# Conceptualizing multi-level determinants of infant and young child nutrition in the Republic of Marshall Islands–a socio-ecological perspective

**DOI:** 10.1371/journal.pgph.0001343

**Published:** 2022-12-19

**Authors:** Stephen R. Kodish, Maryam Matean, Kelsey Grey, Uma Palaniappan, Martina Northrup-Lyons, Akshata Yalvigi, Stanley Gwavuya, Judy Mclean, Wendy Erasmus

**Affiliations:** 1 Department of Nutritional Sciences, Pennsylvania State University, University Park, Pennsylvania, United States of America; 2 Department of Biobehavioral Health, Pennsylvania State University, University Park, Pennsylvania, United States of America; 3 Nourish Global Nutrition, Vancouver, British Columbia, Canada; 4 UNICEF Pacific, Suva, Fiji; 5 College of Medicine, Pennsylvania State University, Hershey, Pennsylvania, United States of America; Jimma University, ETHIOPIA

## Abstract

The East and Pacific region includes 14 Pacific Island Countries where, between 2000 and 2016, indicators of stunting, wasting, and micronutrient deficiencies have plateaued or worsened, while rates of overweight, obesity, and associated disease have risen. The Republic of Marshall Islands (RMI) is no exception: maternal and child nutrition indicators have not improved in decades. A study of the contemporary factors shaping the RMI nutrition situation was needed for informing policy and tailoring interventions. This formative study had an iterative design utilizing qualitative methods. An exploratory Phase 1 included 59 semi-structured interviews with community members, 86 free lists with caregivers, 8 participatory workshops, and 20 meal observations (round 1). Findings were synthesized to inform a confirmatory Phase 2 where 13 focus groups, 81 pile sorts, 15 meal observations (round 2), and 2 seasonal food availability workshops were conducted. Textual data were analyzed thematically using NVivo while cultural domain analysis was conducted in Anthropac. RMI faces interrelated challenges that contribute to a lack of nutritious and local food availability, which is compounded by high food costs relative to household incomes. A decades-long cultural transition from local to processed diets has resulted in infant and young child diets now characterized by morning meals of doughnuts, bread, and ramen with tea, coffee, or Kool-Aid and afternoon meals that include rice with canned meats (e.g., store-bought chicken, hot dogs). Individual preferences for processed food imports have increased their supply. Low maternal risk perception toward nutrition-related illnesses may further explain sub-optimal diets. Improving the RMI food environment will require approaches that align with the multi-level determinants of sub-optimal diets found in this study. As the ten-year 2013 RMI Food Security Policy soon ends, study findings may be used to inform new policy development and investments for improving the nutrition situation.

## Introduction

The East Asia and Pacific region is comprised of 37 countries where economies have grown, health care access has improved, and child mortality has reduced [1]. Despite these gains, the regional nutrition situation remains serious: nearly all forms of malnutrition co-exist among most segments of the population, but especially young children [2]. Stunting (13 million), wasting (4.5 million) and overweight (9.7 million) represent the largest burden of malnutrition among children under 5 years of age in this region [3]. This phenomenon is not unique to the region: globally, countries developing economically are moving from traditional, local diets to food systems that are delocalized and characterized by high-energy, nutrition-poor food choices [4].

A sub-set of this region includes 14 Pacific Island Countries (PIC), where the nutrition situation is just as serious. Between 2000 and 2016, indicators of stunting, wasting, and micronutrient deficiencies have plateaued or only marginally worsened, while rates of overweight, obesity, and associated diseases have sharply risen [2, 5]. Among children, for instance, there has been a 4% increase in stunting and an 88% increase in overweight during this period. At any given time, a third of children in PICs suffer from micronutrient deficiencies. Poverty and inequity, poor maternal nutrition, and sub-optimal health and nutrition practices are leading drivers of child malnutrition in this region; however, their contributions vary by country where the burden of malnutrition also differs [2].

The Republic of the Marshall Islands (RMI) is a sovereign PIC where an approximate 55,000 people live on 29 atolls and five coral islands with 180 square kilometers of land mass [6]. Historically, malnutrition has been a documented challenge for a Marshallese population that lost livelihoods and faced hunger due to food shortages after forced relocation to outer islands due to United States (U.S.) military actions during World War II [7–9]. Since then, then RMI nutrition situation has dramatically changed, and indicators of population hunger are now low, with child wasting at 4.0% [3]. However, the co-occurrence of micronutrient deficiencies, especially clinical vitamin A and iron deficiency anemia, and overweight and obesity have been well documented in RMI [10–14].

Indicators of the maternal and child nutrition situation of RMI have not improved in decades. In 2003, two thirds of adult women were overweight (29%) or obese (31%), while 35.5% of children under 5 years were stunted. By 2020, 70.5% of women were overweight (28.4%) or obese (42.1%) and 37.1% of children under 5 years had stunted growth, a ‘very high’ prevalence classification by the WHO [10]. Also, 25% of households with a child under 5 years included both a stunted child and an overweight or obese mother (i.e. mother-child double burden (MCDB)) [15].

Upstream drivers of malnutrition in RMI are consistent with countries moving through the nutrition transition globally: poverty and inequity, changing demographics, delocalized food systems, and resulting sub-optimal diets reliant on highly processed, nutrient-dense food imports [4]. Specific risk factors for maternal and child malnutrition have recently been investigated in RMI. For instance, short maternal height (<160cm) is a leading predictor of MCDB as well as a risk factor of child stunting in RMI where over a quarter (27.5%) of adult women are shorter than 150cm [10]. But individual-level determinants of nutritional status fall short of telling the full story of RMI whose complex colonial history lends itself to understanding health and disease through more than mere biomedical modelling [16].

Within the RMI food system, individuals make dietary and feeding-related decisions multiple times, every day. Understanding the drivers of those food and feeding choices at multiple, interrelated behavioral levels has not been completed since in-depth work by Gittelsohn and colleagues between 1998–2001 [17, 18]. Therefore, in collaboration with local stakeholders, we carried out a mixed-methods, qualitative study to explore contemporary, community-level drivers of infant and young child feeding practices in urban and rural RMI. This community-based, participatory study had the following aims: 1) understand how household food security may influence diets; 2) contextualize infant and young child feeding practices; 3) explore local conceptions of illness in relation to feeding behaviors; and 4) generate community recommendations for improving the nutrition situation.

## Methods

### Study setting

The RMI has a hot and humid tropical climate that averages 27 degrees Celsius and experiences both a wet season (May–October) and a dry season (November–April) annually [19]. Its societal kinship structure is matrilineal, with a primarily indigenous Marshallese population that speaks both Marshallese and English. Nearly 75% of the RMI population lives in two urban centers, Majuro (Majuro Atoll) and Ebeye (Kwajalein Atoll) [20]. All other RMI atolls are classified as rural.

This study was conducted in both Majuro [urban] and Arno [rural] communities. Majuro is the RMI capital and the economic and political center, where approximately 52% (27,797 residents) of the population lives [20]. Urban fieldwork was conducted in Djarrit town, which is located on the northern end of Majuro atoll and is home to nearly 5,000 people who live on just 0.42 square kilometers of land. Rural fieldwork was completed in Arno and Ine villages on Arno atoll. Arno is the geographically closest atoll to Majuro with a population of nearly 2,000 people. In Majuro, the high population density has put pressure on the delivery of basic social and health services, whereas in Arno the logistics and costs of reaching rural communities only accessible by plane or boat are challenges to service delivery [20].

### Study design and data collection methods

This multi-phase, formative study had an iterative design utilizing qualitative methods. A local data collection team was hired based on previous experience conducting health-related fieldwork, secondary school education level, computer literacy, and language proficiency. Data collection occurred from August until October 2018.

#### Exploratory phase 1

During Phase 1, a total of 59 semi-structured interviews were conducted with female caregivers (*n* = 26), male caregivers (*n* = 13), professional health workers (*n* = 10), senior-level health and agricultural staff (*n* = 3), and community leaders (*n* = 7). Participants were asked semi-structured questions covering key domains including but not limited to community characteristics, health behaviors, food security, feeding and hygiene practices, household member roles and responsibilities, and communication channels. Free lists (*n* = 86) were conducted with caregivers of young children to explore food and illness cognitive domains. Community members joined participatory workshops (*n* = 8) to identify and vote on the top barriers and solutions to optimal nutrition. Direct observations (*n* = 35) of infant and young child meals were conducted in phase 1 and repeated in the same households during phase 2 to reduce reactivity [21].

#### Confirmatory phase 2

Phase 2 was designed to clarify and corroborate findings from Phase 1. Focus group discussions (*n* = 13) were conducted with approximately 6–10 caregivers per discussion to investigate social norms and clarify findings from phase 1 interviews. Seasonal food availability workshops (*n* = 2) generated data to create food availability calendars across seasons. Food availability was specified by symbols indicating foods that are 1) not available, 2) low availability, 3) medium availability, and 4) high availability. Phase 1 free list findings generated items for pile sorts (*n* = 81) to explore local conceptions of food and illness.

### Sampling procedures

Local health workers with knowledge of the community assisted with participant recruitment during both study phases. Health workers escorted research team members to selected communities during fieldwork activities, assisting in courtesy meetings with local leaders and identification of prospective participants. Study participants were purposively sampled at multiple behavioral levels of the socio-ecological model, which served as the theoretical foundation of this study [22–24] (Table 1).

**Table 1:** Theoretical sampling framework with participant types by level of socio-ecological model.

Level of influence	Participant types
Policy	Senior-level health staff from RMI Ministry of Health and Human Services
Organizational	Professional and traditional health workers
Community	Community leaders
Interpersonal	Male caregivers, grandparents
Individual	Female caregivers

Sample sizes were calculated based on achieving data saturation for qualitative methods (i.e., interviews, focus groups, observations) and validity for ethnographic methods generating cultural domain data (i.e., free lists and pile sorts) [25, 26].

### Data analysis

#### Textual analysis

Textual analysis of interviews and focus group data took an inductive approach drawing from Grounded Theory [27]. To do so, an initial codebook was developed using the contents of the data collection instruments. Codes were then used to assign meaning to units of text until themes and sub-themes relevant to the guiding study aims emerged across transcripts. During the coding process using NVivo software, the codebook was continually refined using a team-based coding approach [28, 29]. Textual field notes from direct observations were thematically analyzed by infant or child age range (6–11 mo.; 12–23 mo.). Findings across qualitative methods were synthesized to draw conclusions pertinent to the study aims.

#### Cultural domain analysis

Using Anthropac software, free list data were tabulated to generate a salience statistic for each item based on both frequency of mention and relative rank across lists [30, 31]. Those items with a salience ≥0.30 were used to generate pile sort terms which were analyzing using multi-dimensional scaling procedures. Findings were displayed using maps that fit items of each food and illness domain into two-dimensional figures that considered goodness-of-fit through measurement of a Stress statistic (0 (worst possible fit)– 1 (best possible fit)) [31]. Field notes and interview data were then synthesized to contextualize cultural domain results and create an ethnomedical model of illness.

#### Numerical analysis

Data generated from seasonal food availability calendar and participatory workshops were tallied and ranked based participant votes. Descriptive field notes were reviewed to help explain numerical values.

### Ethical considerations

Ethics approval for the study was granted by the RMI Ministry of Health and Human Services, which approved the use of verbal informed consent due to the minimal risks associated with the study. Verbal informed consent was obtained from participants and recorded with digital recorders prior to data collection. In cases when digital recorders were not used for collecting a specific type of data (e.g., cultural domain data) then written informed consent was obtained.

## Results

### Understanding household food security

#### Food availability

Underlying nutrition-related challenges in RMI are the lack of nutritious food availability and differential levels of nutritious food access for community members in both Majuro [urban] and Arno [rural]. While there are important seasonal influences on local food availability in RMI, the food system relies on processed food imports, which are more readily available than local, fresh foods across seasons. ‘Energy foods’ (e.g., rice, bread) and ‘body-building foods’ (e.g., fresh fish, canned meat) have year-round availability. The majority of ‘protective foods’ (e.g., pandanus, coconut, papaya) have medium availability throughout the year. Local, fresh foods (e.g., breadfruit, taro, makmok, pandanus, coconut, papaya) have variable availability across seasons, especially in Majuro [urban] markets (Tables 2 and 3).

**Table 2:** Seasonal food availability calendar for Rita [urban].

	Jan	Feb	Mar	Apr	May	Jun	Jul	Aug	Sept	Oct	Nov	Dec
Seasons	Wet season (An Ean)				Dry season (An Raak)						Wet season	
Food												
Energy Foods												
Rice												
Bread (bun)												
Ramen												
Biscuits												
Doughnut												
Pancake												
Potato												
Breadfruit												
Breadfruit paste												
Taro												
Starchy root												
Body-building Foods												
Chicken quarter leg												
Canned meat												
Hot dog												
Tuna (fresh)												
Salt fish												
Eel												
Pork												
Steak												
Short ribs												
Octopus												
Giant clams												
Sea snails												
Lobster												
Salted clams with lime juice												
Protective Foods												
Banana												
Watermelon												
Papaya												
Pandanus												
Coconut												
Coconut meat (soft)												
Soft flesh of sprouting coconut												
Coconut nectar												
Others												
Candies												
Ice cream												
Cola												
Chips												
Cheese burger												
French fries												
Color Key	High Availability			Medium Availability			Low Availability			No Availability		

**Table 3:** Seasonal food availability calendar for Arno [rural].

	Jan	Feb	Mar	Apr	May	Jun	Jul	Aug	Sept	Oct	Nov	Dec
Seasons	Wet season (An Ean)				Dry season (An Raak)						Wet season	
Food												
Energy Foods												
Rice												
Flour												
Ramen												
Taro												
Breadfruit												
Biru (breadfruit paste)												
Starchy root (makmok)												
Biscuits												
Body-building Foods												
Pig												
Chicken												
Mackerel												
Tuna												
Ham (processed)												
Sausage												
Corn beef												
Spam												
Luncheon meat												
Corn beef hash												
Eggs (imported, store-bought)												
Quarter leg (chicken)												
Turtle												
Flying fish												
Crab												
Clams												
Shellfish												
Worms (found on Arno beach)												
Protective Foods												
Pandanus												
Banana												
Papaya												
Pumpkin												
Lime												
Apple bell												
Green beans (canned)												
Stewed tomato (canned)												
Corn (canned)												
Mixed vegetables (canned)												
Pineapple (canned)												
Fruit cocktail (canned)												
Soft flesh of sprouting coconut												
Coconut (softer variety, entire coconut, including husk, can be eaten)												
Soft coconut meat (fresh)												
Mature coconut meat												
Coconut tree nectar												
Coconut water												
Others												
Water												
Coffee												
Artificially sweetened fruit juice												
Kool-Aid												
Evaporated milk (Carnation)												
Sugar												
Color Key	High Availability			Medium Availability			Low Availability			No Availability		

The primary challenges to growing food in Majuro [urban] included insufficient space for gardening and refusal of land owner permission to plant. Challenges to home agricultural production in Arno [rural] included not having proper training, lacking the necessary gardening tools and seeds, and disruption by foraging animals which ruins gardens. In addition, copra production, which receives government subsidy, is now more profitable for landowners and, as a result, contributing to reduced agricultural production.

*“Another thing is that we would not think about that* [gardening] *because we take up all of our time collecting coconuts* [for copra production]. *If we distract ourselves by planting in our garden instead of collecting coconuts then there would be no rice*. *That’s the only way…if they need food in the house then it is by collecting coconuts*.*”*

Male focus group, Arno [rural]

#### Food access

Food choice on RMI is also governed largely by what food items are available and affordable on any given day, as imported foods are inconsistently stocked, and household incomes are not always aligned with available supplies.

In Majuro [urban], the key barriers identified were financial as both low household income and high food prices were commonly mentioned. Urban households rely on store-bought foods as lack of space makes homestead production difficult. Fresh fruits and vegetables are often unaffordable so many families purchase cheaper, processed foods to feed large families.

“*When we eat*, *we all gather around and eat*. *Some of the foods we eat are not healthy*, *but we eat them regardless because that is what we can afford…and I cannot afford good*, *healthy foods for all of my family members because there are a lot of us in my house*. *So*, *the reason why we eat unhealthy foods is because of low incomes and expensive*, *healthy foods*.*”*

Male focus group, Majuro [urban]

**Fig 1:**
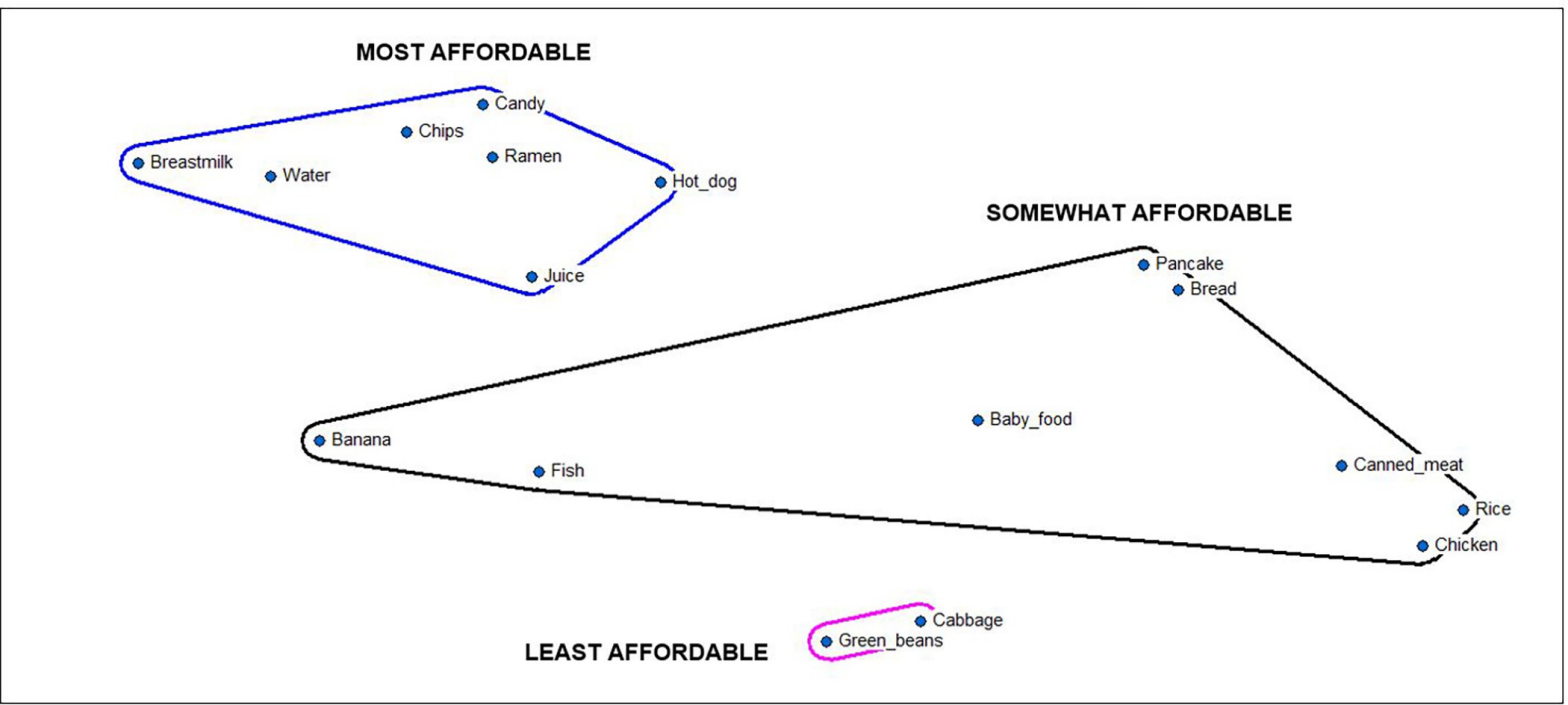
Multi-dimensional scaling map of infant and young child foods by affordability in urban Majuro. *S* = 0.11, Eigenvalue: 11.03, Eigenratio: 7.64.

Structured pile sorting of foods based on affordability corroborated interview and workshop data indicating high affordability of the least nutritious food options (e.g., candy, chips, ramen) relative to others typically provided to infants and young children (Fig 1).

Family size also influences food purchasing patterns, favoring more affordable food items in larger quantities that will be adequate to sustain everyone; household sizes in this study ranged from 4 to 14 including both nuclear and extended family. Food quantity is perceived to be more highly valued than food quality among most study participants whose primary concern is household nourishment.

To the contrary, Arno [rural] community members did not identify financial barriers to food access as the primary challenge for optimal nutrition but instead explained that the lack of agricultural production creates a greater reliance on important food options. Barriers to homestead production of fresh fruits and vegetables, specifically, included poor access to seeds and other supplies, reliance on copra production for household income, and limited knowledge of farming techniques.

*“I’ve heard that we need an RND* [Resources and Development Department which once provided agricultural training] *but we don’t have any RND here* [on Arno island]. *There was one a long time ago but they are gone*. *For us*, *we do not really know how to plant cucumbers and those other things well…like me I do not know how to compost or other* [agricultural] *things*.*”*

Male caregiver interview, Arno [rural]

**Fig 2:**
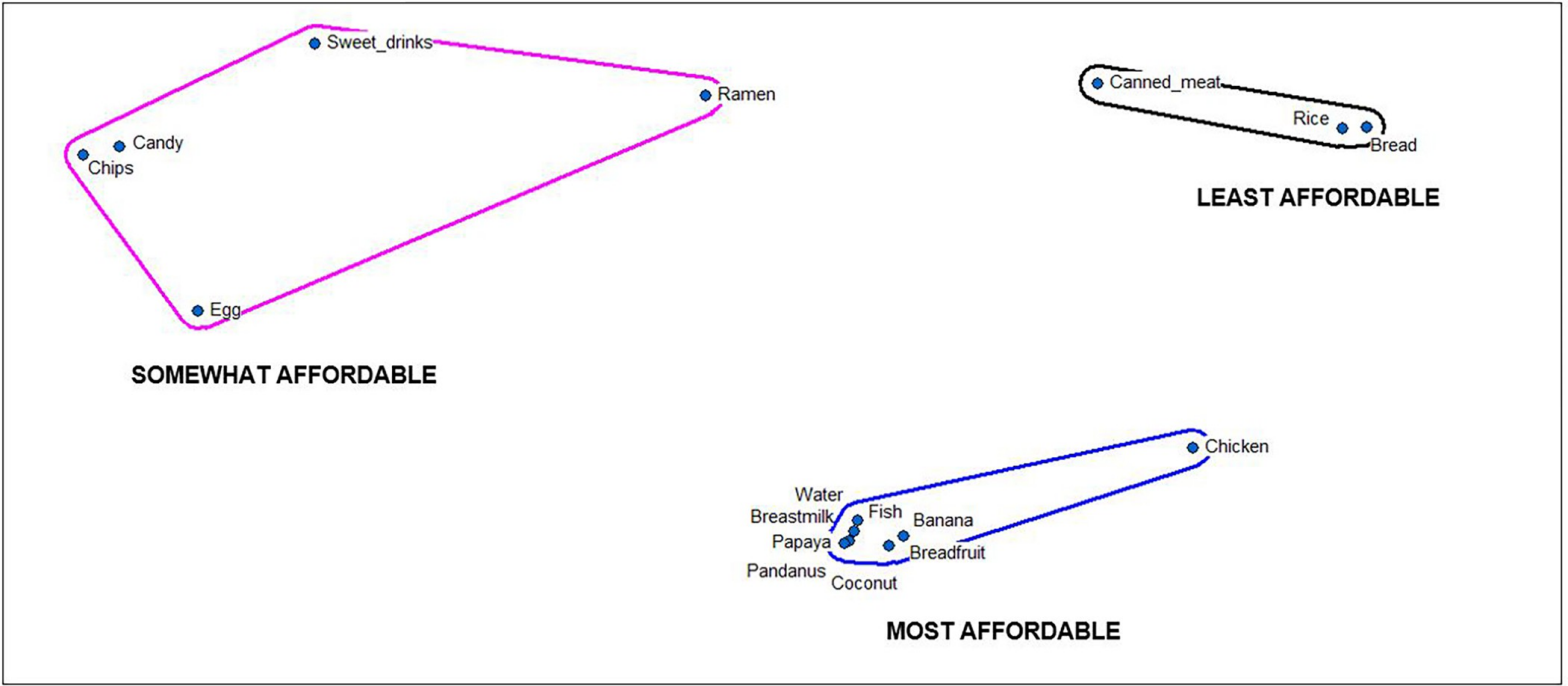
Multi-dimensional scaling map of infant and young child foods by affordability in rural Arno. *S* = 0.06, Eigenvalue: 20.74, Eigenratio: 8.51.

Pile sorting of foods based on reported affordability in Arno [rural] supported interview and workshop data that locally available foods (e.g., papaya, pandanus, coconut) are typically more affordable than imported options (Fig 2).

Further, interview data suggest that over time individual preferences for the convenience of processed foods (higher demand) have increased their supply. Eating processed foods is now an accepted social norm in both urban and rural RMI for adults and children alike. Food purchasing is now considered to be just “more convenient” and “faster” than planting or fishing in both rural and urban settings.

### Contextualizing infant and young child feeding practices

#### Breastfeeding

Caregivers and health workers both reported that early initiation of breastfeeding is now commonly practiced in RMI, a key behavior promoted by professional health workers. In the past, colostrum was not perceived to be healthful, but perceptions toward it are positively changing thanks to health workers who now promote it. Barriers to exclusive breastfeeding 0–6 months do exist: firstly, it is a socially-accepted norm that breastmilk stores are insufficient for infant nutrient needs, particularly in rural areas where health workers reinforce this notion.

Secondly, traditional medicines, including herbal medicines made with untreated water for *kijonkan* (infant jaundice), are common treatments for infants in early life. There is a positive influence of healthcare professionals (doctors, nurses) who do promote exclusive and continued breastfeeding practices which can persist well after a child’s second birthday in some households. Direct observations revealed that continued breastfeeding occurred in 38% (urban) and 56% (rural) of study households with children aged 6–23 months, however. Both individual and community perceptions contribute to breastfeeding cessation before two years.

#### Feeding of infants and children aged 6–23 months

Caregivers reported feeding infants and young children aged 6–23 months three meals per day: morning, midday, and evening meals. Meal observations found that typical household meals consisted disproportionately of store-bought foods in both Majuro [urban] and Arno [rural] RMI. Morning food items included bread, pancake, doughnut, ramen, and either tea, coffee, or Kool-Aid. Mid-day and evening meals consisted of rice, canned meat, and fish in Arno [rural], with the addition of store-bought chicken and hot dogs in Majuro [urban].

Intra- and inter-household food sharing was observed during most meals and was identified as a core cultural value reflective of an underlying interdependent cultural context. The Marshallese phrase, *jake jebol eo* (sharing is caring), which was identified during interviews, was explicitly expressed through mealtime food sharing.

Common first foods for infants included pandanus juice and pandanus paste, both locally available. In Majuro [urban], store-bought baby foods and cereals were also commonly fed to infants. Imported rice, the primary staple of RMI, contributed to the largest proportion of energy intake among infants and young children. Infants and young children commonly ate foods comprised of processed starches and refined carbohydrates such as ramen, bread, and store-bought biscuits during meals. Fresh fruits and vegetables were said to be nutritious and important for good health during interviews, but direct observations revealed a reality of very limited consumption among infants and young children, particularly in rural households. Caregivers largely used food-based incentives (e.g., juices, biscuits) to soothe and to encourage eating during meals.

Observations of infants aged 6–11 months, specifically, revealed very little consumption of animal-source proteins and instead a reliance on a locally-made watery porridge called *likobla*, comprised of flour, coconut milk, and sugar. Among young children aged 12–23 months, approximately half of observed households provided meals to children with at least one animal source, including canned tuna or mackerel, corned beef, Vienna sausage, ham, or hotdogs. All household meals in Arno [rural] and Majuro [urban] included at least 3 sources of refined carbohydrates, typically white rice and flour-based foods such as bread or donuts.

While snacking was not commonly observed among infants aged 6–11 months, it was very common among young children aged 12–23 months and largely consisted of chips, cookies, sweets, and chocolate food items. Interview data suggest consistently high knowledge among caregivers about the importance of dietary diversity for infant and young child nutrition, citing the healthfulness of fresh fish and a wide range of seasonal fruits and vegetables. In practice, however, locally-available, nutritious foods were not central to infant and young child diets during meal observations. Tea and coffee consumption by infants and young children were reported in interviews but rarely observed; sugary drink consumption (e.g., Kool-Aid) was commonly observed though.

### Local conceptions of illness

Local conceptions of infant and young child illness may help to explain the gap between knowledge and practice in RMI. The most salient infant and young child illnesses in RMI were fever, cough, and diarrhea. Nutrition-related illness terms, such as malnutrition, micronutrient deficiencies, underweight, overweight/obesity, wasting, and stunting, did not emerge among the top ten most salient illnesses to caregivers in either Majuro [urban] or Arno [rural], suggesting lower perceived importance relative to other illnesses (Table 4).

**Table 4:** Salient infant and young child illnesses in Majuro [urban].

Rank	Illness term (Marshallese)	Illness term (English)	Frequency (%)	Average Rank	Salience (S)
**1**	*Bwil*	Fever	93.5	2.16	0.74
**2**	*Bok bok*	Cough	80.4	2.19	0.61
**3**	*Bidrodro*	Diarrhea	69.6	3.28	0.39
**4**	*Pilo*	Pink eye	32.6	4.00	0.16
**5**	*Kajjinok*	Asthma Shortness of breath	21.7	2.80	0.14
**6**	*Metak lojeen*	Stomach ache	17.4	3.38	0.10
**7**	*Uwor*	Runny nose	17.4	4.63	0.09
**8**	*Molanlon*	Nausea	15.2	4.71	0.07
**9**	*Metak boran*	Headache	10.9	4.00	0.07
**10**	*Kor kori*	Skin rash	23.9	6.36	0.06

In Majuro [urban], 28 unique illnesses were reported during free listing. Local terms for *jabwe on* (lacking nutrients/malnutrition) and *jabwe botoktok* (anemia) were ranked at 17^th^ and 27^th^, respectively (Table 5).

**Table 5:** Salient infant and young child illnesses in Arno [rural].

Rank	Illness term (Marshallese)	Illness term (English)	Frequency (%)	Average Rank	Salience (S)
**1**	*Bwil*	Fever	97.5	1.51	0.87
**2**	*Bokbok*	Cough	80.0	2.06	0.65
**3**	*Bidrodro*	Diarrhea	67.5	3.44	0.39
**4**	*Kor kori*	Skin rash	40.0	4.94	0.19
**5**	*Molanlon*	Nausea	37.5	4.87	0.17
**6**	*Pilo*	Pink eye	27.5	4.36	0.15
**7**	*Uwor*	Runny nose	27.5	4.45	0.14
**8**	*Ebboj lojeen*	Stomach bump	22.5	5.56	0.09
**9**	*Metak boran*	Headache	22.5	5.11	0.11
**10**	*Wot*	Boil	17.5	6.29	0.07

**Fig 3:**
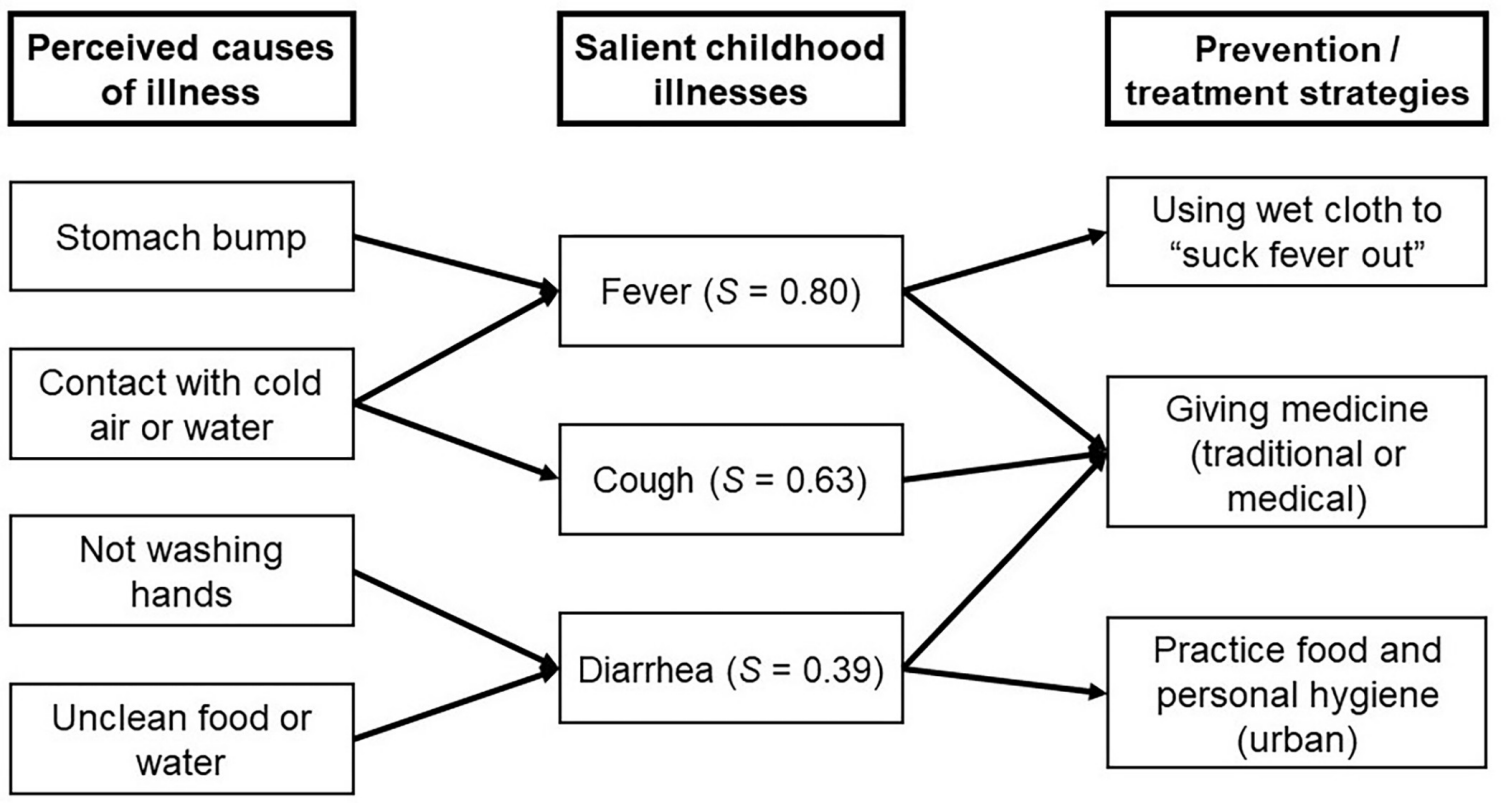
Ethnomedical model of salient infant and young child illnesses. Each perceived cause and prevention/treatment strategy represents a salient theme from interview or free list data.

In Arno [rural], 26 unique illnesses were reported, with nutrition-related illnesses ranking 13^th^ and 14^th^ for *jabwe on* (lacking nutrients/malnutrition) and *pilo in bon* (night blindness), respectively. Interviews further explored conceptions of local illnesses. *Jabwe on* (lacking nutrients/malnutrition), *jabwe botoktok* (anemia), and *pilo in bon* (night blindness) were ascribed to “*missing key foods in the diet*” suggesting an understanding of the connection between illness and diet. Free list and interview findings were synthesized to create an ethnomedical model of illness using the most salient illness terms (Fig 3).

Locally specific conceptions of illness etiology, such as *ebboj* (stomach bump caused from a fall), were found to be important perceived causes of childhood illnesses. Fever was said to be caused by exposure to cold air, cold water, or *ebboj*. *Ebboj* can only be cured through traditional medicines, as it is not a clinical diagnosis and is not formally treated.

The ethnomedical model suggests an underlying medical belief system that considers a combination of biomedical and traditional explanations for disease. Preventative strategies and remedies for *bwil* (fever), *bok bok* (cough), and *bidrodro* (diarrhea) included a combination of both traditional (e.g. ‘using a wet cloth to suck out a fever’) and clinical approaches (e.g. ‘giving Tylenol’).

*“It depends on what type of illnesses they* [infants and young children] *have*. *If the illness can be cured by the traditional healers*, *then they will take their kids to them*. *But if it can be cured by medication*, *then they will take them to the doctors*.*”*

Female focus group, Majuro [urban]

Decisions of when and how to seek care depends on type of illness (e.g., *ebboj* (stomach bumps) are only curable through traditional medicine) as well as consideration of the associated costs, such as those for transportation and clinic fees.

### Community recommendations for improving the nutrition situation

During participatory community workshops, the top voted solutions to improve infant and young child nutrition differed between [Majuro] urban and Arno [rural], given their context-specific challenges. The top-voted solutions to overcome **low financial access** in Majuro [urban] communities included: 1) improving food affordability by decreasing prices of healthy foods, 2) increasing production of traditional Marshallese foods, 3) increasing household salaries, and 4) government-funded food programs for school-aged children.

The top voted solutions to help overcome **low agricultural production** in Arno [rural] communities included: 1) making farming more accessible by providing seeds and gardening tools, 2) implementing programming to teach effective farming methods, 3) improving the nutrition of young children through school lunch programs, and 4) increasing availability of technology to help with food storage.

## Discussion

The RMI nutrition situation is as serious as it is complex. Studies to understand these drivers have illustrated that it is not one but a combination of enabling, underlying, and immediate factors that, in combination, influence population nutrition [32]. In RMI, we observed meals where young child diets were predominantly comprised of nutrient-dense, processed food imports. Seasonal food availability calendars and interviews with agricultural experts, leaders, and caregivers illustrated how those diets are a result of an unreliable food system for ensuring nutritious diets. We found a food environment where nutritious food availability is limited and inconsistent across seasons, coupled with low food access due to high prices relative to incomes. Losses of traditional livelihoods of fishing and agriculture have contributed to a food environment where it is easier to find and feed energy-dense, non-nutritious young child foods than it is locally available nutritious diets. Understanding the present-day food environment of RMI benefits from a historical perspective that weaves together a complex narrative involving colonialism, trade liberalization, aid dependence, and present-day policies.

RMI was occupied by Japan during World War I and the U.S. during World War II when its outer atolls were used as a testing site for nuclear weapons [9]. In addition to the direct consequences on Marshallese health, these occupations have had far-reaching consequences for the livelihoods, health, and nutrition of Marshallese [7, 8, 33]. Historical evidence implicates this foreign influence on changes in food consumption patterns, from diets consisting of locally available foods to those reliant on processed imports [34, 35]. Rising rates of overweight and obesity coincided with the advent of U.S. subsidies in the 1960s and continue today [36]. Nowadays, the United States provides economic assistance to RMI under the Compact of Free Association, an agreement that, coupled with changes in global food trade patterns, helps ensure a foreign food import dependency [34, 37].

Between 1987 and 2013, international food trade increased dietary diversity for much of the world, but at the expense of a heighted dependency on imports [38]. In fact, more than 80% of the countries in the world are now reliant on food imports to meet population demands. RMI is no exception: its food system is delocalized, whereby dietary requirements are met using food items shipped from distant places through commercial channels [39]. Dietary delocalization reflects trade liberalization policies which can result in national economic gains at the expense of population health. There is a positive relationship between trade liberalization and increased processed food imports in the Pacific Island Countries where they amounted to $9,264 million USD, continuing to increase from 2003 until 2013 [37]. The top imported food items included high sodium meat products (e.g., canned meats and fishes), as well as nutrient dense, non-nutritious cereals (e.g., rice). In RMI, at least 90% of the food supply is imported, contributing to a food environment where more than one third of all households are food insecure [36, 40].

RMI communities are now characterized by a cultural context where traditional beliefs and practices persist but have been adulterated through increasing modernization. Caregivers told us that infant and young child feeding is not the sole responsibility of primary caregivers, but an effort shared by health professionals, neighbors, friends, and family. This collectivism is a core cultural value of not only Marshallese communities but also the Pacific region at large [41]. Collectivism is important for understanding food choices that are made within family structures that average 6.8 persons/household [20]. In the modern food environment where nutritious foods are expensive and not always available, food purchasing decisions consider the ability of food items to feed the entire family, resulting in preferences for foods that favor energy adequacy (e.g., rice) to nutrient quality (e.g., locally caught fish).

Modernization also means that caregivers face more competing demands than they did in the past. For families that engage in copra production, a subsidized cash crop in the RMI economy, time for homestead food production, traditional meal preparation, and optimal feeding practices were said to be “*too time consuming*.” Thus, individual food choices on RMI have been slowly shaped by the increased convenience and low cost of processed food imports in an economy where much time is now spent outside of the house working [42]. As a result, personal taste preferences now favor energy-dense, processed food options to traditional staples. Present-day food choice and feeding behaviors are a consequence of a rapidly changing socio-cultural landscape where economic liberalization and modernization have resulted in both high supply and demand for processed food imports among adults, adolescents, and children alike [18, 34, 37, 38, 43].

We designed this study to also generate recommendations to improve the nutrition situation. Among urban participants who primarily source food from stores on Majuro, we heard that improving food accessibility should be a priority. In 2003, Gittelsohn and colleagues implemented a 10-week store-based intervention that aimed to do so by pairing nutritious food stocking with tailored communications (e.g., recipes, cooking demonstrations, point-of-sale prompts) using mass media and in-store promotions throughout Majuro [42]. This trial increased consumer knowledge of diabetes and food label reading, as well as resulted in increased purchasing of promoted foods [44]. Importantly, the intervention was tailored to the cultural context of RMI based on in-depth formative research, one likely reason why consumer exposure to the intervention was high and positive impacts on cognitive and behavioral factors were seen [45]. Working with vendors within food stores of RMI to improve nutritious food accessibility may hold promise.

Nowadays, interventions tailored to RMI may benefit from embracing both the old and the new: designing behavior change communication strategies that value the longstanding oral traditions of storytelling and power of face-to-face communications yet harness the utility of social media and phone usage has potential for both impact and coverage [46, 47]. Increasing modernization is a reality in RMI and thus interventions aiming to reach all population segments may consider using such tailored approaches to blend the old with the new. We found that traditional medicine is an important aspect of Marshallese culture still today. It is not uncommon for infant jaundice (*kinjonkan*) to be treated using herbs and water, a practice reflective of an underlying medical belief system that ascribes illness to both internalizing and externalizing forces [48]. In 2003, a third of RMI survey participants agreed that diabetes is sometimes caused by ‘black magic’ [18]. While much traditional knowledge of agricultural practices and fishing has been lost during over a century of colonialization, our rural study participants suggested agricultural training and provision of farming resources to engage in homestead food production as strategies to improve the food environment. Although a three-year (2011–2014) horticulture project on RMI saw only modest positive effects on fruit and vegetable consumption through provision of resources and training, lessons learned may be applied to designing similar interventions across the food value chain of RMI [36, 49].

Tailored intervention approaches, embodied by biomedical principles that acknowledge traditional medical belief systems, may be appropriate, acceptable, and effective in RMI. For instance, investments in health worker capacity strengthening at both facility and community levels may improve service delivery. Conducting tailored trainings using formative findings that enable health assistants to become qualified nurses within facilities, as well as dually doing so to enhance capacities among the current cadre of RMI health workers to more effectively deliver preventative services, such as nutrition promotion, at community level, may help improve the full continuum of care. To be sure, investments in community health workers has been successful in similar contexts where facility-based approaches fell short of reaching more vulnerable population segments [50–53]. In RMI, community health workers may help to fill health access challenges, especially on the outer islands, where coverage of services is lower than on Majuro [54]. Additionally, Marshallese community health workers would be well positioned to listen and understand community challenges, while problem solving nutrition problems using locally available strategies and indigenous knowledge backed by evidence-informed solutions [55, 56]. Promoting nutrition from Marshallese health workers may be an appropriate, effective, and sustainable interpersonal approach to behavior change.

Improving the RMI food environment will require multi-level approaches that align with the multi-level determinants found in this study. Doing so will also benefit from multi-sectoral synergies that consider not only food and nutrition, but also, for example, water, sanitation, and hygiene, given the importance of infection for infant and young child nutritional status [57]. Regardless of the planned approaches, political will and ample time for sustainable and successful implementation will be foundational. Now is an opportune time to evaluate the effectiveness of the *2013 RMI Food Security Policy*, which will soon be 10 years old and may be coupled with a 2023 health financing transition with implications for public health service delivery [58, 59]. Critically reviewing the policy contents and their implementation, collating the lessons learned from previous interventions, and incorporating research findings, both old and new, may help to shape a forward-thinking ‘do no harm’ policy inclusive of the present-day food environment with consideration to new challenges such as COVID-19 and climate change [19, 60–62].

## Conclusion

Creating a healthy food environment where caregivers of infants and young children find accessing nutritious foods to be convenient, preferable, and affordable is important yet complex. Doing so may require an interdisciplinary, systems-based, and multi-sectoral approach backed by political will, evidence, innovation, and collaboration [12]. Contemporary nutrition challenges on RMI have been shaped by historical events and geopolitical forces that are not easily undone. Without action, the food environment of RMI will remain a place where caregivers of infants and young children struggle to find nutritious, affordable diets for healthy growth and development.

## Supporting information

S1 Data Sources of data underlying key findings. (ZIP)
