# Supplementary material for: Conceptualizing multi-level determinants of infant and young child nutrition in the Republic of Marshall Islands–a socio-ecological perspective
Source: PLOS Glob Public Health. 2022 Dec 19;2(12):e0001343. doi: 10.1371/journal.pgph.0001343 (PMC10022247; doi:10.1371/journal.pgph.0001343)
Supplement: S1 Data — (ZIP) [file pgph.0001343.s001.zip › RMI Supp Data/Focus groups data/F01U_FGD_Female_Rita_Aug 27_Libon.docx]

- Interview code: F01U
- Interview type and interviewee: FGD
- Interview Date: Aug.27.18
- Location: Rita
- Interviewer: Libon
- Transcriber: Marcellina

**I: before we proceed, do you ladies want to participate in this little chit-chat focus group?**

R: yes

**I: okay. We’re going to start now, and we will all introduce each other by telling our name, how many children we have and how old they are. We will start from this side.**

R1: My name is Eoju and I have 12 kids. My eldest is 27 and the rest are 26,21,18,13,10,9,7,6, and my youngest is 1 year old. I lost two of my children.

R2: My name is Terina and I am from Aelonlaplap and Majuro. My father is from Aelonlaplap and my mom is from here. I have three kids; my eldest daughter is 10 years old and she’s in Ebeye with her father, the other one is 5 years old and she’s attending school, and my youngest is 1.

R3: My name is Minne Bokna and I am from Namdrik and Rongelap. I have 4 kids; my eldest is 19 years old, my other two are 15 and 12, any my youngest is 6 months old.

R4: My name is Nina Lakiem and I have a 2 years old kid

R5: My name is Selma and I have 3 kids; I gave birth to my eldest and lost him/her at 4 months old, my second daughter is 3 years and my youngest daughter is 6 months old.

R6: My name is Jenny Jerilon and I am from Ebon. I have a 7 years old daughter and a 11 months old son.

**I: okay that was amazing. Now we will start talking about women’s health. The first question asked, can you describe the appearances of a healthy woman? Like for example, does she have to be fat to be considered a healthy woman or does she have to be thin or in between?**

R1: she must have a sexy body shape

R2: in between

**I: as you are saying in between, what do you mean? Medium size or what?**

R2: not too fat and not too thin.

**I: okay are there more descriptions?**

R: I think that’s it, she’s in between

**I: what about face expression?**

R: [06:58-07:04]

**I: okay what else?.......... what ever you say there is no right or wrong answer. And it’s just us to each other, don’t feel ashamed to express your thoughts.**

R: she doesn’t use glasses...

**I: yes, that’s another one. What else?......... just picture out a healthy woman, what does she have?**

R: she runs a business (store)….

**I: are there no more? Those are the only things that you guys can think of, right?**

R: hmm

**I: okay. Now can you tell me what are the most heathy and righteous foods for pregnant women?**

R: the imported foods

**I: if you are saying imported foods, what kinds of imported foods?**

R: like apples, cabbages, carrots and other imported foods

**I: are there any more foods?...... she stated fruits and vegetables, are there any other foods beside fruits and vegetables?**

R1: local foods. Like fish, banana

R2: the protective foods

**I: what kinds of protective foods?**

R2: like banana, papaya….

**I: are there any more ideas from others?... Jennifer?.... what about can foods?**

R1: well you know us Marshallese women always prefer Saba Mackerel and corn beef

**I: okay Saba Mackerel is good for pregnant women**

R2: yeah these can foods. Especially corn beef since it helps a lot in produce breastmilk

**I: yeah well, we haven’t come yet to the breastfeeding session, we’re still talking about during pregnancy. So, give examples of what kinds of food you ladies were suggested to eat during pregnancy.**

R1: we forgot the foods we were suggested to eat. We only remember the foods we disliked

**I: (giggles) okay so are the suggested foods different from the foods you usually eat?**

R: yes

**I: how are they different?**

R: foods or meals that we usually eat, don’t contain fruits and vegetables. Only rice and meats.

**I: okay good. So, can you tell me the reasons why fruits and vegetables are not in your daily meals?**

R: we are not aware. because at that time that we’re not pregnant, we thought that it was okay if we only eat rice and meats without vegetables and fruits.

**I: okay that’s really good. Are there any more ideas about the differences in the foods?..... why pregnant women must be had different kinds of food?**

R: because it comes from what they are craving for. Some pregnant women craved for uncooked rice, soap, or cigarettes (laughing)

**I: why are they craving for these?**

R1: we don’t know… it’s always depended on what we want to consume even though we know they are not healthy.

**I: okay what are the differences in the foods you ate during your pregnancy?**

R1: I just want them… local foods also.. like banana..

**I: foods you usually consumed.**

R1: sashimi…

**I: now, as for the foods you ladies were encouraged to eat like fruits, vegetables, and local foods. Who or what encouraged you to eat those kind of foods during pregnancy?.. who else beside the doctors?**

R: our family members and the fathers of our kids

**I: good. Is there anyone else?..... okay, as you guys said the doctors, how did the doctors encouraged you guys to eat those kinds of foods?...... why did they want you guys to eat those kinds of foods?**

R: so, we can be healthy especially our babies

**I: okay good, are there more reasons?... what about illnesses?**

R: so that our babies won’t easily get affected by any type of illnesses

**I: good. Now in some places, there are certain kinds of foods that pregnant women should not eat. For example, in some countries they state that if a woman eats eggs during her prenatal period, her baby will be born a thief. This is one of their beliefs. What about people here in the Marshall Islands, do they have beliefs?**

R1: we don’t know any (laughing)

R2: well all I know is that when my kids eat eggs a lot they get heart disease

**I: are there any more beliefs?..... what about octopus?**

R: octopus? What about it?

**I: are there any beliefs on eating octopus?**

R1: they say it’s not good to eat it during pregnancy

**I: why is it not good?**

R1: because it’s kind of like, it’s a bad luck because we might have miscarriage

**I: are any more beliefs?**

R2: we don’t actually know about octopus, all we know is that Marshallese really love to eat octopus.

R3: some people are allergic to it

**I: okay. Are there any other foods that if you eat, it will affect your unborn baby?**

R1: those that we mentioned before that will harm our baby like soap. It is good with some mother because they said that their baby will be clean when they born (laughing)

**I: so, when they eat soap what do they say about it?**

R: good and they want to. They just go along with what they want to eat but it’s true what the doctors said about the foods we should not eat because they will harm our babies.

**I: okay but you were saying that when they eat soap, their babies will be clean when they born right?**

R: yeah with some mother. Eating soap, it’s like drinking warm water during our pregnancy because we believe that if we drink warm water, it will help with cleaning our baby.

**I: We know that some women receive supplements for low blood during pregnancy, like iron-folic acid (pills for blood). Some mothers told us they consumed all the supplements they were given during pregnancy but others did not. Could you explain any reasons why some mothers do not consume all their supplements?**

R1: they hate it

R2: they feel nausea when they take it

**I: okay so one of you said because they hate it, some said because they feel nausea when they take it. Are there any more reasons?**

R: no more

**I: now, what does help a pregnant woman to take all her supplements? What do they do to take all their supplements?**

R: they put up positive thoughts in their mind by telling themselves that they should take all their prenatal bills as it was recommended by the doctors so that their baby will be healthy and won’t have health issues when is born. And so that they will have enough blood at labor.

**I: okay that’s good. Are there any more reasons?... are there anything else pregnant woman should do to have all her supplements?**

R: are there any more ideas from there ladies

**I: there is no right or wrong answers. Whatever you think of..**

R1: we should eat a lollipop to help us swallow the bills (laughing)

**I: good. So, you are saying that when you eat sweet it helps you swallowing the pills right?**

R1: yes

**I: okay good.**

R2: as for me, because the doctors have recommended me to take my bills, I really put my efforts on the recommendation even though I hate the bills.

**I: okay. So, what are the consequences of having low blood during pregnancy and childbirth?**

R: there are many issues of having low blood during pregnancy and childbirth. there are many pregnant women faints at labor, we experience pain in our body, we have tried skin..

**I: okay what else?.. what at labor?**

R: if we have low blood during pregnancy, there will difficulty in giving birth to our baby because we’re weak.

**I: are there any more ideas from the other ladies? You guys seem to be quiet…. Now, were there any advices given from the health workers to prevent or treat low blood?**

R: yes, they told us not to eat foods that will cause low blood like salt, lime, what else….

**I: okay good. Why did the doctors recommend you to take the iron folic acid?**

R: they just gave us and told us that we must have it

**I: they didn’t give the reasons why is it important for you to have the iron folic acid?**

R: they didn’t.

**I: okay. Are there any more ideas from that side?..... now, let’s talk about feeding babies after they are born. Once the baby is born and you begin breastfeeding, can you describe a healthy and nutritious diet for women who are breastfeeding?**

R: fish, local foods, banana

**I: what about can meats?**

R: corn beef, Saba Mackerel

**I: okay. Now similar to what we discussed earlier about “food taboos” during pregnancy, can you describe any “food taboos” that exist for women who are breastfeeding?**

R: we believe that if we eat sashimi during breastfeeding, our baby will bite our breast

**I: good are there any more beliefs?.... You know I once heard it from my grandma about eating octopus during pregnancy. She said that if we eat octopus during pregnancy, it’ll be hard for us to give birth to our baby. It’s like our baby don’t want to come out of their womb just like an octopus when it’s in its hole. The octopus will keep holding on to its hole with its tentacles when we try to get it out of it. That’s a belief about the foods we eat during pregnancy. What about breastfeeding? Are there any more beside eating sashimi?**

R: that’s all we know about.

**I: okay now, what advice have you heard from health workers about breastfeeding?**

R: they told us that it’s very important not to stop breastfeed our babies because they won’t easily get any type of illnesses if we keep breastfeeding them.

**I: yes, that right. What else?**

R2: they told us that we should only breastfeed our babies and not bottle-feed them because if they get sick, breastfeed will help them if they hate foods not like bottle-feed.

**I: good. Are there more?.... what about the family members? are there any advices from them about breastfeeding?**

R: there is one. They advise us not to lie down with our babies and breastfeeding them because they will just want to stay beside you. If we try to stand up from them they will cry.

**I: see? That’s another belief.**

R2: they also said that if we breastfeed and we go and have fun (do the inaccurate stuff), somehow our babies will be affected and might have illnesses like diarrhea (*it’s a belief for single mothers)

**I: okay that’s another belief. What about advices given by our family members about breastfeeding?**

R: they also advise us to breastfeed our babies and not bottle-feed them

**I: okay great. So, we’ve heard some mothers first introduce foods other than breast milk when their baby is 6 months old, while some introduce foods earlier or later than 6 months. Could you describe the reasons why some mothers introduce foods or liquids at 6 months of age?**

R: because as our babies grow more older, their appetite gets more effective like they want to be breastfed more frequently. So, we give them foods at 6 months because their breastmilk is not enough anymore for them.

**I: okay what else?..... what about before 6 months of age? Why some mother introduced foods or liquids before 6?**

R: because its their belief that some babies can eat soft foods like likabla (flour mixed w/water and sugar. Cook it make it really soft for 3mos. old) at 3 months old.

**I: oh okay. So, who’s belief was that?**

R: it’s our ancestors. And at 6 months old they can now eat foods that are little bit more solid like baby food (ones bought from the stores), and atat (made from starch)

**I: okay. But some woman introduced foods after 6 months of age. Why is that?**

R: what can we say, they don’t care about their baby’s health because they don’t feed them foods at the actual month foods should be given.

**I: Many mothers have told us that they did not have enough breastmilk to feed their child. Can you explain to me how children under 6 months are fed when their mothers do not have enough breastmilk?**

R: they are usually bottle-fed and eat foods

**I: okay. Now, are there any advice or ways to increase breastmilk?**

R: eat what we usually eat in the past; fish and coconut [laughing]

R: we should eat those kinds of food that help produce breastmilk

**I: okay. So you said we should eat those kinds of food that produce breastmilk, can you tell me where did you get that advice from?**

R: from the doctors

**I: is there anyone else?**

R: from our parents

R: from our husband

**I: now could you describe for me how mothers in this community know that it is time to stop breastfeeding their child?**

R: there are some babies that just don’t want to be breastfeed anymore. But there are some mothers that they willingly stop breastfeeding their baby because they want to enjoy themselves and have fun.

R: from our own knowledge, we stop breastfeeding them because it’s time for them to eat foods.

**I: okay. Are there any more ideas?...... now, some people have mentioned that they try to feed their young children a balanced diet. Can you explain what people mean by a balanced diet? Like what kind of foods with balanced diet?**

R: protective foods, energy foods, and body building foods

**I: can you explain in detail what kind of protective, energy, and body building foods?**

R: like rice, bread, and local foods like banana…

**I: okay. Is there more?... to you, what does balanced diet means to you?**

R: what can we say, energy food like fish, protective food like bread, and energy foods like banana, papaya, and all local foods

**I: For the last question, we’d like to learn about how decisions are made. Can you explain anything that influences mothers’ decisions about breastfeeding their young children in this community?**

R: we breastfeed our baby so that they won’t easily have illnesses. Some mother feels depressed about their babies being sick because they stop breastfeeding them.

**I: are there any other advices given to you?**

R: we were told to keep breastfeed our baby because they won’t hate it when they get sick not like bottle-feed. If they get sick, they won’t want to be bottle-fed.

**I: great! now would like to ask a few questions about children when they are sick. When children under 2 get sick, some parents take their children to the doctor first and others use traditional healing first. Can you describe the reasons for this difference?**

R: it depends on what type of illnesses they have. if the illness can be cure by the traditional healers, they will take their kid them. But if it can be cure by medication, then they will take them to the doctors.

**I: okay. What about the other ladies? Do you guys take your kids to doctors first or to traditional healers?**

R: to the doctors.

**I: why? Can you tell me the reasons why do you take your kids to the doctors first?**

R: so that they can do a check up on them and decide what kinds of medicine they should take

**I: what about others? Why do they take their children to the traditional healers first?**

R: from their own belief, they believe that traditional medicines can heal their children

**I: now, what kind of illnesses that they usually used traditional medicine for cure?**

R: illnesses like KIJONKAN (kind of illness that every baby is born with--jaundice)

**I: okay, can you tell me what kind of traditional medicines they used for KIJONKAN?**

R: there are lots of traditional medicines for KIJONKAN! Like the small shell that usually stay under rocks, kiep (local medicine), ulej (local medicine)…

**I: okay. can you tell me who influences whether traditional medicine should be used?**

R: there are people that know how to do magic on who you should take your child to for cure. So they are the ones who influence us to use traditional medicines

**I: now, can you describe how children are fed when they are sick compared to when they are not sick?**

R: when they are sick, they are picky on food they should eat. but when they are not sick, they eat every kinds of food.

R: when they are, they don’t eat if they feel nausea

**I: what kinds of food they usually want to eat?**

R: fruits, vegetables, and candies

**I: Now we would like to learn about the foods that you provide for your family. Could you talk about what foods people in this community usually provide for their families?**

R: rice, flour, can meats, chicken….

**I: what else?.... okay now can you tell the difficulties in getting the foods for their families?**

R: not enough money

**I: what else?**

R: that is the most reason [laughing]

**I: okay. Now can you tell me how families deal with food shortages?**

R: they do whatever that can make money. Like selling things like the handicraft they make, snack, or whatever they good at making.

**I: good. Now many families have told us that fresh vegetables are not affordable. Can you describe any other reasons that families do not eat many fresh vegetables?**

R: when they have money, they think more about what they want than buying them.

**I: what else?....okay now in the next section, we would like to talk about water and hygiene. Can you please describe how people typically get water for their families in this community?**

R: some use water tank, some use water well, and some use the government water

**I: okay good. Now are there any difficulties in getting water in this community?**

R: as for the water well, the difficulty is that in someplace, the underground water is salty.

**I: what about water tank?**

R: the difficulty is when the roofs are not clean and need to be repaired.

**I: what about the government water?**

R: the difficulty about using the government water is money. If we don’t have enough money to pay for it, then we will not use it.

**I: these information are really good. Now, are there any difficulties in storing water?**

R: yes, there is. Some household don’t have water tank because they don’t have enough money to buy one for them.

**I: good. Now we’ve heard that some families boil their water for drinking and others do not. Can you explain why some people boil their water and others do not?**

R: some families boil their water because they are aware that their drinking water is unclean since they don’t clean their roof.

**I: what about those families that don’t boil their drinking water. why is that?**

R: maybe they just don’t want to because they feel lazy

R: maybe they don’t have gas for their gas stove

**I: now, are there any difficulties to keeping water catchment systems clean?**

R: yes, the trees. When the leaves of the trees fall down to our water catchment, and when the roofs are not clean because they are rusty.

**I: okay good. Now we’ve heard that some families wash hands regularly while others do not. Can you explain some reasons for this difference?**

R: some families wash their hands regularly because they don’t want to have illnesses

R: and some families don’t wash their hands because maybe it’s one of their habits. Maybe they grew up not being practiced washing their hands.

**I: now, can you tell me the reasons why some people wash their hands with soap and others do not?**

R: those that don’t wash their hands with soap, we can say that because they feel lazy to do it

**I: what about those that wash their hands with soap?**

R: they wash their hands with soap because they are protecting themselves from any illnesses

**I: okay. Now, can you tell me the reasons why some people use hand sanitizer instead of soap?**

R: because hand sanitizer just introduced now a days but, in the past,, we used soap.

R: because its way fast to wash our hands with and its more helping in cleaning our hands

**I: Now for the last questions, we would like to learn about how parents care for their children. We’ve heard that husbands are an important support for their wives during pregnancy. Can you explain what husbands do to support their wives while they are pregnant?**

R: they look for ways to find what we are craving for.

R: they don’t make us feel depressed

**I: yes, that’s right. Are there more..... now, what mothers or other family members do to support their daughters while they are pregnant?**

R: they advise us on what we must eat and what we must not eat during our pregnancy

**I: okay, are there more?..... now, we are interested in learning about how caregivers play with children under 2 years. Can you describe for me in detail how you play with children?**

R: make them laugh by tickling them, bring them toys to play with….

**I: okay, what else?....**

R: sing a song to them

**I: good. now we’ve heard that some parents spend time outside of the home and it may affect the way they raise their young children. Can you tell me about your experiences with this?**

R: as for the mothers. Now a days, mothers like to go from house to house to chat with other mothers or gossip with them.

**I: what else? What are the other reasons why some mother spend time outside their home?**

R: because they’re playing bingo or they’re gambling

R: some spend more time outside their home because they are smoking or chewing tobacco and they want to hide it.

**I: okay now can you tell me how these activities affect feeding their children under 2?**

R: these kind of doing can cause to harm to our children under 2. Like for example, they might get drown or get burn.

**I: what about how these activities affect the hygiene of their children under 2?**

R: when they chat or gossip with other woman, they forgot that they have to bath their kids

R: we forgot to wash our hands because we used coins to cover our cards after we played bingo.

**I: okay now what kinds of activities women usually do outside of their home?**

R: clean their backyards

R: washing clothes, cooking, go shopping

**I: what about men. What kinds of activities they usually do outside of their home?**

R: they go fishing, go to work

**I: are there any others beside these two?....... now for the last question. It’s says, we have heard from some people that they prefer to get health messages from the radio, others say from the newspaper. Can you describe for me the best ways to reach people with information on health in this community?**

R: I prefer doing community meeting maybe once in few months for reminding the mothers about health because some family can’t afford a radio.

**I: so, you are saying doing Outreach every month right?**

R: it can be not every months but may every after three months

**I: okay, what else?..... now can you tell me how usefulness of the internet for receiving health information? For example, Facebook.**

R: the good thing about internet is that it has all the information you need. The bad thing is not every household have access to internet.

**I: good. Now are there any community groups that could deliver health messages, for example women’s groups or mothers’ groups?**

R: it seems there are none

R: none. I think that is exactly what people in this community need to have; community group.

**I: That was great, we are done now. Thank you once again for your generous time and for sharing your thoughts with us. We greatly appreciate your help and we hope this research will help us improve the health of mothers and children in your community.**
