# Supplementary material for: Conceptualizing multi-level determinants of infant and young child nutrition in the Republic of Marshall Islands–a socio-ecological perspective
Source: PLOS Glob Public Health. 2022 Dec 19;2(12):e0001343. doi: 10.1371/journal.pgph.0001343 (PMC10022247; doi:10.1371/journal.pgph.0001343)
Supplement: S1 Data — (ZIP) [file pgph.0001343.s001.zip › RMI Supp Data/Focus groups data/F02U_Male_Rita_Aug 28_Belton_Shante.docx]

- **Interview code: F02U**
- **Interview type and interviewee: IDI MFG**
- **Interview date: Aug 27,2018**
- **Location: Rita**
- **Interviewer: Balton**
- **Transcriber: Shante**

**I: first of all Hi everyone. My name is Belton and I’m the son of Clot and Neni and I am also from here. (rita) I was raised here and went to elementary school all throughout my high school years. I used to walk pass here when school is over. I now most of the council man’s here at Rita. We all grew up together so that was good. But I’ve been gone for a long time, but I am back now. May we all get together and introduce our self?**

R: Anto

R: Jack

R: Nickson

R: DJ

R: Johnson

R: Allen

R: Tom

R: Edwin

R: Simeon

**I: Thank you all for cooperating and being here in this meeting. All the information we are going to talk about we will use it to help the mothers and children. These affects will cause our children to not grow healthy because they don’t have much nutrition in them. This program with UNICEF wants to know the leading causes for children Malnutrition. Our job is to find and prevent these causes. Some of these causes are from illnesses and food and drinks we feed our children. There is no wrong on your answers, everything you say I right… Now for our first question, how do we choose the food we eat in the morning, afternoon and evening? What makes us chose the food that we eat every day?**

R: Our incomes.

**I: Ok. Yeah. True. Our incomes is the biggest issue. If we had a big amount of incomes, we can buy good foods for our family. Is there any other? Other than our incomes? Is there any difficulties other then our incomes that it would make us not feed our children the food that we know aren’t good for them?**

R: Low budgets for vitamin. The food that has vitamin in them are expensive… that’s just my saying.

I: No. it’s ok. There’s no wrong on your answers because what we talk about will show up with **the people we are working with so that they can know about the problem we face so that they can help us. Like example they don’t have this because of this, they need this because of that. Something like that. Is good if we help them with our information because it’s like we help them, and they help us. Can you guys tell me why not enough incomes?**

R: Our work wages are low.

**I: Anything else?**

R: There is one more. We the people here are not willing to do gardening.

**I: Right**

R: Like banana’s, pumpkins and taro. All these things that grow here in our islands.

**I: Ok. Yeah. That’s right I see what your talking about. When I came here I was looking around and I only saw few banana trees and pandanus. Long time ago when I was young I remember seeing lots of banana trees and breadfruit trees here. Why is there bananas and breadfruit are in some places but not at some other places?**

R: One reason is that we are lazy to do the work. We don’t have enough space to do planting. Second reason is that we have no space for gardening and third is we are not from here. (meaning he’s from the outer islands).

**I: I want to know that what if there is no food where we are or at our home like not having a place to plant? How do we feed our family in situations like these?**

R: If it was I usually buy rich. Mackerel and can meats. Local foods from the store because I have no place to grow my own vegetables and things like that. The things I want to know if the foods from the store are good for our health or if It took time for the products to come here. And I might not know if they are expired because I don’t know how to read in English, or have they been in the store for a long time.

R: can you repeat the question again

**I: Oh. It says what does it cause for us to choose the food that we eat. Like why do we eat donut and donut? Not like the American’s because when they eat pancake, they eat it with eggs and bacon. But us we eat just bread with bread and donut with donut. What is the issue that makes us eat the food we eat and not with other foods? like on of you guys said because our incomes and the elder here said because of no space for planting. Is there any other reason we can choose the foods we eat from the foods we eat each day**?

R: 1 short of incomes. For me, especially here on Majuro. When the kids that goes to school comes to my home especially my wife from outer islands, they come and stay at my home because they don’t have any places to stay. When we eat all gather around and eat some of the food we eat are not healthy, but we eat them anyways because that is what we can afford, and I cannot afford good healthy foods for all my family members because there are a lot of us in my house. So, the reason why we eat unhealthy foods is because of low incomes and expensive good foods.

**I: How is work? Are there lots of jobs?**

R: there’s not enough jobs for each person

**I: Not enough? Ok. The information we know a lot about. But we are talking about it so that It can be heard on this recorder so that the people that are learning about us can know about our issues and report them to the higher ranks that are trying to help us. But ok. Thank you for your information. The people from where I work see that we buy too much can foods but not eat local foods. they are trying to figure out the issues on how we can’t eat planted food.**

R: Majuro is full of expensive things. And less space for gardening. We live on people lands and we are not allowed to do what ever we please to do on their land. We need their permission to do so.

**I: Why do we eat imported foods but not foods from our islands? Like fish… but we usually buy can foods.**

R: the people at the market selling the locals food give high price for them. Like for example breadfruit. They took them from near their houses and comes sell it for $5. It’s our island food but they make it so expensive. Lot’s of people would want to eat them but couldn’t because they can’t afford it.

**I: hmm… that’s good. Is there any other answers? Like we have taro and they are at Laura but why do we buy rice? All answers are right. No answers are wrong. Like can food. Why do we eat can foods?**

R: It’s faster do make them.

**I: is the can foods are cheaper then the local foods?**

R: can foods are expensive. Local foods are not expensive. Like for fish. We can just go fishing but we don’t have boats for that. But that just me saying…

**I: no. no. everything you guys say is good. Is there any other reason?**

R: The truth is there can’t be any fishing tools.

**I: some people here grow bananas, and once they bananas are fully ready to be eaten they sell them. Instead of giving it to their family to eat they sell them. The people I work with would like to know why we sell them instead of giving them to our families to eat them?**

R: for our needs.

**I: Can you explain that in more details?**

R: Like for some people, they sell their local foods because they want to buy themselves alcohol.

**I: is there any other reasons?**

R: R. Some sell for their families need like buying hotdogs, electricity bills and things like that. If there no fish well they might sell bananas and other local foods. Like if I were neeeded to go to the doctors I would have to have money for myself and pay my fee to the hospital. Especially for children because they are our priority. has a father or grandparent here, we have a hard struggle of living because money is the motive now days and without it, It’s hard for people who don’t have enough money.

**I: What other types of local food do they sell?**

R. Well there is bananas, pandanas and pumpkins. There actually pumpkin gardens around this village. People who own lands are the ones who have gardens at their place.

**I: there’s pumpkins around here?**

R: yeah. You see that house. Well over there you can see the pumpkins. You can only see gardens in the owners places. Some people houses too but not every house.

**I: Yeah. Those are the same reason the other focus groups I did also said. . Can you tell me about any difficulties that people have with regards to growing food?**

R. Well one of the main causes here is that there no rooms to plant seeds because the place we have here are too crowded and our soil is not that good for growing crops.

**I: is there any more? All your answers will be helpful to the people that will be helping us.**

R: Yes. There are some place to plant and garden but it’s been reserve to other residents.

**I: Oh ok. Is there any more other reason?... if there are no more then lets get on with the next the question**. **We’ve heard that some families boil their water for drinking and other do not. Can you explain why some people boil water and others don’t? what are the difference between these two? Boil water to drink and water we drink straight? Is there any water we boil before we drink?**

R:yes. There are water we boil before we drink.

**I: where does the water you boil come from?**

R: from the fauget or the water tank.

**I: just a question. Where do you guys get your waters from?**

R: from the bantoon. (water tanks)

**I: do you guys take water from the wells?**

R: no.

**I: is there any wells in this community?**

R: yes

**I: what is the use for the water well?**

R: for cleaning, washing dishes and bath**.**

**I: but drinking water usually comes from?**

R: bantoon. (water tanks)

**I: is there water that don’t need to be boiled but drink right away?**

R: yes

R: yes

**I: why do you guys drink it right away? Not I think it’s wrong but I’m just saying because at my house we have a water tank that we drink straight from but when is raining we pour it out and clean it.**

R: Yeah. We boiled our water when it’s not raining and when it’s raining we don’t boil our water but just drink straight out of the bantoon.

**I: yeah. We only boiled them when we take so long to clean the tanks**

R: that’s right.

**I: this other question is about how we wash our hands. One of the reason why the illness that we are working on and learning about our children it comes from not washing their hands and they get diarrhea from it. The reason why kids aren’t growing accordingly to their age is because when they have dairrhea they let out all the nurtition they ate and other then their body to receive the nutrtion they let them out. Also when they get dizzy they don’t want to eat and that will also make them not receive any nutrition. When do we wash our hands during the day?**

R: when we cook food or make food. But some ussually for get to wash their hands before they cook. But truthfully we should wash our hands when we are going to cook food.

**I: is there any other reasons why people should be cleaning their hands other then when they are going to cook?**

R: when we’re done doing our chores/works.

**I: when, during these times we use soap?**

R: before we touch the foods. that’s the time we usually use soap. And also wash our hands when we are about to feed the childrens.

**I: do you use soap other times you wash your hands?**

R: yes. When we’re done using the bathroom then we wash our hands with soap.

**I: you know, I’ve been gone from Majuro since 1990 I think. When I first got here, every where I go, like to the stores and the hospital. I see hand sanitizer. I was surprise becaues we didn’t do these back in my days and now it’s really nice… when did this happened?**

R: a long time ago.

**I: what the different between hand sanitizer and soap?**

R: it’s faster and better to use. And it kills all the germs. And it’s easy to use. Just pout it to your hands a done. (giggles).

**I. We’ve seen that some families keep chickens and other animsl enclosed in a fence while some don’t have fences for their animals. Can you tell me any difficulties to keeping chickens and animals in a fenced area? Can you tell me any difficulties to keeping chickens in a fence?**

R. You know we Marshallese we don’t really keep chickens fenced because there not really a casue to community and you know when children or people wants to eat them. They know better to ask first but children are known to steal our chicken and that’s okay too because they want to take it home and we older one know not to bother them because there only kids. We just remind the parents to come see us before eating the chicken.

**I. In some communities, we have heard that defecating in the open such as the beach side is common. Could you help us to understand this practice, what makes them defacate near the ocean side? What are the issues on why their doing it?**

R. sometimes when you really need to go but your house it too far then you just go to the lagoon side and defecate. Some in the outer islands you can just use the ocean side because there are no bathroom to use.

**I: hmm. Ok. is there anything else.**

R: yes. Manners. Like for example if we wear sitting here and the bathroom was close by and our mother wanted to use the bathroom, she will wait because she know’s that we are here and it’ll make her feel uncomfortable to use the restroom.

**I: you guys have very nice meanings to your answers… Ok next question is about the illnesses of our children under the age of 2. When the child is sick, who do we bring the child to first?**

R: we bring the child to the mother and she usually brings the child to the doctors.

**I: who among you guys has had his child brought to a old lady when their stomach is lumb?mor give them traditional medicines? Do you guys use traditional medicines?**

R: when children has fever and the fever gets higher, we bring them to the lady that knows how to massage the baby. Because children when they get fever for more then 3 days then they probably have stomach lump.

**I: is there any other illnesses other then fever and stomach lump that needs traditional medicines?**

R: mejatoto. (like posess, sometimes when fever is really high they get posess)

**I: is there anything else? What are other illnesses that children have that can make us give them traditional medicines? When I talk about marshallese medicines is like banannas and noni and all those other stuffs that makes a traditional medicines. What kinds of illness that will make parents give traditional medicines to them?**

R: kijon kan. (its when the baby is a few weeks/months old and they have things is there bodies) if they have kijon kan then they use tradition medicines to heal them. There’s a lot of kinds of sickness.

R: sometimes they don’t bring the child to the traditional healer and doctor but bring them to the priest so that he can pray for the child.

**I: Ok. thank you for your answers. We’ve heard that husbands are an important support for their wives during pregnancy. Can you explain what husbands do to support their wives while they are pregnant? What do they do regurlarly to help out there pregnant wives? This is our department. (Long pause) All answer is good. There is no wrong answer**. **The People I work with wants to know that’s why they are asking these questions.**

R: we man help our wives during pregnancy by juur jaki. ( don’t know what it means. Propbably a guy slang. But anways ask belton 😊)

R: it’s really hard to asnswer that.

R: sometimes we man should stay next to our wives because sometimes they can get possess when they are alone. And we also help with what they want to eat. it’s really different how women are pregnant here and the americans women. Women here sometimes don’t go to the hospital and they won’t have a problem. But american they always need to see the doctors.

R: some pregnant women want’s only their husbands to cook their food.

R: some women if they say they want to eat the fish near arno reef and you get her a fish from somewhere else, she would know that you didn’t bring the fish fromo where she wanted.

R: some eat soap. We don’t know why they eat soap but that’s what most of them like when they are pregnant.

R: we call their cravings Domestic Violence! (all laughing) becausing if we don’t get what they crave for they will hit us or do a lot of crazy things. (laughing)

R: sometimes they crave for the food that it’s hard to find or get.

**I: Ok. so we know that mothers take care of the children. What are the duties for fathers with their children and family?**

R: well we provide food for them, bring them breadfruit and grind the coconuts. The mother duties are to take care of the children and our duties are to provide food for them.

**I: ok thank. These information are very helpful… there are some kids that they’re parents are not with them. They usually stay with their aunts or grandparents. So, who usually take care of them?**

R: sometimes the relative of both the child’s parents or maybe the neigbors.

R: sometimes when the child is playing Near his friends house, and his friends families are about to eat, they usually invite him in to eat with them.

**I: who usully take care of a child that has n parents?**

R: sometimes their grandparents**.**

**I: How do they eat and who bath them and? Other then their grandparents? Do the neighbors take part on taking care of the child or?**

**R: yes**

R: yes

**I: can you give me an example.**

R: for me, when I’m not around, my neighbors sometimes give my grandchild a bath.

**I: what kinds of activites makes the mother not pay attention to their child?**

R: they gamble.

R: He said gamble because his wife is always gambling. (everyone laughing)

R: (giggles) bingo.

**I: Wow. Your right, that’s also something very knew to me. Everynight when I drive by lanai I see how crowded it is because lots of women go bingo… is there anything else? How about during the day time? What make a mother to leave the house and children during the day?**

R: sometimes when they cook and they ran out of something then that’s the time they go out and buy.

**I: that’s good. Anytning else.**

R: is when they go to their relatives house.

I: Okay. Now can you tell me the reasons why the fathers are not there to take care of their child during the day.?

R: Because we go to work.

R: sometimes we go fishing.

**I: Jobs. Fishing. Anything else?**

R: there’s also a thing that man now adays do during the day time until night time. Kava.

**I: is there anything the community do to help the children when the parents are gone?**

R: well in this community there is no play ground for the kids to play.

**I: where do you get your information on how to feed the child? Where do you usually get the information from?**

R: from their mother and father.

**I: is there any other you can think of other then the mother and father?**

R: from family planning.

R: from wellness.

**I: from hospital… so, if we were at the outer islands, where would you get the information from?**

R: from the radio station.

R: from our neighbors.

R: Sorry I have to leave because I have an appointment.

**I: No. it’s ok. thank you so much for joining.**

**I: is there anyone using facebook or internet? Well I guess this is our last question about our children that they wanted to know about. I wanted to thank you all for your time. All your information has helped us. We are trying to do all our best to help our children. Thank you guys so much and have a nice day.**

.
