# Supplementary material for: Conceptualizing multi-level determinants of infant and young child nutrition in the Republic of Marshall Islands–a socio-ecological perspective
Source: PLOS Glob Public Health. 2022 Dec 19;2(12):e0001343. doi: 10.1371/journal.pgph.0001343 (PMC10022247; doi:10.1371/journal.pgph.0001343)
Supplement: S1 Data — (ZIP) [file pgph.0001343.s001.zip › RMI Supp Data/Focus groups data/F03U_FGD_Female_Rita_Aug27_Libon.docx]

***Interview Code: FO3U***

***Interview type and Interviewee: FGD_ Female***

***Interview Date: August 27***

***Location: Rita***

***Interviewer: Libon***

***Transcribe: Meia***

**I: Can you tell me what a healthy woman’s body should look like?**

R1: What that you said?

**I: How can you tell a healthy woman body look like?**

R1: She is big.

**I: Okay, she said big.**

R2: she has good foods.

**I: she has good foods, what else? Now when you say big, how big is she? Does she have to be too big?**

R1: she not too big. (in between being thin and fat)

**I: She said not too big, delicious foods, good foods, what else?**

**I: What do you see when you look at a woman with an excellent body?**

R1: When we look at her appearance she looks healthy.

R3: They look clean.

**I: They look clean, What else? To ask another question, how can you picture a healthy person?**

R3: She doesn’t look unhealthy.

**I: Doesn’t look unhealthy, what else? Okay, question 2 it says, what is a healthy and nutritious diet for pregnant women?**

R3: Fruits

**I: Fruits**

R1: vegetables

**I: Vegetables, what else?**

R4: Local foods.

**I: Local foods, what about meats? What kind of meats that good for pregnant women?**

R1: Fish.

**I: Fish, is there any from there? What else is good for pregnant women? So, we said local foods, what kinds of local foods that goods for pregnant women?**

R4: Breadfruits.

**I: Breadfruits**

R1: Those things like pandanus, bananas,

R4: Taro

R1: Papayas

**I: So, are these foods different from your diet when you were not pregnant?**

R2: Yes. They are different.

**I: So, you mention, it different, can you tell me the different how is it different from when you were pregnant to when you were not pregnant? What different in these foods?**

R1: What was the question?

**I: Are these foods different from the foods you usually ate, we are talking about, these foods you guys mention are nutritious foods for pregnant women, and I ask if these are different from the foods that you eat usually ate. and you mention that these foods are different, now I know how are they different?**

R2: It different from local food, we are eating food from processing foods.

**I: Process foods? Like what?**

R2: chickens and,

R3: cow

**I: So, these foods are not good for the health of a pregnant women? Who or what influences women’s diet during pregnancy?**

R1: The doctors

**I: Doctor**

R1: Our mothers and fathers

**I: yes**

R3: it can be your partner

R1: Our husband

**I: Can you tell me how are these people influence you in your diets?**

R1: They told us not to eat foods that are not good for us.

**I: okay, how about from here, there are no wrong or right, we don’t have to be scare, we are here to find what best program for us, and we don’t have to get scare, there are no right or wrong, in your thought what are the influence of other during your pregnancy about your diets? Why did they told you to eat what they told you eat or food that are nutritious, why did the doctors or your father or mother or your partner told to eat these foods?**

R1: So, the baby can have a good health.

**I: uh-uh, what else? are there any other reasons?**

R3: if the baby born he/she won’t lack of vitamins.

**I: In some places, women are discouraged from eating certain foods during pregnancy. For example, in some countries, women are told that if they eat eggs during pregnancy, their child will become a thief. Can you describe any practices like that in this community?**

R1: They will become what?

**I: Thief.**

R1: Thief?

**I: hmm, if they eat egg during their pregnancy, if the child born and grow up will become thief, what about us? Base on ours believe?**

R2: None

R3: There are none.

**I: None? What about over here?........Okay, we know that some women receive supplements for low blood during pregnancy, like iron-folic acid (bill for blood). Some mothers told us they consumed all the supplements they were given during pregnancy, but others did not. Could you explain any reasons why some mothers do not consume all their supplements?**

R4: Nauseous, when they drink them they keep vomiting.

R4: They don’t like them.

**I: What else? Did you drink all your supplements?**

R5: Few of them.

**I: Why only few?**

R5: I feel nausea because of them.

**I: So, when you feel nausea?**

R5: I keep vomiting.

R1: Some can forget to take them.

R3: Forget to drink

**I: uh-uh**

R3: Some are lazy to take them.

**I: uh-uh**

R3: Like me (laugh)

**I: It good, it good that she been honest.**

R3: it not that we don’t want to take the medicine like before I use hate them (supplements), but when I heard what the doctor advice me I took its, but I am lazy to take them.

**I: uh-uh, yes there are some pregnant women like all women have differences in theirs first few months of their pregnancy. what helps some mothers to consume all their supplements?**

R3: Our husbands like force us to drink the medicine. If we don’t drink they like, “drink your medicine”. (laugh) and we don’t forget to drink and if we like don’t want to drink they told us to drink, we drink.

**I: okay, is there any way that you help yourself to drink these medicines?**

R1: If you eager to drink.

**I: uh-uh what about you Christy, you look like you are sleepy?**

R2: I am sleepy because of the wind. Every thing’s good.

**I: You didn’t have any problem with your supplements?**

**I: What are the consequences of having low blood during pregnancy and childbirth?**

R: They are weak, and when they try to give birth, they can’t because they don’t have enough blood.

**I: What about you two? What are the consequences of having low blood? What will happen to women who have low blood during childbirth?**

R5: As for me I don’t know because I haven’t had lack of blood.

R4: They will die.

**I: so, when you say die, can you explain more what you mean, who will die?**

R4: The mother

R3: The mother of the baby.

R3: Or it can be them both.

**I: Yes, those are the consequences of low blood, one of them or both will have something happen to them. Can you tell me if you have received advice from health workers to prevent/treat low blood? What did they usually say?**

R5: They told us to drink medicine.

R1: Drink medicine for blood.

**I: yes.**

R3: Eat a lot of foods that a lot of iron in it.

R4: Drink v8

**I: They also told us to drink v8.**

R3: Eat sashimi.

**I: Eat sashimi. Thing that have a lot of blood in it. Is it raining?**

R1: so, what we have our own tent? You can move to here.

**I: That okay I don’t feel the rain. Now it says, once the baby is born and you began breastfeeding, can you describe a healthy and nutritious diet for women who are breastfeeding?**

R4: Fish

**I: Fish, what else?**

R1: Foods that are right for them to eat.

R5: Fruits

**I: Fruits, what else?**

R1: Food that are for energy, those energy foods**.**

**I: So, when you say foods for energy, what kinds?**

R1: Rice and chickens.

**I: That good, what else? was there something else they told you to eat during when you were breastfeeding?**

R2: Corn beef (canned meat)

**I: Corn beef what else? is this the only canned meat they told you to eat?**

R2: All kinds of canned meat, mackerel.

**I: Similar to, what we discussed earlier about “food taboos” during pregnancy, can you describe any “food taboos” that exits for women who are breastfeeding? Like I said before like in other places when women eat egg during their pregnancy the child going to born thief, what about you during your breastfeeding what will happen? What did you believe?**

R1: For corn beef, if we eat corn beef we will have breastmilk.

**I: What else? what about sashimi?**

R3: And sashimi

R2: Not sashimi

R1: Sashimi make blood.

R4: The baby will bite our nipple.

**I: Yes, our believed is that if we ate sashimi, “oh don’t eat sashimi because the baby will bite your nipple”.**

R2: uh-uh-uh

**I: is there any other believe other than sashimi? This is the only thing with sashimi?**

R1: Uh-uh

**I: What advice have you heard from health workers about breastfeeding? What did they teach about breastfeed?**

R1: About breastfeeding, they told us to breastfeed, so the child won’t get sick.

**I: Yes**

R1: If they have diarrhea, we breastfeed them.

**I: uh-uh, what else? The words that the nurse told you, when they told you about breastfeed?**

R5: They said not to lay down while breastfeed our child.

**I: Why is it not good to lay down while breastfeeding?**

R5: The child will choke.

**I: Good, what else?**

R3: They might die.

**I: Yes, because you might not know that you block his nose. What else?........ Can we move inside? Just bring your chairs. IS there any advice from family members about breastfeeding? From the mother, grandmother, aunties or the husband about breastfeeding.**

R4: Eat a lot so we can have breastmilk.

**I: There is one, what else?**

R4: Don’t go out at night for the child will mejatoto (possessive by spirit).

**I: That one of ours believe. What else?**

R1: My mom told me not to lay down while breastfeeding.

**I: Don’t lay down again while breastfeeding. We heard some mothers first introduce foods other than breast milk when their baby is 6 months old, while some introduce foods earlier or later than 6 months. Could you describe the reasons why some mothers introduce foods or liquids earlier than 6 months of age?**

R1: Because we breastfeed but the baby keep cry.

**I: So, not enough breastmilk.**

**Need assistance from the interviewer.26;40-44**

**I: uh- uh what else?....... it like you guys are afraid?**

R4: It time for the baby to eat.

**I: Yes, you said not enough breastmilk, it time for the baby to eat. are there any other reasons?**

R1: crying a lot.

**I: ay?**

R1: They can cry a lot

**I: They can cry a lot? What the doctor? Did the doctors say?...**

(Then the mother and children are talking nonsense in the background)

**I: Can you tell me why did some women feed their baby before 6 months?**

R5: Some mother forces their baby to eat.

**I: uh-uh**

R1: Some feed their baby because they cannot breast feed their kids with pain in their breast.

**I: what else?....... What about after 6 months, why didn’t they feed their child at 6 months, but they waited after 6 months to feed their child?**

R3: For eating after 6 months, now they say that for the baby not to have bigger gizzard.

**I: During their growing up? When they have bigger gizzard what happen to them?**

R1: They keep getting hungry.

**I: Keeping getting hungry. Many mothers have told us that they did not have enough breastmilk to feed their child. Can you explain to me how children under 6 months are fed when their mothers do not have enough breastmilk?**

R3: Bottle

**I: Bottle, feeding them, what else? are there any other things you guys are doing?......is there any advice or ways to increase breastmilk?**

R1: Eat a lot of fish.

R4: Eat a lot.

**I: Christy? Eat a lot fish, what else?**

R1: Corn beef

**I: But where did these advices come?**

R1: From doctors

R3: Doctors

**I: Doctors**

R4: Our mom and dad.

R3: Our dad and mom.

R1: From the school

**I: From the school, good. Could you describe for me how mothers in this community know that it is time to stop breastfeeding their child? How do you guys know that it time for your baby to breastfeed?**

R3: We follow the yellow card. (yellow card is a yellow paper that the doctor or nurse gave you before you discharge from the hospital after giving birth to your baby in which all the information about the child’s vaccines and weight can be put in when ever the child needs to come back).

**I: you follow the yellow card, what else?**

R4: Some just stop feed from the breast, they like don’t the breastmilk anymore.

**I: They don’t like it? Are there any more reasons?**

R3: Some mothers put tabasco in the breast to stop breastfeed (laugh)

**I: Yes, so they stop feed from the breast.**

R4: about make it sour?

**I: uh-uh, ohh now why did they want to do that? Why did want the baby to stop from breastfeed?**

R2:old enough, they said they are old enough.

**I: they said they are old?**

R1: They said they are old. But some (need assistance from the interviewer 34;20-26)

**I: Can you describe why some people feed young children balanced diet? What are balance diets?**

R1: What was your question again?

**I: Can you describe oh it says some people said they feed their children balance diet, what is, how are the balance diet? What it mean?**

R1: We give the food that are balance diet?

**I: uh-uh**

R1: foods like fruits

**I: fruits**

R1: vegetables

**I: vegetables**

R4: What was the question again?

**I: It says what is the meaning of balance diet? What is balance diet?...... she says fruit, vegetables what else?**

R3: What they usually eat.

**I: Baby foods**

R5: oatmeal.

**I: oatmeal.**

R4: juices

**I: juices, yes now what kind of juices?**

R4: Orange juice

**I: orange juice, what else?**

R3: apple juice

**I: apple juice yes, these kinds. What about local foods? What kind of local foods?**

R1: Bananas, pumpkin

**I: Bananas, pumpkin**

R3: Makwon (pandanus juice)

R4: Makwon

R1: Breadfruit

**I: Breadfruit. Now, for the last question about feeding children we’d like to learn about how decisions are made. Can you explain anything that influences mother’s decisions about feeding their young children in this community? who influence you to exclusively breastfeed?**

R1: Our mothers

R4: The doctors.

**I: The doctors……….. who else?........How did they influence you for you to exclusively breastfeeding? What word did they say to influence you?**

R1: Our mother use to said breast feed exclusively for the baby not to (asked the interviewer pls.)

**I: For them not to get sick more often, right?......... what else?..........When would like to ask a few questions about children when they are sick. It says, when children under 2 get sick, some parents take their children to the doctor first and others use traditional healing first. Can you describe the reasons for this difference?**

R4: The child heals quickly.

**I: When you what? When you bring to the doctors or the traditional healer?**

R3: Traditional healer.

**I: Traditional healer? Okay.**

Need assistance from interviewer40-41;11

**I: What else? what is the differences between these things? Going to see the doctors or the traditional healers?**

R1: It is quicker to go to the traditional healer, but if you take the baby to the doctors it take time for the baby to heal, but when you took the baby to a healer to massage, the baby heal fast.

**I: What else? what about if they take the baby to the doctors?**

R1: We take them to the doctor when the healer cannot heal them.

**I: oh, you take them after they cannot heal from the healer to see the doctors, but some people take their baby first to see doctors……… What illnesses are commonly treated with traditional medicine?**

R1: Medicine for komejatoto (mejatoto is when you possess by demon, so the use medicine to release you from the demon).

R4: Let them drink banana.

**I: When they are what? Why did they let the child drink banana?**

R1: They have stomach pump or cough

R4: stomach pump

R3: Diarrhea

**I: What traditional medicines are used for each illness? Illness like stomach pump? What did they do?**

R4: How did you say it again?

**I: I said what traditional medicine are used for each illness like if the baby had stomach pump? If it were cough? What did they do? What kind of medicine are use?**

R4: There are many kinds of medicine. (again need the interviewer assistance).

**I: What about if they have stomach pump?**

R4: Drink banana

**I: Banana……But who influences whether traditional medicine is used?**

R3: Sometimes our grandmother

**I: Our grandmother or mother?......... Can you describe how children are fed when they are sick compared to when they are not sick? Difference of how they eat when they are sick compare to when they are not sick?**

R3: When they are sick they don’t want to eat.

**I: They hate foods? What else?......... Is there any other?**

R1: When they are not sick they don’t hate foods.

**I: They eat a lot? What else? when a child is sick is it difference how they eat? And what is the difference?**

R3: They usually eat, or there are foods they eat and there are some they don’t want to eat.

**I: uh-hmm, what else?.....What foods are given to the child and the amount of the foods they are eating? (she repeated the questions)**

R1: For the baby?

**I: For the children.**

R4: Orange, apple

**I: Orange, apple,….what else? any more foods? About the children that are not sick what food are given to them?**

R4: All kind of food.

**I: all kind of food? What to drink? What drink are given to them?**

R4: water

R1: orange

R3: orange juice

**I: orange juice? What drink are given to the children who are not sick, and the amount of drink given to them? What kind of drink are given to the children who are not sick?**

R3: Children that are not sick?

**I: Like when they are not sick they eat all kind of foods?**

R4: All kind of drinks

**I: All kind of drink too? Now we would like to learn about the foods that you provide for your family, could you talk about what influences which foods people in this community provide for their families? Food they usually provide for their families?**

R1: Rice and banana

R3: Rice, banana, case of chicken

R4: canned meat, hotdog,

**I: We won’t escape from hotdog it for the children (laugh) what else?**

R1: Sashimi

**I: Sashimi, What are difficulties in getting the foods they want for their families?**

R3: No money

R1: Not enough money.

**I: uh-uh**

R1: Too expensive

R4: Foods are too expensive

**I: What else? Now how did your families deal with food shortages, for example sharing or buying food from the shop? When we food shortage it comes with food that we give each other, what did you go and buy?**

R3: Food that are cheap.

**I: yes**

R1: Food that are .25 cents cost.

**I: It .25 cent cost but is it already cook?**

R1: It already cook

**I: About food share? We share food when we don’t have enough?**

R4: No

**I: Many families have told us that fresh vegetables are not affordable. Can you describe other reasons that families do not eat many fresh vegetables? Why can’t they buy vegetables?**

R3: They are not employee.

**I: They are not employee,**

R1: They don’t want to

**I: They don’t want to, what else?.....but is it cheap?**

R1: What

**I: Is vegetables cheap? They might not buy them because it too expensive. In the next section, we would like to talk about water and hygiene, can you please describe how people typically get water for their families in this community? How do we get water?**

R4: One more time?

**I: How did you get water? How did you get water in your house?**

R3: rain and we fill up.

**I: What else?**

R3: Help

R2: We buy water.

**I: Buy water.**

R1: Fill up when the water from government open.

**I: Yes, that good, are these houses use well**

R1: Some house has well

**I: Some has? So, when it rain, it goes to your water catchment, and you don’t have water for drinking, for drinking only, so where else you get water?**

R1: For only drinking water?

**I: Water for drinking, water for showering, water for wash**

R1: From the water company

**I: The water company, we need to dig well. Now it says, what are the difficulties to have water?**

R1: Not enough water catchment

**I: Not enough water catchment, yes, when we don’t have enough water catchment. What else?**

R3: lazy

**I: we are lazy, we are lazy to go fill up our water container. What else?**

R5: What did you say? What are the difficulties?

**I: What are the difficulties to have or find water? We already say not enough water catchment, to lazy to fill up the water container.**

R5: if we won’t take care of our water catchment.

**I: if we don’t take care, what else?.... Any other reason for us to have or find water?**

R4: Not enough money.

**I: Not enough money, but any difficulties about storing water?**

R3: None.

**I: None? So, can you explain why you said there isn’t any difficulties about storing water?**

R3: When we really care of our water we don’t use it for any other reason except drinking and then clean it all the times.

**I: Is there any, what about**

R1: Any things else?

**I: The difficulties of storing water? The thing you said before.**

R1: oh, right not enough water catchment.

**I: yes, when it not enough, hmm, it difficult to store because water catchments are not enough. What else? Are there any more? We’ve heard that some families boil for drinking and others do not. Can you explain why some people boil their water and others do not?**

R4: Some people are lazy to boil their water.

**I: uh-uh what else?**

R3: Boil so we don’t get sick.

**I: yes,…….. did you always boil your water?**

R2: I don’t.

**I: Why didn’t you boil?**

R4: she is not because her husband bought her water, from his working place

R3: we buy water.

R2; My husband bring water from his working place. His working place also sell water.

**I: Where is that?**

R2: Lanai

**I: And he is always bringing water from there?**

R1: For some people they boil their water because cannot afford to buy their water from the store, but don’t drink from the water they have they boil it, so they won’t get sick.

**I: So, that why they boil water because there are no money to buy water from the store. What are the difficulties to keep our water catchment system clean?**

R4: None

**I: So, that mean your water catchment is clean every time?**

R4: Some time it get dirty

**I: What makes it dirty?**

R4: Dust

R3: Dust on the roof

R2: Cats

**I: Animals, dust**

R3: Dust from the car

R1: Dust from the roof comes down to our water catchment

**I: About flowers**

R3: the flowers

**I: When there are flower on the roof did it dirtying our water?**

R2: yes

**I: We’ve heard some families wash hands regularly while others do not. Can you explain some reasons for this difference? Explain why some wash and some don’t wash their hand all the times? Why did some families wash their hand?**

R2: For when they eat they with clean

**I: Cleanliness**

R4: For them not to have germs

**I: won’t have germs**

R3: For them not to get sick.

**I: Not get sick. So, when you said sick, what kind of sickness?**

R3: Diarrhea

**I: Diarrhea**

R4: Stomach ache

**I: Now, why some did not wash their hands?**

R1: Forget

R2: some lazy

**I: some lazy, forget**

R1: Not use to

R4: in a hurry

**I: not use to, in a hurry. Okay, so now why is it that some people wash their hand with soap and water, but some don’t?**

R3: some use only soap

R1: Some use only water for they are too lazy to get the soap
**I: What else? but what is the main reason why you wash your hand with soap and water all the time? Why is it important for you to wash your hand with soap and water all the time?**

R1: to be clean

R3: not to get sick

R4: not to have stomach ache

**I: not to get sick, not to have stomach ache. Now, why did some people use hand sanitizer instead of soap?**

R2: To kill germs

**I: To kill germs, what else?**

R1: it faster

**I: It faster?**

R1: sometimes we are in hurry.

**I: (laugh) Now for the last question, we would like to learn about how parents care for their children. we’ve heard that husband are an important support for their wives during pregnancy. Can you explain what husbands do to support their wives they are pregnant?**

Need assistance from the interviewer1;02;41-3

R4: Provide our need

R3: When we want some thing but it far they are the one who bring it.

**I: What mothers or other member of the family do to support their daughter while they are pregnant?**

R5: doing chores

**I: Doing chores for you, cook your food, what else?**

R4: When we are pregnant?

**I: yes**

R4: They usually told us to move around

**I: Okay, good they give you advice, what else?.... We are also interested in learning about how caregivers play with children under 2 years. Can you describe for me in detail how you play with children? how did you play with your child?**

R1: We can bring toys

**I: To play with them with it. What else?**

R4: Teach them

**I: Yes, what if they were 6 months old, how would you play with them?**

R4: sing for them

**I: sing for them what else?**

R5: Talk to them

**I: Talk to them, communicate with them**

R1: Make them laugh

**I: tickle them. We’ve heard that some parents spend time outside of their home and it may affect the way they raise their young children. can you tell me about your experiences with this? It for those mothers that are always gone from their children?**

R5: The are having Trouble

**I: Having trouble, what else?**

R3: They are like don’t spend their time with their kids because they are doing what they want more.

R1: What they want it more important to them

**I: What they want more? Now what the want more outside their home?**

R1: Gossiping

R4: Look for lice.

**I: How these activities affect feeding their children under 2? Just like you said they have trouble how will that affect the child**

R1: They will have problem

**I: Maybe they are argue while the child is with them.**

R3: They might not finish their education.

**I: What the child under 2 years. What will they do?**

R3: They will cry a lot, they might be sad

**I: They get sad too. How these activities affect the hygiene of their children under 2?**

R4: Stay near

**I: But now they are not with them**

R4: Their family, their grand parents.

**I: How will the child be clean, it affect their clean like their diaper are wet and nobody clean, now what will affect? When nobody clean the child will the child be clean?**

R1: No

**I: Now the question is asking how will it affect the child cleanliness?**

R1: Their cleanliness will gone.

**I: uh-hmm, what cleanliness? Like what? Wet diapers what else?**

R3: Their hair

R4: skin infections

**I: skin infections, that one of them, if they are not clean there are things appear in their skin, now, what kinds of activities women are doing outside of the home? Why are they not at home?**

R4: They went gossiping somewhere

**I: They went gossiping**

R4: some are gambling.

**I: Gamble**

R4: Bingo

**I: Bingo, What the father what did they do?**

R1: Drinking number 1

R4: Drinking

R4: chew some where

R3: When they what?

R4: Baseball

**I: When they are not at home, some drink, baseball, what else?**

R1: They look for other women

Laugh

**I: So, they are gone from looking after the baby. We have heard from some people that they prefer to get health messages from the radio, others say from the newspaper. Can you describe for me the best ways to reach people with information on health in this community? other than radio and newspaper.**

R4: come to them

R1: Come and make

**I: You can say come and do out reach in this community, okay, go to them what else?....what are the usefulness of the internet for receiving health information (e.g facebook) why is facebook good get these information.**

R1; When we want to reach our family it easy

**I: What about health**

R4: it is easy to look it up at facebook and search it

**I: oh it easy to search it and look it up at face book and internet. Is there any community group that could be a good place to deliver health messages for example women’s group or mother’s group? Is there any group in this community?**

R2: What group

**I: Any group?**

R2: wellness group, the women in this community

**I: okay that one they might share about health it not be only about food, what about mother group, no more**

R3: what?

**I: Group for mother, no more? But did you guys want to have like a mother group? No, okay thank you guys…**

**The end….**
