# Supplementary material for: Conceptualizing multi-level determinants of infant and young child nutrition in the Republic of Marshall Islands–a socio-ecological perspective
Source: PLOS Glob Public Health. 2022 Dec 19;2(12):e0001343. doi: 10.1371/journal.pgph.0001343 (PMC10022247; doi:10.1371/journal.pgph.0001343)
Supplement: S1 Data — (ZIP) [file pgph.0001343.s001.zip › RMI Supp Data/Focus groups data/F04U_FGD_Male_Rita_Aug 28_Balton.docx]

- Interview Code: F04D
- Interview type and Interviewee: FGD Male
- Interview Date: 08/28/18
- Location: Rita
- Interviewer: Balton
- Transcriber: Cendaniel Milne

R1: the fact is, I eat chicken, and I eat rice, those things ham. When getting paid.

**I: can you repeat it, because the answer must not come from me. It must come from you guys, for the consultants that I am working with. How do choose your food? How do you explain it?**

R1: that’s right, that’s right. Thank you for asking, that’s what I mentioned. I said I don’t have a vitamin for buying these things. The fact is, I buy hotdogs. And then buy these things.

**I: because of budget right?**

R2: we buy depends on budget.

**I: depends on budget?**

R1: yes. And during the times I get paid, I usually buy a case of chicken those things because I have money.

**I: during your pay day?**

R1: yes.

**I: perfect. There are places that we visit, families eat local foods but other eat foods from the stores. The question the consultants would like to know.**

R3: the answer is, there is not enough local food now a days.

R1: yes, yes, that’s it.

**I: this is the reason.**

R3: there is no place for local foods.

R2: when there is, they have a high price.

R1: yes, yes. There it is.

**I: Marshallese food?**

R3: our ancestor they were tall and huge, because they ate local foods only from morning to evening. Can you eat local foods from morning to evening? And for years and years?

**I: what are the reasons?**

R: because this is a center (urban area)

Both R: (chattering)

R: when you go to outer Islands, you won’t see this case (referring to local food). Correct?

**I: correct.**

R: the reason why they were tall and huge because they had large land to farm on.

R: yes, everyday eating, eating, eating.

R: these places, there is no places to farm. Center there is no place for food (local food) eating only flour and rice. Outer Islands, you won’t see that kind of type you are saying.

**I: thank you.**

R: do our answers are correct?

**I: your answers are correct. Those are perfect.**

R1: tell us your question so we can answer them.

**I: as of now, families, it has been answered why some families can eat the other food; because they can afford the price. But, those who eat canned foods from the stores?**

R: I have a question. I have a question.

R: many people here on Island mostly eat local foods, those who have their own land they can do gardening.

R: yes, yes.

R: like us, that comes from outer Islands we can’t do gardening because we don’t have our own land.

R: we eat rice and hotdog.

**I: things that are cheap?**

R: yes.

**I: the reason why we eat canned foods because they’re cheap?**

R: correct.

R: you’re correct from what you’re saying.

**I: Thank you, there are few that are, like those from Laura and other places here on the DUD. They take these things that they grew and sell them. The question states that apart from eating these things they grow, what makes them to sell them?**

R: what does it says? What does it say?

R: they don’t have any money.

R: what does it says?

**I: apart from eating the foods they grow, why do they sell them? Why do they sell them?**

R: that’s correct, there is no money.

**I: they just sell them?**

R: I said there’s no money. The way that they are doing, they pawn. Is it true?

R: or they make money for students.

R: yes.

**I: for the family’s need?**

R: yes.

R: same as what the fishermen do. When they go fishing, they will give some of their catch to their families and some they will sell them, so they can buy the needs of their family.

R: yes that’s right

**I: great. The answers in the question above, they say don’t grow food at their homes. Because houses are crowded, there is no place.**

R: well, let me say this to you. Like for our homes, there is no place to farm because it’s crowded.

**I: same goes for Rita, we came across this type of situation. Even lot more in this DUD. Are there any ways to grow food without the need of larger space?**

R: It can’t.

**I: it’s hard right?**

R: it can’t.

**I: apart from being crowded, what other difficulties that make people not to…..**

R: children that are short or by the time that a woman is pregnant. She should have eat body building food.

R: yes.

R: but instead, they eat tuna, spam, corned beef, and those things.

R: things that don’t have any vitamins in them.

R: they should have eaten body building food.

R: hot dog, yes, hot dog.

R: so the baby would have enough vitamins.

R: that’s correct.

**I: yes.**

R: when they eat. They eat unhealthy food

R: yes, foods that don’t have any nutrients in them.

R: and most of all those snack food they eat which are not necessary.

R: maybe that’s where the thing first starts; from a pregnant woman. It can’t be from the time the baby is born. By the time the baby is born, they see that the mother is the one who cause the problem.

R: yeah that’s where that kind of problem first happened from

**I: good, these answers are perfect.**

R: how’s it? Do we answer well?

**I: this next question, is about water and hygiene. The way how the children and the family clean.**

R: you’re giving regarding water?

**I: now it’s about water.**

R: well, now I am saying this to you. I have a question, I have a question.

**I: proceed.**

R: why do you say regarding water? We don’t know if the waters in our water catchments are safe and our children are drinking from them?

**I: I do not know, this is why I came to.**

R: well me too, I do not know.

**I: this question states that there are some families do boil their waters, and others do not. Which waters are boiled and which we go for drink?**

R: well.

R: the waters that are boiled are the water from the well and some boiled the government water.

R: yes, from MWSC (MAJURO WATER SEWER COMPANY)

R: But those that are not boiled are the ones from the water tank.

R: yes, straight from the tanks.

**I: these are the waters do boil?**

R: no

**I: oh just go for drink**

R: they go for drink and also the water from pacific pure water company.

R: well, our babies. For me, it’s true! I boil water for them, for me I just drink. How’s that for your question?

R: if it’s a bottle like this, well they’ll drink without boiling it. Are you talking about a child or an adult or family?

**I: family.**

R: family? Well family, as for my family we drink without boiling.

R: you know family? I have a question, I said family.

R: they drink with each other, I don’t give my child water from the water catchment. Only drinks from the pacific pure water.

**I: from bottle right?**

R: bottle or gallon, all it matters if it’s pacific pure water.

**I: okay.**

R: for me, for me, for me. My young girl, I boil water for her. I drink from the water catchments, but for my little girl, I boil her water.

R: as you know water, they’re different from each other. When you boil a water, you drink and become thirstier, thirstier, and thirstier and there are no nutrients in the boiling water than the water that comes from the water catchment.

R: yes. He’s right, he’s right. Because it comes from the sky, as they say it’s a gift from God.

R: as you see in this water bottle, if you store it for 1 month there will be no green algae in it. But if you fill water from the water catchment, there will be green algae, it’s still moving. this water bottle, the cells are dead.

**I: how do we keep water catchment system clean?**

R: pour it out and clean it inside.

**I: eh?**

R: pour it out and clean it inside.

**I: during the times that rains? Or any times?**

R: maybe every after 6 months

R: when I clean my water catchment, I know the meaning. I can ask a question, you know these things. I tell you, you pour a Clorox. A little spill, pour it and pine sol it. Mix it, mix it, and then pour out the water after. Then you clean it again, and you drink. Oh! I know because I clean the water catchment at home.

R: why what’s wrong? Why are you asking about water?

**I: eh? Because water, children also get diarrhea from it. Because it one of their targets on why many children**

R: getting more sick.

**I: yes.**

R: yes, he’s right.

**I: they want to know because they’re finding a way to help.**

R: about that, they should have, because on the roof tops. As you can see when it last 3 days in a week, there are dust all over the roof tops. And people don’t take out the tanks and wait.

R: to drain it out.

R: yes, to drain it out.

R: when it rains, drain it out first. and when it’s clean then they put it back on again. This is where diarrhea comes from peoples’ homes.

R: they don’t clean their roof tops.

R: when the rain starts, if you put a bucket on the edge of the rooftops. Muds, it looks like a milk. You need to take it out first.

**I: and let it spill out first.**

R: after an hour, then you put it back. The answer for it, the reason why they’re having diarrhea, they don’t take out the tanks before the water comes on the roofs because it’s muddy. The roof tops are dusty, this is where it makes them diarrhea.

**I: yes, wow. Perfect.**

R: let’s say when we did (inappropriate word) inside the water catchment we didn’t. On the roof tops, when you look at it. You think it’s clean, but why do the children have diarrhea. Is it because they don’t take out the tanks first and let it clean itself for an hour.

R: yes, he’s right.

R: wait at least an hour

R: and they do not clean their roof tops.

R: it doesn’t matter whether we clean it, the most thing is take it out and wait for an hour

**I: during the time it rains?**

R: so you are saying taking the tank out?

R: when you put a bucket at that time, milk.

R: well, he’s right. Dirty, so dirty.

R: plus, splashes of salts from the ocean side.

R: yes. So, what else?

**I: perfect.**

R: give another information.

**I: the other thing states that some families wash hands regularly while others do not. Like for cooking, taking care of the children, or going out. What are the things that someone would not, like what. What are things that makes us wash our hands and what are the things…**

R: you know, you know, you the meaning. I have a question, washing hands?

**I: yes, why do other washing their hands before working, but other do not?**

R: correct, correct. Correct, your question is correct.

R: the thing they say.

R: because they lack sanitation.

R: yes.

**I: eh?**

R: when we, let me say this to you eh? Let me say this to you doctor, let me say this to you. I said, if it was me, right? I went, for example. Example, this is an example I am telling you. I go and hold my daughter, I need to wash my hand first. And you know (English version: you know) after, I can hold her.

**I: wash your hands first**

R: yes.

**I: are there any other things besides washing hands before taking care of the children?**

R: brother eh? Can you share your answers?

R: like what man, what do we say?

**I: like…what ways, what times do we wash our hands?**

R: that’s the question?

**I: yes.**

R: in what times?

**I: what times do we wash our hands?**

R: during the times that are breakfast, lunch, and dinner. Is it correct?

R: hey, don’t do that.

**I: can you repeat the answers?**

R: before you make food and eating a food.

R: yes, that’s correct. When preparing meats, you have to wash your hands. When cooking a rice, you need wash your hands.

**I: what makes someone, instead of washing hands but doing other works? What are the reasons?**

R: I don’t know because when I cook, I cook.

R: well, I have a question. Your question is perfect, I am interested in. what were you saying? What were you saying?

**I: what makes someone not to wash their hands?**

R: well.

**I: well, that’s the question.**

R: they are not asking you man! They are asking everyone.

R: just answer man!

R: that’s the question? Just ask why and move on? Eh?

**I: no, I meant by why don’t they wash their hands?**

R: where?

**I: why do people do their works, but they don’t wash their hands?**

R: because they lack sanitation, others may tired and.

R: Georgeton, Georgeton answer.

**I: that would be the fact.**

R: sometimes when they get tired, they planned to wash their hands before eat. But they eat and take nap a little and passed out.

R: I have a question, I have a question. You when you come back, are you exhausted? That’s it. You don’t wash your hands. Is it correct? Is it correct? But you eat. Is it correct? Is my answer accurate for your question? Well, then that’s it. When you come from work, feeling exhausted right? You just go for the food and eat.

**I: I know these answers, but it’s not me that I am seeking the answers. It’s for the consultants that I am working with.**

R: well, that’s another answer.

**I: well, they want to know why it is.**

R: how many??

**I: during the times that we wash our hands. What times do we use soap when we wash our hands?**

R: when we eat.

R: when you eat, and after using the bathroom where you’re heading to the kitchen. That’s the time where you use soap.

R: I said, we soap our hands before we eat. And take a shower, those things. Those things that you’re talking about soap…. Is it correct?

R: first, soap our hands before eating. And after eating you wash your hands again. After doing works, wash your hands too. And those that are not, maybe they lack sanitation.

R: yes.

R: or maybe lazy.

R: yes, yes, it’s true… well, there are those who does that, they do that.

**I: in other places you see animals roaming around. Pigs and chickens.**

R: animals

**I: but others are in fences, they’ve been put inside fences. Like for chickens, those chickens that are not in fences. Why do people let others to be roaming, but other don’t?**

R: Georgeton you may proceed. The answer seems to be difficult.

R: only owners do such thing.

**I: that’s it, can you repeat that again?**

R: only owners do it.

**I: I said, others let their chickens roam free on the environment. But others do put them inside caged fence.**

R: the question seems to be difficult.

**I: because of what? Because of some people……?**

R: well you know!

R: the question is hard man, Warrar (a term for expression of amazed) we can’t answer it, because only the Alap (land lords) can answer it. The question is hard.

**I: the other question is, it’s been shown at my place and everywhere on Majuro. Why do people defecate ocean and lagoon side? What makes people to defecate ocean and lagoon side to these days?**

R: well, there are those that come from outer Islands. It’s a habit that they have. There are others that hate using the toilet.

R: likely to be, I have a question, I have a question. Brother.

R: they get used to it since they were young.

R: yes, yes. True, true, true.

R: urban people don’t.

R: you know us, we people of this land, like our pacific that we live in. we have the habit of using the toilet, but the outer Islanders, they use ocean and lagoon side.

R: others don’t have.

R: yes, it’s a habit they have.

R: other people don’t have.

**I: other people don’t have?**

R: yes.

R: other people don’t have a toilet

**I: they don’t have a choice? Is there a difficulty for?**

R: there are those that lived with many families, if there are many people living there. Well.

R: yes, yes. He’s right.

R: but there is only one toilet … when there are many people.

**I: one toilet place for many people?**

R: and the children often, they let the children do that.

R: he’s right, he’s right about.

**I: yes, it is a fact.**

R: it’s really a tragic situation but what can we do.

R: yes, because they don’t have any bathroom.

**I: are there any difficulties of making any bathroom? What are the difficulties?**

R: there are no difficulties in building a bathroom, because I am telling you. It’s because they don’t have any money, they really, like what, they.

R: but there are some Alaps that if you don’t pay them for building a new bathroom.

R: yes.

R: then you won’t make a bathroom. … like for the Utrikan (village name) that’s how it is when I lived there.

R: he’s right.

R: you need to pay the Alap (land lord) to make another bathroom for the house.

**I: thank you for your answer, the other thing that we would like to know is what things prevent a family from build a.**

R: and you know you have to dig the ground for the water pipe that comes from the government water for your bathroom and you know, you need the Alap’s permission too.

R: correct, correct.

R: and it’s not a small price to pay.

R: yes, correct. Correct. Correct.

R: and people do depend on budget.

**I: budget too right?**

R: but when there are many family members in a household, if they do it together, it will be an easy work. but when they separate from each other, then the budget is also separated.

**I: thank you.**

R: how is it, there’s no more right? Give it, more answers.

**I: now it’s done about water and hygiene, now its children’s illnesses.**

R: okay.

**I: as you know the time when children are ill, who is the first for the child to be brought to? Before you, who is the first that we see? The child is sick, who is the first to take information.**

R: well, I have a question.

**I: is checking the illness.**

R: I have a question.

**I: what will do to him/her?**

R: I have a question.

R: the mother of the child.

R: I’ll proceed on with the question

**I: the child’s mother right?**

R: I’ll proceed on.

R: or the grandfather or the grandmother or the father.

R: I’ll proceed on, for me. My daughter always get sick. We both, the woman and I bring her to the hospital.

**I: hospital? You take her to the hospital first?**

R: yes and see a doctor.

**I: but you mentioned, the mother and the grandmother. why?**

R: because… it is … it is… as for my daughter right. Those times where she’s burning and recover, burning and recover. They said we can bring her to the traditional healer to see whether she have internal injury (for children). And sometimes they said, bring it to the hospital to check if she has illness. And, I am talking about those times that my father was still alive because you know I am new to the fatherhood and I know very little about parenting. And we take advice regarding food, clothes, school places, how to teach them, things that they should not do, and things like that. Because they have…

**I: they have encounter these.**

R: they have encounter these and they really know about these.

**I: perfect, thank you. interesting answers.**

R: we usually take advices from the elders

**I: when is it, that we bring the children, what illnesses that we bring them to take traditional treatment or traditional healer?**

R: well, you know let me tell you. as you know when the children have internal injury (abdomen) that's where we bring them to the people (traditional healers) to treat them. if it was you who knows how to treat, we bring them to you because you know how to heal them.

**I: when they have internal injury?**

R: yes, when they have internal injury

**I: are there any other illness besides internal injury?**

R: there are a lot. there are a lot.

**I: can you give some examples.**

R: well, my daughter. This guy touches her head, this guy Georgeton. that man that is sitting there. he touches her head, she was having a headache. and this guy touches her head.

**I: headache?**

R: yeah, headache. and she recovered till now.

**I: are there any other illnesses?**

R: there is another one, my daughter had rashes on her skin. there are images of rashes that I took. so, our mother was not with us at that time but the mother in law was present, she said, bring her so we can take her to the church. I said, are you guys going to pray for my child? where did you see that? let's take her to the hospital. so the old woman said, oh let's take her to my traditional healer. there is a woman who knows how to do traditional treatment. and this is what I am thinking of, man are they trying to do experiment on my child or what. after I thought it looks like a measles or chicken pox. so they take her to the woman. she treats her, the other day, there were still many with her.

R: you know, local illness? only hospital knows. because hospital knows people's illnesses. it knows, what causing people's illnesses. other kinds of illnesses hospital don’t know about.

R: it may reveal itself as a measel and those things, but they can't treat it.

R: they might not know that's a measel but they keep on treating it.

R: but as they see it, it looks like a measel. The child has measles but then it’s not.

R: if they would have showed how to treat it, that would be a targeted way hospital would have share it with people. people would have treated it too.

R: true.

R: there's a local treatment that can treat many illnesses.

R: there should have two hospitals.

R: yes.

**I: China does that. they believe in this.**

R: they believe in this?

R: there should have been two hospitals, one for local medicines and one for foreign medicines.

R: yes, true, true. true

R: abdomen healers and traditional herbalist

R: or budget maker

R: voodoo specialist, because many people casts spell on each other.

R: the fact is, these things I thought. superstition and they seem barbaric. We should take our time and look closely. the old people truly foretell this to me, wait till you have children and you will cry.

R: yes

R: you will know, you will see, you will feel. Do what we are telling and don’t disobey us. And I said those are not true. when I grew up and see with my own eyes. and experience then teachings that I was brought up with. and it is a fact that there are people who have local illnesses.

R: there are two kinds, traditional treatment for voodoo. we may not go straight to the hospital, because those are the voodoo practitioner. and those other traditional treatment, they don't use voodoo. like for abdomen injury treatment, woman bathing traditional treatment. treatment for wound, treatment for maj (internal illness that grows within ears, eyes, nose, anus).

R: for voodooism and those thing, the reason why they're ill because they've been on a spell.

R: he's right, there should be two types of professions. Marshallese and Foreign.

R: yes. marshallese and foreign

R: do you believe?

R; there are those, my sister. someone healed (breathing onto her hands) and recovered. she tried taking Tylenol, aspirin, ibuprofen. she always taking the traditional treatment.

R: I almost gave some food.

**I: perfect. now we're proceeding to the environments. is this place revealing? husbands are an important supports for their wives during pregnancy. what supports husbands do that time? the consultants want to know, because wives mentioned the supports of the husbands. and why?**

R: because when wives get etta (mood swing, cravings) they hate the husbands.

**I: it's not that, they said husbands greatly supports them that time. they may not... it is true, for now, the consultants want to know what kind of supports husbands do during the wives pregnancy.**

R: making sure they get the food they want. because when they get pregnant, they will want to crave any kinds of food every minutes and seconds.

R: well, for my wife... well it's true. I bring her everything, like pillow, her food, and support her like husbands does to wives. is it true? what can we say, when we have to say this. there's nothing.

R: you know why wives need their husbands? because the egg would not rot (definition of saying something must not happen to the child) when a rooster is not present near the hens, every eggs are rot.

R: physical and emotional something. by the time the woman is pregnant, you know they want the food that might be impossible to get. by the time whether you buy it or go fish for it.

R: if they say, I want to eat an orange from KnK (local supermart) they know, they know it man when you bought it from payless even if they were sleeping. as you looked on they throw them away, " why do you throw these away? " I said buy some from payless not to buy from knk.

R: I have a question, what if they want to eat jato (name of a fish)?

R: the answer for it add up everything including not to let the egg rot.

**I: what if they say, bring the fish inside the shark's mouth?**

R: that's a difficult question, it's difficult.

R: well, if you don't get the fish, the egg rots.

R: yes. what else?

**I: during their pregnancy, what foods do they usually eat?**

R: those things the men said.

R: there are alot, orange, apple, especially the thing that is rare on Marshall now a days. you the smaller coconut (smooth and soft unlike the bigger and stronger coconut)

R: well, as for my wife. well, am saying it truly, she was pregnant with my child. she always eats fish and chicken... and then especially, she ate soap (referring to by tasting it) it's true soap.

**I: soap right?**

R: yes, you know pregnant woman who had mood swings, she ate soap.

R: huh? You are telling the truth?

R: yeah my wife ate soap, it’s like she got possessed.

R: if it's not soap, then it’ll be uncooked rice mix with kerosene.

R: you know, when our wives do these kind of stuff they called it like ghostly manner

R: even the bottom of the zorries, they also eat it

R: well, that's our answer. now, what else?

**I: okay, in every places. they said, women usually take care of the children during the time they're caring for the child. what fathers and grandfathers usually do? men in the community.**

R: there are times they take care of the child.

**I: during the time the women takes care of the child, what are the responsibilities men do?**

R: work, work hard to bring the needs of the family.

R: yes, with what? with what? They need the answer like with what?

R: are there any other?

**I: what responsibilities? work right? they're busy with works? they're bring the family's needs?**

R: you know, I tell you, I tell you. I have a question, you know the men. you know, like yourself. you make money, you work for your family. is this question correct?

**I: yes, correct.**

R: well, that's it. because the children can have diapers and clothes.

**I: in other places, within each family. children are often all by themselves, because their parents are gone, growing up with their grandparents. or might be an adopted child, his/her parents is gone. his/her mother doesn't take care of his/her, she went to do bingo and go out. this question ask for.**

R: there is, there is this girl that we adopted. Her mother is younger than my wife, she's younger than boloti (name of the wife) we took her and raise her because her mother goes out drinking, her mother is alcoholic. she goes out drinking while we care for the girl. till now.

**I: it's both of you that are responsible for the girl?**

R: yes.

**I: type of children, are there?**

R: well, she's in school. I put her in school, because I'm working.

**I: beside both of you two, are there anyone who is taking care of her?**

R: my wife stays home, and I work for both of us. well, the wife takes care of the child.

**I: your neighbors, do your neighbors..**

R: mama, my mother takes care of her.

**I: she often takes care of her?**

R: yes..... how is it, does it answers?

**I: great. you know the information about health related stuffs... like for how to raise a child, ways to give them food. which place do people usually cite information from about child?**

R: well, I have never seen this kind of…. it would be hospital. hospital is where you go get information.

**I: does it shows in other places?**

R: yes, yes. it only shows at hospital, it does not show in other places.

**I: can you see it on internet, radio, are they shown in these places?**

R: Georgeton have you seen in these places?

R: also in the radio

**I: radio too? there are programs?**

R: yes, you know the old man that, do you know Tison? do you know about his program which is about the children who see or grows up with what they see. I know you know, because you always listen to it.

R: are there any water left?

**I: there are, help yourself... well then, is there another place besides hospital and the radio that we might find an information about taking care of a child?**

R: yes, from our parents.

**I: from the parents?**

R: or grandparents

**I: grandparents.**

R: it's better with those professionals grandparents or parents.

**I: well, I would like to thank each and everyone of you. you guys gave a lot with this survey, thank you for your time, you should have work, but you took part in it.**

R: don't worry, I'll let you guys to be excused.

**I: good, good, all of these information we'll help our children. on behalf of those I am working with, take care and have a good one today.**
