# Supplementary material for: Conceptualizing multi-level determinants of infant and young child nutrition in the Republic of Marshall Islands–a socio-ecological perspective
Source: PLOS Glob Public Health. 2022 Dec 19;2(12):e0001343. doi: 10.1371/journal.pgph.0001343 (PMC10022247; doi:10.1371/journal.pgph.0001343)
Supplement: S1 Data — (ZIP) [file pgph.0001343.s001.zip › RMI Supp Data/Focus groups data/F05U_FGD_Female_Rita_Aug 28_Libon.docx]

Interview code: F05U

Interview type and Interviewee: FGD Female

Interview Date: Aug 28 2018

Location: Rita

Interviewer: Libon

Transcriber: Christina

**I: Are you guys willing to do your part in this survey?**

R: Yes

R: Yes

**I: Before we proceed with the questions, we will first introduce ourselves, you will tell us your name, and how many kids you have, and how old are they. We will start from this beautiful lady over here.**

R: My name is Etka and I have two kids, a boy and a girl. My daughter is 6 years old and my son is 1 year old.

R: My name is Eljedrik Bila, I have 2 sons....2 sons. One is 5 years old and the other one is 8.

R: My name is Lucy, I have 5 children. What kind of question is that? Never mind..4 girls and 1 boy.

**I: How old are they?**

R: The oldest one is 18, next is 16, 14, 10, and 1.

R: my name is Regina, I have 4 children, 2 girls and 2 boys, the oldest one is 9 years old, the next one is 5, the third is 3 and the last is 0(not yet 1 year old).

R: My name is Jacklynn, i have 4 children, 3 girls and 1 boy....older one is 13, and the next one 11, third one 7 and the last one is 1.

R: oh, you're done? My name is Emina, I have 7 kids, 4 boys and 3 girls, 19 years old, 16, 13, 11,10,9, and the baby.

**I: How old is the baby?**

R: Zero

**I: How many months now?**

R: 11...

**I: 11, what about that girl?**

R: 10

**I: 10 years old**

R: This one will turn 1 in September, and this one will turn 10 in October.

**I: Okay, now we will talk about woman’s health, the first question says, can you tell me, what should a healthy woman's body look like?**

R: big...

**I: how big? Like as in big big or**

R: good size....

**I: not that big but good size....what about you guys, what do you guys think?**

R: skinny, the good size skinny

**I: the good size skinny, what else? The good size in big, the good size in skinny.**

R: I think those are the only body figure.

**I: only these two, so what about the food? What kinds of food that is healthiest for pregnancy women?**

R: like what?

**I: which do you think is most healthy and nutritious for pregnancy women?**

R: Fruits...

**I: Fruits...**

R: vegetables and those kinds...

**I: vegetables.......anything else?**

R: local foods...

**I: local foods.....so, you said local foods....what kind of local foods?**

R: breadfruit, lime....

**I: yeah...what else?**

R: fish

**I: fish, anymore local foods?**

R: Papaya, banana, taro, bwiro(Marshallese food), any kinds of local foods.

**I: Now, are these foods different from the one you usually eat or those that are not pregnant** eat?

R: like how?

**I: is it different? For these kinds of food, you guys said that they're healthier for pregnant women. Now this question is asking if the foods that pregnant women eats different than the one you eat.**

R: it's different.

R: yeah, it is different.

**I: now, how are they different?**

R: the foods that pregnancy women eat are different than the....

R: mmm....

R: the foods for pregnancy women....

**I: if you were the one that is pregnant, what kind of food you were going to eat?**

R: foods like Kool-Aid and stuff...

R: [laughing] second...

R: second...

R: foods that pregnancy women are not supposed to eat......

**I: now why did you want to, why do some pregnancy women want to eat foods like that?**

R: because we craved for them sometimes.

**I: so, do you think they're good?**

R: they're not good.

**I: now, those ae some differences....anything else? Okay, who or what encourages women about food during pregnancy? Who told you to eat those kinds of foods that you guys mentioned before that are nutritious for pregnancy women?**

R: ourselves...

R: our wants....

**I: okay, who else?**

R: at that time everything is different, if we want to eat something, we eat it right away.

**I: what about the ones that you guys said that are healthier, that are good for pregnancy women, who told you guys to eat those foods that are good for pregnancy women?**

R: doctor

R: the doctors...

**I: the doctors...**

R: the doctors...

**I: who else?**

R: the husbands...

R: our mothers....

**I: now, who did they encouraged food for women, how did the doctors, the mothers, or the husbands encouraged you about the foods you can eat?**

R: as for the doctors, they gave us words of advice.

**I: okay, so you mentioned words of advice, what kind of word of advice they gave to you?**

R: eat the foods that are good for the baby...

R: us....

R: the growth of one child...

**I: what else...anything else?**

I: what about the mothers and the husband, why did they want you to eat those kinds of food during the time you were pregnant?

R: such as what?

**I: such as nutritious foods, why did they want you to eat fruit, or vegetables and fishes?**

R: so the child can be healthy....

R: when the child is born, he won't be sick.

**I: in some places, women are discouraged from eating certain foods during pregnancy. For example, in some countries, they say, they believe that women are told that if they eat eggs during pregnancy, their child will become thief, which is their belief in some countries, what about here in the Marshall Island, do we have something to believe in during pregnancy, what food should not be eaten?**

R: octopus...

**I: what about octopus? Why did they encourages you not to eat octopus?**

R: we will have hard time giving birth.

**I: yeah, that's one thing....**

R: big fish....

**I: big fish? What about it?**

R: because some women, when they gave birth, they have their vaginal tear.

**I: oh.....**

R: yeah that's what we usually heard.....

**I: yeah, that's because it's our belief...anything else?**

R: pandanus that are cut in half....

**I: what about it?**

R: we don't eat it, because we'll also have our vaginal tear...

**I: okay, now we also know that some women receive supplements for low blood during pregnancy, or pills for blood, but some women told us they consumed all the supplements that were given to them but some don't. Now, could you any reasons why some women consume all their supplements but some don't?**

R: some women says they are allergic to the supplements.

**I: okay....**

R: nausea...when i drink those pills i feel nausea throughout the day....

**I: so do you consumed all of your supplements?**

R: nope...

**I: you stop drinking them.**

R: yeah...

**I: what about you guys over there? Why some do consumed all....**

R: me too, I don't drink the pills...

**I: and why is that?**

R: because of nausea...

R: well, I don't know, because as for me, i always consumed all of it...

R: yeah me too,

R: those pills taste goods.

R: when I see those supplements, so eager to drink them....

**I: now, what helps or motivates pregnancy women to consume all their supplements? What helps you to drink all of your pills?**

R: some drinks candies when drinking their pill...

**I: mmm...**

R: eat before taking the pills...

**I: okay...what about you? Because you mention that you always feel nausea.....**

R: i don't drink the pills...

**I: you just don't drink them....so, what are the consequences of having low blood during pregnancy or during childbirth?**

R: we will die, so says the doctor...

R: if there are some problems with the child it'll affect the mothers...

**I: now, were there any advice given to you to prevent or treat low blood in pregnant women?**

R: one more time...? The question?

**I: I said, did you received any advice from health workers to prevent or treat low blood?**

R: yeah...

R: yeah, such as they give us supplements to drink.

R: take them seriously...

R: eat foods that can help us with blood...

**I: okay, now we will talk about feeding baby after they were born....Once the baby is born and you begin breastfeeding, can you describe a healthy and nutritious diet for women who are breastfeeding? What kind of foods are healthy and nutritious for women who are breastfeeding?**

R: fish....

**I: fish...**

R: [laughing]

R: foods that are nutritious...

**I: okay such as what? What kinds of food they encouraged you guys to eat, so that it can provide more breastmilk?**

R: fish...

**I: fish....**

R: crab [laughing]

R: drink milk....

**I: drink milk, what else? What about can good?**

R: mackerel, ligo, foods that smells like fish.....

**I: okay, now similar to what we discussed earlier about food, like we mentioned about, eating big fish, or octopus, now could describe any beliefs that shows nowadays with women who are breastfeeding? what are....just like what we discussed before like when you eat octopus, you will have a hard time giving birth.....foods that you eat during pregnancy, what about foods you eat during breastfeeding, are there any in our country? Like we don't eat this food because....**

R: don't eat salt...

**I: okay, why?**

R: well, it's just like the big fish for those who are breastfeeding.

**I; okay. So why can't you eat....**

R: but for others like the kids, they're allergic to some...

**I: okay, what about.....what else?**

R: foods that we know it will affect the child and caused diarrhea, we know that we can't eat them.

**I: what about.......what if it was sashimi?**

R: the baby will bite my nipple...

**I: that's one belief, so, anything else?...aside from the sashimi and biting the nipple...are there any other food which you were told not to eat because it will affect the way you breastfed the baby......it may not be about foods, but it can also about breastfeeding....what about when you lay in bed and breastfeeding?**

R: yeah, lay in bed and breastfeeding.

**I: what about it? Why did they always say do not lay in bed and breastfeeding?**

R: because it will block the nose of the baby to breath and it will make the baby choke and puke and the same time.

**I: what else?**

R: for some women that like takes care of a child.....

**I: Now, what advice have you heard from health workers about breastfeeding? What are...what did they tell you about breastfeeding?**

R: from the doctors....

**I: the doctors or the nurses...**

R: by the time we breastfeeding the baby, the doctors said that we should always focus on breastfeeding the baby because it is good for their brain and it will also makes the breast full with breastmilk....that's what i heard...

**I: what about you guys? Why did you learn about breastfeeding for the doctors?**

R: when we are done breastfeeding, we put the baby in our shoulder.

**I: why on your shoulder?**

R: so the baby can burp...

**I: okay, anything else?**

R: Elizabeth's turn so she can say a few...

R: [laughing]

**I: is there anything that you learn from the doctor or nurse? What did they tell you about what you should or should not do during pregnancy?**

R: [laughing]....oh you.....

**I: [laughing] you want to get spank....**

R: [laughing] oh my stomach ache...

R: [laughing]....

**I: oh okay, now, were there any advice from the family or the husband about breastfeeding? What did they always encourage you about or say to you?**

R: [laughing]

R: first, breastfeeding is good than formula, because we don't use money...

R: don't get sick

R: they don't get sick, it provides nutritious...

**I: okay good....anything else? things that you heard every day from the family.....what about...like the one that I mentioned before about lay in bed and breastfeeding....about lay in bed and breastfeeding... is there any...is there any advice about laying and breastfeeding?**

R: they say, the baby will choke.... I have seen it before so i have learn not to do it...\

**I: okay, what else? We’ve heard some mothers first introduce foods other than breast milk when their baby is 6 months old, while some introduce foods earlier than 6 months. Could you describe the reasons why some mothers introduce foods or liquids earlier than 6 months of age?**

R: the times when they start eating, right? Because at that time the baby's body really needs it and it's about time when they're not full with breast milk because at that time breast milk has become somewhat water for them.

**I: okay...**

R: and when we feeds them such as baby foods, they're really full and energetic...

**I: they're energetic...what else? Why did you guys first introduce foods to your kids when they were 6 months old? Is there any baby that eats now?**

R: mmm.

**I: how old was he when start eating?**

R: 6 months...

**I: why did you start feeding him food at that age?**

R: because what i heard from the doctor was that baby can first introduce to foods at that age...

**I: okay...**

R: 6 months...

**I: who else? Who else told you that 6 months old is the time to start feeds them food? Aside from the doctors or the nurses?**

R: the family...

**I: the family, now, why do some mothers introduce foods or liquids before they reach 6 months old?**

R: some said to make them used to the foods, and their taste...

**I: okay, what else?**

R: they feed them because they said that they don't have enough breastmilk...

**I: okay, now what about when they are 6 months but they have not eaten any food...some can wait until 8 or 9 months old and start introduce foods to them, why do they wait later than 6 months old?**

R: some wants to wait till then...

**I: okay.... just for what they want**

R: yes

**I: ok. Many mothers have told us that they did not have enough breastmilk to feed their child. Can you explain to me how children under six months are fed when their mothers do not have enough breastmilk?**

R: can you repeat the question?

**I: it says, many mothers says that they don’t have enough breastmilk to breastfeed the child. Can you explain to me how children under six months are fed when their mothers do not have enough breastmilk? How are they fed that mothers do not have enough breastmilk to feed them?**

R: I don’t know

**I: Now if it was you and you don’t have enough breastmilk, what would you do?**

R: give him milk formula

**I: that’s one way**

R: (Laughing) because I have never been in cases like that.

**I: you said you couldn’t feed give him store bough milk? So you meant baby bottle**

R: I should’ve do both breastfeed and baby bottle feed.

**I: and what about you in that side?**

R: I would have also do both.

**I: ok. So are there any advice or ways to increase breastmilk? What can we do to provide enough breastmilk?**

R: breastfeeding mother, please answer. (Laughing)

**I: it says, are there any ways to increase breastmilk? What can we do to provide enough breastmilk?**

R: eat a lot

**I: eat a lot of what?**

R: fish

**I: fish and what else?**

R: foods that smells fish in can food

**I: ok smells fishy in can food what else**

R: drink milk

**I: ok, now where these advice came from?**

R: from our grandmothers

**I: grandmothers, who else**

R: our mothers

**I: who else?**

R: from the nurses

**I: nurses. Ok now could you describe for me how mothers in this community know that it is time to stop breastfeeding their child? How you guys do knew that it was time for the child to stop breastfed?**

R: they were old enough it was themselves that refused to eat from our breast

**I: when you say old enough, how old?**

R: they are in kindergarten

**I: do some children breastfeed until**

R: yes there are some children that breastfeed until they are grown up children

**I: ok that’s great. What else? How do you know that the child has to stop breastfed? Nothing else than they are grown up kids? Any other reasons that you see or know that the child has to stop breastfed?**

R: some mothers are pregnant again while breastfeed can caused sickness for the child

**I: ok so they have to stop breastfeed the child?**

R: yes

**I: ok now some people have mentioned that they try to feed their children a balanced diet. Can you explain what people mean by a balanced diet? What do they mean by that “balance diet”?**

R: what kind of food? Balanced diet?

**I: hmm. From your own opinion, what do you mean by balanced diet?**

R: meats and...

**I: meat ok and what else?**

R: rice

**I: ok, and what about you over there? What are the balanced diet for you?**

R: foods like meat, a little bit of rice, and fruits

**I: ok. And what about you Lucy? What are some balanced diet for you? What is it mean by balanced diet, what kind of food?**

R: any appropriate food

**I: can you share what kind of foods? Can you give two or three kind of appropriate foods?**

R: like fruits

**I: fruits yes**

R: and vegetables. Foods like fishes and can food that smells like fish.

**I: Ok. And what about you? What are the balanced diet and what does these balances diet means to you? Just from you, if you wanted to feed your child balanced diet, what would be the balanced diet for the child?**

R: it should be like corn starch, green beans, pumpkin, breadfruits, and papaya

**I: and what about you there?**

R: the same thing

R the same thing

**I: Ok for the last question, we want to know about how decisions are made.**

R: hey?

**I: why did you mentioned breadfruits, papaya, little bit or rice, or fruits, why did you mentioned these kind of foods, who influenced the choices about food you give the children?**

R: the doctors

R: from ourselves

**I: ok. And what about the family members?**

R: yes also our family members and the our grandmothers or the old ladies

**I: ok now why do you say the old ladies?**

R: because my mother used to advise me to eat these foods

**I: Ok. Now we would like to ask a few questions about illness that children usually get. When children under two get sick, some parents take their children to the doctors first and others use traditional healing first. Can you describe the reasons for this difference?**

R: sometimes we bring them to the doctors and the doctors give medicine but these medicines don’t heal the sick children. These are just some kind of sickness but when we bring them to the traditional healer, they heal them right away.

R: as of my baby, I never know my child is having stomach bump, but I keep giving him Tylenol but when I bring the child to the old ladies, they massage her stomach and the child can be heal from stomach bump.

**I: ok and what else? Ok now what illnesses are commonly treated with traditional medicine?**

R: (Kijon-kan) for small baby.* “when they would never stop crying or their skin is in yellow color and it’s really soft. Or also coughing and fever, asthma.

**I: ok. Any other illnesses that commonly treated with traditional medicine? You mentioned one illness what was that again?**

R: stomach bump.

**I: and what about head ache. Do they commonly treated with traditional medicine?**

R: yes

**I: ok. Now what traditional medicines are used for each illness? If it was *kijonkan* illness how would they do the traditional medicine for that?**

R: *kiop* local plant. Can be pandanus, breadfruits or any kind of green leafs.

**I: oh it can be different leafs that is used**

R: yes

**I: and if it was stomach bump, how would they do that?**

R: they just do stomach massage and give them traditional medicine to drink

**I: what kind of traditional drink?**

R: the medicine that is from the banana stem

**I: banana ok. Now who influences whether traditional medicine is used? Who usually say that they need traditional medicine?**

R: just us

R: us mothers

R: us

R: when we tried to give Tylenol medicine and see that he is not getting better, we look for better ways and try local medicine.

**I: ok so you feel that medicine from the hospital don’t work now you turn to use local traditional**

R: hmm

**I: Can you describe how children are fed when they are sick compared to when they are not sick? What are the differences?**

R: when they are sick, they don’t want to eat.

**I: ok**

R: they just want to breastfeed

**I: ok what else?**

R: they wouldn’t stop crying, they don’t want us to leave them alone, and they want us to carry them.

**I: like they are stick with you**

R: yes they’re sick with us

**I: ok and what else?**

R: they feel sleepy and would sleep longer than they used to

**I: what kind of foods given for them when they are sick?**

R: oranges, apples, bananas, cold foods like ice candy

**I: what else, and what about drinks?**

R: oranges

R: apple juice

R: pineapple drinks

**I: and what if they are not sick? What kind of foods that given to them when they are not sick? What kind of foods that you usually give them when they are not sick?**

R: rice and meat

**I: rice and meat ok and what else?**

R: rice boil with pumpkin and coconut herb or the seat. Boil with rice.

R: any kind of food

**I: what was that?**

R: it can be any kind of food given for the child to eat.

**I: Now we would like to learn about the foods that you provide for your family. Could you talk about what influences which foods people in this community provide for their families? What kind of foods that provide for the family?**

R: rice and chicken

**I: chicken and rice?**

R: yes

R: yes it’s common for families to eat chicken and rice

R: rice and chicken, fish, can food

**I: fish and can food, there is one food that I usually see children buy from morning to night time**

R: hot dog

R: (Laughing) yes hot dog.

R: because that meat is good and delicious.

R: no more like hotdog is like a trash combination of all kind of meats there is no vitamin contain in hotdog.

R: and how come they don’t stop selling hotdog?

R: because they also get money from selling hotdog.

**I: I think they have already stopped people from feeding their children hotdog.**

R: there is no different from feeding the just rice with water.

**I: that’s better. I can say it is better to feed your child just rice and water than giving the hot dog for them. Now what are the difficulties in getting the foods they want for their families?**

R: some foods are not common and some don’t have enough budget to get them and don’t have these foods

**I: but they want to eat?**

R: yes they want to eat

**I: what else? You said they don’t have enough budget, what you mean by that?**

R: some want to buy them and don’t have enough with them

**I: now why they don’t have enough to get them?**

R: three is no money

R: there is no money they don’t work

**I: anything else that make it difficult in getting the foods you want for the families?**

R: we don’t work but we have only small budget

**I: no work and not enough budget and what about food prices?**

R: they’re expensive

**I: expensive. Now how families deal with food shortages, for example sharing foods or buying foods from the shop?**

R: they only share uncommon foods.

**I: ok what else?**

R: sea foods

**I: yes like what kind of sea foods?**

R: fish, octopus, clam, bivalve

**I: and what about foods from the store? What do you buy from the store that you know that there is not enough foods or budget?**

R: like chicken, mackerel, sardines, tuna

**I: u mean that only cheap foods**

R: only cheap foods

**I: Many families have told us that fresh vegetables are not affordable. Can you describe any other reasons that families do not eat many fresh vegetables? They said that they can’t afford vegetables and fruits, what are some reason why they can’t afford these foods? If it was you, do you buy fruits and vegetables?**

R: sometimes

**I: and why not all the time?**

R: they are expensive

R: not all the time because

R: because sometimes there is money and sometimes there is not enough money

**I: what else?**

R: fruits, we cannot bring the fruits and vegetables and share it to the whole family.

R: only one can have fruits or vegetable.

R: I mentioned that some family member can’t afford fruits and vegetables for the whole family. If it was one or two vegetables it’s enough. If we were saying we bring it for breakfast, it’s still not enough.

**I: ok now in this section, we would like to talk about water and hygiene. Can you please describe how people typically get water for their families in this community?**

R: what kind of water?

**I: drinking water, bathing water, any kind of water. How do you bring water to your house, or how do you have that water in your house?**

R: I have water in my house because sometimes it would be raining. Sometimes we used water from the well. And we also get water from the water service company. We paid it from the water service company.

**I: paid for water ok. Now are there any difficulties in getting water?**

R: yes

**I: what are the difficulties that you see?**

R: when we’re out of water, we have to wait again

**I: wait for what?**

R: wait for the rain (Laughing)

**I: ok that’s great.**

R: or we get water from the well

**I: ok what else?**

R: wait for the water service company to turn on the water

**I: ok. What else? Are there any difficulties in storing water?**

R: how to store?

**I: like storing water**

R: there is no difficulties in storing water.

**I: ok. There is no difficulties in storing water**

R: when we store water, we are saving them

**I: and in terms of having water catchment?**

R: when we have water catchment?

**I: what are the difficulties?**

R: if I have water catchment, I don’t need water

**I: if there is water catchment you don’t need water anymore?**

R: hmm

**I: and what about you? Any difficulties in storing water?**

R: when it doesn’t rain we do really need water

**I: ok and what about water catchment, do you have enough?**

R: we don’t have enough

**I: ok. Now we’ve heard that some families boil their water for drinking and others do not. Can you explain why some people boil their water and others do not?**

R: because they have dirty water

**I: ok**

R: they boil their water because they are taking care of their body health.

**I: what else?**

R: they have already teach us how to take care of our water, like even we put water in a gallon and add Clorox in it.

**I: are there any difficulties to keeping water catchment systems clean?**

R: there is none

**I: are you sure about that?**

R: they’re clean we guarantee that they’re clean. (Laughing)

**I: and what about the roofs**

R: Ok so you should have mentioned about the roofs

**I: ok now can you explain difficulties in cleaning our water catchment?**

R: dust

R: dust

R: wind blow the dust into the water catchment

R: salty water blown

**I: ok what else?**

R: houses that have trees on the roofs?

**I: We’ve heard some families wash hands regularly while others do not. Can you explain some reasons for this difference?**

R: some forgot to wash their hands

**I: ok**

R: some are lazy

R: some are in hurry to eat their foods

R: as of some children, we they are really hungry they care if their hands are clean or unclean.

**I: why do some people wash their hands with soap and others do not?**

R: some people are neat and want clean environment and some don’t. (Laughing)

**I: What are the main reasons that prevent washing hands with soap regularly?**

R: when you use the hand sanitizer they said that there is no different in using the hand sanitizer from using soap.

**I: ok so you meant that instead of using water and soap they can use..**

R: the hand sanitizer

**I: ok what else? What else? What prevent them from washing their hands with water and soap?**

R: sometimes they run out of water.

**I: ok**

R: some used to not washing their hands before they eat

**I: what are some reasons why some people use hand sanitizer instead of soap?**

R: it’s the most reliable way

**I: most reliable way for them ok.**

R: it can also kills germs

**I: ok now for the last questions, we would like to learn about how parents care for their children. We’ve heard that husbands are an important support for their wives during pregnancy. Can you explain what husbands do to support their wives while they are pregnant?**

R: what was the question?

**I: what do husbands do to support you during pregnancy?**

R: they help do the house chores. When we feel like we don’t want to do them, they can do the rest of the house chores.

**I: ok**

R: just chores. Some would do laundry because we don’t want to move.

**I: ok. What else? Anything else that your husbands did to help you during pregnancy?**

R: they would always stay near us so that they can give us our needs and wants

**I: ok that is also one reason. And what about ladies over there? Or what mothers or other family members do to support their daughters while they are pregnant?**

R: like what? They push us to do some movement and do works around the house.

R: they don’t want us to stay home for no reasons.

**I: ok**

R: they even tells us not to stay home and do nothing because when we don’t do some moving and energetic our body, we will be in labor of giving birth. Do lot of movement and works.

**I: just do lot of movement. We are interested in learning about how caregivers play with children under two years old. Can you describe for me in detail how you play with children? How do you guys play with your children?**

R: play with them and tell story with them. Teach them the way they walk or always make them feel happy.

**I: We’ve heard interested in learning about how caregivers play with children under two years. Can you describe for me in detail how you play with children? Do you have any experiences on parents that leave their children and stay outside or their houses?**

R: so that they can do what?

**I: in cases like parents don’t watched over their children but they leave them and go out and do whatever they want to do? Telling stories with neighbors or..**

R: and the child is alone?

**I: yes**

R: the child can eat from the trash or eat dirt

**I: what else?**

R: why do they leave their children and don’t take care of them?

R: some mothers leave their children and go out and look for cigarette and betel nuts, and telling stories

**I: How these activities affect feeding their children under two? I think you mentioned it earlier like eating dirt.**

R: caused sickness like diarrhea

**I: ok what else?**

R: danger

**I: ok. Now how these activities affect the hygiene of the child? Will the child have clean hygiene when the mother is not there with them? What would happen to them?**

R: danger

R: dirt can cause sickness

**I: what kind of activities women or men doing outside of the house?**

R: clean

**I: what else?**

R: filling water catchment

**I: what else they do that they don’t really take care of the children, what do they do?**

R: go out and tells stories.

R: riding taxi or gamble like bingo.

**I: ok and what about the men, what do they usually do?**

R: got drunk?

**I: what else? And what about you Lucy?**

R: like what?

**I: things that your husbands usually do outside of the house?**

R: simple works around the house or he also can go fishing.

**I: We have heard from some people that they prefer to get health messages from the radio, others say from the newspaper. Can you describe for me the best ways to reach people with** information on health in this community?

R: Facebook

R: come and work with them

**I: ok what else? Anything else except from Facebook and community outreach. Any community groups that could be a good place to deliver health messages, for example women’s groups or mother’s group?**

R: there is none

**I: there is none? Ok that was great, we are done now. Thank you once again for your generous time and for sharing your thoughts with us. We greatly appreciate your help and we hope this research will help us improve the health of mothers and children in this community.**
