# Supplementary material for: Conceptualizing multi-level determinants of infant and young child nutrition in the Republic of Marshall Islands–a socio-ecological perspective
Source: PLOS Glob Public Health. 2022 Dec 19;2(12):e0001343. doi: 10.1371/journal.pgph.0001343 (PMC10022247; doi:10.1371/journal.pgph.0001343)
Supplement: S1 Data — (ZIP) [file pgph.0001343.s001.zip › RMI Supp Data/Focus groups data/F06U_FGD_Male_Rita_Sep 1_Balton.docx]

- Interview code: F06U
- Interview type and interviewee: FGD- Male focus group
- Interview Date: September 1 2018
- Location: Rita
- Interviewer: Balton
- Transcriber: Marcellina

**I: If it’s okay with everyone we can proceed. Okay?**

R: yes

**I: okay. The first questions are about food availability and security. Now, we would like to learn about the foods that you provide for your family. Could you talk about what influences which foods people in this community provide for their families? Or what made you choose the foods you usually eat?**

R: for me budget. If you have enough money in your pockets, then you can choose whatever foods you want to eat and amount of foods you need depend on how much money in your pockets.

**I: are there anything else besides budget which can help us choose the foods we want? It’s true that budget really help us in choosing whatever foods we want, but beside budget there anything else? Like if were living in the middle of your neighbor’s houses or in the lagoon side, are there any difference in living in the middle of your neighbor’s house or in the lagoon side or ocean side?**

R: at lagoon and ocean side, it’s good to live in because it has lot of space where we can grow local foods or make our own garden.

**I: so, you are saying that it also helps with providing foods on our tables, right?**

R: yes, but living in the middle of our neighbor’s house it’s not good because we don’t have enough space to do our works or make our own garden.

**I: okay, are there any foods that your neighbors at lagoon side consume but not consume by your neighbors at ocean side?**

R: as for the neighbors in the lagoon side, they can just walk to the lagoon side and fish. Neighbors in the ocean side, they can just go the ocean side and find shells or whatever they can have for food. But neighbors in the middle can grow local foods if they want to.

**I: oh okay. Can you tell me how some families deal with food shortage or when there is not enough budget? Let’s just say not enough budget. Where else they can find food?**

R: as for us Marshallese, we usually go fishing for foods. But some families own local animals like pigs or chicken, so they can have these for food. Or the fruits they grow.

R: or we share our own food with the ones they need.

R: Jake Jebol Eo (Sharing is Caring)

**I: Can you really explain more about the word Jake Jebol Eo?**

R: it’s like when you ask for help from your family members or neighbors, they are willing to offer what you ask for.

**I: that’s great. *Jake Jebol Eo * or sharing is caring that word really separate us from other many other countries because Marshallese people share a lot and we’ve never been in the level that says “you are on your own” we share a lot because we care a lot.**

**I: for the second question. It says, we have heard from some families that they eat local foods whereas others eat mostly processed foods. Can you explain why some families eat more processed foods?**

R: as for the local foods, we have to prepare and cook them before we can eat them. As for the processed foods, we don’t have to prepare and cook them for a long time before we consume them.

**I: yes. Are there any differences in costs?**

R: as for local foods, we don’t have to pay for them if we have in our backyards. But the processed foods, we have to work so we can have money to buy them. Like rice it’s really expensive so people have to work so that they can have rice on the table, because many people say that if they don’t eat rice, they feel like they are sick. People here on Majuro are not always complain that they are starve when they don’t have rice on their table, it is the same for people in the outer islands they would also complain because and say that there is famine when there is no rice or flour in the outer islands, but they got plenty of local foods like taro, breadfruits, or pandanus and plenty more local foods.

**I: but there are lot of free foods they can get yes?**

R: yes they can just get free foods without spending money. They can get them and cook them right away and the very important thing is that these foods are really fresh and

**I: okay good. So, we’ve heard that some families sell or trade the foods they grow. Can you tell me why some people sell or trade the foods they grow?**

R: they sell them, so they can get other needs they need.

R: they sell them because they don’t want them anymore. They get tired of eating them every single day.

**I: okay are there anything else? If there aren’t, then can you tell me which foods are usually sold or traded? It can be anything. Just share your information and that you see around you like pig or chicken?**

R: fish.

**I: fish? Mm**

R: some sell pigs. And the foods they grow, usually banana, breadfruits, pandanus, and papaya. I think these are the common local foods that people usually sell. And breadfruits also.

**I: We’ve heard that one of the reasons some people don’t grow food is the lack of space for planting. Can you tell me about any other difficulties that people have with regards to growing food? Especially town here like Rita or Jenrok these areas. Houses are too crowded for people to grow plants. Can you guys tell me anything that makes it difficult for people to grow foods?**

R: permission from the landowners or from EPA (environmental protective Authority) because if they find out that you have dig the ground, they will arrest you

**I: oh! we must have permission from the EPA too to grow food?**

R: yes. Because they are the ones that provide us with local foods to grow.

**I: but not every places, right?**

R: yes, some place they are afraid of the landowners.

**I: what about those households that they already have local foods in their area like banana, how did they grow?**

R: yes what about them?

**I: how did they get their areas to grow their foods?**

R: they asked permission from the landowners

R: some place, the local plants have been there from long time ago

**I: hmm like the pandanus right?**

R: yes

**I: so they have been there from long time ago okay that’s great.**

**I: okay that’s really great. We will now move on to water and hygiene. Now, we’ve heard that some families boil their water for drinking and others do not. Can you explain why some people boil their water and others do not?**

R: some people boil their drinking water because they want to have diarrhea. But some don’t boil their drinking water because they just don’t want to.

**I: can you tell me what type of water that need to be boil?**

R: water

**I: yes there did the water come from?**

R; some people boil their water

**I: from water catchment?**

R: water from the water catchment

**I: where do people in this community get their drinking water from?**

R: from our water catchment. But those people that don’t have water catchment they get water from those who have.

**I: rain water?**

R: yes

R: some buy from the stores like EZ Price Mart, some people get water from there.

**I: is there is water like that in this community?**

R: yes there is one in Rita?

**I: does it work? The closest one in this town Rita.**

**I: are there any water well in this community?**

R: 17:11/51:31

**I: what do people in this community usually do with the water well?**

R: they use it for bathing, cooking, washing clothes they use the hose and pump the water from the water well

R: cleaning the pig’s fence

**I: ok so they use the pump and the water well is not salty**

R: yes water well in this town is not salty like others places

**I: okay good. We’ve heard some families wash hands regularly while others do not before they do something, like in the morning, at noon, and in the afternoon. Can you explain some reasons for this difference?**

R: as for some people, they don’t want any germs in the foods they’re preparing. Some need to wash their hands after they eat. But there are some people don’t wash their hands after they eat, they just lie down or go to sleep.

**I: what other times people need to wash their hands beside after eating?**

R: before they go to sleep and after they wake up. There are some students wash their hands before they write and after they are done with school.

**I: what about the workers?**

R: they wash their hands after they are done with cleaning there are just different types of people.

**I: beside before and after eating, are there any other times people need to wash their hands?**

R: after using the rest rooms

**I: like picking up trashes?**

R: also, after cleaning our yard

**I: can you tell me what time people usually use soap to wash their hands?**

R: some people use soap before they eat.

**I: all the times?**

R: Some use it after they use the rest rooms

**I: by using soap?**

R: yes

**I: what about the times we don’t use soap. What time people don’t use soap to wash their hands? Just share any of your information. What times that people don’t use soap to wash their hands because they are in hurry and all that?**

R: when you are in rush and you forgot to use the soap. Sometimes when you are really hungry you don’t want to wait any longer you can just wash hands and then eat right away.

**I: beside in a rush. What are the reasons why sometimes there is no soap to wash our hands with?**

R: W have to buy soap s that we can have soap. When there is not enough money in our pockets we won’t have soap.

**I: that’s the reason why people don’t use soap**

R: yes we have to have money in our pockets so that we can get soap to wash our hands

**I: We’ve seen that some families keep chickens and other animals enclosed in a fence while others don’t have fences for their animals like pigs, or it cannot be pigs because there is a law against pigs but let’s just say chicken. Can you tell me any difficulties to keeping chickens or other animals in a fenced area?**

R: any kind of animas?

**I: hmm. And what makes some people make fence for their chickens but don’t? Why some people let their chickens go outside of their fence? Like these chickens can be just outside of their houses but the thing is that, they don’t put them in their fences. What is the differences between fence and not fence?**

R: some chicken are in their fence?

**I: yes some do and some don’t. Like this family put their chickens in their fence, and that family don’t, they just let them in their areas. Now these people would like to know why some families let their chicken in their yards and some don’t.**

R: you are talking about only one fence or?

**I: yes just one fence**

R: some chicken would want to stay outside and don’t

**I: no like if it was you, you put your chicken in their case, but that guy over there doesn’t, what makes this different when some chicken are in their cases and some are not? Why do some families put their chicken in their cases and why do some families let their chicken go?**

R: some people don’t keep their chickens in a fence because according to them, if they put them in fence they will feel sick. And so that they can just roam around and find food for themselves.

**I: what about the chickens that are kept in a fence. Why some people put them in a fence?**

R: because they don’t want people to steal them

R: so they don’t want to lose them

**I: okay the last question about water and hygiene. In some communities, we have heard that defecating in the open (such as on the beach) is common. Could you help us to understand this practice, including how common it is?**

R: as I said before, the pockets are weak!

**I: for?**

R: the money in our pockets are not enough to build our own rest rooms

R: also, it’s like it’s a habit and its free we don’t have to build any rest rooms. Some people have enough money to build their own restroom and some people are not willing to do that because they don’t have money. Let just go with the word “common” or it’s their habit.

**I: that’s mean, some don’t have enough money to build their own rest room and some have it as a common practice or it’s their habit.**

R: yes

R: another reason is, the landowner. Some people may have enough money to build their own rest rooms but if the landowners won’t let them dig up their ground then you won’t have.

**I: so another difficulty in this case is the landowners.**

R: these land owners sometimes force people not to build their rest rooms. They also affect EPA from doing their jobs.

**I: ooh so they are also the reasons that makes it difficult for EPA to do their jobs.**

R: all the major issues that we face here in this community are from EPA and landowners and also financial issues, some have strong and weak pockets to afford for a restroom for themselves. We have to think twice that what stopped us from our doing in these areas are the EPA people and these land owners.

**I: in your own areas?**

R: yes because you think that you are the owner of your own place but you would never know that EPA can over control or their power and force you to do and don’t do things.

**I: ok now can this practices not happen to people in this community but also happen to people from other community?**

R: yes these foreigners also can do so.

**I: can it be people in other community?**

R: yes they also do so

**I: how can it be? In what ways?**

R: what you mean people from other community?

**I: like if a guy come from Delap community**

R: he come to this town maybe because he doesn’t have rest room to use

**I: ok so that means there are lot of reasons why people defecate in open beaches.**

**I: Ok now we would like to ask a few questions about children when they are sick. From what we have studies from different families, we found out that when children get sick, some families usually bring them to the hospital and some bring them to the traditional healer. So the foreigners or the ladies that we worked with knows what illness that we usually bring our sick children but now they want to know what kind of illness we bring our ill children for the traditional healer. What kind of illness that parents bring their ill children for the traditional hearer?**

R: stomach pump

**I: stomach pump? What would the traditional healer can to with that?**

R: they do stomach massage

**I: so if the child needs to do massage, what would you do? See the traditional healer first or check her up with the adults in the community? Like who in the family would see that the child is having stomach pump?**

R: it can be their parents or their grandparents but the truth is it would be the mothers. If they know that they can’t do anything about it, they bring them to the traditional healer.

R: they would also found out that the child is having stomach pump because the child always get nausea and they don’t really eat their meals. Now what they would do is go see the traditional healer. It is also happen for boys when they have their balls pump. Parents also bring them to the traditional healer so that they can massage the boy’s pump balls.

R: it’s not happen most of the time for girls, but mostly it happen to boys and it is really serious for boys when they have balls pump.

**I: it is danger for boys? So does the hospital treat illness like that?**

R: no

R: hmm no

**I: is there is... sorry?**

R: they only give us medicine to help prevent the ache.

**I: ok thank you. So are there any illness except for stomach pump? It can’t be just to boys but also can be for girls.**

R: sometimes they got diarrhea

**I: ok diarrhea. So are there any traditional medicine to prevent diarrhea?**

R: yes there is. Sometimes they give traditional medicine that they should be drinking like the *malo* or the seed for the kiop tree* local plants.

**I: hmm**

R: they pound the kiop tree seed and then rinse it in a piece of cloth and make the drink for the ill children.

R: or it can be the …what you call the thing again… it is something that the traditional healer used to stop the diarrhea illness.

**I: oh. So they usually use it. That’s great. Any other illness that you would bring the ill children for the adults? They also used to have fever, diarrhea and stomach pump. I think these are the common illness for children. Ok now this question is all about gender family roles for the children. We’ve heard that husbands are an important for their wives during pregnancy. This resulted in what we have studies when we the guys studied with the males while the girls’ studies with the females. These information were found out that males are responsible enough for their wives during pregnancy. Now these foreigners want to know from the males, what do they usually do to their pregnancy wives during pregnancy? As of you guys, what do you do during wives’ pregnancy?**

R: do fishing because they are craved for eating fish. Give them local foods. If they want to drink fresh coconut juice, even though it is raining but we would go ahead and climb the coconut tree and get the fresh coconut juice

**I: laughing. Yeah that is the reasons why we found out that females would mentioned that their husbands are more responsible than their parents during pregnancy. Now here is another questions that asked “What did you do during your wives pregnancy?**

R: help her

**I: I want to know from all of you guys over there. Just something that you can remember helping your wives during pregnancy.**

R: the first few months of pregnancy, she would always want to be with us (husbands) no matter what. They want to be spoil and want us to help them with everything.

R: it is the time we have to be there because she would want to lay down and tell us to do anything she would tell us to do so. It is the time for the wives and the husbands to be together. They would be together until the wives give birth, now they would start with their own troublesome and arguing for nonsense.

**I: but during pregnancy, they are together**

R: yes they are together

R: we would help together for nine months until her birth

**I: We’ve also heard that mothers are mainly responsible for taking care of children in most communities. Now they say that at that time there is now a baby, and the mothers is the main responsible for the child. Can you explain the responsibilities of fathers in this community in caring for children? If the mother is responsible in taking care of the child, what is the responsible of the father at that time?**

R: they can do house chores like cleaning the house or clearing the baby’s diaper. What you call that thing. I am not talking about the real diaper.

**I: the piece of cloth diaper?**

R: yes that’s it.

**I: anything else when the mothers is at the house watching over the baby and taking care of the child, where is the father at that time?**

R: sometimes he would go fishing. So that the breast can provide enough milk for the child. Some fathers are at work.

**I: work ok. So that means the fathers are out there responsible for bring foods for the family.** Do fathers responsible in parenting?

R: yes they also do.

**I: in what ways?**

R: when the mother is really tired at night time and needs to rest, fathers can do their part and watch over the child throughout the night. Like if the baby is crying all night, the father can carry him/her until the baby stop crying.

R: fathers are also responsible in taking the child to the hospital when the child is sick. And we also can help by giving the medicines for the child when mothers are too tired to do so.

**I: are there any differences for fathers parenting a female child than a male child?**

R: yes there is a big different but is it for the mother or the father.

**I: the fathers. Is there is any differences in raising a female child than a male child?**

R: YES. In our custom, they said that if the father have his eldest daughter, it is not right for him to clean her up when she poop or pee.

**I: hmm ok. And what about the sons**

R: ok for our sons, there is no different because we are men to men.

**I: there are just some families here that their children don’t have parents. Or these children can grow up with their grandparents. Other children don’t have their fathers near them or their mothers don’t stay at home and take care of them but they would go out and do whatever they want to do. Can you tell me who is the main responsible for the child is at that time when their parents are not near them or let just say their parents are there but they working. Who usually responsible for the children?**

R: Their grandparents or their aunties or uncles

**I: so you mean the family member**

R: their parents’ younger sisters and brothers

R: sometimes some parents don’t want to watch over other parents children. They said that they did not gave birth and labor to that child so they don’t want to take care of other people’s child. There are many child on the road watch over themselves. They are like outlaw children.

**I: ok so in cases like that who is the main responsible for the child?**

R: they usually stay by the road or just anywhere and people that cared a lot can take them and take good care of them if they want to or if they cared for them. But I do not believe that people from that house or that community can come to this community just to watch over other people’s child.

**I: Hhm it is hard to raise a child that is not yours. Now as for children like that, who would responsible in bathing or feeding them?**

R: because sometimes their step mothers don’t want to care or support the child but the child’s aunties also don’t take responsible for the child.

R: they take responsible against each other.

**I: what are some reasons why mothers leave their children? Like let the alone. One reason is work. They go for work and they give their children for other people to take responsible for them, any other reasons?**

R: some would go out and have fun like gambling (Bingo)

**I: hm and what about the fathers?**

R: some would go out and get drunk

**I: get drunk**

R: some are cheating on their wives and their children and some have other families out there to take care of.

**I: Our last questions is about information on illness that children often get in this community. Where can we get information on illness children like fever or stomach or something that grow in the body of the children? Where can you get information like these from?**

R: when the child is sick?

**I: yes**

R: hospital

**I: hospital hmm**

R: from the doctors

**I: any other places that you can see, hear or get information on children’s nutritious and health or the way children can be treated?**

R: Public health main office

**I: you go straight to Public health office?**

R: yes and also children’s public health office.

**I: and how about adult children**

R: the ministry of hospital should go out and do community outreach like programs that would help people on how to treat a child or make a child happy or play with a child, programs like the WUTMI (Women United Together Marshall Islands)* Ajiri in Ibwinini* A program for unhealthy children.

**I: what is that mean when you say “ajiri in ibwinini”?**

R: organization that do community outreach and give people information on children’s nutrition and health.

**I: oh these are organizations right?**

R: yes they goes from community to community and especially they give out information on the radio station.

R: also the youth to youth clubs, they also do the same thing.

**I: ohh ok**.

R: they also help public health on information about children’s nutrition and health.

**I: so the radio station have its part in spreading information to families about children’s health and nutrition.**

R: especially people that don’t really visit the doctors with their children.

**I: these programs are really great and helpful. Ok that was my last questions. I want to take this time and thank each and every one of you for sharing your information and like is said, all these information that shared by you guys, we will used them and put them in our report and I would like to thank you all once again on behalf of me and my co-workers especially these foreigners ladies that I worked with and the ministry of health and UNICEF it’s the organization that help our mothers and children on their health. Thank you all and God bless us all.**
