# Supplementary material for: Conceptualizing multi-level determinants of infant and young child nutrition in the Republic of Marshall Islands–a socio-ecological perspective
Source: PLOS Glob Public Health. 2022 Dec 19;2(12):e0001343. doi: 10.1371/journal.pgph.0001343 (PMC10022247; doi:10.1371/journal.pgph.0001343)
Supplement: S1 Data — (ZIP) [file pgph.0001343.s001.zip › RMI Supp Data/Focus groups data/F07U_FGD_Rita_Sep_Libon MHL.docx]

**I: Would you guys like to participate in this survey?**

All: Yes

**I: I will go ahead and introduce myself. My name is Libon Jorkan and I have 3 three children 2 boys and 1 girl. The oldest is 14 years old, the second is 6 and the youngest is 2 some years… We’ll go this way.**

GR: I am Mary Alex, my name is Mary Alice. I am a grandmother and I have 4 children and I think I have 6 grandchildren. The child I look after is 1 years old. My name is Lynn I have a child that’s 2 years old. My name is Atrik I have 2 children and I come on behalf of my grandchild.

**I: How old is she?**

GR: 1 years old. My name is Linda. I have I child and she’s 1 years old. My name is Emily. I am a grandmother and I have 5 children. My youngest is Jowe is in 12^th^ grade but I come on behalf of my grandchildren, 1,2,3,4,5,6,7. Seven (7) grandchildren and I’m always with all the time. My name is Neiland Jeto. I have 2 children, 2 boys. One is 3 years old and one is 1 year old.

**I: Okay that’s good. Now we’ll begin and talk about the health of a woman. It says, can you describe a body of a healthy woman... how would you describe a woman who is healthy or describe the body?**

R (Emily): She’s in her youth, not too heavy not too skinny.

**I: Good what else?**

R (Emily): She’s not lazy.

**I: Anything else? A woman that’s really healthy?**

R (Mary): She doesn’t constantly have plenty of kids.

**I: Doesn’t have plenty of kids… okay… anything else? … If there’s nothing then we’ll go on to the next question. It says what is the healthiest food and the most nutritious food for a pregnant woman? What kind of foods are healthy or nutritious for woman?**

R: Fruit

**I: Fruits!**

R (Emily): We can say all the food from the 3 food group that’s healthy.

**I: Okay from the 3 food group. What about Marshallese food?**

R: Also

R: Also local foods.

**I: What kind of Marshallese food?**

R (Mary): Foods that are not salty

**I: Foods that are not salty**

R (Emily): Almost all of our foods are good (healthy). Pandanas, breadfruit, iu, coconut,

R: ek

R: all of these are good (healthy)

**I: Are these foods different from other woman’s normal diet? When I say other woman I mean by not pregnant woman?**

R (Emily): There’s no difference. But for pregnant woman, they’re really picky about their food. Their diet (pregnant woman) is different from ours (not pregnant woman).

**I: What about you mothers, when you were pregnant what kind of food did you eat?**

R: banana, breadfruit.

R (Emily): Different woman have different kind of craving.

R: Yes

**I: So who and what influenced the woman about these food during pregnancy?**

R: I think what we crave is what we eat, not influence, but what we crave is what we usually eat.

**I: Okay. But what if there were advice on the foods that you should eat, who would advise you?**

R: the doctors

**I: The doctors. Who else?**

R: Their mothers and fathers

**I: Yes. Who else?**

R: Also their husbands

**I: Yes. Now how did these things influence a woman’s diet? What did they say or what did they do?**

R (Emily): They would say don’t eat this because it’s going to affect the child im harm him/her. The salty food.

R: Yes

**I: What else… Anything from that side? Why didn’t they let you eat the things that you ate?**

R: So, it doesn’t affect the baby.

R: Also, to make us healthy.

**I: Yes. Now at some places, woman are discouraged to eat certain food during pregnancy. For example in some countries, some woman say if they eat eggs during pregnancy, their child will become a thief. That’s their belief. So, can you describe any believes in this community or in this islands? During the time you’re pregnant?**

R (Mary): I belief that if we eat food that are torn or cracked or how do you say it?

R (Emily): Crack?

R (Mary): Crack or torn?

**I: Yes. What will happen?**

R (Mary): It will make the baby big. Or what was it?

**I: Yes. That’s it. Now you say when they eat crack foods it will affect the mother or the child?**

**R (Emily):** The mother.

R (Mary): The mother and child.

**I: The mother and child?**

**R (Emily):** And also if you walk behind a pregnant woman, it will affect the mother and child because the umbilical cord is wrapped around the baby’s head or throat.

**I: Oh okay.**

R (Mary): And can also cause diamond, child with diamond mouth.

R (Emily): If we eat crack food? OH…

R (Mary): If they eat cracked food or torn food, it also causes diamond.

**I: Anything else?**

R (Emily): They also say that if we eat cracked food, during birth we might be torn (women’s private part). But a child being born with diamond, well it comes from being depress from what I know. If we’re really depress with our husband or

R (Mary): But we us Marshallese, they say we should not eat torn or cracked food

R (Emily): This is my first-time hearing this.

R (Mary): Because the child will be diamond.

**I: Okay are there anything else during pregnancy that we have believes in?**

R: Usually they when we’re pregnant, we’re not supposed to walk around while we’re eating.

R: Yes

R (Emily): Eating while you walking around.

R (Mary): yes walking around eating.

**I: Now why shouldn’t they be walking while they’re eating? What’s going to happen?**

R (Emily): During labor they might just want to walk around. Instead of lying down, they want to walk around.

R (Mary): They won’t lie down because they only like to walk around.

**I: Oh okay. What the time when you’re in labor and you can’t seem to push, are there any believes that we believe we can do to make it easy to give birth?**

GR: Yes there a lot.

**I: Well?**

R: Medicine

R: medicine

**I: When you say medicine, what kind of medicine**

R (Emily): Our ancestor’s medicine. Any kind of leaves or flower they give us to eat or drink. Especially the eggs that they put on our stomach.

**I: Now we know that some women receive supplements for low blood like the iron-sulfate. Some women say they drink all of their supplement during pregnancy while others not. Could you explain why some mothers do not drink all of their supplements while others don’t?**

R (Emily): Some get nausea when taking it and they get constipated. Pills for blood?

**I: Yes. Pills for blood.**

R (Emily): Yes. Usually they get nausea.

R: light headed.

**I: What about you guys? Do you get nausea when taking the blood pills?**

R (Emily): I think only few. Some do some don’t.

R: We don’t.

R (Emily): Well we all have different side effect for the pills.

R: Yes

R (Emily): We all have different crave. Some have cravings some don’t.

**I: Now what helps/motivates these women to take all of their supplements?**

R (Emily): They would usually say to eat sashimi to produce blood.

**I: Oh okay. What about regarding your medicines like when they’re nauseated are there ways that can help them drink all of the medicines?**

R (Emily): Nothing. Without the medication we’re okay.

**I: Oh okay. But what about other, are there drinks or food that you eat to help you drink the medicines?**

R (Mary): Eat sweet and drink cola.

**I: It’s good if you eat sweet?**

R: Yes. Like lollipop

**I: Now what is the consequences of having low blood during pregnancy and during birth?**

R (Emily): We get dark vision. We feel dizzy when we stand up.

R: Dizzy.

**I: What about during birth?**

R (Mary): We constantly bleed because we don’t have enough blood.

**I: How would that affect you and the child when there’s not enough blood?**

R (Emily): Oh we explain it, because these days is it not the doctors? We have to explain it?

**I: Well for instant the doctors they say the consequences of low blood during birth is this and that.**

R (Emily): Well they would C-section and give us blood.

R (Mary): Give us blood when we’re in labor (C-section).

R (Emily): During C-section.

**I: If they don’t give you blood what’s going to happen, what are the consequences?**

R (Emily): They say it’s bad.

R (Mary): We might get hurt and die.

**I: Okay when you say die, you or the child?**

R (Emily): Might be both. But the mother especially because she is the one they operate on to bring out the baby. And the baby is fine, but she’s in pain with her wound and her body.

**I: Now did you receive any advice from the nurses on how to prevent or treat low blood?**

R (Emily): Every time they would advise us, but our body is weak from the blood pills. This is why we eat sashimi.

R (Mary): Sashimi

R (Emily): So that it can make blood (produce).

**I: Now we will talk about feeding babies after they are born. After the baby is born and you start breastfeeding. Can you explain what foods are good and nutritious for woman who are breastfeeding?**

R (Emily): Drink soup, coconut milk, fish and I would say every kind of food. When we eat, we produce milk.

R: They have breast milk.

**I: What about canned meat?**

R (Emily): Canned meat?

**I: Yes if they said canned meat, what kind of canned meat?**

R (Mary): It’s like when we eat corn beef there is (breast milk).

R (Emily) There is?

R (Mary): Yes.

R (Emily): Well that’s because everybody is different from each other.

R (Mary): If I eat mackerel it doesn’t really make milk but corn beef it does.

R (Emily): So many.

**I: So you mean some say mackerel?**

R (Emily): Yes

**I: but other say corn beef?**

R (Emily): They have different food from each other. But I think the most delicious one is the coconut milk.

**I: So similar to what we discussed earlier about “food taboos” during pregnancy, can you describe any “food taboos” that exist for women who are breastfeeding? Do we have “food taboos” for breastfeeding?**

R (Emily): Yes we believe in breastfeeding.

**I: Well like we said earlier, if you eat eggs during pregnancy, your child will be a thief. What about you, what will happen when you breastfeed that will affect you or your baby?**

**R (Emily)**: When we eat sashimi, they say they bite our nipples.

R: Yes

R: Yes

R (Emily): If we lay down and breastfeed they will be inseparable from us. And those with big breast should not lie down and breastfeed because they might block the baby’s nose.

**I: What advice did you get from health worker about breastfeeding?**

R (Emily): Breastfeeding is good because the child won’t get diarrhea and the child grows healthy.

**I: We hear from some mothers that they started feeding their child other than their breast milk when the child was 6 months, but some started feeding earlier and some later than 6 months. Can you describe the reason why some mothers introduce foods or liquids earlier than 6 months?**

R (Emily): Well as for my grandchildren they didn’t eat at 3 months.

R (Mary): Me too.

R (Emily): It’s like you’re stretching the child’s intestine. But when or from my own belief because all mothers have different ways of parenting their own child. Every time they would say 6 but I would always wait until the child want to eat something then I would start feeding them.

**I: So you mean you can see signs of the baby wanting to eat more than just the breast milk.**

R (Emily**)**: yes

**I: Okay. What else or what about later than 6, why do some wait until 8, 10 or 1 year and then feed their baby?**

R (Emily): To grow what, teeth or something? I don’t really understand.

R: Because at 6 month, when they eat they have teeth.

**I: We hear from some woman that the first drop of the breast milk is not important but other woman say that it’s nutritious. What about you guys, what do you think about the first drop of the breast milk?**

R (Mary): I would also say it nutritious from what I believe.

**I: Did anyone explain to you guys what the first drop of the breast milk mean? For instant the doctors or the nurses.**

R (Emily): Yes. They say it’s good for the baby because it’s nutritious.

R (Mary): Good. They also say it’s nutritious.

**I: It’s nutritious?**

R (Emily): Yes. And some say they don’t breastfeed them because it just water.

**I: Why do they say it’s just water?**

R (Emily): It’s not as clear as the milk when it comes.

**I: Many mothers say that don’t have enough breast milk to feed their child. Can you explain how a baby under 6 months is fed when their mothers don’t have enough breast milk? When there is not enough breast milk what do you do?**

R: Bottle

R: Give the bottles

**I: Bottles and what else? Are there anything else you give other than bottles?**

R (Emily): Like what after 6 months?

**I: Babies that are under 6 months? When the mothers don’t have enough breast milk what do they do?**

R (Emily): Some they give bottle but other cook the rice and take the starch in the water and feed their child.

R (Mary): This is what I usually see some people do.

R (Emily): In a bottle.

**I: In a bottle? And they put it in a bottle. What else? Is there anything else?**

R (Emily): Maybe because at 3 months and they don’t have enough breast milk then they start feeding them.

R: Start feeding them.

**I: They start feeding them?**

R: Yes

**I: Okay. Were there any advice on how to produce more milk?**

R: Eat fish.

**I: Eat lots of fish**.

R (Emily): Woman have different breast. Some are dry, and some are full of milk.

**I: Why are some dry?**

R (Emily): According to their body.

**I: According to their body. Okay but what about their foods?**

R (Emily): Even if they eat a lot, they still won’t produce breast milk.

**I: What if you ate a lot of foods that don’t have nutrients, does that also effect the mother’s breast milk?**

R (Emily**)**: Yes.

**I: So you mean when they eat**

R (Emily**)**: No because every woman have different breast from each other.

**I: They’re different from each other? Now where did these advice come from like for instant “eat a lot of fish”, who or where did this advice come from “eat a lot of fish” to produce more breast milk?**

R: I don’t know but that’s what I usually hear, “eat fish eat fish.”

R (Emily): From our elders (ancestors) that we lived with from the beginning. And when we eat we know that it’s true (it produces milk).

**I: So you mean from the elders?**

R (Emily): Yes from the elders

**I: Can you explain how mothers in this community knows that it is time for babies to stop breastfeeding? How did you guys know it was time for you baby to stop breastfeeding?**

R (Emily): Our body starts feeling weak because they are breastfed a lot (some).

**I: What about others?**

R (Emily): Our body feels weak when we breastfeed. Maybe because there is no more breast milk but they just keep on eating.

**I: Are there any more?**

R (Mary): As for me they just stopped breastfeeding by themselves.

**I: They just stopped? Okay**

R (Emily): Others are shy.

I: Some?

R (Emily): As they grow older they feel shy to breastfeed. Especially places where there are people.

**I: Where there are no people?**

R (Mary): My youngest breastfed until he was in middle school.

**I: Yes there are. I hear that are kids like that. I usually hear that they’re about to start school but they’re still breastfeeding.**

R (Emily): There’s so many kinds. There are some that they’re about to give birth but the child is still breastfeeding. So when that baby is born they breastfeed him/her on the other side if not double it.

**I: What about others?**

R (Emily): Some.

**I: when they’re pregnant but are still breastfeeding? Do they stop?**

R (Emily): Yes some.

**I: do they stop?**

R (Emily): Some keeps going with breastfeeding.

R (Mary): They keep going.

R (Emily): Even though one is born but the other one is still breastfeeding. They don’t stop. Woman have different bodies.

**I: Some people say that they try to feed their children healthy foods (balanced diet). Can you explain what people mean by a balanced diet is? What is balanced diet?**

R (Emily): Foods that gives us energy.

**I: Energy foods?**

R (Emily): They’re healthy because they come together. You have to eat and then have desert.

**What about you, is balanced diet to you that you like to feed your child?**

R: …

**I: Whatever you feel like it’s good to feed your child.**

R: Good

**I: If you wanted to feed them, what would you feed them?**

R: Fish, rice and banana.

**I: Fish, rice and banana. What about you?**

R: Pandanas. I usually feed them pandanas.

**I: You usually feed them pandanas? What about you? What are balanced diet for kids?**

R: Pumpkin.

I: Pumpkin?

R: I also feed the oatmeal.

**I: Oatmeal? What about you?**

R (Mary): All the foods that are healthy for a child.

**I: Like what?**

R (Emily): Some prefer baby food in a can.

**I: Baby food in a bottle? The ones that you buy at the store?**

R (Emily**)**: Yes. Also cereal.

**I: What about when you make your own baby food, what do you make?**

R (Emily): We make them soft with a blender.

**I: The blender? Okay**

R (Emily): Because we usually baby food from makwon (pandanas).

**I: So usually you make baby food out of makwon (pandanas)?**

R (Emily): When it’s pandanas season yes. But not all the times are we going to have them.

R: If there’s none there’s none. When there is there is.

**I: For the last question, we would like to know how choices were made. Can you explain who influenced the mother about breastfeeding in this community? Why did you choose to breastfeed?**

R (Emily): It’s very different because some might don’t have money to buy milk. They have that much money to buy milk so some mothers that work have to go home to breastfeed.

I**: Why did you choose to breastfeed only?**

R (Emily): … It’s also good for the baby.

R (Mary): And it’s also good for us (mothers).

R (Emily): It’s like when we breastfeed, the ovary closes really fast.

**I: Yes… Now I would like to ask some questions about children who are sick. When children under 2 years are sick, some mothers and fathers take their children to the doctors first but others take them to the traditional healer. Can you describe the different reasons for this? Why do some take to the doctors first and some to the traditional healer?**

**R (Emily)**: Because in our belief, like us, we keep on taking them to the doctors but they don’t get well.

R: hmm (yes)

R: hmm (yes)

R (Emily): But when we bring leaves and soak them and bathe them, it helps a little.

R (Mary): Yes like that.

R (Emily): So many things. Like when they have the fever and you give them Tylenol and then you check their stomach and you find bumps (according to our custom).

**I: Now when they have bumps, what do you do?**

**R**: We massage them.

R (Mary): Massage them.

R (Emily): But it’s also prohibited to massage it right? But when we massage it goes away.

**I: Other than massaging their stomach what else do you do when the child has bumps?**

R (Emily): We give them liquid.

R (Mary): Give the local drinks (medicines).

**I: What kind of medicine do you give them?**

R (Emily): The coconut (the juice) you squeeze the juice, the banana (mokadkad).

R (Mary): And also, we sprinkle water over them.

**I: Yes, I’ve heard about that too.**

R (Emily): Oh, we can sprinkle, and it goes away?

R: Yes.

**I: What are the illnesses that are commonly treated with local medicines? What illnesses do you treat them with local medicine?**

**R**: Mejatoto

R (Emily): Usually skin rashes (kodkodi)

**I: Kodkodi. What did you say?**

R: mejatoto (fever that doesn’t go away)

R (Emily): When the fever doesn’t go away.

R (Mary): The fever doesn’t go away.

**I: When the fever doesn’t go away?**

R (Emily): Yes either one of them, mejatoto (fever that doesn’t go away) or stomach bump.

**I: Stomach bump?**

R (Emily): And also those, those children, like that child that has skin rash. Where the skin is really rash.

**I: That we believe.**

R (Emily): We call it jeba (skin rash).

**I: What kind of local medicines are used for these illnesses? Like how would you treat jeba (skin rash) with local medicines?**

R (Emily): They bathe them or give them liquid medicines.

**I: What kind of leaves/plants?**

R (Emily): Our plants like ekkon (traditional leave) with coconut meat

**I: Ekkon with coconut meat?**

R (Emily): Also neen kotkot (traditional leave) make them drink it.

**I: Also the neen kotkot (traditional leaves).**

R (Emily): We have so many.

R (Mary): The red Bukwor.

R (Emily): People have so many different kind of local medicines.

R (Mary): The red rock that floats in the water.

R (Emily): What is that?

R (Mary): Bukwor (red rock that floats in the water.

R (Emily): Oh. That looks like a square rock?

**I: How many of those.**

R (Mary): One of those. You bring them and make it.

**I: They bound it?**

R (Emily): Yes. Also called the bokeneep.

**I: Yes the bokeneep**

R (Mary): bokeneep (rock that floats in the water).

**I: Yes they also call it bokeneep**

**I: So who gives them advice when they need local medicines?**

R (Emily): Our elders.

R: Guardians

R (Emily): Our grandmothers.

**I: Who… what did you say? They say the elders, what about the doctors, what do they say?**

R (Emily): No they just give you medicines.

R: No

R: only medicines.

**I: They just give you medicines?**

R (Emily): They would just give us Tylenol and antibiotic.

**I: Now can you explain the difference between a child that is sick and a child that is not sick. The differences in their diet. If they were sick, how would you feed them from if they were not sick, is there any difference in their diet?**

R (Emily): Yes. They don’t like the foods.

**I: Okay. What else? … When your child is sick do they eat differently from when they’re not sick?**

R: Yes.

**I: How?**

R: We only feed them what they like to eat.

**I: They only… Oh so you only feed them the food they want to eat? Okay.**

R (Emily): They’re picky about their food.

**I: They’re picky about food?**

R: Yes.

**I: What about liquid?**

R (Mary): If it’s liquid, they like cold water.

**I: It’s like they only like cold water?**

R: Yes.

R (Mary): They don’t like warm.

**I: Okay. Is sweet drink okay or just water? What do they like when they’re sick?**

R (Emily): Cause when they’re hot they like water.

**I: When they’re hot they just like water?**

R (Emily): Hmm when they have the fever.

**I: What about when they get nauseated?**

R (Emily): Well they hate water and everything.

R: They don’t eat.

**I: Like they just don’t want to?**

R (Emily): They just feel like it.

**I: So what about when they’re not sick?**

R (Emily): Well they just like everything.

**I: They eat everything and drink everything?**

R: Yeah

**I: Now I would like to learn about the food you provide for your family? Could you talk about what influences which foods people in this community provide for their families?**

R (Emily): Some family depend on their budget. Some are weak where they only eat rice and meat. Here in the Marshalls islands, some usually eats jaibo (Flour, water, sugar and coconut milk) and jokkop (flour, breadfruit, water, sugar and coconut milk). But others who have a lot, they eat vegetables and fruits. Everybody is different from each other according to our culture.

**I: So you mean we’re depending on money?**

R (Emily): Budget. Yes.

**I: Can you explain how people get, no, can you explain the difficulties in getting the food you want for your family**?

R (Emily): That’s when there’s not enough budget.

**I: Not enough budget… What else?**

R (Emily): What can I say? This is the only thing.

**I: So you mean this is the only reason because there’s not enough budget?**

R (Emily): Not enough to look for…

**I: Bring what you need for your family?**

R (Emily): Yes

**I: Now how does family deal with food shortages, for example sharing foods or buying from the stores?**

R (Emily): Just by working and when they have enough they go and by rice and meat just so they don’t run out.

**I: What if there’s like no more and you only have little money with you?
R (Emily): At least there’s enough rice for the next day and quarter leg. But when there is no more than we go around to our family and ask for help.**

R: Hmm (Yes).

**I: Yes. Like long time ago. What if you had little money and you went to store, how do you budget it?**

R (Emily): If what?

**I: If you had little money but you go and buy from the store?**

R (Emily): I would say like if there I can get 1 rice than we’ll survive for 5 days.

**I: And one case of.**

R (Emily): Quarter leg so that you can stew until the next week when there’s no more.

**I: Okay good. Now many families have told us that fresh vegetables are not affordable. Can you describe any other reasons that families do not eat many fresh vegetables?**

R (Emily): Its all out because of just buying rice

**I: The chicken**

R: The chicken

R (Emily): No more money to buy vegetables.

**I: No more money for it?**

R: Yes.

**I: Are there any more reason why you can’t afford fresh vegetables? Maybe because not enough budget.**

R (Emily): Not enough budget

R: Not enough budget.

**I: Not enough for**

R: Enough for.

**I: What about the cost of fresh vegetables?**

R: What?

**I: What bout the cost of fresh vegetables?**

R: Cost of what?

**I: For instant the cost of the fresh vegetables?**

R (Emily): If you buy the fresh vegetables now, it’ll run out fast, you won’t find any rice tomorrow. If you eat apple now, you’ll be hungry later because there is no more rice. Right?

R: Yes

R: Right

**I: Now is it expensive or cheap for you to buy it?**

R (Emily): What

**I: The fresh vegetables is it cheap or expensive for you to buy it?**

R (Emily): Kind of cheap but there is no more budget.

**I: Cheap but no more budget.**

R: Only enough for somethings.

**I: Now in this section, we would like to talk about water and hygiene. Can you please describe how people typically get water for their families in this community? How do you get water for this family or for this community?**

R (Emily): We get water from our water catchment.

**I: Rain.**

R: Yes

**I: What else?**

R (Mary): Buy water

R: We usually wait for the water to open (MWSC).

**I: From where? Where do you buy it from?**

R: The water place, the store

**I: From the water place and the store and what else? Where else do you get your water from other than?**

R (Emily): Water from MWSC.

**I: MWSC**

R (Mary): From the Well

**I: The well. Now it says, do you have difficulties in getting water? What is so difficult about getting water?**

R (Emily): For instant the well you have to fetch water with a bucket.

R (Mary): But when its low tide there is nothing inside.

**I: When there is low tide there’s nothing inside**

R (Emily): Nothing inside.

**I: Okay**

R (Emily): When there is no rain its empty.

R (Mary): Water catchment is empty.

**I: Okay.**

R (Emily): When we don’t have money, we can’t buy

R (Mary): We can’t buy.

**I: Yes. Now are there difficulties in storing water?**

R (Emily): If we don’t have water catchment then we won’t store.

**I: Okay what else?**

R: We need water catchment to store water.

**I: We’ve heard that some families boil their water for drinking but others do not. Can you describe why some people boil their water while others do not?**

R (Emily): Because some don’t care but others care about their health.

**I: Care about their health?**

R: Yes

R: Yes

**I: What else?**

R (Emily): Like what? Some are messy and some care?

**I: Some are messy and some care? So now are there any difficulties in keeping the water catchment cleans?**

R (Emily): There are difficulties when you must fetch out the old water and open the cover and climb down and clean the bottom.

**I: What are the some of the things that makes your water catchment dirty?**

R (Emily): Our tanks.

R (Mary): Our tanks.

R (Emily): Our rooftops

**I: What’s wrong with the rooftops?**

R (Emily): Sometimes the animals goes on top of the roof

R (Mary): Dirty and dusty

**I: Okay what else?**

R (Emily): The trees over our house.

R: Dust

R: Dust

**I: What about the tins?**

R: They’re rusty

**I: Rusty? Now we hear that some family wash their hands all the time but others don’t. Can you explain why some wash their hands all the time while others don’t?**

R: Some are not use to their hands. They’re used to their dirty hands. They weren’t taught.

**I: They weren’t taught? Okay**

R: (Emily): Not use to it.

**I: Not use to it? Now why do some people wash their hands with soap and water and others do not? They can just wash their hands without soap?**

R (Emily): Maybe they don’t have soap.

**I: No soap? No money for soap? What else? Yes, that could be it. Okay. Is there anything else why some** **don’t wash their hands with soap?**

R (Emily): That’s why we say lazy.

**I: They’re lazy?**

R: Hmm (Yes)

R (Emily): When the soap is not in the washing place, then they just hurry and go.

**I: What are some of the things that prevents you from washing your hands with soap daily?**

R: Refuse

R: Don’t want to

**I: Refuse and what else**

R (Emily): They don’t care about their health.

**I: You care about your health?**

R (Emily): We say if you end up sick then you’re sick.

**I: What about our habits?**

R (Emily): Also, our habits. We are used to these.

**I: Why is it that all of a sudden, it’s important to wash our hands? Why**

R (Emily): After the Americans came and taught us how to wash our hands now we know how to.

**I: About the importance.**

R (Emily): Now we know the importance of washing hands

**I: Yes. Now why do some people use hand sanitizer instead of soap? Why?**

R (Emily): Because they can put it in their pocket and when they go places where there’s no water they use it.

**I: So you mean when you go places**

R (Emily): They take it with them

**I: They take it with them and instead of using soap they can use the hand sanitizer?**

R: Yes

R: Yes

**I: Now for the last question, we would like to learn about how parent care for their children. We’ve heard that husbands are an important support for their wives during pregnancy. Can you explain what husbands do to support their wives while they are pregnant?**

R (Emily): They help them and cook them foods.

**I: Okay**

R (Emily): They want to carry buckets

**I: when they fill up buckets for you?**

R (Emily): They want their child to be safe and not in harm or what? Some have love in their hearts.

**I: Yes. What else? What did your boyfriends do to you guys when you guys were pregnant?**

R (Emily): Well some men are different, some are busy sleeping over there but some are busy with chores

**I: Different from each other?**

R: Yes

**I: You mean all men are different from each other?**

R (Emily): Not all are the same. Some don’t care.

**I: Now if they care, what do they do to you? For instance, the men that care about their wife and the child inside her stomach? What do they usually do?**

R (Emily): They broom, they clean, they cook and help look after the kids.

**I: No what do mothers or other family members do to support their daughters while they are pregnant? Like you grandmothers, what did you do to your daughter when they were pregnant?**

R (Emily): You make sure that they go to the doctors

R: Make sure they go to the doctors.

**I: Okay.**

R (Emily): Make them move a lot.

**I: Okay.**

R: Make sure they don’t go out after dark.

**I: Why don’t they go out after the dark?**

R (Emily): They say might get bad spirit and they might not know how to give birth.

**I: Okay good. We are also interested in learning how caregivers play with children under 2 years. can you describe for me in details how you play with children under 2 years old? What do you do with them**

R: Sing to them

R: hmm. (yeah)

R: Do action.

**I: Do action and sing?**

R: Yes

R: Also teach them.

**I: Teach them what?**

R: Like my son, I usually teach the song, “Head, shoulder, knees and toes.”

**I: Oh yes. Good.**

R: Yes

**I: Okay. What else?**

R (Emily): Play kids songs

**I: Okay but how does these… sorry. We hear that some parents spend time outside of the home and it may affect the way they raise their young children. Can you tell me about your experiences with this?**

R: Yes there are lots of different kind of mom and dad. There’s a lot. Some leave their kids and don’t have time.

**I: Okay**

R (Emily): Some they really care about their kids they stay with them

**I: Stay with them?**

R: Stay with them

**I: Okay, what else?**

R (Emily): What else can we say? That’s about it.

**I: Now how does these affect the children under 2 years?**

R (Emily): Some are sad, and some are happy. Children who don’t have their parents near the are naughty and they don’t teach them. And those that are always with their parents I would say

R: I would say they know how to listen than other kids

R: Even though they’re with their parents, but because they say our medicines has gotten to them and has made them naughty.

**I: But how does it affect the hygiene of the children?**

R (Emily): They’re clean because they’re mom is there is clean them up.

**I: What about when they’re not by their side?**

R (Emily): Dirty

**I: What happens when they’re dirty?**

R: They’re really sick.

R: They’re sick.

**I: Now what do women do outside of the home? When they’re away from the house, what do they usually do?**

R (Emily): They always go out.

R: They always play bingo.

**I: Out, Bingo. What else?**

R (Emily): Many things like you know.

R: Things like drinking.

**I: A mother is different from another mother.**

R: They’re different from each other.

**I: What do fathers usually do outside of the home?**

R (Emily): Some they. We’re talking about away from home? Some sleeps, wake up and go but some clean outside the house.

**I: Now as they are away from their family, what do they do? Why don’t they just stay with their family?**

R (Emily): Because there are different kind of father. They go and get drunk and go and look for girls.

**I: We hear from some people that they prefer to get health messages from the radio, others say from the newspaper. Can you describe for me the best ways to reach people with information on health in this community?**

R (Emily): What can I say because we have a habit that even if we hear, they don’t care. Their own love to themselves, because the good but they don’t do them. They know right and wrong. We can say their own feelings.

**I: What if they don’t have radio and they can’t afford to buy a newspaper? What can you do to make a better way to give out these information about health and.**

R (Emily): Like I said, their love will give everything for their family that will make it better. If there is no love in a family, they wouldn’t care about health, food and things like these.

**I: What’s good about internet to get information about health?**

R (Emily): Internet? It’s good because they could go on google and search for things to get information

**I: Okay, are there any group in this community that could be a good place to deliver health messages, for example women’s group or mother’s group?**

R: Like I said, they’re really smart but they just don’t care. If they don’t want it, they don’t want. If they want it, they want.

**I: Anything else over there?**

R (Emily): It depend on a mother who can care for a child or not. There’s a lot of different kind of people in this community. Some know how to care for their child, some don’t. And whoever knows how to care for their child will know how to make this community healthy. And whoever doesn’t know how to care for their child won’t care.

**I: You mean they won’t care to deliver messages information to people.**

R: Yes

**I: You mean they wouldn’t care about how they would be delivering messages from someone who is happy to advice other.**

R (Emily): Yes. We advice them but they don’t want to listen.

**I: They know but they refuse**.

R (Emily): Its up to them. Yes.

R: Yes.

R (Emily): You keep teaching them, but they don’t want to. To the point where you have no power.

**I: That’s great we’re done. Thank you guys for the information you have provided today. We hope and believe that the information you provided today will find ways to improve programs for mother and children especially grandmothers that care for children because we know that they have the knowledge. And its really great that we have two grandmothers that joined us today to tell us what they see today in this community. Information that we did not know about now we know. Its great because they can teach the younger generation.**

R: You mean lifestyle

**I: Yes lifestyle on these islands. Because every country has different culture from each other. So thank you guys.**

R: Thank you too

R: Thank you.
