# Supplementary material for: Conceptualizing multi-level determinants of infant and young child nutrition in the Republic of Marshall Islands–a socio-ecological perspective
Source: PLOS Glob Public Health. 2022 Dec 19;2(12):e0001343. doi: 10.1371/journal.pgph.0001343 (PMC10022247; doi:10.1371/journal.pgph.0001343)
Supplement: S1 Data — (ZIP) [file pgph.0001343.s001.zip › RMI Supp Data/Focus groups data/F08R_FGD_Male_Arno_Sep 26_Balton_Shante.docx]

- **Interview Code: F08R**
- **Interview type and Interviewee: Male FGD**
- **Iterview Date: 9/26/18**
- **Location: Arno**
- **Interviewer: Balton**
- **Transcriber: BM**

**I: Okay, the reason why we are here is to find out how we can help our children have a better life. We are talking about children 6 months to the year under 2. Between those age they can easily get affected with illness like diarrhea and things like that, which means that their body won't grow to how they were so supposed to grow into as long as they have diarrhea. And we're going to have a conversation and tell each other stories and see what we can brain storm. I came with a few questions and those question are about foods that we eat, and also drinks, and illnesses that usually happens from day to day and also what families do with their children. Every answer that you will give, there is no rights and wrongs every answer’2s you'll give is all good. We just want to know what you know about what's happening to children here from day to day since you are the one who take care of the child. It's important to know what you've learn from the doctors about children. Since the doctors sees what's happening with children with their reports. Our first question is about the foods that the children eats and their security. What in the community does the people do to feed their children? What in the community makes people choose the foods for their child? For example, you know like the thing that would make you say wow this child is eating this and that instead of the once that they chose flour and rice what makes a family so that they can feed their child? How can I give an example? Here's one example something that makes people eat other kind of foods that would make them not eat the other kind of foods like formulas and you these things that are in a green bottle for children this is just for an example. Like for you guys what would make you guys do to feed your children with foods on a daily basis?**

R: Well that depends on budget.

**I: Budget? Depends on budget?**

R: Hmm

**I: Umm, when there's no money right for budget and stuff like that, what kind of foods do they eat as in what kind for little children?**

R: Little children under 1 years old? right?

**I: Ah,**

R: Like little children that hasn't eaten and they're still breastfeeding.

**I: Nooo, they're already know how to eat.**

R: Well then that would be foods that are chewy like pandanus, smooshed pandanus, pumpkin, you pumpkin that they would make eat chewy, they would usually eat bread and mix it like a soup, but the thing is there's no meat in it. How do we call that again?

R: Jaepo(Soup)

R: Jaepo(Soup)

R: Jaepo(Soup)

R: Yes. jaepo so that they can dip it with bread. Not jaepo rara (sand worm). (Everyone laughs) I think those are the only foods that we can feed them. But right now, there is no more.

**I: Oh, it isn't time for their season?**

R: It's not time for their season. But right now, my child is 2 and my child is eating for now, my child is eating jaepo. Usually jaepo other than any other foods, jaepo. Sometimes soup rice.

**I: Oh, so what you're saying is that they would eat these foods because pandanus and those kind foods isn't their seasoning yet.**

R: (Everyone at the same time) That's right.

**I: Now during this time that it's not pandanus season, what would you feed your child? Jaepo?**

R: Yes. the kind like jaepo, soup rice, sometime if we have money we would buy biscuit and things like that like nutrition.

**I: Now this question is asking what would make you choose the foods other than because of low budget like there's no budget then you would make local foods? Like if there's no pandanus now there's these 2 problems that we would face which that would be when there's no foods that are grown home and low budget**

R: The closing thing that we can do is rice and flour. Since they can last longer. Is what I say is correct? (Laughs) (Everyone laughs)

**I: There's no right and wrong answers. Ahh, one thing or you know last week we made a chart and talked about with everyone and there's not ah, people in the Marshall they would see that we don't eat much green, like beans and vegetable that are green. The reason we would eat these like in Majuro we would eat these because there in a hamburger and there's cabbage and things like that like for ramen there's this small bag that has mini vegetables. But the truth is that we don't usually say oh I'm going to eat green beans because now our leaders are asking why we're not usually eating these things that are green like lea and these things.**

R: Maybe its because we don't have those kind of things and it's probably because we weren’t used to them. like if it's about lunch there's only 2 things we would put on our plate, rice and the meat.

**I: Rice and meat?**

R: There's nothing else. Maybe if it's beans we don't have any of those around here.

**I: Umm, I don't know if this happened here on this island but in Majuro, Maloelap, and Jaluit during the reign of the Japan. They said that the Japanese would take our local foods because the American took down their supplying ships, so they got hungry and they told the Marshallese that the fish is their fish and the plants are their plants like pandanus and plants that are edible.**

R:(Everyone) Hmmm

**I: Then the Marshallese had to eat leaf’s. Is there any leaf’s that are edible? Do you guys know anything about it?**

R: Starch.

**I: Yes, like starch.**

R: Well yes, we would eat starch, but yes, we would see them eat the leaf’s, but we also saw some kiribati eating a banana leaf’s.

**I: Leaf’s of bananas?**

R: Yes. Soup, there are some that would eat that, but they would like eat the soup of a pumpkin. Well we wouldn't eat it because when they're over grown we would rather mix it.

**I: But us we don't eat?**

R: We don't eat. I don't back then what they would mix, what?

R: Pumpkin leaf’s.

R: I know about the leaf’s of lime but I know that we only mix that to make tea.

**I: Oh yes.**

R: (Laughs) But when it comes to foods I know about coconut meat (cotton meat) and coconut juice but there was one that ... I forgot what's it called. We just saw it nowadays, we know it's not from around here in the Marshall Islands.

**I: What is it?**

R: I don't know how they call it, it's almost like that plant...

**I: Ringway?**

R: Yes, and they mix it with.

R: They make soup.

R: Chicken.

R: They can make soup out of it.

R: They would extract the water from it.

R: Well that plant is from somewhere, but they brought it here. We saw some Philippine that came here and mixed it.

**I: oh… I haven't seen that.**

R: Maybe because what's that called again?

R: There is only one person that planted that that, Mario.

R: What, it has a lot of vitamin?

R: But what is called? Isn't it pele?

R: Pele

R: Yea it's pele.

R: Pele.

R: Yea.

R: It's between those 2 plants.

**I: oh… and only the workers here eat it?**

R: No.

R: No.

R: Not really.

**I: Anyone can eat it?**

R: Well not everyone knew about that.

R: Well that was there since 2010.

R: Some Philippines were the ones who came here and planted it.

**I: The Philippines are the one who planted it and only in this area that has it?**

R: Yes.

**I: Oh man, seems to me I want to see it.**

R: Do you have a box?

**I: What?**

R: Do you have a box?

**I: Box.**

R: Yea.

**I: Yes. I do.**

R: Well you can bring it, so I can cut a root for you.

**I: Hm… well that's a great plan. Do you guys sell plants, what do you guys sell like bananas and things like that.**

R: Yes, but not often.

**I: Not often?**

R: Yes.

**I: Where do you guys sell them?**

R: Majuro.

R: Majuro.

R: If we're going to take a few days there then we would sell them there at Majuro.

**I: Oh, so that would be like your pocket money?**

R: Yup.

R: For taxi and kind of like that. (Laughs)

R: That's the word.

R: The kinds like banana, coconut meat (cotton meat), coconut juice, well that's my understanding.

**I: These are the things you usually sell?**

R: Pandanus.

R: And pandanus

R: And lime.

**I: And lime? Oh, yeah. it seems that this island has lots of lime right?**

R: (All) Yes.

**I: From Ine to here, just lime. Umm, one of the things that we're also seeing from last week umm, there's a lot that wants to make a garden but there isn't any tools right** **what is preventing it?**

R: Tools for working.

**I: Tools for working?**

R: Well when it comes to making taro it's a big work to do. We would need to dig a hole, and if there's only 2 men at the house maybe it would take half of them to be digged, because they need to dig a line for them.

**I: Hmmm, so it takes time and it need tools and stuff like that.**

R: But another thing is that we wouldn't think about that because we take up all our time collecting coconuts. If we distract ourselves planting in our garden instead of collecting coconuts, then there would be no rice. We want to have these but there's no time like if we don't collect coconuts over a week or like 3 days well then, we would have no food. That's the only way, if they need foods in the house it's by collecting coconuts.

**I: Thank you for giving us all this information. But yes, we want to know why like for a day a person would do one thing other than doing other things that can help provide for his family. Does it show, help from other things but because the other important thing is focus on collecting coconuts. What main source is the income for majority here?**

R: Collecting coconuts.

**I: Collecting coconuts?**

R: (Everyone just nods)

**I: Oh yea, I see a lot of small fences you know the red ones?**

R: Hmm

**I: Umm, those are for chicken right?**

R: Yes

R: Hm.

R: Yea.

**I: And does this island raise chickens?**

R: Well we don't anymore. There were, but let's just say that this started from I don't know probably from Australia and those other places. But let's say that a group of wome’s brought this but not every households has this. There were chickens in them.

**I: Now that you have chickens they're not in those fences? You guys just let them free?**

R: Hm

R: Every chicken that are now here on this island they stay out of it.

R: They're not in fences.

**I: Ahh, one of the things we've learned about some question from last week it showed that when we ask what kind of foods you usually eat. and we would see that there's no eggs. Do you usually eat eggs from the chickens?**

R: Well that's one of the things, like if it was me I would get an egg and eat it and my granddaughter would ask grandpa why, would you eat that when you could’ve just left it so that there can be more chickens so that we can cook more and eat them. It's true that we don't usually. or we don't anymore.

**I: Don't anymore? They're trying to make more chickens?**

R: Yes, and so that there can be more chickens. But making eggs there's none, also cooking eggs there's no one doing that.

**I: Just raise chickens?**

R: Just to raise only.

**I: Umm, but this program what is it for, raising or making? This program that made this fencing project?**

R: There was a reason why this program made this project and it was also for making eggs and making chickens at the same time.

**I: Oh, double?**

R: Hmm.

**I: Umm, I asked this question and it was very nice how you explained it to us. Who is responsible for the animals? We would see that they wouldn't stay put they would roam around in their fence. But when we came here there are a lot of animals roaming around, is there any difference? but now there's a law that animals should be in a fence. Why are they roaming around? other than say that they don't have a fence?**

R: Well when we look at it when they're in a fence it's like they're not living well as how they live out there because when they live out there they would eat all sorts of things and when they're in fence they would only eat coconuts, like morning coconuts, afternoon coconuts, and evening coconuts. And it looked like they're having a bad life.

**I: Just coconuts?**

R: If there's leftover foods then we would feed them but there aren't usually leftovers. (Everyone laughs).

**I: Yes, but when they're free?**

R: Well then, they would have a better life. Maybe because when they swim in the mud it would cool them but when they're in a fence we would forget to wash them to cool them down because we're busy with our other chores.

**I: Good, thank you your answers were good. Now we're going to talk about water. How do you ahhh (people coming in) other people would use these things that children drink they would boil the water, and some wouldn't. some would say that they would fill it up from water catchment and some would say you like this bottle here they would fill it up. What are things that would make some people take some water and some wouldn't? For little children.**

R: Well maybe from busyness.

**I: Always busy?**

R: Hm.

R: Because maybe if it was child’' father I could babysit for him but when it comes to saying we need this and that then he would go and collect coconuts. He needs to focus on collecting coconuts so that there would be, but now if it was the mother to watch over the child, she won't have any time to boil, but maybe if they have time to boil they would boil but they're always busy. There's a lot of fire woods we wouldn't say that there aren't any but there are for boil.

**I: Is there any water catchment in this island?**

R: Yea like that kind.

R: There is.

R: Water catchment.

**I: Every household has water catchments, or some have and some are waiting for theirs?**

R: Hmmm

**I: Does everybody have these gutters?**

R: We have gutters.

**I: Do they have these what they're called again?**

R: Filters?

**I: Yes, do you filter for your gutter?**

R: I believe we don't.

R: We only place the filters at the end of the gutters.

**I: Oh, you mean the filter that is at the point where all the water is falling into the water catchment?**

R: Hook.

**I: The hook, oh. They say that some would use tablets right well the tablet it made of chlorine and they would throw it into the water catchment so that it would kill the bacteria and all that. Is there anything like that on island or do you guys just use chlorine?**

R: I think there's none.

**I: So. you guys use the liquid chlorine. How often do you guys use it to clean the water catchment?**

R: When it's all empty.

R: When it's raining. (spoke at the same time)

**I: What?**

R: When it rains a lot.

**I: The days when it's raining, that's the time when you guys clean the water catchment?**

R: Hmm.

**I: Hmm. Why when you clean the water catchment, when you done washing the water catchment how long does it take for it to refill?**

R:

**I: Ahh, the other question is about hand washing. Because umm, everything that we feed our children, we feed them with our hands. When do usually ah, other than taking a bath when do you guys usually wash hands?**

R: Probably when it's time to eat.

**I: Before eating the meal?**

R: Morning, afternoon, evening. Before eating the meal probably everyone knows that they should wash their hands, that's the could be the only time.

**I: Those time?**

R: Other than when we touch these things and we know that it’s very dirty and you would look and see that its very dirty, well than we can wash them.

**I: When do we use soap when it comes to washing hands?**

R: When it's time to eat.

**I: When it's time to eat.**

R: Before we start eating.

R: And after we're done eating.

R: After eating we would wash away our dirty hands after eating.

**I: For soap, is it hard to find on island? Like Lukoj, if I would live there is there any store there?**

R: Well for me, I really don't know if there's any stores there. I only know there were none a few months back. but I don't know if there's any nowadays.

**I: Oh.**

R: What about Benny’s store?

R: Well I don't know about that.

R: Yea there's a store there.

R: It just opened nowadays.

**I: You know small houses and like this house there's probably like almost a lot of stuff in it and in Majuro there are plenty of stores like that and you would usually find them. Like if there were stores like that here, would there be any soap or are there a lot of soap in the store?**

R: Yea.

R: Yea.

R: Yea.

**I: Ahh, there's this thing ahh what's it called ahh, hand sanitizer is there any on island? Do they sell any on island?**

R: I there's none.

R: None.

**I: Oh, so it's usually soap. and things like that. Majuro, when was it that it was over loaded with those things? Other than just soaping their hands they would find it in hospital and government buildings and on the counters when your about to talk to that person. Ahh, our next question is about people defecating the ocean and lagoon side. Is this still a problem here, like in Majuro everywhere did this practice but nowadays it seems to lessen in Majuro. Is this island doing the practice?**

R: Hmmm.

**I: What is it that makes people here defecate the ocean and lagoon side?**

R: There's no place.

R: Some places don't have toilet facility, but I think it's probably a habit. a habit to defecate the ocean and the lagoon.

**I: Defecating the ocean and the lagoon?**

R: Some they have toilet facility, but they'd rather use the ocean and the lagoon side.

R: There's no question that children, every children’s would defecate the ocean and the lagoon. Other than using the toilet it's just that it's a habit.

**I: Is there any households that has this kind of toilet.**

R: Yes. they do. But like I said, when the child says hey mom my stomach hurts, and the mom would say ahh run quickly to the lagoon side. (everyone laughs) But I would be busy doing my work I wouldn't tell my child to quickly go to the lagoon.

**I: Oh.**

R: That's not the only thing but they usually go to the lagoon so that they would swim.

**I: You mean they would wash themselves in the lagoon?**

R: Hmmm.

R: Like I said maybe because of habit.

R: You would grow up with that habit.

**I: Growing up with that habit.**

R: Hmm.

**I: Hmmm.**

R: Well also because the mother is also busy she doesn't have time to wash her child. That's probably why it's better to use the lagoon because it's faster and like a shortcut.

**I: Oh. The schools, is there any toilet facilities?**

R: Yes.

R: But it's not enough.

**I: Not enough?**

R: Yea there should be more.

**I: Okay thank you. Ahh now... You know what kind of illnesses does the children usually get? It's usually what, what kind of illnesses would they first get? What do they usually take? Not just your children, but every child in Arno Arno.**

R: Well that would be flu and diarrhea.

**I: Flu and diarrhea?**

R: Hmm.

**I: Hmmm. are there any illnesses that can pass on to another?**

R: Well things that are like pink eyes. There are times. Pink eyes are probably spread because of the wind. And probably because we stare at each other.

R: Yes. there's sometimes pink eyes but also diarrhea and amoeba, people would easily get catch it from others. But not very often but the thing that usually happens is diarrhea.

**I: Diarrhea because of unwashed hands. are there any illnesses that they would get sick from and do you guys take them to a traditional healer?**

R: Yes, stomach bump.

**I: Stomach bumps?**

R: Yes. We know that they would have stomach bumps when they have a fever and

**I: Oh.**

R: We would give them medicines and still yet they're not getting better.

R: Like if there was something on their body we would take them to have traditional medicines.

**I: Hmmm. Now that when your childrens are sick who do take them to first? The mother or the hospital or traditional healers? Who do usually take them to first or does the the father chooses who to take them to first?**

R: All of the above, some places would take them to their grandparents first but some places their parents would take responsible for themselves. But some would be like oh grandma the child is sick, and she would be like give medicine to the child and when if it's not working then she will say, take the child to the doctor.

**I: Hmmm.**

R: They would know, what to give them like if it was rashes

**I: Rashes and things like that.**

R: The doctor would like oh the child needs this medicine or that medicine. Like if the child is sick where do you go first?

R: The parents.

R: Or the grandparents.

R: Hmmm that is all thank you. (everyone laughs)

**I: Another thing that we also saw is with the women. And what we saw is that when the women are sick they tell us that it's important that the man should be there and help them. They said that it's really important if the man would support them. Like we would ask what is it that they need, and they would answer oh what we needed the most is that our man should be there for us and this is of the top things, other than the mother helping the grandmother the father should take part in too. Now the question is asking what is a man responsibility when the mother is sick?**

R: (everyone is laughing while talking) Like what when their pregnant?

R: Well these guys should know because their women would want to cuddle with them. (they laugh even more)

R: Well it's too bad our son ruined it. (everyone continues to laugh even louder) But the way we see it, I'm serious, like if a father like this guy if they needed him there's nothing that he would do just go and lay down with her and cuddle. Then there's no work that he has finished. Just walk straight to her and lay down next to her but I don't know probably there's a spirit possessing her I don't know.

R: And he doesn't collect coconuts.

R: So. he would be next to her from that day to the next day and yet there will still be no work done.

R: (laughs) No coconut and stuff like that.

R: Instead of like massaging her legs and stuff like that she'd rather make him lay down with her. (everyone laughs) Is that true guys?(everyone laughs again)

**I: What do the women usually eat when they're sick?**

R: There's a lot of things.

R: Like if they want to eat the fish that is always next to the shark then, we have to get it. (everyone laughs)

R: Some would hate eating because they would rather cuddle.

**I: Huh? Oh.**

R: But I know that they would crave for a lot of different kind of foods.

R: What they usually crave for is like cookies, chocolate, and cocoa. They don't usually say oh I want to eat papaya and pandanus it's more they want to eat imported foods. They would like okay, bring 1 reo.

R: So. we got to complain so that they don't bring oreo to this island. (everyone laughs)

**I: It's more like the mother would look after the children while the father is collecting coconuts. Other than that, what else is a father responsibilities on child care? Other than collecting coconuts what else can a father do?**

R: Probably the most responsible thing the father can do is watch over them and distract them so that the mother can finish her work. Like if the mother wants to cook well, then the father needs to watch over the children. Probably those are the time the father would help the mother.

**I: Those times?**

R: Hmmm.

**I: Those times when the mother needs to finish up her chores?**

R: Hmmm. And sometimes when you feel it you would take your child, but it won't take for a long time.

**I: It wouldn't take a long time. How do older kids play with younger kids under 2 years old? How do they watch over them?**

R: Sing

R: Play around

R: Talk

R: Probably the only thing they would do most is play around.

**I: You know, I don't know if there's any on island but in Majuro there are kids that don't have a mother and father near them. Umm, is there and child like that on island? It wouldn't be because their parents ditched them, but it could also be like their parents are working in Majuro. Children that don't just live with their parents but their living with their…**

R: Grandparents?

**I: Grandparents.**

R: I believe there are.

**I: Is there any children like this ahh you the community do they help the family looking after the children like that?**

R: If it was the grandparents of the child or parents of the child and they're at Majuro well they would take them in and

**I: And they're the taking responsibilities?**

R: take responsibility.

**I: And like the responsibility of buying them clothes and feeding them. who is taking this responsibility?**

R: Their grandparents.

**I: The family that's watching over them?**

R: Hmmm. They would ask help from their parents but probably they don't have much and when they do then they would.

**I: Does it show here like in Majuro there are plenty. Does the mother here leave their child at home and the father then has to watch over them?**

R: I think you've asked the right question to the right guys. (everyone laughs) Yes, sometimes.

**I: Like what would be reason why they would do it? Like if it was Majuro than it would be because of bingo.**

R: Well nowadays they would look for Philippines. not Philippines as people but the Philippine movies.

**I: Oh (everyone laughs)**

R: Last time there were 5 women that came from Majuro and there was this group they would go to where the women are. They were going to watch movies with them or have a conversation with them.

**I: But now what about men? What would make men to move around?**

R: Well in the morning that would be coffee.

**I: Coffee.**

R: If it was in the morning you would see most of us outside the store drinking coffee. But the thing is that us men know our timing.

**I: This island is really good there's no problems like oh the guys went drinking because of your law. Now for the last question for today, where do you think information about health and nutrition should be?**

R: Hospital

R: Hospital

**I: Hospital. Is there any where else besides hospital?**

R: Yes. we would also hear it from the radio.

**I: Radio?**

R: Hmmm.

**I: Only these 2 that are usually heard/seen?**

R: The reason why I would know about those information is because of the doctor and V7AB.

**I: V7AB, the health program, right?**

R: Hmmm.

**I: Okay it's all good thank you all for coming here and giving us your time to take part in this survey.**
