# Supplementary material for: Conceptualizing multi-level determinants of infant and young child nutrition in the Republic of Marshall Islands–a socio-ecological perspective
Source: PLOS Glob Public Health. 2022 Dec 19;2(12):e0001343. doi: 10.1371/journal.pgph.0001343 (PMC10022247; doi:10.1371/journal.pgph.0001343)
Supplement: S1 Data — (ZIP) [file pgph.0001343.s001.zip › RMI Supp Data/Focus groups data/F09R_FGD_Female_Arno_Sep 26_Libon.docx]

- Interview code: F09R
- Interview type and interviewee: FGD_Female
- Interview date: Sept. 26. 18
- Location: Arno
- Interviewer: Libon
- Transcriber: Marcellina

**I: okay before we proceed, do you ladies want to participate in this survey?**

R(all): yes

**I: okay good, thank you all. We will now move on to the questions. The first question says, can you describe what a healthy woman’s body should look like?**

R: what can we say, it’s like this?

R: her shape

**I: how’s the shape of a healthy woman?**

R: thin… like as thin as of who?...

R: don’t look for a thin shape here

R: she loves doing works

R: energetic and fully active, never feeling sleepy,

R: not like some of us; overweight and fatty and like to just sleep.

**I: what else?... are there more?... now as you said thin, can you describe how thin is she?**

R: not really thin, but not really big

**I: now what is a healthy and nutritious diet for pregnant women?**

R: pandanus, local foods, crabs, pumpkins, bananas, papayas,

**I: what about sea foods?**

R: fishes, lobsters,

**I: now it says, are these foods different from the regular foods specific women usually eat?**

R: yes

**I: can you describe the difference in these foods from the foods eaten by pregnant women?**

R: because some pregnant women want to eat unhealthy foods like ramen. They don’t want to eat sea foods like fishes, octopus, and these kinds of food.

**I: and when they’re not pregnant?**

R: they eat all kinds of food

**I: now who or what influences the women’s diet during pregnancy?**

R: their parents or their husbands

**I: who else?**

R: their grandparents

R: ourselves

**I: who else? Is there anyone else?**

R: the doctors

**I: now how these people influence a woman’s diet? What do they usually say?**

R: they tell us not to eat salty foods

**I: and why did they tell you guys to eat the foods that are good for you?**

R: so that our stomach (our baby) won’t be harms

R: so that the baby can have vitamins

R: be healthy

R: so that they won’t have skin rash, boils

R: so that they won’t be disable

**I: now in some places, women are discouraged from eating certain foods during pregnancy. For example, in some countries, women are told that if they eat eggs during pregnancy, their child will become a thief. Can you describe any practices like that in this community?**

R: they usually tell us not to eat while walking because it can make you want to have your contractions at different place.

**I: okay. That’s one of our beliefs, are there more?**

R: and also people are not allow to walk behind our back when we’re pregnant because

**I: that’s another one. What else?**

R: pregnant women are not allow to walk at night because they might have ghostly spirit within them

**I: okay. What about with our foods?**

R: they usually said not to eat ramen with Kool Aid because it’s not good.

**I: what about with our beliefs?... during your pregnancy, do they tell you not to eat this because it might cause this?**

R: seems like whatever we want to eat we will eat it

R: they don’t tell us that, the only tell us to eat everything.

**I: well I usually hear this from particular women that pregnant women are not allow to eat foods that have crack on them.**

R: oh and why is that?

R: they believe that they’ll have diamond

R: oh! Well that’s another one

**I: yeah there are a lot regarding our foods… well are there more from you guys?... these are all right?... okay now it says, we know that some women receive supplements for low blood during pregnancy, like iron-folic acid (pills for blood). Some mothers told us they consumed all the supplements they were given during pregnancy but others did not. Could you explain any reasons why some mothers do not consume all their supplements?**

R: because they make us vomit

R: they make us feel nausea

R: the vitamin pills

**I: what about the iron-folic acid pills?**

R: seems like it’s good. Seems like the vitamin pills are the only pills that make us want to vomit.

**I: now what helps or motivates some mothers to consume all their supplements?**

R: sweet drinks

**I: are there anything else besides sweet drinks?**

R: even though the supplements make us feel sick but will restrain our desires in order for our baby to have vitamins

**I: oh okay. Now what are the consequences of having low blood during pregnancy and childbirth?**

R: well if we don’t have enough blood during pregnancy and childbirth, that’s one of the risks during pregnancy.

**I: what else?**

R: baby answer that question because you’ve experience it.

R: we’re blind night because of lack of blood.

R: it is really a threat at childbirth because we don’t have enough blood.

**I: now it affects who?**

R: me

**I: what about the baby? Does it also affect the baby? Like do they tell you that it will also?**

R: yes. It also affects the baby

**I: okay. Now are there any advices you guys received from the health workers to prevent or treat low blood in pregnant women?**

R: yes. They used to say eat sashimi and drink V8 (juice) and those things that can help producing blood.

R: orange juice

**I: Now let’s talk about feeding babies after they are born. Once the baby is born and you begin breastfeeding, can you describe a healthy and nutritious diet for women who are breastfeeding?**

R: we must eat fish so we can have breastmilk

**I: what else?**

R: Mackerel. Those canned foods that are made from fish

**I: what about our own foods?**

R: grated coconut

R: also pumpkins

**I: now as for fish from the ocean, it’s only fish that you must eat to have breastmilk?**

R: I would have eaten aikiu (soft food made from iu mix with flour), pumpkin, rice

**I: so you are referring to those kinds of food that are mixed with coconut milk right?**

R: yes. Everything has to be mixed with coconut milk.

**I: Similar to what we discussed earlier about “food taboos” during pregnancy, can you describe any “food taboos” that exist for women who are breastfeeding?**

R: they tell us not to smoke, not to drink, because these thing can harm our baby

R: not to use tobacco

**I: those things are the things that we’re not allow to do them right? But we are talking about our own beliefs**

R: we don’t have to breastfeed our baby while laying down

**I: why is that?**

R: because they’ll choke

R: they’ll die

**I: what else?**

R: they’ll always cry

**I: why they’ll always cry?**

R: because of breastfeeding them while laying down.

R: because when you breastfeed them while laying down, the warmth that they feel makes them become like a magnet.

R: they’ll always want to be stick with you

**I: now are there anything else?**

R: don’t eat raw fish

**I: because what will it do?**

R: because our baby will bite our breast.

**I: yes. Now it says, what advice have you heard from health workers about breastfeeding?**

R: they told us not to bottle feed our baby

**I: why? If you bottle feed your baby, what will happen?**

R: they’ll be malnutrition

**I: okay what else?... what are the importance of breastfeeding? Why is it important for you guys to keep breastfeeding?**

R: so that they won’t get sick

R: yeah that’s it

R: because if they get really sick and hate foods, they’ll still want the breastmilk. Not like bottle feed. If they get really sick, their stomach won’t have anything because they’ll hate food and the bottle milk.

**I: so these are what the doctors and the nurses advise you guys on? What else?**

R: the reason why it’s important to keep breastfeeding is because if the child has diarrhea, the breastmilk will help cure it.

**I: yes. We have heard that some mothers squeeze out the first milk before breastfeeding their baby, while others do not. Can you explain why some mothers squeeze out the first milk?**

R: because it’s salty

R: they said it’s a DREN WAAN (unhealthy milk)

R: but the doctors said the first milk is the best

**I: okay. And what you guys think of the first milk?**

R: it’s the most important milk

**I: now why is it important?**

R: they said it’s good

R: we also heard it from the doctors that it’s good

**I: so, the doctors and the nurses explained the importance of the-**

R: they said we need to give the first milk because it’s good and important

**I: if you were told to squeeze out the first milk, who or what influences you to squeeze out the first milk?**

R:

**I: who else?**

R: as for me, there is none

R: me too

R: the first thing they say to us is, “breastfeed your baby so he/she can start to feel your warmth”. At the time the baby just got delivered.

**I: okay. Now we’ve heard some mothers first introduce foods other than breast milk when their baby is 6 months old, while some introduce foods earlier or later than 6 months. Could you describe the reasons why some mothers introduce foods or liquids at 6 months of age?**

R: because they cry. They cry maybe because they don’t have enough foods

**I: what else?**

R: we follow the food chart

**I: what about those mothers that introduce foods earlier than 6 months? Why do they introduce foods earlier than 6 months?**

R: because they don’t have enough breastmilk

**I: what about those that introduce foods after 6 months? Why do they feed their children after 6 months?**

R: because they’re lazy. They said it’s the fastest way for their baby to eat hard foods

**I: are there more?**

R: seems like no more

**I: there are some mothers said that they don’t have enough breastmilk to breastfeed their baby, can you describe how the baby under 6 months old breastfeed when they don’t have enough breastmilk?**

R: we bottle feed them with coconut drink.

**I: so, you are saying you switch to bottle feed. Okay what else?**

R: make pandanus juice and bottle feed them with.

**I: what about any formula? Or the milk in the can? Do you guys give that to them?**

R: yes, we do. We mix the formula and give to them

**I: so you are saying that when there is no more formula then you will now switch to coconut drink and pandanus juice right?**

R: yes

**I: now are there any information or ways regarding on how to produce more breastmilk?**

R: we should eat fish and grated coconut

**I: good. What else?**

R: let the breasts loose

**I: yes. Now where these information come from? Like eating fish or eating grated coconut or let the breast loose so you can have more breastmilk?**

R: from the doctors

**I: okay who else?**

R: from our parents

**I: okay now could you describe for me how mothers in this community know that it is time to stop breastfeeding their child?**

R: they just don’t want to breastfeed anymore

**I: okay. What else?**

R: their body just need foods

**I: so that they can be satisfied?**

R: yes

**I: okay**

R: it’s time for them to stop

R: we just stop breastfeeding them

**I: oh okay. Now can you explain that how you just stop breastfeeding them?**

R: we just make them stop breastfeeding. We force them to stop breastfeeding

**I: oh, so you’re saying we, ourselves forcing them to stop. Are there anything else?**

R: there are some kids don’t have ends to their breastfeeding. They can breastfeed up to 6 or 12 grade

R: there are some women when a new child is born, then they’ll stop breastfeeding the other child because they have to breastfeed the new baby.

**I: yeah that’s totally true. Now some people have mentioned that they try to feed their young children a balanced diet. Can you explain what people mean by a balanced diet?**

R: those food like pandanus, papaya, fish, pumpkin, banana-

**I: what else? What are the kinds of food with balanced diet?**

R: aikiu (soft food made from iu mix with flour), ai-rice (iu with rice)

**I: For the last question about feeding children we’d like to learn about how decisions are made. Can you explain anything that influences mothers’ decisions about breastfeeding their young children in this community?**

R: as for me, the reason why I choose to breastfeed is because the breastmilk is important for the baby, it’s good because it has vitamin, and it cost free. We don’t have to buy for formula or milk.

**I: what else?**

R: keep breasting so the baby can grow well and healthy

**I: grow well, what else?**

R: won’t get sick frequently

**I: okay. We would like to ask a few questions about children when they are sick. When children under 2 get sick, some parents take their children to the doctor first and others use traditional healing first. Can you describe the reasons for this difference?**

R: some believes in traditional medicines.

**I: what about others?**

R: they go straight to the doctor

**I: okay what else?... if it was you, would you take your child to the doctor first or to the traditional healers?**

R: both of them. Doctor and the traditional healers

**I: you seek both-**

R: we have to take them to the doctors first and if there’s nothing they can do, then we will take them to the traditional healers for swollen stomach or something.

R: or to the pastors so they can pray with them

**I: now what illnesses are commonly treated with traditional medicines?**

R: Kijonkan (infant’s natural illness)

**I: okay, what else?**

R: swollen stomach

**I: now what kind of traditional medicines are used for these illnesses? Kijonkan for example.**

R: as for the kijonkan, they use local leaves like ATAT (local herb) and MARIKO (local herb)

**I: now do they bath them with the medicines or let them drink?**

R: they let them drink

**I: what about the traditional medicines for swollen stomach?**

R: they let them drink banana juice,coconut drink, kinos (local tree)

**I: what about bathing?**

R: well they also bath them

**I: they bath them with what?**

R: they let them soak in water made from the local leaves called KINO

**I: now who influence on whether the children should use traditional medicines?**

R: us. We use our own understanding and experience. Like when they keep having fever, we will take them to the traditional healers to find out if they have swollen stomach

**I: who else? Who usually give advice?**

R: my parents

R: our family members

**I: Can you describe how children are fed when they are sick compared to when they are not sick?**

R: when they are sick, we check with them what they want to eat

R: we will make iu (white cotton inside the coconut) and see if they want it. Like a spoon at a time. And if they don’t like it then we’ll look for other foods they might like.

**I: okay, what else?... what if a child has a diarrhea? How do you guys feed him/her?**

R: we breastfeed them and give them water

R: let them drink tea

**I: now what kinds of food you give? Like when they have diarrhea for example.**

R: we usually give them aikiu (iu mixed with flour), rice-

R: lukwor (soft food made from iu mix with water&milk)

**I: what about the water? What kind of water you give?**

R: coconut drink

**I: okay and I am talking about when they’re having diarrhea**

R: I usually give water frequently

**I: and why do you give water frequently?**

R: so their body won’t get dehydrated

**I: that’s the most importance?**

R: yes

**I: Now we would like to learn about the foods that you provide for your family. Could you talk about what influences which foods people in this community provide for their families? Or like why is it important for you to provide foods for your family?**

R: so that they can be healthy

**I: okay, what else?... one of the answers was so that they can be healthy, what else?... are there more?**

R: seems like no more

**I: now, are there any difficulties in getting the foods you want for your family?**

R: yes there are difficulties

**I: and what are the difficulties?**

R: sometimes it’s not enough- we want to have other foods but we don’t have enough money to have them.

**I: good, what other difficulties you encounter?... she mentioned not enough money-**

R: not enough copra

**I: now why copra?**

R: sometimes we ran out of copra at our homes

**I: now when there are no more copra, what happen?**

R: we can’t make copra because there are no more

**I: so you are saying that copra is like the money for your needs right?**

R: yes, it’s our money

**I: what else?... okay can we all look around to our surrounding? As we are scanning our surrounding, we can see breadfruit, pandanus, and coconuts. Why is it difficult to provide these kinds of food to your family?**

R: we’re just feeling lazy to cook them

**I: okay. Now, how does families deal with food shortages? For example, sharing foods or buying foods from the store?**

R: go fishing

**I: okay… what else?**

R: picking out breadfruits

**I: what about the foods at the store? If the money with you wasn’t enough to buy food from the store, what would you do?**

R: we eat local foods

R: we can’t bring foods from the store because the money is not enough, we’ll wait until we have enough money and then buy foods.

**I: okay. Now, many families have told us that they eat local foods when there are no processed foods in stores or when they do not have enough money. Can you explain why local foods are not eaten more often?**

R: because every fruits have their own season to be harvested

**I: what about other local foods that can be harvested every time?... like papaya or banana-**

R: banana and papaya can’t be harvested every time-

**I: oh**

R: you mean the garden foods right?

R: it also depends if we have enough ripe vegetables in our garden. But sometimes all the vegetables are harvested at the same time and we have to wait again for a long time for harvesting.

**I: what about sea foods? Why don’t you eat fish all the time? Or crab?**

R: we don’t have fisherman

R: if our husband don’t know how to fish then we will not eat fish

R: if the sea current is not good then they won’t go fishing

R: or if we already have some, then we’re just lazy to cook for ourselves. We want to eat but we’re feeling lazy to prepare them

**I: now, can you explain the purpose of green leafy vegetables? Do the green leafs have any purposes? Like for example, banana leafs, breadfruit leafs, papaya leafs, or pumpkin leafs. Do they have purposes and what kind of purposes they have?**

R: we use the lime leafs for our drink

**I: okay, what about eating it?**

R: I don’t think we eat it

**I: is there a leaf on this island that we can eat?**

R: pumpkin leafs. We eat pumpkin leafs. Like the yellow flower in the middle of the leafs. We use them for ingredients.

**I: now, we see that some families raise chickens. Can you explain what these chickens are used for? for example, for eating as meat or collecting the eggs?**

R: for eating

**I: okay, what else?**

R: we also collect eggs and use them for meat and for our pancake’s ingredients too

**I: now, what makes it difficult to feed children eggs regularly? For example, what are the difficulties in collecting eggs? Why is it difficult to feed the child eggs or collecting the eggs?**

R: we usually don’t know where the hens lay their eggs

**I: okay what else?... why don’t you feed your child eggs every day?**

R: because we feed them eggs once

R: because we don’t have chicken and we don’t have money to buy eggs.

**I: now in the next section, we would like to talk about water and hygiene. Can you please describe how people typically get water for their families in this community?**

R: they fill their water tank when its rain

**I: okay what else?**

R: those that don’t have water tank, they fetch water from other people’s water tank

**I: are there more?**

R: when the water tank goes empty, people buy water from the store.

**I: okay what else? Is it difficult for you to fetch water?**

R: sometimes it’s difficult because we depend on the people’s approval on fetching water from their water catchment. If they refuse to give us water then we won’t have water to drink.

**I: what about storing water, are there any difficulties in storing water?**

R: if we have water storage then we’ll store our water. But if we don’t then we won’t.

**I: what you’re saying is when there’s enough water storage right?**

R: yes

**I: okay. We’ve heard that some families boil water for young children and others do not. Can you explain why some people boil water for children and others do not?**

R: some boil water for their children because they don’t want their children to have diarrhea.

R: but some, they’re not use to boiling water.

**I: are there more reasons?... now are the drinking water safe to drink even without boiling?**

R: they’re good because they’re from the sky.

**I: okay what else? Why do some people don’t boil water? Is it okay if they don’t boil water?**

R: they said it’s okay

**I: they said it’s okay?**

R: yes. Because they clean their water tank frequently.

**I: now, what are the difficulties that make it hard for you to boil water?... why is it difficult for you to boil water every day?**

R: because sometimes we’re busy with our household chores and with our children and we don’t have time to boil our water.

**I: okay what else?... now, are there any difficulties in keeping water system clean?**

R: if there will someone to clean it then it will be clean

**I: okay. What else?**

R: my house for example, we don’t have anyone to climb and clean the house roof.

**I: good. Are there more?**

R: seems like no more. That’s all we know

**I: okay. What about house roof? Is it also make it difficult for water system to be clean?**

R: no because we make filter.

**I: now, what would make it easier to clean or treat water catchment system?**

R: have them covered to protect them from animals and insects

**I: good what else? What did you say?**

R: make water filter to help make our water clean

**I: okay what else?... is there anything else that can help make it easier for our water system to be cleaned?**

R: clean inside of our water tank

**I: We’ve heard some families wash hands regularly while others do not. Can you explain some reasons for this difference?**

R: some are lazy, and some are not

**I: okay what else?**

R: some are impatient when they’re hungry which make them forget to wash their hands

**I: okay. And is that a habit?**

R: yes

**I: okay. Why some people wash their hands with soap and others do not?**

R: to make our hands clean

R: to kill germs in our hands

**I: okay. And why others do not wash their hands with water and soap?**

R: some say it’s a waste of time

R: some just get too tired and forget to wash their hands but go straight to sleep without washing their hands

**I: what are the main things that prevent washing hands with soap regularly**

R: as for me, sometimes when I am too tired, I feel lazy to go back and bring the soap if I already pass it.

**I: okay what else?**

R: when there is no soap in our household

**I: now we’ll go back to what I ask before. Is this a habit for us marshallese?**

R: yes, it can also a habit

R: for some it’s a habit

**I: what about others?... now for example, since when you were a child until now you’re an adult, is this a practice or is this a lesson they taught you about to wash your hands?**

R: yes

**I: okay and why would you say that?**

R: because we don’t want to listen to what they say

**I: now, why some people use hand sanitizer instead of soap?**

R: to kill the germs

**I: okay…**

R: it cleans twice as much as soap

**I: what else?**

R: because we can use it everywhere and use in those place that don’t have water to wash our hands

**I: okay. Now for the last questions, we would like to learn about how parents care for their children. We’ve heard that husbands are an important support for their wives during pregnancy. Can you explain what husbands do to support their wives while they are pregnant?**

R: they help us by supporting us

R: they help us with our chores and do whatever we tell them to do.

**I: what about the mothers? What do they do to support their daughter while they’re pregnant?**

R: they also help us taking care of our kids

**I: what else?**

R: they give us those foods that we’re craving for

**I: We are interested in learning about how caregivers play with children under 2 years. Can you describe for me in detail how you play with children?**

R: play with them by tickling them.

**I: okay…**

R: teach them about our culture

R: teach them how to spell their name

R: teach them how to use the restroom

**I: now we’ve heard that some parents spend time outside of the home and it may affect the way they raise their young children. Can you tell me about your experiences with this?**

R: they want to go to other people’s house and tell stories

**I: what else?**

R: they want to have fun

**I: now how are these activities affect their children under 2?**

R: they might go to the lagoon side and drown

R: they might fall into the water well

R: they might eat unclean stuff

**I: now how are these affect the hygiene of their children under 2?**

R: they get diarrhea

**I: diarrhea what else?... when the parents are not there to wash our clean them, what happen to the children?**

R: they get sick from starvation

**I: good what else?... when they’re not clean, what happen to them?**

R: they get skin rashes

**I: now what kinds of activities women are doing outside of the home?**

R: some goes out to play sports

R: work

**I: yeah some goes to work, okay what else?**

R: some goes out because they don’t want their family members to find out they’re smoking or eating tobacco.

**I: what about the men? What kinds of activities men are doing outside of the home?**

R: they go out to chat with other men

**I: okay. We have heard from some people that they prefer to get health messages from the radio, others say from the clinic. Can you describe for me the best ways to reach people with information on health in this community?**

R: seek them

R: call them

**I: seek them, call them, what else?... now, is there any community group that could be a good place to deliver health messages, for example men’s group, father’s group or church group?**

R: kumit (woman collation)

**I: okay. What about any churches?**

R: there are also groups in the churches

**I: there are? Okay. Now is it okay if these groups would have delivered these messages or information?... would it be easy for you to have these messages?**

R: yes

**I: okay that was great, we are done now. Thank you once again for your generous time and for sharing your thoughts with us. We greatly appreciate your help and we hope this research will help us improve the health of mothers and children in your community.**
