# Supplementary material for: Conceptualizing multi-level determinants of infant and young child nutrition in the Republic of Marshall Islands–a socio-ecological perspective
Source: PLOS Glob Public Health. 2022 Dec 19;2(12):e0001343. doi: 10.1371/journal.pgph.0001343 (PMC10022247; doi:10.1371/journal.pgph.0001343)
Supplement: S1 Data — (ZIP) [file pgph.0001343.s001.zip › RMI Supp Data/Focus groups data/F10R_FGD_Female_Arno_Sep 27_Libon.docx]

- **Interview code: F10R**
- **Interview type and interviewee: FGD MFG**
- **Interview date: SEPT 27,2018**
- **Location: ARNO**
- **Interviewer: LIBON**
- **Transcriber: Shante**

**I: Ok. before we start, do you guys agree on taking your part with this survey?**

R: (everyone) Yes.

**I: Ok. Now, before I start, we will introduce ourselves and I will start with me, My name is Libon Joseph Jorkan, I have 3 children, the oldest is 14 years old the 2^nd^ one is 6 and the youngest one he will turn 3 tomorrow.**

R: my turn now? My name is Jatak. My husband name is Atadrik but he is gone, he’s dead. I have 6 children. The youngest one is working in the office of Education. His name is kentur Kauwe. There is, I am 60 some years old. The boy that I look after, he’s the son of, one of my granddaughters, his father name is, the one that I have a grandchild with, his father name is casper. The girl has 3 children. And the second one to the youngest, that’s it.

**I: how old is he?**

R: 4

**I: good.**

R: my name is winta, I have 3 children. The oldest one is in high school and the second one is in kinder and the youngest is this little boy.

**I: how old is he?**

R: 2

**I: 2?**

R: my name is bearl Jonathan, my husband name is jikalum from wujae (marshall island atolls.) we have 2 boys. They both don’t go to school, one is 3 years old. And the other is 4.

R: well, my name is Lizzy boktok. I have 2 children. The oldest one is 4 years old. And the youngest one is 2 years old.

R: Hi. My name is goldy peter. I have one son and he is 4 years old.

R: my name is Metlin, I was a Jonathan, but I am married now so, I am a buntak. We have one child and he’s 3 years old.

**I: Ok. good. To start, we will now talk about the health of the women. And the first question says, can you tell me how women that look healthy?**

R: she’s neat

R: sexy. (everyone giggling.)

**I: what else?**

R: a women that looks healthy/good? Or what?

**I: body size. One women that looks good**.

R: she’s skinny and she looks healthy and she roam around.

R: move around.

**I: now, what kind of food is good and has lots of nutrition that it’s good for the pregnant women?**

R: local foods like pandanus and coconut juice, breadfruit

R: fish

R: Papaya

**I: so, if it was for foods from the ocean, they can…**

R: if they crave for them they eat.

R: crap and lobster clams and oysters.

**I: Ok. now are these foods different from any kinds of foods that women eat? On how I say any kinds of food I mean from the women’s that are not pregnant?**

R: yes

**I: what is the difference?**

R: they’re foods are difference.

**I: they’re foods are difference? Ok. so, you mean when they are pregnant, they’re picky with what they eat… what about those that are not pregnant?**

R: they eat whatever. Everything is good.

**I: who gave advice to these ladies with the foods they eat?**

R: their husband, their doctors

**I: who else?**

R: their parents

**I: now how did those affect the ladies?**

R: how?

**I: like what word of advice to they tell them?**

R: they say, you eat this and don’t think of yourself because there’s a child now. (everyone giggling)

**I: that’s good. What else?**

R: now what will I say now that you already took my answer? Laughing

R: because that’s what they tell us.

**I: yes. What about why do you eat the foods?**

R: so that the child can be healthy.

R: for you to be healthy.

**I: have nutrition and child feels healthy.**

R: feels healthy inside your stomach.

**I: Ok on some places, there are some kinds of foods that mothers aren’t allowed to eat, for example in some places they say the women shouldn’t be eating eggs because when the child will be born they will be a stealer**.

R: Oh… giggling

**I: what about us in our islands, do we have something to believe during our pregnancy?**

R: we don’t here anything about it.

**I: like for anything**

R: about food?

**I: yeah about food.**

R: eat egg?

**I: No. anything. That’s just what the people in other places believe in. so, now what about us in our islands?**

R: we should not eat the birds flying high because we will be posses.

**I: there’s one, what else?**

R: there’s one, you should not be eating kwole (it’s the round meat inside the breadfruit) because you will fart nonstop.

R: everyone laughing.

R: don’t eat the pandanus that are like cracked.

**I: why?**

R: because the child will have cleft baled.

**I: yes. that’s one.**

R: I just heard about that.

**I: Now, I know some pregnant women take supplements and nutritious when they have less blood. It says, some women finish all their supplements during their pregnancy and some don’t. Now, can you explain the reason why some pregnancy women don’t drink all their supplements and their nutrition?**

R: they don’t like the taste.

**I: Ok. what else?**

R: some women forgets.

R: when they are dizzy/nausea then they won’t drink them.

**I: when they are dizzy they don’t drink them? Did you not like your supplements?**

R: yes.

**I: why?**

R: when I drink them, I vomit…

**I: you vomit? Now, what helps and make the pregnancy women do to drink their supplements?**

R: when we drink them and eat candy after.

R: we usually close our nose and drink them.

**I: Ok. what else?**

R: our thoughts are the one that makes us strong to drink them so that we can be healthy. The child and us.

**I: it’s good for the child and for you?**

R: hm…

**I: Now what are the illnesses when it comes to low blood during the time you are pregnant until the time you are about to give birth?**

R: sometimes we will be weak to give birth.

**I: so, if they are weak?**

R: she won’t have a healthy birth because she doesn’t have enough blood.

R: it can affect the child.

**I: Ok. so, when they are weak, what will happen?**

R: they won’t be apple to give birth.

**I: so, its dangerous for both of them?**

R: yes.

**I: is there any words of advice you guys took from the nurse about preventing illnesses and about low blood with the pregnant women? If you guys had less blood, what do they tell you?**

R: tell us to eat meat, like eat raw

R: eat sashimi

R: sashimi

**I: OK. what about drinks?**

R: drink lots of water.

**I: drink lots of water… we will now talk about how you feed the child after they are born. It says, after the child is born and you start breastfeeding him, can you tell me what kind of food are good and has nutrition with the women that are breastfeeding?**

R: fish

R: number one, squeeze out their breastmilk so that it can be good and bigger.

**I: can you explain how you say squeeze out the breastmilk?**

R: because it can get more milk in the breastmilk.

**I: oh, on how they eat a lot of fish?**

R: yeah, when they eat.

**I: Oh. Ok.**

R: they also eat coconut meat.

R: yeah. coconut meat too.

**I: Ok.**

R: Corn beef.

**I: I heard some women cook rice and when the rice is boiling they take**

R: the thing on top

**I: the water on top… do you guys usually do that?**

R: to feed the child or what? For the women that will eat?

**I: yeah. for them to eat so that the child can eat.**

R: we only know about when the child is going to eat, we cook the rice…

R: and breastfeed

**I: breastfeed the child with it? Oh…**

R: No. when we cook the rice, because the child already know how to eat, so we take the water from on top, not just the water but, it’s like a soft food. eat soft food with the water and let them eat.

**I: Oh. ok. is there any local food that is good for making your breastmilk other then coconut meat and fish? what other foods do they tell you guys to eat more?**

R: I think that’s all of them

**I: that’s all?**

R: meat.

**I: meat and?**

R: fish

**I: OK. And foods that has a lot of coconut milk in them?**

R: hmm…

**I: Ok. Now, like for the things we’ve talked about before about believe, in pregnancy, it says, can you tell me about whatever believes about women when they are breastfeeding? Is there anything we believe about breastfeeding?**

R: like we are not supposed to eat lots of salt because our breastmilk will be salty. Right?

**I: Ok. we can say… let’s say forbidden.**

R: Oh

**I: yes. they usually tell us not to do that. But our believes there is no, like how we said, we don’t eat pandanus that are cracked because the child will have cleft blade. What about during pregnancy?**

R: Oh. we don’t lay down and breastfeed them because they will

R: cry. And wants to be attached with you all the time.

**I: oh. ok.**

R: and chores won’t be done.

**I: Ok. what else?**

R: we should not be attached to our guys?

**I: Not what?**

R: not be attached to our guy

**I: Oh. ok.**

R: because we are breastfeeding.

R: giggling

R: oh…

**I: Ok. what else? Anything else? What about food? what kind of food they tell us not to eat?**

R: there’s one. Salt

R: raw fish.

**I: why?**

R: because thee child will bite your nipples.

**I: Now, what word of advice or lesson you learned from the workers at the ministry of health about breastfeeding?**

R: oh. on how we make our breast. On how we clean them.

**I: Oh. ok. Now, how did they tell you to clean them?**

R: you do this.

R: everyone laughing.

**I: you wash it with what?**

R: hot water**.**

**I: hot water and?**

R: soap.

**I: soap it? Ok. Now, what else?**

R: we should breastfeed them until…

R: we should not feed them more food other then breastmilk. That’s the thing we should give them. Our breastmilk**.**

**I: oh. ok.**

R: they should not be using bottles.

**I: Now, why do they want you guys to breastfeed only?**

R: because they say, a child that is breastfeeding from the time he was born, he will know a lot of things quickly and will be smart.

**I: hmm. are there any words of advice from the families about how you guys breastfeed?**

R: yeah, they usually tell us not to lay down and breastfeed.

**I: is there any other?**

R: we should not be out all night because we breastfeed.

**I: hm… why do they say we should not be out all night?**

R: we will be posses.

**I: bring the spirit to the child?**

R: yes.

**I: ok. we heard that some mothers squeeze out the first liquids in the breast before they breastfeed, and some don’t. can you explain why some mothers squeeze the first liquid in their breast?**

R: they say it’s salty.

R: no use of it.

R: it’s not ine (like not enough nutrition in it.)

**I: what about from you guys? What was your thoughts about the first liquids?**

R: it’s important

R: it has vitamin

R: when we breastfeed for long and then it will turn into like a yellow thing, well how do they call that, they say it’s the vitamin.

**I: who told you to squeeze out the first liquids from your breastmilk? Like they**

R: grandmother and grandfather.

R: hahah I think I don’t know.

R: I don’t think anyone has ever told me…

R: maybe nowadays… oh, the elders told us to squeeze them out. I don’t know why but they don’t explain it to us. But they only say squeeze it out before we breastfeed.

**I: Oh. ok. but now a days they barely do?**

R: they don’t.

R: hmm…

**I: we heard some mothers start feeding their child food other then breastmilk during the time the child was 6 months but some start feeding their child before the child was 6 months and some after they reach 6 months. Now, why did you feed your child when he was 6 months?**

R: because it’s time for him to eat.

R: the doctors only said they can eat when they reach 6 months.

R: their bodies are ready to receive foods.

**I: ok**

R: some feed them from when they are 3 months.

**I: why did they start feeding them when they were 3 months?**

R: they say because their body can be strong.

**I: Oh. ok. what about after they are 6 months? Why do they wit until they are 7 and 8 months or 1 year old and start feeding them?**

R: because they breastfeed, when they give the spoon the child will open his mouth.

**I: hm… ok. now, there are mothers that doesn’t have enough breastmilk to breastfeed the child. Now, can you explain how the child below 6 months breastfeed when the breastmilk isn’t enough?**

R: they give them bottles.

**I: what do you give them inside the bottles?**

R: for the outer islands, we give them coconut juice.

**I: what else?**

R: just coconut juice.

R: and jakro (liquid from the coconut tree.)

R: jakro and milk. They also give those to them.

**I: what kind milk?**

R: those milk for kids.

**I: oh. the formula ones?**

R: yes.

R: milk powder

R: they also drink tea in a bottle.

**I: tea. Ok**

R: koolaid.

**I: and what?**

R: kool aid

**I: koolaid?**

R: hey girl, we are talking about little babies.

R: yeah, 3 months.

R: whoa. Group laughing.

R: not to my child, I saw someone giving koodaid to her child.

**I: hmm… now, is there any understanding and ways to make our breastmilk bigger?**

R: that’s the reason, eat until you have big amount of breastmilk.

**I: what kind of foods?**

R: the foods that I already said. Meat and fish, meat from the ocean.

**I: Ok. other then meat, what else you guys eat so that you can have lots of breastmilk?**

R: foods from the stores.

**I: like what?**

R: can foods.

R: rice and bread, tuna and mackerel.

**I: Ok. where does that information comes from?**

R: from ourselves.

R: on how we take them, we see that it makes more milk to our breast.

**I: ok. who else?**

R: the people next to us.

R: people in our house.

**I: what about the doctors?**

R: the doctors also say so.

**I: Ok. now, can you explain how the mothers in this community know that it’s time for the child to stop with breastfeeding?**

R: no more breastmilk.

R: and the doctors usually say, breastfeed the child until they turn 2 years old.

R: I still have breastmilk in my breast. I breast feed until they are in 6 and 8 grade and until they graduate…

R: everyone laughing.

R: well, he is healthy.

R: some women breastmilk runs out and some don’t.

R: hmm…

**I: what about you? how do you know that it’s time for your child to stop?**

R: because he is grown and also because he has a younger sibling.

**I: Oh. ok**

R: some kids breastfeed with each other. Like the other child in the other breast and the other one on the other breast.

**I: 0h. the child that was just born… now, some people to feed children foods that has nutrition. Can you tell me what they meant my has nutrition?**

R: foods that has vitamin in them.

**I: like?**

R: coconut meat, pandanus, coconut juice

R: papaya.

**I: ok. what else? Anything else?**

R: imported foods that has nutrition?

**I: like what?**

R: orange and

R: local chickens

**I: local chickens… now, for our last questions, we want to know how they choose they decisions? Can you explain what made mothers make breasting important in this community?**

R: how?

**I: why is it important for mothers to breastfeed? What words of advice did they gave you for you guys to breastfeed?**

R: both talking, so that they can be healthy.

R: so that they won’t have rash and…

R: so that they can be healthy and have a strong body.

**I: Ok. Now I want to ask some questions about children that are sick. It says, if the children under the age of 2 are sick the mother and father brings them to the doctors first and some bring them to the tradition healers. It’s says, what are the difference between the two information?**

R: doctor and traditional healer?

**I: yes.**

R: when we bring them to the doctors they give us medicines and it take days.

R: and tradition healers they are faster.

R: the other one is, there is a fee for going to the doctors, we have to pay for the kids to see the doctors. For the traditional healers, they are free.

**I: Ok.**

R: why do we choose what we give them?

**I: hm…**

R: some take tradition healers because it cost to much for them to go to the doctors.

**I: yes.**

R: what kind of illnesses they use tradition medicines with?

R: diarrhea

R: stomach pump

R: fever and coughing.

R: skin rash.

**I: is there any from the back? What kind of medicines do they use with local medicines?**

R: kids medicines.

R: we also use local oil.

**I: for?**

R: massage their stomachs when they have pumps.

**I: oh. ok.**

R: and also when they have fever.

**I: they also use oil?**

R: yes.

**I: now what kind of medicines do they use for these illnesses?**

R: heh?

**I: If it was for fever and runny nose and… what kind of medicines would you use?**

R: the noni leaves. We put in on the child’s body, it takes away the fever.

**I: Oh.**

R: and when they have nose issues they put oil.

**I: if it was skin rash?**

**(can’t hear their answers because of the lady in the background is talking on the phone with Majuro people.)**

R: we usually hear but we don’t know.

**I: ok.**

R: that there is a local medicines for skin rash.

**I: oh. But you guys don’t know what kind of leaves they use?**

R: hm.

**I: ok. who gives you advice when you need local medicines?**

R: our grandmothers and mothers.

**I: now, can you tell me how you feed your child when he is sick and when he is not sick.**

R: when the child is sick, we bring them closer and pretend that the foods is really good, but he won’t eat. but when he is not sick then you will chase after him and feed him because he doesn’t want to.

R: chase him and spank him so that he can sit down and eats.

**I: Hm… what if he has a diarrhea?**

R: we feed him soft foods.

R: when he has diarrhea?

R: we give him water.

R: give him medicines

R: give him medicines and especially waters.

**I: ok. but do you guys feed them a lot or…**

R: we don’t feed them too much because they often poop. We feed them little by little.

**I: why is it important for you guys to let the child drink water?**

R: because their body is dry.

**I: now, what kind of foods you guys usually give them?**

R: like soft foods, we make soft foods.

**I: OK.**

R: cook soft foods.

R: when they have diarrhea?

**I: hm…**

R: the foods they want to eat.

**I: now how often do you guys give it to him?**

R: when they have diarrhea, we don’t feed them as much.

**I: don’t feed him as much?**

R: feed them a little bit, at least so that they can have a full stomach. Just little bit because he won’t stop pooping If you do give him a lot.

**I: oh. ok. now, what kind of drinks do you guys give to them?**

R: when?

**I: when they are sick or diarrhea.**

R: they usually drink water and sometimes they ask for sweet drinks.

**I: Oh. ok**

R: tea.

**I: tea?**

R: hmm.

**I: tea works for what?**

R: make the stomach hard.

**I: ok. now we want to learn about the food that are available for your family. can you explain what influence your family in this community needs to be available for your family?**

R: rice

**I: rice.**

R: flour

R: baking powder

R: those are what we need to eat.

R: things from the stores.

R: if there is no breadfruit and pandanus, we won’t eat because we don’t have those.

**I: so, you mean you guys depend more on the foods in the stores because sometimes there is no local foods?**

R: yes.

**I: what about papaya and bananas?**

R: there are papaya and bananas

**I: now, what about fish?**

R: fish also.

**I: but you guys…**

R: meat

**I: you usually get meats from the stores?**

**R: yes.**

**I: ok. are there any issues on taking the foods you want for your family?**

R: yes

R: yes, because we need to look for coconuts,

R: if there are no coconuts then we will have no foods.

R: some go fishing

R: some look for U. (coconut meat)

R: there are difficulties if these are taking so long, but when it’s quick then it’s good.

**I: what about for those kinds of foods that has time to season?**

R: like I said, that the only issue when it’s not time for breadfruits and not time for pandanus. There is U. we can cook U.

**I: ok. how do families find a way to feed their family, for example, on how they share their foods or buy foods from the store.**

R: one more time please.

**I: how do a family finds a way, when there is not enough foods? for example, on how they share foods to other people in the house or on how they go buy foods from the store.**

R: when it’s not enough?

**I: hmm**

R: cause we, the coconut, brings us foods. if we know that one bag is not enough, then we will make another one. So that it can be enough**.**

**I: what if that was the only money you had?**

R: we will give meat to the child first but not the grown ups.

**I: the kids first you make food for them. What about if that was the only money you had left and you were in a store and you were going to eat? is it an issue for you to buy or what do you buy so that it can be enough?**

R: things that can be enough. Like, what can we say…

R: hotdogs and

R: things that aren’t expensive.

**I: OK. now, there are families says that they eat only local foods if they don’t have imported foods or when it’s not enough. Can you explain why you don’t eat local food all the time?**

R: sometimes there is enough and sometime it’s not enough. When there is fish we eat it with coconut.

R: we sometimes have local foods to eat but we just want to eat rice and things like that.

**I: yeah. because we are used to them. It’s like us these days we eat only from the stores and eat imported foods.**

R: if there is breadfruit we make breadfruit paste, if there is pandanus we make pandanus paste**.**

**I: Ok. so, those are the foods pandanus and breadfruit that has season.?**

R: and bananas we also…

**I: banana also? There is a time for it in the season? Now, can you tell me is there any use of the leaf in the plants?**

R: we use them for medicines.

**I: what about for food?**

R: there are leaf’s that we eat.

**I: like what?**

R: like for the breadfruit leaf’s, we cook our foods with it.

**I: Oh. breadfruit leaf’s?**

R: hm.

R: and the leaf’s of lol. (it’s a tree that I don’t know what it calls in English. Ehhe)

**I: do you eat them?**

R: we don’t.

R: it’s like a tin foil.

**I: Oh. For tasting only?**

R: when we cook them it’s like there is a nutrition in it.

R: we fill in the leaf’s and wrap them and cook them.

R: yeah. cook them.

**I: Oh. yeah, it’s like a tin foil. What about leaf’s that you can take it and eat it and mix it with the foods?**

R: there’s jiak. (the leaf that they cook it with the food)

**I: how do you guys use it?**

R: it like we take it and eat it, it’s like… just food

**I: oh. the leaf on the U?**

R: yeah. it’s like a sweet for kids.

**I: so. That’s the only thing?**

R: I think there is more…

**I: what about pumpkins and bananas?**

R: we also eat the leaf of the pumpkins. We boil it and mix it with soft foods.

R: hmm

**I: Oh. and mix it with it.**

R: there is also one near the that house

**I: what’s it calls?**

R: well, we don’t know the name of it but we also use it for cooking and eating.

**I: you mix it with everything? Any kind cook you mix it with?**

R: make it meat and soap

R: and carrots and cabbage

R: we also use potato leaf. Mix it with the food we eat.

**I: Ok. Now, we see that some families have chickens for pets. Can you tell me what they use for the chickens?**

R: pet local chickens?

**I: for example. Do they eat them all the time or what?**

R: sometimes.

R: they sell them.

R: if we want to eat, we kill them and eat them.

**I: Ok. what about their eggs?**

R: also the eggs. We cook them and eat them.

**I: Ok. what are the issues for you to feed your child eggs all the time?**

R: we say their teeth’s will fall out.

**I: when they eat too much eggs?**

R: the white part.

**I: oh.**

R: the yellow one?

R: the white one.

R: some say if we eat too much eggs we will have high blood.

**I: oh. if we eat too much of the yellow part?**

R: yes.

**I: we will be high blood?**

R: that’s how we heard it.

**I: now who did you hear it from?**

R: the doctors. They say we should eat 2 eggs at the same time.

**I: Oh ok. just to get some strength. Right?**

R: yes,

**I: Ok. on this section, we will now talk about water and hygiene. Can you explain how people look for water for their families in this community?**

R: look for water, they fill up water from their house.

**I: Ok. from what?**

R: we fill up our water catchments.

**I: OK**

R: plus our water wells.

**I: good.**

R: water wells, they dig so we can

**I: take from it**

R: hm.

**I: now, is there any issue on taking or looking for waters?**

R: sometimes.

R: there should be tools to do so.

**I: what else?**

R: is there any issues on what?

**I: on how they look and for water? What are the issues you guys see in looking for waters?**

R: the issue is sometimes there are no water catchments.

**I: are there any issue on how they store they’re waters?**

R: if there is water, store? When they are in the water catchments?

**I: hm…**

R: oh. it’s good.

**I: are there enough water catchments for you to store waters?**

R: catchments are not enough.

R: the truth is, it’s not enough**.**

**I: we heard that some families boil their waters for their children and some don’t. can you explain why some people boil water and some don’t?**

R: some keep safe on how they drink. And some are lazy to boil

**I: ok. what else?**

R: the reason why they boil it so that they won’t get diarrhea.

**I: yeah. so that the kids won’t get diarrhea?**

R: yeah. to keep safe.

**I: so, are the waters good for drinking even though they are not boiled?**

R: not good.

R: no good.

R: it’s not good for them but when the stomach is used to it then it’ll be alright.

R: yeah. if a child is not used to boil water then they will have diarrhea, same for those that are not used to not boiling water.

**I: yes. so, what are the issues you see when it comes to boiling water everyday?**

R: not enough fire woods.

R: not enough water.

R: no tea pots.

R: we don’t have enough utensil.

**I: some has and some not enough.**

R: yeah.

**I: what are the issues of you not boiling water everyday?**

R: those are it. Those are probably the issues.

**I: yeah. I repeated it two times. Sorry. Are there any issues on how your water catchments are clean?**

R: yes, because they need to clean them before they fill it up with water.

R: wait for the rain so that the water tanks can be filled up.

**I: what else? What are the other issues that you guys see for the water tanks to be cleaned?**

R: our roof tops aren’t clean.

**I: Now what makes it easy to protect your water catchments?**

R: if they have covers.

**I: ok.**

R: now put Clorox inside the waters.

**I: what about tools?**

R: not enough tools.

**I: we heard from some families that they wash their hands all the time and some don’t. can you explain to me why some do and some don’t wash their hands all the time?**

R: some are used do it and some aren’t.

**I: yes.**

R: you know some Marshallese,

R: hm…

R: sometimes we’re in a hurry because we are hungry,

R: sometimes when we work and we are hungry, we forget to wash our hands and just go straight to eating.

**I: now, why do some people wash their hands with soap and water and some don’t?**

R: they want to make sure their hands are clean.

**I: what about people that don’t use soap?**

R: they want their hands to be clean but they don’t have…

**I: hm.. they don’t have soap to soap their hands.**

R: it’s better if we wash it other then not washing our hands.

R: because it’s dirty.

**I: now, what are the reason why you don’t wash your hands with soap and water?**

R: when we are in a hurry.

R: when we are in a hurry to eat.

**I: Ok. what else?**

R: when we are somewhere else and there’s nothing to wash your hands with except your drinking water.

R: we can not just wash out hands.

**I: OK. why do some people use hand sanitizer other then soap**?

R: they say it kills germs.

**I: Ok. what else?**

R: they say its cleaner.

R: we can carry it where ever.

R: it’s easy for us to use it.

**I: it’s easy for you to use it?**

R: yes. even if we go to far places we can carry it and use it.

**I: now for our last questions, we want to know how mothers and fathers raise their kids, and it can also be grandparents. We heard that some fathers have lots of responsibilities to their wives during their pregnancy, can you explain what the fathers usually do during the time the mother is pregnant?**

R: stay close to their wives because the wives always wants to cuddle.

R: provide their needs.

R: provides their needs on foods and…

R: some fathers also wants to cuddle.

**I: ok. what about on how they do work? Do they do your chores during the time you were pregnant?**

R: yes.

**I: what do the mothers do to their daughters when they are pregnant?**

R: they cook them food if they don’t want to stand and eat.

R: sometimes they tell us to walk around to exercise.

R: tell them to move around so that they baby can also move around.

R: they also drink local medicines when they are pregnant.

**I: medicines for what?**

R: local medicines.

R: some sit in a tub of local medicines so that when the time they give birth the child will just come out.

R: like get them ready before they give birth.

**I: ok. we are interested on how the babysitters play with the child under the age of two. Can you explain to me how the babysitters play with the children?**

R: the babysitter?

**I: mother and father and grandparents and the older siblings, whoever that watch over the child,**

R: well?

**I: how do you guys play with them?**

R: tickle them and talk to them and tell them stories.

R: teach them about our culture. Right? Like 2 years will understand?

**I: hm… now, we also hear that some mothers and fathers stay out all night other then watching over their children, on how I say stay out at night, other then them watching over their kids, they are out there. Now, can you tell me is there any information about why they are…**

**R: why they are outside? Means they do what they want to do and not watch over their children.**

**I: ok. what else?**

R: sometimes there are girls what watch over their boyfriends.

R: and some guys watch over their girlfriends where they go and…

**I: Ok.**

R: they follow each other and don’t want to be out of each other side.

R: some wouldn’t want to just be with the kids and the other is out**.**

**I: Ok. now, how do these effects the child? How does this effect the child when he is watching over himself?**

R: he can be posses.

R: sometimes it’s not good because it can make problems for the family.

R: the child watches over himself.

**I: now how do these effect the child?**

R: if the mother and father don’t take good care of him.

**I: ok. what else? On how he watches over himself? When you guys look at him? is he clean?**

R: no.

R: he’s not clean because whatever he wants to do he does it.

R: if he wants to touch the garbage he can, because he’s young and he can know…

**I: if it’s dirty it can what?**

R: make him sick.

**I: what do women usually do outside their house?**

R: they take the grass out and cut the grasses and gossip.

(everyone laughing)

**I: what else?**

R: tell lies, make stories.

**I: oh. ok. what about the men’s?**

R: they go look for cigarette.

R: they do some trading.

R: they look for food.

**I: now we hear from some people that people get their information about health on the radio and from the Ministry of health. Can you tell me from you own, what can we do to reach the people with the information about health?**

R: what is good?

**I: like for some they say it’s good if they take the information from the radio and from the ministry of health…**

**R: we need to be together and take information from each other.**

**I: ok.**

R: about the better of it.

**I: hm. What else? Is there any other?**

R: we need to listen and learn the information that we know of and that we know that will be good.

**I: is there a community center that we can show the information at about health? For example, groups for man and group for women or the church.**

R: the church.

**I: the church?**

R: yeah, the church.

**I: do women have groups?**

R: they have in every church. If church elsewhere…

**I: in the community?**

R: I don’t think there is.

R: what about the KUMIT group?

R: it’s not a community. it’s a group. It’s for this island.

**I: ok. is there any group in this community they could pass on the information about health?**

R: the youth.

**I: Ok.**

R: the doctor of the island.

**I: hm… anything else? Women or men groups? Nothing?**

R: I said the groups in the church…

**I: oh. in the church. Ok. that’s good. we are done with all our questions. Thanks so much for giving some information that will help us mothers in the Marshall Islands. And all your answers were good. some answers that we look around and see that it’s easy and hard for us in our community. and thank you guys for taking part in this and leaving your home to come help with this and to make a better for our home and the program for our mother and children. Thank you.**
