# Supplementary material for: Conceptualizing multi-level determinants of infant and young child nutrition in the Republic of Marshall Islands–a socio-ecological perspective
Source: PLOS Glob Public Health. 2022 Dec 19;2(12):e0001343. doi: 10.1371/journal.pgph.0001343 (PMC10022247; doi:10.1371/journal.pgph.0001343)
Supplement: S1 Data — (ZIP) [file pgph.0001343.s001.zip › RMI Supp Data/Focus groups data/F11R_FGD_male_Arno_Sep 27_Balton Fela.docx]

- Interview code: F11R
- Interview type and interviewee: FGD_Male
- Interview date: Sept.27.18
- Location: Arno
- Interviewer: Balton
- Transcriber: Marcellina

**I: okay, is it okay if we start now? So, it can be done. First, I want to take this time and thank all of you on behalf of the Minister of health who couldn’t make it to here. I want to take this time and thank you all for wanting to participate in this survey. My name is Balton, I along with my team mates in this survey, we are representing UNICEF. UNICEF is one of the branches in united nation who are trying to help our children. Last year they also did a survey, I think in this island and other outer islands. They came to observe our children’s health and they found out that our children are not growing well. They found out that our children are short not like those other children in the states that look taller than our children and they want to find the reasons why, so they can help. They found out that there is one reason that make children stunted is having diarrhea. The first thousands days of living, if the child is having diarrhea, then they stated to have stunted. By having diarrhea, their body can’t absorb any nutrient to be able to grow. So, the government ask the united nation and they sent the ladies to work with us and we came here with them to help them. There are twelves of us who came and help in this program. Last week, we were in Ine, and now this week, we are here working with Arno Arno people. From what we are doing right now, we are doing a record and we will translate our talking to English so that these foreign ladies can be able to understand what we are talking about and insert it in their research data. The benefit from this is there is a money granted from the World Bank and I think it’s worth $28 million dollar and I think that’s more than enough to help support our children. Not only for the child but also to this community that these children live on. They choose to make their study in Majuro and in the Outer islands, so you guys are very lucky because they chose Arno atoll to represent all the outer islands here in the Marshall Islands. So, thank you all for coming and the questions are not much. now the first question says, could you talk about what influences which foods people in this community provide for their families?... or like what make you choose the food you provide for your family?... for example, if you make dinner for the family or lunch. What do you usually pick to cook for the family? For some people, they would choose to make this kind of food because they are depending on their budgeting, like they can’t afford for a cereal for the children. These are examples for processed that we depend on how much money we have with us. When it’s come to here in Arno, it can’t be on budget but because we don’t have them here on Island right? What influence us to choose on our meal? Okay you, what did you have for breakfast?**

R: pancakes

**I: okay why pancake?**

R: that’s what we can afford

R: or you can say that’s what we have

R: or it’s simple to do it and it’s faster to make it. For breakfast.

**I: okay are there any foods you want to eat but there is none in here? Like steak, are there any steak in here?**

R: no

**I: so, the reason we can’t provide other foods is because-**

R: there is none

**I: or it’s expensive**

R: yes

**I: now what make us choose our daily foods?**

R: it comes from what we desire. Like if we want to eat this and then we want to eat that, but these is none, then we make our right like “okay let’s eat- “if there are no bread at our homes, then we’ll say, “okay then we’ll eat rice, cook rice for breakfast”.

**I: it depends on our desire**

R: yes

R: or you can say it depends on what we want to eat and on budget.

R: because there are none at our homes. That’s the reason. Some homes have flour, so they can bake bread because they have grease, they have sugar, but others don’t have.

**I: so, it depends on what we have in our homes and on what we want right?**

R: yes

**I: and it also depends on budget to buy the stuff we don’t have at our homes**

R: yes. if we don’t have money then we won’t have them

**I: okay from the data we collected few weeks ago, families told us that they usually eat processed foods, and eat local foods when there are no more processed foods. and our consultants asked us, “why don’t they eat local foods first and then processed foods after when there are no more local foods?” like why do you choose processed foods first instead of local foods like fish, and local chicken? Aside for breakfast like papaya and fish we eat bread or pancakes.**

R: because we depend on the local foods’ season. If there are no more local foods, then we’ll eat processed foods.

**I: and what about fish?**

R: well for fish every time. But it also depends if we have fishing utensils. If we don’t have then we will not eat fish.

**I: what about the fish in canned?**

R: it’s good because it’s doesn’t take too long and much work to consume it

**I: what kind of local foods you have in here? Well I know you have pandanus, coconut tree, lime, breadfruit, do you have taro?**

R: there are some

**I: are there anyone who grow and sell these local foods?**

R: there are none

**I: they’re for eating only right?**

R: yes

**I: what if boats would have come here like everyday, would have been easier for you to sell them?**

R: yes, but we still have not enough. Like if we sell them today, we’ll wait a months for these foods to grow.

**I: so, you must own many gardens, right?**

R: yes, that’s the thing; not enough garden. If you give all your attention to your garden then, you’ll not have money to buy rice.

**I: oh! so what makes money in here?**

R: copra

**I: oh, so you earn money faster when doing copra than gardening right?**

R: yes, we earn money faster when making copra.

**I: people in Ine said that another reason for not making garden is because of not enough gardening utensils**

R: yes, because there are not enough gardening utensils. Even seeds.

**I: beside seeds and gardening utensils, are there any other ways we do to make garden? For example, how much it cost for a bag of copra?**

R: 50cents per pound

**I: like how much pound is that for one bag?**

R: hundred something

R: you’re talking about in Majuro, right? Because it’s 50cents in Majuro.

**I: oh, what about in here?**

R: for outer islands, 48,46,47cents per pound.

**I: oh, the price is lower?! Man, I thought every island is 50cents but Majuro only.**

R: yeah Majuro only. For the field trip 50cents

**I: oh, when the government boats come right**

R: yes

**I: they are doing that because they’re making profit for the person who’s delivering the copra right?**

R: it depends on what ever the price the business will make

**I: so that’s what people been busy with and don’t have time for gardening right?**

R: yes

**I: all of you here have chickens in their houses right? What do you do with the eggs, do you eat them?**

R: sometimes when we need eggs but there are no more at the stores, we’ll then eat the eggs. But we usually let them hatch so there could be more chickens.

**I: one thing we see is, pigs and chickens roaming around but not locked up in cages which is against the law in Majuro. They said that, if you want to own pigs or chickens, you have to make cages for them and make the cages safety. But here, I see pigs roaming around and see that they’re healthy. Now, what is the main reasons for not putting the pigs and chickens in cages?**

R: the reason why people let their pigs or chickens roaming around, is because people of Arno are lazy. As for me, when I let my pigs roaming around, they grow quickly and healthy. When you put them in cages, it’s the opposite because they don’t have enough foods not like if they roam around; they eat many and as much as they want.

**I: now, because the chickens also roam around, it seems like every day the children don’t eat eggs right?**

R: yes not very often

R: if there are some at the store then we’ll buy some for them. Like once in a month

**I: one of the causes for children to have diarrhea is stool. Not only human’s stool but also animal’s. So it says, is there a way to separate the pigs and chickens from where the children are playing?**

R: we can like make their eating place far away from our houses. The more they’re use to their eating place, soon they’ll be adapted to it and won’t come near our houses.

**I: when it comes to drinking water, is every household have water tank?**

R: only few

**I: some don’t have any yet?**

R: yes

**I: the drinking water are from the water tanks right?**

R: yes

**I: and what about the children or babies?**

R: they also drink the water from the water tanks. As for the baby, they breastfeed with their mothers until like 7 or 8 months old, then they’ll drink boil water or water bought from the stores.

**I: are there anything to clean the water tanks?... like in Majuro, we use Clorox.**

R: I think there are none

**I: so you just drink the water from the tank right?**

R: yes we just drink the water. But sometimes when it rain so hard, we pour out the water and clean our water tanks.

**I: are there any filter in the water catchment or in the faucet?**

R: none. Only if your team would love to provide some for us

**I: yes, if they see that it’s another cause for children to have diarrhea, then they’ll also help with this kind of issue. Now this one says, we’ve heard that many families do not wash hands regularly while others do. Can you explain some reasons for this difference?**

R: some don’t wash their hands because they’re in a rush to eat their foods.

**I: why some people wash their hands with soap and others do not?**

R: sometimes there are no more soap

**I: are there any hand sanitizer in here?**

R: no there are none

**I: last question about hygiene is- we all know that this kind of issue happen everywhere; people defecating in the open. So it says, what makes people defecating in the open area? What is the main reason?**

R: the main reason is because they don’t have rest room and they don’t have any supplies to make a rest room.

**I: what is it difficulty for making a rest room?**

R: we can’t afford the materials for a rest room

**I: what if a group or organization help provide the materials, would it be easier to make rest room?**

R: yes it would

**I: land owner’s permission won’t be a problem?**

R: I don’t think so because that’s one of the most important issue in here.

**I: we will now ask few questions about children when they are sick. When children under 2 get sick, some parents take their children to the doctor first and others use traditional healing first. Can you describe the reasons for this difference?... who do you seek first?**

R: the doctor

**I: are there times the mothers take their children to the traditional healers?**

R: yes if the doctor recommend to seek traditional healers

R: also if they have swollen stomach, they take the children to the traditional healers

**I: are there any other sicknesses beside swollen stomach, the traditional healers heal?**

R: yes. When we have issue with our private part.

**I: Now we would like to learn about how parents care for their children. We’ve heard that husbands are an important support for their wives during pregnancy. Can you explain what husbands do to support their wives while they are pregnant?**

R: give them our warmth

R: be there with them during labor

**I: what do they usually eat during pregnancy?**

R: some want to drink coconut, eat fish or lukwor (iu mix with water and milk), chewing pandanus-

**I: are there any foods that people don’t usually eat but they crave for them?**

R: hamburger

**I: so they are here but they want to eat hamburger?**

R: yes

**I: so how do you guys get what they’re craving for?**

R: we make bags of copra right away. As many as we can or at least three bags would do.

**I: We’ve heard that mothers are mainly responsible for taking care of children in most communities. Can you explain the responsibilities of fathers in this community in caring for children?**

R: make money for foods, diaper, formula, clothes

**I: earn money by making copra right?**

R: yes because if we don’t make copra then our children won’t have diapers

**I: when is the right time for fathers to like help their wives taking care of their baby?**

R: when we’re not busy and have time to help then we’ll help parenting.

**I: usually at what time?**

R: usually in the evening

R: when we’re done with our works

R: or maybe in the morning when our wives are busy making breakfast.

**I: that’s also the time the fathers play with their children right?**

R: yes

**I: what about on weekend?**

R: on Sundays

**I: how do the fathers play with their children?**

R: some play with them and tickle them. I think that’s the only play we do.

**I: okay. Sometimes usually at evening, what makes the mothers leave their children and family?**

R: they don’t love us anymore

R: they’re attending programs

**I: where?**

R: like the focus group for example

**I: not like really leave them but like leave them for an hour or so? Like in Majuro, when the fathers came back from work, the mothers will leave the children and them to go to play Bingo or something like that. What about in here?**

R: yes there is a women group called Kumit. It’s for the women to help each other doing copra and handicrafts. So they’ll leave their family.

**I: so at that time, who’s taking care of the children?**

R: the fathers

**I: people in the community, do they also help taking care of the children when the mothers are gone?**

R: yes they do

**I: what about the fathers? Are there anything that make them leave their family? Besides making copra?**

R: yes. When they’re going to help each other cleaning their home town.

**I: wow that’s a big different compare to fathers in Majuro. They’ll leave their family for drinking alcohol. That’s really good. Where do you usually get information about health and nutrition?**

R: from the doctor

**I: is there anyone else beside the doctor?**

R: only the doctor

**I: what about the radio or something like that?**

R: also the radio

**I: these two only?**

R: yes

**I: okay those were the last questions. Thank you again for participating. Are there anything else you wanted to know about but we didn’t talked about?**

R: no we’re good.

**I: okay thanks again and god bless!**
