# Supplementary material for: Conceptualizing multi-level determinants of infant and young child nutrition in the Republic of Marshall Islands–a socio-ecological perspective
Source: PLOS Glob Public Health. 2022 Dec 19;2(12):e0001343. doi: 10.1371/journal.pgph.0001343 (PMC10022247; doi:10.1371/journal.pgph.0001343)
Supplement: S1 Data — (ZIP) [file pgph.0001343.s001.zip › RMI Supp Data/Focus groups data/F12R_FGD_Female_Arno_Sep 27_Libon.docx]

Interview Code: F12R

Interview Type: Female Focus Group

Interview Date: Sep 27 2018

Location: Arno

Interviewer: Libon

Transcriber: Fela

**I: Ok. Do you guys want to participate in this survey?**

R: yes

R: yes

R: yes

**I: ok before we proceed, let’s introduce ourselves. Tell us your name, how many children you have and how old are they and I will go ahead and start. My name is Libon Joseph Jorkan and I have three children two boys and one girls. The eldest is a boy and he is fourteen years old. The next one is also a boy and he is six years old now. My youngest daughter is almost three years old, she will turn three tomorrow.**

R: ok my name is Maley, Maley Ishoda, and I and two children. My eldest son is four years old, and my daughter here is two years old.

R: My name is Miram Namdik, I have three children, the eldest is seventeen, the second eldest is seven and the youngest is five years old.

R: My name is Rulina Antoon, and I have three children, two sons and one daughter. My eldest son is five years old. The second child is three years old, and the youngest is a girl and she is two years old.

R: My name is Eldina Leban, and I have three children. One boy and a girl. My eldest son is thirteen years old, the second one is six and this little girl right here is four years old now.

R: Linda Antoon. I have three children, and all of them are boys. The eldest son is twelve years old. The second eldest is nine and the youngest son is three years old.

R: My name is Ruby Bokadik, I have two children the eldest one is seven and the youngest one is two.

**I: ok that’s great. We will begin by talking about the health of women. Can you tell me what a healthy woman’s body should look like?**

R: good

**I: good body size**

R: not that big size and not that skinny size

R: sexy (all laughing)

**I: ok what else?**

R: no responds

**I: what is a healthy and nutritious diet for pregnant women?**

R: our own local foods the grown foods like pandanus, banana also can be sashimi.

**I: hmm. Let’s say fish. Anything else like sea foods?**

R: fish, crabs, turtles, octopus

**I: do these foods different than foods women normally eat or don’t eat during pregnancy?**

R: can you repeat the question

**I: it’s says “are these foods mentioned different from foods that women normally eat? We are talking here about these nutritious foods. So are these foods different from foods that women normally eat throughout a day? Are they different from the foods that we always want to eat?**

R: yes sometimes we changed our diet and sometimes we usually eat these foods.

**I: and what about the pregnant women?**

R: pregnant women?

R: well these pregnant women usually hate these foods even though they know that these foods are nutritious.

**I: what they usually eat? If not they don’t eat these foods, what kind of foods that they always crave for?**

R: if it’s me, I am making an example about me. I am talking about foods here. I always crave for breadfruits. There is no other foods than eating breadfruit.

**I: just breadfruit**

R: yes

**I: ok.**

R: it’s the same thing for me. I usually eat sashimi during pregnancy. It was the only foods that I always wanted to eat during pregnancy. Mole* (Name of fish that eaten as sashimi)

**I: who or what influences women’s diet during pregnancy?**

R: the nurses

R: the doctors

**I: who else?**

R: our mothers

R: our parents and especially the husband

**I: hmm. Now who or what influence women’s diet during pregnancy? What they usually say?**

R: they usually say “eat nutritious and healthy foods so that when the child born, there shouldn’t be any…

R: illnesses

R: yes illnesses happen to them. The child must born malnutrition.

R: we also can take good care of our health so that the child can live healthy.

**I: and what about you over there. What were some word of advice they usually tell you during pregnancy? Foods that you supposed to eat during pregnancy?**

R: I usually told not to eat salty and greasy foods.

**I: and what kind of food you encouraged to eat during pregnancy?**

R: nutritious foods

**I: Ok. In some places, women are discouraged from eating certain foods during pregnancy. For example in some countries, women are told that if they eat eggs during pregnancy, their child will become a thief. Can you describe any practices like that in this community?**

R: they usually said, we shouldn’t be eating while walking. The reason they say that is when you are about to give birth, it will hurt you a lot and can be really hard for you to give birth.

**I: ok. What else?**

R: no respond

**I: any other practices during pregnancy we can think of? They usually told us not to do because it will affect us during pregnancy?**

R: a pregnancy shouldn’t be sitting in fronts or where people walk because if the person walk at their back, they usually said * erool kijen ajiri en* (when the child eat, they will choke or can be drown in the mothers tummy) when it’s time for the child to come out or the mother giving birth, the thing the child is eating from inside the mother belly will messed up like tangle with the child. It is come from when people walk behind the pregnant lady.

**I: ok what else?**

R: we shouldn’t be waking during night time

**I: and why is that?**

R: they usually said that child can meet ghost at night.

**I: oh ok. And what was that you mentioned over there?**

R: just adding few information from here

**I: ok?**

R: they shouldn’t be showering under the roof tank.

**I: and why?**

R: they also said they’ll meet demons or night ghost.

**I: ok. Now as we all know that some women receive supplements for low blood during pregnancy, like iron-folic acid. Some mothers told us they consumed all the supplements they were given during pregnancy but others did not. Could you explain any reasons why some mothers do not consume all their supplements?**

R: as of me, let me make an example about me. During my first pregnancy, let’s say first time pregnancy. I went to the hospital and they gave me supplements and told me to take them morning, noon and afternoon. I took these supplements and I never liked them. I used to puke until seven month pregnant, I never did so. I never vomited when I took supplements.

**I: you were fine?**

R: yes it was all fine

**I: ok anyone else want to share on why do some women take their supplements during pregnant while some don’t?**

R: for some, um because the pills are too big and when we drink them, we choke. The minute we choke, foods come out too.

R: there are just some who hates the smells of the pills.

**I: ok now where are we? What advice pregnancy women received so that they can take these supplements?**

- **People talk behind the back* (Libon) you have loud noise there. We are doing record!**

**I: ok. One more time. What advice pregnant women to take all given supplements for them?**

R: long pause and no respond

**I: ok was that ok?**

R: again?

**I: what help or advice pregnant women to finish all given supplements during pregnancy?**

R: some drink their supplement while eating candies.

R: some eat before they take supplements

**I: they eat first?**

R: yes

**I: anything else from that side?**

R: no respond

**I: Ok now what are the consequences of having low blood during pregnancy and childbirth?**

R: it is very important for us to take all these supplements because when it is time for us to give birth, it will hard for you to push or hard for you to give birth, and when they want to cut you, there is no way that will work because you have very low blood. Now there is a serious problem that time is to look for pine or blood.

**I: ok anything else?**

R: can you repeat the question?

**I: What are the consequences of having low blood? What will happen to the pregnant woman and the child?**

R: it can cause them dead. And another thing is that when we have low blood, we will be bleeding nonstop or having monthly period without stopping even though we have low blood, we will bleed without stopping. It makes our body weak, and that also affect the lives of the child we carry in our belly.

**I: Now were there any advice you received from health workers to prevent or treat low blood in pregnant women?**

R: Can you repeat that?

**I: it says “were there any advice you received from health workers to prevent or treat low blood in pregnant women?**

R: they usually told us to eat fruits so that it can help make blood or drink orange juice.

**I: ok. Anything else?**

R: take all given supplements and drink them. Especially blood pills.

**I: Ok now let’s talk about feeding babies after they are born. Once the baby is born and you begin breastfeeding, can you describe a healthy and nutritious diet for women who are breastfeeding?**

R: fish, breadfruits,

R: also eat coconut and also eat mackerels or foods that easily make breastmilk for a breastfeeding mother.

R; also eat corn beef

**I: hmm ok similar to what we have discussed earlier about “food taboos” during pregnancy, can you describe any “foods taboos” that exist for women who are breastfeeding?**

R: can you say that again?

**I: we have talked earlier practices during pregnancy like don’t eat crack foods so that the child’s mouth will cut, so now we want to know if we have practices for breastfeeding.**

R: yes for breastfeeding, don’t lay down and breastfeed because the child will never stop crying while growing. They would only want you to hold or carry them.

**I: ok. Anything else?**

R: don’t eat while touch them. They will never share their foods when you eat while touching them when they are little or babies.

R: breastfeed them and when they’re done, make them burb so that they can’t choke or puke.

**I: anything else? Foods that you eat that can affect both you and your child?**

R: don’t eat sashimi because the child will end up biting you while feeding and you will be mad and slap him/her.

**I: you turn mad and slap the child?**

R: yes

**I: ok now what advice have you heard from health workers about breastfeeding?**

R; about breastfeeding, well the advice that I have received from the nurse is when we do exclusive breastfeeding it is very easy for them to receive things or learn. They learn fast.

**I: their brain function well?**

R: it is different when we feed them with different formula, they are slow learner. The store bought milk is not healthy. It makes the child’s brain slow in learning. Because it is the cow animal’s milk that they are feeding from or the store bought milk. That’s what makes them slow in learning. This is the information that I got it from the child’s nurse.

**I: and what about you there? What you have learned from health workers about breastfeeding?**

R: they shouldn’t get illnesses frequently.

**I: anything else? Why is it important for you to exclusively breastfeeding?**

R: there is no cost for it. We don’t have to look for dollars. It’s a free foods for the child.

**I: yes. Now is there any advice from family members about breastfeeding. Can be from your parents, your grandparents or your husbands or people in your house. What they usually advice you about exclusively breastfeed?**

R: they usually tells us about the good thing about exclusive breastfeed. It is important to do exclusive breastfeed so that when the child is sick and refuse to eat. The only thing they will eat is breastmilk.

**I: anything else you have been advised from family members about breastfeeding?**

R: whispering to each other

**I: any advice from family members about breastfeeding?**

R: They advise us to breastfeed exclusively so that when they grow up, they can’t grow malnutrition.

**I: from this side? Any advice from family members about breastfeeding? From your parents or your husbands.**

R: we usually told to exclusive breastfeed so that when there is illnesses on islands, they wouldn’t get serious illnesses.

**I: we have heard that some mothers squeeze out the very first liquid while some don’t. Can you explain why some mothers do and some don’t? Why do some mothers squeeze out the first breastmilk while some don’t?**

R: because they said it colostrum.

**I: colostrum. Ok what else?**

R: what can we say other than that “because it’s colostrum”

R: we don’t know

**I: why do you mean by calling that “colostrum”**

R: it’s not a breastmilk

**I: ok**

R: it’s watery and not the same like breastmilk.

**I: Ok now from your own opinion. Does colostrum important or not?**

R: yes it is important

R: it’s important

R; well as for me, I didn’t squeezed it out, I fed my child with it.

**I: and why didn’t you squeezed it out?**

R: because they never told me to do so

**I: they did not?**

R: they did not

**I: and from you girls, what do you think about the very first breastmilk, is it colostrum or not?**

R: I don’t think that’s a colostrum because it mix with both water and milk. They’re together.

**I; ok now who told you to squeezed out the very first breastmilk? Who you told you to do that?**

R: our grandparents

**I: ok. So who told you not to squeeze out the first breastmilk?**

R: the nurses

**I: did they explain to you any reasons why?**

R: no but they just told me to do so

R: as for me, they never explain anything to me, I just grab a piece of cloth and wipe the nipple. That’s the only thing they told me to do is to wiped the nipple then feed the child.

**I: We’ve heard some mothers first introduce foods other than breast milk when their baby is 6 months old, while some introduce foods earlier or later than 6 months. Could you describe the reasons why some mothers introduce foods or liquids than 6 months of age?**

R: well it’s the months we supposed to feed them according to their birth certificate. We follow the birth certificate.

R: we feed them during six months because when we feed them with breastmilk, they keep crying because they are starve for real meal. When we do breastfeed, the child would still cry but if I give real foods and feed my child, she get full stomach.

**I: ok. Anything else? And why do some parents feed their children during the first three months?**

R: because they said that when children see them eating, they would cry for their parents foods.

R: From their own understanding

**I: and what about the other? And how come there are few children who are more than six months old but still don’t feed with real meal?**

R: some mothers says that the child don’t have spoon to feed the child with

R: some are lazy to make foods for their children

R: the fast and simple way is to feed the child with different liquid the baby bottle

**I: ok. Many mothers have told us that they did not have enough breastmilk to feed their child. Can you explain to me how children under 6 months are fed when their mothers do not have enough breastmilk?**

R: we do both thing. We feed them with breastmilk until we ran out of breastmilk then we would give them different liquid, formula.

**I: so when you do formula, what do you add in the formula?**

R: I used to do formula for my son, the eldest son never tried different formula. My daughter was born three bound weight and she really need to feed from both breastmilk and different formula called smilax.

**I: oh ok.**

R: yes that was the only thing help increase her weight.

**I: ok what else? If you don’t have much breastmilk, what do you do?**

R: we also give them coconut drink as different formula

**I: ok.**

R: we also feed them the (Makon) soften foods made out of boiled pandanus.

**I: Now is there any advice or ways to increase breastmilk?**

R: there are some mothers who massage their breast with the coconut oil so that they say that it is hard for the milk to come out. So if they use the coconut oil, it easily come out and easy for the child to get breastmilk. If the child try to suck for milk, it is hard for them to get it because the mother contain only a little amount of breastmilk. So if we use the coconut oil it help the milk come out easily.

R: there is also one possible way is to drink konat (local plant) * pound it and rinse it in a piece of cloth in a small jar* (Local medicine)

R; yes drink konnat because it does help a lot in providing breastmilk.

**I: what else you can do to help provide breastmilk?**

R: eat fish

**I: hmm ok. So where these advice do came from? Do they come from health workers, family members or from your own?**

R; from family members

R: there is also one “the massaging thing” and it is from the health centre.

R: whenever you do breastfeeding, you have to do massage first.

**I: Could you describe for me how mothers in this community know that it is time to stop breastfeeding their child?**

R: repeat that again please

**I: how mothers in this community know that it is time to stop breastfeeding their child?**

R: they said they can feed from breastmilk until two years old or it is up to us if we want to stop them from breastfeeding.

**I: and what about you ladies on this side? How do you know that it is time for the child to stop breastfeeding?**

R: we do breastfeed until I ran out of breastmilk.

**I: so you did breastmilk until you ran out of?**

R: yes

R; five years old

**I: oh ok. And what about you over there young lady? How did you know that it is time for the child to stop breastfeeding?**

R: until he was five years old.

**I: oh ok. And you pretty girl over there? How did you know that your son has to stop breastfeeding?**

R: he breastfed until he never wanted to do so.

**I: so it was him that he never want to feed from the breastmilk?**

R: yes

**I; and you over there?**

R: because she is too grown for breastfeed. He eats a lot and she has to stop breastfeed.

**I; so it is better to eat real meal than feeding them breastmilk.**

R: yes

**I: Some people have mentioned that they try to feed their young children a balanced diet. Can you explain what people mean by a balanced diet?**

R: If they eat rice with meat, there should also fruits and vegetables in their meals. I am talking from my own understanding.

**I: What else?**

R: we have to eat nutritious food. Eat from the three group of foods. For example, you can have fish, there should be coconut drink, papaya,

**I: so you meant there should be energy food, body building food and protective foods**

R: yes

**I: ok. For the last question about feeding children we’d like to learn about how decisions are made. Can you explain anything that influences mother decisions about feeding their young children in this community? Why it was really important do exclusive breastfeeding?**

R: so that illnesses can’t easily affect them

R: because they have told us that breastmilk is much healthier than any other given formula for the child. It smells fishy and it is healthy for the child.

R: another thing is that. We don’t have to worry about how much money we need to save so that we can be able to buy milk from the store for the child. It is better for the child to feed from my breastmilk so that I can’t worry to buy milk from the store.

**I: yes. Ok now we would like to ask a few questions about children when they are sick. When children under two get sick, some parents take their children to the doctor first and others use traditional healing first. Can you describe the reasons for the differences?**

R; yes there is a quit different. If the child get fever inner the body, they can have fever for too long. We take the child to the hospital and treat him/her with Tylenol medicine. We keep treating children until they finish the Tylenol but still can’t be heal. Then when we bring the sick child to the traditional healer, they check and see that he/she is having stomach lump. After the child do stomach massage, he/she heal right away? There is nothing the Tylenol medicine can do to heal the child from stomach lump. Stomach massage helps a lot to heal the child from suffering. After they do stomach massages, the child heal and can now heal and able to eat or play.

**I: ok. What else?**

R: what again?

**I: what are the differences when you take the ill child to the health care from taking the child to the traditional healer?**

R: the traditional healer doesn’t cost anything while the health care centre cost enough.

**I: Now from your own opinion, which one is better and fast?**

R: My answer would be both.

**I: and what’s fast and reliable?**

R: well when we talk about fever, well let’s say that traditional healer would be more fast and easy to heal the child from illness.

**I: what illnesses are commonly treated with traditional medicine?**

R: stomach massage

**I: if they have stomach lump we do stomach massages ok.**

R: if they have the sickness called “yellow” * illness that cause the body and the eyeballs turn yellow* we use the medicine to heal them called “Kijonkan” and children that can’t stop coughing we make local medicine made out of the banana stem and give them for healing drinking.

**I: so what about the sickness that you just mentioned earlier fever**

R: we do stomach massages. The stomach massager do that.

**I: ok anything else?**

R: there are just some who sometimes have nausea illness. They go to the hospital for medicine but these medicines can’t heal them from nausea or dizzy. If they don’t get better than the situation they are in. We use the local medicine to heal them from nausea. It is just a local medicine to heal from nausea

**I: ok nausea healing local medicine. So what kind of traditional medicines are used for each illness?**

R: for what nausea illness?

**I: If it was nausea illnesses what kind of traditional medicine used for nausea?**

R: the noni tree fruits. Am I right?

R: yes

**I: and what about fever?**

R: we do stomach massage

**I: what do you use to do stomach massage?**

R: the coconut oil

R; there are some children that the fever is inside the body and it’s hard to heal from the fever then we use the “mokadkad” (Type of banana) banana stem to make their drink from it.

**I: Mokadkad banana?**

R: yes

**I: ok so who influence whether traditional medicine is used?**

R: our mothers

**I: ok. Our mothers**

R: just us

**I: ok. Can you describe how children are fed when they are sick compared to when they are not sick?**

R: one more time please?

**I: Can you describe how children are fed when they are sick compared to when they are not sick**

R; if the child is sick, we do breastfeeding.

R: when they are sick? How do we feed them?

**I: yes**

R: we feed them often. We feed them now, later on, minutes after. We don’t feed them morning then wait till noon and feed them. If they’re not sick, well you can feed them breakfast, lunch and dinner.

**I: ok. And what if they’re having diarrhea?**

R: Diarrhea? If they have diarrhea, we give them water and coconut drinks. Since we are there in the outer islands, these are the drinks that we commonly told to give them when they’re having diarrhea. But when we are in Majuro, we usually told to give them IV or medicine from the hospital.

**I: so why do you give water for them when they have diarrhea? What’s the process of water during diarrhea?**

R; because when they have diarrhea, they lose lot of water by pooping water only or pee a lot and it is really important for them to drink water so that it can prevent them from dehydrated.

**I: ok. So what kind of foods given for children when they are sick?**

R; as for me, whenever my children get sick, I usually make “lukor” (Made out of coconut meat mix with water and milk-soften and sweet food) because I think it is the only food they want to eat when they’re sick.

R; some usually want to eat pandanus when they’re sick. They also want to eat banana. They usually want to eat sweets foods.

**I: ok so what kind of foods given when they’re sick?**

R: any kind of foods that they ask for

R: there wouldn’t be anything else aside from rice

**I: so what kind of drinks you give them when they’re sick?**

R: water and coconut drinks

**I: ok so what kind of drinks you give them when they’re not sick?**

R: any kind of drinks

**I: is there any quantity of water given for them when they’re sick or not?**

R; no respond

**I: for example, if they get diarrhea, is there any quantity of drinks give for them?**

R; we just give them the drink

**I: Now we would like to learn about the foods that you provide for your family. Could you talk about what influences which foods people in this community provide for their families?**

R: can you repeat the question again?

**I: can you talk about what influences which foods people in this community provide for their families?**

R: no respond

**I: why is it important to you to provide foods for your families?**

R: it is important to provide foods for the families so that they can’t get any illnesses that people commonly suffered from nowadays. People with diabetics should eat foods that’s appropriate for their health so that their health can’t get worst. Anything else you want to share ladies? “Share something”

**I: so what if it was children?**

R; children?

**I: yes why would it be important to you to provide appropriate foods that you would want to provide to your children?**

R; foods that good for their health since I know all of us here don’t want our children to get sick. We should give them healthy and nutritious foods.

**I: ok so is there any difficulties in getting the foods you want for your families?**

R: yes of course there are sometimes of difficulties in getting foods we want for our families. If we wanted our children to drink juice, sometimes we don’t have money to get juice for them. This is also a difficulty. Another thing is when you want your children to eat fish but the problem is the husband can’t fished.

R: there is no one to climb the coconut tree for the coconut drink. There is no one to climb the breadfruits tree to get us breadfruits.

**I: ok so how do the family deal with food shortages**

R: they have to work a lot

R: make copra and sell them so that they can get enough foods for the families.

R; they should go fishing so that they can sell the fishes to get money to buy foods

R: they also can sell or trade local foods to get money so that they can be able to buy foods.

**I: so what if you wanted to buy foods for the families but you don’t have enough money to buy the food, what would you do so that you can get enough foods for the families?**

R; ask for depth

**I: and what if you are not allow for the depth but you have the last money and it’s not enough**

R: we can buy let’s say a mackerel, a ramen, and a soy sauce and make gravy soup so that it can be enough for the whole family.

**I: ok what else?**

R: ok so if I had the last money but there are lot of people in the family. I knew you would chose to say that only children can eat. Bring the food and feed the children and let the eldest be energized enough for hours later.

**I: hmm that can be one solution. Many families have told us that they eat local foods when they don’t have processed or imported foods or if they don’t have enough money to get foods. Can you describe any other reasons that families do not eat local foods?**

R: in places like outer islands, we get foods when it’s the season for the foods. If it’s the season for breadfruits, then we eat breadfruits. If it’s the season for pandanus, then we get pandanus. Yes there is foods here on Islands but they are seasonal foods and we eat them during the season.

**I: so why don’t you just eat fish if there is fish, or turtle…**

R: the reason we don’t eat fish is because of Rough Ocean or lagoon side, there is no one can be able to go out and fished when we have rough and bad weather.

**I: ok. Now can you describe the process of fruits leaf if there is any? What kind of foods that you can be able to cook foods out of it?**

R: the pumpkin leaf

**I: ok how do you do that?**

R: we cut the pumpkin leaf into pieces and boil it together with meats

**I: oh ok. So do you eat the leaf?**

R; yes the green leaf is eatable we eat it but when only if it cooked, when it not cooked, then we don’t eat it.

**I: oh**

R: when it boiled and has been cooked with the meat, then we eat it

**I: like the Pele? * Local grown food***

R: yes when it cooked you can eat it

**I: oh ok, so that’s just like the cabbage to you right and you can cook it with any kind of ingredient right?**

R: yes

**I: ok. Now we have seen that some families here raise chicken. Can you explain what do people raise chicken for? For example, do they eat them during meals or what do they do with their raise chicken or do they gather the eggs?**

R: maybe I will go ahead and answer that question

R: *laughing*

R; hmm let me make an example about my household. Yes we do collect the eggs, the hen lay eggs in their case then we gather the eggs and cook them for the children or we also can use to eggs to cook our foods with. But you know the chicken, when there is important people visiting the islands, then we cook the children and offer them to our guests.

R: sell them

R: there are times that we don’t have enough cash with us but there is raised chicken, then we can sell them as a trade.

**I: but to eat raised chicken, it’s not common? Like not all the time?**

R: not very often

**I: and why is that?**

R: uh? How come we don’t eat them? Hm what can I say ladies, why don’t we eat our raised chicken?

R: well sometimes we don’t feel like we want to eat chicken

**I: oh ok**

R: the thing is that in my house, my mother doesn’t eat raised chicken. Eating chicken for her is just a disgusting thing. That’s why we don’t really eat chicken at home. We eat less than we offer to our guests.

**I: ok in the next section, we would like to talk about water and hygiene. Can you please describe how people get water for their families in this community?**

R: um seems like we don’t need water here because each household have their own..

R: bantoon or water catchment

R: aside from drought, it is the perfect time that we convert salt or well water to drink water. That’s the water people in this community will live by it.

**I: oh ok. So you guys have that thing you use to convert water?**

R: yes

**I: that’s great, so do you have any difficulties in getting water?**

R; yes there is. Sorry regarding on what?

**I: difficulties in getting water from places, well water or from people’s houses?**

R: yes if we ran out of water, and if the well water is not clean, we goes from house to house and get water.

**I: so is there any difficulties in storing water?**

R: “storing water” when we’re about to ran out of water?

**I: anytime. Do you have any difficulties in storing water?**

R: there is no difficulties

**I: do you have enough water catchment?**

R; we don’t have enough water catchment

**I: ok that can be one difficulties because there is not enough water catchment. Ok. We’ve heard some families boil their water for drinking and others do not. Can you explain why some people boil their water and others do not?**

R: because they are too lazy to boil drinking water, so that cause them to stop boiling water and can just give the water that’s not boiled for the child to drink it.

R; let’s say they have been boiling water from the beginning and in the middle of the family living together, they stopped because mothers are lazy to do the process of boiling water.

**I: what else?**

R: there are some parents who keep boiling their children’s water because their children are used to drink boiled water. If they stopped for only a single day and not drink boiled water, then the child can have diarrhea because they’re not use to not boiled water.

**I: so the water that didn’t boil are good or bad or drinking?**

R; yes

R: the water are clean, let’s say if it rain water then it’s clean

R: the water tank have already tank together with the tanks. We have water filter for the water catchment. We can just drink because there is water filter to clean the water.

**I: what are some difficulties in boiling water every day?**

R; there are sometimes we boiled water because the water comes from the roof and there is rust on the roof.

**I: ok so do you have any difficulties in boiling water? Why do you stopped from boiling water sometimes. What are the difficulties you face?**

R; sometimes we don’t have the wood to start the fire

R: sometimes we don’t have the money to buy gas for the stove.

R; there is also one thing, if we are too lazy to boil water, then we won’t boil the water.

**I: ok so is there any difficulties cleaning your water catchment?**

R: there is none

**I: Is there any difficulties in cleaning the water catchment? Or keeping the water catchment clean? You mentioned that there is no difficulties in keeping the water catchment clean so you mean that you clean your roof, the tanks, and the water catchment every day?**

R: no respond

**I: ok. What makes it easy to keep or clean the water catchments?**

R: your turn now.

R: can you repeat that again please?

**I: it says” what makes it easy to keep or clean the water catchments?**

R: what makes it easy..

**I: why is it easy to keep water catchment clean? We’ve talked about difficulties but now we are talking about the simplicity of keeping water catchment clean. What makes it easy for your water catchment to be clean?**

R: men in our household clean our water catchments. Let’s say it never rained over three days so you have to cover the bantoon with the lid. Then if it rain on the fourth day, you must not just open the lid to catch water. You have to wait maybe few hours then you can now open the water catchment lid. That’s also one thing that can help keep the water clean.

**I: so you mean you have to remove the dust from the roof first. Ok. We’ve heard some families wash hands regularly while others do not. Can you explain some reasons for this difference?**

R: sometimes when they are in rush, they forgot to wash their hands, then after when they remember about hands washing, they already eat.

**I: ok**

R: the family that do hands washing all time is the one that don’t want to have diarrhea. They prevent themselves from having diarrhea.

**I: ok.**

R: prevent their children from germs

**I: but is that a habit for us**

R: yes it is we have to wash our hands before we eat

**I: it’s a habit**

R: yes

**I: Now why do some people wash their hands with soap and others do not? They just wash it water?**

R: because they are in rush

R: or maybe because there is no soap to wash hands with. Or maybe that person save the soap to use it for bathing and wash hands only with water.

**I: yes these are good information**

R: yes but there is a different I see when we don’t use soap for hands washing we can still feel that it’s still not clean. When you use soap for hand washing, you can feel it soft and slippery.

**I: ohh**

R: then we realize and can feel that we have clean hands

**I: ok now what are the main things that prevent washing hands with soap regularly?**

R: I don’t think there is any

**I: what are the main things that prevent you from wash your hand with soap and water?**

R: because we are in rush

R: also lazy. If the soap is not close enough to where you are, you can just use water without soap to wash hands

**I: yes. Ok now what are some reasons why some people use hand sanitizer instead of soap?**

R: because some belief that hand sanitizer kills bacteria and germs better than soap. Hand sanitizer kills bacteria and germs while the soap just clean our hands, it does not kills bacteria or germs.

**I: ok what else?**

R: no respond

**I: anything else? Why do some people use hand sanitizer aside from soap?**

R: it is simple to use

R: just bring it and use it

**I: Now for the last question, we would like to learn about how parents care for their children. We’ve heard that husbands are an important support for their wives during pregnancy. Can you explain what husbands do to support their wives while they are pregnant?**

R: during pregnancy, they have change and take over all of our responsibilities. Women’s responsibilities. They’re the ones to watch over the other children. Like change clothes or diaper, or feed them.

**I: anything else? What do your partner usually do during your pregnancy?**

R: when we crave for lukor (the foods that made our of coconut meat) they go out and bring coconut meat and make our lukor. They carry our bathing water.

R: they’re there to take care of us in times of nausea, dizzy or puke

**I: ok. What mothers or other family members do to support their daughter while they are pregnant?**

R: well I can’t answer that question because I have never been near my mother when I am pregnant. Maybe I’ll let you answer it.

R: can you repeat that again?

**I: it says “what do mothers or other family members do to support their daughter while they are pregnant?**

R: they usually cook our foods, and another thing, they usually advise us

**I: on what?**

R: foods that we supposed to eat, or prevent us from walking roaming around during pregnancy

**I: supplements?**

R: also to take our given supplements

**I: we are interested in learning about how caregivers play with children under two years old. Can you describe for me in detail how you play with children?**

R: We make them feel happy, make them laugh. Take them to the kids play ground so that they can play there.

**I: anything else?**

R: teach them how to count, learning the alphabet or colouring. These are my ways of playing with children

**I: We’ve heard that some parents spend time outside of the home and it may affect the way they raise their young children. Can you tell me about your experience with this?**

R: “parents that don’t really taking care of their children” Parents take their time separately from family doing will never know that their children are sick or not. They will never know that the child is having fever. Sometimes mothers would cook and leave the boiling water on the fire and leave it there then the child can play near it and drown in the pot. These are some example when parents don’t really have time to take care of their children.

**I: How these activities affect the feeding of children under two years old?**

R: like I explain it before, the child will easily get sick like fever, drowning. I think these are the common activities children under two easily affected by.

R: diarrhea also

**I: ok what about diarrhea?**

R: whenever they scrawl on the floor or ground, it easily make them eat their hands and cause diarrhea.

**I: and how these activities affect the hygiene of children under two?**

R: no respond

**I: does the child stay clean by being alone?**

R: no. if the parents are not there to take care of the child. It is easy for them to get dirt or be in danger.

**I: what kinds of activities women or mothers are doing outside of the home? Whenever they go out and roam around?**

R: telling stories

**I: what else?**

R: do their works

R: play games outside of the house. If they could leave their children and go out and play outside.

R: do whatever they want

**I: their wants?**

R: yes

**I: and what about the dads? What do dad usually do?**

R: spare fishing, making copra

**I: ok. Great. We have heard from some people that they prefer to get health messages from the radio, others say from the newspaper. Can you describe for me best ways to reach people with information on health in this community?**

R: go straight to the health centre

**I: ok**

R: get information from there

R: announce the information on the radios.

**I: aside from listening to the radio, what else would be easy for you to reach these information?**

R: you can use the cell phone to call since we can call from here to the health centre.

**I: ok what else?**

R: use the vehicle

**I: for transpiration. So do you have community centre to give these information on health? For example, women. Men, or church organization?**

R: yes there is

**I: ok what kind of organizations?**

R: well for example a church have separate branch of organization.

**I: so it would be easy if the church deliver messages to people in that church**

R: yes

**I: ok. So is there any community groups that could be a good place to deliver health messages, for example women’s group or mother’s group?**

R: can you repeat that again?

**I: is there any community groups that could be good in deliver health messages, for example women’s or mother’s group? Is there any women’s leader who could’ve deliver health messages?**

R: yes we have here like the KUMIT organization. (Coalition Prevention Club)

**I: would it be helpful if these information would deliver by them or they would be the ones to deliver health messages to the community?**

R: yes

**I: it would be easier for the community.**

R: yes

**I: ok we’re done now. Thank you your generous time and for sharing your generous thoughts. We greatly appreciate your help and we hope this research will help us improve the health of mothers and children in this community. It’s a pleasure for all of you leaving your places just to come here and share all your information with us today. Thank you very much.**

R: thank you

R: thanks to you too
