# Supplementary material for: Conceptualizing multi-level determinants of infant and young child nutrition in the Republic of Marshall Islands–a socio-ecological perspective
Source: PLOS Glob Public Health. 2022 Dec 19;2(12):e0001343. doi: 10.1371/journal.pgph.0001343 (PMC10022247; doi:10.1371/journal.pgph.0001343)
Supplement: S1 Data — (ZIP) [file pgph.0001343.s001.zip › RMI Supp Data/Focus groups data/F13R_FGD_Male_Arno_Sep 27_Balton_Marcellina.docx]

Interviw Code: F13R

Interview Type and Interviewee- MFG

Interview Date: Sep 27 2018

Interviewer: Belton

Transcriber: Fela

**I: ok we can start now. We are doing a record and I will need you guys to share your information in this record because this recording will be translate by some of our co-workers who have been assigned to translate these information. We do record here and we will translate it to English to the foreigners so that they can understand what we are talking about. These questions are about water and hygiene, lifestyle in our communities. The first thing is about food and health of our children. Could you talk about what influences which food people in this community provide for their families? What influence us to eat this food and not that food?**

R: no respond

**I: for example, what did you have for breakfast this morning?**

R: I eat rice

**I: rice and?**

R: rice and chicken

**I: ok and what about you?**

R: I had pancake for breakfast

**I: ok now you that each people had different meal right? Now why do some people eat this kind of food while some eat that kind of food? What influence which food we eat?**

R: from my own perspective, there are just some foods that we want to eat and there are some foods we don’t want to eat them. And it also depend on how much money do we have in our pocket.

**I: have you guys had any famine here on island?**

R: regarding local foods?

**I: any kind of food. Any famine on rice food?**

R: yes last year it affect both Majuro and Arno

**I: so how did you survive without rice?**

R: we only ate bread and ramen

**I: bread and ramen hm ok**

R: but when it comes to local foods, there are seasonal food. The famine was on the season that we did not have breadfruits and pandanus

**I: there weren’t any of these foods?**

R: yes both breadfruits and pandanus and it made all people to end up eating only bread and ramen

**I: on what foods people in this community usually eat? Local or processed foods? In different community, do you commonly eat can food or either fruits and vegetables or fish?**

R; well the rest will answer from their own but from me, I usually eat processed foods.

**I: like can foods?**

R: yes. It’s not that there is difficulties and all that but because it is simple and easy to get. I am not really sure about the rest but for me, I eat can meat because I don’t really fished a lot but maybe the rest of the guys here usually eat fish because they can fished while I can’t.

**I: what is the main role of a man each and every day? Men in this community, what do they do throughout a day?**

R: making copra

R: copra making

**I: so you eat can meat because it is the most fast and reliable way to be able to back to your work. Right? Is this happen to all of us here?**

R: yes

**I: yes. Is there any foods- well I don’t know if there is beans or cabbage, is there any vegetable leaf here that we can eat them?**

R: yes

**I: like what?**

R: the plant that was planted by Teruo (owner of the hotel in Arno Arno)

**I: oh the one the foreigners brought here?**

R: that’s the plant leaf people in this community usually use to make their soup or cook different ingredient with.

R: also the pele leaf (local grown plant)

**I: where can we get that pele plant from?**

R: there are just few house that has that pele plant

R: yes we cook our foods and add pele on the foods as different ingredient

**I: so they don’t cook alone? We cook it together with our foods?**

R: yes

**I: also the one in that house over there?**

R: yes

**I: we’ve talk with Ine people and ask them why they don’t make their own plant growing and they have told us that the reason they don’t do planting is because they don’t have tools to use for the farming. And another thing is they’re busy making copra and things like that. Is that the same like here in this community? Is there someone owning a farm or plant grow here?**

R: we can do plants growing here but the problem is that we don’t have seeds to grow plants

**I; seeds?**

R: yes growing foods is not a big deal but the only problem we have here is we don’t have the seeds to grow these foods like green beans or any kind of grown foods. They give to each person so that they can plant their own food.

**I: so they give to every single person to plant?**

R: yes that was few years ago until they stopped I don’t know why maybe the Laura Farm in Majuro has stopped from sharing their seeds to people because they don’t have much

**I: so they stopped because they don’t have much with them. Ok so what about tools and things like that? Is that also a problem here?**

R: there is nothing problem with tools, because we can borrow from others who have them but the only problem is seeds.

**I: I have seen you know the small red houses here on islands, there is no in Ine, and I only can see them here. Is that a cage for chicken?**

R: yes

**I: and I can see that there is no chicken in these small cages?**

R: yes that was like a granted regarding women’s program. It was granted from the Australia. It was a written granted from an Australian woman to women here and some have that red cage for chicken and some don’t have one because those who don’t have ones weren’t there when the organization first started.

**I: hmm**

R: yes she gave us the red cage and foreign chicken so that we could raise them. The problem with these foreign chickens is about food given for them. They’re picky on what foods they want to eat or not. It went that way until we didn’t have enough budget with us to buy foods they chose to eat. They picked on their foods and starved to death.

**I: hmm**

R: they were about to trade these foreign raised chicken to local raised children.

**I: what were these foreign chicken raised for?**

R: for selling or trading with local chicken. Sell the eggs laid by the foreign chicken but the only difficulty in raising these chickens was their picky on foods. We fed them local foods and they start dying and now we lost all of them. They couldn’t survived eating local foods.

**I: maybe they never used to local foods**

R: yes and the women should trained them on what foods they should be eating or the way we raised them. It should’ve been local chicken but maybe because it was a foreigner that involve in the organization she had to give us the foreign chicken to raise.

**I: so what about local chicken? The raised chicken here are led lose or is there any chicken kept in their cages?**

R: few

**I: Ok few**

R: not quite all of them

**I: all are led lose**

R: yes

**I: so the laid eggs, do we keep them or we wait until they hatch so that we can have more raise chicken?**

R: we don’t eat the egg, we make more raise chicken

**I: so why do animals don’t kept in their cages? Let’s say it doesn’t matter for chicken, but for pigs? I heard that there was law enforce on keeping pigs in their cages. Why pigs are not kept in their cages? I can only see few pigs in their cages and most the raised pigs are led lose.**

R: well because most of the people here are bad people. They don’t want to follow the law. Maybe the government is weak in enforcing law against animals that scatter and damage the community.

R: there is a law that if a pig comes in to your property, you report to whom it belong to. Then if they don’t show up and claim it within five day, then you can have it or kill it and eat it.

**I: oh ok**

R: yes but because today there are lot of bad young boys out there-

R: hey I am not one of them. I let go of my animals so that they can roam around and look for their own foods. I don’t want to keep them in the fence because if they keep in their cages for too long, they’re dying and can die in the fence.

**I; how can the pig feel dying or why does it feel that way?**

R: if we don’t feed it more often, then it feel weak because there is no one to give it foods or drink

**I: and what if it let loose?**

R: it can eat whatever is there and is eatable

R: he owns it but he never want to raise it

R: no it’s not like that it live better when it’s led lose

**I: ok**

R: the bad thing about letting these pigs go is they damage our yard. They dig our ground and damage our environment.

R: He only can say he want to keep this raise animal but he refuse to feed them and give them drinks. He let his pigs roam around our areas and damages our yard but he never care. (All laughing)

**I: ok we are now on the water and hygiene section. On our studies in Ine, some families boiled water for their children and some don’t. What is the difference between these two?**

R: well what did these people said, why do they boiled water?

**I: there was one family member mentioned that the reason why they boiled water is when they make tea, coffee or water for the children. Or because they are drinking from the cement water catchment and the reason they are using cement water tank is because they hasn’t get their pontoon share from the government. And some families mentioned that they don’t boil water for drink. What are some reason why people boil water? Or what? Do you boil water or not?**

R: any information from that side guys

R: there are just some who boil water because they said that they are preventing sickness from their children.

**I: and what about the adult?**

R: there are some adult who don’t boil their drinking water. They can just drink water without boiling it.

R: and there are some families that see if their roofs are not clean, then they boil their drinking water.

**I: is there any difficulties in getting pontoon here on Island? Or the local government give each household their own share?**

R: not the local government but the government of the Marshall Islands

**I: so the RMI government share these and there are some houses that don’t have their share yet?**

R: yes few more hasn’t get their pontoon

**I: whenever we ride the vehicle and do our work around this island, I see houses that there is no people living in them but have pontoon outside of these houses. There is also a solar energy standing outside of these houses. So how do keep water catchment or the pontoon clean?**

R: drain out the water and wash both inside and outside of it

**I: you do that all the time?**

R: for example each families have their own time schedule of cleaning their own water catchment. Some clean their water tank months after months while others clean every months. They drain out the water and clean the inside part of the water tank. They usually clean their water tank whenever its rain.

**I: anyone use Clorox to help prevent germs and bacteria in the water tank?**

R: well I do not know about the rest of the male here but I do that all the time. I put Clorox in our drinking water. The reason why I do that is because my roof is damage. The EPA people came last month and taught us the formula on adding Clorox in our drinking water.

**I: oh ok. So you put it in your pontoon?**

R: Yes not only the pontoon but also the one or five gallon drinking water and we kept doing that practice until now.

**I: ok. So now when it’s comes to hand washing. When do we usually wash our hands throughout the day?**

R: any answers from there?

R: early in the morning

R: well for me, I wash my hands before I eat

R: after doing works around the house

**I: when do soap use throughout a day?**

R: what kind of soap?

**I: any kind of soap use to wash hands**

R: well, I usually don’t use soap except from when I take a bath that’s the time that I use soap but I am not sure about the other male here.

R: I only use soap for bathing and not for hand washing. The time I use soap is at ten P.M night time when I take a bath.

R: I do hand washing after I do works.

**I: using soap?**

R: yes I use soap to wash my hands

**I: I heard ten o’clock what was that about?**

R: every ten O’clock at night I wash my hands with soap

**I: In some communities, we have heard that defecating in the open area is common. The question is asking, why do people do defecate in open areas even though they have their own toilet?**

R: um well what if we...

**I: I am sorry not “even though they have their own toilet” What is the main reason why do people do defecating in open areas?**

R: And for others maybe that’s a habit for them

R: they were raised and doing that practice since growing

R: there are some people who doesn’t want to use the toilet bowl. I have an outer Island friend that said he never use toilet bowl because he is used to defecate in the beaches. He is used to that because most outer islands defecating in open beaches it’s either lagoon beach or ocean side beach.

R: it’s not only him doing that. All over the outer islands places do that because it’s a habit for outer islands people.

**I: is that because they don’t have toilet to use?**

R: no it’s just a habit for them to do that in open beaches. There are some people who have their toilet but majority of them want to use beaches as their defecating area.

**I: majority of them**

R: Yes majority

**I: is there any difficulties in building toilets?**

R: there are lot difficulties in building our toilet. Sometimes we don’t have enough budget with us to bring materials so that we can be able to build toilets. The toilet bowl itself cost five hundred some dollars. The fact is that, businesses in outer islands have already increase their prices and everything today cost a lot. It is hard for some people to get their materials when materials is too expensive. If you want to buy materials for spare fishing, the money wouldn’t be enough because the materials are really expensive. You haven’t compare that to a toilet’s material and their costs. It is better to spend our money on foods than on toilets materials because we don’t have enough budget to spend on our needs and wants at the same time.

**I; there should be a someone to host a program of building toilet for people like the one you build for the raise chickens**.

R: well these are the main important things outer islands people really wish to have

**I: do you think that would help prevent people from defecate in open beaches?**

R: yes of course

R: you can help with that project to be done. A project that can help build toilet for each houses here and Arno should be the first place you can think of.

**I: you children’s stools are typically disposed of?**

R: some use the shovel to throw them away and some dig the ground and bury them

**I: what kind of illnesses children here usually suffered from? Can’t be just your children but children here in Majuro. What kind of illnesses they usually suffered from?**

R: Fever

R: coughing

R: runny nose

R: nausea

R: pink eye

R: diarrhea

**I: when children suffered from their illnesses, who would be the first one you bring the child to?**

R: to take care of?

I: yes to check them up, and treat them?

R: the doctors

**I: do you bring your ill children for traditional medicine treatment?**

R: yes

**I: what kind of illnesses**

R: stomach massage

R: yes because sometimes when we bring them for medical check-up, they still can’t be heal. But when we do stomach massage, they heal right away from suffered from their sickness. If you take them to the hospital for medical treatment and they can’t be heal from these medicines, then we know that they are having stomach massages.

R: there is also local medicines for nausea and diarrhea that we used to heal children from with when medicines from the hospitals don’t work for them.

**I: yes because yesterday, there was a little boy fell down from the coconut tree but he was lucky he fell on the sand beach. He broke his arms and couldn’t stop crying. The health assistant was there and he was able to help him. He told him that he was lucky to fell on the sand beach and couldn’t broke both of his arms. Then I told him what did he to do help the boy? He said that the condition that the boy was in was really serious and he couldn’t help it so he brought it to a traditional healer to massage the broken arm**.

**I: ok now let’s move on to gender and family roles. We have studied about women pregnancy and our survey asked what would be the most important thing pregnancy women would want during pregnancy. They mentioned that they always want their husbands to be there during pregnancy. Above all choices, they never mentioned that they want their parents or their grandparents, they just want their husbands. Now the question is asked,” Why”?**

R: who answered that question a woman or a man?

**I: woman**

R: well maybe she was lying

R: * all laughing*

R: no maybe she is right. This is our belief that pregnancy women usually do they need us during pregnancy. They also need their husband during delivery. During pregnancy, she wouldn’t need anyone except from the husband.

**I: what husband usually do during women’s pregnancy?**

R: they bring the fish that the pregnancy woman want.

R: the important thing these pregnancy women usually want is their husbands to be there and kiss them. That’s what they want from their husbands.

**I: what kind of foods they usually craved for?**

R: pregnancy women in outer islands always want to eat “konauwe” (Baby coconut)

R: some craved for soap

**I: soap?**

R: yes. And un-cook rice. They have their different crave practices. Some want to eat the fish under the reef where there the sharks spotted at.

**I: in many communities, the main responsible for women is to take care of the children, so what is the responsible for the fathers?**

R: hunting

**I: in what ways**

R: they go fishing or climbing coconut tree. Bring coconut meat, and getting the breadfruit from the breadfruit tree. The family is able to live because they are the one to bring the food on the table.

**I: is there any time for men to watch over their children?**

R: yes. If there is important even happening here, we watch over the child, or if the women is busy then we are responsible in taking care of the child.

**I: On Majuro, sometimes mothers would just stand up and walk out of the house and tell the husband to watch over the child because it’s their turn to go out and do whatever they want to do. If they want to go out for gambling thing they go and tell their husband that they have be there and take care of the child. Does that happen to women and men here in this community?**

R: sometimes women would tell their husband that they want to go and play volleyball. Then the husbands would scold her to stay and watch over the child because there is men there and he doesn’t want her to show up when there is men there. Sometimes that’s happen but not often

**I: is there any time man do the same thing.**

R: what?

**I: telling their wives to stay because they are going out and play baseball or do things out there?**

R: well men mostly are not there near their children and their mother because they are out there hunting for the family

**I: in the evening**

R: they start hunting from early in the morning. They wake up and hunt for breakfast, then would also go out and hunt for lunch and do the same thing for dinner.

**I: so the reason why they are not there is because they’re out there hunting or doing works around the house?**

R: yes

**I: who usually taken care of children whom their parents can be died or left another islands? They grow up with their family members, with their grandparents or with their aunties and uncle? Is there anything that the community can do to help orphan children?**

R: help regarding on?

**I: anything regarding raising or taking care of children**

R: sometimes when we’re busy doing house chores or works, we bring them to our sisters and brothers to watch over them.

R: that’s the only thing they do by helping us raising children, when it comes to food lending and all that, well we don’t really do that here.

**I: so family members also involve in child care?**

R: yes whenever we’re busy hunting for foods or doing works, they take responsible in child caring. Or if we tell them that we are going to that small islands and hunt for foods, then also take responsible in taking care of the child.

**I: hmm. Ok. Where do you usually get messages on health and children raised here in this community? The most trusted information. Where do you get these information from?**

R: from family planning or the health centre

R: for me, I get messages from my parents

R: from our grandparents

**I: anywhere else? You usually get health and children raised messages from the hospital right?**

R: yes and the program that called “Youth to Youth”

**I: Youth to Youth. Ok. From the radio?**

R: yes

**I: ok that’s great.**

R: they usually give us messages on family planning and how…

**I: so do they also give information on health and nutritious?**

R: yes the cause of malnutrition is when children are not given foods from the three groups of foods.

**I: ok that was the last question and I want to thank you all especially this pastor right here. On behalf of the Minister and my bosses these UNICEF ladies we want to thank you all for sharing your information. Bless us all and May God be with us**
