# Supplementary material for: Conceptualizing multi-level determinants of infant and young child nutrition in the Republic of Marshall Islands–a socio-ecological perspective
Source: PLOS Glob Public Health. 2022 Dec 19;2(12):e0001343. doi: 10.1371/journal.pgph.0001343 (PMC10022247; doi:10.1371/journal.pgph.0001343)
Supplement: S1 Data — (ZIP) [file pgph.0001343.s001.zip › RMI Supp Data/Free lists and pile sorts/Free list illness data (combined).pdf]

*title
"Illnesses Republic of the Marshall Islands"

*respondent data

ID	gender	age	location
1	M	28	U
2	F	26	U
3	F	27	U
4	F	22	U
5	F	35	U
6	F	N/A	U
7	F	18	U
8	F	37	U
9	F	26	U
10	M	26	U
11	F	33	U
12	F	27	U
13	F	37	U
14	M	38	U
15	M	24	U
16	M	32	U
17	M	25	U
18	M	32	U
19	F	22	U
20	F	49	U
21	F	49	U
22	F	24	U
23	F	42	U
24	F	27	U
25	F	36	U
26	M	21	U
27	F	20	U
28	F	33	U
29	F	28	U
30	F	38	U
31	F	28	U
32	F	28	U
33	F	23	U
34	F	18	U
35	F	36	U
36	M	21	U
37	F	49	U
38	M	30	U
39	M	47	U

40	F	34	U
41	F	25	U
42	F	41	U
43	F	30	U
44	F	29	U
45	F	20	U
46	F	38	U
47	M	47	R
48	M	21	R
49	M	32	R
50	M	48	R
51	F	21	R
52	F	25	R
53	F	25	R
54	F	20	R
55	F	28	R
56	F	36	R
57	F	36	R
58	F	24	R
59	M	27	R
60	F	29	R
61	F	30	R
62	F	30	R
63	F	28	R
64	F	28	R
65	F	22	R
66	M	35	R
67	M	28	R
68	M	35	R
69	M	38	R
70	F	44	R
71	M	37	R
72	M	37	R
73	M	49	R
74	F	25	R
75	F	29	R
76	F	48	R
77	F	30	R
78	F	51	R
79	F	31	R
80	F	25	R
81	F	36	R
82	F	35	R
83	M	38	R

84	M	55	R
85	F	64	R
86	M	39	R

*freelists

respondent 1
Bwil
Metak lojeen
Metak boran
Bidrodro
Kajjinok
Ben lojeen
Bok bok
Uwor
Metak di
Kabojak botoktok

respondent 2
Bwil
Bok bok

respondent 3
Bwil
Bok bok
Yillow
Bidrodro

respondent 4
Bok bok
Uwor
Bwil
Pilo
Bidrodro

respondent 5
Bwil
TB
Tonal
Kor kori
Bidrodro
Yillow

respondent 6

Bok bok
Bwil
Bidrodro
Kor kori

respondent 7
Bwil
Bidrodro
Bok bok
Addeboulul
Uwor

respondent 8
Bwil
TB
Bok bok
Bidrodro

respondent 9
Bwil
Bok bok
Kajjinok
Bok
Metak lojeen
Bwil

respondent 10
Bok bok
Bwil
Jabure on
Kor kori
Uwor
Pilo
Bidrodro

respondent 11
Bidrodro
Bok bok
Metak lojeen
Bwil
Addeboulul

respondent 12
Pilo

Bok bok
Bwil
Bidrodro
Kor kori
Karko
Kor kori

respondent 13
Bwil
Bok bok
Bidrodro
addeboulul

respondent 14
Nimonnia
Bwil

respondent 15
Bwil
Nimonnia
Bok bok
Emmoj
Bidrodro
Kijennam
Kor kori
Kor kori
Bok
Ben lojeen

respondent 16
Bwil
Bwil
Uwor
Bok bok
Nimonnia
Kijennam
Ben lojeen
Emmoj

respondent 17
Emmoj
Metak lojeen
Bok bok

respondent 18
addeboulul
Bidrodro
Metak lojeen
Bwil
Bok bok

respondent 19
Bwil
Bidrodro
Bok bok

respondent 20
Bok bok
Kajjinok

respondent 21
Bok bok
Kajjinok
Bidrodro

respondent 22
Bok bok
Bwil
Metak lojeen
Bidrodro
Ben lojeen

respondent 23
Bwil
Molanlon
Pilo
Bidrodro

respondent 24
Bidrodro
Bwil
Pilo
Molanlon

respondent 25
Bwil
Bok bok
Pilo

Bidrodro

respondent 26
Bok bok
Bwil

respondent 27
Bwil
Bok Bok

respondent 28
Bok bok
Bwil

respondent 29
Bwil
Bok bok
Kajjinok
Bidrodro

respondent 30
Bwil
Bidrodro
Pilo

respondent 31
Ebbonejnej
Bok bok
Bwil
Kajjinok
Bidrodro
Metak lojeen
Pilo
Molanlon
Metak boran

respondent 32
Bidrodro
Bwil
Metak lojeen
metak boran
pilo

respondent 33

Bwil
Bidrodro
Molanlon
Pilo
Uwor
Bok
Bwil

respondent 34
Bwil
Bok bok
Kajjinok
Pilo

respondent 35
Bok bok
Bwil
Kajjinok

respondent 36
Bok bok
Bwil
Bidrodro

respondent 37
Bwil
Bok bok
Kor kori

respondent 38
Bwil
Bok bok
Bidrodro

respondent 39
Bwil
Bok bok
Bidrodro
Pilo
Molanlon
Wot
Kor kori

respondent 40
Bwil
Bok
Pilo
Bidrodro
Bok bok
Molanlon

respondent 41
Bwil
Metak boran
Bidrodro
Bok bok
Molanlon
Pilo
Kor kori
Jeen

respondent 42
Bok bok
Bidrodro
Bwil
Pilo
Uwor
Kor kori
Ebboj lojeen
Metak di
Jabwe botoktok
Bwil
Kor kori

respondent 43
Kajjinok
Bwil
Jeen
TB

respondent 44
Bok bok
Bwil
Jabure on

respondent 45
Bok bok

Kajjinok
Bwil
Bidrodro
Emmoj
Kor kori

respondent 46
Bidrodro
Metak boran
Bwil
Uwor
Ebboj lojeen
Kor kori

respondent 47
bwil
kor kori

respondent 48
bok bok
bwil
ebboj lojeen

respondent 49
bwil
bok bok
bidrodro
molanlon

respondent 50
bwil
bok bok
molanlon

respondent 51
bok bok
bwil
kor kori
bidrodro
kajjinok
uwor

respondent 52
bok bok

bwil
kor kori

respondent 53
bidrodro
bwil
bok bok
uwor
kijennam

respondent 54
bwil
bidrodro
bok bok
kajjinok
uwor
bok

respondent 55
bwil
bok bok
kajjinok
molanlon
pilo
bidrodro
kor kori
pilo in bon

respondent 56
bwil
metak boran
molanlon
bok bok
bidrodro
kor kori
jabure on
uwor
nimonnia
kajjinok
ebboj lojeen
wot
mejen wotlok
megatoto

respondent 57
bok bok
bwil
uwor
ebboj lojeen

respondent 58
bwil
bok bok
bok
molanlon
bidrodro
jabure on
wot

respondent 59
bwil
bidrodro

respondent 60
bwil
metak boran
pilo
bidrodro
kor kori
pilo in bon
jabure on
lepa
TB
bok

respondent 61
molanlon
bwil
uwor
bok bok
abnono anbwinin
molanlon
kor kori
bidrodro
kikilok
jabure on

respondent 62

bwil
bok bok
pilo
bidrodro

respondent 63
wot
ebboj lojeen
kor kori
bwil
uwor

respondent 64
uwor
bwil
bok bok

respondent 65
bwil
bok bok
bidrodro
kor kori
pilo
bok
uwor
worlok
metak boran
molanlon

respondent 66
bwil
bok

respondent 67
bwil
bok bok

respondent 68
bok bok
pilo
ebboj lojeen

respondent 69
bwil

bok bok
molanlon
uwor
bidrodro

respondent 70
bwil
bok bok
bidrodro
metak boran
metal ni

respondent 71
bwil
bidrodro
pilo
bok bok
molanlon
metak boran

respondent 72
bwil
bidrodro

respondent 73
bwil
bidrodro

respondent 74
bwil
uwor
bok bok
wot
kor kori

respondent 75
bwil
bok bok
wot
kor kori
bidrodro
molanlon
pilo

respondent 76
bwil
bidrodro
metak boran
bok bok
pilo
molanlon
megatoto
ebboj lojeen
kor kori

respondent 77
bok bok
bidrodro
bwil
molanlon
pilo
ebboj lojeen
kor kori
wot
metak boran

respondent 78
bwil
bok bok
kor kori
metak boran
molanlon
ebboj lojeen
megatoto

respondent 79
bok bok
kor kori
pilo
bidrodro
bwil
wot

respondent 80
bwil
bidrodro
molanlon

respondent 81
bwil
bok bok
metak boran
bidrodro
pilo in bon
jabure on
mej raket anbwinin

respondent 82
bok bok
bon botin
bwil
bidrodro
kajjinok

respondent 83
bok bok
bwil
bidrodro
kor kori

respondent 84
bok bok
bwil
bidrodro

respondent 85
bwil
bok bok

respondent 86
bwil
bok bok
bidrodro
metak boran
ebboj lojeen
molanlon
pilo
