# Supplementary material for: Conceptualizing multi-level determinants of infant and young child nutrition in the Republic of Marshall Islands–a socio-ecological perspective
Source: PLOS Glob Public Health. 2022 Dec 19;2(12):e0001343. doi: 10.1371/journal.pgph.0001343 (PMC10022247; doi:10.1371/journal.pgph.0001343)
Supplement: S1 Data — (ZIP) [file pgph.0001343.s001.zip › RMI Supp Data/Free lists and pile sorts/Pile sort_food data (rural).pdf]

*Title
RMI Rural Q5 Affordability of Foods

*Item Data

ID	Item	Label
1	Rice	Rice
2	Fish	Fish
3	Breadfruit	Breadfruit
4	Canned_meat	Canned_meat
5	Bread	Bread
6	Banana	Banana
7	Water	Water
8	Papaya	Papaya
9	Coconut	Coconut
10	Pandanus	Pandanus
11	Chicken	Chicken
12	Ramen	Ramen
13	Sweet_drinks	Sweet_drinks
14	Breastmilk	Breastmilk
15	Chips	Chips
16	Egg	Egg
17	Candy	Candy

*Respondent Data

ID	gender	age	location
42	F	34	R
43	F	20	R
44	F	28	R
45	F	30	R
46	F	30	R
47	M	38	R
48	F	30	R
49	F	20	R
50	F	30	R
51	F	23	R
52	M	36	R
53	M	28	R
54	M	28	R
55	M	32	R
56	M	27	R
57	M	31	R
58	M	29	R
59	F	31	R
60	M	27	R

61	M	29	R
62	M	20	R
63	F	37	R
64	F	26	R
65	F	25	R
66	F	24	R
67	F	69	R
68	M	31	R
69	F	32	R
70	F	25	R
71	M	35	R
72	M	28	R
73	M	46	R
74	M	46	R
75	M	36	R
76	F	20	R
77	F	41	R
78	F	23	R
79	F	30	R
80	F	43	R
81	F	28	R

*Pilesorts
Respondent 42
Pile 1: 2 3 6 7 8 9 10 14
Pile 2: 13 15 16 17
Pile 3: 1 4 5 11 12

Respondent 43
Pile 1: 2 3 6 7 8 9 10 11 14
Pile 2: 12 13 15 16 17
Pile 3: 1 4 5

Respondent 44
Pile 1: 8 9 3 10 2 14 6 7
Pile 2: 17 16 15 12 13
Pile 3: 1 11 4 5

Respondent 45
Pile 1: 11 9 2 8 3 6 14 7 10
Pile 2: 17 15 12 16
Pile 3: 1 13 4 5

Respondent 46

Pile 1: 6 8 3 10 2 14
Pile 2: 15 16 9 7 4
Pile 3: 1 11 17 5 13 12

Respondent 47
Pile 1: 10 8 14 2 6 3 9 7
Pile 2: 4 15 13 12 17
Pile 3: 11 5 16 1

Respondent 48
Pile 1: 9 14 10 3 8 6 2
Pile 2: 11 5 1 7 12
Pile 3: 17 15 16 13 4

Respondent 49
Pile 1: 10 14 8 2 9 6 3 11 7
Pile 2: 16 17 15
Pile 3: 1 4 13 12 5

Respondent 50
Pile 1: 6 10 8 2 7 9 11 3 14
Pile 2: 15 17 13
Pile 3: 16 1 5 4 12 16

Respondent 51:
Pile 1: 6 9 8 3 16 11 14 2 10
Pile 2: 17 4 15 7 12
Pile 3: 1 13 5

Respondent 52
Pile 1: 2 11 16 12 1 5 10 3 6 8
Pile 2: 7 9
Pile 3: 13 17 4 14 15

Respondent 53
Pile 1: 10 3 6 7 2 11 9 8 14
Pile 2: 13 17 15 12 1 5 4 16

Respondent 54
Pile 1: 16 8 2 10 3 14 6 7 9
Pile 2: 17 13 15 12 4
Pile 3: 1 5 11

Respondent 55

Pile 1: 1 2 4 11 14 9 7
Pile 2: 17 3 12 5 13
Pile 3: 6 8 10 15 16

Respondent 56
Pile 1: 2 11 9 6 3 8 10
Pile 2: 15 16 1 7
Pile 3: 12 5 4 14 13 17

Respondent 57
Pile 1: 6 10 3 8 9 7 6 16 14
Pile 2: 12 1 4 5 11
Pile 3: 13 15 17

Respondent 58
Pile 1: 16 14 11 7 9 2 3 6 8 10
Pile 2: 15 13 5 1
Pile 3: 17 4 12

Respondent 59
Pile 1: 10 9 7 3 2 8 6
Pile 2: 13 11 1 5 4 14
Pile 3: 16 17 15 12

Respondent 60
Pile 1: 3 10 9 8 14 7 6
Pile 2: 5 2 11 15 17
Pile 3: 16 1 12 4 13

Respondent 61
Pile 1: 4 8 2 10 3 6 7 9 14
Pile 2: 15 16 17
Pile 3: 12 5 4 1 13

Respondent 62
Pile 1: 5 7 11 9 2 8 10 3 6 14
Pile 2: 12 17 15
Pile 3: 1 13 4 16

Respondent 63
Pile 1: 16 10 8 3 9 14 6 11 7 2
Pile 2: 12 17 15 13
Pile 3: 1 4 5

Respondent 64
Pile 1: 17 15 12 11 16 6 3 8 7 9 14 10 2
Pile 2: 13
Pile 3: 1 4 5

Respondent 65
Pile 1: 6 8 2 3 9 10 7 14 17 15 16
Pile 2: 13
Pile 3: 4 12 5 11 1

Respondent 66
Pile 1: 7 11 3 14 9 2 8 6 16 10
Pile 2: 17 13 15
Pile 3: 1 12 14 5

Respondent 67
Pile 1: 3 10 16 6 9 7 14 2 8
Pile 2: 4 17 13 15
Pile 3: 11 12 1 5

Respondent 68
Pile 1: 10 8 3 14 7 11 2 6 9
Pile 2: 5 16 12 4 1
Pile 3: 13 17 15

Respondent 69
Pile 1: 14 9 11 3 6 7 8 2 10
Pile 2: 15 17 13 16 12
Pile 3: 1 4 5

Respondent 70
Pile 1: 6 8 3 10 11 2 14
Pile 2: 16 5 17 1 15 14 12
Pile 3: 9 13 7

Respondent 71
Pile 1: 2 7 11 8 14 1 3 6 4 9 10
Pile 2: 5 13 12 15 16 17

Respondent 72
Pile 1: 7 14 2 11 3
Pile 2: 8 9 6 10
Pile 3: 1 17 13 15 12 4 5 16

Respondent 73
Pile 1: 14 2 11 3 8 9 6 10 16 7
Pile 2: 5 4 1 12 15 13 17

Respondent 74
Pile 1: 11 14 7 9 2 3 6 8 10
Pile 2: 5
Pile 3: 16 15 12 4 17 1

Respondent 75
Pile 1: 16 14 11 7 8 6 3 10 2 9 5 1
Pile 2: 4 12
Pile 3: 15 13 17

Respondent 76
Pile 1: 14 8 3 7 11 10 6 2 9
Pile 2: 15 17 12 13 16
Pile 3: 5 4 1

Respondent 77
Pile 1: 10 6 16 8 9 3 7 2 11 14
Pile 2: 15 12 17
Pile 3: 4 5 13 1

Respondent 78
Pile 1: 7 16 3 2 11 6 8 14 10 9
Pile 2: 12 13 4 17 15
Pile 3: 5 1

Respondent 79
Pile 1: 3 6 8 10 2 9 14 7 11
Pile 2: 17 13 15 16
Pile 3: 5 1 4 12

Respondent 80
Pile 1: 7 14 9 11 8 6 10 3 2
Pile 2: 16 15 17 13
Pile 3: 1 4 5 12

Respondent 81
Pile 1: 2 11 3 10 6 8 7 14 19 15 17
Pile 2: 5 13 16
Pile 3: 1 4 12
