# Supplementary material for: Conceptualizing multi-level determinants of infant and young child nutrition in the Republic of Marshall Islands–a socio-ecological perspective
Source: PLOS Glob Public Health. 2022 Dec 19;2(12):e0001343. doi: 10.1371/journal.pgph.0001343 (PMC10022247; doi:10.1371/journal.pgph.0001343)
Supplement: S1 Data — (ZIP) [file pgph.0001343.s001.zip › RMI Supp Data/Free lists and pile sorts/Pile sort_food data (urban).pdf]

*Title

RMI Urban Q5 Affordability of Foods

*Item Data

ID	Item	Label
1	Rice	Rice
2	Chicken	Chicken
3	Canned_meat	Canned_meat
4	Fish	Fish
5	Baby_food	Baby_food
6	Hot_dog	Hot_dog
7	Water	Water
8	Bread	Bread
9	Ramen	Ramen
10	Pancake	Pancake
11	Banana	Banana
12	Juice	Juice
13	Chips	Chips
14	Breastmilk	Breastmilk
15	Candy	Candy
16	Cabbage	Cabbage
17	Green_beans	Green_beans

*Respondent Data

ID	gender	age	location
1	F	21	U
2	F	21	U
3	F	32	U
4	M	20	U

5	F	20	U
6	F	19	U
7	F	21	U
8	F	20	U
9	F	35	U
10	F	22	U
11	F	29	U
12	F	36	U
13	F	28	U
14	F	28	U
15	F	36	U
16	F	21	U
17	M	21	U
18	F	21	U
19	F	37	U
20	F	37	U
21	F	29	U
22	M	35	U
23	F	43	U
24	F	26	U
25	M	38	U
26	M	47	U
27	F	35	U
28	F	33	U
29	F	43	U
30	M	25	U
31	M	42	U
32	M	30	U
33	F	30	U

34	F	25	U
35	F	28	U
36	F	23	U
37	F	18	U
38	F	20	U
39	F	18	U
40	F	23	U
41	F	22	U

*Pilesorts

Respondent 1

Pile 1: 7 13 15 12 5 9 14

Pile 2: 6 11 8 10

Pile 3: 3 1 2 4 17 16

Respondent 2

Pile 1: 14 6 13 15 10 8 7

Pile 2: 9 12 3 5 4

Pile 3: 2 17 16 11 1

Respondent 3

Pile 1: 13 6 9 14 10

Pile 2: 1 2 5 11 4

Pile 3: 3 7 8

Respondent 4

Pile 1: 7 5 9 6 13

Pile 2: 4 12 11 8 10

Pile 3: 1 2 3

Respondent 5

Pile 1: 6 3 16 17 1 2 8

Pile 2: 12 7 15 13 10 9 5

Pile 3: 4 11 14

Respondent 6

Pile 1: 13 15 10 14

Pile 2: 6 3 16 17 4 11 5 7 12 9

Pile 3: 2 1 8

Respondent 7

Pile 1: 6 15 13 8 9 12 7 14

Pile 2: 1 2 3 4 10

Pile 3: 11 5 16 17

Respondent 8

Pile 1: 6 12 9 7 13 15 14

Pile 2: 5 11 8 3 5

Pile 3: 17 2 1 10 16 4

Respondent 9

Pile 1: 4 7 11 14

Pile 2: 1 2 3 5 6 8 9 10 12 13 15

Pile 3: 16 17

Respondent 10

Pile 1: 3 17 1 2 16

Pile 2: 13 15 8 12 5 7 9 6

Pile 3: 10 4 11 14

Respondent 11

Pile 1: 14 7 11 4

Pile 2: 15 13 6 9

Pile 3: 8 5 12 10 17 16 2 1 3

Respondent 12

Pile 1: 14 15 13 9 6

Pile 2: 7 10 5 11 12

Pile 3: 3 17 1 2 8 16 4

Respondent 13

Pile 1: 12 15 13 9 7 14

Pile 2: 11 5 10 8 6

Pile 3: 17 3 16 4 1 2

Respondent 14

Pile 1: 9 12 13 15 8 7 14 11

Pile 2: 6 4 3 10 5

Pile 3: 16 2 17 1

Respondent 15

Pile 1: 8 6 12 13 15 11 7 9 4 10 14

Pile 2: 17 16 5

Pile 3: 2 3 1

Respondent 16

Pile 1: 6 7 16 9 13 5

Pile 2: 10 11 14 4

Pile 3: 8 3 1 2

Respondent 17

Pile 1: 14 13 6 11 7 9 12 4

Pile 2: 8 10 3 5

Pile 3: 1 2

Respondent 18

Pile 1: 14 7 9 13 12 4

Pile 2: 10 11 8 3

Pile 3: 2 6 5 1

Respondent 19

Pile 1: 13 14 15

Pile 2: 4 11 7 6 9 12

Pile 3: 17 16 5 8 2 3 1 10

Respondent 20

Pile 1: 13 7 12 15 14

Pile 2: 6 9 17 16 11

Pile 3: 5 4 2 1 3 8 10

Respondent 21

Pile 1: 15 5 13 2 6 9 14

Pile 2: 12 11 7 10 4 1 8

Pile 3: 16 17 3

Respondent 22

Pile 1: 14 10 11 6 9 13 2 7 4

Pile 2: 5 1 12 3

Pile 3: 2 16 17

Respondent 23

Pile 1: 6 7 9 13 14 15

Pile 2: 1 2 3 4 5 8 10 11 12

Pile 3: 16 17

Respondent 24

Pile 1: 1 14 7 9 8

Pile 2: 2 3 6 10

Pile 3: 16 17

Respondent 25

Pile 1: 14 17 9 13 15 6

Pile 2: 11 5 12 10 1

Pile 3: 16

Respondent 26

Pile 1: 14 7 15 13 10 8

Pile 2: 9 6 2 3 1

Pile 3: 12 4 11 5 16 17

Respondent 27

Pile 1: 6 10 3 7 12 4 14 13

Pile 2: 1 5 8 17 16 11 15 9

Pile 3: 2

Respondent 28

Pile 1: 8 12 2 14 11 16 17

Pile 2: 3 5 1

Pile 3: 9 15 13 6 7 10 4

Respondent 29

Pile 1: 5 12 15 13 7 4 14 11 6

Pile 2: 8 10 3 9 1 2

Pile 3: 16 17

Respondent 30

Pile 1: 7 11 4 16 17 15 13

Pile 2: 14 6 3 5 9 12 10 8

Pile 3: 1 2

Respondent 31

Pile 1: 14 13 15 9

Pile 2: 11 10 12 7 17 5 16 3 6

Pile 3: 1 2 8 4

Respondent 32

Pile 1: 14 15 13 7

Pile 2: 6 17 12 11 9 8 10

Pile 3: 4 2 16 5 1 3

Respondent 33

Pile 1: 10 9 7 14

Pile 2: 8 4 5 13 15 6 3

Pile 3: 11 17 2 1 16 12

Respondent 34

Pile 1: 13 6 7 12 9 15 14

Pile 2: 10 5 2 3 8 1 4

Pile 3: 16 17 11

Respondent 35

Pile 1: 7 14 11

Pile 2: 15 9 13 12 10 8 6

Pile 3: 17 5 16 2 3 4 1

Respondent 36

Pile 1: 12 6 9 13 15 14 7 11

Pile 2: 3 1 10 2 8

Pile 3: 16 5 17 4

Respondent 37

Pile 1: 11 4 14

Pile 2: 9 15 7 12 13 6 8 10

Pile 3: 17 1 2 16 3 5

Respondent 38

Pile 1: 11 4 5 16 14 10 13 15 3 12 8

Pile 2: 7 9 17 6

Pile 3: 1 2

Respondent 39

Pile 1: 14

Pile 2: 13 9 8 15 6 1 7 10 12

Pile 3: 2 3 16 5 17 11 4

Respondent 40

Pile 1: 8 9 13 15 6 14 7

Pile 2: 1 12 10 3 11 5

Pile 3: 16 17 4 2

Respondent 41

Pile 1: 7 9 14 8 13 6 10

Pile 2: 5 2 3 1 11 15

Pile 3: 12 16 4 17
