# Supplementary material for: Conceptualizing multi-level determinants of infant and young child nutrition in the Republic of Marshall Islands–a socio-ecological perspective
Source: PLOS Glob Public Health. 2022 Dec 19;2(12):e0001343. doi: 10.1371/journal.pgph.0001343 (PMC10022247; doi:10.1371/journal.pgph.0001343)
Supplement: S1 Data — (ZIP) [file pgph.0001343.s001.zip › RMI Supp Data/Interviews data/157R_IDI_CL_Arno_Sep 17_Balton.docx]

**I: well, is it okay if proceed on? During our talk, it would be clear for our consultants to understand what we're saying. The question, besides yes and good, from your own understanding. You give things that happens in these places because they can hear and picture things that are affecting these places. A support from the other places can help this community. It’sTo begin with our questions, can you explain your role as a leader in this community?**

R: I am counsel man in this community. me and three others

**I: three for all of this Arno or ?**

R: in this community.

**I: this Ine right?**

R: and this Ajeltorok in.

**I: Ajelto?**

R: I am also a church leader,as a deacon.

**I: perfect, perfect. can you tell me about the community, who lives in it, how many people in these villages?**

R: it’s about many, 300 to 400 about these much. In all these three villages, children to adults.

**I: 300 to 400, are there any foreigners living in this community?**

R: yes, there’s world teach.

**I: world teach, the teachers right?**

R: I think those are the people that usually come and stay in this community. The teacher’s from world teach. Except when there’s visitors, they stay a bit and return.

**I: as one of church leaders, what do you see, are there any support the church does to help the communities, the community. Are they any support to help this community with?**

R: well yes, there are supports we do. Especially delivering the good word. And the works of ecclesia, work together and.

**I: it brings these communities together? If we were to say, like can you tell things that are good from what you understand, the good of this community you worked on. Things that set aside this place from other places. It’s like, other places for example. Other places on Majuro don’t work together. They always compete, this place do they compete? Or do they help? Work together with one heart.**

R: they always work together to help each other with works of community.

**I: within sorry,**

R: the word competing each other is not important because

**I: they’re same.**

R: it’s Marshallese way of life.

**I: it’s really good. Are there any difficulties you see shows in this community you want to change, you want to fix it, what difficulties you want to change?**

R: fact about these days, it’s like you’re taking care of these lands. Lands that are vast and far from each other. May need transportation and these things for work.

**I: are there any difficulties with food?**

R: yes, there are times that is much looking for food in this way because these people from these places don’t want to be in a hurry to make farming places like that. Unlike those people that, foreigners do like to grow food.

**I: growing.**

R: they do grow, we don’t really, maybe these are the things we need take action about.

**I: what you think if other countries would’ve help with agriculture tools. And can you think this may increase in this community?**

R: correct, it would be and one of the thing is there is no tools to work on our rocky land.

**I: the thing that hold all these things there’s no tools? Yes. If we to talk about health, can you explain how children suffer from any illnesses in this community?**

R: yes, sometimes they cough.

**I: cough right?**

R: asthma and.

**I: asthma.**

R: children’s illnesses.

**I: like?**

R: they may, they may go hungry. Sometimes.

**I: there’s no food right?**

R: there’s no food. It’s late

**I: food is late. Do these most often show? Do they frequently show? Like for coughing?**

R: well yes. Coughing and fever and those things. I’d say sometimes, the doctors are lazy. They go to Majuro and return, they don’t bring medicines.

**I: sometimes there’s no medicines? Do you think is it enough for the hospital here to handle people here? Or it’s better if there’s more?**

R: when I look at the places where there’s schools in

**I: yes.**

R: there needs to a doctor. And when there’s an emergency, as I was saying were far from each other. Not enough transportation. Unlike there’s hospitals in these villages. There’s an emergency, bring inside the hospital.

**I: what do you think if the school’s at least have their own doctor? Would it be helpful? Like he/she a nurse or teacher too.**

R: well yes.

**I: would this be helpful? It would be helpful right?**

R: yes. It would be helpful when fainted outside the classroom, just come and help. May reach the doctors but, those are the doctors.

**I: yes, it may prevent the illness to and there needs to be lots hospital right? And people to prevent these illnesses. Perfect. Can you tell how the people in the community find a way to treat the children like for example, if it would be children? Children that are ill, who the first to bring to? Do they usually bring them traditional healers or bring to the hospital right? Or bring them to Majuro? Where do?**

R: Marshall, Marshall (traditional healers). If it’s suitable for foreign treatment well the doctors.

**I: depends on the illness right? If it’s illness for fever? If it was for a mama or baba, see the child really have high temperature. Who is the first person see first? A traditional healer or doctor?**

R: a doctor.

**I: a doctor right? Can you tell what kind of illnesses that come from foods and affecting children in this, are there like for example? If it was toothache, toothache comes when children always eating candies and things. Beside toothache, are there any illnesses affecting children because of their foods? Or it may, it may. I may change the question. Are there any foods that children need to stop eating? That may not keep them healthy? It may what kind of foods that can make children sick? And what kind of food is that?**

R: it may kind of food that it’s preserved for long. And doesn’t keep the food safe where there were flies on it.

**I: yes….. What kind of food does usually affecting children because of their foods?**

R: diarrhea and

**I: diarrhea.**

R: careless of taking care of their foods and (12:45) those things. They just come take the food.

**I: are there any local food that is healthy for children? Are there any food in this community? That is good for the children.**

R: like for papaya, Pandanus.

**I: papaya, Pandanus.**

R: drink coconut.

**I: drink coconut… meats? What kind of meats?**

R: fish meat and

**I: fish meat.**

R: (13:38) healthy with it.

**I: yes. A lot, a lot for being healthy with it. And the growing foods. Banana, Pandanus, Papaya. All those things that are growing foods…. Are there any difficulties to have food in this community? Like is there a time for hunger, do people sometimes don’t have any food?**

R: yes.

**I: what are the things that makes it not enough food for a family?**

R: sometimes there is low income, and looking for food takes too long but, they’re hungry.

**I: what do you think what would help with this thing? Are there, like work will help for money right? Are there any work here that is small, that can be huge to help others that are looking for job? What kind of jobs that this Island needs open up jobs by it?**

R: well we need to, bring those projects to build to have many employees so we don’t go hunger.

**I: like employee that went to school right? Teacher? Or..**

R: or build places and bring the tourists to

**I: about tourism right? Bring, bring, yes, yes. Fact, when there’s more tourist here there will be a lot of workers. It may not only make income for Marshall, but also these places. When you build, yes.**

R: whose keys are those? Who?

**I: do people usually grow foods outside their houses?**

R: yes.

**I: what kind of growing near the people’s houses?**

R: banana and, Pandanus, papaya, pumpkin, these things.

**I: yes. And these are things people do eat from right? Are there any of these foods get sold off when there’s any leftovers? They sell? Like what kind of food they sell?**

R: pumpkin, banana

**I: so the profits that they took from these things, what they usually use it for?**

R: for their needs and food.

**I: for others, profits for other food? And their needs? Great… are there any difficulties, for someone that would like to make an owned farm? Are there any difficulties?**

R: it would be tools.

**I: just the tools right? That’s about it? And if there is, it will be easy to make it? The only difficulties is that there’s no tools.**

R: there’s no tools.

**I: yes….. the foods that have like seasons, like season for breadfruit. There’s season for Pandanus, these things that would be for each food. Is it affecting for having this in these communities? Do people get hungry during the times when.**

R: sometimes we Kabwiro (mashing the breadfruit when riped), (18:33) when breadfruits are all out, we eat Bwiro (mashed breadfruit)

**I: perfect, perfect.**

R: fishing and eat it.

**I: you’d say, doesn’t matter seasonal food and that, there’s always food? Right? From bwiro and those things… is there a time where there’s no food here on Arno?**

R: yes.

**I: when was the last time? Or like every year there are…**

R: sometimes, sometimes there is a habit. Sometimes this Island is a habit.

**I: is a habit?**

R: there are (19:37) because this Island labod (name of an ancient place) half is full of breadfruit and stuffs and the other half is not. And sometimes this place is on hunger, and sometimes this place is not on hunger.

**I: and what do you do when there’s hunger here, what do you do?**

R: we do Marshallese work. Sail and bring from far.

**I: hunt for food right?**

R: hunt for food. Iu (coconut meat)

**I: search for food from ocean side. Where is the fastest way to look for food at?**

R: Islands, small Islands from this place.

**I: the small Islands on this Island right? But, what kind of animals that are usually in this community?**

R: well, pigs and chickens those things.

**I: do these things stay inside a fence?**

R: others are in fence, others don’t.

**I: are there any difference between animals in a fence and animals...**

R: well yes. There’s a time it’s bad for them to stay. They get skinny

**I: inside a fence right?**

R: sometimes well, animals usually in a fence, others don’t.

**I: I’d say it’s easier to let them free right? Because they look for food everywhere.**

R: well, they look for food everywhere, the bad thing is they destroy farming places.

**I: they do usually destroy the farming places right?**

R: when we set them free they eat the potatoes and pumpkins.

**I… Are there any difficulties for making a fence? Do you have any difficulties? Unlike Majuro for example, Majuro you cannot raise a pig unless you have a fence. And you will not have fence if you don’t have a waste sewer. Is there a law here on Island?**

R: well theres only about putting pig

**I: putting pig inside a fence?**

R: there’s no fence.

**I: but there’s no fence?**

R: there’s no fence.

R: they haven’t make them.

**I: there’s no things to make a fence?**

R: this is a thing that is difficult, as for this community. When I fence a pig, I (22:32), I usually let them free. Why I am fencing, but you guys don’t fence?

**I: When you asked this question, what answer do you usually they say?**

R: they usually say, how it can be they don’t have fence they might die.

**I: is it difficult for everyone to have tools to build fences?**

R: well yes.

**I: is it also a difficult right? Well, if everyone has rights. If there was a program for building a fence. Everyone would have fence? The last question about food, who in the family decide the food for the family?**

R: well, let’s say. If it was this for this fire. The elders in this house would say cook pumpkin. With a flour, the cooks come and cook and we eat.

**I: but for the children’s foods? The babies?**

R: babies foods, well the women look into their recipes and if it’s good for (23:52)

**I: well, thank you because when we answer well talk right? You answer by talking and it’s good, when you answer like this it helps our research because, like you don’t say yes or no but you explain why other things and its good you’re showing your answers. Now we’ll proceed on to water and questions regarding about water. And hygiene. Can you explain how mostly to look for waters that are for this community? Where do these water come from?**

R: well, it’s like, the water catchments that were filling.

**I: pontoon?**

R: pontoon because (24:50) EPA (environmental protection authority) made these water to.

**I: EPA? EPA was, are there any difficulties to look for water? Like for pontoon and those things?**

R: well, those that are given.

**I: are there any prices that was given for?**

R: none

**I: wow, well that’s great.**

R: that water catchment, during when, do you the man Lobeba? Lobeba? Ken? Child of Teruwo, the manager for EPA.

**I: yes.**

R: well, first time building of those water catchments him.

**I: and they were divided amongst everyone in the community?**

R: they divided them to everyone here on Island.

**I: wow**

R: I was a councilman those days, and these are things I usually do.

**I: and for these water catchments that are plastic that are big.**

R: those pontoon that are plastics that time Jejwadik (the Senator)

**I: yes.**

R: well, those are the times those things came. And divide them for these places.

**I: also for free? Is there anyone that don’t have these?**

R: there was some.

**I: there’s still some?**

R: they said there’s only one round.

**I: so everyone could have, there’s still others waiting for their turn? That’s very good. And now for the waters, you use the water from the pontoon for shower and drinking or?**

R: drinking and showering too

**I: both right? Do the water filters come too? Do they teach how to clean the waters?**

R: there came others, a group that came to teach how to clean. Chlorination, they come put, there were things for testing.

**I: they do give the testing things right?**

R: when it’s no good, they throw it away

**I: do a cleaning again? It’s very interesting… when it comes for washing hands, when do the people in this community you are working, when do they frequently wash their hands?**

R: they usually wash their hands before having a meal. Wash face and these things. And when they’re working, they usually wash their hands before.

**I: are there, are there any difference between washing hands with water and washing hands with water and soap? Are there any difference between them?**

R: well yes, when we soap our hands it’s clean rather than washing it carelessly.

**I: it’s cleaner right?**

R: who was it?

R: la baaj.

R: hello?

**I: in the schools and other places, the children or adults usually when washing their hands they may not use soap. What makes it for them to forget the soap, or they?**

R: it may emergency.

**I: emergency right? In a hurry? Or there may no soap right?**

R: the soap may far, or there’s no money for it.

**I: good, thank you, plus it’s you both been knowing these things. (Laughter)**

R: yes, sometimes it’s hard for us to do things because

**I: yes, yes, we need help… but for this matolen(name of village), what kind of toilet for each households? Do every households have toilet?**

R: there’s none

R: there’s none. If there’s toilets in these places, there are few.

**I: there are few right?**

R: there’s no every places, matolen may have two.

**I: do you think this thing is important to?**

R: yes

R: we need it very important.

R: it is very important. Toilets because now, when there’s world teach and these things. They don’t take because, there’s no toilets. Then, when there’s world teach well (30:21)

**I: well, yes. You think each households need a toilet is important right? Where do you think it may easy, who do you think it may help these places?**

R: well, it would be the government. (31:01) your projects, who knows maybe your project might. (Laughing)

**I: the reason why we’re here to see what the people needs. And yes, enrich the healthy life of a community. Toilet is important, we talked about this, and there are many places especially on Majuro. And there are Islands Ebeye and people defecate on ocean and lagoon side. And what do you think why they defecate on ocean and lagoon side?**

R: they don’t have any toilet.

**I: yes, now. This thing that if each households have toilets. It will eliminate this thing right? And it will help each communities. Because ocean and lagoon side is where children go and play at.**

R: there was a world teach here. That guy is bad, when I went to the lagoon side he’s defecating on the coconut tree. Well, then why do you do this here? Is our house broken (toilet) he said no it’s not. So why? I’m trying to become Marshallese. I said, “ Bakaro (Japanese word for showing expression)” this guy is bad. Now I forgot what to say. What’s the guy’s name?

R: Seru?

R: yes, I mean what’s his name? Is there someone younger than him? Is there someone who is older than you? He used to work with the mayor.

**I: he’s older.**

R: well, he used to sleep in this house, he slept in this house.

**I: he used to come with you two right?**

R: he and Hudson. He said, you go see your child he’s climbing the coconut tree (way of describing he’s defecating.)

**I: so he has seen the older brother of mine.**

R: that guy and, his your uncle, his my cousin.

**I: and sorry that I am, 20 years I’ve been gone. I’ve just arrived. And sorry for I don’t really know any relatives. Someone that is flunk right, they never gave any advice or prepare me.**

R: la Rilang what are you doing boy?

**I: say, Banton did stay in this house.**

R: every weekend they, really alcoholic those guys.

**I: was Hudson right? Those guys do really always follow the mayor. They started from the water house, they started being friends from the water house. What difficulties that you see that prevent people from building a toilet? Do you think the Alap (landlords) would be against people building toilet? Or the tradition would be against it, it’s like the chiefs saying that’s my land don’t make any toilets.**

R: it’s like it’s hard to

**I: it won’t, they won’t right?**

R: the only thing that would hold it, is there’s not enough supplies to make it.

**I: you say materials and that thing, and if there would be a program or grant to help with these things.**

R: well,

**I: nothing, that’s it? It would fix it. It would eliminate all of these problems. For these communities, you know the children play at the lagoon side under the breadfruit tree and defecate. How do they get rid of the stools?**

R: I think they bury it.

**I: bury it right? At lagoon side bury it? And at environment too? … Where do the children in this community play?**

R: at their playing ground, lagoon side and.

**I: lagoon side and?**

R: sometimes ocean side.

**I: are there any animals at the place they’re playing at?**

R: there’s none. Those are the only animal chickens and pigs.

**I: chicken and pig**

R: dog.

**I: chicken, pig, and dog. If, if government or other place may help this community a place for playground. For children to play, what kind of playground you would like to be in this community?**

R: it’s really, those things we see at the schools. That’s place for playing

**I: playgrounds right? That there’s swings, slides, play areas for volleyball and basketball. Things that are in the schools. if these were to have, or if not playing areas for the children. What makes it harder to be cleaned? There are some places, playing areas but they’re not clean. What, what make it hard to clean?**

R: well, we need to keep it clean. Keep it tight for children’s to play. Playing, there’s none, there’s nothing to be showing. Regarding (37:57)

**I: yes. Yes, now everyone needs to take part in cleaning it? For, for these last questions about hygiene. Can explain ways of preventing illnesses? Like, what does, you know the diseases right? What does it really help prevent disease to spread?**

R: when the environment is clean, the community, the area that you live in.

**I: yes. I agree with that. Cleaning can prevent these from yes. Well, we’ll proceed on with this question about responsibilities for those in a community or a family. Can you explain how do this community take care a child throughout the day? It’s like, who is responsible for taking care of the children?**

R: well his/her mother.

**I: his/her mother. How do, what do mother or grandmother. What do they do show being a good mother for the child? What do, show that they’re a good mother?**

R: sometimes, she sings the child. Well, watch over the child making sure he/she doesn’t eat any dirty trashes (on the ground)

**I: she does really look after right? And for the father, what is the job of the father?**

R: the job of the father is, sometimes help the mother. And support with food.

R: that’s it give it.

R: it’s good. Always sing. He/she can’t go hungry. Always sing to the child.

**I: what are the grandparents’ responsibilities? In the community?**

R: well, it is really huge for the community. Sometimes they both, they both might lead the community. Well like these things.

**I: what do the grandparents do to the child?**

R: for?

**I: it’s like, how do the grandparents like what? Can you…can you just, just, in a community? There’s places where there’s no grandparent inside the house, and there are others it’s good. And we usually see that there’s grandparents are separated, there are things that are same just like the other house. Can you tell what the grandparents bring to show other communities that are different from other?**

R: man, what could that be?

**I: words of advice.**

R: words of advice and wisdom. For the community.

**I: also, also help the mother and father with taking care of the child right? When they’re gone, the mother and father. No child left behind. How do you think in these communities, are there any those like people or neighbors that those may live near these houses, do they help the community take care of their children? Are they together for.**

R: well yes. They,

**I: can you explain they,**

R: sometimes when you’re busy from the child, you may startled they bring the child take care the child don’t lose he/she.

**I: they took part in taking care right? Okay. This question, comes as the last question regarding taking information. Can you tell where do the people in this community, take information from like information that, where can they take hug information from? And they trust, and where do they take information about taking care of a child? Nutritious foods for children. Any information that comes for taking care of a child. Where do they take information from?**

R: well, program that are for the doctors.

**I: on radio or when they come?**

R: they come and radios, when it’s radio they listen. When they come, they sit down and see what’s going on.

**I: hospital too?**

R: hospital too.

**I: which place is easy and good?**

R: in hospital right?

**I: hospitals right? It’s easy. Are there any other information you would like to know? Do you have any other questions about the thing I’m with? You want to know and where we didn’t talk about it and you want have information about it.**

R: well about, do those thing boy.

R: bring the je den (stuff for cooking) bring the je den. Bring the je den. Bring the je den.

R: it would be… movement for the program and when do they work?

**I: the thing, the thing that were doing is. The movement that we do is, everything that we take is, we take it to government. Every word we said, it’s been recorded. And it will go forward to nitijela (government leaders) those with the government to see. Especially your leaders, and friends. And these help, as you already know about. Now, it would be easy for you say how is the thing, how the thing that came to the Island? You can bother the leaders about the thing. But with this program it’s for children right? It’s also for the community and the Island. How long would it be? The thing that we’re doing is the stage two of trying to do it. Stage one is to see what Marshall has, stage two this is it. Find ways to face this thing. Stage three is to attack this thing, it’s like work it. Now, work it it’s where it shows. The results of this thing. It may take time, but it will be achieved. How long? Well, it will be from government to government. These things will stay but, those that I am working with comes from united nation where Marshall ask for help. And these studies will go to our government report it. Or they will go straight to where they send us to work with every one of you. And if there are other questions you would need, with us came the chief nurse of the hospital. She’s there at Ine, along with her, the three ladies that came from United Nations. If there are any questions you may get in touch with them. We’ll be staying here on Island, and we’re leaving tomorrow. But, we’ll be back the next following Monday. We be going to the Arno, and stay long for a week. And if there’s any questions you may get in touch with us that time. Beside this thing, thank you. For your time, and thank you for your council’s lady and this community.**

R: there’s handicraft here.

**I: thank you.**

R: it’s a price for coming. Well, congratulations on your work and trip

**I: yes, thank you.**
