# Supplementary material for: Conceptualizing multi-level determinants of infant and young child nutrition in the Republic of Marshall Islands–a socio-ecological perspective
Source: PLOS Glob Public Health. 2022 Dec 19;2(12):e0001343. doi: 10.1371/journal.pgph.0001343 (PMC10022247; doi:10.1371/journal.pgph.0001343)
Supplement: S1 Data — (ZIP) [file pgph.0001343.s001.zip › RMI Supp Data/Interviews data/I01U_IDI_HW_Hospital_Aug 13_Libon.docx]

**Interview code: I01U**

**Interview type and Interviewee: IDI HW**

**Interview Date: AUG13**

**Location: HOSIPTAL**

**Interviewer: Libon**

**Transcriber: Shante**

**I: Do you agree if we do this survey?**

R: Yes

**I: Ok. Thank you for giving me this time to talk to you. These information like I said it will help us learn to make mothers and children and our environment a better and healthy life in our homes. And to start may I ask what kind of work do you do at the Ministry of Health?**

R: I’m a Health assistance…I usually work in the community, at this moment I’m here working at the office of the outer Islands OISCS.

**I: Now what do you usually do when you wake up from morning until night as a worker from the Health center or as a helper?**

R: As of this moment I take all kinds of sickness from the outer Islands and report it to the Director or give out treatments and other than that I also send out medicines to the outer Islands. The thinks they ask it what they need.

**I: Ok. Lets now talk about illness. I’m interested in the kind of sickness that kids usually suffer from. In your community what kind of sickness can you tell kids from two years below usually suffer from?**

R: All of the community that we work on or we live on, the sickness that usually show is diarrhea, cough, Pink eye and fever. These are the kinds of sickness we usually see in our community.

**I: Okay so you mention diarrhea? Can you tell me what makes them have diarrhea?**

R: It may not be… it may not be right for the mother to use personal (2:25) or it may be the… you know kids… they touch the grounds and touch their mouths and they get diarrhea.

**I: Ok. From your own point of view, what are the dangers effects of diarrhea to a child?**

R: warar! (oh gosh!) kids aren’t allowed to get dried because they are so weak, just a little bit of it and they will go down. That’s what they get really sick from… when they have diarrhea.

**I: You mention dry… can you explain what it really meant?**

R: Its like when you poop a lot and there’s little bit of water in their body, and if mothers don’t breastfeed them often or give them water. We urge them to prevent dryness, because its like when they poop its dry and have diarrhea and its dry and it easily makes them weak. And their body needs water… and ahh… because its goes out when they poop.

**I: Okay… So how do we prevent this sickness… Diarrhea?**

R: The biggest one is personal hygiene because we clean our hands…wash our hands first… before we eat and after we use the bathroom… clean our hands…wash our hands are the most important things.

**I: Now what cures our children from this illness? Diarrhea, in this community.?**

R: Rehydration… it’s the biggest issue… we use ORH and other stuffs. Oral Dehydration solution, this is what we often give, other than give them all kinds of drinks that they usually drinks including breastfeed.

**I: Now… Can you explain what type of treatment people in your community seek for their children? For example… do they use traditional healers? Or do they go with doctors or nurses?**

R: These days people look up to doctors and nurses. They can have diabetes or their kids can have diarrhea and they’ll still go to the doctor or nurses.

**I: Ok. Now that you say they usually go to the doctor and nurse… can you tell me why?**

R: From my own understanding…when I look at it there’s no cure for diarrhea in our own local medicines. Other than they use water and salt for Rehydrate…well they often use ORH and other kinds like IV.

**I: OK. That’s good… Can you tell me about any challenges your community faces in seeking treatment for the illness you mentioned before? Like diarrhea, fever…**

R: its good for us that are in the center (Capital city) because we can just take a taxi but on the outer Islands… If the LA assistance runs out of ORS and stuff… when they discuss to the child mother and father about their yellow cards because there’s a (6:04), its no different from ORS… some use it and some don’t really believe in it and they wait for the medicines from the Center (Capitol City).

**I: Ok that was about outer Islands… what about your community?**

R: Well in this community we usually inform them to keep giving water to their children, and (post flu them?), breastfeed them and go to the hospital on the taxi, one of the problems will be taxi fairs and some other ways.

**I: What are the issue you face when you help out your sick patience?**

R: Not understanding…like I said not understanding…like I discuss about their Yellow card and the medicine…there’s no ORS we can use the formula on the yellow card… and they don’t understand and they don’t really follow what we tell them.

**I: Those are the kinds of information I am looking for. Can you describe any illnesses associated with nutrition that effect children in your community?**

R: There’s nutrition in it?

**I: hmm…**

R: The only problem is the children don’t really know the food that has nutrition in it… like the Marshallese diet…you know…its really different then, its like its not really like our nutrition diet… and that they won’t get used to eat nutritious food, but if you force them it will get better.

**I: Now… what kind of food that makes a child body unhealthy and why?**

R: The food that they like is junk foods… those are the food that don’t have nutritious in them and those are the kinds of food kids loves to eat.

**I: Now… you said junk food? Can you really describe what those junk food are?**

R: Chip and those other kinds…ahhh..kids nowadays they mix stuff with ramen plus those sweets…so that’s the only food for the kids… candies and junk food ahhh chips and stuff like that.

**I: Okay… What about foods that will make a child’s body Healthy? and why?**

R: Well our own food here, Marshallese foods that comes from trees and plants…plus the fish and chicken that we have around here.

**I: We talked about being unhealthy. Could you now describe for me a typical day of someone living a healthy lifestyle, from what time they wake up in the morning until when they go to bed?**

R: Explain the kind that lives healthy?... its really hard to explain but you will just look at that person and knows that he/she looks healthy. From the way they move and…. Just the way they move around us.

**I: is there anything else that you see like it’s the person working a lot or???**

R: Active yes. Doing good with his studies… something like that.

**I: Just few more questions about illness and healthy life… Umm. And that occurs women lives. Can you tell me about what you know about women that don’t have much blood in them?**

R: My understanding its like… women like these are obvious because they have under eye circles, they don’t feel well, its like they’re weak. When you look to their eyes, they’re fingers its like they’re (iub) its like bloods not running to their veins.

**I: Ok… now is there a women that you know she has low blood and thinks that this illness is really dangers for them?**

R: As of this moment that I am still here on this island I haven’t. Maybe if I go to the clinics and roam around and see for myself but at this moment I think I know… or I could tell who or not.

**I: Hmmm… now what does it makes for young girls and pregnant girls gets low blood?**

R: Not enough blood like for the gregnant girls… they go screening…I don’t really know what it comes from. Some it might be a illness but from those screening I realize that there are less blood, but I don’t really know what if comes from.

**I: Okay… Do you have any words of advice to the women or pregnant girls to prevent or to cure this illness? From low blood?**

R: There’s no different from letting them know that hospital is giving back and forth for the next three months for the illness and they need to see the doctors because these are the most important months. And its important for our healthy life and for our children. That’s why hospital insure them to go see the doctors because its important for both of them.

**I: Now we are going to talk about breastfeeding in this community. Umm… Can you tell me how long before the baby is born do the mothers breastfeed in this community?**

R: After they are born?

**I: Yes.**

R: The time the child is born is when they start breastfeed them because that’s the first nutritious thing the baby is going to receive. Also because the breast milk has cholesterol in its nutritious and its what the baby receive.

**I: Ok that’s good. So can you tell me how women in your community breastfeed?**

R: How do they breastfeed?? How they breastfeed…

**I: Like just breastfeed, nothing more, they don’t give food but just breastfeed.**

R: Ohh… there is in our community, we see them breastfeed till the baby is six months and some until they reach two years, but some when their babies reach six months they start feed them foods.

**I: Now do you know what else they give to the babies other than breastmilk given in their first six months?**

R: To those that I’m closer to them and I can see them, I think no one. The breastfeed and breastfeed only, until the child reach six months they start to give them food.

**I: Ok… What are the difficulties faced by mothers in your community to practice exclusive breastfeeding for six months?**

R: Some they don’t really understand that they thought their breast runs out of breastmilk…

**I: Hmmm**

R: And some usually don’t want to breastfeed. And we explain to them that as long as they breastfeed the child the breast will always have milk in it.

**I: Okay… Is there any other issues about breastfeeding that they don’t understand?**

R: To those that they don’t like breastfeeding in public they will only breastfeed their child in their home.

**I: Hmm… Ok so is there any word of advice for the mothers that they should breastfeed throughout that first six months?**

R: Yes. The thing that the hospital says breastfeed…complete breastfeed before feeding them after six months. It’s important to breastfeed them first because it’s the nutritious that the child needs its in those first months. From the first month until six months.

**I: Ok now we are trying to understand how people eat in this community. Could you please describe in detail what most families usually eat and drink throughout the day?**

R: We usually follow our daily diet that depends on our daily needs. Morning we eat bread, pancake and donuts…daily foods… Afternoon Rice will never be missed… its for afternoon and evening. Now about meat… any kind meat they can find as long as they can eat it.

**I: Okay… So what kind of meat do they usually eats during afternoon and evening?**

R: This community usually eats chicken… and I don’t know if hotdog is a meat but they usually eat hotdogs too. Some, because few Men go fishing near the lagoon and their family gets to eat fish. But the chicken is from the store and that’s what the eat the most.

**I: Okay that’s good… So are there ways to make these foods? Or how do they make these foods?**

R: The faster way the see it its boil. Some they fry.

Some they cook it like stew and stuff like that.

**I: Okay… On how they cook these food…do they mix them?**

R: If they can they can mix them, some use cabbage, some mix with cabbage and onions and other stuffs... Some when they can get the ingredients they get it.

**I: What do you mean by when ‘they can get it’?**

R: Its when they can afford it from the stores.

**I: Ok… So who in the family gets to have food first and gets it last?**

R: Ok Marshallese custom the Man of the house goes first, the father of the family. And if there’s a Women that breastfeed then she will eat first. She’s the one that needs the food always.

**I: Is there any difference on how each family share their food?**

R: Its not how we share it but when a Women is breastfeeding then she’s the one that eats most but other than that, everyone eats together.

**I:** **Is there any difference on how the amount of food is being given to each family member?**

R: Who’s giving?

**I: If… if…. For example if we share… if there was three room in the house and you share the food to each room… So this says is there any difference on how they share the food to each family. If it was you and your family and I gave you a big amount of food… and if it was me and my family and I make our food big and those other two family I give them little amount of food.**

R: No… No because we are Nuclear family and we work hard about it and the amount of food to each one is the same. And other than that the Women that breastfeed is always the one that gets to eat more and has to eat first because everyone knows that she breastfeed and that she has a child to feed. That’s the only reason they give food the one that breastfeed first. And other then that everyone eats together because its in our custom.

**I: That’s good… So is there any children that gets more amount of food then the others?**

R: I don’t see it… I don’t see it because we Marshallese we know our manners.

**I: Now could you describe any food sharing between family members during mealtimes (For example children eating together separately from the family, meals eaten from the same plate by all family members)?**

R: Everyone eats together. It can be in one plate or everyone with each Plates but everyone eats together.

**I: Do family share food with their neighbors?**

R: When the food is enough we share because its in our culture we share our food to neighbors**.** But nowadays people barely do it but its really in our culture to share.

**I: Now I want to know about how young children eat in this community. Can you describe in detail what Children under two years commonly ear throughout the day?**

R: Under two years… Under two years they usually eat food that are soft. And because this is the Center, they buy baby foods. And the outer islands there’s already Nutritious food that is already there and ready to be made for the child.

**I: Now soft food. Can you give me in detail what they are?**

R: Papaya, Banana, Taro, but here in the center like I said the baby food is ready and already made and easy for people to get.

**I: Okay… now the child that is already one years old, what do you see that the mother usually feed them? Any kind… any kind food that they give them?**

R: after… after they reach those years… One year and above, I think there’s no difference. They can eat with us grown ups.

**I: Is there any time that they give them snacks or junk foods?**

R: They usually tell the kids to eat orange apples and other stuffs. But like I said about the Marshallese diet, kids one year and up are the ones that usually eat sweets and chips, those are the kind of junk the kids beg to have.

**I: How many in a day do these kids eat?**

R: Three… It has to be three.

**I: How often do the kids eats between every hour?**

R: Between hours?... between hours they usually eat chips and those other kinds of junk foods.

**I: Is there any difference on how to feed the kids when their sick?**

R: Yes. When you see it now, parents give them food that are Nutritious.

**I: Yes. Now other than Nutritious food, is there any other foods you give to the sick child?**

R: Yes. Because around here we usually try to make them feel good when they are sick, they can give them junk food like ice cream and candies. They give these with the nutritious food.

**I: Now is there any differences in feeding girls and boys under the age of two years? And why?**

R: I don’t think there is… I don’t know if there is but from me no.

**I: Can you talk to me about what influences how families feed their children in this community?**

R: Understanding, if they understand… if they understand the important of the food then that’s what they will do.

**I: what do you mean by understand? Understand about what?**

R: Understand about the important of healthy life and the nutritious foods.

**I: We have heard from some families that eat local foods and others that eat processed foods. Could you explain what is typical for most families in this community?**

R: We here in the Center usually eat food that are imported because those are what we can get.

**I: Now is there any…? What makes it difficult and easy to cook our local foods?**

R: Difficulties and what?

**I: Easy to cook our local foods.**

R: what makes it difficult for us to cook?

**I: Hmm. And easy. How we cook our local foods.**

R: well here is Center there’s not enough local foods, because this is the center it is not enough area to plant for our local foods. There can be some people that sells them but on outer islands we plant our own foods. But here we buy.

**I: hhmm…**

R: And that’s also the issue because of the cost.

**I: Now what are the good and bad about the local foods?**

R: good because they are nutritious and good because when your in outer island you can make money out of it but bad reason of it its when you’re here in the Center and you have to buy your own local foods. Because you barely have time to plant.

**I: That’s good. What are the goods and bad of imported foods?**

R: The imported foods don’t have Nutritious in them other than the fruits they send here. And because the food the make in the factories they make it with chemicals and the process them, and they don’t have nutritious in them. When they are imported here we buy them. That’s one of the bad reasons is we have to buy them.

I: Okay now do you have an idea on how they should balance meals that can be prepared with locally available ingredients for children under two.

R: Idea on how?

I: Food that are nutritious.

R: Well…?

I: That its good for children under two years.

R: They should make it affordable for other family to afford it and take it to eat it.

I: Is there any other reasons you wa…

R: Other then that one family has to plant there own food so that it’ll be easy for them to get and not to spend money.

**I: Okay that’s good. Can you talk about what messages about breastfeeding and complementary feeding you give to mothers or other community members?**

R: What do I???

**I: what are the issues of breastfeeding and food for the mothers and the people in the community?**

R: Breastfeeding, As long as the mother eats nutritious food then the baby will also receive Nutritious from the breastmilk.

**I: Is there any specific ways that this nutritious communication could be more effective in this community?**

R: We don’t really…, we can counsel people about nutrition like pregnant Mothers, we barely do any work shop like about Nutrition. Maybe here needs help for the outer islands and for the LA assistance on the outer islands.

**I: Is it important for these to happen in all the other communities?**

R: Yes very. There’s usually a team from here that goes to the outer island to teach about Nutrition but nowadays I don’t see them anymore.

**I: Is there any issues on how to teach people in the community?**

R: No there is none because people wants to learn about nutritious food and food that they have and on how they will make them.

**I: but….?**

R: But like I said we don’t see the programs these days.

**I: How would you want to have the right answers for these?**

R: If they continue to do the programs on teaching about Nutritious and counsel women individually it will help them a lot to learn and understand more about it.

**I: Okay that’s good. Now I would like to talk about pregnant women in this community. Can you describe their diets during pregnancy?**

R: Here in this Center its really hard to monitor what pregnant women eats, people in their household are usually the one to make their food, outer islands they have no problem because they are surrounded by Nutritious food and not imported food. But here if you see a Nutritious food and you can afford it then you can give it to the pregnant women.

**I: Now the women’s that are pregnant, is their food different when their pregnant?**

R: Like I said they should have a big amount of food because they are pregnant.

**I: Is there any more reasons of why they should have different food?**

R: The main reason is because of the child in her womb.

**I: Okay. So what is the big difference about the food the pregnant women eats.**

R: Healthy life for the child and the Nutrition in the food.

**I: What are Women encouraged to eat during pregnancy and why?**

R: They encourage them to eat Nutritious food because its good for the baby, so that they will grow healthy and to be bright in school. They will be good in their studies and can see clearly. As long as the mother continues to eat healthy.

**I: What kinds of food that women are encouraged not to eat during pregnancy and why?**

R: Junk foods. And salt, food that are salty stuff like that… they usually tell them not to eat junk food because it has too much sugar and salt and grease in them.

**I: So you mention sugar and salt… what are the effects of it the pregnant women?**

R: It will affect the baby… there are a lot of illness that’s shows from eating a lot from sugar and salt but to pregnant women it will affect the baby inside her.

**I: So who encourages or discourages eating those foods during pregnancy?**

R: They take their understanding from the hospital counselor or the nurses.

**I: Is there any vitamins in the pills that they take?**

R: Yes, there is its called pre-natal vitamin. And if they see that she don’t have enough blood they will giver her iron (self aid?) there’s already medicine that they already separate for the pregnant women’s.

**I: Ok. What are the issues for women to prevent taking their pills?**

R: Them coming to the hospital and some if they don’t really understand the cause for the medicines they won’t come and take their pills.

**I: Oh ok. During their pregnancy do they smoke or take strong alcohol?**

R: These days yes, they Smoke and they Drink.

**I: From your point of view, do you know why they take these things?**

R: Well I don’t really know why because the ones that I see around I don’t know them but I see them drinking but they are pregnant.

**I: What will be the illness from them taking those things?**

R: The alcohol is not good for the baby and it will affect the baby.

**I: Can you describe women’s diet during breastfeeding in this community?**

R: The community in the outer islands they have breadfruit, banana, Bandanas and papayas. Here they also have it but they have to buy them.

**I: So do women that breastfeed needs to change their eating habits?**

R: They need to change it into a healthy way both her and the baby can have.

**I: So what are the foods that breastfeeding women are encouraged to eat and why?**

R: like I said nutritious food is good for both her and the baby.

**I: What are the food that they encouraged them not to eat?**

R: like said before, junk food and food that are salty and food that are sweet are the kinds that will show complications and problems for the breastfeeding mom and baby.

**I: who gives out the advices to the women’s?**

R: first one is the doctor, second one is the nurses and also from the LA Assistance that they usually do individual counseling. And also they hear it from the radio stations and social media.

**I: As a health worker, what are some of your biggest concerns of the diets of pregnant and breastfeeding women in** **the communities you work in?**

R: Some food that we see them eat that or not ahh good for them is ramen and what they mix it with, and also lime they eat it with salt. And some they eat tobacco with their food.

**I: Hmm. Soo what are the affects of those things?**

R: It will affect the child because salt and those stuff are bad for the child.

**I: For our last section, we would like to learn about ways we can develop health programs in your community. Could you explain where community members usually get’s trusted information about nutrition and health?**

R: We usually get the information from the hospital or for the outer islands they get it from the LA Assistance and some school they put up posters, and like I mentioned before there was a team from here that teaches about nutrition and about healthy life.

**I: Why do they trust the information that got from the people you mentioned?**

R: Because they made them really understand about Nutrition and healthy life, and its clear for them to understand what they we’re thought, if from the school that’s where all the kids learn about these stuffs and about for things.

**I: Where would you prefer for all these Information to go for everyone to hear and know about it?**

R: The school and the health center is where everyone can see these Information.

**I: What about listen to it?**

R: The radio is what we will listen to it about these stuffs, like on v7ab station and 103… and also the churches stations**.**

**I: So ok what kind of device do you use to listen to these kinds of Information?**

R: I usually use the SSP Radio kind.

**I: Is there any other kinds you use for sending out Information**

R: We can also use our cellphones or go online using Facebook.

**I: Ok that’s good. For our last question, could you describe what influences how people raise children in this community**?

R: what’s influence them? How do they influence them?

**I: Like word of advice or…**

R: Ohh… how they influence them…. The only place that will influence them is the church, the school and the health center. If they influence people, they will listen. That’s just the way I see it.

**I: so in your household, who among the people in the house influence them?**

R: in our culture the man is always the one to influence them on doing whatsoever, and the women influence them on house chores and foods.

**I: Do you have a word of advice about influencing our children?**

R: Yes, To the males and women they should influence others on how to care and provide for their family.

**I: Is there any information that pregnant or breastfeeding women typically ask for from health workers?**

R: I don’t think they ask much from the health workers other then just take their medicines and go home

**I: why don’t they ask?**

R: Sometimes they can be shy, but on the outer islands usually the males goes to the health worker but not the women because they are shy. Its good here because there are women nurses and they can help in any way they can. But on the outer islands there’s usually only male workers, that’s why the females will be shy to ask the male nurses for help or to ask them questions.

**I: Ok. Is there a best way you can think of that is best to communicate with caregivers about health?**

R: Yes. If they don’t come with their own aid like for the sick people, they can come with their wife’s or there should be a female working with the LA Assistance with these kinds of issues.

**I: What about the males? Do you have any saying for them about caregiving?**

R: well they can just listen to their wife about that because they are the ones that take care of them and they know more about how to care for them.

**I: Ok that’s good. Is there anything else about the topics we talked about today that we missed or that you would like to tell us about?**

R: You said everything out perfectly so I don’t think there’s anything else.

**I: That was great, we are done now. Thank you once again for your generous time and for sharing your thoughts with us. We greatly appreciate your help and we hope this research will help us improve the health of mothers and children in your community**.

R: No problem anytime.

I: Ok thank you once again.
