# Supplementary material for: Conceptualizing multi-level determinants of infant and young child nutrition in the Republic of Marshall Islands–a socio-ecological perspective
Source: PLOS Glob Public Health. 2022 Dec 19;2(12):e0001343. doi: 10.1371/journal.pgph.0001343 (PMC10022247; doi:10.1371/journal.pgph.0001343)
Supplement: S1 Data — (ZIP) [file pgph.0001343.s001.zip › RMI Supp Data/Interviews data/I02U_IDI_MCG_Rita_Aug 13_Balton_edited.docx]

- Interview Code: I02U
- Interview Type and Interviewee: IDI MCG
- Interview Date: Aug 13, 2018
- Location: Rita
- Interviewer: Balton
- Transcriber: Cendaniel Milne

**I: okay, is it okay if we talk?**

R: okay

**I: we’ll start off with a question regarding the process I am about to explain. First, I would like to thank you for your time and cooperation for helping us with this survey for a greater purpose. The first question to begin with, Can you please tell me a bit about your family? Anything about your family.**

R: like what?

**I: those who live in the house.**

R: Me, and my wife, my three siblings, two boys, and a girl, and my oldest son, my parents, that’s us.

**I: just you guys?**

R: yes.

**I: Can you tell me if there is another child apart from your child who lives in this house? A young female child?**

R: none, it’s just him.

**I: Thank you…can you describe a bit about the community, how is it? , can you explain?**

R: Life in this place, Terong Village had two parties.

**I: Two parties?**

R: so, now is settled after the land dispute case

**I: can you explain how come it has two parties?**

R: because, there was jebta (traditional Marshallese dance group)*.* Those who lived in near the lagoon side and those at the ocean side.

**I: so now, there’s just one?**

R: there’s one.

**I: you’re good.**

R: If only I was more mouthy (Talkative)

**I: Huh?**

R: I’d be more helpful if I were the type that talks from dawn until dusk.

**I: let’s discuss about health and illnesses. Can you tell me some of the illness that your children have suffered from? Illness that is common**

R: Diarrhea and Fever. Oh, and also flu.

**I: can you tell me how frequently he gets these illnesses?**

R: I do not know

**I: do you know what causes him to have a diarrhea?**

R: The other older kids in the neighborhood usually come feed him and spoil him. They adore the younger kids.

**I: what about fever? Can you tell me what causes him to have fever?**

R: it seems I am afraid

**I: it’s okay. There’s no problem. At what time, or when, do you know your child needs to see a doctor, describe how you know, the symptoms you notice that indicates he needs to be treated?**

R: he cries aloud and cough

**I: when he cries aloud and cough?**

R: yes.

**I: During those times he’s really ill, who do you take him first to?**

R: his grandparents.

**I: great. Do you use of traditional healing or traditional medicine?**

R: I don’t, I take him to the hospital.

**I: hospital? Can you describe any illness affecting your children that are associated with nutrition? Are there any foods that makes him malnourished… or not enough nutrients in his foods?**

R: I do not know.

**I: That’s fine, all good, remember there are no right or wrong answers… can you explain what type of foods that can make your child’s body healthy, ones you know of…? Can you give some examples on what foods make him healthy?**

R: I’d say, food’s that are grown at home.

**I: can you tell me more on what type of foods grown at home?**

R: You know, basic Marshallese fruits, papayas, banana, breadfruit, taro… those kinds.

**I: thank you. And what types of food would harm his health? What food best avoid?**

R: Mostly canned food. And rice.

**I: And what?**

R: rice.

**I: what kind of canned food?**

R: Mostly corned beef and spam, things have fats in them…

**I: And what kind of illness that can make him ill, due to the foods he wants? What are the illnesses?**

R: there was this time he ate a corned beef, and had a diarrhea.

**I: well, we’ve talked enough about malnutrition. Could you now describe for me a typical day of someone living a healthy lifestyle? How does a person….like…clarify how does a person become healthy, what does a person do to have a healthy lifestyle?**

R: Typically, they’d brush their teeth, take a shower, have breakfast, enjoying the fresh air, take a walk, doesn’t sit still, always moving, not laying down, eating a noon lunch, enjoy the rest of the day, eat dinner, take a shower and then sleep.

**I: great…what are the appearances or signs of a sick child under 2 years, what are the signs that indicate him being sick?**

R: When?

**I: Lets say, for example, what are the signs of a child under the age of 2 years, that shows a child is sick. Like… shows that he’s sick or not well?... Indicators that shows that shows the child shouldn’t be at the current state, … like doesn’t seem healthy. When a child doesn’t seem healthy, how can you tell?**

R: I can tell when the child doesn’t move, frequently laying down, doesn’t feel like doing anything, everything… everything is a drag.

**I: what about an adult? How can you tell if an adult is in the same condition?**

R: Like if he/she has friends, types he usually hangs out with and do stuffs, but now just stays indoor, becomes a loner and doesn’t speak.

**I: Thank you. Now we will discuss about….food growing on community, we are going to talk about food availability, can you explain how your household gets food to eat on a daily basis? How can be there foods at the house?**

R: There’s two people who works in this house. One who works for the government and one who works for a private company (Pay-days on different weeks). When the first get paid he takes care of all the needed stuffs during the week from Monday to Saturday, then when the private company worker gets paid, he is in charge of paying for food and stuff for the following week, then the government worker takes over the next, they take turn each week.…there are also times when we we go fish or eat off the land.

**I: Off the land? Like what?**

R: Breadfruit, papaya, taro. My grandparents tell us to pick breadfruit sometimes when its in season, sometime we take trips to Arno and get food from there.

**I: Oh wow, that’s great. Are there any foods that are grown at your home? Do you plant?**

R: There are but they’ve gone to ruin

**I: Like what kind?**

R: Like a papaya I planted, we ate from it only once before it died. Then there’s the breadfruit and bandanas. When its in season, because we have neighbors we share when its in season.

**I: Oh wow, what about the other food? Like we mentioned about seasonal reap of plants like breadfruit and bandanas, how do you look for food, can you explain?**

R: there are times when I go fishing in the tide pools on the ocean reef.

**I: are there any times you sold foods like breadfruit, bandanas, sold them for money?**

R: It’s like, my neighborhood, we only just share.

**I: Oh, just share... can you tell me about any difficulties to growing food at your home?**

R: there are just too many houses…

**I: It’s crowded?**

R: Yeah, and with so many kids. They think everywhere is their playground.

**I: if you were to plant, what kind of food you were to plant to help your family? If you were, or if you can.**

R: can you explain more.

**I: if you were to plant a food near your, what kind of food you were to plant it to feed your family?**

R: It’d be something like a breadfruit… Because we grew up on those, and bananas….and bandanas.

**I: That’s it?**

R: Oh, and also coconut trees… and yeah just those.

**I: we discussed the seasonal harvesting for breadfruit, could you explain how easy or difficult it is to get those foods you for your home? If it is not a seasonal harvesting for breadfruit, is it difficult for you to look for food for you guys?**

R: When not in season?

**I: Yes, when not in season, how do you find food?**

R: …... there are times I would contact my father for financial support, if he’s willing, he would give it, but if not, I would resort to my diving gears.

**I: Diving gears…hmmm**

R: Its usually salt fish that we sell, not to people here but to those abroad.

**I: Salt fish? You make salt fish?**

R: Yeah, m wife makes salt fish.

**I: you fish and she salt the fish? (Confirmed with a nod)... Now I would like to ask some questions about animals, if you are raising animals. And, are there any animals you are raising?**

R: I only raised a cat at home.

**I: Ha, just that?**

R: But the other people in the house rises chickens

**I: chickens?**

R: Yeah and my younger brother has a dog. We only raise domestic pets.

**I: Oh, just raise pets? None to eat?**

R: Oh, the chickens…

**I: Chickens? Do the chickens stay inside a fenced area?**

R: Yeah

**I: Like some places that raise pigs, but then you don’t raise pig. My next question inquires about the feces of the pigs So it might not apply to your case so let’s move on to the next question. But out of curiosity, what you do with chicken’s feces?**

R: The chicken’s feces have a use. Because many in my household experienced illnesses of the islands.

**I: what kind of illnesses?**

R: The kind which Marshallese conjure, Illnesses conjured to do harm.

**I: Huh?**

R: Usually when things befalls my home, it’s me who usually sees it. How I can tell the signs are they usually come in the forms of geckos.

**I: They come how?**

R: Creatures. They appear different than when we usually see them, there are times when we see them that look abnormal or different… just from my prospective.

**I: Hmm, and?**

R: Like recently my uncle had pain and swelling encircling his midsection, there were dead geckos for everyone in the house. I found and took all of them. One had a broken a broken front limb. Same like how my limb elbow can now bend backwards without ever been broken. Some had cracked skulls. This was when my uncle was still alive, he was seer, and I asked him what to do with the 1^st^ cursed item I found. He said to destroy it with feces. I asked how and he said to take it to the chicken coop and immerse it in the feces. He said to search the house for strange things and in places that nobody had clues about. When he asked where I found it, I said in the bathroom. He then said to search it again and the whole house. I then found many other things. Like diapers. I didn’t have any kid at the time and by sister was still very little. I then found also the geckos with the broken limb and one with the cut midsection. A week later my arm became limb and my uncle had a swelling line around his midsection. He than said he was going to seek cures for us and tell us who cursed us and to confront them. When we told us the person, we couldn’t confront her because she just recently died. She was one us that died of an headache.

**I: so, the bird feces?**

R: the bird feces nullified the hex.

**I: This is new to me… okay, there are sometimes food that we wish we could eat things, but for some reason we cannot. Could you tell me about any foods you wish your family could eat?**

R: only pizza

**I: pizza? It’s rare. What makes it difficult to eat pizza?**

R: what?

**I: this question asked, there are times we want to eat everything we want, but we cannot. What you want to eat, you mentioned pizza. Why can’t you eat pizza? What makes it difficult for you to eat it? Like every day.**

R: I guess money.

**I: money? …. Okay, for the last question on food, can you explain who decides what food to get for your family?**

R: only the adults.

**I: The adults… How… how do you decide the food, like, let’s say, If someone made dinner, who decide what to makes for dinner and why?**

R: With us, the person cooking.

**I: so, the person cooking. Who usually decide what to eat?**

R: I guess my mom, grandma… and also my wife. They decide.

**I: I see. Who decides what the young children should eat?**

R: Them also

**I: okay, were done about food and health, were going for water and hygiene. Is hard to find water in this neighborhood? Is it difficult to get water?**

R: I don’t think so

**I: how to you bring water to the house?**

R: We have about 4 water tanks in our home. Two small ones and two bigger ones. The smaller ones for bath and washing, one big one,oh and a well for shower and the other big tank for drinking.

**I: Oh wow, that’s good. You are more fortunate than many …. How do you, like for the drinking water tanks, how do you make the drinking water clean?**

R: What we do is we clean the roof and also clean the tanks (gutters in Marshallese) of the house.

**I: water tanks (gutters)?**

R: And the filter too from the tanks to the pontoon (the drinking water catchments).

**I: so, they got filters huh?**

R: Yes, and we also use Chlorine

**I: For handwashing, can you give me an example on how, the question asks about handwashing, and if you could explain how you wash your hands every day?**

R: we usually do wash our hands with soap.

**I: what about the kids, how do they wash their hands?**

R: if there’s hand sanitizer, we wash their hands with hand sanitizer, but it usually with soap. We generally use soap, dish washing soap.

**I: how frequent you use soap to wash their hands?**

R: Usually just before a meal.

**I: Just before having a meal?**

R: and before bed time.

**I: from your own understanding, what is the difference between using water only or water and soap to wash hands?**

R: I think there’s a difference, when we wash our hands with just water, not much changes. But when we wash our hands with soap, everything is clean, even our nails. And if there’s any scent, we cover it with coconut oil.

**I: well, it might not be you, but why does a person wash his hands without using soap? What would prevent him from washing his hands with soap throughout the day?**

R: He’s in a really hurry.

**I: okay. It’s great how you answer these questions, it’s great how you don’t just say yes or no. When you’re explaining and sharing your stories, it gives us rich information for our survey. This is the type of information these foreigners we work with want to get and see why and what makes the way they are. Thank you, your explanations are wonderful. So now we come to restrooms. Could you describe the type of toilet you have at your home?**

R: We have our own restroom.

**I: what kind of restroom are you using?**

R: It has a shower place and a toilet too.

**I: A toilet right? Where you flush… this type?**

R: yeah.

**I: What are the advantages of the toilet you use compared to others, like those outside with just the pit in it?**

R: I think its better. Inside restroom are better to use than the ones outside. My brother lives in a house where there is a toilet outside, and its kind of uncomfortable using it. There are always so many people around it.

**I: Is it hard to get one? Let’s say, if someone want to get a toilet for the house, is it hard to get one.**

R: I do not know, I have no idea about it

**I: What barriers would prevent people from getting those types of restrooms?**

R: Seems like also money related.

**I: How usually are young children’s stools typically disposed of?**

R: I don’t really know because I have not seen it happen around my neighborhood, and my uncles’ house and his neighbors It’s known to happen.

**I: can you explain where the kids at home play?**

R: outside the house

**I: outside the house?**

**I: are there any animals near the house?**

R: There was a dog, but it’s gone. The cat died in the gutters. Reason for last catchment cleaning.

**I: can you explain which place to play that would be suitable for the children?**

R: I think the best place for my kids is RES (Rita Elem. School). The field. It has a huge play field.

**I: what can you think of challenges of keeping a child’s play area clean?**

R: Trash people don’t throw away. Kids scattering their toys after using them. Also their snack wrappings like chip wrappings. They only think of playing and not about cleaning or keeping things clean.

**I: in some communities, people defecate at lagoon or ocean side. Why do you think people do this? Why defecate on the beaches just from your own understanding?**

R: I think maybe because they don’t have a bathroom. Or no bathrooms close by.

**I: Great. Oh wait… let’s retrace back to hygiene, could you explain ways to prevent the spread of disease?**

R: Huh?

**I: From your own understanding, diseases that are spread easily, what’s the best way to prevent it from spreading?**

R: I guess if, let say Rita, had its own place where you can exercise, it could help.

**I: Hmm, great, okay. When you think about it, does spreading of diseases and feces laying about have a linked relation?**

R: Yes, I think they are linked.

**I: Oh? how? Show an example. Your own explanation. How are they linked to each other?**

R: where there’s many feces, and strong wind, the odor spreads with the germs, it’s like, the germs moves around.

**I: now were going through Gender and Family Roles, the first question is could you describe the care of children throughout the day in your community?**

R: … are we talking about one household or more than one household?

**I: Yes, one household. Yes, because we are talking about your kid, any everyone’s role in his care**

R: for me, when I am not busy and his mother is busy, I would pull his baby stroller, and carry him around. And walk with him. I do not look away from since he now know how to walk. When I get distracted, he comes to me crying after hearing a car’s horn. He might cry from being scolded so I take him to his mother for breastfeeding.

**I: Who is mainly responsible for taking care of the child?**

R: Me, My wife, and my mother.

**I: the way you’re taking care of him, what are the responsibility of a mothers? Of taking care, what are the responsibility of a mother caring for a child?**

R: They should not be preoccupied from the child, feed the child, and make sure the child’s play area is clean.

**I: what about responsibility of fathers?**

R: Provide food

**I: how does the caregivers play with the child, can you think of how someone who is taking care of the child play with the child.**

R: ……. My sister usually take cares of the child, they always play hide and seek inside the house. And there are times when they both watch shows together.

**I: can you talk about the role of grandparents having in raising children in this community? ….. Question states, what about the role of grandparents raising children apart from the parents? In the community?**

R: I’d say, there is no significant difference. They do the same things as the parents. Except the spoil them to the point where they scold us for scolding their grandkids.

**I: are there any differences where grandparents are not with the family?**

R: from what I see, there’s difference

**I: there’s difference? Like what?**

R: there are times, usually when I have an headache, the child keep crying and bothering me, I get irritated. Let’s say, when the grandparents are gone, we cannot control our temper. And when we get mad at the child, we get mad. If we want to spank the child, we spank the child. But they’re there, if I get mad, they get mad at me, and tell me to get out the house and away from the child.

**I: what makes good grandparents?**

R: they’re good at treating the child, educating the child, and taking good care of the child’s needs.

**I: can you say a bit about the roles that other people in raising children in this community? Other taking care of your child in the community? About other neighbors treatments towards your kids.**

R: Usually the neighbors next door take my child with them when then see him and most often return him sweets and other treats.

**I: is there any other an older sibling?**

R: there is, but that child is with my Aunty in the states. Life is hard so my Aunty asked me if she could take raise the child.

**I: Are there other older kids who help take care or play with the child?**

R: just my little sister and my little brother.

**I: they always playing with him?**

R: yes

**I: okay, you’re doing a great job answers you’re giving. Now we would like to learn ways we can develop health programs in your community can explain where you usually get trusted information about nutrition and health?**

R: Some when my boy gets sick and I don’t know the cause, I do a research.

**I: at where?**

R: online.

**I: you have internet at your house?**

R: yes, I get connected at the house next to mine. And there are times I get 4g connections, if there’s none, I go hospital... but usually online.

**I: Where is easiest to get information from?**

R: I think the easiest place is to get it online

**I: where do trust to get an information from the most?**

R: also online.

**I: from the time you have become a father, and you have your own family. Was there anyone else who gave help you or gave you an advice the helped you to this day?**

R: I’d say no one, before I become a father, there was none

**I: what are your opinions of the community (people near the house, leaders of the community) what are the people’s opinion about the community? Like, the question is asking about what are the people’s opinion in this community about taking care of a child?**

R: …. From what I’d see in their thoughts about taking care a child is not good.

**I: taking care of a child is not good?**

R: Went they talked to me about it, they say it’s good to have a child because they bring fortune. But truthfully, their opinions about having a child is that it’s bad. We all understand the difficulties of raising for a child. It not easy raising a child. There’s others who don’t want to, but wants to compete who has more kids.

**I: Compete?Oh, that’s just great. Were there other questions you would like to ask?**

R: there’s none

**I: For any questions on things we didn’t discussed or cover, you can get in touch with the hospital, and the UNICEF specialist. You can also contact Hillia, the Chief Nurse, and Caroline, they’re the ones helping us with these surveys. And thank you for time. You have helped us with your answers. I appreciate how you answered the questions. Thank you for your time, bless you and your family.**
