# Supplementary material for: Conceptualizing multi-level determinants of infant and young child nutrition in the Republic of Marshall Islands–a socio-ecological perspective
Source: PLOS Glob Public Health. 2022 Dec 19;2(12):e0001343. doi: 10.1371/journal.pgph.0001343 (PMC10022247; doi:10.1371/journal.pgph.0001343)
Supplement: S1 Data — (ZIP) [file pgph.0001343.s001.zip › RMI Supp Data/Interviews data/I03U_IDI_Pastor_Rita_Aug 13_BM.docx]

- Interview code: I03U
- Interview type and interviewee: IDI Pastor
- Interview date: Aug 13,2018
- Location: Rita
- Interviewer: BM
- Transcriber: Showme Lelwoj

**I. Do we have consent from you that you want to go on with our interview?**

R. Yes

**I. Thank you for giving me your time for our interview today. Can you please tell me more on what are your responsibilities as a community leader?**

R. Sure I would, thank you God for this very pleasant timing. One major responsibility as a community leader is family, another is in the community, and final responsibility is the church. In my family they are my first priority because of their needs like getting into school. Another important factor is the growth factor of a young child is most importance to me. As a father in the community we need money and work just to get pass the school year. As a leader for the youth in church, I help out a lot with giving my advices to the youth and there president to that they could understand a better way to get more youths in this village to know the words of God.

**I. Thank you for those very good explanations, can you please tell me about who is a part of your community is?**

R. Rita we have a lot of mixtures of Marshallese from all over Marshall Islands, every villages in Rita, we have different community leaders. There are mayors, council men and two others I don’t know what they do for the community. There are some outside religions now are here in Rita like the Jehovah witness, another are the Mormons and there here to work with people around this community those are all things I could say about the different religion groups. But for ethnic groups there are Kiribati’s, there are Pohnpeians, chuukese, Chinese, and other ethnics I am not familiar with. The importance of a religion in a community is to the inspire of them to change and make this community a better place not just for us adults but for the young ones, as for a this religion we want people to know that we are growing and are making some differences in the upcoming weeks and years.

**I. Thank you so much for those important details, now can tell me the positive things about your community?**

R. In this community our positive things are with the young adults nowadays, it’s not with the adults or community leaders or the government. Here in Rita there are tournaments for our young adults and people from different community come and watch them play basketball volleyball, baseball, and other competitive sports, there was this one time if I can recall a few years back when the whole Rita communities came together as young adults and had a cleanup team which was one great aspect in my life I remembered we had a really good time and many face showing in hand to make or village a better place for us to live in. Some negative aspects here in this community are car crashes from young teenage driving, drinking alcohol, these things, young teens stealing houses. They make peace out of order. Food related difficulties has to do with low financial aid like money. We don’t eats our own crops but we eat other ethnic foods and want more of it. Now we are so used to these foreign foods that we tend to forget about the nutritious foods we have here in Marshall Islands.

**I. Let’s now talk about health and illnesses in your community, could you please explain to what illnesses children commonly suffer from in your community?**

R. I cannot tell you about every household in my community but I can tell you about some to the major factors to the illnesses that are in my neighborhood, such illnesses such has fever, mild headaches, but there are no more that I know of. What I know is that they get these illness from playing around the village too much and sweetness is still dripping from there body but they take showers.

**I. can you tell how me the seriousness of fever?**

R. The seriousness of fever is death. Only if we don’t treat them right. Like giving them medicine or cooling there body but these are my plan to giving the sick child a better health.

**I. You said headaches right, can you tell me more about it?**

R. For headaches my information about it are not so much about it but usually take medicine. I also would give advice to the person who has the headache.

**I. Are there any ways to prevent each illnesses?**

R. Other than the ways I said previously there are no more ways I know I can give as any use information. My information towards headaches are not much**.**

**I. Can you explain what type of treatment people in your community seek for their children, for example traditional healers, doctors, nurses?**

R. From the sicknesses that come out of nowhere the doctors cant identify what kind of illness they have, for Marshallese medicine, we usually walk around and find these medicines, from the hospitals when they can identify the sickness they usually give them pills or other medicine, other use of medicine here are from our own bananas.

**I. Can you describe any illnesses associated with nutrition that affect children in your community?**

R. From food Like “chunk foods”, also too much sugar sweets, and from not really prioritizing their meals every time they eat like watching out on what they eat.

**I. What foods are make a child’s body unhealthy?**

R. “chunk food” makes their diet low and low vitamins, a lot of not good beverages, parents also play a role on giving these type of foods to their children. This leads too many illnesses and causes such has bad breath from rotting teethes, low care on their hygiene’s.

**I. Now we would like to learn about the foods that are commonly available in your community. I would like you to explain in your community typically get food to eat on a daily basis?**

R. People in this community has food when there are workers in the family. The father is the one who usually helps the family and gets the food for the family. Some types of grown foods that are bananas, coconuts trees, bandanas, and breadfruits. There are a lot of bandanas and a lot of coconut trees. There a lot of house who don’t have the ability to grow in there soil.

**I. Are grown foods sold or traded?**

R. Here is a lot different from places like Laura because they don’t sell their goods and mostly they are eaten.

**I. Can you tell me about any difficulties to growing food in this community?**

R. The thing is difficult to grow is seeds, the soil is bad, the yard is not good to grow, and there are animals that mess with the food that they grow.

**I. What families would need to grow more food?**

R. Tools would be needed, better soil to grow, machinery to make a really good garden.

**I. Could you explain the easy and difficulties it is to get those foods you mentioned every month during the year?**

R. So to get these food we need to be keep a safe garden, do what is right for your garden like feed them water here and there, we ourselves need to be very diligent with our garden and not make it so the sun eats it up all the time.

**I. “mm mm” Any other food shortages throughout the year and their main causes?**

R. From the foods we eat the way it gets shortage is sharing or the bad responsibility not look after it. Mainly the reason is sharing throughout the community. If there are a lot of teenage around we tend to share with them.

**I. Could you please tell me about what type of animals that are commonly raised here?**

R. Cats, chicken, dogs, rats, pigs. Longtime ago there were giant lizards but now days we don’t seem to see them around the island.

**I. Okay thank you now can you please tell me are there any difficulties raising animals?**

R. For raising dogs for them watch there properties, for tabbing pigs we use them to sell or trade for family needs, for chicken we usually tab them so that we eat, for other reason they wake up in the morning times. We also sell chicken for money and buy our family needs. For cats eat tab them to kill of rats.

**I. Can you explain any difficulties to keeping animals in fenced areas?**

R. Yes there are difficulties for pigs to be fenced from the uncleanliness they have and gets people sick.

**I. Again thank you for these information. Now could you tell me what community members typically do with animal feces?**

R. For pigs feces they fertilize them and they use this type of leaf and after they use the fertilizer for soil use for the garden to grow crops.

**I. “Uh Huh” can you explain who decides what food to get for the family in the household?**

R. It is usually the mother or the grandmother if not the head father of the family who chooses the food.

**I. Okay thank you can please tell me who decides which foods young children should eat?**

R. The parents or the elders of the family get to decides what the children should eat.

**I. Can you please describe a typical day getting and storing water for the people in your community?**

R. For water we store them in our big water canteens that mostly every house in this community usually has. Other supply material that are used to store water are the 5 gallons or wells we have also here in the community.

**I. Thank for these important explanations what are the main sources for drinking, cooking, for washing, and for bathing?**

R**.** In this community we get cooking and drinking water from MWSC (Majuro Water and Sewer Company). Then for washing and bathing the large canteens we have here.

**I. Okay what makes it difficult to have water?**

R. If there are no rain for two weeks or for a month. Then we usually wait for MWSC to turn on the faucet water and we wait for the right day or timing for this to happen which sometimes could take like two or less times a week. Times of just sun and the land is dry. Then we buy water which cost money. Another difficulty is outstanding dept. with the water company. Sometimes when there is less washing water it makes it hard for us because we don’t get to wash our clothes

**I. “uh huh” What’s the main difficulty for storing water?**

R. Difficulties with storing water is that we have less material like water canteens to store our water. There has to be more than just one large water canteen for household because it sometimes runs out fast and it will be a better way to store these waters.

**I. Thank you again for these wonderful information. Now can you please tell me more on what ways your community tries to make drinking water safe?**

R. There needs to like a way to test our waters but now we don’t have this right material to test our drinking water. We have the EPA (Environmental Protection Authority) who comes to test our water and lets us know if it’s safe to drink. Another very good information that spreads around the island say a drop of Clorox can clean a lot of dirty dishes.

**I. Good explanations, now we will move on to discussing about hand washing. Can you describes ways you hand wash yourself or practices in your community?**

R. I don’t really see how people in the community hand wash but maybe sometimes it won’t take has long like 10 to 15 min. of hand washing but they take the soap go about around there hand for 3 minutes than finish.

**I. Okay good, now can you tell me the differences between hand washing with soap and just hand washing with water itself?**

R. From what I know from the doctors soap is way better than just water itself. I say this because when we wash our hands with just water sometime our fingernails are long and there are bacteria inside our long nails. We need soap to make these bacteria disappear from our hand. We use also hand sanitizer as well throughout the day.

**I. Thank you a lot. Now can you please tell me are there anything that prevent you or your families from hand washing with soap throughout the day?**

R. The reasoning is that when people see if there is no soap they just straight just use the water to hand wash or maybe there lazy or they don’t tend to look for soap.

**I. Thank you very much for answering our questions. Now can you describe to me the types of toilet is used in your community?**

R. Majority of this community it’s a lot different because a lot use the toilets bowls. From before where people just dig holes on the ground and use a box or to sit on is and just let it go. Nowadays it’s a lot safer.

**I. Good thank you, we’ve heard defecating in the open, is this still common? And how common is it?**

R. Yes there are places where people don’t have restrooms so they go to the lagoon side or the oceans side. Sometimes people don’t use these old types of restrooms because of embarrassment from other community members.

**I. Okay now can you tell me the barriers to using toilets?**

R. if the house doesn’t have salt water running to flush toilet.

**I. Good now can you tell how young children’s stools are typically dispose of?**

R. People sometimes are non-careless so they just throw it away around the house or anywhere they feel it is okay to just throw the diapers. Some other usually are responsible t=so they keep it safe by throwing in their trash.

**I. Now can you please explain where children usually play in this community?**

R. They play where the yard is or they play at the basketball or they play inside the neighborhood. Times before now they usually play by the road. Now it alt better because they have places they could play like the basketball courts.

**I. Good, now can you tell if there are animals around where the children’s play?**

R. Well there are times when there are dogs and other times where there are no dogs around.

**I. What are the challenges of keeping children’s play are clean?**

R. The challenges if people or parents don’t take part to cleaning the play areas and leaves it unclean.

**I. To wrap up these questions on sanitation, could you explain ways to prevent the spread of disease?**

R. To clean your community, clean our disposals, clean our homes right, and clean our children, foods, drinks and our dish cleaners. This way we can prevent diseases.

**I. Thank you for those very important information, now we can move on to gender and family roles. Could you describe the care of children throughout the day in your community?**

R. Okay just for me looking around, the parents usually let go of their kids and go play bingo or the dads around here are too drunk to look after them.

**I.”Uh huh” so who is mainly responsible for child care?**

R. There are and there aren’t any at all sometimes because there are a lot in of members of the family. So when there are a lot in a family member their careless because one reason is that when there is more than one family member looking after the child the other would maybe careless.

**I. Okay now, what makes a mother responsible in child care?**

R. They would wake up early in the morning to clean the child, take them to school.

**I. Good now can you tell what responsibility of a father in child care?**

R. A father does know what he needs to do, when a father is good when there is no problem in the family and takes care of his family, he looks after the house as well.

**I. How caregivers play with the child?**

R. If the child caregiver is under 25 years of age and he or she knows what the kids needs and looks after the child and makes the child joyful.

**I. Good reasons thank you now can you tell me what the roles of the grandparents for raising the child?**

R. They teach them our cultures and what they need to know, what things to know, how to live and what our ancestors told them so they share with their grandkids.

**I. What ways that grandparents support in raising the child as well as the mother and families?**

R. They raise them good, they know that the child is special and want to look after the child, they keep safe food for their grandchild, and another is the grandparents share what information they have only to the grandchild. The mother

**I. Good Information now can you please tell me what makes a good grandparent?**

R. The work they do, the talents they have, and the way they speak out to the grandchildren’s and what they have to share with the grandchild.

**I. Thank you again, now could you talk about the roles that others have raising children in this community?**

R. I have no information for this question.

**I. What are way that siblings (older siblings) help raise young children?**

R. Usually the older siblings help out the young one by getting the young ones needs like if the elder sibling was to work he would help get what the younger siblings needs in various needs.

**I. Thank you so much we are almost finished. Could you explain where members in your community got these trusted information about nutrition and health?**

R. Where I got these information about nutrition and health are from the hospitals and they share what’s right about a child’s life and what is right for them so that they will live a healthy life.

**I. Can you tell me the reasons why these sources are trusted?**

R. The information we caught from the radio station, the local public health. They give out really important information that we know they must be followed for our wellbeing here.

**I. Now can you tell me what type of media the community use the most to communicate?**

R. There are devices, but in the outer islands they ant be reached, Samsung and iPhone are used, there are radios, GP radios, and for information we can communicate.

**I. Thank you so much is there anything you want to add?**

R. Thank you for interviewing me and giving me the time share what I wanted to share. I also hope all these information will help you. Hope that all goes well with the rest of your interview and thank you again and hope all the information I gave to you it.
