# Supplementary material for: Conceptualizing multi-level determinants of infant and young child nutrition in the Republic of Marshall Islands–a socio-ecological perspective
Source: PLOS Glob Public Health. 2022 Dec 19;2(12):e0001343. doi: 10.1371/journal.pgph.0001343 (PMC10022247; doi:10.1371/journal.pgph.0001343)
Supplement: S1 Data — (ZIP) [file pgph.0001343.s001.zip › RMI Supp Data/Interviews data/I04U_IDI_FCG_Rita_Aug 13_Fela.docx]

Interview Code: IO4U

Interview type and Interviewee: IDI FCG

Interview Date: Aug 13

Location: Rita

Interviewer: Fela

Transcriber: Christina

I**: Before we start recording our conversation, can you say it out loud if you are agree, is that okay?**

R: Okay.

I**: Good, thank you. Thank you for giving me your precious time for us to talk to you today. These information that will be given by you will help us find an healthy lifestyle for the women and children and, also for a healthy environment in our country. For starter, can you tell me a little bit about your family? To make the question easier, can you tell me who lives in your house?**

R: Me and my parents, and my three sisters…

**I: your three sisters…**

R: that’s it…

I**: okay. Now can you tell me how many children and their age, or how many girls and how many boys?**

R: Three girls and two boys.

I: **Good, now as for the girls, can you tell me how old are they?**

R: One is 18, the other one is 20, and the last one is 30.

I: **What about the boys, how old are they?**

R: There’s 25.

I**: 25**

R: and I think 28

I**: 28**

R: That’s it.

I**: Now, as for question 2. Can you tell me about this community? to make the question easier, can you tell me the positive things about this community? what you see? like, oh Majuro is good, why? Because it’s this and that….. don’t worry! It’s nothing, it’s like we are brainstorming, any answer that you know is okay. Now, the positive things, the positive things about Majuro, why is Majuro good? Why the community is good? The question is about the positive things in this community and the negative things in this community. Do you understand?**

R: Yes, yes I understand

I**: Don’t rush, this is our time, just take your time and really think about the positive things about Majuro, why Majuro is good? The question is asking about what’s good about Majuro.**

R: It is good because, it has less chores to do.

I**: less chores to do, okay. What else?...you mentioned, less chores, can you explained more about that**?

R: It is good because, we go to sleep, then wake up and eat

I**: mmm, what else? At first you said it is good because it has less chores to do, is there anything else you can think of about the community?**

R: It’s good, because there are lots of people.

I**: lots of people, okay, what else can you think of?**

R: It is good, because it has many different kind of foods.

I**: mmm, different kinds of foods. Now what kinds of foods you see here in Majuro?**

R: Rice, flour

I: **mmm…**

R: many kinds of meat

I**: mmm.. Now, all of these foods are like imported foods,**

R: mmm..
I**: now, as you mentioned that there are lots of foods here in Majuro, do you think there are also many foods that are planted here in the Marshall Islands?**

R: no, not many…

I**: not many from…**

R: not many from other countries.

I**: okay good. Now the other question is about the negative things in a community.**

R: the negatives are, if we don’t have a job, there will be no money to buy foods.

I: **okay**

R: no foods for the kids

I: **there won’t be enough for the kid to do what?**

R: to eat or to see a doctor when they are sick

I: **for the kids, okay. Now we will talk about health and illnesses in your family. Can you tell me what illnesses your child usually suffered from?**

R: usually high temperature or fever.

I**: fever, okay. What causes your child to have fever? how come your child have fever? why is your child usually gets sick?**

R: well sometimes, I’m asleep and I don’t change the baby’s diaper, that’s when the baby gets gold and have fever.

I**: cold and fever, okay. You’re doing a fantastic job in answering the question. Now, what are some seriousness of the illness, I mean for fever?**

R: when the child is having fever inside the body.

I**: fever inside the body, having fever inside the body is the seriousness. Now fever is the only illness that your child usually gets, are there any other illnesses your child gets?**

R: no, I don’t think so.

I**: no…. okay... So, are there any ways that you can prevent your child from getting fever, what would you do to make the fever vanished or disappeared?... what are some ways you can do so that your child can be healed...? okay? do you understand?**

R: what are some ways?

I: **yeah, there are some, I mean what are some ways, like, you can say, oh I’m doing this and that so that the fever can be stopped.**

R: and never have fever again.

I: **yeah and never have fever again…**

R: Drink medicine

I**: medicine…. give medicine, what else? are there any other ways aside from giving medicine to the child to drink.**

R: protect the child.

I**: mmm, protect the child. That’s great! Now, can you please explain how you know when your child needs to see a doctor? What gives you the idea like oh, my child needs to see a doctor. Why do you need to see the doctor? What makes you want to see a doctor?**

R: because the child is sick.

I**: sick, when you know that the child is sick, now, who will be the first person you goes to when your child is sick and why?**

R: And,

I**: and why? Why do you need to bring the child, I mean, who will your child see first?**

R: The nurses.

I**: The nurses, why do you need to bring your child to the nurses? Why do you trust them? it’s okay I know it’s really**

R: a hard question

I**: no, it’s nothing, the questions are not that hard, they are too easy, beside there’s no right or wrong answer. Okay. We’ll go for the next question. So, do you use local medicine when your child is sick?**

R: No, I don’t.

I**: you don’t use local medicine. Okay! Now can you describe any illnesses affecting your child that are associated with nutrition?**

R: I don’t know.

I**: you don’t know. Okay, don’t worry…. like I’ve said before, don’t worry if you don’t know the question or the answer. Now we have talked about being unhealthy for kids. Can you describe a day of a healthy person’s lifestyle, from the time they wake up in the morning until when they go to bed? Can you explain, for example, your child is a very healthy child, and what does he do from the time he woke up until the time he goes to bed?**

R: Playing….

I**: Playing, are there anything else he does aside from playing, when he is living a healthy life? Playing is all you could think of?**

R: Yeah!

I**: Good. Now let’s discuss about hand washing. Can you describe how your family washed their hand throughout the day? Does the children wash their hands throughout the day? For example, the children in your house.**

R: Yeah! Sometimes they washed their hands just before they eat breakfast.

I: **Before they eat breakfast**

R: lunch….

I: **lunch….**

R: dinner.

I**: and dinner. For the kids, in your experienced, can you discuss, children under the age of 2, whether they wash their hands throughout the day?**

R: Yes.

I: **Yes...you said yes, so do you think the children in this community wash their hands?**

R: oh, well, some children don’t wash their hands.

I**: some do wash their hand, but some don’t. Now as for the children that are not washing their hands, how come they don’t wash their hands?**

R: don’t teach them.

I**: they don’t teach them, lack of parenting. Okay…Now can you tell me what times during the day when soaped is used to wash hands?**

R: I’m sorry, but I think I have answered that question, so, in the morning, afternoon, and night.

I**: So, can you tell me the difference between using only water to wash you hands and using soap and water to wash your hand, what’s the difference?**

R: come again?

I**: are there any difference when you washed your hand by using water only, and when you washed your hand using water and soap?**

R: yeah.

I: **okay, can you tell me the differences?**

R**:** if we washed our hands by water only**,**

I: **Yeah?**

R: the dirt won’t vanish or the germs

I**: mmm….**

R: but if we used soap, then the dirt and germs will be gone.

I**: You are doing a great job. Now for the next question, what are some things that prevents a person from washing his or her hands. For example, if it was you, what prevents you from washing your hands? Or the kids, what prevents them from washing their hands sometimes?**

R: I don’t know. [laughing]

I**: don’t worry…. don’t worry, there are other question. Now we will talk about the foods you usually eat when you were pregnant. Now I want you to think back to when you were pregnant, can you tell me what kinds of foods you usually eat when you were pregnant and now that you’re not?**

R: I usually eat IU…

I**: You always eat coconut meat when you’re pregnant. So, what really makes you want to eat coconut meat when you were pregnant?**

R: Well that’s what I craved the most when I’m pregnant.

I: **when you’re pregnant that’s what you want to eat. [laughing]..that’s good, you’re doing great! Now, what kinds of foods you were told to eat during your pregnancy? And why?**

R: what kinds of foods?

I**: Yeah….**

R: IU, pandanus, local foods such as banana,

I**: mmm….**

R: Yeah.

I**: So, for the IU, what do you do with it?**

R: I make lukwor (combination of IU, milk, water and sugar)

I: **you make lukwor. Now, what about the pandanus. What do you do with it?**

R: I cooked it.

I: boiled the pandanus, what about the banana, any kinds of banana?

R: Boiled……

I**: boiled the banana**…

R: yeah!

I**: Good, now what kinds of foods should you not, you shouldn’t want, no, what kinds of foods you shouldn’t like for example, your family don’t want you to eat during your pregnancy and why?**

R: oh, foods that are salty

I**: salty foods…**

R: Kool aid…

I**: mmm...**

R: cholesterol foods.

I**: mmm, foods that contain cholesterol.**

R: I think that’s it.

I**: mmm, and now why did they tell you not to eat these kinds of foods?**

R: they said that it will harm the baby.

I**: mmm, now who told you or don’t want you to eat these foods during your pregnancy?**

R: my husband.

I**: your husband? Okay…and now who took care or supported you during your pregnancy?**

R: my husband, my aunty and uncle

I**: Aunties and uncles and your husband…now, how did they helped or supported you during your pregnancy. What are, what are some things that they lend a hand and help? Is it okay? Do you understand? [laughing]**

R: they usually give me money.

I: **mmm…. money**.

R: foods…

I: **foods...**

R: stuff like that...

I**: mmm…. now can you tell me about any medicine or supplements you took during your pregnancy?**

R: medicine?

I: **medicine. What kinds of medicine they give to you to take during your pregnancy?**

R: they gave me medicine for the blood

I: **blood...**

R: stuff like that?

I: **mmm…**

R: and vitamins

I: **the vitamins**

R: there are only two kinds….

I**: mmm…. did you take all the supplements that were given to you?**

R: Yes!

I: **And why did you take or did not take them?**

R: mmm?

I: **you took them**.

R: mmm!

I: **Now did you drink alcohol or smoke or use any of these?**

R: I didn’t…

I: **You didn’t…now did you use or take any local treatments during your pregnancy and why?**

R: I think, none!

I**: Now, if there’s anyone who advised you to eat more fruit, what makes it so difficult for you to eat it?**

R: I don’t want to.

I: **you don’t want to, okay. Now can you tell me what kinds of foods you eat during breastfeeding?**

R: Fish.

I**: Fish…...any kinds. Any kinds of food you eat during breastfeeding.**

R: fish, chicken, corn beef….

I**: mmm, now what makes you really want to eat these kinds of food during breastfeeding**?

R: I eat them so that there can be breastmilk.

I: **you ate them so that breastmilk can be produced…now, what kinds of foods you were encouraged to eat during…. oh sorry, we’ve already discussed this……what kinds of food you were encouraged not to eat during breastfeeding?**

R: foods that are salty.

I: **salty foods…what else?**

R: I think, that’s all...

I**: mmm, now who gives you advice or words of advice for you not to eat these kinds of food during breastfeeding?**

R: my aunty…

I: **your aunty. Okay…. now, after that we’ll go down to question 12, it says, after giving birth, can you tell me how you breastfed your baby throughout the day? is it okay? Do you understand**?

R: it’s difficult because I only lay on my back facing upward.

I: **lay upward...**

R: yeah because I had C-section…

I**: so that is why it’s difficult because you had a C-section. Now it says, after giving birth, how long it takes for you to start breastfeeding? And why? Is the question okay? To make it easier, how often did you breastfeed?**

R: frequently, because not enough breastmilk.

I: **mmm….**

R: because I haven’t eaten…

I**: so, you don’t eat?**

R: I don’t, I only drink water.

I: **mmm….**

R: the doctor gave me water only…

I**: mmm... now for the next question, it says, did you give a bottle of milk or any kinds of liquid to the baby at first after giving birth?**

R: I didn’t, because the doctor told me not to.

I: **because the doctor told you so, were there anything that makes it difficult or easy to breastfeed exclusively up to six months?**

R: like what?

I**: were there any difficulties or was it easy for you to breastfed exclusively up to six months?**

R: it was easy…

I**: easy, you think it was easy. Now this question says, can you tell me when did you first give foods and liquids other than breastmilk to your child?**

R: when the child is 7 months.

I: **7 months. Why did you start giving these foods or liquids to your child other than breastfeeding or breastmilk?**

R: because when I eat or drink, the child wants to, too.

I**: wants to eat...**

R: it’s like I can feel that he wants to eat and drink.

I: **Great, that’s great! Now, what are some opinions from other to first introduced foods or liquids to the baby, some liquids to the baby at that age, just what are their thoughts?**

R: at that age?

I**: yeah…**

R: they said it’s time for them to eat.

I**: mmm.... some said it’s time for them to eat, what else?**

R: some said, it’s not yet time…

I: **mmm.... good, now it says, what the first foods were and how they were prepared for the child?**

R: Baby food....

I**: the baby food, what about liquids?**

R: Water…

I: **now for the baby food, how did you prepared the food for the baby, do you prepare it from local foods or?**

R: I buy from the store.

I: **from the store, what about the liquids? What kinds of liquid?**

R: I also buy it from the store.

I**: Coffee?**

R: no, I also buy it…water…

I**: oh water! Sorry. Also buy it from the store. Good, you’re doing a really good job. Your answers are beautiful. Okay, now we are in question 14, don’t worry, we are almost done. Now it says, we are trying to understand how people in this community eat. Could you describe in detail what your family usually eats and drinks throughout the day?**

R: they usually eat bread…

I**: okay…**

R: rice…

I**: mmm…**

R: chicken…

I: **mmm**…

R: fish…

I: **fish?...**

R: sashimi…

I: **mmm…now who in your family needs, sorry, can you explain how they prepared the foods? For example, your family, how do you guys prepare the foods? Okay? Do you understand?**

R: how can…

I: **how do…for example you… how do you prepared the foods? What do you do so the foods can be prepared? All good???.... [laughing]**

R: it’s hard for me to answer it.

I**: it’s hard? Okay…no worries…it says can you explain. Oh…who in your family should be served first, and who are next.**

R: the children goes first.

I: **the children and then…**

R: and then the adult

I**: the adults are next…okay. Now are there any differences in the foods served to a different family member? For example, you are the one doing the cooking, are there and differences in the foods you served to the other family member?**

R: mmm... no, I don’t think so…

I**: none. Only one. Okay. Are there any children received more food than the others?**

R: yes.

I: **can you explain more why some children received more food than the others?**

R: they’re not full yet…

I: **they are not full. Okay, that really answer my question. [laughing] Now could describe any food sharing between family members during mealtime( for example children eating together separately from the family members, or meals eaten from the same plate by all family members?**

R: from only one plate?

I: **mmm?**

R: every one has their own plate.

I**: oh, so the kids have their own plate so does the adult…mm. good, good answer. Now does the family share meals with their neighbor? (for example, you, do you give foods to your neighbor)**

R: mmm….

I**: yeah, you do share foods with your neighbor…Now we have heard that some family eat local foods but some eat imported foods, can you tell me what foods your family usually have or eat?**

R: they usually eat IU, when I cooked IU, they ate it…

I: **mmm…. okay.**

R: breadfruit.

I: **breadfruit**…

R: but most of all, rice…

I: **most of all, rice, okay. What makes it difficult or easy to cook local foods? (for example, what make it difficult, what’s so hard for you to cook local foods?**

R: oh, sometimes there’s none...

I: **sometimes, there’s none, what else? now it says, what makes it difficult or what makes it easy for you to cook local foods?**

R: I don’t know…

I: **okay, don’t worry if you don’t know. Now for the next question, it says, what are some positive or negative things about local food? For the positive things about local foods, can you tell me why local foods are good?**

R: they’re good or delicious…

I: **delicious…good. Only delicious you can think of. What about the negative things about local foods?**

R: I don’t know about the negative things[laughing]

I: **don’t worry if you don’t know[laughing]…now it says, now we’ve talked about how your family eats, and now I want to know how your child eat, could you describe in detail what your son or daughter under 2 years commonly eats throughout the day? what you commonly gives to your child to eat throughout the day? is it okay?[laughing] anything. Anything that you know and understand because it’s your child, and you usually feed your child every day.**

R: everyday?

I: **yeah.**

R: in the morning, lunch and dinner.

I: **right!**

R: eat bread…

I**: bread. It can also be between hours, ...**

R: okay…. yeah!

I**: bread, you only give bread. Okay. Now, how many times a day your child eats, snacks too?**

R: usually 5 times…

I**: 5 times…**

R: 5

I: **good, now how do you know that the child has had enough to eat?**

R: the child is full…

I: **full…okay, good. What can you do when the child doesn’t want to eat? (for example, if your child doesn’t want to eat, what can you do so the child may want to eat?)**

R: I don’t know.

I**: you don’t know…okay, don’t worry if you don’t know, now what can you…are there any differences of how you feed your child when your child sick and when your child is not?**

R: one more time…

I: **are there any differences of how you feed your child, when the child is sick and when the child is not?**

R: yeah.

I**: can you tell me the differences?**

R: when the child is sick, he doesn’t want to eat.

I: **okay… he doesn’t want to eat.**

R: but when he is not sick, he eats a lot…

I**: okay, when he is sick, he eats a lot…great! Your answers are great! You’ve told me what your child under 2 usually eats. Now could you explain to me the process, from the start to finish, how you prepare and cook a meal for your child? How you… (for example, your child, how do you prepared or cook your child’s meal, what should you do so that it can be done?**

R: cook it and…

I: **well as for the meal, what kinds of food you should cook so that the child can eat…you just cooked anything?**

R: oh, pancake.

I: **pancake….**

R: pancake…

I**: pancake, what else?**

R: rice…

I**: rice…so you just cook the rice? or can you tell me….**

R: soup…

I**: soup…okay…soup! So, in the soup do you put…...what are the things that you put in a soup?**

R: cabbage, carrots…

I: **Good..**

R: vegetables…

I**: mmm…can you tell what kinds of food that are important for your child to grow well or be healthy? What kinds of food that you know that your child should eat to grow well?**

R**:** fish...

I: **fish…**

R: I don’t know, I usually fed him fish...

I**: fish, you usually feed your child fish…. now, in your own knowledge, what kinds of food you should not give to your child, because it’s bad for their health? This question, it’s says, foods that you’re like oh I should not give it to the child because it’s not healthy.**

R: Who?

I: **[laughing] don’t worry, don’t really….**

R: the chips…

I: **oh, chips and what else?**

R: mmm... chips…

I**: chips, okay. Chip is the only thing you can think of. What is the biggest influence on feeding children?**

R: I don’t know…

I: **you don’t know, okay. Don’t worry, you don’t know. Can you tell me any differences between how you feed your male child and how you feed your female child? Are there any differences?**

R: none

I: **no differences. Now, we are also interested in the roles and responsibilities different family members play in raising children. Could you describe the care of children throughout the day in your community**?

R: they watch over them…

I**: watch over them…how do they watch over them…can you really describe how they watch over them?**

R: they protect them from any harms.

I**: mmm. Now that you mentioned protecting them, who is mainly responsible for the child care?**

R: the adults…

I: **adults…what are the responsibilities of a mother for her child?**

R: protect the child

I**: protect the child, good. What are the responsibilities of a father for a child? For example, the father…**

R: prepare the meal.

I**: Prepare the child’s meal…...what else?**

R: have a job so that there’s

I: **there’s…**

R: money to buy food…...

I**: mmm. Now how does the caregiver play with children under 2?**

R: us, the women or…

I: **it can be anyone, the ones that are taking care of the children. How do they play with the children**?

R: usually play with them...

I**: okay good…play with them…. Now could you tell me the role of a grandparents have in raising children in this community?**

R: I don’t know…

I**: you don’t know…okay... in what ways that grandparents support in raising children, and also support the mothers of the children… what are the things that they do?..............it’s okay, don’t worry about the answer that you don’t know, because there are lots of people…lots of question. Now it says, can you tell me the role of other family members have in raising children in this community? for example, your family, anyone in your community, what do you think of how they raise a child or children, or their family’s children?**

R: I don’t know…

I**: you also don’t know…it’s okay…. you’re doing a great job by answering. And your answers are very helpful. Don’t worry about the answers that you don’t know but we are almost done. So, in this last section, we would like to learn about ways we can develop health programs in your community. can you explain where you usually get information about nutrient and health? It says… sorry…the question is, can you tell me where you usually get information about nutrient food or healthy water, so we can give to the children in the community, where do you get you information from, you usually get information** from?

R: Hospital...

I: **hospital from who? The doctors or….**

R: the doctors…

I**: now, it says, why do you trust the information that you get from the hospital? you don’t know, it’s okay. Now where do the information…from your own knowledge where do the information about the health of a child or mother should be delivered to, so that everyone in the community should hear, like frequently.**

R: what?

I**: it says, where should the information delivered to so that it can be easy for you to hear and see everyday.**

R: V7AB.

I**: V7AB, the radio…good... now what kinds of media you usually use the most to communicate?**

R: cellphones…

I: **cellphones…. mmm. now when you think about your own parenting behaviours, can you explain what influences how you raise your children, do you think there’s difference like through out the day you take care of your own child, are there…. could you explain it…as a mother?**

R: yeah, it’s very exhausting...

I: **exhausting…. anything else? what makes it exhaust?**

R: the child...

I: **the child…okay. Now, the opinions of the people in the community of how they raise their children(for example, leaders, neighbors, church leader, and health workers) are there any advice or information you received related to parenting?**

R: I don’t know…

I: **you don’t know…don’t worry. Now, where or who gives you these information, like, how you raise your child, where or who usually gives you this information?.........it’s okay, [laughing]don’t, don’t really…no don’t cry [laughing].... it’s all good.**

R: really in deep thought...

I: **Now, are there any kinds of information on parenting you like to know about, but you don’t have with you, are there information on parenting like, taking care of this child like this or that you wish to know but you don’t have.**

R: if we don’t have?

I: **mmm?**

R: about me or others?

I**: like you or me...**

R: no, none.

I**: none…. okay. are there any topics you would like to know that we missed to talk about?**

R: no……

I: **no…. [laughing] okay…we are done now, thank you very much even though there were some difficult question you didn’t answer but don’t worry, there are no right or wrong answer, the information you gave to me were good, thank you very much.**
