# Supplementary material for: Conceptualizing multi-level determinants of infant and young child nutrition in the Republic of Marshall Islands–a socio-ecological perspective
Source: PLOS Glob Public Health. 2022 Dec 19;2(12):e0001343. doi: 10.1371/journal.pgph.0001343 (PMC10022247; doi:10.1371/journal.pgph.0001343)
Supplement: S1 Data — (ZIP) [file pgph.0001343.s001.zip › RMI Supp Data/Interviews data/I05U_IDI_FCG_Rita_Aug 13_Meia.docx]

- Interview Code: I05U
- Interview type and interviewee: IDI FCG
- Interview Date: Aug.13.18
- Location: Rita
- Interviewer: Meia
- Transcriber: Marcellina

**I: if it is okay with you we can get paid… oh no not get paid but if you agree to be recorded then you will answer yes or no.**

R: hmmm

**I: okay now… thank you for giving me this wonderful time. Those information we’re going to learn from it or from you will help us find better ways to improve the health life of every mother and children and also the neatness of our islands. Now question number one, can you tell me about your family….. Like, who lives in your household?**

R: the family I am living with which is my husband’s… his mother and his siblings and his auntie and his uncles and their kids.

**I: now, how many children in the household?**

R: three

**I: three kids… ages… how old are they?**

R: I am speaking of five years old, one year old, and one that haven’t have his/her birthday party yet

**I: ooohhh… how many girls or boys?**

R: one girl two boys

**I: good… now can you… question number can you tell me about this community you live within?**

R: what I see… like what people do… oohh…..

**I: like what are your thoughts about this community you are living in**

R: good… this community is good but when I see children playing near the road, playing in the trash, but I don’t see their parents with them, it’s not good with me because the vehicles might hit them or bad causes might happen.

**I: okay [02:54]… now let’s move down… we will now talk about health and the illnesses in the family. It can be your family or other family you know about. Can you tell me what illnesses that your children suffer from?**

R: diarrhea…

**I: diarrhea…**

R: dizziness, je-metak (I don’t know what kind of illness is this. It’s my first time see and hear it☺), and fever…

**I: fever, and je-metak right?**

R: yes and also coughing

**I: and coughing**

R: yes

**I: okay... So you mentioned diarrhea, what are the causes**

R: foods we eat

**I: those foods we eat like…**

R: we don’t store them properly

**I: hmm hmm**

R: we don’t boil them again sometimes but we eat them right away but they are remaining foods from last night

**I: good**

R: dirty hands, when we don’t wash our hands. Sometimes we forgot to wash our hands

**I: so now u mentioned washing hands? Washing hands… how do we wash our hands? How would you preferred us to wash our hands?**

R: wash our hand! And or maybe hand-sanitizer

**I: wash our hands with?**

R: soap

**I: now… can you tell me is diarrhea a serious illness?**

R: yes because we suffer from. And when we have je-metak, it causes our skin to dry which is why sometimes we need I-V

**I: okay… how can we prevent diarrhea?**

R: look after our foods, re-boiled them, wash our hands

**I: wash our hands…. What else?**

R: clean the area where we store our food so that flies won’t land on them

**I: okay let’s move down to question number four… can you describe how do know when your child needs treatment?**

R: it shows on the yellow card… you are talking about?

**I: about that time your child is sick**

R: sick… when my child is starting to have fever I will take him/her immediately… like when I do multiple check up on his/her fever from morning until night…

**I: hmm hmm**

R: and I see and know that she/he still has the fever, I will rush to the hospital

**I: I am sorry we will go back to…. Because I want to know about… these…. I just skipped them….Because you told me about… in question number three… you told me about diarrhea, dizziness, fever, stomach pain, and coughing. Now, as for the dizziness… what cause your child to have dizziness?**

R: when my children stay under the sun, and when they have late breakfast

**I: hmm hmm**

R: then they will have dizziness

**I: what about…**

R: don’t have enough time to sleep

**I: don’t have enough time to sleep…**

R: like wake at dawn

**I: now about fever, I mean dizziness… is it a serious illness?**

R: not really but… we won’t feel alright when our kids are sick

**I: yes.. For the mothers…. What do you do to prevent this illness?**

R: I put them to sleep,

**I: hmm hmm**

R: I wait until it’s not really sunny outside and then let them play outside

**I: hmm hmm**

R: give their meals on time… even though we’re really sleepy but we wake up and feed them

**I: hmm… what about the fever?... what are the causes of fever?**

R: when they have their showers at night,

**I: shower…**

R: when they cool themselves a lot, and when they using fan a lot

**I: hmm hmmm**

R: then they will have fever

**I: now as they…. Do you think fever is a serious illness for?.......**

R: kids?

**I: yes**

R: yes of course..

**I: can you describe the seriousness of fever?**

R: when they have fever, sometimes when their fever temperature is high then they will have to stay in the hospital, they have difficulty in breathing…..

**I: difficulty in breathing….**

R: difficulty in breathing, sometime they give us I-V… my daughter they gave her I-V… when she had fever they gave her I-V also… she is weak when she encounter illnesses

**I: ohh… to prevent fever… what are the ways to prevent fever?**

R: I don’t put my kids in shower at night anymore, don’t let them use fan…..

**I: stomach pain…. What are the causes for stomach pain?**

R: when they are hungry, as I said…. When they are hungry they have dizziness, stomach pain,

**I: hmmm hmmm**

R: also the food they eat, make them have stomach pain

**I: hmm... is stomach pain a serious illness?**

R: as for me yes… every other illnesses make us uncomfortable when our kids have them.

**I: hmmm**

R: because when have stomach pain, they vomit. We use a lot of works when they are sick.

**I: okay, to prevent this illness… how do you prevent this illness?**

R: I let them eat properly and regularly…

**I: hmm hmm**

R: I check the food they make before I get them the food. I checked them if they are still good to eat or not.

**I: are there any food or anything besides food you give to them….**

R: when they have stomach pain?... yes! Fruit…..

**I: hmm hmm**

R: we feed them a lot of fruits first so that when their body gets stronger then they can now eat different kinds of food.

**I: now can you… fruit, you mentioned fruits… What kind of fruits?**

R: apple, orange…. I always feed them apples and oranges

**I: apples and oranges…**

R: hmm

**I: now let’s talk about coughing… what are the causes of coughing?**

R: they get it from other people

**I: hmm hmmm**

R: they use fan a lot

**I: hmm hmmm**

R: they sometimes get it from drink a lot medicines, they also get from the people in the house that are coughing

**I: hmm hmmm… now for coughing… is it a serious illness?**

R: for the kids yeah… because they get sore throat from coughing. And they don’t eat very well.

**I: what are the ways to prevent coughing?**

R: prevent them from coughing?..... Don’t let them use the fans a lot…

**I: hmm hmmm**

R: don’t let them get any closer to those who are coughing…

**I: is this all?**

R: hmm hmmm

**I: because we went back to question number three right?**

R: hmm hmm

**I: we already went through question number four so we will move on to question number five**

R: hmm hmmm

**I: so now… can you describe any illnesses affecting your children that are associated with nutrition?**

R: diarrhea…

**I: diarrhea….**

R: nausea, stomach… that’s all

**I: that’s all?**

R: yeah I’m speaking of my daughter because when she eats food that are lack of vitamins she will feel nausea and have stomach…

**I: now move on to number six… we talked a lot being unhealthy. Could you now describe for me a typical day of someone living a healthy lifestyle, from the time they wake up in the morning until when they go to bed?**

R: huh?

**I: this question is asking if you could describe the ways of a person with healthy lifestyle, from the time they wake up until they go to sleep.**

R: like what shows that they are living a healthy life?

**I: yes…**

R: they are energetic, they eat frequently, and they always happy

**I: so you mention energetic… can you tell more about it?**

R: like when they are energetic, they work a lot instead of just sitting around doing nothing.

**I: now what are the signs of a healthy child under two years old?**

R: eat a lot, energetic, always play sports… like for example, my son.

**I: hmm hmm**

R: for me to tell that he is healthy, when he wakes up every morning he will go outside and call out whoever and go chat with them… but when he is sick, he will just lay in bed the whole day! Also when he is not sick, he eats a lot and eats his food on time.

**I: okay…. Now what are the signs of a healthy adult?**

R: an adult?.... energetic, eat a lot like having their first second and last meals everyday

**I: now we will move on to question number seven. Let’s now discuss hand washing. Could you describe in detail your family’s hand washing throughout the day?**

R: okay… my kids for example… when I’m with them, before they have their meals they wash their hands. But I let them use hand sanitizer usually because of different kinds of illnesses. And also because the Ministry of Health have told us to wash their hands frequently. But, at all time before they have their meals they wash their hand with soap. And when they eat their candies they use hand sanitizer.

**I: now what time during the day your family use soap to wash their hands? Are there times your family wash….**

R: wash our hands with soap?

**I: hmm hmm**

R: yes.. After we done cleaning, before and after we eat, and when our hands our dirty

**I: now can you tell me the differences between using water only and using water with soap to wash our hands?**

R: using soap with water is better than just water only because wash our hands with soap kill germs.

**I: now… what prevent you from washing your hands with soap throughout the day?**

R: wipes and hand sanitizer

**I: we would like to talk about your diet during pregnancy and breastfeeding. Now would you think back to when you were pregnant. Can you describe your diet when you were pregnant compared to when you were not pregnant?**

R: okay… when I was pregnant, I usually eat sashimi, and sweets.

**I: sashimi and sweets?**

R: yes…

**I: what about the time you are not pregnant?**

R: well I eat whatever I like!

**I: hmm… now, what made you want or hunger for these kind of foods? Like the sashimi and sweet?**

R: the reason why I wanted to eat sashimi is because it produces blood and I wanted to have enough blood because I didn’t want to take the bills the doctors suggested me to take.

**I: oohh.. Hmm hmm… what about the other one? The sweets?**

R: sweets? Oh that is always what I want when I am pregnant!

**I: okay… now what kind of foods you were encourage to eat during pregnancy?**

R: fruits, local foods..

**I: fruits like?**

R: like apple, orange, and grape. I eat these but I don’t have them much as sashimi and sweets.

**I: now why did they recommended you to have these foods?**

R: because they said they are healthy for my baby.

**I: now.. What kind of foods you were encourage not to eat during pregnancy?**

R: sweets..

**I: why didn’t they want you to have sweets?**

R: because the baby will not have enough vitamins.

**I: who supported you when you were pregnant?**

R: when I was pregnant?... my husband, my mother in law and my mother.

**I: can you describe on how they supported you during your pregnancy?**

R: buy all my needs and what I wanted to eat, be with me when I’m sick, and always visited me to check up on me and my needs. Even though I didn’t want to be with them but they always buy my needs.

**I: now can you tell me about any bills you took during pregnancy?**

R: I didn’t take any

**I: okaaay…**

R: I only drank V8(juice) and ate sashimi.

**I: now why didn’t you take…**

R: because when I take the bills, I feel nausea and I don’t feel well.

**I: now.. did you drink alcohol, smoke and used other drugs during your pregnancy?**

R: I don’t do all of these…

**I: were there any traditional medicines you took during your pregnancy?**

R: no… but I did take some after I gave birth

**I: okay.. Were there anyone advised you to eat fruits and vegetables during your pregnancy?**

R: yes.. My husband… he always to tell me to eat the grapes and watermelon he bought for me.

**I: okay…now can you describe your diet when you were breastfeeding?**

R: I ate corn beef (can meat), sashimi, fish, sometimes steak and chicken. But I usually ate fish.

**I: now what really inspired you to eat these foods?**

R: breastmilk.. These food helps produce milk.

**I: now can you describe the food you were encouraged not to eat during pregnancy?**

R: sashimi

**I: sashimi.. Why?**

R: because they said my baby will most likely to bite my breast (nipples) when I breastfeed her/him..

**I: are there any other reasons?**

R: none.. That is only reason I know

**I: now can you describe the food you were encourage to eat during pregnancy?**

R: fish, corn beef because it helps produce breastmilk…

**I: now who encourage you to eat or not eat those kind of foods during breastfeeding?**

R: our mothers

**I: we will now move to child feeding practices. After giving birth, could you describe breastfeeding your child throughout the day?**

R: even though my baby is sleeping but I will wake him/her up to breastfeed her/him, and I breastfeed her/him frequently. I usually start breastfeeding him/her at dawn. I give him/her milk and when he/she goes to sleep, I will have my meal and then feed him/her again.

**I: how frequently you breastfeed your baby?**

R: like every after one hour.

**I: now.. After you gave birth, how long you started breastfeed your baby?**

R: I will breastfeed him/her until she/he goes to sleep…

**I: no.. Like after you gave birth…..**

R: like until they are mature enough?

**I: hmm hmm**

R: my older daughter for example, I breastfed her until she was three years old

**I: hmm… did you give her other liquid of bottle-feed her?**

R: I tried to bottle-feed her but she hated it

**I: hmmm… now we will move on to number thirteen. Can you tell me when did you first give foods or liquids other than breastmilk to your child?**

R: when my baby was four months old, I started bottle-feed him/her with water. And for foods, I started feed her/him food when she/he was six months old.

**I: now can you tell me the reasons why you gave foods or liquids at that age?**

R: as for me, I gave the water because I think that’s the time she needs it.

**I: now… do you know the opinions of other people about introduce foods and liquids at that age?**

R: I don’t know…

**I: now.. Can you tell me the first foods your child had?**

R: baby foods and pandanus juice

**I: so you mentioned baby food.. can you tell more about it?**

R: the ones that we bought from the stores

**I: ohhh… and you also mentioned pandanus juice, can you tell me more about it?**

R: we bought pandanus from the local foods market and we then boil it and grate the juice out of it..

**I: so you grate it and what do you mix it with?**

R: milk.. Sometimes water

**I: okay you are doing very well. Your information are really useful!... now we are trying to understand how people eat in this community. Could you describe in detail what your family usually eats and drinks throughout the day?**

R: the family I am living with we eat bread, dough nut, and ramen for breakfast. We drinks Kool-Aid, coffee, tea, and water.

**I: homemade bread or the ones bought from the store?**

R: one bought from the store… sometimes we bake pancake.. And for our lunch we usually eat can meats because it’s too hot in the noon to cook food. Like tuna with aji-kimichi, luncheon meat with eggs, corn beef with corn. And for dinner we cook the frozen meats like chicken and steak.

**I: in your lunch meal, what do you have with the can meats?**

R: rice.. and for my mothers, they sometimes eat can meats with local foods like banana(the ones you boil it), and breadfruits. They don’t usually eat rice. They also eat bread with the can meats.

**I: and you have frozen meat for dinner right?**

R: yes

**I: frozen meat with what?**

R: rice

**I: now.. who in your family is serve first, next, and last?**

R: we prioritize our elders and our kids so we serve them first.

**I: and who serve after?**

R: the couples..

**I: now, are there any differences in the foods served to different family members?**

R: no.. Everyone will have the same

**I: now.. Are there any differences in quantities of food served to different family member?**

R: yes… our elders won’t have much amount of food compare to others in the family because they don’t eat much.

**I: okay.. Now are there some children receive more food than others?**

R: yes there are. Some children eat a lot some don’t.

**I: now could you describe any food sharing between family members during mealtimes ( for example children eating together or separately from the family. Meals eaten from the same plate by all family members?**

R: the kids eat separately from their parents. Sometimes the kids share a plate with each other.

**I: ohh.. now are there any food sharing between household like for example food sharing with neighbours?**

R: yes.. Whenever we have feast we invite our neighbours to come eat with us.

**I: now.. We have heard that some families eat local foods but some eat processed foods. Could you explain what food your family usually eat?**

R: lukwoj (iu w/flour and sugar), breadfruit, fish, chicken, hotdog, ham, rice, and sashimi.

**I: what makes it easier or difficult for you to cook local foods?**

R: for difficulties, when there are no more local foods to cook, when we ran out of butane gas.

**I: now what are the good or the bad things about eating local foods?**

R: for the good things, they are good for our health. And for the bad things, they won’t stay for a long time.

**I: what about the processed foods? What are the good or the bad things about them?**

R: good thing is they last long. And the bad thing is they are not good for our health.

**I: we’re almost done. I know you are tired. Okay now let’s move to question number seventeen. Now that we’ve talked about how the family eats, I would like to learn more about how your child eats. Could you describe in detail what your son/daughter under two years commonly eat throughout the day?**

R: for breakfast, I feed my son cereal and bread. For lunch, I feed him tuna, chicken….. Also for dinner. For snacks, I feed him bread or biscuits.

**I: what water you give for the bread?**

R: Kool-Aid or tea

**I: and as for the cereal….**

R: I add milk to it

**I: and for the tuna and chicken?**

R: with rice of course!

**I: how many times a day you feed your child which is under two years?**

R: five or six plus the snacks

**I: how do you know that your child has enough foods?**

R: he just don’t want to eat further more

**I: what can you do to encourage your child to eat when she/he doesn’t want to eat?**

R: when he/she doesn’t want to eat?... when my son doesn’t want to eat, I give him other things like oranges and he will eat.

**I: are there any differences in feeding your child when he/she is sick?**

R: yes there are.. When he is sick he doesn’t eat much.

**I: and what do you do to encourage your child to eat when she/he refuses?**

R: I let him watch his favourite movie so he would get distracted while I feed him

**I: you’ve told me what your child under two usually eats. Now could you explain to me the process, from start to finish, how do you prepare and cook a meal for your child?**

R: prepare?.. Before I give him cereal, I wash first the bowl he’s going to use even though it’s already been washed. And when I cook him noodles, I let it cool before I feed him. I cover his meals after he is done.

**I: could you now tell me what you think are important foods for children under two years to grow well or be healthy?**

R: for my children I usually feed them cereal and fruits. But I don’t know if these are healthy or not for others..

**I: from what you think… what kinds of food you know that they are important and they need them for a healthy life?... anything…**

R: cereal and fruits…

**I: now what kinds of food that should not be given to children under two years?**

R: candies…

**I: candies.. Why candies should not be given?**

R: because they will lose their appetite and they will have rotten teeth

**I: now from what you know, what are the biggest influences on feeding your child?**

R: feed him foods that contain lots of vitamins, don’t feed him candies because he will lose his appetite, and protect his foods from flies.

**I: can you now describe any differences between how you feed your son and how you feed your daughter under two?**

R: if I feed my son ramen, I will also feed my daughter ramen. If I feed my son cereal, I will also feed my daughter cereal.. so there are no differences.

**I: good! We’re almost done…. We’re also interested in the roles and responsibilities that different family members play in raising children. Now this question is asking can you describe the care of children throughout the day in your community.**

R: I don’t really know about the care of other children but all I know is that I usually see them playing near the road without their parent’s guide.

**I: from you understanding, who is mainly responsible for child care?**

R: the mother… and the father

**I: now what are the responsibilities of mothers in child care?**

R: keep them safe.. Like always check up on them when they are playing.

**I: what about the fathers. What are their responsibilities?**

R: they also help their wife look after their kids and support.

**I: support.. Can you tell me more about it?**

R: for example, they support their families with their needs.

**I: can you explain in detail about the family’s needs?**

R: like.. Buying diapers, foods…….

**I: those are really useful information. And you are totally right, the father should help too. Now can you tell me how the caregivers play with children under two?**

R: caregivers?

**I: like the mother, the father, or whoever is taking care of the children..**

R: me for example as I am the one who’s looking after my child, the reason why I always buy my son toys is because I want him to play with toys inside the house and get distracted. And also stay near me.

**I: okay.. are there any other ways of playing with your kid?.... are there any other things you do?**

R: yes.. I play with him when I am not busy or not at work.

**I: okay… question number 22. Could you talk about the role of grandparents have in raising children in this community?**

R: my children’s grandparents for example. They usually check up on my children. When I sleep, they will look after my kids. They help.

**I: so you’re saying they help. Help in what ways?**

R: like when they are done with their works, they will take them and watch over them for me when I am in the shower or busy.

**I: ohh.. now this question is asking what makes the grandparents be a good grandparent?**

R: they come back home bringing something with them for the kids to make them happy.

**I: we have three more questions to go. Now can you talk about the role that other family members have in raising children in this community?.. Like when we talk about other family, we’re speaking of sisters, aunties and uncles. What are their roles in raising children in this community?**

R: look after them like always stay beside them, always take them with wherever they go and don’t abandon them.

**I: you are doing a great job. We’re almost finished. Now for the last section, we would like to learn about ways we can develop health programs in your community. Now could you explain where you usually get trusted information about nutrition and health?**

R: where do I usually get information from?

**I: yes..**

R: from the hospital, family members, our elders, and also from our self

**I: now, what are your reasons why these sources are trusted?**

R: for hospital, that’s the main place to get information about health and for the family members, I trust them. And for the elders, because they know a lot about what they did.

**I: now, where do you think nutrition and health messages should be delivered so that you would see or hear most easily?**

R: in the radio station like V7AB and other stations

**I: now what type of media you usually use to communicate?**

R: like cell phones?

**I: yes..**

R: well cell phone..

**I: for example like internet and other types of media…**

R: well when they don’t tell over the radio they send text messages, or they do survey, or the community leaders do activities in gatherings.

**I: wow that’s a lot of information girl! Now last question. When you think about your own parenting behaviours, can you explain the differences on how you raise your children?**

R: like the differences between my kids?

**I: yes..**

R: well the only difference is that one of them was really hard to take care of. My daughter, I really had hard time taking care of her. Like I won’t do a lot of work around the house because she just want to be with me.

**I: okay now is there any advice or information you receive or learn related to parenting?**

R: yes.. like they advise me really take good care of my kids, always take them with me wherever I go, something like that.

**I: good!**

R: they also advise me not to abuse them. There are times I get mad and I will just slap them or something. But won’t give them hard slap which will cause bruises.

**I: now what are the opinions of the people in your community about raising kids? For example the community leaders, church leaders, health workers. Like are there any advices they give to the mothers and fathers?**

R: “don’t get distracted while looking after your kids”. Because they said they might be at risk. I’ve heard them tell some mother this kind of advice.

**I: so when they said “don’t get distracted while looking after your kid”, what do they mean?**

R: because most mothers like to sit down and chat a lot while their kids are playing near the road.

**I: now, are there any desired information about parenting you wishes to have but are not available?**

R: maybe there are things I might not know about parenting and I wish to know.

**I: now is there anything else about the topics we talked about today that we missed or that you would like to tell us about?**

R: all of the questions you asked me are perfect. So I don’t think I have.

**I: thank you so much for your time and for the useful information you gave.**
