# Supplementary material for: Conceptualizing multi-level determinants of infant and young child nutrition in the Republic of Marshall Islands–a socio-ecological perspective
Source: PLOS Glob Public Health. 2022 Dec 19;2(12):e0001343. doi: 10.1371/journal.pgph.0001343 (PMC10022247; doi:10.1371/journal.pgph.0001343)
Supplement: S1 Data — (ZIP) [file pgph.0001343.s001.zip › RMI Supp Data/Interviews data/I06U_IDI_MCG_Rita_Aug 13_BM.docx]

- **Interview Code: I06U**
- **Interview type and interviewee: IDI MCG**
- **Interview Date: Aug. 13, 2018**
- **Interviewer: BM**
- **Transcriber: Showme Lelwoj**

***I. Hi before we start do I have consent that you on your own behalf want to share your information to us?***

R. Yes

**I. Okay thank you for this very beautiful timing for me to ask you the question in our interview today. Let’s begin, can you please tell me a little about your family.**

R. Okay in my household it’s just me, my wife and my 3 children.

**I. Good, how many children and how old are they?**

R. There are 2, 6 and 8 years of age and there are 1 girl and two boys. One is still on its way.

**I. Next I’d like you to describe to me about your community?**

R. Good things about this community is that there is peace here. There is

**I. Alright are there any bad things here in the community?**

R. I have not seen any bad things here in the community.

***I. Okay let’s move on to talking about health and illnesses in your family.***

**I. Could you tell me some of the illnesses that your children have suffered from?**

R. My eldest son has heart problems, the younger one have fevers and have stomach aches with headaches.

**I. What causes your children to have fevers?**

R. I can’t tell what the cause was.

**I. How serious is each illness to you?**

R. I have no information on how serious it will get when they have fevers.

**I. Are there any ways to prevent your child from having fever?**

R: monitoring their temperature like applying wet towels or fabrics on their body.

**I: so you also mentioned nausea, can you tell me what causes your children to feel nausea?**

R: sometimes all of the sudden they feel nausea especially when there is a flu going around.

**I: how serious is nausea?**

R: I don’t really know how serious is

**I: good.. So how do prevent nausea?**

R: we feed them the foods they want to eat like oranges. And drinks like Gatorade, orange juice, whatever they want to eat or drink. Also ice-cream

**I: you also mentioned headache. Can you tell what causes your children to have headache?**

R: I don’t know

**I: how serious is headache?**

R: I don’t know too

**I: how do you prevent headache?**

R: as for our older kids, they tell us when they have headache but for our youngest son he doesn’t because he doesn’t know what headache is.

**I: can you explain how do know when your child needs treatment for their illness?**

R: I don’t usually seek treatment for my kids because they don’t usually get serious illnesses. I only seek a doctor on their appointments.

**I: now if you child get sick, who do you first go to for healthcare?**

R: we take care of the kids ourselves when they are not really sick but if it’s really serious then we will go to the hospital.

**I: do you guys use traditional healers or traditional medicines when your child is sick?**

R: yes when they have issue in the stomach from falling several times then we use traditional medicines.

**I: now can you describe any illnesses affecting your child that are associated with nutrition?**

R: I don’t really have any information about any illnesses cause by foods that are lack of vitamins. But we usually feed our children foods that contains lots of vitamins like local foods. They don’t usually eat rice.

**I: what kinds of food that will make your child have an unhealthy life?**

R: I don’t know what kinds of food will lead to unhealthy life.

**I: what about any kinds of food that will make your child have a healthy life?**

R: local foods like breadfruits, fish, coconut drinks…

**I: from your own understanding, why did you pick breadfruits, banana and fish..**

R: because these are nutrition foods and they contain lots of vitamins.

**I: are there any illnesses caused by foods missing from the diet?**

R: I don’t really know what illnesses might be

**I: it’s all good! We talked a lot about being unhealthy. Could you now describe for me a typical day of someone living a healthy lifestyle, from the time they wake up in the morning until they go to bed?**

R: the important thing is they should have all the three meals in a day and drink water.

**I: good.. Now can you tell me what are the signs or appearances of a healthy child under 2 years?**

R: we can tell a healthy child by their looks, when they look lively. And they look lively because they had all their three meals.

**I: what are the sings or appearances of a healthy adult?**

R: for adults….. Sorry I don’t know.

**I: it’s all good! Now we would like to learn about the foods that are commonly available in your community. Can you talk about how your household gets food to eat on a daily basis?**

R: sometimes we sell breadfruits, banana, or other local foods so we can buy foods.

**I: good. So you said you sell your foods, how do you commonly use the profits of the food you sold?**

R: I use the profits to buy rice, flour, diaper, and save some for lunch money.

**I: what foods do you grow in your area?**

R: I grow breadfruit, pandanus, papaya and coconut tree as you can see.

**I: now can you tell me about any difficulties to growing food in your area?**

R: we don’t have any difficulties in growing food here in this house

**I: what your family would need to grow food at home?**

R: we don’t have the seeds to grow whatever and make a garden. But in this area there are lots of breadfruit tree so we can just grow another one by the stems of the breadfruit tree.

**I: so now could you now explain how easy or difficult it is to get those foods you mentioned every month during the year?**

R: as you know these foods have their own season. When it comes to breadfruit’s season there will plenty breadfruits and so do all the other local foods.

**I: throughout the year, what other foods that sometimes not enough and what are their main causes?**

R: there are some local foods die of drought… Yeah I think that’s the reason why some local food are not enough.

**I: now can you describe when there is a shortage of food, what do you do to feed your family?**

R: if there is a shortage of food…… I will go fishing and then maybe sell some of my catches so that we can buy other things we need. But I have a small family, so we don’t usually face that kind of situation.

**I: Now I would like to ask you about animals that you raise at home. Could you please tell me about the animals that you raise?**

R: I raise dogs, cats, chickens, and pigs

**I: can you tell me your reasons for raising these animals?**

R: as for the dogs and the cats, there for pets. And for the chickens and the pigs, I raised these because we can also have them for foods.

**I: now can you tell me the difficulties on raising these animals?**

R: I think there are none, because these animals roam around finding foods for themselves.

**I: Can you explain any difficulties to keeping animals in a fenced area?**

R: as for my dog, I tie him up because he’s wild and he is most likely to bite people whenever they approach our house. For the chickens, I put them inside the fence because there are guys who sneak out at night and steal chickens.

**I: can you tell me what do you do with the animal’s waste?**

R: nothing… they just decomposed

**I: There are some foods that we wish we could eat, but for some reason we cannot do so. Could you tell me about any foods you wish your family could eat (or eat more of) but cannot?**

R: you know those expensive meats like smoke ham, rib and other expensive meats that we don’t usually eat.

**I: can you tell me your reasons why you cannot eat these foods regularly?**

R: the reason why we cannot eat these foods is because we don’t have enough money in our pockets.

**I: good. Now for the last question on food, can you explain who decides what food to get for your family?**

R: me and my wife. But usually my wife is the one who decide what to get for our family.

**I: can tell me who decides which foods young children should eat?**

R: my wife

**I: okay in the next section, we would like to talk about water and hygiene. Can you please describe a typical day getting and storing water for your family?**

R: water tank. Right? We also have water bottles and that 5gallons water tank. What else?... I only that these are things we keep our water in it.

**I: what is the main source of water for drinking, for cooking, for washing, for bathing?**

R: we have water catchment for bathing and two water tanks for cooking and washing.

**I: what are the main difficulties in getting water?**

R: drought. If we encounter drought season, then it’ll be difficult for us to get water. but we buy us drinking water from the store when we really run out of water. we also use water from our water well for bathing and washing.

**I: now can you tell me the ways that your family tries to make drinking water safe?**

R: we usually boiled our drinking water. After we boiled our drinking water, we let it cool down before we put it in our refrigerator.

**I: good. Thank you for your awesome answer for the last question. Let’s now discuss hand washing. Could you describe in detail your family’s hand washing throughout the day?**

R: as you can see over there, we have sink for washing dishes and also our hands. Before we eat we wash our hands. And if it is a soft food, we used spoon.

**I: now can you tell me how does your children wash their hands throughout the day?**

R: before they eat, they wash their hands.

**I: yeah you are right about that, but can you tell me how they wash their hands?**

R: they wash their hands in the sink.

**I: how is hand washing throughout the day use for children under 2?**

R: we help them wash their hands by lifting them up, so they can wash their hands in the sink.

**I: good. Now can you tell me what times during the day when soap is used to wash hands?**

R: every time, because we already put dishwashing soap in the sink, so we just wash our hands in the sink which already has soap in it.

**I: okay now, can you tell me the differences between using water only or water and soap to wash hands?**

R: I am sorry I don’t really know the differences, but I usually buy hand sanitizer for my kids to use.

**I: You are doing a great job providing very detailed answers, thank you. Now, could you describe the type of toilet that you have at your home?**

R: we are using toilet bowl.

**I: could you tell me the reasons why did your family choose to use this type of toilet versus other types?**

R: because we see that most people are using toilet bowl, so we want to use it too.

**I: okay, so in some communities, we have heard that defecating in the open (such as on the beach) is common. Could you help us to understand this practice, including how common it is?**

R: most of our neighbors in the ocean side don’t have rest room so obviously they use the open area. For one reason why they don’t have restroom, is because the landowners don’t want them to dig up their ground to build rest rooms. According to our custom practices, we should ask the landowners before you build or do anything in the landowner’s community.

**I: so, can you tell me how young children’s stools are typically disposed?**

R: we usually let them use diapers and we dispose them in the trash cans which are taken to the garbage dump.

**I: good. Now could you explain where your young children usually play each day?**

R: they usually play around our house and our neighbor’s house.

**I: now, can you tell me of an ideal play area for children and your reasons why?**

R: from my everyday observation, I see that most children like to play in their yard where they feel comfortable playing at because it’s where they grew up in. they don’t feel secure playing in other neighbor’s yard because they are not attached to their environment.

**I: now, does the children play in areas where animals are kept?**

R: yes, they do. Most of the children in this village love to play in the areas where there are animals.

**I: can you tell me what are the challenges of keeping a child’s play area clean?**

R: when there is animal’s waste left in their playground we will shovel it immediately.

**I: good. To wrap up our questions on hygiene, could you explain ways to prevent the spread of disease?**

R: I am sorry I don’t really know the information about that question.

**I: you are doing great don’t worry. Okay, so we are also interested in the roles and responsibilities that different family members play in raising children. Could you describe the care of children throughout the day in your community?**

R: well all I know is that everybody takes care of their own children. Like us, we take care of our own children.

**I: now, can you tell me who is mainly responsible for the child or children’s safety?**

R: the mothers and the fathers.

**I: can you describe the responsibilities of the mothers for the child or children’s protection?**

R: they wash their clothes, wash their dishes, and especially take care of them. While us the father gets drunk.

**I: good. Now from your own understanding as a father, can you tell me the responsibilities of the fathers for their children?**

R: as a father, I help with my family’s needs. I will really try my hardest to get the needs for my family.

**I: now can you tell me** **how caregivers play with children under 2?**

R: my wife and I take care of our children and my older brother’s children when he and his wife go to work. So, we usually pay more attention to our brother’s kids since they are younger than our kids.

**I: good. Thank you. Now could you talk about the role of grandparents have in raising children in this community?**

R: well I don’t really know the roles of grandparents since my wife’s parents are living in Arno, and mine have passed away. But I really do believe that if they were here with their grandkids, they would have look after them better than we do since they know the skills of babysitting way better than we do.

**I: good. Thank you again for your useful information. Now could you talk about the role that other family members have in raising children in this community?**

R: there are some family members treat their kids awfully like torture them. Unlike my family, I don’t want my kids to be treated that way.

**I: can you please describe the ways that siblings (older siblings) help to raise young children?**

R: well I don’t really know about other siblings but like my own sisters and brothers, they don’t treat my kids very well so that’s why we’re (me&wife) looking after our own kids by ourselves. And we also teach our older kids to do the same to their younger siblings.

**I: you’ve given me valuable information thank you so much. We are almost finished. Now for the last section, we would like to learn about ways we can develop health programs in your community. Could you explain where you usually get trusted information about nutrition and health?**

R: I am sorry, but I don’t know where I would get trusted information but all I know is that if we don’t have this and that, I will climb a coconut tree or a breadfruit tree or whatever tree I have and see in my area which can help my family with nutrition and which are easier for me to get for my family.

**I: okay now could you tell me where nutrition and health messages should be delivered so that you would see/hear them most easily?**

R: I don’t know

**I: okay. So now can you tell me what types of media that you used the most to communicate? Like for example, the radio, the internet, or in the newspaper, etc.**

R: I usually get information from women’s club like WUTMI or another women’s club.

**I: good. Now we are on the last question. As you’ve already become a father, can you explain what really influence you on raising your children?**

R: as a father, I don’t want my children to be poor……………….. I am like those who shed tears easily…

**I: it’s alright…**

R: my children…. As someone who care so much about my children, I don’t want them to be poor.

**I: everything will be okay…**

R: it is something that will make me shed tears when I see my children frustrated or see them torture. Especially see them cry. I usually cry when see these kinds of conditions. As a father to my children, I don’t want them to encounter these kinds of conditions.

**I: thank you. Is there anything else about the topics we talked about today that we missed or that you would like to tell us about?**

R: I don’t think we missed anything but all I know is that I really don’t want my children to be mistreated, cry in front of me, or starving. And I will always try my best to help if these things happen to them.

**I: good we are done now. Thank you so much again for your generous time and for sharing your thoughts with us. What you said and the useful information you gave are very important. And don’t worry about whatever happened because everything that have happened here will stay in here. We greatly appreciate your help and we hope this research will help us improve the health of mothers and children in your community.**
