# Supplementary material for: Conceptualizing multi-level determinants of infant and young child nutrition in the Republic of Marshall Islands–a socio-ecological perspective
Source: PLOS Glob Public Health. 2022 Dec 19;2(12):e0001343. doi: 10.1371/journal.pgph.0001343 (PMC10022247; doi:10.1371/journal.pgph.0001343)
Supplement: S1 Data — (ZIP) [file pgph.0001343.s001.zip › RMI Supp Data/Interviews data/I07U_IDI_FCG_Rita_AUG 13_ Libon.docx]

**Interview Code: I07U**

**Interview type and interviewee: IDI FCG**

**Interview date: August13**

**Location: Rita**

**Transcriber: Shante**

**I: Ok. Before we start I would like to ask you if you are willing to do this survey with me or not?**

R: I will.

**I: Ok. Our first question is… First of all I would like to thank you for giving me this time for us to talk stories together. Like I said before that your information you will give we will learn about it and it will help find the better healthy life for the women and children and also for our homes. And to start, can you please talk a little bit about your family?**

R: on how I make them food or…?

**I: on how you…just about your family.**

R: ohh… Well on how I see it I don’t think there’s anything wrong with my family. On how I watch over them and, on how Me and my husband watch over them…everything is good. No harm.

**I: Who lives in your house?**

R: Just us.

**I: Is there any kids?**

R: Oh. Me and my husband and our three kids.

**I: Now how old are the kids?**

R: The child that we we’re interviewing about before is one something year old, and the one that is older than him, he’s our own child and he’s older like around 13 or 12… and our oldest daughter is maybe around the age of 30 or 20 some…

**I: All girls no boys?**

R: all the boys are here attending school.

**I: No how many girls and how many boys?**

R: 2 is our own and the girl is adopted, and if the boys they are three.

**I: Can you tell me about your community or the place you came from?**

R: its good. How we see it its good.

**I: What are the good things in this community?**

R: what are the goods in this community?... I think its good, I like how we’re by ourselves and we fed our animals and we do our own planting.

**I: The people in this community are they doing something together in any kinds of ways?**

R: Yes. they do.

**I: Like what? What do you guys get together about?**

R: For me, my younger sisters and I usually do stuffs together like cleaning and…

**I: What are the bad things in this community?**

R: The bad things in this community?... Well the bad things about it is we…. What?... what would I say?... I think I don’t know how to answer that…

**I: It may not be from inside your home but outside the area or in this community. What are the bad things you see from it?**

R: Oh. The bad in this area… Well the sides are always dirty, that’s the only think I know.

**I: Ok. What about the peoples?**

R: They clean it.

**I: They’re good?**

R: hhmm.

**I: Ok. We will now talk about healthy life and the illness of this family. Its say can you tell us about what kinds of illnesses does your children usually suffer from?**

R: Fever. They usually have fever.

**I: hmm…**

R: and sometimes diarrhea, and pink eyes but… they don’t usually have pink eyes.

**I: Ok. You mentioned fever. What makes them have fever?**

R: sometimes when they take a bath in the afternoon… and when sometimes I get busy and then bath them in the afternoon they will sometimes get fever.

**I: Is there anything else other then when you bath them?**

R: sometimes when they have a bump inside their stomachs they can get fever from that too.

**I: What are the affects of fever?**

R: sometimes they will be seizure.

**I: okay**

R: Sometimes they can, we their fever is high and you look back to them they can have seizure…

**I: Okay. So what are your ways of preventing their fevers?**

R: Sometimes we give them Medicines and if it doesn’t work we give them traditional medicines.

**I: Okay. Can you tell me how you can tell when your child needs to go see the doctor?**

R: If I dee that my child is really sick I will rush to the hospital and bring him there and if the medicines they give us don’t work on him then I will give him traditional medicines.

**I: Ok. Now that you say traditional medicines. What do you mean by that?**

R: From the coconut juice and the leave from (I don’t know what tree is called.) and then I make the child drink it.

**I: Is there any other way other then make the child drink?**

R: sometimes I (jione?)

**I: oh you (jione)… So who do you first bring the sick child too and why?**

R: If the child gets a seizure for instants then I bring him to the priest first. And when I see that’s he’s a little bit ok than I will bring him to the doctor.

**I: Ok. Do you use traditional medicines?**

R: For?

**I: When they are sick? Do you use traditional medicines when they are sick?**

R: Can’t you hear? I said when they have high fever then I will give them traditional medicines.

**I: Can you tell me about any illnesses affecting your children that are associated with nutrition? Is there any nutritious that affect them?**

R: I don’t think there is. They have a healthy life.

**I: What kind of food they eat that there is no nutrition in them?**

R: like rice…

**I: Like rice. What kind of illnesses will they have?**

R: Pink eyes. They will have pink eyes if they often eat rice.

**I: Is there other children that you see they get sick from the food they eat that don’t have nutrition in them?**

R: Say it again

**I: is there any other kinds of illness from the food the eat that has no nutrition in them?**

R: Yes. Sometimes they’re stomach gets big and sometimes they get skinny and have diarrhea and sometimes pink eyes. That’s it. Those are the only illnesses I see.

**I: Ok. We talked a lot about being unhealthy. Could you now tell me a typical day of someone living a healthy lifestyle, from the time they wake up in the morning until the go to bed?**

R: When he moves around and doesn’t want to do stuffs…

**I: hmm hmm… is there any other more that you see that the persons look’s healthy?**

R: He’s big (fat. Saying it in a good way) (saying something not understandable than laughs.) He looks healthy and not look sick.

**I: Now this question says, what are the signs of a healthy child under the age of 2?**

R: Eats good food.

**I: hmm.**

R: He will look healthy and feel healthy with clean surroundings,

**I: What about the adults? How do you know when they look healthy?**

R: there’s barely any adults that look healthy ahhaha. They grow and have diabetes. The food we eat destroy us, we use big amounts of sugar for our drinks. We barely drink (jakro) nowadays.

**I: Ok. Good. Let us now talk about washing our hands. Could you describe how this family wash their hands in a day?**

R: Sometimes we forget to wash our hands, sometimes I say why don’t you wash your hands but your eating?

**I: Now how do you wash your hands?**

R: We use soap. We just use soap. Soap and clean it.

**I: Good. Now the kids… So you mentioned soap… you use soap and…?**

R: just soap, I tell them come and wash your hands and use soap.

**I: so you say you use soap. What do you use other than soap because you know your hands will still be soapy…**

R: Water.

**I: Now do the children wash their hands everyday?**

R: Yes

**I: Now you said they wash their hands everyday. Can you describe how they wash their hands?**

R: they soap their hands and wash them.

**I: Now the children under two years. Do they wash their hands?**

R: No because the mother Wash her hands and then feeds her kids… Laughs

**I: Good. Now when is the important time of the day to wash your hands?**

R: Before we eat and after we use the bathroom. And we wash our face (giggles) and hands.

**I: is there other times you should wash your hands?**

R: Yes. Before we cook the foods and before we prepare the foods.

**I: What about the children? When they play?**

R: They can play and when they come home I immediately tell them to go wash their feet and hand before they come in the house. They know.

**I: Ok. Could you describe the differences between washing your hands with just water and washing your hands with soap and Water?**

R: using the soap is much cleaner. And just the water not really clean.

**I: You said not really clean. Could you explain that.**

R: I don’t use soap and the germs on my hands won’t go.

**I: Now what will prevent you from using soap in a day?**

R: What will prevent?... But I use soap everyday.

**I: What about…**

R: what about what?

**I: Now. When you are in a hurry to do something?**

R: I need to use soap first because I’m in a hurry and I need to do it first.

**I: Ok. Now we will talk bout the food that you usually eat when you were pregnant until the time you were breastfeeding. I want you to look back… (kid crying) to when you were pregnant. Can you tell me what kind of food you usually eat when you were pregnant?**

R: When I was pregnant I only wanted to eat the food that I wanted to eat. I eat fry fish, drink coconut juice and eat banana. Some food I eat but don’t want.

**I: What about the time when you weren’t pregnant?**

R: well every kind food I eat. Laughs.

**I: What was the reason for you to want to eat those food when you were pregnant?**

R: the child in my womb.

**I: what’s wrong with the child in your womb?**

R: its like my wants, it wants…I don’t know.

**I: What kinds of food they wanted you to eat during your pregnancy and why?**

R: maybe**…**

**I: foods that they advices you to eat or the doctor told you to eat.**

R: Well sometimes the doctor gives me medicines to drink but my body needs the food that I want to eat. If I think about eating it I will find a way to have it.

**I: What kind of they told you not to eat when you were pregnant?**

R: I don’t think there’s any food they told me not to eat. For me, I don’t like chicken and Koolaid and donuts, I just don’t want to eat them.

**I: Who was that want and didn’t want you to eat the food when you were pregnant?**

R: The child in my womb did. He made me hate the foods and vomits them out. {Laughs}

**I: Who took care of you during your pregnancy?**

R: My husband. He was the only one who took care of me.

**I: Is there any one else who took care of you?**

R: My mother and father were far away from me, so it was just my husband and sometime his family.

**I: How did the people help you when you were pregnant?**

R: help me? They help me by buying me the food that I want to eat and wash my cloths.

**I: Can you tell me what kind of medicines and nutritious food you took during your pregnancy?**

R: Vitamin and the medicine for Blood.

**I: Did you took all the vitamin or medicines they gave you? And why did you and why didn’t you?**

R: Because some, some of my kids when I was pregnant with them I didn’t like the vitamin for blood. I only take it at night when I’m about to go to sleep.

**I: Did you drink any alcohol during your pregnancy?**

R: No. I haven’t. I haven’t done any of those things.

**I: Was there any traditional medicines you took during your pregnancy?**

R: Just the one for dissines. When I feel dissy I use the traditional medicine and puts it on my face to make me feel better.

**I: Ok. If you were advised to eat more fruits and vegetables during your pregnancy, Could you tell me what would make it difficult for you to take them?**

R: I don’t think no one has ever told me to eat fruits.

**I: No one has ever told you to eat fruits or gave you a word of advice that eating fruits was good for you?**

R: You know when I was pregnant with my oldest son I used to eat peaches.

**I: What did it make it easy for you to eat the fruits?**

R: I want it for my body.

**I: Can you tell me about the food you eat when you were breastfeeding?**

R: When I was breastfeeding I usually eat fish, corn beef, maceral and sometimes coconut because it makes my breast to produce more milk.

**I: Ok. When you mentioned fish, corn beef, maceral. Cam you tell me why you eat these?**

R: Because it has more in it (Like more vitamin and it produce more milk in the breast.)

**I: What were the food they told you to when during the time you were breastfeeding and why?**

R: They usually tell me to eat so that I can have lots of milk in my breast. I usually eat uhhh… you know… I usually eat corn.

**I: Oh ok.**

R: Corn is the one that I usually eat.

**I: is there anymore food they told you to eat when you were breastfeeding?**

R: Fish and maceral. Because its for the breast to gain lots of milk.

**I: who encouraged you not to eat bad food when you were breastfeeding?**

R: my boyfriend did… he told me not to eat dried salt fish and other salty food.

**I: Why did he tell you not to eat them?**

R: He said its not good for both me and the baby.

**I: After you gave birth. Could you tell me how you first breastfeed?**

R: I sat down, took out my breast and start breastfeeding**.**

**I: After you gave birth or… you waited a little bit?**

R: I waited a little because he was sleeping after he was born. Some child when they are born the mother immediately starts breastfeeding them.

**I: Did you give other liquids to the child other then your breastmilk after he was born?**

R: No. I did not. All my children were breastfed.

**I: They were breastfed until what month or age?**

R: years. Some three years.

**I: Oh ok. That’s good. Now did you have any issues on just breastfeeding?**

R: No**.**

**I: did you have any issues on breastfeeding the child until he was 2 years old?**

R: no. I don’t think so.

**I: Can you tell me when you first gave food to the baby and liquids other than breastfeeding?**

R: 8 months.

**I: Why did you start giving them food and liquids?**

R: There comes a time when my breastmilk is not enough for the baby and that’s the time I give them food.

**I: What do others think about giving the child food and Liquids?**

R: Some say the child needs it for their body, and sometimes they don’t have enough from breastfeed that’s why they start to give them food.

**I: What were their first food and how did you prepare them?**

R: When I was in outer island I gave them breadfruit mix with milk and I take the juice out of the bandanas too. And jaibo (its flour mix with water and sugar and coconut milk) and U (coconut meat) mix with flour.

**I: Good. We’re trying to understand how the people in your community eats. Could you describe what your family and the people in your community eats and drinks? Usually eats and drink in day?**

R: for my husband he usually eats rice, rice in the afternoon and bread in the morning.

**I: Ok. What about the others close to you?**

R: same

**I: no difference**

R: Hmm.

**I: Can you describe how they make the foods?**

R: How they cook it?

**I: How they make the foods.**

R: Oh. we cook them like pancakes we cook them in the morning, bring the flour and mix it and make donuts and eat it, go to work or stay home… In the afternoon we cook rice, get the rice ready, if there is fish they eat it or if there is can food they eat it with the rice.

**I: Now if you cook it, is there anything you do other than how you prepare it? From the moment you start making it until its done.**

R: Our hands. We wash our hands…and.

**I: who in the family needs to have the food first and why?**

R: The Man. Secondly the baby and down to the older kids…. Laughs

**I: Ok. Why do they usually say the Man goes first?**

R: So that he can go back to work**.**

**I: Ok. Who are the ones that, can you describe… is there any difference about the food you give to each family members in your household?**

R: is there any difference?

**I: for example, you and your husband and kids eat chicken and the other family in your house eat tuna…**

R: Oh well there’s no one. We all eat the same food.

**I: Is there any difference on the amount of food you give to each family members?**

R: Just the food for the adults, they have bigger amount of the then the kids.

**I: Is there any one that get small amounts of food?**

R: No one. Just the kids because they are small.

**I: could you describe any food sharing between family members during mealtimes? Like for example children eating together separately from the family or they eat from the same plate?**

R: No. I need to separate their plates. The man gets his own plate, the kids get their own plates.

**I: Everyone each gets a plate.**

R: hmm.

**I: Now do the families share their food to their other family neighbors?**

R: yes. Sometimes. Sometimes I bring food to our dad in law next door and sometimes I prepare a plate and bring it to my other neighbor next door.

**I: We have heard from some families that eat local foods whereas others eat processed foods. Could you explain what is typical for your family?**

**R: Here or on the outer islands?**

**I: Either one**

**R: On the outer islands they usually eat Marshallese food. But here we usually eat Rice and can foods. The outer islands we eat Breadfruit and fish and all kinds of other foods.**

**I: what about here? What are the difficulties for you to eat Marshallese food?**

R: Sometimes we don’t have enough money and we can’t afford them.

**I: Ok. What about the easy ways for you to get Marshallese foods? Or take cook Marshallese foods?**

R: here? its far for us to bring the foods and we won’t go far.

**I: What about the outer islands?**

R: The outer islands no problems because they are closer to us and its easy for us to get them.

**I: What are the good and bad things for Marshallese foods?**

R: Marshallese food are not bad. They are good for us. We don’t get sick from it. We don’t get pink eyes, diabetes, we feel healthy. The food we eat here are rice and flour and those other stuffs.

**I: What about the bad things about it?**

R: There’s no bad things about Marshallese food.

**I: what are the good and bad things about imported food?**

R: They give us illness.

**I: What are the illnesses?**

R: We get diabetes, we get less vitamin… Laughs.

**I: Now the we are done talking about the family eats, I would like to learn more about how your child eats. Could you describe in detail what your son or daughter under the age of 2 years commonly eats throughout the day? What do they usually eat?**

R: They eat rice, I already said if we are here they eat rice sausage because it doesn’t cost to much, sometimes tuna, sometimes chicken and fish.

**I: When you cook the fish for your family, could you describe how you cook it?**

R: Sometimes I fry, sometimes we boil. That’s it.

**I: what about fish?**

R: I boil them. Sometimes I fry when I’m tired of boiling it.

**I: Now how many times they eat in a day including his snacks?**

R: well 3 times a day and sometimes snacks between every hour. He usually eats chips.

**I: Now how do you know if the baby eats enough?**

R: Eats enough?... when I see him play around and not he’s not crying.

**I: What would you do if the child doesn’t eat?**

R: not eat? Not eat any kind foods?... well I’ll go straight to the hospital and bring the child to the doctor to get him checked and to see why he doesn’t eat or whatsoever.

**I: What would you do if the child doesn’t want to eat?**

R: When he doesn’t really want to eat? I will find a way to get him eat or find what he wants to eat.

**I: on what ways?**

R: like ask him, do you want to eat this? Or what do you want to eat? And if he doesn’t want anything than I will bring him to the doctors.

**I: Is there any difference on how you feed your child when sick?**

R: Yes. There is a difference**.**

**I: What are the differences?**

R: sometimes he eats a little and sometimes he just doesn’t eat.

**I: But what about when you feed him?**

R: then he will eat more.

**I: Ok. That’s good. Have you done?... Can you tell me what the child under 2 years eat?**

R: can you repeat that?

**I: Sorry. Can you tell me how you prepare you child’s food from the beginning to the end?**

R: when they grow up, they take the same amount as how we eat when two years old from when he was a baby. How do you say it???

**I: How do you prepare their food from start to the end?**

R: When I prepare It, I keep it until they wake up and then I wash my hands and give it to them.

**I: Can you tell me what kind of food is good for your child under the age of 2 that will make him healthy and have a better life?**

R: like fish bananas and things that they can…

**I: is there any other more?**

R: Marshallese foods.

**I: What about food from here?**

R: I give them ice creams. Ehehe. And plus (Juice).

**I: What about foods that has nutrition in them?**

R: I don’t think there is… I usually give him…Oh I give them oranges and apples.

**I: What kind of food should you not give to your child?**

R: Candies.

**I: Why?**

R: Because they won’t want to eat.

**I: Is there any other reasons for you not to give them those kinds of food?**

R: They get skinny and diarrhea.

**I: Could you tell me how you would advice others on taking care of a child?**

R: word of advice?

**I: like word of advice to those who have kids on how to take care of them. What would you tell them and what advices would you have give?**

R: I would tell them, oh look after the kids so they won’t get injured and watch them, so they won’t touch the ground and touch their mouths after.

**I: Is there any other?**

R: tell them not to let them play where is dirty.

**I: Can you tell me the differences on how you feed your son and your daughter?**

R: there’s no difference on how I feed them. I feed them the same. I would tell one of them not to eat because the food is not enough. Both will eat.

**I: What about the amount of foods? Do they have them differently?**

R: they eat the same food and they have the same amount. But sometimes the older child can have a bigger amount of food because they are older.

**I: Could you talk about the role that other family members have in raising children in this community? How can you about how they are doing, like how do the parents raise their children?**

**R: They watch over them. The way I see them taking care of their kids is ok. Its like how I take care of my kids**

**I: On how you say you take care of your kids? Can you tell me how you feed them?**

**R: I make sure they don’t fight and make sure they take care of each others and for the young ones to respect their older siblings.**

**I: Who really took care of the child?**

**R: The woman or the mother of the child.**

**I: What are the responsibilities of the mother?**

R: Prepare their foods, take care of them prepare their cloths, clean them.

**I: Hmm. What are the responsibilities of the fathers?**

R: Buy them foods, buy them cloths, make them succeed in school.

**I: Now how do the care givers play with the children under the age of 2? Like how do they play with them? Like if you are a care giver. How do you play with the child?**

R: I let him play, and make sure he doesn’t get injured and make sure he doesn’t eat what’s dirty.

**I: What are the responsibilities of the grandparents on raising the children in this community?**

R: they teach them our culture, teach them to respect their family, tell them stories about the past.

**I: Now how do the grandparents help the child and the child’s parents…**

R: Teach them.

**I: teach them what?**

R: teach them from what’s right and wrong. Watch their side to side.

**I: What are the good things about their grandparents?**

R: LOVE FOREVER [LAUGHS].

**I: Love forever… 😊**

**I: Could you talk about the role that other family members have in raising children in this community?**

R: Half of the community or just us?

**I: We are now talking about the family in this community… Could you describe how they raise their children. In this community. Not your responsibilities but your neighbor responsibilities.**

**R: Watch them from getting hurt and work together.**

**I: How do the older kids watch over their younger siblings?**

R: they watch them, so they won’t get hurt and play with them.

**I: Ok. Your answers are good. We are almost done. The last section says, we would like to learn about ways we can develop health programs in your community. Could you explain where you usually get trusted information about nutrition and health?**

R: Public health. Hospital…. Hmm where else?... sometimes with the grownups.

**I: Now why do you trust the place where you get the information from?**

R: they know about healthy life. They teach us about the things.

**I: You mentioned good things… Good things on what?**

R: Healthy life.

**I: Yes. Where would you prefer for the information to go for you to easily hear and see them?**

R: We usually hear them on the programs like V7ab and the churches.

**I: Now what kinds of device do you use to listen to the information? Where do you get your information? Like things to talk on or?**

R: cell phone?

**I: cell phone? Internet? Facebook? Either one that you usually get your information from. Is it important for the information to be here?**

R: Yes.

**I: Why?**

R: to listen to them.

**I: Ok. When you think about your own parenting experience, can you explain what influences how you raise your children?**

R: on the kids that are really mine or what?

**I: it could be your own child or not…**

R: What is the difference?

**I: Yes. Is there any difference on how you raise them?**

R: some kids are ok to take care of them and some of them are so spoil that is so hard to time them.

**I: What is the other difference when you take care of them?**

R: I get tired. Sometimes I get so tired but when my husband comes back from work he will help me with the kids and it will give me sometimes to relax.

**I: What is the thought of the people on this community thinks about you taking care of your children? It could be the chiefs, the church pastor and your neighbors. What is their thought about caregiving in this community?**

R: on how I raise my kids they say its good and that I should just keep watching them.

**I: What about the landlords or chiefs in this community?**

R: they don’t have anything to say.

**I: What about the church pastor?**

R: Sometimes they say I am blessed to have them because one day they will help me too.

**I: Is there any word of advice or understanding you learn about caregiving?**

R: what would I say?

**I: Is there any lesson you learned from raising your children?**

R: I don’t think there is…I don’t know…

**I: Who gave you the information about raising a child?**

R: Myself. I took them and raise them myself.

**I: Is there anyone who gave you details about raising a child? Or is there any ideas how you want to learn on raising a child but don’t have with you?**

R: Yes. I want to know.

**I: like what? like, this question says. Is there any thing you want to know about raising a child that you don know off that you would want to know about it.**

R: Oh. There’s none.

**I: Is there anything else about the topics that we talked about today that we missed or that you would like to tell us about?**

R: Everything is good.

**I: Ok. We are done. I would like to thank you for your time. All your important information you gave us will really help our program. Thank you once again.**

R: Thank you also.
