# Supplementary material for: Conceptualizing multi-level determinants of infant and young child nutrition in the Republic of Marshall Islands–a socio-ecological perspective
Source: PLOS Glob Public Health. 2022 Dec 19;2(12):e0001343. doi: 10.1371/journal.pgph.0001343 (PMC10022247; doi:10.1371/journal.pgph.0001343)
Supplement: S1 Data — (ZIP) [file pgph.0001343.s001.zip › RMI Supp Data/Interviews data/I08U_IDI_CL_Rita_Aug 13_Balton.docx]

- Interview Code: I08U
- Interview type and Interviewee: IDI_CL
- Interview Date: 08/13/18
- Location: Rita
- Interviewer: Balton
- Transcriber: Cendaniel Milne

**I: first I would ask you some questions, can you please describe your role as a community leader?**

R: Thank you, my role in a community, I am the youngest among my Leroij (a word term for chief in an opposite sex e.g. queen or empress) sisters. Since they are not present at this moment, I am entitled on behalf of them in announcing community clean ups. If there are any issues, these are to be come directly to me, but if I am to answer any landscape constructions, decision is made by my older sisters. But now, I am responsible for making community clean ups in order to make a better healthy community for our families.

**I: great, Thank You.**

**I: apart from you, can you please tell me whose part of your community is?**

R: yes, there are Alaps (word for lords, land lords, ranked below the chiefs) in a community where they help work with the Iroij (chief) and the Leroij. In a provincial territory owned by the Iroij and the Leroij. If there are any complications, the Alap is the one who comes to the Iroij for the people’s needs.

**I: Thank You**

**I: apart from the Marshallese that live in this community, can you tell me are there any ethnic groups and languages in the community?**

R: Yes, there many Chinese, that are doing their store business.

**I: are there any others beside these Chinese? That are not Marshallese**.

R: there are few, there’s few.

**I: like who?**

R: outsiders, outsiders.

**I: like who are they?**

R: Indians

R: that’s about them

**I: that’s all?**

R: yes, the Chinese

**I: are there any differences on religion and its influence on community members?**

R: yes, there are, there are differences. There are differences because, for example, the Assembly church. There’s a lot of people… let’s say, drunken cannot go into the church. Because when it’s Sunday the churches are full with people. But, in other days, they live their lives. And do what they want. And from what I see, churches help people in a community to change their way of life.

**I: can you explain what type of treatment people in your community seek for your children, for example, traditional healer, doctors, nurses? Where do they seek for treatment for their children, where would you think of?**

R: I am thinking, they always bring them to the hospital. Other than that, if there are necessities on using Marshallese traditional treatment. They might look for a traditional healer. Like these things, traditional or foreign treatment. But, they frequently do go to the hospital for treatment for their children.

**I: who…who is the first, let’s say, if the child cannot, before they go to the hospital, is there a place where they can bring the child, get information for when the child is ill. Before I bring the child, this people might know more about it. Who do think the parents would bring before taking the child to the hospital.**

R: I think, I think, in a family, women do always look for treatment for an infant. Women there are in a community.

**I: do you think, parents frequently do use Marshallese traditional treatment?**

R: Yes.

**I: can you describe any illness associated with nutrition that affect children in your community?**

R: from what I see, they do always having diarrhea….fever, in a, these are the illness we see. And malnourished in foods, they do frequently are having flu illness, because there is not enough. The thing that fights bacteria. The child won’t, because we know, here in the Marshall Islands right? There is not enough food for a child in a community. For example, when they bring the Ramen noodle without any nutrias ingredients. Because of financial issue.

**I: what are types of food that makes a child body healthy?**

R: foods that have nutrients in them.

**I: like what?**

R: like orange, apple, vegetable, meats, small portion of meat not so big, egg, and things we see that can make a child healthy.

**I: what are the vegetables that we add on meals, those that people can frequently buy from the stores?**

R: like apple and orange, for vegetables, cabbage and those things, carrots, these are the things that are can easily bought, where prices are maintained accordingly to their income tax.

**I: what are the illness caused by food missing from diets?**

R: foods like chips that Chinese, sell them. Foods that don’t have any vitamins in them. Like chips, now Kool aids, things that we see that cannot make a child healthy.

**I: thank you, you’re doing a great job answering the questions. Instead of say yes and no, it gives a broad understanding.**

**I: are there any illness that can affect children, that not in foods that are planted. Like, they don’t need to be in the foods we mentioned, but others apart from them.**

R: yes, if they are to be given nutritious food properly. Their brains might not function properly, for they are not treated with the foods that have vitamins in them.

**I: great, great.**

**I: we’ve talked about health**

R: yes.

**I: now, we are going to talk about the foods here on our Islands.**

R: our Islands

**I: yes.**

**I: foods that we always eat.**

**I: can you explain how difficult for people in your community typically get food to eat on a daily basis.**

R: Yes, I know these difficulties, because of money. Their incomes are low to bring these things every day. But, as we know, there many local foods we see that they can bring them. We do know cause, local foods have vitamins in them to help our bodies. Iu (coconut meat) breadfruit, no matter what, they’re good. As am saying about these things, not enough money to bring these things.

**I: are there, are there any foods grown in this community?**

R: foods grown? There are few. Like every family in a community don’t grow foods. Like my neighbors, they can. Because, I have no idea what’s in us, these are some goals we can take local leaves and add them to our meals, but there are few. I think, each family is responsible for this, they can plant at their house, just like those at wellness. They can bring box of dirt and plant in it. One time, it grows. Cutting edges to grow, but I don’t know anything, maybe is it lazy? Or we just don’t want to make them. And from what I see, it’s better if a family is responsible in making what necessary. For health for foods, vegetables.

**I: there are those who plant and happened to sell foods, how profits are commonly used if foods are sold.**

R: let me say, the reason they’re selling, for example, those people form Laura right? When they sell their local foods, they buys things such as, rice, flour for donuts and pancakes things like that. Which are not vitamins. But the foods they are selling, we know that it is healthy for a people. And they buy chickens, steak, spare rib, things that are refrigerated. Rice, white rice.

**I: what do you see, from the way you are taking care of the people in your community, can you tell me about what makes people not growing foods and why?**

R: lazy, this thing I might say. There is no difficulties growing food near a house, there are few, but not enough land to plant. If this would be, plant two bananas. Is not enough, is not enough near the house. But, I would suggest vegetables near the house, they won’t take up any space. I can make a box here, a box here, to make their own vegetables.

**I: there is time where seasonal harvesting takes place right? Every foods has different seasonal harvesting?**

R: yes, seasonal harvesting.

**I: a time for seasonal harvesting right?**

R: Ananrak (foods season)

**I: yes the season, in those times, is it hard for the family to find foods?**

R: these days are different, unlike back in those days, I grew up in the outer islands. When it was time for Rak (plentiful of foods) there use to be a lot of breadfruit, Pandanus, there was a lot, like, now a days, and is different, climate change right? It might be. Unlike back in those days. Lots and lots of breadfruits. Like, just like the fruits that sprout. Now a days, when you looked at the foods grown, like the Pandanus growing, they don’t, they don’t grow. And too much heat, this why we called it climate change these days.

**I: it has changed?**

R: it has changed. Our seasons has been changed.

**I: and by this ways, can you describe if families in the community are having a difficulties looking for foods?**

R: yes it is. Unless, if one of the family members are working, they can pay for foods that are refrigerated.

**I: okay, what kind of animals that are raised in this community. Can you tell about animals that are commonly raised here?**

R: all I know of is Pig.

**I: Pig right?**

R: yes, but there are few. For those who don’t have safety tank they get ticket. Local government is giving them ticket. If they don’t have, done. They’ll close it.

**I: safety tank right?**

R: it’s for flushing out the waste, just like a toilet where wastes are stored in.

**I: a lot has changed now, unlike those days I used to stay.**

R: well, you make your own ten fences but you won’t get a ticket. But today, they do. Do you think is this good? I think it’s good.

**I: I think it’s good.**

R: the pigs.

**I: it is good for cleanliness**

R: yes, cleanliness.

R: and that’s it, if you have safety, but they do checkups, if it dirty. They’ll say, you need to clean this more.

**I: a lot has changed.**

R: and there are few people who does this thing.

R: maybe, only the outer islands

**I: what does? I think I have mentioned this one regarding the safety tank, the difficulties it has.**

R: yes.

**I: are there any difficulties that people can make their own safety tank for their fences?**

R: well, if they are going to give them tickets. Official will be back, they are allowed to have two days after the examining the fence. After two days, and the person says doesn’t have these that are related to the fence. The official will say, that problem is not mine, it’s yours. You need to fix it, that’s what they say. If they don’t have money, they’ll wait. There are others who are struggling.

R: and now some do kill their pigs and sell them

**I: because they want to make their own safety tank.**

R: yes.

**I: what else? See these are some information I did not know of.**

R: maybe you make pig fence those days but they won’t give you tickets.

**I: I don’t have a pig fence near the house**.

R: those days, these days you can’t. You make fence, raise a pig, and you need a safety tank…it is good, it’s sanitation. It doesn’t give you any illness.

**I: well, this last question on food, can you decides what food to get for the family in most households?**

R: the Woman

**I: the woman**

R: the woman because, she’s the one who choose what is good for her family.

**I: who decides which foods young children should eat?**

R: the woman too, but for the one that buys food, it’s the man…If it was a family, an example on me. For my family, I asked my husband, I write these to him, bring this, this, this, this, this, nutritious foods. But other women, they still don’t know about foods that are nutritious for a child…This survey is good, because were learning. They’re a lot women don’t know, they only know of rice and meat that’s it…do you think, it’s good for young children? At the age where you interview with. Must have whole nutritious foods.

**I: well, those questions about foods are done.**

**I: we’ll go through with water and Hygiene.**

**I: can you describe a typical day getting and storing water for people in your community?**

R: there are places for water, as I have mentioned before, if there is money they’ll go buying. But, there are some families do boil water.

**I: boil water?**

R: but there are others who usually drink, unless EPA (Environment Protection Authority) check for contamination for 24 days, till it’s safe to drink. And then they can drink from their water catchments.

**I: for now, what about water for bathing?**

R: same goes for the water catchments and government city water.

R: when it’s turned on, they get billed. But, others do cooking with and drinking from it. Boiling food and drink, the city water.

R: and what we thought and heard about the water is good. They haven’t say it’s bad, actually they boiled it.

R: in a community, a woman is the one boiling the water for her family.

R: I always teach them, teach them how to do their water.

**I: EPA?**

R: yes.

**I: well, this is about hand washing. Can you describe hand washing in your community? Is it something that is important?**

R: yes, yes, other families know. Let me say about my grandson, every time he comes from school, he washes his hands. I have already give out soap, the Dial Soap and a sanitizer before having a meal. But there are others that doesn’t wash their before taking a meal.

R: yes.

**I: can you explain what you think is difference between using only water or water with soap to wash hands?**

R: like, from what I see. Every time were told to use soap to wash our hands.

R: rather than using the water only. You need soap to wash your hand

**I: there’s a difference?**

R: there is a difference.

R: the soap is to kill germs.

**I: what are the. Can you explain anything that prevents families from washing hands with soap throughout the day?**

R: you can’t keep looking on someone who is washing hands with soap, it’s their own decision.

**I: great, thank you.**

**I: can you describe what kind. Each households in your community, what type of toilets they are using?**

R: there are others that are using water.

R: there are those who have their own toilets that use flushing, but there are those that don’t have a toilet, who are using lagoon and ocean side.

R: they don’t have any.

**I: there’s lot households that have toilets?**

R: yes.

**I: and those that are using water?**

R: water.

**I: yes indeed, there’s communities that are using the ocean and lagoon side. What makes people to use lagoon and ocean side?**

R: they don’t have any money to build one.

R: but, if I was an Alap. I would gather the people to tell them to find a way, not to use lagoon and ocean side.

**I: what makes it difficult for someone to build his/her own toilet?**

R: it is difficult, because they don’t have any money to build one.

**I: this is the difficulty?**

R: yes. This prevents from them.

R: and for the Alap, like for instance, this house. If there is a space here, you may build your own toilet.

R: it’s not so bad because it’s small house.

**I: yes it is.**

**I: and how young children’s stools are typically disposed of?**

R: they disposed them at lagoon side, doesn’t matter if it is low tide. They just threw it. (22:16)

**I: don’t they just leave it there**

R: they don’t, they cannot. They need to throw it away from their houses

R: but, disposing them at lagoon side. Each family must teach their children how to use a toilet, teach them and they will know. The child won’t use outside the house.

**I: now can describe where young children in your community play?**

R: next to their house.

**I: next to their house?**

R: on the road, there not enough space.

**I: are there any animals where they’re playing?**

R: there’s times we see, cats, dogs, these things.

R: but, my older sister announced last week that every dogs and cats are to be killed. No matter if they have name tags on them. She doesn’t want them to spread any illness. And cats too.

R: especially where there is a crowded space households. It is also bad, rats can contaminate water tanks through movement from one house to another.

**I: what is an ideal play area for children looks like?**

R: just like places where local government build, basketball courts. There needs to be play grounds. As you know, there is not enough space. Just like the one Katoj Park (near the airport).

**I: what are the challenges for keeping children’s play area clean? And what makes to be clean every day.**

R: cleaning, just like there is place at back of Jenrok (place at Laura) where they’re playing basketball and volleyball. Every time when playing is done, there is a woman there who always tell everyone no to go home instead of cleaning after playing.

**I: you have put someone in charge?**

R: there is woman there. Stay there, after the games are done. Tell everyone to help you clean.

R: there’s time when we have trash bags, she can use to them to put the trashes inside.

R: yes.

**I: that’s great, thank you for your help. Your answers are interesting.**

R: we know something when we stay long.

**I: yes indeed.**

**I: from your own opinion… could you explain ways to prevent the spread of disease?**

R: like, what kind of disease?

**I: like, disease that are easily spread, what are the ways that can help a community to prevent the spread of disease?**

R: let’s say, it’s the hospital’s job to come and explain about a pink eye. Do outreach, if there is a pink eye in the community. Bring them and treat their eyes. Just like that. Like for TB, we know they’re doing the same thing. That’s it, these are the things. Hospital needs to do outreach to help prevent the spread of disease.

**I: yes.**

R: the only thing to prevent is medication. And then, their community needs to be clean. It needs to be clean. Just like what I see there’s place where I came to see, I cannot believe it. Women are sitting, but their place is dirty. There’s a load of dirty laundry in each trash bags, I told them to bring them to the laundromat, and it cost only a dollar. If there is no water, hang them because the there’s sun. I told them to new small rocks near your houses, help to prevent the spread of illness. And I never seen how people react saying, is this how she teaching us?

R: it doesn’t matter, I told that every women needs to make their house clean. There are lot small rocks at lagoon side and ocean side, little by little it will be clean, or sank in sands onto every mud holes.

R: sitting there and wait till the sun comes up. Spreading disease of illness. Done.

R: the families have illnesses.

R: the young children too

**I: is there, is there, a connection between exposure to feces and illness.**

R: could you repeat it.

**I: feces of animals and stools of children that we see on lagoon side and ocean side. That they’re (27:28-27:30) and diseases together, do you think they are the same?**

R: yes

R: same with what?

**I: like is there a connection between them, feces and stools on shore?**

R: yes, it will make illness

R: just as I said, they need to clean their environment.

R: if there’s dog’s feces, they need to throw or buried fast from or near the house. Helps prevent the spread of disease. Just like these.

**I: thank you, your informative statements are great.**

**I: now we will talk about the roles and responsibility in a community, in your community**

**I: can you describe the care of children throughout the day in your community?**

**I: like.**

R: like in a household?

**I: in a household.**

R: well.

**I: who is mainly responsible for child care?**

R: women are, women are

**I: women right?**

R: in a community, when there’s something wrong with the children, women are to be blamed. Because they are the ones who educating them from inside the house and school.

R: and there are many children who happened not to have enough teaching. Children’s respond on learning is from their house.

**I: What reveals the identity of being a good mother?**

R: identity of being a good mother, is that she is neat. She knows how to communicate with others. And lastly, we look by the way her child and her environment is clean.

**I: what about a father?**

R: a father can teach, but not so likely to be presented near the child. To give and teach the child.

R: like, I am talking about other families. When the child has an assignment, the father just takes the assignment. So the mother comes along and teach the child.

R: more likely in a community, every women has to have this responsibility.

**I: can you explain who plays with child.**

R: their mother, their grandmother, and their grandfather. (30:22)

R: the father went out for work, when he comes back it’s night time.

R: usually the grandparents do and those that are in the house.

**I: now, from your own understanding. What are the roles of grandparents have in raising children in this community**.

R: educating them, lastly, I think. These days, grandparents don’t tell stories to their grandchildren. But those days, telling stories, telling stories. (30:58)

**I: are there, what are the ways that grandparents in raising children, support mothers and families. Are there any differences?**

R: I think there are differences, like. If the parents are going off to work, and they give the child to others. They won’t mind educating them, but it’s best for grandparent to be there. Because they’re close as family.

R: blood to blood.

**I: see your laughing.**

**I: can you explain what makes good grandparents. What shows a grandparents to become a good grandparents?**

R: good grandparents?

R: they’re good at teaching them, they don’t spank them, shout at them, and they’re more open to their grandchildren in any ways.

**I: yes, it’s great.**

**I: could you talk about the roles that others have in raising children in this community?**

R: sisters and brothers in the community, and their neighbors when they come visiting them. They do usually look after the children.

R: just like that

R: together.

**I: well, were about to go through the last questions. It’s about communication.**

**I: could you explain where people in the community usually get trusted information about nutrition and health. Like, in a community. Where would you think it would be easier for them to get information from?**

R: with their leaders.

R: like for the Iroij (chief) and Alap (landlords) gathering all the people along with their leaders to information from them.

R: especially the Alaps, are the ones who are going inside a community, and visualizing each households.

R: is it correct?

**I: yes it is. There are no right or wrong answers, statements are great.**

**I: are there any, we’ve talked previously, is there any other concept that you would like us to go through? Since you are the leader here on Majuro. Are there any difficulties you would like to discuss regarding the child’s health?**

R: yes, it is. Since the program have started where you acknowledge mothers of a family.

R: acknowledgement of giving nutritious foods to children.

**I: these are the concepts you would like to say?**

R: yes, these the concepts I would like to say.

R: it is a broad program where every mothers in a family needs to know what to give and how

R: am talking about family that cannot afford, on how they might give food to children in a family. When having no money on her, how can she, is there a difficulty she’ll face. As she knows already, when she is going to buy, it’s not enough. (35:00-35:02)

R: can the program provide something for a family? They can give to the mother to feed the child, is there what? Are there any medications that can have nutritious vitamin for the child?

**I: yes.**

**I: yes, the thing is the Ministry of Health is to help with this survey. To collect information, because these women can collect data for their report. As you can see on the newspaper, it states that it is the thing we are to target**.

R: there is a newspaper, I took from Russell. Am going to read it.

**I: without further a due, you are grateful for your time. Thank you for helping us, a right time to look for participant. Thank You**
