# Supplementary material for: Conceptualizing multi-level determinants of infant and young child nutrition in the Republic of Marshall Islands–a socio-ecological perspective
Source: PLOS Glob Public Health. 2022 Dec 19;2(12):e0001343. doi: 10.1371/journal.pgph.0001343 (PMC10022247; doi:10.1371/journal.pgph.0001343)
Supplement: S1 Data — (ZIP) [file pgph.0001343.s001.zip › RMI Supp Data/Interviews data/I09U_IDI_FCG_Rita_Aug 13_Fela.docx]

**Interview Code:** I09U

**Interview type and Interviewee:** In-depth Interview female caregiver

**Interview Date:** August 13 2018

**Location:** Rita

**Interviewer:** Fela

**Transcriber:** Fela

I: Before we put out record voice, if you are agree to put your voice in this record file, do you agree on that? Is that okay with you?

R: Yes. I do agree.

I: Thank you for giving this great opportunity and the time to talk with you today. Information that are given by you will help prevent ways of living of both the mother and the child along with the cleanliness of this community. To start, can you describe a little bit about your family? To make this question easier. Who live in this household?

R: Umm.. my husband and I, our baby, his parents, his grandmother, and his cousins.

I: Can you tell me how many kids in this house and their ages. How many girls and boys ( Children).

R: Only one kid with one year old.

I: Hmm. Just one kid.

I: Now can you discuss about this community? Or what are some positive or good things about this community? Like what some things you see that this community is good about. And what the negative things about this community?

R: umm.. its good that it has great and big environment,

I: hmm, yes it has large environment…

R: Its windy,

I: Aside from windy, what else can you tell more about this community?

R: um.. there are plenty things like banana tree, coconut tree, and pandanus tree.

I: Great! And can you describe some negative things about this community? ….. What are the negative things about it.

R: Trashes

I: Aside from trashes what else can you think of?...... Ok you mentioned trashes, what else? Only trashes you can thinks of?

R: ( clear throat) umm, its dusty,

I: yeah what else?

R: Um and houses are too close to the street.

I: Great! Now we will discuss about health and illness in this family. Can you discuss what illness that your child often get seriousness sickness of it?

R: Fever

I: Can you discuss more what cause the child to get fever?

R: um.. mostly he stays in windy and cold areas and also he bathe often because he loves to take a bath all the time.

I: Now can you tell me how can you prevent fever illness?

R: umm.. don’t allow him to stay in cold and windy areas ( Air condition R meant)

I: Great! Can you please explain on how can you find out that your child needs to see the doctor when sick?

R: When he has over fever

I: Who would be the first one that you would bring your sick child to and why?

I: The question says that, who would be the first one you’d bring your baby when sick?

R: The doctors so that they would check on him.

I: Can you tell if you are using Marshallese traditional medicines to your child when sick?

R: umm. Yes!

I: Great! Now can you explain sickness that cause you child from foods contain with nutrition they eat.

R: How? What is the question again?

I: Let me rephrase that. “what kind of sickness the kids take from foods that contain without any nutrition?

R: Sorry I do not know the answer.

I: You don’t know” Don’t worry if you can’t answer these questions there are no right or wrong questions as long as you can understand and able to answer the questions.

I: Moving on…Can you explain what some illness that causes you child….ow no. Excuse me.

I: We discuss about unhealthy and now can you please explain life of a person that feeling well from the time he or she wakes up until the time he or she goes back to bed?

I: Do you understand the questions? Just to make it more clearly, it says “What are some symptoms show how a child under two years old have a healthy life.

R: The child feel goods, can play, he/she is not cry of very often.

I: Now can you tell me what some symptoms show that a person is feeling well?

R: um..( pause) they can move very easily. They’re not lazy to move their body… (Thinking)

I: What else aside of these you can think of?

R: That’s all I can things of.

I: Now let’s discuss about washing hands. Can you please explain to me how the family wash their hands during the whole day?

R: They hold the soap in their hands, rinse it together with their hands and wash it with water.

I: From you understanding, can you explain do the children wash their hands on a complete day?

R: Just any children in this community or children in my house?

I: It can be any kids like kids in this community or kids in this house.

R: There are some kids that they don’t wash their hands because they do not know that they should wash their hands. Other kids do wash their hands because their parents tell them to wash their hands.

I: Can you please explain to me more on what time people in the family wash their hands with soap on one day?

R: When we know that we are going to eat our foods, we wash our hands, cook or prepare the foods.

I: Can you describe the differences between washes the hands with soaps while wash it with only water? Make it easier so that you can understand, differences when you what your hands with soap and without soap.

R: Ow!

I: What are the differences?

R: When you don’t use soap to wash your hands, it can’t kill bacteria in your hands and these bacteria can easily remain on people’s hands and it’s too dirty. And if you wash your hands using soap, it results in a very clean hands and also killing all these bacteria.

I: Okay..

I: What are some ways that prevent you from wash your hands with soap on a day? Like, what makes you refuse to wash your hands with soap? Like what are some ways that prevent you from wash your hands with soap?

R: uhhh.. When you are too busy and you forgot that you didn’t wash your hands with soap or also can be when you are in a hurry and you also forgot that you did not wash your hands too.

I: Great! You have great answers that’s perfect.

Now we would discuss on foods that you ate during the time you were pregnant and during breast feeding.

I: Now can you please take back to when you were pregnant, can you describe foods you were eating during pregnancy and the time you weren’t pregnant? Like foods you were eating during pregnancy.

R: umm, rice, bread, chicken and can meat.

I: Now can you discuss what makes you wanted to eat foods like mentioned above during pregnancy?

R: There are none!

I: What kind of foods your pregnancy supporter wanted you to eat during pregnancy and why?

R: Vegetable and fruits that help support the health of both me and the baby.

I: Great! Now what kind of foods your pregnancy supporter to nurses warned you not to eat during pregnancy and why?

R: Salt, it affect the baby and also me, and also cool-aid ow and also sweets.

I: Who wanted or never wanted you to eat or don’t eat these foods during your pregnancy?

R: People in my families, and also my doctors.

I: Who help or took care of you during pregnancy?

R: umm, my families, and also my husband’s families.

I: Can you please explain how did people help you during pregnancy?

R: ahh, they used to buy foods for me, bring me to my appointment, and make sure that I would drink my medicine.

I: Great answer. Now can you describe what kind of medicine or vitamin you drank during pregnancy?

R: Medicine for blood and medicine for vitamin.

I: Please tell me were you able to take these medicine given to you and reasons why you did not take them.

R: I was able to drink all these medicine given to me.

I: hmm that’s great answer.

I: Can you explain more on why did were you able to drink them?

R: So that my baby and I should have a healthy life.

I: Really good answers.

I: Can you tell me were you able to drink or smoke or do drugs during pregnancy?

R: At first during pregnancy I used to drink.

I: Okay…. Soooo, Where there any Marshallese traditional medicine you took during pregnancy and why?

R: I never take Marshallese medicine during pregnancy.

I: If there was someone to tell you to eat fruits during pregnancy, what are some difficulties that prevent you from eats fruits?

R: How? What?

I: To make it more clear, the questions says what make it easy for you to eat fruit and why?

R: Fruits like what kind of fruit..?

I: Just any kind of fruits

R: Ok so what was the question about?

I: What makes it easy for you to eats fruits during pregnancy and what makes it hard for you to eats these fruits?

R: The taste of the fruits makes it hard for me to eat fruits.

I: The taste? Alright okay!

I: Now can you describe what kind of foods you used to eat during breast feeding?

R: um.. Fish, rice, chicken…Vegetable, corn beefs, and yeah.

I: Can you describe what kind of foods they told you that you are supposed to eat because you were doing breast feeding and why?

R: Fish, corn beefs, yeah these two they told me that they can help make breast milk for breast feeding.

I: Now can you discuss what kind of foods they warned you not to eat during breast feeding?

R: ahh.. sweets, cool-aids, chips, and yeah

I: hmmm,, So can you tell me who warned you to eat or don’t eat these kinds of foods?

R: The grandmothers of my babies.

I: Now after gave birth, can you explain how did you breast fed your child on a day?

R: (clear throat) …how?

I: Like how did you do breast feeding to your baby after you gave birth?

R: I just hold him and did the breast feeding

I: So can you tell me after gave birth, how long and then you started giving breast milk to your baby? Why?

R: I gave birth to him and then breast fed him right away

I: Can you tell me if you ever give your baby other kind of milk except breast feeding on the days you just have birth to him?

R: I did not.

I: Can you tell me why?

R: Like baby bottles contain with different milk? What about bottles contain with breast milk?

R: Ow yes. He eat from my own breast fed to him because it was healthier for him.

I: Are there any difficulties or possible ways you do breast feed to your child until he was two years old? And why?

R: There were no difficulties for doing breast feeding for my child.

I: Can you please discuss when was the first time you start feeding your child or give drink instead of gave did breast feed to him?

R: When he was seventh month he stared to eat foods and drink water.

I: Perfect! You answer perfectly. Can you explain why did you feed your start feed your child foods and water except from breast fed?

R…

I: You fed him real meal when he was seven months why did you give foods to him instead of only give him breast milk?

R: So that he could now learn to eat real meal or real foods. ( hard foods)

I: Now can you explain what other people’s thinking by start giving foods or drink in their ages and months?

R…

I: What are they thinking by giving foods and drinks like what do they think would happened?

R: Some mothers say that they have only a little amount of breast milk to feed the child.

I: hmmm. So can you discuss what were the very first food given to them and how did they prepare these foods for the baby? To make it easier, as of your baby, what were the very first food you gave or fed him and how did you prepare these foods?

R: I first feed him the baby cereal that we bought it from the store, now mix it with the morinaga milk and then feed him.

I: Great. Now we are trying to understand how people in this community eat. Can you please discuss what or how the people in your family eat or drink on a day?

R: Chicken, rice, bread in the morning, fish, can foods.

I: So can you explain who among the family are supposed to eat at first and then who eat after?

R: The children have to be fed first and then after, older people.

I: Can you discuss if there are any differences by giving foods to people in the family?

R: No there are no differences in giving foods to people in the family.

I: Can you tell me if there are any differences in giving amount of foods to people in the family?

R: As of children, they have only a little bit amount of foods, and the older people given more foods for them.

I: Now can you tell me if other children were served foods more than the others?

R: ……….

I: Or can be any kids in this family or children in this community.

R: Yes. Some are given only little foods depend on much foods they eat while other given more than enough foods because they eat more than enough.

I: Now question number fifteen…. Can you describe how the people in the family serve their foods during meal?

R: …..

I: For example, children in the family eat in separate plate or the family members eat from only one plate?

R: Children eat in separates plates while older people eat in their separate plates.

Can you tell me if the family share foods to their neighbour?

I: They only served foods when there are enough foods to serve others.

R: hmm great.

I: We heard that some families here eat local foods while some eat from the imported foods. Can you tell me what kind of foods people in your family often eat every day?

R: ….

I: Like local foods or imported or processed foods).

R: Pandanus, chicken, rice, fish, and cow meat (stake meat), crabs, and yes.

I: Now can you explain what makes it easy or difficult to cook these local foods?

R: Make Marshallese foods?

I: Local or Marshallese foods. What makes it easy or difficult to cook local foods?

R: Sometimes it’s hard to find Marshallese foods to my child.

I: Ok great. So can you describe what the negative and positive things about local foods are?

R: Marshallese or local foods are good because they contain lot of vitamins.

I: Great. So what are the negative and positives things about process or imported foods?

R: There are process or imported foods contain with chemicals.

I: Great. You have really great answers.

I: Now have already talked about how the people in this family eats, now can you tell me how you feed your child? Can you please describe what kind of foods your child (boy or girl) under two years old often eat on one day.

R: My child often eat bread in the morning, eggs, cereal, French toast and yeah foods like these. For lunch and dinner, he would eat chicken with rice, fish, or tuna.

I: Okay… Now can you tell me how many times your child eat on a day?

R: Morning for breakfast, lunch, and dinner. And sometimes after nap when he wakes up he would also eat.

I: Now can you tell me how would you know that your child’s food is enough?

R: I would feed him until he refuses continue eating, I will now realize that the food is enough and he is full.

I: No can you explain what would you do to your child when he or she refuses to eat?

R: What I would do is make him watched his movies while I feed him so that he would able to eat.

I: Can you explain are there any differences during feeding the child when sick?

R: Sometimes when my child is sick, he refuse to eat, and he would eat only a little food. And sometimes even though he is sick, he would just eat his food.

I: You describe foods that children under two years old eat, now can you describe how to prepare the foods from the beginning until the food is ready to serve? Like how do you prepare or how do you cook the food for your baby?

R: …

I: Any kind of foods you give or feed your baby, how do you prepare and how do you cook the food for the baby?

R: ummm. Sometimes I would cook fried egg for morning breakfast and also give the bread together with the egg while drinking milk. For lunch, I would cook the rice for him and then feed him with boiled or fried chicken or can be chicken stewed. .. And also fish.

I: Great. Now can you discuss what kind of food are supposed to be given to your child in order for him to grow healthy?

R: umm... Vegetables, foods contain with vitamins.

I: Can you tell what some good examples… What kind of food you are not supposed to give to your child?

R: Sweets like candies, salty foods and also cholesterol foods.

I: Can you explain what would be the best ways or examples in feeding the children?

R: …

I: Can you tell what some influences on feeding children nutrition foods?

R: The child must grow healthy and also a smart kid.

I: Can you explain the differences between feeding your female child or male child under two years old?

R: ?

I: whether you have either boy or girl can you discuss the differences of feeding them?

R: I do not have a daughter I only have a son.

I: We are also interest in the roles and responsibilities different family members raise their children. Can you describe the roles of raising children in this community?

R: …. ?

I: To make it easier, who among the family member is responsible in taking care of the baby?

R: The mother of the baby

I: Can you tell me what are the roles and responsible of a mother for the baby?

R: She make sure the baby is clean, plays with her, prepare the foods, feed him/her, and prepare the child for bed and a lot more.

I: Now can you discuss the roles and responsibilities of a father for the child?

R: Work and earned money in order to buy foods, needs and wants for the baby.

I: Can you explain on how caregiver play with children under two?

R: …

I: Like the people who take care of the child how do they play with children under two?

R: It’s good.

I: I mentioned that it is good, what makes it good.

R: They takes their time and play with their children and do what -ever the child wants them to do.

I: Now can you discuss the roles and responsibilities of the grandparents for the children in the community?

R: The question says that, how do the grandparents of the children in the community raise them?

R: Sometimes they cook foods for the child, watched over the child, play with him/her, also buy milk and diaper for the child, and yes.

I: hmm.

I: Now can you tell on how the grandparents help the parents of the child raise the children?

R: Confuse (same questions as above)

I: Don’t worry you already answer both the first question include this questions. (Sorry about that).

I: Can you explain what makes the grandparents of the children good grandparents?

R: How???

I: The questions says... What makes a grandmother or a grandfather a good caregiver for the children?

R: They are good when parents of the child are good and the child is behave well.

I: Can you explain the roles and responsibilities of other family member in raising the children in this community?

R: um. They also help watched over the baby and sometimes they buy what news for the baby.

I: Now can you tell on how older sibling raise younger children?

R: Some older sibling raise their younger children in a good ways and some raise them in a bad ways.

I: You have very helpful information from your answers and we are almost finish. We would like to learn about ways we can develop health programs in your community.

I: Could you explain where you usually get trusted information about nutrition and health?

R: Sometimes I would go online and search information based on healthy food for children from the internet (like google). Or I usually get information from older people who have enough knowledge on that.

I: Now can you tell me why do you trust where these sources come from?

R: Because I have experienced that these information are real and they worked.

I: Now can you tell me where nutrition and health messages should delivered so that she would see/hear them most easily?

R: They should go to the radio station (radio b7av) so that people can easily hear them and also to the hospital and people’s houses.

I: Can you discuss the opinions of the community influence how they raise their children….

I: Can you explain to me on any advice or information related to parenting you received?

R: Yes I have received information like these.

I: Can you tell me more about it about where did you received information related to parenting you received.

R: Like I have heard that when a parents spend a lot of time parenting their child, the child will soon can communicate with us and able to understand and especially a smart child.

I: Great! Now can you explain where and from whom you get these information from?

R: ….

I: Did you understand the question? The question says, “From whom and where did you get information on parenting and raising children from?

R: From the older people.

I: Can you tell me more on who else you has received these information from.

R: umm. My aunties and also mothers who have already have children.

I: Now can you tell me are these any information related to parenting or raising children you want to know and you do not have with you.

R: hum. No there aren’t any at this moment.

I: Great. So are there anything else about the topics we talked about today that we missed or that you would like to tell us about?

R: There are none.

I: Ok now we are done. Thank you once again for your generous time you shared your supportive information you have given. We are very thankful for your time and we hope these information will help support on better health for both mother and their children.
