# Supplementary material for: Conceptualizing multi-level determinants of infant and young child nutrition in the Republic of Marshall Islands–a socio-ecological perspective
Source: PLOS Glob Public Health. 2022 Dec 19;2(12):e0001343. doi: 10.1371/journal.pgph.0001343 (PMC10022247; doi:10.1371/journal.pgph.0001343)
Supplement: S1 Data — (ZIP) [file pgph.0001343.s001.zip › RMI Supp Data/Interviews data/I10U_IDI_FCG_Rita_Aug 13_Meia FelaEdited.docx]

Interview code: I10U

Interview type and Interviewee: IDI FCG

Interview Date: Aug 13

Location: Rita

Interviewer: Meia

Transcriber: Christina

I: well, if it’s okay with you to do an interview or get information, please answer.

R: yes, I agree with the interview.

I: thank you. Well now, thank you for giving me this precious time for us to talk today. The information we learn will help us find ways to improve maternal and child health and sanitation in our country, now to start off with, can you tell me a little bit about your family?

R: such as what?

I: who lives in this house?

R: oh, there are lots who live here….

I: could you describe?

R: say their names?

I: no, you don’t have to say their names, you just tell who lives in here, how many children, how many adults.

R: 4 kids…14 adults…

I: now we are going to…. how many girls and how many boys?

R: plus, the kids, right? 8 girls, and boys, maybe 8.

I: okay. Now we’ve talked about this family, now can you tell about your community?

R: like what?

I: such as the positive things about this community.

R: it’s, it’s okay because it has more space, it’s not that bad, the only bad thing about it is the street, it’s near the street.

I: mmm…now we’ll talk about health, and also illnesses that affect a family or the people. Can you tell me the illnesses the kids in your house or the community usually suffered from?

R: fever.

I: **fever… this one is different than the ones we’ve covered. Fever, what caused the kids to have fever? can you tell me?**

R: such as, like for example, my son, sometimes he gets fever by the things in his skin or body

I: **mmm….**

R: sometimes when to children spend too much time in the rain, it caused them fever.

I: **you mentioned the things in his skin, can you explain what’s in his skin?**

R: like boil…

I: boil…sometimes in his teeth

I: **okay…boil, well. Now do you think fever is a serious illness?**

R: yeah.

I: **why do you say it’s a serious illness?**

R: some people are dead because of fever.

I: **How do you prevent the child from having a fever?**

R: go see a doctor…

I: **okay. Are there any other ways for you to prevent the child from having fever, aside from seeing a doctor? Are there any other was?**

R: no, drinking medicine only and the fever is gone.

I: **that’s it. Now can you describe how you know when your child needs to see a doctor?**

R: monitoring the child’s temperature but it’s not getting any better, then that’s when we go see a doctor, so that they can give us medicine, and when the child drinks medicine, the fever started to disappear little by little.

I: **when you mentioned monitoring the child’s temperature, how do you do it?**

R: by applying cold water.

I: **cold water…who will be the first person you seek when your child is sick? When your child is sick, who is it that you seek first?**

R: there (the hospital), see the doctor first and then wait till they’re done weighing and checking the child’s temperature and they brought him to another room and then the doctor starts questioning me

I: **mmm….**

R: about the fever….

I: **mmm….**

R: and they wrote a description of medicine…then off to the pharmacy…

I: **okay. Can you explain why you should bring the child first to the doctor?**

R: because they first check how much the child weigh, first they check then go to another room.

I: **so, do you use…do you usually use traditional medicine? Do you use it when your child is sick?**

R: yeah sometimes…like when they’re coughing, we make them drink banana, also when there are like rash or small spots inside their mouth, we made medicine out of the kiden ( a tree that grows only in the Marshall).

I: **Now can describe any illnesses affecting your child that are associated with nutrition?.....do you understand the question? No? okay. Can you describe any illnesses affecting your child that are associated with nutrition? Foods that lack nutrition, are there any illnesses he gets from the foods he eat?**

R: none…

I: **none….**

R: he doesn’t eat frequently. He only eats one time today, one time tomorrow.

I**: oh okay, question 6, now we talked about being unhealthy, could you describe someone living healthy lifestyle, can you describe how or what they do from the time they wake up in the morning until the time they go to bed?**

R: now, how should I…

**I: Can you explain how or what does a healthy person do throughout the day?**

R: Good. For example, the husband and the oldest daughter of the wife, when they wake up in the morning, they feel great, only when they are sick, they’re out of sight.

I**: what do you see that makes you know that they have a healthy life?**

R: I see them running, they’re playing, they’re ecstatic even though they are hungry and thirsty.

I: **what are the appearance/signs of a healthy child under the age of 2?**

R: just as I have said before, they are running, they don’t cry a lot.

I: **mmm, what are the appearance or signs of a healthy adult**?

R: I don’t know. The adults here in this house are not in good physical shape, like the old woman, the one that we talked to…

I: **mmm…**

R: sometimes her finger are sores or numb, and yeah same as the old man, it’s like all of them, it’s like, there’s no one that are in good health.

I: **what about the adults here?**

R: you the girls?

I: **mmm, the women….**

R: sometimes when we wake up, we feel better, we do all the working around the house, we cooked, we moved, it’s not like when we’re sick like the old woman.

I: **Now, let’s talk about washing hand. Talk about washing hand, could you explain how do your family wash their hand throughout the day?**

R: like how they washed…

I: **Describe in detail, how they washed their hands…**

R: they filled up a bucket with water, put a soap near it and the children washed their hands before they eat, before they drink.

I: **mmm….now, do the kids wash their hands throughout the day?**

R: Yeah.

I**: okay. The next one, when will you use soap to wash your hands during the day?**

R: when?

I**: mmm…**

R: the whole day….

**I: you used soap to wash your hand throughout the day.**

R: when we are about to drink or eat, we washed our hand, also when we’re done playing, both the children and the adults.

I: **mmm…. okay, can you tell me the difference between using water, water only, and using water and soap for washing hand?**

R**:** the water doesn’t execute the germs but when you use water and soap, the germs are gone. Sometime when they are using only water to wash their hand, it leads to diarrhea.

**I: okay, good.**

R: because they are not using soap.

I**: Now, what prevents you from using soap when you are washing your hand throughout the day?**

R: prevents us from getting sick….

I: **it’s like what prevents you to not washing your hands with soap.**

R: what is that mean?

I**: what makes you not using soap when you are washing your hands throughout the day?**

R: I don’t know…

I**: don’t worry, it’s all good. Now let’s go down to question 8. Now we will talk about the foods you usually eat, you usually eat during pregnancy and breastfeeding. Now I want you to look back to when you were pregnant, can you tell me what kinds of food you ate?**

R: rice and fish, breadfruit, bwiro(combination of breadfruit and coconut milk)

I**: rice, fish, breadfruit, bwiro…**

R: papaya, sashimi, processed food…

I**: processed food, what about what makes you want to eat these kinds of food during the time you were pregnant?**

R: what?
I: **this question is asking about what makes you want to eat the food that you ate during pregnancy? Just think back to when you were pregnant.**

R: well, I ate many kinds of food, I’m not the picky type.

I**: oh….so, are there any kinds of food they want you to eat during pregnancy**?

R: were there food...…. what???

**I: were there any foods they say…. I mean they want you to eat during the time you were pregnant?**

R: that I don’t want….no, none.

I: **none….**

R: I eat any foods they bring.

I: [**laughing] so what kinds of food they didn’t want you to eat?**

R: foods like salt, Kool-Aid, uncooked noodle, foods that are salty and are not good for us,

I: **mmm…okay, so, why didn’t they want you to eat those kinds of food?**

R: because they’re protecting the baby inside my womb…

I: **mmm…**

R: that is why they didn’t want me to eat these kinds of food such as salt, kool aid…

I: **they say they are protecting the baby so……so the baby will be healthy…**

R: yeah, so the baby will be healthy.

I: **Who took care or supported you when you were pregnant?**

R: my mother.

I: **mmm….so how did your family helped or supported you when you were pregnant**?

R: they’re like, giving me foods…

I: **mmm…**

R: help me lifting stuff that are heavy.

I: **Can you tell me what kinds of supplements you took during pregnancy?**

R: my first visit to the hospital was when I was 8 months pregnant.

I: **you were 8 months pregnant when you first visited?**

R: I took medication for the blood, and also the vitamins.

I: **medication for the blood and vitamins**…..so you took all the supplements that were given to you?

R: yeah, only 2…

I**: Did you drink alcohol, smoke or use any kinds of drugs at that time?**

R: no…

**I: were there any local treatments you took during the time you were pregnant?**

R: none… [laughing]…sorry.

I**: okay…now for question 9…can you tell me what kinds of supplements…. question 10….if you were advised by anyone to eat fruits, what makes it difficult for you eat those kinds of fruit?**

R: such as what???

I: **fruits and….**

R: I don’t think there were any fruits I dislike…

I: **were there any difficulties for you to get these kinds of food?**

R: no…

I: **what makes it so easy for you to get these foods? This question is….**

R: such as the one that is written….

I: **no…such as fruits, and foods that are nutritious for pregnancy. What makes you want to eat these kinds of food?**

R: I just want to eat, like when I see a person eating, then I will start to get the feeling of wanting to eat….

I: **Can you tell me what kinds of foods you eat….can you tell the foods….**

R: you mean fruits…

I: **that you usually eat during breastfeeding…..**

R: you mean, the time when my son was born.

I: **I mean the time when you breastfed him.**

R: mmm…

I**: what foods you always eat…**

R: usually fish and local foods.

I: **local foods and fish. Now makes you really want to eat these kinds of food during breastfeeding? Why do you eat fish and…..**

R: so that there can be breastmilk, so it can be enough for the child….. I breastfed my son and I also gave him a bottle of milk.

I: **oh, so you also gave him a bottle of milk.**

R: yeah….

I: **What kinds of food you were encouraged to eat during breastfeeding?**

R: such as fish, corn-beef, the ones that we usually eat to have breastmilk.

I: mmm, what about the ones you were encourages not to eat during pregnancy?

R: not to eat?

I: were there any food that you were encourages not to eat?

R: food likes the one that are salty, we don’t use sodium a lot.

I: okay, now this question is asking about why don’t you eat the foods that contain lots of sodium?

R: to prevent the child from any harm…

I: mmm….uhh….so who gives you advice to eat those foods or not eat those kinds of food during breastfeeding? Who gives you advices?

R: my mom and grandma, the old woman that lives just across…

I: Now we will talk about Young Child Feeding Practices. Now, after giving birth, could you describe how you breastfed your child through out the day?

R: hold him and…

I: described how you breastfed him….

R: hold him and breastfed him…

I: this question is asking about how long it took for you to breastfeed your child after giving birth.

R: it took like 20 minutes, sometimes 30 or longer.

I: Now when you gave him a bottle of milk or any other liquids after giving birth…..oh did you gave your child a bottle of milk or any other liquids in the first few days after giving birth?

R: yeah, a bottle of milk.

I: bottle of milk, okay….so why did you gave him a bottle of milk?

R: because the hospital in the states encouraged me to do it……also, sometimes the child is not full, so I gave him other liquids.

I: mmm…good. Were there anything that makes it easy or difficult to breastfeed exclusively up to six months? Was it difficult for you to breastfeed exclusively?
R: no….

I: no, none. Okay, were there anything that makes it easy or difficult to breastfeed up to 2 years?

R: were there what?.......difficulties?

I: **was it difficult or easy for you to breastfeed your child up to 2 years?**

R: easy not difficult…

I**: okay, can explain how was it so easy? What makes you say it was easy for you to breastfeed? Why is so easy to breastfeed?**

R: because the paper that was given to me after giving birth says that when a child stop from being breastfed, can lead to malnutrition, so the child should always breastfed in order to be healthy, because sometimes when a child is sick, he/she eats or drinks less, so that’s why breastfeeding is important.

I: **okay, question 13, can you tell me when you first gave him foods and liquids other than breastmilk to** your child?

R: when he was about 6 months.

I**: 6 months, now why did you introduce foods and liquids other than breastmilk at that age?**

R: some say that when a baby is 6 months old that’s when they should start eating baby foods, but still breastfed.

I**: mmm…are there any opinions from others that influenced their decision to introduce foods and liquids at that age? Do you know any other opinions from others?**

R: I don’t know.

I**: okay, no problems, now, what the first foods were and how you prepare them?**

R: I usually buy baby food from the store and reheat it first before giving it to the baby.

I**: only baby foods were first introduced to the baby?**

R; yeah and pandanus juice. Sometimes I gave him banana which is mixed with milk.

I**: how do you do it?**

R: I just slice the banana piece by piece and then poured a cup of milk into a bowl, then add the bananas, then fed the baby.

I**: what about the pandanus juice?**

R: no ingredients for that, but for the JOKOP( Marshallese food), combination of flour, sugar and coconut milk.

I: **mmm…we are halfway there…only 1 page is left now. Okay… now we are trying to understand how people in this community eat? Could you describe in detail what your family usually eats and drinks throughout the day? what kinds of food you guys are eating?**

R: local foods and rice, and can meat, the foods we eat, they also eat.

I: **what about local foods?**

R: yes.a lot of local foods.

I**: local foods such as what?**

R: such as breadfruits, porks….

I**: breadfruits, porks and…..**

R: fish…

I**: fish…**

R: crab…

I: **crab…**

R: bwiro (Marshallese foods)…

I**: bwiro…..can you explain how the meals is prepare, how you prepare your family’s meal?**

R: well as for breadfruit, we cooked it, we put the breadfruit in the fire and wait until it cooked then we take it out from the fire and start peeling it. As for crab, just boil it, and as for bwiro, we just ad sugar and coconut milk to it.

I**: who in your family should be served first and next?**

R: the children first and then next are the adults.

I: **are there any differences in the foods served to different family members**?

R: no.

I**: Are there any difference in quantities of food served to different family members?**

R: well, for the kids, they don’t eat a lot, so we served them little, as for the adults, well they have big stomach so they will get the huge amount of food.

I**: both men and women…**

R: mmm….the adults usually served themselves.

I**: okay. Now, are there some children receive more food than others**?

R: children? Yeah, there are some that received more than others.

I**: Now could you described any food sharing between family members, during mealtimes. (for example, children eating together separately from the family, meals eaten from the same plate by all the family members)?**

R: well for this family they bring their own plate, same goes for other family, so when is mealtime everybody uses their own plate, they don’t eat from the same plate.

I: **Now does the family share their food with their neighbors?**

R: yeah, when there is gathering and visitors……., sometimes we give foods to our neighbors.

I**: Now for question 16, we have heard from some families that eat local foods whereas others eat processed foods.**

R: as for this family, we usually eat local foods but sometimes when there’s a party we eat foods like salad and chicken.

I: **now, what are the positive and negative things about local foods?**

R: local foods are good for their health, unlike imported foods like chicken, chickens contain fats…

I**: and what are the negative things?**

R: some people are allergic to local foods.

I: **so what are the positive and negatives things about imported foods?**

R: some foods are unhealthy…

I**: can you tell me what…**

R: foods like corn-beef, it contains a lot of fats and some have gout caused by imported foods.

I: **yeah, are there any…**

R: no.

I**: Now…..Now….now that we have talked about how the family eats, now I want to know how your child eats. Could you describe in detail what your son or daughter under 2 years commonly eats throughout the day?**

R: I am telling you what they usually eat…

I: **mmm, the foods that you fed your child, any kinds of foods that you give to your child.** R: at breakfast, I gave my child cereal, doughnut, pancake, bread, and sometime banana, and at lunch, well, rice, sometimes breadfruit, fish and can meat, same goes for dinner.

I: **mmm, how many times a day meals, including snacks are eaten by your child, how many time you fed your child throughout a day?**

R: well, first at breakfast then maybe another in 10 am, then at lunch, and another one at 3 pm, then dinner, then goes straight to bed and that’s it.

I**: okay, then how do you know when your child has had enough to eat?**

R: mmm?

I: **how do you know when your child has had enough?**

R: sometimes when he is full but I just keep on feeding him then he spits out the foods, and he doesn’t want to open his mouth again, then I know that he is full…..

I: **so, what do you do or what can you do when your child doesn’t want to eat, that really, really doesn’t want to eat, I mean not wanting to eat, but doesn’t eat or drink….what should you do?**

R: when he wakes up in the morning but doesn’t want to eat, then I will just breastfeed him. Then give him some time to make himself want to eat.

I**: what can you do to the child when he doesn’t want to eat?**

R: when he is upset, he doesn’t….

I: **yeah, so what should you do to make him feel like eating?**

R: sometimes I make him watch movie…

I**: aside from watching movies, what else?**

R: I think that’s the only thing that make him happy, also when there are lots of kids, then that’s when he wants to eat…with the kids.

I**: Do you feed the child differently when the child is sick? (like for example, the child has diarrhea) do you feed the child differently when the child is sick and not sick?**

R: I think none…

I**: he eats the same whether he is sick or not. Okay. Yeah, we are almost done. So, you have told me what your child usually eats, now could you explain to me the process, from the start to finish of how you prepare or cook a meal for your child? For example, if your child is a baby, and he eats only JOKOP, can describe how you prepare it.)**

R: JOKOP, we filled a pot with water and little rice, and wait until the rice is cooked, then add sugar and coconut milk, then wait a few times until the coconut milk is cooked.

I: **okay. Could you tell me what you think are important foods for your children under 2 years to grow well or be healthy?**

R: foods such fruits,

I**: Fruits, what kinds of fruit?**

R: apple, banana, and orange, I think those are the foods that kids usually eats nowadays.

I: **okay, aside from fruits, are there any foods you know that your child should eats?**

R: none.

I**: none, what kinds of food you should give to your child?**

R: just as I’ve mentioned before, foods that contain salt,

I: **salty foods…**

R: foods that are not good for health….

I**: could you explain to me what salt is, I mean, could you tell me some foods that contain sodium?**

R: foods like noodles and…

I**: noodles and**

R: I think noodles contains a lot of sodium and kool-aid..

I**: kool-aid…okay, so now what is the biggest influence on feeding your children?**

R: like what, exactly?

I**: like giving you advice on how to feed your child, what words of advice that was given to you?**

R: words of advice?

I**: yeah, words of advice….**

R: we don’t……..the old woman said that we don’t give huge amount of food to the child because it will harm the child’s intestine.

I**: mmm?**

R: sometimes we don’t give the child huge amount of water because it’ll cause stomach-ache.

I**: Now can you describe any differences between how you feed your male children and how you feed you female children under the age of 2?**

R: UH?

I**: Are there any differences of how you feed your sons and how you feed your daughters?**

R: no differences…

I**: none. Okay, we have 5 more question. We are also interested in the roles and responsibilities different family members play in raising children? This question is like how uncles, aunties and grand-parents play the roles of raising children. Now, could you describe the care of children throughout the day in your community?**

R: okay….

I**: how do they take care of the children?**

R: like, they’re playing with them.

I**: mmm?**

R: protect the children from any harm, like from the seawalls, the streets, and fires.

I**: who is mainly responsible for the child care?**

R: me and his grand-parents.

I: **the mother and the grand-parent. Okay, so what are the responsibilities of a mother for the child? Like you as a mother, what are your responsibilities to your child?**

R: prepare the child’s meal…

I**: mmm?**

R: give the child a bath…

I: **anything else?**

R: boiled water so the child can have diarrhea, that’s it.

I: **what are the father’s responsibilities to his child?**

R: none….

I**: [laughing] [coughing] oh, excuse me……how does the caregivers play with the children?**

R: you mean what kind of games we play with them.

I**: how you play the games with the children?**

R: we make jokes, play with balls, and the child usually plays with other kids.

I**: Could you talk about the role of grandparents have in raising children in this community?**

R: sometimes when I am gone, they are the ones who feed my child, give him a bath…..

I**: mmm…now, what makes the grandparents good? Like what good things the grandparents did to the child? What makes you say like oh, my grandma is good or my grandpa is good?**

R: I see that they are good, they are playing with the child, they do mostly anything with the kid.

I: **Could you talk about the role that other family members have in raising children in this community? like, the older sibling, or aunties or uncles.**

R: same as the grandparents.

I: **now we are at communication channels. Okay, these are last question and you are doing great. We are almost done. Now for the last section, we would like to learn about ways we can develop health programs in this community. now could you tell where you usually get information from about nutrition and health?**

R: my grandparents usually talked about which foods are good for our health and foods that contains no vitamins at all, and they also don’t want my child to eat junk food such as candies, and chip, because they’re malnutrition.

I**: mmm…now I am asking why do you trust these sources?**

R: because I can tell that they are telling the truth, I can tell that the food that they encouraged my child not to eat like candies are not good for our health, but they encouraged him to eat healthy food.

I**: now for the other question, where do this information should be deliver to, so it will be easy to see or hear them everyday?**

R: from our parents.

I: **okay. From our parents, what types of media that you usually use the most to communicate… like to make announcement or to give words of advice, so what types of media you think is easy to use……..what do you usually see everyday that people are using…**

R: you means the one we use to communicate…..

I: **to communicate or**……

R: I don’t understand…

I**: oh, you don’t understand, wait….I’ll make the question more understandable, what types of media we usually use? Example, the radio, cellphones, and internet.**

R: cellphones and internet.

I**: cellphones and internet…okay. When you think about your own parenting behaviours, can you explain what influence how you raise your children? Like, describe the differences of your parenting behaviour**.

R: I don’t think there’s any different.

I: **how about the opinions of the others in the community about raising their children?**

R: you the goodness….

I: **what others in the community think, do they tell you to do this or that**?

R: yeah.

I: **mmm**?

R: they told us not to go anywhere at night and prepare the child’s meal before the child wakes up, yeah stuff like that.

I: **So, were there any advice or information related to parenting you received?**

R: I don’t know…

I**: you don’t know….**

R: yeah.

I: **what kinds of parenting you learned…**

R: the ones that tells us how to….

I: **yeah? Can you tell me more?**

R: like what? Anything?

I: **yeah**.

R: like how to do the diaper, do the laundries. Because as for my son, I don’t do his laundries anywhere but only in the ones that are for babies only, yeah that’s it.

I**: oh, okay. Now where or who the advice or information came from?**

R: from my parents and grandparents.

I**: now for the last one, are there any desired information on parenting you wish to have but doesn’t have available? Are there anything you like to know on parenting?**

R: Yes…….

I: **so????**

R: so, I tell you like what exactly?

I**: what you really want to know about on parenting? How you take care of your child?**

R: I don’t think there is any.

I: **none, okay. Is there any other topic you like to know about or ask about**?

R: nope

I: **it’s all good? Okay. Thank you for your help!!!!!**
