# Supplementary material for: Conceptualizing multi-level determinants of infant and young child nutrition in the Republic of Marshall Islands–a socio-ecological perspective
Source: PLOS Glob Public Health. 2022 Dec 19;2(12):e0001343. doi: 10.1371/journal.pgph.0001343 (PMC10022247; doi:10.1371/journal.pgph.0001343)
Supplement: S1 Data — (ZIP) [file pgph.0001343.s001.zip › RMI Supp Data/Interviews data/I11U_IDI_HW_Hospital_Aug 14_Fela.docx]

**Interview Code:** I11U

**Interview type and Interviewee:** IDI_HW

**Interview Date:** Aug, 14

**Location:** Hospital

**Interview:** Fela

**Transcribe:** Meia

I: To began, can you tell what is you position as a worker in the hospital?

R: I am working as a nurse in the family Planning, which is in public health.

I: What usually to you do from when you start working until you finish as a nurse?

R: sometimes, I went to the clinic for the pregnancy woman to help, but mostly I just stay here wait for any patients.

I: what to you do when any patients come in?

R: so first, the patient come in and sit on the couch and I ask her if I can be of any assistant, if she said came to get birth control either the shot or the bill or the implants I find their charts.

I: Let talk about sickness, I am also interested in what kind of sickness that the children in the community usually get? Can you tell what kind of the sickness that the children under two years usually have?

R: pneumonia, there was a time I used to work in the pediatric

ward, mostly kids there admit because of pneumonia, flu, some malnutrition but it very rare.

I: what cause pneumonia?

R: I learn when I was in school that if the child if expose to cold that can cause the child to have pneumonia and can lead to coughing and fever.

I: So, you also mention flu and malnutrition, can you describe where did the child get the sickness and what makes them sick.

R: Mostly like every year there are always the flu sickness and for the malnutrition, I don’t know why the kids are having malnutrition, but there was a time while I was working in the pediatric ward, there was this mom who bring her kid because the child was malnutrition and the doctor diagnose her to severe malnutrition and the mom said that her child didn’t consume food for like a long time and she didn’t bring her child to the doctor right away until the child like freeze.

I: Base on your knowledge, can you tell me what are the seriousness of these sickness?

R: yes, malnutrition is serious because if you have malnutrition you body can’t function well because you are not eating nutritious foods, as for the pneumonia some die from it, yes and I have an aunt who died from pneumonia in this hospital

I: what are some ways to prevent these Sickness or what can we do to stop pneumonia, flu, and malnutrition?

R: I think it would be better for us not to keep us expose to the cold, like for example, using electric fan for one is not good, don’t keep us expose to the cold too much, and for flu I think as a nurse if I have the flu virus I have to wear like mask so I won’t spread the virus and for the malnutrition I think is better If we like make some lists awareness for the parents to educate them about health and the young teenagers for I know that today many teenagers nowadays get pregnant.

I: what are the treatments for these Sickness?

R: For the pneumonia the doctor admitted the child to the hospital and treat him/her with medicine, and flu I tell them to drink a lot of water and so the sick person like heal from flu sickness in one day.

I: So, you mention drinking a lot of water, how do you think the water help the sick person?

R: A lot, I think the water base on my knowledge I think the water drain out the virus from the sick person, water is very powerful. Then sleep a lot.

I: can you explain what type of treatment people in your community seek for their children, for example traditional healers, doctors, nurses?

R: Come see the doctor what else? is it about the sickness that I said?

I: No any kind of sickness in the community that the child has?

R: Some go the traditional healer I have heard, and I also have seen some go the traditional healer, like Marshallese medicine with some big cases like stomach pump, and the child like getting skinner and skinner so the parents bring the child to the traditional healer. But the parent mostly, bring the child to the doctor for help.

I: who do they go to first for healthcare and why?

R: The doctors.

I: Can you tell me about any challenges your community faces in seeking treatment for the illnesses you mentioned before?

R: what the challenges, can I said I don’t know?

I: it okay, don’t worry about it, there are so many questions that some cannot answer them, and there are no right or wrong answer. Can you tell me any difficulties you face in providing health services to patients?

R: In my working place or what?

I: Yes, it can be in your working place.

R: what are what, I think there are none.

I: there are no difficulties that you face?

R: hmm hmmm

I: Can you describe any illnesses associated with nutrition that affect children in your community?

R: I really don’t know.

I: Base on your knowledge, can you describe what are some foods that can make a child unhealthy? And why?

R: What kind of foods that can make a child unhealthy, oh sweets and oh healthy like the 5 nutrition should be in their food?

I: yes.

R: Oh, rice it has a lot of carbohydrate, chicken it too greasy if they fry it what else, oh and ramen like for example in my house if we like oversleep to cook food there is always ramen in packet in my house which are always ready to go. But if we like feed too much ramen it not good for their health for there are chances that something might happen to them, if they eat too much ramen for it too salty, greasy and it too much carbohydrate plus it was process in in the factory machine and it not healthy.

I: Can you tell me if there was anybody you know from you family has been sick from eating these foods?

R: can I mention stroke, because in my family we have stroke it like we inherit the stroke Sickness, but it can come from what we eat, yes, I have a grandma and my father is also stroke my father’s mom was died because of stroke, I don’t know it maybe from what we eat.

I: Can you tell me what type of foods that make a child’s body healthy and reasons why?

R: Maybe it better if they eat like between eating hours a lot of fruits and foods have a lot of nutrition.

I: Can you describe what kind of fruit that the child mostly eats everyday?

R: My baby always eats bananas and papaya because in my house we bananas and papayas and pandanus so, these are the fruits that my child mostly eats.

I: We talked a lot about being unhealthy. Could you now describe for me a typical a day of someone living a healthy lifestyle, from the time they wake up in the morning until when they go to bed?

R: Happy, because their body already finish having what the body need.

I: Like what exactly?

R: Like, what our body need?

I: yes

R: Like for example the 5 nutrition, to be heathy person, the person had to have all the nutrition in the person’s meals and drinks.

I: can you voice out all 5 nutrition?

R: Water, fats , carbohydrate, protein and vitamin and minerals.

I: Can you describe the appearance/signs of a healthy child under 2 years?

R: They are very active, they look happy they don’t look sad or unwell. And what else, I think that about all I know.

I: Can you describe the appearance/signs of a healthy adult?

R: what age of the adult is it old people or?

I: All adult

R: They look unwell while they are moving around and there isn’t any sickness in their body like diabetic, when we look at them their bodies are still whole they haven’t cut off their legs and they are not blind.

I: I have one more set of illness questions but related to women’s health now. Could you tell me about your experiences with women who have anaemia?

R: Women that have anaemia, like what, what should I say?

I: Like, like from your own word.

R: What we would do or what

I: Like what are doing or what make them have anaemia?

R: For the women to have anaemia is because they have sickness.

I: So, if the have sickness what makes the have anaemia their sickness?

R: Because in body it takes days to__ hmm how I say this process? And then there are some virus in which consume our blood, and for women they might lose a lot of blood during their period. What more could I say?

I: you doing good, but can you explain more about the virus that consume our blood?

R: Cancer, cancer is one the reason why our body have not enough blood in our body.

I: Can you tell me if women who have anaemia think it is a serious concern?

R: yes, because they are the one asking help for giving them blood if they don’t have enough in their body.

I: Can you tell me the causes of anaemia in women of reproductive age (and pregnancy

) ?

R: For a pregnant woman, she need to take some supplement for blood because there is another person in which she is carrying inside her, and for some girls uuhhh, I don’t know maybe they are not eating enough food that can help give them enough blood. It can be because the lost a lot of blood during pregnant.

I: Can you describe the advice given to the women for prevention and treatment of anaemia?

R: First of all family Planning, the reason I said family planning is that it is about all kind of sickness, if you been to like family planning clinic you might won’t have any sickness, for you know those women that lost a lot of blood it can be because they have a lot kids, and they didn’t like have any family planning. It might not from lost a lot blood but other kinds of sickness, if they like have a family plan there might be like several years cap for them to have another child, like for example the implant for the 5 years, if the have the implant it is easy for their body to function well like before and like have a healthy body again.

I: Can you talk about how long after birth most women start breastfeeding in your community?

R: Base on the studies and during when I was in school, I learn that after a baby is born you first lay it on the mother for a while then you take the baby and left it in and a temperature room for a while to keep it warm for like maybe 10 to 15 minute, but you check him/her from time to time and see if the baby is warm enough and if he/she cry if take it to the mother so she can start breastfeed the baby.

I: Am I right that, that is the signal that the baby need to breastfeed?

R: yes, as I know that a baby can not talks, he/she can’t tell the mom that she/he is hungry, but the only the baby can do is cry. And the only the mom can do is give her breast to her baby to be feed.

I: Can you explain if any liquids other than breastmilk given to the baby in the first few days after birth (and reasons why)?

R: ok, you know I am talking about those premature baby they need to be in the temperature room, ok we gave them of course vitamins, and other thing they mix it with the milk that need to feed to baby is the virgin oil, this virgin oil has a nutrition that help the premature baby keep his/her weight going up. And it also prevents any sickness that the premature might get. But for those baby that are healthy I think they only breastfeed them.

I: So, the milk that used to mix with the virgin oil, where does it come from?

R: It from the breast milk of the mom. So, a baby in the temperature room, either the mother went inside the room to feed the baby or she pump milk from her breast to a small bottle and give it the baby.

I: Can you explain exclusive breastfeeding practices in this community?

R: I mostly see women lay down and breastfeeding their baby and when I see that happen I told them to please not to lay down and breastfeeding their baby, for you know if you lay down and breastfeed your baby you baby like lay really flat and that can make water goes into the lung, and the baby have water in their lung can make them have sickness but the first sickness they might get is pneumonia.

I: So, there are some reasons that the baby can have pneumonia?

R: yeah.

I: Good, thanks you for these information, your answers are good, so now, can you tell me if there any liquids other than breastmilk given in the first 6 months after birth (and reasons why)

R: Water

I: why are they giving water?

R: Some mother says that their babies are thirsty, that why they are giving their baby water, but giving only breast milk to the baby for these first 6 months is good for the baby.

I: Can you tell me the difficulties faced by mothers in your community to practicing exclusive breastfeeding for six months?

R: There are none, because breastmilk is free unlike formula you buy and you also but water if don’t want to boil water.

I: Can you tell me if there are some specific ways to better support mothers to exclusively breastfeed for 6 months?

R: Maybe if we explain to them the important of holding a baby and breastfeed them they might have little chances of getting sick.

I: We are trying to understand how people eat in this community. Could you describe in detail what most families usually eat and drink throughout the day?

R: My house, for example there is always rice but if we like tired of rice we bake bread, we eat rice and meat and my parents always told us to drink water, they don’t like us to drink cola or any other soft drinks.

I: So, you talk about meat, can you tell me what kind of meats that your family eat?

R: Chicken, canned meats, fish oh chicken number 1 and second one is fish the reason I said fish is second one because I have an old man for a neighbor who always go out to fish and he always give us fish after he fishes.

I: So, the second one is fish, if there are no chicken in your house then your family eat fish.

R: Yes.

I: Can you explain the process of how the meals are made in your family?

R: We cooked them, oh mixed chickens, we also boil the chicken with soy sauce, for fish we sometime boil them with water or we cooked them on fire and some time we eat them raw.

I: Can you tell who in the family is served first, next and last?

R: My father, he is the one we always prepare his foods first, even if he at work we always prepare his food and store his foods.

I: Can you tell me whether there are differences in the foods served to different family members?

R: there none

I: Can you tell me any differences in quantities of food served to different family members?

R: There are now, hm like different size of foods? Hmm mostly because like my father because he is stroke survival, now he is okay, and he can move around, and every like morning if we cook him oatmeal we also prepare some bread to give him with his oatmeal, he like eating bread with his other meals and some green vegetables.

I: So, the answer for this question is that the healthy people have the different size of foods from the unhealthy people.

R: yeah.

I: Can you tell of some children receive more food than others?

R: I think there are none

I: Now could you describe any food sharing between family members during mealtimes (for example children eating together separately from the family, meals eaten from the same plate by all family members)?

R: Each person has his/her plate

I: Can you tell me if your house is sharing any foods with your neighbors?

R: Yes, foods that are rare like BBQ chicken, fish that are cooked on fire, but for fish if there a many we share them with our neighbor.

I: Now I want to know about how young children eat in this community. Can you describe in detail what children under 2 years commonly eat throughout the day?

R: In my house?

I: It can be in the community or your house base on your knowledge child eating under 2 years.

R: Under 2 years?

I: Yes

R: Children in my house they eat rice and meats.

I: Can you tell me what kind of meats?

R: They eat any meats that we adults in the house eat.

I: Can you tell me whether children are typically given snacks between meals in your house?

R: What kind of snacks, the one that we buy from store or the snacks we give from our house?

I: Any snacks that you give them?

R: Yes!

I: Can you explain if whether children are fed differently when they are sick (eg. When child has diarrhea) in your house?

R: Yes, when they are sick they like picky or choosey when it come to food, like “ I don’t want that, I wanna eat that,” but Laura is very far and there are no big store to buy what they want if they want oranges or an apple we have to come all the way here to buy it, but when they are sick, they eat any things.

I: So, when they are well they are not picky, they eat any things, right?

R: Yes. They will eat any foods given to them.

I: Can you tell me any differences in feeding practices between girls and boys under 2 (and reasons why)

R: None

I: Can you talk to me about what influences how families feed their children in this community?

R: I would have to say hours, because in my house like for example, we eat before 9 am in the morning and we eat lunch at exactly 12 pm and we also eat dinner at 7 in the evening. But, I don’t know about other family in the community.

I: What if someone in the family is not hungry at the meals times, what to you do?

R: Nothing, we don’t do anything. But if that happen the person will make his/her foods. We usually don’t cook when it ‘s pass 9 pm. The foods need to be ready before 9 in the evening.

I: We have heard from some families that eat local foods and others that eat processed foods. Could you explain what is typical for most families in this community?

R: Marshallese foods like bananas, pandanus, aikiu[coconut meat(iu) with flour, water and sugar], aerice[coconut meat(iu) with rice and water and sugar].we also eat processed foods like, chicken and canned meats and meatball in canned.

I: Can you tell me anything that makes it difficult or easy to cook local foods?

R: It might be difficult because the cooking woods might wet because it been raining and then there are no pro butane gas to cook the foods. But about anything that makes it easy I really don’t know.

I: What are the positive or negative things about eating local foods?

R: The negative thing about local foods is that it takes long to cook it like take a long while to finish cook it, and it too much work.

I: Can you explain what you mean by too much work to prepare it?

R: The reason I say it a lot of work because like for example aikue or the aerice, you have to go look for some coconut meat(iu) then you use something sharp to get the (iu)coconut meat from the coconut skin, you crack in half and use a knife to take out the meat, you gather some fire woods to start a fire if you have no pro butane gas to cook you grain the coconut then you cook either with flour or rice. Oh, we also need to make a coconut milk for the foods. But, after we cook on the fire we clean the pot cleans so it won’t stay black.

I: Those were the difficulties so what about the easy?

R: Very delicious, but when I was a child people keep saying our food are more nutritious, but I think our food are not different because it has carbohydrate too same likes rice the only different is it not process and we take them from the trees.

I: Can you tell me any suggestions for balanced meals that can be prepared with locally available ingredients for children under 2 years?

R: How is it? My friend. How will we explain foods that are nutritious?

I: yes.

R: What can I say? I don’t know (laugh) do I need to say I don’t know

I: It okay, don’t worry, there are some questions that some people can’t answer, it not only you. Now I would like to talk about pregnant women in this community. Can you describe their diets during pregnancy?

R: I don’t know about some pregnant women but the one I know I usually see them eat salt, because they say it help them with morning sickness.

I: Are there any other foods?

R: No. I don’t know.

I: That fine. Can you tell me whether pregnant women usually change their diets during pregnancy? Did they like change what they are eating?

R: Like change their foods because of what us health work told them? Yes, we told them to eat a lot of nutritious foods and foods that have a lot of iron, they had to watch what they are eating because of the unborn child.

I: Can you tell me about the foods that have irons?

R: Red meats and that all I know.

I:(laugh) What influences women’s diet during pregnancy?

R: Their hormone influences them.

I: What foods women are encouraged to eat during pregnancy and why?

R: For local food we tell them to eat bananas pandanus etc…

I: Who encourages or discourages eating those foods during pregnancy?

R: The doctor, and we nurse explain to them more why it is important to eat nutritious, because the doctor can only say eat bananas for it important for your health, but he/she will not tell you why it is important. And the nurse is the one telling why it is important and how it important.

I: Can you tell me about any supplements normally given to women during pregnancy?

R: Vitamins, medicine for blood and medicine that help the baby grow it like a supplement or like a vitamin.

I: What prevents women from taking their supplements?

R: They cannot shallow it and if they shallow the always vomits right after, they also don’t like the small, financial it not a problem for these medicines are free. Except if they ask for any Tylenol or amoxicillin then they will pay.

I: Can you tell if pregnant women are drinking alcohol, smoking and using other drugs during pregnancy?

R: I don’t know.

I: Can you now describe women’s diets during breastfeeding in this community?

R: They eat fish, they eat boil, fry and sashimi fish because it is ours believe that fish are good to produce breast milk. But in truth, Ours breast milk is produce from our brain but it take dedication for the breastmilk to produce and as a mother during breastfeeding you have to hold your baby and focus on the baby and nothing else.

I: Can you tell me whether women usually change their diet during breastfeeding?

R: I don’t know

I: Can you explain to me the foods that breastfeeding women are encouraged or discouraged to eat and reason why?

R: The doctor and it can be their family, they might tell them not to eat unhealthy foods.

I: As a health worker, what are some of your biggest concerns of the diets of pregnant and breastfeeding women in this community work in?

R: I don’t know because I don’t know what they are eating, but as for my friends during pregnant they eat kool-aid and I always advice them that it not good for the baby and them but they laugh at me and tell me I am lying and they also tell me that just because I work in the hospital I can tell them lie. Some just don’t mind what I tell them.

I: Could you explain where community members usually get trusted information about nutrition and health?

R: From where I work.

I: Why did you trust this source?

R: Maybe because it health and everything about it in hospital.

I: Can you tell where nutrition and health messages should be delivered so that community members would see/hear them most easily?

R: The people or the public so it easier for them to hear and know.

I: Can you tell me the types of media that community members use the most to communicate (eg. Radio, online apps, websites)?

R: Local radio.

I: For the last question, could you describe what influences how people raise children in this community?

R: I don’t know

I: IF you have kids what influence you to raise your kids?

R: I have friends and I have aunty who have boys, but they want baby girls and they like asked the baby they know who have baby girls to give them their baby girls to adopt.

I: Can you tell me any specific advice or information related to parenting typically given to the community member? For example, as a nurse is there any advice you give to the parents?

R: what can I say? Maybe I will tell the parents to please take care of the child, but if I tell them that they might think that we don’t trust them with their child.

I: Can you tell me any information that pregnant or breastfeeding women typically ask for from health workers?

R: They usually ask about how far pregnant are they and if they can do something (any things they want) during this months of their pregnancy.

I: Any other information?

R: I don’t know.

I: Can you tell me what he/she thinks is the best way to communicate with caregivers about health?

R: What! Maybe we need to educate them about health and the important of health and maybe they might do what we educate them at home.

I: Is there anything else about the topics we talked about today that we missed or that you would like to tell us about?

R: There are none…
