# Supplementary material for: Conceptualizing multi-level determinants of infant and young child nutrition in the Republic of Marshall Islands–a socio-ecological perspective
Source: PLOS Glob Public Health. 2022 Dec 19;2(12):e0001343. doi: 10.1371/journal.pgph.0001343 (PMC10022247; doi:10.1371/journal.pgph.0001343)
Supplement: S1 Data — (ZIP) [file pgph.0001343.s001.zip › RMI Supp Data/Interviews data/I12U_ IDI_HW_Hospital_Aug 14_Fela.docx]

Interview Code: I12U

Interview type and Interviewee: In-depth Interview

Interview Date: August 14 2018

Location: Majuro Marshall Islands Hospital Centre

Interviewer: Fela

Transcriber: Fela

**I: OK. Before we start, I am asking you if you would tell on the recorder that you agree in this recording survey. Do you agree?**

R: That is fine with me

**I: Thank you**

**I: Thank you for giving me this time to speak with you today. The information we learn here will help us find ways to improve maternal and child health and sanitation in our country.**

**I: To begin with can you please tell me about your job in this health centre/nursing station?**

R: I am nurse patient

**I: Can you please explain more on how do you nurture your patient or what do you do to them?**

R: Be there and nurture them in terms of needs, pain, dizzy and what I supposed to give them, but before I give them supportive medicine, first, I would call the doctor and medicine given with information following with the hours that they supposed to take them

**I: Now the other question says, what is your typical day from morning until evening here as a health are or worker?**

R: be there to in terms of patient to needs and wants, giving them their medicine..

**I: Yes?**

R: Responsible for the doctor’s order and carry them out.

**I: that’s great. Let’s now talk about illness. I am specifically interested in illness that children suffer from. In this community, what illnesses would you say children under two years commonly suffer from?**

R: Like… fever, diarrhea, dizzy, some child do, and yeah I thinks these are the illness.

**I: You mentioned fever? What are the causes of fever?**

R: Some children under two years old?

**I: Yeah**

R: We Marshallese often called it, sickness between months.

**I: Yeah? Between month?**

R: Yeah like before they can do or able to do something, or the sign of beginning of doing something. Yes. That is the cause of “between month” as in sing of a child to do something or can say a word, its fever, and diarrhea

**I: And what about dizzy?**

R: As of that, I think is comes in both with fever and diarrhea.

**I: Now can you tell me what the seriousness of each sickness you mentioned are?**

R: Fever... if the fever is too high, the reason why you would know that the fever is comes inside, the hands are too cold, and also the feet. Then you would rush and do something like give him medicine or use the piece of cloth to suck out the fever.

**I: Now can you tell me on ways to prevent each one of these illness you mentioned in this community?**

R: Medicine

**I: Yeah medicine**

R: and for diarrhea, children who have diarrhea, they should drink water a lot, so that it cannot cause dehydrated. We both know that children now a days often get sickness of dehydrated. If you are not able to take care of the children, he or she should be taken to the hospital.

**I: Can you tell me more on dehydrated? What cause the dehydrated?**

R: When they got diarrhea and they poop non -stop, that is the causes of dehydrated when they are having diarrhea.

**I: HMMM**

**I: Now can you explain what type of treatment people in your community seek for their children, for example traditional healers, doctors, nurse? What do they seek most often?**

R: As we Marshallese people, some people do not really trust in bringing their children here, or health care, But some, as a mother of a child, it seems like they do really trust or believe in traditional heal or Marshallese medicine. And then I believe each and every one of us have different believe.

**I: Now you explain about Marshallese medicine or traditional medicine same like you explain medicine from this hospital. That’s a very helpful answer.**

**I: Now can you tell me who are the first people mothers bring their sick children when they are sick and reason why? Who are the first people mothers bring their sick children for?**

R: As of our age? You and me?

**I: hmm?**

R: We usually bring them to our mother, or the grandmother or the grandfather of the child. Because as of our age, we never know ways to heal the baby from sickness because we are still young and we haven’t had any ideas on babies’ sickness to be treated.

**I: great. That’s really answer my question.**

**I: It says, can you..? The next question says. Can you tell be about challenges your community faces in seeking treatment for the illness you mentioned before?**

R: um. Today, some people have money, and some do not and the most challenging thing in this community is when you do not have or as of bringing a child to see the nurse or doctor, some people are scared or shy to come because they do not have the money to buy the medicine or they do not have money for taxi to bring their babies to the hospital

**I: Now can you tell me any difficulties you face in providing health services to patients?**

R: hmm... Difficulties by providing health services to patients? Hmm.

R: Difficulties... Like for an example for me. Sometimes when you do no really know how to communicate with the patient’s attention, because when you trying to give something for the patient and if they do not want to, especially when they do really need to take it at that exact time,

**I: hmm?**

R: You really don’t have power to go straight to them and tell them that they should take the medicine right away because the choice is theirs

**I: So you mention that the choice is their own, what you they are really sick and they do really need to take the medicine right away? What can you do in order for them to be able to change their mind and can take their medicine?**

R: You have to be really good in counselling a person or a patient. You have to be good in giving good explanation on why you do really have to give them the medicine or tell them that it is really important for them to take it because.. Something like that.

**I: You are doing really great. You answer the question clearly thank you.**

**I: (clear throat) that is exactly the level of detail I am looking for in your answers- thanks you- Can you describe any illness associated with nutrition that affect children in your community?**

R: Foods that are?

**I: Nutrition food and can affect children when they eat in this community.**

R: Some children, for example the banana. When they eat banana, they can’t poop for a long time.

**I: Now this question is about types of foods that make a child’s body unhealthy and reasons why?**

R: If you keep feeding the children from foods bought from the store and less from local food that makes a child not grow fast, slow in moving, because the problem is that a mother of a child always give junk food especially when a child is not having breakfast but parents can just give them unhealthy foods.

**I: Now this question says. “What types of foods that make a child’s body healthy and reason why?**

R: hmm what can I say? Local foods or foods we have here in the Marshall Islands and are really healthy.

**I: Can you tell me more about local foods like what kind of foods?**

R: Like pandanus, coconut meat, breadfruits, foods that now days we Marshallese people start hating our own foods.

**I: Good. Moving on. We talked about being unhealthy. Could you now describe for me a typical day of someone living a healthy lifestyle, from the time they wake up in the morning until when they go to bed?**

R: Like a child?

**I: Can be a child or can be anyone. Some one that’s being healthy.**

R: Someone that is being healthy is someone that balance the hours of sleeping throughout the day. Go to bed at exact time to bed and when they wake up they won’t feel any sickness, not like someone that have lack of sleeping hour by staying up late and got only two hours of sleeping, it causes themselves to feel headache or any kind of sickness.

**I: So briefly you explain that they will feel better?**

R: Yes. When someone has overall hours of sleep for the night.

**I: yes have enough hours of sleeping**

R: If he or she eats healthy foods, and having meal at exact time.

**I: that’s great. Now can you explain the appearance or signs of a healthy child under two years?**

R: Appearance for child under two years old or one years old?

**I: Under two years old … signs of a healthy child under two**

R: When you look at the child and see that he or she plays the whole day, he or she is not sleeping or taking nap frequently but would keep playing. Not like some children they feel sick and when a child eat nutritious food, or the mother of the child feed the baby exact meal time or bed time.

**I: Now what are the appearance or signs of a healthy adult?**

R: Healthy adult…… When you look at an adult person that is healthy, it is because he is can do house chores, moveable, smiley face, not a sad face, every hour he or she is happy.

**I: So you mention chores, can you explain more on what kind of chores they do?**

R: One good example from us women, we clean around the house, we have clean cloth and yeah something like that.

**I: I have one more set of illness questions but related to women’s health now. Could you tell me about your experiences with women who have anaemia?**

R: Women that are having anaemia, when you look at a women who is having anaemia, you will see that the lady is getting sick and even got worst each and every day. And today, mostly women got anaemia comes from unhealthy nutritious food, some women also got anaemia by having monthly period without stop.

**I: Now can you describe more on what makes a women having nonstop monthly period?**

R: There are some women who have wound in their important spot (vagina) the wound is the reason for women to have nonstop monthly period.

**I: Ok.**

**I: So can you describe if women who have anaemia think that this is a serious concern?**

R: Some women really don’t think that this sickness is a serious sickness but others do care and they believe that it is a really dangerous sickness.

**I: Could you explain causes anaemia in women of reproductive age and pregnancy?**

R: As of young mothers, sometimes the problem comes from themselves when they don’t clean their important spot of their body.

**I: So that is the cause of anaemia/?**

R: hmmm

**I: Now are there any advice given to women for prevention and treatment of anaemia? Like what are some helpful advice that can help prevent them from anaemia?**

R: The first thing that I would say is “ if they feel it or know that they are having nonstop monthly period, it is important for them to come for medical check-up and if their result tell them that they have sickness like anaemia. Another thing is that, some women even though they are having monthly period, they are having sex with their partner and that is also one reason for having monthly period and what I would advise them is to limit themselves a little bit of having sex frequently with partners.

**I: That’s great. Now can you tell me if there are any advice given for women…. Hmmm I am really sorry I asked this question before. ..Moving on, now I would like to talk about breastfeeding practices in this community.**

**I: Can you talk about how long after birth most women start breastfeeding in your community**?

R: From the time the baby born?

**I: Hmmm.**

R: Long breast feeding when the baby born the mother should start breast feed the baby until two years old. Today, many mothers out there stop breast feeding their baby from few months to one years, they would start giving them different liquid using the baby bottle.

**I: Now the other questions is like that, except the exclusive breastfeeding, you mentioned other liquid?**

R: Hmm yes.

**I: Now can you tell me why they give other liquid than breastfeeding them?**

R: For example, some women say that the child eat from their breastfed, and they don’t really get full milk from them and that is why they give different liquid for the baby but the best food for a baby is the breast milk.

**I: Now could you discuss any difficulties faced by mothers in your community to practicing exclusive breastfeeding for six months.**

R: can you repeat the question?

**I: Can you discuss the difficulties faced by mothers in this community when they do exclusive breastfeeding for six months?**

R: What are the difficulties?

**I: hmm. Like what makes it difficult for them not to do breastfeeding.**

R: Like is said before, some mothers says that they don’t really contain enough breastmilk to feed the baby. Other mothers would say that they always feel sad and worry with the father of the baby and would not give breast feed to the baby. When the parents separate, or divorce, the father can either take the baby from the mother, that’s also when the baby don’t get breast milk from the mother.

**I: Ok, Now are there any specific ways to better support mothers to exclusively breastfeed for six months?**

R: hmm … like what…

**I: Are there any specific ways that health worker should support mothers to exclusively breastfeed their baby for six months?**

R: As of the mother of a child, when they come to the hospital, nurses always support them and show it to them how to feed the child by breast feeding and why breastfeeding is really important for the child. And health worker always remind the mothers that the breast milk of a women to the baby is really important than the liquid given for the child. It is what we see from a child that has been breast fed by the mother, when you look at the child, he or she grow fast and healthy. The child will not have pink eyes and all kind of sickness, but would feel really healthy.

**I: Healthy**.

R: Hmm yes.

**I: you mentioned by exclusively breast feed from the mother, the child is really healthy yes that answer the question. Thank you.**

**I: Okay, moving on to the next question. Now we are trying understand how people eat in this community. Could you describe in detail what most families usually eat and drink throughout the day?**

R: alllriight! They usually eat tuna, today, money is not enough so some people cannot afford healthy food. Now they usually eat hot dog, tuna, some people here in the Marshall Islands, example here in Majuro, sometimes they would eat only rice without meat.

**I: Okay. Are there anything else?**

**I: Like what do they drink every day?**

R: Today in this community, people like to drink soda. Like most of the important event people would bring soda for drink. They don’t drink water frequently than drinking sweet drink.

**I: Yeah that might be right.**

**I: Now as of the meat they eat or their everyday meal, what is the process of how the meals are made?**

R: Some families do not cook the meal, as of the tuna, we do not cook the tuna, they can just get it from the store and bring it and eat it with the rice mix it the soy sauce or tobacco and salt all these together they mix them with the rice and the tuna. Also can be the same like the hot dog, they bought it from the store and can eat it without cooking it.

**I: hmmm. Yes that is right.**

**I: Now can you tell me who in the family is served first, next and last?**

R: The person should be served in a family, is the land lord or land owner if any. Or can be the sick people in the family especially people who are having diabetes. Second is the child. These are the main people we should serve them first.

**I: Hmm yes that right.**

R: And then lastly, we people. Can be anybody.

**I: So you mentioned the land lord or the land owner, if there is no land lord or land owner in the family, who would be served first in the family.**

R: The children

**I: hmm ok the children.**

**I: So now are there aby differences in the foods served to different family members? People in the family, are there any differences by serving food for them?**

R: hmm….. If there were two or three couple in the family. Today we are no longer together as a family, different family in one household have separate meals to serve themselves. Wwe are no longer sharing food to each other. *Jake jebol eo* (sharing is caring)

**I: No more “sharing is caring”**

R: hmmm yes.

**I: so people eat separately?**

R: Yes people eat separately

**I: Now are there any differences in quantities of food served to different family members?**

R: There are is a different when people eat because some eat more than the other.

**I: Can you tell me more on people who eat more than the other, what they do?**

R: They get foods more than enough so that they can have full stomach. Like they never think about other people but they would just get foods depend on their diet.

**I: Now do some children receive more food than others?**

R: Yes some children eat more than the other. So the child that eat more, you can feed him/her twice, you can fill the plate one time, and then when the child is not full yet, you can fill the plate for the second time.

**I: Now could you describe any food sharing between family members during mealtimes (for example children eating together separately form the family, meals eaten from the same plate by all family members)?**

R: Some parents don’t want to share the same food with the children, they give their children’s food in separate plates

**I: Ok. Now do the people in the family share food between the households? Like to their neighbours**.

R: Today, some people share food. If you have enough food, you can share it to your neighbour or your friend live next to you. Let me make a good example of me.

**I: hmmm?**

R: People next to my house, if there are food or local food come from the outer islands, I usually share with them.

**I: hmm so you share with them**

R: Yes I do share with them. Like my neighbour.

**I: What about just any kind of food?**

R: Well these kind of food, you do understand that they can get them anytime anywhere, I only share foods that we don’t get or have them all the time.

**I: So you don’t give foods from the store or?**

R: yes I don’t give foods like that because they can get them here only local food or uncommon food that we don’t have them here.

**I: ok. Now I want to know about how young children eat in this community. Can you describe in detail what children under two years commonly eat throughout the day?**

R: Today mostly children eat only can meat. They usually eat food from the can meat. They don’t really eat cook chicken, fish, they don’t really eat them every day. Only if the parents of the children can afford the food, the children can have healthy food. If the parents can’t afford enough, so they only eat cheap food.

**I: Now can you tell me how many times a day meals are eaten by children under two?**

R: To be really sure about that, they should eat only three times a day. It needs to be only in the morning breakfast, lunch, and the dinner.

**I: hmm**

R: But today. When the child parents don’t have the money to feed the child in the morning, the child can wait and fed by noon or during lunch.

**I: That is great. You answer my question. Now moving on, the question says, “do children typically given snacks between meals?**

R: Yes

**I: Moving on... Do children fed differently when they are sick from the time they are not sick?**

R: Yes

**I: What makes it different from the time they are sick than the time they are not sick?**

R: The time the child is sick…..

**I: hmmm?**

R: The child refuse to eat. He is picky at that time he/she sick. So what you do when the child is sick is look for what kind of food they would want to eat.

**I: Can you tell me about the time the child is not sick?**

R: When the child is not sick, he/she can eat the foods they would want to eat.

**I: You mean any kind of foods?**

**I: Now can you talk to me about what influence how families feed their children in this community? (Asking repeatedly)**

R: you mean, advise or support a mother so that she can feed the child or? Because when some mother refuse to feed their child they can’t feed them no matter what.

**I: hmm... So what are some ways to help support them to feed their children?**

R: We should talk to the mother and tell her that if the child is not eating, it can cause the child to be sick. Today, many children do not eat and they feel weak. Now when you look at the child, there is a big change, he/she is not moveable, we should encourage the mother “you should feed your child” is already meal time.

**I: Like giving advices**.

R: Yes always give her advices.

**I: ok now moving on…. We have heard from some families that eat local foods and others that eat processed foods. Could you explain what is typical for most families in this community?**

R: Local or process food?

**I: Both. Local and imported foods.**

R: When is come to local foods, they usually eat breadfruits, banana, pandanus, and yes I think these are the foods I usually see them eat. And when is come to imported foods, today, people usually eat noodle, or the ramen.

**I: hmmm so they should not eat ramen.**

R: Yes at workplace and the community are the same people eat ramen.

**I: So you usually see that they eat ramen every day**

R: Yes

**I: Now can you tell me what makes it easy or difficult to cook local food?**

R: long pause

**I: What makes is easy.. And what makes it difficult to cook local food.**

R: hmm what makes it easy to cook local food? Ok, as of local food, the easy thing to do is using the grill or make fire to cook them so that they can be cook real quick. What makes it difficult is... For example here in Majuro, when you start your fire, your neighbour would complain because they don’t want the smoke of your fire so you won’t be able to cook the local food.

**I: Now can you tell me what the positive things about local foods are? Or why local foods are good?**

R: The reason why local foods are good is that, they give us good health. They help prevent us from diabetic sickness, high blood, heart diseases. Diseases that are common today.

**I: Are there any suggestions for balanced meals that can be prepared with locally available ingredients for children under two?**

R: Can you explain it more?

**I: What are people’s suggestion for balance the meal together with local food available ingredient for children under two?**

R: A mother should….. They should practice giving real foods for the baby because some child can’t eat the pandanus like the taste of the pandanus. The mother can practice giving the food from the time the child can eat real meal in order for the child’s growth, they can eat easily eat the food.

**I: Now at that time when the mother start giving food, does she give the food or does she have to soften the food and feed the child?**

R: Yes. She need to soften the food and feed the child.

**I: Alright. Moving on to the next question. Can you talk about what messages about breastfeeding and complementary feeding you give to mothers or other community members?**

R: Pause…

**I: About breastfeeding and complementary feeding mothers take from you. Why is it really important for them to take food like these foods?**

R: A mother?

**I: Yes during breastfeeding.**

R: A mother should be eating foods that provide milk.

**I: what kind of foods?**

R: Foods that are fully contain with nutritious.

**I: Can you tell me more on what kind of foods?**

R: From what we believe, we should be eating meat like fishes and something like that. Compared to foods we bought from the store, they usually eat the corn beef so that corn beef and fish can provide breast milk for a mother so that they can be able to feed the child.

**I: Now next question says... … it says…it says. …ow sorry I already asked this question I am sorry.**

**I: Are there any nutrition education activities with community members as part of health work? Or can be part of your health work, do you help spread the health and nutritious message to people like what would you do or say to them in terms of local foods?**

R: For example, we the nurses, we usually see our patients are full with tuna or can meat, and the patient helper or the people who is there with the patient give them the food they want to eat. And we always tell them that the food that are given to them from the hospital, are the best food for their health.

**I: Are there any difficulties to deliver nutrition messages to caregivers?**

R: Yes there is because some people when you discuss to them about the foods they would refuse to listen to you. They only want to get the food that they just want to eat it. Let say, they would go ahead and eat the food they want it.

**I: So you meant they only follow what their brain tells them that they want to eat?**

R: Yes

**I: Ok that’s fine. Now I would like to talk about pregnant women in this community. Can you describe their diets during pregnancy?**

R: For some pregnant lady, as we both know, we are too picky and we pick different food we want to eat. Some might hate the food and would never want to eat during pregnancy. Now some want to eat not real meal or junk foods like piknik and something like that.

**I: You mention piknik, what else?**

R: Piknik with cook aid powder. And some pregnant lady’s partner or their parents force them to eat healthy food so that the child can be born healthy.

**I: Can you explain on foods women are encouraged to eat during pregnancy and reason why?**

R: Foods that pregnancy women should be eating are food that we have here in the Marshall Islands or local foods.

**I: Like what?**

R: Like buwiro ( made our of pandanus tree) fish, and when it’s come to here on Majuro, they should be eating apple, orange, and things like that or just healthy foods. Foods that we know and understand that they are good for our health.

**I: Now can you explain whether pregnant women usually change their diets during pregnancy?**

R: During pregnancy, some women are too picky. Some get dizzy, and don’t want to eat this and that, they only want to eat the food they just want it. It is different from one another pregnancy women change their diets during pregnancy.

**I: So what kind of food a health worker or a nurse should advice the pregnant woman to eat? What kind of food they should eat and why they should be eating the food?**

R: A health worker or a nurse should advice the pregnant woman to eat nutritious food or healthy food.

**I: Ok. Healthy food.**

R: These foods are not only important for the mother of the baby, but it is really important for the baby’s health.

**I: Can you discuss on what foods women are encouraged not to eat during pregnancy? And why?**

R: Like the cool aid powder, salty food, they should not eat ramen or noodles.

**I: Now during pregnancy, who encouraged or discourage eating those foods?**

R: Health or nurses and the family members.

**I: You mentioned the family, can you tell me more who among the family do really encourage the pregnant women?**

R: Can be the mother or the husband.

**I: Can you tell me about any supplements normally given to women during pregnancy?**

R: Yes pregnancy women get the medicine for blood and their medicine for vitamin.

**I: Anything else?**

R: Yes the medicine to help grow the baby

**I: So where they get the medicine?**

R: The doctor write in their medical record and they will get it from the pharmacy and it’s free. They get free medicine.

**I: That great. Now can you tell me anything that prevents women from taking their supplements?**

R: Some say that they can’t swallow the medicine because it’s too big for their throat. Some say that they feel nausea or dizzy. They said that when the take these supplement, they keep vomiting or puke.

**I: Can you tell me if these pregnancy women drink alcohol, smoke, and using other drugs during pregnancy?**

R: Yes. Some women do.

**I: alright. Moving on… Can you now describe women’s diet during breastfeeding in this community? Like any breastfeeding mother.**

R: just any?

**I: Hmm yes**

R: Some breastfeeding mother don’t really care about the food they eat.

**I: some don’t care?**

R: hmm they eat whatever they have to eat like whatever food they see in front of them, they eat it. They don’t really think that it is not important for them to eat the food and it might affect the child but still they would eat it anyway.

**I: Now, do women usually change their diet during breastfeeding?**

R: Some women do and some don’t. Mothers who really care for the child eat good and healthy food. But mothers that don’t really care for the child can just eat whatever they want. They usually say “we can get whatever food we will get breast milk any” like they don’t really care.

**I: As a health worker, what are some of your biggest concern of the diets of pregnant and breastfeeding women in the community you work with and why?**

R: Health worker that work with pregnancy patient, they usually advice a mother to eat only nutritious foods.

**I: So you mentioned nutritious foods. Can you tell me more on what kind of nutritious food?**

R: local foods and foods from the store like fruits and vegetables

**I: Can you list what kind of vegetable that are really important for them to eat?**

R: can be orange, apple, water melon something like these.

**I: so can you tell me what kind of foods that breastfeeding women are encouraged not to eat and reasons why?**

R: salty food and junk food

**I: what kind of junk food?**

R: like chips or can be any kind of chips

**I: Now for the last section, we would like to learn about ways we can develop health programs in this community. Now could you explain where community members usually get trusted information about nutrition and health?**

R: Example for me… I usually get it from the doctors, some of the nurses. Youth to youth program, what is the name of the place that is partnership with youth to youth program?

**I: I do not really sure?**

R: yes. WUTMI program (Women United together Marshall Islands) or can be the KUMIT or (* team that prevent coalition here in the Marshall Islands*)

**I: So these places that you have mentioned, do they also umm…..**

R: Yes these places that I have mentioned usually delivered messages to peoples about how to live a healthy lifestyle what food they should eat and shouldn’t eat.

**I: As a health worker, what makes you trust where these sources come from?**

R: From my own opinion, I can see that these information are trusted especially when the talk it does really work when they arrange what food should be eaten and what shouldn’t be eat. They also delivered good information on what’s good for people’s health.

**I: Can tell me on where nutrition and health messages should be delivered so that community members would see/hear them most easily?**

R: The health centre

**I: The health centre. Anywhere else you can think of?**

R: like is said earlier, the prevention coalition team, and then youth to youth team. These are the teams that usually come to public and do their program about nutritious and health.

**I: Ok that is great. Now for the last question, could you describe what influence how people raise children in this community?**

R: the mother or the father, they should be good in influencing their children from the time the child was young until he/ old enough. They should influence them on what’s good and bad for their health.

**I: Great. You are doing well you answer my question perfectly.**

**I: Now can you tell me are there any specific advice or information related to parenting typically given to community members?**

R: hmm?

**I: Can you discuss any typical advice for parenting that should be given to community members?**

R: they usually give advice to community members like a mother on parenting or raising their children. Sometimes parents got mad with their children and can hit them in order to discipline them. Sometimes parents can’t control them and would hit them harder than they expect. Today when parents hit their children, that’s call an abusive so they can take the child away from parents who hit their child.

**I: So when it’s comes to advice to mothers that hit their child more than that, what can you say or what are some word of advice can you help that mother in order to stop her from keep hitting the child?**

R: we should tell them that they should balance the way they discipline them.

**I: so are there any information that pregnant or breastfeeding women typically ask for from health workers?**

R: mother that’s the first time for them to have child or mother that have no parents near them, what they would ask is things that they would need for the baby. So what they would need for the baby is the stuff for the baby or things that a baby would need.

**I: hmm like what?**

R: like the clothes for the baby, diapers something like that.

**I: so are there any way to communicate with caregivers about health?**

R: caregive?

**I: hm yes**

R: we should advice a caregiver and give information on how to raise a child. The caregiver should discipline the child in a best way. Foods that are giver to the child should be healthy and nutritious. They should be eat by the exact time of meal and bed.

**I: you are doing really well and give really answer. So is there anything else about the topics we talked about today that we missed** **or that you would like to tell us about? (Giggling)**

R: I think that’s fine.

**I: You’re good?**

R: yes

**I: okay we’re finish. Thank you so much for your generous time, we appreciate your help and we hope this research will help us improve the health of mothers and children in this community. Thank you so much.**
