# Supplementary material for: Conceptualizing multi-level determinants of infant and young child nutrition in the Republic of Marshall Islands–a socio-ecological perspective
Source: PLOS Glob Public Health. 2022 Dec 19;2(12):e0001343. doi: 10.1371/journal.pgph.0001343 (PMC10022247; doi:10.1371/journal.pgph.0001343)
Supplement: S1 Data — (ZIP) [file pgph.0001343.s001.zip › RMI Supp Data/Interviews data/I13U_IDI_MCG_Rita_Aug 14_BM_Cendanieledited.docx]

Interview Code: I13U

InterviewType and Interviewee:IDI_MCG

Interview Date: Aug14

Location: Rita

Interviewer: BM

Transcriber: Balton

**I: Do you agree to take part in this recorded discussion?**

R: Yes

**I: well, thank you for your wonderful time. Our talks and any information from you will help in our efforts to provide help infants, their mother and our communities.**

R: Hmm

**I: To start us off, could you say a bit about your family and household.**

R: good, about my family, we have two children. One is 6 years old and the younger child is four years old.

**I: Anyone else in the house?**

R: We live with my wife’s parents.

**I: You mentioned having two kids, how old were they again?**

R: one, the older is six years old. And the youngest one is four years old.

**I: How many boys and how many girls?**

R: They’re both girls.

**I: Oh okay, thanks. Can you say a bit about your community?**

R: Well, as for this community, it’s nice, it’s really great.

**I: you mentioned great, what is so great about this community?**

R: For once, because we just moved here from the outer islands, the people are very nice and they cooperate with each other very well. It’s

**I: What’s bad about this community?**

R: Well, the downside of this community it that there’s too many drunks just hanging around drinking and smoking, and they’re mostly girls.

**I: Thank you for your answering. Now we’re going to talk about health and illnesses within this family. Can you tell me what illnesses your children usually get?**

R: My kids usually get the flu and fever. Those the two most common ones they get. They’ve have not caught anything very serious yet.

**I: You said they had flu, do you know the cause?**

R: First, as you can see I am trying to think why. Because in the other islands they also get flu and fever but not as much. But here, maybe because there so many people in the neighborhood and everywhere, and if any one them get sick, they (his kids) also get it.

**I: From your understanding, what are the dangers of flu?**

R: Well, from my own understanding, if not treated and the flu get worst, it can get to a point where the doctors can’t do anything about it.

**I: Okay, now can you explain what to you do to help prevent from getting flu?**

R: Every time my kids’ gets sick I usually rush them to the hospital. Check them, especially giving medications.

**I: Now you mentioned about flu, no but fever, how did your child get the fever?**

R: Hmm… Usually when they play in the rain for too long, because they always insist on joining their friends playing in the rain. This makes them to have illness of fever.

**I: Perfect, thank you for your information. But can you tell, explain from your understanding the dangers of fever?**

R: Well, I think fever is not too dangerous because we have a hospital and as soon as the kids get sick we rush them there. Bring them so they can see the doctors.

**I: Great, And how do you prevent fever?**

R: Well, I always give them Tylenol because hospital always give the kids medicine and our room is full of them and we just give them medicine to help them and then take them to the hospital.

**I: Thank you for your answer. Your answers are very important, thank you. How can you tell you need to take your child to the doctor when they’re sick?**

R: Well, as a father, or parent of a child, when you see your child even a little bit sick you just want to take your child to the doctor.

**I: When they get sick, who do you take your child to first and why?**

R: Well the doctors. And if it’s night time, we always take the kids to the emergency. It just makes sense to take the kids to the doctors because we do not know as much as the doctor on how to treat illnesses.

**I: Do you use traditional medicine or healers when your child is sick?**

R: Yes, there have been times I have taken them to local healer when they have abdominal muscle strain or pains to be massaged.

**I: Great thank you, what illnesses you think your children will get from lack of nutritional food?**

R: I know for sure they will be malnourished and have problems functioning in places like schools and they will very sick and the type that resulted from malnutrition.

**I: And what kind of food would get your sick sick and why?**

R: For one, if we just give them junk food like chips and only food from just one of the food groups instead from each of the groups.

**I: Thank you for your answer. And what food would make your child healthy and why ?**

R: I believe they need to eat a lot of vegetables, lots of greens vegetables. Also stuffs like fruits and less of stuffs like rice. They may have rice but it must also include also vegetables.

**I: Because you referred to fruits and vegetables, why do you feed your kids those?**

R: Well, although I teach, I am also always learning and also teach my students the importance the food from each of the food groups like vegetables, like green beans and food like apples and oranges are types of food that will help children stay healthy.

**I: From your understanding, what health issues might result from food missing from diet?**

R: Well from my understanding, lack of food with vitamin C and other nutrients might result with poor eyesight and not see well in the dark and the likes…

**I: What do mean by “and the likes…”?**

R: Like illness that effect the body like the head bigger than the rest of the body and arms… Like those resulting from lack of vitamin C.

**I: Thank you for your answers. We appreciate your answers. We have been talking a lot about health. How would you describe a typical day for a healthy person from morning to evening.**

R: Say that again?

**I: What’s a typical day for a healthy person like from when he wakes to when he sleeps?**

R: A person wakes up, wash his face, brush his teeth, eat breakfast, do his work during the day but also make time to rest and eat his meals. It’s also important to have plenty time to sleep at night, like go to bed from 8- 10pm to have enough rest before the following day.

**I: Very useful information, thank you. What are the signs for a child?**

R: Sign? Could you ask the question again I didn’t really get it?

**I: What are the indicators of a healthy child less than two years of age?**

R: You can tell by their appearance or persona. Also by the body language, like whether they’re active or not. You can just look at them and notice it.

**I: Good, thank you. What indicates an healthy adult?**

R: Just from me, you see that they are active, they don’t just sit around and they exercise also and are always doing things. They aren’t lazy, they don’t just sit around but are always doing something and you see it. See that they are always active. You look at them and say to yourself, “that guy is healthy and active.”

**I: Thanks for the description. Now if you can tell what type of food you usually eat in your home? What type of vegetable or food you eat? Can you tell me how you get food for your home each day?**

R: Hmm… As I understand, or… How we get our food…Because we do not have anywhere to plant or have a small home garden where we could get food from, we typically buy from the stores, usually the Chinese stores.

**I: You have no garden?**

R: Uh hmm…

**I: Thanks. Are there any challenges in planting in your home?**

R: The first challenge is not enough space at my home to make a garden to grow food. And another one is compose and materials to start a garden is expensive. It’d be wonderful if the Hospital could help with this. If they could help provide what we need it’ll be awesome. The copra compose in Tobolar (Gov’t Copra facility) is expensive even if it’s not imported. Also the seeds for crops are also expensive.

**I: Thank you for your answer. Please tell me how hard or easy is it for there to be food throughout the year.**

R: Say that again?

**I: How hard or easy is it for there to be food throughout the year.**

R: To me, I don’t think it’s that hard, like with us here, we all help bring food and always help where help is needed. By us working and helping each other, its easy to provide food.

**I: During a year, what food are scarce and what makes it so?**

R: Maybe more so in the other islands than here, when local food starts to get scarce and when crops run out and no recent field-trips (Gov’t boats rounds to atolls with supplies and people) for supplies, like rice and flour, those are the times I’ve seen it happen.

**I: Where food supplies run low, what to you do to feed your family?**

R: As you know, it’s our lifestyle to go to the ocean to fish if food get low. Our home in the outer islands, we have our own taro patch that we get taro from when food get low and we just bring them and boil or whatever.

**I: Thank you. Now for our next question, do you raise any animal?**

R: Yes, I only just started raising some chickens. I have about twenty to thirty chickens and two pigs, a female and a male. I am taking care of them so they can multiply. We only just recently decided to raise animals.

**I: Okay. Could you tell me what made you decide to raise animal?**

R: Well, first off, it’s because I most usually buy pigs from other people for my kid’s kemems (birthday party). We lived in Aur (an island in Aur Atoll) but bought pigs from Tobal (another island in Aur atoll). And that’s what made us decide to get young pigs to raise.

**I: Good. Those were good information. Now tell me the difficulties of raising animals.**

R: One of the difficulties is maybe because heat from the sun is getting worst nowdays, it causes fewer coconuts to feed on so we feed them banana leave and other types of leaves to feed on instead of coconuts which are better and can help them bigger faster.

**I: Good. Thank you for he information. Now can you explain challenge to putting animals in pens?**

R: One challenge in putting animals in pens is they grow sickly or not like if they were to roams around to forage for food. And now with laws on putting animals in pens and not let them roam free, sometimes no matter how you take care of them, some still die out. Maybe its because they need to also be free to forage also to feed themselves.

**I: Thanks for the information. But what do you usually do with the animal feces?**

R: Well, I don’t do anything with them. I just throw them away.

**I: Okay good. There are times when we wish to eat something but we couldn’t. What do your family wish to eat but can’t not because there’s none of it but because they’re rare or very few of…?**

R: Well, from being raised and living in the other islands for years, this makes us start to wish we could eat food from the centers (Majuro and Kwajalein), usually because we get tired of local food.

**I: And what prevents you from eating these?**

R: First off, because we’re in the other islands, sometimes when the ships come in after like 4 months, it makes us wish we had food from the center.

**I: Thank you for your information. For the last question regarding food, who in this family decides the food for this family?**

R: Well now while we are living with my in-laws, it’s them that decide what we should bring to eat. In the outer islands, where we’re independent, it me who decides.

**I: Who in this family decides on the food to eat?**

R: I, alone, decide what we eat since I am the head of household. But I always ask them what they wanted me to bring.

**I: And who decides what the kids eat?**

R: Their mother. Their mother usually decides what I feed them.

**I: Now we’re going to talk about water and hygiene. Okay?**

R: Okay

**I: How do you find and store water?**

R: In the outer islands, we have water tanks, the kinds that are given to people in the outer islands. And we also have wells. These are what we used most.

**I: And where do you usually get water for dinking, cooking, washing and bathing?**

R: We usually get our drinking water from the water tank, as for baths and other stuff like watering the pigs and other animals, we use the well.

**I: And what are the challenges of getting water?**

R: When it doesn’t rain for a long time, like for six to seven months, them the water tanks empties. That’s one of the challenges.

**I: And what are the challenges to storing water?**

R: One of the challenges is when the tanks that were given break causing it to leak. When that happens, more water leaks out than get stored. This is one of the challenges. And it will take time, or until next summer to get a replacement.

**I: How do your family clean their drinking water?**

R: I don’t think I know. We’ve never treated our drinking water, we don’t know how to treat our water. We don’t even know if the water in the tank in the outer islands is clean.

**I: Thank you for the information. Now let’s discuss washing hands. Can you describe how your family wash their hands throughout the day?**

R: We usually wash our hands in the dish washing basin that has detergent already inside. The mother usually takes the kids there to wash their hands before they eat. That’s our common practice.

**I: And how do the kids wash their hands throughout the day?**

R: When after the play and they come to eat we ell them to go wash their hands.

**I: How do the kids under two years old wash their hands?**

R: They don’t usually do it themselves, their mother takes them to wash their hands.

**I: When is soap usually being used?**

R: When do they wash their hands?..... After they play, after they touch dirty things, they wash their hands with soap but especially before they eat.

**I: What is your opinion about washing hands with water and washing with soap and water?**

R: I think washing hands with just water doesn’t clear all the germs from your hands but using soap clear maybe not all but enough not to get you sick.

**I: What prevents from using soap when washing your hands during the day?**

R: Maybe, from my understanding if there no money to buy soap causes people to just go and use just water when you run out of soap.

**I: Thank you. I like how you have been answering my questions. Now, can you tell me what type of toilet you have in your home?**

R: Restroom? Like bathroom? There’s a restroom over there for the family, it has a flushing toilet and bathing area. It’s what we use.

**I: Can you explain why use this one and not the other kinds?**

R: I don’t know. The elders made that decision, it was already made when we came.

**I: Thank you. Okay, it is heard that in some communities’ people defecate on the beaches, can you explain why this practice is happening and how frequent?**

R: Honestly there are lots that do this especially in the outer islands. You would see people go into bushes or you you see them run to the beaches. Maybe its because they don’t have a bathroom.

**I: Can you explain why it’s happening in some place and not in other places?**

R: From my understanding, because in some places they have restrooms because, I don’t know, the government help with funding for restrooms… and some places they don’t get funding and they can’t afford since they’re copra (coconut byproduct) workers, outer islands people are usually copra workers. They can’t afford bathrooms and materials to make one.

**I: What do think are the challenges to getting a restroom or toilet?**

R: Again?

**I: What do think are the challenges to getting a restroom or toilet?**

R: I think the only challenge is… because it’s expensive, buying the toilet seat, hooking up all the plumbing and water, those are the obstacles from my understanding.

**I: Now can you describe how you get rid of the kid’s feces?**

R: When the kids were younger they used diapers and maybe the mom takes them to the dump or throw them away somewhere… I don’t know.

**I: Where do your kids usually play?**

R: They usually play here, close to the house. They are usually around, close-by, near the house.

**I: Can you describe a good playing area for the kids and why?**

R: Umm… I think because space is limited, it’s hard to get a good play area, a nice playground, only because of limited spaces. The houses are tightly packed.

**I: You can go check what your wife needs you for, we’ll just let this run while I wait… Now let’s continue. Do the kids play where there are animals?**

R: No, no. There’s no dogs or anything except the other houses that has dogs but they’re far.

**I: Okay, from your understanding, what prevents playground from staying clean?**

R: What keeps them from being clean? From my understanding, it’s people in the community not getting involve in cleaning the place the kids play at.

**I: Now for the last question regarding hygiene, how do you prevent the spread of diseases?**

R: Say that again?

**I: How do you prevent the spread of diseases?**

R: Maybe by washing our hands, cleaning our waters, our sleeping areas, and also our surroundings. Around our houses should also be clean.

**I: What are your thoughts on feces laying about and the diseases that are spreading around.**

R: Its seems…. I don’t understand it.

**I: If I could rephrase it, do feces cause diseases?**

R: Oh yes, yes!!! That’s the number reason for diseases to spread, when the animals take the germs from the feces and spread them when they land and place the germs in the food we eat, making us sick. So we have to be careful of this.

**I: Now I would like to know the roles of everyone in the family in taking care of the child. How do the community help the children each day?**

R: Hmm… Like… The community?

**I: Yeah, the community, like your neighbors. How are they in taking care of your kids?**

R: Oh, very good. They treat them very well.

**I: And who has most responsibility in taking care of the kids?**

R: Foremost is the mom, and then me. The two of us are most responsible.

**I: What do you think are the responsibilities of the mothers in taking care of the kids?**

R: They are responsible to make sure they are safe, make sure they are fed and have water to drink. Think these are their responsibilities, also clean their things and sleeping area.

**I: Do they have any other responsibilities?**

R: Breastfeed and keeping them warm.

**I: And what are the responsibilities of the fathers in taking care of the child?**

R: They help the mothers take care of the kids when the mothers get tire. They help the mother.

**I: Do they have any other responsibilities?**

R: Yes, fish. They also provide food. They also have to go fish to bring home food.

**I: Thank you for your input. How does the caregiver play with the child? How do you or the person with the child play with the child?**

R: They play with them, or just talk with them so they can learn to understand.

**I: And how do you play and talk with them? What kind of game do you play?**

R: Hmmm… like I don’t know, like give them a ball and play with them…

**I: Okay, good. Can you tell me what happens when grandparents help take care of the children in the community?**

R: They teach the children about our culture and help teach them things, and because they are the future, they also need to learn from the grandparents also.

**I: Okay, thank you. Now can you tell me how the grandparents help the kids, the mothers, and the family?**

R: They also help take care of the kids. If the parents are busy, they are also help watch over them, take care of them, and help keep them safe.

**I: Thank you. Now what identifies a good grandparent?**

R: Huh?

**I: From your understanding, what makes a good grandparent?**

R: I don’t really understand…

**I: If you don’t understand, let me rephrase it, how can you tell the grandparents are good at taking care of the children?**

R: Oh, you know from how they are eager to be with the kids, to play with them, and to take part in their lives.

**I: Anything else?**

R: Just this in my opinion.

**I: Can you tell me the responsibilities of other members of the family with taking care of the kids?**

R: Hmmm…. Responsibilities of others? They have to care for the kids, know not to talk to the kids a way that would hurt them, teach them respect by also showing respect.

**I: How do the older kids take care of the child?**

R: They take care of the them, help care them also. They take them playing and also watch over them when they play by themselves.

**I: Good response, we’re almost done. Now we’re finish asking question regarding family and now going ask about the community. Can you tell me where you take trusted information about food nutrition?**

R: At the hospital. The hospital tells us what food are healthy and nutritious. And what food to avoid.

**I: Why do you trust the information from this place?**

R: Because they’re the experts who understand people and what they should eat to be healthy. These are the people that are experts in the human body. That’s why I trust the hospital.

**I: Because the hospital, are there other place you can go to find or hear information about health?**

R: Oh, in the schools, the churches, youth-to-youth programs, and other places that people usually gather to help teach this type of information.

**I: Thank you for sharing your information. Now how do you usually get these information?**

R: Usually from the radio. This radio here is usually on and get information from it.

**I: When do you usually hear this on the radio?**

R: During the announcements and…. The advertisements that comes on the radio.

**I: Now for the last question, from your experience, what motivated you to take care of your kids?**

R: Repeat that again?

**I: What motivates you to take care of your kids?**

R: Because they’re my kids, and I helped bring them to this world, this motivates me to do everything I can for them. This also include also me and my wife advising each other on how best to take care of the children.

**I: Thank you. In your own opinion, that is the community’s opinion on raising children?**

R: I see some people leave their kids to go hang out and drink. I think they need to change and take more time with the kids and also respect their kids.

**I: Did you get any advice or word of wisdoms on raising kids?**

R: Hmm… No.

**I: You never got any advice from anyone on raising kids?**

R: Oh, I learned from the older folks, they advise me to take care of the kids.

**I: Who helped advise you on taking care of your children?**

R: Family Planning

**I: Okay. Were there any questions you wanted to know regarding nutrition and child caring?**

R: No

**I: Anything you wanted to know in parenting?**

R: Yeah, I got them from pamphlets that are handed out at Family Planning.

**I: Okay, we are done. Is there anything would like to know but we never covered?**

R: No, everything was taken cared of in the questions.

**I: well we are done and thank you once again for your time and information, your identity will remain confidential. Thank you.**
