# Supplementary material for: Conceptualizing multi-level determinants of infant and young child nutrition in the Republic of Marshall Islands–a socio-ecological perspective
Source: PLOS Glob Public Health. 2022 Dec 19;2(12):e0001343. doi: 10.1371/journal.pgph.0001343 (PMC10022247; doi:10.1371/journal.pgph.0001343)
Supplement: S1 Data — (ZIP) [file pgph.0001343.s001.zip › RMI Supp Data/Interviews data/I14U_IDI_MCG_Rita_Aug 14_Balton_Fela edited.docx]

- Interview Code: I14U
- Interview Type and Interviewee: IDI_ Male Care Giver
- Interview Date: Aug 14, 2018
- Location: Rita
- Interviewer: Balton
- Transcriber: Cendaniel

**I: well, first, I would like to thank you for your time. Helping out the Ministry of Health, by participating on how children are taken care of. Is it okay for us to proceed on with our survey?**

R: okay.

**I: okay, to begin with, can you tell me a little about your family? Give me a little bit information.**

R: this family?

I: yes like who live in this house, please tell me a little about this family

R: let’s say, me and my wife, our daughter and her infant son. And a girl, my wife’s cousin, and our grandson.

**I: ok. That’s great thank you. How many children live in this house?**

R: two, two little boys. One year old and a two year old.

**I: great.**

**I: can you describe about your community.**

**I: in your town that you live in.**

**I: how’s it, what are the advances, what are the challenges? It cannot be your house, but it can be the neighborhood that you live in.**

R: it’s like, it’s near the water. We live near the ocean side. We don’t really have stores in this community.

**I: is there, okay proceed on.**

R: In this town, we only have one store or the person that doing that business is a Chinese man.

**I: are there any negative things you see needs to be better?**

R: our sea wall is wreck but, has not been fix to this hour. And one of the things that are affecting, when is high tide. Flood comes inside the houses, it’s also affect the environment and all that. The flood also cause disease and affect people’s house and their stuff.

**I: great.**

R: the thing that is affecting this neighborhood drinking Alcohol is huge. Drunken people disturbing the peace, children often fright everyday of hearing shouting and that is also one disturb the peace of other people. We don’t sleep peacefully sometimes. Let’s say in other word law enforcement is weak by these doings.

**I: yes, great.**

**I: now we’ll talk about health and Illness.**

**I: your answers are great, thank you for answering. With your informative comments, they will help this survey. And thank you because it will help us with this survey.**

**I: now can you tell me about some of the illness the children in your community suffer from?**

R: I always see children with malnutrition, some has huge belly, is it hunger or what? Others suffer from fever. Maybe it’s the affection from climate change, but there are many children I’ve seen having fever. Running nose, pink eye, and coughing. All because of what is effecting them but it is really from air pollution and changing of climate change.

R: Maybe because some people don’t want to…. there are others with malnourish, not enough money inside their father’s pocket to provide for them. For medical care like these things.

**I: good, what do you know of, like if it’s diarrhea. These illness you mentioned, can you tell me what causes each illness?**

R: what causes what?

**I: like, when it’s diarrhea, pink eye, fever, every illness that you showed. Can you describe what cause them?**

R: number one, their foods, their water doesn’t boil. And the air, the air can cause illness. When flood rise from the ocean side, goes inside their houses and damage people’s stuff, lack of sanitation, throwing trashes near their houses. Making insects, flies and mosquitoes flying. These are the things I know of.

**I: and, your grandchild. What are the illnesses the he frequently suffer from?**

R: he usually had fever, where he must not use fan for cooling. Having internal injury from playing and fall from upper things like the tables or stairs caused him to have fever comes from stomach pump.

**I: regarding the illnesses that you mentioned. Children in your community including your grandson, are there any ways to you think that might prevent them from having these illnesses?**

R: yes, each household needs to clean, boil water for their children to drink this prevents them from having diarrhea. Bring the ill children to the doctors and it would be very helpful if people clean their environments so that there wouldn’t be any sickness happening around town.

**I: great, Thank you.**

**I: can you describe how you know when your grandchild needs treatment for his illness. Or ways that he needs to see a doctor.**

R: when I see that he’s having a fever, high burning fever, coughing, and when he has diarrhea. That’s where I bring him to the doctor.

**I: when the child is sick, who do you think he goes first for healthcare and reason why**

R: they usually can be brought to their grandparents, if they can’t be healed. They can be brought to church pastors for a healing prayer. Frequently, they bring them to hospital to the doctors

**I: are there any times where you use traditional healers, or traditional medicine to heal the child**

R: I brought my grandson to a lady for traditional healing because he has stomach pump and I had to bring him for his grandmother so that she can massaged his stomach, he’s been having fever, but when he was treated by the lady he recovered. He’s been taking medications but, nothing change.

R: has he was been treated by traditional medicine, because of his internal injury, after the treatment, he recovered and healthy. I think this is the best way we can do to them when they have stomach pump, is doing stomach massage.

**I: can you describe any illnesses that are affecting your child or grandchild that are associated with innutrition and foods that are given?**

R: diarrhea, weakening, weakening of eye sight, and lazy.

**I: what types of foods that make your child’s body unhealthy?**

R: like feeding them fat foods, junk foods, having them to drink soda, foods that have fats in them. Junk foods and those.

**I: can you explain what kind of junk foods?**

R: like, gums, lollipops, sodas, and ice-creams. Things that have sugar in them.

**I: can you explain what kind of foods that have fats in them?**

R: about foods that have fats in them, chips. Chips have fat in them, chips. Breads, fats usually add into breads. Pigs, there’s fats in pigs, that’s it. I guess these are the only things I can think of.

**I: what types of foods that make your child’s body healthy?**

R: regarding healthy foods, such as apples, grapes, papayas, and breadfruits. Local foods, oranges and apples.

**I: great thank you.**

**I: are there any illnesses caused by foods missing from diet? What kind of illnesses that might expose?**

R: foods missing from diet, we know there are young children with unhealthy body growth. Poor eye sight, tooth ache, problem with hearing. I think these are the things I know of.

**I: great, thank you. We talked a lot about being unhealthy. Could you now describe for me a typical day of someone living a healthy lifestyle, from the time they wake up in the morning until when they go to bed?**

R: the person is not lazy, works daily, looks healthy, doesn’t sit but do walk around. Always playing, just like these.

**I: what are the appearance/signs of a healthy child under 2 years?**

R: always playing, is not lazy, is not weak, just like these. Always get into moving, doesn’t cry a lot.

**I: but, what are the appearance/signs of an adult?**

R: always working, always picking up leafs, always working and moving, and doesn’t sit around.

**I: good, thank you. Now we’ll proceed on with the questionnaires about Food. And how we eat our foods, can you explain how your household gets food to eat on a daily basis.**

R: their parents provide, they need…to work to buy for their children. Cook their food, I think that’s it. Others bought food from the stores, but others grow foods to feed their families.

**I: in your community, how to you put food on the table every day? What are…how do you find food for the house?**

R: we go fishing, we go to work to buy food for the house. There are others who grow food, things that we know of.

**I: what kind of foods are grown at your home?**

R: man, there’s banana, lime, Pandanus, papaya, and there’s coconut around the house. I think that’s about them.

**I: among from these foods grown at your home, are there any among them you take and sell?**

R: yes, bananas and sometimes I put the limes inside a plastic bag and sell them to the stores.

**I: great, Thank you. Now, as you were selling these foods. How do you commonly use the profits?**

R: for buying foods, taxi fare, buying the children’s needs, diapers and foods.

**I: Can you tell me about any difficulties to growing food at home?**

R: there are places that are having difficulties, but here at this house is not, it’s easier.

**I: what do you think about what prevents someone from growing food at his home?**

R: like what?

**I: like, if someone is to make a farm at his home, is it easy to grow, what are prevents it?**

R: there are some places is difficult, land owners doesn’t like moving dirt on their lands. The Alaps (ranked below the chiefs), but there are some place it is easier for them.

**I: Food availability varies across the year in some places. Could you explain how easy or difficult it is to get those foods on the table?**

R: like I said, we go fishing, work to provide for food, and fishing is easier to look for food.

**I: Great, thank you... now, am asking about animals in your community. Could you please tell me about animals that you raise?**

R: like what?

I**: any kinds of animals for food**

R: the place that I am in, doesn’t have any animals.

**I: raising?**

R: raising. None

**I: thank you… as you can know, can you give me some difficulties of someone raising animals?**

R: well yes, EPA (Environment Protection Authority) and the land owners prevent them. Because of complications of polluting the air.

**I: can elaborate more on these things, these difficulties?**

R: for pigs, they cannot raise pigs because they’re dirty. Causing bad smells. Dogs, dogs carry diapers and bites and this is why many Alaps don’t like raising these because they think it affect people’s health lives.

**I: as you know based on your understanding, for instance, pigs are put inside fences. Do you know, for those that raise pigs, what do they do with the animal feces?**

R: there are some who drained them out into the ocean side, inside the safety holes, and there are others who do them for their farms.

**I: There are sometimes foods that we wish we could eat, but for some reason we cannot do so. Could you tell me about any foods you wish to eat (or eat more of) but cannot?**

R: yes there is, turtle but it cannot be bought at this moment.

**I: what makes it difficult for you to eat turtle at this moment?**

R: we need dive for it, but since turtle is one of the extinct species that are not to be killed. As coast guard mentioned it.

**I: great, thank you. For the last question on food, can you explain who decides what food to get for your family?**

R: at my house, each and every one decide their own food. If I want to eat something I want, there’s no one to object to it.

**I: who cooks the food?**

R: the woman. During the times she’s at work. And mostly, she does the cooking.

**I: who decides which foods young children should eat?**

R: well, the mother is the one who decides the food for the child, she knows what food is good for the children.

**I: Thank you, now we’ll proceed on to water and hygiene. When it’s about water, we’re talking about waters that are for drinking, bathing, and for foods. Hygiene, is about sanitation of our environment. And first question about, water and hygiene is Can you please describe a typical day getting and storing water in your community?**

R: getting and storing water

**I: how do you store water? Where do you store water?**

R: inside the water catchments, water containers. It is also defined as (bantoon) water catchments. Storing rained water, there’s no well to store water from it. It is common to get and store water from the rain. We fill and store our water in containers or in our water catchment the bantoon.

**I: and the water inside the water catchments are used for what?**

R: there are two water catchments at the house, one is used for cooking and those things. The other one is used for drinking, people in this house use faucet for bathing straight from the government’s water supply MWSC (Majuro Water Sewer Company).

**I: how do the family clean their water for drinking and cooking?**

R: there are others who would boil and chlorination of water. There was a survey done long ago, conducting on how to treat your water with correct measurements of chlorination. But, many people do use boiling water.

**I: are there any difficulties of storing water?**

R: the difficulty is when there is a drought, that’s the difficulty.

**I: during the time there is a drought, what do you do to get water?**

R: we use the government water for cooking and bathing, but for drinking water. We usually get water from the stores such as EZ Price, Cost Price, where you fill up your water machines with 5 gallons by entering your money.

**I: are there any difficulties for someone to have water catchment?**

R: yes, it is. Price of water catchment now a days is huge. And there are others don’t have enough water containers. Today stuffs are getting expensive like bantoon it is not a joke to just get a bantoon right away because bantoon is really expensive nowadays. And there are few people that don’t have enough water catchment to store their water.

**I: great, now let’s discuss hand washing. Could you describe in detail your family’s hand washing throughout the day.**

R: we usually fill up a bucket and use soap, and there are others who mix the cool water and the hot water together and wash their hands with soap.

**I: how do children’s hand washing throughout the day?**

R: …. Their mother usually does the hand washing for them, since they’re still young to do it.

**I: how hand washing throughout the day for children under 2?**

R: Like I said, their mother is the one who wash their hands for them, they’re still young to wash their hands.

**I: …. what time do the children wash their hands?**

R: when done eating, or when they are done using the bathroom.

**I: from your own understanding, what you think is the difference between using water only or water and soap to wash hands?**

R: washing hands with water does not clean and the smell of the food you ate, but when washing hands with water and soap, it cleans and the smell of the food you ate is gone. That’s the difference.

**I: as you know, what prevents someone from hand washing with soap throughout the day?**

R: can it be what?

**I: by the time … what makes someone not to use soap when hand washing?**

R: wash…

**I: they don’t use soap.**

R: they forgot. But, there are others that don’t have any soap at their place. They don’t have any money to buy soap.

**I: thank you, you’re doing a great job answering these questions. Could you describe the type of toilet that you have at your home?**

R: our toilet is inside the house, is not at outside. The place that were living in is that, the toilet is inside the house.

**I: can you explain what type, the ones you use flush or water?**

R: Flush, the ones using toilet paper, are the ones that are using flush. (31:00)

**I: there different kinds of toilets that, we see. There are houses that have toilet holes, and those that use water.**

R: yes, but, there are other people that don’t have bathroom. They use the ocean side.

**I: yes, why, why do you use toilets that use flushing instead of these others?**

R: because it is safe, it is environmentally safe, and there’s no difficulty for people to use. It is more safety to use our own restroom than the ocean side because it is for public people and it is not a place to defecate.

**I: as we mention a little bit about. There are some communities where people use ocean and lagoon side.**

R: yes because there are people who don’t have places to stay or defecate there are those who doesn’t have bathrooms.

**I: can you explain how come this happens?**

R: there are those who doesn’t have much to build their own toilet room, there are those who don’t have jobs, and there are those who happened to be lazy, making the ocean side look bad.

**I: can you explain reasons why this practice exists in some places but not others.**

R: because the Island is thin, there are some places where is prohibiting and there some places that are not. There needs to be laws for these kinds of things.

**I: what difficulties for a family to build its own toilet place?**

R: number one, budget, number two, land owners if the land owners won’t allow them to build their own, that’s another difficulties like the Alaps. These are the things I know of.

**I: there are just some places that the children can play and do defecate at … as you see, how would a person dispose of children’s stools? What do they do about that?**

R: they always use shovel, coconut shells, and cans.

**I: and what do they do to them usually?**

R: they throw them out to the ocean side, to the trash, trash can, and others do throw them out to the lagoon waters.

**I: Could you explain where your young children usually play each day. Where do the child play?**

R: the child always play around and near the house, and inside the house, the child doesn’t play far.

**I: is there an animal where the child plays at?**

R: well, yes of course. Dog, there’s dog at the child play area. The dogs of this house, dogs that are from the other houses, animals that usually comes near the house. Cats, there are times there are cats. Chickens, chicks and those. He does always play inside this house.

**I: are there any places where children can go and play?**

R: there are, the parks, school play grounds that’s where they play at. But they always play on grass fields. Basketball courts, they also usually go and play there.

**I: what are the challenges of keeping a child’s play area clean? What makes them not to be clean every day?**

R: people do litters, and animals. They always move around the litter them. Trashes and diapers where they bring them to the children’s playgrounds. People too. They come and throw trashes instead putting them into the trash areas.

**I: Great, Thank you. To wrap up our questions on hygiene, could you explain ways to prevent the spread of disease?**

R: if the community cleans their environment, their places. Cleaning, is most important. And lawn mowing, like these things. Boil their waters. And … how do we say this one? Make a group where they can clean from one place to another to clean the areas and government involvement with cleaning supplies, helps clean the environment. The government is willing to help with cleaning supplies, that’s all. Not just theirs but also the people, especially the council men in a community. Bringing them trashcans and trash bags. Cleaning their surroundings where people can come out and help cleaning. That’s about these.

**I: good, thank you. What you think of the connection between exposure to feces and illness?**

R: well yes there is, how can there be no connection. They spread disease, the flies come and the animals, where you’re not happy about it.

**I: Thank you, your information are helpful. The question that we are going to discuss, gender or it’s about families in the community. Could you describe the care of children throughout the day in your community?**

R: like about what?

**I: like, who takes care your grandson in every day? On what time who takes care of him?**

R: Usually just me, and my nieces at the next house. When his grandmother comes home from work, she’s the one who takes care of him in the evening. We help each other take care of him every hours, the people in this house, father, grandmother, and my brothers.

**I: who is mainly responsible for child care?**

R: his grandmother, were the ones taking care of him. His parents are in the states.

**I: what are the responsibilities of mothers or the grandmother in child?**

R: things that are, taking care of food. His needs, water, taking care of medical care.

**I: what are, okay...? Proceed on.**

R: these are the things I know of.

**I: and what are the responsibilities of fathers in child care?**

R: keep the child from harm, help out with the child’s needs. Buy foods, same as the responsibility of his mother. Except when not busy. Things that he can do.

**I: how caregivers play with children under 2?**

R: there those who would play with them at picnic places, there those who would play with them at the playing grounds, there are those who would play with them outside and inside their houses. Trying to make them not to be boring. Let’s say, lazy. Doesn’t want to do anything. If they have time, they take them and play with them. They usually take them to picnic areas, go enjoying the waters with them

**I: about your grandson, where do you usually play with him?**

R: we usually play fun with him, we bought toys for him to play, a television for him to watch kid’s songs and these things.

**I: from both you two, as grandparents. Are there any difference when taking care of your grandson?**

R: there’s things he learns, as his parents are away from him. He knows how to play all by himself, like he knows how to play with himself, and work with himself.

**I: is there difference when the child is with the parents and the grandparents are in the community too? What you think is there**

R: yes there’s a big difference. From the child that the grandparents and parents are far away from the child.

**I: … what are the grandparents support in raising children, support mothers and families?**

R: like what, advisements? ... Giving advisements about how to take care

**I: what makes good grandparents?**

R: they are really good in taking care and raising the child. They always take care of them.

**I: great thank you. Could you talk about the role that other family members have in raising children in this community? (46:05)**

**(Interruption cause 46:10 – 47:09)**

**I: pardon me, I’ll say this again. . Could you talk about the role that other family members have in raising children in this community? Apart from you two, who are those and what do they do to take care of the child?**

R: other family members?

**I: yes, other ones.**

R: how can we know, because we don’t be at their houses?

**I: inside your home. Others apart from you two**

R: well.

**I: others that who took care of your grandson.**

R: like?

**I: like our aunty, those or your other daughters**

R: taken care of while we are not here right?

**I: yes do they, help out?**

R: yes they do, sometimes they help out with Jenjen’s(name of the child) needs. And there are times where they check if were gone, they come and take care of him. There are times they help out,but they do help out.

**I: okay, these answers are great. And were almost done. And for this last question, we would like to cover about how we can develop health programs in this community. Like, Could you explain where you usually get trusted information about nutrition and health? Where you usually get,**

R: we usually get from radios, and newspaper. Well we’ve just common hear from these things announcing on the programs that usually comes from radios. I thought Nitijela (Parliamentary government hearing) and newspapers. And when walking from one place to another, I usually get information from friends, like there selling food at this place, like that.

**I: and where nutrition and health messages should be delivered so that he would see/hear them most easily?**

R: I usually hear from hospital’s radio, program that is legal services, not legal services but those who are responsible delivering health. Health service and them.

**I: When you think about your own parenting behaviors, can you explain what influences how you raise your children?**

R: I don’t really have an information regarding that kind of question.

**I: what are the opinions of the community influence of raising children (e.g. leaders, neighbors, church leaders, health workers?)**

R: where?

**I: let’s say, the influence of raising a child.**

R: well, there some families do, and others don’t. Some do care for children and parents don’t.

**I: are there any advice or information from your parents, grandparents related to parenting you received?**

R: well, yes they used say take good care of the child, don’t harm the child, but the very important thing is help them finish their education. If you don’t finish your education that’s a problem in the future for your family. Just like now a days, our children didn’t finish their education that’s why they’re having complications. If there is no money for diapers, they come asking “baba”, if they don’t have any money for food they come asking, “Baba”, when they don’t have money to buy foods for the child they asking “mama” now they’re in suffer because they never listened to fathers talking to them.

**I: great thank you, Is there anything else about the topics we talked about today that we missed or that you would like to tell us about?**

R: what I see, everything is perfect. Those questions were great. As I can see everything that we covered were really helpful and I think there’s nothing from me, thank you.

**I: well, I would like to thank you for your generous time. By helping the ministry of health, especially UNICEF, thank you. Thank you to the member of the family. Thank you and May God bless us, that’s all.**
