# Supplementary material for: Conceptualizing multi-level determinants of infant and young child nutrition in the Republic of Marshall Islands–a socio-ecological perspective
Source: PLOS Glob Public Health. 2022 Dec 19;2(12):e0001343. doi: 10.1371/journal.pgph.0001343 (PMC10022247; doi:10.1371/journal.pgph.0001343)
Supplement: S1 Data — (ZIP) [file pgph.0001343.s001.zip › RMI Supp Data/Interviews data/I15U_IDI_FCG_Rita_Aug 14_Meia.docx]

Interview Code: I15U

Interview type: In-depth interview

Interview Date: August 14 2018

Location: Rita

Interviewer: Meia

Transcriber: Fela

**I: Thank you for giving me your time to speak with you today. So if that is fine with you can you please give me your answer?**

R: Yes.

**I: Thank you for offering your time. The information we learn here will help us find ways to improve maternal and child health for both mothers and the children and sanitation in this community. To begin with, can you please tell me a little about your family/ household?**

R: About what?

**I: Like who lives in the household?**

R: Tell you the names or what?

**I: No you can just tell me who lives in this house.**

R: OKAAAAY! My mother, and also my father, my brother’s son, my son, my sister and our grandmother.

**I: Now how many children in this house?**

R: Two children, one with one years old and the other one is one year old.

**I: Great, and thank you.**

**I: Now can you please describe a little bit about this community? Or let’s say, this town.**

R: Um like what?

**I: Like what are the positive things about this community or what are the negative things about this community?**

R: Ummmm I don’t know.

**I: As of good and bad things, what are the good and bad things about his community? And what you know about that are not good in this community.**

R: You mean in this town or?

**I: Or can be this community.**

R: You mean sickness?

**I: No the bad and good things about this community.**

R: The good thing about this town is there is a new church that has been build.

R: The bad thing about this town is there are lot of drunk people. People drink from Monday to Monday even though there is a new church building there. Both boys and girls drink alcohol every day and we face trouble every day because people got drunk all the time.

**I: What else**.

R: I think these are the only good thing and bad thing I can mentioned about this town.

**I: Alright alright. Now can you tell on what kind of sickness that often happened to people in this family? Can you tell me what kind of sickness that often happen to your child?**

R: daheia and fever.

**I: So can you tell me what the causes of daiherrea are?**

R: Maybe it’s the water catchment or sometimes he used to get water from our neighbour’s water catchment but I don’t know because when he is here in this house, we buy him water from the stores. But I truly believe that people who come and take him with them, they give him water from anywhere or water from water catchment that are not clean enough.

**I: What cause the fever sickness?**

R: The cause of fever is the wind from the fan.

**I: Now do you think diarrhea sickness is a seriousness sickness?**

R: Yes because it can make his body dehydrated and what else can I say about it…..pooping without stopping. Sometimes he is too weak and sometimes he can’t eat.

**I: What about fever sickness what are the seriousness about it?**

R: Well as of every child, when the fever is too high for them, the most seriousness about it is the child can be stuck or when it get higher than that, then we might lost the child.

**I: Now can you tell me how do prevent diarrhea sickness? Can you explain how you can prevent diarrhea sickness?**

R: So that it will never happen again?

**I: For example, when I used to get diarrhea, I always drink soda the coke or I get medicine from the hospital and as of my son, when he get diarrhea, I would buy him Gatorade and also medicine from the hospital or what we called (IV) or the watery thing inside the bag that given from the nurses to heal diarrhea sickness.**

**I: And what about fever? How would you prevent fever?**

R: Well what I would do is put water in a cloth and rinse it with water so that it would help suck out the fever inside the body. Or I would also put oil in his body to help calm the fever down.

**I: Can you describe how you know when your child needs treatment for their illness?**

R: When he is really sick and getting ever worst.

**I: How do you know when he is really sick can you really explain on that?**

R: I can say that when I try to rinse the piece of cloth on his body but the fever is not getting lower than before or it’s getting worse. He doesn’t want to eat.

**I: Now can you explain who would be the first one you bring the child for healthcare and why?**

R: Where?

**I: When he is sick. Do you bring him to anybody when he is sick or? Is there anyone you bring your child to when he is sick?**

R: The doctors.

**I: The doctor? Ok**

**I: Now can you explain why you bring your child to when he is sick?**

R: So.. who do you think I would bring him to except the doctor?

**I: Yes yes that right.**

**I: So do you use any traditional medicine?**

R: For my child?

**I: hmm**

R; Sometimes when.. yes when I know there was something wrong with him like they say that it’s the demon or ghost I don’t know I never bring him to the doctors so I brought him to the traditional healer and she just put some traditional medicine and the sickness was all gone. Tylenol or medicine from the hospital never worked for him. The only thing that heal him was the traditional perfume given to him. The father of the son ( my husband) work as an observer on the chips and I think he bring all these evil sickness that can’t be heal by the doctor from where ever ocean he goes and I think that happened last few months.

**I: Last few months? Mm I feel sorry for him.**

**I: Now can you describe any illness affecting your children that are associated with nutrition?**

R: Uh what?

**I: Illness that affect your child that associated from food contain with nutrition.**

R: What could it be?

**I: Because the question ask about illness that are affecting the child from eating foods that are contain with nutrition**.

R: I don’t understand that question.

**I: Ok. I will find the easiest way to make it more understandable to you. Let’s say are there any sickness he gets by eating healthy foods? Or foods that are not healthy food**.

R: OH… Maybe because he always eat sweets.

**I: So what kind of illness he get by eat too much sweet?**

R: dizzy or nausea.

**I: When he is dizzy he vomit.**

**I: We are now in number six. We talked a lot about being unhealthy. Could you now describe for me a typical day of someone living a healthy lifestyle, from the time they wake up in the morning until when they go to bed? Can you explain the lifestyle of someone healthy from the time he wakes up until he wakes up?**

R: How can I explain that?

**I: I can make an example, someone wakes up and you would say ow the person is healthy so now can you describe a typical day of someone living a healthy lifestyle form the time they wake up in the morning until the time they go to bed? What do the healthy person do from the time they wake up until they go to bed?**

R: They move and all that.

**I: hmmm. So can you explain more on their movement?**

R: They wakes up and do some movement like do some house chores, wash the dishes, and things that they think that they can do throughout the day. What else?

**I: Now moving on, can you describe signs of a healthy child under two years old? This question is most likely the same as the other one that you answered.**

R: The child wakes up without crying, he or she loves to eat, and he also play

**I; thank you now the question ask about old people. Can you describe the typical day of an older or old people**

R: They feel healthy, they don’t just sit down all day, the love to take a walk.

**I: Hmm okay. The next question is let’s discuss hand washing. Could you describe in detail your family’s hand washing throughout the day? Hand washing throughout a day can you discuss in detail how they wash their hand through the day? How they wash their hands.**

R: They wash their hands and their faces in the morning and then come and eat their food. Wash their hand before eat lunch and before dinner. They usually wash their hand before they eat breakfast, lunch and before dinner. Whenever they eat they wash their hands.

**I: So when do you use soap to wash your hands?**

R: One is when you wash your face, then three times as for breakfast, lunch, and dinner.

**I: Now can you explain what do you think is the difference between using water only or water and soap to wash hands?**

R: What?

**I: Can you tell the differences between wash your hand with water only and wash your hands with water and soap.**

R: What you mean water and water only?

**I: The differences when you wash your hands with soap and water and with only water itself?**

R: The differences with water is that bacteria can remain in people’s hand not like the soap when we use the soap to wash hands, it kills bacteria.

**I: Now can you tell me anything that prevents washing hands with soap throughout the day?**

R: Sometimes we forgot to wash hands, sometimes we are too lazy.

**I: Now we are on nutrition. Now we would like to talk about your diet during pregnancy and breastfeeding**.

**I: Now I would like you to think back to when you were pregnant. Can you describe your diet when you were pregnant compared to when you were not pregnant? Like can you describe food you used to eat during pregnant compared to when you were not pregnant?**

R: When I was pregnant, I loved to eat only local foods

**I: Like?**

R: Pandanus, breadfruits, papaya and piknik mix it with the cool aid.

**I: Now as of pandanus and breadfruits where do you get them, you buy or what?**

R: I buy them from the store.

**I: Now could you discuss what influenced your diet during pregnancy?**

R: What influenced me?

**I: From what you wanted?**

R: Yes from what I wanted to eat.

**I: What foods was encourage to you to eat during pregnancy and why?**

R: They told me to eat fruits, nutrition foods, the foods were healthy for me except that sometimes I eat cool aid and piknik. And yes I was told that the piknik and the cool aid is bad and too dangerous for both me and the baby it can cause like what, it too hot and… yes as of the cool aid it also can cause the health of the baby.

**I: Now moving on, who who….**

R: Who what?

**I: Who encouraged you to eat these food during pregnancy?**

R; Just me.

**I: Who primarily cared for or supported you during pregnancy?**

R: Supported me?

**I: Supported or cared for you during pregnancy?**

R: My mother.

**I: Now can you describe in detail on ow your mother supported you during pregnancy?**

**I: From the first time you were pregnant and all that.**

R: She gave me everything that I need as a pregnant lady. For example, I do not have a job, she offered me money so that I can buy food that I want to eat.

**I: Ok now can you tell me about any supplement you took during pregnancy?**

R: Supplement that given from hospital like vitamin, medicine for blood and yes.

**I: Ok. Now supplement that was given to you were you able to take them all or drink them?**

R: Yes.

**I: Now why did you take these supplement that were given to you?**

R: HMM? Because they told me to drink them

**I: For?**

R: To prevent sickness. Let’s say to help the baby grow healthy inside the womb.

**I: Did you drink alcohol, smoke, or use other drugs during pregnancy?**

R: I did not. Like that was before

**I: ohh?**

R: That time I never realized that I was pregnant. I was three month pregnant when I realized that I was pregnant while I was drinking. There were like signs that let me know that I was pregnant. It surprised me by hate different kind of alcohol like vodka, and I took the pregnancy test and I found out that I was pregnant then I stopped I started hated drinking alcohol.

**I: Great. You have great answer. Thank you for answering. Now can you tell me were there any Marshallese traditional that you took during pregnancy?**

R: Yes the medicine for (ass) ** painful thing that the one that was given for me to drink.

**I: Was is a seriousness sickness?**

R: Yes. It is more painful than giving birth.

**I: Can you tell me more on how painful it is? Ow and don’t worry only ladies are there to check our recording.**

**I: Can you share you experiences on that ass painful compared to giving birth?**

R: When I get that kind of sickness, I couldn’t pooped but I couldn’t able to. And it took one week long for me to suffer from that sickness. Yeah like one week long I suffer a lot. I was pregnant to my female child and my mother was the one who use to make me the Marshallese medicine for this kind of sickness. I freed from the sickness when I was four month pregnant. Like I never eat because I never want to poop even though I was too hungry, I couldn’t eat. But yes this is the most seriousness sickness for me during pregnant. Even though I was too hungry, I couldn’t eat because I couldn’t pooped because the pain is in the spot where I used to poop. The painful spot was too big and too painful for me. So they took me to the hospital but I refused to go to the hospital.

**I: Why did you refuse to go to the hospital?**

R: I was scared and afraid at the same time. So they tried Marshallese medicine for me and then they found out that I was been blacked magic by others.

**I: hmmm**

R: soooo. An old lady, from Maloelap, she came and checked up on me and tried the traditional medicine and then she found out that…. Like what. Or how can I say it? Like she tried to swift away the illness and at the same moment, the thing popped out. The evening after the illness popped out I was able to use the bathroom. It was too painful but I tried my best so that I could drained out urine and pooped from that I hold for too long. The ass painful is really bad for women especially pregnant women. The paint in my pooping spot scattered and reason why I say that it is more painful than giving birth.

I: If you were advised to eat more fruits and vegetables during pregnancy, could you describe what would make this difficult?

R: uh?

**I: If someome was encouraged you to eat fruits,**

R: I don’t think someone has told me to do so.

**I: ok no problem… moving on to number eleven.**

**I: Can you describe your diet when you were breastfeeding?**

R: long pause

**I: What kind of foods you used to eat when you were pregnant?**

R: Foods like fish, most of the time fish, corn beef, because we believed that they create breast milk a lot.

**I: ok that what make breast milk.**

**I: Now who encourage you to eat these food during breastfeeding?**

R: I heard it from people

**I: Moving on… After giving birth, could you describe breastfeeding your child throughout the day**?

R: long pause

**I: The question is asking on how long you did breastfeed your child throughout the day.**

R: How long?

**I: Yes how long.**

R: It took one hour and few minutes. But I feed my child both from breast feed and the baby bottle.

**I: So from what month you started giving him baby bottle?**

R: From the time I gave birth to my child because I did not have enough breast milk to feed him/her.

**I: Now how much milk you fed him with? Like how many time you fed him throughout the day?**

R: I started feeding him with different liquid like other milk bough from the store one hour after born. I made the baby bottle for him with different liquid like the milk for baby, what I did was put two ounce of water and after I feed him at eleven thirty, and then at twelve thirty I would also feed him the same ounces of milk and water or when I have enough breast milk I give him, but when I do not have enough, I give him the baby bottle. The reason why I feed him with different liquid is to feed him until he is full.

**I: so you mean even now he is feeding from your breast milk, you still can’t give him enough to feed him?**

R: He really can eat from the bottle because he is use to it.

**I: So when did you stop giving the baby bottle for him?**

R: I think seven or eight month?

**I: So you think you gave enough to feed him?**

R: Hmm yes.

**I: Now could you tell me when you first gave foods and/ or liquids other than breastmilk to your child?**

R: You mean gave him other liquid like..

**I: Yes like milk and …**

**I: Yes it is clear that you said that you did breastfeed to him since he was born, you feed him from breastfeeding.**

R: Feed him what?

**I: Food like real mean, when did you first gave him foods like that?**

R: When he was five month, I first gave him the cereal he first stated with the cereal. Just the milk mix it with cereal.

**I: Now what are other’s opinion that influenced their decisions to introduce foods and liquids at that age? Do you know?**

R: I do not know

**I: Ok skip that since you said you do not know.**

R: Are we done yet?

**I: Giggling,, please bear with me we are almost done.**

**I: Now we are trying to understand how people eat in this community. Could you describe in detail what your family usually eats and drinks throughout the day?**

R: They eat rice with meat. Like the can meats, sashimi, chicken, soda, coffee, tea.

**I: Can you describe in details from breakfast, lunch, and dinner?**

R: For breakfast, pan cake, bread, and oatmeal, and for lunch, rice, chicken, sashimi or fish, and for dinner, rice or pandanus.

**I: Could you describe the process of how meals are made? Like the process of doing meal for breakfast, lunch and dinner?**

R: What you mean how? We just cook them.

**I: Yes. How do you cook the foods? Can you explain in detail how you cook the foods?**

R: Pan cake?

**I: Yes as of pan cake, can you describe in detail the process of cooking pancake?**

R: The pancake, we just bring all the ingredient and put them all together and then just cook the pancake. As of rice, we just wash the rice first and then boil it with water, and then for chicken, we fried the chicken. We would take it out from the freezer ant hen put them in a container and then put water so that it would not be cold anymore. After, we put different recipe like put the garlic, soy sauce, it depend when we want to eat fried chicken or boil chicken.

**I: Now who in the family is served first, next and last?**

R: Everyone get their foods at the same time.

**I: Now are there any differences in the foods served to different family members?**

R: No. Everyone have the same kind of food.

**I: Do some children receive more food than the others?**

R: No

**I: Now can you describe any food sharing between family members during mealtimes (for example children eating together separately from the family, meals eaten from the same plate by all family members)?**

R: Like I said, if the meal is cook and ready to serve, it depend on whether the person want to eat that time it depend if he or she is hungry or not they can eat right away or can wait later on when they hungry. But we often serve the children first, as for me, when I feed the baby, the children and then last, the family member.

**I: Now does the family share food between households for example sharing food with neighbours?**

R: Yes when they come near the house and see that we are eating, we would call them and tell them to come and eat, but yes sometimes when the foods is enough to share then we share.

**I: Now we have heard from some families that eat foods whereas others et processed foods. Could you explain what is typical for your family?**

R: Mostly they eat rice and process foods from western country. Like rice, chicken, and fish this family often eat.

**I: So you meant that they usually eats from imported foods?**

R: Fish is not imported food it’s a local food. We eat balance from both imported and local foods. …. Ow and oat meals too that really important.

**I: What make it is easy or difficult to make local food?**

R: Hey?

**I: What makes it easy to cook or difficult to cook local food?**

R: What is that mean?.... Propane or what?

**I: What makes it difficult for you to cook local food?**

R: Nothing. Sometimes we eat Marshallese foods.

**I: So what are the positive or negative things about Marshallese foods?**

R: There are no negative things about Marshallese food.

**I: So what are the good things about Marshallese food?**

R: They’re delicious

**I: I am really sorry for these kinds of questions. This are the questions that should be asked out.**

R: That’s okay don’t worry.

**I: What are the positive or negative things about eating processed foods?**

R: One example for chicken, when we eat chicken a lot, it makes my foot feel hurt or my body does not feel the same, it’s really disturbing maybe because they contain too much cholesterol.

**I: hmmmm**

R: But yes they can be good because they are delicious too.

**I: Ok. Now that we have talked about how the family eats, I would like to learn more about how your child eats. Could you describe in detail what your son or daughter under two years commonly eats throughout the day? Foods that your child eats throughout the day**.

R: He usually eat rice and sausage and bread sometimes for snacks. For breakfast, sometimes he eat ramen and egg or pancake.

**I: So how many times a day meals and snacks are eaten by your child?**

R: …long pause

**I: How many times you feed your child throughout a day?**

R: During breakfast, and then snacks, then lunch and then snack again, and the dinner. That count as five.

**I: So how do you know that the child has had enough food to eat?**

R: I feed him until he refuse to eat

**I: Ok. What can you do to encourage your child to eat when he do to eat? Not when he do not eat when he refuse to eat.**

R: Give something that he might like so that he can be able to or like to eat.

**I: Now what can you do to the child when he refuse to eat? The word refuse is different than the word do not**.

R: Give him traditional or medicine for feel dizzy.

**I: So you use traditional medicine or medicine from the hospital**

R: Traditional medicine like that one that’s the one I give him

**I: Ok now can you describe how do you do it in detail?**

R: I would bring the medicine from the noni tree root and pound it and then wrap it in a piece of cloth and then give it to him so that he can smell it or inhale in the medicine.

**I: Now are there any differences whether you feed the child when he is sick for example when the child is acing diarrhea?**

R: silent

**I: The question is asking on any different when feeding your child when he is sick from the time he is not sick? Are there any differences? Are there any differences by feeding your child during sick or when he is not sick? Example for diarrhea, when he is having diarrhea what are the differences when he eats when he get diarrhea from the time he is not having diarrhea?**

R: He sometimes go out.

**I: Hmmm. The differences?**

R: He always drink water.

**I: Number eighteen, we are almost done** **…**

R: How many more?

**I: hmm. One two three four five six and seven. It’s going to be real quick bear with me. If we answer these questions good and understandable we’re going to finish this things real soon.**

**I: Now you have told me what your child under two usually eat. Now could you explain to me the process from start to finish, how you prepare and cook a meal for your child? How do you prepare or cook the food for you child?**

R: What is that mean?

**I: Can you describe in detail how do you prepare or how do you cook the food for our child throughout the day? What do you do? Like the little kids for example, can you tell me the ingredient?**

R: Now that he is old enough, he can eat with us (the family member)

**I: What about the time when he was a child? Like you said you give him the pandanus juice, how did you make the pandanus juice for him?**

R: When it’s time for him to eat, I would take out the pandanus juice and feed him with it.

**I: Now as you take out the juice, is it hard or soft for him to eat?**

R: Medium size. Soft as the oatmeal. I use the spoon and feed him.

**I: Sometimes other mothers would put it in baby bottle and feed babies right?**

R: Yes.

**I: So how do they do it?**

R: They ummm, do it like, put four ounces of water in the baby bottle with two table spoon of the pandanus juice then shake the bottle and then it became the real pandanus juice.

**I: Now could you now tell me what you think are important foods for your child are under two years to grow well /be healthy? Can you explain what kind of foods your child under two is to be eaten in order to be healthy?**

R: Orange

**I: Orange?**

R: Orange and what else, sometimes he would ask for ice cream

**I: This question is asking about important foods the child supposed to eat to be healthy. You cannot give him, but are there any important foods that are supposed to be given to your child so that he can grow or be healthy?**

R: I don’t know what it would be because we give him rice so that his body can be energized

**I: Rice? Ok**

**I: So what kind of foods that are not supposed to be given to the child under two years old?**

R: Cool –aid and things like that

**I: Ok so the question says, why it should not be given for the child?**

R: Because they will have kidney problem or something like that.

**I: Now do you have the biggest influence on feeding child?**

R: None

**I: Can you describe any differences if any between how you feed your male children and how you feed your female children under two?**

R: There is none

**I: hmmm we are on the last page.**

R: Now how many more?

**I: We have five more questions. (Laughing)**

**I: OOWW**

R: What time is it?

**I: We have only minutes not even an hour now.**

**I: You might think it’s too long, but because it took long time for us to do our conversation**

**I: ok. Could you describe the care of children throughout the day in your community?**

R: What over them from harmful things… for example they can slid by the door or just any harmful things. Watch them from eating trashes or dirt.

**I: hmm**

R: They are kids they do not know whether it is good or not for them to eat.

**I: So who is responsible is taking care of the child?**

R: The mother

**I: Can you please explain the roles and responsibilities of a mother in this community?**

R: The roles and responsibilities of a mother is to do baby- sitting, cook food for the child and people in the house, do house chores, make sure the students ready to go to school, ( if any) or get the husband ready for work something like that. Everything are the responsibilities for a mother. They do not sleep well because they take care of the child throughout the night. Something like that

**I: What about roles and responsibility of a father?**

R: hmmm responsibility of a father……..what kind I say? They also help take care of the baby or they work and make money for the family.

**I: Now this question is how caregivers play with children under two years old? How do you take your time and play with them**

R: We both watch movie, play with each other, play hide and seek, something like that, or can be stroller on the road.

**I: Could you talk about the role of grandparents have in raising children in this community?**

R: When they come back from work, and see that the child is poop they can go ahead and clean the child up from the popping. They also can feed the child when they see that the parents are not here they do our responsibilities.

**I: What ways that grandparents support in raising children, support mothers and families/…. What makes good grandparents (grandmother/ grandmother?)**

R: What is that mean?

**I: Like what are the good things grandparents do like help so that they can be good**

R: Who can be good?

**I: The grandparents of the children**

R: They play with the child or they also watch over them from harmful things, buy those candies and buy those toys and many more.

**I: Could you talk about the role that other family members have in raising children in this community? Like for example, aunties or uncles siblings... Yes**

R: They watch over them like parents do. They also take them out and buy them food, carry them around the places.

**I: Now how do siblings or older siblings help raise young children?**

R: hmm?

**I: How do older sibling raise their younger sibling?**

R: Play with them

**I: You have really great explanation and we are almost finish. We are on the last section**

**I: Could you explain where you usually get trusted information about nutrition and health?**

R: LOOONG PAUSE.

**I: Could you explain where you usually get trusted information about nutrition and health?**

R: silent moment… hospital.

**I: Why you trust where these sources came from?**

R; because they are nurses and doctor and they know everything better than us.

**I: Ok. So where do you think nutritious and health messages should be delivered so that you would see or hear them most easily?**

R; what is the meaning of that question?

**I: Like where would these information should be delivered so that you can hear them every day?**

R: Sometimes they bring them to the radio station radio B7av.

**I: Last question: or let’s say second to the last. What types of media that you use the most to communication.**

R: I don’t know

**I: what kind of media you usually use?**

R: Cell phones and things like that.

**I: When you think of raising child, can you explain the influences of raising a child?**

R: What is that mean?

**I: Like are there any differences when you raise a child from others?**

R: Differences. There is no differences

R: Last question right?

**I: Yes but the last probes and then we’re finish.**

**I: What** are other people’s thinking about raising children in this community?

R: hey?

**I: This question is asking about people in this community opinion about raising children. Like the land owners, leaders, church leaders, health care?**

R: They said that raising children is not an easy thing to do.

**I: Were there any advice or information related to parenting you received?**

R; No. there is none.

**I: Are there any desired information on parenting you wish you had but you do not have available?**

R: No

**I: Ok thank you so much we’re finish. Thank you soooo much**.
