# Supplementary material for: Conceptualizing multi-level determinants of infant and young child nutrition in the Republic of Marshall Islands–a socio-ecological perspective
Source: PLOS Glob Public Health. 2022 Dec 19;2(12):e0001343. doi: 10.1371/journal.pgph.0001343 (PMC10022247; doi:10.1371/journal.pgph.0001343)
Supplement: S1 Data — (ZIP) [file pgph.0001343.s001.zip › RMI Supp Data/Interviews data/I16U_IDI_FCG_Rita_Aug 14_Meia_Showme edited_FelaShanteEdited.docx]

- Interview Code:I16U
- Interview type and interviewee: IDI FCG
- Interview Date: Aug. 14, 2018
- Interviewer: Meia
- Transcriber: Showme Lelwoj

**I. Do you want to move forward with our interview today?**

R. Yes

**I. Okay, thank you, now we can move forward. These information we are looking for is to help out mothers and children’s and also the hygiene of our islands, to begin, could please tell me a little about your family and household?**

**R. Like what?**

**I. Like, Who lives with you, you don’t need to tell me their names. Just tell me who lives here, how many children, how old they are, how many girls and boys?**

R. There is me, my husband, my two kids, my mom, my dad, and there an older sister and her husband but they have no kids. It’s just only us here. I’m only 22, my husband is 25, my youngest boy is 8 months old, and my eldest boy is 3 years old, my dad is 50 years old, mom is in her mid-40. My older sister is 25 years old, her husband I’m not sure how old he really is.

**I. Now, I’d like to ask you about your community or about you home?**

R. Here the community I stay at?

**I. “Uh Huh”**

R. This community I stay in is a good one and it’s where I grew up in since I was very small to nowadays. Everything here seems good to me.

**I. Now are there any negative in this community?**

R. There aren’t any negative in this community.

**I. Now, we will talk about health and illnesses in the family, Can you tell me about some illness that your children have suffered from?**

R. One very constant illness that my baby boy tends to have is fever and its long has a week and a half. For the girl she been good lately.

**I. Now what is the cause for your boy to have fever?**

R. The sickness when it flows around he tends to get it from others who have it.

**I. Do you think when he has fevers is it serious to you?**

R. It is not.

**I. Can you tell me any ways to prevent fevers?**

R. I just give them medicines.

**I. Yes take medicine, who does your child see first when they need treatment?**

R. When it gets really serious and when he starts breathing heavy and it’s hard for him to breathe. When that happen asthma occurs and it is really serious when he takes it. Especially when he starts getting dizzy.

**I. when you say dizziness what is that do you see from looking at him?**

R. He gets weak, he tends to eat less than usual, and he cries a lot. All these things.

**I. Who does your child sees first when he gets really sick?**

R. I usually take him to see his doctor and I take him there because it’s the place that can take his illness away. They also give him medicine

**I. Do you use traditional medicine?**

R. I do not.

**I. Good, now can you tell me what kinds of illnesses that occur from the food they eat?**

R. Yes there is some foods that causes like diarrhea, they are not well because of what they eat, some foods that we feed them we tend to forget to heat them up and when we feed them they tend to get sicker.

**I. Can you tell me what illnesses they get when they have no healthy foods in their diet?**

R. Yes there are I think like rice and things like these. I think there would be no health in rice and flour…

**I. Could you please describe for me a typical day of someone living a healthy lifestyle, from the time they wake up to the time they go to bed?**

R. They are more wide awake and feeling good about themselves, they’re not lazy, always busy all the time, moves around a lot and their basically the opposite of laziness. Kinds that are not lazy but is always healthy.

**I. Could you tell me what appearance and signs of a healthy 2 years old?**

R. When they signs of getting bigger, they’re appearance looks good and it’s not like all the time their sick. Always in a good mood, he is always laughing and smiling all the time. Plays fair with other children’s.

**I. Now could you please tell me about what appearance and signs of a healthy adult?**

R. Well, my dad is a lot more different than before because he has gotten skinnier and has been through many sicknesses.

**I “Uh huh” can you tell me about hand washing in your family in a whole day? How do you guys wash you hands?**

R. We use soap and wash our hands.

**I. Now, how do the children wash their hands?**

R. Well we the adults wash their hand, like me I usually wash the children’s hands before they eat. Then when it’s time to eat they will eat.

**I. How many times is soap used to wash hands during the day?**

R. We always use soap to wash hands. Times like when we do work in the house, we wash our hands, times like eating we also wash hands, yeah all the time we wash our hands.

**I. Can you explain to me the differences in hand washing with just wand and hand washing with soap?**

R. When we use soap and wash our hands, the soap will kill bacteria from our hand and I think it’s no different from just using water. It’s like using soap.

**I. Now what prevents you from washing your hands with soap though out the day?**

R. We are always in a hurry to do things. That we tend to forget to wash our hands. Out thought will go for what we are doing that keeps us from washing our hands

**I. Now, could you think back to when you were pregnant and can you describe your diet when you were pregnant compared to when you were not pregnant?**

R. I ate a lot of choice ramen, which was the only thing I ate. Everything else I never was into them, so I ate just choice ramen. When I ate other foods I didn’t like it.

**I. Now I want to know what your reason for eating just choice ramen was.**

R. I only crave only for choice ramen. In my first trimester I always want to eat choice ramen.

**I. What foods were there that you were encourage to eat during your pregnancy?**

R. They wanted me to eat nutritious foods, like Marshallese foods such as pandanus, and fruits like these, my boyfriend would bring me apple and things like that.

**I. Oh okay, now what foods were there that your parents or boyfriend discourage you not to eat?**

R. There was choice ramen, salty foods, salt dried fish, Kool-Aid with salt and these things.

**I. How come you were not supported with these foods?**

R. Oh because it was not good for my child. Plus it wasn’t good for me as well.

**I. Who supported you during your pregnancy?**

R. Going inside, or what did you mean?

**I. From the time you started being pregnant till you gave birth, who supported you?**

R. During the time I was pregnant, my mom usually gave me massages when I was hurting, and my boyfriend as well during that time I was pregnant. During these times.

**I. Can you tell me any supplements you took during pregnancy?**

R. I took the vitamins that pregnant should take while their pregnant. I also took pills for blood.

**I. Did you take all the supplements given to you?**

R. I did not take them all because I usually was vomiting a lot and I did not want to eat any foods. I was about 4 months into my pregnancy and I stopped taking these medications because I was always vomiting. When I drink them, I always vomit.

**I. Did you drink alcohol, smoke or use any other drugs?**

R. I did not do or take anything during the time I was pregnant.

**I. Did you ever took any traditional medicines during pregnancy?**

R. There was none that I took. I did not.

**I. If you were advised to eat fruits or vegetables during pregnancy, could you describe what would make this difficult?**

R. When there was no money to buy them was the only way it was difficult to eat these things.

**I. What would make it easier to eat more fruits and vegetables?**

R. If there was money to buy these things.

**I. After giving birth, could you describe breastfeeding your child throughout the day?**

R. I ate store bought products like can meals, chicken, and fish.

I. Okay, Why do you eat fish?

R. Well because my parents say fish will produce more breastmilk.

**I. Did you give any other liquids did you give to the baby in the first few days after birth?**

R. There was none.

**I. Now can you tell me anything that makes it easy or difficult to breastfeed exclusively up to 6 months and reasons why?**

R. There wasn’t any.

**I. Okay what made it so easy for you to breastfeed till he was 6 months?**

R. Oh because all the vitamins are all in the breastfeeding and I’m used to breastfeeding my kids because I do not use baby bottles or anything else.

**I. Can you tell me what makes it easy or difficult to breastfeed up to 2 years?**

R. It kind of depended on my child because if they would have stopped breastfeeding but if they did not they would have just kept going.

**I. Could you please tell me when was the first time you gave your child food instead of breastmilk?**

R. When he was 6 months old.

**I. Why did you introduce foods or liquids other than breastmilk to you baby?**

R. The reason why I fed him when he was 6 months old was because during this time it when they start eating from my understanding. When my family and I were to eat, he would drool and want to eat with us. So I immediately thought he wanted some food. So I just stared feeding him since that time.

**I. Any opinions from others that influenced their decision to introduce foods and liquids at that age?**

R. There weren’t any.

**I. What was the first food were and how they prepared?**

R. The first thing I gave to him was baby foods from the store. Like the rice cereal. This was the first thing that I ever fed my baby boy. Sometimes there was pandanus. The baby food I used fresh milk to mix it with it. With the pandanus, I boiled it and take the liquid textures from it.

I. Did you feed him with a spoon?

R. Yes I fed him with a spoon.

**I. We are trying to understand the people in community. Could you describe to me what your family eats and drinks throughout the day?**

R. In my family there are those who are ill, like my parent’s diets are different from the rest of us. So they eat like brown rice and these things, there only for them two. As for the rest of us we eat white rice and different kinds of meat products.

**I. How are the meals made?**

R. In our household we usually cook food and if we are slow to cook, we buy them at the stores. In morning time if we over sleep we just buy breakfast in the mornings from the stores.

**I. How about drinks?**

R. We drink from the water catchments here at home.

**I. In your family who is served first, next, and last?**

R. First my parents and children are fed first then the rest of us who are strong enough there won’t any problems for us.

**I. Are there any differences when you serve meals to your family?**

R. There are no differences.

**I. Are there any differences between the quantities you serve to your family members?**

R. There is not.

**I. Do some children receive more than others?**

R. There are no differences. Like if one of my girls were to eat they would eat with the same plate. But as for my boy, he is different.

**I. Are there any food sharing between family members during meals, example children eating together separately from the family, meals eaten from the same plate?**

R. The amount I give. Sometimes they would serve themselves. The amount they want they would make their own amount.

**I. Any food sharing between households? For example, the children would eat with each other or do everyone eat with one plate?**

R. It’s different. The children eat by themselves and the adults eat by themselves.

**I. Now do the family share food to people around their surrounding?**

R. Yes. We share food when there are a lot from my family or when my next door neighbours have a lot of food they would share to us.

**I. We have heard from some families eat local foods whereas others eat processed foods. Could you explain what is typical for your family?**

R. There are times we eat local foods as to processed goods we eat most of the times.

**I. Anything that makes it difficult to cook local foods?**

R. It’s sometime difficult because it’s kind of rare to eat local food. When we want to eat local foods there kind of far ways to find them. Then when there aren’t any around we go and buy them at our local markets. Other times we have local foods from my dad’s family’s side from the outer islands.

**I. Now can you tell me anything positive or negative things about eating local foods?**

R. There are some negative things about some local foods because they are too greasy. For pandanus it’s too sugary and we easily get diabetes.

**I. What’s greasy that you mentioned?**

R: Oh pigs.

**I. Can you please tell me what any positive or negative things about processed foods?**

R. There are some processed foods like rice makes us people in the Marshall Islands have more diabetes. Other are not good for us.

**I. Like? Can you be more specific?**

R. Like rice and those sugary goods.

**I. Are there any positive aspects about processed foods?**

R. Yes

**I. Can you describe to me why you say yes?**

R. When I eat processed foods they are good. Like rice I get full as well as bread.

**I. Almost there, thank you for all your information’s, there really great. Now can you describe in details what your son under 2 years commonly eats throughout the day?**

R. In the morning, he eats ramen noodles, cooked bread. In afternoon, he eats rice.. In the evening also rice and when there is no more chicken, he eats store bought can foods.

**I. How many time a day meals and snacks are eaten by him?**

R. He eats from morning, afternoon, and dinner. I usually give him snack every time he eats during the day and whenever he craves snacks.

**I. How do you know that your child has enough to eat?**

R. Whenever we try to give them food, they are not likely to want to eat it because they will just turn their heads away and that how I know.

**I. What do you do to encourage him to eat?**

R. I figure it out, by seeing what he wants to eat.

**I. What do you do to when he refuses to eat?**

R. I figure it out myself so he can eat.

**I. Do you feed him differently when he is sick?**

**R. Can you repeat that question?**

I. Do you feed him differently when he is sick? for example like when he has diarrhea, is there a difference in how you feed him?

R. Yes there is a different, when he is having diarrhea, are times he doesn’t want to eat but he only wants to drink and give him medicine, you know like the one where their body doesn’t get to dry. I usually give them this medicine so that their body won’t get dry.

I. This medicine, you get it from the hospital?

R. I got it from the hospital.

**I. Can you describe to me how you prepare your baby boy’s foods?**

R. Some times when they don’t want to eat from the cooked meals, I usually find food from the store and feed him soup or things like that. When they don’t want to eat, they usually drink water. That’s because they’re stomach is upset or have diarrhea and things like that

**I. What types of foods do you think is important for your child? How do you prepare and cook a meal for your child?**

R. First I bring water from our water catchment or the store. Then I cook with rice pot. Then finally I fill up a plate and make sure its cools down and give it to him.

**I. Could you tell what do you think are important foods for your child to be well and healthy?**

R. Foods that are important like store bought products. Most of the time they eat rice. But not all the time. Some foods like vegetables and the ones with a lot of nutrients in them. Also fruits and those good things.

**I. Now what type of foods you should not give to your child under the age of 2?**

R. Foods like what because all types of foods they eat.

I. No, foods that you are not supposed to give them

R. Oh

I. What we want to know is what foods are we not supposed to feed children under 2 years?

R. Foods that don’t have any nutrients in them like junk foods. Not all the time we feed them these foods. We also would not feed them junk foods, not every day.

**I. What’s the biggest influence on feeding your children, this is a very important question, and we want to know your thoughts and what’s your biggest influence on feed your children?**

R. Yes there are. We really have to feed our kids types of foods that are right for them and their body. So that they can grow better and live better. Foods that have a lot of vitamins.

**I. Can you tell me any differences between how you feed your baby boy and you little girls?**

R. There is no difference.

**I. This is the last page and all thank you so much for all these great information’s. Could you describe the care of children throughout the day in your community?**

R. With my son I look after him, he is a good kid because it’s not hard to watch him and he barely cries and is very different from all other babies.

**I. Who mainly responsible for child care?**

R. Mainly it’s only me.

**I. What are the responsibilities as a mother?**

R. If I was not to watch him, he would not be well. Like if it was not for me, he would not be capable of himself because he’s only a child. **I. Now what are the responsibilities as a father?**

R. Okay his father watches him but not all the time. His responsibility is to buy him what he needs like foods and these things.

**I. Can you describe to me how you would play with your child?**

R. I play with him.

**I. Can you explain how you play?**

R. Like really explain it?

**I. Yes, we really want to know, can you please explain how you play with him?**

R. My son, he likes being silly and played with like when we tickle him, he likes it and make him laugh. He really like to be played with. Sometimes when his older sister plays with him and makes him laugh, he really gets playing with her.

**I. What’s the responsibilities of the grandparents are when they care for the child in the community?**

R. There are times, they both would watch over him when they want me to go and buy thing. Sometimes when I’m tired and exhausted, I would be sleeping and he would play and his grandparents would take him for car rides. They really help out a lot.

**I. Now, what makes a good grandparents?**

R. What makes them good?

**I. Yes**

R. They will watch over him because he is their grandson and he will listen to them.

**I. What do they do that makes them good to him?**

R. They really help me out a lot, when they see me and I’m tired and these things, they would take him from me and take care of him and when he falls asleep. They would bring him right next to me to sleep. The good things about them is that no matter that they know I’m grown, they will take care of him because when they see me tired they will watch him.

**I. Can you describe to me how does other family members care for children in the community?**

R. The other family members?

**I. Yes, aunts and uncles, brother and sisters?**

R. My older siblings would also take care of him but when there are busy and some have their own kids. They don’t seem to be around as much to be there for him but they are wonderful watching him. As for my aunts, they really know how to care for my kids and listen. They like watching children.

**I. when you say they like watching children, can you tell me more?**

R. More of what? Well when they are around and my boy was to be playing on the ground, they themselves would see and come and take them and carry him around. They would be gone for a while and come back to the house and play with him. Sometimes when he was to be laying there, they would go to him and be silly with him. They watch my kids very well.

**I. Okay now, how do the older sibling care for their younger sibling?**

R. You mean my kids, well with my older daughter, her love for her younger brother is not as much, what can I say maybe she did not want a younger sibling because whenever I put him next to her she would beat him up and she would say she does not like it. She says bwebwe (stupid), she doesn’t like him. Sometimes she would play with him but she does not enjoy being with him as much as she should have.

**I. Now could you tell me how does older siblings in other families care for their young sibling?**

R. They watch them, play with their younger siblings, they really watch them because the homes here are right next to the roads. They really watch them to make sure they don’t get in bad situations. They watch them a lot. Whenever the young sibling are playing, they would stay there and watch them till they are done playing. Then they would both leave.

I. **Could you explain where you usually get trusted information about nutrition and health?**

R. Where, the hospital and whenever I used to go there, they would tell me not to eat foods that don’t have any nutrients in them. They also sculled me whenever I were to eat sugary content because I might get sick and have diabetes and things like that. They scull me to no eat foods that are not good for me. I also studied in school and now usually I know because of what I was taught and know about foods. Foods that we need to eat.

**I. Good.**

R. Foods that are good for our body.

**I. Okay, why did you mention the hospital and the other things you said and why are you influenced by them?**

R. For hospital that where we look for being healthy and look for help from doctors. Usually it’s where posters that are put on walls and they show what types of foods we should eat and the foods that are bad for us. From the schools, we study and that’s how we know these things and are really influenced from school and hospitals yes because hospitals are a place to get good health and we really depend on it.

**I. Okay, where you think these information should be at and for it to be easy for you to hear every day?**

R. These information’s?

**I. Where do you think these information should go so it would be easy for you to see and hear about it every day or for people to hear about it? are there a place where it would make it easy to hear about these information?**

R. V7AB or you can make it through online.

**I. What type of media do you use the most to communicate? Do you like use cell phones or the radio or television?**

R. Cell phones and the internet.

**I. Now, when you think of parenting your own behaviours, can you explain what influences you to raise your children?**

R. In what?

**I. When you are parenting.**

R. Like?

**I. When you look at other parents parenting, what is the differences from your parenting?**

R. My children, what going to say. Parenting is hard, somewhat hard because you really need to what, you really need to be focus on your child. You are like what.

**I. Giving your time to them.**

R. “Uh huh” you really need to give them time because they depend on the mother than the father and I give my time to them all the time ad they really need my help and care and love I have.

**I. Any opinions of the community influence how you raise your children for example like from leaders, neighbours, church leaders, health workers.**

R. Can you tell me again sorry?

**I. Are there any thoughts on parenting from other people about parenting that you was influenced by for an example like abusing children? Now we asking are there thoughts from leaders, church leaders, or health workers, about parenting?**

R. For the health workers at the hospitals, they know, they think of knowing if the child was abused because they have tools and these things knowing the child was abused. For leaders they will what, they will enraged.

**I. Is there words from them?**

R. The leaders would scull the people and tell us that it is not right because their only children.

**I. Where/who advice or information came from parenting?**

R. Yes there are, they told parenting it’s a job that is very hard. They said if it weren’t for you, your child would’ve not be anything. because I was to do everything for the children like preparing meals and this is how my mom would give me advice. Parenting is one of the hardest jobs for women.

**I. Is there anything else about the parenting we talked about that you would want to know?**

R. Yes.

**I. What is that you want to know about parenting?**

R. What am I supposed to say? Can I say how valuable it is child care? But I don’t know.

I: anything. This is your time to show what you want to know. There’s no right and wrong in your answers. We just want to know what your thoughts are. If you have any questions, you can go with the nurses at family planning or anywhere else in the Ministry of health to give your questions. But at this moment, thank you so much for all your good information, and for giving me this time to get all your information. Believe that all these information are useful. Thank you so much.
