# Supplementary material for: Conceptualizing multi-level determinants of infant and young child nutrition in the Republic of Marshall Islands–a socio-ecological perspective
Source: PLOS Glob Public Health. 2022 Dec 19;2(12):e0001343. doi: 10.1371/journal.pgph.0001343 (PMC10022247; doi:10.1371/journal.pgph.0001343)
Supplement: S1 Data — (ZIP) [file pgph.0001343.s001.zip › RMI Supp Data/Interviews data/I17U_IDI_CL_Rita_Aug 14_BM.WMA.docx]

- Interview Code: I17U
- Interview type and Interviewee: IDI_CL
- Interview Date: 08/13/18
- Location: Rita
- Interviewer: BM
- Transcriber: Cendaniel Milne

**I: do you consent to proceed on recording?**

R: yes.

**I: thank you for your time, to talk to you today.**

R: yes.

**I: The information we learn here will help us find ways to improve maternal and child health and sanitation in your country. Before we proceed on, can you please describe your role as a community leader?**

R: first, I am a pastor at Majuro independence Baptist. Additionally, I am an administrator or Principal for Majuro Baptist Christian Academy.

**I: great, thank you. Can you please tell me about who is a part of your community?**

R: church members, students, staffs, foreign staffs that we hired from outside so that they can stay in these apartments.

**I: great. Can you tell me about the community structure and hierarchy?**

R: leaders, well first of all, I have two deacons in the church and officers. The officers are the secretary of the church and treasurer of the church. I would also say, the staffs for these students in the school.

**I: good, thank you for the information. Now, other than Marshallese. Who are the ethnic groups and languages in the community?**

R: who or how many?

**I: it can be who.**

R: it can be who, well. There are, there are, a Chuukese, a Pohnepian, three Americans, I don’t know how many that are from outside of Marshall Islands that are not Marshallese.

**I: it’s okay. Now, can tell me about the religion and its influence on community members?**

R: as one of the church members, it is our aim to help people with the Gospel. It is a mandate that every Christians believe in the Bible. It is our responsibility, and it is stated in the school’s hand book to help develop students of the Marshall Islands to become a better citizens.

**I: good, that was an interesting explanation. Now could you tell me about the positive things about your community?**

R: the positive things, the positive things about this community I am thinking. (Statistic wise, we are in the mist of the most populated are in the urban center. And the opportunity to be able to reach out and contribute, be part of people’s lives. Any which way we can try to help) [English speaking] try to help for the better of life, especially when we’re talking about spiritual life. Other ways we can contribute to the community, regarding the young youths, to work with. As a church, there is a group. Ministry of youth that we reached out into the community and work with young youths. I am thinking of something that is good to engage with these youngsters.

**I: great, those are good explanations. These are the informative explanations, because every words you give can help by finding a better solution to come up with. Now can you tell me any negative about your community?**

R: the negative things that affect the community are, I am thinking because of too many people. And there times I am thinking of congested. There are times there is lot children that are neglected. I am thinking there are too many people’s children to be able to support them. As one of the things I see it’s been increasing here at Rita Majuro, there are lot of children that are not in school. And it is one of the devastating thing. I am thinking of that there are other parents that don’t have sense of responsibility of caring for their children’s growth. They roam everywhere, I am doing my best to open this campus where there would be a place for them to play. Opportunities, such gathering them during praying sessions, Sunday schools, and gather them with any programs that would can make them enjoy. Especially teaching the words of God, we believe it will help them in one or many reasons. I am thinking as one of those things that I am unhappy about, neglects people tend to neglecting children.

**I: Thank you for your explanations. But, if there’s difficulties for foods. Can you tell me any food-related difficulties in this community?**

R: well, the difficulties about this community is there is no place to farm. Because it’s been too many people, and it’s hard for us to see. Except we can go the stores and buy, we don’t have any place to grow. There is no place where we can isolate for farming, it is limited. And I think there is none, we can do to farm, except there’s about coconut tree here. Pandanus here, this place is full of people. I think the problem is there is so many people.

**I: great, thank you for the explanations. Let’s now talk about health and illnesses in your community. Could you explain what illnesses children commonly suffer from in this community?**

R: well, because there is a mass screening for leper and TB. It is one of the thing that I heard and see, it is stated that every household. At least, two people with Tuberculosis. From time to time, the illnesses that are familiar to us. Flu, diarrhea, and let’s say pink eye. They’re easily spread, when it’s outbreak in this community. The disease that we see that spread easily excluding diabetes. And I think it comes from the foods we eat, and how we move. Not enough space for resources to exercise, no place to grow nutritious food and local food. These are the things I see there’s a lot to it. Affecting this Rita.

**I: great, great. Now, you mentioned about flu, diarrhea, and pink eye right? Can you tell me what causes Flu?**

R: one of the major problem I see here in Rita, with children. We frequently eat together, drink together, sharing a piece of food, one soda but it can be passed onto 5 to 6 children. Sharing is big, then it is consider to be one of Marshallese sense of responsibility family ties to help out children our family members. But my thoughts about sharing is that it can be contagious too. Drink or drink, water of water. Sharing is big by food, from mouth to others. That’s it.

**I: good, what if.**

R: out clothes too, I can see they can share clothes.

**I: yes.**

R: and I don’t know if this can spread disease but.

**I: yes. Can you describe the seriousness of Flu?**

R: flu, like this flu right? From my own understanding, flu comes and go. It is a way that is important, that we can get the flu because it helps our immune system. I have, a small knowledge from those who work at the ministry of health. And from my own understanding, because I have had the flu many times. But, there are times when I am having the flu, I suffered form it greatly. Frequently having the flu, I thought of when having it could help my immune system. But I have no knowledge of what makes it strong and why, for us in this campus. Even though when there’s visitors, when the flu is strong, pink eye, because we have little in sharing.

**I: good**

R: don’t usually have it, because we’re in a surrounded campus.

**I: and now, from your own understanding. What ways to prevent flu?**

R: I am thinking, when our foods good. And we move a lot. Work or work, exercise. Be more cautious about someone who has it. And we go to the hospital and look for medications.

**I: now, you mentioned diarrhea. What causes diarrhea?**

R: I am thinking, they don’t do sanitation. Doesn’t matter if this is an urban place, but people can do their own toilet place, they do use it a lot. And they do use it a lot, I am thinking if. When they use the ocean side, there’s flies and the flies land on our foods. From my own understanding, they fly in with the disease and especially when we don’t prepare our foods neatly. And there are times when don’t carefully prepare our foods, we don’t look into the meats, how we fridge them in a way they can still fresh, that they won’t be spoiled. I am thinking these are the ways, we’re in lack of storing them perfectly.

**I: great, you explain perfectly. And, from your own, what are the seriousness of diarrhea?**

R: I think it’s not too serious, about average. Because I haven’t seen any deaths about it, but there’s times when taking medications from the hospitals or over the counters at the stores. For me, when I am taking these medications it’s gone. For me, it is not a life threatening.

**I: yes... but, you also mentioned about pink eye. Now, from your own, what causes diarrhea?**

R: from my own, lack of sanitation. But, as we understand about it, we usually have it from those who are contagious. It’s contagious, from us, but it comes from person to person. And lack of sanitation by washing their hands, there’s sanitizers, we have soap to clean.

**I: great, but pink eye, what you think of the seriousness of pink eye?**

R: I think it’s not so serious, I’d say on the average of 3, I would say diarrhea would be 2 but pink eye is 1.

**I: good, good. You explain it amazingly. Now, can you describe on how to prevent pink eye?**

R: prevention, that’s what I am thinking, sanitation. Hand sanitizer, there was how many? Two years past. It was a disastrous, we hear and saw ways to prevent us from having pink eye. I am thinking of this way, sanitation. The truth is, there are times pink eye is huge here on Rita Majuro. But, my family they don’t, because were well careful of. If go somewhere else, wash your and before you eat, wash your hand.

**I: great, well. We’ll proceed on to the next question. What type of treatment people in your community seek for your children, for example traditional healers, doctors, nurses?**

R: healing them as if they…?

**I: if, for example. If they’re one of those traditional healers, doctors, and nurses.**

R: and the question is, what are…?

**I: what does the people in the community, or what does the people in the community seek to treat their children?**

R: What I frequently seeing is that, we always seeking the hospital. Just like the Rita people, when having sickness they go to the hospital. Unlike the outer Islands, we tend to make our own traditional medicine. But, what I am seeing is we always go to the hospital.

**I: from your own, in this community, do you see anyone who use traditional healers or traditional medicine?**

R: I usually heard, but I haven’t seen any with my own eyes. I usually heard, but I think they always go to the hospitals instead of using local medications.

**I: good, now can you describe any illnesses associated that affect children in your community.**

R: sorry, could you repeat that again?

**I: Pardon, what kind of illnesses in foods that affect children in this community?**

R: okay, what kind of illnesses that affect the people?

**I: children that…**

R: children.

**I: from food.**

R: well I have no knowledge, when it comes to food, I have no idea of what we see in this place about food.

**I: when you, let me explain, or I can rephrase this question. It can be, what foods that makes children unhealthy? And why?**

R: I am thinking it is huge, what I see is we is huge giving junk foods. Candies, sodas, it is big on these here on Rita. What I am thinking, children get malnutrition because of junk foods in the stores that sell them and soda. It is something that is big.

**I: yes, but, what types of foods that make the child’s body healthy?**

R: from my own, as I’ve seen them healthy, live, and grown is their food. Even though they may not enough, but as I’ve seen from my grandparents. They didn’t have diabetes, they didn’t have an early blind eyes. Now, I am starting to have blind eyes. High blood, they didn’t even have these diseases, NCDs. They didn’t feel them, and I am thinking it’s our own foods in our country. But for us, we like rice so much, I am thinking rice is good but not every time. And people eat rice so much.

**I: perfect, well. We have talked about health. Now, were going to talk about food that are commonly available in this community. Now, can you explain how people in your community typically get food to eat on a daily basis?**

R: well, they need to work to buy their own foods. But I am thinking not enough money, frugal. Maybe not enough vegetable, fruits, juice, foods that they don’t have to buy or bring to plant. Foods that are nutritious.

**I: good, perfect, but as you walk or move here in Rita, what foods are commonly grown in Rita?**

R: there are few, breadfruit trees that I see, but the frequently seeing growing here on Rita are Pandanus and coconuts. These are commonly grown foods that grow increasingly. As I was saying there are few breadfruit trees, but there’re grown at the far end of Alwal (village name) where there are few people. The most thing that commonly grown is Pandanus. Secondly, coconut tree. Between these two, breadfruits are few, but don’t usually ripe like the coconut trees from outer Islands. That’s about those.

**I: okay, what about the foods grown at home that are eaten by families, do they eat them or sell them?**

R: well, from what I am seeing. They usually eat them, but there are those who would steal them and sell them. I don’t believe if there are those selling them. Because it is rare, they usually eat them. For instance, children would take coconut trees and drink them.

**I: now you mentioned about stealing, what do they do after profiting from selling them?**

R: well you know, young men, they’ll be drinking alcohol. Smoking, chewing, these three things that they get busy with and addicted.

**I: now, can you tell me about any difficulties to grow food in this community?**

R: I do not believe there’s much plant’s food in this environment because we do not fertilize or compose the soil. This house and this house, there is no soil for the roots to spread. It’s not growingly spread on the soil, I do not believe the soil has enough nutrients in it. Because of crowd, and our development of houses. These things that are everywhere playgrounds.

**I: great, perfect. Now, from your own, what do the community need to grow foods in this place?**

R: need right? I am thinking we need to reduce the crowd, make space for each household. That there can be a farm of their own, I am thinking is how that can it possible. We can do many things but, as long as there are many crowd, there won’t any foods that can hardly to grow. And if it grows at this house, the next door can steal it. It would young men, young men, we know because we have done these things. I am thinking if there is space, there will a place the foods can grow.

**I: great, now could you explain how easy or difficult it is to get those foods you mentioned every month during the year.**

R: it is very difficult, the only way is, and you need to get a job to be able to buy food. Because we can’t, when it comes to growing foods in Rita, there is no chance. This is a way that we can have food. It is clear that it’s difficult, it is difficult because there’s no place for growing foods.

**I: perfect, can you explain the food shortages throughout the year and their main causes?**

R: food shortage here in Rita is out of stock of Rice. But, I don’t believe the shortages of food because it is a stable food. That many think is, even though people don’t have enough money. It is hard, as I am thinking. They prioritize food so there can be food. When there is a drought, there is not enough food.

**I: great, now as walk, what kind of animals you see in this community?**

R: first, dog, cats are precious to people, in the trashes which there are many. I’ve seen rats, at the back side where there is a trash place. I think dogs can be seen everywhere.

**I: now from your own, why are these animals raised by the community?**

R: first, sorry, pig, because I have seen pigs except dogs and cats. People tend to like dogs, there is many times where we see people raise them it’s for the children as a pet. And cats too, dogs are not just for pets, but it’s for guarding our homes. These are the ways we see, as we been valuing dogs. It doesn’t matter here, or outer Islands we been valuing dogs. Pigs can be raised for occasional events such as Christmas, birthday, and these are the big events that I see people doing for it. They don’t kill them for no reason, there are those who does it. They raise them to ensure there’s pigs for those important events. And these are reason for birthdays, and Christmases.

**I: but, are there any difficulties of raising these animals?**

R: the difficulty is that its dirty, lets the difficulty is there are limited space. And now, there are regulations of how to raise a pig. There is no difficulty in dogs and cats, from what I see, because people don’t have any complaints against them. But for pigs, it about their place and their fence. For now, we have policy or regulations of protecting the environment of being dirty. I think these are the difficulties.

**I: from your own, can you explain any difficulties to keeping animals in a fenced area?**

R: yes, because these are ways we’re wasting money on making fence. As I mentioned, since we’re having regulations I find it difficult. Where few people can build a fence, build a sewage, feces pipes. This is the law that every fence has to have a drained outs, waste from pigs. And these are the things that are difficult for people. And their foods, if it wasn’t for the waste foods from the house, it would be hard to look for food.

**I: now, as you can people that raise these animals, can you tell me what community members typically do with animal feces?**

R: well, as was saying. Before there was no regulation, we used to drain out feces at the ocean and lagoon side especially the ocean. But for now, there’s inspection of whose following regulation on draining to sewer system. Majuro’s system, DUD that we live in.

**I: good, thank you for your information. For the last question on food, can you decide what food to get for the family in most households?**

R: who decides? Well, I may explain from experienced. I always give this responsibility to the wife. We always talk about this, it’s a good thing she’s always taking care of it. Taking care of the nutritious foods. Especially when she knows were sick. There are times this responsibility goes to her, because the man goes to work and she stays to take care of the children. It’s a good thing get to interact with each other, for me, I entrust her to have the responsibility of cooking the food. Because she loves the children and she loves me. She’ll cook the foods that are nutritious, and good for us. I think this is a great responsibility, especially when the man is too busy working. As a pastor, it is important for two of them to talk about it for the good of the family. The man should take part too.

**I: perfect, perfect. From your own, can you explain on how families decide which food to get?**

R: I believe, they decides to eat on what is available. And it is one of the challenge that is big for families in the Marshalls. Sadly, the foods that are nutritious have high price in them. But, for a simple tuna with rice, that’s according on what they can buy. And, I am thinking this is how people live. When it’s hard, that is how they can buy. I think this is the reason, they have no choice.

**I: when they need to eat it, they eat it?**

R: yes they need to eat it, they’ll starve.

**I: great, what you think about who decides which foods young children should eat?**

R: I am thinking, its mothers and fathers. It needs to be mothers and fathers. We are the ones that have been blessed for having the children by God. If it is a precious gift from God, we should cherish them. Because children will choose what they want, not for those that are important to them. We know, we want to eat ice-cream. Eat an ice-cream over the broccoli, so we need to choose a decision for the kids. They don’t know better.

**I: good, great. Now, we would like to talk about water and hygiene. Can you please describe a typical day getting and storing water in your community?**

R: in this community, when talking about this community. There are concrete made water catchments and plastic made water catchments. The government’s water is not enough due to the low pressure to eat. Since from the start, we started to invest on building concrete water catchments and buying plastic made water catchments. And it is enough, I’d say, in a year. Just a regular year of rain, as expected. We don’t need. During the times of drought, it’s the time where we bought water. And it is enough, because there are 300 students. Along with the staffs that live in this campus. When the church members need water, they too go with the function of using the water catchments. It is important, water is important to us. We take good of it so they won’t play with waters. Arrangements that I have set them up with the staffs that live on this campus. We don’t let children to play with waters, because it is important.

**I: but, where do you get water for drinking, for cooking, for washing, for bathing?**

R: okay, for us on this campus. Water for drinking we bought them from the water vending machines, because it is safe especially when they use the ultraviolet light for inspection. Bathing, well from the water catchments and for washing dishes. Laundry, those who live in this campus always go out doing laundry at the laundromats. And to use water greatly carefully, we don’t allow them to buy their own washing machine. If they want to rinse, they can. And this is how we use water here. For washing and for bathing.

**I: perfect, thank you for your information. For now, can you think of what are the main difficulties in getting water?**

R: when there is no rain, its weather. There are times there’s rain and there’s not. We sought to see how much we use water and made these water catchments to help with the needs here on campus, so that we don’t need to buy or connect with government water. It’s enough.

**I: good, those information were interesting. Now, can you explain the main difficulties of storing water?**

R: that’s it, when we don’t have money. So that we can buy water catchments, this is the difficulty for some people. As I was saying, it is not enough for people that have three or two families. And few workers, these are the difficulties for many families. We thank God that the church and the school have the resources to buy.

**I: great, good. Now, on what ways that this community tries to make drinking water safe?**

R: we work with EPA (Environment Protection Authority) with their regulations, because these are water catchments. We need to treat them every months, every time, in order to make it safe from germs.

**I: great, now we’ll talk about washing hands. Can you describe hand washing practices in your community?**

R: for this place, there are faucets and sinks. You go and wash your hands, we don’t usually follow the public health regulation, as for the school. It is required to bring hand sanitizers, there are sinks to wash their hands. And are other students that bring their soap, but each families are taught differently on how they wash their hands. The students, it is their studies of how to wash their hands.

**I: perfect, from what you think. What is the difference using only water or water and soap to wash hands?**

R: for hands, what I am thinking is not really cleaning it. We know of soap how it cleans, I am thinking there is a difference. This is why it is required to have soap every time, for our houses. At my house with my family, if not. Well, in this school it required for parents to bring not only hand sanitizers but soap. If they don’t have much to buy it, we try to buy it so there needs to be in the bathroom. There is difference, I believe there is a difference. Because when I clean my hands, with soap or without soap, there is a difference I can see it.

**I: can you think of anything that prevents families from washing hands with soap throughout the day?**

R: they’re lazy, maybe because they never learn from their houses. I know, because I grew up, even though it was not enough for us, they made us to wash our hands with. And then, it’s culture, it can be culture. But, but, because people don’t have. They can’t afford. I think these are the two majority things.

**I: perfect, thank for helping by answering these questions. Could you describe types of toilets in your community?**

R: what types right? Well, there are bathrooms, all of these apartments have their own bathrooms, and kindergarten have their own bathrooms. And elementary and high schools are together using the bathrooms. Separating girls from boys, so there are four for the students. I say four, and there are stalls. Whether urinals or sinks.

**I: and pardon for this question, if it’s too much, but what reasons you guys chose these types of bathrooms?**

R: the reason that we chose, how do we say, culture? It’s the habit of using these things on Majuro. For me, the fact is, there are those before me who took care of these bathrooms and made arrangements here on campus. So, things they made before I follow them. However, I have been in this school before. And I have a small knowledge of sanitation, cleanliness from I saw them doing it and I think it was good for me. Unlike outer Islands where you have to take it here. But I think is proper, it’s good, it’s healthy, it’s clean and simple to use.

**I: now, what types of toilets you’re using, regularly or?**

R: yeah, regularly toilets, like anywhere you go. Things that we buy from the stores.

**I: in some communities, we have heard that defecating in the open (such as on the beach) is common. Could you help us to understand this practice, including how common it is?**

R: yes, yes, there are times that people, because I didn’t fence the back side of this place. When it’s high tide, when they come asking for using the bathroom, you know they need to, I won’t say no. but, I always ask them to go to where there is a high ground from water. But when there is a high tide, they defecate near the classrooms where we can’t see them. It is bad, because we need to clean it. The students, I will fence this place. Especially, the ocean side and go through. But, they do, they do always especially young men. Am saying about the young men, because.

**I: can you tell me why this practice exists in some but not others?**

R: I am thinking, because they’re some places where the land lords prohibits. But other places don’t have any. Secondly, there’s none in our homes. If there is in our homes, as you know according to our culture. If there’s family near it, it would be not good to see each other coming in and out. For young men, it is easy access to use and no people can know you’re using the toilet room. These are things that can make come out of our negative minds, but I am thinking there are some households that don’t have any toilet rooms so that’s why they use these places. They can’t afford it.

**I: what you think from you own, what are the barriers to using the toilets?**

R: there are some people who comes from outer Islands don’t know how to use toilets. And it is hard, but as I was saying, culture and easy access because they don’t have any.

**I: great. But in this community, how young children’s stools are typically dispose of?**

R: the great thing is, I have commonly seen people using baby diapers. And one of the good thing about Majuro is the trash bins. Because back in those days before collection, they mainly throw them ocean side. And we don’t usually come to the ocean side, they do from time to time we see them throwing them at the ocean side. But I am thinking it’s good to throw them inside those small trash bins that were divided among each households. I think its protective way because there is place for littering. After cleaning the child, they throw it inside the trash bins.

**I: good. Thank you for your answers. Could you now explain where young children in your community usually play each day?**

R: they usually play at the hug area, like on this field. Am talking about from here to the lagoon side. RES (Rita Elementary School) that’s where I see many children there frequently. If not, they come inside this campus. Last year, local government donated playground equipment to this school. I let the children play, because I know they don’t have anywhere to play. I’ve seen them many times playing on the beach if tide is low. These are the places I’ve seen them playing.

**I: perfect. Do the children play in areas where animals are kept?**

R: places, places that people have pigs, they don’t usually go there. I have, I have never seen places they got to, but dogs and cats roam in our places. I think there’s no problem children and animals playing. But, yes.

**I: in this community, (can you like) how do we say? (English language).**

R: I am thinking, if we to build a park on ground. And make a playground for children, because I have seen new parks that are building. So, there are families where they bring their children. The children are happy, it’s far and it’s clean. Because there are not enough people visiting there, they’re happy to go there. And the children are happy to be there. Like, Rita’s community would have build 3 or 4 higher grounds at the ocean side, since there are none at the middle. They would have extend, how do we say? Extend it, not a sea wall but maybe fill in rocks to the ocean. And then make the playing ground place. It won’t be a disturbance to the land lords and the people because is at the far end.

**I: great, perfect. Thank you for the picture who have showed. What are the challenges of keeping children’s play area clean?**

R: that’s it, it’s like there are none. For children, it’s hard for them to look for a place. For instance, this campus is limited. There needs to be a time where they need to go home and I care. And that’s the least place they frequently spend their time at is RES. And there are times where it is hard for the staffs, as you know children can throw dirty stuffs. There are times where it is hard to open this place, but if parents present and watch over them. By the time they are done, they can come in and clean up after them. They can clean up but, but there are times they are busy with work and responsibilities. They just let them go and play, they don’t know children need to be learn how to clean. This is why it’s dirty they just let them play by their own.

**I: well, great. Those were excellent explanations, but to wrap up our questions on sanitation, could you explain ways to prevent the spread of disease?**

R: cleanliness, I think it’s a major thing. I see it from the way we run the school. Cleaning is one the thing that sanitize and reduce us from eating with each other.

**I: what do you think of the connection between exposure to feces and illness?**

R: what I think, I always quickly clean it and bury it right away.

**I: good. Now, we’ll talk about the roles and responsibility of each family raising a child. Could you describe the care of children throughout the day in your community?**

R: inside this campus its perfect, like I was saying, in this community they just let them roam free. Not enough adult supervision, leadership. They don’t have time to stay with them. That’s how we say neglect.

**I: great. Who is mainly responsible for child care?**

R: from what I believe is that both parents, mother and the father. Both of them, sometimes we say father’s but mother’s do usually care for the child. It’s a collective responsibility.

**I: now, can you describe to me the responsibilities of mothers in child care?**

R: a responsible mother is the one who working hard and taking care of her child. She always think about her child. I believe God gave every mothers, this is why we always respect every mothers. Because God gave them the knowledge that is bigger than fathers. Relate, especially their emotional connection with them. They usually act to responses when child is in danger. For me, God gave each person the ability to take care children and their needs.

**I: and with father, what are the responsibilities of fathers in child care?**

R: fathers, I believe they are the ones that can lead that’s a first. He’s the head of a household as I understand it from bible. And he’s the one who protects them, and he’s the one who brings. It’s not likely that mothers can bring, it is the great responsibility that many fathers bring the needs of a family.

**I: now from what you’ve walked, and seen caregivers taking care of children. How do the caregivers play with children under 2?**

R: there are times I have seen them not taking care of them, they don’t response on what the child is doing. Anything the child does, there are many times we’ve seen. As we grew up with it, mothers and fathers always led the older siblings take care of the child. There are times where they make things to the children sad, you know children do angry and they don’t usually know what they are doing. And I think this way is inappropriate, we’ll say take care of the child but might the whole half day.

**I: could you talk about the role of grandparents have in raising children in this community?**

R: from what I believe, with a child it’s for mother and father responsibility. They might still ask to take care of the child, but I don’t believe its grandparent’s responsibilities to take care a child. If they to look after them because there’s responsibility where mothers and fathers from time to time, they will do it. But it does not need to a way where grandparents take care of the child because a young adult is having a child. Grandparents are good, they have a place with us. But, I disagree that they are the ones who are responsible taking care of the children. So, their responsibilities are done, those that have children they their own responsibility now.

**I: but what ways grandparents support in raising children, support mothers and families?**

R: am thinking every responsibility is within every mothers and fathers care. Because, it is their responsibility from God. I think grandparents have limited roles, there are times where we tend to make jokes. Grandparents do spoil their grandchildren, but they are not the ones to take care of everything. As you can see how grandparents treat their grandchildren, is completely different from their own children. It is different, I believe it’s a structure or design from God. For me, I have was raise by my grandparents. They wanted to take care of me, and there were others in the family too. They can play the role, where the parents missed absent. I am thinking they can take over, but it’s not the way it should be.

**I: but what makes good grandparents?**

R: what makes? Well because they’re grandparents. The word grandparents, they’re good. Like for instance, when the person have unstable mind maybe that’s why the person is bad. But, what I have seen the word grandparents are good. Everyone, even though they were bad back then but they were good with their grandchildren.

**I: great, but could you talk about the roles others have in raising children in this community?**

R: I think the responsibilities are, the roles of parents are immediate. The roles of others are secondary, because if something happens to a child. Maybe if they didn’t notice the child going to the road, when we see a child going to the road, and we respond quickly. But if they see the child being naughty, it is their responsibility to say what the child did. They may look after them from things that are bad. It’s not their responsible to discipline but it’s the parents. They may contribute to what they bring them to their parents.

**I: Thank you, but as you have been walking. In what ways that siblings (older siblings) help raise young children?**

R: as I was saying, it depends on what older siblings’ age to take care of the younger child. And I think there are times where we see many families let the young siblings taking care of the younger child, which they should have not. But we grew up with our culture, our younger siblings we always protect them. But as I was saying, it’s not their role in taking care of them. Staying with them for hours and hours taking care of the children.

**I: great job answering, this is the last question. Last question. Now for the last section, we would like to learn about ways we can develop health programs in this community. Is it okay? Could you explain where members of your community usually get trusted information about nutrition and health?**

R: the fact is, we have not much knowledge to know. I believe a parent needs to know to teach his/her family. There’s no problem for Public Health implementing informational programs. Because there are some people do not care about, we don’t care. And it is a huge thing, and I don’t know of if Public Health can help now because they even care too. But this is one the important thing I see with Public Health doing outreach of teaching people with anything they don’t know of. There are other people who wouldn’t find a way for them to understand. And there are times that Public Health do take part of giving out information fortunately.

**I: now, can you explain the reasons why these sources are trusted?**

R: Public Health, they’re the experts I believe. Not everyone in the Marshall Islands knows what is a best information regarding health. And this is where Public Health is good for help. But as I was saying, it is parents’ responsibility to know. There those who didn’t finish education, maybe they don’t have a high school diploma. It would be hard for them to understand, this is good part where Public Health comes and help others what they want to know.

**I: great, Thank you. And where nutrition and health messages should be delivered so this community member would see/hear them most easily every day?**

R: schools, because the students and children they be, and I know there’s science and biology all of these things in the school. But, one of the places that might be reachable is the school. There are times Public Health would like us to deliver these information inside the church. But I think it is affective such as been doing in school. Because it is where, you knowledge everyone wants to achieve it and there are families who are putting their children through school.

**I: thank you that was a good explanation. And what types of media that community members’ use?**

R: cellphone, cellphone and those gadgets especially I think social media. And now there a few adults don’t know how to use, but I think their children knew how to use them and they share with them. I think this is the best way of probable the best way of communicating. All of that social media, and for people of the Marshall Islands in these years. Facebook, as we know we’re talking about, Instagram those things. Radio maybe the second, and especially for adults. Social for newer generation, radios for adults, and if we want to reach the outer Islands. And the other one is, TV well there so many who use it. And if there is a way we can go through it NTA. We can say NTA can set aside a channel for this purpose.

**I: great.**

R: things Public Health is making.

**I: well, is there anything else about the topics we talked about today that we missed or that you would like to tell us about?**

R: well, I don’t have any from what I am thinking. You do have many questions, those are some questions where person is thinking of. They are much more related and revealing, about our health, our health, I think is perfect.

**I: great, it’s perfect, well we’re done.**
