# Supplementary material for: Conceptualizing multi-level determinants of infant and young child nutrition in the Republic of Marshall Islands–a socio-ecological perspective
Source: PLOS Glob Public Health. 2022 Dec 19;2(12):e0001343. doi: 10.1371/journal.pgph.0001343 (PMC10022247; doi:10.1371/journal.pgph.0001343)
Supplement: S1 Data — (ZIP) [file pgph.0001343.s001.zip › RMI Supp Data/Interviews data/I18U_IDI_FCG_RITA_AUG 14_LIBON.docx]

**Interview Code: I18U**

**Interview type and interviewee: IDI FCG**

**Interview Date: Aug 14**

**Location: Rita**

**Interviewer: Libon**

**Transcriber: Shante**

**I: Before we start. Do you agree on doing this survey with me?**

R: Yes.

**I: Ok. Thank you for giving this precious time for me to talk with you. The information you will give, we will learn from them and it will also help make the women and children life healthier. And to start, can you tell me about your family?**

R: like what?

**I: anything about your family.**

R: like what? What’s wrong with my family?

**I: Who lives in your house and how many kids you have, how many boys and how many girls and…**

R: Me and my 2 sisters and my brother and my kids and my sister’s kids. That’s it just us.

**I: How many kids are there?**

R: I have 6 kids but 3 are with me. My 2 sisters have 3 kids each.

**I: Now how old are they?**

R: 10 years something… ends around 19 years.

**I: and?**

R: There’s no 20 years above.

**I: What about the other kids?**

R: There’s 12 years, 10 and 6. And the baby hasn’t turn 1 yet.

**I: How many boys and girls?**

R: 5 girls and 5 boys.

**I: Can you tell me about your community? Anything about your community**.

R: like what…?

**I: this town…**

R: Plenty drunk people. Plenty people with no jobs…

**I: That’s good. Is there any other? About the people or your chief leaders…?**

R: They said people here should quit drinking… I think that’s it.

**I: What are the good things about this community?**

R: the good in this community…? Hmm… What can that be…? Oh. Our chief leader is good to us but the people here in our town don’t listen to him, some listen to him and some don’t. but the chief is nice and kind to us here.

**I: What are the bad in this community?**

R: lots of drunk people. They don’t work… the males here don’t work expect the women’s.

**I: Let’s now talk about health and illness in your family. Can you tell me about some of he illnesses that your children have suffered from?**

R: Sometimes they get stomach ache, sometimes they get sick… I think that’s it.

**I: You mentioned sick… what kind of sickness?**

R: ohh. There’s diabetes in this family…

**I: What about your kids?**

R: oh well they aren’t.

**I: You said they usually get sick, can you tell me what kind of sickness do they usually get?**

R: Cough, runny nose, diarrhea, headache and stomach ache… all these kinds.

**I: What makes them sick?**

R: When we have flu here, they will get it. And when they don’t get enough sleep they will also get sick

**I: Is there any other reasons that…**

R: Now when they sleep and wakes up in the afternoon to eat they can also…

**I: You mentioned cough diarrhea and headache… Can you tell me what causes them?**

R: sometimes they cough because its too much cold and sometimes they get it from others.

**I: What are the affects of these illnesses?**

R: they can get asthma, fever, diarrhea and stomach ache… those are the cause. And headache.

**I: Ok. Now how do you prevent these illnesses?**

R: I take their sleeping stuffs outside and put them under the sun and clean where they sleep…

**I: What about their food?**

R: I clean the dishes they eat with and wash their hands.

**I: How would you know when your child needs to go see the doctor?**

R: When I see that my daughter is coughing I will take her to the hospital and when is has diarrhea too. I would take her to the hospital so that they can give me her medicines for her to take.

**I: Do you take her to the hospital when she’s really sick or when she just started to get sick?**

R: when I see that she’s going to be sick I take her straight to the hospital. When my other kids get runny nose I take them straight to the hospital also.

**I: Who is the first person you bring your child too when he is sick and why?**

R: I used to bring them to the children nurse first… I forgot her name. When they get in the hospital that’s the nurse they go to first.

**I: Why do you bring them to her?**

R: Because she said when they get sick I should bring them to her**.**

**I: do you use traditional medicines?**

R: Yes. I used traditional medicines. Both traditional and hospital medicines.

**I: What kind traditional medicines do you use and why do you use them?**

R: Because they say they can cure them. When they cough we have Marshallese medicines to cure it.

**I: can you tell me what kind of affects your child gets from eating nutritious foods?**

R: the illness I already told you…

**I: We are talking about nutrition, what kinds of affect will your child gets from eating nutritious foods?**

R: Oh. Nothing.

**I: What kind of illness will the child gets from not eating food that have no nutrition in them?**

R: If they don’t eat the kind of foods that… its like they will starve more… things will grow on their body like rash and stuff… they will feel weak.

**I:** **We talked a lot about being unhealthy. Could you now describe for me a typical day of someone living a healthy lifestyle, from the time they wake up in the morning until when they go to bed?**

R: they look chubby and healthy, they feel healthy and they don’t feel sick.

**I: ok. What are the sign of a healthy 2 year old that you look at them and see that they look healthy?**

R: when the child is healthy?

**I: Yes.**

R: When he eats good food… they eat on the right time and they are chubby. And we feed them on the right time.

**I: What are the signs of a healthy adult?**

R: they eat healthy food. They are big, they feel healthy. They eat on the right.

**I: ok. So if it was you that was feeling healthy, could you tell me what would the signs be of you knowing that you are feeling healthy?**

R: I would say not lazy…

**I: is there anymore?**

R: I don’t think so.

**I: Let’s now discuss hand washing. Could you describe in detail your family’s hand washing throughout the day?**

R: we soap it and after we wash our hands with water and then dry it.

**I: Do the kids wash their hands in a day?**

R: yes. they do.

**I: How do you wash their hands or how do they wash their hands?**

R: for me I would go soap my youngest daughter’s hands and the wash it water and dry her hands after.

**I: What about kids under 2 years?**

R: They also do it. Use soap wash it with water and dry their hands.

**I: When is the time you guys wash your hands in a day?**

R: morning noon and night. When we eat in the morning we wash our hands, same for afternoon and evenings. We wash our hands with soap and wash it with water and then dry our hands after. An after they play they will wash their hands too.

**I: Other then washing their hands before they eat. Is there other important times they should wash their hands with soap?**

R: Yes. They do.

**I: After what? Can you give me in detail exactly when they wash their hands?**

R: after they play they wash their hands, before they eat they wash their hands, after they use the bathroom they wash their hands. When they make their food, they wash their hands.

**I: What is the difference on washing your hands with water only and the difference on washing your hands with soap and water? Can you tell me the difference?**

R: I don’t there is right?

**I: Now that you said there’s no difference. Can you explain why there’s no difference.? Cause there’s water only and water with soap only.**

R: there’s difference because the water is by itself and the soap its by itself.

**I: when you use the water and soap together is it cleaner? Are your hands clean?**

R: yes.

**I: on how you use water to clean your hands. Is it clean?**

R: No. I need to add the soap with the water.

**I: What are the good things about washing your hands? When are times that you don’t use soap?**

R: When I am in a hurry and sometimes we just don’t soap are hands because we forgot.

**I: Now I would like you to think back to when you were pregnant. Can you describe your diet when you were pregnant compared to when you were not pregnant?**

R: When I was pregnant. If I said I wanted to eat that food I would eat it right away. I won’t eat anything else other then the food I want to eat.

**I: What about on the time you weren’t pregnant?**

**R: I eat everything.**

**I: What made you want to eat the food you liked during the time you were pregnant?**

R: I really liked eating plum. The sweet plum. That’s the food I liked the most.

**I: Ok. Now what made you want to eat the plum?**

R: it was my pregnancy cravings. That was the main thing I usually eat. oh, and orange. Those are the things that I wanted to eat during my pregnancy.

**I: What kind of food did they want you to eat during your pregnancy and why?**

R: they gave me fish and vegetables, those are the things they wanted me to eat. Orange and apples. They told me not to eat food that are greasy and… those are the food they gave me to eat.

**I: Why did they want you to eat those food?**

R: Because it will make the baby in my womb healthy and grow.

**I: Ok. What kind of food they told you not to eat during your pregnancy**?

R: Those foods, [laughs] like candies and drink soda’s and all those kinds that aren’t good to eat.

**I: You mentioned candies. What kind of candies?**

R: Candies like chocolate and cookies, all those junk foods… I only had orange and apple.

**I: Now, why didn’t they want you to eat those kinds of foods?**

R: huh?

**I: why didn’t they want you to eat those foods?**

R: Because its bad for the baby and it will harm the baby.

**I: Who want and didn’t want you to eat those food during your pregnancy?**

R: Myself.

**I: is there anyone else who told you not to eat those food?**

R: No one else. Just me.

**I: Is there anyone that told you, oh. Don’t eat that eat this one?**

**R: Just myself.**

**I: just you?**

R: hmm.

**I: is there any teachers or anyone that told you not to eat those foods?**

R: No one just me.

**I: What about the workers at the health center? Or the doctors? Did they give you word of advice about the food not to eat?**

R: Yes. They did.

**I: Oh ok. Who took care of you when you were pregnant?**

R: hmm?

**I: Who took care of you and help you during your pregnancy?**

R: my sisters and aunties.

**I: Ok. How did they look after you and helped you?**

R: They took care of me and when I was in the hospital they brought my stuff and the baby’s stuffs. They brought food, drinks, cloths and towel… all the things we need inside the hospital.

**I: Can you tell me what kind of medicines and nutrition you took when you were pregnant?**

R: they gave me vitamin and medicine for blood.

**I: did you took all those medicines?**

R: yes. I took all of them.

**I: Why did you need to take the medicines?**

R: They said its for the baby and its also good for me.

**I: are there times that you didn’t take the medicines?**

R: Nope.

**I: did you drink alcohol or took some strong medicines during the time of your pregnancy?**

R: I haven’t.

**I: Is there any traditional medicines you took during your pregnancy?**

R: when I was pregnant with my daughter, I was coughing really bad. I drank Marshallese medicines and I stopped coughing.

**I: Ok. If you were advised to eat more fruits and vegetables during pregnancy, could you describe what would make this difficult?**

R: I don’t think there is… my husband family side and sometimes mine…I usually eat what I want. The things that I want to eat is the food that I’m going to eat.

**I: Ok. Now what will make it easy for you to eat vegetables and nutrition foods?**

R: I went for my appointment and the doctors told me to eat vegetables and nutritious food. Sometimes I don’t like to eat them, but I eat them anyways. I usually eat orange everyday.

**I: What are the important of it for you to eat them?**

R: For my healthy life and the baby’s.

**I: Can you tell me what kind of food you ate when you were breastfeeding?**

R: fish and vegetables and sometimes chicken but with less salt.

**I: Ok. Is there any other food you eat that you know its good for you when breastfeeding?**

R: I usually eat breadfruit.

**I: Can you tell me what kind of food they told you to eat during the time you were breastfeeding?**

R: The one thing they tell me to eat is fish. And I usually eat fish. When I was in the hospital they told me to eat the food from there too.

**I: Is there anything other than fish?**

R: all those vegetables.

**I: Ok. What are the foods they told you not to eat during the time you were breastfeeding? And why didn’t they want you to eat those foods?**

R: what?

**I: I said, what kind of food they didn’t want you to eat during the time you were breastfeeding?**

R: they told me not to eat candies and drink sodas.

**I: Ok. Who told you not to eat those food during the time you were breastfeeding?**

R: The doctors told me not too. Also, not to drink soda because its bad for me and my baby’s health.

**I: Who was the one to give you advice on what not to eat and to eat during the time you were breastfeeding?**

R: the doctors.

**I: Anyone else other then the doctors that gave you advice?**

R: My parents and the ones older then me.

**I: After you gave birth, could you describe how you first breastfeed your baby throughout the day?**

R: I was sitting and breastfeeding him. I usually don’t like laying down and breastfeed my babies. I don’t lay down and breastfeed my babies.

**I: Can you describe why you don’t like laying down while breastfeeding?**

R: Because if I do the baby will always want to laydown and makes me breastfeed him. because if he is used to laying down and breastfeed he will be hard to control because if I wont laydown and breastfeed him he will cry until I laydown and breastfeed him… that’s why I never breastfeed my children.

**I: After you gave birth, how long did it take you to breastfeed and why?**

R: When I went to the hospital I went straight to the labor room and gave birth, right after I gave birth they put me in a wheel chair to my room and told me to wait because they will bring my baby out once they are done cleaning him. once he arrived the first thing I did was breastfeed him. I said down and breastfeed him because they told me to breastfeed him.

**I: Now, did you give your baby bottles or any other liquids on the first few days he was born?**

R: When I saw that my breast hasn’t gain any milk yet then that was the time I first gave him baby bottles to feed him. I gave him milk in a bottle and he was full, and he slept right away. I only gave him that because my breast wasn’t producing milk faster. I gave him Emfamil. (Powder milk in a can).

**I: How long did you gave him bottle until he was breastfeeding again?**

R: my breast took a week to produce… he’s my only child that took him awhile to use the bottle.

**I: Did the doctors tell you the reason why it took your breast too long to produce milk?**

R: hmm, it was different for each my children. One of them it took 5 days for my breast to produce milk, my other child I think it took 6 days. But my youngest child her took longer for my breast to produce milk that’s why I had to give him milk in a bottle.

**I: Did you have any difficulties on breastfeeding your child to the day he was born until the day he was 6 months?**

R: no.

**I: hmm? Did you have any difficulties on breastfeeding your child from the day he was born to the day he was 6 months? Did you have any difficulties?**

R: With me…? Oh. No, I wasn’t breastfeeding him.

**I: ohh…**

R: He only used milk in the bottle because it took long for my breast to produce milk, so I gave him Enfamil**.**

**I: Did you have any difficulties on breastfeeding your child from when he was born to when he turned 1?**

R: Yes, because when I gave him my breast he didn’t want it and he was crying because there was no milk in them yet so when I gave him milk in the bottle he stopped crying and went to sleep. And I’ve been giving him bottle ever since.

**I: Could you tell me when you first gave foods and/or liquids other than breastmilk to your child?**

R: I first gave him food when he was 6 months.

**I: Ok. What kind of drinks did you first gave him?**

R: Water and milk.

**I: Could you explain why you started to give him food and water?**

R: Because it was time for him to start eating.

**I: Ok. Are there any other reasons why you started giving him food?**

R: Milk wasn’t enough**.**

**I: What are the thoughts of the people on giving food and drinks to the children in that age? From your point of view, what do you think about people giving food and water in that age?**

R: Because sometimes the baby usually cries a lot and we think maybe they are hungry so and the milk wasn’t enough for them and that’s why we started giving them food and drinks. So we feed them so they can be full and to make the crying stop.

**I: Oh ok. Now, what was the first food you gave to your child and how did you prepare it?**

R: I first gave him baby food and I bought them from the stores. How I fed him was, wash my hands and then feed him.

**I: hmm… Now you mentioned you bought your child baby food. Do you usually make his own baby food?**

R: Yes. Sometimes I make him food from the Pandanus and breadfruits and Biscuits.

**I: Now how do you prepare them? For instant the pandanus. How do you make it?**

R: first I wash them then boil them and after boiling them I take the juice out of it and then feed it to my child after.

**I: What about the breadfruit? How do you make it?**

R: boil it and make soap out of it.

**I: What do you add in the soap?**

R: Chicken and Fish and vegetables.

**I: You mentioned biscuits. How do you feed him that?**

R: I dip the biscuits into the water to make it soften so that it would be easy for the child to eat it.

**I: You dip It into what?**

R: Water and milk.

**I**: **We are trying to understand how people eat in this community. Could you describe in detail what your family usually eats and drinks throughout the day?**

R: eh?

**I: We are trying to understand what the people in this community eats. Can you give me in detail what the community usually eats throughout the day?**

R: They usually eats chicken, fish, big, ham and rice… they eat different kinds of food everyday.

**I: Can you tell me how they prepare their foods?**

R: before they prepare their food, they wash their hands first and start making their family’s food, so they can eat.

**I: Now, who in your family needs to have its food first and who comes last?**

R: the eldest eats first. Like my grandparents and parents. They are the one who gets to eat first.

**I: Is there any difference on how you share your foods with each family members?**

R: I don’t think so. If my family was eating chicken, then everyone will be eating chicken. But to the eldest their food is separate.

**I: now you said the Elders food are separated. Can you tell me why? What do they eat other then not eating chicken with you guys?**

R: they will eat fish and vegetables.

**I: Now is there any difference in the amount of food you give to each family members?**

R: everyone has the same.

**I: is there any children that his foods are bigger then the rest?**

R: No one. This house one person fills up their own food so that they can know the amount of food they want.

I: **Now could you describe any food sharing between family members during mealtimes (for example children eating together separately from the family, meals eaten from the same plate by all family members)?**

R: Everyone each get a plate. All the kids will have their own plate to eat.

**I: What about the adults?**

R: Me and my husband share the plates because we are couples, but each child used their own plate to eat from.

**I: Now, do your family share food to your neighbors?**

R: Of course, we share our food to our close neighbors. We are one in this community. if their house is closer to ours then we will give them a plate of food.

**I: We have heard from some families that eat local foods whereas others eat processed foods. Could you explain what is typical for your family?**

R: We eat anything, the only reason sometimes we have Marshallese food is when they send it from the outer islands. When we received the foods are already cooked so we eat them and share them with our neighbors.

**I: What are the difficulties for you to cook Marshallese foods?**

R: well I don’t have any planted Marshallese food so if I have money I would go to the store and buy breadfruit.

**I: What are the goods and bad on eating Marshallese foods?**

R: sometimes its bad because sometimes we want to eat them, but we don’t have planted plants for them, so we will only eat them when we have money to buy them. In our home in the outer island we wouldn’t worry about getting them because we are surrounded by them.

**I: What are the good things about Marshallese foods?**

R: Marshallese foods are so good. They are good for our health and body.

**I: What are the goods in imported foods?**

R: I don’t think I know the…. [giggles] I don’t know… to make are tummy full.

**I: How about on how you go buy them in the stores? Is the imported food expensive or not?**

R: the imported food is not expensive.

**I: What are the bad things about them?**

R: well they are greasy, salty and not good for our health.

**I: Now that we’ve talked about how the family eats, I would like to learn more about how your child eats. Could you describe in detail what your son/daughter under 2 years commonly eats throughout the day? Now we will talk about your daughter first. Can you tell me what she usually eats in a day?**

R: She usually eats sausage and rice.

**I: How about when she eats snacks? What snacks does she usually eats?**

R: I give her banana, orange and apples.

**I: How many times do your child eats in a day including her snacks?**

R: she eats morning, afternoon and evening.

**I: How do you know when she will have enough food?**

R: enough what?

**I: her food. Like how would you know if she is full?**

R: I would know when she’ll full because she will stand up from where she was eating and go play.

**I: What would you do if the child doesn’t eat?**

R: I would give him biscuits or apples and oranges. He usually eats that when he doesn’t feel like eating other foods?

**I: What about when he doesn’t want to eat those foods? What do you do?**

R: I breastfeed him.

**I: If the child doesn’t want to eat, like he really doesn’t want to eat. what do you do?**

R: I would stand up and carry him around and give him water to drink.

**I: How about when he is sick? Do you feed him differently?**

R: this child when he’s sick he doesn’t hate food.

**I: You’ve told me what your child under 2 usually eats. Now could you explain to me the process, from start to finish, how you prepare and cook a meal for your child? [baby crying in the background]**

R: I wash my hands, make his food and feed him so he could be full. {Johnny, take him].

**I: Can you tell me what type of food do you give your baby that is good for him and that it will make him to grow and be healthy?**

R: I would say fish and rice.

**I: Is there any other food that you feed him that you know that it will help him grow and that it is good for the child?**

R: breadfruit, banana, coconut juice.

**I: Now, what kind of food you shouldn’t feed your child under the age of 2 and why?**

R: I don’t give him candies and those other kind junks because if I will feed him real food he won’t eat them.

**I: is there any other food other then candies?**

R: chips, chocolate and lollipops.

**I: Can you tell me the responsibilities on taking care of a child?**

R: take care of them when they are sick. Make sure they are safe.

**I: Is there any more other then you making sure they are safe and…**

R: look after them to make sure they won’t get sick. Feed them on the right time so that they won’t get sick and weak. Make sure they feel good.

**I: Can you tell me the difference on how you feed your daughter and how you feed your son under the age of 2? What is the difference on how you feed them? Your daughter and your son.**

R: What is the difference…? Well if there was this meat, they will both eat it. Not separately but eat it together. They eat the same food.

**I: *We are also interested in the roles and responsibilities different family members play in raising*** ***children.*** **Could you describe the care of children throughout the day in your community?**

R: we wash their hands, clean them, make sure they are clean and feed them. [baby crying]

**I: is there any other reasons?**

R: take care of them and make sure they won’t get hurt.

**I: who’s in charge of taking care of the children?**

R: the women.

**I: Why should it be just the mother who takes care of the children?**

R: the father’s too.

**I: What are the responsibilities of the mother to their children?**

R: look after her child from getting hurt, take care of them so that they won’t get sick. Make sure they aren’t hungry and weak.

**I: while the children are growing, what should the mothers do to them while they are growing up?**

R: talk to them and teach them about manners so that they would know what do to when they grow up.

**I: yes. That’s right. What are the father responsibilities on taking care of his children?**

R: they bring them food so that they can eat and not be starved and also take care of them.

**I: How do the caregivers play with the children under the age of 2? What kind of games do the caregivers play with the children?**

R: They read books to them. Look after them and let them watch movies.

**I: Could you talk about the role of grandparents have in raising children in this community?**

R: Sometimes they help us with the needs of my children, sometimes they bring them food and sometimes they watch over them. And teach them.

**I: Teach them what?**

R: Teach them manners so that they would know how to respect others.

**I: Ok. That’s good. How do the grandparents help their grandchildren and their parents?**

R: they tell us to talk to our children so that they will listen to us and let them know that they should respect their elders and other people.

**I: Now, what makes the grandparents good?**

R: they are good because they give their all to their grandchildren and they love them very much.

**I: Ok. Now could you tell me about the role that other family members have in raising children in this community?**

**R: They take care of their children, talk to them, make sure they are clean.**

**I: how do the older siblings look after their brother and sisters?**

R: they make sure their siblings don’t go to bad dirty places**.**

**I: your youngest child… how would her older siblings take care of her?**

R: they would carry her around and check if she pooped or play with her.

**I: Ok. You’ve done a very great job on answering. We are almost done. Now for the last section, we would like to learn about ways we can develop heath programs in your community. Could you explain where you usually get trusted information about nutrition and health?**

R: the hospital, the church and everywhere else.

**I: Now beside hospital and the church… is there anywhere else you could get the information from?**

**R: I would say from my grandparents and my parents. The people around me and also from the schools.**

**I: hmm. Ok. Why do you trust the people you took your information from?**

R: because I trust their information.

**I: Could you tell me where would you rather want the information to go and will be easy for you to see and listen to them**?

R: I would say the station V7ab.

**I: Can you tell me why?**

R: because that’s where the news gets out and that’s where I usually listen to news.

**I: What kind of device or social media do you use to listen to the information other then the radio station.**

R: Online and face book.

**I:** **When you think about your own parenting behaviours, can you explain what influences how you raise your children? Is there any difference on how you raise your children and on how others raise their children?**

R: I myself take care of my baby, only when I am doing chores then I give him to my family members to watch over him while I do the chores.

**I: when you give your child to someone else. Do they take care of him like how you do?**

R: when I give my child to someone else to take care of him he won’t do a nice job on it because its not his child and she won’t take her time with him.

I: can you describe that?

R: Its like if she wants to put my child on the ground on go do something else but I told her to watch over my child, its like she doesn’t really care because its not her child and it won’t be her responsibility if my baby get hurts.

**I: How do your community influence how you raise your child? What do they think about you raising your child? Is there any word of advice you learned about caregiving from your community?**

R: they told me to take care of my child so that they won’t get sick. Make sure they won’t get hungry and make sure they are always clean.

**I: Who did you take this information from? And where?**

R: from hospital.

**I: Is there any information you would want to know about parenting, but you never knew about?**

R: yes.

**I: like what?**

R: oh. never mind, I don’t think there is.

**I: Is there anything else about the topics we talked about today that we missed or that you would like to tell us about?**

*R: No.*

***I: Ok that’s good. We are done now. Thank you for your time. All your information will help us with this program. Thank you so much.***

*R: Thank you also.*
