# Supplementary material for: Conceptualizing multi-level determinants of infant and young child nutrition in the Republic of Marshall Islands–a socio-ecological perspective
Source: PLOS Glob Public Health. 2022 Dec 19;2(12):e0001343. doi: 10.1371/journal.pgph.0001343 (PMC10022247; doi:10.1371/journal.pgph.0001343)
Supplement: S1 Data — (ZIP) [file pgph.0001343.s001.zip › RMI Supp Data/Interviews data/I19U_IDI_HW_Rita_Aug 14_Libon.docx]

Interview Code: I19U

Interview Guide and Interviewee: In-depth Interview

Interview Date: August 14 2018

Location: Majuro Hospital

Interviewer: Libon- Traditional Healer

Transcriber: Fela

**I: Before we proceed, do you agree in taking your part on this survey?**

R: yes

**I: Ok. Thank you for giving your time to speak with us today. The information we learn here will help us find ways to improve maternal and child health and sanitation in your country.**

**I: To begin with, can you tell me your job title? Where do you work?**

R: I don’t work.

**I: and what about in this community? Is there is anything that you do in this community?**

R: yes. I cook, do laundry, I clean around the house and I am also a traditional healer.

**I: Now, as a traditional healer, what you usually do throughout the day?**

R: silent (long pause)

**I: for example, when someone come and seek for help in need of traditional heal, what do you?**

R: silent

**I: to make it easier so that you can understand it more, if someone bring a child for you so in term of healing, what would you do?**

R: I can massage their body, and some children brought with damage body and I also bathe them with Marshallese medicine.

**I: anything else?**

R: (uno in kijon) “a special medicine for child in term of prevent the child from crying all the time or also can help them move or walk before they turn one”.

**I: before you give these traditional medicine, what do you do first? Do you think first what would be the suitable medicine you’re giving them or you just give them any kind of medicine? And how would you know that the traditional medicine is the suitable one for them?**

R: there are just some traditional medicine that suit children best because whenever I use these medicine, it does really heal the child. I know the medicine and I positively know that these medicine can heal children.

**I: ok. Let’s now talk about health and illness in this community. Can you tell me any illness that children under two years old have suffered from?**

R: some children get illness from their mothers. (Meaning- when the mom is pregnant carrying another baby while the child is under one year olds, that’s also one cause of illness causes to children). Sometimes the mothers don’t really take care of the child’s health. Or spending their times with their children. They don’t really take care of their meals.

**I: and what kind of sickness or illness that happen to the child when the mother don’t really take care of them?**

R: these illness can be caused when children don’t get their meals in their exact time of having them.

**I: and what kind of sickness that caused by that?**

R: illness like diarrhea, or can be amoeba

**I: Anything else except diarrhea or amoeba? When you look around, what kind of illness you usually see in this community? Like the common illness that you usually hear or see that children get almost every day in this community?**

R: when mothers don’t take care of them or watched over them but they let them play in sanitation area. They play in dirt and come straight and eat their meals without washing their hands.

**I: ok**

R: mothers are responsible in washing their children’s hand when before they eat their foods.

**I: what are the seriousness of these illness?**

R: like,

**I: is there is any seriousness for diarrhea or amoeba?**

R: yes when the child is having diarrhea and the mother is not giving enough water for the child, the body would get dehydrated.

**I: and what about amoeba, what is the cause of amoeba or diarrhea?**

**I: why do children often get illness like diarrhea or amoeba? What are the causes?**

R: caused by sanitation, or dirt, or also can be when mothers don’t take care of cleaning the foods that are given for the child.

**I: what are some ways to prevent these illness?**

R: sometimes I massage their stomach because amoeba can be one caused when the child is having stomach bumps. When I do massaging, it helps reduce amoeba sickness.

**I: ok. Now who do you thing the child would bring to when he/she is sick? Or why would parents bring their sick children to the hospital?**

R: children are supposed to be brought to the hospital when they have asthma illness, coughing, fever, or toothache.

**I: and from your opinion, why would they bring to the hospital or the traditional healer?**

R: because some people don’t trust these traditional medicine. They only trust these supplements from the hospital.

**I: so you meant that they usually used traditional medicine but they seek for health in the health centre at the hospital?**

R: yes:

**I: what are some difficulties for a child when seeking for health at the hospital?**

R: in this term, what makes it difficult for children to get health or medicine from the hospital is financial issue. Parents don’t have enough money to bring their children to the hospital.

**I: anything else. On what issues you are talking about the financial issue?**

R: there is no money for taxi fair, they don’t have money to pay for the pill, and also they don’t have the money to pay for the chart in order for them to get the medicine.

**I: ok that’s great and these are the right information that I am looking for. Now can you described any illness affecting your children that are associated with nutrition?**

R: there is none

**I: can you describe more on reasons why you mentioned there is none?**

R: the reason why I am saying that there is no illness affect the child associated with nutrition is because these are nutritious foods.

**I: can you describe any foods that affect the child’s growth? And reasons why?**

R: unhealthy foods like ramen, rice, soda, ice candy, chips and any kind of foods that are unhealthy for children.

**I: and what kind of foods that give children good health?**

R: pumpkin, papaya, coconut meat, crab, fish …

**I: you are arranging only local food. What about the imported foods? Imported food that give healthy food for children in this community? Healthy food from other countries**

R: like grape, apple, orange, mango, water melon.

**I: why would you say that these are foods are health for children’s body?**

R: because they are nutritious foods. They are contain with vitamins.

**I: we talked a lot about being unhealthy. Could you now describe for me a typical day of someone living a healthy lifestyle, from the time they wake up in the morning until when they go to bed?**

R: silent (long pause)

**I: let me read the question one more time. Could you now describe for me a typical day of someone living a healthy lifestyle, from the time they wake up in the morning until when they go to bed? Can you describe the lifestyle of a healthy person throughout the day?**

R: the healthy person would wake up with a good health, they wakes up and do some works and that’s make the body feel healthy.

**I: what are the appearances or signs of the healthy person?**

R: when I look at the person, I know as I can see that the person is feeling healthy due to do some movement around the house. The person doesn’t look skinny, when they move around, they are sweating, and they have smiling faces all the.

**I: and what about the appearance or signs of a healthy child under two years old?**

R: silent (no respond)

**I: what do you see in a healthy child under two years old? How would you know that the child is healthy?**

R: when I see that the child is moving. The child always play, and the child doesn’t look skinny.

**I: can you also tell me the appearances or signs of a healthy adult?**

R: the person is not skinny, they do house chores in order to move their body, and always have smiling faces.

**I: I have one more set of illness questions but related to women’s health now. Could you tell me about your experience with women who have anaemia?**

R: they always sleep without moving around.

**I: ok**

R: anaemia also can be caused when people always sleep without doing movement so that blood can circulate inside their body and the foods they eat.

**I: can you tell me if these women who have anaemia think it is a serious concern?**

R: from my own opinion, yes there is. There are some women who are struggling with anaemia and they have weak and dying body.

**I: anything else that concern for them?**

R: some might die.

**I: ok**

R: some would survive when we bring them foods that can help provide blood for them like sashimi.

**I: what are the causes of anaemia in women of reproductive age or pregnancy?**

R: they usually eat cool-aid with salt or spicy foods like the takis chips. As you know, foods that affect reproductive age like salty foods.

**I: ok. And what are the causes of anaemia in pregnancy lady?**

R: what can I say about that question? Some pregnancy are having anaemia because they don’t eat foods that can be good in providing blood in their body. Can be sashimi or drink coconut drink, or jokaro (the juice taken from the coconut catch in a water bottle)

**I: do you have advice given to women for prevention and treatment of anaemia?**

R: yes. Sometimes when I see those eating foods that can caused anaemia, I would tell them not to eat salt or cool-aid. Look for foods that would help provide blood in your body and not foods that are caused anaemia illness.

**I: any advice for pregnancy women that have anaemia?**

R: yes

I: can you describe more on reasons why you say yes.

R: sometimes I tell them to eat a lot, drink lot of water.

**I: you say eat a lot. What should they eat?**

R: they should eat fish, pandanus or bwiro (also made out of pandanus) or they can eat any kind of foods that appropriate for their body and can provide blood in their body.

**I: ok. Now I would like to talk about breastfeeding practices in this community.**

**I: could you explain about how long after birth most women start breastfeeding in your community?**

R: no respond

**I: how long after birth women start breastfeeding?**

R: can be …..

**I: to make it more understandable. You daughter is giving birth, now after she give birth, how long after she start breastfeed the baby?**

R: no respond

**I: during pregnancy, how long after you started breastfeed your baby?**

R: they took the baby to the room where they can clean him while the other nurses cleaned me and them when I went to the room where the baby was inside, I started breastfeed him

**I: why was it very important to breastfeed the baby?**

R: so that my breast could contain breast milk, he needed to eat so that when the nurse came back and measure his bound, there shouldn’t be any problem.

**I: the very first breast milk in women’s breast, do you thing that is really important for the child?**

R: I think it’s not really that important because the very first milk in women’s breast milk is not that really important.

**I: were there any liquids other than breastmilk given in the first few days after birth and reason why? If someone in this community has given birth, do you have any ideas whether they give other liquids few days after gave birth?**

R: some birth mothers give other liquids like bought store milk. They put it in the baby bottle and feed the child with it.

**I: why do they give that to the child? Can you explain it more?**

R: some mothers says that they don’t have enough breastmilk to feed the child.

**I: ok. Any other reasons?**

R: some say that they want to make the baby used to feed not from them but from the bottle.

**I: so, are there any reasons why they say that they want to make them used to feed from the bottle? Why do they want to do that?**

R: each mothers have different thoughts. Some mothers would leave the child with their parents and go out and do whatever they want to.

**I: ok. And what about some that work? Does she do breastfeed or she give other liquid?**

R: yes. She can do both thing. When she is at work, the baby feed from any liquid, and when she is home, the child feed from her.

**I: can you explain breastfeed in this community? How long would a mother do breastfeed to her child?**

R: sometimes I see mothers feed their child for a very short time period. Even though they know that they child is not full yet, but they would stop anyway. They would leave the child with their mothers and go out somewhere else.

**I: any other liquids given for the child rather than the breastmilk in the first six months?**

R: some give tea, coffee, water, carnation milk, or cool-aid.

**I: in your own opinion, why would they give other liquids to the baby except from breastfeeding?**

R: silent (long pause)

**I: what are some reasons why mothers would give their babies other liquids than breast milk?**

R: the reason to that is to let go from the child’s breastfeed from them. They usually give them tea or also can be coffee.

**I: does it affect their breast when they give other liquids to the child?**

R: no. it does not affect them.

**I: what are the difficulties faced by mothers in your community to practicing exclusive breastfeed for six months?**

R: yes because when they don’t have enough foods that help provide breastmilk, they can’t do breastfeed for the child. If there is no fish, then they don’t have enough to feed the baby because fish is also one foods that help provide breast milk for the child.

**I: are there any specific ways to better support mothers to exclusively breastfeed for six months?**

R: yes. Mothers should feed children exclusively because they are supposed to feed by breastmilk because they are just babies. We don’t pretend to feed them any kind of liquids, they should be given fresh and nutritious food like the breast milk.

**I: you mentioned fresh food, can you explain more on that? What’s in the breast of a baby’s mother?**

R: there is vitamin in them, and don’t hit by flies.

**I: We are trying to understand how people eat in this community. Could you describe in detail what most families usually eat and drink throughout the day? What kind of foods or drinks they usually eat or drink throughout the day?**

R: someone in this family would wake up in the morning and have bread and coffee. For lunch, he/she would have chicken, rice and drink water. Dinner, the person can have soft food.

**I: what kind of soft food they usually eat?**

R: the coconut meat (more like the coconut herb) they would cook it with rice and boil it with water and make it soft. They would eat that with fish and drink water.

**I: as you look around and see people buying their foods, what do you usually see them buying for their children or for the adult?**

R: silent

**I: as of the kids, what do you usually see them buy to eat in the morning, during lunch or dinner? Or what do you usually see the adult buying for breakfast, lunch or for dinner?**

R: the adult usually eat rice with fried chicken. The children eat rice and hotdog for lunch and they would also do the same thing for dinner.

**I: what is the processed of doing the meals?**

R: some children would buy one or two eggs and also buy the hotdog and give to their mothers to cook these two kind of food so that they can eat. Adults ones can do fried or boil. Either way they would want their foods to be cooked.

**I: ok. Now who is the family is served first, next and last?**

R: I can make an example about my family. In my family, the people that should be served first are my husband’s parents, next, the children’s parents and then the children.

**I: is there is anything else? Why would you make arrangement like that?**

R: because sometimes when my husband’s parents don’t want to share foods with the other family member, I can share foods with them.

**I: is there is any differences in the foods served to different family members?**

R: there is. If it were you, I am you family member and you were the one to serve food for the family member, you could have fill my plate with only rice but you have plenty of meats on the table to share but you don’t want to share with me. You will share the meat with your favorited people in the family member and the rest that you hate, you won’t share it with them.

**I: ok. Ok. So are there any differences in quantities of food served to different family members? For example, one family member have bigger quantity of food than the other family? Is there is any quantity of foods?**

R: they have the same quantity of foods.

**I: do some children receive more food than the others?**

R: yes there is

**I: can you explain more on that why some children received more food than the other?**

R: sometimes it would go that way because for example, if I had hated the child, I would give only little food to him/her than the ones that I loved.

**I: ok. Now could you describe any food sharing between family members during mealtimes (for example children eating together separately from the family, meals eaten from the same plate by all family members?**

R: everyone get a separate plate. For example, this child get his/her own plate than that child. I have my own plate than my husband.

**I: is there is any food sharing between households or your neighbours?**

R; yes. When we make special food for special occasion, we share one plate to our neighbour. We share one plate to this house, or one plate to that house.

**I: Now I want to know about how young children eat in this community. Can you describe in detail what children under two years commonly eat throughout the day? From what you have been observed all day?**

R: some children when they eat, they usually eat rice with meat. But they usually eat junk food and sweet.

**I: so you mentioned meat, what kind of meat?**

R: sausage, luncheon meat (can meat) with egg, or corn beef fried with egg, they also can cook the corn beef with pine apple, with ooh no not pine apple. It’s the tomatoes!

**I: so when they eat sweet, what kind of sweets?**

R: like, ice candy, they usually eat ice candy and can be chips that can be fit their mouth and they can’t be chocked from it.

**I: now how many times a day meals are eaten by children under two?**

R: three times a day

**I: ok. Do children typically given snacks between meals?**

R: silent

**I: do they typically given snacks or can be sweets or let’s just say snacks like fruits or something like that.**

R: not frequently.

**I: now can you explain why they’re not given any snacks between meals?**

R: when there is no money to buy their snacks, they don’t usually get snacks.

**I: do children fed differently when they are sick from the time there are not?**

R: for example, when they get diarrhea, we look for the best food that can be given for them when they get sick.

**I: ok**

R: they have to eat healthy food so that they can be healthy.

**I: hmmm**

R: I don’t know how many kind of foods supposed to be on the table in order for the child to pick whatever they would want to eat. I can put papaya, pumpkin, or the coconut meat, and other kind of foods like biscuits because we are trying our best on the best food they would want to eat so that they can feel better.

**I: so is there is any differences in feeding practices between girls and boys under two years old?**

R: long pause (silent)

**I: is there is any differences when you feed your female child than your male child?**

R: there is no differences. When I feed my daughter, I also feed my son but the only different is their separate plate. They eat with separate plate than each other.

**I: so when they have separate plate, do you give more foods to your female child than your male child?**

R: they have the same quantity of foods.

**I: can you talk to me about what influences how families feed their children in this community?**

R: silent

**I: what influence how families feed their children in this community?**

R: silent

**I: why is to really important to feed your children?**

R: so that they can be healthy, they cannot live with hungry stomach, they cannot go house to house and beg for food, or so that they cannot eat from the ground.

**I: hmm. So as of health and nutrition, why important to you to feed your child healthy and nutritious foods?**

R: so that the child can live healthy

**I: healthy.**

R: grow, or getting tall

**I: Ok. We have heard from some families that eat local foods and others that eat processed foods. Could you explain what is typical for most families in this community?**

R: some family members would eat rice with tuna.

**I: why just rice and tuna and what about the other kind of meat?**

R: they think they rice and tuna is their favorited meal throughout the day. There is meat here in this house but they don’t really eat them as much as they eat tuna and rice because they say that tuna is their number one can meat and it’s really delicious.

**I: and what about Local food? What if it was Marshallese foods?**

R: Ok. There is no problem at all when it comes to local foods. If there is pndanus, crab, pig turtle any kind of local meat on the table, they eat them.

**I: So what kind of food they eat more, local or processed foods?**

R: silent

**I: people in this community eat more local food or processed foods?**

R: processed foods

**I: why do they eat more processed foods?**

R: they are healthy foods

**I: and don’t they really eat from local foods?**

R: Some people eat local foods and get allergy, they get skin rash and something like that. They eat a lot of greasy foods, they also drinks sweets, eating rice, and more.

**I: what makes it difficult to cook local foods?**

R: silent

**I: what makes it difficult to cook local foods?**

R: as of fish, we go and fished so that we can be able to eat fish. It you have pandanus, you have to cook them on the fire, if you want to eat crab, you have to dig the ground and get them, if you want sea food like bivalve or mussel you’ll have to go into the deep water in the ocean and get them.

**I: hmm. Ok. And what makes it easy to cook local foods?**

R: we can just cook them on the ground, like boil, or used underground over.

**I: ok. So what are the positive things about local foods?**

R: local foods do really make us fell healthy and live healthy.

**I: hmmm**

R: when we eat more local foods, it makes us eat more and more local foods and not really focus on processed foods. Processed foods can caused us to get diabetes sickness not compared to local foods that they do really make us feel healthy and live healthy. When we go to the hospital for check-up, the doctors see that we have good health and we don’t have diabetic illness. There is no problem in our kidney because there is no salt contain in these local foods.

**I: ok. So what are the negative things about local foods?**

R: there are some local foods that are not good for our health. We get allergy when we eat them. There are just some local foods that weaken our body. You get allergy, weaken the body, or also can caused diarrhea. If it was not appropriate for you to eat the fish but you eat it anyway, you will get diarrhea or you get allergy.

**I: and what are the positive things about processed foods?**

R: the reasons why we say that processed foods are good is because we think of what? Chicken, rice, bread, and we do really think they are delicious foods.

**I: anything else about the good thing of processed foods?**

R: there are some processed foods that make us healthy. That’s the reason why we say they are delicious, they make us feel they and live healthy.

**I: what kind of processed food you are talking about?**

R: like fruits and vegetables.

**I: like what?**

R: like orange, apple, or grape.

**I: ok. Now, you have any suggestions for balanced meals that can be prepared with locally available ingredients for children under two?**

R: silent (long pause) you can feed the child under two year so that they child can have healthy food. You can balanced the meal with local food with process food by putting the breadfruit juice (*more like the boiled breadfruit that taken out for the child to eat*) mix it with fish and feed the child.

**I: hmm. Anything else?**

R: umm. Now you can cook the pumpkin and mix it with papaya and also feed the child.

**I: Can you talk about what messages about breastfeeding and complementary feeding you give to mothers or other community members?**

R: there are sometimes we can give them rice with fish, or rice with local chicken or coconut meat that cooked with sea foods like clan with the cook coconut meat.

**I: hmm. Ok. Now is there is any nutrition education activities with community members as part of health work?**

R: silent

**I: to make the question easier and more understandable, are there any education from the ministry of health that given to the community members as part of health work?**

R: yes there is.

**I: can you explain more why you say yes. What kind of education given to the community member.**

R: they educate us on healthy food like these nutritious foods or local foods. Like taro, or just any local foods like, coconut meat.

**I: so is there is any difficulties to deliver nutrition messages to caregivers?**

R: silent (long pause)

**I: What makes it difficult to deliver messages about nutrition to people in this community?**

R: when nutrition is deliver to caregiver, sometimes some caregiver don’t want to learn anything about nutrition and health.

**I: so do you have any ideas why they don’t want to learn about nutrition and health? What makes it difficult for them not to study?**

R: their needs and wants. If there is something they’re up to, they would just go even though they know that there is a program happening in their community about nutrition and health.

**I: ok. What are some specific ways that nutrition communication could be more effective?**

R: silent

**I: what are the possible ways that these health programs can do in order to help their communication about nutrition and health?**

R: come straight to our homes and give us information at the same time they get information from us or people in this community.

**I: so you meant give them these information**

R: yes give these information to people in this community.

**I: Now I would like to talk about pregnant women in this community. Can you describe their diets during pregnancy?**

R: there are some pregnant women when I see them eating, they usually eat fish,

**I: hmm**

R: rice boiled with the coconut meat (more like the herb or the thing that grow inside the coconut *the food) these are the foods that I usually see them always wanted to eat during pregnancy. Eat the cooked pandanus and drink coconut drink.

**I: so in your own opinion whether these pregnant women usually change their diets during pregnancy?**

R: some pregnant women change their diet like when they eat, they usually eat chicken, like they want the fried chicken but the chicken has to be cook in medium rare cook. Medium rare chicken with rice and drink cold water.

**I: hmm. Now what really influence women’s diet during pregnancy?**

R: what makes it different is their diets. When mothers change their diets like before they have a son, then after when they change their diet, we know that the baby they are carrying is going to be girl.

**I: what kind of foods women are encouraged to eat during pregnancy and reasons why?**

R: nutritious foods that are good for the child they are carrying in their womb. They should eat local foods a lot. They also can eat rice but they shouldn’t be eat chicken a lot because the chicken contain enough grease or cholesterol.

**I: ok. How can that affect the child? How can healthy food affect the child?**

R: when they eat, the foods go straight to the baby and the child is eating from what the mother is eating.

**I: so the child can feel good or bad?**

R: the child will feel really healthy because the mother is eating nutritious food.

**I: what kind of foods women are encouraged not to eat during pregnancy and reasons why?**

R: like salt, cool-aid,

**I: anything else? They encouraged you not to eat during pregnant?**

R: drink soda

**I: hmm. Anything else instead of soda they encouraged pregnancy not to drink?**

R: coffee

**I: who encouraged or discourage eating those foods during pregnancy?**

R: silent

**I: who encouraged them?**

R: their parents

**I: anyone else?**

R: people around them or people in the community

**I: what if from the hospital? Who encouraged them?**

R: the doctors

**I: Can you tell me about supplements normally given to women during pregnancy?**

R: yes there is. Like the vitamins

**I: what kind of supplements they take from the doctors during pregnant?**

R: pill for their blood and vitamin pill

**I: ok. Umm. What are some ways that prevents women from taking their supplements?**

R: some feel nausea, some vomit, some have head ache when they take these supplements

**I: are there any times women drink alcohol, smoke or use other drugs during pregnant?**

R: yes

**I: Can you explain why they take these during pregnancy?**

R: because they said they are craving for drugs and alcohol during pregnant and they just want to get drunk and smoke

**I: ohh. So does it affect the child? And how does it affect the child?**

R: it will end up when the child born and how the child would look like. Some children look malnutrition, some have unmoral mouth (the mouth shaped like diamond or there is a cut in between the upper or lower lip), polio

**I: hm. Can you now describe women’s diet during breastfeeding in this community? What do breastfeeding women usually eat?**

R: rice with fish, mackerel

**I: do women usually change their diet during breastfeeding?**

R: yes. Some do and some don’t

**I: why do they change their diet during breastfeeding?**

R: so that they can provide enough breastmilk

**I: ok. What kind of foods encouraged women to eat during breastfeeding? Like really encouraged them to eat the foods**

R: they usually encouraged to eat fish.

**I: anything like fish**

R: mackerel also can provide enough breast milk for breastfeeding women

**I: anything that you were encouraged to eat during breastfeeding?**

R: also can be corned beef, apple, something like that

**I: what kind of food they were encouraged not to eat?**

R: silent

**I: what kind of foods they were encouraged no to eat?**

R: like salty foods, salt, unhealthy foods like chicken, turkey tail foods like that.

**I: who encouraged or discouraged eating those foods while breastfeeding?**

R: their mothers, and especially their nurses.

**I: As a traditional healer, what are some of your biggest concerns of the diet of pregnant and breastfeeding women in the communities you work in?**

R: silent

**I: as traditional healer, what are your biggest concerns of the diet of a pregnant woman or a breastfeeding woman should eat?**

R: can be like salt, cool-aid, ramen…

**I: ok. Now for the last section, we would like to learn about ways we can develop health program in your community.**

**I: Could you explain where community members usually get trusted information and nutrition and health? Information on nutritious foods and health?**

R: from the doctors. From the nurses. From when we go to churches, the radio station

**I: ok. Now can you tell me reasons why these sources are trusted? For example, why do you trusted sources that comes from the health centre?**

R: because it is the exact and right place to get our information from is from the nurses

**I: ok**

R: especially from the doctors

**I: Where nutrition and health messages should be delivered so that community members would see or hear them most easily?**

R: radio b7av (Marshall Islands International Radio Station), school, churches and can be anywhere around the Marshall Islands.

**I: Ok now what types of media that you use the most to communicate?**

R: telephone, online, and face-book

**I: ok. You give the right places because today people use them a lot.**

**I: For our last questions, could you describe what influences how people raise children in this community?**

R: silent (long pause)

**I: can you explain or describe what influence how people raise children in this community?**

R: they grow up and lived healthy. They don’t get any kind of illness. They don’t feel lazy or nausea, they feel healthy.

**I: ok. Now is there is any specific advice or information related to parenting typically given to community members?**

R: silent

**I: is there is any advice given to the community in term of parenting? Like mothers, fathers, or can by any caregivers is there is any advice or information given to them?**

R: they really have to take good care of the child and watched over the child from playing in dirt, go and play in danger areas, like they have to watch over them carefully from harm things.

**I: So now is there is any information that pregnant or breastfeeding women typically ask for from health workers?**

R: silent

**I: any information that pregnant or breastfeeding mothers ask for from the health workers?**

R: coughing.. Silent (long pause)

**I: let me rephrase the question, like is there is any advice or information that breastfeeding or pregnant women ask from the health workers?**

R: there is none. I don’t know

**I: ok. What do you think is the best way to communicate with caregivers about health?**

R: silent

**I: do you have any ideas or what is the best you that you see that it would help give advice to caregivers or parent in raising children?**

R: caregivers have to take good care of the children from illness, harm …

**I: ok. So is there is anything else about the topics we talked about today that we missed or that you would like to tell us about?**

R: no there is none

**I: ok that’s great. Thank so much once again for your generous time and for sharing your thoughts with us. We greatly appreciate your help and we hope this research will help us improve the health of mothers and children in your community. Once again. Thank you**

R: thanks to you too.
