# Supplementary material for: Conceptualizing multi-level determinants of infant and young child nutrition in the Republic of Marshall Islands–a socio-ecological perspective
Source: PLOS Glob Public Health. 2022 Dec 19;2(12):e0001343. doi: 10.1371/journal.pgph.0001343 (PMC10022247; doi:10.1371/journal.pgph.0001343)
Supplement: S1 Data — (ZIP) [file pgph.0001343.s001.zip › RMI Supp Data/Interviews data/I20U_IDI_HW_Rita_Aug 15_Fela.docx]

**Interview Code: 120U**

**Interview type and interviewee: HW**

**Interview date: Aug 15**

**Location: Rita**

**Interviewer: Fela**

**Transcriber: Shante**

**I: Before we start, I would like to know if you agree on what we are about to do? Ok?**

R: Yes**.**

**I: Thank you… Thank you for giving this time for us to talk. Your information you will give me will help make our community a better place and for mothers and children to have a better and healthy life. Before we start can you tell me what you, health workers do?**

R: I work with the postpartum local treatments.

**I: What kind of medicines to you give them?**

R: they usually drink bobo and wutilomar (local medicines).

**I: Can you tell me how to make the bobo?**

R: I pound them and add local leaves and some coconut and put them in a rag and squeeze the juice out of it for the mother to drink it.

**I: Other then those medicines, is there anything else you do for the mothers?**

R: I give them kidren (local medicines) to drink.

**I:** ***Let’s now talk about illness****.* **I am specifically interested in illnesses that children suffer from. In your community, what illnesses would you say children under 2 years commonly suffer from?**

**R: Cough, fever, and diarrhea.**

**I: If they have fever, what causes them to have fever?**

R: its when they have a pump inside their stomach.

**I: Can you tell me what you do when they have stomach pump?**

R: I give them stomach massage.

**I: Is there anything else you do?**

***R: I also make them drink local medicines. Like meria (local medicines for fever).***

**I: As a traditional healer, do you think your strong medicines are strong?**

R: I think so because when I make the medicines for my child she heals quickly.

**I: Ok. For cough fever and diarrhea, what would make the kids not to have these kinds of illnesses?**

R: I usually give my kids traditional medicines and they barely get sick because I usually give them local medicines to drink. Sometimes I give my children both medicines. Traditional medicines and medicines from the hospital.

**I: You mention traditional medicine. can you give me the names of the medicines?**

R: Meria and Noni.

**I: Which part of the Noni do you take?**

R: the seed and the little leaf on the noni.

**I: Can you tell me what are the affects of these illnesses?**

R: they will have asthma when they cough a lot and they will get shocked when their fever is high.

**I: Ok. How do we prevent these illnesses?**

R: Is there on the things that I said, I use meria and noni. For me I think all mothers should stay close with her child when they are sick so that they can take care of them and make sure their sickness won’t get worst.

**I: So, your understanding is that all mothers should closely pay attention to their child when to are sick or when they’re getting sick?**

R: Yes, because its better for them to pay attention to them when they know that they’re getting sick because if they are they should rush to the hospital or give traditional medicines to the child before their sickness gets worst.

**I: Now what do you usually treat those sickness with?**

R: like I said Meria and Noni. And sometimes the doctors.

**I: Who do you bring your child first when he/she is sick?**

R: No one. I take care of them on my own.

**I: What kind of medicines do you use for your child?**

R: I use traditional medicines and sometimes I take my child to the hospital so that they can give me medicines for my child.

**I**: **Can you tell me about any challenges your community faces in seeking treatment for the illnesses you mentioned before?**

R: I stay in the outer islands and I’m still waiting for the ship to get ready to leave. Anyways on the outer islands where u live, we don’t have enough imported medicines. And its to far for us to reach where the doctor is.

**I: Is there any difficulties on getting the Marshallese medicines?**

R: No because we are already surrounded with our traditional medicines. They are planted all around us.

**I: What difficulties do you have on helping other mothers or helping the sick people that you use your local medicines with?**

R: No. I don’t have any difficulties.

**I: That is exactly the level of detail that I am looking for in your answers – thank you. Can you describe any illnesses associated with nutrition that affect children in your community?**

R: On outer islands kids there eat Pandanus, pumpkin and bananas. All those foods they eat, they don’t get sick from them. All those foods have lots of Viber and Calcium.

**I: What kind of food that will make children body not healthy?**

R: On outer islands we don’t have any problems on that… {giggles} kids don’t eat lollipop.

**I: What about on this community?**

R: well here on Majuro, kids here eat too much rice, rice, and rice, rice with chicken and they don’t add vegetables. But its good that they drink water a lot. Sometimes when they are given milk to drink they don’t want to. Other islands kids there, drink lots of coconut juice, eat fish, breadfruit and Pandanus and more healthy food.

**I: What is the most important thing that mothers should feed their baby?**

R: Breastmilk! {laughs} that’s the most important meal for a baby that should always be given to him/her. And we also feed them soup.

**I: Can you tell me what you put inside the soup?**

R: fish vegetables and rice.

**I:** **We talked a lot about being unhealthy. Could you now describe for me a typical day of someone living a healthy lifestyle, from the time they wake up in the morning until when they go to bed?**

R: For my child when he wakes up feeling good and healthy, he walks around, play, eat a lot.

**I: What are the signs of a healthy adult?**

R: For that lady over there, my grandmother. She’s 93 years old and she’s doing very good. She eats ok. Drinks ok and move around as if she’s still young and she doesn’t forget things. She remembers what she did on her early teen days. She’s the example to all elders and all grown ups. [giggles]

**I: Ok that’s great. I have one more set of illness questions but related to women’s health now. Could you tell me about your experiences with women who have Anaemia? Can you tell me about those women that doesn’t have enough blood? That you know of.**

R: sometimes the girls or women have their monthly period and it won’t stop. They will just have their period nonstop. Sometimes eating sashimi its good for those that has less blood. Sashimi can make their body to gain more blood. Or sometimes they don’t have enough blood because they don’t drink their medicines the doctors give them.

**I: Do women ever think that having anaemia is bad?**

R: some care about it and some doesn’t. some women can have anaemia in 2 weeks 4 months or so… some if they see that they are sick they will go straight to the doctor and get checked up. And some others just won’t care. Sometimes they don’t want to go to the doctors because they are afraid if the doctors say they have other illnesses like cancer. So instead of going to the doctors they use traditional medicines.

**I: What kind of traditional medicines are they looking for?**

R: Like madok nin jojo, wutilomar, kar, med kipwepwe, uleej. (few of the local medicines name use for all kinds of illnesses). These are all add up together to create medicines for cancer.

**I: what makes the young girls to have less blood?**

R: Sometimes they can have less blood from having their periods. And maybe because they don’t have enough vitamin in them…

**I: Is there any advice given to women for them to prevention and treatment of anaemia?**

R: Yes. They should use traditional medicines because they have a cure for all kinds sickness. Some they ask the people who makes their medicines to help make their medicines and when they are done preparing their medicines they don’t finish drinking them. Sometimes because they don’t have any freezer to keep their medicines cool or because sometimes its doesn’t taste good.

**I: The one that makes the medicines, does she get mad about the girls that waste the medicines she made?**

R: yes. She will get mad because making traditional medicines is not easy and its a lot of work. For me I would first get all the leaf for the medicines. Look for coconut and grind it and the pound the other leaf’s… it’s a lot of work and I would be upset if the women I made the medicines for would put them to waste. Some they don’t want to waste the medicines but it’s the taste of it that they don’t like.

**I: Now we would like to talk about breastfeeding in this community. Could you explain exclusive breastfeeding practices in this community? Can you tell me how long does it take for a mother to breastfeed right after she gave birth?**

R: For me and my daughter we breastfed, her child is now 2 years old. The moment her baby was born she breastfed her, the first water in our breast when we first gave birth is colostrum, and for the first few days of the baby’s life colostrum is the perfect first food for the newborn because it is high in protein. That’s why breastfeeding is good for the child. It will make the child grow healthy and live a healthier life.

**I: Ok. That was good answer thank you. Now are there any other liquids the mother gave to her child other then breastmilk?**

R: I don’t know what the doctors usually give the babies. I saw them squirt something to their mouth, but I didn’t know what is was, when I was in the hospital with my sister who gave birth I saw the doctors squirt some liquids inside the newborn child’s mouth, but I didn’t know what is was.

**I: Oh ok. Can you tell me how women in this community breastfeed only?**

R: Some women don’t want to breastfeed, and some women wants.

**I: So, what if the child wants to be breastfed**?

R: Sometimes the mother will get upset.

**I: Why would she get upset?**

R: Because they would want to finish their laundry but the couldn’t because the child would be crying for her to be breastfed. The mother would want to finish all her chores, but she can’t because the baby kept crying for her to be breastfed and sometimes the mother would be upset because she wants her chores done but her child is on her way of doing it. And sometimes when the mother of the father’s child see’s that women laying down and breastfeed her child she would say,’’ she’s so lazy! All she does is lay down and breastfeed her child, but she doesn’t want to do her chores. She should be cooking food and washing her husband’s cloths but she just there laying down and breastfeed her child all day. ‘’ That’s what some mothers in law would say about their daughters in law here. They would think they are lazy, but they are not, is just that the baby always wants to be breastfed. That’s why sometimes they get upset. They want to finish their chores. but can’t because they breastfeed their baby.

**I: Ok… are there any other liquids they gave the child other then breastmilk on their first 6 months?**

R: they usually give them Simlac and Enfamil (milk powder in a can) and all those other formulas that I don’t know what they are called.

**I: What are the difficulties for the mother to breastfeed on those 6 months?**

R: Some mothers says they sometimes can’t breastfeed their child because their nipples hurt from the child biting it. And sometimes it’s difficult for them because they need to do their chores and cooking, but they can’t because they are breastfeeding. Some say it’s difficult, but the truth is one Women should find a way to do everything an make it right and good. That’s their duties.

**I: Ok… What are some better ways to support mothers to exclusively breastfeed their baby for 6 months?**

R: Its simple, if they want their kids to have a better healthy life and don’t want their kids to be sick and go to the hospital then they should breastfeed their child until then.

**I: We are trying to understand how people eat in this community. Could you describe in detail what most families usually eat and drink throughout the day?**

R: For my household, we usually eat donuts and drink coffee, sometimes bread, meat and rice. The kids in my house always drink water. Even when they eat they will always drink water. Unless they are eating cereal then they will use milk. But other then that they will drink water with everything they eat**.**

**I: ok so, you mentioned rice bread and meat. Can you tell me how you make them?**

R: for the meats, we boil them. If we don’t have enough money for vegetables, then we just take papaya from outside my house and add them on our plate.

**I: In this family, who gets to eat first and who gets to eat last? And why?**

R: The kids first. Sometimes the workers get to eat first because they are in a hurry for work. And my mom also gets to eat first.

**I: Is there any difference on how you give the amount of food to each family members?**

R: No difference.

**I: Is there any difference on how you make your food then your family members?**

R: No. I don’t think so. If we eat pancake we all eat pancake. The only different is when the older kids come grab 3 or 4 pancakes. They will have more pancakes then the younger kids. That’s the only difference.

**I: Ok. Is there any difference on the amounts of food each child gets?**

R: Some other kids in their homes gets different amount of food but not kids in my household. Every Children gets the same amount of foods.

**I:** **We are trying to understand how people eat in this community. Could you describe in detail what most families usually eat and drink throughout the day?**

R: No. everyone eats separately. The kids have their own plates along with the elders. Everyone eats separately with their own plates. Except if the couples here want to eat together on the same plate then they do.

**I: Ok. Now do you share your foods with your neighbors?**

R: Yes. We do. Sometimes when we have enough food then you share them.

**I: Now I want to know about how young children eat in this community. Can you describe in detail what children under 2 years commonly eat throughout the day?**

R: they usually eat bananas and papaya. Some houses they hang bananas in their house and children just go grab the bananas and eat them every time. When there is pandanus too they eat.

**I: How many times in a day a child below 2 years old eats?**

R: they usually eat 3 times a day and sometimes I usually make them snacks. I usually bake cookies and cake and make ice candies for them to eat for their snacks.

**I: Ok. Is there any difference on how you feed your child when they are sick?**

R: Yes. The food that they used to eat when they are not are the foods that don’t like to eat. and sometimes when they see other kids eating something they would say they want to eat the same food the child they see is eating. If they see other kids eating orange, then they would want to eat orange too.

**I: Is there any difference on how you feed the girl and how you feed the boy?**

R: sometimes the girls don’t like to eat too much then the other boys. Girls just like to eat a little and boys like to eat too much.

**I: We have heard from some families that eat local foods and others that eat processed foods. Could you explain what is typical for most families in this community?**

R: for my house. The boys in my house usually goes out fishing. We barely buy imported food because the boys in my house usually goes fishing. And we have bananas and pandanus and breadfruit tree out my house so its easy for us to just take it and cook it. All those that lives in the house eats the same food. If I said everyone is going to eat banana this evening, then everyone will eat. they like it and they don’t complain about it.

**I: We have heard from some families that eat local foods and others that eat processed foods. Could you explain what is typical for most families in this community?**

R: like I said before, we eat banana and sometimes pandanus but not all the time because there are times for pandanus. And breadfruit.

**I: Can you tell me what makes it difficult or easy to cook local foods?**

R: for here on Majuro it will sometimes be difficult because when we’re going to start the fire to cook the foods some of the neighbors might get mad because the smoke will go to their houses. There’s so many houses close together and sometimes they complain because when we make fire for cooking the smoke goes to their house and they don’t like it. That’s one of the reasons why its difficult to cook local foods.

**I: Ok. Now can you tell me how easy it is to cook local foods?**

R: the easiest way to cook our local food is using the stove. Its easier and faster.

**I: What are the good and bad in eating local foods?**

R: the good things about local food is sometimes when we don’t have enough money we can just take bananas and breadfruit and pandanus from outside our house and then cook it and eat it, that’s one of the reasons why its good. And the bad things about it if we can’t find any local food then we won’t get to eat them. And we don’t have enough money to buy them from the store.

**I: Ok. Thank you. Your information will really help our program. Can you tell me what is bad about imported food?**

R: For me, I don’t like the taste. And some of the imported food are hard to cook. And some other people don’t know how to cook them**.**

**I: Can you tell me about your thoughts of balanced meals that can be prepared with locally food available ingredients for children under 2 years old.**

R: Yes. We can make breadfruit soup mix with carrot and chicken and other vegetable. The children under 2 years old love to eat breadfruit soap and fish with carrots and potato. They also love to eat breadfruit chips.

**I: Can you talk about what messages about breastfeeding and complementary feeding you give to mothers or other community members?**

R: The food we give to the mothers that breastfeed is too make their breast produce and gain lots of milk for the baby. They eat banana and fish to make their breast produce lots of milk and it will be enough for the child to eat from. the mothers that breastfeed, they eat for themselves and for their baby. So, they eat for 2 Person. Her and the baby.

**I: Is there any advice on nutritious food given to caregivers in this community?**

R: Well that’s what your job is, to teach them and make them understand. It’s your job to let them know about the importance of Nutrition and their health.

**I: Is there any difficulties on teaching them about Nutrition foods?**

R: No. I don’t think so… someone is responsible for themselves if they want to learn about it or not. If there’s no difficulties with the ministry of health with teaching about Nutrition, then it won’t be a problem to the people here and there. Whoever want’s or care about their health, then they will seek the ministry of health or any workers at the hospital to help them out and their needs of understanding about nutrition and health. Long time ago there use to be a team from the hospital that goes around to houses and teach the people about how to cook healthy foods and the important of healthy foods.

**I: Could you tell me more about some specific ways that nutrition communication could be more effective?**

R: Like I said before, if the people care they will be interested in joining the programs about Nutrition. And you guys that do the surveys should always make people understand about these things. Let them understand about Nutrition and health. Give them in detail so that they can truly understand. Some people might say they understand but they don’t because they are shy or will be afraid to say they don’t understand. So, if you guys go around and do surveys please let people really understand.

**I: Ok. Thank you… all your information will surely help us with our programs**. **Now I would like to talk about pregnant women in this community. Can you describe their diets during pregnancy?**

R: Pregnant women are always choosey with their foods. Some won’t eat anything because what they crave for aren’t available. Some if they say they want that chicken to eat it, they’re husbands will kill the chicken just so that his pregnant wife could eat it. IF what they want to eat isn’t available they will be miserable! They will be moody and wont’ be happy until they eat what they want to eat.

**I: Can you tell me about the difference on their food when they are pregnant?**

R: well I don’t know. [giggles]

**I: Can you tell me what kind of foods women are encouraged to eat during their pregnancy?**

R: they encouraged them to eat the 3 groups of food. {I don’t know what they are so ask fela 😊} usually when we go see the doctors for our appointment during our pregnancy, they would remind us not to forget to eat our 3 groups meals. But sometimes when we don’t have money we won’t eat them. [giggles].

**I: What kind of foods do they encouraged pregnant women not to eat?**

R: salt… Usually salty foods because that’s what pregnant women’s sometimes usually wants to eat. Sometimes they smoke but we won’t say that because it’s not a food. We tell pregnant mothers not to eat salty foods because it will affect both her and the child.

**I: Can you tell me who encourages women to eat healthy food during pregnancy and why?**

R: Some women trust the advice of their spouses and they will listen to them about what food they should eat and not to eat. sometimes the doctors encouraged them to eat healthy foods just for the sake of both the women and the child.

**I: Can you tell me about any supplements normally given to women during pregnancy?**

R: Just that the medicines they need to take its for her child and herself.

**I: Can you tell me what prevents pregnant women from taking their medicines?**

R: Some women doesn’t like taking the medicines because they can get nausea and dizziness and they might vomit from taking the medicines. It depends and their hormones. Some women they want to take it but once they taste the medicines they vomit right away.

**I: Is there anytime that pregnant women smokes, drink alcohol and take strong supplement during their pregnancy?**

R: yes.

**I: Can you describe what kind of foods does the mother’s that breastfeed eats in this community?**

R: Fish and sometimes chicken. For the women on outer islands where I’m from, they eat fruits and sea foods.

**I: from your own understanding as a traditional healer, can you tell me what bothers you about the food mother’s that breastfeeds eats?**

R: I don’t think there is because the food that she eats is I cook them/make them for her. And the food that I make for her are healthy foods for both her and the child.

**I: *Now for the last section, we would like to learn about ways we can develop health programs in your community.* Could you explain where community members usually get trusted information about nutrition and health?**

R: On the radio and from the programs that the Public Health usually Host.

**I: From your won point of view. Could you tell me why you believe the information from where you hear it from**?

R: because they usually come over to our place and give us medicines and remind us about Nutrition and health. They told me that if there are no illnesses, then everything will be ok.

**I: Now can you tell me where you would want all the information to go for you to easily see and hear about it everyday?**

R: do you guys have your own radio station for your programs that you do?

**I: I think there is.**

R: Ok. Well I think you guys should schedule a day for your programs to be out on the radio station, so we could listen to it. And people in the outer islands will always listen to it because they always listen to the radio. And I wouldn’t say we hear it from the churches because that’s not where they usually mention those kinds of stuffs. Or you guys can post it up on internet because that’s what everyone uses nowadays.

**I: Can you tell me what kind of device do you usually use to listen or to see the information with?**

R: radio.

**I:** **For our last question, could you describe what influences how people raise children in this community?**

R: sometimes I see children not wearing shirts and I would ask they’re parents why aren’t they putting shirt on their chid. But sometimes I want to ask parent why their child keeps wearing the cloths for 2 days. I want to ask them, why are they not cleaning their child? but I couldn’t because it would be rude, and it might make them embarrassed. Sometimes the kids in this neighbor comes around here in my place and ask for quarters or ask for foods and stuff like that.

**I: Is there any word of advice you can tell the parents in this community?**

R: Yes. I usually tell them that they should clean their children and get them dress and bring them to the place where the surveys usually do their programs, so that both them and their kids should go, and listen and learn from what the survey team are going to discuss.

**I: Ok. Is there anything that the breastfeeding or the pregnant women usually ask from the doctors?**

R: Some asked, and some don’t. for me I asked doctors, what are the medicines are for and what are the causes of them.

**I: Is there any better ways for you to communicate to caregivers about health?**

R: there’s a lot that we see and want to talk about but everyone just too lazy or they have their own excuses.

**I: Is there anything else about the topics we talked about today that we missed or that you would like to tell us about?**

R: Ok. Now that you’ve done your interview now, where will your information go for everyone to learn it from? will it be only here in this community or everywhere else?

**I: For this community and to all mothers and children out there. Including other adults in our country. And for those in the outer islands, we can make a radio station for them to listen to so that they won’t have to miss out on anything**.

R: So, if you guys are done with your survey, Will the mothers here that have pre-mature child get any help in this? Because some mothers here don’t have enough to feed their child and they won’t be fill with vitamin and Nutrition…

**I: Ok. That’s good to know. Honestly, I am not prepared to answer your question, but I will hand it over to my boss and the ones that are in charge, of this survey for them to answer this.**

R: Ok. Thank you. At least for someone to come see the ones that truly need help.

**I: Ok. I will surely give your statement to the ones in charge of this survey. Thank you so very much for your precious time. I really enjoyed this interview. Thank you once again.**

R: you are welcome and thank you also.
