# Supplementary material for: Conceptualizing multi-level determinants of infant and young child nutrition in the Republic of Marshall Islands–a socio-ecological perspective
Source: PLOS Glob Public Health. 2022 Dec 19;2(12):e0001343. doi: 10.1371/journal.pgph.0001343 (PMC10022247; doi:10.1371/journal.pgph.0001343)
Supplement: S1 Data — (ZIP) [file pgph.0001343.s001.zip › RMI Supp Data/Interviews data/I21U_IDI_MCG_Rita_Aug 15_BM edited.rtf]

I: Before we start, can you tell me a little about your family?
R: In my family there are 2 girls, one of them goes to school and she's in 1st grade. The other is younger, she is about 1 years of age. Also that old man that just came in, he is the father of my wife and the owner of this house. 
I: Okay you have 2 children, your wife, and your father in law, is there any other people that lives here?
R: One of our niece and my wifes mother.
I: You said you had 2 children, can you tell me their ages?
R: The eldest one is 7 years old. The youngest one is 1 year and 6 months now.
I: Next, I'd like to ask you to describe your community?
R: Well this community is really great. Theres no problem.
I: You said this community is really great, can you tell me more about it?
R: Okay. First, it's not noisey, second when I'm busy my neighberhood would come help me out with anything that needs to be done around. The problem here is power outage here in rita. And it's all good that's it, lets just say that here is calm.
I: Can you describe the negative things about this community?
R: The only negative thing is kids. When they play at night they would sometimes throw rocks and nothing else.
I: Let's now talk about health and illnesses in your family. Can you tell me about some of the illnesses that your children have suffered from?
R: Fever, warm, coughs, sometimes coughs. What else...? When I look at it I think these are the only illness my children catches. You know, these illnesses that children's always get, fever and coughs. There's nothing else.
I: Can you tell me the cause of your child getting warm?
R: Well there are times... When they shower under the rain, well from my thoughts but it's not like they get sick all the time. Sometimes you know the coconut juice, the small one, we would give them to drink it when they get sick. It's not like they take it all the time because they don't get sick very often. Just sometimes.
I: Can you tell me the seriousness of your child getting warm?
R: Well they would burn up and it would reach up to 105. So we would turn of the AC in the room or come out here and sleep. And there was this time that the older sister got really burned up and she had it for a week.
I: Can you explain on the ways to prevent the warm?
R: Well it's a good thing I would go to the hospital and they would give me medicines. Also I would take medicines from K&K and Payless. Because it has tasty flavors. And also because the flavor of the medicines from the hospital doesn't taste very well. Like my daughter she hates the taste but when we bought the medicines at the market she likes it better.
I: You also said fever. What was the cause of the fever?
R: Well about the fever, when my youngest daughter was still a baby and the time when she's starting to have teeth also the eldest one had this too. You know that we say kata(babies getting sick before knowing and having the abilities to do so) right. There were some times when they would go swimming in the lagoon side and when we look at it, it's probably the reason why they get fever. Sometime they would cry and whine because they want to swim in their mini pool. And when they swim for a long time and we would take them out next thing you know OOOH!! Their sick.
I: Can you tell me on ways to prevent the fever?
R: Oh yes, we don't sleep because we have to keep monitor to make sure they don't sleep bad, you know kids. They would cry and give us attitude and bad behavior, they wouldn't eat, man it is so bad. The thing is they won't eat they just want to drink. That's the thing, when they don't want to eat, they wouldn't for the whole day. They only drink. That's serious to me because, we try and force them eat or try and see what they would eat.
I: What were the ways you prevented the fever?
R: Oh, they would drink medicines. We would bring them medicines sometimes we would use towel to lower their warmness.
I: Can you describe how you know when your child needs treatment for their illness?
R: We have a thermometer to check their heat. We would feel the heat and we use the thermometer and when goes high we would say, okay lets take them to the hospital. There are some times we would go in the day time there are times we would go in night times. Because they're overheated. There's nothing else I would do, just go straight to the hospital. You know medicines, we would need medicines.
I: Can you tell me besides going to the hospital, do you use traditional healers and tradition medicine?
R: Naaaah.
I: Can you tell me why you don't use traditional healers or tradition medicines?
R: Oh that's because we don't believe in those type of stuff. Just the massage part for the stomach. But we'd rather go to the hospital.
I: Can you describe any illnesse affecting your children that are associated with nutrition?
R: They would... what, weak. For my children I would keep on track with their snacks, like these you see oranges and apples.
I: Can you explain the types of foods that make your child's body unhealthy and reasons why?
R: Well nowadays a lot of kids would eat junk foods. That's the thing, my target is to make sure my kids don't too much junk food. Like eating chips and drinking sodas. Also nowadays a lot of kids usually eats raw ramen. Like in the morning, lunch, and dinner. Ramen taste really good with kids and I'm making sure that my kids don't eat so much. That's what I'm trying to do, making sure my kids don't eat so much junk food and espicially the ones that are salty.
I: Can you explain the types of foods that make you child's body healthy and reasons why?
R: The foods that they eat most of the time like in the morning they would eat french toast, scramble eggs, and cereal. My eldest daughter likes to eat pancake and eggs. But when it comes to feeding them cereal though out the day, well we don't. We would feed them randomly, like today they would eat pancake, tomarrow french toast, and from the looks of it from me everything seems fine.
I: Can you tell me any illnesses caused by foods missing from the diet?
R: Not enough vitamins.
I: Anything else than vitamins?
R: Well ahh... I don't know, maybe lost of weight. Yes probably that as well.
I: We talked a lot about being unhealthy. Could you now describe for me a typical day of someone living a healthy lifestyle, from the time they wake up in the morning until when they go to bed?
R: Hmm, oh. Like my daughter, the one that doesn't go to school. When she wakes up, like today, for example today, she would eat pancakes and eggs. I would cook her ramen, in the morining. Also she would eat cereal. When it comes to lunch time, I would think of cooking her ham with eggs. Then between hours I would give her these kind of snacks, orange and apples. The most improtant is dinner. That's the most improtant part for me is dinner. If we have a bit of money we would buy them food, because my daughters like to eat chicken katsu. So we would go and bring chicken katsu. But for a week they would eat chicken katsu only 3 times in a week for dinner only. But this one, boil chicken is her favorite. Without salt and other spices. Just boil and just that. But when it comes to eating fruits, they would eat between hours. 
I: Can you tell me the appearanc/signs of a healthy child under 2 years?
R: Can you say that again?
I: Can you tell me any signs of a healthy child under 2 years old?
R: Ohh, well like my daughter, just for an example. Since she's under 2 years of age. Before her weight was very low, but we took our time feeding her and she grew. Not just that but when we looked at her she was skinny like her arms and legs and I know if you would of looked at her you could tell that we did't feed her enough. Like myself when I looked at my daughter see that she's scronny and looked like she's unhealthy I would use my time with her, feeding her. Then I notice that she would grow and gain more weight. And that's how I know that she's healthy.
I: Can you tell me the appearance/signs of a healthy adult?
R: They feel lively.
I: Anything else?
R: Ah, what else? Their strong. If I can make an example of my father in law. He is living very well but the only problem is the injure that he has on his feet. It seems like that the only thing that makes him look weak. But he is very strong, he would work on anything that needs to be work on. But the only thing that is bothering him the most is that injure on his feet. I know that when I see old people they would easily get sick. Like around 50's or 60's, they would get body ache like mostly their legs. Like when their legs are usually in pain and back aches it's the only thing they would suffer from. But when it comes to other things but only when they have bruises on their body it's the only thing that they would suffer from. 
I: Now we would like to learn about the foods that are commonly available in your community. I would like you to explain how your household gets food to eat on a daily basis.
R: Well you know this is Majuro, like since we're here in the Majuro. If we want so that there would be foods in the household you know when there's green paper, you know money, when we get paid. Well that's the time when we would say okay, we need this and that and this and that. Since there's only 4 us here in this house, uhh, me, my wife, our parents, oh there's 6 of us. Like for us for cooking rice we would use 4 cup and it would last until dinner time. For us to make food in one day, since there's some of us that goes to work and the only ones home are me, our father, and our daughter. But everyone else is gone. So they would eat lunch at a fast food or restruant. So the only important time of the meal is in the morning and at dinner because those are the only time we get to eat with eachoher. In the refrigrator we would fill it up with food not that I'm saying we would fill it up with the same supplies or products but it would be filled randomly. Like it takes a month until one of the product would be out so we would say we're out of this and that. But when I look at it, it depends on how many people lives in the house. Like in this house since we have a few people it takes a long time until our food is out.
I: Can you tell me what foods are grown here at your home?
R: Oh, you mean like the ones that are planted right? Well we don't have any, there use to be bananas but there's no more.
I: Can you tell me about any difficulties to growing food at home?
R: Well the only problem which is the biggest problem here is because the ocean is right here at our backyard. That's the reason why we can't plant. Because the salt water. There use to be banana outside of the house but when it's too windy the bananas don't grow very well.
I: Please explain what your family would need to grow food at home?
R: Man, what would it be..? I think planting here wouldn't work. Probably if we made a fence for gardening. Like probably somthing that could prevent the salt water from wetting the plants. That would be the only thing we would need.
I: Could you explain how easy or difficult it is to get food every month during the year?
R: Oh yes, there would be times when is easy and there are times when there is difficulties. But here in Majuro, for example here in Majuro. There should't be any difficulties here, since we're here in Majuro only one type of food that we usually put in our refrigrator which that is a case of chicken quarter legs. Also we would get a bag of rice and a bag of flour. But it comes to produce and the other stuff, well those will be the only things we would buy everyday. But when it comes to supplies for a month, those will be it. Those will be the foods that we would take, chicken quarter legs, rice, and flour. That will last for a month.
I: Can you tell me any other food shortage throughout the year and their main causes?
R: Nothing. Everything is good.
I: Can you explain what do you do to feed your family if there is a shortage of food in the household?
R: I haven't been through in that case before.
I: Okay, now I will ask you about animals. Do you have any animals here at home?
R: We don't have any.
I: Okay, then I'll ask you, from your own perspective, can you tell me any difficulties to raising animals?
R: Well around the house there aren't much animals around like pigs, but there are dogs. But when it comes to pigs there wouldn't be because the community is so crouded that they wouldn't bring pigs cause the pig's feces smell very bad. Yea I think that's the reason, there's no space for pigs. When there's a lot of houses here we don't want to raise any animals.
I: There are sometimes foods that we wish we could eat, but for some reason we cannot do so. Could you tell me about any foods you wish your family could eat but cannot?
R: Well if it were our parents, my wife's parents. They usually want to eat breadfruit, local foods. Then we would have to go and check at the local stores or local markets. When they want to eat coconut meat (iu) we would go to the local store or markets to get some. The only thing they would want to eat is local foods. It would also be difficult, because we wouldn't find any or there wouldn't be any on stock. Then we would have to go to Laura to find any so that they would eat until they're satisfied. 
I: Can you please explain reasons why your family cannot eat these foods recularly?
R: Nothing, that would be the only reason why we couldn't get any of the foods because there aren't any left to find. That's the problem, sometimes there wouldn't be any and we couldn't find any.
I: For the last question on food, can you explain who decides what food to get for your family?
R: No one. Everyone decides on what they want to eat. Like our parents, if they don't want to eat fried chicken then we would boil chicken for them cause they like boil chicken more. Like if I was the one cooking I would seperate their food just in case then boil it and if we want to eat fry then I would cook it. I sometimes bring fish, I would also boil them and fry them so that everyone gets what they want.
I: Can you tell me who decides which foods your children should eat?
R: Well that would be me and my wife. There are times that I make foods for my children.
I: In the next section, we would like to talk about water and hygiene. Can you please describe a typical day getting and storing water for your family?
R: If it comes to drinking water, there are three place of water at this house. Two water catchments and one home made cement of water. Now one of these water catchments there is one that we use for drinking and cooking. Now when it comes to storing the water we would make sure the cover of the water catchment is secure. It has filters. Now when it comes to the other two, we use one them as cleaning water and the other one to shower with. But usually we use both of them for cleaning and showering. Well that's it, that's how we store our water.
I: Can you tell me any difficulties in getting water?
R: Well let me give you an example about the time when it was really hot. Do you know about that time? It kept going until we ran out of water for cleaning and for showeing. But there was still water for drinking and cooking. And that was the only thing we tried to prevent it from running out. And we made it happen. But that time when we ran out of water we had to buy water. Which was also one of the difficulties, we had to buy water. But if we bought the water today, they wouldn't deliver it now, tomarrow. It would take two days until they deliver the water. That was one of the most difficulties at that time.
I: Can you tell me any difficulties in storing water?
R: Nothing. Everything is okay when it comes to storing water. But the thing is, we don't have enough to storing water. That's the only problem. Everything is okay for storing water but it's just that we don't have any more tanks to store more water. Well in this family, we have a small family and I just don't believe that our tanks would easily run out but since the time when it took a long to rain, we ran out.
I: Can you explain the ways that your family tries to make drinking water safe?
R: That I told you, that the water tank is very secured. Since that time the EPA put filters and cleaned it. When it rains we don't just let it filled just yet. We let the rain pour first then we would connect it to the water tanks. Our kids only drink water from PPW, but when comes to us and our parents, we would drink from the water tank.
I: Let's now discuss hand washing. Could you describe in detail you family's hand washing throughout the day?
R: My children would wash their hands befor they eat. We usually wash their hands for them, theres soap in the bathroom but we use the hand soap there in the kitchen. We would wash their hands all the time. We're talking about the kids. We only wash their hands in the kitchen only. But us adults we wash our hands in the bathroom. When it comes to the kids that's why we put a hand soap in the kitchen for them.
I: Can you tell me about hand washing throughout the day for children under 2?
R: I usually wash their hands, still no differnce. I would wash their hands, use soap, and handsanitizer. I would also use the handsanitizer where ever we go. There are handsanitizer in our room, in the kitchen, and on the table.
I: Can you tell me about times during the day when soap is used to wash hands?
R: That's what we would do, you see the hands soap in the kitchen we would also wash our kids hands before the eat their snacks. Also we would wash their hands before they eat but it depends on what they have touch and where they have been. Like if they ever touch the ground, guarantee we will wash their hands. But it really depends on how their dirty like if they play a bit we would give them handsanitizer.
I: Can you explain what you think the difference between using water only or water and soap to wash hands?
R: Haaa, there's a big difference. Like for me and my thoughts, there's no point of washing your hands if it's just water. Because, gaurentee there will still be bacteria. The way I see there's no point on washing hands if we're only using water.
I: Can you tell about anything that prevents washing hands with soap throughout the day?
R: The thing is I really don't know. But when it comes to the people who lives here I really don't watch how they wash their hands. So I don't know if they wash their hands with just water or water and soap. All I know is my kids.
I: You are doing a great job providing every detailed answers. Thank you. Now, could you describe he type of toilet that you have at your home?
R: Oooh, regular toilet bowl. Toilet bowl.
I: Can you tell me reasons why you have this type of toilet versus other types?
R: I don't know. Maybe because i think every house is using this type of toilet.
I: In some communities, we have heard that defecating in the open is common. Could you help us to understand this practice, including how common it is?
R: If it was this town, yea sure it still is common. Not just here but everywhere, if you go from the end of Alwal to the end of Laura. But here in rita most of the weeks when you go at the ocean side just to hang out you can smell the defecate. It really stinks and the truth is we really don't know where it's coming from. How can I explain it, maybe because there are still people here doing it.
I: Can you tell me reasons why this practice exists in some places but not others?
R: Okay, I thought about that once and the reason why is there isn't enough restrooms to use. Like some houses they don't have any toilets. Like this town it also has that problem, but if not just that, there are some toilets that the salt water doesn't get to so that they can flush. Those could be the problem. If not, there are some people that I would ask why would they keep on defecating the ocean side? They would say, I'm not use to using toilets. I'm only use to using our ways back then.
I: Can you tell me the barriers to using the toilet?
R: Barriers, there's no barriers. But like I said, there are houses that the salt water doesn't reach to the toilets so that it can flush. That would be the problem. But here in this town some houses are jus to close to eachother that people are embarras to go to the bathroom so that would be the problem why they choose the ocean side to defecate. That's what I think.
I: Can you explain how young children's stools are typically disposed of?
R: About that, there are trashes here to throw away their diapers and then we would take out to the trash pins. Because, you know this town. You would see a bunch of dogs and you know these dogs would carry around diapers. I saw this before so I don't just throw around my daughter's diapers. Like if my daughter would poop, I would quickly change her and take out and throw it in the trash pins. But if it was just pee, I would just throw it in the trash.
I: Can you explain where your young children usually play each day?
R: They mostly play in this house. Sometimes they would go outside and play. Let's not say sometimes, but every evening. Every evening they go outside and play. Just outside of this house.
I: Can you tell me what an ideal play area for children looks lik and reasons why?
R: When it comes to my kids and we would tell them, oh, let's go to the airport you know where theres a park there. They would get excited. When I look at it, that's the only place they like to go play at. You know where there's playground at. Like the at CMI. But the place they mostly like to go is at the airport. It seem like that's the only place I like to take them to and play with them. There's nowhere else.
I: Can you tell me whether children play in areas where animals are kept?
R: Well there's no animals around the house. But there are sometimes dogs.
I: Can you explain the challanges of keeping a child's play area clean?
R: If it was here in the house, there's nothing. But outside, only the mud that concerns me. But they only play on grass. Oh, the other problem is the dogs feces. That's the other problem we would quickly throw it away. When it comes to everything else, it's fine.
I: To wrap up our questions on hygiene, could you explain ways to prevent the spread of disease?
R: Well in this house, washing the dishes is important. There are bathrooms in each room, so we keep it clean, we would use pinesol to clean with. These are the most two things we would prevent it from getting dirty. Most of all washing dishes is the most important part... We would prevent flies from getting on the food because if a fly goes on the food we just throw it away and cook another. Also the kids here would share, like some kids would come over and play outside and my daughter would come and get a gallon for them to drink. I would seperate a cup for her to use for herself.
I: Can you explain what you think of the connection between exposure to feces and illness?
R: ... Well I know that feces are good for planting. But I don't know, oh yes. The feces do cause illnesses. I would know that because when there's flies they would go on the feces and when they come to our food I would throw away the food. It's really nasty and bad if we would of eat the foods that the flies landed on.
I: We are also interested in the roles and responsibilities driffent family members play in raising children. Could you describe the care of children thoughout the day in your community?
R: In this house our father is old, really old. Our mother, is old. Our niece is in grade 12. The reason why I quit working is because I needed to look after my yougest daughter. Also because our babysitter flew to the states. Like, example for today. If my daughter wants to go with her grandfather in the room, there she'll just go to her grandfather. I'm the one who actually looks after our daughter while my wife is at work then when she comes home, she'll take her turn to look after our daughter.
I: If it was your community, how would they look after the children thoughout the day?
R: When ever the childrens comes and play there would also be adult supervision. Like, it would be me and the father of one the child. We would just hang around and talk while looking after the childrens. Like if there were kids outside, my kids grandmother would be outside looking ater them or me. Like let me give an example on how we look after them. We would like, make sure they don't play near or on the street, make sure they don't fight, you know kids. We would make sure they play nicely with each other, like make sure they don't take something that it's not theirs and fight over it and we would make sure no one cries. The only unsafe place is the road and we make sure none of them go play near it. The kids know how to look after each other. Like our youngest daughter would go play and when our our eldest one see her playing she would go and play with her and watch after her.
I: Can you tell me who is mainly responsible for child care?
R: In this house I'm responsible for our children's care. I mean like they are children.
I: Can you explain the responsibilities of mothers in child care?
R: Well, when it comes to my children mothers responsibilities, ... she would take responsibilities in the evening. If it was me, I would be responsible from the morning till evening. Since I'm father I would look after them while their mother is at work. I make them food, look after them, clean them, no different in how a mother looks after their children. And when their mother comes home in the evening. She knows that she would feed them, clean them, prepare them for bed, and wash their clothes.
I: Can you explain the responsibilities of fathers in child care?
R: I would look after my children like every every day. At first I thought looking after a child would be so easy, but the things I would do is more like a mothers responsibilities. But since I'm a father, my responsible should be what their mother is doing now. I would make foods for them and give bath, like I was gonna give my daughter a bath when we got here.
I: Can you tell me how caregivers play with children under 2?
R: Well we would play all sorts of stuff with them. Play volleyball, you see all these toys, we would play with these toys with them. There are sometimes I would take them out and watch them ride their bikes. These are the only things I would play with them, play volleyball, play with balls, throwing the ball and catch it, bycicle. But when there's other kids every kind of games is fun with them.
I: Could you talk aout the role of grandparents have in raising children in this community?
R: Nothing, everything is good, everything is good... The only thing is the grandparents would spoil their grandkids. So we would observe them and watch them because they would buy a lot of toys, they would feed them junk foods, these would be the things we have to watch out for. Like when the kids would choose something they would say okay. So we keep our eyes on them because they would spoil our children way too much. And when their grandchildren would say this and that, they would say oh yea.
I: Can you explain the ways that grandparents support in raising children?
R: There's a lot of ways they support us. Because sometimes we would be busy. Like for me since we have two children, one would go with their grandparents while I'm feeding the other one. But when I'm busy with cooking food, guarantee the grandparents would take them outside and play with them. They would would also feed them when I'm busy. It really helps a lot when their around.
I: Can you tell me what makes a good grandparents?
R: From my prespective, it's their choice if they want to play with our kids or watch over them. But I know that when I'm busy they take them and keep them busy while I finish up with my chores. Like if they were watching they would take them and let them watch.
I: Can you explain how the grandparents are good?
R: Like I told you before, I'm here because I followed my wife here and we're living under her parents roof. There's nothing I would tell them or show them how to be good, because they have their own consciousness on how to be good. And it depends on themselves on how they wanna be good.
I: Can you explain the ways that siblings (older siblings) help raise young children?
R: Our eldest daughter, because she's still young she doesn't have to help us raise her. She only watches her, like you see these stairs, she would watch her little sister from falling. 
I: You are doing a great job We are almost finished. Now for the las section, we would like to learn about ways we can develop health programs in your community. Could you explain where you usually get trusted information about nutrition and health?
R: From me, I would listen to my wifes parents telling me I should feed our children this I should feed I children that. I would take some advices from them. Like in this house our parents would teach us how to cook the food and how to make the food. 
I: You said you would listen to your wifes parents about nutrition and health. Who did they get the information or where did they get the information about nutrition and health?
R: Well I don't really know who or where they get their information. But I believe it's because they're older and they know more. And there are times we would ask from them because they've been through this kind of situations. Like when we try to feed our children and they don't eat it we would tell our parents about it and they would tell us to cook the food they tell us to cook then next thing you know our children eats. But when we go to the hospital, the doctors would tell us some information about nutrition and health. We would do what ever the doctors would tell us to do to keep our children healthy.
I: Can you tell me where nutrition and health messages should be delivered so that you would see/hear them most easily.
R: You know how you guys move place to place, it's really good. Make some posters and make some signs around. They were better. Also radio program was good. Now, espicially the  schools, there should be people going around schools giving them information.
I: Can youand  tell me the types of media that you use the most to communicate?
R: I would take some advice on how parenting should be with my wifes parents when there's time when I don't know what to do. Like if it was my youngest daughter, I have a older sister who works at MCH at the hospital who knows about children. She's usually the one who we would call and ask some information on how to look after our child. But if they didn't know about anything on parenting I would have probably gone to the hospital and ask.
I: When you think about your own parenting behaviours, can you explain what influences how you raise your children?
R: From me, it's really important for me to look after my children. There shouldn't be... late dinner, they should be washed up and clean, I got to make sure they don't eat to much junk foods, I would make sure they drink plenty of water. Because I'm a father I should be also teaching them on how to wash their hands and make sure that they eat at the right time.
I: Can you explain on how opinions of the community influence how you raise your children?
R: If it was this town, I haven't ask any opinions from anybody. But when I see on how they look after their children I see no difference on how they look after their childrens. Their children would play outside then the parents would call out to them saying, come home it's time for shower and dinner.
I: Can you explain any advice or information related to parenting you received?
R: ... Yes, there were plenty. Like we shouldn't do anything bad infront of them. Because if we let them see bad things happening around them, they might as well do the same. We shouldn't put any anger on them. We should stay open to them because if they needed something they would come to us. yes, there were a lot of things I've learned like, how to feed them, how to make them food and how to play with them. I know what's bad for them and what's good for them. I would try to keep them from doing what the others kids would do, like, when they see other kids playing in the mud I would tell them that the mud is not a good place to play in. Also if they want to play under the rain I would tell them that they will get sick.
I: Now can you tell me where/who the advice or information came from?
R: I got them from around the house.
I: Can you tell me about any desired information on parenting you wished you had but is not available?
R: Yes, there are plenty. Like how a birthday should be for a child, when they have a fever and we have no way to the hospital what can we do to lower down their fever. That's it.
I: Is there anything else?
R: Like what kind of foods they should be eating. Like what they should eat for breakfast, for lunch and for dinner.
I: Alrighty then, we are all done, thank you for your information these information you have given to us are very important. Is there anything else about the topics we talked about today that we missed or that you would like to tell us about?
R: You know what you guys are doing, you guys are doing great. I hope you guys can make a workshop at schools to show them on how to look after children, because nowadays people don't know that much on how to look after children. That's all.
I: Again thank you.
