# Supplementary material for: Conceptualizing multi-level determinants of infant and young child nutrition in the Republic of Marshall Islands–a socio-ecological perspective
Source: PLOS Glob Public Health. 2022 Dec 19;2(12):e0001343. doi: 10.1371/journal.pgph.0001343 (PMC10022247; doi:10.1371/journal.pgph.0001343)
Supplement: S1 Data — (ZIP) [file pgph.0001343.s001.zip › RMI Supp Data/Interviews data/I22U_IDI_FCG_Rita_Aug 15_Meia.docx]

- Interview code: I22U
- Interview type and interviewee: IDI_FCG
- Interview date: Aug.15.18
- Location: Rita
- Interviewer: Meia
- Transcriber: Marcellina Timus

**I: okay thank you. Now do agree to proceed with this interview with me this morning?**

R: yes.

**I: thank you again for giving me your time to speak with you today. Okay to begin with, can you please tell me a little about your family?**

R: like?

**I: you may not mention their names but who live in the household, how many children and their ages and gender**

R: well our parents, and there are 8 girls and 4 boys

**I: now could you please tell me a little about your community? Like what are the good or the bad things about your community.**

R: the good thing about my community is it’s breezy, we don’t hear and see troubles anymore, it is safe, like my house has fences around it.

**I: good. Now what are the bad things about your community?**

R: the bad thing was, there were always troubles occurred

**I: can you tell me more about troubles?**

R: troubles like fighting, car accident

**I: now let’s talk about health and illnesses in your family. Can you tell me about some of the illnesses that your children have suffered from?**

R: there are none

**I: none? They don’t get sick at all?**

R: no

**I: Now can you describe how do you know when your children need treatment if they ever get sick?**

R: when the illness gets severe

**I: can you tell more details about how severe?**

R: fever for example, when the temperature gets higher. And, when they really don’t want to move around at all.

**I: now, who do you first go to when your child is sick?**

R: the nurses. But sometimes my family along with the church(mormen) we seek the pastors first so we can do a prayer with.

**I: now I am asking you as you said they do a prayer, why?**

R: for blessing and to reduce the effects of the illness

**I: do you use any traditional medicines?**

R: no

**I: okay can you describe any illnesses affecting your children that are associated with nutrition?**

R: none. I haven’t seen any illnesses in them

**I: okay we talked a lot about being unhealthy. Could you now describe for me a typical day of someone living a healthy lifestyle, from the time they wake up in the morning until when they go to bed?**

R: when they wake up in the morning, they go brush their teeth, wash their face, have their breakfast, and then take a shower. After that they will go to play and come back to have their lunch at lunch time. So many things they do! They sit down, stand up, walk around, playing around, and as the sun staring to set down, they will go play volleyball and chat with friends. Before they go to sleep, they will take a shower and have their dinner.

**I: you are doing good thank you. Now can you tell me the appearance/signs of a healthy child under 2 years?**

R: they wake up early in the morning, have their breakfast, pooping, and take a shower. After that, they play alone with our attention. They also eat foods frequently.

**I: Now, can you tell me the appearance/signs of a healthy adult?**

R: a healthy adult. When he/she does the household chores, he/she will do it until it’s done. He/she loves to walk and exercise, love to chat in the public with strangers, and when they rest they don’t rest for too long because they need to do more works.

**I: Let’s now discuss hand washing. Could you describe in detail your family’s hand washing throughout the day?**

R: throughout the day… well as for me I wash my hands before and after I eat, after I used the rest room, before I cook the meals, after I am done with my household chores, before I touch my baby

**I: that’s wonderful but can you tell me how do you wash your hands?**

R: well I wash my hands with soap. I rubbed the palm of my hands, between my fingers, and all the way up to my elbows.

**I: okay. Now can you tell about children’s hand washing throughout the day?**

R: I recommend them to wash their hands with soap too or sometimes they use hand sanitizer.

**I: now can you tell me how children under 2 years wash their hands throughout the day?**

R: we wash their hands using wet towels

**I: now I am asking, what times during the day your family use soap to wash their hands? Like do you wash your hands with water and soap or do you just wash it with water alone.**

R: yes, sometimes we wash our hands with water only but sometimes we wash our hands with soap.

**I: now can you tell me when do you use the soap?**

R: after I used the rest room, before and after I eat, and when my hands are dirty

**I: now can you tell me the differences in washing hands using water only and water with soap?**

R: when we wash our hands using water only we think they are clean. But when we wash our hands using water and soap, there is a different feeling right there. Our hands are slippery because of the soap. But after we rinse our hands, they feel smooth.

**I: okay. Now what prevent you from washing your hands with soap throughout the day?**

R: how can I say.... keep forgetting things instantly. Like for example, I planned to go wash my hands with soap but when something came up and I am in rush, I will just wash my hands with water.

**I: Now we would like to talk about your diet during pregnancy and breastfeeding. Now I would like you to think back to when you were pregnant. Can you describe your diet when you were pregnant compared to when you were not pregnant?**

R: when I was pregnant, I ate variety of foods. I ate bread--every food that are made from flour. for meat, I like my meat to be not fully cooked. And when it comes to fish, I always preferred sashimi. I rarely preferred fried fish. And for can meats, I usually eat one whole can meat alone!

**I: what about now that you are breastfeeding. What kinds of food you like to eat?**

R: I eat chicken, can meat but I share with other, papaya, and banana

**I: okay. Now what made you starve for these foods when you were pregnant?**

R: I just saw them and want to eat them. But I will be mad when my family won’t get them for me.

**I: now, what kinds of food they encourage you to eat when you were pregnant?**

R: they never told me what foods to eat

**I: how about what kinds of food they encourage you not to eat when you were pregnant?**

R: they didn’t tell me that too

**I: now, who took care of you or supported you when you were pregnant?**

R: the nurses

**I: can you tell me in detail how did the nurses supported you?**

R: they helped me with all my checkup appointments.

**I: okay so can you tell me about any supplements you took during pregnancy?**

R: I took the vitamin bills and the other bills which are for helping produce blood.

**I: can you tell me did you took all the supplements given to you and the reasons why you took them?**

R: yes, I did. I took them because the nurses said it’ll help me and my baby a lot with vitamins and from lack of blood.

**I: can you tell me did you drink alcohol, smoke, or use any other drugs when you were pregnant?**

R: no… I didn’t drink, smoke, nor used any drugs. But one thing I did, and I am still doing it is chewing tobacco(grizzly)

**I: okay. Can you tell me did you used any traditional medicine during pregnancy?**

R: no, I did not

**I: If someone told you to eat only fruits during your pregnancy, what made it impossible to follow through with it?**

R: nothing. you know I am food lover. during my pregnancy I ate mostly anything. If you would give me food, no matter what kind I would take it anyway.

**I: what type of foods you ate when you were breastfeeding?**

R: mostly fish…as well as chicken, hot-dog, rice and canned meat.

**I: what made you eat these foods when you were breastfeeding?**

R: because I’m hungry

**I: what type of foods you were told to eat when you were breastfeeding and reasons why you ate them?**

R: when I first breastfeeding, I ate the meals provided in the hospital. But other than that, I mostly ate fish because it helps produce breastmilk.

**I: what type of foods you were told not to eat when you were breastfeeding and reasons why you were told not to eat?**

R: raw fish like sashimi since they claim my baby will bit me. That’s the only food they told me not to eat… sashimi

**I: who really advised you and helped you to make sure you eat or don’t eat all these foods when you were breastfeeding?**

R: my husband and my mother

**I: after you gave birth can you explain how you first get your baby to breastfeed in one day?**

R: after they cleaned my baby, they wrapped my baby in a blanket and brought her to me and they told me to feed her first since she’s hungry. When I saw my baby, I was really happy and excited. I immediately sit up and just looked at her. I gave her the left breast first, but before I thought she was staring to sucking up milk, her mouth just closed like she just doesn’t want to have her breastmilk. So, me and the nurses tried to make her breastfeed until I got her standing up and that’s when she opened her eyes. When I tried to breastfeed her again, it didn’t take too long for her to start sucking up her breastmilk. She really loved to be breastfed that she won’t stop until I pulled it out.

**I: did you give bottle-milk or any other liquids in the first few days after giving birth?**

R: no, I didn’t

**I: were there anything made it difficult or easy for you to breastfeed your baby?**

R: there are none. Everything was just fine.

**I: can you tell me when did you first gave foods or liquids to your baby other than breastmilk?**

R: when she was 6 months old

**I: can you tell me the reasons why did you give her foods or liquid at that time?**

R: there was this time I brought her back to the hospital for her second appointment. and when one of the nurses was checking up on her, she was like opening her mouth to whatever comes near her mouth. So, the nurse asked me if I have start giving her food. I said no I haven’t. she told me that I can give her food now that she’s 6 months old. So, that is why I started give her foods and liquid at that time. I was encouraged by the nurses and it shows in the baby chart what food should be given at different age.

**I: now this question, what are the opinions of others which influence their decision to start giving foods or liquids at that age?**

R: when they come over to my house and see me feeding her, they said “wow! Sooner there will be no more foods left because she will eat them all”.

**I: now, what were the first foods you fed her and how did you prepare them?**

R: the first food I fed her was bread. I bake it myself and boil water and let them cool down for a while. When I gave her first bite, she didn’t refuse which I thought she would have but she ate all of it.

**I: now, can you describe in detail how did you prepare the bread?**

R: I just added water and mix it with the flour and bake it. I didn’t add sugar.

**I: your answers are great. Okay, we are trying to understand how people eat in this**

**community. Could you describe in detail what your family usually eats and drinks throughout the day?**

R: in the morning we buy us bread for breakfast and drink tang (Kool-Aid). The kids usually drink water. for lunch, as usual rice with chicken. For dinner, most of the time we eat fish with rice.

**I: now, how do prepare the lunch and the dinner foods?**

R: I prepare them before the meal time and everybody eat at the same time

**I: who is the family is serve first, next, and last?**

R: the first to serve is our parents, next the older ones, last will be the girls

**I: are there any differences in the foods served to your different family members?**

R: there are none. Everybody will have the same

**I: are there any differences in the amount of food served to different family members?**

R: yes, there are. Like our parents, they will have two pieces of meat but all of us will have one

**I: are there any children received more food than the others when you served them?**

R: no, they will all have the same amount

**I: Now could you describe any food sharing between family members during mealtimes (for example children eating together separately from the family, meals eaten from the same plate by all family members)?**

R: everyone eats with their own plate

**I: We have heard from some families that eat local foods whereas others eat processed foods. Could you explain what is typical for your family?**

R: we usually eat processed foods.

**I: can you tell me what makes it difficult or easy for you to cook local foods?**

R: in my place, we don’t have any local foods around. The only place that have local foods are the stores rural area. That’s the difficulty.

**I: what are the good or the bad things about eating local foods?**

R: the good thing about eating local foods is they are healthy. The bad thing is we don’t have in our place.

**I: okay this question is asking, what are the good or the bad things about eating processed foods?**

R: eating processed foods depend on our money and that’s the negative side of it. The positive side is they don’t take too much of our time to cook them not like local foods.

**I: Now that we’ve talked about how the family eats, I would like to learn more about how your child eats. Could you describe in detail what your son/daughter under 2 years commonly eats throughout the day?**

R: as for my daughter, she commonly eats baby foods, oatmeal, cereal, biscuits, meat, and rice

**I: now can you describe how many times in a day she eats including her snacks?**

R: she eats in the morning, lunch time, dinner time, and have her snacks in between the regular meals time.

**I: how do know when your daughter has enough foods to eat or she’s full?**

R: she just doesn’t pay attention to her meal anymore. That’s how I can tell she’s full.

**I: what would you do to encourage your daughter to eat?**

R: make her distracted

**I: what would you do to encourage your daughter to eat if she refuses?**

R: I will breastfeed her if she refuses to eat

**I: are there any differences in feeding your daughter when she’s sick? Like for example if she has diarrhea and the reasons why.**

R: when she’s sick they don’t eat much

**I: okay you’ve told me what your child under 2 usually eats. Now could you explain to me the process, from start to finish, how you prepare and cook a meal for your child?**

R: bread for example, before I start bake it I will bring all the ingredients I need and then combine them all together and then bake it. Before I feed her what I cook for her, I will let it cool down first.

**I: Could you now tell me what are the important foods for your child to grow well/be healthy?**

R: those foods that contain vegetables and fruits like baby foods which she usually eats.

**I: okay, now can you tell me what kind of foods should you not give to your child?**

R: junk food like lollypops, chips because they are sweet

**I: what is the biggest influence in feeding your child?**

R: she has different time to eat different types of foods including snacks

**I: Can you describe any differences (if any) between how you feed your son and how you feed your daughter?**

R: the different between them is, my son eats all by himself but my daughter, I am the one who feed her.

**I: okay we are also interested in the roles and responsibilities different family members play in raising children. Could you describe the care of children throughout the day in your community?**

R: my neighbors for example, when they see that the mother is tired they will take her baby and look after her baby, so she can rest for a while. Me, I will just cuddle with my baby until it’s time for her meals. When she’s done with her meals, she will play with the other kids while I look the undone chore around the house.

**I: now, who is mainly responsible for the child care?**

R: the mothers

**I: what are the responsibilities of the mothers in taking care of her child?**

R: they are responsible in keeping them safe from any dangers, sicknesses, and make sure that don’t play in the dirty area. Above all, teach them to be loved.

**I: now, what are the responsibilities of the fathers in taking care of his child?**

R: because they are the head of their family, they are responsible in taking care of all the family’s needs. They support their family. Also give advices to their children when they do wrong and teach them to be loved too.

**I: how does the caregiver like you for example, play with their children that are under 2?**

R: we cuddle with them, kiss them in the cheek or in the forehead, make them laugh like we’re sharing our love with them.

**I: now, can you tell me the roles or responsibilities of the grandparents in taking care of the children in this community?**

R: they also help in taking care of our children

**I: can you tell me what makes them good grandparents?**

R: they care and love our children so much. For example, if we shout at them because were mad at them, they will take them from us and comfort them.

**I: Could you talk about the role that other family members have in raising children in this community?**

R: they also take care of their children the same way we do to our children.

**I: okay. So how do the older siblings help in raising young children?**

R: they give advices and set good examples for the children.

**I: You are doing a great job. We are almost finished. Now for the last section, we would like to learn about ways we can develop health programs in your community. Could you explain where you usually get trusted information about nutrition and health?**

R: number one is hospital like the wellness center, in school, and in the community

**I: can you give me the reasons why these sources are trusted?**

R: because it is where we usually hear the information from

**I: where do think nutrition and health messages should be delivered to so that you would see/hear them most easily?**

R: in the radio stations and in the newspapers

**I: When you think about your own parenting behaviors, can you explain the influences on how you raise your children?**

R: there are no influences

**I: now, are there any advices or information related to parenting you have received?**

R: yes, there are. They told me really take good care of my children and don’t abandon them. And give my attention to them especially. Always feed them food and don’t let them hungry.

**I: who gave you these advices or information?**

R: my mother, my older sister, my husband’s older sister, and my husband.

**I: now, are there any information about parenting you wish to learn or know about but are not available?**

R: well yeah, I know about parenting, but do you have any information to help me with?

**I: well if you wish to know more about parenting, you can get more information from the health center and mrs. Caroline and mrs. Helia in the family planning center. Once again thank you so much for your time and your useful information.**
