# Supplementary material for: Conceptualizing multi-level determinants of infant and young child nutrition in the Republic of Marshall Islands–a socio-ecological perspective
Source: PLOS Glob Public Health. 2022 Dec 19;2(12):e0001343. doi: 10.1371/journal.pgph.0001343 (PMC10022247; doi:10.1371/journal.pgph.0001343)
Supplement: S1 Data — (ZIP) [file pgph.0001343.s001.zip › RMI Supp Data/Interviews data/I23U_IDI_FCG_Rita_Aug 15_Meia_Fela edited.docx]

Interview Code: I23U

Interview type and Interview: In-depth Interview

Interview Date: August 1 2018

Interviewer: Meia – Female Caregiver

Transcriber: Fela

**I: Do you agree in doing this interview this afternoon?**

R: yes

**I: Thank you for giving us your time to speak with me today. The information we learn here will help us find ways to improve maternal and child health and sanitation in your country.**

**I: To being with, can you please tell me a little about your family?**

R: no answer from respondent (people talk at the back)

**I: like who live in this house, how many female child or male child live in this house?**

R: like what?

**I: you don’t have to tell me the names, you can just tell me who live in this house**

R: in this side right here, it just me, my husband and our child. That lady over there together with her three sons live in that side. Just in this house but different side that has been divided for my family and that lady and her three sons.

**I: ok**

R: this house is part of that house, and my mother in law lives in that house together with her grandchildren.

**I: is there is any man live in that house?**

R: that man is younger than my child’s father. And his wife. Just them.

**I: ok. Now how old is your child?**

R: one years old

**I: one years old ok.**

**I: Now, I’d like to ask you to describe your community?**

R: no answer

**I: like what are the positive or negative things that you see in this community?**

R: long pause. (Silent)

**I: was the question understandable to you?**

R: it confusing

**I: you can just tell me everything or just anything that you see happening in this community. We won’t know who.**

R: still no answer.

**I: what are something that good or bad that you see around you?**

R: there is none

**I: ok. Let’s now talk about health and illness in your family. Can you tell me about some of the illness that your children have suffered from?**

R: fever

**I: fever ok. The child usually have fever, now what is the cause of fever?**

R: when she usually sleeps in cool-air or when the air condition is on and it’s cool inside the room, which is the reason why she get fever sickness. If I leave her sleep in a long time in cool air, she get fever.

**I: what else?**

R: windy air

**I: ok. Also from windy air. Anything else that cause her to have fever?**

R: there is nothing else. But sometimes when we leave her sleep there, outside and it’s too windy, she would have fever.

**I: ok**

R: We even passed out from her even though we say that we would limit our night sleeping outside but we would passed out from her anyway. When we wake up, it’s already day time. And when we check her, we find out that she got fever.

**I: now do you see that fever is a serious sickness?**

R: it also can be serious for children.

**I: now can you tell me why you said that fever is a seriousness illness?**

R: because when their fever get too high, they can have asthma and the can be stuck or can’t even move.

**I: how would you prevent fever illness?**

R: cool down a piece of cloth and massage the cloth in her body so that the cloth can be suck out fever inside.

**I: ok**

R: when we do it urgently and cool down the fever, the child can be heal right away, but when we don’t do anything about the illness, it can cause the child to have asthma.

**I: ok. Anything else that can help prevent fever?**

R: nothing else. The only thing that I can do is rinse a piece of cloth with water and use the piece of cloth and help prevent fever

**I: Ok. Now can you describe how you know when your child needs treatment for illness?**

R: when we find out that the child have illness, and these illness are really serious, I take them to the hospital. We would realize that they have illness.

**I: ok**

R: the first time she got sickness, I brought her to the hospital and they found out that she needs to be in the hospital.

**I: can you tell me in detail what kind of illness that cause her to be in the hospital because there are lot of kind of illness?**

R: she had malnutrition. As you can see that she have only few hair, look at her.

**I: ohh**

R: she kept losing her hair and when they checked her, they found out that she had malnutrition illness. She refused to eat, she couldn’t stand alone.

**I: ohh**

R: she was really weak. They didn’t treat her with IV, but the only thing they did was treated her with medicine. From their treatment, she was able to eat. I can’t remember how many weeks she couldn’t eat, it took too long for her not eating her foods.

**I: hmm**

R: she was suffered a lot.

**I: it was like that when she force herself not to…**

R: not to eat.

**I: until she had the illness?**

R: when we tried to feed her, she refused to eat. That is the reason why we had to bring her to the hospital, we realized that she couldn’t eat her food and we didn’t really know what was happing.

**I: she didn’t have illness right? But the problem was that she refused to eat?**

R: when she stand on the ground, she couldn’t be able to walk.

**I: ooh ok. How old was she when she was like that?**

R: now that she is one year old, I can’t remember what month was that. I think it was last May.

**I: how long she spend inside the hospital?**

R: one week

**I: just one week and she got out from the hospital. Now you can easily tell that she fine now?**

R: yes

**I: ok. Now who would be the first person you would go to for healthcare and reason why?**

R: no respond

**I: for example, they usually bring their children to the traditional healer. (Giggling, “it’s just an example”) But the question is asking, who would you first go to for health care when the child is sick? And reasons why you would bring the child to the person?**

R: long pause (No respond)

**I: was the question understandable to you?**

R: no respond

**I: is there just someone that you would bring your sickness child when the child is really sick? Who would be the first one that you bring the child to when the child is sick?**

R: No respond

**I: or do you use traditional medicine?**

R: no respond to the interviewer (talking to family member) “Mitchel go bring me the child” “I think that’s the person that you hated” (Woman giggling)

**I: Any traditional medicine you used to use?**

R: for the child?

**I: yes**

R: I usually bring her to her grandfather so that he can do stomach massage. That old man is my mother’s father.

**I: ok. Stomach massage.**

R: before she got into the hospital, I brought her to that old man so that he can massaged her stomach.

**I: Ok. Stomach massage.**

R: he massaged her stomach and she was healed from her illness.

**I: so you meant that he was the first person that you brought her to when she was sick? You brought her to the traditional healer. Ok that’s great.**

**I: Now can you describe any illness affecting your children that are associated with nutrition?**

R: uh?

**I: this question will apply to you. Can you describe any illness that affect your children that are associated with innutritious?**

R: no answer*

**I: let’s skip to question number six. We talked a lot about being unhealthy. Could you now describe for me a typical day of someone living ha healthy lifestyle, from the time they wake up in the morning until when they go to bed? Can you describe how a healthy person live in a day, from the time the person wakes up until they go to bed, how can you tell that the person is healthy? What do the person do throughout the day so that you can say that “oh that person is healthy?”**

R: they move a lot and do works.

**I: yes**

R: that person is not feeling lazy, they do lot of movement. They always do works around the house.

**I: hm. Ok. Now what are the appearance or signs of a healthy child under tow year old? What are the signs that’s you see that the child is healthy, what do the child do throughout the day?**

R: the child is not crying, he/she only play and not crying

**I: hmm**

R: the child also do lot of movement

**I: hem**

R: the child also don’t sleep all the time but would play all day.

**I: ok. Now what are the appearance or signs of a healthy adult? What do the adult do so that you can tell that they are healthy?**

R: the adults?

**I: yes**

R: like what? Adult that are?

**I: that looks healthy.**

R: like the person that is not lazy, they do lot of movement and house chores. When they do lot of work, we can tell that they are healthy. They only move, they don’t sleep but they always like to do works. That’s why we can tell that they are healthy. They don’t relax their body but they do lot of movement throughout the day. They work each and every day. They can do works from morning until night time. They can just move all day. Like my mother in law. She can do lot of movement and do works around the house from morning to night time. Even when she do cloth hand washing, she can do that from morning until night time.

**I: hmm. She is just a hard working woman**

R: she did laundry by hand washing yesterday under the rain without feeling tired.

**I: ok. Let’s now discuss hand washing. Could you describe in detail your family’s hand washing throughout the day?**

R: “baby talking to the mother” mother talking to baby “ah where is daddy? Long pause. (Baby crying) this child is crazy. Go with daddy hurry. Mother- Gibson? Daddy: yeah? *conversation between mother, father and the child*

**I: Now this question is asking about hand washing. It’s asking you to describe how this family wash their hands throughout the day?**

R: how can I explain that? For example for me and my husband.

**I: hmm**

R: We can go around the house or somewhere else, and when we come back, we wash our hand using the hand sanitizer. We now usually use the hand sanitizer

**I: hmm. Do the children also wash their hands?**

R: yes they also wash their hands too. They also use the hand sanitizer.

**I: and what about children under two years old. Do they also wash their hands?**

R: yes. That old lady in that side, my mother in law, usually the one to wash their hands. Even before they drink, they have to wash their hands.

**I: Now what time during the day when soap is used to wash hands?**

R: can times before they touch the meal dishes. They have to… the old lady bring all the children together and wash their hands using soap them after would use the hand sanitizer after.

**I: now...**

R: lady in background shout really loud (Respondent talking- that’s the crazy lady she doesn’t know we are doing recording uh?

**I: Can you tell me the difference between using water only or water and soap to wash hands?**

R: family member talking at the back (Respondent silent)

**I: was the question understandable?**

R: I don’t know how to answer that question (Giggling)

**I: It says, is there is or can you describe the differences when you wash your hands using water only or by using water and soap?**

R: describe the what? Differences?

**I: yes**

R: the differences between using soap and not using soap is when we don’t use soap for hand washing, there will be Bacteria and viruses in our stomach. But when we use soap, we won’t have viruses or bacteria in our body especially in our stomach. When we use only water, the germs affect our body.

**I: ok that’s great. You answer is perfect. Remember that there is no right and wrong in your answers. All the answers that you are sharing with me are great and don’t worry when you can’t answer these questions. Just give your answer as possible as you can.**

**I: Ok now what prevents you from washing your hands with soap throughout the day?**

R: hand sanitizer

**I: from using hand sanitizer**

**I: Now I would like you to think back to when you were pregnant. Can you describe your diet when you were pregnant compared to when you were not pregnant?**

R: fish that should be cooked on the fire.

**I: ohh**

R: I wanted the fish to be cook on the fire and I never wanted to eat it with rice

**I: ohh just the fish?**

R: yes just the fish but the fish should be cooked on the fire.

**I: hm that’s delicious girl**

R: that’s funny like pregnancy women are really funny because we are too picky on our foods. It was really funny that I never wanted to eat fish with rice, I wanted to eat fish only but my fish has to be cooked on the fire.

**I: every pregnancy women have different diet during pregnancy**

R: yes you are right. Some want to eat the mosquito coil.

**I: ok. Now what influenced your diet during pregnancy? What influenced you to eat these foods?**

R: baby talking (Mom whispering to the child)

**I: from your own opinion, what influenced you to eat fish during pregnancy?**

R: long pause.

**I: was the question understandable?**

R: * woman talk to family member*

**I: so on what foods you were encouraged to eat during pregnancy and reasons why?**

R: they encouraged me to eat a lot of fruits and vegetables and also eat fish but not drink coffee, if I want to drink hot water, I was encouraged to drink only tea.

**I: ok. Now why would they encouraged you to eat these kind of foods?**

R: long pause… they said that it should help giving the child good health. And also the child can’t have any kind of sickness. And these are the appropriate food that should be given for the child and me during pregnant.

**I: ok. Now on what foods you were encouraged not to eat during pregnancy and reasons why?**

R: like the salty and greasy goods. Fried foods and greasy foods or salty foods.

**I: Ok now why you were encouraged not to eat greasy and salty foods?**

R: so that these foods can affect my child

**I: Now can you tell me who encouraged or discouraged eating those foods during pregnancy?**

R: like what?

**I: who encouraged you or discouraged you eating those foods during pregnancy?**

R: oh yes the doctors.

**I: ok moving on to the next question. Who primarily cared for or supported you during your pregnancy?**

R: when I was in the hospital?

**I: During your pregnancy, who cared for or supported you?**

R: oh my husband and my mother in law.

**I: ok you answer perfectly to the question. But the next question is asking, how these person supported you during pregnancy? Like in what ways.**

R: they do my house chores that I used to do. They also can do laundry for me, bring foods for me

**I: ok. Now can you tell me about any supplements you took during pregnancy? If you took any supplement during pregnancy.**

R: just these supplements for pregnancy

**I: like what?**

R: vitamins and the blood pill.

**I: ok. Now why did you take any of these supplements?**

R: they said that I should take these supplements so that when the child born, she shouldn’t be feeling sick and all that. The child can’t be born skinny but chubby and healthy. And there shouldn’t be any problem for both me and the child during giving birth.

**I: ok**

R: and yes when she was born she was really chubby and big

**I: how many bound she was?**

R: seven bound

**I: hmm seven bound (Giggling) ok**

R: let’s say finished all the given supplements

**I: and were you able to finish them?**

R: yes I used to finish all of these given supplements and would return to my next appointment until I have birth to her.

**I: ok. Now can you tell me if you drink alcohol, smoke or used other drugs during pregnancy?**

R: I did not

**I: so were there any traditional medicines taken during pregnancy?**

R: there was none

**I: If you were advised to eat more fruits and vegetables during pregnancy, could you describe what would make this difficult?**

R: I never eat fruits during pregnancy I only ate the, how do you name the circle fruits

**I: tomato?**

R: grapes. That was the most delicious fruits that I wanted to eat during pregnancy. That fruits children usually called (kauroor) * Marshallese plant* I don’t know

**I: don’t worry if you can’t say the names of these fruits because these are not fruits from here. They are imported fruits.**

**I: Ok. Now we are in question number eleven. Now can you describe your diet when you were breastfeeding?**

R: I usually eat fish. They usually bring fish for me to eat.

**I: what influenced you to eat fish during breastfeeding?**

R: no respond

**I: why did you eat or liked fish during breastfeeding?**

R: to provide milk

**I: ok that’s the perfect answer.**

R: * talking to family member to look for the baby*

**I: Now can you describe your diet when you were breastfeeding? What kind of foods you were encouraged to eat during breastfeeding?**

R: I was encouraged to eat fish, banana, pandanus and any kind of local foods, or can be boil fish

**I: ok. Now on what foods you were encouraged not to eat during breastfeeding?**

R: salty foods.

**I: why did they encourage you not to eat salty foods?**

R: no respond

**I: they encouraged you not to eat salty foods can you tell me some reasons why?**

R: sot that the child’s health can’t be in danger

**I: Who encouraged or discouraged eating those foods while breastfeeding?**

R: the doctors

**I: doctors ok. Moving on to questions number twelve.**

**I: After giving birth, could you describe breastfeeding your child throughout the day?**

R: I just breastfeed her right away. I didn’t give her any other liquid.

**I: ok. And what about after giving birth, how long after giving birth you started breastfeed your child? Did you give other liquid or anything else?**

R: I didn’t give her any other liquid. My children didn’t feed from any other liquids, they never liked it. Even this girl right here, my baby she keep on feeding from breastfeeding.

**I: ok that’s great. Now could you tell me when you first gave foods or liquids than breastmilk to your child?**

R: when she was five months

**I: five months ok. Now why did you introduced foods or liquids other than breastmilk at that age?**

R: when I saw that she kept crying, I realized that maybe she was hunger for foods

**I: Now do you know any opinions from others that influenced their decision to introduce foods and liquids at that age?**

R: hm? In your opinion, do you know other people’s decision to introduced foods or liquids to their children?

R: I don’t know

**I: ok. No problem. What was the first foods that you gave to your child and how did you prepare the foods for the child?**

R: baby food. Am I right? You were asking about foods?

**I: yes**

R: yes the baby food

**I: that you bought it from**

R: yes the stores.

**I: We are trying to understand how people eat in this community. Could you describe in detail what your family usually eats and drinks throughout the day? Can you describe in detail what do they eat from morning during breakfast until dinner meal?**

R: they usually eat rice and drink water.

**I: ok. Rice and drink water.**

R: but some usually drink sweets drink or colour water. Like when they see someone drink sweet while they drink water, they would turn to the other person and say “oh I also want to drink sweet” and whenever I drink water, I feel like I am full already.

**I: you mentioned colour what kind of colour drinks?**

R: if it’s not the coffee kopiko, it can be the eight o clock tank, or not the kopiko coffee. I meant the white coffee.

**I: ok now how do they eat their breakfast? When they eat breakfast, it’s the right time for them to drink colour drink when they have bread for the breakfast.**

**I: ok now who in the family is served first, next, and last?**

R: the children them after the adults

**I: is there is any differences in the foods served to different family members?**

R: there is no, everyone has the same kind of food

**I: ok. So are there any differences in quantities of foods served to different family members?**

R: yes there is. There is because some eat more than the others. For example, if we eat pancake for breakfast, the person that eat less can eat only half of the pancake.

**I: do some children receive more food than the others?**

R: hmm. Children that eat more. We usually give more food for them. I always give them same quantity of foods and when the child that eat less see that the foods is too big for them, they can reduce the quantity of their foods. Because they usually eat sweet, when they eat their real meal, they eat only a little of their food. The fact is that, we usually feed them until they are full.

**I: ok now question number fifteen. Now could you describe any food sharing between family members during mealtimes (for example children eating together separately from the family, eaten from the same plate by all family members?**

R: we eat separately.

**I: children have separate...**

R: the children have separate plate than the adults

**I: ok.**

R: they also have separate cups like each child has already marked their own names on their cups. (Giggling)

**I: ok. Does the family share foods between households?**

R: yes. We share foods to this and that house because we are one family but different houses.

**I: oh ok. Now number sixteen. We have heard from some families that eat local foods whereas others eat processed foods. Could you explain what is typical for your family?**

R: we usually eat rice. If we don’t have fish, we usually buy hot dog and mackerel like these are the foods that we usually eat every day.

**I: ok. Now can you tell me what makes it difficult to easy or difficult to cook local foods?**

R: how?

**I: if you don’t usually eat local food. What makes it difficult for you to eat local foods? Or what makes it easy for you to eat local food?**

R: If I have local foods near me then I can just get them and eat them, but when I don’t have local foods near me, I can’t eat because I don’t have. If there is, we can eat, but if there is none, then we cannot eat.

**I: so that’s depend uh?**

R: yes

**I: it depend on whether there is or there is none**

R: yes. If we have we eat, but if there is none, we can’t eat (laughing)

**I: ok. If I am craving for local foods, I can just go the local market and get whatever foods I want to eat from there.**

**I: ok now can you explain the positive or negative things about local foods?**

R: no answer

**I: what are the good or bad things about local foods?**

R: there is no bad things about local foods, all local foods are good because they give us good health and help us make our body feel healthy. Not like the processed foods that people usually get in and out of the hospital

**I: ok so they get in and out of the hospital because they eat foods from where?**

R: processed foods.

**I: ok that’s great.**

R: when look at sick people inside the hospital, their family members usually visit them and bring them local foods.

**I: Now that we’ve talked about how family eats, I would like to learn more about how your child eats. Could you describe in detail what your son or daughter under two years commonly eats throughout the day? What does this child eat throughout the day?**

R: mackerel and fish

I: fish and mackerel ok. Now can you describe how you prepare the foods for the child?

R: please wait, I am really sorry.

**I: how many times a day meals and snacks are eaten by children under two?**

R: long pause (no respond)

**I: how many times your child eat throughout the day? How many times?**

R: she eat frequently throughout a day.

**I: can you guess how many times you feed the child throughout the day?**

R: no respond

**I: what would be the closest number on how many times you feed your child on a day?**

R: I feed her during breakfast, lunch and when the father is there eating, he also feed her with him *talking to family members*

**I: how do you know that the child has hand enough to eat or the child is full?**

R: uh?

**I: how can you tell that the child has had enough to eat?**

R: she eat until she refuse to eat.

**I: what do you do to encourage the child to eat?**

R: give her water

**I: what do you do to encourage the child to eat if the child refuses to eat? When the child is really refuses to eat what do you do to encourage so that the child can eat the foods?**

R: buy foods for her like the snacks.

**I: can you tell me if there is different when you feed your child when she is sick from when she is not sick? For example when the child is having diarrhea, is there is any different when you feed her?**

R: very long pause

**I: are there any different when you**

R: feed her?

**I: when she is having diarrhea and not having diarrhea?**

R: *lady talking to husband*

**I: was the question understandable? What is the different when you feed your child when she is sick from when she is not?**

R: no answer

**I: do you see that the child eat more or less?**

R: yes

**I: what you mean?**

R: when the child is having and not having diarrhea?

**I: yes**

R: when the child is having diarrhea, she refuse to eat. She eat only a little amount of food.

**I: ok**

R: when she have stomach ache, little by little she cannot eat any more but when the children poop, they only poop water, like their stomach is really empty.

**I: now we are in number eighteen, don’t worry. We’re almost finish. Ok. You’ve told me what your child under two usually eats. Now could explain to me the process, from start to finish, how you prepare and cook a meal for your child?**

R: no respond (talking to the baby and the father of the child)

**I: ok. Now this question wants to ask how you prepare the food that given for your child. Can you explain in detail how do you prepare the food from start to finish so that you can feed her?**

R: bring water to cook the food with.

**I: ok**

R: then put the food in a dish and then feed the child.

**I: ok**

R: wait and cool down the food and then feed the child.

**I: Could you now tell me what you think are important foods for children under two years to grow well and be healthy?**

R: papaya, pumpkin, just any kind of local foods also can be fish

**I: *couching* excuse me. Anything else? Pandanus and corn starch**

**I: the next question is asking about foods that should not be given to the child under two and reasons why?**

R: lolly bob

**I: can you explain more why did you say lolly shouldn’t be given to the child?**

R: they can eat lolly and the lolly candy can broke their teeth or they can be chocked from it.

**I: Now we want to know what your biggest influence of feeding the children is.**

R: no answer

**I: like what would be your biggest influence on feeding the children?**

R: still no respond

**I: for example, if it what your brother, you give advice to him, what would be the biggest influence for your brother in term of raising child or parenting? What would be your biggest influence on feeding child you would give your brother? This is just an example.**

R: take good care of them. Watched over them from the main road from eating from the dirt or from the ground.

**I: and what about feed the child?**

R: wash their hands before eating

**I: yes**

R: give foods that are good for their health

**I: ok. We are almost finish. These are very helpful information. Thank you for sharing.**

**I: can you describe any differences between how you feed your make children and how you feed your female children under two?**

R: there is no differences

**I: there is none ok**

**I: we are now in the last section of our interview. Let say we are now in family roles. We are also interest in the roles and responsibilities different family members play in raisin children. Could you describe the care of the children throughout the day in your community?**

R; no respond

**I: like people in this community, how do care about the child?**

R: let me make an example in this house

**I: ok**

R: when I discipline one of the children in that side, their parents would be mad. And when they discipline my child, I can also be mad with that. It is really hard to discipline other people’s children. Even though they realized that the child has made a mistake and we want to discipline them, they would be mad no matter what. So my husband and I discipline our own one year old child.

**I: ok so in your opinion, how do they raise their own children?**

R: they raise them in a wrong way like they love them in a wrong way.

**I: now we want to know who is mainly responsible for child care. As of that child, who is mainly responsible is care for her?**

R: just the two of us.

**I: now I want to know what are your responsible as a mother in child care. What are your responsible in caring for her?**

R: I make her food and feed her. This child is different from the rest of the children in this house. She doesn’t really eat a lot. Not like when she eats with her father, she would eat a lot until she is full.

**I: ok. Now what are the responsibilities of fathers in child care?**

R: he usually bring meat for the family like he is responsible in bringing food so that the baby can be eaten.

**I: ok. Ok now we would want to know how caregivers play with children under two years old. With the child**

R: no respond

**I: is there is anything that you do when you play with the child?**

R: I usually play with her

**I: yes**

R: she doesn’t come and play with me more often than come to this side or side and play by herself. She mostly play with the other children.

**I: ok. Number twenty two.**

**I: Could you talk about the role of grandparents have in raising children in this community?**

R: no respond

**I: as of the grandmother, what are her responsibility? What does she do?**

R: when I take a shower, she come and take her and watched over her.

**I: ok. So what makes the grandmother a good grandmother or a grandfather?**

R: no answer

**I: what do the grandparents do so that they can be good grandparents?**

R: they usually play with her in their side (Mother talks to the child)

**I: Now could you talk about the role that other family members have in raisin children this community? This question is talking about aunties and uncles.**

R: what?

**I: what are other family members in raising the child in this community?**

R: long pause

**I: is there is someone like her aunties or her uncles that or let’s say in what ways that sibling help in raising children?**

R: long pause (No respond)

**I: did you understand the question so that I can repeat it and make it more understandable.**

**I: like it is asking about other responsibilities of family members in raising the child. In terms of raising the child, what would be their responsibilities in help raising the child?**

R: no answer

**I: like your aunties or uncles, do they help raisin the child? Or take out the baby with them?**

R: no respond

**I: do you have family members that help you raise the child?**

R: no respond

**I: to make it more easier, how do siblings or older sibling help raise their young children?**

R: no answer

**I: in what ways they help in raising young children?**

R: no respond

**I: was the question understandable to you?**

R: yes

**I: so do you have any answers to this questions?**

R: silent (Long pause)

**I: ok. How was the question?**

R: they raise the child like the way we do

**I: ok. You answer perfectly. And we are almost finish this is the last section. The last two question and this interview finished. Could you explain where you usually get trusted information about nutrition and health?**

R: hospital from the nurses.

**I: can you give me one reason why these sources are trusted to you?**

R: they give us information with picture on them

**I: Now in your opinion, where nutrition and health massages should be delivered so that you would see or hear them most easily?**

R: radio b7av * Marshall Island International radio station*

**I: b7av ok. What types of media that people uses the most to communicate?**

R: telephone

**I: Now in this last question, it says, when you think about your own parenting behaviour, can you explain what influences how you raise your children? Is there is differences when you raise your child.**

R: there is no differences

**I: what are other’s opinion in this community about raising child?**

R: yes

**I: what are people’s opinion can you share with me?**

R: I don’t know what other people’s opinion

**I: ok you don’t know their opinion. Were there any advice or information related to parenting you received?**

R: there is none. I learn from myself.

**I: yes that’s true because I also do the same thing I learn from myself. So now is there any desired information on parenting you wishes to know but you don’t have available?**

R: there is none. I can learn from myself

**I: ok. Thank you so much for sharing your information and I know they would help us a lot.**
