# Supplementary material for: Conceptualizing multi-level determinants of infant and young child nutrition in the Republic of Marshall Islands–a socio-ecological perspective
Source: PLOS Glob Public Health. 2022 Dec 19;2(12):e0001343. doi: 10.1371/journal.pgph.0001343 (PMC10022247; doi:10.1371/journal.pgph.0001343)
Supplement: S1 Data — (ZIP) [file pgph.0001343.s001.zip › RMI Supp Data/Interviews data/I24U_IDI_FCG_Rita_Aug 15_Libon.docx]

Interview Code: I24U

Interview type: In-depth Interview- Female Care-giver

Interview Date: August 14 2018

Location: Rita

Interviewer: Libon

Transcriber: Fela

I: Before I proceed, I would like to ask if you agree to take your part on this survey.

R: Yes

I: Thank you so much for giving this time for me to speak with you today. Like is said earlier, the information you will share, will help us find ways to improve maternal and child health and sanitation in this community.

I: To begin with, can you please tell me a little about your family/household?

R: Like?

I: About you and people who live in this house.

R: what we do?

I: Noting, just tell on who live here with you, number of your children, how many girls and boys?

R: There are six people live in this house.

R: We have a first born son, but we have adapted a child before this child so we have two child in this house with us and my husband’s younger sister. Only six of us live in this house.

I: Ok. Now can you tell me the ages of your children? And how many female child or male child?

R: Only one female child and four male children

I: Can you tell on their ages?

R: My husband’s younger sister is twenty eight years old. The younger male child is sixteen years old, the second younger male child is twelve years old, and the youngest female child is eleven years old and then the baby is now seven months old.

I: Next, I’d like to ask you to describe this community.

R: Like what?

I: What happened in this community when you look around every day?

R: When I look around, I see that most children get sick and maybe the reason why is because they always play in sanitation areas. Another reason for children to get sick is they refuse to eat at the house because they would go outside the house and eat sweet and junk foods. These are the things that I usually see happen in this community.

I: Now can you tell me the positive things about this community?

R: It is good because the people are nice

I: hmmmm

I: Now you say the people are nice how nice they are? What make them nice, like what do they do?

R: Like they always get together…

I: Yes. Anything else?

R: you will see that every Saturday, people in this town do general clean up. Because the land owners want the town to clean all the time.

I: hmm that’s great. What about the negative things that you see about this community?

R: Like what?

I: things that you see don’t agree on them and still happen in this community and need to be fix.

R: people need to clean more and make it a better surrounding. Today is really different and like the same like before. Before was even better.

I: Better

R: yes

I: Let’s now talk about health and illness in this family

I: Can you tell me about some of the illness that your children have suffered from?

R: I think the only sickness that my children have suffered from diarrhea

I: Anything else instead of diarrhea?

R: When they got diarrhea, you know that they will get fever too

I: hmm

I: Now what are the causes of diarrhea?

R: I can see that what makes my children get diarrhea illness is from the water they drink, and I always boil the water so that they can drink from it.

I: hmm

R: Before, I never boil water for them, but now I always boil water and I can tell that it is the water that they drink and also can be from places where they go out and play.

I: What are the seriousness of diarrhea?

R: they’re having diarrhea and don’t what to eat, sometimes they eat but still they gain nothing, they get ever thinner each day.

I: Can you tell a story about a time when your child get diarrhea? What happen to the child and what are the seriousness of the illness happen to the child?

R: last few weeks, my child just recover from diarrhea and I don’t know how many times I went to the hospital and get medicine for him. When the child get diarrhea, he get multiple illness like nausea, fever, refuse to eat for days. I even go to the hospital and get him the vitamin medicine in order to help him feel strong. Even she get only little amount of food, she would poop a lot.

I: so you said you bring her to the doctors’ right?

R: Yes

I: What kind of food or drink did the doctor tell you to give or not to give to the child?

R: they told me to give more than enough water in order to help prevent the diarrhea, even though she can’t eat but the important thing is to keep water.

I: so when they told you to give water, was that helpful?

R: yes

I: what were the signs of differences that you saw with the child?

R: the child is also feed from any liquid and is using baby bottle. So I stopped giving other liquid to feed him and start giving her only water. By giving just water, I saw a big different, he was even better than before.

I: what are some ways to that you can prevent diarrhea?

R: I have to clean the dishes that she use to eat with, clean the food, and especially the water that is given to the child.

I: what about places where they play?

R: yes I also have to clean their playing areas.

I: Can you describe how you know when your child needs treatment for their illness?

R: when I see that my children don’t like their meal, or they change their diet, I don’t want to wait until they get worse than that. I would take them immediately to the hospital. When they get sick today, I don’t wait for tomorrow and bring them to the hospital.

I: what makes you rush and bring them to the hospital?

R: because I don’t want the illness to get even worse so when my children get sickness, they only get sick for only three days long because on the very first day when I realized that they are sick, I would bring them to the hospital immediately.

I: that is really great. Now who would be the first one that you bring the child to the health care and reason why?

R: the doctor first because I know they are doctor and they know what to do for the child’s illness.

I: so do you use traditional medicine?

R: for what?

I: diarrhea

R: no I haven’t use any traditional medicine for diarrhea.

I: you don’t use traditional medicine when the child get diarrhea?

R: no I haven’t

I: now we talked about... Ow no sorry...

I: Can you describe any illness affecting your children that are associated with nutrition? Are there any sickness or illness affect your children from eating nutritious food?

R: there is none

I: now why do you think there is no sickness affect the child?

R: maybe because these food contain with vitamin

I: so are there any illness caused by foods missing from the diet?

R: malnutrition

I: yes and what else

R: getting skinnier

I: now can you imagine your children when they would eat unhealthy food, what would they look like?

R: They would look really thin and even skinnier.

I: so were there any illness that would have affect them?

R: yes any kind of illness

I: now can you describe any illness that would affect them from eating unhealthy food?

R: like malnutrition, skin rash also can be one illness,

I: like what?

I: what kind of skin rash?

R: can be skin rash or ringworm or skin itchy.

I: We talked a lot about unhealthy. Could you now describe for me a typical day of someone living a healthy lifestyle, from the time they wake up in the morning until when they go to bed?

R: long pause…… (Someone giving her the baby)

R: when you see the person, he/she look healthy. They would move and do some works around the house from the time they wakes up until the time they go to bed, they really look healthy. (Baby crying)

I: Now can you tell me the appearance/signs of a healthy child under two years old?

R: the child have a beautiful and nice skin, the child is really active, and there is not skin rash

I: what about the appearance or signs of a healthy adult?

R: adult?

I: hmm

R: they look good, always wake up with a smiley face, and they feel healthy all the time.

I: Yes. Let’s now discuss hand washing. Could you describe in detail your family’s hand washing throughout the day?

R: people in this family usually wash their hand at the time they are going to eat. That’s the time they wash their hand, and for the children, we force them to wash their hand before eat.

I: Now how do they wash their hand?

R: they wash it with water and soap. We have the hand soap before they eat.

I: do the children wash their hand throughout the day?

R: for breakfast, they wash their hand, eat their snacks, they also wash their hand. They wash their hands before they eat. After using the bathroom, they also wash their hands.

I: what about the children under two year’s old, do they also do hand washing? For example your child, do you usually wash her hand?

R: Yes

I: Now can you tell the difference between using water only or water and soap to wash hands?

R: there is a big difference when we use only water, it’s not really clean. When we use soap, it does really show the dirt.

I: Now can you tell me about anything that prevents washing hands with soap throughout the day?

R: sometimes we forget to wash our hands.

I: yes.

I: are there any reason that prevent you from wash your hand?

R: There is nothing else than when we forget to was our hands.

I: Now we would like to talk about your diet during pregnancy and breastfeeding.

Now I would like you to think back to when you were pregnant. Can you describe your diet when you were pregnant compared to when you were not pregnant?

R: during not pregnant, I can eat any kind of foods. Compared to pregnant, I eat only few kind of food. When it comes to local food, I love to eat breadfruit. During pregnancy, the breadfruit is really delicious.

I: And what about imported foods?

R: I usually buy food from the store, foods like watermelon.

I: hmmm. What else?

R: except from fruits, I usually don’t eat meat.

I: ohhh

R: I can say that I usually eat cook breadfruit and fruits.

I: you mentioned fruits, what kind of fruits you like?

R: water melon, apple, and then grape. These are the only things that I liked to eat. And then when it comes to meat, I eat only one time at a day.

I: oh ok.

I: What kind of foods you were encouraged to eat during pregnancy and reasons why?

R: I have serious sickness, and my doctor or nurses told to me to eat nutritious food.

I: ohh ok. Now what kind of foods your nurses or doctors encouraged you to eat?

R: because I have diabetes sickness, they encourage me to go and eat at the wellness canvas. They told me that I should control the level of diabetic at that time.

I: now what kind of food you ate from the wellness canvas?

R: I ate there for only one or two times I think? I didn’t continue eat there because I couldn’t like the foods there.

I: now at these times that you mentioned only one or two times you ate there, what kind of food you ate at the wellness canvas centre?

R: pumpkin, the one that they cook it and mix it.

I: now what kind of food you were encourage not to teat during pregnancy and reason why?

R: there were lot of foods I was encouraged not to eat like the junk foods without any nutrient. They didn’t really tell me not to eat, but not to eat them frequently.

I: like what kind of food?

R: foods that we usually eat, like greasy food, salty food.

I: hmm. And what about drink? Were there any drink they encouraged you not to drink?

R: they usually told me not to drink soda. Drinks that are not good for my health.

I: so who encouraged or discouraged you to eat these foods during your pregnancy? Like who really advice you not to eat or to eat these foods?

R: hospital

I: hospital

I: now who from the hospital encourage or discouraged you to eat or not to eat the foods?

R: health worker from the public health because they were the one to treat my diabetic

I: oh okay. Now who primarily cared for or supported you during pregnancy?

R: in what ways?

I: in many ways during pregnancy, who cared and help or give you?

R: hospital like the doctors.

I: now you mentioned hospital, in what ways did they help you with?

R: like encouraged me

I: yes

R: to eat foods that are good for my health

I: so like when it comes to the community or your house, who cared or support you during pregnancy?

R: my husband and people in this house

I: now can you explain how they supported you during pregnancy?

R: the gangs here?

I: in this house, your neighbour, or hospital during pregnancy.

R: They only advise me and encouraged me on foods that I supposed to eat and not eat. Mostly people in this house, my husband and my friends and sometimes my husband’s friends.

I: hm ok. Now how do they help you? Like what do they do to you?

R: when they have local foods in their house, they bring to me.

I: can you tell me about any supplements you took during pregnancy?

R: I didn’t drink the vitamin medicine, the red button medicine. But I did drink the medicine for blood.

I: so were there any supplement you took during pregnancy?

R: oh yes the big button one. The one that it’s too big.

I: now can you tell me if you took all the supplements given to you and reasons why?

R: yes

I: were there any times that you did not take them?

R: no

I: Why do you think these supplements are really important?

R: to help both me and the child

I: great. Did you drink alcohol, smoke, or used any other drugs during pregnancy?

R: I did not

I: why didn’t you take these things?

R: because they are not good for my health.

I: Now you said that it’s not good for the health, can you explain more on that?

R: alcohol can affect the child

I: were there any traditional medicines you take during pregnancy and reasons why?

R: there was none

I: you didn’t take any traditional medicine during pregnancy?

R: yes

I: if you were advised to eat more fruits and vegetable during pregnancy, could you describe what would make this difficult?

R: sometimes we don’t have money to afford enough.

I: okay. Is there is anything else you can think of and you see that would make it difficult for you to eat fruits and vegetables?

R: I don’t think there is difficulties, the only difficult thing is money, when there is no money. I usually don’t eat and I don’t buy food that much.

I: what would make it easier to eat more fruits and vegetables and reason why?

R: it’s good for my body and it also can help give good health for the child.

I: you mentioned that “help” how does fruits and vegetable help you and the child?

R: the child grow well, and healthy.

I: and when it’s come to you, what would affect you?

R: I also feel healthy

I: Now can you describe your diet when you were breastfeeding?

R: pause or silent

I: like what kind of food you eat during breastfeeding.

R: I usually eat fish and fruits.

I: what kind of fruits?

R: when we do breastfeeding, we usually eat fruits and broccoli.

I: hmm now what kind of fruits

R: apple, orange, and just any kind of fruits.

I: what makes you eat these food during breastfeeding?

R: during breastfeeding, I couldn’t give enough breast milk for my child, and that’s why I really had to eat these kind of foods. By the time I started eat fruits and fish, I was able to give enough breast milk for the child.

I: what kind of food you were encouraged not to eat during breastfeeding and reason why?

R: unhealthy food.

I: now can you explain more on what kind of unhealthy food?

R: like the can meats, and chicken.

I: like what?

R: can meat and chicken.

I: who encouraged or discouraged you to eat those foods while breastfeeding?

R: hospital

I: ok hospital anyone else?

R: people in this house

I: after giving birth, could you describe breastfeeding your child throughout the day?

R: “confuses”

I: after giving birth, can you describe breastfeeding you child throughout the day?

R: how?

I: after you gave birth?

R: ok?

I: how did you do breastfeeding after gave birth? You gave birth and then just feed the child from breast milk or you waited and then later you did breastfeed? How long did you do breastfeed or not breastfeed?

R: it took three hours not do breastfeed.

I: can you explain why did you wait after three hours?

R: because the child was with the child for too long and then they came and tell me to put hot water on my nipple before I feed him

I: so can you tell me more on why did they tell you do that?

R: I just don’t know. They just told me to clean it or put hot water so that it can provide enough breast milk to feed the child. And it took times for the breast to spill with breast milk.

I: now after giving birth how long you started breastfeed the child and why?

R: it took a little bit longer because my breast didn’t really contain enough milk.

I: oh ok. Now did you give any liquids to the baby in the first few days after birth and reasons why?

R: I did not

I: were there anything that makes it easy or difficult to breastfeed exclusively up to six and months and reasons why? What makes it difficult or easy to breastfeed exclusively?

R: nothing. It was fine. It was only that time after gave birth and I did not really have breast milk. Now that I have enough breast milk to feed the child, there is no more problem or difficulty to breast feed the child.

I: were there anything that makes it easy or difficult to breastfeed up to two years old and reason why?

R: my child is no longer eat from my breast because he is now six months and she can now eat real food. And I don’t know what makes him refuse to eat from the breast milk I give her.

I: does it comes from when you don’t really provide enough breast milk?

R: maybe. That also can be one reason why. Sometimes when I don’t really provide enough breast milk for her, I can give her double from breast milk and other liquid. Today she really hate breast feed and liquid bottle feed.

I: We are trying to understand how people eat in this community. Could you describe in detail what your family usually eats and drinks throughout the day? For example, people in this house, what you eat or drink throughout a day?

R: they usually eat rice, bread, yes I think these are the things people in this house usually eat. And also meat for breakfast.

I: so you mentioned about meat? Can you tell me more on what kind of meat you eat?

R: we usually eat chicken, and can meat.

I: Now can you describe the process of how meals are made?

R: sometimes we would just eat the food and other times we mix food tougher so that it can be enough for the whole family.

I: so you mentioned ingredient? Or mix foods together, what do you use?

R: sometimes we would mix the corn with cabbage.

I: now can you tell me who in this family would serve first, next, and last?

R: in this family, our children served first.

I: ok, now who would be next?

R: me and my husband

I: are there any differences in the foods served to different family members? For example, your younger sibling, your older children, you and your husband.

R: everyone eat the same kind of food. (Baby crying out ☺ )

I: are there any differences in quantities of food served to different family members?

R: there are none.

I: can you explain more on why there is no quantities of food given to the family members?

R: like what?

I: when you served food do people receive different quantity of food?

R: yes there is

I: now for the children, how much food you would give them?

R: I give one or two scoop of food for the each child.

I: and what about the adult, how much food you served them?

R: two scoop of food

I: is it bigger than the children?

R: yes two scoop or bigger than the children

I: do some children receive more food than others?

R: I don’t think there’s any…only the older sibling, they eat more than the younger sibling

I: what about the way you make their food, do you give amount or quantity of food for the children?

R: depend on their diet (baby crying)

I: maybe he is hot, you can turn the fan to him

R: He is sleepy and really annoying

I: is the breast okay? Or does the baby get enough milk?

R: sometimes

I: Now could you describe any food sharing between family members during mealtimes for examples children eating tougher separately from the family, meals eaten form the same plate by all family members?

R: everyone have their own plate, children have different plate from the adult.

I: can you explain why you give separate plate to each person?

R: so that each person can balance their own diet.

I: Do the family share food between households?

R: yes sometimes we share foods. And if there is enough food to share we can share.

I: ok. We have heard from some families that eat local foods whereas others eat processed foods. Could you explain what is typical for your family?

R: foods like rice, can meat, chicken, and hot dog.

I: now why the family usually eat processed food?

R: because we don’t have local food. Not all the time we have local food.

I: so you mentioned that you don’t have local food, why don’t we have local food? What you mean by saying you don’t have local food?

R: we don’t have farm to plant local food. Local foods are too expensive and process foods are cheaper and are easy and ready to cook.

I: what makes it difficult or easy to cook local food? Or can you discuss the difficulties?

R: sometimes when we want to cook local food, we don’t have wood to make the fire. For some local foods, we have to use the coconut milk and sometimes we don’t have it. We have lot of things to mix with our local food and we don’t have them.

I: ok. Now what makes it difficult to cook local food?

R: I can cook them easily because I know how to cook them

I: that’s great. What else that make local food easy to cook?

R: sometimes we have coconut to provide coconut milk for the food, and that also on thing that makes it easy to cook these local food.

I: now can you explain to me the positive and negative things about local food?

R: The good thing about local foods they’re new, I meant brand new. We take them down and then we can cook them right away. These foods and fresh and healthy.

I: ok. What about the negative things about local foods?

R: I don’t think there negative things about local food.

I: Now can you tell me about the positive and negative things about processed food?

R: processed food are good because we can just get them from the store and eat them or cook them in the simplest way.

I: and what are the negative things about them?

R: the negative things about them is they give us poor health. They are greasy and salty food.

I: so you mentioned they give you poor health and can cause sickness, can you tell me more what kind of sickness causes by these foods?

R: serious sickness that we see today happened from these foods, are diabetic, high blood pressure, and kidney.

I: Now that we’ve talked about how the family eats, I would like to learn more about how your child eats. Could you describe in detail what you son/ daughter under two years commonly eats throughout the day?

R: can be any kind of food that we can prepare for her. Can be rice or hot dog.

I: can you explain more because your child is not yet two years old what do you feed the child?

R: My child can eat cook breadfruit, bread, papaya and potato.

I: how do you cook or prepare the foods for your child?

R: I just cook them.

I: like do you add ingredient to the foods or?

R: as of potato, I can mix the potato with the pumpkin, and for the papaya, he can just eat the papaya itself or sometimes I can mix the papaya with bread and feed him or can be the pumpkin with bread.

I: can you tell me how do you know that the child has had enough to eat?

R: I balanced the diet

I: now how do you know that the child is full?

R: I would feed him and until he refuse to eat.

I: what do you do when to encourage the child to eat?

R: silent

I: when the child don’t want to eat, what would you do to make the child eat?

R: breast feed him

I: is there is anything else that you try so that he can eat?

R: Sometimes when he refuse to eat, I would try my best and look for food that would make him eat.

I: what if he really refuse to eat, what do you do so that he can eat?

R: I breast feed him and give him water

I: what is the different when you feed the child during sick?

R: yes there is a different, because when the child is not sick I can feed him any kind of food, not like when he is sick, he pick on what kind of food he would want to eat.

I: You’ve told me what your child under two usually eats. Now could you explain to me the process, from start to finish, how you prepare and cook a meal for your child?

R: what you mean?

I: how do you cook or prepare the food so that it could be ready for the child to eat? From the time you are ready to cook the food, what are the steps in doing the food or the process of making the food so that it can be ready for the child? Different people have their own ways of cooking or preparing the food, so when it comes to you what are the technique of cooking food you do?

R: before I cook foods, I wash my hands first, then cook the foods

I: what do you do at the very last part of your cooking?

R: I wash my hands

I: can you please discuss in detail the way you prepare the child’s food?

R: like is said, I wash my hands first, then if I would cook pumpkin, I cut in into pieces and boil the pieces. Next, I add a little bit of sugar to the boiling water and also add a little bit of flour. Then I also add the starch and mix it with the coconut milk. Finally, I also wash my hands and leave the food and until it cool down and then feed the child.

I: that is great. Now could you now tell me what you think are important for the children under two years to grow well/be healthy?

R: nutritious foods

I: can you list them down

R: papaya, pumpkin, and pandanus and can be any kind of local foods

I: what about processed food?

R: fruits like apple, orange, and mango

I: now when it comes to meat, what kind of meat you can give to your child that make him grow well?

R: chicken, fish and crab

I: what are the biggest influence of feeding your child?

R: the child live healthy, cute and chubby, feeling well every day. He/she doesn’t feel lazy or sick.

I: Can you describe any differences (if any) between how you feed you male children and how you feed your female children under two? Are there any differences?

R: silent

I: for example, if you have twin? One girl and a boy, were there any differences in feeding them? Would you give the boy food more than the girl and reason why?

R: I don’t know

I: from your own opinion?

R: I would say that I could have given them the same amount of food

I: we are also interested in the roles and responsibilities different family members play in raising children. Could you describe the care of children throughout the day in your community?

R: they watched over them, when it meals time, they feed them. They raise them good. They bathe them and wash their hands before eat.

I: now who is mainly responsible for child care?

R: the mothers

I: you mentioned the mother, what are the responsible of the mothers for the child?

R: she cook, do laundry, and feed the child…

I: is there is anything else? As of you, what are your responsible for your child?

R: I watch over the child.

I: all the responsible are for the mother

I: like what responsible

R: take good care of them and their food. Teach them.

I: what are the responsibilities of a fathers in child care?

R: bring food for the baby, watch over them and teach them, over them from sickness.

I: how caregivers play with children under two years old?

R: they play with them, read books to them, and they also can sing to them

I: anything else?

R: play with them and teach them

I: could you talk about the role of grandparents have in raising children in this community?

R: grandparents? They also watch over them, protect them from sick and dirt, and teach them

I: what ways that grandparents help support in raising children, support mothers and families?

R: sometimes they bring foods for them.

I: ok and what else

R: they also help babysitting the child, and discipline them

I: are there any advice grandparents give to parents

R: yes all the time

I: like what? Can you discuss in detail?

R: they teach them and make sure they don’t play in dirt places, and sometimes they make sure that the food and drink we give to them are good for their health.

I: what makes good grandparents (grandfather/grandmother)?

R: they always advise us and discipline us is good ways

I: Could you talk about the role that other family members have in raising children in this community?

R: they also make sure that they don’t play in sanitation areas, and also discipline them. Make sure they don’t get sick.

I: how do older siblings help raise young children?

R: they do the same thing like play with them, teach and make sure they don’t play in dirt places.

I: you are doing a great job. We are almost finished. Now for the last section, we would like to learn about ways we can develop health programs in this community.

I: Could you explain where you usually get trusted information about nutrition and health?

R: can be anywhere

R: hospital, or places that people usually go to.

I: could you please give me one reason why you trust these information?

R: because I know that they are trusted information

I: can you explain more on reason why you trust these information.

R: they tell me to do so and I can see that their information and real and helpful

I: where do you think nutrition and health messages should be delivered so that you would see or hear them most easily?

R: churches, the schools, and the hospital

I: what types of media that you use the most to communicate

R: Facebook, online

I: so you mentioned Facebook or online, do you usually get information like these?

R: yes

I: when you think about your own parenting behaviours, can you explain what influences how you raise your children? Can you describe in detail the difference when you raise children?

R: what makes it different is giving them foods to eat. I think that is the only thing that makes different is kind of food caregiver gives to the children

I: what are the opinion of the community influence how they raise their children

R: they give advice

I: like what kind of advice?

R: the way we should raise children. Prevent them from sickness

I: are there any advice or information related to parenting you received?

R: yes

I: can you explain them?

R: feed them, bathe them and prevent them from any kind of sickness

I: so where or who gave you these advice or these advice came from?

R: hospital, from my friends or my parents

I: are there any desired information on parenting you wished to have but doesn’t have with you?

R: I think there is none

I: is there is anything else about the topics we talked about today that we missed or that you would like to tell us about?

R: everything is perfect

I: that you so much once again for your generous time and for sharing your thoughts with us. We greatly appreciate your help and we hope this research will help us improve the health of mothers and children in this community.
