# Supplementary material for: Conceptualizing multi-level determinants of infant and young child nutrition in the Republic of Marshall Islands–a socio-ecological perspective
Source: PLOS Glob Public Health. 2022 Dec 19;2(12):e0001343. doi: 10.1371/journal.pgph.0001343 (PMC10022247; doi:10.1371/journal.pgph.0001343)
Supplement: S1 Data — (ZIP) [file pgph.0001343.s001.zip › RMI Supp Data/Interviews data/I25U_IDI_FCG_Rita_Aug 15_Libon FelaEdited.docx]

**Interview code: I25U**

**Interview type and Interviewee: FCG**

**Interview Date: Aug.15.2018**

**Location: Rita**

**Interviewer: Libon**

**Transcriber: Libon**

**I: Before we proceed, do you agree to participate in this survey?**

**R:** Yes

Thank you for giving us your time to speak with us today. The information we learn here will help us find ways to improve maternal and child health and sanitation in your country.

**I: To begin with, can you please tell me a little about your family/household?**

**R:** … can you say that again?

**I: Can you tell me a little about this family?**

R: about this family?

**I: hmm (Yes)**

**R:** About this family they’re always together. They’re cooperative, they eat together, drink together, Party, birthday party and anything that they do together.

**I: who lives in your house?**

**R:** My grandmother, my aunties, my older brothers… and my little sisters

**I: How many children are in this house and how old are they?**

**R:** There is a five (5) yrs. old, three (3) yrs. old and one (1) yr. old

**I: Now how many are there boys and how many are there girls?**

**R:** One boy and three girls

I: Next, I’d like to ask you to describe your community:

**R:** About this community?

**I:** Or this town, what are some of the things that happens in this town?

**R:** There are drunker, the community leader is not good to the community and that’s about it.

**I:** what about the kids that are in the community?

**R:** They’re always playing in the dirt

**I: Anything else?**

**R:** Nothing

**I: What are the good things about this community?**

**R:** … Because they play volleyball, basketball, baseball, bonnie…

**I: What about how people are always cooperating?**

**R:** Like they’re always together telling stories, parties, birthday parties, and other things that Marshallese usually do.

**I: And what are the bad things about this community?**

**R: …**

**I: Can you tell me the bad things that you see in this community?**

**R:** Sometimes they argue with each other, sometimes they’re happy with each other and tell stories with each other

**I: What about the children in this community? What are some of the bad things you see with the children living in this community?**

**R: they would hurt each other, there’s no playground, and they play in the trash and near the road.**

Let now talk about health and illnesses in your family**.**

**I: Can you tell me about some of the illnesses that your children have suffered from?**

**R:** Asthma, coughing and vomiting and that’s it

**I: Now that you say asthma, coughing… what causes them to cough and have asthma? Why do they cough and have the asthma?**

**R:** It comes from wet diaper, soaking in water

**I: Are there any other kind of illnesses other than these that you see that the other children that are older than him had when they were two years old**

**R:** diarrhea, high temperature, fever and stomach ache

**I: Why do they get the illnesses that you say like, diarrhea, fever, high temperature and stomach ace?**

**R:** It comes from dirts, they play with dirt

**I: Yes, what about their food**

**R:** Usually orange, apple

**I: No! Their food, does it affect them having diarrhea?**

**R:** Usually because when they eat it, it’s new to their stomach (new diet)

**I: what about the way you prepare their food does also affect having the diarrhea?**

**R:** hmmm (yes). They don’t wash their hands (also hand sanitizer) and they don’t chase flies away from their foods while they’re eating.

**I: Now what are the seriousness of these sickness that you listed before?**

**R:** asthma?

**I: Like asthma, what are the seriousness of asthma?**

**R:** they would vomit, get weak,

**I: what about diarrhea, what are the seriousness of diarrhea?**

**R:** Because they don’t wash their hands and they don’t watch flies

**I: But what are the seriousness that can occur to the child because he/she has diarrhea?**

**R:** they get skinnier, get stomach ache… they don’t eat

**I: Now are there ways you can prevent these illnesses?**

**R:** drink medicine

**I: drink medicine**

**R:** wash their hands

**I: good, anything else**

**R:** no more

**I: Can you describe how you know when your child needs treatment for their illness?**

**R:** They usually give the day or when they say

**I: What about when he’s sick, how do you know when to take him?**

**R:** when he’s sick?

**I: When he’s sick?**

**R:** When we would hold him there is like this atmosphere, then we would rush him

**I: Now what kind of atmosphere do you see?**

**R:** usually shortness of breath (asthma). That’s his illness (asthma) and coughing

**I: Now who do you first go to for healthcare (and reasons why)?**

R: Usually the… American doctor

**I: So usually you take him first to the doctors?**

**R:** hmm (yes)

**I: But are there anybody else other than the doctors that you go to or take your child to?**

**R:** Just the doctors

**I: Why do you take him to the doctors first?**

**R:** So that they can take a look at him, check him and see what kind of illness he has.

**I: Do you use Marshallese medicine (traditional medicine)?**

**R:** No I don’t… When he coughs I would give him banana juice. That’s what he usually drink and if not then kiop(Local herb)

**I: Now those medicine, how do you use it on him?**

**R:** We pound them and you know the water bottle or the bottle (baby), we would put it inside… It would take three days and it goes away

**I: Can you describe any illnesses affecting your children that are associated with nutrition?**

**R:** Nothing

**I: Can you explain the reason why you say “nothing?”**

**R:** … He’s doing well and he’s bigger and that’s about it

**I: What kind of illnesses are caused by food missing from the diet? If your child’s food is unhealthy, are there any illnesses that can occur**

**R:** hmmm (yeah)

**I: What kind of illness or can you explain what kind of illness and why does it affect him?**

**R:** Because not enough food, doesn’t eat on time, when he sleeps, usually seven, eight or nine o’clock he would wake up and eat.

**I: But what kind of illnesses would affect him?**

R: Nothing except for his illnesses, asthma and coughing

**I: For example: If you were to feed your child… rice hotdog, rice hotdog, rice hotdog, rice tuna. How would his health be?**

**R:** He wouldn’t be healthy because he’s only eating rice hotdog.

**I: And why do you say that he wouldn’t be healthy?**

**R:** He’s malnutrition not enough nutrition

**I: We talked a lot about being unhealthy. Could you now describe for me a typical day of someone living a healthy lifestyle, from the time they wake up in the morning until when they got to bed?**

**R:** Because he/she eats apple, orange, chicken, rice and he’s really full and big. The way he look is really happy.

**I: What about a child under two years old? What are the signs indicating that the child under two years is really healthy?**

**R:** He’s bigger, he’s taller, and he takes his naps….

**I: Okay anything else?**

**R:** Nothing

**I: What are the appearances/signs of a healthy adult?**

**R:** He exercises and he’s bigger. He usually eats what diabetic patient would eat

**I: Can you tell us some of the food that he eats to be healthy?**

**R:** Usually eat bread, soup, drinks milk and water, eat apple and orange and eat meat.

**I: Let’s now discuss hand washing. Could you describe in detail your family’s hand washing throughout the day?**

**R:** They hand sanitize their hands. One room one hand sanitizer. If not the dish washer. They wash their hands with water and soap.

**I: Does the children wash their hands throughout the day?**

**R:** Yes.

**I: Can you tell us when it’s important for the children to wash their hands?**

**R:** When they eat… usually when they eat sweets, when they touch the dirt. When the touch the dirt and don’t wash their hands.

**I: okay…**

**R:** after they use the bathroom

**I: what about when they’re about to eat?**

**R:** They also wash their hands

**I: What about children under two years old or your son, do you wash or when do you wash their hands throughout the day?**

**R:** every minute or every second

**I: Now how do you wash his hands?**

**R:** I use hand sanitizer. Usually hand sanitizer

**I: Is there anything else other than hand sanitizer that you use to wash his hands with to get rid of the germs?**

**R:** when he takes a shower, I usually scrub his hands really good, his feet, his cheeks, and really soaps his face because people are always kissing him.

**I: When do you use soap to you wash your hands throughout the day?**

**R:** everyday

**I: Now can you describe in detail what you mean by every day? When every day? When do you wash your hands?**

**R:** In the morning, the afternoon and evening… and when we’re preparing food… and when we’re touching the dirt and using the bathroom**.**

**I: Can you tell me the difference between using water only or water and soap to wash your hands.**

**R:** … I soap my hands before washing it.

**I: During the time when you’re washing your hands, what’s the difference if you were to wash your hands with just water?**

**R:** The germs are gone.

**I: Good. Now what prevents you from washing hands with soap throughout the day?**

**R:** … What did you say?

**I: What prevent you from washing your hands with soap throughout the day? Why don’t you wash your hands with soap?**

**R:** … we forget because we’re rushing…

Now we would like to talk about your diet during your pregnancy and breastfeeding.

**I: Now I would like you to think back to when you were pregnant. Can you describe your diet when you were pregnant compared to when you were not pregnant?**

**R:** Usually sashimi and pizza. There are the things I wanted to eat.

**I: What about when you were not pregnant? What kind of food did you usually eat?**

**R: Bread. I think only bread.**

**I: Is that the only food you ate when you were not pregnant?**

**R:** hmmm (Yeah)

**I: What influenced you to eat these food when you were pregnant? Like you mentioned before, you only wanted to eat pizza and sashimi?**

**R:** Because when I crave, these were what I craved for.

**I: oh okay. Now what foods were you encouraged to eat during pregnancy?**

**R:** They told me to eat orange, apple and foods that are for sick people.

**I: Anything other than orange and apple?**

**R:** Usually… that’s about it

**I: What about healthy food like vegetables, were there vegetable that you were encouraged to eat?**

**R:** hmm (yeah)

**I: Like what?**

**R:** Green leaves

**I: Green leaves?**

**R:** hmmm (yeah)

**I: Now why did they want you to eat this green leaves?**

**R:** So that the baby can grow and get bigger and be healthy. Not like the other baby where they’re born premature.

**I: What kind of foods were you encouraged not to eat during pregnancy?**

**R:** Can you say that again?

**I: What kind of foods were you encouraged not to eat during pregnancy?**

**R:** They told me to eat less rice

**I: Good, what else?**

**R:** Hotdogs and the one Marshallese usually eat and also salt, Kool-Aid, ramen

**I: Now why did they encouraged you not to eat these foods?**

**R:** Because if we eat ramen with Kool-Aid it will damage the ovary

**I: Now how would that affect the baby? Would it have affected the baby during the time you ate the foods that they didn’t want you to eat?**

**R: …** The reason why it would affect them is we’re eating foods that’s not good. The doctors are telling us not to eat them but we eat them anyways. But that’s just how pregnant women are.

**I: Now can you tell me one reason why it would affect your baby?**

**R:** Won’t be healthy and won’t grow healthy.

**I: Now can you tell me who encouraged or discouraged you to eat those food when you were or during pregnancy?**

**R:** The Pilipino… I think the Pilipino

**I: Pilipino from where?**

**R:** At the hospital. From public work… oh health and the doctors and nurse, mom and dad

**I: Now… who helped or supported you when you were pregnant?**

**R:** My grandmother and my aunties

**I: Now how did they help you when you were pregnant?**

**R:** Because I had caesarean… and they told me to hang it there, but because my water broke they had me rush to the hospital. We both almost didn’t make it but we did. And they told me to surgery right away. When I got to the hospital, they told me they were going to do caesarean so they did.

**I: Now when you had your caesarean, how did they help you?**

**R:** They gave me the epidermal shot.

**I: What about your mother, grandmother and your aunties, how did they help you when you had your caesarean?**

**R:** They said… The only thing they said was, “this is a woman’s war, do you best because you’re a woman.”

**I: What about the other people in this community, how did they help you when you were pregnant?**

**R:** … They visited me, talk stories with me

**I: Now can you tell me what kind of supplements you took during pregnancy?**

**R:** Usually vitamins and Iron-sulfate

**I: Okay. Now did you take these supplement they gave you?**

**R:** hmmm. Yeah

**I: Did you at one point stop taking these supplements?**

**R:** I stopped when I was eight months.

**I: Why**

**R:** I just started vomiting and…

**I: Now did you drink alcohol, smoke or use other drugs when you were pregnant?**

**R:** I don’t think so

**I: Why didn’t you take these? What prevented you from taking these things?**

**R:** Because they say alcohol is bad. Alcohol comes from can harm, will affect the baby. Includes alcohol, betel nut and smoking.

**I: Were there any traditional medicines you took when you were pregnant and why did you take these medicines?**

**R:** Can you say that again?

**I: Were there any traditional medicines you took when you were pregnant... When you were pregnant were there any traditional medicines that they gave you?**

**R:** I don’t think so

**I: If you were advised to eat fruits and vegetables during your pregnancy, could you describe what would make this difficult?**

**R:** I don’t think there’s any.

**I: Now… What would make it easier to eat fruits and vegetables… why was it easy for you to eat these fruits and vegetables?**

**R:** Because they make us full. When we eat them we feel good…

**I: Are they any reasons or are there any other reason why it’s easy for you to eat these fruits and vegetables?**

**R:** I don’t think so.

**I: What about the growth of the baby inside of you?**

**R:** Because of the fruits and vegetables we eat…

**I: Can you describe your diet when you were breastfeeding?**

**R:** Usually rice, fry fish, breadfruit and banana

**I: Now what influenced you to eat these foods when you were breastfeeding?**

**R:** So that I can produce more breast milk, so that the baby is full/has a full stomach.

**I: Now what kind of food did they want you to eat when you were breastfeeding?**

**R:** They told me to eat sashimi…. Iu with flour (IQ), rice, soup and corn beef, mackerel, tuna.

**I: Now why did they want you to eat these?**

**R:** To produce more breast milk, babies are full.

**I: What kind of food they didn’t they want you to eat?**

**R:** I don’t think there’s any… oh ramen, kool aid, piknik and food that are not good for eating.

**I: Now why didn’t they want you to eat these foods?**

**R:** Because, they reason why they want us to eat them is because our breast is going to hurt and we won’t be able to produce breast milk and the baby won’t be healthy.

**I: When you say healthy, what do you mean by healthy?**

**R:** Full stomach because we have a lot of breast milk, he sleeps long.

**I: Now who encouraged or discouraged you not to eat these foods during breastfeeding?**

**R: Our mothers and fathers, the doctors, the nurses…**

**I: Now what is the real reason to why you should or should not eat these foods?**

**R:** So that we breast milk, the baby is full, the baby grows well, healthy and gets bigger.

**I: Now after giving birth, could you describe how you breastfed your baby throughout the day?**

**R:** As I was going to breastfed, they told me to lie down and breastfeed. So I did because I couldn’t move.

**I: Why did they tell you to lie down and breastfeed?**

**R:** Because I couldn’t move

**I: So did you give, how long after giving birth did you start breastfeeding you baby?**

**R:** Nine o’clock I gave birth, ten to eleven o’clock I started breastfeeding. It took almost two hours of sitting and breastfeeding. I lied down and breastfed but when they told me to sit up and breastfed I did.

**I: Now what…… Why didn’t you breastfeed right after you gave birth but you waited two hours and** breastfed?

**R:** Because he was in the incubator. They had to wash him first and put him in the incubator and weigh him

**I: OH… Did you give him other kind of milk other than the breast milk during that time?**

**R:** No

**I: But… Why wasn’t there any other milk you gave him other than the breast milk?**

**R:** There was breast milk, he was breastfeeding

**I: Were there anything that made it easy or difficult to breastfeed exclusively from birth up to now?**

**R:** I don’t think so.

**I: Can you explain what made it easy or whatever made it easy just to breastfeed from birth up to now?**

**R:** There’s breast milk. When he’s breastfed he feels good. There’s nothing on his body.

**I: When you say there’s nothing on his body, can you really explain what you mean by there’s nothing on his body, it’s not what?**

**R:** It’s not… There’s nothing like skin rashes (karko, rajjia and kito).

**I: okay. But what about his appearances, what are the sign that tells us that he’s healthy?**

**R:** He’s bigger, he’s always smiling.

**I: So are there any difficulties or easy to breastfeed up to 2 years old? Does he still breastfeed until now?**

**R:** Yes.

**I: Are there any difficulties you see in breastfeeding until now?**

R: **There are difficulties**

**I: Now can you really explain what difficulties you’re facing?**

**R:** Sometimes there is not enough breast milk, when we’re sick and we don’t want to eat it can also affect the producing of breast milk (not enough).

**I: What makes it easy just to breastfeed until now?**

**R:** I don’t think there’s any.

**I: Now can you tell when you first gave foods and /or liquids other than breast milk to you child?**

**R:** when he was six months… He ate and drank.

**I: What did he eat and drink?**

**R:** Baby, now orange, apple and banana… and that’s about it.

**I: When you give him orange and apple, how do you feed him?**

**R:** We slice them, bound them and feed them.

**I: oh okay**

**R:** and make it soft.

**I: What drinks did you give him on the first day you fed him?**

**R:** Apple juice, water, milk

**I: Why did you give these foods to?**

**R:** Say that again?

**I: Why did you give these foods or drinks other than your breast milk at that age, at six months?**

**R:** Because it was time, the doctors said it was time for to eat…

**I: What are the opinions from others that made them choice to give foods and drinks at this age (6 months)?**

**R:** To be healthy, grows well, to be full, to get bigger.

**I: Now what were the first foods and how did you prepare them?**

**R:** Usually baby food and water.

**I: Now the baby food that you feed him where do you get them from, do you make it yourself or do you buy it from the store?**

**R:** Baby food from the store.

**I: Now what flavors do you usually buy or what flavors does he like?**

**R:** Usually banana, orange? Or what’s it call? Yeah orange, carrots and vegetables and.

**I: Good. But do you make him or do you make your own baby food for him?**

**R:** I feed him.

**I: When I say make, I mean by do you make your own baby food other than the ones you go and buy from the stores?**

**R:** I do. Like breadfruit, papaya, pandanas and banana

**I: Now how do you make it, for instant the banana? How do you make it or prepare it?**

**R:** We dice it up and cut it into pieces and we bound them.

**I: Okay. What about for instant the pandanas? How do you prepare the pandanas?**

**R:** We boiled them, grate them (get the juice out)… and mix it with milk.

**I: Good… Now when you feed your son how do you prepare or make his food?**

**R: I wash my hands, cook him food, let it cool and bring it and feed him. And hand sanitizer.**

**I: Now we are trying to understand how people in this community eat. Could you describe what your family eats and drinks throughout the day?**

**R: They usually eat rice and sashimi if not rice and chicken. I think these are the usual. Oh and drink water and rice and hotdog.**

**I: What about can meat, does this family usually eat these foods?**

**R: Usually tuna, mackerel, corn beef and spam.**

**I: Now what about drinks? What do they usually drink?**

**R: Water and soda.**

**I: Who in this family get served first, next and last?**

**R: The kids, the elders, our mom and dad**

**I: Now are there any differences in the food you give to each family?**

**R: No everybody is the same.**

**I: But are there any differences in the amount of food given to each family?**

**R: No**

**I: Now are there any kids that receive more foods than others?**

**R: No**

**I: No? Now can you like give me an explanation for instant how this family, how they, how they serve food whose separated, who get served first and last?**

**R: The children first, then the elders, our parents and visitors if any.**

**I: Now can you explain how this family share food during meal time (for example children eating together separately from the family or meals are eaten from the same plate?**

**R: Everybody eats from the same plate**

**I: Can you really explain what you mean by the same plate?**

**R: Because you know children, when they want to eat here and there.**

**I: What about the adults?**

**R: Well they eat by themselves.**

**I: Does this family also share food with their neighbors?**

**R: Yes… When there is a lot of food then we share**

**I: Now how much do you share with your neighbors, or how much do they share with the neighbors?**

**R: Usually little to each. Little by little with love.**

**I: We heard that some families eat local foods whereas others eat process foods. Could you explain what foods your family usually eats?**

**R: They usually eat sashimi and chicken. That’s it.**

**I: What about local foods?**

**R: Usually breadfruit, pandanas, banana, roasted breadfruit, fish and that’s it.**

**I: Now what makes it difficult or easy for you to cook local foods?**

**R: … Can you say that again?**

**I: What makes it difficult for you to cook local foods?**

**R: Because we boil it. For example, if it was a breadfruit then we boil the breadfruit, pandanas, we boil the pandanas**

**I: But…ummm… now you’re telling us about the how easy it is to cook but can you tell me the difficulties. What are the difficulties in cooking local foods? What’s difficult?**

**R: There are no woods and propane gas to cook. There is no money to buy the foods. There are a lot of difficulties.**

**I: You mentioned earlier that it’s easy because you boil the breadfruit and boil the pandanas. Are there any other reason why it’s easy for you to cook these foods?**

**R: I know how to do them. And it’s easy to find them.**

**I: Good. But what’s… good about local foods?**

**R: Makes our body grow, be healthy, bigger, good facial expression and don’t get sick.**

**I: So what’s bad about local foods?**

**R: When we don’t eat local foods, we don’t feel good, our body doesn’t grow but gets weaker.**

**I: What’s good about processed foods?**

**R: There’s a price for it… it’s cheap. It’s already just bring it and eat.**

**I: What’s bad about processed foods?**

**R: Because when there’s no food everybody suffer. People stop working, a lot of illnesses.**

**I: Now what kind of illness are you saying usually occurs?**

**R: … Diabetes, high blood, cancer, kidney**

**I: Good answer. Now we’re done with how the family eats, now I want to know how your child eats. Can you describe what your son under 2 years commonly eat throughout the day? Now you’re going to talk about what your son usually eat.**

**R: Usually eat, in the morning he eats nutrition (cracker) if not ramen. In the afternoon he eats rice, chicken or hotdog. In the evening, he eats rice and spam or corn beef.**

**I: Now how many times does your son eat include his snacks?**

**R: …**

**I: How many times a day does he eats?**

**R: He can eat morning, lunch and dinner… Umm and snacks**

**I: If he eats snacks, how many times does he eat snacks?**

**R: Maybe once**

**I: Now how do you know your child has enough to eat?**

**R: He cries because he wants something. When someone walks by with something he’s cry for it.**

**I: What about you? How do you know he has enough?**

**R: He gags signs that he’s full. He vomits when he’s full.**

**I: Now what can you do when you child doesn’t/ refuses to eat?**

**R: Huh?**

**I: What can you do when your child refuse to eat?**

**R: He cries a lot. When he doesn’t eat he cries**

**I: So what do you do?**

**R: Wake up and feed him…**

**I: Now when he doesn’t want to eat, what do you do?**

**R: Breastfeed him**

**I: What can you do to him if he doesn’t want to eat? What can you do?**

**R: Huh?**

**I: What do you do or what can you do when he doesn’t want to eat?**

**R: Breastfeeding**

**I: Are there anything else that you do so that he eats?**

**R: …**

**I: Do you feed your child differently when he’s sick (for eg. When he has diarrhea)**

**R: I usually feed orange and apple**

**I: Apple and orange? Why do you feed him apple and orange?**

**R: So that he doesn’t get the diarrhea and make drink a lot of water.**

**I: So you’ve told me what your child under 2 years usually eats. Now could you explain the process of preparing food for your child from start to finish? How do you make or prepare your child’s food?**

**R: We fix their food, let it cool and then feed them.**

**I: Now… What kind, for instant, what, can you explain a food that he usually eat, how would you prepare the food? For example, if it was a pumpkin that you were cooking, how would you cook it from start to finish?**

**R: We wash our hands and cook.**

**I: How do you mix it?**

**R: We mix it with carrots, vegetables…**

**I: Now can you tell me what foods do you think is important for children under 2 years and why?**

**R: Local foods… apple, orange, banana**

**I: What food should you not give to children under 2 years and why?**

**R: Because they’re healthy**

**I: Okay but the question says, what foods shouldn’t you or should not give to your children?**

**R: …**

**I: To make the question easier. What kind of food don’t you want to give him to eat?**

**R: Things like chips, lollipop, gum and chocolate**

**I: So why don’t you want to give these things to them?**

**R: Because they’ll lose their appetite, their body is good (skin rashes) and not heavier (big)**

**I: Okay. What the biggest influenced on feeding the children?**

**R: Really feed so that they get big and healthy.**

**I: Now can you tell me the difference between feeding your son from your daughter... For example: is there any difference in feeding your son than your daughter?**

**R: No they have to be the same**

**I: Why should they be same?**

**R: Because I love my kids so I have to make it fair.**

**I: That’s really good. : We are also interested in the roles and responsibilities different family members play in raising children. We want to know what the responsibilities the family have in raising the children. Now the question is asking, could you describe the care of children throughout the day in your community? How do people look after their children in this community?**

**R: … Watch the kids so they don’t touch the dirt and dirty water. Play near the road and touch the dirty walls.**

**I: Good. Now is there any other reason umm ways to watch other than when they are watching them from touching the dirt watching them from going near the road are there any other reason?**

**R: No**

**I: Now who is mainly responsible in watching the kids?**

**R: The mother and father**

**I: Now can you really explain why you say the mother and the father?**

**R: Because they know how to watch them, they’re learning.**

**I: Are there any other reason?**

**R: No**

**I: Now but what is the responsibility of a mother… what is your responsibility to your son?**

**R: I give love to him, protect him and teach him.**

**I: Now when you say teach him, teach him what?**

**R: Culture, don’t beat up people…**

**I: And what is the responsibility of a father to the child?**

**R: Bring them and feed them and then… protect them, love them**

**I: So how does caregiver play with children under 2 years?**

**R: They sing, read…**

**I: Now if you were to for example; how do you play with your son?**

**R: How do I play with them?**

**I: hmmm (yeah)**

**R: make him laugh, play with them, sing to him, tell story to him and talk to him.**

**I: Good. Those are really good. Now could you tell me the responsibilities of the grandparents in watching the children in the community?**

**R: Love them, teach them, play with them, bring them and feed. Teach them the culture.**

**I: Good. So how does the grandparents help in raising the child(ren) and also help the mother and father of the child(ren)?**

**R: … Help them, teach them…**

**I: okay. What make a good grandparents?**

**R: They love them and teach them the culture.**

**I: Good. Now can you tell the responsibility of the other family member in raising the children in this community?**

**R: Watch them, prevent them from touching dirt, sing to them, read to them and love them.**

**I: Now how does the older siblings look after the children?**

**R: They love them, play with them and make them laugh.**

**I: Yes. You’re answers are really good. Your answers will help us find ways to develop health program for the children and mothers. We’re almost done. In this last section, we want to know how to know how to develop health programs in this community.**

**I: Can you explain where you get these information about nutrition and health from?**

**R: From the stores, hospital, radio, the schools and the church**

**I: Oh okay. Now why do you trust where these information comes from?**

**R: Because there are some kind of foods and… because it’s the hospital.**

**I: If it’s the hospital, why do you really trust the hospital?**

**R: Because there are certain food for diabetic and it a place for treatment.**

**I: Now where can these information go to so that it can be easy seen and listen to?**

**R: Radio, school, churches, hospital and the stores**

**I: Good really good. Now why do you want these information to go there?**

**R: So all the people can hear and know where it is.**

**I: When you think about your own parenting, can you explain what influences how you raise your children? Can you explain the difference in how your raise your children and how other raise their children?**

**R: …**

**I: Do you understand?**

**R: Not really.**

**I: Can you explain how your raise your son?**

**R: How I raise my kid?**

**I: Yeah. How is it that the way you raise your son is different from how others raise their kids? Do your raise your son different from the other girl?**

**R: Yes.**

**I: Now what is the difference?**

**R: Because they don’t look after them but I watch mine, I show my love and teach him.**

**I: Good. What about the opinion of others in this community regarding raising children or how you raise your child. If it was the alap (land owner) or the pastors of the church, your neighbors or the health worker. What do they think about raising your child?**

**R: They make sure that we’re really watching our kids. They come and teach the people.**

**I: Are there anything else?**

**R: No**

**I: Were there any advice or information you learned from parenting?**

**R: There are. Parenting is not easy… we stay up late with them and when they’re sick we care for them.**

**I: Now are the advice or information they give you?**

**R: I don’t think so.**

**I: Who and where did you get these information from?**

**R: Hospital, our family**

**I: Now what kind of information do they give you?**

**R: Food and Health**

**I: What about the family, what kind of information do they give you?**

**R: To feed them and watch the kids**

**I: Are there anything else you want to know about parenting you want to know that you don’t know about?**

**R: Say that again?**

**I: Are there anything else you want to know about parenting that you don’t know about and you want to know?**

**R: regarding parenting? I watch them, protect them…**

**I: Is there any other information you want to know that we didn’t talk about?**

**R: No**

**I: Okay thank you. Thank you for giving this time. Hopefully all the information you provided today will help develop program for mothers and children. Once again thank you.**
