# Supplementary material for: Conceptualizing multi-level determinants of infant and young child nutrition in the Republic of Marshall Islands–a socio-ecological perspective
Source: PLOS Glob Public Health. 2022 Dec 19;2(12):e0001343. doi: 10.1371/journal.pgph.0001343 (PMC10022247; doi:10.1371/journal.pgph.0001343)
Supplement: S1 Data — (ZIP) [file pgph.0001343.s001.zip › RMI Supp Data/Interviews data/I26U_IDI_FCG_Rita_Aug 20_Libon.docx]

- Interview code: I26U
- Interview type and interviewee: IDI_FCG
- Interview date: Aug.20.2018
- Location: Rita
- Interviewer: Libon
- Transcriber: Marcellina

**I: are you giving me your consent to do this survey?**

R: yes

**I: okay before we start, I want to thank you first for giving me this wonderful time to negotiate with you. The information we’re going to learn from you will help us find ways to improve maternal and child health and sanitation in your community. Now the first question is asking, can you please tell a little about your family/household? Like who live in this household, how many children and their ages and gender**

R: my name is Limen and my husband name is Jeremy and we live with our grandmother. We have two children, 1 girl and 1 boy. One is 6 years old and the other is 1 year old

**I: now, can you describe your community? Like what are the good or the bad things about this community**

R: the bad things about this community are, it’s not clean, lots of people, lots of drunkard, no community playground, and it’s not peaceful every time. The good things about it are, it has a church where we can gather and do praise meeting, and the women in this community cooperate with each other. Like if there is a birthday party at our relative’s house, we will all go together and join the birthday party.

**I: Let’s now talk about health and illnesses in your family. Can you tell me about some of the illnesses that your children have suffered from?**

R: stomached, coughing, fever, runny nose, headache, and diarrhea

**I: now what you think are the causes for these illnesses?**

R: when they play outside a lot and when they are curse

**I: what is the seriousness of each illnesses?**

R: for diarrhea, they don’t eat but drink only. For fever, they like to just sleep. and when they sleep, they don’t sleep well because of difficulty in breathing through the nose.

**I: are there ways for you to prevent these illnesses?**

R: seek doctors, but if we don’t have cash for the taxi cab medical fee, we will do home medical care. Like if they have high fever, we will reduce their fever by applying wet clothes all over their body and get what they like to eat so that they won’t have empty stomach.

**I: now, can you explain how do you know when your children need treatment for their illnesses?**

R: when their illnesses get severe. Like don’t eat at all, and they sleep a lot.

**I: who do you first go to when your children are sick and why?**

R: my grandma and then she will tell me what to do next. And I seek her first because she knows better and a lot about parenting.

**I: now, do you use traditional medicines when your children are sick?**

R: yes, I do use traditional medicines when my children are sick. I use them for like swollen and those kinds of illnesses that need to be heal by traditional medicines.

**I: so, you mentioned swollen, can you explain what kinds of swollen and how do you use traditional medicines to heal it?**

R: like swollen stomach. And they apply body oil to the stomach using bare hands and rubbing it up and down.

**I: how do they know that the children have a swollen stomach?**

R: when they have the symptoms of swollen stomach. Like on and off fever, they don’t eat, and they sleep a lot

**I: Can you describe any illnesses affecting your children that are associated with nutrition?**

R: there are none. My children don’t get sick when they eat nutrition foods

**I: can you describe any illnesses affecting your children caused by foods missing from the diet?**

R: malnourish, and lose weight

**I: now, we talked about a lot about being unhealthy. Could you now describe for me a typical day of someone living a healthy lifestyle, from the time they wake up in the morning until when they go to bed?**

R: they look energetic, they eat from all the three group of nutrition, they exercise a lot, and they have enough time to sleep

**I: can you tell me what are the appearances or signs of a healthy child under 2 years?**

R: they play a lot and move a lot, they have healthy body shape, and they always smile

**I: now, what are the appearances or signs of a healthy adult?**

R: they look joyful, have sexy healthy body shape, and look energetic

**I: now let’s talk about hand washing. Could you describe in detail your family’s hand washing throughout the day?**

R: before we eat we wash our hands, after we used the rest room we wash our hands, and after the children done playing they wash their hands

**I: can you describe the ways your children wash their hands throughout the day?**

R: they wash their hand before they eat, after they use the rest room, and when they came back from school

**I: good. Now, can you describe the hand washing throughout the day for children under 2?**

R: we adult help them wash their hands. We wash their hands wit soap, hand soap, or sometimes we let them use hand sanitizer

**I: now, can you tell me what are the differences between washing hands using water only and water with soap?**

R: when we wash our hands using water only, it doesn’t help killing the germs in our hands. Washing our hands using water and soap helps with killing germs that are in our hands

**I: can you tell me what does prevent you from washing your hands using soap throughout the day?**

R: only thing that prevent me from washing my hands using soap is, when I am in a rush and I forgot to use the soap.

**I: We would like to talk about your diet during pregnancy and breastfeeding. Now I would like you to think back to when you were pregnant. Can you describe your diet when you were pregnant compared to when you were not pregnant?**

R: when I was pregnant, I ate mostly fish and rice. And when I was not pregnant, I ate mostly everything!

**I: what made you want to eat these foods you mentioned?**

R: well I think it was the baby that made me carve for those foods

**I: what kinds foods you were encouraged to eat during your pregnancy and the reasons why?**

R: fruits like apples, bananas, and oranges because they said they’re good for me and my baby’s health

**I: what kinds of foods you were encouraged not to eat during your pregnancy and the reasons why?**

R: rice, salty foods, sodas, greasy foods, and chips because they said they will affect my baby’s health.

**I: who encouraged or discouraged you to eat these foods during your pregnancy?**

R: my grandmother, husband, and the doctors

**I: now, who helped and supported you during your pregnancy?**

R: my grandmother and my husband. Sometimes my mother

**I: can you describe how they helped and supported you during your pregnancy?**

R: they support with my foods, my drinks, give me advices, and especially my medicines

**I: If you were advised to eat more fruits and vegetables during pregnancy, could you describe what would make this difficult?**

R: money. I can’t afford to buy any since they are expensive.

**I: what would make it easier to eat more fruits and vegetables and reasons why?**

R: make our own garden, and reduce their prices

**I: can you describe the foods you ate when you were breastfeeding?**

R: I usually ate fish because it produces breastmilk

**I: what kinds of food you were encouraged to eat when you were breastfeeding and reasons why?**

R: fish, can meat like Saba mackerel and corn beef, fruits and vegetables like carrot, papaya, and banana. And they told me eat these because they are healthy for my baby and they produce breastmilk.

**I: what really inspired you to eat these foods during breastfeeding?**

R: to make sure my baby has enough breastmilk, so that my baby will grow healthy, and so that my baby will not likely to have sicknesses

**I: what kinds of food you were encouraged not to eat during breastfeeding and the reasons why?**

R: chips, sodas, Kool-Aid… and they told me not to eat these because they are not healthy for my baby and they are most likely to cause illnesses to my baby when he/she grows.

**I: who really encouraged or discouraged you to eat these kinds of foods during breastfeeding?**

R: the doctors and the ones I am living with

**I: after giving birth, would you describe how you breastfeed your child throughout the day?**

R: before I breastfed my baby, I first drank fresh milk. And when she/he cried, I brought him/her and breastfed him/her.

**I: can you give me the reasons why did you drink fresh milk first before you fed you child?**

R: to help produce breastmilk

**I: now, who inspired you to drink fresh milk before you fed your child?**

R: the nurses when they brought my baby to me

**I: now can you tell me how long after giving birth you started breastfeeding your baby?**

R: after I gave birth, they took my baby and clean her/him while they took me to one of the rooms in Maternity ward. After they clean my baby, she/he falls asleep. And maybe hour later she woke up and cried. That’s when I started to breastfeed her even though my breasts haven’t had milk yet.

**I: can you tell me did you give any liquid or bottle milk to your baby in the first few days after giving birth?**

R: I did not because it’s one of the policies in the Maternity ward. We’re not allow to give bottle milk to our baby. It was really hard for me and my baby because it took so long for my breast to produce milk.

**I: were there any traditional medicines your baby drinks while she/he was in the hospital?**

R: there were none

**I: what about the time when you and your baby went back to your house?**

R: when my baby and I got out of the hospital we went back to my house and days after, my baby drinks some traditional medicines

**I: can you tell me the reasons why your baby drank traditional medicines and what kinds of traditional medicines?**

R: it’s Marshallese traditional medicines that help make the baby cry less, have strong and healthy body

**I: now do you know what do they mix with the traditional medicines?**

R: yes, water. they mixed it with water. they called it UNO IN KIJON (the name of the traditional medicine)

**I: now what are the ingredients for the UNO IN KIJON?**

R: usually local medicines (NIN [noni], KIEB, that kind of GRASS which is eaten by the chicken, and EKKON)

**I: now what made it difficult or easy for you to fed you baby from the time she/he was a baby until 6 months old?**

R: the only difficulty was my baby didn’t have enough breastmilk. What made it easy was that my baby only loved breastmilk. She/he doesn’t want other foods.

**I: what do you mean when you stated that your baby doesn’t like other kinds of foods other that her own food? What do you mean by her/his own food?**

R: I only breastfeed him/her only

**I: now did you faced any difficulties or was it easy for you to breastfeed your baby up to 2 years old and why?**

R: there wasn’t any difficulties during breastfeeding time for both of us

**I: what about the easy things during that time?**

R: it was good because I could just grab her/him whenever and breastfeed him/her

**I: Now as a worker, were there any difficulties for you to feed your baby at the time you were working?**

R: there were no difficulties because my workplace was close to my house, so it didn’t take too long for me to go back to feed my baby

**I: Could you tell me when did you first gave foods and liquids other than breastmilk to your child?**

R: I started gave foods and liquid to my baby when she/he was 6 months old. The first food I gave is baby foods.

**I: as you said baby food, can you tell me what kinds of baby food. The ones you made by yourself or the ones buy from the stores?**

R: the ones we buy the stores

**I: can you tell me the flavors of the baby food?**

R: banana, mixed vegetables, and apple. I usually chose the fruits and vegetables flavor than the meats flavors.

**I: can you tell me the reasons why you introduced foods or liquids other than breastmilk at that age?**

R: because according to the baby chart, it shows that we can feed our baby at that age. And the nurses told me too

**I: now, beside people telling you to feed your baby at that age, are there any other reasons why you started feed your baby at that age?**

R: yes. Because there wasn’t enough breastmilk anymore to feed my baby

**I: now, what kind of opinions from others that influenced their decision to introduce foods and liquids at that age?**

R: they also believe in the fact that baby should be feed at that age. And, they wanted to feed their baby at that age because they want their baby to grow well and healthy

**I: what were the first foods you fed you baby and how they were prepared?**

R: first food I gave to my baby is baby food and water. I washed my hands first before I fed my baby.

**I: so, as you mentioned before the different kinds of flavors of the baby food you feed your baby like banana, mixed vegetables…. Do you mix them all together and feed your baby with?**

R: yes, I do. When I feed her/him banana, I will mix it with mixed vegetables, so she/he will like it. So, when I mix them all together, she really loves it!

**I: now, at the time your baby started eating baby food, can you tell me exactly how many bottles/trays of baby food your baby eats in a day?**

R: usually one. Let’s say two because I combine two flavors at a time.

**I: We are trying to understand how people eat in this community. Could you describe in detail what your family usually eats and drinks throughout the day?**

R: we usually eat rice and chicken (fry, boiled, stew). Sometimes we eat can meats like tuna, Saba mackerel, corn beef, and other can meats

**I: now can you describe the process of how the meals are made?**

R: before the foods are cook, we wash them first and then cook them. Rice for example, we wash the rice first and then add water to it and then put it the rice cooker to cook. For the chicken meat, if its frozen we put it in water and when its not frozen anymore, we take them out and cut them in pieces. If we planned to cook them with soy sauce, we will boiled them first with water for about 5 minutes and then pour out the boiling water. after we poured out the boiling water, we add water again and soy sauce too and let them boil again until they are fully cook.

**I: are there any ingredients added beside the soy sauce?**

R: yes. Cabbage and onion if we have.

**I: can you tell me who in the family is served first, next, and last?**

R: usually our children served first. Next will be my husband and my grandmother. And I will be the last to be served.

**I: are there any differences in the foods served to different family members?**

R: no there are none. If chicken meat is for dinner, everybody will eat chicken

**I: are there any differences in quantities of food served to different family members?**

R: well yeah. Children served less than adult because you know, they are kids and they don’t eat as much as adult.

**I: are there any children receive more food than others?**

R: yes, there are! There are some children in this family that really love foods, so they eat more than the others

**I: Now could you describe any food sharing between family members in this family during mealtimes (for example children eating together separately from the family, meals eaten from the same plate by all family members)?**

R: children eat separately from their parents and they eat alone by their own plates. And so, the adults in this family.

**I: do your family share their food to their neighbors?**

R: yes, sometimes if we have enough to share or when we celebrate birthday party or we when we cook rarely eaten foods like barbeques.

**I: We have heard that some families eat local foods whereas others eat processed foods. Could you explain what is typical for your family?**

R: my family prefer local foods and we usually eat local foods like taros, breadfruits, pandanus, and other local foods that we know that people from outer islands usually eats. But sometimes we eat processed foods like apples, steaks, and usually chicken if we don’t have local foods anymore.

**I: now can you tell if there are anything that makes it difficult or easy to cook local foods?**

R: as we know that this island has become one of the centers of the Marshall Islands and the more of the processed foods are imported, the less are the local foods. So that’s the difficulty. Sometimes it’s easy to cook local foods when our family members from outer islands send some to us.

**I: now what are the good or the bad things about eating local foods?**

R: the good thing is they are very healthy, they won’t cause illnesses like diabetes. The bad thing is not everybody have local foods grow in their area. And they are expensive when we buy them from the stores.

**I: what are the good or the bad things about eating processed foods?**

R: the bad things about processed foods are they can cause diabetes, cause high blood pressure, some are expensive. The good things are they are close to us not like local foods, and we don’t have to put much effort on prepare or cooking them. Unlike local foods, we do a lot of work to cook them.

**I: Now that we’ve talked about how the family eats, I would like to learn more about how your child eats. Could you describe in detail what your son/daughter under 2 years commonly eats throughout the day?**

R: you know my child was born in the outer Islands, so he/she prefers eating local foods than processed foods which we usually eat here. If I give her/him bananas, she/he will eat them until there are no more. But if I give her rice and chicken, she/he will refuse to eat it. For snacks, our older kids sometimes buy him/her chips and plus(juice)

**I: can you tell me how many times a day you feed your child which is under 2, his/her meals and the snacks?**

R: my baby will have all the three meals but will also eat whenever he/she sees someone eating even though he/she just finish his/her regular meals.

**I: now can you tell me how do you know when your child has had enough to eat?**

R: before I feed my child, I will put him/her on my laps, so he/she won’t walk around while eating because he/she might lose his/her appetite and I might think that she/he is full already. So, when I put him/her on my laps, she/he will eat until he/she had enough. I can tell that she/he is full already because she/he will push my hands away when I try to give him/her more.

**I: what do you do when your child doesn’t eat?**

R: I usually look for foods which will get him/her to eat. Like I will buy what he/she wants to eat.

**I: what do you do to encourage your child to eat when she/he refuses?**

R: when my child refuses to eat, I usually give some time to let him/her have whatever she/he wants at the moment. And then whenever I try again, she/he doesn’t refuse. But if she/he still refuses, I will try give him/her something else.

**I: are there any differences in feeding your child when she/he is sick? Like for example, when he/she has diarrhea.**

R: yes. When my baby has diarrhea, he/she hates foods or doesn’t want to eat at all.

**I: okay, you’ve told me what your child under 2 usually eats. Now could you explain to me the process, from start to finish, how you prepare and cook a meal for your child?**

R: before I cook a meal for my child and before I feed him/her, I wash my hands and his/her eating utensils just as plate and cup.

**I: now, what kinds of foods you usually prepare or cook for your baby?**

R: I usually prepare him/her soft foods. For example, when I give him/her rice (already cook) I will add water to make it soft and then add raw eggs to make it softer.

**I: Could you now tell me what you think are the important foods for children under 2 years to grow well/be healthy?**

R: I think those foods that have lots of nutrient in them. For example, oranges and banana.

**I: can you tell me what kind of foods that should not be given to children under 2 and the reasons why?**

R: those kinds of food that are lack of nutrients. For example, ramen and junk foods.

**I: now can you tell me what is the biggest advice on feeding your child?**

R: wash my hands before I feed my child and wash my baby’s eating utensils before use them.

**I: Can you describe any differences (if any) between how you feed your son and how you feed your daughter under 2?**

R: yes, there are differences in feeding them. As for my daughter, she eats almost every kinds of food. Local foods and processes foods as well. My son, he likes to eat local foods onnly.

**I: We are also interested in the roles and responsibilities that different family members play in raising children. Could you describe the care of children throughout the day in your community?**

R: those children in this community stay awake until maybe at midnight. So, I don’t know how their parents take care of them. It’s like they don’t care about their children being absent from their house at night until midnight.

**I: can you tell me who is mainly responsible for the child care?**

R: the mothers

**I: okay, can you tell me the responsibilities of the mother in taking care of their children?**

R: they take care of them, protect them from danger, feed them, make sure they stay clean, and teach them to be good.

**I: what the fathers. Can you tell me their responsibilities in taking care of their children?**

R: they help their wife looking after the child when the mother is busy with household chores, support with foods and clothes, and give advice to their children.

**I: okay. Now can you describe how the caregivers play with the children under 2 years?**

R: they play with them hide and seek, run around with them, tickle them, read with them, sing with them, and usually watch movies with them.

**I: can you tell me what are the roles of grandparents in raising children in this community?**

R: taking care of them, protect them from danger, and teach them about our culture and how to treat old people politely.

**I: now can you tell me the ways that grandparents support mothers and families in this community?**

R: they help us with babysitting our children, and give advice to our children

**I: from your own knowledge, what makes them good grandparents?**

R: it’s the way they treat our children and how they teach our children.

**I: Could you talk about the role that other family members have in raising children in this community?**

R: make sure that the children don’t play near the road, don’t stay awake until midnight, don’t play gamble, make sure they stay clean, and make sure they go to school.

**I: can you tell me the ways that older siblings help in raising young children?**

R: they also help in looking after them, play with them, make sure they stay safe and clean.

**I: You are doing a great job. We are almost finished. Now for the last section, we would like to learn about ways we can develop health programs in your community. Could you explain where you usually get trusted information about nutrition and health?**

R: from the hospital

**I: can you tell me why this source is trusted?**

R: because it is usually where I get that kind of information from and they know more nutrition and health

**I: okay can you tell me where nutrition and health messages should be delivered so that you would see/hear them most easily?**

R: radio station, and Facebook

**I: now can you tell me what types of media that you use the most to communicate (eg. radio, online apps, websites)?**

R: online apps like Facebook

**I: When you think about your own parenting behaviors, can you explain the influences on how you raise your children?**

R: [01:25-01:26] *sound of the wind is stronger than the respondent’s voice

**I: okay. Can you tell me the opinions of the community leaders on how you raise your children?**

R: they usually told me that my baby looks healthy. It’s like they want to praise me or something[laughing]

**I: are there any advice or information related to parenting you have received or you have learned from?**

R: yes, there are. [01:28:07-01:28:38]* didn’t get the rest of the respondent’s answer because of the sound of the wind.

**I: okay. So, where and who the advice or information came from?**

R: from the nurses in the hospital

**I: are there any desired information on parenting you wish to have but doesn’t available?**

R: no. there is none
