# Supplementary material for: Conceptualizing multi-level determinants of infant and young child nutrition in the Republic of Marshall Islands–a socio-ecological perspective
Source: PLOS Glob Public Health. 2022 Dec 19;2(12):e0001343. doi: 10.1371/journal.pgph.0001343 (PMC10022247; doi:10.1371/journal.pgph.0001343)
Supplement: S1 Data — (ZIP) [file pgph.0001343.s001.zip › RMI Supp Data/Interviews data/I27U_IDI_FCG_Rita_Aug 20_Meia.WMA.docx]

- **Interview code: I27U**
- **Interview type and Interviewee: IDI_FCG**
- **Interview Date: Aug 20**
- **Location: Rita**
- **Interview: Meia**
- **Transribe: Meia**

**I: To begin with, can you please tell me a little about your family/household?**

R: My family don’t have enough of everything in life.

**I: Can you tell me who live in the household and how many kids and their gender?**

R: My aunt, my uncle and I live in the house. And there are four children, my one years old boy and two girls.

**I: Next, I’d like to ask you to describe your community? what are negative and positive of the community?**

R: Well, as you can see around my community is not good because it dirt every where.

**I: Are there any more negative about your community?**

No answer.

**I: What are the positive?**

R: The negative thing is that people cooperate with one another (looking after one another).

**I: Let’s now talk about health and illnesses in your family or other family in your community. Can you tell me about some of the illnesses that your children have suffered from?**

R: Only fever and cough.

**I: Are there any other illnesses that kids in this community have suffered?**

R: Some are polio and some are pink eye.

**I: Are there any other illnesses?**

R: Some children are slow learner.

**I: Okay, so you mention blind What make the child pink eye?**

R: Dirt

**I: Can you explain it a little bit more?**

R: The child play where it dirty and like rub their eyes with their dirty hands.

**I: About polio? What makes a child polio?**

R: From what I know, the child has from when he/she was born.

**I: So, you mention that your child sometimes has fever and cough, can you tell me the causes of these sicknesses?**

R: Maybe because I always let him nap or sleep naked while sleeping using electric fan.

**I: Okay, about slow, can you tell me what makes a child slow learner?**

R: I don’t know.

**I: How can you tell that a child is slow learner?**

R: Because sometimes when you speak to a child he/she might not register what you are saying until you repeat it again. And it might have come from the mother who took drugs.

**I: Good, thank you, can you tell me if these illnesses that you mention are serious? You mention fever, polio, cough, and slow learner?**

R: Yes.

**I: Can you describe why you say these illnesses are serious?**

R: Fever for example my child might have high temperature, so it might cause him to freeze.

**I: What about the other illnesses, you mention polio, cough and slow learner, are these illnesses serious?**

R: well, from what I heard the doctor if a child is coughing, we brought him/her to the hospital because if the child cough for a long time it might cause pneumonia and the child might die.

**I: What about the other illnesses that we mention?**

R: I think for pink eye some child might have infections and damage their eyes.

**I: Okay, so let talk about how to we prevent these illnesses we mention pink eye, polio, fever, cough and slow learner. Are there any methods you can do to prevents these illnesses?**

R: We really had to take care of our kids.

**I: Can you explain what you mean by really take care of our kids?**

R: we had to check where they are playing, watch them from touching their eyes so they might have pink eye.

**I: What about slow learner, could you explain what we can do to prevents a child from slow learner? You mention that child that become slow learner it can be because of the mom, so what can a mom do to prevent her child from become slow learner? For example, if it were me, to prevents a child from becoming slow learner a mother had to quit smoking during pregnancy, so can you tell me any other reasons that a mother can do protect her child?**

R: oh, just like you said, a mother had to stop using drugs.

**I: Can you describe how you know when your child needs treatment for their illness?**

R: When my child starts to get sick, I bring him to the hospital.

**I: Can you tell me who you first go to when your child is sick? You mention you bring him to the doctor, so can you explain why you bring him to the doctor?**

R: For the doctor to check him up with his illness and treat it.

**I: Can you tell me if you are using traditional medicine?**

R: Yes. Sometimes.

**I: Can you please explain more about how you use Marshallese medicine and what kind of illnesses you are use traditional medicine and why?**

R: I use traditional medicine when sometimes like for example my kid if he has high temperature he is coughing and It difficult to go see the doctor because I have no taxi fair, I used Marshallese medicine.

**I: Can you tell what kind of traditional medicine you use for the high fever? And how to you use it?**

R: I use the leaf of a flower tree we call meira (it a white flower tree). So, I took off 9 green leaf of the flower, put the flower(meria)’s leaf on his bed then laid him on them, I arrange the leaf so when I laid him on his back or front the leaf should cover all his back and front for it like the leaf are absorb the fever. And when you take off the leaf from his bed they look dead because his fever burned them.

**I: Are there any other illnesses that you use Marshallese medicine?**

R: Yes, Like cough.

**I: Can you describe how you treat cough using Marshallese medicine?**

R: I used noni tree, I took tree small noni’s fruit the one that have tree flowers in it, I crush them together and put it in a small cloth and give him three drops of it juice.

**I: Do you mix it with water?**

R: No, I don’t.

**I: you just give him drop of it?**

R: yes.

**I: Any other illnesses used for these medicine for your kid?**

R: Stomach pump, if, he fells down I gave him stomach massage.

**I: Thank you for these good answers, Let go to question number 5. Can you describe any illnesses that affecting you child associated with nutrition?**

R: no answer

**I: Can you describe any illnesses caused by foods missing from theirs diet?**

R: skin infections and boils in their head.

**I: We talked a lot about being unhealthy. Could you now describe for me a typical day of someone living a healthy lifesty**le, **from the time they wake up in the morning until when they go to bed?**

R: He/she is moving around and working all the times.

**I: Can you explain what does he do from when he wakes up until he goes bed, tell me a story about what does a healthy person do through out the day? Do you understand the questions?**

R:no

**I: Can you tell me, what does a healthy person do through a day like for example, a healthy woke up do all his/her morning and prepare breakfast and stuff like that and you go on with whatever a healthy person is doing through the day and he/she doesn’t look sick. So, I am asking you how does a healthy person be through a day?**

R: How will I explain how a healthy being?

**I: For example, for an unhealthy person we can tell because the person looks grumpy, he/she look unhappy and sleepy all the times and does look well. But a healthy person can do anything so can you explain what a healthy person is doing to show that he is doing well?**

R: I have a grandma who just left from here, every early morning she woke up prepare breakfast, doing laundry and cleaning the house, she was very active.

**I:** **Can you explain the appearance/signs of healthy child under 2?**

R: He doesn’t look sick and he is not lazy.

**I: What else does he do?**

R: He always playing around.

**I: What about the appearance of a healthy adult?**

R: They have happy faces.

**I: What about their actions?**

R: no answer…

**I: Let now discuss hand washing. Could you describe in detail your family’s hand washing throughout the day?**

R: We wash our hands with soap only.

**I: Could you describe more in detail how you wash hands?**

R: We usually wash our hands before eats but it we rarely used warm water to wash our hands.

**I: Can you tell me how children in your household wash their hands throughout the day?**

R: I wash my baby’s hands, I wash it with soap and water.

**I: when did you wash his hand?**

R: After he play and before his meals.

**I: Can you tell me the different between washing hands using water only or water and soap?**

R: Base on my knowledge, I think washing hands with water is like the germs in your hands are not died and you are not getting of the germs. But with soap and water the germs are like gone.

**I: Can you tell me anything that prevents you from washing hands with soap throughout the day?**

R: Base on my wants, if I want to I wash my hands soap but if I don’t well…

**I: If you don’t want to wash your hand you don’t?**

R: Yes, I don’t.

**I: Thank you for being honest. Now I would like you to think back to when you were pregnant. Can you describe what you eat when you were pregnant compared to when you were not pregnant?**

R: Sashimi only, some local foods.

**I: Local foods? What kind of local foods?**

R: Breadfruit and bananas.

**I: And sashimi, right? you also mention sashimi?**

R: Yes!

**I: What makes you want to eat these foods?**

R: I curve for them.

**I: What kind of foods were encourages for you to eat during your pregnancy?**

R: Food like nutritious foods.

**I: Why were you encourage to eat these foods?**

R: To helps my unborn health.

**I: What kind of foods that were not encouraged for you to eat during your pregnancy? Like what foods they didn’t want you to eat?**

R: Ramen with kool-aid.

**I: What else?**

R: Uncooked rice. (laugh)

**I: Why did they not want you to eat these foods?**

R: Ramen with kool-aid for example, they it might destroy the baby’s skin.

**I: What the uncooked rice?**

R: Uncooked rice can like make the skin of the baby dirty when he/she born, and the baby might not enough blood.

**I: Who support you during your pregnancy?**

R: My mom

**I: Can you tell me how did your mom support you?**

R: She supported me by all my needs. When I want something, she bought it.

**I: Can you tell me if you took any supplement during your pregnancy?**

R: Those vitamins for babies and medicine for blood.

**I: Can you tell me if you took all these medicines? Why you took and not took these medicines?**

R: I didn’t take them?

**I: Can you explain why you didn’t take them?**

R: Because whenever I took them I feel

**I:** **Did you try to take another medicine?**

R: Only medicine for blood.

**I: you took it? But the vitamin you don’t?**

R: Yes.

**I: Could you explain to me if you have drinking alcohol, smoking or take any other drugs during your pregnancy?**

R: Only smoking.

**I: Can you tell if you use traditional medicine during your pregnancy?**

R: Yes.

**I: Okay, can you explain what kind of medicine you use and why?**

R: I went to the hospital for a check up and the doctor say that my baby wasn’t in the right place, so I went home and asked my grandma what I can do for my baby wasn’t in the right place, she said to me to drink medicine I don’t because she was the one making the medicine.

**I: For?**

R: For the baby returned to the right.

**I: Is there anything she did to you?**

R: She has me stomach massage me.

**I: She stomach massage you and let you drink medicine, that good let go to the other question, if you were advised to eat fruits and vegetables during pregnancy, could you describe what make this difficult?**

R: It not for my liking.

**I: But if you were like likes them, could you have eaten them?**

R: Well, yes.

**I: Let move on to the next question, now can you describe your diet when you were breastfeeding?**

R: Fish and …. I usually eat fish.

**I: Only fish?**

R: Yes.

**I: Can you tell me what influenced you to eat fish during breastfeeding?**

R: So, the baby had foods.

**I: Can you tell me the foods you were encouraged to eat during breastfeeding? If there were anybody tell you to eat certain food, what kinds of foods?**

R: No answer mmmm…

**I: If there anybody who encourage you to eat certain foods. What kind of foods they encouraged for you to eat during the time your breastfeeding? Was there anybody encourage you?**

R: No body.

**I: Can you describe any foods you were not encouraged to eat during breastfeeding?**

R: Salty.

**I: Can you tell why do they tell you not to eat salt?**

R: Because they said it gonna affect the baby.

**I: Can you tell me who encouraged you or discouraged you to eat salt?**

R: Some of my neighbor.

**I: After giving birth, could you describe how you breastfeeding your child throughout the day?**

R: I sit, and breast feed him.

**I: My question exactly is how, like for example did you have to wait for a day to breastfeed the baby or?**

R: I breastfeed him right away.

**I: Now I want you to tell me did you give him any bottle or other liquid other than the breastfeed?**

R: No, I think I didn’t.

**I: You didn’t?**

R: Hmmhmm

**I: Did you give him any thing at all, this question I want to know if you give him any bottle or anything after giving birth, like our Marshallese medicine for example?**

R: Can you repeat the question again?

**I: Okay, could you tell me if you had giving the baby any other liquid other than the breastmilk? For example, did you give him traditional medicine?**

R: Oh! Yes.

**I: Can you explain?**

R: Medicine for kijonkan (when a child has yellow skin or eye and it can be when the child can’t sleep peaceful and when the child is always stretch up during the first few mean of his/her life after birth). I let him drink it.

**I: Can you tell me a story of how you made him drink the kijonkan medicine?**

R: I took something we call kaboj form the kiop flower[ a Marshallese flower(kaboj is like a small pump in the bottom of a kiop, it not the roots and you can see it clearly in the kiop’s stem, so to take it out you peel out the stem of the kiop till you see the kaboj which start to come out of the kiop’s stem or grow our of the stem of a kiop.)] I use it, I take one for a day I grind it and put three drops in is mouth. I use it three times a day. I continue this medicine for three days.

**I:**  **Can you tell me if you give him any other traditional medicine in his mouth?**

R: There were many medicines.

**I: What kind of medicine?**

R: Medicine for him to no get sick frequently.

**I: Can you explain, how you do it and how long you do it?**

R: It takes a week for this kind of medicine.

**I: What did you do with the medicine?**

R: I used kone tree, I used the leaf for a medicine.

**I: Can you describe how you made the medicine?**

R: I took three leaf of the kone tree and grind it then I drop the liquid of in to my baby’s mouth, but I mix.

**I: What did you mix it with?**

R: I mix it with my breastmilk.

**I: okay! Thank you. Now could you tell what makes it easy or difficult for you to breastfeed you baby exclusively up to 6 months?**

R: Sometimes it difficult because I sometimes feel pain in my breast and I didn’t breastfeed him, but I use the other breast when it okay.

**I: Now can you tell me is it easy or difficult for you to breast feed you baby up 2 years and why?**

R: Yes, it easy.

**I: Why?**

R: For my baby to have healthy life.

**I: Could you tell me when you first gave foods and/or liquids other than breastmilk to your child?**

R: Like I mention before that I gave medicine. I gave him after he was born.

**I: When did you start giving foods?**

R: when he was 6 months old.

**I: What kind of foods?**

R: I feed him baby food.

**I: Baby foods that you bought from the store?**

R: Yes.

**I: Why did you gave him baby foods at that age?**

R: Itis ours believe that a baby eats at that age.

**I: Can you describe any opinions from others that influenced their decision to introduce foods and liquids at that age?**

R: There are none.

**I: How did you prepare his foods?**

R: I mix the baby foods like beef with bananas.

**I: We are trying to understand how people eat in this community. Could you describe in detail what your family usually eats and drinks throughout the day?**

R: Today we eat pancake for breakfast and for lunch we eat rice with fry chickens and we drink water sometimes coffee.

**I: Now could explain you prepare the foods? How you prepare your breakfast lunch and dinner?**

R: Like I said we eat pancake this morning, we mix the flour and cooked it.

**I: Can you tell me the ingredients?**

R: Because we are few in this house we make only three cup of flour, 6 teaspoon of baking powder and half cup of sugar.

**I: To bake a pancake?**

R: Yes.

**I: What about to drink?**

R: coffee

**I: About the kids, are they drinking coffee with adults?**

R: Only the child drink water.

**I: Who in the family is served first, next and last?**

R: Children, I always gave kids their meals first.

**I: Can you tell me whether there are differences in the foods served to different family members?**

R: I think there are none.

**I: Okay. Can you tell me if some children receive more foods than others?**

R: Yes

**I: Can you explain?**

R: For example, the girl who is in elementary school, she has lot more food from my baby because she eats more.

**I: Now could you describe any food sharing between family members during mealtimes (for example children eating together separately from the family, meals eaten from the same plate by all family members?**

R: Separately.

**I: Could you explain?**

R: My aunt and my uncle had their own plate, my sister has her own, and my baby and I have our own.

**I: Do you share foods with your neighbor?**

R: Yes sometimes.

**I: We have heard from some families that eat local foods whereas others eat processed foods. Could you explain what is typical for your family?**

R: Foods that are here.

**I: What kinds of foods?**

R: Foods like rice and…

**I: So, processed foods?**

R: yes

**I: Could you tell me anything that makes it difficult or easy to cook local foods?**

R: The difficult is because we don’t have any.

**I: Can you tell me any positive or negative things about eating local foods?**

R: I think the local are good.

I: Why?

R: They are fresh, and the are very nutritious.

**I: Could you tell me the negative or positive of processed.?**

R: Some rice may not clean some food greasy.

**I: What about positive about processed foods?**

R: It good because we eat and be full.

**I: Now that we talked about how the family eats, I would like to learn more about how your child eats. Could you describe in detail what your son/daughter under years commonly eats throughout the day?**

R: My baby always eat rice…

**I: Rice with what? Any meats or liquids? What else?**

R: Rice, ramen, chicken sausage.

**I: Any liquid while he ate.**

R: I bought 8o’clock for him to drink.

**I: Can you tell me how many times a day meals (and snacks) are eaten by your children?**

R: He eat 6 times a day.

**I: How did you know that your child had enough to eat?**

R: He throw out the foods from his mouth.

**I: Can you tell me what did you do to encourage your child to eat?**

R: Nothing I let him drink water and breastfeed him.

**I: Can you tell me what you do to encourage the child to eat if he refused?**

R: I play some movies, so he can watch and eat.

**I: Can you tell me if you feed you kid differently when he is sick, (ex. When he has diarrhea) and reason why?**

R: When he was sick with diarrhea, he doesn’t want to eat and the only thing he ate was oranges(fruits).

**I: You’ve told me what your child under 2 usually eats. Now could you explain to me the process, from start to finish, how you prepare and cook a meal for your child?**

R: I cook his foods like every morning, I cook a little just for the breakfast.

**I: Can you tell me a story of how you prepare his food? Like for example if it were me I would have go to the store buy a certain food came back and prepare it for him, so can you describe how you prepare your child food?**

R: In the morning I went and buy bread for his breakfast, sometimes when I overslept when I woke up his food already prepare so I feed him.

**I: Any liquid you gave him with his breakfast?**

R: Yes, I bought him fresh milk and pour it in his cup.

**I: Could you now tell me what you think are important foods for children under 2 years to grow well/ be healthy?**

R: Fish, I take out the meat from the bone.

**I: This question is asking what kind of foods you think it important for you child for him to grow well and healthy.**

R: I cook him ma jokkob(it a breadfruit, you boil it and when it soft you grind it and mix it with coconut milk, add a little bit of sugar)for I think it good for his health.

**I: Any other foods you think is good for his health?**

R: I think that all I know.

**I: What foods you should not be giving to the children under 2 and reason why?**

R: Junk foods.

**I: Do you understand the question?**

R: huh, huuh

**I: You understand this question? This question is asking what food you should not gave to your child and it bad for his health?**

R: HUH huh

**I: My other question the one I ask before this question was asking for the food that are good for his health, so what do you think? Can you tell me the food that are good for his health?**

R: Food that very nutritious.

**I: Okay can you give me some examples? You said fish and breadfruit.**

R: Pandanus.

**I: What is the biggest influence on feeding your child?**

R: Don’t always give him junk foods.

**I: Thank you, you are doing great. Can you describe any differences between how you feed your male children and how you feed your female children under two?**

R: No

**I: Could you describe the care of the children throughout the day in your community?**

R: Take of them from dangers.

**I: Dangers like?**

R: Look after them so they won’t go play in the road.

**I: Who is the mainly responsible for the child care?**

R: Their mothers

**I: What are the responsibilities of mothers in the child’s care?**

R: They look after them, shower them, prepare their foods.

**I: What are the responsibilities of the father?**

R: Like take responsibilities.

**I: Like?**

R: Buy them food and support them with their need.

**I: How does you play with the child?**

R: I give him toys to play with.

**I: Could you talk about the role of grandparents have in raising children in this community?**

R: They give them word of advice.

**I: Could you describe how did the grandparents give them advice?**

R: As you can see this house is located near the ocean side and some children on the sea wall where it not safe they told not to paly on the sea wall.

**I: What are the ways that grandparents support in raising children, support mothers and families?**

R: Take care of them when the parents are busy.

**I: Can you tell me what makes the grandparents good?**

R: they are treating the child good.

**I: Could you talk about the role that other family members have in raising children in this community?**

R: I am talking about my kid my relative come and take him and buy him stuff.

**I: Can you tell me the ways that sibling (older sibling) help raise young children?**

R: There is this young girl who always came by our house, she is neighbor, and when her little brother came and play we told her to look after him and she said she doesn’t want to, she always hurt her little brother.

**I: Could you explain where you usually get trusted information about nutrition and health?**

R: In the health or the hospital.

**I: Why did you trust this source?**

R: Because when I follow their advice all their advice works.

**I: Where nutrition and health messages should be delivered so that she would see/hear them most easily?**

R: To the radio station.

**I: What type of media that the person uses the most to communicate?**

R: Cellphone.

**I: When you think about your own parenting behaviour, can you explain what influences how you raise your children?**

R: The way I raise my boy is different from other mother because I raise him with discipline but them, they just let or watch their children do what ever they want.

**I: Can you tell me the opinion of the community influence how they raise children (e.g. leader, neighbor, church leaders, health workers)?**

R: Nothing, in this town we have Kiribatian people, and when their children like play or make too much noise and people who have sick people in their house if their high blood pressure like high they just swear at them (the Kiribatian kids).

**I: Can you tell me of anybody tell you advice or information related to parenting?**

R: None

**I: Can you tell me if you have any desire information on parenting you wish to have that you don’t have?**

R: None

**I: IS there anything else about the topics we talked about today that we missed or that you would like to tell me about?**

R: No, none.

.
