# Supplementary material for: Conceptualizing multi-level determinants of infant and young child nutrition in the Republic of Marshall Islands–a socio-ecological perspective
Source: PLOS Glob Public Health. 2022 Dec 19;2(12):e0001343. doi: 10.1371/journal.pgph.0001343 (PMC10022247; doi:10.1371/journal.pgph.0001343)
Supplement: S1 Data — (ZIP) [file pgph.0001343.s001.zip › RMI Supp Data/Interviews data/I28U_IDI_MCG_Rita_Aug 20_Balton.docx]

Interview Code: 128U

Interview type and interviewee: IDI-MCG

Interview Date: August 20, 2018

Location: Rita

Interviewer: Balton

Transcriber: Maybelline

**I: You ready? Will it be okay if we ahead and start?**

R: Yeah. Sure

**I: To start of our survey, I will start us off with a few questions. I will go ahead and start. Could you tell me a little bit about your family, who lives in this house and how long have you been living in this community? Also how many kids are living in this house?**

R: You’ve start recording? What am I supposed to start off with?

**I: Just talk a little about your family first.**

R: Oh okay. I just move into this community. I have living here for almost 2 years now. I am living here with my wife and her parents.

**I: How many kids are in this household?**

R: Kids in this house? There are three kids, two are mine and one is my sister in laws.

**I: How many girls and boys?**

R: There are two boys and one girl.

**I: Okay. If you can tell more about this household and how you live? Is living in this community okay and is it okay living in this neighbourhood?**

R: Yes of course. This neighbourhood is safe. Also we in this community look out for each other. If someone needs help, we in this community help by lending a hand.

**I: What are the good things in this community?**

R: One thing good about this community is the electricity. We all help each other pay for it. Because in this community, this house and four other houses share the same electricity box. We all contribute in buying cash power. Each household, if they know that they have enough money to buy electricity card, they’ll just go ahead and buy and other houses will buy later if they have money. Everybody in this community are good people. I know can survive because they all lend a hand when I’m need. This community really knows how to look out for one and other.

**I: Are there bad things that you know or see in this community? If you could explain?**

R: From what I see every day, there is not enough spaces between houses and it is very crowded. No space to breath.

**I: Thank you. Now I am going to ask about health. If you could tell me of what kind of illness kids usually have or get?**

R: Fever and coughing. These are the two most common ones that I know they usually have.

**I: Coughing and Fever?**

R: Yes

**I: Is there any other that you can think of? Such as skin irritation, diarrhea and etc.?**

R: Oh yeah. Sometimes they usually get diarrhea. But not often. There has not been anything that has grown on their skin.

**I: So fever and coughing are the ones you know? Is it risky if they get these illness?**

**I: If you are not able to understand the question? You can just ask me to rephrase it. Or you can just say that you don’t really know. You can just answer in any way that you know. Or you can just say you go to your wife the mother to ask about it because they are the experts on kids. You can also say that you give them to their grandparents or the mother for they know how to treat them when they are sick. What I’m trying to say is that if they get fever and it goes on for way to long, the child can get pneumonia and they can die. Also if they get diarrhea, they can get dehydrated. This can also lead to death. These are the risk I wanted to hear from you. In this survey were trying to get information from the communities on how they are taking care of their kids. Also were trying to encourage parents especially the mothers to breastfeed their babies from 0-6 months. Within these six months, the baby will absorb all the nutrient that it needs as it grows up fast. So it really important to breastfeed the baby. When time comes for the baby to eat thick food he/she already have the nutrients from being breastfed and can now absorb other nutrients from the food he/she will be eating. You have to really take care and to make sure that the kids don’t get diarrhea. Diarrhea is also one cause/effect of a child growth. So in order for the kids to grow is by preventing them from getting diarrhea. Also by feeding them healthy food. Greens and sea foods are good for the kids. Every food that you know that are healthy are good to give to the child. Foods that are unhealthy are the ones that are boiled too much. Rice and porridge are also foods that you boil and are bad because foods that are boiled, there is no nutrients in them. So it is good have mekwan(pandanas pudding) and fresh pandanas. Foods that are not boiled a lot are good. Foods that are not boiled too much are good because all the nutrients are gone.**

R: Oh okay.

**I: Okay. Let us continue. This question says, are there any risk you know that always occurs when having fever or diarrhea?**

R: Now I don’t know. What am I supposed to say?

**I: Huh? What I’m trying to ask is, is it too risky if kids are having diarrhea or fever?**

R: I don’t know.

**I: You can say, if they get worse I just take them to the doctor, if it way too worse, they can be admitted into the hospital. Or, you can you give the kids to the mom or the grandmother to take care of them or take them to the hospital. Something like that.**

R: Hmmm… Okay...

**I: Let go on. While the kids are ill, like fever and diarrhea. What are the risk you know that will affect them?**

R: When they get sick, I take them to see the doctor. Not I. The mother or the grandmother are usually the ones to take them to the doctor.

**I: Like these illnesses especially diarrhea. Is there a way to prevent it from kids to have?**

R: There’s none other than to take them to the hospital. We go straight to the hospital.

**I: When the child is sick. What are the signs that you see if the child is getting sick? When you look at the child, what is the first thing you see if the child is getting sick at that time?**

R: Sometimes I can tell when the child is crying a lot and by the look in the eye. If the nose is runny, I know that the child is getting a fever. Also if the child’s temperature is rising, we run straight to the hospital.

**I: When the child is sick. Who is the first person you bring the child to?**

R: The mother and if the mother doesn’t know what is wrong. She usually goes to the mother and ask the mother for help because she always know what to do. She usually bok bwil (puts a wet towel soaked with cold water) if doesn’t work she advises us to go straight to the hospital and see the doctor.

**I: Are there times when you take your child to the local doctors? Like for stomach massage. Liker the traditional healer?**

R: Yes. Because sometimes the kids have stomach lump. Like one of them, the girl had a lump in the stomach months ago. We got someone to massage the stomach.

**I: What are illnesses that you know your kids get if there’s no nutrients in the food they are eating?**

R: What was that again? If they don’t have enough food?

**I: If there’s no nutrients in the food. What will affect the kids if there’s not enough nutrients on the foods they are eating or they don’t get enough food to eat. Do you know if they are going get sick?**

R: Yes. Yes, they’ll get sick because they don’t have enough food.

**I: What kind of illnesses will the child have? For example, you can say if the child don’t eat too much, he or she will get sick a lot. Or they have to eat all the time or will be like kids who don’t have the nutrient who their body don’t grow and are small and their stomach is big. Also some are sickly and are not active they don’t run around.**

R: Oh yeah.

**I: Yeah. You know sometimes, not all families don’t have the foods with the right nutrients that the child needs. So what the question is trying to say is, do you know what will happen to a child if there is no nutrients in the food they eat?**

R: Yeah. The child will get sick a lot, not happy because he/she don’t have enough to eat. Their body won’t grow accordingly because they don’t have enough food and the food that they are eating don’t have the nutrients in them.

**I: What are the kind of food do you think will make your child healthy?**

R: It’s better eating local foods. Local’s foods that don’t…

**I: Like what kinds?**

R: Foods that grows on trees plus foods that are fresh.

**I: As you mention foods that grows on trees. Would you give me some examples of foods that grows on trees?**

R: If they were to eat breadfruits, bandanas and bananas. But here where we’re living in there are not so much growing.

**I: What kind of food that you know that the child will eat that are unhealthy and will make them sick?**

R: Foods that we are eating everyday here in the centre like rice and foods that are from the store that are not fresh. These are the food that make a child sick.

**I: Hmm. These are good answers. Can you tell me some examples of illnesses that will make a child sick if they eat bad foods or there is no nutrients in the foods and also don’t have enough food? Like what kinds of illnesses they will get?**

R: The child will be malnurtrest. The child’s body will be small and the stomach will be big.

**I: Hmmm… We are talking a lot about health. So can you list down what your child is doing in a whole day, from morning to evening? Like how healthy the child is from describing what the child is doing from when he/she wakes up to the time he/she sleeps? For example your kids, when they are healthy what do they do from that time they wake up until they go sleep?**

R: They usually wake up and eat their foods if they are done eating than they go out and play or if they are not playing, they are inside the house watching television then they play. These are the usual. At lunch, they come in and eat than go back and play. This is how, also they don’t cry. They just like to go out and play. They don’t like to stay inside, but like to go out and look for other kids to play with. All they want to do is to go out with other kids and play.

**I: Usually at what time they come home and prepare for bed?**

R: They usually play up until 5pm, then when they are called to come home. They come and take their evening bath. When they are done taking their bath than they eat their dinner. By the time they are done with dinner around 7, than they go to bed.

**I: A healthy child that is at the age of 5. Can you explain more on what do they do throughout the day?**

R: I think it is the same like the other kids that I mentioned earlier. From the time they wake up, they look for their own foods then after they finish eating, they go straight to the playground and play with them. They would come back for lunch and then would go back to play again. There is nothing else they can do except for playing

**I: and what about the adults’ ones, what are the appearances or signs that you see that they are healthy people?**

R: what ages?

**I: the adult ones, like parents, or grandparents of a child, what are the signs of a healthy adult?**

R: they wake up early in the morning and do their house chores like if they have work, they have to prepare the household before they left to work. They have to prepare foods for the children, and people that do not have work, they stay home and do works around the house.

**I: great thank you that was the last question on children’s health and nutrients. Now we would like to learn about the foods availability. Can you explain how your household gets food to eat on a daily basis?**

R: like what?

**I: how do you feed the family like how does the family have foods on the table? How do you bring food on the table so that the family can have breakfast, lunch and dinner how?**

R: like so as of me, I am talking about me I do not have work, what I do is I go fished sell the fishes so that I can have money to buy rice, flour, and foods for the children. I bring these foods to the house so that they can be cooked for the family. If the family get tired of eating fish, the money that sell the fishes about I can use it to buy other kind of meats for us.

**I: that great: are there any foods grown in this house that are eaten for this family?**

R: no. there is no grown food here in this area or around this house. We don’t have enough space for gowning plants we only get foods from the stores.

**I: that’s great. Can you elaborate more on why you mentioned that you don’t have enough space to grow foods can you explain on what are the difficulties in growing foods in this community?**

R: what was that?

**I: what are the difficulties in growing foods in your community?**

R: we can’t grow a plant in our yard because we don’t have enough space to grow these local foods. There is no difficult in grow foods except we don’t have enough space to do that.

**I: that’s great. But were there any times that you used to bring local foods in this community?**

R: yes there are times that we get local foods but it is not me that bring these local foods, it the parents of my wife but I am not really sure where they get these local foods from.

**I: ok great. Like you said that before, its been two years now that you have been here in Majuro, from where you came from I know you have better understand on seasons of foods. Like the pandanus, breadfruits, from where you grew up in, like these foods that I mentioned breadfruits, banana, pandanus, or taro, if it is not the season for foods like these, how would you have foods at the house?**

R: if it’s the season for breadfruits, we would make breadfruits food and store them into the foods we called “bwiro” foods that has to be store for months or years* so that when we are run out of breadfruits, we can get them make foods so that we can eat. If it’s the season for banana, we have to eat a lot of banana because there is no way we can store banana, all we do is eat a lot before we ran out of banana. That’s how we live in the outer islands. If we are in the season of that kind of foods, we have to turn to somethings that can be store for the future.

**I: ok great thank you. In terms that there is not enough foods, what do you usually do when there is not enough foods in the community so that you family can have foods to eat?**

R: if we have banana and it is not the season for banana, we cannot just boil the whole banana because it wouldn’t be enough for the whole family to eat and we would ran out of banana right away. We would cook Jokkep “mash food made out of banana” so that the whole family can have enough to eat. We cook it like that so that we can still have left over banana for the upcoming days. If we cook the whole banana, we won’t have something to eat the next day.

**I: ok that’s great thank you. I am asking questions and you cannot just answer questions from here on Majuro but you also can say something from where you came from Ebon (Outer islands). Except from growing foods, is there any animals you raise here in Majuro?**

R: there is none

**I: and what about in the outer islands?**

R: yes

**I: who is now responsible for them since you are here on Majuro?**

R: I gave them away to people

**I: you gave the away? Are you two planning in going back?**

R: yes but we are not really sure when will be the right time

**I: still not sure yet ok.**

R: if she is done with her education

**I: You have really good answers, you should run for council men chair. Yeah I mean it you should run for the council men chair. I am also plan do to that, it John silk (Minister) retired, I think it would be better, do you think he is a good leader to Ebon people?**

R: I never had any experiences on the negative and positive things he had done.

**I: you never experience that? Do the leaders goes the places?**

R: yes

**I: the mayor lady in your community back home, does she help people in different ways?**

R: yes she does but some people tells negative and positives things about her.

**I: because she used to come to me and tell me when I was in State, she used to tell me that she used to visit people in Toka (Name of place in Ebon and island) she even told me she visited my family and she had a changed to tells story with my little sister named “Darlene” she visited them. She said that she saw you there and you never had time to visit her you were busy fishing and seemed like you were afraid of getting near her.**

R: I never know what is right and wrong our leader do but all I knew is that I can do something in order to feed my family

**I: who is the council man for the place Ebon Toka?**

R: there is no councilman, only the Alap (Lower rank from the chief)

**I: so it’s just them? They are the one to make rules and enforce people?**

R: yes

**I: so who is the Alap in our places?**

R; I am not really sure about that but Jeiban is there.

**I: Jebiban again? So he is the councilman?**

R: yes but now that he is getting sick, I am not really sure who is helping him out

**I: who are your older sibling there since Mart is here on Islands.**

R: Mejwadik

**I: geeeez that guy man.**

R: yes

**I: ok that great he is talkative and he also can be a chief of police**

R: yes

**I: so he is the one that live in my dad’s place?**

R: yes like Mart and them

**I: he also take chief of police positon?**

R: yes but he left his job and moved here.

**I: someone told me about that man I never stop laughing**

R: yes I was only little at these times I never knew between what is right and wrong. Damn man that wife of that guy knew that I was too young for her but she never stopped

**I: I knew that from the beginning but don’t worry that’s not your fault. Ok now I would like to ask you about animals that you raise at home. Could you please tell me about the animals that you raise?**

R: as I was backed in Ebon, I used to raised pigs and chicken. There were also dogs, but that is not a big deal to mention because we don’t eat dog, just to pet them, yes and I think these are the only animals that I used to raise. In the morning, I have to wake up and feed these animals before I do other works around the house.

**I: is there any animals that you raise here on Majuro?**

R: there is none because we don’t have enough space to raise animals,

**I: what are the difficulties to raise animal here on Majuro?**

R: if I had enough space to make their cases or fence so that they can be kept in there. If I had enough money with me I could have make a fancy safety tank to drained out the animal waste. If I had enough budget I could have built my own case and fence for these animal and also made safety tank use to drain out their waste, the only difficult is that I don’t have enough budget with me to do these things that I want to do.

**I: that’s great. As of budget to make fence for raisin animal, what would be some difficulties for to make fence for the animals? What is the difficult for you to afford for these things, the fence and the safety tank?**

R: like I said before, I haven’t got any job at this time and I only lived my life from fishing and diving and what earned from that is not enough to afford for a fence and a safety tank, I only can afford for needs and wants for the family like pay for electricity bill, needs for the family, and buy foods for the family. I think these are the only thing I can afford based on how much budget I have with me.

**I: that’s great. There are sometimes foods that we wish we could eat, but for some reason we cannot do so. Could you tell me about any foods you wish your family could eat or eat more of but cannot?**

R: I would take that as a yes. There are lot of healthy and nutritious foods that we could get but because we don’t have them or they are too expensive, it is better for us to get the cheapest food that can be enough for the whole family to eat. We do really know that it is the right food to get because it is healthy, but if we get it, only two people can have the food, it is too small for everybody, but is too expensive to get it. We know that the healthy food that we want to get is not enough for the whole family so all we have to do is the food that is not healthy and can be enough for the whole family to eat for the rest of the week or can me months. Yes there are lot of healthy food that we could’ve get to make us healthy but the problem is that we don’t have enough budget to afford for healthy foods.

**I: ok that great. Among all these kind of foods, what would be the top food that you wish to have at time moment? Just give me one example of these kind of food you mentioned and its too expensive, it’s not common, and you wished to eat.**

R: I am an outer islands boy and I am used to eat only local foods and I wished to eat local foods and not any other foods, I just wished to eat local foods right now. I wish I would’ve chew the pandanus, eat fish and breadfruits. I do really want to eat these kind of foods but I cannot afford them so that I can eat them.

**I: ok. For the last question on food, can you explain who decides what food to get for your family? Like dinner, breakfast who decides on what food to get for the family?**

R: as for this family, each one of us decide on what food they want to eat. Everyone bring whatever foods they want to eat. Nobody pick on foods, if the foods is there on the table and ready to serve, they would go ahead and eat.

**I: who decide which food young children should eat?**

R: the mother, or it also can be me or the grandparents depend on whom the child point to get the foods for them.

**I: thank you. You have very supportive information by answering these questions. The way you are able to answer yes or no and share your information do really help this survey a lot. Thank you for answering. Now we would move and talk about water and hygiene. Can you please describe a typical day getting and storing water for your family? Like how do you get water in this house and how do you store your water?**

R: in this house that I lived in, there is a bantoon. We use to fill it up with water and use the water for drink. If we know that we are almost ran out of water, we would conserve our water so that our children can’t be thirsty, but sometimes we would also use the water for dish washing. If we have enough, we can use it for shower, but when we have only little, we use water well since there is in this town and it’s close to this house, we can go them water well when we need water. Only if we almost ran out of water, we know that we have to conserve the water for the children, but if we do really ran out of water, we can go and get water from the stores.

**I: that’s great. What are the difficulties in getting water in this community? Like what makes it difficult for you to get water? In you bantoon or your water catchment, whatever that makes it difficult to you to bring water to this house?**

R: bring from where?

**I: it cannot be bring, like what makes it difficult to have water inside the water catchment? Or make it not enough water for everyone in the house to use water?**

R: one reason is the roof is not big enough to catch enough rain water to fill the bantoon, like even though its rain a lot but still the bantoon might not catch enough rain water.

**I: what makes it difficult for the family members to get drinking water?**

R: bring water?

**I: so that the family members can have drinking water throughout the day, is there any difficult for you to get water? Like others would say that they go to DO IT BEST stores and get water like the one they bring their gallon and put it in the machine so that they can have water, where do you get drinking water from?**

R: from the water catchment, the bantoon

**I: ok so you can say that there is no difficulties. If there is no difficulties, you can say there is none. Let move on. Is there any difficult for you to get bathing water or laundry water?**

R: I don’t think there is difficult in getting water because there is water well and when we ran out of water in our water catchment, we can use the water well. There hasn’t be any difficult in getting or bringing water from other houses because we have never ran out of water.

**I: your drinking water, how do you clean the drinking water?**

R: we drink straight from the water catchment the bantoon and the children sometimes we boil their drinking water.

**I: that’s great. Let’s now discuss hand washing. Could you describe in detail your family’s hand washing throughout the day?**

R: when they wake up they wash their face at the same time they wash their hands before they cook breakfast, and after eat they also wash their hands. Before cooking lunch, they would also wash their hands yeah I can say that they wash their hands morning, at noon, and in the evening.

**I: how do children wash their hands throughout the day?**

R: children that can’t be able to wash their hands, their mothers do the hands washing for them

**I: and what about the older kids?**

R: we have already prepare a water and a soap so they can come and wash their hands by themselves. We have prepared a bucket and a soap and they know that before they eat, they have to wash their hands.

**I: when do they use soap throughout the day?**

R: they wash their hands in the morning, during lunch, and in the evening.

**I: from your own understanding, is there any differences between using water only or water or soap to wash hands?**

R: yes there is a big difference, when we wash our hands and don’t use soap, we won’t see that there is still germs in our hands, but when we use soap to wash our hands, we will now recognize dirt in our hands. It is different when we use water only and water and soap to wash hands.

**I: what are something that prevents washing hands with soap throughout the day?**

R: I don’t see any difficultly that prevent someone from washing their hands, except when they don’t have soap or they don’t have money to buy soap, but I think there is nothing to prevent someone from washing their hands.

**I: You are doing a great job providing detailed answers- Thank you. Now, could you describe the type of toilet that you have at your home?**

R: the toilet that we used here in this is we are using the toilet bowl. And my father in law made out his own safety tank and it’s different from the ones that are in the government safety tank line. He dug the ground by himself and made the safety by putting cements together so that we can now have toilet and a safety tank today.

**I: the toilet in this house is a flush one?**

R: yes

**I: that’s great. What is the difference between the toilets that you used in this house from the toilet used in the other houses?**

R: the only different is that we don’t use the government safety tank line, and in our toilet, we have separate a water catchment for the toilet so that we can use to flush the toilet. It is not like the other houses that they have the government line for their safety tank, they can just flush their toilet without using water to flush it. I think that is the only difference

**I: is there any difficult in building toilet in the government’s safety tank line? What prevent people from doing their own safety tank in the government line?**

R: I don’t think there is difficult in doing that but the only problem is when people don’t have enough budget to do their own safety tank and they also can’t afford the salt water that make the toilet bowl flush.

**I: how do young children stool typically disposed? In the environment or?**

R: in this house, children only disposed in their diaper and the mother is responsible in cleaning them and put these diapers in a plastic and throw them in the trash can.

**I: that’s great. Could you explain where your young children usually play each day?**

R: they usually play in our backyard, at the lagoon side when the tide is low, or can be side of the road.

**I: are there any animals kept in areas where children play?**

R: I don’t think there is

**I: can you picture a playground that would be perfect for your children to play?**

R: this area is too small and we can’t make playground for children

**I: and what if there was enough budget to make play ground or areas to play for child, where would you want the playground for the child to be build at? You know this kind of question, it can apply to our government leaders like also to your councilman. We can share these information to your leaders and tell them that your children do really need playground so in your answers, so they can hear you from this interview. So this questions says, if there was a playground for the children, you can say that so your council man or the government leaders can hear you out that you really want a safe playground for your children because if you don’t say it then the councilman or the government leaders might think that the children don’t need playground. then they will use the government money for other purposes. We’re not doing this only but as we are doing this kind of interview, we also give these information to the government leaders so they can know that children in this community need playground, a place to build school buildings, school fee, money for lunch, they need know that children need to have lunch in school because the lunch provide in school is nutritious.**

R: well in this community, they go to the playground

**I: okay let’s repeat the question again. If you want your child to play, where would you want your child to play at? What kind of playground you want your child to play at?**

R: maybe for my desire, it’s better to bring my child to play in the play ground that has lots of playing stuffs and it’s clean for the children to play in.

**I: as for the children’s playground, what prevent it from being clean?**

R: what can i say, because it’s an open area it will always not clean because trashes can come and go through it.

**I: are there any ways to make it clean?**

R: beside that, if there was a fence build around it with a door and someone to make sure to keep it clean or something like that, I don’t know..

**I: good good. In some place, we’ve heard that some people defecating in the lagoon or in the ocean side. Do you know the reasons why or what make some people defecating in the lagoon or in the ocean side?**

R: it comes from lack of people’s needs to provide supplies to build their own rest rooms so they won’t use the ocean or the lagoon side. And I think that’s the only reason for some people to use the lagoon and the ocean side.

**I: good thank you. Now we’re in the questions about the roles of different family members. Can you describe the care of children throughout the day in this community?**

R: as for my family, when we wake up in the morning I will go and wash my face first while my wife looks after our children and when I am done, my wife will go wash her face while I look after our children. When she’s done, then she will go ahead and make breakfast for our children and when she’s done making breakfast, she will then come and take them to feed them.

**I: who is mainly responsible for a child?**

R: usually the mother since I am always busy doing works that will help me provide needs for my family.

**I: what are the responsibilities of the mother?**

R: as for my wife, her responsibility is to take care of our children. She has to prepare food for them, wash them, and I think those are responsibility for my wife.

**I: what about your responsibilities or the responsibilities of the fathers to their children?**

R: as for me, my responsibility is to make money or work so I can support my family with their needs. Or whatever I think that a father is responsible for.

**I: how do caregivers play with the children? Or how does your wife or you play with your children?**

R: as for our children, because they are still young we just let them play and running outside while we look after them.

**I: okay good. Can you tell me what happen when grandparents take care of the children? Or are there anything they help with in taking care of the children in this community?**

R: well yeah. Some parents bring their children to the grandparents and they look after the children and play with them too. Sometimes the grandparents bring toys to the children to play with. They also help.

**I: good. What are the ways that can show that they are good grandparents?**

R: I believe that grandparents are also nice to our children. We can see that the grandparents are really happy when they interact with the children, they spend a lot of time with them, the children play with them, and because they love their grandchildren so much they show their happiness toward them.

**I: good thank you. Are there any family members in this community take responsibility in taking care of the children? Instead of you and your wife, is there anyone else take care of the children?**

R: well yeah. The other reason why this community is good is because as I said the people in this community help each other with everything. They also help in taking care of the children. Wherever they see them, it’s also their responsibility to take care of them.

**I: the older children in this community, do they help in playing with your children? Or the older children,**

R: oh yes they do. The older children play with the younger children because they are close to each other.

**I: okay thank you and I am sorry because I forgot to ask you the last question about hygiene. Can you explain the ways to prevent disease from spreading? There are lots of disease like pink eye, coughing, flu, so what can we do to prevent these diseases?**

R: I think the only way to prevent disease from spreading is to help each other clean our community. Even though its not our area but if we see that its not clean, we give our hands and help clean it. And I think that’s the biggest way to help prevent disease from spreading.

**I: You are doing a great job. We are almost finished. Now for the last section, we would like to learn about ways we can develop health programs in your community. Could you explain where you usually get trusted information about nutrition and health?**

R: as for me and my wife, we usually ask our children’s grandparents. Every time we ask them about how to take care of the children.

**I: do you get information from the internet, or from the radio, or from the government’s program, or from the posters in the hospital, or from school? Instead of the grandparents
?**

R: sometimes from the radio. sometimes I listen to the radio on how to take care of our children.

**I: where do usually get and its easy for you to get information from?**

R: I think its easier to get information from the radio because we can just listen to it while we are doing our chores

**I: okay good we are on our last question. From your own understanding as a father, were there any advices given to you or were there anything influence you as a father to take care of your children?**

R: okay as for me there are none because I haven’t meet someone that like to give advice on how to take care of my family. I just see what I can do as a father to take care of my children or my family.

**I: in this community, how do people think about raising children? Like for example, the community leaders or the neighbours. Do you think the people work together on raising children?**

R: well yeah. In this community, people help each other in every activity. As for the children attending school, everyone is taking responsibility in taking care of them

**I: are there any information about parenting you wish to know but it’s not available?**

R: I don’t think I have come to that kind of thought

**I: okay our very last question. Are there anything else you wanted to know about children’s health and parenting, but we didn’t discuss?**

R: I think there none either

**I: okay thank you. Thanks for the quality time you share with us so we can get some useful information from you.**
