# Supplementary material for: Conceptualizing multi-level determinants of infant and young child nutrition in the Republic of Marshall Islands–a socio-ecological perspective
Source: PLOS Glob Public Health. 2022 Dec 19;2(12):e0001343. doi: 10.1371/journal.pgph.0001343 (PMC10022247; doi:10.1371/journal.pgph.0001343)
Supplement: S1 Data — (ZIP) [file pgph.0001343.s001.zip › RMI Supp Data/Interviews data/I29U_IDI_FCG_Rita_Aug20_Meia_MarcellinaEdited.docx]

**Interview code: I29U**

**Interview type and Interviewee: IDI**

**Interview Date: Aug20**

**Location: Rita**

**Interview: Meia**

**Transcribe: Meia**

**I: TO began with Can you tell me something about your family? Who live in the house? How many kids? You don’t have to mention your names.**

R: My husband and I and our five kid, and our baby granddaughter.

**I: Next I want you to describe your community?**

R: In this community, we gather together during May day (it a constitution day), party and mother day. We always gather together during these event.

**I: Can you tell me any negative in this community?**

R: Many animals are roaming around this town making this town dirty.

**I: Any other negative about this community?**

R: When there are drunken guys yelling and shouting between houses.

**I: Can you tell me about some of the illnesses that your children have suffered from?**

R: The illnesses that the child always had are headache, nauseous and stomach ache.

**I: Can you tell me what causes these illnesses?**

R: They just appear in their own time from my opinion. Time for headache and stomach ache.

**I: Can you tell if these illnesses are serious?**

R: No. not for my children

**I: Can you tell how you prevent these illnesses? Do you use medicine from hospital or you use traditional?**

R: I used medicine from hospital for stomach ache and headache like Tylenol, but for the nauseous I use Marshallese medicine.

**I: What kind of Marshallese medicines you use?**

R: Kino (Marshallese tree we use for medicine, it a small that has a lot of small leaf).

**I: Can you explain how you use the kino tree.**

R: I took some leaf and grind them and put them in a small piece of cloth and give it to the child to sniff.

**I: Can you describe how you know when your child need treatment for their illnesses?**

R: I always bring her to the hospital when she is sick, and the doctor always said that whenever she is sick just bring her right away.

**I: Can you tell me who did you bring her to first when she is sick?**

R: The doctor.

**I: why did you bring her to the doctor?**

R: So, the doctor can check her and see what kind of sickness she had.

**I: Can you tell me if you use traditional medicine?**

R: Yes, I use traditional for many kinds of sickness

**I: Can you example some of the sickness you use traditional medicine?**

R: sickness like maj (it very dangerous and painful type of illness where it grows in our nose, eyes but not inside of our eyes just the outer part of our eyes, ears, inside of our stomach and it very commonly grow in our anus or vagina).

**I: What about kids during sickness what traditional medicine you use when the child is sick.**

R: Yes, I use traditional medicine for diarrhea, nauseous, and stomach ache.

**I: Could you describe how you use traditional medicine for these illnesses?**

R: For diarrhea I use kiden tree and kolani (kolani is the smallest coconut in the coconut tree). I mix them together and let them drink it. For nauseous like I said before I use kino tree and for stomach ache I use noni tree’s leaf like i took three green and three yellow leafs and grind together and squash the juice to cup and let her drink it.

**I: Can you describe any illnesses affecting your children that are associated with nutrition?**

R: There haven’t any sickness that affecting the children that are associated with nutrition.

**I: We talked a lot about being unhealthy. Could you now describe for me a typical day of someone living a healthy lifestyle, from the time they wake up in the morning until when they go to bed?**

R: They woke up eat breakfast, eat lunch to get energy throughout the day, for them to get by 24 hours or to feel energetic throughout the day.

**I: Can you tell me the appearance/sign of a healthy child under 2 years?**

R: They don’t feel lazy, they don’t cry a lot.. that’s all I know.

**I: Now could you describe the appearances/signs of a healthy adult?**

R: They don’t get tired easily, they eat vegetables and fish for them to be healthy and energetic.

**I: Let now discuss hand washing. Could you describe in detail your family’s hand washing throughout the day?**

R: The children use soap and hand sanitizer.

**I: Could you explain how they wash their hand?**

R: They wash their hand before they eat, before and after they use bathroom, they wash their hands with soap and use sanitizer.

**I: How about the child under two, how did they wash their hands?**

R: I wash my granddaughter’s hands using soap and hand sanitizer.

**I: Can you tell me when did the children in the house use soap to wash their hands during the day?**

R: when they just woke up, between breakfast and lunch and dinner and before they go to bed.

**I: Can you tell me the differences between using water only or water and soap to wash hands?**

R: seems like before there were no hand sanitizer, we use soap with water but it’s no different because after we wash our hands we still see dirty in our finger tips, but now with hands sanitizer we can tell that our hands are clean.

**I: What prevents you from washing hands with soap throughout the day?**

R: Nothing we use soap to wash our hands all the times.

**I: Now I would like you to think back to when your daughter was pregnant. Can you describe her diet when she was pregnant compare to when she was not pregnant?**

R: She wasn’t with me during her first few months of pregnancy, but when she did come she was 7months old pregnant and she didn’t like had any problem in her diets. She eats anything and didn’t had any pregnant sickness issue.

**I: Can you tell me what food you encourage your daughter to eat during pregnancy?**

R: I didn’t force her to eat, she can drink any things and eat any things.

**I: Can you describe how you primarily cared for/supported your daughter during her pregnancy?**

R: During her pregnancy, as I was saying before that she wasn’t with me until she was 7 months pregnant, when she was with me, I advice her not to go out at night, always carry a torn page of a bible with her whenever she goes out and I took her to see doctor.

**I: Can you tell me about any supplement your daughter took during her pregnancy?**

R: She took vitamins and supplements for blood.

**I: Did she take all the supplements?**

R: yes.

**I: Can you tell me if your daughter drink alcohol, smoking or use other drugs during her pregnancy?**

R: She chew, I only saw her chew betel nut but the other drugs I never saw her use them.

**I: Did she use traditional medicine and why?**

R: She only use traditional medicine for back pain.

**I: How did she take the medicine?**

R: She drinks them.

**I: If she was advised to eat more fruits and vegetables during pregnancy, could you describe what would make this difficult?**

R: she never had those during her pregnancy

**I: Why didn’t she have these foods?**

R: Because at that times we didn’t have work yet or we were unemployed at that time during her pregnancy. Her parents didn’t work but now we are working.

**I: Now can you describe her diet during breastfeeding?**

R: Rice, fish aikue (iu mix with flour and coconut milk with sprout come out of it) aerice (rice with iu) and egg.

**I: What makes her eat these foods?**

R: The baby;she eats these to have breastmilk to feed her baby.

**I: Can you describe what food you encourage your daughter to eat during breastfeeding?**

R: Fish, egg and meats.

**I: Can you tell me why you wanted her to eat these foods?**

R: For her to make breastmilk. And for her baby to not have any problem.

**I: Can you tell me what kind of food that you did not encourage your daughter to eat during her breastfeeding? And reason why?**

R: Greasy foods, like pork, turkey tails, shortening (it is commonly eaten with breadfruit), salty soup base of ramen (children and woman eat this with rice when they don’t have any meats with their food or they can just eat it like their snack, people with low income) and salt.

**I: Can you tell me why you didn’t want her to eat these foods?**

R: So, the baby won’t have problem with her health.

**I: Can you tell me some problem that can be the result of the food you mentioned?**

R: as for the greasy foods, they’ll cause problem with her lung, she would have diarrhea and she would have body limb.

**I: After giving birth, could you describe how your daughter breastfeeding your grandchild throughout the day? Like did she wait like for a day to breastfeed the child or did she waited for like several hours to breastfeed the child.**

R: She waited for hours because the nurses took the baby to the incubator because the baby drown inside her mom stomach because she took a long time to push her out. The doctor tried to bring the baby’s life back after they had success in bring the child’s life back, they took her and put her inside incubator, for after like three hours then she started to feed her child.

**I: now when she breastfed her baby, were there any difficulties in breastfeeding the baby?**

R: no there were no difficulties but they brought her back to the incubator and like whenever the baby needed to feed they just bring her

**I: Can you tell me if you or the mom gave any liquid to the baby in the first few days after birth (and reasons why)?**

R: I think none.

**I: Was there any medicine our Marshallese medicines because base on ours believed and culture, we always give our baby traditional medicine so was there anything you gave after birth?**

R: To the mother?

**I: No, to the baby**

R: the baby? No, because the baby stayed in the incubator and I cannot go inside the room where the incubators are. But after the baby discharge and we went home, that is the time I used traditional medicine.

**I: Okay about that time, that’s what I wanted to know, can you tell me the medicine you gave to the baby?**

R: After she was discharge, I gave her the medicine for kijonkan (kijonkan = we believe that a child with kijonkan is possess by spirits but not evil spirit, the child will either have yellow skin or eyes, the child will stretch a lot and the child will always startle during his/her sleep). It had to be finish for three days, after that I gave her another medicine for her not to cry a lot, then I gave her another medicine after that to protect her if her mom be with men (have sex with any male).

**I: Can you explain what kind of medicine you use and how you did you use these medicines?**

R: For the kijonkan I use mariko (mariko is a grass that have violet flowers and it leafs look like a heart shape), so I grind the leaf and give it to the baby to drink, I also bath her with the medicine twice a day like in the morning and evening for three days. After I finish with this medicine, I gave her another medicine which is called ekkon (local tree) to protect the baby when her mother had sex with another male who is not the father, the baby won’t get sick and the baby won’t have weak body or weak muscles.

**I: All these medicines the baby drink?**

R: Yes, and bath with these medicines.

**I: What’s the other medicine?**

R: Medicine for the baby not to cry a lot.

**I: Can you tell me about the medicine?**

R: The medicine for the baby not to cry a lot is konnat tree.

**I: Are all these medicines mix with water?**

R: Yes, I bath her with them and let the baby drink them except the konnat tree because It sour, so I just bath the baby with the konnat tree. The mariko medicine for the kijonkan illness she baths and drink and the medicine for weak muscles and body whenever the mom have sex with another man, I use the kone tree, I bath the baby and for the medicine to drinks I mix it with her mother breastmilk. I made the baby drink it and rub the medicine all over the body of the baby.

**I: Thank you for these good in formation. So, were there any difficulties for her to breastfeed her baby exclusively up to 6 months?**

R: No. None

**I: Is it difficult for her to breastfeed her baby up to 2 years?**

R: none also

**I: That means she still breastfeed. Okay, Now, can you tell me when you first give foods and liquids to your grandchild other than breastmilk?**

R: at 6 months, I started to feed her when she was 6 months old, the first food that I gave my grandchild is baby foods; bananas and vegetables flavor and the liquid was fresh milk.

**I: Can you tell me why you introduced foods or liquids other than the breastmilk to your grandchild at this age?**

R: This is the month for the child to start eating, the doctors have advise us and we follow what they told us.

**I: Can you tell me what are the opinions from others that influenced their decision to introduce foods and liquids that age?**

R: To grow and have a healthy life, and to grow and adapt to the taste of our foods so that the baby can eat our own foods.

**I: So now is there.. oh no. Now what are the baby’s first foods and you did you prepare the foods? Can you describe how you prepare the foods?**

R: If I took one banana and vegetables, I took three spoons from each of the baby food and mix them together, I feed her at 8 o clock in the morning, 11 o clock at noon, 2 o clock in the afternoon and 4 o clock in the evening.

**I: We are trying to understand how people eat in the community. Could you describe in detail what your family usually eats and drinks throughout the day?**

R: My children drink water, but if we drink coffee the children also drink. As for foods, this family eat any kind of foods, and as for the food from the store- the foods that we bring from the store are like chicken, gizzard, turkey tail and turkey neck, these are the only meats from the store and hotdog.

**I: Are there any other foods along with these meats?**

R: Rice and bread, for flour we can make tonet (it’s round and you cook like a donut but it round and smaller that donut), donut, kotaban (it’s a really small donut and it sweeter than the donut), aekui ( it a coconut meat with flour) and likobla ( it made of flour, water, sugar and coconut milk, and it like pudding).

**I: Now, I want asked how you process the meals of your family?**

R: I made them pancake in the morning and drink 8 o clock tang, and we gone to work then back at night they’ll eat rice with the meat that we bring.

**I: Who in the family that you serve first and last?**

R: The children first and then the adults.

**I: Can you tell me whether there are differences in the foods served to different family members?**

R: There are none, all the foods are the same.

**I: Is there any differences in the quantities of foods served to the different family member?**

R: All are same amount.

**I: Now can you describe any food sharing between family members during mealtimes (for example children eating together separately from the family, meals eaten from the same plate by all family members?**

R: there are five kids, one girl is eating with my grandchild in one plate, the other two girls they in one plate too, same for the two boys and my husband and I with one plate.

**I: Does your family share foods with your neighbors?**

R: yes,

**I: Can you tell me a story of how your family share food with your neighbors?**

R: If we have a lot of foods, we share a plate of food to the neighbor, but if it not enough then we keep it to our self.

**I: We have heard that some families eat local foods whereas others eat processed foods. Could you explain what is typical for your family?**

R: as for processed foods, we eat rice and processed meats. But for local foods, we eat breadfruits, and all the other Marshallese foods. There are no local foods that we hate.

**I: What about the foods that they eat like every day?**

R: Rice, meat and bread for breakfast.

**I: What make it difficult or easy for you to cook local foods, can you tell me anything that make it difficult or easy for you to cook local foods?**

R: Marshallese foods; the foods that we can make and are easy to cook are lukoj (coconut meat with flour, water and sugar), coconut meat pancake (flour with coconut meat we make pancake), aerice,--rice with coconut meat or rice with flour, pumpkin with rice or pumpkin with flour, breadfruit, for breadfruit we have biliturok (you boil the breadfruit with coconut milk and sugar), stem breadfruit, biro (it also made out of breadfruit) and jokkob (it’s the same as biliturok but you smash the boiled breadfruits and make it soft like a paste)

**I: Is it difficult for you to have these foods?**

R: No

**I: Can you tell me the positive or the negative of eating local foods?**

R: There are no negative in local foods.

**I: Okay, tell me the positive of local foods?**

R: All local foods are good because they were our foods from before and we should have known how to make them. Also it was what we usually eat from our mother and father.

**I: Is there any other positive thing about local foods that you know about? Base on our health?**

R: we don’t get diabetic and high blood pressure from local foods.

**I: (laugh) you are right! What about processed foods? What are the positive or negative things about eating processed foods?**

R: For processed foods like rice we get diabetic. Base on my knowledge it has lot of starch. For sugar, we have our legs cut off because of it, and the processed meats, we get fat and high blood pressure.

**I: Now that we’ve talked about how the family eats, I would like to learn more about how your grandchild eats. Could you describe in detail what your grand son/daughter under 2 years commonly eats throughout the day?**

R: we feed her foods.

**I: Okay. What kind of foods you feed or usually feed her?**

R: Rice and- lunch food like rice and meats but if we have fish we give her too. And we feed her at her meals time.

**I: What about breakfast?**

R: For breakfast she will have bread but if she doesn’t want bread we will give her rice too if she wants.

**I: What about liquid, what did you give her?**

R: The liquid she usually drinks is 8 o clock (tang)

**I: Good thank you, okay! Now can you tell me how many times a day meal (and snacks) are eaten by your grandchild?**

R: If my grandchild eat breakfast, if she eat bread because she doesn’t want to eat rice then she is going to eat two times in the morning, and if it noon between 10 and 11 o clock she will eats again, two time again and if she doesn’t want to eat dinner she sleeps, and then woke up at midnight crying because she is hungry again.

**I: How did you know that your grandchild had enough to eat?**

R: She doesn’t want to- she pushed her plate away and she doesn’t want to eat.

**I: What do you do to encourage your grandchild to eat?**

R: We usually- if she doesn’t want to eat from the foods in the house, like today she doesn’t want to eat, we took her to the store and see what she wants to eat and when she pointed out what she like we buy it for her to eat.

**I: Can you tell me what you do to encourage your grandchild to eat if the child refuses.**

R: we do what she wants- let just say if she doesn’t wants to eat, we walk her around until she is happy and then we’ll return her because she doesn’t cry anymore. And at that time she’ll ask for foods. And what can we say? She is tire of here and want to carry her to other places.

**I: now are there any differences on feeding your child when he’s sick? For example, when he has diarrhea?**

R: Yes because she has stomach-ache and she only lays down. When we give her food she doesn’t want to eat. And that’s why I make her a local medicine to drink and she sleep. And when she woke up she’s hungry. I usually prepare her food just in case she woke hungry, so I prepare a bowl of food and her drink because if she woke up hungry if feed her.

**I: You’ve told me what your child under 2 usually eats. Now could you explain to me the process, from start to finish, how you prepare and cook a meal for your grandchild? Can you tell me a story about how you prepare her foods?**

R: Before she woke up, I usually prepare her food and put it in a bowl and then put the bowl besides her for if she wakes up, she’ll see her food. And I always warn my children not to touch her food but to make their own foods for they are old enough to make their own foods. And she’ll eat when she wakes up, and I also prepare her a drink in a cup.

**I: Okay, this question is asking the process of how you prepare her foods from start to finish? What do you do from start to finish on preparing her foods?**

R: If it were pancakes, I usually mix the ingredients, after that I bake it and put it in a small bowl for her to eat. And then take some eggs and cook them and put them too in the bowl together. For her drink, I also prepare it and put it besides her bowl. There are always two choice of drink for her; it’s either water or color drink (it either coffee, kool-aid, or any soft drink) for her to choose her own drink. But if she sleeps and wakes up whenever, she’ll then have her meal.

**I:**  **Could you now tell me what you think are important foods for your grandchild under 2 years to grow well/be healthy?**

R: Fish, vegetables, but she doesn’t like vegetables much. The only vegetables she likes are corn (canned) and mix vegetables (canned) because as for the mixed vegetables, she loves choosing what she likes from the mix vegetables.

**I: Can you tell me what type of foods that should not be given to your grandchild and the reason why?**

R: Fish, for she might get some small bone stuck in her throat. So we have to feed her. And the foods that are cook but aren’t really cooked, we also don’t give her.

**I: you already say why you can’t give fish. What about sashimi or raw foods. Can you tell me why can’t you give her these?**

R: what I meant by raw foods was, the foods that are cook but are not really cook. And for sashimi, we only give that kind of sashimi that has lots of meat and no fish bones.

**I: are there any foods that you don’t have to give her because it’ll ruin her health?**

R: Sweets- foods that are sweet because she always wanted to eat sweets before she eats. And we usually try not give her any sweet before she eats her meal. And after she eats her meal then we give her the sweet.

**I: Can you tell me why you should not give your grandchild sweet or sweet foods?**

R: Because she will have tooth cavity and it’s not good for her health.

**I: Can you tell me what your influence on feeding your grandchild?**

R: I always told my kids not to feed her raw foods, foods that have bones in them like fish, they have to chew it first before give it to her, and don’t put too much foods in her mouth for she might choke.

**I: Can you describe any differences (if any) between how you feed your male grandchildren and how you feed your female grandchildren?**

R: No different, they are both same.

**I: wow! Your information are good thank you for these good information. We are now in the last page. We are also interested in the roles and responsibilities different family members play in raising children. Could you describe the care of children throughout the day in your community?**

R: The people in this community look after their grandchildren or children from danger like climbing trees otherwise they might fall, not drowning in the ocean while swimming, and other stuff while they play like throw rock at each other, fight one another.

**I: Who is the mainly responsible for the child?**

R: The mother.

**I: Can you tell me what are the responsibilities of the mother in the child care?**

R: The mother look after her child and don’t sleep at night when their baby is awake, look after them in the house, play and feed them.

**I: Can you tell me what are the responsibilities of the father in the child care?**

R: also help the mother taking care of the children and support with needs like hospital bills.

**I: Can you tell me how you play with your grandkid?**

R: I give her toy like ball, I roll the ball on the ground and she brings it back. I keep doing this and if she doesn’t want to play anymore I look for something else for her to play.

**I: Could you talk about the role- oh this question really applied to you because you are the caregiver of the baby, Roles of the grandpa or you in raising your grandchild?**

R: We take care of her as our own, and we bought whatever she needs. Same like our own kids, but we really give more to our grandchild than our children.

**I: (laugh) just like all grandparents to their grandchildren. Okay, now what makes you a good grandparent?**

R: We buy what she wants and we play with her, if she want us to carry her around either on our back or front we do whatever she wants.

**I: Can you talk about the role that other family members have in raising children in this community?**

R: They are doing the same things we do for the child. Taking care of the child like their own, playing and looking after the child and do what ever the child wants.

**I: Can you tell me the ways that sibling (older siblings) help raise young children?**

R: For example, children in my house. Whenever we go to work and leave them alone at home, the older sibling will prepare their foods, have them bath before they sleep, and prepare their beds before they go to sleep.

**I: Thank you, you are doing a great job. We are almost finished, now for the last section, we would like to learn about ways we can develop health program in your community. Can you explain where you usually get trusted information about nutrition and health?**

R: from the wellness group. When the people from wellness do outreach and teach us about the nutrition foods.

**I: Can you tell me why you trust the wellness program or where you heard the information from?**

R: Because all the three groups of food are in wellness center’s meals, but at our house we only have two group of food out of three.

**I: Where should nutrition and health messages should deliver so that you would see/hear them most easily?**

R: It should have been with us.

**I: But where do you want the message to go to so it could be easy for you to see and hear?**

R: At the stores and at the wellness center as well.

**I: as for the stores, they have to have Poster right?**

R: yes.

**I: What types of media you usually use to get these information?**

R: telephone, oh cellphone not telephone.

**I: Good, we are in the last question. When you think about your own parenting behaviour, can you explain what influences how you raise your grand child?**

R: I took care of my grandchild

**I: yes, can you explain the differences? Like are your parenting skills different from others?**

R: as for me because I took the child from the mother, the difference on how I take care of the child verses the mom, I can buy her needs but the mom can’t because she is un employee.

**I: Can you describe the opinion of the community influence how they raise their children?**

R: Good, everyone has the same opinions on raising kids.

**I: Was there any advice or information related to parenting you received?**

R: as for parenting, the thing I used to hear from our ancestor, regarding discipline.

**I: Can you explain about that?**

R: We can discipline our children or our grandchildren but not too much for blood to flow from the body. After I heard this, I kept it in my mind and heart. But before, if I had to beat up my younger sibling, it’ll be very bad like they’ll be bleeding or swelling. But now after I know this I don’t do it anymore. Now that I have kids, I am not treating them that way. But now when see my younger sibling doing this, it makes me reminisced the time we use to be together and it makes me afraid. That’s why I don’t treat my kids like the way I used to treat my younger siblings. Now whenever they ask me to help them financially I help them. But before if they ask something from me I beat them up.

**I: Is there any desired information on parenting you wished you had but you don’t have? so that I may give it to the health worker for help?**

R: it’s all good, I understand everything.

**I: Thank you for giving this time for me to collect these good informations.**
