# Supplementary material for: Conceptualizing multi-level determinants of infant and young child nutrition in the Republic of Marshall Islands–a socio-ecological perspective
Source: PLOS Glob Public Health. 2022 Dec 19;2(12):e0001343. doi: 10.1371/journal.pgph.0001343 (PMC10022247; doi:10.1371/journal.pgph.0001343)
Supplement: S1 Data — (ZIP) [file pgph.0001343.s001.zip › RMI Supp Data/Interviews data/I30U_IDI_SLHS_Rita_Aug 21_Maryam.docx]

I: Okay start with, can you please descibe your role here.

R: I'm the deputy general manager here at EPA. My main role is most of what I do on a daily basis is as a administration, while the GM takes care of anything above that. But my role is mainly taking care of the staff and administration and finance. So I'm not too involve with the you know with the international, all of these conventions that we're part of and all these inernational rights and anything like that. But I do sometimes when she's on a fetch when I take on the role but my main work is at administration and finance here at EPA and Human Resources.

I: How long have you been here?

R: I've been here for a year and a half. Not too long. So this will be a learning experience for both of us. Because I'll be trying to see if I know what my position is. (Both laughs). We'll know because of you. You'll be like I was talking to the deputy and she doesn't know anything.

I: Okay so in terms of getting into the need of the project. Can you describe what you think are the biggest challenges to accessing safe water in Majuro?

R: Not every household has catchment. Some have access to well water but not everybody. Depends on where you live and how the loaner of these wales are, but not all wells are safe. So we try to encourage people if they are using well water for preparing food or drinking or anything, that they have to boil them. If it's the water catchment system they have, we'd provide them with treatment plans which is using clorox or bleach. But not everybody does it because you know how you put it and have to wait for a certain period before the bleach takes affect. We try to do as much to resourness for making sure they keep their tanks clean and making sure they keep their gutters clean. But it's really hard because there are a lot of animals. We have the birds, we have the cats, and we have the little geckos that walks over the roofs so you know that. So we do have a lot of e-coli results and we do testing. So in most cases most serious espicially the food at restraunts and the delis and all that. We do those on weekly basis. So we do consistenly testing, especially the water provider like RRE and all of the mini water, you know where you go and refill your water bottels and yeah. So we do test those consistenly. So like I said the population of Majuro is what... 20, 25,000 now. So not everybody has water in their house. Not everybody has access to public water system. Even if they do it doesn't work all the time. And of course it has hours when it's on or off. It's not on 24hr/day. So most houses if you see that they have running water it's probably they have water catchment and a pump. If it's running water all the time it's 24hrs not the public water system. So it's mainly sharing, that's how people usually access to having water but it's not always safe so we have make sure that they have to boil it before they drink it or use it to prepare food but we really don't know if they do that or not. But we still have to find out. But we do try to our share too. Like all of these little stalls that you see by the road, they're suppose to have... Sorry, a health certificate. And before they get their health certificate they need to get their water source tested. So we've been doing that for, ever since I came in. When the e-coli came out last year and Hep B and all of that came out. So we took that on and took on the testing so it's actually time again to do another round, it's been a year now. So it's funny I was just thinking about it this morning and I have to meet with the Tibuk and tell him to go and check all the ladies are around there and check out all the stalls and see if their health certificate are still up to date. So that's one, they can't get their health certificate unless they get a clearing from here, clearance from EPA on their water. So in most cases, it's church groups and womens groups so there is only one water source which is good. So it doesn't take too long for us to get that clearance and those are headache in the beginning because we had no reason to do it before, that's when we went there and actually saw what they were doing. How they were preparing their food, most of them we had to say you can't do this. There was no way we're gonna allow you to sale food because some were using wale water, right there and no boiling or no anything like that and it wasn't pretty. We got to see all of that when we get that so that was good. So yes we need to do another round I think we need to do another round. It's kinda different for me from my personal perspective because, ... how I was raise I always have access to water in my family so it's kinda sometimes it's hard to you know, how can you have no water, you know things like that? You know when you were small and you really didn't think about it, but as you grow older and you find out that a lot of people do not have access to waters here. Because I think not only that but the population was not as how it is now. A lot of these people are coming, but when you go to outer islands, it's a whole a different story. Almost everybody has access to water. But the issue now is, because they're raising a lot pigs and waste, solid waste. That's getting into the water system and now we just did the study in Wotje working with SPC and they went Wotje and did a survey. When they did that we also tested their water and there were a lot of e-coli results from the wale waters. Even the tanks you could understand but this is wale water this is wale. Their wale water are affected as well so we're gonna be doing a whole a lot more work with them to try and clean those up. On EPA, maybe we'll do this last before we know what our plans are. Because we did a vocal area for safe and fresh water supplies. Our second focus is clean safe marine water, this is just marine and the third one is safe fresh water supplies. So we need to develope a program to improve households water management and of course continue with our water testing and our reporting. But we haven't, we're just implementing this this year. So these are some of the things we're gonna be working on, but the plans are to improve awareness on making sure they keep their tanks clean, at least once a month, they need to empty it out, scrub the thing down and then you know that's the plan they need to do that. And of course continue with the other... Clean... Bleach, use of bleach which is not good in all terms but yea, that's what you have to do you know. Making sure your roof is clean, making sure your gutters are clean. I discourage animals but my Aunt next door has a lot of cats. (Laughs). But yes that's an issue for my house. There's cats all over the place. (Both laughs).

I: So when you are trying to raise awareness to get people to clean their gutters and clean their you know keep that safe. How is that awareness raising done, like how do guys, what do you guys do?

R: We go. Like we go to their homes, we do it on the radio we do it once in awhile, and the newspaper. But we do have a awareness environment solve and educational awareness group. She sits here in this office. She's only by herself but all of the other divisions are supposed to work with her and help when we do any work in all the different area espicially in the communities. What we are most active now is a clean school program so we do it in the schools that has trash. Incinerating your trash, that's the one we focus mainly about. But our water division is the one does all the awareness, you know whenever they go and do testing they go through the whole processes of keeping the water clean and trying to work. Well you can do it so much right, you tell them what to do and help but you never know they do it all the time. I don't even do it all the time. It's a lot of work, it's water work. Sometimes I do it without telling my family. (Laugh). Sometimes I don't tell them you have to wait 24 hours and you can't tell a household to wait 24 hours and not use the water for 24 hours. So I try to do it before everybody goes to bed. Pouring all the tanks, the good thing about my house is there's two systems. My parents house like the main house has it own system and I have my own system but we share. Like if the water in the main house runs out then we use my own water. So I get my water treated while we use the other one. Then after that we switch the control system. Like there's pump in my house and the pump in my parents house, we switch. But that's us. And there are a lot of homes that uses just wale water, they pump their water from wale. That's how my old house use to be. So we never ran out water and it never got salty because back than, 30 years ago. So we never ran out, so that's how we had access to our water, we never had a catchment, we had a small one but we hardly ever use it. So we just use our wale water.

I: Can you give a little bit of explaination of the difference between back then and now interest of wale water?

R: (Laughs). Well yea like I said. My household what 30 years ago, we only had to use mainly our wale water for everything. We had running water in my house but it was from the wale. We didn't have access to public water back then, at our old house. And it was fine, it was clean and we never really had any issues with diaharrea and all of these different things that you see now. While these illnesses are coming and all these water bourne illnesses there happening now and of course you'd understand that because it's so crouded here then it was back then. Only in my 40's but if you'd see the whole difference in the last 30 or so years from how it was when everybody had access to clean water but now it's just, it's so crouded. When you go to these places where there is like... Not too much end of Rita but a little after end of Rita, Jenrok that area, Delap of course, and I'm sure Small Island now. It wasn't that populated it just boom like in last maybe 10, 15 years all of these houses just started. But it wasn't that over populated as it is now and we've been trying to work with the land owners because all the whole ocean side of that place in Small Island it's all trash. So it's really...(Laughs), you try, you try to but you can't take all of this to dump because it's already full where else can you put it? Like well, what so put it in the water, and then you go swimming in the water and then you go fishing in the water and you don't know what this does to anything that's in the water? Yea, it's pretty hard, espicially the lifes here Majuro and Ebye. Not so much in the outer islands, because they don't have too many people anymore everybody's moving out.

I: Coming where? Like are they coming to Majuro?

R: They come to Majuro and I assume they are just moving to the States. Yea.

I: Why is that, why is there migration away from the outer islands?

R: They're going for a better opportunity you know work or school. There's people that they've been there for the longest time and I don't think they have any plans to come home. I want to stay here as much as long as I can. If it ever comes to that I just wanna stay here as long as we can. And than if you wanna go into nowadays it's not just the over crouded, over populated areas. Now you have to think about climate change affects and how the salt is envading our wale waters and our lands water. Because that's a whole different (Laughs). There are wale waters in the outer islands that are no longer in use because they're just too salty. There are some here there are times it's salty and they just wait when it rains a lot then they should be usable again. Yea, that's the scary thing now, is when we have our king tides.

I: How often does that happen?

R: Usually when it's drout they come in. Because we're on drout now but the tides are not too high now. Usually king tide are, they're usually in just February. And then it bacame February, May, a few months a year. But this year we had them in February of course and then I think in April, I'm not too sure. But than it's just low now because we're in a different climate, different wheather pattern now. It hasn't been raining so much, still raining at least it's not super dry. We got visitors come in, like the crop agents they were like, it seems to be raining a lot you know, (Laughs). Are you sure it's not a dry season (End of story). And it's dry season coming up so like October to oh November through March is usually dry season because that's when winds come up. But we'll see, we'll see what happens. But going through a climate change is a scary thing, I'm sure you understand some other big issue. Especially right here.

I: So you mentioned there was like last year e-coli and hep b came up?

R: Well e-coli is almost here all the time but hep b and zika just came last year.

I: So for e-coli was it perusing high last year?

R: Maybe if we can get those number. Well yes, for us there were a lot of negative results. But that's almost all the time kind of varies. In the food service business now we have the, you can't get your card until your facility is tested we've seen some improvement in that area. But homes you can't really tell you know but we don't usually test homes too much, unless they are actually selling food we have to go test them. But deffinetly the water facilities and delis and the restraunts we've seen a little bit of improvement. We do have numbers if you want to see some of the data we do have some. Of course we have some modern data here.

I: So do you think people take e-coli seriously like if they're told that there's e-coli contaminated in water? Do you guys have awareness between like e-coli and illness?

R: Yes we see it everywhere. How do they keep that in... I do, we do. But I don't know if the rest of the population takes it seriously.

I: Like what do you think would it be preventing them from taking this seriously?

R: Laziness.

I: Yea, but laziness just come from lazy or from not having that knowledge. Is it educational factor or is it?

R: It's definetly not cultural because when you see how Marshallese were back then when you talk to the older folks, the elders. It was very clean, they were very clean. I don't know what happened? Because it's not just in our drinking in fresh water also in the lagoon, I mean we all go swimming in the lagoon that's just part of culturalism and it's there as well and it's probably worse there. I can't tell you about the fish I only know about the fish in Kwajlein. That's why I feel sorry for the hospital. I know they try to do it as much as they can but how much more can you do when they just don't want to listen I guess. I'm sorry I really don't know how to get this, I think it's... I mean how can you be a parent if you can't teach your kid, you know, your just teaching your children the wrong stuff. And you can't say this is how I was taught, because no parent would teach there kids to be like that. It's a lot of neglection, people are just popping out babies, I'm sorry, there just popping out babies all over the place without even concidering how to take of them most of these people have kids and they don't have jobs. Maybe they have jobs but their income is next to nothing. Like even my family, both of me and my husband work my daughter my oldest son they work. But it's still hard, still hard, most of us would date check to check. But then you look at these people that are getting 5 dollar checks 1 dollar checks it's like how the hell do you live? I mean if I go and buy food today for my family for like the next couple of days just grocery shopping it's almost a hundred bucks. It's really expensive. I mean like, I don't know how these people survives. I washed my clothes in the laundry mat and I see a lot of parents, I know they're parent. They come and eat there, they buy their ramen and anything like that I'd be looking and like, I wonder what their kids are eating? Cause here there are both of them eating here and just getting their fill and then they go home. So you wonder if they actually feed their kids. Like most of these schools they don't have feeding program. So when they do, in most cases my sister works at the education, she said it's probably their own meal of the day. In most cases. So that's most probably are stunt, no nourish children. And not only that it's also the westernesation of our culture. Because we used to be very dependent on our ocean and our local foods we're not as much now you know the staple have changed it's just rice and flour and only can, uglyness that's out there. It's all over the pacific. There's spam, cornbeef, and your yea. And we all know what's in there, they all know what's in there, but because it's cheap and it's rairly available where ever you go it's right there. And then ramen, (Laughs) the salt are the worse one, it's so cheap. And then you see these kids just walking around with their little packages they just pour hot water in and that's what they eat from like 50 cents or something like that. They dump the egg and eat the donut with the chocolate on top you know. It's hard, I don't want to say her name but the former secretary of health there was another workshop and these varies workshop that we have and this came up with the teen pregnancy and we're like, we're the number one in the whole pacific. RMI is number one in teen pregnancy, that is so embarrassing. You know what the secretary said? Well what can you do? We have to teach them how to take care of their kids.(Both Laughs). I was like WHAT! So it's not just the culture thing it's a religion thing also. You know this go forth and multiply?

I: You think that's having an impact on?

R: That's a huge impact. They don't teach these girls to prevent that. I mean all these years when I used to go to get my birth control, it's empty. I always joke with the nurses, I would be like, GO TO THE OPGY IT'S FULL! (Both Laughs). I always come like by myself like the service is like right to the end, I don't have to wait. You just walk in and get your shot and you leave. It's always full, continue to see it it's really sad. But now no, my daughter is pregnant. YEEEH, you know. And than it ends up with a lot of neglection and all of these abuse with all of that because they can't take care of their children and their self. So I'm sure that's incredible with all that domestic stuff.

I: Is that a huge issue here what you said?

R: From what I can see, yes. From my personal pointerview yes I see all over the place.

I: Like child abuse?

R: Yea. Neglection and abuse. I use to go, well, me and my husband we go walking in Marshall Island High School for exercise, and we'll see kids, when we go late like six, we go at six kids are still playing when we leave there they're still playing. Sometimes I like, aren't you supposed to be eating dinner? Go home take a shower and eat. They just stare at you. So you don't know if they you know? Yea it's sad it's just really sad, it's even more sad that most parents don't really like, they don't care or I don't know. I hope we get more positve respond in other places but it's a culture aspect, it's a religious aspect and I don't wanna say class I really hate that word I really hate that differentiate between... You take care of your child or you don't. Unfortunately the class, you can see there is a class and it's sad.

I: So which comes down to education.

R: But then yea, I don't. Our biggest issues are education and health. Obviously. There's too many kids, too many children being born and not all of them are able to go to school. And there's this issue on what happens after they graduate after high school not all of them go to college. So most of them just hang out and make trouble.

I: I don't know if you know but is there any national prevention strategies for teenage pregnancies?

R: Oh sure. Well we'll ask the health for that. And we've heard of some cases where the mothers or the grandmothers actually incourage their girls to go forth and get what you need. (Laughs). Get what we need, you know. So they send them to these boats and, you know the longest work in the history of women, they make them do that. And when they go home without anything they get beaten. That's one. Another one with the teen pregnancy thing well all of us pretty much know that it comes from somewhere in the house in most cases. Because these girls when they go they're single. So we hear that it comes from inside the house.

I: So is it inside the family members?

R: Yes, because when we walk into their house it's full there could be a little maybe a room like this but there's like 25 people in there or something like that. And when they do toilet... surveys then they come to my house, my house and my parents house, then they'd be like how many toilets facilities do you have? (whisper) 2, 3. They caught how many of us like yea that's just like 3 people to one toilet, something like that. Than you don't think that in most places they don't even have toilets facility, they don't have catchment systems you know. There's like 30 people living in that house, I mean it's here in the island. Maybe if I lived in a different area, I'd probably would be able to give you better answers or what your looking for, but where I live like I said it's almost all family in my area we all, like if my neighbors cousins needs something they come to my house or anything like that, if there's a power outage and we have something we'll share you know like that. So we share, you could only see it where I live. But if I lived somewhere else I'd probably be able to tell you, you know who the best people I think to interview are, the Mormons and the Jehova Witnesses. Because they walk into these communities and they talk with these people. I usually tell like when we do surveys we need to talk to these people espicially the elders, because they just walk around. They walk into the communities and they're there everyday you know. Just tell Helia, she'll know, but I think those are the ones that really see it because they're in these areas everyday. Like there are a lot of places in Majuro I've never been in years. I'd admit Even 20 years still, I've never walked into.

I: It feels like anywhere that you live and there are some places that you don't go right?

R: I don't go yea, it's like I just, it's either the main road or the back road but in between. I recomand talking... We're not gonna say their name.

I: Can I call you back to the public water system? Can you explain that a bit in terms of like who would have access to it like how's it a cost you, can you afford it?

R: I think it's... it's 12 dollars a month. That's how much I pay.(Laughs). Because, well it's not in my house, but I do have a tap outside my house. Like when it doesn't rain than that's when I use it to fill up my tank. But I just have a tap outside that's connected to the public water system but hardly use it but yea, I have one and it's 12 dollars a month. Befor you... Well when you applied for it EPA needs to clear it. You need to get a permit from EPA because it's a lot of digging. But anyway you need to get a permit from EPA, yea it's 12 dollars a month.

I: How much does the permit cost?

R: Oh god I don't know.(Laughs). I don't think it's that expensive just to get water. I think it's like 20. But it was just a one time thing that we had to spend 20 dollars. Like I said it has days like maybe 3 days a week if it's raining a lot it would probably go up to 5 days a week for 8 hours or so. When they're shortage it goes down to 4 hours in 3 days a week, it depends. Access to it I can't tell you, I don't know. You can get that from them on how many customers they have, but they would know that they would know how many homes have sewer systems in their homes toilet facility because you can get access to that as well. The salt water systems for flushing, the salt from the salt water in Majuro I'm sure. Because most use salt water for flushing.

I: Does it corrupt the pipes? The salt water.

R: Of course it does.(Both Laughing). I really didn't think about that. Yeah but so we've been talking using that for waste energy project as well. But because it's salt water the bacteria is different so we can't use it to develope the method that it needs to produce energy but it's all different.(Both Laughs). Sorry I used to work in energy source. So anyway, your asking me how many people have access to it? I can't really tell you.

I: But public water system is usually not use as the main source of water?

R: I don't think so. But our catchments the ones in Laura are the public water system there.

I: And by the airport?

R: Yea. Access to water I think the public access, I think you need to get because they can give you how many people are using it. But I don't think it's their main source, because like I said it's not on all the time. I know communities when it's on they all fill up whatever they needed to fill at home.

I: In Majuro you know your talking about how the water catchments can be contaminated and you guys go out and test you guys outreach programs to kind of educate people on boiling water espicially wale water and treating with clorox. In terms of count affects that is your not sure in terms that people are listening and actually boiling and things like that. Because we're trying to figure out like doing this research like what would people are trying to do to keep their water clean?

R: Yeah because clorox is not expensive, it's not expensive here. You hope that they do it, but you don't know if they actually do.

I: It might come down to not having any of like their attaching risk to not treating their water.

R: This is common sense. It's all about education, the parents should teach their kids because there's teen pregnancy it all contribute. Teen pregnancy...(Laughs) it all contribute to how that child is being raised. Because like, you know how you would say my parents used to disciplen me so much when I was young and then I don't wanna do that to my kids but and there are some where they would say I was beaten when I was young so your gonna get beat as much as I was. Some cases, well most cases is like that, this is how I was raised, this is how I was taught. Even in the schools, when I went to school you get the ruler or you get the yea, but now you don't. These days they don't usually do that.

I: So in schools, but at home it's a different story.

R: Yea at home it's a whole different story. When I used to work in energy, when we used to visit schools because you know we tell the kids to go back home and play with your parents. You know it's kinda like that, so we do that here too when we go to schools we tell the kids go and tell your parents that they need to do this. Sometime you know they say you can't teach the old dog new tricks. I understand their situation. It's hard. The sanitation issue is...

I: Yea yea that's a big one.

R: And I know I mention that every home has toilet. One of our staff here used to work in, he used to work in a project where they build compose toilet. Which is awsome, espicially when you do every culture and all that I'm also into compose system but here they're like yea. They think it's gross. So and I said so it's not gross to use this, the ocean? And then you go after and take a bath in it take a swim in it. That's not gross?(Laughs) I think our workshop are needed to be more forth right, I think they need to be more blunt. But that's when the cultural aspect comes in, I can't wait to do that here.

I: How would you go about that?

R: See it's hard. And then you can't go to a chief and tell them all this and say you need to tell these to your people.

I: Why not?

R: (Laughs) Yea, I mean cultrally you can't. You have to in most cases, oh yea you have to. Like I said how we teach our kids needs to be more blunt.

I: So when you said we can't go to the chief and say those things, what would prevent it?

R: It's not prevent, it's the culture. And to an old person you can't do that as well, it's a cultural thing.

I: Like tell them what to do?

R: Tell them what to do. Because if you did, they would like these kids come in and tell me what to do like, what the hell are you like you know, and I've heard that a lot. Like you can't come and tell me to put my trash here or not use this to because it's my property. We get that here a lot. Like it's my property I can put my trash anywhere I want you know. And even if you say this is what your trash is doing to your water, what it's doing to your if you have a garden or whatever. Like what do you care. Yea, we've had a lot of this like that where they chase our boys anywhere. Like I own this land I can do whatever I want to it.

I: Who would they listen to?

R: Well if it's the chief that's telling you this. So that's the clash between the government and the landowners. That's why it's really hard to do any development here. Sorry I used work at tourism too.

I: Oh yeah!(Both Laughs). Like a track of all traits?

R: I worked with a whole bunch of different hats. Don't play this because if anybody hear me they'll know who I am.(Laughs)

I: Don't worry about it.(Laughs)

R: Yea but that's where the clash of the leaders come in where the government and landowners. Let's not even mention the church because it's a whole a lot different.

I: So what's going on with the church?

R: Well it's what they say that the patient would listen to. So we've actually tried to when they do meetings during the summer, we've actually tried to... I think we did one time, do awareness in the church, we'll tell them whats your program can you like put us in and we'll come and talk about this, we've done that, so we try to do that again. We try to incourage the leaders of the church to continue to do it as well. Like everyday before you finish the program we ask them to say make sure to do this make sure to keep your... but they don't do it all the time. Even our radio station, if you do an announcement they'll just say for that period and that's it. They don't continuesly do it. Which is why we're trying to work with FM the radio station, because they play everything all the time. They don't stop playing it. Record something and they just continuesly play it including all the music and everything, so when you sing a little thing from the, they know it cause it's all the time. So that's what we wanna do with them and hopefully they'll pick it up and do it here but they just haven't, like make it consistent.

I: The message should be consistent?

R: Message should be consistent. So I know I can, my kids go to Assumption and they used to go to Coop as well so all of these messages are consistent in those schools. But you don't know how much it is in the public schools. About keeping your hands clean, keeping yourself clean and all the sanitation stuff. So those are taught in school we know that they do but we don't know if they do that in public schools. Like Coop for example, their menu is all healthy, they only have brown rice and for each meal, and they do breakfast as well but you have to pay for it of course. For lunch, they provide the monthly schedule to the parents, and it's all brown rice, it's all juice and they have the fruit and the vegetables in every meal, at that school. The restraunt is just short, you have to pick it up at home.

I: So Coop and Assumption are private schools?

R: Yea.

I: Are they for elementry school or?

R: Also high school. Of course Coop is probably the most expensive here. Assumption is okay it's not too bad. But than again you have to be able to affort to send your kids to those schools you know. That's why it's really important to be able to provide them with you know how they say it's all I can, I'm able to do. I send them to public school cause I can't send them to a private school, so you need to make sure that the public school has all of the resources and the capability needs.

I: So the public schools are there any like hygiene programs within the schools? You don't know. Yeah that's something to look into.

R: But deffenitley the private schools I know. And of course there are parents that take the initiative to buy hand sanitizers and all of that to donate to the class and we do that. Like you know how some moms you open their purse and there's sanitizer that's my purse.(Both Laughs). It's hard. But it has to start at home, so if it's difficult at home than there's actually no way that child will improve like you've given them the best education that they can get. When you see it you hear it, and it's really sad. There are many times where I've actually told women that, beat their kid, like that's a child how can you do that? They just stare at you like, stupid. That's what I tell them. And I've done it a lot. Yeah like what the hell are you doing?

I: So do think it's like, I don't know if you'd say, is it social norm to beat your child?

R: Yea. It's all over the place.

I: Have there been any programs like public health programs?

R: Yea. I know they do it, they do it a lot but, the social aspect is hard as well here. So I feel for education, I feel for health, and I feel for Internal Affairs because they're the ones really doing all things that are in it. We see it from the water part, we see it from the trash and solid waste part, but they're the ones that actually see how these kids are developed and that's hard, and you think it be managable considering, yea we do have a small population prepared in comparison right we'll be able to manage it.

I: It's hard to create culturalship to create...

R: You know it was all fine but you know when the westernazation coming in and the religions started coming in it just changed everything. Our diet changed our life style changed and yea. And of course the introduction of alcohol. Access to it, it's even more accessable now than it was. Because these chinese are bringing in all these cheap stuff that they can buy for a dollar. Yea like the hard harsh stuff for a dollar, 2 dollar or something like that. Yea they have access to it, easy.

I: Are there any messages coming from the church about drinking?

R: Oh yea all the time. So like pregnancy they talk about that in the church here. I went to church in the FSM in Pohnpei, I went to church one time and the lesson for the day, the teachings for the day was about pregnancy and sex and I thought that was awsome. Like wow I wish they can do that back home. You know talking about family and having kids and you know.

I: What's stoping them here?

R: Culture. Well you think that it is the culture because that's how you were told when your growing up you can't talk about things like that it's kinda taboo to talk things about that. So they won't talk about it in the church. Yea, I came back and I was telling my uncle he works at the church, he was like, I didn't say it like that, but I said they talk about things like that in the church and then they like discuss it afterwards you don't have discussion amongst the patient and I thought that was awsome, why can't you guys do it here. Like sex education is not taught in the schools, so public school I don't know, but private yes. Public school is exstensive it is being taught. You know kids right they have a different mind set but yea. But when that secratary said that thing back then when she said, we have to teach them how to take of their kids, that just blew me away and I was like how the hell are we supposed to control this if our leaders are like this.

I: Can we go back to the churches when you went to the church leaders. What information were you trying to get?

R: Here I have to check what they've done it. You can do it, they'll let you do it but for them to continue with that message you don't know how far they will continue with that message, but they'll let you go and do your presentation. The thing about the protestant church is when they have their annual or bible meetings... so many people come in especially outer islands they don't usually have a place to stay. So they stay in that old school like the protestant church in Uliga they used to have a school in the back, it's not a school anymore. So they use those like apartments when these people come in. So of course there's not enough toilet facilities because sometimes there are over a thousand people that come in just for this church activities. All around, couple of states so yea they'll come in and we'll have like a thousand people. So where do they go to use a toilet?

I: The beach.

R: Yea, those that live in those area they always get so mad when church meeting season, they always get so mad because they're like (whooosh) all over there. So when we have our king tides and all this stuff washes up into the yea it's terrible.

I: Like where children play?

R: Yea. I don't even want to talk about ebye.

I: About where?

R: Ebye. On Ebye there... a place I'm sure you've probably heard that are Majuro Water and Sewage Company, we just pump everything out into the ocean right, okay but it goes way out there in the ocean. It mainly just disolves but in Ebye it's a different situation. It goes into the lagoon. Yea, so right now they're redoing it of course, it's an envy project but yea. So they have that going into the lagoon and anything else goes into the lagoon. They have all of these old military and even new, the old military poisonus PCBs and things like that, so right now there's actually a fish contamination unit that's been set up by the President because of the lead situation on Ebye. So that's one place you guys need to track, because there's a lead devomilate fish there. So we're dispersing the population from eating the fish on Ebye and yea that's serious. The sewage water goes into the lagoon and the fish are contaminated. So like I said my sister works in the education she said they're already seeing it the decline. They're already seeing the decline on educations of kids on Ebye.

I: How long has it been contaminated do you know?

R: Well the military been there for how long..? So we're working with them with cleaning up and they're removing all of the old stuff they're supposed to remove the old transformers and everything like that.

I: Who's doing that? The US Government?

R: Yes. So we're part of there's this group we say USACA this is Kwajlein, USACA the environmental group that's us and USPA included NOAH we're all part of this group, so we manage that which is fine then in Kwajlein it's fine they just are here for everything in Ebye it's a whole different story. When I'm there I always tell them why can't you just do what your diong in Ebye but when you go to Kwajlein, because when your in Ebye you see all the trash from the, you throw it everywere you want and then you go to Kwajlein you look for a pin and dump it in the pin.

I: What's the differents?

R: What's the differents. You know if it was a toilet, you look for a toilet in but here you go everywhere you want you know, why is it so different, there right there and you see it all the time, you think you learn from it because your doing it there but you don't do it here, why?

I: Do you have any answers for that?

R: Well because it's enforce, if I do something wrong they won't let me go there anymore.

I: In Kwajlein?

R: In Kwajlein.

I: So the law is being enforced there?

R: There enforced there, they even enforce our laws as well on Kwajlein. So it's education, it's enforcement, it's yea. Here going into sanitation it's a lot to do so much because... limited space, even more limited space than how it was then before. I'm sure if you walk into Jenrok you'd probably know there's not enough space to connect all of those houses to the sewer system or build toilet facilities. I don't know I'm just talking all over the place.

I: That's just great your giving such great information. In terms of enforcement, Are there regulation for houses having tap toilets here in Majuro?

R: Not for them to have because not homes, we don't have building codes, they're developing building codes now but, I'm sure you've seen a lot of houses that they're just like matching putting little piles together so not everybody has toilet facility. But when they do they have to come here to get a permit to dig to get their sewer. So we may have, but I don't know if it's collected I'll have to check on how many homes actually have toilet facilities, but I'm sure EPPSO has that. Because they do their annual surveys, on how many homes have, cause they just did it when they did the survey in a couple years ago. So yea, get those numbers.

I: Isn't the demographic health survey the DHS?

R: Yea, I think that was one of them. That's the one that caught how many toilets you have.(Both Laughs).

I: Yea we'll look into that. So you guys tried to...(Disrupted)

R: Yea but we do, but not... it's not a requirement.

I: It's not a requirement?

R: Yea. So we don't go to homes and say yea your required to have a toilet. Unless it's the homes that are build under the Development Bank or anything like that housing programs and yes they do have toilet facility. But the ones there they're just patching up than.

I: How common you say it like open defecation in using the the beach?

R: What I have noticed is even if they do have toilet house they don't keep it clean. There are a lot of people they think that's where what you do is dirty in the toilet so it's like why do we clean it? Because it's like where the dirty stuff. You know it's like where's the perception, sorry it's a lot of common sense but, you say that to people too like where is your common sense? Can't you just think and they say no I shouldn't do this because that's what...yea.

I: So when their toilets are not dirty it's like...(Disrupt).

R: If it's dirty they're not gonna wanna use it because it's dirty so they'll go somewhere else. Like they'll go to the beach cause the water comes and washes it away and from their thinking it disappears. Even if you tell them don't swim in the lagoon cause we do costal testing as well. So we'll do announcements saying this area this area this area can not go swimming because it's contaminated with e-coli for example. We do announcement on the radio and they don't care they just don't listen and they continue on what they're continueing. We still have pigs in the costal area even they're not supposed to. So whenever we so those we try to stop them but they still do it.

I: The pigs, are there regulations in terms of keeping animals in Majuro?

R: ... Not regulation but your not supposed to have pig pin that goes washes straight into the lagoon, all pig pins are suppose to have either connected to the sewage system or have their own insulin collection they have to have cement at the bottom. Like in Laura, Laura is doing so well they have a dry litter system in Laura.

I: Okay, what is that?

R: That is use... greens, like old greens and stuff, and than compose. So they use that there but not here in Majuro. We try to discourage it as much as possible but like I said, it's my land who are you to come tell me what to do with it. So there are so much you can do even though you go with the regulations and you go with your fines. So yes we've been making, we're surviving with fines here at EPA.

I: (Laughs) It's good bussiness.

R: It's good bussiness unfortunately. We're not supposed to be making this much money from waste and (Laughs) violations. We just wanna tire those people out there. I think improving the education system, I think there's a lot more that the health facilities can do. I know they're trying but I don't think they're doing as much as they can, there are so much more they can do. I have an uncle that work there, he's a doctor there. He was based in Palau, so I'm sure you've heard of Palau or...

I: Not too much.

R: Oh not too much. They didn't have a bigger population as we do but... I think they do so much better they have a whole lot of programs, they're in the community a lot, they do a lot of awareness and you know because whenever you go there they're doing it. Here they'll do it but not as, they can be more consistent I think, they need a stronger awareness group at the health facilities. I think consistency is important. They need to be more consistent. WUTMI and education have this parents as teachers program I don't know where that is I wonder if it's still. This is where they actually teach the parents on how to raise their kids and education at home I don't know it's PAT, they it's the PAT system. I think it's still there but I don't know or I haven't heard anything about it. But than again it goes all the way back to all of these kids having babies, babies having babies and how much can you teach them when they're not educated enough to work. So all they can do is 2, 3 dollars an hour jobs that are you know. Our minimum wage was just raised which it's raised this year and it's 3 dollars an hour only 3. Like the highest employer in private sector which are the fish companies, it's like $1.50 an hour. And that's where a lot of these people work because that's as far as they can go you know. Which is sad but, I think we need to, I don't know where it is now, I haven't really followed up on where their salaries are at now but the government has raised it to 3 dollars an hour for both public and private. But those were kind of weighed because they're new, this is one they were new, they were just starting up but they've been here for awhile now I don't know if it gone up or if they're actually following up the minimum wage.

I: That's really low for the price list.

R: Really though it's really expensive here. And you go to these places and you look at your bag you know your shopping bag and your like how the hell is this 80 bucks you know?

I: Exactly what I thought. Yea.

R: And you try to eat healthy but you can't because it's so expensive you know. All of the vegetables here most of them are important but it's expensive.

I: Yea and they're not always fresh?

R: Yea yea. Good thing for us is my aunt has a garden in Laura. So she'll bring like eggplants and cucumbers whenever she has and lend us anything that she has to bring to my mom. So we usually have those all the time, but to buy additional stuff it's expensive. We don't need greens all the time but I try to have as much as possible as I can.

I: In terms of the Ministry being more, face of they can do more what would the consistent...(Disruption)

R: I think consistent is improtant especially in hearing, because hearing and seeing, cause when we do like presentation like when I came here I told them we need to do more like pictures and illustrations rather than just reading the words, because if it's just reading or if you go and give this to a Marshallese person they won't they'll just throw it you know. But if I gave you one with photos and drawings you know, they'd be like ooh, fun. They'll pay attention to it, but if you just put all of these report and everything like that especially numbers. When we were doing presentation and MOH are doing their presentation they're like in two thousand whatever this and this and this and we're like (whisper murmuring) like who cares. That's what I've been working with Christina is, our reporting now when we do our quarterlies, it's almost all pictures and it's like this it's all different colors and it's like that. Just this year we just started this year with different format for quarterly reports and all the different reports are like wooow your report is so fun.(Both Laughs). I was like wow so this facility was like this and this and they pay attention now, we've been telling them it's been negative for the longest time and we're just toying them and nobody noticed it and now they do. Which is good because now they're paying more attention if they're seeing these things that are happening. So we've kinda change our format reporting to the public. We're doing more photos and more with these different color and just making it more interesting and our presentation as well we are also changing those, making them more see than more hearing. Like I'd go up there and read, okay our focus area for this year are this and this. I wouldn't lose a crowd like (Snaped her finger) like that.

I: Where do think the message should be given so that people could hear it?

R: Schools, that posters and things, those should go into the schools. Community areas, where they tend to group... Churches, most churches have like meeting areas. If they allow us to post those on the wall we'll do it you know.

I: Have you tried?

R: I don't know if they've tried.

I: It seems like the church leaders would be they're willing but kinda have that champion that will grow.

R: Yea, we need a champion that's a real champion like the champion is just sits there and. We've had champions that there was a water champion I can't remember and she was really active. That was a few years back. We had like drout back then and they were just like all over the place talking to the community. So that's a good champion we need champion like that, that actually show face and not just the name and the title.

I: And like from the community they know people?

R: Yea. So diffenetly churches some place to go. We need to do more in community instruction like going into these communities setting up community meeting and things like that. I think those things need to be more. I know they're doing house to house but that is so much work.

I: What would be your propose?

R: ....... Oh god, where do you start? ... Okay in the local government they have a councilman for each jurisdiction like Majuro has 7 jurisdictions. Maybe we could work with that, on doing more meetings like bringing the community together, but than that takes money that's the thing. Like if you set up a meeting most would say is there any food? Because if there's no food than we won't come. You think they're making a joke but they're serious. There's no food they won't show up. When I used to work in tourism we used to group up and go to like if there's a councilman in Laura, and fine a town hall meeting in my jurisdiction you get whatever you want to present you know anything like that. But you don't hear those all the time.

I: Like a location or...?

R: Like a call from the Councilman calling for a meeting. You don't hear those all the time I think they need to work more with these, these local leaders. Because like I said all these jurisdiction like in Laura they have their own like some of the areas they have their own, like a town hall. Like Laura they have, Jaydeff have a town hall and that's where EPA base is as well. So we'd have our, he's here most of the time but when he's in Laura that's where he works with the community. That's where we have all of our fliers and things like that, there in this town hall in Laura. So we utilized it and we're there so Juluis is there so he's consistent, he's there and he goes through with all the, he works with all the community there with the water and all of that. So it's like, somebody needs to be there all the time and consistent go through the house all the time and it can get irritating but, maybe they'll changed if we continue consistent. Because if we do like these one offs. Then most cases everything is one off. Well I say it's one off because we'll do it now and we'll do anything until 2 years later or something like that.

I: Interest of higher key the Councilman compared to the land owner?

R: The Councilman works with the land owner.

I: With the land owner, okay.

R: So it's the Mayor of course in that it's the Mayor and the Councilman and than they will. Mayor sorry Executives and than the Councilman. But they all have to work with land owners. So the land owners like to keep and sell it to fews that actually sits on the Council local government. You know when we go back to this champion. Can you use your authorities as much as you can to educate? Not use to not use your authority, but use your authority to educate where you stand. I mean it could it could work but it just takes a lot of work. Bring them and convince the land owners. There are some land owners here that have actually taken initiative, so there are some that have taken initiative to like keeping their area clean they do want their area to stay clean. We've been trying to as much as we can like incourage it like... We want to do like competition on things like that but again it takes money. Whenever earthday comes along like anything like that but these all comes once year thing. So we can't, the consistency can't. Like if we do like weekly, like Rita weekly beach clean up. Can you imagine? It would be great, but than they're gonna be like how come nobody is paying us to do this. It's always about the money. Initiative, money, consistency (Laughs).

I: Just in terms of garbage they were talking about, is there, can you talk about garbage disposal and collection system?

R: We have a weekly collection system, yea but it's mainly DUD area.

I: What's DUD area?

R: Rita to the airport.

I: Oh from Rita to the airport, okay. What happens in Laura than?

R: That's where the Coucilman takes initiative. So they work with their community their jurisdiction and... This is a good person for you to talk to for what he's doing, Jina David... He is a Councilman for one of the jurisdiction in Laura but this is a person with initiative, you can tell Helia I think she know him. But he has utilized his funds in the local government to build... collection pins in Laura and the dogs can not get into any animal can not because it's a full enclosure that he provides each household in his jurisdiction and they all fill it up with their trash and weekly he uses his local government truck and local government goes and pick it up and collect it from the. He makes sure they put it in a trash bags and if it's a green waste he makes sure they put them in blue bags. Yea he's really good. So we need more people like him, so he does that and works with the local government but the local government is the ones that pretty much collects the trash pins out there because there's not one collection point where that area, there trying to Malcus is working on it trying to because so far it's a lot of work for the collection trucks. It's mostly the local government that brings in the trucks, but not everything is collected of course because there's not enough collecting system, here it's easy to dump anywhere you want and not in a pin. The urban area.

I: Why is it easier?

R: I don't know, for them I guess, for people. Yea you know everywhere you go there's trash everywhere.

I: Why is that, is there like no pins so people can dump them?

R: Even if you say, you put a pin they'll put a pin there and some they do some but I guess it's just easier for them to throw trash where ever they want. But again it goes down to education and again how your being taught at home and yea. This is my land thing again I'll throw trash where ever I want. You don't see it when it goes into the water you know.(Laughs) I mean if you dump it in the ocean it disappears, that's what they think. So it all goes down to the education the consistency of cleaning up and disposing of it. Because here land is very limited I think that's our third... dump and the last 40 years maybe, on Majuro. 1,2,3,4... maybe 4 sights. But I think that's the one that's been used the longest before and it's here now and you see the mountain of trash. I know they're thinking about waste energy project but who knows if that'll ever go through.

I: Does it get washed out or how do they get rid of the garbage?

R: It's there. I don't how many years of trash it's been there. Does it like get in the king tides or storm surge and typhoon even?

I: Yea, do they burn it at all?

R: Some. They did used incenarator back then but it didn't work well. We've talked about incenaration before but... the response was we don't have enough trash to keep it to sustain it because most incenarators have to be on all the time, but I think it's changed now you know the technology has changed, but if they're not getting it it's already there, but they can't burn everything. So not everything can be burned. This waste energy project is... I mean they're burning waste oil which is good and some trash and of course your gonna have to deal with the ash and all of that, that's when EPA comes in. Like on Thursday when they collect our area, our area's on Thursdays in Uliga. We always look in our trash pin and then I'm just like my house produces so much trash, not my house like the main house. Then I'm just looking at these pins and they're totally empty I mean like where's it going? Because whenever we pulled out our trash pin everything is almost open because there's so much trash and we just pile everything thean, which is bad.

I: Are people still throwing it in the ocean side?

R: Yea... Oceans day are my... I have a granny that passed away so we did a foundation for her, so our group did a clean up behind the you know office mart?

I: I've seen office mart yea.

R: Yea, so in that costal area in the back we did a clean up. We collected a... there were hundreds bags of trash, I think we had like 60 blue bags, like cans and recycle items. We won the second prize for the most trash collecter for that area, for that day and then we won another prize here for the weight of the trash we've collected because we took scrabs, everything like that. And all the kids from that town were like looking over their seawalls and sat there and staring at us and I was like do you like this? And some were in the water they were like just paddling on these made ships, and I knew one of them and I was like what the hell are you doing in this water? Get out of the water. And she was like, what do you think?(Laughs) It was (gag) it was gross, there was food to everything, animals dead animals, you name it it was there.

I: So what were peoples perception of that?

R: That's the thing it's like why has this become normal to them. I guess they see it everywhere they think it's normal cause they see it everywhere. When EPA took the initiative to banned plastic bags.

I: When did that happen?

R: It's been on going now for this will be our 30 years, but it took affect last year. But they're still around, because you'll see them the palastic shopping bags, this is not the grocery bag or a trash bag but this is the actuall shopping bags that's the one we banned from the stores. You still see it some of it but you know those are illegally coming through because they can not come through the government, this is a government regulation. You don't see that as much as you used to before. Have you been here before?

I: No this is my first time.

R: Yea yea yea, but if you were here like 2 years ago, you would of seen the difference because there were plastic bags all over the place. So last month or was it this month we... This month, what month is this? August?

I: August.

R: We just launch our CDO the Container Deposit Program, this is for every cans, bottles and glass bottles we get 5 cents back. So we just launched it like last month. So when you come back you won't see any cans or your not gonna see anymore hopefully. So people are doing it, they're sending us their little copies of receipt to ensure that people are going and deposit their cans and... So the cans are out they're exported for recycling. Right now we're working with the waste company to find recyclers for the bottles and or anything that is plastic to collect. But than the glass we're not sure if they want it to get crush or crush it for like cement or anything. Diffenetly the cans are exported for recycling they already have a contest. So they make money there but they're also making money from the facility that gets a penny back for each can, so whenever one of these come in they're shipped in we charged them for addition 6 cents per can, so it's probably gonna get expensive for cans and all the storage, but we haven't seen that yet it's still a dollar for a soda. But they pay back, it's not a tax, it's a deposit fee. So that goes to the waste company and you go and get your claim your 5 cents back. We're worried if that would harm the insurance, the consumer, but so far it hasn't. There are some stores that you see that we charge a environment fee is what they call it, for 6 cents. So you'll probably go buy a soda for a dollar 6. So if you see a dollar six than that's what it is. You pay for it then, that was launch last month, but the plastic banned that's been in affect for 2 years and plastic and styrofoam. Styrofoam plates, styrofoam cups, so those are banned as well. So you'll see mostly of those now.

I: Paper cups?

R: Paper cups and we've tried, of course we've tried incouraging using reusable bags, so you'll probably see those in stores as well. So those are all PPA initiative. So we're thinking of doing some other things as well like okay we'll give them a couple years to get that in their brains and then next we'll do something else. You know slowly but surely. But at least these are consistent and we enforce. Like I said we're making money. And you see it, we saw it like immediately that's when we get started and we don't see the plastic as what they used to be before when they're all over the place. I don't dive, but if I did dive I would probably tell you what it looks like under water I think you'd probably ask somebody who knows how to dive. My daughter dives but she's not here. She's in school. But she works for MIMRA so most time she'll come and say it's really clean there's nothing there so. Like when we had our storm in 2014, there was a big storm and we had couple months of waves just coming in so it cleaned out the lagoon I think, there was a lot of trash coming over and just for the past 6 to 8 months I've noticed and I'm not used to, because we have a ramp at our house a pool ramp, so it's always full of trash but then for the past 6 to 8 months it's been cleaned and then again it's like a couple weeks ago of complaining again because there is a lot of trash coming in so people are they're doing it again because it was clean for awhile and now it's dirty again.

I: Why do you think it was clean for awhile?

R: Like I said, just a couple months the waves coming in. So everybody just clean it up everyone like, when it came over my ramp, I was telling my cousins and my sisters and they were like I'm sure if it's like that in other area they would just swim in it. But we were collecting and almost everyday we were collecting trash from my house.

I: Is there a perception like when you throw garbage into the ocean the water takes it away?

R: Yea yea. That's a perception.

I: But yea at the same time there's so much garbage in the beaches that are still there?

R: Yea, and then like eventually the water will just take it away.

I: Eventually?

R: Yea.

I: We're also interested in how people dispose of like young children stools?

R: Mostly diapers. You know like said that place that we cleaned up there were a whole bunch of those as well. We actually issued a fine for the land owner at that office mart area, we actually issued a fine and they've been monitoring but we told those houses there if they're gonna keep doing it we'll charge them. So they'll have to think where are they gonna get the a thousand, 2 thousand fine to pay off their fine, if they'll continue.

I: Do they pay their fines?

R: They pay, like mostly the company they pay. We don't really issue individual fines but we warn them if they're gonna do it, some get scared and stop doing it for awhile. So we have to be consistent on our water part as well. So when we're consistent with plastic bags and like that that is working but being consistent going through homes you can't do it all the time, there's only, here, there's less than 20 of us here in this building. There's not enough of us to go out and enforce it as much as we want to.

I: In terms of fining the land owners, so when you fine the land owners they have to pay or is there anything about the land owners being involved?

R: They'll come and negotiate most time they don't actually pay money but they'll say we'll clean up here and we'll make sure that the homes or the bussiness there have trash pins or anything like that. You know they come and try to negotiate with the general manager to get rid of that fines that we've given to them. Like I said they'll do it for awhile. So we have to be consistent to continue to do it.

I: Behavior change and your consistency and you need enforcement right?

R: Yea, so... But don't ask me when it comes to politics. Those above the law and stuff (Laughs). The ones that they think they're above the law (Laughs) those are the worst.

I: Oh yea so we heard that, like you said that pigs are suppose to be in pins that are attach to the sewer but some land owners have their pigs running around and the police don't fine them because they're land owners.

R: But however if the pig runs into your land you can claim it.

I: Oh really?

R: That's the law. So lets say if they break out of their pins and they go roaming and come into your property you can kill it when it comes into your property or you can claim it and your gonna say well it was on my property you know. But you know there is a really funny story of the record if you want, we have couple islands out here and one of our islands there's a family that owns pigs and when they're roaming around the island we'll like (whispers) okay it's in our property now take it take it (Both Laughs). So they have to make sure it's safe in their pins or it's their lost. Same as chickens. Yea you could do it here, but than of course if it's the Chiefs nobody's gonna touch it. That's the issue out here one of the Chief out here has pigs all over the place and you'll see them crossing the road but nobody will really touch them.

I: Yea because there's a huge issue that children are playing around and you have pigs kinda defecating or dogs all over the place.

R: Yea, it's not as bad as it was before I mean there was a lot of stray dogs there were a whole lot of stray dogs before. But here is we don't train our pets, like how it is in the state or where ever. So if you have a dog you have a dog. Well they just stay at your house of course but if they start roaming then it cause troubles.

I: Was there a stray dog for family like animal conrol?

R: Yes. Our local government do that but people started complaining because they, because say you have to register your dog and most dogs have to have tags. The issue of tags actually for your dog but see that's not consistent this is like what 2 years ago, but I haven't seen any new tags. But diapers for babies and children we're hoping that most of that goes into the dump again the ocean is right there.

I: I know and the kids are playing right there too.

R: Yea. I hope you've notice but there's a lot of growth here that's all from increase protein from the water. I went to MIR but I barely go there and we went there one time and I was like this is here too ewwww, (Laughs). So there was one at my house I saw it after awhile but it's gone now. But the seaweed is gone, because when they come they did a survey by our house because Micheal was building a seawall and he was like I don't know if I wanna allow you guys to that because your placing, the coral is healthy there's so many fish and it was just like it was just that block from where house is(Laughs), there's that many trash but right now we have neighbors that are collecting tires, because he wants to use it for seawall but there just there and when there is like waves come in there's like tires all over the place.

I: When like for example if you have an area in your back yard and you clean and you make sure it's clean, does that inspire people to do the same?

R: No.

I: How that precieve like what do they what do people think about other people?

R: They have a clean house. Their place is always clean.

I: So they're just thinking maybe like if there's positive examples or if there's a...?

R: Nope. Like you'd think that it would but I don't know. I don't see it, like you'd go to a town I guess I'm just saying and maybe 2 or 3 houses are really beautiful, they have a lot of plants and everythings clean and then maybe that house would be like that and than this one would be like (gags) and another one would be like you know (claps).

I: There like more individual?

R: Yea it's more like they're more individual. Again it's my place what do you care you know. I guess you'd also say you'd also think like if I was a Chief if I was a main land owner or Alap that how what your call, for this area, you'd think that I'd take the initiative talking to the people that are living in my land to make sure that they're keeping it clean. Like I said there are some that have taken initiative but not all. What is important is, okay I have a birthday coming up and I need 25 dollar from each household. That's what they do here.

I: The land owners?

R: Yea. Sorry that's...

I: Yea no no that's interesting.

R: I'd probably get in trouble for that. (Both Laughs)

I: How many land owner are there in Majuro?

R: Oh god... Okay for a parcel the weto is a parcel... how many wetos are in Majuro? It could be 300 maybe. Not all have different, but most land owners can like own 3, 4, 5 different wetos. But Chiefs... maybe 4.

I: 4 Chiefs in Majuro? Okay.

R: I think, yea maybe 4. Because there's some area where there are no Chiefs. When the Chief died he didn't pass on to anyone else. So they are managed by the Alaps, so there are areas in Majuro like that they don't have Chiefs. So you think yea.

I: No we're just trying to figure out like what would be the way to get messages that people would be more likely to take action and yea.

R: Your land owner deffinetly, your Chief, your church leader and your Councilman. Because those, if you wanna say our government leader they're so way up there, their message won't go down as much as they would wanted to and they probably won't even, they're probably the worst defenders. Like said they're above the law. And they're the ones that partner up with these chinese companies and these chinese companies that we fined then when they want to do something you'll see the Senator coming in like why are you fining my store? And we're like your store? We kinda have that kind as well. I think community level is always the best way, but educate your community I think that's where consistent the community.

I: The case where Jina at laura like how that was very effective what would you say where the factor that needs that case?

R: His backround.

I: So was he like a champion?

R: He has been working with the community for a long time he used to work with the Youth to Youth so I think it's just how he's been, he's been working with the community. Like somebody that takes initiative and he's close land as well he's a Majuro person like I'm a Majuro person and I do what I can on where I live I can't really do anything everywhere. Like when we clean up our area, the weird thing about my house is off the main road there's just one road that goes into most of the houses that goes to the like if this is my house the houses on this side you can only access to this house from the road that goes into my house. So when we clean, clean from the main road and the back road that goes to those houses in the middle of our area and sometimes I say if your gonna use this road you have to clean it up as well it doesn't mean that it's in our area we're only suppose to clean it because you litter it as well. They don't, and there was a time where we actually said we're gonna shut this road down.

I: You can do that?

R: Yea I can do it because it's part of my list. That road is in my list this road that accesses to their houses off the main road, it's in my moms list and I said we're gonna close it up because you guys are just you know, your not doing anything your not cleaning it your not doing any, what's the use of being there inservicing you when you don't service it. So they've been trying to I guess it's not consistent. So if you come off the main road if you just go straight you'll come into my house but if it's just the road at the back the one that has access to the houses than it's to the right of our house I mean to the left of our house. And you clean it up but they don't but their houses are clean but the ones that lives around the water one is a Senator well two of them are Senators. One is okay the other Senator has a family has a seawall that even when it was enclosed they were dumping trash they were like crazy. This is before I worked for EPA and we had one of our king tides and they all came up, because my house is one of those homes where you can actually sit and look at the lagoon the lagoon is right infront of you we have parties there it one of homes like that and auntys house as well. But most home when you go there it's closed and they don't have access to the beach. I have access to the beach, so this time we woke up in the morning right after the king tide like 4 A.M where it's usually the highest king tides our highest tides there was this line of trash bag from that house all the way up maybe like 30 big trash bags like actuall trash bags. Like we called our Councilman number 1 call up EPA when they come in there's like trash bag full of trash but at least there weren't any trash bags. What you think they would just go out and collect it but nobody collect it I don't know what they did here we just report it. But the cosmic came in and like ohhhhh so that's from their area and they're like okay alright.

I: At this point garbage pins are there and they couldn't just throw it?

R: Yea, they couldn't just but they were building a seawall so here most seawalls they'll dump their trash in first to fill it up and then put sand on top, but this was like when the seawall wasn't enclose yet, but they started dumping in it, they were just starting to build it.

I: So the garbage was for creating the seawall?

R: No no no, they were building the seawall but it wasn't fully enclosed yet, they just started building it. But they were already starting fill it up when it wasn't even enclosed yet. So that's one thing you will do here, with the last storm we had we recieved funding the RMI recieved funding to build seawalls so in the last 2 years we have like almost all Majuro is not seawalled not like before. So it's gonna be interesting when we have our storm surges and things like that, the winds that comes in because it's gonna be a whole different circulation, so it's gonna be scary for some maybe scary for most because all of the seawalls should of been built. Like I said my uncle lives in a building that's one because his seawall was destroyed and his boat hit our house. His boat started coming it was low tide so it was like we got the people off the boat it was really hard because the waves were really hard it was strong but when we finally got them off the boat and the tides started coming. And all of us was standing outside of my house because my parents house was higher and it's elevated so all of was at the bottom just like staring at this big boat coming toward our house so it came wabling up and down towards our house and then it turns this is my house it just started (whooosh) this is my parents place it's higher and it just went like that like right under my parents and I was just like... we have pictures it was scary and we were like staring at it it just went like that it never really hit our house but then as soon as it got to where the ramp is it turned and head straight into my aunt seawall and went like (boooom) destroyed one of their old shacks destroyed that and ran into the end of the seawall and just broke everything and then park there for a couple of years after maybe this is in 2014 and I think they were finally able to move it in 2016 it was the last one that they had to move but it was so hard to move it. But yea that was really scary. This is when my dad was really active with all the climate changes. I have pictures of him and the boat was like. But yea that was really scary. Well anyway talk to Jina about the trash stuff e-mail me anytime you have questions about the water stuff we can give you some numbers we're more than happy to work with you we've done a whole lot of work with hospital especially on the zika stuff and hep b stuff on the sanitation we've worked with them as well we're part of a group that goes out and check the water and restraunt for expire food and anything like that. We also check the kitchen and things like that. But anyway if you have any other questions I'm sorry I'm just jumping all over the place.

I: No it's amazing thank you.
