# Supplementary material for: Conceptualizing multi-level determinants of infant and young child nutrition in the Republic of Marshall Islands–a socio-ecological perspective
Source: PLOS Glob Public Health. 2022 Dec 19;2(12):e0001343. doi: 10.1371/journal.pgph.0001343 (PMC10022247; doi:10.1371/journal.pgph.0001343)
Supplement: S1 Data — (ZIP) [file pgph.0001343.s001.zip › RMI Supp Data/Interviews data/I31U_IDI_FCG_Rita_Aug 20_Libon.docx]

Interview code: I31U

Interview type and interviewer: In-depth Interview

Interview Date: August 20 2018

Location: Rita

Interviewer: Libon- Female caregiver

Transcriber: Fela

**I: Ok. We can start now. Do you agree and take your part doing this survey.**

R: Yes

**I: Thank you for giving us your time to speak with us today. The information we learn here will help us find ways to improve maternal and child health and sanitation in this country.**

**I: to begin with, can you please tell me a little about your family/household?**

R: silent (long pause)

**I: can tell me who live in this house, or who live here with you, how many children you have?**

R: in this house, me and my parents, my older sister and a younger brother, my brother’s son, my two sons, and the head of this family is my husband.

**I: how many children in this house and can you please tell on the ages of these children?**

R: my eldest son is seven years old, my other son is nine months old. Sam is my six and he is the son of my older sister and Miel, I am not really sure how old he is now.

**I: there is no female child?**

R: yes there is no female child in this house

**I: Next, can you describe a little about this community?**

R: in this community, I think it is only a small town but there are plenty people live in it.

**I: Now can you describe the positive things about this community?**

R: The good things in this community or this town, people sometimes gather together during parties, funeral or also can be during birthday celebrations.

**I: what about the negative things in this community?**

R: the negative things in this community is lot of drunk people, and there is no playground for the children. The children don’t have playground so they go and play at the main road, dump site, or can be at the lagoon side.

**I: Let’s now talk about health and illness in this family**

**I: Can you tell me about some of the illness that your children have suffered from?**

R: the common illness that children get are diarrhea and fever.

**I: are there any traditional illness happened to them?**

R: my older son got illness like skin rash and I used local medicine to heal the skin rashes from his body

**I: what are the causes of the skin rash illness or fever?**

R: this can be resulting in traditional doing or black magic. Diarrhea happen when children don’t wash their hands before eat or after using the bathroom. Also happened from dirt or unclean drinking water.

**I: and what are the causes of fever?**

R: can also cause from the weather, or heat from the sun or places where they play and there is no wind or air in the atmosphere where they play.

**I: what are the seriousness of diarrhea?**

R: seriousness?

**I: when he get diarrhea, what happen to the child?**

R: dehydrated, getting skinnier, and can cause the stomach to get bigger.

**I: and what about fever?**

R: they can be stuck or can’t even move and also refuse to eat.

**I: how would you prevent illness that you just mentioned?**

R: give them medicine, give foods and water.

**I: Can you describe how you know when your child needs treatment for their illness?**

R: silent

**I: How do you know that the child really need to go see the doctors?**

R: there is nothing else I can do for him.

**I: you said there is nothing else you can do for the child, like what?**

R: I am trying the thermometer for the fever and the fever keep getting higher and higher.

**I: who would be the first one you bring the child to the healthcare and reason why?**

R: my mother

**I: why do you bring him to your mother?**

R: because she has better experience with the sickness than me

**I: do you usually used traditional medicine?**

R: yes sometimes when the fever is too high I used traditional medicine like the noni leave to help prevent the fever.

**I: can you describe in detail how do you make the medicine for the kid and what are the steps in making the medicine for the child?**

R: I bring the noni seed and pound it together with the oil and then massage the baby with it.

**I: when you do the massage with the medicine, does the fever go away?**

R: yes the fever quick heal.

**I: can you describe any illness affecting your children that are associated with nutrition?**

R: there is none

**I: are there any illness caused by foods missing from the diet?**

R: skin rash, skinny, the stomach get big, get sick most frequent.

**I: we talked a lot about being unhealthy. Could you now describe for me a typical day of someone living a healthy lifestyle, from the time they wake up in the morning until when they go to bed?**

R: people with good health, when they wake up, you can see that they feel very well and healthy. They move around the house and do house chores. They’re being nice to people and they don’t hate people for no reason and they have good or nice faces.

**I: that’s great and yes you are right. So what are the appearances or signs of a healthy child under two years old?**

R: under two years old?

**I: yes**

R: the child have a clean body. They always play and not cry all the time

**I: and what about a healthy adult. What are the appearance or signs of a healthy adult?**

R: they also have nice and smiley faces and would walk or run around. They always feel healthy and can do movement things around their houses.

**I: Let’s now discuss hand washing. Could you describe in detail your family’s hand washing throughout the day?**

R: the family wash their hands, the adult would bring a bucket so that whenever someone come inside, they wash their hands before eat and they wash their hands with soap. Whenever the children play outside, and before they come inside the house, they would need to wash their hands.

**I: do the children wash their hands throughout the day?**

R: yes they wash their hands before eat, after using the bathroom and before they get inside the house.

**I: and what about the children under two years old, do they also wash their hands throughout the day?**

R: yes they also wash their hands.

**I: how do you wash their hands?**

R: I just wash their hands with soap and also whenever I bathe them, I also used soap to clean their body with the soap.

**I: what is the difference between using water only or water and soap to wash hands?**

R: the bacteria in our hands will remain there when we use only water to wash our hands compared to wash hands using the soap kills the bacteria on our hands.

**I: can you tell me what something that prevents washing hands with soap throughout the day?**

R: sometimes we are lazy to do, there is no soap to wash hands, or sometimes we forget to wash our hands.

**I: Now we would like to talk about your diet during pregnancy and breastfeeding.**

**I: Now I would like you to think back when you were pregnant. Can you describe your diet when you were pregnant compared to when you were not pregnant?**

R: silent

**I: what kind of food you used to eat when you were pregnant?**

R: anything. I ate any kind of food. I never pick on food that I wanted to eat. I eat whatever was there on the table.

**I: and what about the time you weren’t pregnant?**

R: it’s the same thing. I ate any kind of food that was on the table.

**I: what influenced your diet during pregnancy?**

R: Nothing

**I: what food was encouraged to eat during pregnancy and reason why?**

R: I was encouraged to eat fruits and vegetable, fish, and drink juice and I was also encouraged to drink a lot of water.

**I: and what foods was encouraged not to eat during pregnancy and reasons why?**

R: food was encouraged not to eat during pregnancy were cool-aid, drink soda, eat salty food, and eat chips.

**I: and reasons why?**

R: because these kind of foods can affect the health of the child.

**I: and reasons why they encouraged to eat healthy food?**

R: so that the child can grow well and healthy

**I: who encouraged or discouraged you to eat those foods during pregnancy?**

R: the father of my children, the doctor and my parents.

**I: why did they encouraged you not to eat these kind of foods?**

R: so that the child can grow well and can avoid sickness

**I: who primarily cared or supported you during pregnancy?**

R: during pregnant, my mother cared for me and also my husband

**I: so you mentioned that your mother and your husband cared for you, how did they cared or supported you?**

R: they supported me for foods and gave word of advice on what’s good and what’s bad for the health of both me and the child I carried in my womb.

**I: can you tell me on how each person supported you during pregnancy?**

R: silent

**I: cannot be just your parents or people in this house, it also can be anybody.**

R: they also gave word of advice telling me not to eat foods that are not good for my health.

**I: anything else?**

R: nothing

**I: ok now can you tell me about any supplements you took during pregnancy?**

R: just the medicines for blood and vitamin

**I: just these two**

R: yes

**I: did you take any supplements given to you and reason why?**

R: yes

**I: why was it really important for you to take these supplements?**

R: to support me with blood and help the child’s health.

**I: did you drink alcohol, smoke or used drugs during pregnant?**

R: yes

**I: can you please explain more on what drugs you took during pregnancy?**

R: I used to have headache and the reason why I used drug like drinking beer is to help relief from the headache.

R: this was the only thing that I wanted to do was to drink beer during pregnant, and I never understand when I drank beer, pimples would show in my face.

**I: ok. How long did you drink beer during pregnancy?**

R: until I gave birth

**I: were there any traditional medicines taken during pregnancy and reasons why?**

R: yes I used to bathe with traditional medicine

**I: can you explain more about how they did the medicine for you?**

R: they pound leafs together and that’s make the Marshallese medicine. Then they put leafs in the water and tell me to wash myself in the bath tub together with the medicine and that would be in the morning, noon, and afternoon.

**I: can you tell me more why did you encouraged to bathe with traditional in the bath tub?**

R: so that when I get to give birth, it wouldn’t be hard on me to deliver the baby.

**I: who made the traditional medicine for you?**

R: my mother

**I: If you were advised to eat more fruits and vegetables during pregnancy, could you describe what would make this difficult?**

R: silent

**I: what would make it difficult for you to eat fruits and vegetables during pregnancy?**

R: long pause... silent

**I: for example, the doctor tells you to eat fruits and vegetables, what would make easy or difficult for you to eat fruits and vegetable during pregnancy?**

R: there is no difficult in eating fruits and vegetables the thing is there is sometimes I don’t have enough money to buy fruits and vegetables. It is also fruits and vegetables are too expensive and it’s not common for local people to plant their own fruits and vegetables at homes.

**I: what would make it easier to eat more fruits and vegetables?**

R: fruits and vegetables should be prices in the lowest price so that it could be easy for pregnant women to get them anytime they want to eat. Also have our own plants to plant them and the hospital should also provide for us.

**I: OK that’s great. Now can you describe your diet when you were breastfeeding?**

R: during pregnancy, I would rather eat fish

**I: is there is anything else instead of fish?**

R: it can also be can meat like mackerel or tuna

**I: what influenced your diet during breastfeeding?**

R: so that I can produce enough breastmilk for the baby.

**I: what kind of foods you were encouraged to eat during breastfeeding and reasons why?**

R: I was encouraged to eat fish and foods like fruits during breastfeeding

**I: what kind of foods you were encouraged not to eat during breastfeeding?**

R: they told me not to eat tobacco. Cool aid, salt and greasy foods

**I: who encouraged or discouraged eating those foods while breastfeeding?**

R: the doctors and my mother.

**I: can you tell me why did they encouraged you not to eat these kind of foods?**

R: because these foods are not good for the child’s health.

**I: can you tell me why they encouraged you to eat the foods?**

R: so that the child can grow big with a good health.

**I: ok. That’s great. Now can you tell me, after giving birth, could you describe breastfeeding you child throughout the day?**

R: breastfeeding?

**I: how did you breastfeed the child throughout the day?**

R: I hug my baby and feed him

**I: did you wait for them to wash the baby or you just breastfed him right away?**

R: it took one hour so that I could breastfed him

**I: why did you wait one hour later and then breastfed him?**

R: they wash him, check the his hearings, and bound him

**I: ok. Now did you give other liquids to the baby in the first few days after birth?**

R: I did not. I didn’t give him other liquid, I fed him with my own breast.

**I: so were there any traditional medicine give for the baby?**

R: there was none

**I: were there anything that makes it easy or difficult to breastfeed exclusively to now?**

R: right now he is no longer feed from my breast, he is now using other liquids.

**I: can you tell me since when he did not fed from your breastmilk?**

R: since five month

**I: ok. Can you explain why you don’t give him breastfeed?**

R: because he don’t get enough food from my breastfeed and he rather want to feed from other liquids like the store bough milk. He hates it when I feed him from my own breast.

**I: ok. Were there anything… ok that’s good?**

**I: now can you tell me when you first introduced foods or liquids other than breastmilk at that age?**

R: he start ate when he was six months.

**I: why did you gave him food at that age?**

R: when he stated eat?

**I: why did you wait until he was six month then give him foods or drinks?**

R: oh. Because I followed the certificate card that tells us when the baby is supposed to eat or not. And the doctor also told me to do so. My parents also told me that it is okay to give him goods during six month.

**I: what were others opinions from others that influenced their decisions to introduce foods and liquids at that age, six month? People who give their six month babies real food or liquid rather than breastfeeding, what are their opinion on that?**

R: they also follow the birth certificate card and also world of advised from the doctor.

**I: what were the very first foods and how did you prepared these food for the baby?**

R: the very first food that was given to him was the baby food that I bought it from the stores. I don’t mix it with any other kind of food, I give the baby food itself and feed him.

**I: how did you prepared the food for the child?**

R: I washed my hand before I feed him, wash his dishes, and then I would feed him after.

**I: ok. That’s great. We are trying to understand how people eat in this community. Could you describe in detail what are your family usually eat and drinks throughout the day? Let me make this question easier. Can you discuss in detail what kind of foods your family eat and drinks throughout the day?**

R: this family usually eat rice, and drink water.

**I: what kind of meat you eat throughout the day?**

R: we usually eat can meat like mackerel.

**I: what about processed food, what kind of food you usually eat?**

R: chicken, turkey tail, fish hot dog, and salt fish

**I: can you describe the process of how meals are made? As of the chicken, how chicken are made or can meat food, how do you made the foods?**

R: I cut the chicken into many pieces and then add cabbage with it, onion, and garlic.

**I: and what about the can meat, how do you made the meals?**

R: I fried the can meat with onion or I can do gravy so that it can be enough for the family to share.

**I: can you tell me in detail how do you make the gravy?**

R: I need the mix vegetable, onion, soy sauce and a little bit of flour to make the gravy

**I: can you tell me who in the family is served first, next and last?**

R: I usually served the children and then after the adult.

**I: are there any differences in the foods served to different family members?**

R: silent

**I: is there is any differences in food you give to them, like your parents or your children?**

R: sometimes I would make different food for my father. I would bring him potato and cook them for him.

**I: you mentioned you give different food for your father, can you explain why you give him separate or different food?**

R: because he is having a sickness and I prepare separate and good food that can help control his sickness

**I: what kind of sickness does he have?**

R: high blood pressure and diabetes.

**I: are there any differences in quantities of food served to different family members?**

R: what is that mean?

**I: do you give more foods to others than the others?**

R: the adult are served more foods than the children

**I: can you tell me if some children receive more food than others?**

R: yes. Some children served more because they eat more than the others.

**I: oh ok.**

**I: Now could you describe any food sharing between family members during mealtimes (for example children eating together separately from the family, meals eaten from the same plate by all family members?**

R: in this house, I usually give different family members a separate plate. Each children have different than the other, each adult also have different plate to eat, except for me and my husband we are sharing the same plate.

**I: ohh. Okay. Do you usually share food between households? For example sharing your foods with your neighbours?**

R: yes I usually share food to my neighbour because he is my dad’s uncle.

**I: ok. We have heard from some families that eat local foods whereas others eat processed foods. Could you explain what is typical for your family?**

R: we typically eat imported foods

**I: ok. So you mentioned imported foods, can you explain what kind of imported foods?**

R: rice, chicken and can meat.

**I: what makes it difficult for you to cook local foods?**

R: what makes it difficult because we don’t plant these local foods and we don’t have local foods like pandanus, breadfruits, and we don’t also plant our own papaya.

**I: What makes it easy to cook local foods?**

R: cook the local foods?

**I: hmm. Yes.**

R: cook the pandanus on the fire. It’s an easy thing to do.

**I: so why does is easy. What makes it easy for you to cook local foods?**

R: it is really easy because we can just bring the pandanus and cook it on the fire and also the breadfruits, we can just boil it. Bring the fish and then just boil it or cook it on the fire.

**I: how do you bring the fish? Do you buy it from the store or where do you bring it from?**

R: sometimes we buy it from the store or sometimes my dad and my husband can go spare fishing.

**I: that’s great. And now can you tell the positive things about local foods?**

R: local foods are good because they give us good health. And local foods are just our own foods.

**I: when you look around, do you see any local foods or not?**

R: I can see many local foods around me but it hard to just go straight and get them because I wasn’t the one to plant them.

**I: you mentioned that it’s too hard to get the local food right away whenever we want to because we did not plant them, you can say that as the negative things about local foods. Are there any negative things about local foods?**

R: one negative thing about local food is when we want to get them from the stores, they’re too expensive.

**I: what are the positive or negative things about eating processed foods?**

R: processed foods caused a lot of sickness and unhealthy body.

**I: anything else except for sickness causes.**

R: they are too expensive because they are processed foods

**I: and what are the positive things about processed foods?**

R: the good things about them is that we can just buy them and eat them right away.

**I: anything else about the good things about processed foods?**

R: some foods are cheap and that’s why it can be easy to get them

**I: Now that we’ve talked about how the family eats, I would like to learn more about how your child eats. Could you describe in detail what your son/daughter under two years commonly eat throughout the day? For example, what food that your son common eat throughout a day?**

R: my son usually eat can meat, like tuna, sausage and mackerel with rice.

**I: ok. How many times a day your child eat include snacks?**

R: include snacks, he usually eat in the morning, lunch and dinner and also for snacks that means five.

**I: how do you know that your child has had enough to eat? Can you tell me a story about times when you feed him and you know that he has had enough foods?**

R: I feed him and the time I know he is full, he would throw out the foods from him mouth.

**I: what do you to encourage the child to eat?**

R: silent

**I: what would you do when the child doesn’t want to eat?**

R: when he refuse to eat, sometimes I would give him the baby bottle or different liquids to feed him. I would also bring chips so that he can eat.

**I: what would you do to encourage the child to eat if the child refuses to eat?**

R: I usually forced him to eat.

**I: and what if he really refuses to eat?**

R: look for the best way and the best food he would like to eat or drink.

**I: don’t cry baby... Are there any differences when you feed the child differently when the child is sick? For example when the child has diarrhea are there any differences when feeding him?**

R: there is no different but the thing is that, it is really important to let them drink so that their body would not end dehydrated.

**I: so you said give him drink, what kind of drink you give him?**

R: water or juice.

**I: You’ve told me what your child under two usually eats. Now could you explain to me the process, from start to finish, how you prepare and cook for your child?**

R: silent

**I: how do you prepare the food for your child from start to finish?**

R: first, I have to wash my hands. Then, wash the dishes before they use them.

**I: ok. Now what are the process in preparing the food for the baby?**

R: I cook the rice first, then buy a can meat and warm it before give it to the child

**I: when you warm the can meat, are there anything else that you add with the can meat?**

R: I usually add mix vegetable and warm it so that there should be vitamin contain in the food

**I: yes that’s really important.**

**I: could you now tell me what you think are important foods for the children under two years to grow well/be healthy?**

R: he can eat fish, banana, orange, breadfruits, pandanus, papaya and apple.

**I: what kind of foods that should not be given to children under two years old?**

R: lolly pop, chocolate, and chips

**I: and what about water, what kind of drinks that shouldn’t be given for the child?**

R: drinks like soda, tea, plus and …

**I: ok. What is the biggest influence on feeding your child?**

R: influence on?

**I: influence on feeding your child?**

R: make the child grow healthy

**I: can you describe any differences between how you feed your male children and how you feed your female children under two years old? If you had a girl and a boy under two, were there be any differences in feeding the boy than the girl?**

R: there shouldn’t be any different. I would feed them the same

**I: hmmm. Now you mentioned it could be the same, can you explain more what could be the same?**

R: same food

**I: same food?**

R: yes.

**I: ok**

**I: We are also interested in the roles and responsibilities different family members play in raising children. Could you describe the care of children throughout the day in your community?**

R: don’t allow them to play in the main road, or side of the road, dump side, lagoon side or play in dirt places.

**I: who is mainly responsible for the child care?**

R: mothers

**I: ok. Now can you explain more why mothers are the main responsible for the child care?**

R: silent

**I: example. If you were the main responsible for the child care, why?**

R: because he is my child and I should take care of him and raise him

**I: you mentioned take care of them, take care of them from what?**

R: from going to dangerous places, like the mud, lagoon and ocean side and from eating dirt or trashes. And also let them away from the trashes so that these trashes can cause them sick.

**I: ok. Now moving on to the next question, it says, what are the responsibilities for mothers in child care?**

R: mother’s responsibilities for a child is to take care of them, raise them, feed them, bathe them, and hug them.

**I: ok. Now what are the responsibilities of fathers in child care?**

R: they bring them foods, and also help take care of the child.

**I: are there any times of difficulty for fathers to take care of the child?**

R: Yes. Sometimes it might be hard because the he have to go to work, or can be get drunk.

**I: that time when he is drunk, what are the troublesome for you and the child?**

R: sometimes they don’t buy foods, they don’t help babysitting the baby because they are drunk.

**I: ok, now how caregivers play with children under two?**

R: sing to them, disco dance with them or play with them

**I: could you talk about the role of grandparents have in raising children in this community?**

R: they also help in raisin the child, if we are too busy, they can help us watch over the baby when they are also sick they also take care of them.

**I: for example, what are their responsibilities for you and the child?**

R: they buy foods, and watch over the child, teach them the cultural way, and also sing to them.

**I: How do grandparents support in raising children, support mothers and families?**

R: advised them, and help them raise the child

**I: what makes good grandparents?**

R: they buy food, hep raise the child

**I: anything else?**

R: babysitting the child

**I: Could you talk about the role that other family members have in raising in this community?**

R: they raise them and teach them cultural way.

**I: anything else except from raise them and teach them?**

R: give word of advised

**I: how do siblings help raise young children?**

R: they play with them and raise them or feed them.

**I: You are doing a great job. We are almost finished. Now for the last section, we would like to learn about ways we can develop health programs in your community? Could you explain where you usually get rusted information about nutrition and health?**

R: from the health canter and the radio station.

**I: what are some reasons that make you trust where these sources come from?**

R: Because they come from the health place

**I: where would nutrition and health messages should be delivered so that you would see or hear them most easily?**

R: Marshall Islands radio station (b7av) schools, churches, and the stores.

**I: what types of media that you uses the most to communicate?**

R: cell phones and text messages

**I: anything else?**

R: social media like Facebook and especially the radio station.

**I: when you think about your own parenting behaviours, can you explain what influences how you raise your children? Are there any differences of raising child from that woman in that house raising her own child?**

R: yes there is different in raising child.

**I: can you explain the differences that you see in raising child**

R: some parents spoil their children

**I: what you mean by spoiling them**

R: they don’t let them walk or move around, they don’t teach them.

**I: what are people’s in the community opinion influence how you raise your child?**

R: They give word or advice and help me raise the child.
**I: how do they help you raise the child?**

R: when I am not there, they can take care of the child, so that the child cannot go to the ocean, or eat trashes from the dirt.

**I: were there any information related to parenting you received?**

R: yes

**I: like what?**

R: related to parenting is about foods they supposed to eat, watch over them carefully and don’t let them get sick.

**I: where or who the advice or information came from?**

R: from my mother and my husband and my doctor

**I: Are there any desired information on parenting you wishes you had but you don’t have available?**

R: there is none

**I: is there anything else about the topics we talked about today that we missed or that you would like to tell us about?**

R: there is none. Everything is fine

**I: That was great, we are done now. Thank you once again for your generous time and for sharing your thoughts with us. We greatly appreciate your help and we hope this research will help us improve the health of mothers and children in this community. Thank you very much.**
