# Supplementary material for: Conceptualizing multi-level determinants of infant and young child nutrition in the Republic of Marshall Islands–a socio-ecological perspective
Source: PLOS Glob Public Health. 2022 Dec 19;2(12):e0001343. doi: 10.1371/journal.pgph.0001343 (PMC10022247; doi:10.1371/journal.pgph.0001343)
Supplement: S1 Data — (ZIP) [file pgph.0001343.s001.zip › RMI Supp Data/Interviews data/I32U_IDI_SLHS_Rita_Aug 22_Maryam.docx]

**Interview Code: 132U**

**Interview type and Interview: IDI SLHS**

**Interview Date: Aug 22**

**Location: Rita**

**Interviewer: Maryam**

**Transcriber: Shante**

**I: So, we can just start… let me know about the challenges… yeah.**

R: I think public awareness has… The ministry of health has done a lot of public awareness. I wouldn’t say its weak, its just that their message, its not reaching the targeted audience. Its reaching the wrong audience. Ahh through… I’m not sure if they were doing any awareness through social media…

**I: Oh ok.**

R: That would be ahh strong flat form.

**I: Right**

R: To do some public awareness materials. Right now, I only know about their public awareness materials advertise publish in the local news papers.

**I: hmm ok.**

R: Not that many people read news papers, but I would say many people here are using Facebook.

**I: Oh yeah?**

R: And it would be a nice flat form to raise this profile of this issue and… Going back to a Fort ability, we import more then we produce. Most of the vegetables you see in the stores on the shelves are mainly imports.

**I: They’re so expensive… yeah…**

R: they’re so expensive. People tent to buy those unhealthy foods because they’re a lot cheaper then the imported vegetables. I guess another problem here is we lack. I mean the people here on islands needs, I’m sure if we have Nutritionist, maybe we could have more then what we truly have in the moment. I’m not sure how many Nutritionist are there in the ministry of health, ahh or maybe there are not enough to accommodate the entire nation. In terms of… let’s say land uses. You don’t actually see any farms here on the RMI. But plenty of empty space that are actually good for farming. I’m not sure why we are not taking advantage of it… I would if I would have Own a land over here, but I don’t. I only own lands out there on the outer islands. But really its all about the market and excess to those. Ahh… coming back to the thing that you mentioned… what’s it called? The Nutrition Survey?

**I: Oh ahh its called the Integrated child health and Nutrition Survey.**

R: Is this survey like available online? Is there something we can like share?

**I: Um… yeah I think you can share… yeah.**

R: Yeah. Because I don’t recall taking this survey, but I would like to…

**I: Yeah yeah. Absolutely. I know different Ministry should be aware of all Nutrition related activity site that go on… yeah**.

R: We here in the Ministry do have… This ministry focuses mainly on policy development and we do have what’s called the full security Policy… I’m not currently sure where we at the moment, in terms of implementing the activities… I’m actually new to this Position. But one of them is, I mean the objective of the, I mean the government objective to… as well as other countries is to provide access to food and all… and all the entire country.

**I: Right.**

R: And that’s the eligible of the food security policy. And there were several activities listed in the action planning, along with the development planning that we work with, as well as the local partners like the ministry of health, Ministry of eternal affairs. As of… I’m not sure if I answered all of your questions. I can’t speak on the parent to the size because I don’t have kids. I’m single.

**I: [Giggles] Ok.**

R: My job is my Wife actually. I spend too much time at work then at my home.

**I: Yeah… Kind of go back too… you know you were saying… Let’s talk about social media first for a second. So, you mentioned that ministry of health is not messaging getting to the target population.**

R: I think so because I only see they’re materials in the local news papers. But if they could’ve maybe… well maybe they’re using social media but I’m not so sure, but there was ahh. Public service commission of a new policy where we cannot use facebook at work place. And I just don’t know why we are not allowed to do that. We at the trade division promote local products through social media because its such a horrible flat form. But now the PSC wants to get rid of that. So that would be an issue if the ministry of health there try to implement.

**I: So, there’s a bit of the police to allow for that….**

R: but you know the PSC they’re quite flexible, we can talk with them and they can recommend the new policy. But those social media… you know if you… I know your using Facebook… well I’m not on Facebook. I’m only on google flat form that nobody uses. (Both laughing). Well yeah, every minute there’s a new update and… but many people here, I would say about more then half the population.

**I: Oh ok. What about the causes associated using Facebook, you know using phones and internet, so what would you think it would capture the most vulnerable population or what ways…**

R: oh that I have never thought of that… believe it or not but many people here are, you know… using many smart phones. And I kept wondering how, can they afford those smart phones since they are not working, they have rich parents or… But access to Facebook is… You know I don’t think it’s an issue here as long as they have a smart phone and have access to a free Wifi Hotspot and it shouldn’t be…

**I: Are there a lot of free wifi hotspot here on Majuro?**

R: Not that many. We have it at the airport and at the resorts. I would say we have about to 7 to 8 free wifi hotspots. But what people would do is find a wifi signal and if they find it they will ask people around the place to ask them for the password. That’s why everyone is connected to every.

**I: That makes sense [giggles] And ok. And messaging like, suggesting the Ministry oh health could create like a page…**

R: Not like a website but through Facebook.

**I: Yeah… Ok.**

R: Because there are many users there.

**I: Yeah… Ok. What are other ways you could get communication channel of getting into… kind of the most vulnerable population?**

R: You know just doing some community consultation, you know go to each district around here on Majuro and preach…

**I: like in person…**

R: Or fairs like the… You know, to get them and the community involved… like what we do on Saturdays we usually do what’s call the Laura Farmers Markets. So they do it at CMI, they sell produce and do some demonstration on how to cook soap using the locally ground produced food. And some ‘’other Saturday we target like the grass food level people who are like ‘’ Oh we can’t afford to buy local products, so we sell them with very cheap price with no profits.

**I: Ok. Where is the [tent]?**

R: We call it the (unadre peium) farmers market. It’s like we mobilized it in every district here. Last one was near Coop. school, we targeted the community near coop. all of the product are grown in the farm at Laura but the Local farmers at Laura are the one that most people Attends. The farmers at Laura farmers markets the one that sells the produce, so their prices are a bit higher from what we see and do the Mobil farmers markets.

**I: Right… Ok. And so far the Mobil pharmacy market. What’s your reach? Like you said you go to different comm…**

R: We go to different communities targeting the grass roots and low incomes.

**I: How long has this been going on for**?

R: since last year.

**I: You find there’s a demand for it? When the prices are low are the people buying it?...**

R: you know, I think because we just started last year and it’s not really… not that many people, low income people come and buy them. It’s probably because of the variety like the lemon grass and you know, not the poplar, not like chines cabbage and egg plants. Now that’s the biggest farmers market. [giggles].

**I: So, now at low income market, you said lemon grass and what else?**

R: Some pumpkins.

**I: Oh ok. So other produces like cabbage and?**

R: Cabbage, egg plants and the white reddish.

**I: Those are sold at the local…**

R: And the bigger, well… [giggles]…

**I: laughs. Yeah ok. What are your plans with low income markets?**

R: I guess the plan is to increase the numbers of verities of the (?), suppose to be sell because right now farmers from Laura comes to the farm to collect and take the vegetables that are grown and take It to their farm or to their families. But the plan is to, well because of limited, we don’t have that money on stock… So that’s why we do it like every 2 to 3 weeks.

**I: Yes. Yes.**

R: Imagine if we could do it like everyday, but we will run out of probs.

**I: Yeah, so sorry. I don’t know anything about your farm. Could you explain a little bit about it?**

R: Laura farms is currently operated by the government of Taiwan, it’s funded by Taiwan but through this Ministry though. They do have not only produce but also a bakery over there. There is a program that we do with people through out the RMI and for households to receive 2 piglets and any veracity of and seedlings, it’s just that we can offer them…

**I: Oh. Ok.**

R: Its just the communication channel, the local government that issues that we’re facing right now with them because they’re suppose to come and collect them and deliver it to their Constituents, but I don’t know… we will have to work on them to improve the movement of the transport of the seedlings and the piglets.

**I: So, right now the piglets are?**

R: In laura, it’s quit far but we did provide transport, all they have to do, Is come here and sign up we’ll just bring it, but then when we bring them there is no one who comes and claim them.

**I: Oh ok. The funding for the piglets and the seedlings?**

R: Through the Taiwan’s governments

**I: Ok. And now the ministry of health?**

R: this ministry? Oh no. the Natural Resources.

**I: Ok So, your coordinating?**

R: Coordinating with the… I’m like the man between local government and the ROC government.

**I: So, the local government consists of?**

R: 24 local government all together.

**I: Oh ok. So, all across Majuro?**

R: All across RMI.

**I: Ok. When did this program started?**

R: I would like to say around 10 years ago but I have to confirm it first. But it started many years ago.

**I: What’s holding the local government?**

R: I think because they’re busy dealing with other stuffs. I guess again we need to improve Communication channel with them or encourage them to come here and collect the stuffs that they requested, but I really don’t know why they don’t come and claim the piglets.

**I: Are the piglets here or…?**

R: They’re at Laura

**I: So, the local government, you said they requested the piglets, so this is the program?**

R: The local government we do work with them, some just difficult. And all together there are 24 of them.

**I: Ok. Where in Majuro would the people be keeping the piglets?**

R: You know pass the airport there’s an area there and you’ll see them when passing by, but not as big as the ones in Laura. I’m not sure about the people here in town because the EPA, they have strict regulation on setting up piggeries here in town. But I just don’t know where they are. They are probably hiding from the EPA, from the regulations. [Laughs].

**I: My understanding is that once you have pigs in town you have to have a pin that’s connected to…**

R: Yeah, the regulation. EBA are really strict. But I’m sure there are piggeries here in town.

**I: So. I’m just figuring out the logistics of the… if we purpose to have the piglets on where would it be kept and how would that be maintain, such as places like Rita and it’s very congested.**

R: The President started this Program called 1 Island 1 product 2 years ago. The mail goal of this program was to encourage the people in the outer islands to think like an Investor, Like a businessman with their handicrafts. And he helped them look for a market as well as to encourage the farmers in the outer islands to use up their unused lands to grow more vegetables and fruits, like for an example Ebon (an atoll) It’s quit far but it’s near Jaluit, and Jaluit has a high school there and that would probably help the people in Jaluit the cost of shipping because it’s quit far from here so maybe if all these atolls help each other… I think there’s 4 of them. There’s kili, Jaltuit, Namdrik and Ebon. (all 4 are atolls). They are right next to each other and If they can work together and buy a fusel to transport their goods within that cluster it would help them out. And in terms of trading, like trade their goods with each other.

**I: (Laughs while talking) The piglet story is very Interesting. The piglets are still there waiting for a home? Is it on going?**

R: Yeah. It’s on going.

**I: And your trying to get?**

R: The local governments to come here and… well not come here first but to request what they need and how many piglets they need, and then we bring them here and someone suppose to pick them up. Some comes and pick them up and some don’t.

**I: Was the piglet program suppose to help with food security and some household levels?**

R: hmm.

**I: Ok. Interesting. And the Laura farm, So, basically the farm Taiwan’s funds it, farmers in Laura use that land**

R: Not use the land but they go and take seedlings and even piggeries. There was a training center over there. There are some experts over at the farm and they provide training to the people, but mostly people in Laura use it.

**I: And the Taiwan Mission. Who do they work with?**

R: They work with our ministry. This Ministry.

**I: Do you know if they have any contacts with the Ministry of health or?**

R: I believe they do have contacts because there’s some Taiwan’s volunteers also working with the Ministry of health, They are in the same group called the ICDF (International Cooperation Development Fund). That’s the program that funds the Taiwan funds. And I believe there are also volunteers ICDF’s working with the ministry of health.

**I: Oh ok. Do you know if there’s any school filling program from the…?**

R: I think there is but you’ll need to confirm that with the Ministry of education.

**I: Ok. I kind of want to go back to food security. So, you mentioned that affordability is the main barrier for people accessing fresh fruits…**

R: Not only fresh fruits but also imports. I’m not sure if you have visited EPPSO and their prices, but there is this division within the EPPSO called the Prices Monitoring Board. Like for a bag of rice here on Majuro cost $10 and there on the outer islands cost $20. I just don’t know how they come up with $20 dollars. The prices there on the outer islands are quite expensive.

**I: So, in the outer Islands are they relying on the foods they grow or…?**

R: Umm. I would like to say that, but I don’t really know… I’m not always at the outer islands. I was at Likiep and Arno (atolls). But this was many years ago. Wotje and Jaluit (Atolls). These atolls I wouldn’t really say they are outer islands because they are dev like Urban… Compare to the outer islands, usually is the northern islands that are usually affected, when there is a draught. Over come here manually, the southern islands are not usually affected by the draught because we get more rain then them, because of the trade winds, so the future you move up more trade winds and more trade winds we no rains, less rain not more rain… so, the northern now is windy then the south.

**I: Right. Ok. And so, in terms of the food security there your, not sure of how their growing food or buying imported or…?**

R: I would say in the south they grow some vegetables and fruits but on the southern they rely too much on their family members and their leaders like the Mayors and the Senators. You know over the year the kids you know becoming a politician here, you need to have a lot of money because the people will be contacting you and say ‘Hey can you give me some money to buy stuffs and’…

**I: Yeah… right. OK. We accomplished so many different things right now 😊. So, in terms of foods…We’re interested in umm… because you mentioned that there’s lots of space in Majuro but there not planting anything or? Do you know why that is or what community improves they’re?**

R: I guess one of the obstacles is the land owners demanding more payments especially here on Majuro. Outer islands I don’t think it should be a problem on leasing lands and growing foods… out here in the outer Islands I guess they have no choice. But over here because it’s the Capital and the lands Owners here they’re demanding a lot of cash. So that’s one Obstacle. Another one… I can’t think of another one… but I guess Majuro is reserved mainly for to develop like for building and house holds programs. But it all falls back to the land owners demanding lease payments and all that…

**I: Right. Right. Ok. Umm. What about umm. Do you know anything about a home garden some people who grow foods umm in their own kind of backyard…?**

R: I think yeah… we should have that information. Like our Ministry should go door to door to interview and do some surveys. I really don’t know, maybe we do have them, but I don’t have their numbers with me, but I’ll ask them do home gardening. I do know that the President is doing that. Yeah. Several people but I can’t give you the concrete number. But all I can say is all they do, there are some home gardening.

**I: So, the soil here? What can it be grown here?**

R: ehehe. I’m not an expert (both laughing while talking) but I’m not sure because there are Coconut trees and Pandanus and those are the common trees here. Um in terms of vegetables I guess you will need some sort of like special top soil fertilizer compose, but in terms of the soil we currently have I guess several invasive species (?), but definitely Coconut Pandanus trees that we have. I’ll note your question down. (both laughing)

**I: It was interesting knowing what you’re (both laughing, and I can’t understand what they are saying.) So, what about in Laura, um the farm there they’re growing different crimes of crops, they’re growing cabbage and egg plants and cucumber as well…?**

R: Yeah. I think they have cucumbers.

**I: Um yeah. So how do you know…**

R: Yeah. Well you know Laura is big compare to uhh I’ll say here like you can see like the lagoon and the ocean side but in Laura you cannot see because the land is very good and also you know with the water vents in Laura I think it’s also helping the plants grow and also making it a lot easier for other crops to grow in Laura. Where it’s quite difficult for smaller area’s like here and…

**I: And the production in Laura, So it’s that just enough for people in Laura and the Saturday like ever other Saturday’s markets…**

R: I would say so um the production in Laura can… it will not meet the total demand, but for a day yeah. They can meet the, like the daily demand the currently what they sell can… Because you know when they sell not to many people buy them.

**I: hmm…**

R: When the market is over they go to the restaurant and sell them.

**I: Oh ok.**

R: But when the… the chines restaurant are the ones, they’re actually the market. They’re the one buying the produce from the farm in Laura.

**I: So, the demands from people is low?**

R: hmm.

**I: OK. So, you mentioned that’s kind of more related to the portability?**

R: Yeah. The portability.

**I: Ok.**

R: But home gardening…. That is something that we really need to strengthen… work on and insure, well not insure but try to make some people more interested in home gardening.

**I: Right. Do you think it’s a possibility to some how lower prices for produce growth here so that many people could have more access to it?**

R: the locally growth produce. Well I mean, I guess us farmers we did provide, the government has already given them intensive, I mean the government are not taxing them and they’re not paying any GR tax and uhh resonated tax and sale tax… not like the regular business here where they use Social security and all these taxes. I just don’t understand where and how they come up with the prices and I really thought that was a goal of the price monitoring board to set their price and… but they’re not actually not setting the price, they only selling the price of the goods in outer islands but not here on Majuro.

**I: Yeah… ok.**

R: But yeah, the price monitoring board should look into setting up price throughout the country.

**I: um. Yeah… so, I was just wondering if in the outer islands… are there more lands available to grow food there?**

R: I would say so. Because you know people in the outer islands are actually moving to Majuro moving to Ebeye, so there are plenty of lands out there. You know there are many homes there that no one lives in because they’ve all moved. And people here are moving to the United States to get a job and…

**I: Yeah. I’m interested if you know that if there’s more land over there to grow food. Could there be some kind of… you know between Islands and trading…?**

R: What the Ministry trying to do starting next physically years to employ more extension accents and to dis patch them to the outer islands so that there is like a specialist in the area agriculture, help the community, And maybe setup a small nursery maybe about 3 or 4.

**I: Ok.**

R: And then maybe there will be our contact, you know there’s going to be a lot of contributing… we’re going to start working with the local government because they are unreliable but we will sent our own people there.

**I: Yeah. Right ok. So, these are new plans? Kind of? And with that insecurity policy is that? Ok implementing policy…?**

R: Yeah. It’s an implementing policy…

**I: Ok um yeah. So, hopefully I will get my hands on food security policy. Umm. In terms of the idea of home gardens, how would you? Do you think people would be resected to that?**

R: I don’t see why not… I mean it’s a… they… you know it could be a good evening activity and morning activity rather then you know finish working or going to a bar and going to… (laughs while talking) because there not too many activities going around here after work and after school. There used to be a movie theater it’s close down because not too many people go there but I don’t see why people would not like this home gardening. We do have these seedlings in Laura that we can distribute to every households, maybe not piggeries but um home gardening is good, I just don’t see why not, I think home gardening people should be receipted because… I’m not into home gardening because I don’t have a big land spot or backyard or, maybe a small pot could do. I have a small backyard so I don’t do home gardening. [giggling]

**I: yeah…**

R: [laughs while taking] I guess it depends on your backyard if you but it that way.

**I: [laughing along] Yeah… what about in terms of ahh peoples umm reception, you know eating healthy and the importance of eating healthy uumm…**

R: I guess when you have too uhh change that person’s mentality or attitude because you know spinach, When I first taste it spinach, I was just a young kid. The reason why I ate spinach is because of pop bai [both laughing] I wanted to be strong like popey … so you know when we could do something like that to change that persons attitude I think it might work. {laughs)

**I: Yeah that was a great toy… yeah… so if you wanted to change children’s… we could create cartoons for adults to…**

R: Unhealthy food they’re delicious but for the healthy foods they’re not.

**I: right**

R: but you need to look for like a role model somewhere like popey… (both laughing)

**I: Yeah. Um ok. I Just remember the cooking demonstration is at the market, the Saturday market?**

R: they did it last uhh past Saturday so maybe another… They usually do it on the first Saturday of a month and then the third Saturday.

**I: Would it be this Saturday?**

R: No. it would be on the other Saturday.

**I: Next Saturday ok.**

R: Because next Saturday would be September 1st. so, that’s a new one.

**I: Ohh. Ok. And do you know who does the cooking demonstration?**

R: Cooking demonstration umm…? I think there are several people who does… the nutritionist from… I think… I think the ministry of health are a part of…also involve in the… through their volunteers Taiwan’s volunteers.

**I: ohh. Through the Taiwan’s volunteer…**

R: Yeah. And also the fish market…the fish that they sell they do some uhh cooking demo.

**I: Ok. Where’s the fish market?**

R: you know where allele building is?

**I: hmm. Noo.**

R: ahh ok do you know where uliga dock is like over on that side?

**I: Yeah yeah. Like over at Raimers?**

R: Yeah that’s shoreline.

**I: Oh ok.**

R: ok. But before you go to shoreline, Ok it should be across the church…

**I: Oh ok. I got cha…**

R: ok well the fish market is there.

**I: So, in terms of you know getting people too be interested in home gardening and to change their perception maybe have a champion like popey. And the taste of food you said… (both laughing) let see…ok…yeah I’m just trying to think of ways to make it easy for families to food at home…not grow food at home but just to have access to have fresh fruits and vegetables because umm that’s seems to be the main issue that access in there because portability and umm, and the portability is driven by the fact that, the supplies is not**…

R: yeah. The supplies is not there I mean if you look at the local res… if you look at the restaurants here, specially the locally local restaurant here… you don’t see like healthy foods, they just like to forget what the wellness center is doing, but not coming up with their own Menu or… but I would say we need more nutritionist. I think there are several with the ministry of health, but I think we will need more than that. Ant the Taiwan government through the IGDF program uhh I think they are willing to provide Nutritionist, they even asked me to sign up for Nutritionist, but we don’t need a Nutritionist. The ministry oh health needs that because it’s under their like man (faders) (I don’t know if I heard the word right, but I think it’s sounded like faders.) All we do is food security and then coordinate with them on the Nutrition Part. We cannot take what Ministry of health suppose to do because you know there could be like a conflict or a war between the 2 Ministries. But I would say we need more Nutritionist. And encourage our students to become Nutritionist it’s a Nutritious field uhh…

**I: yeah… How would you encourage them?**

R: Give them free scholar… yeah, we do have scholarship program for them it’s just that I guess people are just not interested in becoming a Nutrition or I mean the local people here. But you know the tide might change so they’ll you know, want to persuade on that career.

**I: Have you notice, you know on your field of work… level of Nutrition…are people aware of what they should be eating or?**

R: The only time that they’ll be aware is when they are sick, when they’re dying and then that’s when the doctor will be saying ‘ok you need to go on a diet or start eating this and’… Yeah… like me. I’m unhealthy, pretty soon ill have to go see the doctor and he will recommend that I, you know star consuming vegetables and (giggles) But I think, yeah… I guess going back to the attitude… and that’s something that we really need to change.

**I: Yeah. Yeah, right. That’s what we’re trying to figure out like what will resonate with people better like how do you do that? Um…**

R: How do I do that? Um. Well I can guarantee you one thing. If I leave the government then I’ll have time to go see the doctor… I’m not sure I’m just throwing you words.

**I: No. no. that’s ok. So. Um you mentioned more Nutritionist… wait before we get into some kind of coming up solution… um. Have you notice any difference in umm…through… um… fruit ability on what crops could be grown here now compare to let’s say uhhh… like 10 20 30 years ago, has there been a change?**

R: 10 20 years ago? Uuhhh… well… uhh… I don’t know, I’m only guessing here. I think there’s been a lot of changes because before we didn’t have a lot of varieties of vegetables, locally grown vegetables and fruits, now we have many varieties now and… but yeah 30 years that’s a long time… and yeah, I would say there’s been a lot of changes in terms of varieties.

**I: and is that varieties coming from the Taiwan the Laura farm? Or where does that varieties coming from?**

R: From… its really really difficult to import seedlings because the quarantine officials works there and they’re very strict on that, you’ll need some sort of like SPS certificate and… uhh but yeah, It all started in the I would say Laura farm and they stared to distribute seedlings to the other communities, and that’s how I guess they all started. I can’t think of any other Prior project other than the Taiwan ORC farm. But if I were to guess I would say they were the first one to start.

**I: Ok. In terms of uhh growing foods in Majuro, umm What would be the difficulties in growing foods here?**

R: I guess safety… You know, there are many many people here in Majuro and many people live in like I would say poverty and then you know a lot of chef here in Majuro, I mean another Issue you know when your planting when your farming, some people might come and steal your crops and I’ll say that’s an issue that we people farmers need to concentrate more on, not just grow it but If they plan on farming they need to look on… you know because it’s an investment. and you’ll need to hire people not to keep the wealth to themselves but too spread the wealth, then a higher additional people to work with them, insecurity people to look after the farm when the farmer is taking a rest or something so that no one can destroy the property. I would say security is number one uhh… another issue is land owners, uhh don’t be surprise if a land owner demands like a hundred cabbage or… land owners here in Majuro are quite powerful, not like the people in the outer islands because they uhh, I mean there are no investments in the outer islands, no ones is interested in investing in the outer islands because there’s really no market over there. But then suppling from the outer islands is another challenge because the transportation, they only do it like 4 times a year.

**I: Oh. Oh really? From?**

R: from… because the shipping corporation they only have limited boats and again there are 24 islands, so they need to, they can only work on 4 trips per islands.

**I: right. Right. Ok. So, 4 trips per year?**

R: So, places like probably Wojte or Jaluit (atolls) they would get more trips because semai Urban because there’s a park plan over there so fuel needs to get over there too to fill up the tanks farm. I can’t (sorry I couldn’t understand what the word he was saying 47:37) because I’m not an expert in the area…

**I: Yeah. Yeah. Ok umm. So, that um you we’re thinking those we’re for the bigger farming, what about** um home gardens, like if we we’re too recommend home gardens for…

R: What would be the challenge?

**I: Yeah. For people growing**

R: What would be the challenge uhh… that would be an extra cost uhh, thing with the people here is that they don’t like to spend money, they like to keep the money and uuhh… I just don’t… I mean I just don’t understand, to make money you need to spend money. Uhh I guess additional cost to the, it the only issue I could see, but I don’t see why home gardening, why there should be an issue on home gardening unless the land owner comes again and demands… ”Okay I need that pot or” that would be the only issue I could think of.

**I: Right. Right. Umm, and the issue of security you mentioned earlier…?**

R: well is in your land, as long as you have a fence. Have you ever been to Ebeye? (atoll)

**I: uhh not Ebeye. No.**

R: you’ll be surprise if you go to Ebeye. It’s a lot different from here, every house every home they have their own fence. Because uh it’s very very small, like you have maybe every 2 feet, that’s like the space between 2 houses. It’s uhh many many houses in Ebeye. One household you talk about an average of 2 to 3 people… it’s just about guestimate but yeah, a lot of people in one household.

**I: Yeah. Ok. Um. In terms of food security that we talked about price being to high uhh the supply not being enough uhh transportation difficult between outer islands to bring food here… So, are there any other challenges that you see to food security here?**

R: I guess ahh, other then what I mentioned earlier on, uhh because food uhh I’m not sure what’s the exact term of the food they use uhh, food (poisoned?) I think we do have that, you… I think I’m not sure, I forgot the saying but a lot of people throw away foods. And we just… I mean growing up I don’t recall throwing away left over foods. We always eat the smallest bit, we always make sure our plates are clean no waste or anything, it’s just that we were discipline in a very strict way, maybe my childhood wasn’t every that good, but uhh there are a lot of food waste over here, but all those wasted foods, well some people give it to their piggeries to feed their pigs, some go straight to the dump. Umm and I cannot say that maybe those wastage foods are Nutritional foods or, but that’s… I mean how can you say we have a (dinful?) security when we have a lot of…(giggles) that’s not the word uhh, I guess we eat, we take more then we can consume or… but food wastage is something that we see a lot here and I’m not sure if there’s a campaign on that food wastage or maybe there’s a, maybe FAO is doing something about that… but other then what I mentioned earlier, I cant think of anything… especially that I’m not an expert in the area of Agriculture…

**I: One think that we notice when we go to the grocery store sometimes there’s fresh produce area, the fresh produce are rotting, so you know they’ve been left there for a long time, um like the lettuce would be molded away or the carrots would have mold on them, we’ve seen that, so we’ve been wondering if you know why that happens, if it’s ?**

R: why that happens? Uhh, usually when the importers, well let’s say the retailers at K&K and Payless because they are the main 2 competitors groceries sector. They… I guess what they do is they don’t do like a survey or anything before they import the produce, but most of the produce that get rotted first are, I would say the ones that we local people are aware of is the fruit pomegranate, I mean no Marshallese people know what pomegranate is, and they sell it at a very high price. No one buys that only my mother of all people would be what is that? And she couldn’t even pronounce it, and like what? Why buy it if you cannot say it? (laughing) but yeah, most of the foods that get rotted first are the ones that many people don’t understand about what they are. And you know the other ones are like onions and potatoes. Now those ones are like I would say more demanding ones because of corps and carrots as well, no corps in onion but the flavor. What payless or K&K does is umm, before… I guess every produce there they have like an expiration. And once they reach the expiration date they start selling them on a very low price. But yeah, I’ve seen a lot of thrown away, you know thrown away produce that they’ve thrown away, but I guess they should’ve build like a market testing before they could import a lot of, in terms of quantity.

**I: Yeah, super market is kind of known as food wasted everywhere…ok um yeah… So far we’ve been kind of talking about the challenges. So, a question we like to ask is that, in a radical situation, like if you’ll have unlimited resources, you like had all the money you need, all the funding, um what programs or services would you like to see in place?**

R: relating to promoting Nutrition?

**I: uhh yeah. Promoting Nutrition and improving access, people access to have healthy foods and fresh produce, yeah.**

R: Program I would give, provide intensive to the farmers.

**I: intensive? Ok.**

R: to encourage them to… you know to use up their lands and to grow foods and vegetables. Piggeries, we have a lot of pigs here in the RMI and… but then we still import pork and chicken, we import meat. That is one area that I would look into, you know to start consuming or commercially sell our local pork, uhh guess settle like uhh, something like uhh, what’s it called?... (slaughter house?) or something. We can sell local locally grown pork.

**I: is the supply high enough? Would be high enough?**

R: Maybe to certain area, maybe not Majuro but uhh the current supply… yeah, I would’ve say it’s high enough to meet the… we can still, I mean we can import few to meet the local demands. What else in terms of Nutrition uhhh? Oh, the Scholarship program specially in the area of a becoming a Nutritionist. And prop transportation you know within, probably increase from 4 trips per islands to probably minimum 6 trips, not 12 because that’ll too much but… Oh. You said if I have unlimited? Uhh. Realistically I would stick to 6. (both laughing)

**I: Yeah. Yeah. That would be enough time to harvest to grow…**

R: And then um, I don’t want to talk about tax because, um leave it be

**I: (talks while laughing) uhh why not?**

R: because then you were…. Well actually, the tax purpose of a import tax is to protect the local production. Like for the kids for the fishery cycle (?) like for example tuna. We don’t import tuna we only import can food tuna but if we start importing fresh tuna, then we’ll need to probably increase 4 sets of (?) so that we don’t need to import tuna.

**I: yeah. Yeah. Because you have fresh tuna here.**

R: and we have unlimited supply. But for the case of vegetables, uhh we cannot go there yet, we do not have the local supply to meet the demands so, we’ll need to import more too…

**I: Ok. What about importing from other islands? Do you meet the demands?**

R: importing from other islands as in other countries?

**I: no, just the outer islands of Marshall in terms of if they can grow more land there or more food there.**

R: if we can import, I mean for the one that I listed down was for transport, and so that we can bring the produce from the outer islands then sell it here on Majuro, either here or on Ebeye where the demands is and the need is. But provided supply is, doesn’t meet the demand and still would need to import, I guess from people like for Kosrae. I don’t want to say plain. Unlimited resource is very very tempting. (both laughing) and um, and then again beef up the Ministry responsible, well beef up the Ministry responsible variety culture. Um especially in the outer islands like the intension agent program that we’re going to initiate over here. Because you cannot send only 1 person to the outer islands. At The moment we can only afford to send 1. But for places like Jaluit and Aelonlaplap (atoll) where the land is too big you have plenty of communities, 1 person cannot handle them. So, probably expend the program if we have unlimited resource. I think I’m going to stop there, I can’t think of anything else… I was thinking of buying a plane or, but uhh I’m like I’m not going to go there.
(both laughing) Speaking for the needs right now.

**I: Yeah. Those are great solution.**

R: Oh, probably one more thing, peace up, strengthen and public awareness.

**I: Public awareness. Yes. Yes, so, on pubic awareness you mentioned earlier using social media yeah in doing kind of in out reach such as going to the community and stuffs…**

R: You know the Ministry of health is doing a pretty good job, you know the area public awareness, uhh I guess maybe it depends on the resources they have available and maybe they can, they cannot expend the uhh strengthen it anymore but uhh if they can you know look into social media, social media is a very strong flat form everyone is using it and you know right now they’re doing a good job. They can do better.

**I: Yeah. I guess it starts with the Public awareness to increase the demands yeah, make the changes there.**

R: Ok. One more.

**I: Oh yes, absolutely go on.**

R: Ok. Probably like a training center or you know for farmers, not just for farmers but for people just to go to you know the training center and learn how to cook Nutrition food and then uhh some training on a home gardening…

**I: Yeah, absolutely.**

R: Now I’ll stop there… (giggles)

**I: No, it’s ok go on. Yeah. The training center, where would it be the best place for it?**

R: again, unlimited resources… probably Ebeye, here in Majuro, here in town. We already have the one in Laura and then probably if 2 more maybe 1 in Wotje or Jaluit or to semai Urban because Wotje and Jaluit are the only 2 outer Islands that are with High schools. So, other than the high schools here, Majuro and Ebeye they also have 2 other High school. So, probably set up the training there as well in the high schools.

**I: And if you do it in the high school we can target the next generation.**

R: And then uhh, maybe for the kids in Ebon (atoll) they can always fly to Jaluit because it’s near by, they don’t have to come here which is more expensive, they’ll just go to Jaluit go to the training center and do something new. Those are my wish lists.

**I: those are great wish list. So, a lot as come up to a lot of funding… uhh and so, what in terms of policies, what policies do you think should be in place that would facilitate, better food security place here?**

**R: What sort of Policies… like at the regional or national level in between?**

**I: uuhh either.**

R: ok. Let’s take it to the national level, don’t want to go regional. There are… like for the transport sector, I don’t think they have a transport policy and I think they need to set up one to transport policy that could address the uhh movement of the goods between… you know within the country. There is a transport policy but on mainly on sustainable transport but more relevant to greenhouse gas production but noting in terms of uhh, like trading. There is a national development plan they call it the net plan, I’m not sure where we at at the moment in terms of the net plan but I think its house it’s at the chief Secretary office. I’m not sure f you’ve met with Jennifer Debrum?

I: uhh. No.

R: Ok. But the net plan focuses on like disaster risk management. I think this is a existing policy, it’s just that I don’t know where we at, at the moment due to implementation and if food security is related its link to the net plan.

**I: ok.**

R: Going to education

**I: Yeah**

R: education policy… I don’t think there is one but maybe I am not aware of it

**I: yeah**

R: but, where the public awareness uhh should start with the students and, and we should link food security if there is an existing education policy on food security. Ok, now the food security policy sort of captured health so, I’m not going to go there in terms of health policy because I think it’s to specific…there’s a lot of things I want to say right now but my mind is kind of stuck. (both laughing) Yeah. I don’t know. But when it comes uo I’ll let you know. I’m having some kind of brain stuck.

**I: no this has been very great, there’s been a lot of interesting points that you brought up. Yeah. Ok. No this is been great. Um so, the education policy that kinds of relate too…**

R: I guess it’s the curriculum…. Is that what it calls?

**I: yeah yeah. It’s putting like agricultural nutrition programs in schools…**

**R: yeah**

**I: Ok. Perfect. Let’s see… yeah, we’ve covered a lot of topics here**

R: that’s a world record for me. (both laughing)

**I: yeah this is great. So yeah, we talked about challenges in terms of you know lands owner kind of bring Perrier to growing food here, the trade here between islands, transportation there, you know some kinds of policy…**

R: Going back to the land owner think. Don’t go cope me on that, you can cope someone else but don’t cope me on that. (both lauging)

**I: Yeah, don’t worry about it. Nothing will go back to you don’t worry. Yeah. So, these issues um yeah, we talked about those trust funds on how to come up with solution, yeah you came up with a great list. Is there anything that you can think of, on top of your head that we missed in terms of?**

R: In terms of nutrition?

**I: yeah.**

R: in terms of nutrition… you know nutrition it’s not just a issue that involves one state holder cross cutting issue that’s all sectors, food security sector even education like the childhood so, I mean students… effect every sector… I mean it’s not a one man goal. Everyone needs to work together

**I: absolutely**

R: to address those issues. I can’t say much on the stunting part because I have no information whatsoever on that. Maybe there is a report… I haven’t seen that report yet. I guess I have nothing else to add because we pretty much talked about it maybe, is it been a hour now?

**I: yeah.**

R: but uhh, yeah. I mean I’ll like to do the survey if it’s available

**I: Um. Yeah. Just contact Helia or maybe caroline**

R: Let me give you my card, actually old card printed 3 years ago it’s kind of out dated the cards because the Ministry it’s now called the, the ministry is not, ministry of natural comers so, that’s the only difference as well as my title. I’m no longer the chief of trade I’m the deputy secretary right now.

**I: oh ok. but you know it’s the same but that’s all it matters. (laughing) perfect. Well thank you so much I really appreciate it**.
