# Supplementary material for: Conceptualizing multi-level determinants of infant and young child nutrition in the Republic of Marshall Islands–a socio-ecological perspective
Source: PLOS Glob Public Health. 2022 Dec 19;2(12):e0001343. doi: 10.1371/journal.pgph.0001343 (PMC10022247; doi:10.1371/journal.pgph.0001343)
Supplement: S1 Data — (ZIP) [file pgph.0001343.s001.zip › RMI Supp Data/Interviews data/I33R_IDI_MCG_Arno_Sep 13_BM FelaEdited.docx]

**Interview Code: I33R**

**Interview type and Interviewee: Male Caregiver**

**Interview Date: Sep 13 2018**

**Location: Arno**

**Interviewer: BM**

**Transcriber: Shante**

**I: Now you will answer if it’s ok for us to use this recorder, you will answer. Is it ok?**

R: yes

**I: Good. Ok, thank you for your time. Now can you tell me about your family?**

R: About what?

**I: your family.**

R: like what?

**I: Like who lives in your house and how many kids lives there and how old are they?**

R: Me and my wife and our 7 children.

**I: who lives in your house?**

R: me and my wife, our kids and grandkids.

**I: how many kids?**

R: 3 kids.

**I: what about your grandchildren’s? your 3 grandchildren’s?**

R: yes

**I: and you and your wife?**

R: yes

**I: so, you mean just you 5?**

R: yes

**I: how old are your grandchildren’s?**

R: one is 5 years old and the other is 3 years old and the youngest hasn’t turn 1 yet.

**I: Good. What about boys and girls?**

R: 2 boys and 1 girl.

**I: 2 boys and 1 girl?**

R: Yeah. My grandkids.

**I: Good. Can you tell me about this community?**

R: Like what? This house or my house?

**I: yes, or around your house… there’s no right and wrong. Everything you say or want to say it’s good. How is your community? good or bad? Tell me the reason why it’s good and bad. We’re just talking stories about these things.**

R: well, I would say it’s enough because we eat food from the plants we grow.

**I: like what?**

**R: Like bananas. We take them to Majuro and pound them.**

**I: hmm… so, how much do you reach?**

R: like if one banana, it’s $1 per pound, but if we bound the big bananas, then $100 will come out of it. For the medium size bananas then $80 something. If I bring 4 bananas, then I would make like $400 out of it. And we also survive with coconut. If we make for bags of coconut, we bring it to Majuro Tabolar. (it’s where they bring their bags of coconuts and pounds them to make money.) and Pound it there. When I bring and pound them there, I would make $5,000 out of it with 40 bags of coconut.

**I: That is a lot… So, what about the bad things in this community?**

R: Like what?

**I: the bad things in this community.**

R: Well, we see that we are struggling when there’s a lot of people in the house because sometimes the things we had will run out faster. And we don’t stop working. For me I didn’t go to school so now I survive with coconuts.

**I: Ok.**

R: The first time it was just me and my wife and we weren’t struggling as much. But now that we have our children’s and our grandchildren, we work twice as hard to find our ways to live.

**I: hmm… those are good information. Now can we talk about illnesses that your children have suffered from?**

R: fever…. And like stomach bump. When they like fall and they will get a stomach bump and sometimes they will get a fever from it and sometimes the fever will get high and they might die from it so, we use traditional medicines to heal them. We let them dip in the medicine and let them drink the medicines and soon their stomach bump will be gone. When they have stomach bump they don’t eat. this is usually the main sickness they have. Because they fall a lot and their stomach will get a bump. Especially to boys… when they get a stomach bump usually it takes longer for them to heal… they can drink medicines from the hospital, but it will only make the fever go away but not the stomach bump. They won’t eat because of the pain in their stomach. They need to be dip in a tub of traditional medicines to help the pain and to get rid of the stomach bump.

**I: So, you guys use traditional medicines?**

R: Yes.

**I: So, can you tell me why, do they get fever?**

R: It’s when they go outside they get fever… like to the place that are windy. Like the lagoon side, and they bath them when it’s windy, and when we bring them to the doctor they said the child is really burning hot and they give us medicines for the child to drink it.

**I: oh…**

R: That’s what the doctor said… he said that we let the child be in the cold wind too much, when we put them on the ground cement that is cold then they will get fever too. When they see that they have fever, these are what the doctors tell us. So, they give us medicines for fever.

**I: that’s good because you guys bring them to the doctors. What are the dangerous issues on high fever?**

R: it’s really dangerous because sometimes, you know their eyes. They will get stuck, like their eyes won’t close, like their eye balls rolls. When they get fever, sometimes they can’t breathe, like they can’t shut their eyes. So, we bring them to the doctors and they give them medicines to heal them. Sometimes when their fever is really high we dip them in a tub full of water mix with traditional medicines. When my granddaughter was fever I brought her to the hospital, her fever was really high, like high fever. They just dipped her in the traditional medicines and wrap her around with a sheet to keep her warm. Her fever was really high that the hospital medicines took so long to work, so we just dipped her in the water of traditional medicines. Dip her in the water and after, wrap her around with the sheets and finished. She’s healed.

**I: Good…**

R: It’s like they can’t hold there breathe, that’s how bad their fever is.

**I: hmm. That’s how you guys prevent the fever, and the doctor helped, not just that but, you guys gives her hospital meds.**

R: yeah. After every time we dipped her in her tub of traditional medicines, then we give her, her hospital meds to drink. We do this to her the medicines twice a day and she gets better.

**I: Ok. now can we go back to the part about stomach bump. What makes them have stomach bump?**

R: The old ladies says because they fall a lot, they play and when they tripped, they land on their stomachs. Sometimes they won’t know that they have stomach bumps. 5 months from now when they have fever, like really high fever they will check their stomach, and they’ll see that their stomach has a really big lump in it. They go see the doctor about it, but their stomach won’t be healed with the hospital medicines. They are only cured with traditional medicines. When they are being dipped inside the tub.

**I: what is the danger of stomach bump?**

R: if they have stomach bump they won’t want to eat. they won’t eat. they can’t sit and eat, and if they like breath it’s hard for them because of the pain of their stomach. When you press their stomach, it’s like really hard and they’ll cry because it’s hurts. They say because we let them fall. Like for example, if they are learning to walk, they fall and sit down. the traditional healers say because they fall to much. For those little children, sometimes they tripped with the baby or they run around with them and they won’t know if they have a stomach bump until we know the baby has a fever and check her stomach then we’ll know she has a stomach bump, but when we give them traditional medicines they are cured.

**I: how dangerous is stomach bump?**

R: it’s really dangerous because some kids can die from it. The stomach bump is really painful, and they won’t want to eat because of the pain.

**I: Oh. So, it’s really dangerous.**

R: Yes. Very

**I: woror. (Like OMG) So, we have to be really careful. Ok. thank you for your information. Can you tell me how you can tell when your grandchild should see the doctor when she is sick?**

R: I take her to the hospital because there’s nothing much I could for her. I bring her to the hospital when she is sick. Sometimes I tell them, why don’t you guys bring her to the hospital? She’s really sick… sometimes she is cured with hospital medicines. When we bring her to the doc they gave us her medicines and give it to her to drink and she will get better.

**I: is there any signs that you see in your grandchild when you know she is sick?**

R: Well yes. When I touch her head is really hot and she looks weak and just want to lay down and sleep. When you look at her eyes, it’s like she looks weak and when you touch her whole body from her feet to head it’s hot. So, we rush to find a pickup truck to bring her to the hospital.

**I: So, do you guys use a traditional healer or traditional medicines?**

R: yes, we use traditional medicines like UNO IN KIJON KAN. (It’s some leaf’s mix with each other Medicines for weak child, makes their bodies strong.) when they are born that’s the time we usually bath them with that medicines. If we don’t bath them the medicines then some of them might get like, like the body is really tight and straight, (almost like a stroke but for kids) they don’t know how to do this, just this. (Doing an action mode to BM while saying that.) when daylight they bath the child with the medicines and the child will be ok. they won’t get tighten again and that’s why it’s called UNO IN KIJON KAN. There’s a lot of traditional medicines here, like for crying and medicines that will prevent them from injuries, like if their parents are with some other people, we have medicines that will make the child not be affect by what their parents are doing with some other people. It will make them not have diarrhea. Even though the mother is pregnant, but the child won’t get hurt because they already prepared the child when he was still a baby.

**I: good. Your information’s are good. Thank you for your information’s. What kind of illness will it effect your grandchild when he doesn’t have much Nutrition and nutrition in his food? Like what kind of illnesses will he get from eating different foods, like if he eats just rice and he gets sick from it?**

R: it won’t be enough vitamin and they might get pink eyes so, they’ll need medicines and we’ll go to the hospital so that they can give him a medicine because they the child can’t see us, when is night them they can’t see us and that’s from us just feeding him rice. Rice and those other foods that has no vitamin in them. When we give them crab and fish, give them food that has vitamin in them like papaya and coconut meat. My granddaughter had night blind, at night she usually bumps into the walls, when it’s really dark she bumps to that house but at day time she can see well. But at night, not really. Bring her to the doc and she’ll be ok. and feed her the food that they told me to feed her.

**I: like what?**

R: Papaya, pandanus, banana, fish and all the things that will make her eyes strong. And when I feed her these. She looks healthy. They give me vitamin pills and I give it to her. There’s a lot of children that they get night blind because of the food they eat. when is time for you to give them food, the food you give them don’t have enough nutrition in them. Children gets night blind.

**I: what kind of food will make your child not healthy? Like what kind of food does the child will eat and won’t get healthy because of it? Like what kind food will make the child go weak and not make him strong?**

R: make him weak?

**I: yeah, like make him weak and make him not grow healthier? What kind of food are those?**

R: the foods that don’t have vitamin in them.

**I: good. So, how do you see it that the food, don’t have vitamin in them?**

R: what?

**I: because you said no vitamin in the food, so why won’t it make the child not grow healthier?**

R: because it’s not a food for him to grow healthier.

**I: like if it would say, junk food, if the child eats lots of junk foods and eat chocolate and chips and…**

R: yeah.

**I: what kind of food would your grandchild eat to be healthy and why?**

R: those foods that has vitamin in them.

**I: and why do you say so? Why does he eats food that has vitamin in them makes him healthy?**

R: because it’s good for his body and make his whole body strong.

**I: What kind of illness will the child gets if they don’t have nutrition in their food?**

R: skin rash (rajia), pink eyes and they don’t grow fast, I think those are the only things I know.

**I: that’s good. Thank you. can you tell me in detail in a day how someone takes care of their health from the time he is awake to the time he goes to sleep? Do you understand?**

R: wake up in the morning… throughout the day?

**I: all of that day…**

R: like 24 hours?

**I: yeah. 24 hours. How does someone take care of his health in a day?**

R: if for me, I wake up, they cook me food. I eat in the morning, then I do a lot of work, and again at 12 o’clock they make me food again and then work again and then eat again at evening. And then I shower then sleep.

**I: can you tell me the signs of a healthy child under the age of 2? From your own point of view. Like what are the signs? You see signs of healthy life of a child.**

R: at 2 years old?

**I: 2 and below**

R: I think…

**I: do you see it in your grandchildren’s? like if they are healthy…**

R: yeah, when I see it, they don’t have illnesses because they are always playing, and they don’t look weak, just strong and they don’t feel weak because they are not sick.

**I: ok. what are the signs of a healthy adult? It’s like how you said it before, like you worked all day and then eat, it’s like your full of energy and fully active, and not being lazy and wants to lay around…your healthy, like if you see someone who is healthy its when they are active, and they move around a lot. Do you usually see any adults that are like this?**

R: hmm… (Yes)

**I: now, we would like to learn about the foods that are commonly available in your community. can you explain how your household gets food to eat on a daily basis?**

R: we make 2 bag of coconut and go pound them and then exchange it with rice, sugar and bread. And we cook it and then eat it.

**I: where do you go to pound the coconut bags?**

R: the stores.

**I: so, you guys pound coconut huh?**

R: yeah, we pound coconut and they work by 47cent 1 coconut. So, if a total of a coconut is 150, then that’ll be $70 something a bag. Or if it’s 100 something coconuts, then it’ll be $90 something dollars.

**I: hmm.**

R: but if it’s 2 bags something coconuts, then you’ll get 200 something cash. So, you’ll get want you need and then if you have change they will give you your change. If you have 100 something change, then you’ll have more to buy, this is how we survive.

**I: hmm. What about fish?**

R: Well, when they come home, they come with fish so, we will have meat for our food. Everyday we eat fish at my house because the guys go fishing so we could have meat.

**I: what kinds of plants grow near your house?**

R: banana, lime, papaya, breadfruit and coconut juice. But they don’t grow well because the soil there is salty. Some area for planting is good, like Ine. There are a lot of spots for planting. The plants there looks alive. But here the area is salty, but it’s ok because at least the banana grows and that’s where we eat and how we live. And it helps us with for other supplies like school supplies.

**I: the plants that grow at the house, your family sells them?**

R: lime, we sell them.

**I: so, what do you guys do with what you earn?**

R: we buy our foods from Majuro because it’s cheaper there. But here, rice cause $20 but at Majuro it cost 9 to 10 dollars. But when we ride to Majuro, we buy a lot of food there because it’s cheaper there than here. Like here rice cost 20 dollar and flour cost 23 dollars. It’s a struggle here we live in so we find a way to pound our things just to go buy things at Majuro. That’s the reason we plant coconut tree so that we can collet them and pound them and get money, so we can get our needs.

**I: good. Now can you tell me if it’s difficult to do planting around here?**

R: No. I don’t think so.

**I: no difficulties?**

R: difficulties against what?

**I: against planting**

R: on how we block it or make fences?

**I: yeah. On how you blocked them…**

R: No. no difficulties because the pigs are tied up. We tie them up so that they won’t eat the bananas.

**I: what else would your family wants to plant near your house? Like what other plants would they want to plant?**

R: the plants that I see that are alive is pumpkin, potato, lime and I think those are the things I know that can grow alive, not like breadfruit, it can grow and die because of salty air, the salty air can damage the plant to grow healthier, we plant them and they grow healthy, but it’s the salty air that destroys the plants. It’s the salty wind and waves that makes the plants die. We can spray the plants and have of the plant will die because of the salt, it damages the branches. Those are the only plants that will die because of the salty air. The plants that we see them remain standing are the only healthy plants we have.

**I: when you get paid, different places…can you tell me how easy or hard it is for you to have food in every month in a year? Like can you tell me the easy and difficult ways to get the food that you mentioned like pumpkin and potatoes because you said the plants are weak from the salty air. Now can you tell me the easy and difficult ways for you to get those foods.**

R: on how we plant them or?

**I: yeah. On where you plant them and… like how you see it, if its easy and hard to make the plants. What do you guys need for you to easily plant potatoes and lime.**

R: I would bring fertilizers and things like that and spray them so that they can grow. But if we use this soil it will grow but will die easily. If we buy the seeds in the stores it will grow good and we spray them with water, they will stay alive and good.

**I: good. In 1 year. What make it not enough and why? If like the plants you grow, and it’s not enough, What, do you do when it’s not enough? Why is it like that?**

R: yes

**I: why was it like that?**

R: sometimes it takes so long for them to grow or maybe because there are a lot of family members coming in or, I don’t know, sometimes the plants grow slow but that’s where we eat to survive, but if we have them we pound them and sometimes they grow slow, like for 5 months from now they grow. Coconuts, fish… we live also because of fish. We fish and then go pound them.

**I: like when you guys are not having enough food. That’s the only think for you and your family to eat is only fish…**

R: yeah, fishing and make money to buy rice.

**I: you guys sell fish too?**

R: they buy, and we pound them at Mimra on INE near the dock. All that house and at Mimra. They bring ice and we fish and what we caught will make enough money and we buy food.

**I: thank you for your information. Now we will talk about the animals that you raise at home. Could you tell me about them? And what kind are they?**

R: pigs, chickens. Those are the only things I raised

**I: what about dogs?**

R: Oh, and dogs. There’s dog I also raised dogs. I thought dogs are not important to mention in this talk.

**I: can you tell me why you raised them?**

R: my pigs, I raised them so that I can eat them, same goes for the chickens. And the dog, he’s my pet and he follow me around like to get the pigs away from my plants. And the cats to get the rats.

**I: there’s also cats?**

R: yes.

**I: can you tell me the difficulties and raising your animals?**

R: yes. Sometimes we run out of coconuts I feed the pigs and chickens with it, so we feed them rice because we’re running out of coconuts because people usually get them. So sometimes we feed them rice and fish. Sometimes we give the pigs breadfruit when we run out of breadfruits. And we sometimes cook the breadfruits and feed the pigs.

**I: can you tell me is it difficult to put them in a fence?**

R: yes. There is difficulties on putting them in a fence. When you put them in a fence, they grow slowly, even when you feed them for 8 months. If we put them in a fence and feed them there they will die. It’s good if they roam around and eat because they will grow faster and get big. If you feed them they will grow. And if you just feed them coconut, they will get tired of eating them and they won’t like it. They’ll get tired of eating it. We let them go so that they can eat wherever and its good for them.

**I: what do you do with the animal stool?**

R: I take the cooler near the lime tree and put garbage inside and the pig poops and throw it to the lime tree to keep it alive, feed it.

**I: good.**

R: I don’t throw them in the lagoon, I gather them and take them to the lime tree. That’s how my lime tree is alive. When we don’t sprinkle them with like that they die. Even though you say its alive and big but no matter they will die, because it’s salty the weather and it’s to hard for the tree to handle, but if we sprinkle the tree with that then it will be good.

**I: good. It’s good how you know how to plant. Both giggles. There are times that we want to eat the food we want to eat but sometimes we won’t get them plus they are barely available. What kind of food do your family wants to eat?**

R: we eat a lot of fish and we get tired of eating fish, there are times my family wants to eat can foods from the stores, like cornbeef, if they get tired of eating it they’ll say they want to eat maceral, if they get tired of eating maceral then they would want to eat tuna. If they get tired of it, ill go find craps and they eat them. Those are the foods I bring them to eat because they get tired of eating some other foods. So, I bring them the food they want to eat from the stores.

**I: what about the foods that we barely see that you guys want to eat them?**

R: well I go bring them from Majuro. It’s right. The food we want to eat don’t got them here, so I get them from Majuro

**I: what prevents you to eat those foods all the time? You know the foods you guys wanted to eat yeah? Like the foods you go find at Majuro that you guys want to eat?**

R: orange and…

**I: fruits?**

R: yeah.

**I: what prevents you to get those foods?**

R: when we eat them, we don’t think about them anymore, so we just stopped. We’ll just stay around and all a sudden we would want to eat them again and we will look for them, but not all the time because they are far from me. I’ll just stay and say ohh, I already eat that and after a few months I go on the boats to bring food from Majuro.

**I: That’s the only way to prevent**

R: I won’t go if there Is no money. That’s also a big issue because if no money then we won’t get what we want to eat because there is no money, wait until we pound.

**I: for the last question on food, can you tell me who decides what food to get for your family?**

R: sometimes my wife cooks, she will cook from morning and tells me what kind of meat we will eat? and I’ll say I don’t know, did the guys go fishing because the rice is cook, I’ll say I don’t know, she’ll say well go bring corn beef. I’ll bring corn beef and open them and we eat, no sauce or anything, we just eat.

**I: How do your family choose your foods? Do you and your wife choose the foods or the people in your house?**

R: me and my wife.

**I: you two choose the foods?**

R: yes.

**I: who choose the foods for your children?**

R: Themselves, they tell their mother what they want to eat and she tells me and I’ll go find what they want to eat.

**I: their mothers tell you what they want to eat?**

R: yeah. Like she’ll say, oh the child wants to eat this and that, so ill find want they want to eat.

**I: ok. we talked about foods. We will now talk about water and cleaning. Is it ok?**

R: good.

**I: can you tell me about a typical day on getting and storing water?**

R: water for drinking?

**I: yes. Drinking water or water to bath…**

R: well, what about it?

**I: how you get water and store them?**

R: Oh, I fill up my water and take care of it so that it won’t run out quickly and fill up my other water tanks over there, those containers over there, I fill them up and close their lids. And keep them because if there are no water, it will be difficult, if we run out of water we use the water wells.

**I: you guys have tanks? (Pontoon)**

R: Yeah. over there.

**I: Ok. good. Where do you guys get water from? like for drinking and for bath?**

R: My house. When it rains I fill the buckets up… they are right there.

**I: the tanks there?**

R: yeah. those. Those are for showering and washing. That water tank there is for drinking only.

**I: this pontoon is for drinking only?**

R: yeah.

**I: what are the difficulties in getting for water? Is there any difficulties?**

R: yes. There are difficulties.

**I: can you give me in detail why is it difficult?**

R: not enough spots to get the waters.

**I: Not enough spots to get water?**

R: Yes, and they like measure the waters for us and give it to us. Like we fill in 10 gallons and we save some for tomorrow, the other day we go again and fill up 10 gallons. We pay for them and go to INE and fill them up, but we go on the lines, we pay the trucks $10 to go to INE, and get our drinking water from there. But the thing is we lined up to get our water. There is a water filter there and we are using it to get our clean water because we get our drinking water from the well. But you have to ten dollar for the vehicle to get to Ine.

**I: Geez. That’s cost a lot**

R: a lot! It’s really difficult when we don’t have enough water. Especially for this family. It’s not good. We need to save lots of water. Or make a cement water tank to keep our own water.

**I: what are the issues in keeping the waters? If your tanks are empty? How else do you save your waters?**

R: we bring water from INE and we fill up the tanks. Until it rains, and we will now fill up the tanks for drinking.

**I: how do your family clean the water tank?**

R: we open them and clean inside and pour Clorox and scrub inside and clean the water after. And now we fill it up.

**I: let’s now discuss hand washing, could you describe in detail your family’s hand washing throughout the day?**

R: I think 3 times a day because we eat in the morning, they wash their hands, eat in noon, wash their hands, at evening, wash their hands. We wash our hands before eating. Soap it and wash it after. Make it clean before eating.

**I: good. What about the kids? How do they wash their hands in a day?**

R: me and my wife wash their hands before they eat.

**I: how do you guys wash their hands?**

R: we take the soap and soap their hands and wash them and finished. We take them and now feed them.

**I: good. What about in a day? How often do you guys use soaps? In a whole day, like how often do you guys use soap in a day?**

R: well usually we won’t finish a soap in a day, so the soap we use will last long for another day. Tomorrow we will use the exact same soap.

**I: how many times do you guys use the soap in one day?**

R: like 5 or something, wash our hands in the morning, noon and evening, when showering, that’s 4 times.

**I: hmm. Good. Can you tell me the difference between washing your hands with water only and washing your hands with soap?**

R: for me I think washing our hands with soap will make all the dirt gone, if we wash our hands with water it’ll be only like… I don’t really know, sometimes we wash our hands with soap for washing cloths, some say it’s good because it has Clorox in it, so we just wash our hands with it. And the time we wash our hands is when we are going to eat.

**I: good. What prevents washing hands with soap throughout the day? What prevents you from using soap? You might run out of it or?**

R: we run out. When we run out of soap, we go to the stores and buy them. The only thing that prevent us from washing our hands with soap is when there is no more soap.

**I: could you describe the type of toilet that you have at your home? Do you guys have any toilets in your home?**

R: No. we only use the ocean side.

**I: can you tell me in detail why you guys don’t have a toilet bathroom?**

R: we don’t know how to make one, plus we don’t have money to make one. We only have enough money for food and for the students. We have enough like, the body for the bathroom, like walls for it but it’s far from here.

**I: the next question would really necessary for you to answer because you say you don’t have a toilet. You would probably know about why others don’t use the rest rooms even though they have. Some people have money to buy the things to make a bath room, but they don’t. they rather use the lagoon side. Can you tell me why they do that in some places but other places they don’t?**

R: they probably are not used to use the bathrooms. Because they have bathrooms at their houses, but they just want to use the lagoon side. If they use the lagoon side, it’s probably because they don’t have enough water.

**I: Maybe. What are the issues in using the toilets? Oh. No. sorry. What are the issues on building a toilet?**

R: for me, I don’t have enough money to build a toilet. If I have enough money, then I will build one so that it would be safer and better environment.

**I: how do you take away the kids stools??**

R: we take it and dig a hole on the lagoon side and throw it in there and cover it with the sand so that flies won’t go there.

**I: can you tell me where your children usually play at?**

R: well, each place’s they play at, like near those houses and near the lagoon side and near the road. Just they play in different areas. Like near this area.

**I: can you imagined a playground that is perfect for your grandchildren? Like where would you prefer for your grandchildren and kids in this area should play that is safer? Can you imagine where it would be nice for them to play?**

R: near there school. Because they go to school then after they can play.

**I: can you explain why you chose the school?**

R: because that’s where they go everyday from morning to evening. From the time they start their class to the time they finish school then they will play there.

**I: do kids play where there’s animals?**

R: they don’t. the dogs go next to them but nothing there’s no other animals where they play at.

**I: oh, just the dogs?... what are the issues for the kid playground to be clean… like you know the kid playground? What are the issues in keeping it clean?**

R: to be clean?

**I: to be cleaned only.**

R: if we don’t clean it, it will be dirty.

**I: that’s the issue?**

R: yes

**I: now to end the second question, can you tell me how we stop or prevents illnesses that spreads?**

R: illnesses that spread from every person.

**I: can you tell me a little bit about it. Illnesses that spreads.**

R: be safe and clean. Clean our area, keep trashes where they supposed to be keep…

**I: that’s good. Is there any other?**

R: I don’t know why I have little information…

**I: on how you think about stool that are all over the place and illnesses that shows? Do illnesses comes from that?? On how you think about it. Can we get sick from feces?**

R: yes.

**I: we will now go on to the next question. Could you describe the care of children throughout the day in your community? like how does your neighbors look after your grandchildren in a day?**

R: they feed them, and they watch them from the shore so that they won’t get drown, day and night we watched them so that they won’t go to the other places because they are still young, and they can’t watch over themselves. Watch them so that they won’t go to other places and get hurt.

**I: who is responsible on taking care of the child?**

R: My wife and I are responsible in taking care of them

**I: from your own point of view. What are the responsible for mothers on caring for the children? What is the responsibility for a mother as a caregiver?**

R: she breastfed the child, when the child pooped or pee, she cleans them, put them to sleep, when they wake up she watch over them. Feed them and stuff like that.

**I: anymore? One responsible for a mother? Those are the only ones? Well other then the mothers and father duty… for a father duty, what’s his responsible?**

R: bring the food to their mother so that she can cook for the kids. Look for what the kids cry for and wants. If the child wants to drink coconut juice I will climb and get it. All the things that are right for the father’s responsible, we do them.

**I: good. If there are others taking care of your child other then you two, how does the person play with your child?**

R: she plays with them and watch them.

**I: how does she play with them and watch them? Can you give me in detail how she does that? What do they do? They play tag or race or read books or?**

R: they watch movies.

**I: movies?**

R: they watch movies in the house and sleep.

**I: ok. what else? Anything else they do?**

R: they just watch over them make sure they don’t get hurt.

**I: ok. could you tell me about the role of grandparents have in raising children in this community? what will happen if a grandparent watched over their grandchild?**

R: we watched them when their parents are busy, like when the mother is busy washing the cloths and when their dad is collecting coconuts, things like that.

**I: is there any other, other than that?**

R: I think no more.

**I: can you tell me about the way you grandparents support the child and the parents?**

R: help them when the child is sick or help them take the child to the hospital.

**I: what makes a good grandparent? Make them good and nice. If it was you that you were watching over the kids and teach them and…**

R: it shows that I love them.

**I: on how you say that you care for them, how do you show it in action to show that you love them?**

R: bring them close and hug them and watch them. And kiss them.

**I: is there any time that you tell them stories?**

R: yes, I do.

I: stories about long time ago and, teach them about our culture and how we lived long time ago.

R: yes.

**I: Could you talk about the role that other family members have in raise young children?**

R: and how they watch over the kids?

**I: yes, on how they watch over the kids.**

R: I tell them to cook their food and boil their water and…

**I: how do the older kids take care of their siblings? For example, your grandson’s, how do they take care of her sister? Do they play with her or watch over her or?**

R: they play with them and watch over her.

**I: can you tell me a story about that, if your busy and no one watch over the kids and they watch over their sister…**

R: I’m usually busy and they call out to me and say, ‘hey he’s on top of her, they ye’ll and say, he stepped on her stomach, so I just ran over and taker her.

**I: do the kids know how to watch after each other?**

R: yeah.

**I: ok. your information is good. we are almost done, is it ok?**

R: hmm.

**I: only 3 more questions and we are done. could you tell me where you usually get your trusted information about nutrition and health?**

R: family planning.

**I: family planning? Family planning is located where?**

R: Majuro

**I: you were at family planning at Majuro? The place you take your information from is far. Sorry. I need to come closer so that the recorder can record our voice. Why do you trust the information from the place you heard it from? because that’s Majuro and Majuro it’s far. Because Majuro is far, what about here? At Arno. Where do you think the information should have been gone too for you to easily walk around and see the information? Where would it be easy for you to see it? Where should the information should be at?**

R: where people usually gather together.

**I: like where? Here?**

R: hmm… the school, the church.

**I: the school and church… good. Good. Other then you going to Majuro, how else did you get the information?**

R: we hear them on the radio.

**I: radio… good. Here they use radio… is there internet?**

R: there is.

**I: there is internet… Ok. we will now come to the last question. For the ones that are parents, like you are a father and a grandfather, can you tell me what influence you on how to raise your child or your grandchildren?**

R: regarding what?

**I: what influence you…**

R: watch them and stay with them and protect them.

**I: what are the thoughts of the community in the family raising his child? Like for an example, the leaders or the people around your house or the pastors in the church or the public health workers? That’s for an example. But the question is, what are the thoughts of the people in this community show how to care for their children?**

R: umm. I think the school.

**I the school? The school also tell them about parenting?**

R: hmm…

**I: is there any advice or an understanding you learned about a father or being a father?**

R: about what? The child’s father?

**I: yes, or any other understanding you learned about… is there anything you learned from this? About being father. Word of advice or the understanding of another child’s father?**

R: I don’t think so…

**I: you haven’t been in this kind of issues? No. its ok. I’m just following up. No. sorry, you didn’t learn about this and know one talked story about this and is there any thing you wanted to learn about a mother and father child, but you don’t see the information about it? What kind of lesson did you want to know about the mother and the father of the child? Is there anything you wanted to learn from?**

R: yes.

**I: can you tell me what are the things you want to learn from it? Because a mother and a father, how they watch over their child or how they play with them or how they feed them or what they need to do for their child so that they can be healthy like that… are these what you wanted to learn about?**

R: hmmm…

**I: yeah, these are what we should learn about… both giggles… because once you turn to be a mother and a father it will become a big responsibility because now we won’t think about ourselves because now we won’t think about ourselves, we now think about our children. Because that’s the line, they crossed the line, we are at the line and it’s good to be a mother and a father. Because if we crossed the line to be a mother and father then there is no turning back. We will no longer think about us, just about our kids. But it’s good. Thank you. we are done with our questions. Is there anything you want to say about what we talked about?**

R: no.

**I: ok. thanks a lot.**
