# Supplementary material for: Conceptualizing multi-level determinants of infant and young child nutrition in the Republic of Marshall Islands–a socio-ecological perspective
Source: PLOS Glob Public Health. 2022 Dec 19;2(12):e0001343. doi: 10.1371/journal.pgph.0001343 (PMC10022247; doi:10.1371/journal.pgph.0001343)
Supplement: S1 Data — (ZIP) [file pgph.0001343.s001.zip › RMI Supp Data/Interviews data/I34R_IDI_MCG_Arno_Sep 13_Balton word pad.docx]

**Interview Code: 134R**

**Interview type and Interviewee: IDI_MCG**

**Interview Date: September 13, 2018**

**Location: Arno**

**Interviewer: Balton**

**Transcriber: Cendaniel Milne**

**I: is it okay if we proceed on to talk? Well, as I was saying, my name is Balton. I come on behalf of Ministry of Health. We’re trying to do a survey with people, with people on young children. and things, things that are affecting them. Especially their way of growth life, and way of community. and these things. And to begin, can you explain a bit about your family? who else is with you or how many are you living?**

R: all of us.

**I: yes, all of you. Yes.**

R: us, let’s say.

**I: yes, good. We’re doing.**

R: two of us, my wife’s parents, her sister, and another one, the sister’s husband. And the children.

**I: how many children?**

R: two.

**I: how many children you two have?**

R: it’s the first

**I: how many months now?**

R: none

**I: still young right? … what are the total of adults in this house? … can you..**

R: five.

**I: five adults, and children?**

R: four children

**I: four children… what about in this community?**

R: all these people.

**I: how houses beside this house?**

R: all these houses are one.

**I: they’re the same right?**

R: all are family.

**I: how far is your neighbors? Which of these houses are near?**

R: well, they’re about this near.

**I: a bit far?**

R: near the school buildings.

**I: well, it seems far. What you think that is good about this side, of village, other than the side of protestant. Are there any good things or thing?**

R: I think every place are good.

**I: every place is good? No comments, it is a fact. Here on the ocean is cleaner than Majuro. We’ll start talking about children’s health. Can you explain any kind of illnesses you have seen many children have? It may not only the child but these two children from these two houses. What kind of illnesses they get infected by?**

R: fever and coughing.

**I: fever? what causes them to have fever?**

R: … they all of sudden in the evening they have fever.

**I: in the evening they get fever? how long they have, is it big?**

R: our daughter is really burning

**I: she used to have high fever?**

R: last night she had fever again.

**I: and how do you two?**

R: we took out the fever.

**I: just monitoring the temperature only? And what is the other thing? Coughing?**

R: coughing.

**I: and how long has she been sick?**

R: it may be deep.

**I: it may, so what did you two do?**

R: we brought her to a doctor.

**I: with the doctor junior?**

R: sometimes Zed, there is no medications.

**I: so what are the symptoms, the question is asking can you describe how you know when your child needs treatment for their illness? The questions that I am asking, we may know the because it’s our way of life, these the consultants would like to know these things. Because they don’t know how we, they want to know why, and they want to know these explanations from these people. And now for the question is what do you see in the child when having illness? What are the symptoms? For coughing? Well not for coughing it is much revealing, what if it’s fever, what are the symptoms you see?**

R: foreheads and body, when we touch the forehead is warming. Wait for a while, when touching it, it’s warmer.

**I: are there any other illnesses the child suffered from? Like diarrhea. diarrhea? she hasn’t suffer from diarrhea? very good. … when times that having fever and cough, who is the first you bring the child to before bringing to (05:32).**

R: bring to who?

**I: I said, is there anyone you let them care first, do you give to the grandmother or you give to someone that can treat or heal before you bring to hospital. (05:53)**

R: the mother, the mother touches her and knows.

**I: after knowing, she brings her to a doctor?**

R: we hastily, we feel her having fever, we make trip for the transports for a doctor.

**I: are there any time that you use traditional treatment for children’s illness?**

R: well, there are many.

**I: there are many times? What kind of illnesses that you do to**

R: internal injury.

**I: internal Injury? … and what else? can you give some examples?**

R: … internal injury, rubbing (traditional treatment on the stomach)

**I: it’s rubbing. So can you describe any illnesses affecting your children that are associated with nutrition? Is there an illness that might comes from malnutrition… you don’t know right?**

R: yes.

**I: do you know, the question says, what types of foods that make your child’s body unhealthy? What are the foods that makes our children unhealthy? For Majuro, candies from morning to the afternoon. Things that you know don’t, on this Island in this community, are there any foods that might make your child’s health not growing?**

R: there’s none in these places. Just local foods.

**I: yes. So what about the foods that you think can be healthy for the baby, what kind of foods are in these place?**

R: always eating pumpkin.

**I: Pumpkin.**

R: sometimes we look for ripe papaya

**I: oh!**

R: those that are riped.

**I: what else do you give to eat?**

R: fish

**I: fish.**

R: coconut meat (Iu)

**I: coconut meat (Iu)**

R: (08:33-08:34)

**I: poor majuro kids. you know this question that I am telling you, I did ask, but I have never heard these kinds of answers.**

R: crabs.

**I: hmm**

R: they do really eat.

**I: crabs in water or land?**

R: crabs on land

**I: on land?**

R: carbs in the far water too.

**I: hmm**

R: lobsters and those

**I: yes... are there anything else beside papaya, pumpkin, what other foods from.**

R: banana what other foods?

**I: banana and pandanus juice**

R: pandanus juice, we fed her since young

**I: since young?**

R: yes, since young. pandanus juice.

**I: hmm**

R: we grate and put inside a baby bottle and fed her

**I: poor majuro kids, there are no answers like these on majuro. we see them eating lolly pops and cereals.**

R: ... when we came from majuro, they don't be she will be alive.

R: they don't be she will be alive.

**I: that's where she was born, Majuro?**

R: she was small, she was premature.

**I: those that are premature?**

R: they were twins but the other died

**I: twins? so they told you that she would also die?**

R: yes

R: we came and fed her pandanus juice since she little till she's grown.

**I: that's her first food, pandanus juice?**

R: yes. when we went back to see a doctor they said is that her? they don't believe

**I:**

R: they don't believe it's her. they carry her and

**I: (10:33-10:35) but one that was born early? so the other twin is gone and she is the one that stayed? and they said she would die too? and you two came here and fed her pandanus juice**

R: fed her and when going back they said, she have grown so fast.

**I: man, what would you think if she would have not have these foods what kind of illnesses would be shown? things that she is eating.**

R: well, were looking everywhere. were looking, when we see a pandanus, she eats for a week.

**I: if she would have not eat these, she might have gone just like the doctors said? but from these..**

R: coconut she drinks

**I: she drinks coconut? ... we talked alot about being unhealthy, could you now describe for me a typical day for someone living a healthy lifestyle, from the time they wake up in the morning until when they go to bed?**

R: she plays

**I: she wakes up and?**

R: she does not cry, she plays. playing, when they make her laugh, she laughs and laughs.

**I: she does not always sleep?**

R: sometimes she sleeps

**I: during the times she's tired? she does not cry does she?**

R: she does not cry.

**I: what times she goes to sleep?**

R: it can be six o'clock, sometimes twelve in the noon.

**I: nap? so she wakes up and play?**

R: play, eat, and back to sleeping.

**I: she's healthy now? ... now, for an adult that is healthy, like two of you. let's say how do those with sickness, (12:51 to 12:53) those that are sick, how do get recover? they just stay, but how do, from your own, how does someone that is healthy, what are the things that a person does from the time the person wakes up till time of sleeping, what are the things that a person do? just in this place.**

R: working.

**I: can you list it down from morning to evening, if it was you, what do you do in the morning, the thing you do till evening. what do you frequently do?**

R: we work, doing the copra, (13:25)

**I: wake up in what time?**

R: seven o'clock.

**I: after?**

R: after eating, work till you rest in noon, then you work on the evening. eveing meal.

**I: when it's evening? eat and then sleep... those that are sick can't do nothing just stayed indoor. always (13:54) this land has good weather. when you work, our body is healthy. now, we'll talk about the foods that here on Island. can you explain how your household gets food to eat on a daily basis.**

R: ... food?

**I: yes. where do you get food from?**

R: from the stores.

**I: there are stores? where are they?**

R: there's one there.

**I: the big house next to the coconut trees there? that's a store?**

R: we go and trade with copras.

**I: selling the coconut copras? and then take those things?**

R: taking foods and meats

**I: what kind of foods you usually take?**

R: rice and meats in cans. flour.

**I: are there any foods that you grow here? that you ripe from? leen wojke (general foods grown on ground)**

R: there's none.

**I: what kind, what kind of meat that you**

R: fish and canned foods

**I: fish and canned foods, breadfruit and those?**

R: well it's been, we ate last years but there all out.

**I: breadfruits in this place are all gone? are there anything else you sell to store beside the coconut copra.**

R: I think it's coconut all over this place.

**I: .... Majuro, the people from laura, they always. you know the pandanus and breadfruits, they take them to town and sell them. does this place do this?**

R: they don't

**I: pigs and chickens?**

R: they don't.

**I: thank you becuase, when you answer you don't just say yes or no. but you tell story, and these are the answers were seeking because when you tell story the way of, it's good because we have a friend who transcribe from marshallese to english. the consultants that we work with, they look into these, and it's good for them to visualize way of life in these communities. from what you see for been living in this place, are there any difficulties in growing foods?**

R: there is none, every, every place are in this together to farm.

**I: there's no restrictions, there's no difficulties**

R: there is none.

**I: if you want grow something?**

R: you plant it.

**I: is there anything you have planted? I am looking around, pandanus, banana, is that a lime?**

R: lime.

**I: papaya, this is a breadfruit near the house right? there is no difficulties to grow right? majuro, they do, because it's crowded with houses, it's hard to farm near... there are some growing trees just like breadfruit and pandanus. there is a season, they say, how do they say? it's Anan-rak (season for pandanus) and Anan-ean (season for breadfruit). during those times, does affect you to eat? when it's not the time for pandanus or breadfruit, in those times, how do you look for food for the child. when it's not time for pandanus, where do you frequently look?**

R: we always, pumpkin because

**I: we'll let's change it to pumpkin, because pumpkin it's everyday.**

R: everyday.

**I: everyday right?... bananas.**

R: bananas.

**I: when it goes to the times where rice is all out. not enough food, what other places you can find food?**

R: other stores.

**I: there are in other stores?**

R: all of these places to the end, only stores.

**I: ... now, I will ask about the animals that you are raising at your home. are there any animals you're raising?**

R: chickens.

**I: chickens?**

R: chickens and pigs.

**I: ... where, and where do these pigs come from? lagoon side or ocean side**

R: they're everywhere. at the back

**I: are they in fence?**

R: they're not. they're roaming around.

**I: what about the chickens?**

R: they're not.

**I: is there a reason for you to raise chickens and pigs?**

R: none.

**I: (19:51)**

R: everyone just raise and let them go.

**I: let them go. are there any difficulties, are there any restrictions, do you want to..**

R: before it was, but it's gone now. because they say let them die, because they're lazy to make them food to their fence. they can let them go.

**I: let them go? when they're inside the fence they don't think about them because they're busy?**

R: they don't think about them.

**I: when letting them go, they just survive by their own? ... the thing that is affecting them by putting them inside a fence, because there are times we get busy and we don't feed them, and they die. but, the reason for letting them go is.**

R: they can feed themselves.

**I: (20:48) there are times where we would like eat something. it's not because we don't have any money with us. or there's none in any place, is there a food you would like to buy for your family and it's hard to find because of not enough money and there is none on Island. is there something you think you guys would like to eat? just give an example, every food that you would like to buy but you can't afford.**

R: in these places?

**I: anywhere. you can eat it on majuro, you can eat it on america and those places. is there any kind of food you might want the family to eat. it's like, having an evening meal or in the morning, during having a meal. Is there a food that you want it but not always.**

R:like...

**I: like for example, a family that want to eat pizza. how can they find a pizza on Island? is there a food you would like to bring for the children and adults in this place to eat? you saw it's delicious and you would.**

R: well, from chicken leg and those

**I: you wanted to eat a chicken?**

R: but the chicken's place is far

**I: the difficulty is that is far? chickens from the stores? yes... the kind of chicken on majuro?**

R: quater leg and those.

**I: quater leg?**

R: and these place because we're tired of eating

**I: chicken (local one ) ?**

R: chicken (local one).

**I: really?**

R: we want to eat majuro's chicken.

**I: opposite for majuro, wanting to eat chicken (processed one), and we want to eat chicken (local one). now, on this last question about food. who in this family decide which food to eat? the family's food. even in you community, who decide the food for the family? let's say tonight we'll be eating this.**

R: the old woman.

**I: the old woman?**

R: she's the one making foods.

**I: your job is to provide the food? (23:27) and who decides the food for the children?**

R: the woman, the mother.

**I: the mother?**

R: she's the one making the food.

**I: now we'll be talking about water and hygiene, can you explain how to store a water? how do find water and store water? how do you look for water in this house? water for drink and water for shower.**

R: water for drinking here it is. the water catchment.

**I: these houses use water catchment right? and these are thing you store, are there any wells?**

R: these places, there are no wells in these places.

**I: just water catchments only?**

R: it's salty.

**I: now, where do you take your water catchments?**

R: they were divided among, we don't know..

**I: government right?**

R: government.

**I: do they have price?**

R: the government bought them.

**I: it's for every communities? well, there's no difficulties for everyone are having each.**

R: well, everyone are having each one of them.

**I: I look around, every households are having solar right?**

R: solar and M.E.C too. it was here on Island, it was.

**I: and it's free too?**

R: I think there are on majuro right? the small islands at majuro.

**I: the small islands yes.**

R: there are

**I: alot of work with that. are there any difficulties to find water for drinking and showering and these things? what are things that can make the water catchment empty?**

R: well, when it's drought, really drought, drought, drought.

**I: when it's rarely to be rained.**

R: it goes to be empty.

**I: beside these things, now for these tanks. where do get them?**

R: those tanks, maybe they're from majuro.

**I: let's say, they were included with the water catchments?**

R: they did, they did not.

**I: is it hard to find these things, like if, for example if one of these things were broken. are there any difficulties to bring?, you need to go to majuro and bring them? this is the difficulty? but the only thing is, they've already gave you water catchment? ....... now, how do you say, how do you, are there any times you clean your water catchment?**

R: there are times. we clean.

**I: how?**

R: we take out the water.

**I: take out the water? when? when is raining or**

R: when is raining.

**I: none, when is raining. so you can take out the water so it can be filled again? is there a way to clean, do you clean, during that time, taking out the water is the cleaning time.**

R: some people came and show how to use, man what was that, cholorex.

**I: cholorex?**

R: others, came from majuro. came and cholornate waters

**I: they give out to families? cholorexs?**

R: they came and make cups and pour them inside.

**I: do they, do they teach your families? how to do it?**

R: they said, don't do it, I don't how many hours you begin to drink.

**I: wait till it.**

R: the bacteria dies.

**I: now, were going to talk about washing hands. how do the family washes their hands a day? like when do they wash their hands?**

R: times when eating.

**I: when eating?.... how do the children wash their hands? when, for children. exactly when do they wash their hands? during eating time too?**

R: during the times when they're taking shower.

**I: yes.**

R: their mother do clean them. there are times when she's busy, they don't.

**I: who washes the childrens' hands? let's say**

R: their mother.

**I: their mother right? when do they wash their hands with soap?**

R: ... after eating they wash their hands.

**I: before and after eating?.... from your own, are there any differences between washing hands with water and soap and washing hands with water only. what is he difference between them?**

R: others when washing with. not washing with soap it only makes it dirty. when you're working, .like cleaning outside the house.

**I: yes.**

R: when you come and wash your hands with water only, the stains can't wear off, when using soap, it's gone. and it's clean.

**I: there are times we don't wash, we can, there are time we don't wash our hands with the soap. just wash it, can tell me what time we don't wash our hands with soap. what makes someone not to wash his/her hands with soap? during the times of washing hands. can you just give me some examples. during the times when people don't use soap. when there is no soap they can't use it, for example. can there be anytime? some examples?.... they're in a hurry?**

R: they don't rub, when there's no soap we can't take a shower.

**I: well, we did (32:18-32:20) washing hands. are there any toilets in these houses?**

R: there are none.

**I: can government help with building toilets? there is no houses that does digging?**

R: there are some who makes them.

**I: are there any difficulties to make?**

R: none

**I: everyone can make them? ... now, which part of these places for toilet? lagoon or ocean side?**

R: ocean side.

**I: ocean side? ... it's so good, (33:32-33:35) the place is good. so now, where do the children use the toilet?**

R: well, the lagoon side.

**I: lagoon side because it's near.**

R: it's near.

**I: when using the lagoon side, do their stools stay there or get buried?**

R: they get buried.

**I: they get buried?... where do they usually play? children always play?**

R: near the houses.

**I: near the houses?... if there was a, if you see, a playground for the children. where would it, we would say, counsel build a playground. to build, you want to make a better place for the children to play. and where would you build it, what kind of play ground is it?**

R: the first play ground that was made was a basketball court. and place for ground skating.

**I: skating?**

R: we mainly build it in the schools. the schools.

**I: you usually bring kids, and where are these things are they still available?**

R: they're damaged. they've already...

**I: rusty? gone, they never renovated.**

R: they never did.

**I: .... where do the children often play a day?**

R: near the houses

**I: near the houses? ... and are there, what animals are there near the houses?**

R: pigs

**I: pigs**

R: chickens

**I: chickens... how do you guys clean? which days you guys clean all over this town?**

R: times when there's occassions, but they do clean outside of their houses. everyday

**I: everyday... the other qestion says, what are the difficulties that makes it hard clean the children's play area. what make it difficult to make children's play area clean, clean, clean, what things may make it hard for places where children play to make not clean?**

R: dirty

**I: what are, what are they?**

R: coconut trees, they are not to be under these things.

**I: when they fall, they fall by themselves?**

R: it may hit them. under the breadfruit, they are not to be.

**I: to wrap up all of these questions about hygiene, can explain ways of preventing illnesses from spreading? what are the things we can do to help prevent diseases from spreading? what do you think it might help prevent spreading of disease? for this Island, it's not like majuro, the thing is.**

R: everywhere is clean.

**I: yes. because cleaning? (37:51) you do every everyday, this is why it would be hard for children here on Island get sick unlike people on majuro. clean, clean, clean. now, this question, these other questions are about responsibilities of each family members. can you explain how do your community take care of the children in a typical day? like who's in charge of taking care of the child?...like who does take, always taking care of the child each day?**

R: the mother

**I: the mother right? what are the responsibilities of mothers? the mother of the children? what are their responsibilities?**

R: clean them

**I: clean them**

R: clean everywhere

**I: their surroundings right?**

R: doing the laundry

**I: doing the laundry..what about the fathers?**

R: work, doing the copra

**I: doing the copra**

R: cleaning up trashes.

**I: cleaning up trashes.**

R: sell, sell, when it's time to sell

**I: these are the things to bring foods? are there any older siblings for these children? like their cousins like that. how do they play with the children?**

R: they play with each other.

**I: they do help you two taking care of them?**

R: they play with them

**I: play with them sometimes taking care of them? from the way you see it, from your own understanding, can you say what outcome for grandparents in your community take part in taking care of the children in this community? what you see the way grandparents take part in it, is there a change to it? from not taking part in taking care of the children.**

R: I think they do, they do take care of them. when we get busy and went to places, they take care of them.

**I: they do take part in. and they also help the community too?**

R: when we're in far places, we call to take something, it helps alot.

**I: okay, okay, okay. and they do responsible for.... what are like, our grandparents, what are, how do they take care of the children?**

R: they play with them

**I: play with them? help babysitting?... the other question says, what makes grandparents being a good grandparents? how do you the grandparents that are good and bad? what shows that their good or bad? what are the things that they do?**

R: ...... I think it's good, it's good that they're..

**I: I said, the question says, there other grandparents that don't really take part and they don't usually, like what, take this grandparents that are best at taking care of their grandchildren. for other's, they're really good at taking care of their grandchildren. what things show good grandparents and bad grandparents? what are the things good grandparents do to show that they're good grandparents?**

R: there are some that take care of the needs that they want.

**I: taking care of their needs. anything else? give some examples. making sure their needs are done. what they want. how do the elders help you two?**

R: our needs, they do fufill them.

**I: for you two? not for the children but for you two?**

R: they help us.

**I: for baby sitting?**

R: baby sitting, everything.

**I: everything? are there any relative that do have the responsibility of taking care of your children?**

R: yes.

**I: like, what are the responsibilities for the family for the children, what do they do usually?**

R: they usually bring, when there are no diapers and these things. help out.

**I: okay, are there any siblings they usually help out right? other chidlren from these place help out by playing with them? and their siblings too. you know it's , in this place, the statements that are related to health. ways of taking care of children, where do you usually take information from, for taking care of a children? where do you first took information from? and often take.**

R: ....... health places

**I: hospital?**

R: yes.

**I: for majuro people they usually look into internet, because there's internet on majuro, watch tv, for this place, these things are gone. and what other places where you would listen to or watch, and take information about health? Radio?**

R: there is radio but, it's like they're gone, they're damaged.

**I: none. now, the information you remembered you took from hospital during the time you guys were in they showed. what you think that can help people here on Island or the families help spread a way how to take care a family, is there something that can be easily done for helping out a families here? spreading the information that can help you. and it can help to give information help you, to help you about health, how to take care of the children, is there a way that can help you? any answers are good. none? there are no right or wrong answers, now this is our last question for our interview. how many children do you have?**

R: it's the first.

**I: it was suppose to be two but, the other one is gone. you mention about after being born, the doctors gave up. but you didn't, you tried harder to make your child healthy. what makes, is there something that makes you to stay strong? was there any advice from an elder? what moves you to do the thing you needed to do?**

R: nothing, when I kept on feeding her. kept on look for foods anywhere. like pandanus.

**I: yes, what makes you move to try harder? are there any advices from elders, is there something that moves you to, who advice you with words of motivations, or by yourself, what moves you to.**

R: my mother, she said, you need to take good care of her because it's your first child.

**I: this is the thing that moves you? the elders said. you must take care of her, it's your first child. now, which of these twins? the younger one or the older one?**

R: the younger twin.

**I: the older twin is gone? yes. the first one came out died? but her when she came out, she cried? but they said she would not be alive?**

R: yes, they said she would not be alive.

**I: so, the grandmother (male care giver ) said, try your best because it's your first born.**

R: she came and took care of her, now she's alive.

**I: now the doctors are amazed by her?... hows, what is the community's thought about taking care a child?...like, what are the people's thought in the community, about way of taking care of their children? for example, leaders of the house, leaders of the church, like how do they thnk about this concept of taking care of a child? do they support it or do they take part in taking care of their children?**

R: yes. they do help people.

**I: people in these places? they help each other?**

R: they help each other.

**I: but are there any other things you wanted to learn about taking care of a children but, we didn't talk about? like are there anything you would like to know about?**

R: for?

**I: to take care your child, is there something you would wanted by we didn't, the questions you wanted me to ask but I didn't say. is there something that would, like it's, because the statement that were talking about is, are recorded and can be a way to help. government will help because all of these we reported it to the government. are there anything you wanted for the government can help families about health, but we didn't talk about. if there none, along with us came a nurse, chief nurse of majuro. she came with us too, if there are any questions you would like to understand about. she's with us, at the hospital, I don't know the place where is at the protestant church?**

R: Ine

**I: Ine? she's there, we're staying at the hospital. come visit anytime, there's coffee, (51:11) once again, thank you for your time. thank you for participating on our survey. on behalf of us that we came along with ministry of health. we thank you for participating by giving a precious time. this about it, thank you.**

R: your most thankful

**I: Balton. I will never forget your name.**
