# Supplementary material for: Conceptualizing multi-level determinants of infant and young child nutrition in the Republic of Marshall Islands–a socio-ecological perspective
Source: PLOS Glob Public Health. 2022 Dec 19;2(12):e0001343. doi: 10.1371/journal.pgph.0001343 (PMC10022247; doi:10.1371/journal.pgph.0001343)
Supplement: S1 Data — (ZIP) [file pgph.0001343.s001.zip › RMI Supp Data/Interviews data/I35R_IDI_FCG_Arni_Sep 13_Fela.docx]

- Interview Code: I35R
- Interview Type and interviewee: IDI_FCG
- Interview Date: Sept.13.18
- Location: Arno
- Interviewer: Fela
- Transcriber: Marcellina

**I: okay before we start recording our voice, I am asking you to tell it in your own voice that you are agreeing. Are you agreeing?**

R: yes

**I: thank you. Thank you for giving us your time to speak with us today. The information we learn here will help us find ways to improve maternal and child health and sanitation in your country. To begin with, can you please tell me a little about your family/household? Like for example, who lives in the household, how many children and their ages, how many daughter and son you have? like the people in this household.**

R: my mother and father, my older sister and her husband and their child. They have one child

**I: they have one child?**

R: yeah they have one child.

**I: what about how many children in this household and how old are they?**

R: one 5 years old, one is 4 years old, other one is 1 year old, and that one hasn’t have year yet

**I: might be how many months?**

R: 8

**I: eight months? Okay good. Now how girls or how many boys?**

R: 1 girl and 3 boys

**I: thanks. Now this question state, can you describe this community? Like what are the good things about this community?**

R: it’s good because we eat many kinds of local foods

**I: eat many kinds of local food. Okay good. can you raise your voice a little bit more, so the recorder can record all your information? It’s really good that you are eating local food. Are there any more good things about this community? Like is it peaceful…**

R: yes, because there are no drunker

**I: no drunk people, what else?......okay what about the bad things about this community?**

R: seems like there are none

**I: there are no bad things in this community. Okay good. Now we will now discuss about health and the illnesses in this family. Can you tell me about some of the illnesses that your children have suffered from?**

R: we also state the illnesses?

**I: yes**

R: the illnesses they usually get are fever, coughing, and asthma

**I: fever, coughing, and asthma. Now the other one was just an activity. But now we really need to answer in detail about fever, coughing, and asthma. Now this question is asking, what are the causes of fever?**

R: because we keep them a lot in the cold

**I: now coughing. What are the causes of coughing?**

R: when they have high fever, they will cough

**I: good. Now asthma?**

R: asthma. When they have fever and coughing, they will guarantee have asthma.

**I: good. Now this one state. As for fever, from your own understanding, why fever is really serious? Or what are the seriousness of fever?**

R: it is serious because when they have fever they might reach the stage where their body freeze or stuck

**I: freeze right? Okay good. Now what about coughing, why do we say it’s a serious illness? what are the seriousness of coughing?**

R: when they cough, they might cough until they are almost out of breath

**I: what about asthma? What is the seriousness of asthma?**

R: it’s same as coughing.

**I: now as for these illnesses you listed down, like coughing for example. How can you prevent coughing?**

R: give them medicines. We usually let them drink medicines.

**I: good. What about fever?**

R: we also give them medicines, but we also let them soak in water with local medicines because they said they might have swollen stomach which might be the cause of the fever.

**I: good. And also asthma? You guys also give local medicines when they have asthma?**

R: yeah. We give them medicines and also local medicines

**I: good. Now the next question state, can you describe how you know when your child needs treatment for their illness?**

R: when I see that the illness is getting worse

**I: now I am saying, who do you first go to when your child is sick and why do you seek that person first?**

R: in this community?

**I: yes it can be in this community or in this house. Who do you seek first when your child is sick?**

R: his/her grandmother.

**I: the grandmother?**

R: and my mother

**I: and the question is also asking why do you seek them first?**

R: because they know more better on how to take care of children

**I: and now it asking, do you use traditional medicines for the child?**

R: yes, we also use traditional medicines for the child

**I: for his/her illnesses?**

R: for the illnesses like when they’ve fallen and have swollen stomach

**I: good. Now it states, can you describe any illnesses affecting your children/child that are associated with nutrition? Like the foods that are nutritious. Are there any foods that your child consumed and have illnesses from?**

R: none

**I: good. Now it states, what kind of illnesses caused by foods missing from the diet? Like the foods that are not nutritious. What illnesses can affect your child from the foods that are lack of nutrient?**

R: things grow on their skin

**I: things grow on their skin. What else? Like korikori (skin rash) right?**

R: yes

**I: are there anything else?**

R: seems like no more

**I: okay. Now it states, we talked a lot about being unhealthy. Could you now describe for me a typical day of someone living a healthy lifestyle, from the time they wake up in the morning until when they go to bed?**

R: we describe his/her what? Children? People?

**I: like… yeah might be a person or any person. How do you observe them from the time they wake until they go to bed?**

R: not lazy; when he/she wakes up, he/she do his/her chores.

**I: good. Now it states, what are the signs or appearances of a healthy child under 2?**

R: sizeable

**I: good sizeable. What else?**

R: they know how to move around prematurely

**I: good what else? Are there anything else that can make you say “oh that child is healthy because..”**

R: they don’t get sick frequently

**I: good. Now it states, what are the signs or appearances of a healthy adult? Like you can said “oh that old lady or that old man is healthy because..”**

R: he/she is healthy because he/she loves to do chores and they don’t like to just lie down all day long.

**I: now the next question states, let’s now discuss hand washing. Could you describe in detail your family’s hand washing throughout the day?**

R: in where?

**I: in this household…. Well how do this family wash their hands?**

R: we wash our hands using soap.

**I: good. Now it states, do the children wash their hands throughout the day?**

R: yes, but sometimes they don’t

**I: sometimes they don’t wash their hands.**

R: yes, when we don’t help them wash their hands

**I: now, what does prevent the children from washing hands?**

R: if we don’t wash their hands then they won’t wash their hands

**I: now, what does prevent you from washing their hands?**

R: sometimes we got distracted and when we come to them they already eat their foods, but they didn’t wash their hands.

**I: when you got distracted, that’s the time you’re busy doing chores right?**

R: yes.

**I: good. Now it states, children that are under 2 years old, do they wash their hands throughout the day?**

R: yes.

**I: do they wash their hands by themselves or the mothers help them or?**

R: we usually wash their hands

**I: now the next question states, when do you use soap to wash your hands throughout the day?**

R: when we are about to eat

**I: when you are about to eat only?**

R: and also when I am done eating

**I: those are the only times you use soap to wash your hands?**

R: yes

**I: it states, can you tell the differences in washing hands using soap and washing hands with water only?**

R: when we eat foods that have fishy smell, and when we wash our hands with soap, the smell fade away

**I: and when you wash your hands with water only?**

R: the fishy smell won’t go away

**I: good. Are there anything else besides fishy smell?.... like do you think it would be okay if do your chores and just wash your hands with water only, would it be okay?**

R: no it won’t be okay

**I: well can you tell me why?**

R: because the germs won’t be killed

**I: so, when you wash your hands with water only the germs won’t get killed right? And when you wash your hands with soap, you really know that your hands will be very clean yeah?**

R: yes

**I: good. Now we will talk about your diet during your pregnancy and during breastfeeding. Now I want you to look back to when you were pregnant, can you tell me what types of food you eat during your pregnancy and the foods you eat when you are not pregnant?**

R: when I was pregnant, I usually ate breadfruit, ripe pandanus,

**I: good what else?**

R: ma chip (fried breadfruit), and papaya

**I: now about the pandanus, you just grabbed it from the pandanus tree and chew it right?**

R: yes

**I: papaya. Ripe papaya right?**

R: yes

**I: now what about the foods you eat when you were not pregnant?**

R: I usually eat rice

**I: good what else?**

R: rice and fish and…

**I: rice fish what else? Whatever you eat/ate during the time you are not pregnant.**

R: crab, rice, fish…. And sometimes canned foods

**I: now it states, what made you want to eat those kind of foods during your pregnancy? Like the breadfruit, pandanus, and papaya.**

R: I don’t know. Suddenly I just craved for them

**I: okay now it states, what types of food they encouraged you to eat during your pregnancy and the reasons why?**

R: who’s they?

**I: like you’re your husband or your family members. They wanted you to eat during your pregnancy and why did they encouraged you to eat the foods?**

R: they encouraged me to eat local foods

**I: local foods like what for example?**

R: banana, pumpkin, fish….

**I: good. Now what types of foods they encouraged you not to eat during your pregnancy?**

R: they usually tell me not to eat the ramen that I cooked it and mix it with Tabasco

**I: good. Are there anymore besides that?**

R: ramen mix with KOOL-AID

**I: good. What else? What about water?**

R: hot coffee

**I: good. Now who encouraged or discouraged you to eat these kind of foods during your pregnancy?**

R: my parents and my husband

**I: oh okay good. Now it states, who took care of you or supported you during the times you were pregnant?**

R: my husband and my parents

**I: how did they helped you during your pregnancy? Or like what did they do?**

R: they did whatever to get what I wanted to eat

**I: like when you wanted to eat what?**

R: like when I craved for breadfruit, they will cook some for me. And when I craved for pandanus and there is no more at our place, they go find some from other places.

**I: good. Now to the next question, it states, can you tell me what kind of medicines or supplements you took during your pregnancy?**

R: supplements that were given from the doctors

**I: supplements from the doctors. Now it states, what kind of supplements?**

R: the vitamins one and the one that helps producing blood

**I: are there anymore beside these two?**

R: that’s all I took

**I: okay good. Now it states, did you take all of your supplements given to you during your pregnancy and why did you take them all or why didn’t you?**

R: well I did take the supplements but every time I take one, I will vomit it out

**I: you will vomit it out?**

R: yes

**I: now why do you vomit out the supplement?**

R: I don’t know. I just don’t like the taste

**I: you don’t like the taste. Good. Now it states, did you drink any alcohol or smoking or take any drugs during your pregnancy?**

R: no I didn’t

**I: now it states, were there any traditional medicines you take during your pregnancy and the reasons why?**

R: yes there were

**I: traditional medicines?**

R: yes

**I: now it states, why did you take tradition medicines?**

R: they gave me traditional medicines so that I won’t really suffer a lot when delivering my baby

**I: good! You are really doing great on answering. Now it states, if there was someone encouraged you to eat fruits or vegetables, what made it difficult for you to do so? Like for example, your husband or your parents encourage you to eat fruits and vegetable, what made it difficult for you?**

R: the thing is, I won’t want to eat it if I don’t want to

**I: oh so that’s the difficulty, if you don’t want to eat it you won’t eat it?**

R: yes. If I want to then I’ll eat it

**I: now this one states, what would have make it easier for you to eat fruits and vegetables and the reasons why?**

R: if I see them all day

**I: you see them all day. Good. Now to the next question, it states, can you tell me the foods you eat during breastfeeding?**

R: fish,

**I: fish,**

R: foods that contain meats

**I: meats?**

R: local chicken

**I: local chicken and what else?**

R: rice

**I: good. Are there anything else?**

R: breadfruit and coconut drink

**I: good. Now it states, what really made you want to eat these kind of foods during your breastfeeding time?**

R: they recommended me to eat them so there would be enough breastmilk

**I: good. Now this one states, what types of foods they encouraged you not to eat during breastfeeding?**

R: salty foods

**I: salty food like what for example?**

R: salt fish because it’s really salty

**I: are there any other foods? Only the salty foods?**

R: yes

**I: okay. Now it says, who gave advice to you on what you should and what you should not eat during breastfeeding?**

R: my parents and my husband

**I: now it says, after you gave birth, can you describe how did you breastfeed your baby throughout the day? Like how long after giving birth you started to breastfeed your baby? Like did it take long for you to breastfeed your baby?**

R: no it didn’t take long

**I: can you really describe in detail at the time you gave birth**

R: I gave birth and after I gave birth, at the same moment I breastfeed my baby

**I: now why did you breastfeed your baby right away?**

R: because she/he was crying

**I: because of crying. Good. Now it says, did you give bottle milk or any other liquid to your baby in the first few days after giving birth?**

R: no, I didn’t

**I: and the reasons why?**

R: I breastfeed my baby until he/she was mature enough to be bottle feed. So, I bottle feed with milk because she/he consume a lot breastmilk so there wasn’t enough breastmilk. So, I gave him/her both breastmilk and bottle milk.

**I: now this one state, were there any difficulties or easy things for you to breastfeed your baby from the time he/she was born? Like what are the things that made it difficult or the things that made it easy for you to breastfeed your child from the time he/she was born?**

R: it’s difficult because I just gave birth but I breastfeed. I was still hurt.

**I: now what is the easy thing for you when you were breastfeeding?.... okay don’t worry about that question let’s move to the next question. Now this one state, can you tell me when did you first gave food to your child or give any liquids other than breastmilk?**

R: when he/she was 4 and 5 months old

**I: at 4 and 5 months old. Good. Now it says, why did you gave food or other liquid other than breastmilk to your child at that age?**

R: because the breastmilk are not enough anymore

**I: oh so when your baby breastfeed, he/she don’t have enough and want more right?**

R: yes, he/she will cry

**I: now this one states, what are the opinions of others to give food or other liquid to their child at that age? Like you for example, what came to your mind to start give food to your child at that age?**

R: because they are not full when breastfeed

**I: it says, what were the first foods and how they were prepared? Like what were the first food you gave to your child to eat?**

R: soft bread (made from flour mix with coconut milk)

**I: soft bread**

R: and pandanus juice

**I: now as for the soft bread, how do you prepare it or how do you cook it?**

R: just cook the flour with…

**I: like what are steps you do so that the soft bread can be done for your child?.. do you understand the question? Like do you cook it on the ground or do use the stove or?**

R: I cook it on the ground

**I: okay. Now can you explain how do you make the pumpkin?**

R: bring it and cut it and cook it

**I: cook it with what?**

R: like let it boil first and when it cook, we pour out the boiling water and then make it soft

**I: good. And you mix the pumpkin with what?**

R: mix it with coconut milk too

**I: the pumpkin and the coconut milk only?**

R: and then cook it again

**I: now, it states, we’re trying to know how people in this community eat. Can you describe in detail what your family eat or drink throughout the day?**

R: we usually eat rice, fish, and breadfruit.

**I: now can you describe how your family prepare these foods?**

R: they go fishing and the guy bring down the breadfruit

**I: bring down the breadfruit and cook it how?**

R: we also cook it on the ground. Make fire and cook it on the ground

**I: and how do you called that kind of cooking? Kwanjin?**

R: yeah kwajin ( kind of cooking when you cook breadfruit on the ground and scratch the skin using whatever is sharp for scratching like broken glasses)

**I: now this one states, who in the family is serve first, next, and last?**

R: first my parents.

**I: now who is serve next and last?**

R: us

**I: you guys after. Okay. Now it says, are there differences in the food given to different family members?**

R: there is none. If that family eat this kind of food, so is everyone else. Everyone eat the same food.

**I: it says, are there differences in the amount of food given to different family members?**

R: same amount

**I: good. Now it says, are there any children receive more amount of food than other?**

R: none

**I: they also have same amount of food?**

R: yes

**I: now this one states, can you describe how the family share their food during their mealtime? Like for example, children eating together and separately from the family, or meals eaten from the same plate by all family members?**

R: they eat together

**I: everyone share the same plate?**

R: no. like as for my mom, she usually feed the two children

**I: now the children eat with the adults?**

R: yes

**I: now this one says, we’ve heard that some family eat local foods whereas other family eat processed foods. Could you explain what types of food your family usually eat?**

R: rice, fish, and crab

I: good. Now it says, what does make it difficult or easy for you to cook local foods?

R: it’s easy for us to cook local foods because we see them everyday

**I: everyday? Now what does make it difficult to cook local foods?**

R: when there are no more or when they are not ripe yet

**I: that’s the difficulty?**

R: yes

**I: now this one says, what are the good or the bad things about local foods?**

R: local foods are good because they make us feel energetic

**I: good. Now what are the bad things?**

R: there are no bad things about local foods

**I: there are none? Okay good. Now what are the good or the bad things about the processed foods?**

R: we don’t see or have them everyday

**I: now do think it’s a good idea to eat a lot of processed foods? Like rice, chicken, or flour. Are these food good?**

R: they are good but not like local foods

**I: why what wrong with local foods?**

R: it’s good because we can have them everyday

**I: are they nutritious?**

R: yes

**I: what about the processed foods?**

R: they are not nutritious

**I: they are not nutritious. Okay. Now you’ve told me what your child under 2 usually eats. Could you explain to me the process, from start to finish, on how you prepare and cook a meal for your child?**

R: at what time?

**I: anytime. Anytime you prepare food your child.**

R: cook it and feed him/her and store it when he/she is done

**I: like, can you really describe it in detail. What is the first thing you do, what do you do to get the food done, and ways you feed your child.**

R: when we wake up, first thing we do is prepare their meal

**I: okay. Just cook it? Like if it was a pancake, how would you cook the pancake?**

R: we also make fire on the ground

**I: and mix the pancake and then?**

R: and then bake it

**I: good. And then feed her/him?**

R: yes

**I: do you wait until it cool down or do you just give it right away?**

R: I usually cool it down first

**I: good. Now it says, can you tell me what you think are the important foods for your child under two to grow well and be healthy?**

R: local foods

**I: local food like? Can you really describe in detail about the local foods?**

R: I can say the local foods I’ve already mentioned before?

**I: whatever food you think your child can eat and will grow well and healthy**

R: banana, pandanus, and coconut drink

**I: now it says, what kind of foods you should not give to children under 2 and why?**

R: salty foods

**I: what else?.... now this one says, what are the advices on feeding the children? Like what really encouraged you to feed your child?**

R: feed them on the right time

**I: good what else? Feed them on the right time and what else? Okay just feed them on the right time. Now this one says, can you tell me the differences on how you feed your son and how you feed your daughter? Do you have a daughter?**

R: no I don’t have a daughter

**I: okay let’s move on. Now this one says, we’re also interested in the roles and responsibilities that different family members play in raising children. Could you describe the care of children throughout the day in this community?**

R: who? all of the people in this community?

**I: yes**

R: they take care of them as same as we take care of their children

**I: I say who is mainly responsible in taking care of the child?**

R: which child?

**I: that child. Or every child in this community. Who is mainly responsible in taking care of them?**

R: their mother and their father

**I: now it says, what are the responsibilities of mothers in taking care of their children? Like you for example as a mother, what are your responsibilities for your child?**

R: make sure he don’t go to the lagoon side for he will drown

**I: good what else? What are your other responsibilities for you child? Do you feed him or do you husband feed him or your mother feed him**

R: I feed him most of the time but sometimes his father and his grandmother

**I: now it says, what are the responsibilities of fathers in taking care of their children?**

R: make food or bring food for their children

**I: like what kind of food? What should they do to bring food to their children?**

R: fish, they should go fishing

**I: good what else?**

R: go find crabs for them

**I: good. Now this one says, can you talk about the role of grandparents in raising children in this community? Like what do they do to them every day to help you in raising your child?**

R: they also take care of him

**I: they also help you taking care of him?**

R: yes

**I: now it says, how does caregivers play with children under 2? Like you for example, how do you play with your son?**

R: I let him play in the swing

**I: okay good. What else?**

R: take him to the shore and let him play in the sands

**I: going down to the next question it says, in what ways grandparents support in raising children and support the mothers or the fathers?**

R: they also help in buying diapers when there is no more

**I: what else?**

R: they also help in feeding the children

**I: okay good. This one says, what makes the grandparents a good grandparents?**

R: they are a good grandparents because they help us in taking care of our children

**I: good. Now could you talk about the role that other family members have in raising children in this community?**

R: when they see our children cry but we are busy, they will come and take them and comfort them. And also when they are hungry but their meals are late, they will take them and feed at their house.

**I: good. Now how does the older siblings take care of the young children?**

R: play with them and make sure they don’t go to the lagoon side

**I: you’re doing a great job, we are almost finish. Now for the last section, we would like to learn about ways we can develop health programs in your community. Could you explain where you usually get the information about nutrition and health?**

R: from the doctors

**I: from the doctors, okay. Now it says, why do you really trust where the information came from?**

R: because they are doctors

**I: okay, now it says where these information should be delivered to so you would see or hear them most easily?**

R: radio

**I: radio okay. What else?**

R: health centers in this atoll

**I: okay good. Now this one says, what types of media that you use mostly to communicate?**

R: GP radios

**I: GP? Okay what else?**

R: and the radio

**I: good. Now when you think about your own parenting behaviors, can you explain the differences in your parenting behaviors? Like when you thinking of taking care of your own child by yourself, what are the differences?**

R: I don’t like it when I am taking care of my child alone because it’s really tiring.

**I: what are the opinions of the people in this community influence you on how you raise your child? For example the leaders, neighbors, church leaders, or the health workers. Like do they give advice on how to raise children?**

R: yes they do

**I: like what?**

R: they tell us to take care of our children so they won’t drown

**I: okay. Now are there any advices or information related to parenting you have received?**

R: none

**I: okay it says, are there any desired information about parenting you wish to have but doesn’t available?**

R: none

**I: now there is one question I skipped. It’s about the first breastmilk. So, you know that first time you breastfeed your baby, did you give the first water or the colostrum to your baby?**

R: I don’t know because I breastfed my baby right away

**I: but from your own understanding, do you think that first breastmilk is nutritious?**

R: no because it’s a dan waan (colostrum)

**I: okay is there anything else about the topics we talked about today that we missed or that you would like to tell us about?**

R: none

**I: okay were done. Your information are really useful. Thank you so much.**
