# Supplementary material for: Conceptualizing multi-level determinants of infant and young child nutrition in the Republic of Marshall Islands–a socio-ecological perspective
Source: PLOS Glob Public Health. 2022 Dec 19;2(12):e0001343. doi: 10.1371/journal.pgph.0001343 (PMC10022247; doi:10.1371/journal.pgph.0001343)
Supplement: S1 Data — (ZIP) [file pgph.0001343.s001.zip › RMI Supp Data/Interviews data/I36R_IDI_HW_Arno_Sep 13_Fela.docx]

Interview Code: I36

Interview Type: In-depth Interview

Interview Date: Sept 13 2018

Interviewer: Fela

Transcriber: Fela

**I: Ok it started already. Before we start record our talk, I ask if you agree in this survey record, do you agree?**

R: Yes.

**I: Thank you. Thank you for giving me this time to speak with you today. The information we learn here will help us find ways to improve maternal and child health and sanitation in our country. To begin with, can you please tell me about your job as a traditional healer?**

R: I do local medicine only for children. There is a way that I bathe these children so that they can’t have stomach lump. Or make them drinks medicine made out of the banana tree. And I also let them bathe in a tub that has been put different local medicine with.

**I: what kind of medicine?**

R: I use the banana stem to make the medicine and do I have to tell you all my local medicine ingredients?

**I: yes please**

R:* Biliklikka* old Marshallese Word.

**I: Biliklikka? * laughing* sorry I don’t know what that is.**

R: it’s just a local plants how can I called that? I put use this together with the banana stem and pound them together so that it can turn to be a child bathing medicine healing thing.

**I: what do you put in your medicine, do you use water or?**

R: yes I rinse the pounded banana stem and the baliklikka tree in the water so and let the child bath in a tub or a bucket.

**I: ok that’s great thank you. Now what do you do on a typical day as a traditional healer?**

R: I usually bathe children using local medicines.

**I: like how?**

R: when there is sickness happening to them, I usually there to help healing their sickness with my local medicines.

**I: that’s great. Let’s now talk about illness. I am specifically interested in illnesses that children suffer from. In this community, what illnesses would you say children under two years commonly suffer from?**

R: fever like couching,

**I: ok that’s anything else?**

R: diarrhea

**I: anything else?**

R: I think that’s all I can mentioned about illness in this community

**I: alright. So that next question is asking about what are the causes of these illnesses. You mentioned fever, coughing, or diarrhea. As of fever, what caused the child to have fever illness?**

R: stomach lump caused the child to have fever

**I: ok so what I the cause of coughing?**

R: when they’re sick they cough too.

**I: what kind of sickness?**

R: when they’re having fever it also cause them coughing.

**I: and what about diarrhea? What cause the child to have diarrhea?**

R: the foods they are eating. If their foods are not really taken care of.

**I: now can you tell me how serious these illnesses are? Having fever, coughing and diarrhea**

R: having fever and diarrhea

**I: fever and diarrhea are serious illnesses?**

R: yes

**I: so can you tell me why do you say that these illnesses are serious? How serious are they?**

R: because as of diarrhea, when they can’t stop pooping, they would feel weak and their body is weak to move.

**I: and how serious is coughing?**

R: they would cough until they have asthma

**I: asthma?**

R: yes

**I: ok now in what ways that can help prevent these illnesses? Like fever, how can you prevent fever?**

R: I prepare for them a bathing local medicine

**I: what kind of local medicine?**

R: yes the same thing that I mentioned earlier, the banana tree and the baliklika tree.

**I: ok now how can you prevent coughing illness?**

R: I use the coconut oil to massage their neck.

**I: and what about diarrhea?**

R: I give them medicine for diarrhea

**I: local medicine or medicine from the hospital?**

R: there is local medicine but children don’t really use

**I: you mean for diarrhea?**

R: yes

**I: so you only give them medicine from the hospital to prevent diarrhea?**

R: yes

**I: how each one of these mentioned illness typically treated in this community? As of fever, coughing and diarrhea. How these illness typically heal in this community?**

R: stomach massage and the bathing medicine thing

**I: so it’s local medicine?**

R: yes

**I: Now can you explain what type of treatment people in your community seek for their children, for example traditional healers or doctors and nurses?**

R: local medicines and medicines from the hospital

**I: ok. Now can you tell me who they first go to for health care and can you give me reasons why?**

R: no respond

**I: if the child in your house is sick, who would be the first person that the mother would go to for health care?**

R: they usually bring them to the pastors so that they can pray with them or if not, they go the doctors.

**I: why does the mother belief so that she needs to bring the child for these people?**

R: because she knows that there is nothing else she can do to heal her child.

**I: do mothers use traditional healers to heal their children?**

R: yes they use local medicine and local healers to help heal their children’s illness

**I: Can you tell me about any challenges your community faces in seeking treatment for the illnesses you mentioned before?**

R: the challenges is that when they don’t have transportation to seek the doctors in the hospital.

**I: anything else?**

R: when they don’t have enough money with them to pay for their transportation to the hospital.

**I: is there is any difficulties you face in providing local medicine for your patients?**

R: so respond

**I: what are some difficulties you face when you do stomach massaging or providing local medicine for children?**

R: like what?

**I: any difficulties that you face, do you face any difficulties when you provide help by making local medicine for children? Any difficult you face when you do stomach massage or when you prepare the local medicine? Any difficulties you faced?**

R: there is no difficulties that I face when I do local medicine

**I: Now can you describe any illnesses associated with nutrition that affect children in your community?**

R: silent

**I: nutritious foods, when children eat healthy foods, do they get sick from eating these healthy or nutritious foods?**

R: they don’t get sick by eating nutritious foods

**I: What types of foods that make a child’s body unhealthy?**

R: I am sorry I do not know

**I: like what kind of meal children eat and resulting in having unhealthy body? It can’t be just children in this house but you can also talk about children in this community.**

R: I don’t know because in this community, I usually see children eating papaya.

**I: they usually eat papaya? And what if you would go to town, where there is lot of foreign foods, what kind of foods you’d see children eats? What kind of foods that would make a child’s body unhealthy?**

R: silent, no respond

**I: if a mother would only feed her child junk foods like chips and all kind of junk foods, would that child live healthy or not?**

R: no

**I: ok, so what kind of foods that children are given to eat and have unhealthy body?**

R: like noodle and chips.

**I: so now can you tell me what kind of foods that make a child’s body healthy?**

R: papaya, pandanus,

**I: ok what else?**

R: drink the coconut juice. Just local foods and drinks.

**I: We talked a lot about being unhealthy. Could you now describe for me a typical day of someone living a healthy lifestyle, from the time they wake up in the morning until when they go to bed?**

R: no respond

**I: like when you see someone that live healthy, can you describe to me the typical day of that person from the time they wake up until the time they go to bed?**

R: living healthy? I am not really sure.

**I: how would you describe a healthy person, what does the person do throughout the day?**

R: doing works around the house.

**I: ok what else? How can you tell that the person live healthy?**

R: the person lives healthy because he/she do works all the time, they don’t feel nausea.

**I: ok now can you tell me about the appearance or signs of a healthy child under two years old?**

R: that child is healthy and not feel dizzy or nausea, but always play.

**I: what are appearances or signs of a healthy adult?**

R: they don’t feel sleepy throughout the day, they are strong enough to move their body.

**I: I have one more set of illness questions but related to women’s health now. Could you tell me about your experiences with women who have anaemia?**

R: anaemia?

**I: yes**

R: because they eat a lot of salt and salty foods.

**I: now the question was asked about your experience with someone who live anaemia? When you look at the person, what do you see in them? Or how can you tell that the person is having anaemia?**

R: the person look skinny or their lips turn to a different colour like from normal colour (pink) to purple and does not ..

**I: does not what?**

R: move easily.

**I: do women who have anaemia think that it is a serious concern for them?**

R: yes they have serious concern about that.

**I: can you tell me the causes of anaemia in women of reproductive age and pregnancy?**

R: because they use stuff that are not good for their body.

**I: like what?**

R: they smoke, they eat salt and cool-aid and salty food.

**I: ok now are there any advice given to women for prevention and treatment of anaemia? If it was you, what would some word of advice that you would give to women who have anaemia illness? Like what would you advice these women about anaemia illness as a traditional healer?**

R: they should drink drinks or foods that help provide blood for their body.

**I: so you mentioned drink, what kind of drinks that help provide blood.**

R: local medicine drinks.

**I: you make their local drink medicine randomly or there is specific medicine you make for their drinks?**

R: there is specific medicine that I use to help provide blood for our body, and these women with anaemia needs to drink milk, water and eat sashimi fish a lot so that they can have enough blood. I: and what kind of foods that provide blood for the women?

**I: anything else aside from sashimi that help provide blood?**

R: no respond

**I: was the question understandable or not? Is there is any other foods aside from sashimi that can help provide blood?**

R: eat green leaves like the pele tree. (Local plant)

**I: pele plant also help make blood?**

R: yes and we also can make local medicine drinks out of it.

**I: can you tell me how do you make the pele drinks?**

R: I just bring the pele leaf and pound it and rinse it in a piece of cloth and add water to it then drink it.

**I: ok now I would like to talk about breastfeeding practices in this community. Can you talk about how long after birth most women start breastfeeding in your community?**

R: how long the child breastfeed?

**I: how long after birth women start breastfeeding? After they give birth, how long after they breastfeed the child?**

R: when the child cry they give her the baby for breastfeed.

**I: can you describe more after giving birth.**

R: ohh, it doesn’t really take too long for the nurses to hold the child with them, after they clean the child’s body, then they will give to the mother for breastfeeding when the child cry.

**I: when the child cry, she do breastfeeding right?**

R: yes

**I: are there any liquids other than breastmilk given in the first few days after birth and reasons why?**

R: there is none

**I: there is none? Is there is any reasons why?**

R: she needs to feeds her child breastmilk

**I: can you tell me the difficulties faced my mothers in this community to practicing exclusively breastfeeding for six months?**

R: no respond

**I: how do they do exclusive breastfeed in this community?**

R: still no respond

**I: did you understand the question?**

R: * shyly answered* “not really”

**I: can you explain how mothers do breastfeed in this community?**

R: they just do breastfeed

**I: you said breastfeed, can you elaborate more breastfeed in this community. Like how long do they breastfeed their children or how do they do breastfeed in this community does it take too long or they breastfeed real quick?**

R: it takes a little time for them to breastfeed their children because it deepened on how long the child will have enough to be fed.

**I: until they have enough to be fed okay. Are there any liquids other than breastmilk given in the first six months after birth and reason why?**

R: they usually give them the fresh coconut juice

**I: coconut juice ok, now why do mothers give other liquids like that coconut juice?**

R: they said that it is fresh and healthy and they can get it whenever they want and it’s also free to have it

**I: now can you tell me the difficulties faced by mothers in this community to practice exclusive breastfeeding for six months?**

R: no respond

**I: what makes it difficult for them to exclusive breastfeeding for the first six months? After gave birth**.

R: the difficult is that when they do breastfeed until they run out of breastmilk and the child would cry most of the time because he/she needs to be fed but the mother can’t give enough breastmilk to feed her child.

**I: any other difficulties to practice exclusive breastfeeding?**

R: they don’t do breastfeed because they don’t have breastmilk to feed the child.

**I: now can you tell some specific ways to better support mothers to exclusively breastfeed for six months?**

R: they usually told to do exclusive breastfeed so that the child can’t easily get illnesses.

**I: alright that’s great. Now we are trying to understand how people eat in this community. Could you describe in detail what most families usually eat and drink throughout the day?**

R: no respond

**I: in this community or in this family, can you describe what your families usually eat throughout the days. Like people in this house or people in that house, what do you usually eat throughout a day?**

R: rice

**I: rice okay what else?**

R: eat noodle for breakfast.

**I: ok what else?**

R: breadfruits

**I: okay what else?**

R: and banana

**I: now how do you prepare the foods? As of breadfruits, how do you prepare the breadfruits meal? How do you prepare the breadfruit meal for the family?**

R: *Konij* or cook it on the fire. I roll it over on the fire and then when I know the breadfruits is cook enough, then I would take it out and clean it then we have our breadfruits we called “Konjin”.

**I: any other ways that you cook your breadfruits meal?**

R: I steam it.

**I: any other ways?**

R: I cut it in many pieces and boil it with water and add coconut milk.

**I: any other?**

R: “Jokkep” We grate the breadfruit then boil it again and make it real soft then mix the food with coconut milk.

**I: and what about the banana, how do you cook the banana?**

R: I boiled it

**I: only boiled?**

R: I also make banana balls * banana meals that shape like balls*

**I: ok. Now can you tell me who in the family is served first, next and last?**

R: the elder people serve first then the children would be next.

**I: the adult serve first then the children serve next?**

R: Yes?

**I: is there any differences in the foods served to different family members?**

R: there is no differences

**I: Is there is any differences in quantities of food served to different family members?**

R: yes

**I: can you tell me why?**

R: because the adult eat more than the children we give more food for the adult ones than the children

**I: and why do you give more foods for the adults and give little quantity of foods for the children?**

R: because adult people eat more than the children when we give more foods, they can’t finish their foods.

**I: do some children receive more food than others?**

R: yes

**I: yes and why?**

R: some children eat more than the others.

**I: Now could you describe any food sharing between family members during mealtimes (for example children eating together separately from the family, meals eaten from the same plate by all family members?**

R: they have separate plate. Children have their own plate while the adult ones have their own separate plates.

**I: ok. Do your family share foods between household and your neighbour?**

R: yes we do share foods with our neighbours or between household.

**I: you share the foods like sometimes or all the time?**

R: all the time I share one plate of our meal for them

**I: Now I want to know about how young children eat in this community. Can you describe in detail what children under two years old commonly eat throughout the day?**

R: they commonly eat noodle, crackers, and soft food like soften breadfruits meal and rice

**I: How many times a day meals are eaten by children under two years old?**

R: hours between hours

**I: can you explain more on meals eaten hours between hours?**

R: they eat their breakfast then hours later they would also eat because they are hungry. Then during lunch time, they eat their lunch and hours later, they eat again.

**I: so you meant that they eat breakfast, lunch and dinner and eat hours in between their regular meals.**

**I: do children typically eat their snacks between meals?**

R: yes

**I: do children fed differently when they are sick? For example when they have diarrhea? Is there any different when you feed them?**

R: yes

**I: can you explain the differences?**

R: the different is that when children do not want to eat.

**I: and why is that?**

R: because they are sick and they don’t feel like they want to eat.

**I: and what if they are not sick?**

R; if they are not sick, they eat more than they have enough.

**I: so now can you tell me any differences in feeding practices between girls and boys under two years old?**

R: there is no differences

**I: there is no?**

R: yes

**I: Can you talk to me about what influence how families feed their children in this community?**

R: no respond

**I: for you as a grandmother, what influence how you feed your grandchildren in this community?**

R: feed them in order for them to grow.

**I: We have heard from some families that eat local foods and others that eat processed foods. Could you explain what is typical for most families in this community? As of here in Arno what is typical for people here to eat typically?**

R: Rice, we usually eat rice

**I: anything else?**

R: rice and bread our seasonal foods like the local seasonal foods. When we really run out of rice and bread we eat local foods it there is any in the season.

**I: now if you have bread or rice for your meal, what meat you eat with bread and rice?**

R: if we have fish, we eat fish, can food like tuna, mackerel or any kind of can foods.

**I: Ok now what makes it difficult or easy to cook local foods? Let’s first talk about the difficult in cook local foods?**

R: when we don’t have materials to cook the foods.

**I: what kind of materials?**

R: woods to make fire

**I: ok and what else?**

R: gas propane for the stove

**I: westernize stove gas?**

R: yes

**I: these are the difficulties in cooking local foods? Any other difficulties?**

R: I can only think of these difficulties.

**I: Now what are the negative or positive things about eating local foods? Let us first talk about the positive things about local foods.**

R: what kind of foods?

**I: any kind of local foods, what are the positive things about them.**

R: they are delicious

**I: anything else aside from that**

R: I can say they are delicious because they are really fresh, we cook them and eat them while they are really fresh and healthy. We eat and have satisfy stomach with fresh foods.

**I: and what are the negative things about local foods?**

R: when we eat too much of these foods we can have diabetes

**I: what kind of local foods that cause to have diabetes?**

R: the breadfruits, it is also unnecessary for diabetic people to eat frequently because it is also contain a lot of starch in it.

**I: oh ok. Now are the good things about processed foods? Foods that are imported, what are the good things about them?**

R: processed foods?

**I: yes**

R: they are good because when they are cooked, they have good taste and we love them a lot.

**I: and what are the negative things about processed foods?**

R: because it took a long time for them to arrive here

**I: and what if it took time for them to arrive? What about that?**

R: they caused us illness. They stored for too long and not fresh compared to local foods

**I: Is there any suggestions for balanced meals that can be prepared with locally available ingredients for children under two?**

R: no respond

**I: do you put available ingredient of local foods with any other kind of foods to feed your children?**

R: yes

**I: what kind of foods you put in the available ingredient?**

R: we can use the flour together with the pumpkin and cook boil it and feed the child.

**I: anything else? Aside from pumpkin?**

R: bread

**I: bread is not a local food, are there any local foods that you put different ingredient so that you can feed your children?**

R: yes I also can make banana with flour and boil it.

**I: ok. Now can you talk about what messages about breastfeeding and complementary feeding you give to mothers or other community members?**

R: no respond

**I: was the question understandable or not?**

R: can you repeat it again?

**I: can you talk about what messages about breastfeeding and complementary feeding that you give to mothers or other community members?**

R: no respond

**I: as of a breastfeeding mother, what are some message you would give them on foods that they supposed to eat or can be anybody in this community?**

R: what like what?

**I: did you understand the question? Are there any nutrition education with community members as part of health work? No I am sorry. Not that question. Is there is better ways in supporting breastfeeding mothers or mothers to feed their children under two years old on what kind of foods they supposed to feed their children under two years? Like you as a traditional healer?**

R: yes?

**I: what would be the perfect and helpful advice you would tell your breastfeeding mother or people in the community to eat?**

R: eating pandanus and do exclusive breastfeed for the child.

**I: ok anything else?**

R: they should also eat breadfruits and eat fish a lot so that…

**I: yes?**

R: so that it can provide breastmilk.

**I: are there any difficulties to deliver nutrition messages to caregivers?**

R: no respond

**I: what are something that would make it difficult for you to deliver nutrition messages to mothers or people in the community? What are the difficult for you in delivering nutrition messages to people in your houses or your neighbours or breastfeeding mothers?**

R: there is no difficulties

**I: there is none?**

R: yes there is no difficulties.

**I: what are some specific ways that this nutrition communication could be more effective?**

R: no respond

**I: how would you make specific ways that can help nutrition communication be more effective to your traditional healing patient, or a breastfeeding mother, or just a ill person. How would you help nutrition communication be more effective and easy for them to understand?**

R: no respond

**I: how would you tell them that they should eat this and that kind of food?**

R: talk to them or give them helpful advice that they should eat foods that are good for the child’s health.

**I: alright that’s great. Now I would like to talk about pregnant women in this community. Can you describe their diets during pregnancy?**

R: some pregnancy women always crave for fish and breadfruits

**I: anything else aside from fish and breadfruits?**

R: the yellow banana

**I: do these pregnant women usually change their diets during pregnancy?**

R: yes

**I: Now what influence women’s diet during pregnancy?**

R: because they are pregnant and they always crave for different kind of foods.

**I: what kind of foods that are encouraged for women to eat during pregnancy and reasons why?**

R: no respond

**I: like what kind of food you encourage the pregnancy women to eat?**

R: foods that can help give the child good health.

**I: What kind of foods women are encouraged not to eat during pregnancy?**

R: like salt, they should not eat salt

**I: ok anything else?**

R: they usually told not to eat salt

**I: who encouraged or discourages eating those foods during pregnancy?**

R: their mothers

**I: just their mothers?**

R: and their grandmothers

**I: Can you tell me about any supplements normally given to women during pregnancy?**

R: medicine from foreign country?

**I: can be yes**

R: that they normally take during pregnancy?

**I: yes were there any supplements normally given to them during pregnancy?**

R: no answer

**I: were there any supplements given from the doctors**

R: the blood pill and the pill to provide vitamin.

**I: what prevent women from taking their supplements? Sometimes pregnant women don’t take these supplements, so what are some difficulties that prevent them from taking their given supplements?**

R: because they feel dizzy and nausea whenever they take their supplements.

**I: do women drink alcohol, smoke, and use other drugs during pregnancy?**

R: yes there are some women who take drugs or some during pregnancy, there are just few or some of them.

**I: Can you now describe women’s diet during breastfeeding in this community?**

R: they usually eat rice and fish

**I: just rice and fish, anything else?**

R: whenever they feel weak and starve, they make themselves noodle, so that it is the simplest way of cooking and do breastfeeding right away.

**I: ok. Now can you tell me if women usually change their diet during breastfeeding?**

R: yes there are some breastfeeding women that change their diet and would only want to eat fish.

**I: What kind of foods that breastfeeding women are encouraged to eat and reasons why?**

R: so that the child can live healthy

**I; yes and what kind of foods breastfeeding women re encouraged to eat?**

R: local foods

**I: like what?**

R: eating pandanus, and fish.

**I: what else?**

R: that’s all I can think of breastfeeding women to eat.

**I: Now what kind of foods that breastfeeding women are encouraged not to eat?**

R: salty foods.

**I: aside from salty foods, what else?**

R: salts and salty foods that not good for their health.

**I: who encourages or discourages eating those foods while breastfeeding?**

R: their mothers and their grandmothers

**I: As a traditional healer, what are some of your biggest concerns of the diets of pregnant and breastfeeding women in this community you work in?**

R: eating lime with salt.

**I: anything else?**

R: these are the common foods for pregnant women here in this community, lime and salt.

**I: Now for the last section, we would like to learn about ways we can develop health programs in this community. Can you explain where community members usually get trusted information about nutrition and health?**

R: from the doctors

**I: anyone else aside from the doctors?**

R: from the parents and our grandparents

**I: what are some reasons why these sources are trusted?**

R: trust their grandparents?

**I: yes why do pregnant women trust they get these sources from?**

R: because they are adults and they have better experiences

**I: Now do you think nutrition and health messages should be delivered so that community members would see or her them most easily?**

R: no respond

I: nutrition and health messages, where do you thing would be the easy way you can hear or see these messages most easily?

R: no respond

**I: this question is about information we talked about like breastfeeding, pregnancy, nutrition and health, where do you think would be the easiest way that people in this community can hear or see these messages most easily?**

R: from the health care centre

**I: anywhere else?**

R: from the governors

**I: and what about hear them most easily?**

R: from the radio station

**I: ok and what types of media that community members use the most to communicate?**

R: cell phones

**I: cell phones, ok anything else?**

R: CB radio

**I: ok what else?**

R: any radios that we use to communicate

**I: For our last question, could you describe what influences how people raise children in this community?**

R: no respond

**I: what influence someone to raise children’s health or the growth of the child in this community?**

R: no respond

**I: did you understand the question?**

R: no

**I: it says, what influence you as a mother or a grandmothers to raise children in this community?**

R: grandmothers

**I: yes what about our grandmothers**

R: they influence us to take good care of our grandchildren

**I: ok. Now are there any information that pregnant or breastfeeding women typically ask for from you as a traditional healer?**

R: the mothers of the child?

**I: yes, the mother or mothers in this community, are there any specific advice or information related to parenting typically given for them?**

R: yes

**I: like what?**

R: That they should take care of their children from swimming in the ocean and drowning.

**I: what else?**

R: goes to unknown places where they can be caused any serious problem for themselves. Parents should always be there for their children in case of emergency and danger for the child.

**I: thank you. You have very good answers. Now are there any information that pregnant or breastfeeding women typically ask for from you as a tradition healer? When your local bathing medicine or stomach massaging for the children and their mothers, do these women typically ask from you as a traditional healer?**

R: yes

**I: what do they usually asked from you?**

R: anything that has to do with their children’s healing medicines or traditional local healing stuff.

**I: what do you think would be the best way to communicate with caregivers about health?**

R: again?

**I: what would be the best way to communicate with caregivers about health? As caregivers raise their children, what would be some ways that you would communicate with them about health?**

R: watched over their children so that they can’t be in danger

**I: ok so you said they should be really taken good care or their children**

R: yes they should be serious in taking good care of raising their children

**I: Is there anything else about the topics we talked about today that we missed or that you would like to tell us about?**

R: there is nothing else

**I: nothing else? Okay we are done now. Thank you so much for sharing your helpful information. Thank you once again.**
