# Supplementary material for: Conceptualizing multi-level determinants of infant and young child nutrition in the Republic of Marshall Islands–a socio-ecological perspective
Source: PLOS Glob Public Health. 2022 Dec 19;2(12):e0001343. doi: 10.1371/journal.pgph.0001343 (PMC10022247; doi:10.1371/journal.pgph.0001343)
Supplement: S1 Data — (ZIP) [file pgph.0001343.s001.zip › RMI Supp Data/Interviews data/I37R_IDI_FCG_ArnoSep 13_Meia.docx]

- **Interview code: I37R**
- **Interview type and interviewee: IDI FCG**
- **Interview date: SEPT 13,2018**
- **Location: Arno**
- **Interviewer: Mei**
- **Transcriber: Shante**

**I: ok. Before we do the interview. Do you agree to take part in this afternoon?**

R: yes

**I: Ok. Thank you for giving me this beautiful time for us to discuss with each other. The information you give we will learn about it and it will help to make a better life for mother and children. and for our islands. For m e to start. Can you tell me a little bit about your family? Can you tell me a little story about your family? Like who lives in your house and, you don’t need to say names because we don’t need names, but you can just tell me who lives in your house and how many are there.**

R: On how many family members I have?

**I: yes. And who lives in your house.**

R: my total family members is 4

**I: 4 only?**

R: yes 4 only, we live with the father of my husband.

**I: now we live here?**

R: me and my husband and our kids and his mother because his dad is gone, he’s at Majuro, and his sister’s kids and their brother. Just us.

**I: Now how many people is in your house?**

R: me, my husband, the old lady, Kabe, Robert, Selana, Bryan, Racheal…11

**I: 11 of you guys in total… now how many children?**

R: kids. Kabe, Robert, Salena, racheal, melina, Ronny, Bryan, kijen… 9.

**I: 9 kids and 3 adults?**

R: 3

**I: now can you tell me how many boys and girls?**

R: 2 girls and 7 boys.

**I: 7 boys?**

R: hmm.

**I: now I want to know how old they are.**

R: well I don’t really know what the age of the other kids is…

**I: What about the age of your kids?**

R: our oldest child is 8 years and in grade 3. Her brother, when October he will turn 4. And my youngest is 1 year old.

**I: he’s one years old? Oh ok. Thank you. Now can you tell me about your community that you live in?**

R: like say what? About this area?

**I: the community. What’s happening in this community? What are the good and bad about this community?**

R: well. It’s good here where we stay, it’s not that bad… it’s not bad because we are ok.

**I: yes. But can you tell me why it’s good? When you say it’s good, it’s good why?**

R: it’s good because, like because of the area is wide and clear for us to look around.

**I: what else that you see that is good that you see in this community in our island? Because this is an island where we are. What are the good in this island?**

R: it’s good because… what?

**I: about how you live and…**

R: Oh. How we live is we make amimano. (handcrafts decorations/many more). Coconut. Like that.

**I:is there any activities you guys do for this community?**

R: Yes. There’s time we play Volleyball and play on the roads.

**I: What about for kids?**

R: well, little kids…

**I: is there anything good in this community for the little kids? Like for example, Majuro, we would say the bad thing about it is near the road because of the cars that goes by. And if that house had seawall, the child might fall down from the seawall. Now can you tell me the good thing about the community in this island for the children?**

R: it’s good because they there’s no any dangerous things near them. When they play on the road, we don’t really mind because there’s barely any cars around here. They mostly play near this area.

**I: on how they are together. do each children, are there any other good things? When they are together, like for me, if I was new to one place and my child wouldn’t want to play because he will be scared and stuff like that. What about the kids here?**

R: well, I think… picnic and stuff like that?

**I: hmm.**

R: well, if picnic… we go to the picnic place and the kids play at. And the program that they have for kids, the go from here to INE and play with the kids there. That’s how they…

**I: is there, and you guys make these programs like every year or every time?**

R: not all the time. Barely.

**I: barely.**

R: like they usually do it when it’s a special day for the islands.

**I: is there any bad things that happens here in this community? The way you see it, is there any thing you see bad about this community that needs to be improve for the kids. Is there anything that there should be for the kids?**

R: dangerous things?

**I: No. what are the dangerous, like for me, my place was, if it was, I would say, oh the bad thing in this community is…it’s like…like what… not enough money to , not really lot’s of money to bring like, the government need to bring like things to play with because it’s good for the kids. The women need to make little programs for the little kids. Stuff like that. Is there anything bad in this community that you don’t agree with in this community or your island?**

R: when the kids go around and play, like we don’t know…

**I: Oh. Because they play, and you don’t know what is bad and everything is good…**

R: yeah, when they play and come back, we don’t see…

**I: Oh ok. Thank you. Now let’s, let’s, we will now talk about health and illnesses in your family. Can you tell me about some of the illnesses that your child have suffered from? Like we talked about it on our free list, can you tell me what are the illnesses to the record, the illnesses that we usually get?**

R: children in this community?

**I: yes. And your child. Children in this community or your kids.**

R: I already said it before.

**I: kids under the age of 2.**

R: oh. Like fever and cough…

**I: now you said fever, so. Fever cough and asthma and headache, dizziness, pink eyes, diarrhea, and rash and pink eye in the dark. Now that’s all the things we learned about on the paper that we are going to talk about them. The things that you mentioned. Now I want to know about the illness that you mentioned. Now what makes the child gets fever? You can tell me what your thinking about what makes the child get sick. Make your voice heard in that. What makes the child get fever?**

R: when we… what… get them cold.

**I: yeah.**

R: when we keep them cold they start to get a fever and cough and those kinds of illnesses.

**I: now, and you said when they also play in the rain.**

R: when it rains and get’s cold.

**I: what about, on how you see it, is fever dangerous?**

R: yeah.

**I: can you tell me why you said fever is dangerous? Can you explain?**

R: when they have fever for a long time, their body get’s dry, and they don’t feel good, they sleep because it’s like they are weak, they can’t handle the fever.

**I: yes. Now how do you prevent fever?**

R: I give them medicines.

**I: traditional Medicines or you go to the hospital?**

R: the doctor.

**I: the doctor. Ok. Now when you go to the doctors, do you pay fee’s?**

R: yes.

**I: they have fee’s? how much does it cost?**

R: 25cents.

**I: 25cents?**

R: hmm

**I: oh. Ok. Thank you. Now, every time you go to the doctors, but where do you go to the doctors? Is there any doctors here at matland where we are, is there any hospital for this community or do you go over there to see the doctors?**

R: you know here doesn’t have any hospital. We only go to INE.

**I:so, how do you go to INE to see the doctor?**

R: there’s bicycle, bicycle and pick up.

**I: you take the child and put him in the car and go…**

R: if the diesel is busy then I would use the bicycle.

**I: yes. Now every time you bring your child to the hospital is you use the bicycle or the pick up.**

R: yes, bicycles or pick ups.

**I: ok. And you pay 25cents for seeing the doctor.**

R: yes.

**I: Ok. Thank you. Now. We will go down to coughing. Can you tell me how they get the coughs?**

R: maybe because too much fever. when their fever is long they might cough. Like. They cough and get runny nose.

**I: Now, the way you see it, do you think coughing is dangerous?**

R: when they cough a lot, they can have bruises inside their throat when they cough for a long time. Coughing can make their throat hurt and when cough and runny nose can make them have bruises inside their nose.

**I: yeah…**

R: sometimes when they cough for a long time, they will vomit.

**I: do you think the child can die from coughing?**

R: they can have asthma too, when they cough they get asthma

**I: when they cough, they will have asthma, and that will be the time it will be difficult for the child… ok thank you. Now how do you prevent coughing from the child or how do you make the child not to cough?**

R: I go to the hospital and bring her to the doctor.

**I: Ok. Good. For the child not to cough, is there something you could do for the child not to cough before he gets it? Like… what made the child cough, fever. now what can you do to make sure the child wouldn’t get cough and fever? is there anything other then medicines? Like there’s a reason why the child get fever and make the child cough because if put him where is cold.is there anything you should do to make the child not sick?**

R: maybe prevent the…

**I: Yes, prevent the child from getting the child fever and coughing. What can you do before…**

R: hold him from where it’s cold.

**I: hold him from where is cold, ok. Good. Thank you. Now about the asthma you said, what makes the child gets asthma?**

R: from coughing. When they cough, they get asthma.

**I: and you said, when they have asthma, it’s dangerous and they might die from it because they’ll run out of breath. Now what prevents coughing?**

R: we get them medicines, bring them to the doctors.

**I: bring them medicines…good. What about the headache?**

R: we also bring them to the doctors.

**I: you bring them to the doctors?**

R: hmmm…

**I: what make the child gets a headache?**

R: I don’t know, maybe because they stay under the sun or what. At the, or maybe because when they are sick. They get hurt from the sickness they get.

**I: so, when they sick it makes them have headaches?**

R: yes.

**I: good. Do you think headaches is a serious illness?**

R: yes. I think so, because if they have a headache, they won’t be happy to eat and drink, they won’t want to move around because they have a headache. They only lay down.

**I: when the child doesn’t want to eat and drink it will make them get skinny and weak and it’s dangerous. Now to prevent headache, what do you do?**

R: give her medicine for her pain. Like when I finish bringing her to the doctor, then I also give her medicines.

**I: from hospital?**

R: hmm.

**I: now we reached illness number 4, we have 4 more illness to talk about. What made the child feel dizzy?**

R: maybe because from when he is sick.

**I: gets it when sick?**

R: yes.

**I: sick like what?**

R: maybe from headache and pain stomach. When his stomach hurts they can get dizzy.

**I: yeah, when they have stomach pain they can get dizzy. Now do you think dizziness is dangerous?**

R: when they vomit, maybe not really because once they have it, it goes away.

**I: you said, not often because your fast to bring hm to the doctor to get him meds. Now you said, I want to know, when you give the meds to the child in he doesn’t get better is there any other things you do to the child?**

R: yes.

**I: yes, ok. Can you give me in detail about what you do when the child gets better?**

R: I bring them to the ones that knows how to massage their stomach when they have a stomach bump. They usually see if their stomach has a bump and then massage it.

**I: do you pay the traditional healer?**

R: No. I don’t.

**I: you don’t pay for the traditional healer… ok. So, it’s free from you going to the doctors. now pink eyes, dirty pink eyes. What makes a child gets pink eyes?**

R: pink eyes they usually get it from where they play because it’s really dusty, maybe from when they get sweaty and they touch their eyes, but their hands are dirty. Maybe that’s why they get pink eyes, because their hands are dirty, but they rub their eyes with their hands.

**I: is there any thing else that cause pink eyes?**

R: well I don’t think…

**I: do you think it can spread?**

R: yes

**I: yeah, because people can get it from others. Is there anything other then spread and dirty?**

R: when gets pink eyes?

**I: hmm**

R: when he gets sick, like what would I say, if it’s like dirty and get pink eyes, it’ll make other people get pink eyes.

**I: now, the way you see it, do you think pink eyes is a serious illness?**

R: well, it will also be serious because it will hurt the child’s eye.

**I: like hurt how?**

R: like bruise and… when we,

**I: so, when it gets bruise, what will be the effect of it? What will make him see?**

R: doesn’t get pink eyes?

**I: No. like…**

R**:** Like pink eyes that’ll get effected?

**I: yeah.**

R: because they get pink eyes and they’ll get like scratch like near their eyes.

**I: is there anything that pink eyes will affect the life of a person?**

R: yes. Maybe because when the eyes get hurts, it get affected by his brain, maybe it affect their head.

**I: so, your understanding says, when he cant’s affection and pink eyes and hurts upward to the brain, It makes the head hurts.**

R: when their eyes are hurt, and they look around, they won’t barely look around because their eyes hurts.

**I: we want to know when he has pink eyes, what do you do to make sure he won’t get pink eyes or how do you prevent him from pink eyes?**

R: I bring him to the hospital to look for medicine for him…

**I: when you give him medicine, Is there anything else you give him?**

R: protect him from having pink eyes…

**I: so, how would you protect the child from getting pink eyes?**

R: probably I will clean his hands and face. Soap their face like around here so that…

**I: Ok. Good. Thank you. Now diarrhea. We reached diarrhea, now we want to know what makes the child gets diarrhea?**

R: it comes from the food that are not good.

**I: not good like?**

R: like if we don’t put the food in the containers and it can still be hot but we put them in and they don’t clean their hands and that’s why it makes the kids have diarrhea.

**I: so, you mention that you put hot food inside containers, can you explain that?**

R: when we put them inside the containers?

**I: No, when you said, if you don’t put them inside the containers it will make the food?**

R: Have flies in them.

**I: Ok. Do you think diarrhea is a serious illness?**

R: yes. If children get diarrhea, it takes long for them to…

**I: hmm… what are the issues? When they get diarrhea, what affects them?**

R: Stomach ache, headache and dizziness.

**I: do they eat?**

R: No, they don’t eat.

**I: now if they don’t eat, their body…**

R: get’s weak.

**I: Ok. What makes their diarrhea stops?**

R: bring them to the doctors.

**I: you make them drink?**

R: make them drink American medicines.

**I: now let’s get down to korkorri (skin rash) what makes them get korkorri?**

R: when we don’t clean them, bath them, and them playing on the dirt. What else would I say… the korkorri also comes from not enough food with no nutrition, that’s also how they got them.

**I: is korkorri a serious illness?**

R: probably a serious illness because it affects their body.

**I: it affects their skin… now what prevents getting korkorri? What will prevent a child from getting korkorri? Like, what would make a child not to get korkorri?**

R: clean them, clean there sleeping things and maybe they need to eat more healthy foods like meat so that…

**I: what kind meat? And what kind food? Can you give me in detail?**

R: fish and you know the Marshallese foods like pandanus and pumpkin.

**I: what else? Is there any other more?**

R: and papaya

**I: yes. Is there any other thing you can make their korkorri gone?**

R: yes. I also bring the child to the doctor so that they can give me medicines so that I can give it to my child.

**I: Now the last sickness. Now I want you to tell me, about blind at night, what make a child or children get blind at night? From your own point of view.**

R: it also comes from not enough food, like we don’t feed them enough nutrition like papaya, pumpkin, feed them fish and we let them drink more color water then just water only. I think that’s some of the issues… maybe comes from not having nutrition on their foods. It’s like we’re not serious on feeding them nutritious food.

**I: Now when a child is blind at night, what do they say about it?**

R: they usually say less nutrition.

**I: less nutrition… Ok less nutrition in is food?**

R: Yeah, we barely give them planted food.

**I: Now the child had this illness since he was young, or he just got it now?**

R: maybe he just starts getting it.

**I: yeah because now that he starting to know how to talk they…**

R: they just found out that…

**I: the child is blind at night.**

R: we just found that that he’s blind at night. Like we see that the child doesn’t go to the dark.

**I: the dark because…**

R: he can’t see in the dark.

**I: Now, do you think blind at night is a serious illness for the health of the child?**

R: it’s bad because when he is blind at night, his eyes are always looking around…

**I: Now we say because not enough Nutrition and it affects the health of the child…**

R: on how he has less nutrition?

**I: yes… what will it make for the child not to be blind at night.?**

R: I think we could give the child vitamin to drink, feed him papaya and I think when we are blind at night we usually eat papaya.

**I: papaya?**

R: yeah, makes us not to drink more sweets like sugar, less drink sugar and eat lots of papaya and other planted foods.

**I: planted foods? Ok… now can you tell me how, we are going to come down now. Can you tell me how you know that your child needs to see the doctor when he is sick?**

R: on how the child needs to see the doctor?

**I: yes. How would you know when you need to bring your child to the doctor? You can just tell me your own understanding, like on how you said you bring them to the hospital when they are sick, is it right, like you said it?**

R: yeah.

**I: Ok. can you make your voice heard so that they’ll hear you…**

R: on how we bring them to see the doctors?

**I: yes.**

R: well. You know when the child is sick, I bring them to the hospital, maybe the time when we see the doctors, it’s the time when we bring our children when they are sick.

**I: Ok. now this asks. Who do you first bring your child too when he is sick? Like you know some Marshallese, they bring their child to, there is… do you usually bring your child to a traditional healer? Is there any time that you bring your child to a traditional healer?**

R: when I’m done bringing them to the doctors…

**I: so, you would say, you bring the child to the doctor first before the traditional healer?**

R: yeah, I usually take them…

**I: and when your done with the doctor then you go to the traditional healer if they’re not cured with hospital meds?**

R: yeah.

**I: for what?**

R: your talking about illnesses like fever and…yeah. when we see that they take so long to get better like for fever and coughing. When we bring them to the traditional healers, they would check them and say, the child has a stomach bump and that’s why it takes long for them to have fever and cough.

**I: ok. can you give me in detail what kind illnesses that affects your child from the nutritious food they eat? is there any illnesses that you child gets from eating nutrition foods?**

R: Nutrition like what?

**I: any kind.**

R: when they eat these foods? Right?

**I: yeah**

R: I don’t think, they don’t get sick.

**I: what about what kind of illnesses that the child will get from not eating nutrition?**

R: When they don’t eat nutrition food they get blind at night…

**I: yeah…**

R: when they get blind at night they can also get skin rash from less nutrition.

**I: now when they have less nutrition in their foods they can get skin rash and blind at night. (like don’t have a good vision at night.) could you describe for me a typical day of someone living a healthy lifestyle, from the time they wake up in the morning until they go to bed? Can you explain how a healthy person acts in a day? From the day he wakes up until the time he sleeps?**

R: well, if for someone that it’s healthy, they usually wake up at probably 6 and 7 o’clock morning. And once they wake up they work. They work, there’s time when they rest, when they sleep.

**I: when they wake up, what kind of work do they do throughout the whole day? What do they do all day, like give me in full detail?**

R: morning they wake up and… like us. We wake up and make breakfast, after breakfast we now go to our kids and after we pick up trash and clean. Clean the areas.

**I: What else?**

R: maybe when we work like bring coconuts and pick up trash and bring coconut and… that’s the work we usually do in a day. Clean near our houses bring coconut and before you know it, it gets dark.

**I: Now before you sleep, what do you do?**

R: before we sleep?... like eat before we sleep.

**I: what is the sign of a healthy 2 years old child? You explain how he acts from when he wakes up to until he goes to bad. How the child, you explain…**

R: on how they…

**I: On what they do activities that’s shows that he is living healthy…**

R: when he plays around, he doesn’t cry a lot and don’t like… just play around.

**I: what about on how they eat?**

R: eat before they go to play, when it’s time for them to eat lunch they eat.

**I: is there anything that the child doesn’t like?**

R: on what?

**I: on how good he’s feeling. does he hate foods?**

R: no.

**I: No? ok. what signs are there that shows that an adult is feeling good? So, like If a grown up is awake and is feeing good. What are the signs that you’ll see that an adult is feeling great?**

R: well, for the grown ups, when we know that they are feeling better is when they are not laying down but work only. They work and it’s good for them to move around. Like moving around, they’re strong. It’s good for them to move around.

**I: Ok. we will now go down to our next question on how to wash your hands. Could you tell me how your family wash their hands? Can you tell me a story about hand washing? On how families wash their hands in a day**.

R: when we do chores and stuff, and when we see that our hands are dirty we wash them. And when we eat we wash them. We usually wash our hands after we touch something dirty before we eat.

**I: because when we interviewed you mention diarrhea, sometimes they get diarrhea and sometimes…**

R: sometimes they touch dirty things…

**I: Now, how do they wash their hands in a day? Do they wash their hands everyday or sometimes?**

R: sometimes.

**I: sometimes? So, why do you sometimes wash your hands or sometimes for the child to wash their hands?**

R: there comes a time when we are busy, and we didn’t see the children touch their foods, but their hands are dirty and touching their mouths. Sometimes those affects diarrhea.

**I: ok. sometimes when… I’ve seen this before. Sometimes when we don’t watch over a child, sometimes they can eat what’s on the ground.**

R: yeah, they can eat from the grounds too

**I: it may not come just from the food they eat but from what they eat on the ground. They can just take a food from the ground not knowing is dirty but just eats it. Now children that watch their hands in a day, can you describe how they wash their hands in da day?**

R: when they wash their hands…the kids in my house usually wash their hands, when they play and wash their hands, they don’t usually wash their hands after they play but just when they are going to eat.

**I: do they wash their hands everyday?**

R: I think some kids do wash their hands everyday.

**I: now kids below 2 years, do they wash their hands? How do they wash their hands in a day? Kids that are 1 years old.**

R: well I don’t know. Usually it’s us that wash their hands.

**I: Now do you wash their hands everyday?**

R: sometimes we forget to wash their hands. When we don’t wash their hands then we feed them.

**I: ok. now that you said you usually forgets. can you tell me why you usually forget? Sorry if I ask too much.**

R: because I’m usually busy because there’s a lot of chores in front of me and I don’t really think about the kids because I’m so focus on doing the chores.

**I: yeah. that’s how it is. We ladies have a lot of things to do because we are the only ones who handles everything. Now can you tell me how you use soap in a day? Or do you guys wash your hands with soap?**

R: yes. like I said they we wash out hands before eating and…

**I: like how much soap do they use to wash their hands and how long do does it take to wash their hands with it?**

R: Like 3 minutes…

**I: 3 minutes you wash their hands?**

R: yes. They usually wash their hands and their finger nails. But maybe only when they are going to eat and during the time they play, comes back from playing, like maybe before they watch their hands, but we don’t see them wash their hands except when it’s time for them to eat they wash their hands with soap.

**I: Now ca you tell me the difference between washing your hands with water and with soap and water?**

R: what’s the difference? Yeah, the difference is when we wash our hands with soap and it turns to color brow to white, shows that its clean and we see it clearly.

**I: hmm. But when we wash with water?**

R: it’s clean too but not as much as using the soap.

**I: Ok. when you don’t… what prevents you from not washing your hands with soap?**

R: we umm…

**I: I said, what prevents you from nit washing your hands with soap? Because you use hand sanitizer?**

R: well, yeah. when we don’t use soap we just crap the thing and wash our hands with it.

**I: oh. So that’s why you don’t wash your hands with soap?**

R: but there’s time that we are not really dirty, and we just use soap to wash our hands.

**I: is there any other things that prevents you from not washing you hands with soap everyday?**

R: we usually use soap to wash our hands and clean them.

**I: we will now talk about the foods you ate during your pregnancy and breastfeeding. Can you look back to the time when you were pregnant, can you tell me what kinds of food you used to eat during your pregnancy and when you were not pregnant?**

R: during the time I wasn’t pregnant, I ate every food. I eat any kinds of foods and I drink all kinds of drinks.

**I: Ok. can you tell me all the foods you ate?**

R: Like, the foods we usually eat. Rice because we usually eat it. Rice and bread. Drink coconut juice and water and those other drinks.

**I: what about the time you were pregnant?**

R: I usually eat breadfruit. During the time I was pregnant I eat breadfruit.

**I: oh… is there anything you eat other then breadfruit.**

R: I think that’s the only think I ate when I was pregnant. I don’t usually eat fish, crap, pandanus and coconut juice.

**I: so, you only eat breadfruit when you were pregnant?**

R: yes.

**I: everyday? But when there is no breadfruit, what do you eat?**

R: well only a little bit of breadfruit, not like breadfruit because I eat too much breadfruit. When there is no breadfruit I eat rice.

**I: what made you want to eat those food during the time you were pregnant?**

R: I usually drink pregnancy pills. Like vitamin.

**I: now when you drink those pills. You only wanted to eat breadfruit?**

R: When I drink the pills it’s like I don’t hate many foods anymore.

**I: Oh. so, when you drink the pills you will want to eat other foods?**

R: yes. I will want to eat other foods.

**I: the pills help you want to eat other foods.**

R: hmm…

**I: Can you tell me why you wanted to eat breadfruit?**

R: the baby wanted to eat. (both laughing) that’s the only reason.

**I: what kinds of food you didn’t want to eat during your pregnancy?**

R: the usual. Pandanus and rice. The food that I usually eat when I wasn’t pregnant. Except the foods I ate when I was pregnant. I hated them.

**I: why did you hate the foods?**

R: I don’t like them the way I look at them, but when I eat them I vomit them out.

**I: Now why didn’t like them?**

R: because I always vomit them out. That’s why I don’t eat them. Because when I eat them I vomit them out.

**I: oh ok. Now can you tell me what kind of foods they wanted you to eat during your pregnancy?**

R: what kind of foods?

**I: yeah. what kind of food did they wanted you to eat during your pregnancy and who told you? and why did they wanted you to eat it?**

R: any kind foods?

**I: yes. Any kind food that they wanted you to eat and why they told you to eat the foods your suppose to eat?**

R: I don’t think there is. They only ask me if it’s there anything I want to eat.

**I: Oh. they only ask what you want to eat?**

R: yes.

**I: what do they don’t want you to eat?**

R: like salt fish and…

**I: what else?**

R: foods that are spicy.

**I: now here’s the question. why didn’t they want you to eat spicy foods salt and lime?**

R: make us have less blood (anemia) and we can have hypertension and it will cause the child to have a problem. They don’t want me to eat salty foods because they say I can have less blood.

**I: Now, when you eat salt, lime and other spicy foods, they say the child will have anemia? Who told these words?**

R: My family. Mom and dad.

**I: do you usually go to the doctors?**

R: my husband side of the family and… yes, I usually go to the doctors.

**I: do the doctors give you words of advice? Is there anything they tell you or?**

R: yes. They give us our supplements and they give us advice on how important we should take them.

**I: Ok. so, who helped you during your pregnancy? During all the time of your pregnancy? Who took care of you?**

R: My family.

**I: how did your family take care of you? what did they do to you?**

R: they give me the good things, they see what good things I wants, and they give it to me. Like for example drinks. And the foods that I tell them they bring them. They make sure I drink my pills.

**I: Now. Can you tell me what kinds of supplements you took during your pregnancy?**

R: supplements for pregnant women. The red ones.

**I: how many pills did you take?**

R: I drink them until I gave birth.

**I: No. like how many pills did you take?**

R: 2.

**I: Now. Did they tell you what the supplements was for?**

R: to help us.

**I: that medicine and the red medicines?**

R: hmm

**I: Now. Did you drink all your medicines?**

R: yes.

**I: you had no problems on drinking them?**

R: No.

**I: Did you drink any alcohol or smoke or take anything strong during your pregnancy?**

R: I didn’t.

**I: is there any traditional medicines you took during your pregnancy?**

R: no

**I: all the time during your pregnancy you didn’t use traditional medicines?**

R: I didn’t.

**I: Now who usually encourage you to eat during pregnancy and why? What made it so hard for you to eat those foods?**

R: like what?

**I: on how you take local foods, if there was a person telling you to eat local foods because it’s good for your pregnancy, is there any issue on how to get those local foods?**

R: maybe because I hated them.

**I: oh. you hated them? That’s why you don’t like local foods? but anyways there’s always local foods here.**

R: hmm… because you can eat.

**I: but like planted foods like…**

R: like orange and that kind of stuffs?

**I: yeah.**

R: well I don’t usually say I want to eat those kinds.

**I: oh. but is there anyone that usually tells you to eat things like that? Is there anyone who has ever told you to eat foods like that and what will make you take those foods? like would eat those planted foods. any kind planted food. It could not be apple and orange but other planted foods. planted trees are also like pandanus and papaya and bananas**

R: I don’t think anybody has ever told me to eat those things.

**I: you don’t usually eat them?**

R: well I usually eat when I’m not pregnant, but I don’t usually eat them.

**I: now why don’t you want to eat local foods? oh. sorry not local foods. why don’t you want to eat planted foods?**

R: I don’t know, maybe the only the foods I crave for I eat.

**I: Now. Can you tell me about the foods you eat when you breastfeed, can you tell me about what you ate during the time you were breastfeeding?**

R: I usually eat fish.

**I: what else?**

R: can foods.

**I: can foods like what?**

R: usually Mackerel and tuna.

**I: mackerel and tuna. Ok**

R: and rice and those other kinds.

**I: what makes you eat those foods during the time you were breastfeeding? Why did you eat those foods during the time you were breastfeeding?**

R: I eat them because… how do they say it… we tap our baby. (like eat a lot to make our breast full so that the child can have lots of milk to drink to make him bigger.) fill up our breastmilk.

**I: Ok. these foods are the foods to fill up our breastmilk. Now what kind of foods they told you to eat during the time you were breastfeeding?**

R: they told me to eat lots of fish, eat… they told me to eat lots of foods so that I can have lots of milk for the child.

**I: what kinds of food they told you not to eat during the time you were breastfeeding?**

R: those kinds of food that aren’t good for the kids. Like when the child just got born the foods aren’t good for them.

**I: can you give me in detail about the foods they told you not to eat that aren’t ok for you to eat?**

R: foods that been there for awhile.

**I: for the foods that’s been there for awhile, why do they tell you not to eat them?**

R: they tell me not to eat the foods that’s been there for a while because it might affect the child and might make the child to have a stomach ach. Like diarrhea.

**I: yeah, because when you will breastfeed the child he might have a stomach ach… is there any other foods that they told you not to eat? like for example poisonous fish.**

R: yeah like poisonous fish.

**I: like what kind of fish because I don’t know what the fish are called.**

R: like sahlia. (Name of a fish. idk if I spelled it right but it sounds like that.)

**I: now why do they tell you not to eat that fish?**

R: because it will be poison for the child.

**I: when he gets poison, what will happen?**

R: the child might die.

**I: oh. when you breastfeed him, when your done eating the fish and you breastfeed the child he might die from the poisonous fish you ate… what else food did they tell you to eat during the time you were breastfeeding?**

R: they told me not to eat salty foods. they told me that they will have a bump on their face and they will… they usually say not to eat salty foods because the child might have bumps on their face.

**I: I want to know who usually tells you to…**

R: usually my husbands and his parents.

**I: Ok. thank you so much… after giving birth, can you tell me how you first breastfed your child throughout the whole first day? like when we were talking earlier you said, you didn’t…**

R: I didn’t breastfeed my child till late night.

**I: can you tell me why you didn’t breastfeed your child?**

R: because he didn’t cry the whole night and I was sleeping.

**I: nobody woke you up and tell you to breastfeed the child?**

R: I don’t think so. he cried the next day, and that’s when I started breastfeeding him.

**I: did they say that there will be any problem with the child?**

R: no.

**I: just sleep? He takes a long nap. From your own knowledge, we want to know from your first breast milk, the first liquid in your breast, do you think it’s important for your child?**

R: hmm…

**I: it’s important? The first liquid in your breast?**

R: when we look at it we don’t see that its bad.

**I: like the first liquid?**

R: well the first time I had my first child, my first breastmilk liquid I throw them away because it’s like it’s bad.

**I: why?**

R: like dren waan. (the first liquid in the breast means not useful liquids,) its like milk but the color is difference.

**I: oh, and you squeezed it out? The first time you breastfed, you squeezed it out because you thought it’s not useful**?

R: yes.

**I: who told you that is not useful? From your own thought because that’s what I want to know.**

R: the midwives.

**I: the midwives… where did you gave birth?**

R: here

**I: this island. Now the midwives told you that the first liquid in your breast is not useful, so they told you to squeeze it out because why?**

R: it’s because they say it’s salty.

**I: they say it’s salty and that it’s not good for your child?**

R: hmm….

**I: is there anything that they say that will affect the child because of the first liquid?**

R: maybe because they say it’s salty and that’s why they are not healthy…

**I: the child is not healthy?**

R: hmm.

**I: Ok. good. Thank you so much. after you gave birth, how long did it take you to breastfeed your child? Oh, you said that you didn’t breastfeed him until how many days?**

R: 1

**I: because he was still sleeping?**

R: like from the morning till evening.

**I: Oh. you mean 1 whole day of you not breastfeeding?**

R: when it was around 9 o’clock he just started breastfed. and I kept on breastfeeding him until he slept.

**I: now after you gave birth, like have the child was born. Is there anything else you gave to your child? Milk or any other liquids.**

R: nothing

**I: like for our own trust, is there anything you did to your child? Did you give him medicines?**

R: Oh. just the medicines.

**I: Ok. I want to know about the medicines. Can you explain to me what kind of medicines you gave to your child, and what will it do? From our own knowledge what will the medicine do?**

R: because when we give it to the child they don’t cry anymore. diarrhea and their skin don’t turn yellow anymore.

**I: Now I would want to know what kind of medicine you make. What liquid do you give your child first? Other then your breastmilk, what do you give?**

R: the local medicines? medicines that we add with water.

**I: can you tell me what kind of medicines?**

R: medicines for kids? medicines for Kijon. (local medicines for kids so that they won’t get weak and stuff.)

**I: don’t worry about anything because we won’t say anything. What’s between us stays between us. We will transcribe them and translate them to Marshallese and give it to the ones that we work with and let them work it out. And those information’s won’t stay in the hospital, the people will take them to the states and work on it. They will take them and report them to the ones higher up. Ok. now they want to know what kind of medicines you give to your child? And what do they do to the child? Other then your breastmilk that you give to your child, what other liquids do you give to your child?**

R: just the local medicines that we usually give our child.

**I: Kijonn kan? (local medicines.)**

R: hmm…

**I: Ok, so when you use the local medicines, what do you bring and make it and what do you mix it with and do you, where does the water comes from and all those kinds. We want to know all that. Where you get the water? Where you take the medicines? what do you do to the medicines and do you wash them before you use them and how do you prepare them? You tell me in detail because they want to know.**

R: we usually make them with, you know the skin of of the coconut tree and some from the skin of a banana tree. And kiden and konut. (both local medicines)

**I: so for the skin of the coconut, what is it for? Do you mix all the medicines together or they go separately?**

R: the medicines from the coconut tree we mix it with water.

**I: just water?**

R: and kipwe in wujaj.

**I: what’s kipwe in wujaj? I don’t know what it means. Can you tell me?**

R: it’s the dust of the fire.

**I: oh… ok. now the skin of the tree mix, with water and the dust of a fire. So how do you do it? Where do you get the water?**

R: the waters that we drink.

**I: in the tanks?**

R: yeah.

**I: ok. do they boil?**

R: no

**I: you just make them? Ok. so, you bring them and mix them together and give it to the child to drink?**

R: yes. Give it to him, morning, noon and evening.

**I: morning, noon and evening, how many days?**

R: 3

**I: now the banana tree?**

R: it works for, when they have things growing on their necks.

**I: oh. when it’s… have skin rash…**

R: yeah, and we take the banana tree and add with the grass that is near the coconut tree and mix them together.

**I: and you come and pound them together?**

R: grate the banana and mix it with the medicine and squeeze it to the coconut shell.

**I: coconut shell? So now do you wash them?**

R: the grass

**I: you wash it. You only wash the grass?**

R: we don’t usually wash it. We only take the dust out from it.

**I: yeah because you don’t want to wash away the strong meds from it. You mean all that you don’t wash** it**. Just mix them together? Can you explain to me again? Sorry for disturbing you talking.**

R: I cut them and add them to a coconut shell and put them under the sun and when it froze…

**I: oh. that’s for their body?**

R: hmm.

**I: is there any meds that you give the child to drink?**

R: kiop en (local meds)

**I: ok. what was the other medicines?**

R: the kiop that we make for the child so that the color of the child would be different from the skin they were born with. We bring it and pound it and add the circle thing on the coconut tree. (it’s a medicine but they call it quarter.)

**I: what’s the circle thing on the coconut tree?**

R: yeah. the circle one.

**I: like the color green one or…**

R: the grey one. We grate it with the kiop and pound them and add water to it. And squeeze it and give a little drip to the child’s mouth.

**I: you drip it to his mouth? Ok. good. Thank you. is there any issue on how you breastfeed your child from the day he was born till he turned 6 months?**

R: is there what?

**I: any issues? On how you breastfeed your child.**

R: I don’t think there is because when I breastfeed him I don’t see any problems and buying milks and…

**I: but he still his being breastfed until now… do you think there will be a problem about you breastfeeding him until he turns 2? Do you think there will be any difficulties if he breastfed until he turns 2?**

R: the reason why I don’t stop breastfeeding is because when he get sick and doesn’t eat he will always be breastfed, my breastmilk will always helps when my child doesn’t want to eat. my breastmilk makes my child not to be weak. That’s why I only give him my breastmilk.

**I: Ok. so, it’s not an issue?**

R: No. because when he’s sick he won’t want to eat and my breastmilk covers that.

**I: can you tell me when you first feed your child food?**

R: I usually star at 6 months.

**I: 6 months? Now they want to know why you started to feed your child and gave him liquids instead of your breastmilk in that month?**

R: because the way I see it he wants to eat and wants to drink.

**I: how do you know?**

R: he points at them and look at them and when we feed him he eats right away and when I give him a drink and he drink right away.

**I: what is the thought of the people when they feed their child and give them drinks?**

R: for me when I think about it, it will make them strong when they eat and drink. When they eat and drink it’s good because it help the breastmilk make enough for them to be full.

**I: what was his first foods and how did you prepare them?**

R: when I first make them food, I made them foods that are soft.

**I: yes. but what kind of food?**

R: pandanus paste.

**I: how do you make the pandanus paste?**

R: we boil the pandanus and then I grind the paste out of it. Like if I boil it in the morning and I cool it of after and then I grind the paste out of it and then feed my child.

**I: is there anything you add to the pandanus paste?**

R: I usually make the sweet of it gone by adding a little bit of water. Mix it with water so that the sweet of it can lower down.

**I: is there anything, like for his first food?**

R: pumpkin. I boil pumpkin for him. boil it with sweet water and make it soft for him to eat.

**I: sweet water… you add sugar to it?**

R: add a little bit of sugar in the water and boils it with the pumpkins. When it’s cooked I scrambled it.

**I: and feed the child. Pandanus paste you feed him with a spoon?**

R: hmmm…

**I: Now. We want to know how people in this community eat? Can you describe what you and your family eat throughout the whole day?**

R: we usually eat rice and fish. and it’s like we’re doing good. Everyone is healthy.

**I: yes. But what kind of foods do they eat? you said rice and fish…**

R: because there’s no breadfruit and pandanus now.

**I: so, what do they eat now that there’s no pandanus and breadfruit?**

R: rice and bread.

**I: rice and bread. Drinks?**

R: some drinks water, some drinks coffee, some drink kool-aid and some drinks lime.

**I: Now we want to know who gets the foods first and who gets it last?**

R: we usually say the kids eat first.

**I: is there any difference on how you serve food to each one?**

R: for this house, we eat the same food.

**I: is there any difference on how the amount of food you serve to each one?**

R: some have small amount of food, some has enough amount of food and some has big amounts of foods.

**I: so, you mean it depends on how much they want to eat?**

R: yes.

**I: do some kids have bigger amount of food from the other kids?**

R: I don’t think so, the older kids usually eats a lot because they are hungrier then the younger kids, that’s why I give them big amount of food then the younger ones.

**I: can you explain how your family share your foods during the time you guys are eating? Like for example children eats together separately from the family meals eaten from same plate by all family members.**

R: when I make them food. kids and kids. Sometimes I usually let them have each plate and for my parents they have to same plate and the same with me and my husband.

**I: so, everyone has their own plates?**

R: hmmm

**I: so, does your family share your food to you neighbors?**

R: when we have the foods that we barely have then that’s the time we share our foods to the neighbors. But yeah, we always give them a plate of foods.

**I: we heard from some families that eat local foods whereas others eat processed foods. could you explain what is typical for your family? What food do you guys usually eats? Local foods or imported foods?**

R: we usually eat local foods like rice. When it’s not time for the local foods grow we eat imported foods.

**I: Ok. thank you. what makes it easy for you to make local foods?**

R**:** what makes it what?

**I: what makes it easy for you to make it local food? like easy for you to make local foods?**

R: on how we make breadfruit or biro (made out of breadfruit.)

**I: no… we talked about food that your family usually eats and what makes it easy for you to make local foods.**

R: maybe during the time that we eat

**I: when there is, that’s when it’s easy to make?**

R: hmmm… when it’s time for pandanus we make pandanus, like make paste out of it. When there is breadfruit then we make breadfruit. All the things that have their own time to grow we make them.

**I: what is the good and bad of local foods?**

R: local foods are good because we are healthy from it. But the bad thing about it is when they take long to grow. But it’s good, when there’s local food they last long. Because when we make biro (made from breadfruit) it will last for a year. When we also make Pandanus paste and Jakmai (juice made from the coconut tree) they also last long.

**I: oh. ok thank you. what about imported foods? what are the good things and the bad things about it?**

R: I think it’s also good because we eat them right away. and we don’t have much nutrition from them.

**I:so, that’s the bad thing about it? Not enough nutrition. But the good thing about it is fast to get it and eat it.**

R: it’s fast because we just see it and they don’t have season for it.

**I: now we are done talking about how your family eats, now I would like to learn about how your child eats. Could you describe in detail what your child under 2 years commonly eats throughout the day?**

R: eat in a day? well our children eat morning, noon and evening. Those are the time they eat.

**I: what do they eat?**

R: morning he eats bread, noon he eats rice and evening he eats rice.

**I: now how many days does he eats in a day?**

R: for my son, I usually feed him every hour. For this size he his always hungry.

**I: you mean every hour. So, when you feed him every hour, how many times does he eats in a day?**

R: I feed him in the morning then I wait 2 hours and feed him again other food and in the after noon I feed him again.

**I: do you give him snacks? You add his snacks?**

R: hmm… sometimes he doesn’t have snacks.

**I: sometimes he doesn’t? so, why does he sometimes don’t have snack?**

R: because sometimes there’s no snacks.

**I: how do you know that your child has enough to eat?**

R: I feed him and know and see that he is full, and he Is not crying.

**I: Now what do you do when your child doesn’t eat?**

R: like what?

**I: like what do you do when your child doesn’t want to eat?**

R: when the child doesn’t want to eat real food then we bring him snacks like biscuits.

**I: oh, so when you your child doesn’t want to eat you give him snacks so that he can eat?**

R: yes.

**I: Ok. what if the child doesn’t want to eat?**

R: like don’t want to eat?

**I: is there anything you do so that the child would want to eat?**

R: I don’t know because my son didn’t usually don’t want to eat.

**I: oh. is there any difference on how you feed your child when he is sick?**

R: the difference is when he is sick he eats only a little bit. But when he is not sick and he eats, then he **eats a lot.**

**I: oh. Now you told me about what your child eats under the age of two. Now can you tell me how you prepare their foods. how do you prepare and make your child foods?**

R: how we prepare them?

**I: prepare them and cook it. Can you give me in detail or tell me a story about how you prepare your child’s food? from the beginning and to the end of the time your done cooking their foods.**

R: from 2 years and below

**I: yes. The size of your baby.**

R: when I cook. Cook in the morning and feed him. then I keep the foods for later in the after noon and feed him again. Put the food in the containers and wait until he wants to eat again and then I’ll feed him again**.**

**I: when you cook them, how do you prepare them in the morning? Because the reason is… where do you get the waters from? for example, of I brought the water from the tanks. And bring it and cook it with it, or you can say I brought it and boiled it on the ground or with a propane stove or whatever you use?**

R: when I bring water, I usually fill it up from and make the fire, wash the pots and fill the up with water and make foods to the pots and put the barbeque grill on top of the fire and cook the food and after the food are cooked I feed my child.

**I: Ok. can you tell me what kind of food it’s good for your child that will make him feel good and healthy?**

R: the foods that are good for the child on this age is papaya and pumpkins.

**I: papaya and pumpkins.**

R: and feed them fish and craps.

**I: craps… now why are those foods good for your child?**

R: I think it’s better from rice, mackerel and tuna.

**I: what kind of foods you not suppose to feed your child under the age of 2? What kind of food that you not suppose to give?**

R: like food that are strong for the kids? Like what? Food like can foods, corn beef?

**I: hmmm. Can food and what else?**

R: can foods and…

**I: you said rice… rice is not good.**

R: yeah.

**I: what else? Why are the food bad for your child’s health?**

R: they won’t feel good. Like when I feed my child rice and meat, they eat but, it’s like they don’t look healthy. But when I feed my child the other food he feels healthier.

**I: is there any difference on how you feed your daughter and son? Is there any difference on how you feed them?**

R: no.

**I: thank you. your information is very good. We have 5 more questions to go? This is a very long interview but thank you so much for being patient with me. Your information is very helpful. Now. We are interested on how each member work on taking care of child. Can you tell me how the people in this community watch over their children? Like if there were lots of kids playing together can you tell me how the people in the community watch after each children?**

R: they will watch them while they are playing and when they are under the coconut tree the parents will tell them to go away from under the coconut tree because the coconut might fall under their heads.

**I: oh. so. they give them words of advice. So. Who takes responsibility for the child?**

R: their parents.

**I: their parents? Ok. can you tell me what’s the mother’s duty for her child?**

R: her duty is look after them when they roam around, like if they go to the lagoon side, the mother brings back the child and places that is dangerous for the child.

**I: how about on how they take care of them? What do they do?**

R: when we take care of them we watch over them. Watch over them when they roam around**.**

**I: yeah. like what do you do to your child? Like for example. I cook for them I wash their cloths…stuff like that. are there any other duties you do for your child?**

R: like cook and wash cloths. I think we those are the main duties for us mothers. When they are done eating we clean them.

**I: what are the father’s duties? Is there anything he helps you with?**

R: his duty is to make money to buy us food. go fishing. He does man’s work.

**I: how do you play with your child?**

R: I usually play with them.

**I: is there other ways on how you play with your child? Just play?**

R: hmm.

**I: ok could you talk about the role of grandparents have in raising children in this community?**

R: on what?

**I: on how they take care of them when they are sick and…**

R: when our kids are sick they help us. Like if the child doesn’t want to eat the grandparents will see what they can get for the child to eat and what they would want to drink. They help us by bringing what the child wants.

**I: what make the grandparents good?**

R: good on what?

**I: on anything they do.**

R: for our kids?

**I: hmm.**

R: they are good because they provide the needs of our children.

**I: good. Thank you. can you tell me about the role of other family members have in raising children in this community? is there any brother or sister they help take care of the children?**

R: me and my husband usually the ones that take care of them.

**I: do you have siblings there to help you?**

R: no, because they are usually with their husband side of the family.

**I: ok. how do the older siblings take care of their young brother and sister?**

R: good.

**I: they take good care of him**

R: yes.

**I: what do they do when, taking care of their brother?**

R: play with him, take care of him. if they have goodies they will share it to their brothers.

**I: well. You have been very helpful on your answers. Number 24 says, could you explain where you usually get your trusted information about nutrition and health from?**

R: can I say like from each club that usually comes. Kumit and hospital usually comes and tell us about it.

**I: you mean from the ministry of health. You usually get your information from the Ministry of health?**

R: hmm.

**I: can you tell me why you trust the information from the Ministry of health?**

R: I think they are good and because they help us with our kinds and help with their health. From the other one community they give us advice too.

**I: and the question says why you trust the people from the Ministry of health?**

R: because it’s a place of health. (both giggling)

**I: where would you want the information to go for you to easily see and hear about them?**

R: they should do it on the radio stations.

**I: Oh. radio. Ok. so, is there any device you use?**

R: we usually use the GB radio.

**I: Now, we are on the last question. when you think about your own parenting behaviours, can you explain what influences how you raise you children?**

R: I think there is. For me when I take care of my child, I wouldn’t want him to get hurt from the bad things he does. I teach him.

**I: what about the others? The other mother’s? they don’t teach them?**

R: well I don’t know. Maybe there are some they do.

**I: what are the thoughts of people in the community on how they raise their children’s. like for the chief and the leaders and preachers and the health workers. What is their thoughts on raising a child?**

R: maybe their thoughts they would want our kids to have manners. Know how to keep their manners. Knows how to live well.

**I: Ok. thank you. is there any word of advice you learned from raising a child?**

R: when they told me advice about our child, they told me to take good care of them.

**I: who gave you these information’s?**

R: our parents.

**I: is there any other information on raising a child that you want to know that you never knew before?**

R: I think there is but I don’t know what it is.

**I: (both laughing) but you don’t know what? (giggles) Ok. thank you. we are done. Thank you so much for your time and giving me useful information.**
