# Supplementary material for: Conceptualizing multi-level determinants of infant and young child nutrition in the Republic of Marshall Islands–a socio-ecological perspective
Source: PLOS Glob Public Health. 2022 Dec 19;2(12):e0001343. doi: 10.1371/journal.pgph.0001343 (PMC10022247; doi:10.1371/journal.pgph.0001343)
Supplement: S1 Data — (ZIP) [file pgph.0001343.s001.zip › RMI Supp Data/Interviews data/I38R_IDI_FCG_Arno_Sep 13_Libon_FelaEdited.docx]

**Interview Code: 138R**

**Interview Type: FCG**

**Interview Date: Sep.13.18**

**Location: Matelen, Arno**

**Interview: Libon**

**Transcriber: Libon**

**I: Okay before we go on would you like participate in this survey/program?**

**R: Yes**

**I: Okay. Thank you for giving me this beautiful time for me to talk with you. The information we’ll learn from you will help us find ways to improve maternal and child health and sanitation in our islands.**

**I: To begin, can you please tell me a little about your family/household?**

**R: The house we live in we have five sons and two girls. One is eighteen years of age, one fourteen, two twelve, one five, one years old and two years old.**

**I: Okay. Now can you tell me a little about this community?**

**R: Yes. I see good things about this community. There are many things we see that is good. When there are people, we see a lot of people. There are but sometimes there are not (people). And there are lot of things we see that is good in this community.**

**I: Now what do you see that is good?**

**R: We see that there are things like water for drinking, when it comes to doing work like, when it comes to doing work like making copra, we have oven (atiti) to cook coconut in this community. And it’s like it good, the way we live is good.**

**I: What are the bad things about this community?**

**R: Sometimes we see that this community is bad because when we look at it there are lot of things we want for our children cause when we’re busy and things like that and we need some things for them to keep them busy like playground or something to keep them busy so we can finish our chores.**

**I: Okay about the leaders?**

**R: Community leaders?**

**I: Yes**

**R: Well maybe the community leaders are probably**

**I: Are they good**

**R: Yes they’re good to the community and their people are also good.**

**I: Now we will talk about the health and the illnesses in this family. It says can you describe the illnesses that your child suffers from?**

**R: Yes there are many kind of illnesses these kids suffers from. They suffers from high fever, headache all these illnesses they suffer from.**

**I: High fever, headache and what else?**

**R: Nausea… All these illnesses that they usually. Like when it comes this month they get sick. Like there are months to get them.**

**I: So when you say there are months when they get it, like from what month and what do they get these illnesses?**

**R: That’s if they might say in December they might get skin rash (rajjia) or something like. And from March or January and on they get the flu.**

**I: From March and on?**

**R: Maybe from January and on.**

**I: January to March? Month of what?**

**R: Month for the flu**

**I: So when we say flu, what kind?**

**R: Illnesses such as, fever, coughing**

**I: Okay. Now when you say fever, what causes the child to have fever?**

**R: Sometimes when the child has fever it’s because of growing of the tooth or starting to crawl. Lay flat on their stomach and all these other stuff (kota)**

**I: You mean kota? What about when they have headache?**

**R: Maybe because they don’t get enough sleep, they don’t eat on time.**

**I: What about nausea?**

**R: Nausea is also related. Or maybe its also related to headache. Also when they don’t have enough food, their stomach doesn’t feel good things like that.**

**I: What about when you say from January to March they usually get the flu or illnesses such as fever, headache or nausea. Why is it that its only from these months?**

**R: Because that’s what we usually hear they say from this month or from this month to this is the month for flu, yeah they say flu.**

**I: What about December and onward?**

**R: Well that’s skin rash (rajjia) if skin rash. It’s like skin rashes starts growing on their body.**

**I: Now can we say because of the weather, like from January to March the weather is cold or what?**

**R: Maybe because of the cold weather.**

**I: What about December and onward can it come from being rainy all the times and there are plenty of muds everywhere?**

**R: It’s dirty**

**I: So you can say dirty, dirt from the mud?**

**R: The dirt from plus dirty air.**

**I: What are the seriousness of the illnesses for instant fever. What are the seriousness of having fever?**

**R: The seriousness of fever that we see is that when they have really high fever they might have seizure and things like this. But if not seizure, how would I say it, like if the fever is really high they might die. Will go into the brain and like**

**I: Mental?**

**R: It’s like they’re sick and their brain is malfunction**

**I: Oh okay. So what about headache?**

**R: Headache is like having headache for too long. Like how would I say it, our hair is about to fall out**

**I: What about children under 2 years of age when they have headache, what the seriousness of this illness for them?**

**R: The seriousness is that they’re little and we don’t know where it hurts and we’re just guessing. We’re guessing because they can’t talk and they won’t say it hurts here and there. We only know that we give them medicines.**

**I: Oh okay. So also nausea? What about the children in this community, do they usually get diarrhea?**

**R: Yes some children gets diarrhea. They usually gets diarrhea.**

**I: What about the children in this family?**

**R: Well sometimes but not always.**

**I: Okay. Now how would you prevent these illnesses from your child?**

**R: Maybe by cleaning them from places that are dirty they don’t touch bad things to get sick and also their food. Wash their hands before eating.**

**I: Now it says can you describe how you know when your child needs treatment when their sick?**

**R: We would take them first to the doctors and when we see that they’re not getting better when we take them to the doctors, then we bring them to our grandmothers or mothers so they can see what kind of illnesses they have.**

**I: Okay but how do you know that she/he need to go see the doctors?**

**R: Well because when we look at them and when they first get it they’re sick. We see that they’re sick and we take them to the doctors. Also when they get sick for too long then we bring them back and bring them to the local healer that knows local medicines.**

**I: So who do you first take child to when they’re sick and why do you take them there?**

**I: If not our mothers then our grandmothers because they might know what illness they have. Especially when you ask about it they will help you with it.**

**I: Like they know better. Now do you use local medicine?**

**R: Yes sometimes when their illness doesn’t go away for a long time then I bring them to the local healer. And when they use local medicines its then gone. Their illness goes away.**

**I: So what kind of local medicine do they use?**

**R: It’s like um what do they call it… kijon kan (bathe them so that they’re not always startle, make their body stiff or strong and also when they have hepatitis). Children’s illnesses that we use to kakijon.**

**I: Kakijon and what else?**

**R: Taking the spirit out of the child (mejatoto), messaging the stomach and illnesses that we know about.**

**I: So when they use medicine for reddish skin rash (jeba) how do they do it, do they give them local liquid or bathe them?**

**R: I usually hear or they say that they bathe them. Because I haven’t and my children haven’t done that.**

**I: Now when they bathe them do you know what kind of herbs they bathe them with? For instant kojebaba (jeba)?**

**R: I don’t really know but I usually hear them say the little rock that floats in the ocean that is red. Well that’s the one I usually hear.**

**I: Do they mix it with leaves?**

**R: They probably mix it with leaves. Maybe they mix it and bathe them with it.**

**I: Can you explain any illnesses affecting your children that are associated with nutrition? For instant, the foods are nutritious, what kind of illnesses would affect them? Do they get sick from the nutritious food that they eat?**

**R: The reason why they would get sick is because of the foods that are not nutritious to their body that it makes them hate food. Not enough food in the body.**

**I: Vitamins?**

**R: Yes not enough vitamins**

**I: What about when they eat the foods that are nutritious?**

**R: When they eat nutritious food they have vitamins, which make them healthy, healthy skin, don’t get sick all the time.**

**I: So what kind of illnesses occurs from the foods that don’t have nutrients in them? Like how you mentioned earlier when you say,” When they don’t eat foods that have nutrients they often get sick.” Like what kind of illness do they take?**

**R: the illnesses that they usually get is that they usually get the fever, not eating, skin rashes and I think these are the usually illnesses that they get.**

**I: Now we’ve talked a lot about being unhealthy. Could you describe for me a typical day of someone living a healthy life from the time they wake up in the morning until when they go to sleep?**

**R: From when they wake up till the time when they go to sleep, the reason why they’re healthy is because of their nutritious diet that makes them healthy that it makes them doesn’t feel like they don’t want to do anything. As soon as they wake up they move around. Because they have all the nutritious foods for their body.**

**I: What are the sign of a healthy child under 2 years of age?**

**R: Because they are some children that are healthy. They haven’t had their birthday yet but they’re walking, they play by themselves. Maybe because of the foods that they’re eating is nutritious that their body is stiff/strong because they move around a lot.**

**I: What are the signs of a healthy adult?**

**R: Maybe because he doesn’t just sit around but he’s always moving around. And when he’s always moving and not just sitting around it’s like he feels like moving around with his body. Because he’s always moving around and doing his chores.**

**I: He doesn’t feel lazy?**

**R: Yes he’s not lazy and doesn’t feel like he doesn’t want to.**

**I: Now let’s discuss hand washing. Could you describe in details your family’s hand washing throughout the day?**

**R: Before they what come to food and stuff? Before they eat in the morning they wash their hands and then eat. And after that they come and do their chores again and when lunch time again, they come again wash their and same goes when it’s dinner time, they wash their hands.**

**I: Are there other times when it’s important to wash hands other than before eating?**

**R: Doing chores. After doing chores we wash our hands. After using the bathroom we also wash our hands. Animals like dogs, after touching them we also wash our hands.**

**I: Good. Now it says, does the children wash their hands throughout the day?**

**R: Yes when we tell them all the time to wash their hands. When they touch something they wash their hands. If they touch something that is not clean they come and wash their hands.**

**I: Okay. What about children under 2 years old, do they wash their hands throughout the day?**

**R: Us mothers we come and wash their hands because they won’t wash their hands by themselves from the things that they touch.**

**I: From the things they touch are there any other times you wash their hands?**

**R: When they eat we also wash their hands.**

**I: It says, when do you use soap to wash your hands throughout the day?**

**R: When we’re done everything because we use everything with soap. After using the bathroom, after touching the dogs we also wash them and the animals we also soap it. After doing our chores and cooking we also come and wash our hands.**

**I: Now can you explain the difference between using only water to wash your hands and using soap and water to wash your hands? What are the differences?**

**R: There is a big difference. When we use water only we see that its not clean but when we use soap and water we know that it’s clean.**

**I: Now what’s preventing you from washing your hands with soap?**

**R: A lot. The things we wipe our hands with, we also bring them and wipe our hands with.**

**I: Yes but the question is asking, “What’s preventing you from washing your hands?” Why are there times that you don’t wash your hands with soap?**

**R: Because we’re not touching things that are not dirty. We don’t touch or we’re not cooking and touch the firewood and the things that are dirty that we use. We don’t pick up leaves. The reason why we wash our hands is because it dirty and the things that we use.**

**I: So other than that, you don’t usually wash your hands because you don’t touch dirty stuffs.**

**R: Yes**

**I: Now we would like to talk about your diet during pregnancy and breastfeeding. Now I would like you to think back to when you were pregnant. Can you describe your diet when you were pregnant compared to when you were not pregnant?**

**R: During the time when I was not pregnant I ate everything and when I was pregnant, there were some food that I didn’t eat.**

**I: Like what?**

**R: Sometimes I don’t want to eat rice or eat bread. I only wanted to eat things that are. There are something that I want to eat that are not good for the baby, like I would eat uncooked rice. It’s really good eating uncooked rice. And I would, I love to drink water and sugar. I hated drinking colors (kool-aid, luau, tea, coffee)**

**I; why did you like to eat uncooked rice and water and just sugar?**

**R: Maybe because the child changed my diet. The child change my diet from the food that I want and when inside the body it’s when our diet change.**

**I: So it changes back to? But why didn’t you eat bread or rice?**

**R: I don’t know, its like every time we try to swallow it down it can’t.**

**I: Okay. Now what encouraged you to eat these foods when you were pregnant? When you said uncooked, why did you eat uncooked rice?**

**R: The reason why we eat uncooked rice is because we want to. The child has also changed or because the things that we want is also what the child wants.**

**I: What kind of foods did they encourage you to eat during pregnancy? And why did they want you to eat these foods during pregnancy?**

**R: when our stomach was big?**

**I: Yes when you were pregnant. What kind of food did they tell you to eat?**

**R: Oh. Everything like papaya, drink coconut and things that are worth it are have vitamins like papaya, pumpkin also drink coconut and eat breadfruit.**

**I: Why did they want you to eat those things?**

**R: Because they say it can grow healthy inside or what? So that it’s worth eating because when we eat they also eat.**

**I: Okay. What kind of food we you encouraged not to eat when you were pregnant? And why did they discourage to eat these foods?**

**R: You know, stuff like salt, soy sauce, lime**

**I: Well why did they discourage you to eat and drink these things?**

**R: They say we’ll get sick. It’ll affect the baby inside me.**

**I: So who encourage or discourage you to eat these foods when you were pregnant?**

**R: Maybe the baby inside me. If I want to eat those foods, the baby doesn’t allow it.**

**I: Okay. What about your family?**

**R: They also support us in these things saying not to eat them. But we always want to eat them.**

**I: So who supported or helped you during your pregnancy... For example did they help you with your chores that you would usually do? Did they help you bring the foods that you wanted to eat?**

**R: Yes. Yes he did. He brought the things that I needed.**

**I: What else? What about your daughter, did anybody help you watch her? The older one?**

**R: Yes my family helped. They helped me watch her.**

**I: Now can you me what kind of supplements you took when you were pregnant?**

**R: Vitamins**

**I: What else?**

**R: Vitamins and the foods that I ate. Vitamins and Iron-Sulfate.**

**I: Did you take all the supplements that they gave you? *Repeat the same question.***

**R: … Yes.**

**I: Was there a time when you didn’t take the supplements?**

**R: Sometimes I don’t take them and sometimes I take them.**

**I: What about when you drink them?**

**R: I drink them. When I drink them I drink them all.**

**I: Did you drink alcohol, smoke or use other drug during pregnancy?**

**R: No I didn’t.**

**I: Were there any local medicine you took during pregnancy?**

**R: Yes because when we’re pregnant they give us local medicine.**

**I: What kind of local medicine did they give you?**

**R: Medicine for pregnancy. Medicine so that we don’t suffer when you’re pregnant.**

**I: Why did you take the medicines?**

**R: Maybe to help us and prepare us for birth so we don’t suffer (time of giving birth).**

**I: Okay good. So if you someone wanted you to eat fresh vegetable during pregnancy, could you describe what would make this difficult?**

**R: The only difficulty is that there is none. If there was but then there’s none.**

**I: What about our own fresh vegetables?**

**R: Like what?**

**I: Papaya**

**R: Ohh…**

**I: What would make it difficult to eat papaya or pandanas?**

**R: Because not all the time they’re ripe because they have season.**

**I: What makes it easy to eat fruits and vegetables?**

**R: When it’s their season and they’re ripe.**

**I: Now can you describe your diet when you were breastfeeding. What kind of food did you eat when you were breastfeeding?**

**R: When I was breastfeeding I ate everything. I ate rice, fish, and breadfruit. I ate all of these foods.**

**I: What kind of food did they want you to eat when you were breastfeeding and why did they want you to eat these foods?**

**R: Probably because to produce breast milk and to have full breast.**

**I: Now what kind of foods did they say to eat to produce breast milk?**

**R: It was important to eat fishy meat like fish.**

**I: What kind of food did they encouraged you not to eat when you were breastfeeding?**

**R: Like salt and foods that are not good because it won’t produce any milk.**

**I: So what kind of foods are those?**

**R: Like salt and foods that are greasy.**

**I: What about chicken? Did they say it was good? Corn beef or spicy foods?**

**R: They say spicy foods are bad because they’re hot.**

**I: Who encouraged or discouraged you to eat these foods when you were breastfeeding?**

**R: This old lady. This old lady that I’m with and my husband. I wouldn’t say my mother because she’s far away.**

**I: What about the doctors?**

**R: Also the doctors.**

**I: After giving birth, could you describe breastfeeding your child throughout the day?**

**R: When he wakes up I breastfeed him and goes to sleep and wakes up again and breastfeed him again.**

**I: Can you tell me a story about when you gave birth or can you tell me a story about after you gave birth? What did they do to the baby and how many hours did you wait until you breastfed him and how did you breastfeed him? Were there any difficulties in breastfeeding?**

**R: After birth or after I gave birth I breastfed him. Well maybe 20 some minutes in between I breastfed. I only lay down and breastfeed.**

**I: Why did you wait that long that it took you 20 some minutes then breastfed? And why did you lay down and breastfeed?**

**R: Because after giving birth I didn’t feel like moving around. I didn’t want to sit up and move.**

**I: Did you have C-section or normal?**

**R: Yes**

**I: you gave birth?**

**R: yes**

**I: Did you give other liquid to the baby in the first few days after birth and why?**

**R: I didn’t.**

**I: You kept on breastfeeding? Okay. Now what makes it easy or difficult to breastfeed only up to 6 months?**

**R: Like what?**

**I: Why did you give him other liquid other than your breast milk? Did you breastfeed until 6 months or what or did you double (bottle and breast milk)?**

**R: When eating, he eats with me (breastfeed).**

**I: When he eats food, he breastfeed?**

**R: Yes**

**I: But the question says, are there any difficulties in breastfeeding from birth until 6 months? Were there any difficulties in breastfeeding from birth until 6 months?**

**R: I only breastfed him from when he was born until he was 6 months.**

**I: Okay. So what made it easy just to breastfeed until 6 months?**

**R: Maybe because there weren’t enough breast milk. Or there were breast milk?**

**I: So mean when there is breast milk it easy for you to breastfeed?**

**R: Yes. When there is breast milk because that’s why he’s still breastfeeding.**

**I: So are there any difficulties or easy in just breastfeeding up to now 1 year of age and why?**

**R: There is no difficulty because he still breastfeeding up to now.**

**I: What about easy? Why is it easy to breastfeed up until now?**

**R: Because there is breast milk.**

**I: There are some women that say the first breast milk is the vitamin but some women say that its not. What about you?**

**R: After birth we breastfeed them right away.**

**I: Right away?**

**R: Yes.**

**I: Why is it important to breastfeed right away?**

**R: So that we could produce breast milk. It’s the vitamins.**

**I: So you mean the vitamins is in the first breast milk?**

**R: Yes.**

**I: Okay. So how do you know that’s the vitamin in the first breast milk?**

**R: The elders that we stay with that are used to it every day.**

**I: What about the nurses or the doctors, did they tell you that you have to breastfeed right away because those are the vitamins in the first drop of breast milk?**

**R: Yes they also said that.**

**I: So now could you tell me when you first gave foods and liquid other than the breast milk to your child? What month did you start feeding?**

**R: 6 months.**

**I: Now why did you start giving foods or liquids other than the breast milk at that month?**

**R: Because I already started feeding her and maybe the body needs liquid because she already started eating.**

**I: So you mean the body needs the food and liquid?**

**R: Yes.**

**I: Why do you say that? Are there any differences when you’re breastfeeding?**

**R: The reason I feed her is because every time I breastfeed her she keeps crying and it’s like she’s not full.**

**I: oh okay. What are the opinions from others that influenced their decision to introduce foods and drinks at that age?**

**R: Maybe because they breastfeed them but they keep crying they’re not full. It’s time for them to eat.**

**I: What about the nurses or the doctors, did they give you any information about what month the child should eat and drink?**

**R: Yes they said that.**

**I: And when they do they say what month they can eat?**

**R: 6 months.**

**I: What were the first foods given and how were they prepared?**

**R: Soft food**

**I: What kind of soft food?**

**R: Likobla (pudding)**

**I: Likobla. Now can you describe how you prepare the likobla?**

**R: I boil the water and add flour, sugar and coconut milk.**

**I: Now we want to understand how people eat in this community. Could you describe in detail what your family usually eats and drinks throughout the day?**

**R: I can just explain or do I explain this entire village. I don’t know because I’m not always with them. But still do need to explain?**

**I: Yes**

**R: Well we usually eat bread in the morning.**

**I: What kind of bread?**

**R: All kinds. Some make pancakes while others make donut.**

**I: Okay and what do they drink in the morning?**

**R: Some drink coffee others drink color like cool aid.**

**I: What about in the afternoon?**

**R: Well some eat rice, canned meat or fish each has different ways of making them.**

**I: Okay what about in the evening?**

**R: In the evening we can also make rice because that the usual and it makes you full.**

**I: Now can you explain how they prepare the foods? How the community prepare their foods? This household or this community, how do you prepare the foods?**

**R: Like how we eat at places or in this house?**

**I: How do you prepare your foods?... like how you said earlier, in the morning bread, in the afternoon rice, canned meat or fish and in the evening also rice, canned meat and fish. Now how do you prepare these foods?**

**R: We tell you how we cook them? Well the rice you cook it with water and add coconut milk put in the fire. For the fish, you bring it and boil it, fry it or roast it on a fire.**

**I: Now who in this family is served first, next and last?**

**R: In this family it’s the old lady (grandma) that is served first and then the rest of the people.**

**I: are there any differences in the foods served to different family members? If you were to serve food, are there any differences in the food you serve?**

**R: …**

**I: Everybody gets the same food or they’re all different?**

**R: oh if we prepare food? Yes everybody gets the same food to eat. If we prepare the food it’s the same.**

**I: Are there any differences in the amount of food served to different family members?**

**R: The amount?**

**I: Yes**

**R: If we serve that amount that’s that.**

**I: You mean everybody is served the same. Nobody gets served bigger than others?**

**R: Yes all the same.**

**I: What about the children, are there some children who receive more food than others?**

**R: Yes some children eat little while others eat a lot.**

**I: Now could you describe any food sharing between family members during mealtimes (for example children eating together and separately from the family, meals eaten from the same plate by all family members)?**

**R: The children eat separately. They have separate plates. And adults have separate plates too.**

**I: Does the family share foods between households (for example do the share food with their neighbors)?**

**R: Yes they serve plates. For instant, this house serves that house and that house serves this house.**

**I: We’ve heard from some families that they eat local foods whereas others eat processed foods. Could you explain what is typical for your family?**

**R: We usually eat local foods.**

**I: Now can you describe what kind of food your family usually eats that’s local?**

**R: the foods that are usual like pumpkin if pumpkin, breadfruit, IU, pandanas and banana.**

**I: Okay. What about processed foods. Why don’t you eat processed foods?**

**R: Because we don’t usually have them here. And when they’re not usually here that’s why we don’t usually eat them.**

**I: So you mostly depend of shipments from Majuro to here?**

**R: Yes. If there is shipment then you know you’ll eat.**

**I: Okay. Are there any difficulties in cooking local foods?**

**R: The only difficulty in cooking local foods is that when there is no sugar, there’s no mixture to cook with. No flour to mix with.**

**I: What about when they are ripe (season of ripe)? Is that also a difficulty?**

**R: Well if they’re not ripe then we won’t cook them because they are not ripe. When we have then we cook.**

**I: What makes it easy to cook local foods?**

**R: Because they are ripe and we cook them we make them. Everywhere that they make like all the community when there is they make them.**

**I: What’s good about local food?**

**R: It makes our body healthy so we don’t get sick and everything else.**

**I: And what’s bad about local foods?**

**R: The bad thing about local foods is that if there is then we make them but if not.**

**I: What’s good or what is good about processed foods like canned meat and chicken?**

**R: It’s good because we just bring and eat it. It’s fresh and ready to eat. Just bring it and put it and eat.**

**I: Okay what about the bad things about processed foods?**

**R: Processed food you get sick like, amputated, high blood pressure, diabetes and everything.**

**I: Now that we’ve talked about how the family eats, I would like to learn more about how your child eats. Could you describe in detail what your son/daughter under 2 years commonly eats throughout the day? What does she usually eat or what do you usually feed her?**

**R: Rice. She usually eats rice and bread and canned meat.**

**I: Now what kind of canned meat?**

**R: Tuna and mackerel**

**I: Now how many times a day does your child under 2 years eats also include the snacks? When she eat, how many times does she eat other than eating in the morning, lunch or dinner? About how many times does she eat and also count the times when she’s eating lollipop and chip?**

**R: Anytime in between. When they eat and sees others eat, they’ll eat again.**

**I: So in could be like how many can you give me a number like how many?**

**R: In a day? I can’t really tell because not all the time there is. Only when there is then I could be 3 times a day.**

**I: Three times?... Now is says, how do you know your child has enough to eat?**

**R: The reason I know she’s full is because she spit out the food from her mouth. And when I feed her again, she refuses.**

**I: What can you do to encourage your child to eat?**

**R: That’s under 2 years of age? We can breastfeed her and…. What else?**

**I: What can you do to encourage your child to eat when she refuses? When she refuses, what do you do?**

**R: I please her to eat.**

**I: Please her with what?**

**R: I can breastfeed her and when distracted then I feed her food.**

**I: Do you feed your child differently when she’s sick (example; when your child has diarrhea)?**

**R: When she has diarrhea and I feed her, she refuses to eat maybe because she has diarrhea.**

**I: Now are there ways that you do to make them eat?**

**R: Like what? Because when she doesn’t want to eat.**

**I: Like are there ways because when you look at her, she has diarrhea. Like some child when they have diarrhea they refuse to eat.**

**R: They don’t want to eat but they want to breastfeed.**

**I: Oh they just want to breastfeed?**

**R: Umm (yes).**

**I: Good. Now you’ve told me what your child under 2 usually eats. Now could you explain to me the process, from start to finish, how you prepare and cook a meal for your child? Can you explain how you prepare her breakfast?**

**R: If it was pancake, we bring it and mix it with but first we wash our hands and then mix it. We mix it and then cook it.**

**I: What about her lunch?**

**R: Her lunch, you also wash your hands and then come and, if it was rice then you bring it and cook it.**

**I: Okay what about meat?**

**R: If it was canned meat, you just bring it and open it. Open it onto the rice.**

**I: So there’s no different with the breakfast I mean dinner?**

**R: umm (yes)**

**I: So what if there was no meat?**

**R: When there’s no meat, then we make rice pudding.**

**I: Rice pudding? So you put rice and what?**

**R: Rice with water and sugar.**

**I: Now can you tell me what kinds of foods you think are important for children under 2 year to grow healthy? What kinds of food are good for your child to be healthy?**

**R: Our local foods like pumpkin, papaya, banana, seafood like fish, clams (the bigger one), and chicken (locally raised).**

**I: Okay. What kinds of food are you not supposed to give your child under 2 years?**

**R: The kind of food that are spicy, salt and grease.**

**I: What about the biggest influence on feeding your child?**

**R: Like what?**

**I: Maybe we should bring foods that are good for their body?**

**R: Now why do you want to give them those foods?**

**I: So that her body grows healthy. No skin rash (kodkodi), not sick**

**R: Now can you explain the differences between how you feed your son and how you feed your daughter under 2 years? Are there any differences in feeding your son than your daughter? If you had a daughter that was 7 months or if you had a son that’s one year old going on two, would there be any difference in feeding them? Or if it was your twins, one boy and one girl, would there be any difference in feeding them?**

**R: Maybe there would be because one of the twins is unhealthy while the other one is healthy. Maybe because he has a healthy diet but the other one doesn’t.**

**I: Now how would you feed them, would they have the same amount and same kind of foods or would it be different?**

**R: I would have made them different because one of them knows how to eat while the other one because she doesn’t really eat that much that she doesn’t… Some food she doesn’t really eat.**

**I: So you mean one has little food than the other because one doesn’t really eat while the one eats a lot. It says that we are also interested in the roles and responsibilities different family members play in raising children. Could you describe the care of children throughout the day in your community? How do the people in this community raise their children?**

**R: In how they’re interested in raising their children?...**

**I: How do you raise your child?**

**R: I’m really interested in raising my child every day.**

**I: What do you do? When raising your child, what do you do?**

**R: I usually make them busy, bathe them, clean them and feed them. Make sure they don’t touch the ground.**

**I: Now who usually looks after the child?**

**R: Us mothers.**

**I: Why do you say mothers?**

**R: Because she is the one that prepare the family in a community.**

**I: What is the role of mother?**

**R: Clean them see what they need, feed them; put them to sleep all of these stuff.**

**I: What is the role of a father to the child?**

**R: Make them food or prepare them food.**

**I: Also bring what?**

**R: Bring from the ocean.**

**I: Now how do caregivers play with children under 2 years old? How do they play with the children?**

**R: This size (age)?**

**I: Under 2 years. So if it was, not you but any caregiver, how would they play with children under 2?**

**R: They would sing to them, tell them stories, make sure they don’t touch the ground.**

**I: What is the role of the grandparents in raising the children in this community?**

**R: Things like, making sure they don’t touch dirty things, make sure they don’t get in harm’s way.**

**I: In what ways would the grandparents support in raising children or support mothers and families?**

**R: Things like give them advice, watch what we give to the child and do to the child and things like that.**

**I: What makes a good grandparent?**

**R: They give them their needs, watch them, feed them, and teach them**

**I: Okay good. Now could you talk about the role that other family members have in raising children in this community? What are the roles of the other people in this community in raising the children?**

**R: Teach them and give them advice.**

**I: How does the older sibling look after the younger ones?**

**R: They also teach them, play with them and sing to them.**

**I: You are doing a great job. We’re almost done. Now for the last section, we would like to learn ways we can develop health program in your community. Could you explain where you usually get trusted information about nutrition and health?**

**R: Usually from hospital and radio. I usually hear from them. Also from the newspaper.**

**I: Why do you trust where these information come from?**

**R: Because these are the people that bring these information.**

**I: Where should these information be delivered to so that it would be easy to see or hear?**

**R: Radio V7AB**

**I: What types of media to you use to communicate with?**

**R: Radio**

**I: So you mean only radio because this is the outer islands and that the only source you used?**

**R: Yeah, usually they would broadcast it in the radio.**

**I: When you think about your own parenting, can you explain about what is different about how you raise your child than how others women in this community raise their child? What difference do you see?**

**R: Regarding what?**

**I: How you raise your children. Is raising your daughter different from how the other women raise the children?**

**R: I don’t think there’s any difference**

**I: There’s no difference?**

**R: Yeah**

**I: Now what are the opinions of the community in regarding on how you raise your children (for example; leaders, neighbors, church leaders and health workers)? What are their opinions about how your raise your children? For example when they say oh you’re really doing a great job raising your children.**

**R: I would say the same thoughts.**

**I: Now are there any advices or information you received regarding parenting?**

**R: Yes. From the hospital, nurse and the old lady.**

**I: Now where and who did the advice come from?**

**R: The old lady and also from the hospital.**

**I: Now are there any information about parenting you wish to know but don’t have available? Are there any parenting information you like to know that you don’t know about? You don’t know but you want to know?**

**R: Because there are so many question, I feel like I’m out of answers. There is nothing.**

**I: You mean everything is good? You understand everything? Okay good. We’re done. Thank you once again for you time with us. We appreciate you help and we hope this information that you gave us will help us improve the health of mothers and children in your community. Once again thank you!**
