# Supplementary material for: Conceptualizing multi-level determinants of infant and young child nutrition in the Republic of Marshall Islands–a socio-ecological perspective
Source: PLOS Glob Public Health. 2022 Dec 19;2(12):e0001343. doi: 10.1371/journal.pgph.0001343 (PMC10022247; doi:10.1371/journal.pgph.0001343)
Supplement: S1 Data — (ZIP) [file pgph.0001343.s001.zip › RMI Supp Data/Interviews data/I39R_IDI_HW_Arno_Sep 13_Libon_Shanteedited.docx]

Interview code: **I39R**

Interview type and Interviewee: **IDIHW**

Interview Date: **Sep 13**

Location: **Arno**

Interviewer: **Libon**

Transcriber: **Christina**

I: **To start, are you willing to do your parts with this survey?**

R: Yes.

I: **okay, well thank you very much for giving your time for us to talk with you, the information that will be learn by you will help us find a healthy lifestyle for women and children, and a healthy environment for the country. So, to start, can you tell me what kind of work you do…...in this family?**

R: in this house?

I: **mmm…**

R: well, doing handicrafts and helping them babysitting.

I**: okay. Now, what do you usually do from the morning till night?**

R: making handicrafts…collecting cobra [brown coconut].

I: **that’s it…now it says, now let’s talk about illnesses, I am also interested about the illness that children usually suffer from. In this community, what kind of illnesses you can say children under the age of 2 usually suffered from?**

R: such as what? fever and stuff like that? Well yeah, they suffered from fever, and when they fall from higher ground, that’s when they will have fever.

I: **okay**.

R: something like that.

I: **what else?**

R: about what? That’s one of the……. that they usually

I: **what about diarrhea?**

R: diarrhea…any kind of illnesses, they get diarrhea, fever, coughing…

I: **what about medicine…...**

R: and…...

I: **local illness?**

R: we help them, as for me, I do massage for the stomach for kids that have bumps in their stomach, so when I massaged their stomach, the bumps vanished.

I**: okay, so are there any other local illnesses a child usually suffered from?**

R: well, usually they have stomach bumps [fall from higher ground will cause stomach bumps]. And spirit entering their body.

I: **mmm……**

R: for stomach bumps, we kakijon [ local treatment] them. Usually the age…...the age of 2 usually have stomach bumps, things like that. They don’t usually suffer from other illnesses.

I: **okay.**

R: unlike stomach bumps.

I: **Now, what caused them sick, like fever, what caused fever, what causes diarrhea?**

R: well, for diarrhea, it caused by the food they ate, or foods that are not good for their stomach.

I: **mmm……**

R: when we feed them foods that are not good for their stomach, it caused diarrhea, things like that. So, reason they have fever and diarrhea is from…. reason that have fever, because they have diarrhea…some.

I: **so, what are the seriousness of the illnesses, fever and diarrhea?**

R: well, it is very serious, because when they have high fever, it can kill them. And when they have a bad diarrhea, and they don’t go to the hospital to see a doctor, it can also kill them.

I: **now, how can we prevent these illnesses?**

R: we take them to the hospital to see the doctors, as for diarrhea, only the doctors can help prevent it, but us, we can’t, because we don’t have any… ,but for some children that suffer from other illnesses that we can treat them by using local medicine for children. we can treat them with local medicine and that’s enough to cure it. They can get better by using local medicine, but if it’s fit for the illness.

I: **Now, what usually cure these illnesses in this community?**

R: well, as I’ve said before, only the doctors or

I: **local treatments**.

R: yeah.

I: **Now, can you describe what ways people in this community usually find to cure the children, for example, from traditional healer……from traditional healer or from the doctors or the nurses.**

R: traditional healers and doctors are the same.

I: **yeah but who is the first one that the people usually see or visit?**

R: see who?

I: **see the doctors or nurses or the traditional healers…… if the child is sick, who will be the first one you bring the child to?**

R: we bring the child to the doctor or if not, we bring to those who do traditional cure. Because as for some children, if we bring them to the doctors but are not getting better but than when we bring them to traditional healers, they got better.

I: **getting better**?

R: yeah.

I: **now, why do they see the doctor first when a child is sick, but not the traditional healer**?

R: maybe because they are doctors, and they know what to do.

I: **mmm…. okay, now do they use traditional healers, or do they use local medicine?**

R: used what?

I: I **said, do they use traditional healers or local medicine?**

R: when we brought them to the traditional healers?

I**: yeah.**

R: well, yeah when bring them to the traditional healers and they use….

I: **local medicine….**

R: yeah local medicine

I: **okay, can you tell me, what difficulties this community faces as they looking for cure for the illnesses that you have mention?**

R: difficulties?

I: **mmm…….**

R: well, when there is no like doctor….let just say there are traditional healers but when it’s so difficult to reach the doctor, or where the doctor is staying is too far from here, we find ways or if there is no money to rent the truck, it is really difficult for the children, because we just have to pay for the truck and go….

I: **so that you can reach….**

R: yeah…

I: **the doctor’s place…...**

R: yeah, yeah

I: **so, you can say that this is one of the difficulties**,

R: one of the difficulties is that there is no

I: **there is no way or access to reach the doctor’s place, so this is one of the difficulties…**

R: difficulties like there is no way for us to bring them to seek the doctor.

I: **what about…… what difficulties you faced when you help your patient?**

R: such as what?

I**: like when they bring a child to you, so you can cure him or her, what difficulties you might face?**

R: well, the difficulties that I faced is that I might be able to help some but some I can’t, because some of the illness that they have, I can’t cure them.

I**: okay…...now those are some information that we are looking for in your answer and it’s really good, but now can you tell me what kind of illness that is cause by the nutritious foods and are affecting the children in this community? foods like……we are talking about nutritious foods…**

R: just like papaya?

I: **yeah, well does it affect the children?**

R: but when they eat these kinds of food, it’s okay, foods that contain vitamins have no problem. It’s good.

I**: so, what kinds of foods might cause for an unhealthy body for one child?**

R: what?

I**: I said, what kind of foods might cause for an unhealthy body for one child?**

R: such as salty foods, fatty foods and things like that, they’re not good, and chilli foods

I: **okay,**

R: you know, food like ramen are not good for children.

I: **why?**

R: they’re chilli and have fats in them, and are salty, the ramen nowadays contains fat

I: **fatty….**

R: there is a package of ramen that have a small package of oil inside and also salt

I: **fat and salt…**

R: yeah

I: **so, what kind of food that cause a healthy body for a child? Why?**

R: the ones that contain vitamins… every kinds of food that contains vitamins.

I: **such as what?**

R: such as pumpkin, papaya, coconut drink……coconut drinks can also prevent kids from having diarrhea. when we gave them coconut to drinks, it prevents them from diarrhea.

I: **okay, we have talk about an unhealthy lifestyle, can you describe a day of a healthy person’s lifestyle, from the time they wake up until the time they go to bed? What do you see when you are looking at a healthy person?**

R: when we are looking, we see that the person is a very healthy person, and energetic, no such thing in their body, what can we say, their body contains nutrient…

I: **you mean they’re chubby…**

R: yeah

I: **they’re not**

R: they’re not because their body contain vitamin and they’re energetic…

I: **what about their movement?**

R: yeah

I: **when you see them moving, are they lazy or…**

R: no, because their body contains these things….

I: **now what signs or appearances that shows that a child under the age of 2 is healthy?**

R: signs….

I: **mmm……what do you see when you are looking at a healthy child?**

R: when we see them moving, we see that they’re healthy, and eager to do anything

I: **oh okay.**

R: they don’t usually get sick and you know, they’re very eager to play and….

I: **okay…what signs or appearances of a healthy adult?**

R: when they are eating healthy food and well taking care of and you know, and their body works well with the food that are given to them and yeah

I: **okay, and they’re working**

R: yeah working, we don’t just sit around………

I: **okay……now I have one more set of illness questions but relate to women’s health now. But could you tell me about your experiences with women who have anaemia?**

R: now how can answer that question about women who have anaemia…..maybe because of eating too much salty food and you know….

I: **mmm**……

R: have less blood because the salt makes their body dry……and coffee, because coffee is also not good for us, it also dries our body and stuff like that.

I: **so that is why we have less blood in our body….**

R: yeah, women in this island usually drink coffee

I: **oh okay.**

R: is that your friend?

I: **mmm…….do the women have knowledge of how serious this illness is?**

R: of course. They noticed that they’re not feeling okay, but they just want to use the stuff, because nowadays if not salt, they are like, bring the salt so that I can dip in with…….

I: **mmm, so what are the seriousness of this illness?**

R: like they know that salt is not good, but they just eat it….

I: **okay, now what cause of anaemia in women of reproductive age or pregnancy women?**

R: well as for pregnancy women, they don’t have enough supplements, but they just use stuff that are not healthy for them like salt.

I: **fats and……**

R: they don’t drink their supplements and stuff like that.

I: **okay, what about the foods they are eating? What kind of foods that can cause anaemia?**

R: foods that cause anaemia? Well, salty food and yeah, the one that I mentioned earlier like foods that are like strong for their body.

I: **so, when you mentioned strong food, what kinds?**

R: salt and……

I: **sal**t

R: ramen soup base

I: **ramen soup base…...okay, now were there any advice given to women to prevent or treat anaemia?**

R: yeah, we give them advice not to take any of the stuff….

I: **what would you have said to them?**

R: like, don’t eat this, don’t eat that.

I: **why?**

R: so, they can have much blood.

I: **now let’s talk about breastfeeding practices in this community, can you talk about how long after birth most women start breastfeeding?**

R: well, giving birth in an outer island, they just gave birth and some minutes later they start breastfeeding.

I: **why is it important to start breastfeeding right away?**

R: because midwife from long time ago says that breastfeeding right away is okay because it makes…….i mean the baby cries and……..as you know that the hospital forbids breastfeeding right away because…….why the clinics make us wait for a few minutes to breastfeeding, but long time ago when we gave birth we just breastfed right away….

I: **okay, now why did they always say, why like long time ago, they just told us to breastfed right away?**

R: maybe because the baby is crying and stuff like that…0

I: **what about the mother’s breastmilk….**

R: so, the breastmilk….

I: **like for the first breastmilk, is it important?**

R: it is important.

I: **then who……...**

R: you know that at first when we breastfed them, there’s no milk but just water, then minutes later there’s milk.

I: **mmm…...**

R: but sometimes, there’s no breastmilk at first, but maybe 2 days later there is…...

I: **there is….**

R: yeah…...the first day, there’s some….

I**: mmm, now do they always, like the doctors or midwifes or the old ladies, do they say that the first liquid, first liquid, I mean the first liquid or breastmilk is important for the baby.**

R: yeah, they told us.

I**: okay…... now, are there any liquids other than breastmilk given in the first days after birth?**

R: such as what? Milk….

I: **mmm….**

R: well as for some babies, they don’t give like a baby bottle which is filled with milk at all…only breastfeeding….

I: **what about the others?**

R: the others, if they cry, they just give them their bottle of milk…...

I: **how come they don’t just breastfeed them, but why giving them other liquid?**

R: because some when they don’t have breastmilk in their breast they just bring the bottle with milk in it and fed them.

I**: okay. Now can you explain exclusively how the women breastfeeding in this community?**

R: breastfed the baby?

I: **mmm,**

R: maybe they just want to breastfeed, no, because they are not able to…...it’s not enough……

I**: you mentioned it’s not enough, it’s not enough what?**

R: not enough money to bring milk, they just want to breastfeed, it’s not enough…….

I: **mm…. okay…. are there any liquids other than breastmilk given to the baby in the first 6 months?**

R: no, only breastmilk…...

I: **only the breastmilk…**

R: yeah

I: **okay, but why, how come they don’t give other liquids other than breastmilk?**

R: because they’ve been breastfeeding from the beginning and they don’t want to give other liquids.

I: **okay, okay.**

R: only the breastmilk

I: **now what are the difficulties faced by the mothers in your community to practicing exclusively breastfeeding for 6 months, are there any difficulties they face as they breastfeeding?**

R: I don’t think there’s difficulties, because breastfeeding is the only thing that works for them, so it’s good for them, unlike milk in a baby bottle because it would have difficult for them, because there would have no access to these kinds but breastfeeding, there will be no problem.

I: **no problem because it’s free of charge**

R: it’s free of charge, just the breastmilk.

I: **okay, so are there any ways to support the mothers to breastfeed for 6 months only? What would you have said to them to only breastfeed?**

R: because we give them………say like, you must breastfeed only because it’s this and that, we can’t bring this or that, only breastfeeding can…….

I**: but what about a healthy life for the baby…**

R: oh yeah, breastmilk also gives healthy, it helps….

I: **okay,**

R: other liquids don’t, when you bring like milk, some babies can have diarrhea just because of it, but breastfeed won’t

I: **it won’t**

R: yeah

I**: so, you can say that the baby doesn’t get sick because of it**

R: yeah

I**: but have a healthy life, okay, now it says, we are trying to understand how people eat in this community. It says, could you describe in detail what families eat and drink throughout the day? what they usually eat and drink throughout the day?**

R: food nowadays like rice, and if there is breadfruit, then yeah, if there’s fish, we eat them, foods like that, if we have them then we eat them.

I**: okay,**

R: we don’t usually go for can meat in here because there aren’t any can-meat with the families, just these kinds of foods

I: **okay, now how do you prepare these kind of foods….**

R: we cook them, like for example fish, when they bring them, we boil them, the rice, you just cook it and feeds the family.

I: **what if it was local foods?**

R: local foods like breadfruit, you cook it and eat.

I: **how…how do you cook it?**

R: you just put it on the fire or buliturok [breadfruit that is cooked with coconut milk, water and sugar], just for breadfruit, but as for pumpkin, you may not cook it with coconut milk but just boil it with water and sugar.

I: **oh, you may not use coconut milk**

R: yeah it may not cook with coconut milk, just boil it and eat it

I**: what about pandanus?**

R: pandanus, it can also be boiled, but some just want to eat it right away.

I: eat it right away…

I**: So, who in the family should be served first, next and last?**

R: well, the family, the adults should be served first, we should respect them and served them and then served the others

I: **can it be like, as you mentioned the adults should served first, then after that who are next?**

R: the men, the children and the women

I: **so, the women are the last…**

R: yeah

I**: Are there differences in the foods served to different family members?**

R: no, it’s same

I**: same**

R: yes

I**: Do some children, oh, are there any difference amount in the food serving to them?**

R: differences in food?

I: **the amount of food**.

R: the amount of food… no… well some eat less, so they received less, but some eats more, so they received more.

I: **so, you can say that if they are not full, they can go refill**

R: they can refill

I**: but by the time they are serving the foods, everyone has the same amount of food.**

R: yeah.

I: **So, do some children received more food than others?**

R: yeah, for those that eats more, received more, but for those that eat less, received less

I: less food

R: yeah, just for the perfect amount….

I: **now, could you describe any food sharing between family members during mealtimes (for example, children eating together separately from the family, meals eaten from the same plate by all family members)?**

R: no, separately, like in this house, one kid with one plate, and one adult with one plate, they don’t do sharing.

I**: okay,**

R: one plate for a person

I**: everyone has their own plate**

R: yeah, they have their own plate.

I**: Do the family share food with their neighbors?**

R: yeah, if they are really close by, then they share food with them. Well, like what, as for this family, we are the only one here so it’s just us

I: **what about the other families......because they’re too far from here, right?**

R; yeah, because they are too far.

I: **what if there’s not enough food?**

R: it’s just enough for

I**: will you still share it?**

R: sharing, how?

I**: like some places they go like, oh, reason why we’re sharing because there’s a lot of food.**

R: well as I have said before that if we have lots of food, than we will share but if not then we don’t, only the family will have them.

I**: okay, that’s great. Now I want to know about how young children eat in this community. It says, can you describe in detail what children under 2 years commonly eat throughout the day**?

R: like what?

I**: any kinds of food that is commonly eaten by the children**.

R: Rice…

I**: same age as her like 6**

R: rice and fish, and other kinds of food, papaya and pumpkin, yeah those are commonly eaten, as for the kids in this family, those are the foods that are commonly eaten by them.

I**: okay, now, how many times a day meal are eaten by children under the age of 2?**

R: three times, breakfast, lunch and dinner.

I**: what about between hours?**

R: they don’t usually eat between hours.

I: **yeah, not food foods but snacks**

R: yeah, not usually but if there is then they eat, because we rarely have snacks.

I: **so, no snacks, then they eat breakfast,**

R: breakfast, lunch and dinner

I**: mmm, now, do the children usually have snacks between meals?**

R: no, they rarely have snacks, but if there were any, then we would have given to them

I**: you mentioned that if there were any snacks, what do you mean by that?**

R: if there were like, cookies or something else that we make then we would have given to them to eat between hours

I: **okay, so you’re basically talking about import?**

R: yeah, we rarely have these kinds of things in here like money

I: **so, you’re saying that it all depends on transportation**

R: yeah

I: **and money to buy……now, are there any differences…...are there any differences in feeding their children when they are sick? Are there any ways that is different**?

R: I don’t think I know

I**: if the child**

R: if the child doesn’t like the food then they find something else but if not then they find ways to make the child eat.

I: **to see what the child likes to eat**

R: yeah, what the child likes to eat

I**: okay, are there any difference……what if, like, when they have diarrhea? are they fed** differently when they have diarrhea?

R: well, when some children have diarrhea, they hated some food, but once we brought another food then they ate, and when we brought something else, they don’t like it, because of the diarrhea…

I: **is it important for them to have full stomach when they have diarrhea?**

R: yeah

I: **why is it important?**

R: because if they have empty stomach, they’ll get weak, yeah for some children.

I: **okay**

R: they are weak when they have an empty stomach.

I**: okay, now, are there any differences when they are feeding the boys and feeding the girls?**

R: I think none, they have the same amount of food

I**: same amount of food**

R: yeah, same.

I**: okay, now can you talk to me about what really influence the family to feed their children in this community?**

R: what did you say?

I: **why is it so important, what influences the families to feed their children? why is so important for them to feed their children?**

R: to get healthy, yeah things like that.

I**: what else?**

R: [laughing]

I**: mmm**

R: [laughing] yeah, it’s important for them to get healthy, get healthy body and things like that

I: **okay.**

R: because if they don’t eat, then they will

I**: they’ll really….when they’re looking at us, they’re like**

R: yeah

I: **okay, what else? why is it important for the children to eat?**

R: what can I say, I mean I already mentioned, to get healthy or energetic and things like that

I**: what about illnesses**

R: well, as for illness, when some don’t……we find ways for them to eat this kind or that kind

I**: what about when they are healthy and eats a lot**

R: then you see that they’re not

I: **they are healthy**

R: they don’t usually get sick

I**: they don’t usually get sick**

R: yeah. they don’t

I: **now, we have heard that some families that eat local foods and others that eat processed foods, such as can meat and chicken, hot dog. It says, can you explain what, what is typical for most families in this community?**

R: local foods such as breadfruit and sometimes rice, when there is rice, eat bread and stuff like that. We usually eat local foods because we have those

I**: so, you mean half and half**

R: yeah

I**: because if there are**

R: when there are rice and other stuff then we eat

I**: but if there aren’t any, now**……

R: then that’s when we eat the local foods, such as IU

I**: IU, what else?**

R: and yeah IU

I**: so, when you mention IU, how do you prepare it?**

R: when I cook I,t I can also add rice, but if there is no rice then I add flour

I**: oh**

R: or corn starch, yeah things like that.

I: **what about pumpkin?**

R: pumpkin, you can also mix rice with it and flour

I: **oh**

R: you can also add flour to the pumpkin.

I: **what about sea foods?**

R: well, like…….

I: **what kinds of sea foods you guys usually eat?**

R: fish and other things, other things in the sea, like shellfish. So, when it ‘s low tide and we don’t have any meat then we tell the kids to go and fine some shellfish, so when they came back, they have lots of it, so we just bring them and boil them.

I**: mmm, the shellfish,**

R: yeah, any kind of shellfish

I: **oh, so, what are the positive things about local foods? Why local foods are good?**

R: lots of nutrients, free of charge, we just take and eat them, it doesn’t damage our body, things like that.

I: **what about the negative things about local foods?**

R: the negative things about what? Local food? Such as what?

I**: those food, are there any negative things about local foods? Are there…what negative**

R: no, none, they’re good, local foods are good.

I: **so, what are the positive things about processed foods?**

R: well, processed foods, we don’t have money to bring them, things like that…

I**: those are the negative things?**

R: yeah…

I**: what about the negative things about them?**

R: well, they are easy to cook, if you cook the rice, it will cook right away so you just eat it.

I**: what about can meat?**

R: well can meat, you just open the lid and eat it

I**: now, any balanced meals that can be prepared with locally available ingredients for children under 2**

R: yeah, foods are important

I**: okay, can you talk about what messages about breastfeeding and complementary feeding yu give to mothers or other community members**.

R: well, breastfeeding for kids, it’s so important.

I: **what would you have said to them about breastfeeding?**

R: yeah, when they………. when babies are not breastfed, and they get sick and when you try to make them eat, the babies won’t want to eat. But if they are breastfed, never mind feeding them food because the breastmilk prevents them from anything

I: **so, all……...all nutrients are in the breastmilk**

R: yeah in this breastmilk, but we you don’t breastfed them and you try to feed them with food, they will not want to

I**: mmm**

R: so, breastfeeding is important because it’s good

I**: Are there any nutrition education activities with community members?**

R: yeah.

I: **okay, what kinds of studies would you have given to them about nutritious foods?**

R: what can I say, or what should I do to make them just eat

I: **mmm**

R: make them just eat foods that are nutritious, and have vitamins and things like that

I**: why?**

R: because it gives nutrients to their body and things like that…

I**: mmm, it doesn’t get them sick.**

R: yeah, any of those things.

I: Now, are there any difficulties to delivering nutrition message to the people?

R: no.

I**: no, so you’re saying that it’s easy for you**

R: it’s easy

I**: for you to teach the people here in this community**

R: mmm

I: **about nutritious food**

R: food

I**: how about ways that this nutrition communication could be more effective? It says, how should be the information about nutrition, how would you have made it a better communication about nutrition.**

R: what can I say, well I can just tell to eat them, because they’re good for their health

I: **so, you mean, to advice them**

R: yeah, to give them advice to make, because it helps them

I: **mmm. Now I would like to talk about pregnant women in this community. can you tell me what kinds of foods that are eating during pregnancy?**

R: when they’re pregnant, there are some food they dislike, but they like other foods

I: **okay**

R: and the foods that they like, well we get them but for those that they dislike, we don’t

I**: so, what are the foods they usually grave?**

R: they usually grave foods like coconut drink, IU, pandanus, the things that pregnancy usually do

I**: so, those are like the things that they usually want**

R: yeah, want to eat

I: **to eat, what about…. are there any……it says, for pregnancy women, are there any changes in their diets during pregnancy?**

R: yeah, some do change their diet, they don’t like these, but they like those, the only difference in their diet is when they hate the foods.

I**: oh**

R: because they only want some

I: **so, you mean, they’re too choosy when it comes for foods**

R: yeah, they’re too choosy

I**: oh okay**

R: they don’t like the foods that we prepared for them because they want something else.

I**; okay, now, what really influences women’s diet during pregnancy?**

R: what changes?

I**: mmm, what really influence them to change their diet?**

R: maybe because the child [laughing]

I: **[laughing**]

R: maybe changes because of the baby, maybe babies do the changes because they don’t want to, because they are also eating when the mothers are eating…when mother is eating something, the baby is also eating

I**: so, you mean it also affect the baby**

R: it affects the baby

I: **okay, now what kinds of food women are encouraged to eat during pregnancy?**

R: foods that contain lots of nutrients.

I**: can you give me some examples**

R: such as our local foods, papaya, pandanus, pumpkin, nutritious foods

I**: okay**

R: they are healthy, so are the babies

I**: okay, what are the foods that women are encouraged not to eat during pregnancy?**

R: fats and salty foods and things like that, because when they eat too much salty food, the baby’s skin will be dry or peeled off

I**: mmm**

R: that’s because, they’re like oh that baby’s skin is like that because you ate too mush salty food

I: **oh okay, so now, who encourages or discourages eating those foods during pregnancy?**

R: us, the mothers, we encourage them

I: **okay**

R: for this family, I am, I mean I can encourage them to do this or that

I: **mmm, who else?**

R: their husbands

I**: okay, what about the doctors or….**

R; the doctors also encourage not to…because when they gave supplements, they told us not to eat those, but use that, the doctors gave supplements

I: **mmm**

R: for them to drink, some don’t want to drink the supplements, but we encouraged them to drink, because those are vitamins and are good for their body

I: **now, are there any supplements normally given to women during pregnancy?**

R: yeah, the ones that the hospital gave to them

I**: what kinds of supplements?**

R: the vitamins, and the one for blood

I: **the vitamins and the one for blood?**

R: yeah, they gave those

I: **now, what prevents women from taking their supplements?**

R: when they are nausea, and their body is weak, it’s like they don’t like the tastes, as for the vitamin, it tastes like something else, so when they are about to drink it, they hate the tastes

I: **okay, so do they sometimes drink alcohol, smokes, or use other drugs during pregnancy**?

R: yeah, sometimes

I: **so, now, why do they want to use these kinds of stuff?**

R: well

I**: but they know that they are pregnant, but they are using those**

R: well, that’s the part that we’re lacking off, because we know that they are pregnant but still using those, usually for the women in this community.

I**: mmm, now can you describe women’s diets during breastfeeding in this community?**

R: well, things that have vitamins, but some usually eat rice, but usually the ones that contains vitamins.

I**: okay, what about fish?**

R: fish, well you know that fish is commonly eaten here

I: **like uh?**

R: when they go spear fishing tonight then they eat fish

I: **so, you’re saying that fish is like the main source of meat here, unlike Majuro**

R: can meat

I**: unlike in Majuro, we say chicken**

R: chicken

I: **is the main source of meat**

R: yeah

I: **okay**

R: well in our island, we rarely have chicken

I**: mmm, only chicken (local food)**

R: well when there’s birthday party, you’ll have chicken, because they usually bring chicken for the birthday party,

I**: mmm**

R: but we rarely have those

I**: now, do women usually change their diet during breastfeeding?**

R: like

I: **do they change their diet during pregnancy?**

R: they don’t, when they eat, they eat

I: **you mean they eat any kinds of foods**

R: yeah, they eat any kinds of foods

I**: it’s different than when they were pregnant**

R: it’s different, there’s no food they dislike, they just eat everything

I: **Now, what kinds of foods that breastfeeding women are encouraged to eat and why are they being encouraged to eat the foods?**

R: as for fish, they encourage them to eat fish so there can be breastmilk for the baby

I: **mmm**

R: all kinds of foods, we’re like, you must eat a lot so there can be breastmilk in your breast

I**: oh**

R: yeah

I**: so, what kinds of foods that breastfeeding women are encouraged not to eat and why?**

R: salt, those foods that are not good for the baby’s body, also for them. Because when they eat salty food, it’ll make the breastmilk taste like salt.

I: **so, who encourages or discourages eating those foods while breastfeeding and why?**

R: us, the mothers, also the husbands, we encourage them not to do those because it’ll affect their children

I: **now, could you…...for the last section, we would like to learn about ways we can develop health programs in your community. Could you explain where you usually get information about nutrition and health**.

R: from the doctors, they gave us the information……they gave us information about this and that. We get our information only from the doctors.

I: **Now, why do you trust these sources**?

R: because that’s where health and things like that are at and they are our doctors, and they give advices to us

I: **where nutrition and health messages should be delivered to so that community members would see or hear them most easily?**

R: the radio, because that’s where every program are coming from and we listen to them everyday.

I: **oh, now, what types of media that community members use the most to communicate.**

R: the radio and the outer islands

I: **the radio and the outer islands**

R: yeah

I: **for our last question, could you describe what influences how people raise their children in this community**?

R: what influences them? We are the one influenced, yeah, we are the one influenced people on how to raise children in a community, we, the adults, give advice to do this or that….

I: **okay**

R: oh look, the child is going to fall

I: **now are there any information that pregnant or breastfeeding women typically ask for from health workers?**

R: yeah

I: **such as what**?

R: asking for help

I: a**bout how to breastfeed**

R: the doctors also teach us how to breastfeed, things like that, they help us a lot

I: **Now, is there a best way to communicate with caregivers about health?**

R: encouraging them, gives them advice to breastfeed or feeds them with food, we can say, cooks them healthy foods

I: **okay, is there anything else about the topics we talked about today that we missed or that you would like to tell us about.**

R: I don’t think there is, because every information is in there

I**: did we**

R: it’s all good

I: **did we talked about how you used to, you know when there is a sick child and they brought to you, how do you take care of that.**

R: yeah, I said that I can massage them, we already covered that

I: **okay good, just double checking**

R: [laugh]

I: **good, we are done. Thank you so much, the information that was given by you, we hope it will help us improve the health of mothers and children**

R: thanks to you too

I: **in our country**

R: for helping

I: **yeah**

R: and encouragement

I: **thank you so much.**
