# Supplementary material for: Conceptualizing multi-level determinants of infant and young child nutrition in the Republic of Marshall Islands–a socio-ecological perspective
Source: PLOS Glob Public Health. 2022 Dec 19;2(12):e0001343. doi: 10.1371/journal.pgph.0001343 (PMC10022247; doi:10.1371/journal.pgph.0001343)
Supplement: S1 Data — (ZIP) [file pgph.0001343.s001.zip › RMI Supp Data/Interviews data/I40R_IDI_MCG_Arno_Sep 13_BM.docx]

- Interview Code: 140R
- Interview type and Interviewee: IDI_MCG
- Interview Date: September 13
- Location: Arno
- Interviewer: BM
- Transcriber: Cendaniel Milne

**I: you will reveal your voice that you agree that you will participate on this record?**

R: yes.

**I: thank you for your wonderful time. Before we proceed on, can you tell me about your small family?**

R: about?

**I: your family.**

R: my family, about what?

**I: about, who’s in your family? How many of you? How old is your children?**

R: we only have one child, and there are two one is adopted. No, every one of them are adopted.

**I: every one of them are adopted?**

R: there are three younger brothers, two, and two that are living with us now. Our parents, one is on Hawaii, and other is on Majuro. (00:59)

**I: so, just all of you in this house? Can you tell me the ages of the adopted children?**

R: one is one years old, the other boy is ten something years old, I don’t remember how old he is.

**I: how many girls or boys?**

R: with us here? There’s no girls, but boys only.

**I: boys only? Thank you very much. Now can explain about the community?**

R: this community?

**I: yes, can you explain what’s good about the community?**

R: well, a bit good, were good stay in, there nothing.

**I: can you tell a bit story what’s good about your community?**

R: about how we live in? Well, we see it’s good to say. We don’t get hungry and these things.

**I: but, are there any difficulties in this community like challenges?**

R: there’s nothing.

**I: nothing?**

R: is it good?

**I: everything is good, thank you. Now we’ll talk about illness about your family. Can you explain about illnesses that youngest child always suffers from?**

R: mainly fever and diarrhea.

**I: fever and diarrhea?**

R: frequently always having fever.

**I: frequently having fever?**

R: diarrhea no, not really, few times

**I: so, these are the illnesses that have suffered from? Well, for fever. What causes to have a fever?**

R: we only monitor the temperature. Watering clothes and towels things like that.

**I: what do you think about the serious of fever?**

R: well, it’s huge because there are times they usually lead to seizure. And there are times it leads to affecting their minds. They’re deaf.

**I: so, can you explain what causes to have a fever?**

R: well, I don’t know.

**I: don’t know. Now for diarrhea, do you know what causes to have a diarrhea?**

R: maybe, when, sometimes playing and biting things, we don’t see dirty things biting like that.

**I: so, what you think from your own, how serious is diarrhea?**

R: well, I think is not. There are times, they hate food. For this boys, sometimes he hates food when having diarrhea.

**I: and how do you prevent him from diarrhea?**

R: well, we bring to the doctor.

**I: now doctor, where do you bring to see a doctor?**

R: there times with this doctor here. If not, Arno Arno.

**I: arno, arno, there is a doctor here on jebo right?**

R: jebo? Well, there’s none. At ine.

**I: at ine? You go to ine to see a doctor for this boy? Great. And you went to see a doctor, they gave you medication right?**

R: yes. Medication for diarrhea for children.

**I: good. Can you explain how do you know if the child that you’re adopting is ill? So, you two see a doctor.**

R: how do we, how do we know if sick right?

**I: sick. Because you two to see the doctor.**

R: when he’s often defecate, or when he poops only water. And we take him to see a doctor. We see that he’s having diarrhea in his stomach that’s why we see a doctor. So the doctor said.

**I: treatment for diarrhea or fever?**

R: well, fever.

**I: what other illnesses that you would show to**

R: the doctor?

**I: the doctor. But, who you first bring the child to when your youngest child is having illnesses?**

R: the doctor.

**I: the doctor? But, do you use traditional healers or traditional treatments?**

R: for these illnesses? Well, no.

**I: now, why not?**

R: we don’t have any ideas for local healing for these kind of illnesses. This is why we go to see a doctor.

**I: okay, perfect thank you. Now, what illnesses that are affecting your children caused by malnourished in their food?**

R: what did you say?

**I: you know, the foods that are nutrients? Foods that are malnourished? Would your child be ill if eating malnourished foods? (06:49) eating only candies, drinking sodas, is he going to be sick?**

R: well, we don’t know about that.

**I: now hasn’t he eat these junk foods? Are there any food that he eats and become ill of it?**

R: well there are none.

**I: there’s none? Perfect. What kind, what kind of food that can make your child not being unhealthy?**

R: I don’t know.

**I: good, it’s good, it is perfect. But, do you know what kind of food makes your child healthy?**

R: local foods, fish, leen wojke (often consider breadfruits, pandanus, and papaya-foods that are plants) (07:57)

**I: perfect, but any illnesses caused by foods missing from the diet?**

R: it can be, like what, malnutrition and those things? Foods that are, local foods?

**I: when it’s not enough local foods it will lead to being illness?**

R: well I don’t know, maybe. I don’t know.

**I: what about the foods that has nutrients in it, will he be**

R: well, we didn’t

**I: it’s fine, there’s no right or wrong in this question. We can tell from what you see and believe. There’s no right or wrong in this. Well, Could you now describe for me a typical day of someone living a healthy lifestyle, from the time they wake up in the morning until when they go to bed?**

R: how to live a day?

**I: a person that is healthy. From the time they wake up in the morning until when they go to bed.**

R: well, there are others that when they wake up in the morning, they’re healthy. They wake up, eat and moving, work. Till time to rest, eat again and rest.

**I: do you know what signs of a healthy child under 2 years?**

R: they’re healthy, they don’t cry. Always playing.

**I: what if, good. What if it was an adult? What if it was an adult? What appearance of a healthy adult?**

R: someone that, there’s no difference from child, someone that is healthy, doesn’t, sick and those things. Moving.

**I: always active? Good. Well, we’re done talking about health. We’ll now talk about food. Can you explain how your household gets food to eat on a daily basis?**

R: daily basis. Well, there are times we make copra and sell and other times we bring food from Majuro, my wife has salary, she’s a teacher in here, and that’s it, this is it.

**I: what kind of food you bring from Majuro?**

R: things like Rice, flour, meats like quarter leg and those things. Canned foods.

**I: and what foods are grown at home?**

R: coconut tree, papaya, banana, pandanus, pumpkin, lime, I think those things, those foods grown. Breadfruit.

**I: great, and the foods that grown at this house, is your family selling them?**

R: well, not all the time, most often.

**I: most often? Perfect. Now when times of selling, what do you do with profits from selling?**

R: we do buy foods with them, like foods of pelle (definition for foreign country)

**I: thank you very much, but, can you tell me about any difficulties of growing food at home?**

R: growing food? You’re growing foods? Well, it’s good, there’s none.

**I: well, can you explain how easy or difficult to have food? Foods that you get from Majuro, to be easy, like, it’s like how easy or difficult it is to have these foods here on Island?**

R: how easy is it right?

**I: easy or difficult.**

R: how they come from Majuro to here? Well, it’s easy to be here. There’s rides servicing the channel, there are each. And when we stay here, we just call and they deliver to here. For this place only, we make account books with the businesses and take foods. I think these are the things.

**I: great. Well, in a year, what foods are in shortage and what causes it? For this house, when there’s not enough food, what causes for not having much food?**

R: there are times when every stores are down (considered for every merchandises are sold out) because the sea is rough for transportation to deliver cargos. Things like that. This is it. When its transportation is difficult.

**I: so, when the water is rough, there’s no food for you to bring.**

R: either way we do eat, when the leen kein ekkan (foods grown at homes) have bear, we eat from them.

**I: but, during the times when there’s shortage of food. What do you do to have food?**

R: I always fish, fishing for food like that. But when there’s banana or breadfruit. Climb a coconut tree for us to drink like that. Just like these.

**I: perfect, thank you. Now I will ask about these animals that you are raising. Please tell a bit about these animals and what kind?**

R: mainly pigs and chickens.

**I: pigs and chickens? Well, there was a dog here right?**

R: one dog in this house.

**I: that’s your dog? Great. Now, tell about what makes you to raise these animals?**

R: about pigs and chickens, we raise them, there are times we eat. There are times when we don’t have foods in pelle (foreign country food) we sell them to take for our food.

**I: perfect, but are there any difficulties for raising these animals?**

R: like we don’t know.

**I: everything is fine. Perfect, well. Can you explain the difficulties of putting animals inside fences?**

R: (16:51)

**I: it’s like difficulties of making fence for the animals that you are raising.**

R: it’s like its good, there’s no difficulties.

**I: there’s no difficulties? So for the animal feces, what do you do with it?**

R: we collect them and put them in the farming places, the farms. The growing plants.

**I: good…well, there are times we want to eat some other foods. But we can’t, what kind of food the family wants to eat, but they can’t because there’s no money or most often.**

R: there are times, foreign food when it goes to be rarely to have in these places. And no transportations like these from Majuro.

**I: you mentioned foreign food, what kind of food?**

R: there are times when, foreign meat quarter leg when it goes to be difficult for transportation.

**I: so that’s why you wanted to keep eating? Are there any other foods that you want to eat but it’s rare or you cannot afford to take?**

R: … well, there’s also none like that.

**I: good, thank you. But, what are those that prevent you to eat the quarter leg every day?**

R: about?

**I: quarter leg right? You want to eat quarter leg right? What makes it difficult to eat it every day?**

R: prevent? Prevent us from eating? ... Well, how do I say? What do you mean about?

**I: if you don’t understand about this question, (19:23-19:27) why you don’t eat these foods? Let’s say you want to eat quarter leg right? Why you don’t eat it every day? Why do you not eat it every day?**

R: it’s rare in these places. When it’s rare, when there’s a bit. And it’s not every time we get to eat.

**I: when it’s not every time? It’s rare right? Depends on the ocean? When the ocean is good?**

R: and times when there’s like kemem (a child 1^st^ birthday) times where school’s break. When there’s programs in these places. And churches.

**I: thank you. And for the last question about food. Who in this family decide the food for the family?**

R: every time it would be us.

**I: so how do you decide the food for the family?**

R: we’re together to decide what food that is good for us to eat. What our children and siblings are eating.

**I: you two decide the food for children right? Both of you?**

R: things that they want to eat. Not just us, also them.

**I: also them? Well, now we’re going to talk about water and hygiene. Can you explain how do you find or store water?**

R: storing water?

**I: getting or storing water.**

R: water for drinking?

**I: yes. For you to store or get water. Not just water for drinking, water for shower, water for laundry.**

R: we get, when it’s raining, we pour into our water storage and store them.

**I: what kind of water storage you have? Water catchments and?**

R: water catchments and this, plastic container.

**I: and there’s**

R: well

**I: well.**

R: yes.

**I: where do you usually get water for drinking, cooking, washing, and bathing?**

R: from the water catchment. For bathing, container for well water. Well, containers that we’re getting.

**I: what are the difficulties in getting water? What makes it difficult to get water?**

R: difficult, what makes it difficult? Well, there’s no difficulties to it.

**I: what about difficulties to store water?**

R: when there’s not enough containers.

**I: so, how do your family make drinking water safe?**

R: well, we would pour them out. And pour soap inside them, or we use Clorox to clean too.

**I: and then let it stand to be filled again... great, well let’s now talk about washing hands. Could you describe in detail your family’s hand washing throughout the day?**

R: using soap. Showering soap.

**I: soap and water? But, how do the children wash their hands throughout the day?**

R: for these young children, let’s say. Children that can’t wash their own hands. We clean them. Soap their hands. For those children they know how to.

**I: for those children that knows how to, do you watch them how they wash their hands?**

R: yes, they do use soap. There’s soap we give them to wash their hands.

**I: perfect. Throughout a day, when do you usually soap to wash hands?**

R: throughout a day?

**I: throughout a day. When do you wash your hands?**

R: during times when eating. Morning, noon, and evening when they’re eating during morning, noon, and evening.

**I: morning, noon, and evening? Times when eating? You wash your hands? From what you think, what is the difference between washing hands with only water and washing hands with water and soap?**

R: washing hands with water only it’s like. Not cleaning unlike using water with soap. (25:14)

**I: but what are the difficulties for using soap for washing hands throughout the day? If not for you, but the people that you see or hear, what prevents you from using soap?**

R: what prevents? When it’s like not enough or no soap or what?

**I: yes. When there’s no soap or if there is soap but they don’t wash their hands with soap. Or if there’s no soap to wash their hands with.**

R: well, if there doing what? In a hurry like that. Or there are others who are really hungry, there in a hurry.

**I: so, are there times where there’s no money for soap?**

R: like what?

**I: if for soap, where do you get soap? The stores right?**

R: the stores.

**I: here on Island, how much here on Island?**

R: I think one, one dollar right?

**I: exactly one dollar?**

R: for soap soaps, joy (dish washing soap) and those things

**I: if it’s soap soap?**

R: soap soap I think it’s one

**I: one? Perfect. Well, thank you, thank you for helping us with your answers.**

R: yes.

**I: now, can you explain what, what kind of toilets you’re using in this house?**

R: bathroom.

**I: bathroom? Toilet? (26:54) what is the reason you choose this kind of bathroom?**

R: it’s like, it’s been … the idea from the family to make a better to use

**I: better bathroom? Well, in some communities. I heard there defecation on ocean and lagoon side. Is this practice still exist in these places?**

R: in the outer Islands there is.

**I: it will always be outer Islands, it will.**

R: places that, homes that don’t have bathroom in them. They use ocean and lagoon side.

**I: well it shows, so, what you think is difficult to build a bathroom?**

R: well I don’t know if, they can’t afford or, like these.

**I: so now for your children’s stools where do you throw them away?**

R: we do, always using diaper. We use diaper and (28:36) his mother washes it.

**I: so can you tell me, where do your children play in a day?**

R: well, in this community only.

**I: community? Can you picture a place, playground that’s perfect and good for children? Where, where would be a good place for the children to play in?**

R: in these places right?

**I: can you picture a place, a place where it’s clean and good for children to play in. where would it be, and why would you choose it?**

R: a place for playing right? … Because, these places there’s no playground for children. It’s like, they usually play in the community.

**I: if there was to be a playground.**

R: maybe there should a playground in these places. This would be the places that would’ve.

**I: so, do these kids play near the animals? Are there any animals in the places that they’re playing?**

R: yes, there’s animals like chickens and those things.

**I: so, what makes it difficult for children’s playing area to be clean?**

R: when, when we say don’t clean and those things. When we don’t clean it. They won’t play in it because it’s no good.

**I: well, to wrap up these questions about hygiene, can you explain how to prevent disease from spreading?**

R: prevent diseases? Go see a doctor. (30:56) from my own, doctor is better.

**I: see a doctor and ask for what? Why doctor now? For us to prevent diseases from spreading?**

R: give medications, for, for our illnesses. These things like that.

**I: sorry, I had to fix my sneakers. After, now for, what is your thoughts about stools that lay around and illnesses that are emerging? Are there any people who is ill cause of stools, do you know what illnesses that come from stools?**

R: yes.

**I: well, great. We have talked about hygiene. Now we’re going to talk about responsibilities of each family members of how they care for the children. Can you explain how this community take care of the children throughout the day?**

R: how do they take care of them? We feed them, clean them and for those children that are young. Have primary mind. One years old and those things. We look after them from dangerous things.

**I: if it’s not you or others, your children goes to play in other houses, how do they take care of them?**

R: they bring them and bring them to us. When we’re distracted or busy from them. Things like that.

**I: so, when you get distracted and go to other houses, they bring him to you? And who is really responsible taking care of the children?**

R: it’s us, the mother and father.

**I: you two? Well, for you. From your own, what are the responsibilities for mothers for taking care a child?**

R: cooking, cook for his food. Children’s food. Clean them, and teach them, that’s it.

**I: thank you, so for fathers, what are the responsibilities of fathers about taking care a child?**

R: taking care of them too. I think that would be it, taking care of them. Make food for them. See what they want to eat. Like that.

**I: good. Are there anything beside that?**

R: how do we say, playing with them. Look after them so won’t get hurt.

**I: do you have any babysitters? Are there any who take care of the children when you’re busy from each other? When you two are busy from the children, who takes care of the children?**

R: it’s like, when two of us are both busy. Its like, were often busy because I always take care of the children.

**I: it’s you when you…**

R: their mother, their mother works.

**I: perfect, and how do you play with your children?**

R: I gather them and play with them. or the road, inside the house.

**I: what kind of play you guys do?**

R: give a ball to or things like that. Playing toys, basket and.

**I: there’s a basketball place here?**

R: nope I

**I: nope you just make**

R: I bring a things for basket and toys for children.

**I: from Majuro right?**

R: yes, put it on the wall so he can play with it.

**I: it seems perfect, don’t say the elders are gone right?**

R: yes.

**I: how long they have gone?**

R: for our father, one week or two weeks have passed.

**I: two weeks.**

R: he just.

**I: he have (36:08) this child right? (36:10) what about the lellap (definition for grandmother or mother, formal way)? Was she here when this child is here?**

R: she was

**I: she was here, great. Can you explain how grandparents take part in taking care a child in this community?**

R: it’s like, when they take part in taking care of him/her. They always give words of support to us. And they give us how to care for a child, things like this. Advise us, words of preparing.

**I: so, tell me ways for the grandparents of these children help taking care of these children and also help you two?**

R: they usually… take care of them sometimes when our children is happy with them and go with them. They play with him... And there are times they eat together, like these.

**I: the child always play with elders?**

R: when they’re here.

**I: they always moving around? Moving around right? But what you think shows and clearly reveal grandmother and a grandfathers that are good and perfect?**

R: can you say that again?

**I: what shows and reveal grandmothers and grandfathers, grandparents. Makes them good and perfect?**

R: about? Things that they do right?

**I: for their grandchildren.**

R: well, teach them about good and bad.

**I: do they tell stories with them about the past? Show the difference about the past and today?**

R: yes.

**I: our cultures? Great. Well, can you talk about the responsibilities of others in this family for them to take care of the children in this community?**

R: for who?

**I: for this family.**

R: this family, others in the family? How they?

**I: taking care of, taking care of the children in this community. If for children in different places comes, how do you care for them? Do you play with right? Do you care for them?**

R: we let them play with children when they’re happy with them. we let them play with the children, their toys when they have balls and those things. We give them to play with them.

**I: great. Perfect. So, is that the youngest? How do the old children take care of their youngest siblings?**

R: well, they play with him/her. Take care, no different from how we take care of them. they know.

**I: they know how to take care of a child right? They take care of them, like how you take care of them.**

R: we teach them how to take care of them, and play with them.

**I: well, it’s so good thank you. It will be over soon. We only have two questions left. Is it okay? Could you explain where you usually get trusted information about nutrition and health?**

R: that would be hospital.

**I: which hospital?**

R: sometimes when we stay at Majuro, they give foods that are good for our children. There are times when, their grandparents they do ice-cream and taking care.

**I: they did explain it to both of you? Reasons why these sources are trusted? Why do you trust these sources?**

R: how we say, let’s vote right? Let’s be clean (41:46) for the family.

**I: prefect, and where do these information should go to, so it can be easier for you to know every day?**

R: for me, I think it should come from radio v7AB, those places because.

**I: great, (42:15) for these schools a poster would have posted like these. Others on the road when you walk.**

R: the schools, the hospital, those places.

**I: it would be easier for you to see right? When you walk around you can see it.**

R: yes.

**I: good for you to stop by and see it and learn to take information. But, how, how do you, how do you get sources from?**

R: these sources?

**I: these sources, these sources, how do you get them?**

R: we usually ask, we usually ask from their grandparents.

**I: unlikely you mentioned radio right? You sometimes listen from radio?**

R: sometimes radio does.

**I: are there any newspaper here on Island?**

R: well, there’s none. We only listen from

**I: from radio? What about place for internet? Are there any places for internet?**

R: there’s none. There only Arno, Arno.

**I: Arno, Arno. Thank you. Let’s proceed on to the last question. How do you think about the way that you have become a father. What really moves you to take care of your child?**

R: it’s like, … my wife does help with taking care of the child like these. It’s like, it’s good when we love them. (44:16) if it was for no making them go hungry. We decide what’s good for them.

**I: great.**

R: these things like that.

**I: so, what are the opinions for people in the community show a way to take care of their children? For example, for they show how to take care of the child from the leaders, neighbors, or church leaders, and health workers. How do they take care a child? What are the opinions for the people in this community of taking care of a child?**

R: how they take care of them? it’s like, they take care of them from being hurt or what?

**I: everything. If it’s for… we come to take care of the child because (45:32) children because the preacher told us from the bible how to take a child. Something like that.**

R: well, we take care of them it’s like, how do we say? How do take care of them from being hungry. Away from harm, these things. Take care of them from dangerous things. And when they get ill and we find a way to go to see a doctor. These things like that.

**I: if it was for you, or other from what you see. How do they take care of their children?**

R: it’s like there’s no difference

**I: no difference? Perfect, but are there any words of advice you learned about being a father?**

R: well … how do I say, they advise us because we need to take care of them. Keep them from harm’s way.

**I: who gave these words of advice and these information to you?**

R: those that are our parents. (47:12)

**I: but, where do, sorry. Are there any other information you wanted to learn about child’s mother or father but you can’t find these information? Are there any information you want to know?**

R: it’s like, I don’t know. I don’t have any plan on ideas like that.

**I: perfect. Perfect. But, are there any question from there? Our questions are done, do you have any questions from there?**

R: regarding our questions right? (48:00) what can we ask about?

**I: everything**

R: everything?

**I: if you have any questions, give it. Is there anything you would like to ask? Give it.**

R: if we have, now if we, we don’t have any questions. If I have questions but you’re gone. how do we get in touch you?

**I: you will come see the doctor, you can give questions to him, and he can get in touch with those boards that are for the doctor.**

R: well, this is about it. I would ask about it, just like our questions. About our siblings or children, well this could be a way to get nearer. (49:05)

**I: hospital will always answer you and help you with your questions.**

R: well, this is about it, we have gaps in our questions?

**I: there’s none. There’s no problem. Everything is good?**

R: yes.

**I: thank you**
