# Supplementary material for: Conceptualizing multi-level determinants of infant and young child nutrition in the Republic of Marshall Islands–a socio-ecological perspective
Source: PLOS Glob Public Health. 2022 Dec 19;2(12):e0001343. doi: 10.1371/journal.pgph.0001343 (PMC10022247; doi:10.1371/journal.pgph.0001343)
Supplement: S1 Data — (ZIP) [file pgph.0001343.s001.zip › RMI Supp Data/Interviews data/I41R_IDI_FCG_Arno_Sep13_Meia_MarcellinaEdited.docx]

**Interview Code:** I41

**Interview Type and Interviewee:** IDI_FCG

**Interview Date:** September 13

**Location:** Ine Arno

**Interviewer:** Meia

**Transcriber:** Meia

**I: To begin with, do you agree to participate in this interview?**

R: Yes.

**I: Thank you for giving me this time to talk with you, these information that I will collect from you will help find and ways to make Marshall island clean and healthy for the women and children, so, to begin with, can you tell me a little bit about your family? Tell me a story about your family, you don’t have to say their names, just who live in the house? How many girls and boys and then their ages together with the baby?**

R: We are seven in this house; my husband and I and three younger siblings and our two children.

**I: So, now how many boys and girls?**

R: Only one girl and four boys.

**I: Okay, how old are they?**

R: The oldest boy is 13 years old and the younger is 11 years old and the other one is 9 years old and this girl she is 5 years old, and then the small child is 1 year old, almost 2 years old.

**I: So, now can you tell me a little bit about this community? Or this island for we are in an island?**

R: about what?

**I: regarding the good and the bad things in this community. What do you see that are good around you and what do you see that are bad around you?**

R: The good things about this community or this atoll is because it’s good and the way of life is easy. But the bad thing is that there isn’t any enough lights at night for us to look around at night.

**I: Now we are going to talk about the health or the illnesses in this family, Could you describe the illnesses that your child faces and suffers from?**

R: Like what? Sometimes my child have flu, coughing, fever, chicken box, nausea, malnutrition, boil on the head and diarrhea, these are the sicknesses he usually faces.

**I: okay good thank you. So you mention flu, how did the child get the flu?**

R: The child get the flu during when it’s flu time, like when there is an announcement in the radio that there is a flu going around, okay then it’s easy for the child to get the flu.

**I: Okay, can you tell me is the flu sickness serious for the child?**

R: Yes, it is serious

**I: Can you tell me why you think the sickness is serious or why flu is serious for the child?**

R: The sickness flu for the child, the reason why I say is serious because sometimes if there is no medicine for the sickness then it will be hard for us to treat the child back to health.

**I: How can you prevent the flu sickness? Or what can you do to stop the flu sickness?**

R: We go to the hospital for treatment and let him drink the right medicine for the sickness.

**I: So, when you go to hospital, is there any hospital in this community or you go all the way to Ine?**

R: I go to the hospital at Ine.

**I: So, when you go to the hospital at Ine, what kind of transportation you use? Because it’s very far between here to there?**

R: Sometimes when it sudden and there are no transportation to use, I have to walk because it’s really important that I should go to the hospital.

**I: Is there any medical fee?**

R: It’s 25 cents for medical fee, but if it’s not working hours, then it cost $1.

**I: It $1? But what if it’s an emergency?**

R: If it’s an emergency then there is no fee.

**I: What if it’s not working hours-?**

R: If it not working hours but we go-

**I: But it’s an emergency for the child, do you need to, do you need to like, do you need to pay the fee?**

R: Yes, I need to pay $1

**I: $1? Okay, if it pass working hours but you really need the doctor you need to pay $1 dollar? Except if it working hours and it’s an emergency there is no fee right?**

R: yes

**I: Good, Thank you so let go down to coughing, what makes your child cough?**

R: Cough also comes from when he has the flu, and sometimes when he eats sweet which make his throat itchy.

**I: Okay, is coughing a serious illness?**

R: It’s not really serious

**I: Why do you think coughing is not a serious illness?**

R: Because it just cough, doesn’t matter if he cough because he will still drink and eat.

**I: So, what do you do when your child is coughing, or how can you prevent coughing? When your child is coughing what do you do to him?**

R: I let him drink medicine for coughing and I also brought him to seek help from the doctor.

**I: Okay, medicine from the hospital. Is there any other medicine you use? Because you also mentioned in the paper that you use Marshallese medicine. So, what kinds of medicine you use and how do you make it?**

R: The Noni tree. We bring the noni fruit and bound it and drain out the juice and let him drink it.

**I: Do you mix it with water or anything?**

R: if- it depends on their desire but the juice of the noni fruit must be strong.

**I: So, now does it burn, have you drink noni before?**

R: No, I haven’t- oh yes I have drink noni juice.

**I: When you drink the noni does it burn the back of your throat or?**

R: Yes it burns because when you cough it hurts, and when I drink it burns.

**I: It really burns?**

R: Uh-Uh Yes

**I: So, when your throat burns do you want to-? You vomit right?**

R: I don’t vomit.

**I: But it only hurts?**

R: It only hurts but I have to drink it.

**I: Let’s go down to fever, why do your kid usually have fever or why he always have fever?**

R: When it’s too cold, when he plays with cold water, when he stays where it’s too cold, and fever also comes when have sickness like chicken box, because then he will have fever.

**I: So, is fever really serious for the life of the child?**

R: Yes, a little bit, not really.

**I: It’s not really serious? So, when you say it’s not really serious, why is it not really serious? Is there any,,,**

R: He might have fever but he will still love to drink and eat.

**I: For the treatment, what do you do to him?**

R: I also take medicines from the doctor, and I also take his temperature when he have high fever with water and cloth.

**I: So, we are now in chicken box, what makes our child have chicken box?**

R: Chicken box base on my knowledge, it also comes with the flu.

**I: Flu? It goes together with flu? So, the sickness chicken box base on your knowledge, is it a serious illness?**

R: not really

**I: It’s not really serious? But what do you do to prevent chicken box?**

R: It seems like we only use coconut oil because there are like, no medicine for chicken box.

**I: There are no medicine for chicken box? So what kind of oil you use?**

R: I use Marshallese oil

**I: you also mention that use-? Because you said that when we have chicken box we have high fever, so we drink?**

R: We drink Tylenol

**I: You drink Tylenol, Okay good, so now nausea, what causes the child to have nausea or the child to be nauseous? Why do the child get nauseous?**

R: It’s also flu

**I: Yes, you mentioned that, you said that when he have the flu he feels nausea, and you also mentioned that because he feels hungry.**

R: Hungry, yes when he also gets hungry

**I: So, when you say hungry, like how long does he stays hungry?**

R: its long- it can be like, if he didn’t eat breakfast but he eat lunch, then he’ll feels nauseous.

**I: So, you can say he feels nauseous but doesn’t really feel nauseous. And so when he feels nauseous, what do you do?**

R: We- nothing, we just give him sweets.

**I: you gave him sweets to eat? So, when he feels nausea, is he doing something like laying down or vomit?**

R: He feels dizziness.

**I: Dizziness-**

R: He does not vomiting but only feels dizzy.

**I: Dizziness, so now what about diarrhea? What make a child have diarrhea?**

R: It comes from dirt. It can comes from the dirty dishes or it can be also from the left over foods we feed our kids with.

**I: You also mentioned that it can also come from the dirty hands of the person who is feeding the baby.**

R: yes when the person’s hands are dirty.

**I: And the person didn’t wash his/her hands. So, now do you think this sickness is serious?**

R: Well, yes because whenever he ate he poops

**I: It’s serious because he keeps on pooping? so when he keeps on pooping, what will happen?**

R: It’s serious because he will get dehydrated

**I: He will get dehydrated and this will affect him how?**

R: It can make him-

**I: like when he is not eating but keeps on pooping and vomiting, what will happen to the baby? What will it affect?**

R: His health, he will have weak body.

**I: So, when he have a weak body and he doesn’t eat and he gets skinnier, how do we labelled that?**

R: malnutrition.

**I: hmm, Thanks, so now base on your knowledge, is this sickness serious?**

R: yes

**I: What are your treatments for malnutrition?**

R: We have to feed them nutritious foods like the foods that are from the three groups of food. And also wash our hands and our dishes before we eat.

**I: so regarding malnutrition? What makes a child malnutrition?**

R: eating the foods that are not nutritious

**I: Yes, when our foods are not nutritious. What else, you also mentioned in the paper that because they also eat a lot of ….**

R: Junk foods

**I: Okay… so as you say he is not eating the foods that are nutritious, can you tell me what kinds of nutrition food he should have eaten?**

R: He should’ve eat breadfruit, fish and bananas.

**I: Breadfruit, fish and bananas, these are great examples. So what can we do to treat it, or what can we do to make him healthy?**

R: We need to give him foods that are nutritious and let him drink vitamins so that he can be healthy.

**I: So now boil in the head, what make a child have boil in the head?**

R: Boil in the head also comes from lack of vitamins.

**I: So, is boil in the head a serious illness?**

R: I think…..

**I: You said no.**

R: No it’s not

**I: What can you do to prevent the child from having boil in the head?**

R: We also feed him nutritious foods and let him drinks vitamins.

**I: And medicines?**

R: yes

**I: Can you describe how do you know when your child needs treatment for his/her illness?**

R: When he is not getting better and I have try my best but he is not getting better but only worse. That’s the time I bring him quickly to see the doctor.

**I: So who do you bring your child to first? Is it the doctor or the traditional healer?**

R: I bring him to the doctor first to have him check.

**I: But what if the child is not getting better after the doctor treat him?**

R: We can find someone to find out what’s happening to him.

**I: but are you always using medicine when your child is sick, do you usually use traditional medicine?**

R: yes

**I: What sicknesses that you usually use traditional medicine for?**

R: those kind of body sicknesses that the doctor can’t cure.

**I: So the sicknesses that the doctor can’t cure it, you use traditional medicine?**

R: yes

**I: Good thank you, Can you describe any illnesses affecting your children that are associated with nutrition? Are there illnesses that he encounter from the nutrition he consume?**

R: Sickness like boil in the head and-

**I: But he is eating nutritious foods?**

R: Oh, no, there are none.

**I: What about the illnesses caused by the foods missing from the diet?**

R: Yes, the sicknesses like boil on the head….. He also have diarrhea

**I: So, when he have boil on the head and diarrhea, what else?**

R: Nauseous

**I: Nauseous…. It’s a sign that shows the child have malnutrition?**

R: Yes.

**I: Okay, so we talked a lot about being unhealthy. Could you now describe for me a typical day of someone living a healthy lifestyle, from the time they wake up in the morning until when they go to bed? Can you describe how a healthy person do in a day?**

R: He is energetic.

**I: He is energetic, okay but what does he do?**

R: He is playing, he is walking around the area, he is doing household chore alone, and he is doing whatever he is doing.

**I: What about a child under 2 years old, what does he do that shows that he’s healthy?**

R: He doesn’t cry a lot, he doesn’t really need adult’s supervision, when he is playing, he will play until he is hungry then he come and eat. And there isn’t anything he doesn’t like.

**I: There isn’t anything he doesn’t like? So about an adult, how can you tell the appearances of a healthy adult?**

R: When they have already reach the old age but they still moving around doing chores.

**I: Let’s now discuss hand washing. Could you describe in detail your family’s hand washing throughout the day?**

R: We wash our hands sometimes with soap and sometimes we don’t use soap.

**I: Can you tell why sometimes your family is not using soap to wash their hands, what are the difficulties for your family to wash their hands with soap?**

R: What can I say… we have soap but maybe we’re too lazy sometimes to wash our hands with soap-

**I: Or you are not use to it**

R: Yes, I am not use to washing my hands with soap

**I: or because there were nobody to educated you on-**

R: Because nobody ever educated me on washing my hands with soap.

**I: goo thank you. But now are you teaching your children to wash their hands with soap?**

R: Yes I am teaching my children to wash their hands with soap.

**I: So, now can you describe how you oh- the difference between using water only or using soap and water to wash hands?**

R: The difference is that when you use soap to wash your hands, our hands are really clean. but when you use only water to wash hands the dirt are still there.

**I: Okay so what prevent you from using soap to wash your hands?... Like you said you forget and you are not use to it. These are the things that prevent you from washing your hands with soap. Now we would like to talk about your diet during pregnancy and breastfeeding-?**

R: I haven’t got pregnant myself, but the child I am taking care of is my brother’s.

**I: Oh so you adopted him**

R: Yes

**I: okay, now that you adopted him-**

R: I took him when hasn’t have age yet

**I: So, what about his parents, where are they?**

R: They are in Majuro.

**I: So you took him from Majuro**

R: yes I took him from-

**I: So were you with them when the mother was like- you took the child when he was how old?**

R: He haven’t reached one year yet; he haven’t reached one month yet.

**I: He haven’t has any months yet?! Like he just discharge from-**

R: Like he was like how many weeks, three?

**I: He was three weeks old when you took him?**

R: yes so I took him here. But his parents stay in Majuro.

**I: So, from when he was three months old you feed him bottle-milk?**

R: From when he was three weeks old!

**I: so from when he was three weeks old he only feed from bottle right?**

R: yes

**I: From when you took him here to this island?**

R: yes

**I: Okay these doesn’t apply to you, number 8 number9 any more number? Number 10 and number 11, we’ll skip 1234, so now I want you to explain, like- like- were you at the time the baby was born?**

R: No I wasn’t.

**I: You weren’t with him? And you don’t know if they gave him anything when he was discharge?**

R: Oh he was in the incubator

**I: He came out and went to the incubator, why?**

R: Because his skin was yellow.

**I: oh his skin was yellow? So did they give him anything when he was little?**

R: I think there are none, the only things that they give him at the hospital is the breastmilk from the mom.

**I: But was there any medicine the mom give him like Marshallese medicine?**

R: No

**I: None?**

R: Oh the grandma

**I: The grandma give it?**

R: Yes

**I: Is there any things the grandma gave? What kind of medicine the grandma gave if you know and still remember the medicine she gave when he was in- oh why did she gave him any medicine in the first place?**

R: Because she said his skin was yellow and she was trying to help the doctor treat the baby

**I: Because of his yellow skin and she was trying to help the doctors heal the baby faster?**

R: after she gave the medicine his skin colour is gone really fast.

**I: What kind of medicine she gave?**

R: I don’t know what kind of medicine. I only remember that she gave a medicine

**I: How did she give the medicine, did she let him drink it or?**

R: she put it in the mother’s breast and let him drink.

**I: so you can say she mix it with the mom’s breastmilk so they can let him drink it? Is it an oo (it a medicine that you wrap up in small piece of cloth) that she made?**

R: Maybe because she was-

**I: use drop?**

R: uh uh yes use drop

**I: How sad, we should have known. Now when you feed your child with bottle**, **right? You feed your child with bottle since you say he was three weeks old right?**

R: yes

**I: you took him and feed him with bottle up untill now?**

R: yes up till now

**I: Does he still use bottle?**

R: No, he doesn’t, he stop using bottle when he start consuming foods

**I: When he start eat foods?**

R: No, he stop when he was like how many months? Oh, he was already 1 year old, he only stop like when he was like how many months?

**I: So, you mean he just stop now using bottle. So he stop when he eat any kinds of foods and any kinds of drinks right? You also mention in the free lists that he also drink coffee. Can you tell me when did he start drinking coffee?**

I: He start drinking coffee after he stop with bottle-feed; when he was 1 year old. He drink anything we give except for milk.

**I: So, you mean he started drinking, he drank anything and coffee is one of his drinks. That’s good thank you. So now what else… now when you fed him in bottle, what did you feed him?**

R: I feed him bottle milk.

**I: What kind of milk?**

R: He feed morinaga

**I: Morinaga? So milk what else?**

R: Coffee

**I: coffee**

R: Kool-Aid

**I: Kool-Aid**

R: Coconut

**I: Coconut**

R: I thinks those are the only things

**I: Only these, how old was he when he drink Kool-Aid or Coconut?**

R: Coconut, he drank before the- he drank coconut and milk when he was still months old.

**I: oh! Okay what about kool-aid?**

R: when he was 1 year old.

**I: so, now could you tell me when did you first give foods to the child?**

R: When he was- you are referring to month’s right?

**I: yes**

R: He was four months old.

**I: Four month old, so why did you feed him at that age?**

R: because whenever he sees people eating, have watery mouth or he’s craving for the foods we eat.

**I: How do you know that he’s craving for your foods?**

R: He cries for the foods and want us to give him.

**I: Oh he cries for the foods. So is there any opinions from others that influenced their decision to introduce foods and liquids at that age? Is there anybody you know also feed their kids at four month?**

R: Yes, but there are some feeding at 3 months old.

**I: Ooohh! There are also some people feeding at 3 months old?**

R: Yes

**I: So what- base on your knowledge, why did they feed their kids at that age?**

R: It’s usually occurs to the child that are not with their parents but are with their grandparents.

**I: Oh okay, they feed them in this months. Who told them to feed the child?**

R: Base on my belief, themselves. Because they want to see if the child is ready to be feed at that time.

**I: To feed okay. So when they feed them at that age, is there any problem? Does he really consume the foods?**

R: Yes he really know how to eat, he is really consuming the foods.

**I: He is really consuming food up to today, his feeding process is goods, and there aren’t any problem. So, his first foods, what were his first foods and how did you prepare them?**

R: usually baby foods and mash potatoes, breadfruits-

**I: Yes you mentions breadfruit**

R: Pumpkin

**I: Baby foods, pumpkin, breadfruits, mash potatoes, cereal for baby**

R: yes

**I: so as for the baby foods, what- oh no baby foods and others are ok. The breadfruits, how did you prepare the breadfruits?**

R: The breadfruit that you kwanjini (kwanjini is when you cook the breadfruit on fire until it turns black like all the outer part turn black, then you use a sharp piece of glass to scratch out all the black skin). So you separate it skin from the meat and you grind the meat with a spoon to made it soft and mix it with milk. And then you stir it, and you add a little bit of sugar to sweeten it then you feed the child.

**I: Then you give it to him. What about pumpkin, how did you gave it to him?**

R: The pumpkin I cook first, then when it’s fully cook, I also grind it then spray it with milk and gave it to him.

**I: You also spoon it to him right?**

R: yes

**I: Was there any food he start eating using the bottle?**

R: Yes, the pandanus paste

**I: The pandanus paste like the makwon? He start eating it using the bottle when he was four months old.**

R: yes

**I: Good thank you for these informations, so now we are trying to understand how people eat in this community. Could you describe in detail what your family usually eats and drinks throughout the day?**

R: we bake like dough nut (round one), bread, dough nut, and pancake for breakfast.

**I: What about liquids?**

R: We drink kool-aid, coffee and water. These are the drinks we drink.

**I: What about for lunch?**

R: For lunch, we drink water, sometimes cold coffee, eat rice, eat meats like canned meats if we have, fish, and turtle sometimes.

**I: What about for dinner is it the same?**

R: Yes, like rice or soft food like aikui (iu mix with flour)

**I: How does the foods are made?**

R: how do we cook them?

**I: Yes, Cook them.**

R: as for pancake, I cook it on fire

**I: What are your ingredients for the pancake?**

R: flour, oil, sugar, and baking powder

**I: What else?**

R: Only these

**I: What about dough nut?**

R: same as the ingredients for pancake but we add yeast to it

**I: All these thing you cook on fire right?**

R: Yes, but sometimes when I have propane gas I cook on propane stove

**I: When there is propane gas?**

R: Yes when there is propane gas. But if there are no propane gas then I have no choice but to cook on fire.

**I: So, who in the family is served first and who is served last?**

R: who is what?

**I: Who is serve first and who served is last?**

R: The children first. We prepare the foods for the children first and our parents. but since they are not here, the baby first then us.

**I: Can you tell me if whether there are differences in the foods served to different family members?**

R: There are none

**I: None? All have the same, but is there any differences in the quantities?**

R: Yes, the child just not too much, and the older child is just enough and the adult a little bit more. Amount that it’s enough to be satisfy.

**I: Are there any children receive more food than others?**

R: Yes. It depends on how much they eat. some eat a lot some eat only little.

**I: Now could you describe any food sharing between family members during mealtimes (for example children eating together separately from the family, meals eaten from the same plate by all family members)?**

R: the children eat separately from the adults.

**I: Meaning all the children eat in one plate together right?**

R: Yes, like two children can eat in a plate or three children eat in a plate except for the baby he eats separately.

**I: He eat separately. Is your family sharing foods between household (sharing foods with neighbours)?**

R: Yes we give foods to our neighbours.

**I: how often?**

R: It almost ever times because we always share foods to each other.

**I: Good, now we have heard from some families that eat local foods whereas others eat processed foods. Could you explain what is typical for your family?**

R: we often eat processed foods, sometimes local foods. But we usually processed foods like rice and-

**I: food like rice and flours? Could you tell me what makes it difficult or easy for you to make local foods? Cook and eat local foods?**

R: For local food, there are times we have them and there times we don’t

**I: Yes, yes so what are the positive of eating local foods? Or the negative of the local foods?**

R: The positive thing about local foods is when we eat them, we don’t have any discomfort in our body. But whenever we eat processed foods, we have discomfort in our body like in our legs.

**I: so now is there any negatives about local foods?**

R: The negative things about the local is- I don’t see any negative things about local foods.

**I: Isn’t it when you have to wait for too long for it and it takes long time to prepare them?**

R: oh! (laugh) the negatives are, there are certain time we have them and certain time we don’t have, and when we have them it take long for us to cook them.

**I: What the negative of process foods oh you already told the negative about process foods but what are the positive things of process foods?**

R: The positive of process foods is we don’t have to wait for their seasonal. Except they cost money.

**I: So you mean it depends on money? Now that we’ve talked about how the family eats, I would like to learn more about how your child eats. Could you describe in detail what your son/daughter under 2 years commonly eats throughout the day?**

R: He usually eat in the morning pancake or dough nut. These are the thing that he usually eat in the morning. And for drinks, any colour drinks like kool-aid or coffee. For lunch, he eats rice and canned meats. And for dinner, if there is fish he’ll eat fish but if there aren’t then canned meat.

**I: Can you tell me how many times a days (and snacks) are eaten by the child?**

R: He eats three times a day

**I: Three times, what about the snacks?**

R: He doesn’t eat snacks very often.

**I: How do you know that your child have enough to eat?**

R: When he’s like- get up and walk away

**I: What do you do to encourage the child to eat if he refuses to eat?**

R: Like- I had to find any-

**I: When your child refuse to eat what do you do?**

R: I need to go to the doctor to check if he have any sicknesses or anything.

**I: For he doesn’t want to eat? What do you do if he’s like, doesn’t want to eat? like the food is there but he doesn’t want to eat?**

R: I need to hold him to me and like beg him to- like bribe him that after he eat he will do anything and he will eat.

**I: could you tell me whether you feed the child differently when the child is sick?**

R: Yes, when he is sick he eat only little but when he is not sick he eat more.

**I: So when he is sick and he eat little, could you tell me how amount of little does he ate?**

R: It like three spoon or two spoon and that’s enough.

**I: You’ve told me what your child under 2 usually eats. Now could you explain to the process, from start to finish, how you prepare and cook a meal for your child? Tell me a story of how you prepare the food from start to finish?**

R: I am talking as I am always busy, so I always prepare his breakfast at night and store it so when it’s morning I feed him.

**I: So what kind of foods you prepare for him for the morning?**

R: Foods like dough nut or tonaj (dough nut but it’s round)

**I: To prepare for the morning?**

R: To prepare it for the morning.

**I: What about for lunch?**

R: Now lunch I have to wake up in the morning and cook rice for lunch and then go.

**I: And you go to work?**

R: But sometimes his dad cook and feed him.

**I: Okay, thanks you, could you now tell me what you think are important foods for children under 2 years to grow well/ be healthy? What are the foods that are good and fit for him to eat?**

R: He needs to eat the foods from the three group of foods?

**I: Hmmm**

R: I need to feed him and after he’s done-

**I: Like what kind of foods in the three group?**

R: There need to be meats with the foods and then what? The snack**.**

**I: so now what kind of foods is it?**

R: Pancake with eggs and hotdog. these kind of foods.

**I: Then the snacks? What is it?**

R: Coconut meat (cotton) or lukor(is coconut cotton add water milk and sugar).

**I: What are the food that you should not be given to your child under 2 years?**

R: Foods like, what? The sweets.

**I: yes sweets?**

R: The sweets and the chips

**I: So, why you don’t you need to give your baby junks foods or sweets or chips?**

R: There is no vitamins in it base on my knowledge.

**I: So, what if there is no vitamin, how will that affect your baby?**

R: He will like- if he eats a lot, he won’t think about his foods that he needs to eat.

**I: What about the sweets what will happen or how will the sweet affect?**

R: It will damage his teeth.

**I: Thanks, What is the biggest influence on feeding the child or what are you influence on feeding the children at the age of 2? Do you have any words on advice?**

R: advice?

**I: Hmmm**

R: My advice is that anybody that feed their child, need to wash their hands with soap, and feed the child nutritious foods.

**I: Like?**

R: Like for breakfast, pancake with egg and hotdog and then snack. In order for the child to be healthy.

**I: Good we have reach almost to the end, Thank you for these good informations, it night but it okay for us to….. now could you describe any differences (if any) between how you feed your male children and how you feed your female children under 2?**

R: Like what?

**I: If you had two children, is there any differences on how you feed you son from your daughter under 2 years of age? The child under two years?**

R: If I had two kids or-

**I: Hhmmm are there any differences like in other places, boys are more important and the girls. Like they feed him more and girl only a little because she had little work?**

R: Ooohh, well, no it depends on if he is full then I will stop.

**I: You informations are good thank, we are also interested in the roles and responsibilities different family members play in raising children. Could you describe the care of children throughout the day in your community?**

R: like what?

**I: How do they take care-**

R: Prepare their foods?

**I: No, if you were gone, like for example, if there was a neighbour relative child came to your house to play, how would you take care of the child?**

R: Ooohh, well if any children came while I am feeding my children, I will also call them and feed them

**I: So, you mean you are also taking care of them like your own children?**

R: I also take care of them as my own children.

**I: Or the people are taking care of your child like they take of their own children?**

R: Yes

**I: Who is mainly responsible for the child?**

R: What child?

**I: Your child. Who is mainly responsible for him?**

R: Mostly his father, for I am always gone.

**I: Because you are working? What are your responsibilities as a mom?**

R: My responsibilities are, I have to prepare him before I go. like make his foods and make him ready like clean him and make him looks good.

**I: So, now what are his dad’s responsibilities to care for the child?**

R: His responsibilities are, he had to find meat for him and make his snacks and also look after him for I am busy.

**I: How caregivers play with the children? Like you as a caregiver, how do you play with your kid? How do you play with the child?**

R: Oh yes, I tickle him, tell stories with him

**I: Tell story with him**

R: what I meant by telling stories is, like I chat with him an adult so that he can learn fast how to listen and talk.

**I: Hmm, Could you talk about the role of grandparents have in raising children in this community?**

R: The responsibilities of the grandparents are, whenever the parents of the child are busy they replace them to care for the child, they are also like their parents; they feed them, they give them whatever is not enough for them or like their needs.

**I: what makes the grandparents good? Why the grandpa or the grandma is considered good to the child? What are they doing to make them good for you to say that they are good?**

R: because they love them and take care of them like we do.

**I: So, now could you tell me how siblings (older siblings) help raise the child? Is there any time that they take responsibility and look after him?**

R: Yes whenever we are gone and busy from him, then they come and look after him throughout the day.

**I: What about his older sibling how does he take of him?**

R: They are not close each other sometimes. Like the older one who doesn’t really understand about parenting, whenever she see us carry him, she will have problem with that because she said we are not doing the same for her but she is old.

**I: You are doing a great job. We are almost finish. Now for the last section, we would like to learn about ways we can develop health programs in your community. Could you explain where you usually get trusted information about nutrition and health?**

R: I usually heard about on the radio and from the doctor whenever I visit, the doctor give me advice about the nutritious foods.

**I: Why do you trust the information from the radio or the doctor?**

R: I have to trust them because this is where the health come from and I have to follow their advice about health.

**I: Where should nutrition and health messages should be delivered so that you would see/hear them most easily every day?**

R: It should be in the hospital, the school and the churches for us to hear them often and know.

**I: What types of media you are often using?**

R: GP Radio and telephone

**I: Oh there are phone, you guys are using phone here? Oh how nice**

R: They are using phone in the place that you are in.

**I: Yes but where exactly?**

R: You can stand at the lagoon side or the pastor home near the house that made from pandanus leafs or the place where you had your meeting last night and call from there.

**I: oh it has service from there how nice, so when you think about your own parenting behaviour, can you explain what influences how you raise your children? Is there difference of how you take care of your kids?**

R: Yes, while I raise my child I learn about life. Like when he is sick, I know what make him sick. Things like these.

**I: how about if you compare how you raise your kid from other people is there any difference on how you raise your kid from others?**

R: Like what?

**I: Is there any difference of how you take care of your kid from how are the other women take care of their kids?**

R: Yes,

**I: Like how**

R: The way I take care of my kid, when I take care of my kid- some mom leave their kids, some let their kid naked

**I: So you can say they are not spending their time with their child, they are only doing what they want right?**

R: hhmmm

**I: What are the opinions of the community influence on how they raise children like leaders, neighbours, church leaders and health workers?**

R: Theirs influences are very important about how to raise a child

**I: So you can said they give advice?**

R: They give advice to me on how to follow the steps to raise a child.

**I: They help you when they?,,,**

R: They help me and gave me advices.

**I: Did you learn any advice or information related to parenting?**

R: Yes

**I: uh uh**

R: I hear when I- there was a team before, they were here before and they give advice to the women and the men.

**I: To do what for the child? Like what advice did they give to you?**

R: They give us like-

**I: What did they recommend you to do?**

R: Take good care of them, and make their environment good, always take care of them

**I: like don’t leave them?**

R: yes

**I: Okay, where and who give you these informations? Oh you already said the leaders and also a team. Is there any desired information on parenting you wishes you had but doesn’t have available?**

R: Things like what?

**I: Anything you want to know about parenting or is there any desired information you wish to know about parenting?**

R: Like what should I say?

**I: Is there anything you wish to know about parenting? Or you want to be told about parenting?**

R: Oh show me what, how to take care of him?

**I: hmmm, if there is anything you wish to know but you don’t know about?**

R: I don’t really understand

**I: Oh you don’t really understand? Is there any information about parenting you don’t have and you wish to have or know like you haven’t know about it about parenting you wish.**

R: Like I want to know how to take care of him?

**I: Is there anything you wish to know about parenting?**

R: seems like none

**I: none? Is there anything else about the topics we talked about today that we missed or that you would like to tell us about?**

R: I think it’s all good.

**I: Thank you**
