# Supplementary material for: Conceptualizing multi-level determinants of infant and young child nutrition in the Republic of Marshall Islands–a socio-ecological perspective
Source: PLOS Glob Public Health. 2022 Dec 19;2(12):e0001343. doi: 10.1371/journal.pgph.0001343 (PMC10022247; doi:10.1371/journal.pgph.0001343)
Supplement: S1 Data — (ZIP) [file pgph.0001343.s001.zip › RMI Supp Data/Interviews data/I42R_IDI_FCG_Arno_Sep 14_Fela.docx]

- Interview code: I42R
- Interview type and interviewee: IDI_FCG
- Interview date: Sept.14.18
- Location: Arno
- Interviewer: Fela
- Transcriber: Marcellina

**I: okay before we start recording, I am asking you to tell it in your own voice that you are agreeing. Do you agree?**

R: yes

**I: okay thank you. Thank you for giving me your time to speak with me today. The information we learn here will help us find ways to improve maternal and child health and sanitation in your country. To begin with, can you please tell me a little about your family/household?**

R: like what?

**I: like who lives in this household…**

R: oh! Well me, my husband, my uncle, and our little siblings. That’s all.

**I: now it says, how many children and their ages?**

R: 12, 10, 18, and that baby 10 months old.

**I: now, how many girls or how many boys?**

R: 3 girls and 3 boys

**I: good. Now the second question state, can you describe your community?**

R: like?

**I: like what are the good things about your community?**

R: it’s good because lots of things we see, we play volleyball, and lots of different kinds of game we play

**I: okay are there any things else good besides these that make you love this community?**

R: well yes. It’s good because we can learn how to make copra, and we girls in here combine together and create group on making coconut oil. They cooperate with each other.

**I: good. Now it says, what are the bad things about your community?**

R: the bad thing about this community is that some people are nice and some are not.

**I: like how are they not nice?**

R: they’re just not nice. Like when you “good morning!” some will respond you back but some will not.

**I: oh okay good. We will now talk about health and illnesses in your family. Can you tell me about some of the illnesses that your child has suffered from or children in this community have suffered from?**

R: well I haven’t seen children in my household got sick but there is a child in our neighbour’s house who’s always got sick. Like after 3 days she/he got sick again. He/she always have headache.

**I: headache? Are there any other illnesses you see in other children in this community?**

R: there are some children when I see them, they’re like feeling lazy or unenergetic

**I: when they’re feeling lazy, they are like what?**

R: I don’t know! Maybe they are not well or they are hungry or I don’t know because I don’t really go to other people’s house

**I: well in the free list that we just did, you mentioned diarrhea, headache, coughing, and fever. So as for diarrhea, what are the causes of diarrhea?**

R: when they don’t wash their hands before they eat and when they eat leftover night foods.

**I: okay good. What about fever? What are the causes of fever?**

R: if they have fever it’s because they sunburn themselves a lot and also when they swim in the lagoon.

**I: as for malnutrition like you mentioned in the free list, what are the causes of malnutrition?**

R: when there are enough nutrients in their meals

**I: oh okay good. What about blind at night time?**

R: they said they’re malnutrition when they’re blind at night

**I: malnutrition good. Now what about korikori (fungus on the skin)?**

R: it’s also from malnutrition or mosquito bites

**I: good. What about headache?**

R: when they don’t have sleep because they stay up late

**I: okay. Now what are the seriousness of each illnesses? Like blind at night, what are the seriousness of blind at night?**

R: if they walk at night they might tumble down or fall into holes

**I: good. What about the seriousness of fever?**

R: fever is also serious because if we have high fever, there are some people like my cousin. She wasn’t deaf when she was born, but after she got high fever she became deaf

**I: it can also affect our brain**

R: yeah our brain. That’s what up with my cousin! She’s grown up but she’s deaf.

**I: oh poor girl. Now what about headache? What is the seriousness of headache?**

R: if they have headache, it can also affect the brain

**I: good. Now what about malnutrition? What is the seriousness of malnutrition?**

R: if they are malnutrition, they will be lots of thing will happen to them or they might live long enough.

**I: now it states, how can we prevent these illnesses? Like how can we prevent our children from having fever, headache, or blind at night?**

R: we must let them stay with us inside our house and don’t let them go to other people’s house because it’ll make them have illnesses. Lots of things will happen to them because they’re going out and inhale whatever.

**I: good. Your answers are very good. Now to the next question, can you describe how do you know when your child needs treatment when he/she is sick?**

R: when they are sick

**I: okay. Now who do you first go to when your child is sick?**

R: the doctor

**I: good. Why?**

R: so the doctors can check him/her up because he/she is sick

**I: okay. Now do you use traditional medicines for your child?**

R: yes sometimes when he/she is has fallen

**I: when they had fallen, what kind of traditional medicines you use?**

R: we let them drink coconut drink or the roots of the coconut. Sometimes they can just spill out drops of water into their stomach

**I: now who make the traditional medicines?**

R: my aunties. I have plenty aunties here

**I: now it states, can you describe any illnesses affecting your child/children that are associated with nutrition?**

R: there are none

**I: now it states, what kind of illnesses affecting your child/children cause by foods missing from the diet?**

R: nausea

**I: now this one states, we talked a lot about being unhealthy. Could you now describe for me a typical day of someone living a healthy lifestyle, from the time they wake up in the morning until when they go to bed?**

R: when they wake up, they do their household chores right away. They don’t feel lazy

**I: can you tell me the signs or appearances of a healthy child under 2 years?**

R: they don’t get sick frequently, and they look healthy

**I: now what are signs or appearances of a healthy adult?**

R: they look healthy, they like to roam around, and for the elders, the still like to do chores even though they are weak to move around

**I: good. Now the next question states, let’s now discuss hand washing. Could you describe in detail your family’s hand washing throughout the day?**

R: I don’t know because sometimes I see some of them wash their hands but some don’t

**I: okay. Now do the children wash their hands throughout the day?**

R: yes. Before they eat they wash their hands. If they don’t wash their hands then they’ll use hand sanitizer.

**I: now it says, do children under 2 years old wash their hands?**

R: yes the mothers help wash their hands

**I: during what time in a day soap is used to wash hands?**

R: at all time before we eat, we wash our hands with soap and then go eat. In the morning, noon, and evening.

**I: can you tell me the differences between washing hands with soap and washing hands using water only?**

R: when we wash our hands with soap, it really clean. But when we wash our hands with water only, the dirt are not really remove from our hands

**I: good. Now it says, what does prevent some people from washing their hands with soap throughout the day?**

R: the hand sanitizer

**I: are there anything else besides the hand sanitizer?**

R: when we are really hungry

**I: Now we would like to talk about your diet during pregnancy and breastfeeding. Now I would like you to think back to when you were pregnant. Can you describe your diet when you were pregnant compared to when you were not pregnant?**

R: when I was pregnant, I usually eat ramen and raw fish

**I: what about when you are not pregnant?**

R: well I eat everything

**I: everything good. Now what really inspired you to eat ramen or raw fish?**

R: I don’t know. I just craved for them when I was pregnant.

**I: okay. Now what types of food they encouraged you to eat during your pregnancy and why?**

R: they usually recommend me to eat fruit, drink coconut, eat…..

**I: as for fruits, what kind of fruits?**

R: my mother usually bring me fruits from Majuro like watermelon, grapes, and candelope

**I: now why did they recommend you to eat these foods?**

R: so that my baby can be healthy

**I: good. Now what kind of foods they encouraged you not to eat during your pregnancy?**

R: ramen and Kool-Aid, pik-nik with Kool-Aid, everything that is mix with Kool-Aid. And also raw fish

**I: raw fish? Why didn’t they want to eat raw fish?**

R: I don’t know why but I really liked it. They said I don’t have to eat raw fish every day because it’s not good for me.

**I: now it says, who encouraged or discouraged to eat these foods during pregnancy?**

R: my husband and my mother

**I: now it says, who took care of you or supported you during your pregnancy?**

R: my husband and everyone in my household

**I: okay good. Now how did each people help you during your pregnancy?**

R: they help me with my needs like when I ask them to cook me food they will, and they wash my clothes.

**I: good. Now it says, can you tell me about any supplement you took during pregnancy?**

R: I took all of the supplement. But I usually hate the vitamin one; sometimes I take it sometimes I don’t

**I: like can you name the supplements?**

R: I don’t what they called but the one for helping producing blood and the vitamin

**I: you take the one that is for helping producing blood? Why didn’t you take the vitamin?**

R: when I take it, I am most likely to vomit and I don’t like it

**I: do you have any understanding on why you are most likely to vomit when you take it?**

R: I don’t know. I only know that I always feel nausea when I take it

**I: and you are okay with the other one?**

R: yes. And you know when I drink the vitamin ones, I must prepare a sweet drink or maybe have a lollipop so that I won’t taste it but instead I taste sweet.

**I: good. So, did you drink alcohol, smoking or use other drugs during pregnancy?**

R: I didn’t

**I: you didn’t, good! Now did you use any traditional medicines during your pregnancy and why?**

R: yes I did. They let me drink a traditional medicines which is for feeling nausea.

**I: okay, why did they let you drink that?**

R: so that I would love to eat food because I vomit whenever I eat food

**I: oh. What do they make it from?**

R: nin (noni) and kino (local leaves which usually grow beside the coconut trees)

**I: okay good. Now, if you were advised to eat fruits and vegetables during pregnancy, could you describe what would make this difficult?**

R: I don’t like it. I hate it

**I: you don’t like it? Okay. Now what would make it easier for you to eat more fruits and vegetables and why?**

R: I don’t know

**I: oh okay. Now it says, can you describe your diet when you were breastfeeding?**

R: I usually eat fish, corned beef, pumpkin, breadfruit, and mere (the white food inside the coconut drink) I really love mere.

**I: what really inspired you to eat these foods during breastfeeding?**

R: breastmilk. I ate these because they help in producing breastmilk

**I: now what kind of foods you were encourage to eat during breastfeeding and why?**

R: they recommended fish, corned beef, drink milk or foods that contain milk so that I would have enough breastmilk

**I: okay good. Now what kind of foods you were encouraged not to eat during breastfeeding and why?**

R: they recommended me not to eat salty foods like spam, chicken, and salt fish.

**I: now it says, who encouraged or discouraged you to eat those foods during breastfeeding?**

R: my mother

**I: good. It says, after you gave birth, could you describe breastfeeding your child throughout the day?**

R: breastfeed her/him

**I: you just take her/him and quickly breastfeed her/him? Like how long after giving birth you started breastfeeding and why?**

R: at that same time I gave birth. They wait for a while and give him/her to me to breastfeed her/him

**I: and why?**

R: because the baby was crying. They told me breastfeed my baby because he/she was crying so I take him/her and breastfeed him/her

**I: okay. Did you give bottle milk or any other liquids to your baby on the first few days after birth and why?**

R: I breastfeed my baby until he/she was 3 months old. That the time I gave bottle milk to my baby.

**I: good. Now it says, were there anything that make it difficult or easy for you to breastfeed your baby until 6 months and why?**

R: the only difficulty is when my breast don’t have enough breastmilk then I will give bottle milk to my baby

**I: now this one says, could you tell me when did you first give foods or liquids other than breastmilk to your child?**

R: at 6 months old

**I: can you tell me why did you gave foods or liquids to your child at that age?**

R: because the breastmilk are not enough anymore and he/she cries every time when she/he not having enough. So when I gave water and foods, she/he doesn’t cry anymore.

**I: oh okay. What are the opinions from others on making decision to introduce foods and liquids at that age?**

R: they said, as for those mothers who introduce foods at 3 months old they said that because they don’t have enough breastmilk

**I: now I am saying, what were the first foods you gave to your child and how did you prepared them?**

R: I gave first baby food and makwon (pandanus juice)

**I: so how did you prepare the makwon?**

R: I brought pandanus and boiled some and they are cook I grate them and mix it with milk

**I: good. Now to the next question it says, we are trying to understand how people eat in this community. Could you describe in detail what your family usually eats and drinks throughout the day?**

R: as for breakfast, we usually eat pancakes. For lunch, we usually eat rice and chicken. Sometimes gravy. And for dinner, we usually eat fish

**I: good. Now it says, can you describe the process of how meals are made?**

R: as for fish, we grate out the scales and then cut it and fry it. And for chicken, we also fry it.

**I: you are answering perfectly. Now who in this family is serve first, next, and last?**

R: first will be our parents and the kids, and after will be us teenagers

**I: good. Are there any differences in the foods served to different family members?**

R: yes there are. Like for children they will have to eat canned foods if they are having fish. Because they might hurt their throat with the fish bones.

**I: good. Are there any differences in amount of food served to different family members?**

R: yes. Some have more than others

**I: now it says, are there any children receive more food than others?**

R: yes there are

**I: and why is that?**

R: like some love food more than others

**I: Now could you describe any food sharing between family members during mealtimes (for example children eating together separately from the family, meals eaten from the same plate by all family members)?**

R: everyone eat separately from each other

**I: now it says, are there any foods sharing between households?**

R: yes we share our foods to our neighbours

**I: now it says, we have heard from some families that eat local foods whereas others eat processed foods. Could you explain what is typical for your family?**

R: as for this household, we usually eat processed foods like chicken, turkey tail, and hot-dog

**I: now what does make it difficult or easy to cook local foods?**

R: there are no difficulty in cooking local foods. The only difficulty is when there is one to bring the local foods like fish or crabs for example.

**I: okay. What are the good or the bad things about local foods?**

R: good thing about local foods is they are healthy. The bad things is we don’t know how to cook some of them.

**I: now what are the good or the bad things about eating processed foods?**

R: as for chicken and ham, I like these but they said they are not good for our health because they are very greasy. And for rice, sometimes it make our legs hurts.

**I: Now that we’ve talked about how the family eats, I would like to learn more about how your child eats. Could you describe in detail what your son/daughter under 2 years commonly eats throughout the day?**

R: my daughter?

**I: the children**

R: well as for my daughter, she usually eats local foods like lukwor (iu mixed with water and milk), banana, and the pohnpeian apples. as for the son, well he usually likes rice and ham. And as for the baby, he/she eats everything. He/she usually eats ramen and nutrition biscuits for breakfast. and for lunch, he/she eats rice with sausage or with fried ham with eggs or rice with mackerel.

**I: good. Down to the next question it says, how many times in a day child under 2 eat their meals and snacks?**

R: we as usual, three times a day for meals and for snacks, usually twice a day

**I: good. Now it says, how do you know when a child has enough to eat?**

R: they look healthy and they don’t feel lazy because they are having enough foods

**I: it says, what can you do when your child doesn’t eat?**

R: look for other ways to make him/her eats. Bring him/her and find the reasons why he/she doesn’t want to eat. Like if he/she feels nausea, I will do the nausea treatments, so he/she can eat.

**I: okay. Now it says, what can you do to get your child to eat when he/she really refuses to eat?**

R: what can say? Well maybe we can say “hey come and eat and I will give you chocolate when you finish”. Like lie to them so they can eat because there are some kids really refuse to eat so we lie to them.

**I: you lie to them. Okay. Now it says, are there any differences in feeding your child when he/she is sick? like when he/she has diarrhea for example.**

R: when he/she is sick, she/he barely eats, or she/he doesn’t eat much. And when he/she doesn’t feel sick, he/she eats a lot.

**I: okay good. Now you’ve told me what your child under 2 usually eats. Now could you explain to me the process, from start to finish, how you prepare and cook a meal for your child?**

R: sometimes I must cook early in the morning, so they can have their breakfast before they go to school. Sometimes I cook in the faster way like bring ramens and boiled them and let them eat. Or sometimes I just give them nutrition biscuits. And I cook rice for lunch and dinner so that they can just come back from school and eat with can foods like spam.

**I: good. Could you now tell me what you think are important foods for children under 2 years to grow well/be healthy?**

R: we must prepare healthy foods like lukwor (iu mixed with water and milk) so they can eat

**I: now it says, what type of foods you should not give to your child under 2 and why?**

R: we should not give them salt fish because it’s not good

**I: salt fish, okay. What else? Are there any other foods you should not give to your child under 2?**

R: ramen

**I: okay good. Now what is the biggest advice on feeding the children?**

R: we to tell them to wash their hands before they eat, and before they eat they should pray.

**I: good. Now it says, can you describe any differences between how you feed your daughter and how you feed your son under 2?**

R: there is a different. As for my son, there are certain kinds of foods he dislikes which my daughter likes. They must eat separately because they have different appetite.

**I: good. Now it says, we are also interested in the roles and responsibilities different family members play in raising children. Now could you describe the care of children throughout the day in your community?**

R: like what? Like as for the community, what about it?

**I: like who is mainly taking care of the children?**

R: well we ourselves like the mothers. As for this house, we take care of our own children.

**I: okay now it says, what are the responsibilities of the mothers to their children?**

R: they must prepare everything for their children. Like they have to prepare food for them, bring them and help them with their homework, and teach how to work.

**I: okay. What are the responsibilities of the fathers to their children?**

R: the responsibilities of the fathers are, they must do everything for them or support them like go fishing and make copra.

**I: good. Now how do the caregivers play with children under 2?**

R: play with them. Like play with them inside the house or take them outside and play with them.

**I: now it says, could you talk about the role of grandparents have in raising children in this community?**

R: I don’t know because the grandparents of the children are gone.

**I: what about the grandparents in this community? What are their responsibilities?**

R: they usually said “hey you guys go and study” like recommend them to go and study and do their homework.

**I: what about the grandparents of the children under 2?**

R: they take them and feed them and give them bath

**I: it says, how do the grandparents help or support in raising children and support mothers and fathers?**

R: as for me, my parents, they always helped me with my child

**I: like in what ways?**

R: like taking care of her/him and cook food for her/him

**I: good. Now it says, what does make the grandparents a good grandparent?**

R: they also take good care of our children

**I: okay. Now could you talk about the role that other family members have in raising children in this community?**

R: like what?

**I: like your relatives or your aunties or your uncles. What are their roles in taking care of your children?**

R: there are sometimes they come and take them to their house and take care of them.

**I: okay good. Now how do the older siblings help in raising young children?**

R: they look after them. Like as for my older brother, he treats me nicely.

**I: good. You are doing a great job. We are almost finished. Now for the last section, we would like to learn about ways we can develop health programs in your community. Could you explain where you usually get trusted information about nutrition and health?**

R: we must go to the doctor to get the information

**I: okay. Now why do trust where or who the information came from?**

R: because all the informations are with the doctor

**I: okay. Now where nutrition and health messages should be delivered to so that you would see/hear them most easily?**

R: me. I must seek them, so I can get the informations

**I: now it says, what types of media you use the most to communicate with? Like the radio, online apps, websites for example.**

R: the GP radios

**I: okay besides GP radios, is there any other?**

R: cell phones

**I: good. Now it says, when you think about your own parenting behaviours, can you explain the differences on how you raise your children?**

R: when I am taking care of my children by myself?

**I: yes**

R: well when I take care of my children by myself, I like it. Even though I feel tired but still try my best.

**I: now it says, what are the opinions of other people in this community about raising children? Like the leaders, the neighbours, the church leaders, or the health workers.**

R: sometimes they tell me that I know how to take care of my children because I take care of them by myself. They say it’s good that I take care of them by myself and not depending on others to take care of them.

**I: good. Now it says, were there any advises or informations you received about parenting?**

R: yes. At hospital, there are informations about parenting.

**I: from who?**

R: from the nurses

**I: okay good. Now are there any desired information on parenting you wish to have but doesn’t have available?**

R: none

**I: good. well is there anything else about the topics we talked about today that we missed or that you would like to tell us about?**

R: none

**I: okay that was great, we are done now. Thank you once again for your generous time and for sharing your thoughts with us. We greatly appreciate your help and we hope this research will help us improve the health of mothers and children in your community.**
