# Supplementary material for: Conceptualizing multi-level determinants of infant and young child nutrition in the Republic of Marshall Islands–a socio-ecological perspective
Source: PLOS Glob Public Health. 2022 Dec 19;2(12):e0001343. doi: 10.1371/journal.pgph.0001343 (PMC10022247; doi:10.1371/journal.pgph.0001343)
Supplement: S1 Data — (ZIP) [file pgph.0001343.s001.zip › RMI Supp Data/Interviews data/I44R_IDI_FCG_Arno_Sep 14_Meia.docx]

I44R_IDI_FCG_Arno_Sep 14_Meia

Interview Code: 144R

Interview type and Interviewer: FCG Meia

Interview Date: September 14, 2018

Location: Arno

Transcriber: Joie Heine

**I: Are you willing to participate in an interview?**
R: Yes
**I: Thank you for giving up your time to do this interview. All the information we gather from you will help improve the health of mothers and their children, and also sanitation of this island. To begin, can you tell us a little about your family?**
R: Like what?
**I: Like who’s living in this household? How many children and how old they are?**
R: Oh, I see. My husband and I, also there are others; Chad and his wife, and only two children. One is in kindergarten.
**I: How old?**
R: The girl is one.
**I: What about the other kid/child?**
R: The kid is 5.
**I: Is the child a boy or a girl?**
R: She is a girl.
**I: Can you tell us a little about this community? What are some of the good things or bad things happening here? What are some barriers/challenges you face in this community?**
R: Let me think of some challenges? I think life’s pretty good considering the fact that we’re making a living from making copra. However, we’re out of coconut apple (iu) at this time. I cannot think of any challenges.
**I: What about basic necessities, anything you lack?**R: Yes, there are times when we lack some basic needs.
**I. Are there things you see here that are bad? Are there things you need or want to have?
R: …
I: We’re good, right**.
R: Yes, we’re good.
I: **Now, we’re going to discuss health and sicknesses in this family?** **Can you tell me about some of the illnesses that your children have suffered from?**R: There are many illnesses…
**I. Yes, can you explain more so it can be recorded?**
R: Fever, common cold, coughing. Sometimes, they got burns from fire or from hot water. They got medications when they are burning up or antibiotics.
**I: You mentioned on the paper that they also get pinkeye and diarrhea.**
R: Yes, they do too.
**I: Now, we’re going to talk about the illnesses. If a child got a fever, what are some of the causes for the fever?**R: If a child catches a cold and if the child is breathing unclean air such as dust.
**I: Do you think fever is very dangerous to a child’s health?**

R: Yes, very dangerous. They get seizures, fever, deaf.
**I: If they have a high fever…**
R: Yes, when they have a high fever, they have seizures. That’s when we are told to soak them in cold water.
**I: So, if they have a high fever, that’s why some go deaf.**
R: Yes
**I: How can you prevent a child from getting a fever?**
R: They would put damp towels to help ease the fever. They also can get medications to help ease the fever.
**I: What about coughing? What causes the child to cough?**
R: If the weather is too windy or rainy, then my child sometimes would start to cough.
**I: What about the other children on this island?**
R:…Sometimes, they get it (coughing) from others.
I: Yes
R: Now, do you think coughing is dangerous to a child’s health?
**I: How do you prevent a child from coughing?**
R: We give them cough medicines
**I: From the hospital?**R: Yes, from the hospital.
**I: Can you tell me does it cost you to go see a doctor?**R: Yes
**I: How much?**
R: .50 cents
I: That’s good. Everytime a patient sees a doctor he/she has to pay.
R: Adult pay .50 cents, and children costs .25 cents.
**I: What about emergency cases? Do they pay?**R: They don’t pay.
**I: What about pink eye? What causes a child to have pink eye?**R: Pink eye is contagious. If it’s too dusty and if a child is playing where it’s too dirty that’s when they get their pink eye from.
**I: Do you think pink eye is very dangerous to a child’s health?**
R: Yes, it can cause a child to have permanent damages to his/her eye.
**I: What do you do when your child has pink eye?**
R: Sometimes I use breastmilk on their eyes, or I gave them medications like the eyedrops.
**I: Where do you get these eyedrops?**
R: I get them from the local doctor.
**I: What about diarrhea? What causes a child to have diarrhea?**
R: Unclean drinking water or they don’t handwash before eating and drinking.

**I: Do you think diarrhea is dangerous to a child’s health?**
R: Yes, there are some who have died from diarrhea They have diarrhea and they become weaker and weaker; they lose their appetite.
**I: What can we do to prevent diarrhea?**R: Medicines
**I: How do you know to go see a doctor if your child is sick?**
R: I see that she is very sick and I go bring her to the doctor.
**I: Who do you go see first? Do you bring her to the traditional healer or the doctor? Are there times you go see the traditional healer?**R: They go see the doctor first and afterwards they go see the traditional healer.
**I: Why do you think they do this?**R: I don’t know. They go see a doctor to see if the child has a fever, and then they bring to the traditional healer to check the stomach for cyst.
I: After they’ve gone to see the doctor, then they go see the traditional healer.
R: There are times when mothers give medicines to their children while they go see the traditional healers. They can feel if a child has a cyst in the stomach.
**I: Can you describe any illnesses affecting your children that are associated with nutrition?**R: I don’t think there are any.
**I: Are there illnesses affecting your child due to lack of nutrition in their diet?**
R: Can you repeat your question?
**I: Are there illnesses affection your child due to the lack of nutrient**?
R: Maybe, she lacks proper vitamins and because she’s not eating papaya..
**I: What does she usually eat?**R: She usually eats rice, pancakes, and often she eats papayas when they’re available, which is rare.
I: **We talked a lot about being unhealthy. Could you now describe for me a typical day of someone living a healthy lifestyle, from the time they wake up in the morning until when they go to bed?**R: He/She is active. He/She doesn’t look sickly.
**I: How does a child under 2 look healthy to you?**R: He/she is also active, and the child can look chubby, has good hair, doesn’t have rashes on their skin, the child seems happy.
**I: What about an adult? What are the signs that show that they’re healthy?**
R: They look healthy, active, and happy. They don’t look tired and drowsy.
**I: Let’s now discuss hand washing. Could you describe in detail your family’s hand washing throughout the day?**R: I buy them soap and they wash their hands. Before eating, I wash their hands. After we’re working outside, I wash their hands for them.
**I: When do you use soap during the day to handwash?**
R: Before we eat and after using the toilet. Sometimes when we’re out of soap, I just wash their hands with water.
**I: What is the difference between using water only and between using water and soap?**
R: I feel that using water to handwash doesn’t feel clean. I can still see dirt in my hands and fingernails unlike using water and soap which really can eliminate germs.
**I: What prevents you from washing hands with soap throughout the day?**R: If I’m too tired from work during the day, then I won’t use soap. I will just wash with water and rest for a while. If I see that we’re almost out of soap, I will save the soap just for washing before a meal.
I: **Now I would like you to think back to when you were pregnant. Can you describe your diet when you were pregnant compared to when you were not pregnant?**R: Foods I usually eat when I was pregnant were cooked ramen noodles with rice and jaipo (Marshallese dish which is made of floury dough mix with sugar).
**I: Can you describe your diet when you were not pregnant?**
R: I eat almost anything: crabs, chicken, canned meat, sometimes fish.
**I; what encouraged you to eat ramen and rice during your pregnancy?**R: I really don’t know, all I know is that I was craving those foods. Other foods gave me heartburn.
**I; Who encourage or discourage your eating during your pregnancy?**R: My husband told me to eat foods that are full of vitamins like papayas and pumpkins.
**I: Why do you think he encourage you to eat those healthy foods?**R: He said that it’s good for the baby.
**I: What other foods your husband discourage you to not eat?**R: He wouldn’t like that I eat ramen and corned beef. He said that they’re not good for the baby. He said they’re too greasy and salty. Food that he recommended was fish, but I really dislike fish during my pregnancy.
**I: Who primarily cared for/supported you during your pregnancy?**
R: My husband cared and supported me. He helped me with my chores and also he would bring me food that I craved.
**I: Can you tell me about any supplements you took during pregnancy?**R: Prenatal vitamins
**I: Did you take all the supplements?**

R: Yes**.** The doctor advised me to take them because they’re good for me and the baby.
**I: Did you drink alcohol, smoke or use drugs during pregnancy?
R: No
I: Did you use traditional medicine during pregnancy?**R: No
**I: If you were advised to eat more fruits and vegetables during pregnancy, could you describe what would make this difficult?**R: It’s not hard since my husband is buying them.
I: **Now can you describe your diet when you were breastfeeding?**R: I would eat chicken, canned meat, and fish.
**I: What influenced your diet during breastfeeding?**
R: I would eat mostly fish since eating fish can make breastmilk.
**I: Is it important to eat foods that are good for you?**

R: Yes, it is important since it also affects the baby.
**I: Were there foods that were recommended for you to eat during breastfeeding?**

R: I would eat foods that are available, and if I’m tired of eating those foods then I would eat other foods.
**I: Were there foods that were not recommended for you to eat during breastfeeding?**R: Corned beef, ramen, and colas. I was told not to eat these foods because they’re too salty and they contain a lot of fat. The colas can affect both me and the baby. The cola can damage my kidneys.
**I: Who encouraged or discouraged eating those foods while breastfeeding?**R: My doctor.
**I: After giving birth, could you describe breastfeeding your child throughout the day? H**ow long after giving birth you started breastfeeding?
R: After the baby was born and cleaned, I breastfed her right away.
**I: Did you squeeze out the colostrums?**

R: No, I did not.
**I: Why didn’t you squeeze out the colostrums?**

R: My doctor told me that the colostrum is good for the baby.
**I: Did you give other liquids to the baby after you’ve conceived?**
R: Yes, this was for the traditional medicine.

**I: What was this for?**

R: It’s called “uno in kijon kan”.
**I: How did you administer this “uno in kijon kan”?**
R: I gather these plants near the beach wash them, pound them. I would add little water to the plants, and squeeze out 3 droplets a day for 3 days.
**I: Do you use boiled water?**

R: No**. I** got it from the water catchment

**I: Why do you give traditional medicine to the baby?**

R: This will help the baby since some babies have nonstop coughs. Other times, they may have diarrhea. Some have the yellowish color in their eyes.
**I: Do the traditional medicine work?**

R: Yes, they stop coughing, and the diarrhea stops.
**I: What makes it easy or difficult to breastfeed exclusively up to 6 months?**
R: It wasn’t difficult to breastfeed since I don’t have a job. I stay with them and care for them.
**I: What would make it easy or difficult to breastfeed up to 2 years?**R: There won’t be a problem breastfeeding since I’m watching over them and taking care of them.
I: **Could you tell me when you first gave foods and/or liquids other than breastmilk to your child? Why?**R: I started feeding her soft foods when she was 7 months old. My husband and I felt like it was time for the baby to eat other foods.
**I: What about others?**
R: Some feed their baby when they’re 5 and 6 months old.
**I: Why do you think people have different views in feeding their babies in different months?**
R: There are some babies who would want to try other foods. For example, if everyone was eating around a baby, the baby would then cry and want to try the food everyone is eating.
**I: What were some of the foods that were given to the baby and how were they prepared?**
R: I would wake up early in the morning to prepare the pumpkin. I would grate them and boil them. After it’s soft and cooked, I would add flour. If there was baby cereal, I would give the baby the cereal for breakfast. Sometimes when there are pandanus available, I would cook them and prepare them for the baby to eat. After the baby has eaten her breakfast, I would prepare the pandanus for the baby to eat after lunch and for dinner.
**I: We are trying to understand how people eat in this community. Could you describe in detail what your family usually eats and drinks throughout the day?**R: **For breakfast** we would eat pancakes or bread. We drink coffee if there’s any. For lunch and dinner, we usually eat rice.
**I: You mentioned coffee, do the children drink coffee too?
R:** Sometimes, but we make the coffee lighter.
I; **When did she first drank coffee?**R: When she was one year**.
I: Can you describe how the meals are made?**R: I would prepare breakfast the night before. When it’s daytime, it’s when I prepare lunch. After everyone has their breakfast, I now will begin to prepare for lunch.
**I: Do you cook on fire or on stove?**R: I cook on the fire.
**I: You mentioned earlier you usually make pancakes. What are your ingredients?**R: Just sugar, water, and baking powder.
**I: Who in your family is served first, next, last?**R: I usually serve children first, then the workers, and I’m the last one to serve.
**I: Are there differences in the foods served to different family members?**R: Everyone eats the same meal. Adults have larger portions of food than children. Each person is served with their own plate but my baby and I share the same plate.
I: **Now could you describe any food sharing between family members during mealtimes (for example children eating together separately from the family, meals eaten from the same plate by all family members)?**R: After I cooked dinner, I served everyone. After everyone is served, I gave a plate to our neighbor everytime.
I: **We have heard from some families that eat local foods whereas others eat processed foods. Could you explain what is typical for your family?
R:** We usually eat processed foods.
**I: Why is it difficult for you to cook local foods?**R: Sometimes they’re not available; pandanus and breadfruits come in season. It takes a lot of time and effort to make local food. For example, it takes time to bring in the fish from the ocean.
**I: Can you tell me any positive or negative things about eating local foods?**R: Local foods are delicious, nutritious, and fresh. Negative things about local foods are that it takes too long to prepare.
**I: Are there any positive or negative things about eating processed foods?**R: Positive things about processed foods are that they’re readily available. Negative things about processed foods are old, not fresh. They have high sodium, high fat, and very expensive.
**Now that we’ve talked about how the family eats, I would like to learn more about how your child eats. Could you describe in detail what your son/daughter under 2 years commonly eats throughout the day?
R:** She usually eats what the whole family eats. She eats bread, coconut apples.
**I: How many times a day meals (and snacks) are eaten by your child under 2?**R: 4 times
**I: When does she usually have her snack?**R: After she eats a meal, sometimes I don’t give her snack.
**I: Why not?**R: When I’m too busy I don’t make snacks. But, when I’m not busy, then I will make and give her snacks. Sometimes I give her coconut apples for snack.
**I: How do you know the young child has had enough to eat?**
R: She eats and then when she has enough she refuses to eat more. **I: What do you encourage the child to eat?**R: Healthy foods. **I: What do you encourage the child to eat if the child refuses?**
R: I wait a while and feed her again. I look for other food that she wants to eat.
**I: Do you feed the child differently when the child is sick (eg. when child has diarrhea) (and reasons why)**
R: I look for other food to eat. If she still refuses, I would breastfeed and give her water.
**I: You’ve told me what your child under 2 usually eats. Now could you explain to me the process, from start to finish, how you prepare and cook a meal for your child?
R:** For breakfast, I would tell my husband to look for 2 coconut apples. In the morning I would husk it, grate it and cook it with flour, put a little sugar, and add coconut milk.
I: **Could you now tell me what you think are important foods for children under 2 years to grow well/be healthy?
R:** She has to have a balanced diet. She needs to eat proteins to grow and body building foods. **I; Can you give us examples?**R: Fish and fruits and vegetables. Sometimes, we can’t provide these foods to the child because as I say before, a local fruit comes in season. My husband doesn’t fish and we buy fish if we have money. If we don’t have money we made credits in the store and the store takes our copra in exchange for the food we buy in the store. For meats, if no other foods are available; our main food is rice and canned meat. We know that we have to feed our kids from the 3 food groups, but we lack these foods.
**I: What foods should not be given to children under 2 (and reasons why)?**R: Chips and candies.
**I: Why not?**R: They’re bad for the kids. They’re junk foods.
**I: What is the biggest influence on feeding their child(ren)?**R: My husband would ask if I’ve handwash the kids, and also he ask if the children had meat in their meals.
I: **Can you describe any differences (if any) between how you feed your male children and how you feed your female children under 2?**R: I only have girls**.**I: **Could you describe the care of children throughout the day in your community?**R: My neighbors usually watch my kids when they go play near their house or they watch them in case they go to the beach. They would send them home if they see them in the beach.
**I: Who is mainly responsible for child care?**
R: My husband and I.
**I: What are your responsibilities of mothers in child care?**R: I would care for them, feed them, bathe them, make sure meals are prepared before every mealtime, and wash their clothes.
**I: What are the responsibilities of fathers in child care?**
R: He provides for their needs.
**I; How caregivers play with children under 2?**R: Sometimes I watch the baby play, other times I play with her, tickling her.
**I: Could you talk about the role of grandparents have in raising children in this community?**R**:** Our parents are in Majuro and we don’t see a lot of them.
**I: What if they come here, what are their roles?**R: They can watch over them while I go and do chores.
**I: What makes good grandparents (grandfather/grandmother)?**
R: Grandparents watch over the children. Sometimes they feed the kids and play with them.
I: **Could you talk about the role that other family members have in raising children in this community?
R:** We don’t see a lot of them, and my kids stay with me and their dad.
**I: What about older siblings?**
R: The older sibling can watch the baby or she can bathe and clean the baby. She can also babysit.
I: **Could you explain where you usually get trusted information about nutrition and health?
R:** When I go see the doctor, that’s how I get the information.
**I: Why do you trust these sources?**
R: It’s because I trust my doctor.
I: Where nutrition and health messages should be delivered so that you would see/hear them most easily?
R: My parents should have told me and taught me about nutrition.
**I: What types of media that the person uses the most to communicate (eg. radio, online apps, and websites)?**R: Radio
**I: When you think about your own parenting behaviours, can you explain what influences how you raise your children?**R: I raised my kids the way I know how and what’s worked.
**I: How are your parenting behaviors any different from other mothers in the community?**
R: There are some mothers who really take care of one kid rather than all of their kids. There may be favoritism among the children where some mothers tend to keep foods only for the ones they favor.
**I: And you don’t show favoritism?**
R: I don’t. What I feed everyone is the same, the older kid and the baby.
**I: How opinions of the community influence how they raise their children (e.g. leaders, neighbours, church leaders, health workers)?**R: They would say that some kids have bad conducts, and parents should teach their kids to be well mannered when they are around other people. **I: Were there any advice or information related to parenting you received?**
R: Nothing.
**I: Do you have any desired information on parenting you wish you had but don’t have available?**R: I would like to know more about parenting and family planning.
I: **Is there anything else about the topics we talked about today that we missed or that you would like to tell us about?**  *R:* I think we covered them all.
I: Thank you again for your time and for all the information you gave us.
