# Supplementary material for: Conceptualizing multi-level determinants of infant and young child nutrition in the Republic of Marshall Islands–a socio-ecological perspective
Source: PLOS Glob Public Health. 2022 Dec 19;2(12):e0001343. doi: 10.1371/journal.pgph.0001343 (PMC10022247; doi:10.1371/journal.pgph.0001343)
Supplement: S1 Data — (ZIP) [file pgph.0001343.s001.zip › RMI Supp Data/Interviews data/I45R_IDI_FCG_Arno_Sep 14_Meia.docx]

I45R_IDI_FCG_Arno_Sep 14_Meia

Interview Code: 145R

Interview type and Interviewer: FCG Meia

Interview Date: September 14, 2018

Location: Arno

Transcriber: Joie Heine

**I: Are you willing to participate in an interview?**

R: Yes

I: Thank you very much. **To begin with, can you please tell me a little about your family/household? How many people are living here and the number of children living in this household? Please show us their gender and ages as well.**R: There are five adults and four children including an infant who is almost 1 month. I have a kid who is one year old.

**I: Next, I’d like to ask you to describe your community:**R: Like what?

**I: Are there positive and negatives in this community?**
R: There aren’t any negative things. Some positive things are people are making copra for a living. Also, people are making coconut fronds for handicrafts.

**I: Are there any challenges here?**

R: I don’t think there’s any.

**I: Do you have plenty drinking water?**

R: Yes, we do.

I: Now we’re going to talk about health and illnesses in your family*.* **Can you tell me about some of the illnesses that your children have suffered from?**R**:** Some of the illnesses include fever, coughing, common cold?

**I: What causes the child to have fever?**
R: If the weather is cold and if he’s in the water playing or bathing frequently, that’s when he catches a fever.

**I: Do you thing fever is dangerous to the child’s health?**

R: Yes, the fever can trigger his wheezing.

**I: What are some of the ways to prevent coughing?**

R: I gave the baby cough medicine from the doctor.

**I: What causes the child to cough?**R: If it’s too windy and he stays outside for a long time while it’s windy, he can start to cough.

**I: Do you think coughing is dangerous to a child’s health?**

R: If he’s coughing, he will have a fever which then can lead to him wheezing.

**I: What can you do to prevent the baby from coughing?**
R: I would also give him medicine from the doctor.

**I: What about the runny nose? Do you think runny nose is dangerous to a child’s health?**

R: No

**I: What do you do to prevent him from having the runny nose?**

R: Sometimes, it’ll go away on its own. Other times I give medicine for fever and coughing.

I: **Can you describe how you know when your child needs treatment for their illness?**

R: If I see that he’s really sick, I take him to the doctor.

**I: Do you use traditional medicine when he’s sick?**

R: I don’t.

**I: Who do you first go to for healthcare (and reasons why)**

R: The doctor.

**I: Can you describe any illnesses affecting your children that are associated with nutrition?**

R: Nothing

**I: What about any illnesses caused by foods missing from his diet?**

R: I don’t think there are any.

I: **We talked a lot about being unhealthy. Could you now describe for me a typical day of someone living a healthy lifestyle, from the time they wake up in the morning until when they go to bed?**

R: He/She is active; he/she looks clean

**I: What about appearance/signs of a healthy child under 2 years?**

R: He/she is chubby, likes to eat, looks clean

**I: What about appearance/signs of a healthy adult?**

R: They don’t look sickly, they’re active.

I: **Let’s now discuss hand washing. Could you describe in detail your family’s hand washing throughout the day?**R: Sometimes they wash their hands and sometimes they don’t. But before a meal, they wash their hands.

**I: Why do you think they wash their hands and sometimes they don’t wash?**

R: They’re too lazy.

**I: How does the children hand wash throughout the day?**

R: We wash their hands after playing and before they come inside the house.

**I: What children under 2? Who’s washing their hands throughout the day?**

R: The mothers wash their hands with soap and after I use hand sanitizer

**I: What is the difference between using water only or water and soap to wash hands?**

R: It’s not really clean when you only wash with water. Washing hands with water doesn’t kill germs and bacteria.

**I: What prevents you from washing hands with soap throughout the day?**

R: If I’m too tired and lazy, I won’t use soap. *.*

**I: Now I would like you to think back to when you were pregnant. Can you describe your diet when you were pregnant compared to when you were not pregnant?**

R: During my pregnancy, I would eat ramen, chips, and drink juice, rice, sashimi,

**I: What influenced you to eat those foods during your pregnancy**?

R: I don’t know. Maybe the baby wants those food.

**I: Were there foods you were encouraged to eat during pregnancy (and reasons why)?**

R: They would encourage me to eat apples, oranges, local foods which are good for the baby.

**I: Were there foods you weren’t encouraged to eat during pregnancy (and reasons why)?**

R: They would say that salty foods aren’t good for the baby.

**I: Who encouraged or discouraged eating those foods during pregnancy**?

R: The baby’s father would discourage eating salty food.

**I: Who primarily cared for/supported her during her pregnancy?**

R: The baby’s father cared and supported me. He would take care of my needs.

**I: Can you tell me about any supplements you took during pregnancy?**

R**:** I took prenatal vitamins.

**I: Did you take all the supplements given to you (and reasons why or why not)?**

R: I took all the supplements because it’s good for me and the baby.

**I: Did you drink alcohol, smoke or use other drugs during pregnancy?**

R: NO, I did not.

**I: Did you use any traditional medicines taken during pregnancy (and reasons why)?**

R: No

I: **If you were advised to eat more fruits and vegetables during pregnancy, could you describe what would make this difficult?**

R: When I was pregnant, fruits and vegetables made me want to throw up.

**I: What would make it easier to eat more fruits and vegetables (and reasons why)**R: They are delicious and healthy.

I: **Now can you describe your diet when you were breastfeeding?**

R: I would sashimi, fish, rice, chicken

**I: What influenced your diet during breastfeeding?**

R: I would eat so I would have milk to breastfeed.

**I: What foods were you encouraged to eat during breastfeeding (and reasons why)?**

R: I was encourage to eat fish because it makes more milk for breastfeeding.

**I: What foods you were encouraged not to eat during breastfeeding (and reasons why)**

R: I was discouraged to eat salty food because it’ll make my breastmilk salty and empty.

**I: Who encouraged or discouraged eating those foods while breastfeeding?**

R: My grandparents and the baby’s father would discourage me from eating salty foods.

I: **After giving birth, could you describe breastfeeding your child throughout the day?**

R**:** After I gave birth, I started breastfeeding right away.

**I: Did you squeeze out colostrum when you breastfed?**

R: No, I didn’t. My doctor told me to breastfed right away.

I**: Did you give other liquids to the baby in the first few days after birth (and reasons why)?**

R: No.

**I: What about the older child? Did you use traditional medicine?**

R: Yes, I use the traditional medicine which is “uno in kijon kan”. It’s to stop the baby from crying a lot.

**I: How do you make the traditional medicine?**

R: I use these plants, wash them, pound them, and put a little bit of unboiled water from the water catchment.

**I: How long do you give the traditional medicine?**

R: 3 weeks. I put a few drops of water in the baby’s mouth.

**I: What makes it easy or difficult to breastfeed exclusively up to 6 months (and reasons why)?**

R: It’s not difficult. I am always available if he wants to breastfeed.

**I: Did you wean him?**

R: I stopped breastfeeding him after his first birthday. I stopped because he bites my breasts.

**I: Could you tell me when you first gave foods and/or liquids other than breastmilk to your child?**

R: I gave him food when he was 6 months old.

**I: Why did you introduce foods or liquids other than breastmilk at that age?**R: It was time for him to eat food.

**I: Were there opinions from others that influenced your decision to introduce foods and liquids at that age?**

R: There was none.

**I: What were the first foods and how were they prepared?**
R: I bought baby cereals from the store.

I: **We are trying to understand how people eat in this community. Could you describe in detail what your family usually eats and drinks throughout the day?**

R**:** They eat coconut apples cooked with rice, coconut apples cooked with flour, breadfruits, rice fish. For breakfast, they would eat pancakes and drink water.

**I: How are the meals made?**

R: We cook coconut apples with rice add a little sugar and some coconut milk. For the coconut apples with flour, we add flour, sugar, water, and coconut milk.

**I: Who in the family is served first, next, last?**

R: Children are served first and adults last.

**I: Are there differences in the foods served to different family members?**

R: There is none.

**I: Are there any differences in quantities of food served to different family members?**

R: No

**I: Do some children receive more food than others?**R: Some may have more than others.

I: **Now could you describe any food sharing between family members during mealtimes (for example children eating together separately from the family, meals eaten from the same plate by all family members)?**

R: Children have their own plates.

**I: Do you share food between households (eg. sharing food with neighbours)**
R: Yes, sometimes we do.

**I: We have heard from some families that eat local foods whereas others eat processed foods. Could you explain what is typical for your family?**

R**:** We eat rice and canned meat.

**I: What makes it difficult or easy to cook local foods?**

R: I can’t think of any.

**I: Are there any positive or negative things about eating local foods?**

R: There’s none on negative things. Positive things are they’re nutritious

**I: Are there any positive or negative things about eating processed foods?**

R: Negative things about processed foods are that eating them can lead to diabetes.

I: **Now that we’ve talked about how the family eats, I would like to learn more about how your child eats. Could you describe in detail what your son/daughter under 2 years commonly eats throughout the day?**

R**:** They usually eat rice and canned meat, and drink water.

**I: How many times a day meals (and snacks) are eaten by children under 2?**

R: My kids don’t eat snacks.

**I: How do you know the young child has had enough to eat?**

R: He eats and refuses to eat more.

**I: What do you do to encourage the child to eat if the child refuses?**

R: I gave her candies.

**I: Do you feed the child differently when the child is sick (eg. when child has diarrhea) (and reasons why)**

R: No, I don’t.

I: **You’ve told me what your child under 2 usually eats. Now could you explain to me the process, from start to finish, how you prepare and cook a meal for your child?**

R: I cook it and serve it.

**I: Could you now tell me what you think are important foods for children under 2 years to grow well/be healthy?**

R: Important foods are the ones that are nutritious and healthy.

**I: Are there foods that should not be given to children under 2 (and reasons why)?**

R: Junk foods and uncooked ramen noodles

**I: What is the biggest influence on feeding you children?**

R: There is none.

I: **Can you describe any differences (if any) between how you feed your male children and how you feed your female children under 2?**

R: There is no difference.

**I: Could you describe the care of children throughout the day in your community?**

R: My neighbors

**I: Who is mainly responsible for child care?**

R: I am.

**I: What are some of your responsibilities as a mother in child care?**R: I watch over them and take care of them.

**I: What are some of the responsibilities of the fathers in child care?**R: Fathers take care of their needs.

**I: How caregivers play with children under 2?**

R: I play with him.

I: **Could you talk about the role of grandparents have in raising children in this community?**

R: They take care of them just the same way I care for them.

**I: What makes good grandparents (grandfather/grandmother)?**

R: I don’t know.

I: **Could you talk about the role that other family members have in raising children in this community?**

R: They watch over them.

**I: What are some of the ways that siblings (older siblings) help raise young children?**

R: The older sibling can also watch over the young child.

*You are doing a great job. We are almost finished. Now for the last section, we would like to learn about ways we can develop health programs in your community.*

**I: Could you explain where you usually get trusted information about nutrition and health?**

R: I get them from my own household, from other family members.

**I: Why do you trust these sources?**

R: They’re the ones I look up to and I also learned from them.

**I: Where nutrition and health messages should be delivered so that you would see/hear them most easily?**R: I don’t know.

**I: What types of media that the person uses the most to communicate (eg. radio, online apps, and websites)?**

R: Nothing.

I: **When you think about your own parenting behaviours, can you explain what influences how you raise your children?**

R: There is no difference.

**I: How opinions of the community influence how they raise their children (e.g. leaders, neighbours, church leaders, health workers)?**

R: I don’t know.

**I: Did you received any advice or information related to parenting?**

R: My mother gave my advice.

**I: What were the advices?**

R: She told me to really take care of them and watch over them.

**I: Do you have any desired information on parenting you wish you had but doesn’t have available?**

R: No.

**I: Is there anything else about the topics we talked about today that we missed or that you would like to tell us about?**

R: No.

I: Thank you for your time.
