# Supplementary material for: Conceptualizing multi-level determinants of infant and young child nutrition in the Republic of Marshall Islands–a socio-ecological perspective
Source: PLOS Glob Public Health. 2022 Dec 19;2(12):e0001343. doi: 10.1371/journal.pgph.0001343 (PMC10022247; doi:10.1371/journal.pgph.0001343)
Supplement: S1 Data — (ZIP) [file pgph.0001343.s001.zip › RMI Supp Data/Interviews data/I46R_IDI_FCG_Arno_Sep 14_Meia.docx]

- Interview code: I46R
- Interview type and interviewee: IDI_FCG
- Interview date: Sept.14.18
- Location: Arno
- Interviewer: Meia
- Transcriber: Marcellina

**I: hi, do you want to do this interview this afternoon?**

R: yes

**I: Thank you for giving us your time to speak with us today. The information we learn here will help us find ways to improve maternal and child health and sanitation in your country. To begin with, can you please tell me a little about your family/household?**

R: regarding what?

**I: like who lives in this household, how many children and their ages?**

R: well me and my husband, another two couples, our mother, my children and the couple’s children

**I: how old are they?**

R: there are 13 years old, 6 years old-

**I: what about your children?**

R: 1 year old and 6 years old

**I: and how many girls and boys in this household?**

R: 3 girls and 1 boy

**I: okay thank you. Now can you me a little about your community?... what are the good or the bad things about this community? If you would love to describe it to me.**

R: it’s good because we’re feeling good staying in it

**I: okay and what makes you feel good about it?**

R: there are local foods and our lives depend on it

**I: regarding what?**

R: money; when we make copra we’ll have money and we don’t have to buy drinking water because we have water tanks

**I: okay thank you. Let’s now talk about health and illnesses in your family. Can you tell me about some of the illnesses that your children have suffered from?**

R: seems like our children haven’t got any illnesses

**I: what about the illnesses you give in the free list we did? Can you explain them?**

R: oh! Sicknesses like fever, skin rashes, boil, and swollen stomach

**I: now can you tell me the causes of fever?**

R: when they’re starting to have teeth and when they fall down and have swollen stomach.

**I: now do think fever is a serious illness for your baby’s live?**

R: yes. Because if they have fever we won’t get a good sleep because we have to prevent them from having high fever by giving them medicines.

**I: what about boil? What are the causes of boils?**

R: because of malnutrition

**I: now why would you say malnutrition?**

R: because I am referring to our daughter. When I was pregnant with her, I have all the supplements they gave me and she doesn’t get illnesses commonly. Unlike our son, lots of illnesses he encounters because I didn’t take the supplements.

**I: okay good. Now do think boil is serious to your baby’s live?**

R: not really because it’ll be gone anyway.

**I: what do you do to get rid of it or prevent it from happening?**

R: I apply coconut oil and massage it so it can be open up.

**I: what about running nose? What are the causes of running nose?**

R: because of catching cold

**I: now do you think running nose is a serious illness?**

R: not really

**I: okay. And what can you do to prevent running nose?**

R: nothing. It’ll be gone anyway too

**I: what about skin rash, what are the causes of skin rash?**

R: it’s from being unclean

**I: now do you think skin rash is a serious illness?**

R: not really but my son doesn’t know what to do when his skin rashes are itchy.

**I: okay and how do you treat the skin rashes?**

R: I bath him with local medicines

**I: what kind of local medicines?**

R: WUT KONAMNAM (local tree). I boil the leaves until the water turns really green and then, pour it into a tub and let him soak in it.

**I: okay good. Now, can you describe how do you know when your child needs treatment for their illness?**

R: when they have fever

**I: okay. Who do you seek first when your child is sick and why?**

R: the doctor to prevent it from getting worse

**I: okay. Do you use traditional medicines when your child has fever?**

R: no I don’t

**I: Can you describe any illnesses affecting your children that are associated with nutrition?**

R: seems like there are none

**I: none? And what the illnesses caused by foods missing from the diet?**

R: boil in the head

**I: boil in the head, good. Now, we talked a lot about being unhealthy. Could you now describe for me a typical day of someone living a healthy lifestyle, from the time they wake up in the morning until when they go to bed?**

R: when they wake up they clean outside of their house, make copra, doing laundry…

**I: what else you see in a healthy person? You mentioned cleaning, doing laundry.. What are the other things?**

R: seems like nothing more

**I: is it right that a healthy person should look clean and fit?**

R: yes

**I: well that is. What about a child? What are the appearances of a healthy child?**

R: they walk a lot

**I: are there anything else they do beside- do they like to sleep a lot or do they always keep smiling-**

R: sometimes they sleep at day time

**I: what about an adult? What are the appearances of a healthy adult?**

R: they work a lot

**I: okay. Let’s now discuss hand washing. Could you describe in detail your family’s hand washing throughout the day?**

R: we wash our hands before we eat and after we’re done with cleaning or with chores.

**I: what about the children? How do they wash their hands throughout the day?**

R: we help them wash their hands. When they’re done playing, we take them and give them a bath.

**I: what about the child under 2 years of age?**

R: we wash their hands

**I: okay. Now can you tell me when do you use soap to wash your hands during a day?**

R: like I mentioned before. After I am done with chores and cleaning.

**I: now, can you describe how do wash your hands?**

R: I apply soap in my hands and then rinse them.

**I: okay. Now what you thinks is the difference between using water only or water and soap to wash hands?**

R: we use water in both

**I: I am asking about the difference between washing hands with water only and washing hands with soap and water?**

R: oh! If you wash your hands with water only, the germs will be still there in your hands.

**I: okay. Can you tell me what prevent you from washing your hands with soap?**

R: when I am having my period I don’t use the soap

**I: okay when you’re having your period. Are there anything else?**

R: nothing

**I: every time you have soap right? There is not a time when there is no soap?**

R: every time we have soap.

**I: okay thank you. Now we would like to talk about your diet during pregnancy and breastfeeding. Would you think back to when you were pregnant? Can you describe your diet when you were pregnant compared to when you were not pregnant?**

R: when I am pregnant I usually eat rice, luncheon meat (canned meat) and fish.

**I: what about when you’re not pregnant?**

R: well, I eat every kinds of food.

**I: now what made you eat the foods you ate during pregnancy?**

R: I craved for them

**I: okay. What foods they encouraged you to eat during pregnancy?**

R: seems like none-

**I: no one told you eat whatever food they encouraged you to eat?**

R: nobody

**I: okay. And why didn’t they tell you eat whatever foods they want you to eat?**

R: I don’t know (laughing)

**I: okay it’s all good. Now what foods they encouraged you not to eat during pregnancy?**

R: salty foods like Kool Aid and salt.

**I: now why did they encouraged you to not eat salty foods?**

R: they said they’ll cause problem

**I: now who encouraged you not to eat these?**

R: my parents and my husband

**I: okay thank you. Who took care of you during your pregnancy?**

R: my husband and my family

**I: and how did they help taking care of you during your pregnancy?**

R: they helped me with my chores like bring water for washing dishes. And help me carry my bathing water.

**I: okay what about your needs? Do they also help you with?**

R: yes

**I: okay thank you. Can you tell me what kind of supplements you took during pregnancy?**

R: pregnancy supplements only

**I: are there any others?**

R: none

**I: now did you take all the supplements given to you?**

R: some I take some I don’t

**I: which one you take and which one you don’t?**

R: I take the folic-acid pills and I don’t take the vitamins pills.

**I: why didn’t you take the vitamins pills?**

R: because they make feel nausea

**I: and what about folic-acid pills? Did you complete taking them?**

R: no I didn’t

**I: why?**

R: I hate it too but I tried my best

**I: now how long you been taking the supplements? Or at what month of pregnancy you stopped taking the supplements?**

R: I started taking the supplements when I was 8 months old pregnant because that’s the time I decided to see the doctor. And I stopped when I was 10 months old pregnant.

**I: okay thank you. Did you drink alcohol, smoked, or used other drugs during pregnancy?**

R: I didn’t

**I: were there any traditional medicines you took when you were pregnant?**

R: no there were none

**I: good. Now I want to know, why you didn’t took any traditional medicines during pregnancy?**

R: because I didn’t want to take any

**I: okay good. If you were advised to eat more fruits and vegetables during pregnancy, could you describe what would make this difficult?**

R: I hated fruits and vegetables

**I: you hated fruits and vegetables. Are there any more difficulties? Were you able to buy them every day?**

R: yes

**I: okay. The only thing was, you hated them. Right?**

R: yes

**I: Now can you describe your diet when you were breastfeeding?**

R: I ate rice, crab, fish, corned beef, and mackerel

**I: what really made you eat these foods?**

R: so that I can have enough breastmilk

**I: okay. And what kind of foods they encouraged you to eat during breastfeeding?**

R: usually crab and fish

**I: and why they did preferred crab and fish only?**

R: because it’s easy for them to get. Not like the canned foods.

**I: so you’re saying that they can get them because they don’t cost money not like the canned foods right?**

R: yes

**I: okay thank you. Now what foods they encouraged you not to eat during breastfeeding?**

R: salty foods

**I: why didn’t they want you to eat salty foods?**

R: because the breastmilk will have salty taste

**I: okay. Who encouraged you not to eat salty foods during breastfeeding?**

R: my husband

**I: After giving birth, could you describe breastfeeding your child throughout the day?**

R: I breastfeed when he/she cries

**I: okay. Now did you squeeze out the first milk after giving birth?**

R: no I didn’t

**I: and why didn’t you squeeze it out?**

R: I don’t know

**I: nobody tell you anything?**

R: no

**I: okay good thank you. Now how long after giving birth you started breastfeeding your child?**

R: it took hours for me to start breastfeeding

**I: and why did you wait for hours to start breastfeeding?**

R: because they were cleaning or baby bathing so I waited

**I: oh okay. Did you give bottle milk or other liquid to your baby after giving birth?**

R: I didn’t give bottle milk

**I: what about any other liquid? Like the traditional medicines for example.**

R: no

**I: you didn’t use traditional medicines for your baby after he/she was born?**

R: oh! I did

**I: well?**

R: well I let him drink the traditional medicines called KIJON KAN (infant natural illness)

**I: now where did you take the traditional medicines and how did you make it?**

R: I took it from anywhere and bring it and bounce them all together.

**I: do you wash it?**

R: yes I do. I wash it before I bounce it and then give it to my child

**I: now the water you made from the medicines, do you boil it before you give it to your child?**

R: no I don’t. I give it right after it’s done

**I: okay good. Now were there any difficulties for you to breastfeed your baby up to 6 months? Is your child still breastfeeding?**

R: I stopped breastfeed him after he has his first birthday

**I: okay why did you stopped breastfeed him after he has his first birthday?**

R: because my breasts were hurting

**I: now was it difficult- did you breastfeed him until he was 6 months old?**

R: yes

**I: now could you tell me when did you first gave foods or liquids other than breastmilk to your child?**

R: nothing. He only drank water.

**I: when did he start eating?**

R: when he was 9 months old

**I: good. Now why did you start give him foods at that age?**

R: I gave him rice

**I: he didn’t eat any other foods?**

R: he did. But I usually feed with rice at 9 months old

**I: what about the very first foods? I am talking about the very first foods you started feeding your child with? What month you first start giving foods to your child?**

R: at 6 months old

**I: okay and why did you start giving foods to your child at 6 months old?**

R: because I think it is time for him to start eating foods

**I: okay and who told you that it was time for him start eating foods?**

R: me because I know

**I: now what are the opinions from others that influenced their decision to introduce foods and liquids at that age?**

R: they all started giving foods at 5 months old

**I: what were the first foods and how they were prepared?**

R: soft foods

**I: soft foods? How did you prepared soft foods?**

R: I wake up in the morning and cook them

**I: yeah like how do you cook them? What was the first soft food?**

R: MAKWON (pandanus paste)

**I: okay now how did you prepared the makwon?**

R: first we boiled the pandanus pieces and when they are fully cooked, we cool them down and then rubbed out the pandanus paste using spoon or pandanus grating. And then mixed the pandanus paste with water

**I: do you add sugar to it?**

R: little sugar and coconut milk and give it to him

**I: good. Now we are trying to understand how people eat in this community. Could you describe in detail what your family usually eats and drinks throughout the day?**

R: we eat in the morning, noon, and evening

**I: what do your family eat?**

R: for breakfast we eat pancake, drink Kool-Aid or coffee. For lunch we eat rice with meats and drink the same drink in the morning.

**I: does your child also drink coffee?**

R: yes he does

**I: when did he start drinking coffee?**

R: when he was 1 year old

**I: okay, and he’s still drinking coffee until now?**

R: yes

**I: good. Now can you explain how your family prepare the meals during the day? As for the pancakes, how do you prepare it, how do you prepare the lunch, and how do you prepare the dinner?**

R: as for pancakes, we mix it

**I: mix it with what?**

R: mix it with sugar, baking powder,… that all

**I: okay. What about the lunch meal?**

R: rice

**I: rice? Who in the family is served first, next, last?**

R: the children serve first and then us; the adults after.

**I: are there any differences in the foods served to different family members?**

R: no. there are no differences

**I: what about differences in the amount? Are there any differences in quantities of food served to different family members?**

R: it depends on how much they desire because everyone serve themselves

**I: okay. Are there some children receive more food than others?**

R: yes there are. As for my son, he eats more than my daughter

**I: okay good. Now could you describe any food sharing between family members during mealtimes? For example children eating together separately from the family, meals eaten from the same plate by all family members?**

R: separately. One child with its own plate.

**I: now do your family share foods to the neighbours?**

R: yes

**I: every time your family share foods?**

R: yes. When there are foods like chicken, fish, foods that are plenty to share

**I: now we have heard from some families that eat local foods whereas others eat processed foods. Could you explain what is typical for your family?**

R: we usually eat breadfruits, rice, fish, iu (coconut cotton), local chicken and pork

**I: okay. So you are saying that your family eat both local foods and processed foods as well.**

R: yes

**I: what does make it easy for you to cook local foods?**

R: it’s easy because- as for local chicken, we just grab it and kill it.

**I: you don’t have to buy it right?**

R: yes

**I: so you’re saying that it’s easy because there a lot right?**

R: yes

**I: okay. What are the good things about local foods?**

R: they are good because they have vitamins

**I: are there any bad things about local foods?**

R: seems like none

**I: now what are the good or the bad things about processed foods like rice or canned foods?**

R: as for rice, it causes pain in the legs. And as for chicken, if you don’t fully cook it then it will cause sickness.

**I: what about the children? What are the good or the bad things for them about processed foods?**

R: we have to- as for chicken, it must be fully cook because it can cause sickness for our children. And as well as hot dogs, it has to be also fully cook.

**I: what is wrong with hot dogs?**

R: they said it’s not good

**I: why is it not good?**

R: they said it’s poisonous. You know before you cook it, you have to take the hotdogs out of their plastic bag.

**I: which one is poisonous, the plastic bag or the hotdogs?**

R: the plastic bag

**I: so the hotdogs are good but the plastic bag it’s not good right?**

R: yes

**I: okay good thank you. Now that we’ve talked about how the family eats, I would like to learn more about how your child eats. Could you describe in detail what your son/daughter under 2 years commonly eats throughout the day?**

R: they eat the same foods we eat

**I: okay good. Good. Thank you. Now how many times a day your children under 2 eat their meals and snacks?**

R: only 3 times a day

**I: how do you know your child has had enough to eat?**

R: they just don’t want to consume more

**I: good. What can you do when your child doesn’t eat?**

R: I give him water and sweet water

**I: and what can you do to get your child to eat when he/she refuses to eat?**

R: if they refuse to then I just let them be

**I: are there any differences on how you feed your child when he/she is sick? Diarrhea for example. Are there any differences in feeding your child when he/she has diarrhea?**

R: when they are sick, they don’t want to eat anything because they feel nausea.

**I: You’ve told me what your child under 2 usually eats. Now could you explain to me the process, from start to finish, how you prepare and cook a meal for your child?**

R: before we prepare it we have look for a way to do it like bring it. For example aikiu (iu mix with flour), we have to find some iu in the jungle, take out the cotton inside, slice them all together and then boil them. When they are fully cook, then we add flour to it and let it cook for another 5 minutes.

**I: okay thank you. Could you now tell me what you think are important foods for children under 2 years to grow well/be healthy?**

R: they have to eat the foods that have vitamins like bread, local meats… that’s all

**I: are there anything else besides these your children have to consume?**

R: ramen

**I: ramen? Nothing else?... okay then what kind of food you should not give to your child?**

R: things like salt, soy sauce…

**I: now why should you not give salt or soy sauce to your child?**

R: because they’ll harm them

**I: what is your biggest backup on feeding your children? Do you have any backups on feeding your children?**

R: none

**I: none? Can you describe any differences if any, between how you feed your male children and how you feed your female children under 2?**

R: it seems like there are no difference

**I: no difference? Okay thank you. We are also interested in the roles and responsibilities different family members play in raising children. Could you describe the care of children throughout the day in your community?**

R: they take care of them from any harms, they feed them

**I: are there any more?**

R: seems like no more

**I: okay. Now who is mainly responsible for child care?**

R: my mother and father

**I: what about you as a mother? You’re not responsible for your child care?**

R: oh yes I am

**I: okay. Now what are your responsibilities as a mother to your child?**

R: there are times we discipline them so can be obedient

**I: good.**

R: we teach them

**I: good what else?**

R: and look after them

**I: and what are the responsibilities of the fathers?**

R: support them

**I: now you as a caregiver, how do you play with your child?**

R: make them happy by let them watch movie, play with them-

**I: how do you play with them?**

R: like tickling them

**I: good. Could you now talk about the role of grandparents have in raising children in this community?**

R: they said they’re busy- their parenting time has come to an end (laughing)

**I: oh the grandparents don’t take their part on taking care of the children?**

R: they’ll take care of them only when they’re not busy or something.

**I: okay. What makes the grandparents a good grandparent? What do they do to the children?**

R: they take care of them

**I: how do they take care of them?**

R: like when they’re sick, they support them.

**I: okay. What about other family members? Like you sister or brother for example. Can you tell their responsibilities for the children?**

R: they also look after them when we’re- support them.

**I: what about the sister of your child, how does she take care of his baby brother?**

R: she also take care of him like keep an eye on him when they’re outside the house.

**I: You are doing a great job. We are almost finished. Now for the last section, we would like to learn about ways we can develop health programs in your community. Could you explain where you usually get trusted information about nutrition and health?**

R: from the doctors

**I: from the doctors, why do you trust the doctors?**

R: because they give us good health

**I: okay. Where nutrition and health messages should be delivered so that you would see or hear them most easily?**

R: to the kumit organization

**I: okay kumit. Now what types of media you use the most to communicate?**

R: cell phone

**I: cell phone good. When you think about your own parenting behaviours, can you explain the difference on how you raise your children? Is there a difference on how you parenting?**

R: none

**I: none? Good. Are there any thoughts or opinions from others like the leaders, the neighbours, church leader, or health workers on raising children?**

R: they advise us on taking care of our children so that they won’t be harm

**I: okay. Now who give that advice on taking care of the children so that they won’t be harm?**

R: my parents

**I: your parents. Is there any desired information on parenting you wish to have but doesn’t available?**

R: none

**I: now is there anything else about the topics we talked about today that we missed or that you would like to tell us about?**

R: none

**I: That was great, we are done now. Thank you once again for your generous time and for sharing your thoughts with us. We greatly appreciate your help and we hope this research will help us improve the health of mothers and children in your community.**
