# Supplementary material for: Conceptualizing multi-level determinants of infant and young child nutrition in the Republic of Marshall Islands–a socio-ecological perspective
Source: PLOS Glob Public Health. 2022 Dec 19;2(12):e0001343. doi: 10.1371/journal.pgph.0001343 (PMC10022247; doi:10.1371/journal.pgph.0001343)
Supplement: S1 Data — (ZIP) [file pgph.0001343.s001.zip › RMI Supp Data/Interviews data/I47R_IDI_MCG_Arno_Sep 14_Balton.docx]

Interview code: I47R

Interview Type and Interviewee: IDI MCG

Interview Date: Sept 14

Location: Arno

Interviewer: Balton

Transcriber: Donald Duck

**I: So is it okay for us to start?**

R: Yes.

**I: Okay, to start off on our survey, the first question asks: Can you say a bit about your family. Like who lives in your homes, how many kids and what are their ages…?**

R: Well my kids… I have two kids… well three now. The infant, a girl, and another boy.

**I: How old are the two older kids?**

R: I don’t really know the exact age of the two older kids. They are around seven and ten.

**I: They’re both in school?**

R: Yes

**I: And when was the infant born?**

R: Last November

**I: Last November, so almost a year old now**

R: Yes

**I: Congratulations. So how is this this community?**

R: It’s good

**I: Good?**

R: Really good.

**I: How would you…. Have you been to Majuro?**

R: Yes

**I: How are things here compared to Majuro?**

R: Its better here because there are less dangers to kids here and the air is cleaner.

**I: Are there disadvantages or things that are not as good as in Majuro?**

R: I feel that there are none

**I: Everything is better here than in Majuro?**

R: Hmmm (affirmative grunt)

**I: Okay, we’ll now proceed on and with the health of the children. Can you tell me what kind of illnesses inflict the kids, not just your kids, but also all the kids here in Arno that you know of?**

R: Usually pinkeye and that last one that just ended.

**I: Which one?**

R: The vomiting.

**I: The kids were vomiting? Pinkeye and vomiting.**

R: Yeah

**I: How bad was the pinkeye?**

R: Quite bad, it effected the whole island.

**I: The whole island? So how did you treat it?**

R: With local medicine, from the stuffs from the kinnut bush(Local shrub)

**I: Kinnut? You mean from the white berries on them? The ones we usually squeeze into our eyes after swimming to relief the redness?**

R: Yes

**I: I see. And the vomiting, how serious was it?**

R: It was not that serious. It only effected the kids.

**I: Were they vomiting often?**

R: It didn’t last long.

**I: Didn’t last long but effect many? So how did you treat that?**

R: Well we took the kids to the hospital to be treated

**I: Another question the ribelle(foreigners) wishes to know states, “Can you describe how you know you child is sick and needs to see the doctor, what are the symptoms?”**

R: When they feel hot and when we see stuffs like them vomiting and loss of appetite.

**I: When the kids are sick, like with fever, who is the first person the kids are taken to?**

R: We usually try to take care of them but if it very serious it’s the doctor.

**I: So when you notice them getting sick you don’t take them to your wife or their grandmother or…?**

R: No, usually it’s just us that usually take care of them

**I: Have the kids ever had an illness that required you to take them to a traditional healer?**

R: Yes

**I: What type of illness?**

R: When the girl was….err… I mean when the boy had a stomach lump

**I: Stomach lump? So he was taken to have it massaged?**

R: Yeah

**I: This next question is related to your kids’ health, “What illness will your kids have resulting from lack of nutrition in their food?” It’s like from your own thoughts, what will happen if your kids don’t eat nutritious food?**

R: They will get sick often.

**I: They get sick often? There’s no wrong answers in these questions, everything you says is important because it’s the parents that are always with the kids and whatever you says will be accepted. What do the kids eat to be healthy? What type of food are healthy? Kids here seem healthier than some kids in Majuro, what you feed them?**

R: Mostly Iu(Cottony fruit inside shells of young coconuts) and those kinds of food. When people go gather coconut and there are iu, they feed them those. When people are cracking those coconut open they feed them iu and also give them coconut to drink. Also apples, like this one here…

**I: That’s an apple?**

R: Yes, it an apple tree from… somewhere. It’s the small one. When it bears fruits you will see lots of kids under it and outside around here.

**I: What type of food would make a child unhealthy, or which food are unhealthy for kids?**

R: Imported food

**I: Like which kind?**

R: Imported food like spam and other meat products?

**I: When we don’t eat vegetables like breadfruit, pandanas or drink coconut, what illnesses usually do people get?**

R: People that don’t usually get sick easily. Whatever illnesses that is currently spreading they will get. Those that eat lots local vegetables and greenery don’t but when they do it doesn’t stay long.

**I: Eheheh, we’ve been talking a lot about illnesses but can you describe a typical day for someone that is healthy from dawn until dusk. Describe a typical day for someone who takes care of his health and doesn’t drink stuffs like soda. What does he typically do? Does he sleep all day? Does he move around? What does he do from the time he wakes up until he goes to sleep?**

R: He can do anything. He doesn’t just sleep. He can do anything he want because he is healthy..

**I: So he is able to wake up early each day, wash his face, do a bit of work…. And???**

R: And then he does more work…

**I: Then has lunch and then…**

R: Then he pretty much do more work until the evening then he eats, take a shower and go sleep.

**I: And what’s an unhealthy person’s day like?**

R: He sleep most of the day and be lazy. He doesn’t do much but sleep and be lazy.

**I: What are the signs of a healthy child?**

R: The kind of kids that are healthy are always active. Don’t want to stay still.

**I: And signs of an unhealthy child?**

R: They are always sleeping or don’t want to do much work. They just want to sleep and lie?

**I: Lie?**

R: Yeah, if you ask them to do a task or for fetch someone, they will just say okay and not do it or say the person is not home without even going to check.

**I: Okay we will now talk about food availability. How do people in your home get food on a daily basis? How or where do you usually get food?**

R: Well this home is also a store. We gather coconut but we also eat and sell food from the store.

**I: So where do you get meat or food from?**

R: From inside the store

**I: I meant where do you get food to sell, do you bring them from Majuro?**

R: Yes, the owner (father-in-law) get stocks from Majuro.

**I: I see lots of coconut bags, do people bring those or are those for the store?**

R: Yes, these bags you see belong to the store.

**I: Instead of paying with cash people use those pay?**

R: Yes, people bring their coconuts, weigh them, and trade them for food and stuff.

**I: That’s a fast and easy way of collecting coconut**

R: Yes it is

**I: So this is a business. That explains all the vehicles here. I first notice that of all the houses here, yours is the only one with lots of vehicles. I had a feeling this was a business will all the vehicles, boat engines and stuff around. Is that the store?**

R: It’s through that door

**I: There’s even a street bike**

R: They all are working fine except this one, there’s a part that needs fixing.

**I: Nice, you know how to live. Are there any crops growing around this house?**

R: Yes, but they were always here. The panadas, the breadfruit and the apple tree.

**I: The fruits from these trees do you sell them here or in Majuro?**

R: Oh no, we don’t sell them. If people in Majuro want some fruit we just send those to them but we don’t sell them. When someone here is going to Majuro, they just take the fruits with them.

**I: And how does he buy merchandise for the store?**

R: The coconuts is used to purchase stuffs.

**I: How long the store been opened?**

R: It’s been here quite a while

**I: Ah, so the owner’s is quite used to this. He must have a set routine….**

R: Do you know the owner?

**I: Huh?**

R: He also used to live in Majuro

**I: Where at?**

R: Rita.

**I: Who?**

R: Heran.

**I: Heran? From Rita?**

R: Heran Helkena.

**I: Helkena? From Alwal?**

R: Yes

**I: Heran Helkena, I know the family. Heran, I might know him but just didn’t recognized him.**

R: He was the one that came and stood here and talked to me.

**I: If he’s an Helkena from Alwal I might know him.**

R: Elsi’s son

**I: I know him if he’s Elsi’s son.**

R: Yes, that’s his son

**I: He’s probably one of the older sons, is he older than the older girls?**

R: He’s younger

**I: Man, I have been away for twenty years, I won’t recognize anyone right away. Does he have a son in Inne? Elson?**

R: That’s not his son, that’s his brother. They’re brothers. This house is belong to the brothers, including Fredly too.

**I: Heran, he did looked familiar. He probably knows who I am, I don’t know. In this community, are there any challenges that might prevent you to grow crops? Like if you wanted to grow a breadfruit here, will someone be upset?**

R: The landowners here, if they see that you are planting something that will be beneficial, they’re all for it. Because they feel you are growing something that will benefit the land.

**I: So no challenges, unless you decided to plant something in the middle of the road, eheheh!!!**

R: Eheheh, that will definitely be met with disapproval because it’s against the law. The road is state property.

**I: So when you grow anything there’s no complication because they see it as you helping the land?**

R: Yes, you are planting the land, not ruining it.

**I: So if you want to plant anything, it’s not a problem?**

R: No, they will not disapprove.

**I: You know anyone on the island that is growing stuffs?**

R: Yeah. That over there the neighbors will grow something there.

**I: That’s going to be a garden?**

R: It will probably be a banana patch. If you see coconut husks lined and piled up like a fence like that it mean there is something going to be grown there.

**I: Oh, okay, like how you’re doing it there.**

R: That’s not ours, that’s also our neighbors.

**I: Do you grow anything?**

R: I do not.

**I: Do you eat from that breadfruit?**

R: Yes

**I: You know how there’s a season for many crops also breadfruit, where do you get your breadfruit when it’s not in season?**

R: Oh, that breadfruit is the type that produces all year round.

**I: Oh wow, so you get breadfruit all the time. Do you fish?**

R: I don’t because people bring fish here to our dad.

**I: He buys fish from them?**

R: No, he doesn’t pay. They just bring him fish because they are very close to him.

**I: So there literally isn’t any time when there’s no food in this household? If you don’t get food from the store they get brought from friends of the family.**

R: Yeah

**I: Do you raise any animals**

R: Yeah, there was a pig but it was slaughtered some time ago. The carcass was used to feed the The Red Cross people who came.

**I: was it penned in or did roam freely?**

R: No it was bind by ropes and tied to that tree

**I: Are there laws here for animals, like in Majuro pigs are all required to be gaged.**

R: There are but those laws are dormant

**I: So the laws are there but just are not being exercised**

R: They were at one time, by the elders before.

**I: No restrictions in raising animals?**

R: Yeah, none.

**I: I see a lot of animals run free, like chickens and pigs. Are there preferences or…. Why the preferences for the animals being loosed than in pens?**

R: On this island, I think you lose more caging a pig.

**I: How so?**

R: Here, the reason for why prefer putting animals in pens and letting loose is if a pig been fed leftovers all its life, if diet changes to coconut it will not adjust overnight. Also, some family don’t have any leftovers from their meals. Then the pig starves and does not reproduces as much. But if it were to be let loose, it could feed itself everywhere. It could feed on the wild vegetation that pigs here feed on. And you know how there are worms underground? It also digs the ground for those.

**I: So there’s a preference to let them loose?**

R: Well, it’s not really a preference because them loose is not a good thing. They ruin the grounds everywhere. People are just probably lazy. Like those who owns pigs get tire of always having to take care of the pig.

**I: And when the pigs that used to be in pens are set lose, they don’t adjust well, they still wait around to be fed?**

R: Yes

**I: What about chickens?**

R: Well chickens are fine. There are no restrictions to chickens because they don’t damage properties.

**I: Why is there some red pens that I see in many places here, what are those?**

R: Oh, those were chicken coops.

**I: Was that another project?**

R: It was a project from another organizations called KUMIT.

**I: I was very curious about it when I saw them. But they’re solely here since I didn’t notice any in Ine right?**

R: Yeah. The project was done here strictly here. A elderly Kumit member brought the project here. She lived in Jungle (Oceanside of Arno, Arno). She is of the Kalles family.

**I: She brought the project here?**

R: Yes:

**I: I was very curious when I first saw them then surmised they were chicken coops when I saw the fence at one end of it. But they were all empty.**

R: They all used to have chickens in them. But they were all set loosed. Like that one over there, there used to be lots of chickens in it. A chicken died there when the people there visited Majuro. There was only a young son that stayed behind and he didn’t stay there when it died. So the other chicken probably ate the maggots from the carcass. When the people returned all the chickens were dead. Probably died from feeding on the maggots from the dead chicken.

**I: Is that why there are no more chickens in every coop? They let them all loose after that incident?**

R: Yes. The chicken coop was a great idea but it was just too small. The inside Is just too small.

**I: Looks big enough just for two. The next question states, there are times when we wish we could eat something, but we can’t, either because there is none or not enough around. What would your family like to eat but couldn’t because there’s none or not enough of?**

R: Shellfish.

**I: Shellfish? Which kind, like crabs, lobsters, or…?**

R: Coconut crabs are very rare because pigs get them first. Also there are lobster out there but they are mostly on the other side of the lagoon at the other islands. They are very rare here. When people here do manage to get some, it’s usually just one.

**I: On this last question regard food. Who in the family decide what food the family should eat on a daily basis?**

R: The old man.

**I: Is there an old lady?**

R: Yes, but she just left for Majuro.

**I: So who typically decide what to eat, or what to take out from the fridge?**

R: The old man decide everything.

**I: And who decides what the children eat?**

R: Well, for my infant, it’s me and my wife who picks what the child eat.

**I: You and your wife decide what the infant eat?**

R: Yes

**I: Now we move on to drinking water and washing of hands. This survey and reason for children to be stunted is malnutrition. One reason for this is diarrhea. This could may be the main reason, or not, I am not sure, but is why I am asking you these question, to find out why they are not growing to their fullest potential. Could you describe how you get and store water? How do you find water for your home?**

R: We bring from homes with pontoons (Water tanks).

**I: For drinking?**

R: Hmm hmm (affirmative nod)

**I: Oh, there no pontoons here?**

R: There’s none

**I: How is it that most homes in Ine (other end of Arno) have pontoons?**

R: It’s just that this house just hasn’t gotten its share of pontoons.

**I: Most have but this house’s amongst those that hasn’t?**

R: Yes. There also some homes on the island that are still waiting for their shares.

**I: So in the meantime?**

R: I the meantime we get water from the homes using plastic gallons.

**I: For drinking?**

R: Yes

**I: And for bathing?**

R: For bathing, that over there (old cement water tank).

**I: And for Cooking?**

R: From that same tank.

**I: So drinking from neighbors tanks but shower and cooking from this tank.**

R: yes

**I: oh ok. What about the well water? Or what is that? Is that a well?**

R: yes

**I: and what is that for?**

R: for washing dishes

**I: so it’s just of dish washing and clothing washing and the gallons are for drinking water. And for bathing you use this water tank right here?**

R: yes

**I: so there are few houses that hasn’t get their pontoon share? Who is responsible in sharing pontoon to houses here?**

R: the local government

**I: so what is the difficulty is getting your pontoon?**

R: well I don’t have any idea

**I: do you think the pontoon are still in the Council men’s house or they’re still in Majuro and hasn’t been shipped here?**

R; they have already shipped but not on this Islands, the small islands close to this islands.

**I: so you mean Ine town or?**

R: yes

**I: so where do you store the water that you get?**

R: we store the water in the plastic gallon

**I: these big white plastic gallons?**

R: yes

**I: is there any difficulties in bring water to this house?**

R: there is no difficulties

**I: is there any difficulties in storing water?**

R: I don’t think there is difficulties because the last time we had rain water was last month.

**I: we haven’t have drought here on islands**

R: yes

**I: do people in the house where you get water usually clean their pontoon?**

R: they used to but nowadays they hasn’t clean their pontoon maybe there is times for them to clean their water tank.

**I: do you boil the drinking water?**

R: we never did but only the water given for my father in-law and for my child are boiled water

**I: Let’s now discuss hand washing. Could you describe in detail your family’s hand washing throughout the day? Like when hands washing usually happen throughout the day?**

R: before they eat

**I: and what about the elder people?**

R: the same, but for me. I don’t usually wash my hands because I usually use spoon to eat.

**I: and what about the child?**

R: the mother feed him

**I: what time during the day soap is use to do hand washing?**

R; before meal

**I: from your own opinion, is there any differences in washing hands with only water or with soap and water?**

R: we won’t have clean hands if we don’t use the soap to wash hands. But whenever I make copra and my hands get dirt, I usually go to the lagoon side and wash my hands with salt water and rinse it with sand.

**I: what prevent someone from washing their hands?**

R: when people are in rush

**I: is there any times that you wash your hands but there is no soap to wash hands with?**

R; yes

**I: is there any soap in the stores here?**

R: yes

**I: so soap there is no problem with the soap, it’s just when people are in rush. You have very nice answers instead of answering yes and no. These are the information we are looking and by sharing your information is really helpful. The people who will read these information would be glad to get your good information. We will translate these to English and the person that would read these information will have clear visualize on what we are trying to talk about here.**

**I; is there any toilet in this house?**

R: yes there is one right there

**I: this one?**

R: yes

**I: it’s a flushed type?**

R: yes

**I: there are some toilet that are dig underground and use a toilet bowl while there are some houses that don’t have any toilet. Is there any differences between toilets like that from toilet that has been dug? Which one is better in using or better having it?**

R: they’re both good

**I: is there any difficulties or law against building toilets here on Islands?**

R; there is no difficulties

**I: is there any programs from the local government to help build these toilets?**

R: I’m not really sure about that

**I: how long have you been living with this family?**

R: it’s been a long time

**I: were you here when the toilet was build??**

R: no I wasn’t here that time

**I: you came here and it was just here?**

R: yes. This house is much older than I am

**I: “laughing” you were born and the toilet was already there?**

R: I am new here. I just moved here.

**I: where did you grow up?**

R: I am from Ebeye

**I: Ebeye! What town?**

R: the last part of the islands is where I used to live. Near to Rilong’s house.

**I: coast guard town?**

R: near the Catholic Church.

**I: Catholic Church?**

R: yes

**I: My town is loan aelon in (Town name in Ebeye) if you know loMaet and them. When was the last time you moved from Ebeye? How old were you when you left there?**

R: old enough

**I: do you know George Luther the council man?**

R: um

**I: Aleak town (another town name) Lijabet, Lanio or Loran, well the house is just right across from the hospital there.**

**I: there are some people who defecating in open area and there is much defecate in Majuro. What do you think would make people defecating in open areas like ocean or lagoon side? For example here on Islands?**

R: well in here or in this house, if there is someone using the toilet and we want to use it at the same time and can’t hold t, then we rush to the lagoon or ocean side. But in other places, they don’t have their own toilets.

**I: do you think people face difficulties in building their own toilets?**

R: it can be budgeting

**I: ok for buying stuff to build a toilet. Sometimes the children defecate under the threes or around this area. How do young children’s stools are typically disposed of especially in the lagoon side?**

R: in the lagoon side, they usually told be dig the sands before they poop.

**I: and what if they defecate under the breadfruit trees?**

R: one example is when they’re out there and gather copra in the bushes and would want to use the toilet, then they would go ahead and defecate in the bushes. Then they would just leave them there because they don’t know that they should dump them.

**I: where do your children usually play every day? Where do they usually go and play?**

R: around this area and sometimes they goes to the houses near this house and play with the other children.

**I: if you can think of a place that should be built for the child to play in, where would be the perfect place you can think of?**

R: at the school

**I: what about the school?**

R: I think it’s the only place that have bigger and better playground area for children in this community.

**I: is there any animals in places that children usually play?**

R: what kind of animals?

**I: any kind of animals**

R: just the dogs but they never disturb them playing

**I: just the dogs and the children and a little pig. What would be some challenges of keeping children’s play area clean?**

R: like what?

**I: let’s say if it was the school area, the field where they play at. Does it clean or not?**

R: The only thing that make it unclean is their school stuff that they tear them and scatter them around the play area.

**I: and what if they play in this area around this house?**

R: well they usually do the same thing. They come back from school and make air plane out of paper and play with them. That’s what make this area unclean sometimes.

**I: To wrap up our questions on hygiene, could you explain ways to prevent the spread of disease? What would help prevent diseases from spreading?**

R: what kind of sickness?

**I: any kind of illnesses it can be the pink eye that affect people here or can be any kind of illnesses**

R: the pink eye, well if the child is having pink eye and is not a child from this house, we tell them to go their houses so that they can’t share it to the other.

**I: and what about illnesses like coughing or any other illnesses?**

R: the same, we do the same thing we tell them to go to their own houses so that their parents can take care of their illnesses.

**I: Ok now we are on the section on gender and family roles. We are also interested in the roles and responsibilities different family members play in raising children in this community. Could you describe the care of children throughout the day in your community?**

R: from what?

**I: like how they raise them, take care of them and it can’t be the two of you but your neighbor. Are they together when it’s comes to children raised?**

R: so you mean if the child is not from this house, do we participate in taking care of them or what?

**I: yes do you take care of them or**

R: yes there are two children that don’t live here. Their parents asked with my father in-law if they can stay here with us. They asked if we can take good care of them while they’re away.

**I: so people in this community are good in help each other raising children**

R: yes

**I: if your children would have gone somewhere danger and play, do you think your neighbor or people who see them would take care of them?**

R: I think they will because we all know each other

**I: in this community, who is the main responsible in raising the children?**

R: people here are all family and we all help each other taking care of our children. I think that’s one reason why people easily care for each other. We all related to each other here on islands.

**I: who is the main responsible for child care?**

R: my wife

**I: what are the responsibilities of mothers in child care?**

R: teach them the way we should live. She teach them to do work around the house, cook foods and help each other.

A: And what about you. What do you do for the child care?

R: I make copra so that I can trade them for foods for the family

**I: so that’s your main responsibilities here?**

R: yes

**I: so who usually spend time with the child?**

R: well everyone in this house

**I: everyone is responsible in taking care of the child**

R: if the person is not busy and is doing nothing then they can watch over the baby

**I: do the older sibling play with the child?**

R: yes

**I: what do they do?**

R: they play with him when their mother is busy doing house chores

**I: is there any differences when there is grandparents from when there is no grandparents to be there for their grandkids?**

R: I think there is differences

**I: like what?**

R: sometimes they feel like they want to spend some time with their grandparents

**I: what do grandparents usually do so that grandkids would always love to spend their time with them?**

R: my son usually spend his time with my wife’s parents. My father in-law usually take him for fishing and also when he is out gather copra, then he would also take him with him.

**I: they’re like best friends**

R: yes they’re best friends

**I: what makes the child’s grandparents good?**

R: they’re good in taking care of their grandchildren because they love them

**I: Could you talk about the role that other family members have in raising children in this community? Like is there anyone else in this house aside from your parents in-law and you and your wife live in this house**?

R: eh?

**I: is there any more people live in this house aside from you and your wife and your father in-law?**

R: the girl that was raised by my mother in-law. She has her different father from the old man in this house. And that guy and my wife, they’re my father in law’s own children.

**I: so do these people take responsibilities in taking care of the children?**

R: yes

**I: you have really nice answers. Now we are on the last part and you are doing really great. We are now on communication channel on how do you get information about raising children, or making their foods. Where you usually get trusted information about nutrition and health?**

R: I am the oldest sibling among us, and I usually get information on nutrition and health from them by taking care of them each and every day. Then when I have children, I knew what to do.

**I: so that happened from your childhood growing experience?**

R: yes

**I: do you usually get information from the internet, the TV or the radio about health and nutrient?**

R: no I have never heard from any of these sources

**I: where do you think would be the easiest way for you to get messages on health and nutrient from?**

R: the hospital

**I: this is our last question. When you think about your own parenting behaviors, can you explain what influences how you raise your children?**

R: I have always want a child. I grew up with my parents. I never met my grandparents, so I have always wanted to have children before my parents pass away.

**I: what are people’s opinion on raising children in this community?**

R: well if it was me, I would never want my children to grow up and have their own babies in their young ages. I always see them pointing some to be their girlfriend or boyfriends but they are still in their young ages. I always advise them about that. They should finish their education then have their babies later when they’re done.

**I: is there anything else about the topics we talked about today that we missed or that you would like to tell us about?**

R: all good

**I: ok. Thank you so much for your generous time. Thank you for your information on behalf of the organization that I work for under the ministry of Health and Human Services we want to thanks you two for taking your time and answer questions in this survey. We are in Ine Arno right now and next week Monday we will be here and if you have any questions please don’t hesitate to come to the hotel where we will be staying at. There will be coffee provided see you there and thank you once again.**
