# Supplementary material for: Conceptualizing multi-level determinants of infant and young child nutrition in the Republic of Marshall Islands–a socio-ecological perspective
Source: PLOS Glob Public Health. 2022 Dec 19;2(12):e0001343. doi: 10.1371/journal.pgph.0001343 (PMC10022247; doi:10.1371/journal.pgph.0001343)
Supplement: S1 Data — (ZIP) [file pgph.0001343.s001.zip › RMI Supp Data/Interviews data/I48R_IDI_MCG_Arno_Sep 14_Balton_MarcellinaEdited.docx]

Interview Code: I48R

Interview Type and Interviewee: In-depth Interview

Interview Date: Sep 14 2018

Location: Arno

Interviewer: Belton

Transcriber: Fela

**I: ok if that’s fine with you, can we start now?**

R: yes

**I: To begin with, can you please tell me a little about your family household? Like how many people in your family, who live in the house or how many children and their ages and gender?**

R: there are four people in this family. Me, my name is Ray (Laughing)

**I: no that’s fine go ahead**.

R: My wife and my two daughters, one of them is four more months so that she can turn four and the other one is one year and three month.

**I: one year and three months ok she is fifteen months**

R: yes

**I: Now I’d like to ask you to describe your community?**

R: well in this house

**I: no you don’t have to tell about the owner. You can talk about the community like are there many people live in this community or how far neighbours are close to each other.**

R: like what?

**I: um like people live close to each other or just you and your family live in this community?**

R; ow yes people live close to each other in this neighbour

**I: the next question we are going to talk about is all about the child’s health. Can you tell me about some of the illness that your children have suffered from?**

R: fever, coughing, yes I think these are the two common illness I can tell

**I: what are the causes of fever?**

R: like what?

**I: do have any ideas, the reason or the cause of the child having fever?**

R: well the fever came out of nowhere, we never know what happened, and the child just got fever

**I: how serious was the fever, was the child suffered from fever?**

R: the fever was fine, the child wasn’t really suffered from it.

**I: then how did you prevent fever?**

R: I rise cloths with water and wipe the body with the cloth to prevent or calm the fever down

**I: so you cool down the fever hot temperature? Ok and what about coughing? Do you know the cause of coughing?**

R: we give her medicine from the hospital

**I: to prevent coughing?**

R: yes

**I: and what about the cause of coughing?**

R: is the same thing, the coughing illness came out of nowhere.

**I: well was it serious or did she suffered from coughing?**

R: Not really

**I: so you just went to the hospital for check-up?**

R: yes

**I: the time when your child was sick, what were the signs that you knew that the illness was serious and you knew that you have to take the child to the hospital?**

R: it was really different because the child couldn’t move, she just lie down and did not move.

**I: hmm**

R: yes that was the sign that made me realize that she was sick.

**I: it was not normal to her ok. When the child is sick, who would be the first person you bring the sick child to? Do you bring her to the mother or the grandparents or is there anyone you first go to before you go straight to the doctors?**

R: straight to the doctors

**I: do you and your wife ever use any traditional medicine to heal your child’s illnesses?**

R: we have never been used traditional before

**I: never. When it comes to foods that given to her, or nutrition is there any illness caused the health of the child by not eating healthy or nutritious? Nutritious or healthy foods, do you think they can cause any illnesses to our or your children?**

R: I don’t think there is

**I: there isn’t any illnesses cause by nutritious and healthy foods**

R: yes

**I: what kind of foods that caused the child to get sick?**

R; eating can meat

**I: hmm and what kind of foods that make the child’s body healthy**

R: local foods

**I: local foods right? Like what?**

R breadfruits, banana, fishes,

**I: yes because the people I work for are from Canada and they don’t know what kind of local foods, and that’s why I asked you to mention what kind of local foods you that makes the body of the child healthy. They don’t really know much about foods that common here they only know about one foods and its breadfruit. Aside from breadfruit, they don’t know what else we eat. It is he only foods that they first came here and eat it and they love it a lot, but when you say local foods, they are going to ask you again to list down what kind of local foods that you eat or give to your children.**

**I: um let’s say if children eat foods that are not local foods, like can meat, candies or ship, what kind of illnesses that would happen to them?**

R: toothache when they have cavity

**I: anything else, just tell the your answer I am listening**

R; diarrhea. What else? I think these are the think I can only think of

**I: hmm. Aside from healthy foods, can you give any example of a healthy person? What does the person do from the time they wakes up until the time they go to bed? What do they usually do throughout the day?**

R: they do works

**I: alright can you be more specific on what they usually do throughout a day? Their typical day what do they do from morning until evening? Like what did they do yesterday, or what do they do today? If the person wakes up, what do the person do throughout the day? Do works, eat, or only doing work?**

R: they do lot of works

**I: lot of work?**

R: Yes

**I: and what about unhealthy people?**

R: well they lie down all day long

**I: and what about the children? How can you tell that the child is healthy?**

R: the child always shout or play like we can just see how the child move throughout the day and can tell that the child is feeling healthy they also do lot of moving they when they feel unhealthy, the eyes are red and the child would sleep all day. They just feel weak and lie down because they’re sick.

**I: Ok now we’re done with health, we are now on foods available here in this community. I would like you to explain how your household gets food to eat on a daily basis? Where do you gets foods from throughout the day?**

R: from the store

**I: the store?**

R: yes

**I: you two live in the store?**

R: I bought foods from the store

**I: ok. Is there any foods grown at home?**

R: it belong to the owner here

**I: so you are the property care here in this community?**

R: no only here in this area. Care taker for..

**I: this area**

R: for the tourists

**I: oh ok so you are the tourist care taker? Hmm**

R: people talk to respondent

**I: is that a tourist?**

R: no he is a world teach here for the school here in this community

**I: what is a world teach?**

R: he is a teacher from the State and he only come here to help students and teacher or schools that really needs help within their education level

**I: ow wow, so where does he live?**

R: yes but there is also a women that is like a host family here and I am also responsible for her. She is my responsible

**I: hmm. Umm so the foods grown at houses here, what people do about them? Do they sold them or trade them?**

R: well we don’t have the program for planning

**I: ow so you mean the cucumber planning?**

R: yes

**I: so what they usually do about the cucumber planting? Sale or trade?**

R: we do shipping to Majuro and trade or sold them

**I: ok. ok now I remember. So that’s the planting spot is where the tanks has been dug underground?**

R: yes

**I: oh, so now what about the things inside the tanks?**

R: Noni fruits juice

**I: oh so it is here that they made the noni fruits juice from?**

R: yes

**I: so noni fruit juice and cucumber planting are made here?**

R: yes

**I: ok now that’s make sense to me, noni fruit juice made here ok now I know, and also the foods grown here you eat from it and sell from it at the same time?**

R: yes

**I: ok. Now do you have your own foods growing spot or do you do planting?**

R: no I don’t have

**I: you don’t have, so what if you were to make your own foods growing, was it possible enough for you to do so? Is there any difficulties?**

R: I would rather do so

**I: there is no difficulties in that?**

R: there is none, everything is fine here there is no difficulties and there is also pumpkin in that foods growing?

**I: so you have pumpkin too?**

R: yes as in right now.

**I: do they sell them?**

R: no we offer people for free but we also eat them

**I: you eat the pumpkin? Was it not part of the farm here?**

R: no

**I: they are your own foods, if you want to sell them it is up to you?**

R: yes

**I: do you usually eat breadfruits**

R: umm

**I: um as you know there is season for breadfruits right?**

R: yes

**I: there is season for breadfruits, and if this is not the season for breadfruits, what do you usually feed your family when it’s not the season for breadfruits?**

R: I feed the rice that I bought from the store (Laughing)

**I: that’s fine, it is better when we don’t have local foods (Laughing)**

**I: is there any times that the family run out of foods or food or shortage of foods?**

R: well, we don’t face that right now.

**I: you still have available foods on the table**

R: yes. There is available foods at the house, but if there is no meat to eat with the foods, that’s like we don’t have foods available on the table to feed the family. I hate it a lot when my family don’t eat meat, but only rice or breadfruits, I hate it when they eat rice with the sodium salt or rice with the soy sauce that’s not a freaking foods.

**I: so what are the available foods you commonly eat?**

R: I take meat from the store, or if I not, I can go fished using the kayak.

**I: oh so you guys have the kayak? Can we use it for rental? Or is it your own?**

R: yes it also include staying here

**I: *yes* whispering ok I am looking forward to our visit here. That’s going be the first thing I will be up to here is the kayak.**

R: yes

**I: nice! Ok now I forgot what I was going to say (Laughing)**

**I: is there any animal raise in this community?**

R: yes there is

**I: like what?**

R: chicken

**I: they are kept in their case or?**

R: there is chicken everywhere, they kept in their case, and they scattered all around this community

**I: who own these chicken?**

R: I owe some of them

**I: so you also raise some chicken that’s part of this compound?**

R: yes

**I: so the animals that you raise, do you keep them in your fences or you let them lose?**

R: the pigs and the children that I raise are kept in their fences or cases. I don’t let them go

**I: ok so do you have difficulties to raise animals in this community? Like building material and stuff like that?**

R: well I can say that there is a little difficult, sometimes I do really need materials to use but it is hard to bring them from Majuro. There is no difficulties in buying it but the only difficult is that transportation from Majuro to here.

**I: so the only difficult is shipping thing?**

R: yes I have to save enough money so that I can bring all these materials that I need. I have to buy the material and also pay for the freight for shipping

**I: hmm**

R: yes pay for the freight or the shipping that I put the stuff on, we also have to pay

**I: how come you don’t make any good connection with your friend or the guys that own this property? (Laughing) Robert and his gang, telling them that you need their help (Laughing)**

R: if I am in Majuro and I know there is a boat leaving Arno, now I have the chance to catch the boat up and bring all my stuff on.

**I: your supplies**

R: yes if it is the owner’s boat, then I don’t have to pay for the chipping from Majuro to here Arno, it’s all free

**I: so you don’t have any difficulties in building any fences here in this community since sometimes the owners would refuse to let people build any fences for their animal?**

R: no they don’t. They are good people

**I: hmm I know. So there is no difficulties in raising animals?**

R: yes

**I: um. There are sometimes foods that we wish we could eat, but for some reason we cannot. Could you tell me about any foods you wish your family could eat or eat more but cannot? Not just that we don’t eat, but sometimes it’s expensive, or it’s not common, or let’s say it’s rare, is there any kind of foods that you want to feed your family?**

R: foods from the restaurant, or pizza, I want these foods but it is a little difficult for me to get them

**I: What are the difficulties? Is it because there is no one to cook them?**

R: yes

**I: is there is over to use to cook these foods if you knew how to cook foods you could’ve cook?**

R: I do not cook, I can’t cook.

**I: is there any over here on islands?**

R: local over

**I: oh ok local over that you make the fire under it?**

R: yes

**I: yes I think we can make our own oven. So it’s just pizza or foods from the restaurant right?**

R: yes

**I: if you really want to eat, then you go straight to Majuro?**

R: yes

**I: For the last question on foods, can you explain who decides what foods to get for your family?**

R: the wife

**I: the wife?**

R: yes

**I: Who decides which foods young children should eat?**

R: also the wife

**I: so who would be the one to look for or bring the foods? (Laughing out loud)**

R: well you know the husband

**I: (Laughing) ok now foods section is now taken care of. In the next section, we wold like to talk about water and hygiene. Can you please describe a typical day of getting water and storing water for your family? Do you have water at home?**

R: yes

**I: where do you get water from?**

R: the bantoon (water catchment)

**I: where do you get the water inside the bantoon from?**

R: rain water

**I: and what about shower water?**

R: the same we also use rain water in the bantoon for shower.

**I: and what about water to cook foods and drinking water?**

R: the same rain water in the water catchment, the bantoon.

**I: was it difficult for you to have water catchment at the house? Where did you get your bantoon (water catchment) from?**

R: well I bought it from Majuro and it is provide from the owner of this place

**I: oh ok I see. So the water catchment is also include with properties that are in this area right?**

R: yes

**I: ok. So you never spent for anything there? It was all set up before you came here?**

R; yes but there are some houses here in this area that have cement water catchment

**I: cement water catchment?**

R: yes

**I: do you usually clean your water bantoon water catchment?**

R: yes

**I: how do you clean it?**

R; I drain out the water and use the water for showering before I dump the rest of it. If the bantoon is dirty, I drain out the rest of the water inside. But I have to take a shower and clean myself before I go inside the bantoon and clean the inside part. If not from my water catchment, I clean the rest of the water catchment around this area, there are lot of them, there is one here, two over there, three and four, and plus that cement water catchment. There are also two over that make makes it six, but the sixth is down, it is no longer used.

**I: wow! You don’t have any problem with water catchment**

R; yes this house also use well water

**I: there is also well water?**

R: yes. In this place, there are two water catchment here, two over there, and two on top of the house. There is also water for the bathroom, there is also one over there.

**I: flush toilet bowl?**

R: yes

**I: wow**

R: it’s like what we called, RRE( Robert Reimers Entertainment) hotel, the one in Majuro

R: people here called this place RRE

**I: hmm ok. That’s really great**

R: yes if someone would pass that gate door, they feel like they are in Majuro (Laughing)

**I: hmm**

R: there is provided generator

**I: not solar**

R: there is solar but they both work, the solar and the generator.

**I: ok. Let’s now discuss hand washing. Could you describe in detail your family’s hand washing throughout the day?**

R: before we eat

**I: before you eat?**

R: yes

**I: aside from before meal, is there any other time?**

R: after we are done in our chores, we wash our hands before we hold the child. For example, whenever I want to hold my children, I would take a shower first or wash my hands before I hold them.

**I: hmm. So when do you use soap when you wash your hands?**

R: there is hand sanitizer

**I: there is hand sanitizer? What is the differences between the hand sanitizer from the soap?**

R: well I would say that hand sanitizer is better than soap.

**I: hmm. That’s what you commonly use right?**

R: yes

**I: there are some people who hold the child or do works around the house and then after hold the child without washing the hands, what would be something that prevent from doing hand washing?**

R; when they are in rush

**I: rush?**

R; For example when the child goes to upper lever, while the mom or the dad is cooking. When they see their child staring at them from the higher lever, they would leave the food and rush to get the child from falling from that spot.

**I: thank you for sharing your answer. That’s what we are looking for in this survey aside from giving us answers like yes or no but you are taking your time and sharing your information. The record catch lot of information from you. When we do transcribe, our bosses will have clear mental picture of what you are trying to tell us here. They will really happy about that because that’s what we are looking for in our survey is your full and supportive information and thank so much.**

**I: Can you describe the type of toilet that you have at your house?**

R: it’s a flush toilet bowl.

**I: is there any difference between the flushed toilet bowl from the hole that was dug and made from cement or ply wood? The one that it just the cement or the ply wood and the hole inside, what is the difference between these two?**

R: oh yeah toilet like that?

**I: you haven’t seen toilet like that?**

R: yes I have seen but I never use one before. There is toilet here in some houses but not in this house, we use flushed toilet.

**I: Since you first came here, you used to use the real flushed toilet so you never use toilet like the one people here use. Ok that’s great. In some communities, we have heard that defecating (I don’t know if it’s happening there) is common. Could you help us to this practice, including how common it is?**

R; well because maybe they don’t have money to build their own toilet. Do they have to wait for their material to build the toilet bowl or they have to do defecating? They do defecating and when their toilet bowl is all set up then there will be no more defecating. Right on to that, let’s do it using the toilet bowl.

**I: *Laughing* yes I see that’s happening. Is there any problem like that in this area?**

R: there hasn’t been any problem like that.

**I: you are monitoring that practice from happening here?**

R: yes

**I: people in this area know better**

R: people in this community knows that this place is for tourisms

**I: what are the barriers to using the toilet aside from what? The landowners, or is it easy for them to allow people to build their own toilet?**

R: yes

**I: it’s not a big deal right?**

R: yes

**I: and what about funding and stuff like that? Were there any programs to help people or**

R: there hasn’t any programs for things like this

**I: you haven’t heard that there is program for this?**

R: yes. I only know that I asked for seawall

**I: for where, ocean side or lagoon side?**

R: ocean side

**I: I have been to the ocean side and it is really beautiful, most likely the same as the lagoon side. There is times of high tide right?**

R: yes. That’s the only thing that I asked from the IOM (International Organization of Migration) team but it never happen until now.

**I: the senator is just right next to you, you can go and tell him that you want to go out and fished with him then tell him all about your unanswered questions about your needs.**

R: Mike

**I: he haven’t visit these days?**

R: not quite often

**I: hmm. Um so how do your children do disposed? Do they do typically defecate or using the toilet?**

R: they do defecating outside

**I: outside, so how their stools are typically disposed of?**

R: using the shovel

**I: the shovel? And where do you dump it?**

R: where I plant the bananas

**I: so you mean the farm?**

R: the only thing the child do is defecating outside the house then I use the shovel to dump it in places where there is plants grown in.

**I: that’s great. Could you explain where your young children usually play each day?**

R: sometimes inside the house and sometimes outside the house

**I: do your children play in areas where animals are kept in? Like chicken, pigs and dogs?**

R: yes but there is no dog

**I: so what kind of animals are there?**

R: only chicken

**I: only chicken?**

R: yes

**I: is the playing area clean like is there any trashes in their playing area?**

R: it’s good I clean it up everyday

**I: ok. To wrap up our questions on hygiene, could you explain ways to prevent the spread of disease? How can we prevents diseases from spreading or help prevent illnesses from spreading? What’s the best way you can think of?**

R: illnesses like

**I: diseases that children easily can get?**

R: yes?

**I: what are some best ways we can do to prevent them from spreading?**

R: give them medicine

**I: medicine?**

R: yes

**I: We are now in family and family roles and responsibilities family members play in raising children. Could you describe the care of children throughout the day in your community? Who is the main responsible for raising children?**

R: you are referring to family or?

**I: your family who is mainly responsible in raising children?**

R: the wife, the mother of the child

**I: what is the main responsible of the mother of the child?**

R: she takes care of raising the children, she do cooking,

**I: anything else aside from feeding them and raising them?**

R: clean them and make sure they are clean and neat. She also makes sure she clean both the children and the house.

**I: what is the responsible of fathers in child care?**

R: make sure there is foods on the table

**I: hmm (Laughing)**

R: make sure the family don’t starve

**I: yes that’s true. Does the baby have sibling? How many sibling?**

R: only two girl sibling

**I: how old are they**

R: one of them is few more months and she turn four

**I: she is three years old now?**

R: yes

**I: can she walk by herself?**

R: yes

**I: does she usually play with her younger sibling?**

R: yes they always play together every day and the other one only one year old now.

**I: hmm. Do grandparents help raise the children in this community?**

R: yes

**I: ok. From your own opinion, is there any differences when grandchildren raised by grandparents or raised without grandparents? What is the difference, is there any good or bad influences when grandparents raised grandchildren?**

R: differences between what?

**I: when grandparents help or does not help raise their grandchildren?**

R: yes that’s right there is differences

**I: ok like what? Can you give me some examples?**

R: the differences is that... what can I say?

**I: what are some good influences that grandparents help in raising grandchildren?**

R: it is difference when children don’t have grandparents, and their grandparents are not there to help raise their children. But if children have grandparents near then, then they have someone there near their parents to help raised them. It depends when there is grandparents or not.

**I: how can you tell what makes good grandparents? What makes you say that grandparents are good because they do this and that? What do they do to their grandchildren so that you can you tell that grandparents are good?**

R: if they know the parents are busy doing work around the house, they help feed the child, or they also buy the child clothes or something like that

**I: so they take their part in raising children like parents do?**

R: yes

**I: is there any family members that help raise your children?**

R: raise them? No

**I: so only the two of you raised your children by yourself?**

R: yes

**I: ok. We are almost finished. If you wanted to get some information about health and nutrients, where do you think would be easy for you to get possible information from? Where do you think would be easy for you to get information about nutrients and health from?**

R: Usually from the hospital

**I: hospital?**

R: yes

**I: anywhere? Do you get information by listening to the radio station or from watching television?**

R: well we don’t watch movies here, we can only get information from listening to the radio station.

**I: where do you think is the most trusted information that you get these information from?**

R: I can take both

**I: health programs?**

R: yes the hospital also do health information but I meant that I get information from radio B7av

**I: ok so it’s where you commonly get trusted information from.**

**I: ok this is our last question. Right now you have already become a father, like a father of two, you have two daughters. Was there anything that influence you to be the father you are right now? Like is there anything that motivate you? Was it people or it was just yourself?**

R: it was just me

**I: what influence you to...?**

R: like I said, I have children

**I: what really influence…**

R: yes my children motivated me a lot

**I: influence you a lot to care and loved like what you mentioned earlier, you don’t want to eat only the rice or the sodium salt or the soy sauce, or just rice itself, what makes you think that way?**

R: I want my family to feel energized all the time and not feeling weak. I do my best not to let them starve for foods and water

**I: ok. Is there anything about nutrition or parenting you wanted to know?**

R: I can say that I am good and I get good information from what we have talked about

**I: we are in Ine Arno right now?**

R: yes?

**I: we are together with the hospital chief nurse, along with the two foreigners. They came know a lot better about nutrients and it is their field of study. They are mastered and doctors’ degree in nutrition, umm they were sent from the company or the organization called UNICEF and if you have any questions and something that you would want to know more about? We are just there but maybe two weeks from now, we will be here on islands, Arno Arno.**

R: hmm

**I: and I am sure we will live here in this place, and you have any questions at that time, please don’t hesitate to come and ask questions. I will also be here with them along with some of my fellow hospital workers, we’ll be here. And I want to thank you so much for taking your time and sharing your information with us. Together with the ministry of Health and Human Services, we want to thank you and bless you and family.**
