# Supplementary material for: Conceptualizing multi-level determinants of infant and young child nutrition in the Republic of Marshall Islands–a socio-ecological perspective
Source: PLOS Glob Public Health. 2022 Dec 19;2(12):e0001343. doi: 10.1371/journal.pgph.0001343 (PMC10022247; doi:10.1371/journal.pgph.0001343)
Supplement: S1 Data — (ZIP) [file pgph.0001343.s001.zip › RMI Supp Data/Interviews data/I49R_IDI_MCG_Arno_Sep 14_BM.docx]

- Interview Code: I49R
- Interview type and Interviewee: Male Interview
- Interview Date: 9/14/18
- Location: Arno
- Interviewer: BM
- Transcriber: BM

**I: Do you approve of letting me record our conversation through this recorder?**

R: Yes.

**I: Thank you for your time. Let's start with, can you tell me about your family?**

R: Like as in?

**I: Your family.**

R: Yea but like what do you mean?

**I: Like how many are in your family.**

R: Oh, there is 3 girls and 2 boys amongst our family so 5 of us.

**I: Who lives in this house?**

R: Well there is only 3 kids. So me, the 3 kids and my wife.

**I: How old are the kids?**

R: The kids, the youngest one (Silent thinking) when it's November she will turn 2. The older one she is in kindergarden she is 5 years old. The eldest one is 11 years old, she's at Majuro for this time being.

**I: How many boys and girls?**

R: 3 girls, there's no boys.

**I: Now can you explain about this community?**

R: About?

**I: About this community as in the good things or the bad things about this community?**

R: Oh about this community, about the kids here in this community if it were the kids. The ones that goes to school, well their parents aren't really pushing them towards school. If the kids says they're lazy well they're lazy. Also about the foods that the kids always eat, there are a lot of foods from the outside of our island, there are sometimes no local foods because we're not having enough space for planting.

**I: Is there anything good about this community?**

R: Well yes it's really good here. It's good to live here, we're not scared of the flies. So yea living here is good, it's good. There are coconut, breadfruit, pandanas. But the thing is it's barely any.

**I: Now we will talk about any illness that this family has ever taken. Can you tell me about any types of illness the kids in this family usually get?**

R: If it's the kid’s maybe the illness they usually get is flu the illness flu, they usually get it.

**I: Is there any other illness besides flu?**

R: Also diaharrea but they don't usually have it. The thing that they usually get sick from is flu.

**I: Can you explain what makes them have flu?**

R: Well I think maybe because we breath in dust and things that are dirty if not maybe the climate, maybe we're too close to the ocean I don't know but maybe.

**I: Can you tell me if you know the seriousness of flu?**

R: Well every illness is very serious I know that it's very serious like if it was a fever and their fever is very high they would have a headache, they would become deaf, and also they can die from it so yea it's really serious.

**I: Can you tell me how you prevented the flu?**

R: Clean her up like making sure she washes her hands, keep her clean, let's just say making sure she's clean.

**I: Like if she has a flu what do you do to cure her from it?**

R: Well we take her to the doctor.

**I: Since you take her to the doctor, where is the doctor?**

R: The doctor’s house we would take her to the doctor’s house and check her up with the doctor.

**I: And does he give you medicines?**

R: Yes.

**I: You told me that your daughter don't usually get diarrhea but she had diarrhea, can you tell me what made her have diarrhea?**

R: Well I believe it's because of their hands. Like if they don't wash their hands or we would get distracted and they would touch anything and eat it. Sometime it could be because of the water. But like I said it could be because of their dirty hands because they would drink the water but don't get diarrhea and next thing you know they would have diarrhea. Maybe because they have bacteria’s on their hands.

**I: About the diarrhea, do you think it's serious?**

R: Yes, every illness is. I believe so yes it's very serious because I've heard that if their diarrhea gets really worse and their body is all dried up they can also die from it. So yea it's very serious.

**I: And during the times that your daughter had diarrhea how did you prevent her from diarrhea?**

R: Well we would listen what the hospital, the doctors would instruct us, give her medicines and give her lots of water. And this is what we would do except the thing is when it's been a day but when we monitor her to see if it got lesser and it doesn't we would take her back to the doctor so he would give us medicines.

**I: Can you tell me how would you know that your child needs to see the doctor when she has illness?**

R: Well like a father I'm used to it when I look at her like sometimes she would just keep crying and that's when we know that there's something up with her. When we see her and she's not happy and not lively like how we would play with her all the time, when we play with her and she's not playing we would know like if she had the flu we would touch her and we would feel heat from her body then we would quickly take her to the doctor.

**I: Can you tell me when your child get sick who do you take her to first?**

R: Her mother would take her to the doctor.

**I: Can you tell me do you guys take your child to a traditional healer or use traditional medicines?**

R: I don't believe in traditional medicines but I do believe in massaging the stomach. But our youngest daughter, she hasn't had that traditional healer massaging her stomach but my eldest child had gone through that case before.

**I: Can you tell me a story about when your eldest child had that kind of case?**

R: Let say when she had her stomach massaged, she had a fever, we took her to the doctor and we would give her medicines but when we monitor her, her fever just comes and goes, her fever doesn't disappear. So we would talk with her grandparents and they would tell us that she might have stomachache. So we would take her to a traditional healer and the traditional healer would say yes she has stomachache, then the traditional healer would massage her stomach.

**I: Can you tell me what kind of illness your child would get coming from less nutrient in her food?**

R: There's no doubt about it that I know she wouldn't grow well. She would ... how we say that again, she would slowly grow not just that her brain would also grow slow during in school. That's what I believe if my child would have less nutrient. My child would also easily get any type of illness.

**I: From your own perspective what kind of food would make your child unhealthy?**

R: Well I believe the kind of food would be the imported food, lollipops, chips, and all of those foods that are sugary. Within my knowledge that I know.

**I: Can you please explain why your child would get sick from lollipops and all of these sugary foods?**

R: Well I'm kind of one of those persons that would listen to the radio. Every program especially the health program I would also listen to that and that's one of the things I've heard. Lollipops are bad and you know those doctors that would usually come, there are times that they visit around these parts. Well they taught us that there's no vitamins in these sugary foods and it would give them toothache. That's why I know, I learned a bit.

**I: Can you tell what kind of foods would make your child healthy and why?**

R: Well I really don't know, but I've heard from the doctors and the nurses and the people that are also announcing from the radio, they say it's best to eat the foods like papaya, you know fruits and vegetables, papaya, pandana and those type of foods well that's what I know.

**I: Can you tell me what kind of illness your child would get if she eats any missing food from her dish?**

R: Maybe she would have fungus/rash on her skin and she would have wounds on her skin from less nutrient, she would become blind. Those are the only thing I would know like she would go blind, her eyesight would be weaken, and she would get wounds on her body.

**I: Can you describe one day of someone keeping themselves healthy from the time they wake up in the morning until they go to bed?**

R: In the morning they need to eat, like they should eat healthy foods. Even in lunch time and evening time. There should be vegetables and fruits in their dish every time they eat. That's how much I understand.

**I: Can you tell me the signs of a healthy child under 2 years old?**

R: Like I said before, just giving an example on my children. Almost every time, when they wake up they would play around and make us play with them they would slap us and laugh and have fun. When it's not like this we say maybe there's something wrong with them, they look sick, they're lazy and they don't eat.

**I: Can you tell me the signs of a healthy adult?**

R: Well from myself, maybe the thing that might show an adult healthy is they're active. Almost like little kids, they're not lazy. When you look at their movements it's not slow and when you look at their body it's all muscly and you would like wow you’re living a healthy life because your nerves are showing.

**I: Okay we have ended our conversation about health now we will talk about what kind of plants are planted here. It wouldn't be just plants but any kind of foods that are available here in your house. Can you explain how do you have food in your home in a day?**

R: In this house we would gather coconuts so that we would have foods, I'm talking about the foods that we buy at the store. If it comes to breadfruits, well there would be breadfruits in some times, like if we eat breadfruits this month then it would take a long time to eat them again like since we're talking about breadfruits we don't have breadfruits now. Same thing goes to pandanas they also take time to have. Ever since you got here there hasn't been any breadfruit and pandanas let's say there are but it's not time for them to be picked. So now that means we'll be eating imported foods. For us to have rice and foods, we gather coconuts.

**I: What kind of plants grow here at your home?**

R: There right there as you see them, pandanas, bananas, breadfruits and coconuts. There's no potatoes and those other plants that's all it is.

**I: Can you tell me if you do, do you sell your plants?**

R: We can sell them but for us we usually eat them and we would give some to our families at Majuro. Probably the only thing we sell is coconut meat, if people wants to buy. We don't tell people that they should buy but when they needed it at Majuro and they say they'll but well then we'll sell it. That's what we usually sell the coconut meat.

**I: Okay, you said you'd sell coconut meat. What do you do with the money?**

R: We would see what we would need, like for our children we would buy diappers, we would buy them food that there's barely available, orange, apple anyways there's barely any like it's not every week. But we would usually buy foods with the money we gain from selling.

**I: Can you tell me if you have any difficulties in growing plants at home?**

R: ... I believe I can plant, but like I said if it was me here in this house since I'm the only man probably there's not enough ... I don't know, how can I explain. But I believe I can plant, but the thing is that there's not enough time because I'm busy with gathering coconuts, because if I don't gather coconuts well then there wouldn't be any rice, flour and other foods. But I believe I can plant.

**I: Can you tell me what kind of plants would you need to plant here at your home?**

R: There's a lot of plants we want like our wants are very high, but to get it is very low. We want taro which came from the people that we hear from and those things, what are they called again? We also want to eat kiwi and those things that there aren't any here.

**I: Can you explain how easy or hard it is to get those foods you mentioned every month during the year?**

R: You want me to explain what kind of food?

**I: Like the foods you mentioned, taro and ... (Interrupted)**

R: Well about taro it's kind of hard like for myself I haven't ate taro over a year. Kiwi, it would be lucky if someone bring some or either we would be at Majuro. But here in this island we won't see any cucumber and other foods it's hard, for now those kind of foods are hard to find. The only foods that are easy to find which is rice and flour.

**I: Now tell me about the foods that you mentioned are hard to find, why is it hard?**

R: The reason why I said it's hard as in right now there's none here where I'm at like where can we get these? Like if it's Ine, I don't know but I've heard that we need a RND but we don't have any RND here. There was one a long time ago but there gone like for us, we don't really know how to plant cucumber and those other things well like me I don't know how to make compose and other things. Well for taro you know taro, you need to dig a hole well it's just me with one shuffle to dig a hole for taro I believe it would take a week. If I get busy on planting taro well then there wouldn't be any rice.

**I: Now I will ask if you raise animals. Can you tell me about the animals you raised?**

R: Yes, here at this house we only have 2 kinds of animals, pigs and chickens.

**I: Can you explain what made you raise animals?**

R: Maybe because as I was growing up and we raised animals I believe it could be because of our custom. It's almost every time we have pigs, so as I grew to be an adult I decided to raise pigs and chickens as well. Well we focus more on raising pigs like for important events like kemem (when the child turns 1 years old) and those kind of stuff, but we rarely eat them maybe only when there's a party we would eat them.

**I: Can you tell me any difficulties in raising animals?**

R: Well yes, if it's about chicken raising it is good but when there's no fence we want a fence but as I said we don't have any money to make a fence because making fence would need nails and those kind of stuff. We want to buy them but like I said I'm always busy collecting coconuts because it's the only thing that can provide us food. Now when we raise animals here and we're asleep and there's a lot theives there won't be any. No difference with the pigs, we also want them to be in a fence because the area here is so wide that when someone kills the pig we won't hear it from a distance. The thing about the pigs is that we feed it with coconuts and when there's more pigs then we'll feed them with more coconuts and we'll have less coconuts to you know. (Laughs) But a pig needs to eat like 10 coconuts, maybe 30 coconuts in a day.

**I: Then it comes to working more on collecting coconuts.**

R: That's one of the thing because we lost some coconuts to do trades.

**I: Wow, you don't say, collecting and gathering coconuts is a hard work.**

R: Yea it's really hard.

**I: Can you tell me what do you do with the animal’s feces?**

R: Now at this time like I said there's no fence to put the animals in right, there's nothing I had done to the animals feces because they're out there roaming around. There hasn't been a time.

**I: There are sometimes foods that we wish we could eat, but for some reason we cannot do so. Could you tell me about any foods you wish your family could eat but cannot?**

R: Well this family I don't know if they're following other families I don't know but my wife and I we wish we could eat cabbage but I know where we could find cabbage if it was here in this island. Like the things we would like to eat is cabbage, eggs well there's eggs, but the eggs here are very expensive. But the food we wish to eat is cabbage so we can mix it with other foods.

**I: Can you tell what is preventing your family to eat these cabbage?**

R: Well the difficulty is that there aren't any here. If it's here well there's no cabbage. But I believe if there were cabbages here we would eat them every day.

**I: Okay for our last question on food. Who in this family decides what food this family should eat?**

R: Everyone, let’s just say me and my wife. We would talk to each other okay bring mackerel, corn beef, not good not good, so bring just mackerel because if we bring corn beef it would be like cook it like this like that. I also say that it's expensive and it's not enough.

**I: How do the family decide their foods?**

R: It's like what I said me and my wife decides the foods we would plan it, like what will we eat for breakfast for tomorrow? Oh pancake, what about lunch? You go fishing, I'm feeling lazy but I need to cook the coconut, then I guess we'll eat mackerel. But the foods that we already have here in this house which is easy to find, it's rice and flour. Then when it comes to other days to decide what foods we should get then that's when my wife and I decides what to eat for the day. We would talk and see like if its meat then we'll only talk about what kind of meat.

**I: Who decides the food that your children should eat?**

R: Also both us.

**I: We have finished our conversation on foods, now we will talk about water and hygiene. Can you please explain how do your family get and store your water?**

R: Drinking water?

**I: Drinking water, water for showering ... (interrupted)**

R: At this we have water catchments. We don't have wale water.

**I: Where do you usually get your drinking water, cooking water, washing water and showering water from**?

R: From the water catchments.

**I: What difficulties do you face when it comes to getting water?**

R: Well maybe because there is only a few of us here and we have plenty of water catchments and to us it's plenty enough for us. Except, when was it? A few months back when the wheather didn't rain that much well that's one of the difficulties here.

**I: What about the difficulties in storing water?**

R: ....

**I: Is there any?**

R: Well I'll talk about nowadays, how many water catchments we have there 1, 2 water catchments and 1 cement water catchment. The way I see it for us it's enough.

**I: It's enough. There's no difficulties in storing water?**

R: But also that's what we want, we want more water catchments for our house.

**I: Can you explain why do you need more water catchments?**

R: Because like I told you we don't have wale waters, but we wash, we shower, let’s say this water catchment one water catchment is only for drinking or cooking, while the rest are for showering and washing. So I want it so that we can have more water catchments because we want to be prepared for like what happened a few months back. Well we want to be prepared and ready for that.

**I: Can you explain how do your family keep your drinking water clean?**

R: About... The thing we would do is check the roof of this house and the gutters. Those are what we check on. And if it's dirty, then we would dump out the water and clean it but it's not every often we do it.

**I: Okay about the water catchments that you drink from, can you tell me how would clean it?**

R: If we see that the water catchment is dirty we would dump the whole water and wash it and prepare it for refill.

**I: When you wash out the water catchments you use only water?**

R: Water, soap, sometimes we use chlorine.

**I: Okay now we will talk about washing hands. Could you describe in details on how your family washes their hands in a day?**

R: Okay about washing hands we would usually use soap and water. We would soap our hands and wash them, we would tell our kids to use soap when they're washing their hands.

**I: How do your children wash their hands in each days?**

R: They would usually wash their hands only when they're about to eat. When it's time to eat us adults we would tell them heeeey wash your hands before you eat. Maybe I think, maybe they would wash their hands 3 times a day because we would remind them to wash their hands when it's time to eat, before they eat.

**I: How would a child under 2 years old wash their hands?**

R: The mother would wash her hands but the mother usually feed her.

**I: In a day how many times do your family use soap?**

R: When it's time eat, in the morning, afternoon and evening they would use soap.

**I: In your thoughts, what is the difference between washing your hands with water only and washing your hands with water and soap?**

R: As we know, the soap would clean your hands. The reason we would believe it is because as we were growing up and they tell us to always use soap maybe that's why we would use it so that our hands can be clean. For me I think it's better to use soap than just using water. Maybe because I've heard it a long time ago and I've been remembering it.

**I: Can you tell me what's the prevention of using a soap in a day?**

R: It could be maybe the time when everyone is busy like both of us we could like yell at these kids that are old enough but when they're hungry like, let me give an example when they're about to eat, don't forget to wash your hands. But I believe sometimes they wouldn't because they would be in a hurry. They would like wash their hands and show it to us but we're a bit far and we see their hands and there are we and we're like okay they washed their hands but we don't know if they used soap. We didn't really check because like for me I would try and finish up gathering coconuts because it's closed to dawn and it has to be finished.

**I: Thank you for answering. Now can you describe what type of toilet facility you have here at your home?**

R: This house uses toilet bowl.

**I: If you know, can you tell me why this home chosen to use toilet bowl?**

R: Well me this isn't my home, this is my wife’s home and ever since I first came here they already had a toilet bowl. I believe maybe my wife’s parents chose it but I really don't know or maybe because it's better to use toilet bowl than using around there.

**I: In some communities, we have heard that defecating in the ocean and lagoon side are still happening. Can you tell me is this practice still on going and how common it is here?**

R: Well from my own thoughts if it's about this community. I believe this practice won't disappear, maybe it's inherited (laughs) I don't know. Maybe people are used to it. Also kids like in this neighborhood we would wash up our kids. Let me try and explain. If a child says he/she wants to poop to if it's their mother, the mother would already know that she'll need to fill up a bucket to wash the child so she would tell her child to quickly go to the beach. That's probably why this practice won't disappear. Maybe it could if there could be ... I don't know maybe lessons and those kind of stuff so that it would stop.

**I: Can you tell me why some communities does it and some don't?**

R: That's another thing, if it's about here maybe some would have toilets. Maybe half of this island don't use toilet facilities. Also that could be the reason why people don't use the toilet because they don't have a toilet at their home. But this won't disappear I believe so. Like even if they have toilets for example this like this house, even though we have still the children would use the beach. When their stomach isn't feeling well they would quickly run towards the beach.(Laughs)

**I: It's faster right?**

R: It's faster and closer.

**I: About your children, do they use diapers?**

R: Yes.

**I: What do you do with it?**

R: We bury it. At this time there is a whole that I already made. It's for diapers for me to bury them.

**I: Where do you bury them at?**

R: Next to the lagoon side. I would make a whole next to the lagoon.

**I: For us to end our question on cleaning, can you explain on how to prevent spread of disease?**

R: Like what kind of disease?

**I: All type of disease.**

R: One is clean our hands, keep telling your kids to always wash not just the kids but also us adults. I believe because sometimes when we're busy, if it's coconut, we're still gathering coconuts and it passed 12 and we haven't ate and we're still busy they would bring us food but we haven't washed our hands but still we would just eat anyway maybe the thing is that we should teach each other on cleaning. And I believe cleaning would prevent getting disease not just that but also we should tell them not to defecate the beach but if they do I think maybe it's better if they bury it if they're defecating.

**I: Now can you tell me within your own perspective do people get illness from feces?**

R: Yes. I understand that a fly would land on a feces and then come and land on our food and I know there is 2 types of illness we can get. I still remember it back then when I was listening to the radio. We would get amoeba and diarrhea.

**I: Okay we have finished our conversation on cleaning. Now we would also like to know the role and responsibilities different family play in raising their children. Could you describe the care of children throughout the day in your community?**

R: Well I know that a mother’s role and responsibility is to raise the children like look after the kids and maybe the father’s role and responsibility is to make sure there is food on the table. But about watching over the kids from the morning till evening is the mother. Sometimes when the mother is busy she would give her youngest child to those who are older than the child, as in if the child is below 2 years old or even 2 years old. That is what I see in different families’ role and responsibility within the community.

**I: From your own perspective, what is the responsibilities of a mother in child care?**

R: They need to make sure to feed their children first, washing them up, look after them, be with them. Watch over them so they don't play in unsanitize area. Let's just say that a mother is the most person who would be with their child.

**I: Do you know what the responsibilities of a father in child care?**

R: Well about the life here that I have seen, I see that a father doesn't really have time for a child care. They're busy with gathering coconuts and stuff like that maybe the only thing that they would be with their children is if the mother is sick and when the mother is busy cooking. Also at night time when there's no more work to do then they would be busy with their children. But a father’s responsibility is to make sure there is food so that there's food for their children.

**I: Is there any caregiver that would look after your children when both of you are busy?**

R: Yes, sometimes we would have a babysitter. We would sometimes give our children to one of our family members.

**I: How do the caregiver play with children under 2?**

R: Well if it's the kids here they would play by the road. They would play with marble balls and those things, they would run around. But we don't really see it because we're busy with other stuff. But they would play around the road.

**I: Has the grandparents been around when your youngest child is still here?**

R: Yes.

**I: Could you talk about the role of grandparents have in raising children in this community?**

R: Well if it were this house when the grandparents were here, well their grandchildren would keep bothering them. Like if the grandmother would go somewhere outside she would take her grandchildren with her. She would do whatever she's doing while her grandchildren is just sitting down next to her and playing.

**I: Can you tell me ways that a grandparents support in raising children, support mothers and families?**

R: Yes there are times when they were here they would bring foods from where they're coming from. When they see the mother or the father and they're busy well then they would take their grandchildren and care for the child.

**I: Within your own perspective, what makes good grandparents?**

R: Well maybe from my thoughts, when they’re with their grandchildren and they would watch over them and teach them also tell them stories about our past and teach them about bibles. They would be happy to be with them and they would spent their time with them.

**I: Could you talk about the role that other family members have in raising children in this community?**

R: You’re asking about any children or the children in this house?

**I: The children in this community.**

R: Well what I see in life nowadays, it's getting hard. For example, if the children from that house plays around and we see them the only thing we would do is telling them to make sure they don't play in the mud. Like stuff like that and I think it's not good.

**I: Tell me about the ways that a siblings help raise young children?**

R: For example, if the child is 2 years old and a child that is 9 or 10 years old. Well they love them if it were some other kids but they would still want to have fun while they're still in a young age, even though they see that there's a mud or an area that is not good for child to sit and touch but they would watch over them they want to watch over them because they love them, but they would easily get distracted.

**I: We're almost finished just a few more questions thank you very much. Could you explain where you usually get trusted information about nutrition and health?**

R: I believe that would be with the doctors. I've also heard about it with those doctors that knows about nutritious and stuff like that. But I believe at the end of the day the doctors and teachers would teach others about nutrition and stuff like that.

**I: Can you tell me the reasons why these sources are trusted?**

R: Well from my knowledge I know that a doctor has the knowledge and know about the nutrition and what illness we would have and that a nutrition can help us with our health because they've studied it. Also in schools there's also lessons about foods which is why I also trust the teachers.

**I: Can you tell me where nutrition and health messages should be delivered so that you would see/hear them most easily?**

R: Sorry can you say that again?

**I: The messages on nutrition and health where do you think it should be delivered so that you would see or hear them every day?**

R: Well one of the things is that there's no newspapers here. Probably the only thing we could do is go to the doctor I don't know if the doctor could draw so that we would have a paper but there aren't any here. But if there were more doctors they could assembly everyone so that everyone would learn. They would meet the pastors so that they can tell them about health.

**I: So what you mean that these messages should be with the doctors so that it would be easier for all of you to see or hear. Where else do you think that these messages should be so that all of you be able to see or hear it every time?**

R: Well yes also that these messages should be delivered to the pastors in every church like you know that there's this program called Sunday school. I believe that this is a good idea to teach the pastors for the times when they're teaching them bible there should be a bit of time to teach them foods, cleaning hands and the groups here are weak, there's groups for women’s, but they mostly focus on making money. Instead of just the doctor I believe these messages should also be delivered to the pastors so that they can teach these groups. So that when it comes to Sundays they should teach each other especially to the children’s.

**I: Can you tell me what types of media do you use the most to communicate?**

R: Well for me, I mostly use radio. But there are rarely any radio.

**I: So you would take messages from doctors and radio.**

R: Yes.

**I: When you think about your own parenting behaviors, can you explain what influences how you raise your children?**

R: Well for me I don't know, maybe spirits or what..? Just myself I would say spirit. Not spirit as in spirit but maybe how can I explain this? Like when I had my child it's like there's something that I felt that it hit me. Like if my child cries I would want to know why my child is crying like if someone made my child cry I would make that person cry as well. Because of this it just like just click within myself like for example when it's in the afternoon let's say she's hungry she needs to eat but still I don't know, maybe it's just something that I feel. When its evening I would like has my child ate yet? And they would say oh she's about to eat now then I would like has she washed her hands? I don't know because no one has told me anything about this feeling that's why I say it's probably spirit. Like this feelings that I'm having I put myself as my daughter and every time when she's about to eat I want her hands to be clean. I don't know how to explain it in a very well detail but I just say spirit. It's not spirit as in the Holy Spirit, I don't know how to say it. It just automatically appears. You know what I mean?

**I: I understand what you mean I think the word that best fit would be love.**

R: Right. Maybe love, because they don't ask if we're hungry but I would first ask has our children ate? They ate, are you sure? Did you really feed them? Like that, maybe it's love, your right it's love.

**I: Can you tell me how opinions of the community influence how you raise your children?**

R: Well if it's here, I don't know if you've heard an old news from v7ab that I've heard a long time ago. Children’s are growing up with what they see and I believe if it's what you said about teachings well there's none here. But if it was a person like me maybe I saw how they raised me and I'm just following what saw in raising. But teaching about that, there's rarely any, I don't know if there's been any maybe it still hasn't. Except when your children is sick and you take them to see the doctor then the doctor would give instructions and we would learn from it. But if it was raising our children maybe how we were raised is what we would do the same for our children.

**I: Can you tell me has there been any advice or information related to parenting you've received?**

R: Yes. I've heard that we should love our children and cherish them. I would say that it's better to lose money for them to have a healthier life and like be able to provide for them. But yea, I usually hear it from our children’s grandparents.

**I: So your wife’s parents are the ones that usually give you advice and information?**

R: Yes.

**I: Is there anyone else other than your wife’s parents that gave you advice or information?**

R: Yes, the doctors and the nurses. There's this team of nurses that usually come here and give shots to children’s here. It's also good enough that the nurses come here and help out and tell us how we should look after our children’s. But as I said before, they barely come, it would take about 2 to 4 times we would see them again. Like the team that came and gave shots here they really focus on their job, they would scold us on how we should take care of our children and sometime we be scared. Like instead of trying to teach us professionally they would rather scold at us when they see our mistakes in taking care of our children. But anyways about your questions yes I've heard it from the doctors and the nurses.

**I: Can you tell me is there any desired information on parenting you wished you had but is not available?**

R: yes, I really want to. Like about nutritious and foods, I really want to learn more about foods that are good and foods that are meant to do what they're supposed to do. Also I wanted my children’s mother to understand what she's supposed to do like what they’re supposed to know what to do with 1 or 2 years old. Yes I really want to learn from these things but because we're here in this island I don't know who we can get close to, to learn.
