# Supplementary material for: Conceptualizing multi-level determinants of infant and young child nutrition in the Republic of Marshall Islands–a socio-ecological perspective
Source: PLOS Glob Public Health. 2022 Dec 19;2(12):e0001343. doi: 10.1371/journal.pgph.0001343 (PMC10022247; doi:10.1371/journal.pgph.0001343)
Supplement: S1 Data — (ZIP) [file pgph.0001343.s001.zip › RMI Supp Data/Interviews data/I50R_IDI_MCG_Arno_Sep 14_BM.docx]

- Interview Code: I50R
- Interview type and Interviewee: Male Interview
- Interview Date: 9/14/18
- Location: Arno
- Interviewer: BM
- Transcriber: BM

**I: Do you agree to let me record our conversation with this recorder?**

R: Yes.

**I: Let us begin with, can you tell me about your family?**

R: About my family?

**I: Yea.**

R: Good.

**I: Can you tell me how many are in your family in this house?**

R: There is only 4 of us.

**I: Who lives here?**

R: Cola, Fibay, Kobe and Mike.

**I: How many boys and girls?**

R: 4 boys and 1 girl. 2 of them are in Majuro for school. 2 of the boys are in Majuro. Also the sister is also in Majuro.

**I: But what about in this household, how many are you?**

R: Only 4.

**I: Only 4, which means it's you, your wife and your 2 children. Your children how old are they?**

R: One is 3 years old and the youngest one is 1 years old.

**I: And they're all boys?**

R: Yes, all boys.

**I: Can you tell me about this community?**

R: Like about what?

**I: Like what's the positives about this community.**

R: Well about this community ... It's good to be here ... Sometimes it's good to be here but sometimes, you know, because there's no light and things like that. But it feels good living here in this community. I'm from Ailinglaplap, I a drifted all the way here and I caught a fish here and made 3 more fish. I've been here since 2006 until now which is 2018.

**I: Wow that sure is a long time.**

R: Yea it is.

**I: Can you tell me if there is any negative things here in this community?**

R: I haven't seen any negativity here in the community.

**I: Oh so there aren't any?**

R: Yea, everything here is very good.

**I: Now we will talk about health and illness within this family. Can you tell me what kind of illness has your child had?**

R: They usually get fever and cough, I think these are the only illness they had. Fever and cough.

**I: Can you tell me what was the cause of your child to have fever?**

R: I don't know.

**I: Its okay, if you don't know it's fine. Do you know how serious it was when your child had the fever?**

R: It was very serious.

**I: How did you prevented the fever?**

R: We wet a cloth and put it on their heads. We cool their heads so that they don't get even worser.

**I: Okay, now on coughs how did you prevent it?**

R: I don't know as well.

**I: Do you know the serious of your child having the cough?**

R: I don't know.

**I: If you don't it's still okay. Can you explain the ways you prevented the cough?**

R: Give them water.

**I: Can you describe how you know when your child needs treatment for illness?**

R: Because we would know like when they've been sick for a few days then we would take them to see the doctor. We would go with the doctor and sometimes he would give us medicines. He would tell us to give our child a medicines and timing it so that we can give him another. We would follow the doctor’s instructions. Then it'll come down to when our child is healed.

**I: Who is the first person you take your child to when he has illness?**

R: My son that is the youngest, the one that is 1 years old, well he rarely get sick. We would take him to the doctor. So the doctor would give us medicines to treat his illness.

**I: Do you use traditional healers and traditional medicines?**

R: No we don't.

**I: Can you explain why don't you use traditional medicines and traditional healers?**

R: Because we don't know.

**I: Could you describe any illness affecting your children that are associated with nutrition?**

R: What was that?

**I: What type of illness that would affect your children that are associated with nutrition?**

R: ... Man like what would it be? Like he doesn't eat enough food or what?

**I: Just within your perspective what kind of illness would your child have associated with nutrient? There are no right or wrong answers.**

R: Nothing. I think there's no type of illness.

**I: Can you tell me what kind of foods your child would be unhealthy with?**

R: What kind of foods?

**I: Yes.**

R: That is unhealthy for my child?

**I: That's right.**

R: ... (Shrug his shoulder)

**I: It's fine if you don't know. Can you tell me what kind of foods that will make your child healthy?**

R: Foods that we make for them. Like bananas, coconut meat (the cotton part), we would give them coconut juice for them to drink, we would feed them fish.

**I: From your own thoughts do you know what kind of illness would your child have coming from foods missing in the diet?**

R: I really don't know.

**I: It's okay. Could you now describe for me a typical day of someone living a healthy lifestyle, from the time they wake up in the morning until when they go to bed?**

R: Yes. Like what can we do? (Talking to himself) What like what can keep them healthy?

**I: Can you describe someone living a healthy lifestyle in a typical day?**

R: We would look after them and feed them these foods that are... healthy lifestyle? We would feed them and when it's night time it's time for them to sleep. They stop sleeping when they're awake.

**I: Can you describe more about living a healthy lifestyle?**

R: I think that's pretty much it.

**I: Can you tell me the appearance/signs of a healthy child under 2 years?**

R: ... I don't know.

**I: Can you tell me the appearance/signs of a healthy adult?**

R: I also don't know.

**I: Now we will talk about foods that are available here. I would like you to explain how your household gets food to eat on a daily basis?**

R: Yes like what, changing?

**I: Can you explain how you get foods for your house on a daily basis?**

R: We'd collect coconut, like if the store we would loan foods so we would have food every day. Like if it's in the morning we would have flour, then when it's lunch we would go and loan foods for lunch and also for dinner. Let's just say that we would loan foods from the store like foods for that day.

**I: So how do guys get foods, like you would trade the coconuts that you gathered for foods at the stores?**

R: Yea basically. Like if you would gather coconuts form Monday to Friday then you would trade them on Saturday to clear your debts so that you can keep loaning for foods.

**I: What kind of foods our grown here in this house?**

R: Every kind of foods like coconut, pandana, breadfruit, papaya but the thing is there's no pumpkin.

**I: Just those are grown here?**

R: Yes.

**I: The foods that are grown here in this house, do you sell them?**

R: Sometimes we do sometimes we don't.

**I: Okay you said that you would sometimes sell the foods that are grown here in this house... (Interrupted)**

R: Just last year.

**I: Last year? Okay then what did you do with the profits that you have gained from selling the foods?**

R: We would make about 100 then we would buy small things. Like if diapers, diapers... The things we would buy are the things that are for kids, we would buy them like cereals, and other things like that.

**I: Can you give me more details on the other things like that?**

R: Like clothes, tang and waters.

**I: Can you tell me about any difficulties to growing foods here in this house?**

R: What was that?

**I: Is there any difficulties on growing foods here?**

R: I don't know.

**I: There's no difficulties on growing foods here?**

R: None, there's no difficulties.

**I: There's nothing?**

R: Yes.

**I: What foods would your family need to grow here at your home?**

R: Pandana, foods that are barely grown here like potatoes, pumpkins, and watermelons. But there aren't any.

**I: Could you explain how easy or difficult it is to get those foods you mentioned every month during the year?**

R: We can like what? We can go to Majuro and go to the farm like buy seeds and things that we would need. We can bring them from Majuro.

**I: Is there any other food shortages throughout the year and their main causes?**

R: Sometime there wouldn't be any foods.

**I: Can you explain what was the causes?**

R: Sometimes when we're sick, like for a man, he should look for foods but when we're sick it's hard to get any.

**I: Like to gather coconuts so that there can be foods?**

R: Yea like gathering coconuts or like fishing or like climb a coconut tree.

**I: What do you do to feed your family if there is shortage of food in the household?**

R: I would bring iu (coconut meat that is cotton) and aekiu (coconut meat soup).

**I: Now we will ask about animals that are raised here. Could you tell me about the animals that are raised here?**

R: None.

**I: There's no animals that are raised here?**

R: None.

**I: Well then can you tell why aren't you raising animals?**

R: There hasn't been any animals.

**I: As in you haven't had any time to look for animals?**

R: I haven't had the time to look and raise. Before there was only pigs and chickens, but the people here would steal and steal and steal until we have none.

**I: So you don't say it's because you had no fence for the animal’s right?**

R: Yea. Also you know Katwil? He would come and take a lot of animals here. He would usually come and kill the pigs so that he would take them and eat them.

**I: And we can't say no because he's the chief.**

R: Yea. Like the times when I had animals the chief would come to me and take the animals since this is his land. Not just me but also the people that lives here.

**I: Okay, may we proceed to the other questions?**

R: Sure.

**I: Could you tell me about the foods you wish your family could eat but cannot?**

R: Foods on Majuro.

**I: Like what?**

R: Meats and kinda like that.

**I: What kind of meat?**

R: Chicken, cow, hotdogs not hotdogs. Just those, chickens and cow.

**I: Like cases of chickens and cows?**

R: Yea, we rarely eat any of them.

**I: Now that you told me about the foods that you wish your family could eat. What is preventing you from eating these foods every day?**

R: There's none here in the island.

**I: Is there any other reasons?**

R: (Disagreement with their head)

**I: Okay than, for the last question on foods, who in this family decides what food this family should eat?**

R: Me.

**I: Just you?**

R: Yea.

**I: Can you tell me how your family chooses the foods?**

R: Well they would go to a store and just pick the foods. Like they would get rice because there's no rice back home. They would get milk, milk carnation they would also get it. Can foods if they want can foods, usually corn beef or mackerel, ramen, onions they would dice it. That's about it.

**I: Who in this family decides what the children should eat?**

R: Also me.

**I: Now we will talk about water and hygiene. Can you describe a typical day getting and storing water for your family?**

R: There's a drum and there's a water catchment, we usually fill up the gallons and store it here in the house. Like we would be prepared when we wake up there's water to drink.

**I: Can you tell me the main source of water for drinking, cooking, washing and bathing?**

R: The water catchment.

**I: What are the main difficulties in getting water?**

R: There's none.

**I: What are the main difficulties in storing water?**

R: There's also none.

**I: There's no difficulties in storing water?**

R: Yes.

**I: Can you tell me the ways that this family tries to make drinking water safe?**

R: When it rains. When it rains we would use chlorine and soap to clean inside the water catchment then we would empty it. Then we would rinse it and fill it up with the rain.

**I: Now we will talk about washing hands. Could you describe in details your family's hand washing throughout the day?**

R: Sometimes they would wash their hands sometimes they wouldn't wash their hands.

**I: How do your children washes their hands throughout the day?**

R: Their mother would bring a small bucket and fill it up with water. She would wash their hands when they want eat, like this one she would take him and wash his hands.

**I: How do children under 2 years washes their hands?**

R: Their mother would soap their hands.

**I: The mother uses the soap?**

R: Yes.

**I: She just use the soap or soap and water?**

R: She soap their hands and rinse it with water.

**I: Can you tell me the times during the day when soap is being used?**

R: Every day.

**I: Like every day, how many times would it be used in a day?**

R: Only the times when we're about to eat.

**I: The times when you’re eating, like morning, afternoon, and evening?**

R: Morning, afternoon, and evening. 3 times a day.

**I: Can you tell me what prevents washing hands with soap throughout the day?**

R: Can you explain that again?

**I: Has there been a day when you don't use soap?**

R: Oh yes yes yes.

**I: What prevented you from using soap throughout the day?**

R: There's no soap in the island. Like we would run out of soap.

**I: Can you tell me what type of toilet facility do you have here in this house?**

R: There's a bathroom.

**I: Bathroom, as in what type of toilet?**

R: Toilet bowl.

**I: Can you tell me the reasons why you chose this type of toilet?**

R: Because it protects the community.

**I: We have heard that some people would defecate the ocean and lagoon side. Can you tell me if the practice is still on going?**

R: Tell you about what?

**I: The people defecating the ocean and lagoon side.**

R: Like if it was other households and they don't have any toilet bowl they would usually defecate the ocean and lagoon side. Because they don't have any toilet facility. If it's day time they would defecate the ocean side, if it's night time they would defecate the lagoon side.

**I: Can you explain why this practice is done in some places and not the other places?**

R: Because some places have toilet facilities and some don't. That's just one of the difficulties.

**I: Within your own perspective what are the barriers in using the toilet?**

R: With us there's no barriers.

**I: Okay with you and your families but what about other places?**

R: Probably because they're lazy to build a toilet facility. But one of the importance in a community is toilet facility.

**I: How are young children's stools are typically disposed of?**

R: We would dig a whole at the beach at the lagoon side and bury them.

**I: Can you explain where your young children usually play each day?**

R: On the road.

**I: On the road?**

R: Yes.

I: Does your children play where there are animals?

R: There's none. There's none because we watch over them. Just ants, the ones that are red that are so annoying. Like these ones here.

**I: What are the difficulties where your children’s play at clean?**

R: ...

**I: Does the area where your children play in always dirty?**

R: Yea.

**I: Then tell me what are the difficulties in keeping it clean?**

R: Nothing. Like if our children would play on the road, we would look and see that it's clean.

**I: It's always clean?**

R: Sometimes I would go and rake it in the morning.

**I: Rake it?**

R: I would rake it so that it would be better for them to play in.

**I: To wrap up our questions on hygiene, could you explain ways to prevent the spread of disease?**

R: Don't know

**I: Can you tell me what you think of the connection between exposure to feces and illness?**

R: Yes.

**I: Can you explain?**

R: Because it's dirty, it would be dusty and stuff like that.

**I: Now we will talk about the responsibilities of different families in this community. Could you describe the care of children throughout the day in your community?**

R: They would wheel barrow them. Sometimes they would take them to the beach. They would play with them. They would put them in a wheel barrow and walk around with them. At night when they would sometimes put them in the wheel barrow to put them to sleep.

**I: Who is mainly responsible for child care?**

R: The mothers.

**I: What are the responsibilities of mothers in child care?**

R: I don't know.

**I: Then do you know the responsibilities of fathers in child care?**

R: The fathers, their always tired. Because they would look after the child like they would carry them from the morning till evening. They're always tired, it's hard and when they're about to rest they would eat a little bit because they want to rest because they're tired.

**I: Can you tell me how caregivers play with children under 2?**

R: They would play with them.

**I: Can you give me more details?**

R: They would build a toy car and they would push the car around on the road, they would run around with it. They would also take them to the beach and play with them.

**I: Could you talk about the role of a grandparents have in raising children in this community?**

R: I don't know.

**I: Could you explain the ways that grandparents support in raising children, support mothers and families?**

R: They would watch over them. They would help out with the family. If it was my child that is 3 years old, they would talk to him.

**I: Anything else?**

R: No more.

**I: Can you tell me what makes good grandparents?**

R: I don't know.

**I: Could you talk about the role that other family members have in raising children in this community?**

R: I don't know about the other families.

**I: You don't know about the other families?**

R: I don't know, just my children.

**I: Could you explain where you usually get trusted information about nutrition and health?**

R: With Jack.

**I: And where is this Jack?**

R: At the hospital. That's where I would go to.

**I: Can you tell me why these sources are trusted?**

R: Because there where we would get information from, from the hospital.

**I: Tell me where nutrition and health messages should be delivered so that you would see/hear them most easily?**

R: It's better if these messages were by the road. Like billboards.

**I: What types of media you use the most to communicate?**

R: Radio, V7AB.

**I: So you would listen to radio?**

R: Yea I would but now I don't have one.

**I: You would listen to a radio with your neighbors or what?**

R: I would usually walk around because I would look for a radio to listen to because I don't own a radio.

**I: When you think about your own parenting behaviors, can you explain what influences how you raise your children?**

R: I would like if they were like at the beach swimming I would look after them so that they wouldn't drown, or like a tub that's been filled and they forgot about it, or like the wale waters that are around the houses, I would really watch them. So that they wouldn't fall into. They're stuck with me. When I gather coconuts I would bring them along sometimes. One would be with me and the other would be with the mother. I would tell my wife, make sure they don't go to the beach, and make sure they don't go under the coconut trees because a coconut might fall and hit them on the head, make sure they’re not under the breadfruit tree. That's how we would remind each other, this family would remind each other about the kids.

**I: Tell me about how opinions of the community influence how you raises your children?**

R: From our mother and father. From our grandmother and grandfather.

**I: They taught you?**

R: Yea, they taught us.

**I: Was there any advice or information related to parenting you received?**

R: Yes.

**I: Where/who the advice or information came from?**

R: From my parents and grandparents.

**I: Do you have any desired information on parenting you wished you had but is not available?**

R: Yes I want to learn.

**I: Like what would you want to learn?**

R: Like how to take care of a child, watch over people and stuff like that. Like how you would say it, I want to achieve a lot of goals.
