# Supplementary material for: Conceptualizing multi-level determinants of infant and young child nutrition in the Republic of Marshall Islands–a socio-ecological perspective
Source: PLOS Glob Public Health. 2022 Dec 19;2(12):e0001343. doi: 10.1371/journal.pgph.0001343 (PMC10022247; doi:10.1371/journal.pgph.0001343)
Supplement: S1 Data — (ZIP) [file pgph.0001343.s001.zip › RMI Supp Data/Interviews data/I51R_IDI_CL_Arno_Sep 14_BM_Shanteedited.docx]

- **Interview Code: I51R**
- **Interview type and Interviewee: Community Leader Interview**
- **Interview Date: 9/14/18**
- **Location: Arno**
- **Interviewer: BM**
- **Transcriber: BM**

**I: Do you agree on letting us record our conversation with this recorder?**

R: Yes.

**I: Thank you for your time. Before we start can you describe your role as a community leader?**

R: Yes, my role here would be the responsibility of a head teacher. I would check on reports, I would check about the school areas, and I would look after the students when they’re in campus, I would work with the teachers here on how to work in school.

**I: Can you please tell me about who is a part of your community?**

R: We have a world teach here that works here in this school. We have teachers that were hired from PSS. We have pastors, councilman, and mostly parents in this community.

**I: Can you tell me about the community structure and leadership hierarchy?**

R: Yes, the leaders of this community would be pastors, councilmen, and leaders of each community and teachers.

**I: Can you say, other than Marshallese what other foreigners are in the community?**

R: The only foreigner here is the world teach that teaches here in this school.

I: Where did the world teach come from?

R: He came here from Majuro in one of the departments in PSS.

**I: But do you know what country he came from or what country he’s from?**

R: They didn’t say where he came from.

**I: Can you tell me about religion and its influence on community members?**

R: The importance of a church in a community would make people’s life a much better life. They would put everyone in the community to combine as one and work with each other and mostly change their behavioral lifestyle so that nothing bad would happen.

**I: Now could you tell me about the positive things about your community?**

R: The positive things in this community, number 1, I would see that the people here in this community they would combine together as in they would work together in cleaning and kind of like that. They would gather and have a meeting like to keep this community pushing forward.

**I: Can you tell me any negative things about your community?**

R: The negative things in this community is that sometimes we would have problems with water if there’s lack of water catchment, sometimes we have lack of transportation to move from one place to another and the most thing that gives us a little problem is the lack of lights like solar and stuff like that. It’s not enough in the community that the students can’t do their studies and the people here in the community doesn’t able to finish their work.

**I: Tell me any food-related difficulties in your community?**

R: Well about the foods, the foods here in the Marshall Islands and because we’re Marshallese, the difficulties on food is that sometimes we would have no time to prepare it, like bringing in fish from the water they want fish, prepare coconut meat (cotton part) if they want coconut meat but still we would prepare food even if it’s past time to prepare food.

**I: Let’s now talk about health and illnesses in your community. Could you explain what illnesses children commonly suffer from in your community?**

R: The kids here in this community usually get asthma, some of them would get rash, and when it comes to the part where they don’t have enough nutrition some of them would have problem with their children and some of the students would usually go home because they say that they have a stomach ache.

**I: But is there been any that has diarrhea?**

R: Yes, there are times when they have diarrhea.

**I: What about fever, has any child ever taken fever?**

R: Yes, also fever.

**I: Can you tell me the cause of asthma?**

R: Asthma, well they say that asthma is also a cause here in the Marshall Islands like they would use any medicines to prevent the asthma from getting worsen. But sometimes some would just grow older and just get asthma and there’s some times that people would automatically just get it.

**I: Can you tell me the seriousness of asthma?**

R: Well it’s a bit serious. But if they have way too much asthma well then they could die from it.

**I: Can you tell me ways to prevent asthma?**

R: Well the asthma I’ve heard of its prevention and I would sometimes see them when they’re giving medicines and lower their fevers but I don’t know if there are any other ways to prevent asthma. There are some people that would take their children to massage them.

**I: Okay you also said that children here would also have rash. Could you tell me what was the cause of it?**

R: They would get rash if they have fever if their fever is high.

**I: Can you tell me the seriousness of rash?**

R: Very serious, the fever. Like if their fever keeps getting higher and they don’t lower it down then they could also die from it.

**I: Okay, but what about rashes, how serious is rash?**

R: Oh, rash they’re fine, not that serious. The rash would stay then disappear, it’s not like fever and asthma.

**I: Can you tell me ways to prevent rash?**

R: One of the ways to prevent the rash from getting worsen is using Marshallese oil on the skin where there’s rash on. That’s what I usually see when their kids have rash they would use Marshallese oil and put it on their skin.

**I: Do you know the type of rash it was?**

R: Well they didn’t say.

**I: They didn’t say what type it was?**

R: Nope they just say rash.

**I: Can you tell me what was the cause of a child to have a stomach bump?**

R: It comes from dirty water if not then it comes from when kids would play and they would fall down and land on their stomach which would lead to stomach bump.

**I: Can you tell me the seriousness of stomach bump?**

R: Well stomach bumps is really serious it can also cause fever. But you know when the pain gets really worse than the child could die from it.

**I: Can you explain ways to prevent stomach bumps?**

R: Number one would be that some would notice that the water might be the cause of it and they would boil the water. The second is so that they can prevent from stomach bumps, they would use traditional medicines.

**I: Like what kind of traditional medicines?**

R: Like give them coconut to drink or give them green noni to drink. They also need stomach massage.

**I: Now diarrhea, tell me the cause of it?**

R: Diarrhea comes when the water or the food is dirty. Also when they’re eating different types of foods like mix of foods all at once.

**I: Tell me the seriousness of diarrhea?**

R: Well it’s also serious. As in if they keep having their diarrhea and it keeps going then it’ll come to the point when their body is all dried up and kids can die from it.

**I: Can you explain the ways to prevent diarrhea?**

R: Some would give them medicines for diarrhea while some would use traditional medicines.

**I: We talked about fever before. Can you explain the causes of fever?**

R: Well their fevers usually comes from when they sweat from under the sun and they would stay under the cold then they would catch a fever. Also some would catch a fever from sleeping under the coldness like they would sleep outside and they wouldn’t use blanket to keep them warm.

**I: Can you tell me the seriousness of fever?**

R: Well fever is also serious because if the fever gets worsen then they could get paralyzed from it and they can die from it.

**I: Can you explain the ways to prevent fever?**

R: They would lower their fever, they would use water to cool down their fever and also to prevent them from getting paralyzed.

**I: Can you explain what type of treatment people in your community seek for their children, for example traditional healers, doctors, nurse?**

R: They would take them to the doctors.

**I: Can you tell me who they first go to for healthcare?**

R: They would first take them to the doctors.

**I: Can you explain to why?**

R: Because the doctors know how to heal their children from their illness also because they know how since they learned about illnesses.

**I: Can you tell me about the use of traditional healers and traditional medicine?**

R: They also use traditional healers and traditional medicines.

**I: Can you describe any illnesses associated with nutrition that affect children in your community?**

R: Well the types of illnesses that they would get is diarrhea, hook warm, and they would also get TB if their foods are dirty.

**I: Can you tell me the types of foods that make a child’s body unhealthy?**

R: Well when they get sick they would usually eat rice well they wouldn’t eat much because they’re sick like obnoxious.

**I: What are the types of foods that make a child’s body healthy?**

R: They would eat coconut meat (sprouted coconut), pandanus, like these types of foods that are fun to eat when they’re sick.

**I: Can you tell me any illnesses caused by the foods missing from the diet?**

R: There are foods that can cause the children to have illness. Like ramen they would just eat that and just that with nothing like meat or vegetables in them they would eat uncooked. This would cause them to have illnesses.

**I: Now we will talk about foods that are commonly available in your community. I would like you to explain how people in your community typically get food to eat on a daily basis.**

R: Yes. like in typical day a family would gather together and see what they would want to eat like coconut meat (sprouted coconut), breadfruit, and mix it together or they would want to eat breadfruit and see how they want to cook it. If coconut meat (sprouted coconut) they would make it with rice or soup or mix it with pumpkin. They would try and see what they can do to keep on mixing up their diet so that they can be lively and healthy.

**I: What foods are commonly grown at home?**

R: Pandanus, breadfruit, coconut, papaya, and taro.

**I: The foods that are grown at home in this community are eaten by the family or sold/traded?**

R: Yes.

**I: Can you explain the profits are commonly used if foods are sold?**

R: They would usually bring like if its coconut meat (sprouted coconut), breadfruit, pandanus and things like that. They would sell these kind of things and once they’ve sold them they would use their profits to buy imported foods like rice and also they would buy things for them to wash clothes with soap, they would bring things that they would need.

**I: Can you tell me about any difficulties to growing food in this community?**

R: Well one of the reasons why we have difficulties in growing food is that there are animals that would roam around and destroy the plants and the soil isn’t good to plant.

**I: Food availability varies across the year in some places. Could you explain how easy or difficult it is to get foods?**

R: Well they would have fun planting foods so that they would have foods like breadfruits, they like only show like they have their own seasons that they would become ripe. So when there’s like no breadfruits than we steer ourselves towards the pandanus. So it would be fun planting.

**I: Can you tell me what families would need to grow at home?**

R: Well when it comes to planting they would need shuffles, they would need machetes, and they would also need pigs so that they can make compose for planting. They would also need a fence for the garden, but we could also use compose with dead plants.

**I: Can you tell me any other food shortage throughout the year and their main causes?**

R: Sometime the community would run out of food and the reason why like for local foods they have their own seasons but when it comes to imported foods like rice, there are times that Majuro would run out of rice then we wouldn’t have rice.

**I: What do the community do to feed themselves if there is a shortage of food?**

R: The thing is when they have food shortage what they would do is they would eat local foods like fish, coconut, and these foods that can produce faster and easier every day that it would be easy to find when the community runs out food.

**I: Now I would like to ask you about animals that are raised in this community. Could you please tell me about animals that are commonly raised here?**

R: Mostly pigs, chickens, and dogs.

**I: Can you explain the reason why those animals are raised?**

R: Well the reason why they raise pig and chickens is because they would eat these animals but not just for eating but they would also sell them. For dogs the reason why they raise dogs is for them to help the master chase other animals like mostly pig and chicken. The dogs would also be the guard of the house so that no one wouldn’t try and do anything at the house.

**I: Is there any difficulties to raising animals?**

R: Well one of the difficulties to raising animals is that when they’re not in a fence then people would steal them, we wouldn’t know where they’ve gone to. Another one is when we feed them like we would mostly feed them coconut meat (spouted coconut and coconut meat) which we would run out and starve since these are the main thing to make money out of.

**I: Can you explain any difficulties to keeping animals in a fenced area?**

R: I think there’s no difficulties.

**I: None?**

R: Yes.

**I: Then can you tell me what do the community members typically do with animal feces?**

R: Well if they have a fence that has a safety tank, then they would just wash it but if they’re in a fence that has just the ground then they would shuffle them into the plants.

**I: What about the animals that aren’t in a fence?**

R: Well they would just go where ever they want and drop feces everywhere. That’s one of the reasons why pigs are difficult.

**I: For the last question on food, can you explain who decides what food to get for the family in most households?**

R: That would be the father the father would choose the food, as a man of the family he should always be the one to choose the food for his family since he’s the one who works to have food for the family.

**I: How do the families decide which foods to get?**

R: Well they would say okay bring this like they would try and buy something that could feed the whole family so that they can save more money, so that they can use the money to buy again if they don’t have the time to prepare the meal.

**I: Who decides which foods young children should eat?**

R: The mother and the father.

**I: So both of them. As in both of them would decides the foods for their young children and for the rest of the family the father decides.**

R: Oh I think they both would also decide what the family should so that the mother would know what to cook and how to cook it.

**I: Now we will talk about water and hygiene. Can you please describe a typical day getting and storing water for people in your community?**

R: They would store water in water catchment some would store water that’s for washing and stuff like that they store it in a cement water catchment.

**I: But is there a place for getting water?**

R: Well the houses that don’t have any water catchment or anything to store water in they would go to houses that has water catchments.

**I: So the community would share water?**

R: Yes they would share. They would go to other people’s house and get their water.

**I: Where do your community get their main source of water for drinking, for cooking, for washing, and for bathing?**

R: If it’s raining water we would use that but if we’re out of raining water then we would use the wale water.

**I: What are the main difficulties in getting water?**

R: That would be the lack of water catchment or anything that can store water because if we don’t have those then there wouldn’t be a place to get water.

**I: Okay, what about the main difficulties in storing water?**

R: Well there’s no difficulties in storing water but the thing is the lack of it.

**I: How are some ways that your community tries to make drinking water safe?**

R: Well if it’s the house and the house alone and they have their own drinking water they would clean it out as in when their water catchment is about to be empty they would clean it out and prepare it for its refill when it rains. They would clean it out with soap and things like that.

**I: Can you describe how would they clean it?**

R: The water catchment they would tilt it over and soap it and when they’re done cleaning it they would tilt it back up and let refill again. But for wale water they would take out the water and they would wash it in with chlorine.

**I: Let’s now discuss hand washing. Can you describe hand washing practice in your community?**

R: They would fill up a bucket and if not they would go to the lagoon and wash their hands but if they have dirty hands they would use soap and they would soap their hands and rinse it with a bucket of water or the lagoon as long as they use soap.

**I: Within your own thoughts what you think is the difference between using only water or water and soap to wash hands?**

R: Using just water is good if you want to rinse of the dirt and stuff like that but using water with soap is better because it kills bacteria’s and germs.

**I: What prevents families from washing hands with soap throughout the day?**

R: Well the thing that would prevent them from using soap is that when they’re always in a hurry they wouldn’t use soap and sometimes they wouldn’t have soap so they would just use water.

**I: Is there any other reasons?**

R: Well if they’re not using soap to wash their hands well then it must be because they’re in a hurry and there would be sometimes when there’s no more soap in the store.

**I: Now, could you describe the types of toilets in your community?**

R: The types of toilets in this community in some house they have bathrooms and some don’t have.

**I: As in they have toilet bowl?**

R: Yes some would have toilet bowls and some don’t have. Now with the houses that don’t have any you know they would use the bushes and the beaches.

**I: Do you know reasons why one type of toilet is used versus other types?**

R: It’s much safer, it helps prevent from getting sick. Also when there’s like sick people and they use it, it would be better to use it so that other people wouldn’t get sick.

**I: In some communities, we have heard that defecating in the open is common. Could you help us to understand this practice, including how common it is?**

R: People defecating in the open is still happening.

**I: Can you tell me some reasons why in some communities they would defecate in the open while in other places they don’t?**

R: Because in some places they have toilet bowls and some place as I said before they don’t have any toilet bowls, that’s the reason why they would defecate in the open.

**I: What are some barriers to using the toilet?**

R: The reason why some wouldn’t use the toilet is because some wouldn’t have any toilet paper and some wouldn’t have any water to flush the toilet.

**I: Can you tell me how young children’s stools are typically disposed of?**

R: Some would shuffle it and throw it at the lagoon.

**I: Could you now explain where young children in your community usually play each day?**

R: They would usually play around the school area.

**I: Does the children play in areas where animals are kept?**

R: There’s no animals around.

**I: Can you picture what an ideal play area for children looks like?**

R: Well the area that the children play in is the area in schools in which they made a place for children to play at. The reason why I said school area is because we made this area a safer place for children to play in.

**I: Can you tell me the challenges of keeping children’s play area clean?**

R: Well there’s not that much of cleaning tools and sometimes when schools is over the children would go home and come back to the school to play and they would throw around trashes.

**I: What challenges to keep the playing area clean?**

R: There’s not enough cleaning tools and things like that, like lawnmower and things like that.

**I: To wrap up our questions on sanitation, could you explain ways to prevent the spread of disease?**

R: Well to prevent the spread of disease the bathrooms should be clean, people should use soap very well so that people wouldn’t get easily sick.

**I: What do you think of the connection between exposure to feces and illness?**

R: Yes, because if the feces would be there for a long time and the flies would land on them and the flies would land on our foods then we eat from it and then we would get sick.

**I: Now we will talk about the responsibilities different family members play in raising children. Could you describe the care of children throughout the day in your community?**

R: Throughout the day how the community would look after the children from getting injured, they would teach them the things that they’re doing is bad so that they would know, and mostly they would clean them from time to time so that they won’t get sick.

**I: Who is mainly responsible for child care?**

R: Well that would be the mother.

**I: Since you said mother, what are the responsibilities of mother in child care?**

R: A mother’s responsibility is that all chores in the house should be done, the family in the house shouldn’t have any worries, for the children she should watch over them prepare them so that they would grow up the way she wants them to.

**I: What about the responsibilities of fathers in child care?**

R: Well a father’s responsibility should change his life and become a Christian. (Both laughs)

**I: Can you explain why?**

R: Because they get drunk and stuff like that, well they should change their life from do that because alcohol would bring problems to a family.

**I: Is there any other reasons?**

R: A responsible father should have a responsible wife, and his children should always be good to him and never give worries and things like that, so that he can do his best for his family.

**I: How would caregivers play with children under 2?**

R: Like they would walk around them and stay by their side so that they wouldn’t get into accidents.

**I: Is there any other reasons?**

R: Not just walking around but also carry them and giving them a cruise around.

**I: But do you know how they play with them?**

R: They would play with them but also they would watch over them so that they wouldn’t get into accidents.

**I: Could you talk about the role of grandparents have in raising children in this community?**

R: Grandparent are worthy because they would teach them our ways of custom, they would teach them how work with the family so that when they grow up they would know what to do.

**I: What are some ways that grandparents support in raising children, support mothers and families?**

R: They would like stay next to them and they would give some advice to the mother and father plus their grandchildren’s. They would also see what they can do for the family. They would like prepare them.

**I: What makes good grandparents?**

R: Good grandparents, well if there’s nothing that would show upon them that would worry them about their family and kind of like that well then they would live a life that they’re all bond together. They would like work things out together.

**I: Could you talk about the role that others have in raising children in this community?**

R: The role of others in this community they would watch over the children from getting hurt anything that could hurt them. They would teach them what’s wrong and what’s right for this community, and they would teach them how to get a long and love each other.

**I: What are ways that siblings help raise young children?**

R: Well the siblings that are older they should know how to watch over their young siblings like make sure that they know how to listen to one another and know how to love each other.

**I: Thank you for your answers, we are almost finished. Could you explain where members of your community usually get trusted information about nutrition and health?**

R: They would get trusted information about nutrition and health then they would have to take those information from their leaders like the once in the families that know about these information. Especially they should get these information from the doctors.

**I: Why are these sources trusted?**

R: Because some leaders in a community would tell his people some ideas and information about illness and how to keep the community to move forward.

**I: Can you tell me where nutrition and health messages should be delivered so that community members would see/hear them most easily?**

R: Well I think the messages should be delivered to our community leaders like our government should combine all community members and have a meeting about these information so that everyone could listen and share some ideas with each other to fix it. I think it should be delivered to our leaders.

**I: Other than the leaders, is there any other ways?**

R: Yes, pastors so that they can pass them out to each group of the church.

**I: What types of media that community members use the most to communicate?**

R: Well nowadays they usually use CV radio and cellphone, these are the things that they would use.

**I: Thank you very much for giving your time and having a conversation with me, we are all done here but is there anything else about the topics we talked about today that we missed or you would like to tell us about?**

R: Yes. about the program that you brought today, you said that you came on behalf of the hospital right?

**I: Yes.**

R: About what program?

**I: There is this program that was brought up by the UNICEF which stands for United Nation Child Fund. They came with a survey from last year and they looked at the report and they asked why children in the Marshall Islands aren’t growing to their full potential. So they send us so that we can find out the reason why so that they can create a program that can help the mothers and fathers and children’s so that it can help them have better life and help them how to keep their life healthy. They said that our children aren’t growing well and when they’re bodies aren’t growing well so does our brain and they called that stunted or stunting. And they’re trying to figure out why is it like this.**

R: Okay I understand now. That’s very good what you guys are doing for us. Thank you

**I: Thank you as well.**
