# Supplementary material for: Conceptualizing multi-level determinants of infant and young child nutrition in the Republic of Marshall Islands–a socio-ecological perspective
Source: PLOS Glob Public Health. 2022 Dec 19;2(12):e0001343. doi: 10.1371/journal.pgph.0001343 (PMC10022247; doi:10.1371/journal.pgph.0001343)
Supplement: S1 Data — (ZIP) [file pgph.0001343.s001.zip › RMI Supp Data/Interviews data/I52R_IDI_HW_Arno_Sep 14_Libon.docx]

**Interview Code: I52R**

**Interview type and interviewee: IDI HW**

**Interview Date: SEPT 14**

**Location: ARNO**

**Interviewer: Libon**

**Transcriber: Shante**

**I: Do you agree on taking parts on this survey?**

R: yes.

**I: thank you for giving us your time to speak with us. The information we will take from you, we will learn from them and help make our program good for our mothers and children’s and also the healthy of our islands. And to start, can you tell me with kind of job do you do in this community?**

R: my job in this community, I work with a group. We work together. Group for cleaning. Looking for coconuts. Work together. Move around with the girls.

**I: what’s your group called?**

R: well, the group doesn’t have any name. the group just started. We will give it a name. new group. (giggling)

**I: Now, the group belongs to who?**

R: KUMIT.

**I: Oh. ok. now, what do you usually do from the morning until evening as a worker for KUMIT or as your group?**

R: I usually, here at my house, I wake up a wash dishes and cook, pick up trash and then when finish I will go with my groups and work. Look for coconuts and pick up trash.

**I: Ok. so, what kind of works do you guys usually do?**

R: pick up trash and look for coconuts.

**I: pick up trash and look for coconuts. Ok. let’s not talk about illnesses. I am interested in illnesses that children usually suffer from. in your community, what illnesses would you say children under 2 years commonly suffer from**?

R: fever, diarrhea and coughing. And when they get these illnesses we bring them to the doctors. And when the doctors are not there, we bring them to the traditional healers so that they can make them traditional medicines.

**I: ok. now, what makes them to get sick?**

R: they fall. And sometimes the mother doesn’t make their food right, like the foods can have flies in them, and she doesn’t wash her hands before she feeds the children’s. and that’s how they have diarrhea.

**I: Oh. ok. what is the seriousness about these illnesses?**

R: the seriousness of these illnesses, sometimes they can get seizer and will die, also from fever. They also die from diarrhea. And sometimes they will turn handicap and it would make them not know how to talk. Comes from when their fever is high.

**I: Oh. ok. now, how would you prevent these illnesses?**

R: we go to the doctors so that they can check them. And they give us medicines. and when the doctor is gone, we bring them to the traditional healer so that they can help.

**I: Ok. now, how do these people treat these illnesses in this community?**

R: on how they treat illnesses in this community, they go to the doctors so that they can give them medicines to prevent their illnesses.

**I: Ok. if they are not cured with the medicines that the doctors give them, is there other ways to prevent their illnesses?**

R: yes. we come back for traditional medicines so that they can help them.

**I: Oh. ok. now, can you explain what type of treatment people in your community seek for their children, for example traditional healers, doctors, nurses**?

R: first they usually go to the doctors first. And when the doctor is gone, then they will go to the traditional healer.

**I: Oh. ok. good. who do they go to first and why?**

R: first they go see the doctors so that they can help their children.

**I: why do they bring them to the doctors?**

R: because they are sick. They have fever, diarrhea, coughing, asthma and all the other illnesses that kids usually have. the illnesses they usually get is diarrhea, asthma and fever. That’s what children usually gets.

I**: Now, why do they bring them to the doctors?**

R: so that they can give them medicines to prevent the illnesses.

**I: Ok. do they use the traditional healers or just local medicines?**

R: they use both of them.

**I: Ok. how do they use all of those?**

R: they… the tradition healer goes and see, or the doctor comes and see if the child is very sick and will give them medicines to drink.

**I: ok. can you tell me what challenges your community faces in seeking treatment for the illnesses you mentioned before**?

R: sometimes there is no more medicines. and now we will use our local medicines because there are no medicines in the hospital.

**I: Ok. what difficulties do you face when you provide help to your patients? For example, if they bring a child so that you can check if he is sick, what prevents you from checking your patients?**

R: well, when they bring them, for me, if they bring a women’s, you know the issues with women, they tell the person, ‘’I’m not going to be able to check the person because I have a wutamwe. (like someone having issues). The struggles of a women.

**I: Oh. that is exactly what we are looking for in your answers. Thank you. can you describe any illnesses associated with nutrition that affects the children in your community?**

R: nothing.

**I: why do you say nothing?**

R: because it’s good. the foods that they eat.

**I: because what?**

R: it has nutrition in them. The foods that they eat.

**I: Ok. let’s say, when they have nutrition in them, they don’t get sick?**

R: they don’t get sick because they have lots of nutrition in them.

**I: when there is lots of nutritious in them, what will happen?**

R: they can grow healthier, they’re chubby, have a healthy life, nothing on their skins that we see.

**I: yes. now, what kind of foods can you say that won’t make a child’s health healthy? And why?**

R: number one, when they eat salty foods. that also affect them. The sweets that they usually eats also affect them. And they have no nutrition for them. Those are the foods we don’t want them to eat, salt and sweets, because they can’t grow with those kinds of foods.

**I: Oh, because they cannot grow because of that?**

R: yes.

**I: do they usually get sick?**

R: yes. yes, they get sick because they eat too much sweets and salt.

**I: what kinds of foods that will make a child’s body healthy?**

R: Pandanus, breadfruit, U and banana. Foods we usually give them are local foods.

**I: why are those foods good?**

R: because there’s no bad thing about those foods. they have lots of vitamin in them. When they eat them, they will grow faster, they’re body is good, they are chubby and they are not weak.

**I: Ok. so, we talked about unhealthy life, could you describe for me a typical day of someone living a healthy lifestyle, from the time they wake up in the morning until when they go to bed?**

R: when we look at them, they look so good and healthy because the foods they eat has lots of nutrition in them, when you look at them they look healthy and not weak. They look clean. When we look at them we see and know that they are healthy, and he doesn’t feel weak.

R: yeah. they are not lazy. I: Oh. so, they are not weak and lazy?

**I: Ok. I have few more questions but related to women’s health. Could you tell me about your experiences with women who have anaemia?**

R: One, the reason why women has anaemia is because they eat too much kool aid. They mix it with soy sauce and ramen and eat it uncooked. It will affect their body and it’s not good for their body but that’s their choice because they want to eat it.

**I: Ok. what about their iron sulfate medicines? do they drink it?**

R: they don’t care about them because they don’t like them.

**I: Oh. so, you mean when they have morning sickness they don’t want to drink the medicines?**

R: yes.

**I: are there any women that knows that anaemia is not good for them?**

R: yeah. they know about it but they just want to eat the foods that they want to eat. they think about them having anaemia, but they just want to eat what they want. They don’t want to stop doing it.

**I: what makes young girls have anaemia and pregnant girls?**

R: I already told you. because they eat too much of the bad food for their bodies.

**I: is there any word of advice given to women for preventing ad treatment of anaemia?**

R: yes. we give them word of advice to them not to eat those kinds of foods. they don’t want to listen to the advice we give them. We tell them not to eat them because it’s not good for them and their health.

**I: we will now talk about breastfeeding. Can you talk about how long after birth most women start breastfeeding in your community?**

R: yes. the ones that are breastfeeding needs to breastfeed their child once they are, until they reach 1 or 2. But now, we see now a days that they use milk more other for their children.

**I: Oh. ok. you know when the time they are not pregnant, when they just gave birth, did they needed to breastfeed the child right away or do they need to wait a little?**

R: they need to breastfeed once they give birth.

**I: that time?**

R: yes.

**I: now why do they need to breastfeed right away?**

R: it’s important for them to give breastmilk, it’s like to prevent the illnesses from them. They give them to breastfeed.

**I: Ok. from your own self, when your daughter gave birth, did you have any idea about the first liquid in the mothers breast?**

R: yes. when they give birth, and the first liquid, it’s like a dren waan. (not useful liquid) it’s not like a milk. But the child will kept on breastfeeding and soon the real nutrition will show.

**I: from yourself, is the liquid important for the child to eat it?**

R: it’s important because the doctor says it’s important. They said they should give it to the child so that the real nutrition can show.

**I: Oh. ok. so, the doctor said the liquid is important at first.**

R: they should breastfeed them so that they’re real food can show.

**I: Oh. ok. good. now, is there any other liquids they gave the child after they were born?**

R: no. after one week ahead, you should give milk and water. But at first, you only give them your breastmilk.

**I: Oh. ok. why didn’t they give other liquids other then the breastmilk?**

R: on how we know, the breastmilk is the first one we give them.

**I: Ok. Can you tell me how the women in this community breastfeed only?**

R: the reason why it’s important for them to breastfeed only it’s because the child so grow good and healthy, they won’t get hurt, they won’t get diarrhea, all the kinds illnesses that children usually gets, if they eat too much with their mother, they will be healthy because their food is safe.

**I: oh. ok. Now, is there any other liquid given to the child other than breastmilk in the first 6 months?**

R: yes. first you give them water. Other then the breastmilk you give them water. But now a days they often give them milk with water, they give them milk and water.

**I: now, why do they give them water?**

R: because they say that it will make the child bodies healthy. So that their body wouldn’t be dry.

**I: what are the difficulties faced by mothers in your community on breastfeeding for six months? are there any difficulties?**

R: I don’t think they have any difficulties on how they breastfeed.

**I: Ok. Now, why do you say there are no difficulties?**

R: because the breast, you know that that’s their first food that has been ready for them and they won’t get affected from it. It has no cost and they don’t spend money on it.

**I: Ok. are there any words of advice for the mothers to breastfeed exclusively for 6 months only?**

R: yes. you need to give them word of advice so that they won’t stop breastfeeding them, make them breastfeed their child only because it’s very important and it doesn’t cost anything.

**I: Ok. we are trying to understand how people eat in this community. could you describe in detail what most families usually eat and drink throughout the day? foods that they usually…**

R: well, they usually eat, they usually eat bread and rice. those kinds Imported foods.

**I: imported foods? what about meat?**

R: they usually eat imported meats. Can foods and those kinds. they barely have fish.

**I: how do they prepare their foods?**

R: sometimes they don’t mix their foods. they eat them right away.

**I: Ok. Now, is there any difference on how meals are being prepared for each family? sorry. Who in the family needs to have food first? And who comes after?**

R: first, when you make food you need to make food for your family, mother and father, you prepare for them first and after that you will now turn to your kids…

**I: Good. is there any difference on sharing your foods with each your families? If you were to share foods to your house members or your neighbors and the other households share their foods to some other households, is there any difference between sharing your foods?**

R: yes. sometimes we see the difference.

**I: Ok. what are the differences?**

R: when we go and see, it’s like they don’t let their families know and feed someone, but some eat alone. There is a difference. It’s difference from what we do to our families.

**I: oh. like it’s a different fire. (meaning like each family members have their own foods to eat)**

R: yes. it’s different.

**I: Ok. is there any difference on the amount of foods they serve to each family?**

R: yes. there is a difference on how they give food to the family.

**I: how is it difference?**

R: when I look at them make the foods, I see the difference.

**I: hm. So, like some can have a big amount of food and some don’t.**

R: some and some.

**I: ok**

R: Some has big amount, some has small amount.

**I: Ok. are there some kids that have a big amount of food from the other kids?**

R: yes. we see that too. They do that. Some kids have big amount of foods sometimes.

**I: why do they do that?**

R: it’s like for us, our culture, there are some that are high and some are low.

**I: Oh. so that’s the reason why some has big amount of food from the rest.?**

R: yes. when you have a special child, you will give them a big amount of foods. they will get big amount of foods from the other ones. You know when you’re a parent and a grandparent you will always have a favorite child, and that’s when the child gets different amount of foods then the other ones.

**I: Oh. ok. Now, can you tell me how the family shares their foods with family members during meal times? For example, children eating together separately from the family, meals eaten from the same plate by all family members?**

R: we separate. Everyone don’t get together. We do separate the kids foods from, they don’t use the same plate.

**I: Ok. Now I want to know about how young children eat in this community, can you describe in detail what children under 2 years commonly eat throughout the day?**

R: when I look in this community, the usually eat rice.

**I: kids on the age of 2 years and below.**

R: the 2 years old they make them rice. They often eat rice.

**I: does the rice have meat. Or just the rice?**

R: sometimes there’s no rice, sometimes there is.

**I: so, when there is no more, is there anyone, they eat the rice only or do they eat rice with soy sauce or rice beto (rice mix with any kind liquids).**

R: yeah. sometimes with rice with soy cause, sometimes rice beto and sometimes they don’t. that’s for kids under the age of 2.

**I: Ok. now how many times in a day, children under the age of 2 are eating?**

R: sometimes when the mother is busy, they can eat 1 or 2 times. 3, when the mother is very, not busy. Well, sometimes they eat healthy on the right time. Sometimes when she’s very busy they can eat only 2 times a day.

**I: Hm… Ok. do children usually eat snacks between hours?**

R: barely. They barely eat snacks between hours.

**I: why?**

R: there are nothing.

**I: Now, are there any different on feeding a child when they are sick? Or when they have diarrhea. is there any difference on feeding them?**

R: yes. when they are going to feed them their food they wouldn’t want to eat them. They won’t ever find what’s good for them.

**I: is it important for them to eat when they have diarrhea?**

R: sometimes the doctors say, ‘’don’t feed them first because they poop a lot so that it can stop. But their mother will just feed them. But it’s important to stop because the doctors told us to stop, don’t feed them yet so that it can stop, so that the germ can be gone.

**I: Oh. ok. Now is there any difference on how they feed the girls than the boys under the age of 2?**

R: I don’t think there is a difference.

**I: everyone is the same?**

R: hm.

**I: Now, can you talk to me about what influences how families feed their children in this community?**

R: because the reason why we feed them it’s so that they can grow faster and won’t get illnesses.

**I: we heard from some families that they eat local foods and others that eat processed foods. could you explain what is typical for most families in this community?**

R: we usually see families in this community eat imported foods. 1 or 2 families eat local foods.

**I: Oh. ok. now, what makes it difficult and easy to cook local foods? why is it sometimes difficult to cook local foods?**

R: the first one, you know about our life’s these days. It’s like we are lazy to make food. lazy to cook. They only want to use imported foods because it’s faster, just take it and open it and eat it.

**I: hm…**

R: but our foods, before we eat them we cook them. If it was U. you will poke it out. For breadfruit you would take the breadfruit, it’s a lot of work for our local foods. and that’s why some people don’t want to make local foods.

**I: what makes it easy to cook local foods? why is it easy for you to cook local foods?...**

R: it’s easy for us because we have local foods. and we make them good.

**I: so, you say you can…**

R: I know how to make them…

**I: cook them**

R: cook them.

**I: Ok. what are the goods and bad of local foods?**

R: there are no bad things about the local foods. the local foods are good because it gives us strength. We are healthy, and we don’t get sick.

**I: what are the goods and bad of imported foods?**

R: the imported foods give us lots of different kinds of illnesses.

**I: on how you say lots of difference kinds of illnesses, what do you mean by lots of difference illnesses?**

R: diabetes and high blood. These are the things that affect us.

**I: now, are there any thoughts about nutritious foods to add them with local foods for children under the age of 2?**

R: the foods that are good for us to give the children under that age, 2 years below. It should be pandanus and banana. Those are the ones that are good for them when they eat in those years.

**I: Ok. can you talk about the information about breastfeeding and the other foods you give to mothers and other people in this community?**

R: the importance of breastfeeding, the first one, your child should grow well. And they can be healthy. That’s the importance of breastfeeding.

**I: Ok.**

R: they won’t get sick, you won’t see your child get sick If you only feed him.

**I: Ok. is there any lesson about nutritious food for people in this community? based on your work in this community. is there any lesson you tell the people in this community about foods that has nutrition in them?**

R: yes. we usually take part with the people and let them make the foods and plant bananas, pandanus and other kinds so that they can grow. There are lots of nutrition in local foods.

**I: Ok. Now, how would you give it to the children? Is it important for mothers and fathers to give these foods to the kids?**

R: yes. we tell them to plant some plants so that they can have plants to eat from.

**I: are there any difficulties on teaching the people in this community about nutritious foods?**

R: like how we say it, they have some difficulties. There are some things they say.

**I: like what?**

R: like if they say, go do that and the other person would say, oh we don’t have this for that. There are many kinds of difficulties when it comes to planting. They don’t have fences. Lots of things they say. It’s like they are not happy.

**I: How would you explain and give them the perfect information about nutritious food? how would it be good and perfect?**

R: it’s like we say, it’s good if we are together to find ways to plant our foods so that our foods would be perfect.

**I: OH. ok. now, I would like to talk about women that are pregnant in this community. can you describe what kind of foods do women eat during their pregnancy?**

R: you know all pregnant women loves all kinds of foods. sea foods, some wants to eat clam. Some wants to eat kukor (who ever is checking this, please define kukor for me. Thanks.) some wants to eat, I’m talking about sea foods. when it comes to island foods, some eat U. some wants to eat breadfruits. Some wants to eat pandanus. It’s different what pregnant usually wants to eat.

**I: Ok. now, women’s that are pregnant, are their foods different during the time they are pregnant?**

R: yes. their foods are different because they choose they’re own foods.

**I: what really makes their foods different from when they are pregnant?**

R: because they have morning sicknesses and they don’t want to eat the good foods, they just want to eat foods that are, they only get what they want to eat because they are pregnant.

**I: because of the child?**

R: because if the child.

**I: ok. what kind of foods do they encourage pregnant women to eat? why do they want them to eat those foods?**

R: the foods that the old ladies always encourage them to eat is, if for like fish and other things, they would give it to them to make them have more blood. Make them healthy. Local foods. they usually encourage them to eat them and not the foods that are strong for them. (strong as in bad).

**I: Oh. ok. what kind of foods do they encourage pregnant women’s not to eat?**

R: not to eat salty foods. foods that aren’t good for pregnant wonmen.

**I: what else? Salt and what else?**

R: greasy foods. they should not eat grease. Kool aid and ramen. These are also what the old ladies encourage them not to eat.

**I: who encourage or discourage the women from eating those foods while breastfeeding?**

R: her mother and her doctor. Every time she goes to her doctor, the doctor tells her these words. Don’t eat this one. The things that the doctor gives her.

**I: is their medicines for nutrition women usually takes during thee time they were pregnant?**

R: yes. they usually give them medicines for anaemia and vitamin.

**I: what prevents the women from taking their supplements?**

R: sometimes they can’t take them because they feel dizzy and they won’t drink them.

**I: is there any time the women smoke and drink alcohols during the time they are pregnant?**

R: yes. some women’s do, and some women do.

**I: why do some women do them, and some don’t?**

R: you know how we live, we live like what…? You know.

**I: like anemkwoj (when you don’t care, and just want to do whatever you want to do.)**

R: yeah. rianemkwoj.

**I: Ok. can you describe what kind of foods the women eat during their breastfeeding?**

R: the foods that the ones that breastfeed, their mothers tell them to eat fish, coconut so that they can make breastmilk so that the child can eat. more for the child to eat.

**I: fish and coconut?**

R: yes.

**I: do the women change what they eat during breastfeeding?**

R: yes. they change what they eat. if her mother tells her to eat this, she will. Their foods is always different.

**I: what kind food of food do they encourage the women to eat during their breastfeeding? And why?**

R: that what they usually tell them, to eat lots of fish so that they can have a strong body plus the child will have lots to eat.

**I: what kind of foods did they encourage women not to eat during the time of their breastfeeding? And why?**

R: well, the foods that are salty and, like what… those foods that aren’t good for the women to eat.

**I: salty foods?**

R: yes.

**I: what about foods that has grease?**

R: and grease.

**I: Ok. who encourages or discourages them not to eat those foods while breastfeeding?**

R: their doctors. They usually encourage them not to eat those kinds of foods.

**I: who else?**

R: especially their mothers and grandmothers.

**I: Ok. as a worker, what are some of your biggest concerns of the diets of pregnant and breastfeeding women in the communities you work in?**

R: the foods they are sad about are these kinds of foods that they eat. like salt and, we are very sad with them because they eat them.

**I: Oh. ok. good. on our las section, we want to know about ways we can develop health programs in your community. could you explain where community members usually get trusted information about nutrition and health?**

R: we usually get them from the hospital, they give It to us, about foods and other things and we take it back to our islands. The hospital gives it to us.

**I: ok. why do you trust the information from where they come from?**

R: because that’s the place to give us health and information for us to take.

**I: OK. where wold you have wanted the information to go for you to easily see and hear about it everyday**?

R: we usually hear it from the radio. We take the information from the radio and learn from it.

**I: Ok. where should they also put it for you to easily see and easily hear them?**

R: the schools and the church.

**I: Now, for our last question, could you describe what influences how people raise children in this community?**

R: so that they won’t get hurt. We protect them so that they won’t get hurt. Get sick and… so that they won’t touch the ground and where is dirty.

**I: Ok. is there any words of advice about raising a child they usually give to people in this community?**

R: yes. they do. They usually say, you guys should protect your children so that they won’t eat what’s dirty and won’t get sick. You guys should protect your children’s.

**I: are there any information pregnant women’s and mothers ask for from the ministry of health?**

R: they usually tell them to go to the doctors.

**I: Now, when they go to the doctors, do they ask questions to the doctors or the nurses?**

R: I don’t think they give them any questions when they go see the doctors. They don’t ask about health and things like that.

**I: they only go for their appointments?**

R: appointments and finished.

**I: are the anyways for you to teach the caregivers about health?**

R: yes. we need to tell the caregivers to take good care and protect the children so that they won’t touch where is dirty, so that they won’t eat dirt’s… things like that. Protect them.

**I: OK. well, is there anything you want to talk about that we missed? Is there anything you want to say or add in?**

R: well, the only word is, you should really be careful and protect your children’s so that they won’t get sick.

**I: Oh. ok. thank you. we are done. Once again, thank you for this beautiful time and the information you gave, I believe that those are some information that will help better our programs for women, mothers and especially our children’s. and thank you.**

R: thank you also for bringing these questions so that we can answer. Even though they are wrong… laughing.

**I: good.**
