# Supplementary material for: Conceptualizing multi-level determinants of infant and young child nutrition in the Republic of Marshall Islands–a socio-ecological perspective
Source: PLOS Glob Public Health. 2022 Dec 19;2(12):e0001343. doi: 10.1371/journal.pgph.0001343 (PMC10022247; doi:10.1371/journal.pgph.0001343)
Supplement: S1 Data — (ZIP) [file pgph.0001343.s001.zip › RMI Supp Data/Interviews data/I53R_IDI_FCG_Arno_Sep 14_Libon.docx]

- Interview code: I53R
- Interview type and interviewee: IDI_FCG
- Interview Date: Sept. 14. 2018
- Location: Arno
- Interviewer: Libon
- Transcriber: Marcellina

**I: okay are you willing to participate in this survey?**

R: yes

**I: okay good. We will now proceed. And thank you for giving us your time to speak with us today. The information we learn here will help us find ways to improve maternal and child health and sanitation in your country. To begin with, can you please tell me a little about your family/household?**

R: well my name is sako, my husband name is Ray, and our daughter names are jonita and rakko. My older daughter is 3 years old and the other one is 1. My mother name is jowaan and my father name is loko.

**I: now who is living in your household?**

R: me, my husband, our children, and the world Teach teacher. But in the other house is my parents and my little sister.

**I: now can you describe your community?**

R: like what?

**I: anything about this community?**

R: well this community is dirty, and it has lots of bushes

**I: and what are the good things about this community?**

R: the good thing is it has local foods which are cost free.

**I: and what are the bad things about this community?**

R: it is dirty and sometimes there are drunken guys

**I: are the people in this community good or bad?**

R: some are good, and some are bad. One thing they are bad about is, they are not good at welcoming visitors.

**I: Let’s now talk about health and illnesses in your family. Can you tell me about some of the illnesses that your children have suffered from?**

R: fever, diarrhea, coughing, headache, nausea, and skin rash (RAJIIA)

**I: what are the causes of fever?**

R: when it’s really windy, swimming in the lagoon, and touching dirt and eat without washing hands.

**I: and what are the seriousness of fever?**

R: seizer and then die

**I: now how can you prevent fever?**

R: l let them drink Tylenol and treat the fever by applying wet fabric all over the body.

**I: now what about coughing, what are the causes of coughing?**

R: also touching dirt and eat w/out washing hands and walking when it’s windy

**I: oh okay. Now is there seriousness for coughing?**

R: yes, there are

**I: can you give examples?**

R: having pain in the body, head, and throat

**I: and how can you prevent it?**

R: by let them drink medicines

**I: are there any local medicines for coughing?**

R: none

**I: Now can you describe how do you know when your child needs treatment for their illness?**

R: when I see at they are sick, I take them to the doctor

**I: the moment you see that they are sick, you suddenly take them to the doctor, right?**

R: yes, at that very moment I found out, I take them to the doctor, so he can give medicines

**I: now who do you first go to when your child is sick?**

R: the doctor

**I: why do you seek the doctor first?**

R: so, he can do check up on them and give medicines

**I: and do you use traditional medicines when your children are sick?**

R: no, I don’t

**I: Can you describe any illnesses affecting your children that are associated with nutrition?**

R: there are none. They grow well and healthy with the nutrition foods. And they don’t get sick frequently.

**I: good. Now can you describe any illnesses caused by foods missing from the diet?**

R: rajjiia (skin rash), wot (boil), and malnutrition.

**I: We talked a lot about being unhealthy. Could you now describe for me a typical day of someone living a healthy lifestyle, from the time they wake up in the morning until when they go to bed?**

R: they work a lot, roaming around a lot, don’t get sick frequently, energetic, and healthy

**I: and now, what are the appearances or signs of a healthy child under 2 years?**

R: they walk around, they eat local foods, and they don’t get sick frequently

**I: and what are the appearances or signs of a healthy adult?**

R: they don’t just sit around but they work a lot

**I: Let’s now discuss hand washing. Could you describe in detail your family’s hand washing throughout the day?**

R: we wash our hands with soap to get rid of the germs

**I: do the children wash their hands throughout the day?**

R: yes

**I: how do you wash their hands?**

R: I apply soap on their hands and rinse them with water

**I: and what about the children under 2. Do they also wash their hands throughout the day?**

R: yes, they also wash their hands

**I: now how do you wash your youngest daughter’s hands?**

R: I take her to the dish washer and wash her hands

**I: okay. what do you think is the difference between using water only or water and soap to wash hands?**

R: when we wash our hands with water only, the germs are still in our hands which cause diarrhea. And when we wash our hands using water and soap, it feels good and it kills the germs.

**I: now can you tell me when do your family use soap to wash hands throughout the day?**

R: when we are going to eat, after using the toilet, and after touching dirt.

**I: what prevent you from washing your hands with soap throughout the day? Or why can’t you wash your hands with soap throughout the day?**

R: sometimes we just forgot to, because it’s not habitual

**I: Now we would like to talk about your diet during pregnancy and breastfeeding. Now I would like you to think back to when you were pregnant. Can you describe your diet when you were pregnant compared to when you were not pregnant?**

R: when I was pregnant, I usually ate breadfruits, fish, pineapple, drink coconut or soda, and ate sweets like chocolate.

**I: and what about when you are not pregnant?**

R: I eat everything

**I: what foods they encouraged to eat during your pregnancy?**

R: fish and breadfruits

**I: and why did they encourage you to eat fish and breadfruits?**

R: so that my baby can grow well and healthy

**I: what foods they discourage you to eat during pregnancy?**

R: salty foods like salt fish.

**I: okay. Now who encouraged or discouraged to eat those foods during pregnancy?**

R: my parents

**I: who helped or supported you during pregnancy?**

R: my mother and my husband

**I: how did they help or support you during pregnancy?**

R: they helped me taking care of my children when I am busy, and they bring me foods.

**I: okay good. Now can you tell me about any supplements you took during pregnancy?**

R: I took the vitamins and the iron-folic acid

**I: and did you take all the supplements given to you?**

R: yes, I did

**I: were there anything made it difficult for you to take your supplements?**

R: as for the iron-folic acid, sometimes I don’t take it because it makes me feel nausea

**I: and why did they encourage you take the supplements?**

R: for my baby to be healthy

**I: now did you drink alcohol, smoke cigarette, or took any drugs during your pregnancy?**

R: I didn’t

**I: and why didn’t you?**

R: because it’ll cause harm to my baby

**I: now were there any traditional medicines you took during pregnancy?**

R: yes. I drink the traditional medicine called medicines for suffering (it’s a traditional medicine for helping pregnant women have little pain or slightly suffer at delivery)

**I: who made the traditional medicines for you?**

R: my grandmother

**I: how did she made it? Like do you know what kind of herbal she used?**

R: KIDREN (local tree), and KONNAT (local tree)

**I: and how did she make it?**

R: she bounds the leaves together, so it could be easy to squeeze the leave’s liquid and then mixed them with water.

**I: If you were advised to eat more fruits and vegetables during pregnancy, could you describe what would make this difficult? Like were there any difficulties for you to eat fruits and vegetable?**

R: there were none

**I: what about, what made it easy for you to eat fruits and vegetables? Like why it wasn’t difficult for you to eat fruits and vegetables?**

R: because they are good and healthy for me and my baby

**I: Now can you describe your diet when you were breastfeeding?**

R: I usually eat fish, mackerel, and those foods that can help producing breastmilk

**I: what really made you want to eat these foods during breastfeeding?**

R: so that I can have enough breastmilk

**I: now what foods they encouraged you to eat during breastfeeding?**

R: foods that help in producing breastmilk like fish, and canned foods like mackerel and corned beef

**I: what foods they encouraged you not to eat during breastfeeding?**

R: foods that are salty like salt fish and other salty foods

**I: now who encouraged you to eat or not eat these foods during breastfeeding?**

R: my parent

**I: now after giving birth, can you describe how you breastfeed your baby throughout the day?**

R: after I gave birth, they clean my baby and then bring her to me and I breastfeed her

**I: did you give bottle milk or other liquid other than breastmilk to your baby after giving birth?**

R: I never give bottle milk or any other liquid, I only breastfeed my baby

**I: now what made it easy or difficult for you to feed your baby up until now she’s 1 year old?**

R: nothing. Everything was okay

**I: and are there anything that can make it easy or difficult for you to breastfeed your baby until she’ll be 2 years old?**

R: nothing

**I: now why there are no difficulties for you to still breastfeeding?**

R: because it cost free and it makes our babies grow healthy

**I: now can you tell me when did you start giving foods or liquids to your baby other than breastmilk?**

R: when she was 5 months old

**I: and why did start at that age?**

R: because I wanted her to grow faster

**I: now what are the opinions from others that influenced their decision to introduce foods and liquids at that age?**

R: it’s what they think it’s right, but I don’t know why they think that way.

**I: what were the first foods you gave to your baby and how did you prepare them?**

R: biscuits and breadfruit

**I: as for the breadfruit, how do you prepare it?**

R: I boil it and then later add coconut milk and sugar.

**I: and how do you call that kind of cooking?**

R: JOKKOP and BWILITUDROK

**I: and what about the biscuit? How do you prepare it?**

R: I cracked them up into pieces and then let them soak in water or milk.

**I: We are trying to understand how people eat in this community. Could you describe in detail what your family usually eats and drinks throughout the day?**

R: we eat rice, fish, canned meats, and drink water

**I: can you describe how do your family prepare the foods? Like the fish for example?**

R: we fry them, boil them, and ground grill them.

**I: and what about the canned foods?**

R: we make gravy with the canned foods

**I: now who in the family is served first, next, and last?**

R: the children always serve first. Next will be our parents, and last will be me, my husband, and the boy

**I: are there differences in the foods you served to different family members?**

R: none

**I: none? Everyone always have the same foods?**

R: yes

**I: and are there differences in the amount of foods you serve to different family members?**

R: there are none. Everyone receives the same amount of foods

**I: are there some children receive more amount of foods than others?**

R: none. My children always receive the same amount of foods

**I: Now could you describe any food sharing between family members during mealtimes? for example children eating together separately from the family, meals eaten from the same plate by all family members?**

R: everyone eats separately from each other; children eat by themselves and so as the adults

**I: now does your family share foods to the neighbors?**

R: yes, most of the times we share foods to our neighbors

**I: We have heard from some families that eat local foods whereas others eat processed foods. Could you explain what is typical for your family?**

R: hot-dog, chicken, fish, crabs, and lobsters

**I: now what would make it difficult for you to cook local foods?**

R: there are no difficulties in cooking local foods because the fire woods cost free

**I: what are the good or the bad things about local foods?**

R: they are good because taste good, make us grow healthy and make us barely have sicknesses

**I: and what are the good or the bad things about processed foods?**

R: they are bad because they cause illnesses like diabetes and high blood pressure

**I: Now that we’ve talked about how the family eats, I would like to learn more about how your child eats. Could you describe in detail what your son/daughter under 2 years commonly eats throughout the day?**

R: as for my younger daughter, she usually eats bread, rice, chips, crabs, lobsters, and fish

**I: okay. Could you now tell me what you think are important foods for children under 2 years to grow well and be healthy?**

R: fruits like bananas, breadfruits, papayas

**I: now what is the biggest influence on feeding your children? Or why is it important for you to feed your children?**

R: so that they can grow well, be healthy, and barely get sicknesses

**I: if you had son and a daughter under 2 years old, would you have feed them differently?**

R: no. I would do the same to each of them

**I: Now we are also interested in the roles and responsibilities different family members play in raising children. Could you describe the care of children throughout the day in your community?**

R: as for me, I watch out my children from harms; I make sure they don’t go near the well, play near any deep holes, or touch unclean stuff and eats without washing their hands.

**I: now who is mainly responsible in taking care of your child?**

R: me

**I: why is it always have to you or the mothers?**

R: because they are our babies

**I: instead of saying they are your babies; how do you take care of them?**

R: I take care of them

**I: okay. Now what are the responsibilities of the mothers in taking care of their children?**

R: they take care of them from any harms and take care of them, so they won’t have illnesses

**I: and what about the fathers? What are their responsibilities as a father to their children?**

R: they make sure their children don’t starve by providing foods and water.

**I: now how do caregivers play with children under 2 years old?**

R: they sing with them and chat with them

**I: now could you talk about the roles of grandparents in raising children in this community?**

R: they also take care of the children and make sure they don’t get sick and make sure their hands are clean before eating.

**I: how does the grandparents support in raising children and support mothers and fathers?**

R: they support with needs like diapers and foods for the children

**I: and now what does make a grandparent be a good grandparent?**

R: the way they help us taking care of the children when we’re busy, and feeding them

**I: okay. now could you talk about the role that other family members have in raising children in this community?**

R: they also take care of them, feed them, sing to them, and put them to sleep

**I: and how does the older sibling take care of their younger siblings?**

R: they carry them, feed them, and make sure they don’t touch any dirt

**I: well you are doing a great job. We are almost finished. Now for the last section, we would like to learn about ways we can develop health programs in your community. Could you explain where you usually get trusted information about nutrition and health?**

R: from my mother and from hospital

**I: now as you mentioned hospital, why do you trust the information you get from hospital?**

R: because it is where we always go to when our children are sick

**I: what types of media you use the most to communicate? Like for example the radio, online apps, websites?**

R: the radio

**I: When you think about your own parenting behaviors, can explain the differences on your parenting skills, are there any differences on how you parenting from other mothers?**

R: I don’t there are because I don’t know how other mothers take care of their children

**I: now opinions of other people in this community on how you parenting, how do they think about your parenting skills? What do they usually tell you?**

R: they usually tell me not to walk around with my children when it’s raining and windy; they tell me let them stay inside the house and give them medicines

**I: are there any advices or information you have receive about parenting?**

R: they gave me advice on the foods I should not give to them like the salty foods. and to make sure they don’t touch any dirt.

**I: okay. Is there any desired information on parenting you wish to have but doesn’t available?**

R: none

**I: Is there anything else about the topics we talked about today that we missed or that you would like to tell us about?**

R: none

**I: okay that was great, we are done now. Thank you once again for your generous time and for sharing your thoughts with us. We greatly appreciate your help and we hope this research will help us improve the health of mothers and children in your community.**
