# Supplementary material for: Conceptualizing multi-level determinants of infant and young child nutrition in the Republic of Marshall Islands–a socio-ecological perspective
Source: PLOS Glob Public Health. 2022 Dec 19;2(12):e0001343. doi: 10.1371/journal.pgph.0001343 (PMC10022247; doi:10.1371/journal.pgph.0001343)
Supplement: S1 Data — (ZIP) [file pgph.0001343.s001.zip › RMI Supp Data/Interviews data/I54R_IDI_HW_Arno_Sep 15_Fela.docx]

Interview Code: I54

Interview Type: Traditional Healer

Interview Date: September 15 2018

Location: Arno

Interviewer: Fela

Transcriber: Fela

**I: Okay before we proceed, do you agree to take your part in this survey?**

R: Yes

**I: Thank you for giving your time to speak with me today. The information we learn here will help us find ways to improve maternal and child health and sanitation in your community. To begin with, can you please tell me about your job as a traditional healer, can you explain what do you do around the house or to the children in your community?**

R: here we go, let’s just say around my house because I don’t go out and do my local medicine I only do it at my house, like for my grandchildren or people who need my help can come to me to my house. I do local drinking medicine for children that fell from higher places, or stomach massaging.

**I: what do you do when the child fell from higher places?**

R: I make them traditional medicine that we called “fallen drinking medicine” and when they have fever and can’t be heal from having fever. There is also medicine that I do for that.

**I: ok. Now can you please tell me your typical day from morning until evening as a traditional healer?**

R: I usually go out and look for plants and trees that are necessary for my local medicines healing thing. There are just specific medicine that I look for, I don’t just go out there and pick whatever I want to pick. It takes time for me to get these seeds or plants to make healing medicine for my patient.

**I: Let’s now talk about illness. I am specifically interested in illnesses that children suffer from. In your community, what illnesses would you say children under two years commonly suffer from?**

R: they usually ill from, under two years? Illnesses like fever?

**I: yes that can be one**

R: they usually get illnesses like fever, but we also have local medicine to treat fever illness.

**I: now can you.**

R: diarrhea

**I: also diarrhea?**

R: yes

**I: can you explain more on fever, when the child get fever, what do you do to heal the child?**

R: I usually do local medicine that I was told from other traditional healer. There is local medicine for child when they fall. They get fever because they fell and that caused the fever hardly go out from the child’s body. I gather different leaf and seeds that can help heal from fever.

**I: you also mentioned diarrhea, what do you do to prevent diarrhea?**

R: yes I also do medicine for diarrhea because children under diarrhea caused by when parents are not hiding their sexuality in front of their child or they are doing it while the mothers is taking local medicines. Children also have diarrhea when their mothers don’t take local medicine seriously and they breastfeed their children.

**I: what are the causes of these illness? As of fever, what caused the child to have fever or diarrhea?**

R: sometimes we think that they have fever and diarrhea at the same time because there are some parents that don’t really take good care of their child in ways like, leaving them in cool-air or air condition room because they only think of how hot they are and not the care of the child. And as of diarrhea, parents don’t take care of the way they make the child’s foods. Sometimes some mothers are not really interested in making their children’s foods and that end up with unclean foods that given for the child to eat.

**I: ok that’s great. How serious is these illnesses?**

R: it is very serious because there are some children who died. Some parents lost their children from diarrhea illness, and some parents lost their children from fever when they stuck or have high fever and count move their body. I only can say that but I am not really sure, there are some children who have over fever they lost their life from that illness.

**I: hmm ok. Now can you tell me ways to prevent each illness in this community?**

R: well, there are times parents can take them to the doctors, or from our own belief, we think that there are traditional healing medicines.

**I: Can you explain what type of treatment people in your community seek for their children, for example traditional healers, doctors, and the nurses?**

R: I think the very first thing they do is to see the doctors. But there are people who don’t have healed children because they don’t give their children medicines according to hours they supposed to have their medicine. But there are just some children who have illnesses like these. I have seen a mother that needed treatment for her child and she tried taking the child to the hospital and there was nothing the doctors could do to heal her child, then she brought the child to the traditional healer, and then the child was able to recover from the illness.

**I: alright.**

R: I don’t really know what kind of sickness was that.

**I: Who would be the first person parents would go for their child’s health care and why?**

R: let’s say to people that have storing belief, like the pastors, and it’s common to bring them to the doctors.

**I: thank you. Now can you tell me if parents use traditional healers and traditional medicine to heal their children?**

R: I speak from my own perspective that I know sometimes doctors heal the child, and sometimes local medicines heal them. There are times they do stomach massage to heal the child’s pain in the stomach.

**I: alright. Now can you tell me about any challenges your community faces in seeking treatment for the illnesses you mentioned before?**

R: like what/

**I: medicine for diarrhea, fever, or also can be headache.**

R: western medicine?

**I: the question says, what are the challenges your community faces in seeking treatment for these illness, it can be western medicine or local medicine. Is there any challenges?**

R: transportation also can be one challenges that we face. There are some families that face struggles in transportation to the hospital. There are just some families, but when it comes to local medicine, it is more simple and reliable for us. Sometimes we face challenges in local medicine when we run out of plants or seeds for the medicines we are looking for. But the most challenging thing is transportation.

**I: ok now when it comes to you, do you face any difficulties when you provide health services to your patient as a traditional healer?**

R: there are sometimes I face difficulties, but not serious challenges. The only difficult thing is that I have grown old enough nowadays and I feel like I don’t want to walk far away just to get the necessary leaf or seed that I need for the medicine. It is difficult for me sometimes when I feel tired and I don’t feel like I can make a walk from my house to the bushes just to get my ingredient of the medicine.

**I: hmm. That is exactly the level of detail I am looking for in your answers. Thank you. Now can you describe any illnesses associated with nutrition that affect children in your community?**

R: illnesses associated with nutrition affect? I don’t think I have heard questions like that before?

**I: yes is there any illnesses?**

R: what can I say to that, what could be nutritious foods?

**I: is there any children eat nutritious and get sick from these kind of foods?**

R: ok. Let me tell you something true about the children in my house. Not that they get sick from eating nutritious foods, but when they eat they feel dizzy or nausea because they don’t like them, they just don’t like the taste of the food. Whenever I make foods with different ingredient and add mix vegetables to the foods, they won’t eat no matter what. They just hate the food.

**I: okay. Now can you tell me what types of foods that makes a child’s body unhealthy and reasons why?**

R: unhealthy?

**I: yes**

R: there we go, you know foods that are not healthy for the body. We can only eat rice itself. Rice without meat or without green vegetables. We also can eat only the breadfruits itself, without complete foods, which is also consider unhealthy meal.

**I: and what kind of foods that make a child’s body healthy and reasons why?**

R: geez! Foods that are healthy and nutritious for the child, like fruits, papaya, and pandanus and rice, the rice can’t miss from a single meal. Sorry are talking about children under two years old?

**I: yes children under two years old.**

R: well, children under two year old, I can say, yes I mentioned it. Papaya, banana and any kind of given local foods that’s good for the body. Fish is also important for the child’s body.

**I: We talked a lot about being unhealthy. Could you now describe for me a typical day of someone living a healthy lifestyle, from the time they wake up in the morning until when they go to bed?**

R: like what?

**I: someone that live healthy…**

R: adult people?

**I: yes how do you observe that healthy person throughout the day?**

R: they wakes up and play, they don’t stay just stay home and do nothing, they do lot of moving and that is why we can tell that they are healthy.

**I: great. Now what are the appearances or signs of a healthy child under two years?**

R; what are the sighs, hmmm.. When the child moves, eat, when you give the foods the child eat the food. I also can tell that the child is healthy because they can eat any kind of given foods.

**I: Now can you tell me the appearances or signs of a healthy adult?**

R: the minute they wakes up, they start doing works around the house. They just want to do works just to move their bodies.

**I: they just love to do works**

R: yes they wakes up and do works until the time they goes to bed

**I: OK. I have one more set of illness questions but related to women’s health now. Could you tell me about your experiences with women who have anaemia?**

R: women who have anaemia illness are the ones when you look at them, they can stand there and would fall down. And I also can tell from their skin body. It turns to dark purple colour from their original skin body.

**I: do women who have anaemia think it is a serious concern?**

R: some think that it is a serious concern and some don’t really care.

**I: what are the causes of anaemia in women of reproductive age and pregnancy?**

R: there are some reproductive ages that don’t have their monthly period normally, but then when they have it, it takes long time for them to end it. They can have monthly period, they lose lot of blood. I think these are the two thinks that we can tell that women are having anaemia, also when they lose lot of blood.

**I: ok. Now is there any advice given to women for prevention and treatment of anaemia?**

R: yes we always tell them to eat a lot of sashimi. Exercise their body and eat foods or drinks that help provide blood for them. They should eat nutritious foods and drinks like orange juice and especially they should drink water.

**I: water is the best solution**

R: yes

**I: Now I would like to talk about breastfeeding practices in this community. Can you talk about how long after birth most women start breastfeeding in your community?**

R: after the mother give birth, she told to do breastfeed for the child.

**I: she do breastfeed right away after breastfeed or does she have to wait longer?**

R: when they see that she is feeling well enough to do breastfeed right away, then she can feed the child when the child cries after they clean him/her up.

**I: is there any liquids other than breastmilk given in the first six months after birth?**

R: some mother gave birth to the child and still have no breastmilk to feed the child, so they would give different formula like milks or other liquids to feed the child.

**I: ok now can you could you explain exclusive breastfeeding practices in this community?**

R: they just do exclusive breastfeeding. They don’t give different liquids like water or milk for the child. Whenever the child cries, mothers would feed them with breastmilk. They don’t feed their child with different liquids, they don’t also give water for the child to feed from a baby bottle.

**I: and what about the very first breastmilk? From your own understanding, do breastfeeding mothers squeeze out the very first breastmilk for the child?**

R: yes that’s the thing, I have heard it from doctors that the very first milk that came out of the mothers breast is the most unique milk that should be given for the child.

**I: so why do you think the doctors says that it is a very unique breastmilk and needs to be given for the child?**

R: well I don’t really understand why but I have heard it many times from the doctors that mothers should give to the child because it is really vitamin for the child’s health.

**I: what are some difficulties faced by mothers in your community to practicing exclusive breastfeeding for six months?**

R: first six months?

**I: yes? What the difficulties they face during exclusive breastfeeding?**

R: I don’t think there is difficulty they face during exclusive breastfeeding, the only difficult is when they stick around only with their baby. If mothers spend a lot of their time more than enough, they are practicing the child to never let go of them. If they would want to do some house chores or some food for the child, it won’t work for them. The child will cry because he/she just want the mother to hold her/him. The child is used to be only with the mother, so when the mother stand and do something for the child, it will really hard because the child would never stop crying.

**I: so is there any specific ways to better support mothers to exclusively breastfeed for six months?**

R: yes we always tell them to exclusive breastfeed so that the child can live healthy and one good example I did when I used to be a breastfeed mother, I never want to wash baby bottles so I did exclusive breastfed for my children and I get better benefit from that because I never want to wash these bottles, and my children never had any illness due to exclusive breastfeed. There is nothing to bother me, if I want to go somewhere else, I just crab my baby and go. I can do breastfeed on our ways to places we go, I don’t have to worry about baby bottles and all that.

**I: that is really great. Now we are trying to understand how people eat in this community. Could you describe in detail what most families usually eat and drink throughout the day?**

R: geez there is nothing else except from rice or bread every day. Even though we still got local foods to eat, but we commonly eat rice or bread throughout the day. Bread in the morning, like pancake, or bun, and rice for lunch and dinner.

**I: okay pancakes, bread, and what else?**

R: doughnut, and all kind of flour ingredient baked and we commonly eat throughout the day.

**I: how do you make the foods?**

R: how do I what?

**I: if you make pancake, how do you make the pancake?**

R: well, we just bring the ingredient like the baking powder, vegetable oil or adding milk something like that.

**I: who in the family is served first, next, and last?**

R: well, it is common to serve the children first because they will cry when they’re hungry, and then the people who have office work or work around the house would be the next to be served, or let’s just say students and employers or worker around the community.

**I: is there any differences in quantities of food served to different family members?**

R: yes there is

**I: What are the differences?**

R: the only difference is that we can serve adults more foods than the children. It depend on how much people eat.

**I: do some children receive more food than others?**

R: yes because there some children eat more than the others.

**I: Now could you describe any food sharing between family members during mealtimes (for example children eating together separately from the family, meals eaten from the same plate by all family members)?**

R: who am I going to talk about? Such that my family members are using separate plate during meal but sometimes when I go to other people’s house, I see that children share the same plate to eat with the adults people. Mothers can let two people share the same plate because maybe they don’t have enough foods for the family members or they don’t have enough plate to use.

**I: do you and your family members share foods between households or to your neighbours?**

R: yes this habit will never stop, except when we don’t really have enough foods, then we don’t have to share if we know that we don’t have enough for our own family members.

**I: Now I want to know about how young children eat in this community. Can you describe in detail what children under two years commonly eat throughout the day?**

R: now what can I say about that because we are outer islands people. We don’t have anything else except from cooking or baking doughnut or pancakes for breakfast. It depend on whether people like the foods or not, we don’t have any choices, we just do whatever foods that should be ready on the table for our family members to eat. And for the children under two years old, they also can eat with us but we make different foods for them like soften food that they can be able to eat.

**I: ok and what kind of food?**

R: if we can make soft food like the iq, that we cook the coconut meat with flour and boil it with water. We add too much water in the food so that it can be soft for the child to eat it. Or we can also make boil rice and add water to it to make it soft and add soft meat to it in order for the child to happy to eat it.

**I: hm that’s great.**

R: and it is common to feed children under two years foods that are necessary for them to eat, healthy and soft foods.

**I: How many times a day meals are eat by children under two years?**

R: children under two years let me think? I think they eat more often than us

**I: yes and how often or how many times a day they eat?**

R: they can eat their breakfast and play for a while, then minutes later they feel hungry again so they cry for food and we know that they are hungry then we feed them again. That happen the same thing for lunch and after lunch until its dinner time. The fact is that we would never know if they are hungry or not, but we realize that they are hungry and they want foods when they cry now we feed them. It can be three or four to five times they eat meals throughout a day.

**I: do children typically given snacks between meals?**

R: well I can say that there are more children under two are not given snacks than children that snacks are given for them. Children in my house are not given snacks only when we have are not busy we can pill the papaya or give the yellow banana so that they can have as their snacks. Or they can have their snacks from the food they eat for their regular meal. Sometimes they also given chips or right ships are consider snacks right?

**I: yes**

R: ok right so they sometimes given cereal or chips for snacks but not all the time only if we have and we can afford for them since it is really expensive in the outer islands and we don’t really rely on these kind of foods because we have available snacks for them like papaya or banana only if there is chips, cereal or papaya and banana but if we run out of these kind of foods then they can’t have snacks throughout the day.

**I: is there any different in feeding the children when they are sick?**

R: oh yes there is

**I: what is the difference?**

R: there are lot of food given for them, I mean for these sick children, we prepare lot of foods because we are trying to figure out what kind of food they want to eat the time they are sick and picky on foods.

**I: ok that’s great. And what about the time that they are not sick?**

R: well, we give whatever foods for them to eat. They can get any kind of foods given for them to eat.

**I: is there any differences in feeding practices between girls and boys under two years?**

R: hm I am not really sure about that. Difference in feeding practices between girls and boys?

**I: is there any differences in feeding girls from boys?**

R: yes there can be any differences in feeding girls from boys. Sometimes girls are too picky on their foods or boys eat more than girls do

**I: Can you talk to me about what influences how families feed their children in this community?**

R: what, what?

**I: what influence how you feed your children in this community?**

R: yes there are times that doctors, nurses or health assistant visit us here and give us advice on feeding our children. Telling people that they should feed their children. Even adults people in this community telling parents that they should be take their time and feed their children.

**I: We have heard from some families that eat local foods and others that eat processed foods. Could you explain what is typical for most families in this community?**

R: well there are just some families that eat local food and there some eat processed foods. There is season for food like breadfruit and there are some families often eat breadfruits. But rice and bread are the common foods that we eat every day.

**I: can you tell me anything that makes it difficult or easy to cook local foods?**

R: What makes it easy to?

R: cook local foods, what makes it easy to cook local food.

R: it takes too long for local foods to be cook. But it is really simple and easty to make local foods.

**I: What makes it difficult to make local foods? Do you face any difficulty when you cook local foods?**

R: yes when I don’t have wood or propane gas to make a fire to cook the food. This is outer islands and the only difficult is that we don’t have wood to start the fire or no stove to cook the foods on. But there is no difficult in cooking local foods. And another thing that makes it difficult is that it took a long time for these foods to be cooked.

**I: that’s great. So what are the positive or negative things about eating local foods?**

R: I say that there is no negative things about local foods, local foods are really healthy and fresh foods.

**I: why do you say that local foods are good? What are the positive things about local foods?**

R: there is no way we refuse to eat local foods. When we see local foods around, we always want to eat. There is times that we crave to these foods and not like processed foods like rice and all that, we used to eat and when we eat them nonstop, we now hating them and don’t want to repeat the foods over and over again. It is not the same thing like local foods, they are fresh foods and there is no way of hating these foods.

**I: what are the positive or negative things about eating local foods?**

R: we can’t just get them whenever we want to, we have to buy them. Only if we can afford for these processed food then our families can eat.

**I: ok. Now is there any suggestions for balanced meals that can be prepared with locally available ingredients for children under two?**

R: what?

**I: nutritious or healthy foods, is there any suggestions for balanced meals that can be prepared with locally available ingredients for children under two?**

R: Yes we can balanced meals with locally available ingredients for children, there is no problem in doing that. I don’t think there is problem in putting different ingredient with the fish or add fish to any kind of local foods available.

**I: Now can you talk about what messages about breastfeeding and complementary feeding you give to mothers or other community members?**

R: differences on what?

**I: like what messages about breastfeeding you would give to mothers or other community members?**

R; we would prepare foods or drinks for them, foods and drinks that help provide breastmilk for the breastfeeding mother. And it is also important to feed children under two more because when they get sick, they won’t refuse to eat, they still get their changes to love their foods. We make them soup that should add fish in it because the breastfeeding mother can have enough milk for her child.

**I: is there any nutrition education activities with community members as part of what you are doing as a traditional healer?**

R: what?

**I: as a traditional healer?**

R: yes

**I: is there any messages that you give to people you help them or mothers that you heal their children form your local medicine. Do you have any education activities with community members as part of health work?**

R: no respond

**I: do you usually give messages on nutritious or healthy to breastfeeding mothers that you see or that you help them with local medicine?**

R: yes for breastfeeding mothers, I usually them that they should not eat sashimi

**I: yes and why they shouldn’t be eating sashimi?**

R: it is a belief that when breastfeed mothers eat sashimi, the child can bite the nipple while feeding from the breast.

**I: ok that’s great. So is there any difficulties to delivering nutrition messages to caregivers?**

R: I don’t think there is difficulties to deliver nutrition messages to caregivers.

**I: there is no difficulties?**

R: oh no. yes there can be difficult when some people don’t eat healthy food, sometimes they would eat only rice without meat. They can just eat rice without meat or without any vegetables in their foods.

**I: why do you think these people don’t eat healthy foods?**

R: what can I say? People are lazy to bring or make themselves nutritious foods. They are lazy to grow healthy food and they are lazy to make themselves nutritious foods.

**I: what would be some specific ways that nutrition communication could be more effective?**

R: we have to advice each other that it is very important to us to eat healthy and nutritious foods. It is important to have healthy foods in one meal, like for example we can have breadfruits and fish for lunch or fish and breadfruits for dinner something like that. We should eat from the three groups of foods to make have better body growing especially for to the children.

**I: alright. Now I would like to talk about pregnant women in this community. Can you describe their diets during pregnancy?**

R: well pregnancy women have different diets form each other. This woman have different diet than that woman, we have different diets than each other. There are some pregnancy women who hate fish while some love to eat fish. Some like to eat crab while some don’t want crab but the truth is that, they have to eat healthy and nutritious foods.

**I: great. Now do pregnant women usually change their diets during pregnancy?**

R: yes they do

**I: ok now what really influence their diets during pregnancy?**

R: that is the thing I do not understand about pregnant women. Maybe it is because they are pregnant and they just want to. It is like they like they just want to change their diets. If they want to eat the foods, it very necessary for them to have the foods. If they don’t want the given foods, then we don’t have to give them the foods because they don’t like it.

**I: what kind of foods women are encouraged to eat during pregnancy and reasons why?**

R; they should be encouraged to eat healthy foods. But is it really funny sometimes when some women crave for stinky or rotten coconut and there are also uneatable foods they want to eat.

**I: who encourage eating those foods during pregnancy?**

R: what kind of foods?

**I: the healthy foods**

R: well sometimes their husbands, or the elders in their families or it can be any people that have better experiences on women pregnancy but mostly they are encouraged to eat healthy foods by their husbands.

**I: Can you tell me about any supplements normally given to women during pregnancy?**

R: if they go for medical records

**I: ok and what kind of supplements given for them? Do you have any ideas about the given supplements for these pregnancy?**

R: I don’t know and I can’t name these supplements but supplements for blood or vitamin. But the fact is that, most of the women said that they feel so ill when they take these supplement and it make them feel even worst. There are few of them don’t take these supplements at all and there are just some who take them.

**I: ok that’s great. What prevent women from taking their supplements during pregnancy?**

R: the truth is that some women said that when they take these supplements, they feel dizzy and don’t feel good. They have bad feeling when the take these supplements. But there are some women who don’t have any difficult in taking these supplements. They finish their supplements before upcoming pregnancy medical check-up.

**I: is there any times women drink alcohol, smoke or use other drugs during pregnancy?**

R; I don’t think there is here in this community, only few but they only smoke.

**I: ok. Can you now describe women’s diets during breastfeeding in this community?**

R: common foods that we have here in Arno since we live in outer islands. It is common for us to eat rice or bread every day.

**I: aside from rice and bread, is there anything else?**

R: Can be can meat or fish or it can be any kind available meats here.

**I: do women usually change their diet during breastfeeding?**

R: some do that because they belief that they should change their diet during breastfeeding but some eat whatever foods is there and ready for them to eat during breastfeeding

**I: yes. What kind of foods breastfeeding women are encouraged to eat and reasons why?**

R: well they mostly have advice from the doctors that they should eat foods

**I: what kind of foods?**

R; fruits and vegetables or healthy and nutritious foods.

**I: ok now what kind of foods breastfeeding women are encouraged not to eat and reasons why?**

R: I think I have mentioned before, like what kind of foods? Foods that breastfeeding women shouldn’t be eating? Foods that are not good for breastfeeding mothers. If it is not necessary for them to eat sashimi, they shouldn’t be eating sashimi. Fishes that’s not appropriate for them to eat, like fishes that cause illness or can cause diarrhea and illness like vomiting or headaches.

**I: Who encourages or discourages eating those foods while breastfeeding?**

R: usually their husbands or their parents

**I: ok that’s great. As a tradition healer, what are some of your biggest concerns of the diets of pregnant and breastfeeding women intis communities you work in?**

R; common foods that we have here in this community. We want them to eat healthy foods. They need to eat healthy foods like fish and fruits or vegetables that we have here in this community.

**I: you answer perfectly. Now for the last section, we would like to learn about ways we can develop health programs in your community?**

R: I usually get information from nutritionist people who sometimes visit us here in tis community and sometimes I listen to these information from the radio station. It can be anywhere I go I also get information from my friends or from places where I do physical exercises. Places where people gather together we also share information.

**I: why do you trust where these sources come from?**

R: because we know that they are from the health centre and people bring these messages are well educated people.

**I: ok. So where do you think nutrition and health and messages should be delivered so that community members would see or hear them most easily?**

R: I can easily hear them from health assistants or from the school teachers especially from the doctors and also from nutritionist that usually come to outer islands and bring health messages about health and nutrition.

**I: great. So what types of media that community members use the most to communicate?**

R: as of right now, I am only use cell phones

**I: ok what else?**

R: CB radio or the radio station we use to get information from.

**I: For our last question, could you describe what influences how people raise children in this community?**

R: one more time please can you repeat that again?

**I: what influence how people raise children?**

R: well are told to take good care of their children. Give them foods that are good for their health and see the doctors when the children get sick.

**I: is there any specific advice or information related to parenting typicall given to community members?**

R: yes sometimes I tell them to do exclusive breastfeed because it does really help give the child healthy body. If they get sick, they refuse to eat then they can only feed from breastfeed. Children that only feed from breastfeed are the healthy ones because they get vitamin from breastmilk they feed from their mothers.

**I: is there any information that pregnancy or breastfeeding women typically ask for from you as a traditional healer or any traditional healer you know? From the way you help breastfeeding mothers or pregnancy, or as a traditional healer, is there any information women or your patient typically as for from you?**

R: well yes, there are some mothers that sometimes ask how do they take good care of their children or raise their children. I usually tell them that it is very important to raise our children in the most loving and caring way because when they grow up they will know what is right and what is wrong in lives. And children are very unique to be taken care of because they are our blessing and we as mothers, it is our responsibilities to watch over them and raise them. I usually tell them to never depend on other people in raiser or take care of our children, wherever we go somewhere else, we have to take them with us.

**I: alright. So what would be the best way to communicate with caregivers about health?**

R: yes it is important to tell then to be there with their children throughout the day or the night because it is their responsible to take care of the child’s health and growth.

**I: anything else?**

R: we also advise them to feed their children and make sure the child is clean and neat so that children can live healthy.

**I: alright that’s great. Is there anything else about the topics we talked about today that we missed or that you would like to tell us about?**

R: I don’t think there is anything else. I don’t have anything else to say.

**I: aright we’re done and thank you so much for your generous time and information that you shared with me.**
