# Supplementary material for: Conceptualizing multi-level determinants of infant and young child nutrition in the Republic of Marshall Islands–a socio-ecological perspective
Source: PLOS Glob Public Health. 2022 Dec 19;2(12):e0001343. doi: 10.1371/journal.pgph.0001343 (PMC10022247; doi:10.1371/journal.pgph.0001343)
Supplement: S1 Data — (ZIP) [file pgph.0001343.s001.zip › RMI Supp Data/Interviews data/I55R_IDI_CL_Arno_Sep 17_BM FelaEdited.docx]

- **Interview code: I55R**
- **Interview type and interviewee: IDI CL**
- **Interview date: SEPT 17**
- **Location: ARNO**
- **Interviewer: BM**
- **Transcriber: Shante**

**I: Do you agree to let me record our discussion on this recorder?**

R: yes. I do.

**I: Ok. Thank you for your beautiful time. Before we start, can you tell me what your responsibility as a leader in your community?**

R: my responsibility as a leader in this community, first I would change people’s life here and protect and teach them, and second, I don’t just change their lives, I would help them to achieve whatever it is in this community. How to teach them about life and not on how they act. it’s important because one that stays in this community should know our strength and know their rights in their community. And these are my responsibilities in this community and especial teach them the words of God.

**I: good. Your information’s are good. Can you tell me who stays in this community?**

R: at this moment, every people in Jebo, and from jebo the preachers from BNJ. (it’s a church) preacher waju and his wife. And johnty and his wife along with their grandmother, my wife and I, and Deacon Lechon and his wife and okabah the businessman and preacher Junior and his family, and Sam and his family, John and his family and Cory and his family, Junia and his family and Joshua the driver of the diesel, and Daniel and his family and Robinson and his family.

**I: can you describe about the leaders in this community?**

R: at this moment, from my own knowledge the Lerooj (queen) her name is Lemtanur, and his son that takes her place and his kids are watching over the community. Junia and the other one is jalo mailia the daughter of the school head teacher, Ezekeil and danile. Our councilmen is not here.

**I: Ok. Good. Thank you. Other than the Marshallese here, who are the foreigners that stays here?**

R: at this moment I think all the people here in this community are all Marshallese. I believe okabah we won’t count him as a foreigner because he already has is signature to be a Marshallese citizen.

**I: Ok. Good. Can you tell me the important of the churches in this community?**

R: it’s important because it keeps our culture and peace in this community.

**I: Now can you tell me the good things about this community?**

R: at this moment, this community is doing great. They join on the big events and they know how to respect each one, knows how to work together, together in school and work together in school and take the good things about this school so that they can take the programs in the school. When it’s time, our culture when it comes to funeral, when the ainekien Manit (words from the chiefs) in this community are together in this community they go to the funeral.

**I: what are the issues in this community?**

R: the issues that effects this community is that the hospital is far, and things cost too much in the store that we stay at in this community. We need transportation to go to the hospital because going to Arno it’s far and you need transport and it cost $20 and $5 to use the truck of the councilmen. And we are also having issues on living and foods. at this time that it comes on season, and when the season is done. We will start eating from the stores, but the foods are so expensive. Pandanus comes during Pandanus season. If pandanus season is over then there will be no more pandanus. Banana we barely eats. If you have banana plants, the issue is there’s rule to prevent big from destroying the banana plants. We see this issue that the law doesn’t work because the pigs kept destroying the banana plants. Our government Is weak on implementing by what goes by. Those are the issues at this moment.

**I: Hmmm… good. Thank you for your information, they are very important. Ok. We will now talk about heath and illnesses in your community. Could you explain what illnesses children commonly suffer from in your community?**

R: in this community, from my own knowledge, the kids comes from the chief of this island, they have titles. They’ll be the new future for the future generation. They have a right to sit on the chairs, and also the children of the land lords. They also have a title.

**I: sorry if you didn’t understand my question. Can you explain what kind of illnesses that children in this community usually gets them?**

R: the illness that children in this community usually gets them is diarrhea, cough and fever. those 3.

**I: Oh… fever, cough and diarrhea. What makes the kids cough?**

R: I think what makes the kids cough is from us parents.

**I: Ok.**

R: we barely watch over them from playing where It’s sunny, and when it’s raining if they want to go shower in the rain they go, but we don’t limit them from showering in the rain. I know about where it’s sunny and when rain comes, the weather will be different. And it makes us to be sick. Diarrhea comes from us not taking care, there are times…. Not just grown ups but kids to use the lagoons to poop and the flies fly and that is the reason why they have diarrhea.

**I: hmm…**

R: coughing is also from fever.

**I: hmm. Good. On these illnesses, how serious are they? How serious are these illnesses?**

R: diarrhea sometimes are serious because sometimes… bring them to the hospital on Majuro because our doctors and our medicines here are not useful. They are not allowed to use strong medicines. And these are the serious illness for the sickness. For fever, last year, there was one child that died from fever. the child’s parent they thought the fever wasn’t very serious, they tried to give medicines and rush to the hospital and the doctor wasn’t there, he was at Majuro. He had a patience there. They rush the child to the emergency and once they got to the hospital the child was dead. He was 2, almost 3 years.

**I: how about on coughing?**

R: coughing when, you know when change climates…

**I: yeah?**

R: you will see the child, not the child but the adult itches and have a long cough.

**I: Oh. Ok. Good. Can you tell me how would you prevent these illnesses?**

R: only one way it’s go to the hospital. And I believe parents should know how to protect their children. That is the way to prevent the illnesses. So, the first place we should bring our children to is the hospital.

**I: can you tell me what the people in this community usually do to seek treatments for their children in this community? Like for example traditional healers, doctors or nurses?**

R: I think from my own knowledge when they have illness they go to the hospital.

**I: hmm**

R: but there are other illnesses people try to use local medicines, like local doctors. Like Marshallese black magic people, those who are really good in doing black magic also can heal the very sick child right away.

**I: Ok. Good. Thank you for all your information. Can you describe any illnesses associated with nutrition that affects children in your community?**

R: illnesses that comes from the foods that affects the people in this community are kids having diarrhea…

**I: what type of food would make your children unhealthy?**

R: the junk foods from the stores…

**I: hmm**

R: those are the foods that make the children’s not healthy. And the food that you keep from the other day, our motto these days is, be careful because life it’s hard these days and we don’t have enough money. So, we will reheat the food from yesterday and eat it again.

**I: oh… ok. Good. What type of food that will make a child healthy?**

R: well, from my own knowledge, we use planted food from our community that we use is green beans to eat for lunch. We have lots of that here and we can boil it and mix it with fish. I believe that there is lots of vitamin in the karteeb (local plants) than cabbage. There is one book that read a study that was made in the university of New Zealand and it shows that karteeb (local plant) it’s really important. And the vitamin in it it’s bigger then the cabbage that they bought from the stores and they are still fresh. And there was one tree and it’s the tree of life. It’s called Malunggay (kind of like a spinach) it’s from the Philippines. There is here. I planted it here in this community. it’s really important, you can just boil the water. Let the kids drink it, and they get their energy from it.

**I: is there any other words that can prevents the child from the foods that are not planted?**

R: come again?

**I: is there any other words that can prevent the child from eating the food they eat that aren’t planted?**

R: oh yeah. It can come from foods like ramen and, junk foods. they can affect the health of the children’s, and junk foods, now a days they are already selling chips in the stores and I believe these are making the children eating it more because it’s tasty, but the truth is they have no nutrition in them.

**I: Ok. Thank you for your information. Now we are done talking about health, we will now talk about planting. is it ok?**

R: good.

**I: can you explain to me how people in your community typically get food each day?**

R: the difficulty is people should have their own foods first, when we talk about food from the stores then there is not enough money. At this moment, the price of a coconut is .50 cent one pound. At some stores they can get it with .46cents and some other stores with .40cents. and other stores with .45cents. When you take 85 dollars from a store, when you pound your coconuts you will lose .75cents. no, you’ll lose 10 dollars. And the cost you earn is 75. And 75 dollars you use for rice and spam, mackerel and it will double the price in Majuro. The price got higher.

**I: what type of plants are usually planted here?**

R: there are like breadfruit, coconut juice, coconut meat, pandanus and bananas. These are the things that grows here. I believe there are more that we can plant to have more. We could do cucumber, tomato and melon, pumpkin…. We can also plant those… sweet potatoes we can also plant that.

**I: Ok. Good. The plants that grow in this community, do you guys sell them?**

R: at this moment, the plants that you sell them is… biro (made from breadfruit) we can also sell them.

**I: biro? (made from breadfruit)**

R: yeah. And pandanus paste, we can also sell them. Bananas, there are some people that take them to Majuro and sell them. And also lime, they also take it on the boats and bring them to Majuro and sell them there. And coconut meat, they can also take it to Majuro and sell them there.

**I: Ok. They take them there and sell them?**

R: yes.

**I: Ok good. Now when they sell these things, what do they do with their earnings?**

R: with their earnings they buy food.

**I: food for them?**

R: yes. And their needs.

**I: their needs. Ok good. Thank you for your information. Can you tell me about any difficulties on growing food in this community**?

R: there is planted foods here, but we need something to prevents the animals from destroying the plants. Sometimes we plant bananas at near our home… sometimes you can plant, and the pigs will destroy them. And that is my own knowledge of it. How I see planting in this community.

**I: good. What do the family need to grow food at home?**

R: they need a lot. They need implement and fence for the pigs, but from our culture, if the chief doesn’t want the pigs to be in a fence, then we will stop. But we need a wire to fence the plants that we planted. And we need tools for planting, so we can move forward on planting. and we also need seeds. I don’t know where I can find them on Majuro, but we sure do need seeds.

**I: Ok. Thank you for your information. Can you tell me how easy or difficult it is to get those foods you mentioned every month during the year?**

R: make each family to work together. And one community to do community work and agree with each other to make, like the work KUMIT, we can come together and work together with each family like, one, one, one. We can make 3 to 4 groups on planting mines and tomorrow help plants yours. If not, we can skip another day and do it the next day and the other day. I believe it will be easy and there will be foods everyday for each family in this community.

**I: is there any time that you guys run out of foods? and why is that there are no more?**

R: well there are times in this community that runs out of foods. sometimes you can hear on the GP radio that they say the island is nita (meaning when the island is at hunger and they run out of food.) and the reason why because the ocean is not good. The small boats can’t bring them. And the stores won’t supply all the families with the foods because under 3 weeks they will run out of foods and the boats hasn’t arrive to bring the foods.

**I: What do the community do to feed themselves if there is not enough food in the community?**

R: on the days that we are running out of foods, we go fishing and eat coconut meats and eat coconuts. And we drink coconut juice. These are the foods that we eat when we run out of foods.

**I: good. Thank you for your information. What kind animals that are here in this community and what kind of animal that this community usually have?**

R: well there are pigs, there are dogs, there are cats, there are chickens in this community. These are the animals that we have and that I know of.

**I: why do the community tap (feed the animals) the animals?**

R: the reason why they tap them is so that they can live by them, eat them sometimes. And one other reason is selling them.

**I: they tap them and sell them?**

R: yeah. And sell them. Exchange it to money.

**I: is there any other reason why they tap the animals?**

R: there is a word that we usually say… our church usually say… kakonana (when each members bring a food to a botlak.) to the church when they are together, they kill the pig and eat together.

**I: ok. Good. What are the issues on tapping the animals?**

R: the issues on tapping the animals is their foods. it’s hard for us… if you think about feeding the pig coconut only, it will be hard for you to find more coconuts to pound them. And that’s why you can’t have plenty animals. That’s the issue. There is no other reason, there is no other food for these animals that we can give. Only coconut we can give to them to eat.

**I: Good. Good. Are there any difficulties on putting the animals in a fence?**

R: the way I see it… the difficulty is there foods when you put it in the fence and feed them, if you feed them coconuts, you will throw away lots of coconut and give them to the animals. If you had 5 to 6 pigs and up, it will make you have less coconut. This is the issue.

**I: to put the animals in a fence?**

R: yes. Put them in a fence.

**I: OK good. When they are not in a fence then they can eat anywhere?**

R: yes. You can not feed them, but they can just roam around and feed themselves.

**I: Hmm. Good. The people here, do they usually step on animal poops?**

R: I think, I don’t know.

**I: you don’t know?**

R: yeah.

**I: Ok. It’s good. Good. For the last question on food, can you explain who decides what food to get for the family?**

R: well, all children?

**I: the children?**

R: yeah. the children especially because the children are important in this community and because they are the main ones in the family.

**I: ok. But who in the family that chooses the foods? who? The mother or the father?**

R: mother and father. They are the ones who choose the foods.

**I: they are the one who choose?**

R: yeah.

**I: good. Who chooses what food the children should eats?**

R: the mother is the one that choose the foods.

**I: the mother choses?**

R: yeah.

**I: thank you for all your information. In this lesson, we will learn about water and cleaning. Is it ok?**

R: hm…

**I: can you please describe a typical day in getting and storing water in this community?**

R: find and storing water, there is one family that they have their own water tanks. But not us because we haven’t had water tanks. We keep them in containers.

**I: containers?**

R: yeah. We keep them in containers and drums, plastic drums. That is where we store our waters. Especially some of the families have water well.

**I: water wells?**

R: yeah.

**I: Ok. Good. The way you see it, they um, in this community, where do you guys get water for drinking and cooking, washing and bath**?

R: there is in the bantoon. We can take water from them for drinking and cooking and for bath.

**I: good. What are the difficulties to have water?**

R: the thing that is difficult about water is less catchments for waters.

**I: not enough water catchments?**

R: yes. Less water catchments.

**I: what are the difficulties in storing waters?**

R: on storing water is when the water catchments are not good that you are using. You won’t be able to drink from it. There are some houses that have cement bantoons. And they can put the pipe so that the water can fill up the tank, the water cement, but it’s cracked, the water cement.

**I: oh. So, you mean the water cement is broken?**

R: yes. Is cracked.

**I: hm. Ok. Good. What ways do people do to make their drinking water tanks clean?**

R: at this moment, they clean it, the things I know is they clean their tanks at their houses, like the…

**I: gutters?**

R: yeah.

**I: you guys clean the gutters?**

R: yeah.

**I: and the bantooms, how do you guys clean them?**

R: if the bantoon is no good, you can take lid and pour out the water and clean it with Clorox.

**I: Clorox?**

R: Clorox.

**I: good.**

R: finish.

**I: Ok. Good. We will now talk about washing hands. Can you describe how people wash their hands in this community?**

R: well, according to what I know of, people bring bowls and soap

**I: bowl and soap.**

R: make 2 drips of Clorox and wash their hands.

**I: they use Clorox?**

R: yeah.

**I: 2 drips of Clorox in the bowl of soap?**

R: yeah

**I: Oh… Ok. On what you think, what’s the difference between washing your hands with water and water with soap?**

R: with water only, it’s not that clean.

**I: Not clean.**

R: it won’t be clean because the germs will stay, they won’t die but if you sue soap and Clorox the germs will die and will be gone from your hands.

**I: Hmm… good. What prevents people from washing their hands with soap in a day?**

R: sometimes they lack, sometimes there is no soap.

**I: no more soap?**

R: yeah. There is no money to buy soap.

**I: Oh Ok. No money to buy soap. But there is money to buy cigarettes…**

R: yeah. Cigarettes and chips…

**I: hm…. Ok. Good. Can you describe the types of toilets in this community?**

R: there are some people who use the toilet bowls.

**I: toilet bowls…**

R: and some others use the lagoon.

**I: Ok. So, majority use the lagoon?**

R: yeah.

**I: (giggles.) good. Good. What about the houses that have toilets bowl, why is the reason they choose toilet bowls? From your own point of view?**

R: well, it’s easy for them, night and day.

**I: easy for them?**

R: yeah. Easy for them to just go inside.

**I: easy for them to use.**

R: yeah. Easy for them to use.

**I: good. But on some places, they use the lagoon, for one reason they use the lagoon. Can you me if this is still happening?**

R: giggling.

**I: in this community, does this situation still happening? For people using the lagoon?**

R: I believe so, because there is no…

**I: toilet, no bathroom.**

R: yeah.

**I: and why, what are the issues on using the toilet?**

R: if there is not enough water.

**I: not enough water?**

R: yeah.

**I: ok. For flush?**

R: to flush and…

**I: I see, I see. Good. Ok. Can you tell me how people in this community take their kids poops?**

R: the things I usually see is, there are usually some that dig…

**I: dig? Dig holes?**

R: yeah. Dig holes and throw it in the hole and bury them.

**I: Oh. Ok. On the lagoons or near there house or wherever?**

R: wherever. Everywhere.

**I: ok. Good. All right. Thank you for your answers. Now, can you tell me where the children’s usually play in this community in a day?**

R: usually they play on the road.

**I: on the road?**

R: yeah.

**I: hm.**

R: the lagoon, on the sand. On the road.

**I: do the kids play where there are animals?**

R: there is.

**I: there is?**

R: yeah.

**I: hm. Can you imagine a playground for kids, imagine a place for kids to play? Where do you see that the kids can play at?**

R: the place that I think it’s better is the school.

**I: the school?**

R: yeah.

**I: can you tell me the reason why you say the school?**

R: because the school is in the middle of this community, and it’s easy for the kids from both side of this community to get there and easy for the parents to get there when it’s almost evening to look for their kids near the school.

**I: Ok. Good. What are the difficulties in cleaning the children’s playgrounds? Is the children’s playground dirty? The places that the children’s play, is it usually dirty?**

R: I think it’s good.

**I: it’s good? It’s not usually dirty?**

R: yeah. It’s clean.

**I: good. Ok. To end these questions about cleaning, clean you explain ways to prevents the spread of disease?**

R: we should know how to be clean/cleaning. And not just garbage, people’s garbage, animails garbage, because some of the animal’s garbage are far away. Like pigs, chickens and dogs. It’s important for us to clean because it prevents us from the disease and sickness. And we need to clean, take them and dig a hole and put them in, and bury them so that there won’t be any flies to be around it and comes flying to our foods. that’s the important thing. And the garbage, it’s important to clean. This is an island that has lots of mosquitos and we need to watch our garbage and clean the dirty waters and make sure there are no mosquitos inside because it will prevent us from some illnesses from the mosquitos. These are what I know about the important of cleaning in this community.

**I: good. What is your thought about feces and illness? Do people get sick from feces? Do they?**

R: yes.

**I: yes. Good. Ok. Thanks a lot. Now, we will talk about the roles of each family members in raising the children. Is it good?**

R: hm…

**I: could you describe how they care for the children throughout the day in your community?**

R: well, each family how they care for their children is, from my own understanding on how I see it day by day, there is families that they look at children and they care about what they eat and watch them play where it might be dangerous. And this is the thing I know about the importance on caring for our children’s in this community.

**I: Oh. Ok. Who is the one that is responsible for the children’s?**

R: the mother.

**I: the mother?**

R: yeah.

**I: ok. What makes a mother good?**

R: the things that makes a mother good for the children’s is, she knows, she usually, and when is enough for a mother, for her to live, makes a mother not to be stressful about the kids, and when the father is helping the mother to be a good mother and not to depend on the mother only, but for both of them to work together and… this is my announcement for mothers to be nice, needs to work with the father, needs to work with the family members and should make the family to be a good family.

**I: good. Ok. What makes a good father?**

R: the mother needs to stand behinds the father, and the kids needs to stand behind their father and watch over each other and help each other’s in the family.

**I: what are the roles of the father?**

R: the roles of the father in the family is, he is the one bringing foods and give It to the mother to cook it. He’s the one protecting them from danger. These are the roles of a father in the family. The father roles is to find ways to make sure his kids are in school and have registration…like that. in one community.

**I: how do the caregivers…? are there any babysitters on this community? Like how do the** **babysitters play with the children?**

R: I don’t think there is.

**I: hm?**

R: I don’t think there is.

**I: no babysitters? Then who usually watch the children when they play?**

R: Oh… family members only.

**I: family members only? How do they play with the children?**

R: there’s time when you see them play with ball, and there’s some family members that play with the kids, sometimes you see the father and mother playing with their kids.

**I: hm. Good. Can you tell me how do the family play with their kids?**

R: there are times when the family are together, and they go swimming in the lagoon and play, there are times they play in the road when they play volleyball.

**I: volleyball?**

R: yeah. The family are always together, together on playing volleyball, baseball on the roads, and long ball, you will see them play together. They usually play together.

**I: hm. Ok. Good. Ok. Can you tell me the role of the grandparents on raising the children in this community?**

R: well the grandparents, from my own knowing, they watch over the kids so they won’t get hurt**.**

**I: hm. So, they won’t get hurt?**

R: yeah. There are times that I know, the times for school, I usually see grandparent, the kids ask help school works with their grandparents.

**I: how do the grandparents help the children and help the parents of their grandchildren’s?**

R: their grandparents of the kids, they help their mother and father, leave their mother and father to do chores in the community while the kids stay with their grandparents. The grandparents will go looking for the kids when they are not at home. And these are the responsibilities of grandparents. Sometimes when is close to night time, they will walk from the end of the town and look for their grandkids and bring them back home. And these are what I usually see and know about.

**I: hmm. Oh. What makes the grandparents good?**

R: well. When the kids, the mother and father of the child, and the kids are standing near their grandparents, in our culture and knows manners and respect their grandparents. One grandmother is good, and the one grandfather is good in this community.

**I: good. Good. Very good. Can you tell me about the role of others in raising children’s in this community?**

R: to others, on watching over the kids, sometimes… I see kids in this community, there are times that they fight when their parents and grandparents are not around, for other… that I saw and learn from, from the little kids, I talk to them and let them play together and, learn how o cooperate with each other and let them not to hate each other.

**I: hm. Ok. Your answers are so nice. We are almost done. Oh. And how do the older siblings watch over their younger siblings?**

R: there are some families, there are some kids that don’t watch over them. They are usually jealous. The life of jealousy comes from how they see the action of the family, like the mother and father, if they see the father and mother spend all their times with the youngest ones, it will make the older child thinks that the parents love the younger ones then the older ones. And that’s why you see them fight, there are times…

**I: few kids?**

R: yeah. Some kids. But there are families that you see that they care for each one.

**I: Ok there are families that cares?**

R: yeah.

**I: they watch over their little siblings?**

R: yeah. Their little siblings. If the parents are gone then the older sibling will feed them and bath them.

**I: hm. Good. Can you tell me where the people in this community take their information from and that they understand them about foods that have nutrition I them and health?**

R: the people in this community took their information about foods from the lessons in the school about health, teach the kids, and the kids can go home to their mothers and fathers and tell them what their teachers thought them. But the thing I remembered before like last year, there was one program on radio, they usually thought us about food that are good and that will make you healthier. Today I haven’t heard about it.

**I: hm… why do the people in this community trust the place they get their information from?**

R: from my own knowledge, when the program comes from anticultural, they trust it because it’s a place where they move forward and knows the importance of the foods. when the programs come from the ministry of health, they will know because it’s a place where they learn about health. The kids in this community, when they come from the school, they will know because their teachers in their school don’t just learn about math and science but learn also about health.

**I: good. From how you see it. Where do you think those information’s should go for everyone to easily see it and hear about it?**

R: the way I see it, I think the information should go to the radios.

**I: radios?**

R: yeah, it should be out in the radios, but the only problem is, not everyone has radios.

**I: everyone… yeah.**

R: there are few that has radios, but when you work with the local government, there are some that do they roles, and there are that doesn’t. I believe that we give it to the churches.

**I: the church?**

R: yeah. And the church preachers will take responsibility and teach the church members, and at the church, I think it will be easy, not just teach them the words of God but to teach them about life. Keep their body healthy.

**I: hm. Good. What kind of devices do people usually use?**

R: at this moment there is GP radio.

**I: radio?**

R: there is phones.

**I: there are phones?**

R: yeah.

**I: Ok. Good. Thank you for answering all the questions. We are done with all my questions. But is there something you want to talk about?**

R: understanding against communication between these islands and Majuro. About phones. There are times phones are hard to reach Majuro, if you call there are no signal, no service and you will go to a spot to reach, it might be from here to where you guys are at, at Ine… like spot locations you can reach Majuro, for the kids that are in good size, they usually go there easily…

**I: there are antennas in each community to easily reach each one.**

R: I was telling stories to one worker at NTA, he said the location where the antenna is at it’s good, but it can’t catch all signals. we can just find a better place to catch signals. like the GP radio is not useful for some people, because it’s expensive and they can’t afford it.

**I: only some has… Ok. Good. Are we good?**

R: yeah.

**I: Ok. Thanks a lot.**

R: your welcome.
