# Supplementary material for: Conceptualizing multi-level determinants of infant and young child nutrition in the Republic of Marshall Islands–a socio-ecological perspective
Source: PLOS Glob Public Health. 2022 Dec 19;2(12):e0001343. doi: 10.1371/journal.pgph.0001343 (PMC10022247; doi:10.1371/journal.pgph.0001343)
Supplement: S1 Data — (ZIP) [file pgph.0001343.s001.zip › RMI Supp Data/Interviews data/I56R_CL_Arno_Sept17_Balton.docx]

**Interview Code: I56R**

**Interview Type and Interviewee: IDI_ CL**

**Interview Date: September 17**

**Location: Arno**

**Interviewer: Balton**

**Transcriber: shante**

**I: is it ok if we do our interview? I’m going to put it here so that it can be closer. The question says, can you tell me what is your role as a leader in this community?**

R: my role is to know the ways of this community on how I give lessons to the students, not just as a head teacher but as a teacher in the classroom in this school.

**I: good. Thank you. these other questions that I’m going to ask you is about community, on how you look around and see, can you tell me who stays in this community and who do you usually work with?**

R: Ok. thank you. people I usually work with and stand behind this school and they sleep at my house especially this community. when it comes to the activities in this school, like cleaning up and having meetings on finding the better cause for this school against the students. Everyone in this community comes together. Not just the parents and the PTA staffs but the people in this community, for us to talk about the better and useful things for our kids, for us to be together and talk and to know and the future of this school. Like the people that usually, the PTA members plus the community and the preachers in the church, the businessman plus the chiefs and the leaders in this community.

**I: hm. Good. The chiefs and leaders help you with your work?**

R: yes. Our culture helped us a lot in the needs of our school to lift up our level of knowledge in this school. For example, in the school in this community, it has rules, one student should not be walking around after it’s 7 in the evening. After 7 in the evening, they should be in their houses and to study. If they will see the student, then the mother and father, the parents of the student, they will get some word of advice to let their kid stay inside their house and learn his studies.

**I: and the leaders help in the same situation?**

R: yes.

**I: they help enforce them?**

R: yes. They enforce the rule so that it can be still be alive. Plus the policeman, let’s say the policeman of the government…

**I: Oh, not the chief leaders?**

R: not just the chief leaders but the government.

**I: wow. That’s very good. Other than the Marshallese that work with you, is there any foreigners that helps you with your duties?**

R: yes. There is one world teach volunteer that works in this school.

**I: with the other schools, do you think there are other foreigners working there?**

R: yes. There are 2 school here and there are other foreigners helping there.

**I: now do you know if the churches are helping you with your duties? The preachers in the church?**

R: yes. They help us teach the students, on how they understand. the students also learn from the church when they are in Sunday schools and they teach them about the bible. On how they learn the bible it also helps them reading and writing. The church helps a lot with the school.

**I: good. From your own self, can you tell me what are the good things in this community? is there anything that shows that this community is good? What are the good things you see in this community?**

R: yes. There are good things in this community because when it comes to, let’s say… brokuk (means everyone stands together and help each other.) everyone in this community brokuk and they think the same on getting the achievements for the school.

**I: that’s very good.**

R: work together in this community.

**I: is there any, from your own point of view as a leader, head teacher and also a teacher, is there any difficulties you see about food in this school or in this community that you see that you will need help, and need help from other places to address your difficulties?**

R: yes. Food right?

**I: yes.**

R: first we need our clutch program to come back for our students because I believe that they can have more nutrition in their foods if the clutch program comes back. The issue is because when there is no lunch food in the schools, it will make the students not come interest with their school studies and it will also affect their health and knowledge.

**I: Ok. good. Other than food helping the school, can it also go to the community?**

R: yes. it can also go to each family for them to… not for the school only put to the student house, the parent house so that they can have nutritious in their foods. and the foods that they give to the students to eat before they go to school, they can eat it the morning, and evening. And I think that there should be help for this community if they can, yes. when it can come to the community.

**I: Hm good. Thank you. it’s good because on how you answer like this other than just say yes no yes no. these are the type of answers that the people I work for wants. Because when you tell stories, in their heads they imagine what your explaining what you need, they know. And thank you for answering like that. Those are the kinds of answers we are looking for. Answers that has lots of information from. our next question is about the health of this community, about illnesses and other things. Can you explain what kinds of illness children in this community usually get?**

R: kinds illness are fever and runny nose, like how they say it… flu right?

**I: hm hmm.**

R: yeah. and headaches, stomach aches plus toothache, kids here usually have a toothache. I think this situation should lower down and if it would make one hospital for this community

**I: hospital?**

R: yes, because when we go to the house of the doctor in the centre he is gone, not gone but they ran out of medicines. when the doctor goes to Majuro then no one is there to work. Some people have less incomes to go to Majuro hospital to get medicines.

**I: if the child was like having flu and fever, headache, stomach ache, how serious are these illnesses, are they serious or do you as a teacher/headteacher can those illness affect their school works and you would say, ‘hey you can go home because you are sick’…**

R: yeah, because when they have stomach ache and headache, now they will ask, I will bring them home so that they can go home and rest and laydown all that other stuffs. When they have medicines, they will drink and then rest so that the child wouldn’t get sick anymore.

**I: I’ve seen the hospital here, but is there, one of the illness you gave me about toothache, Is there any dentist here?**

R: good. Thank you. well, when it comes to toothache there is no, the hospital here doesn’t do any dentals. If one student tooth hurts, they usually give them Tylenol to prevent them to have feel the pain, other than going to Majuro and seek the dental than they won’t. because the hospital here doesn’t do dental clinics.

**I: wow. So, you mean all the kids will be in pain?**

R: I have a child that goes to school here and when they have toothache, my wife and child will go to Majuro…

**I: is that the only way?**

R: that’s the only way.

**I: there’s no other place to help here?**

R: other than, what? Drink the Tylenol to kill the pain, I think that’s the only way they do it here. Drink Tylenol.

**I: the way you see it, as a leader that watch over the school that has lots of kids, there should have been a nurse in this school, like for some other school’s they have their own nurse, she’s a teacher and also a nurse.**

R: Oh… yes. well, that is very good. And it’s better if someone comes. Not just a doctor but also a teacher so that she can teach the children about how to take care of their teeth when they are in school. Yes.

**I: it’ll be something that would really be helpful. Now the children can’t go to the hospital and can’t skip school because the nurse can help them and send them back to class.**

R: yes. hm hmm.

**I: our second question is, can you what the people in this community usually look for to cure their children? For example, from the traditional healers, the doctors or the nurses? If one sickness effects your students, where would they bring the children to? The traditional healers or to the hospital or whichever? Can you tell me a little story about it?**

R: Ok. thank you. when it comes to illnesses like stomach ache… and when they have cuts on their bodies, then they go to the hospital. Other than… break. Like if a child fell down and broke his arms, one Marshallese person comes and massage him and make him medicines, I saw one kid that had a cut under his feet, from the bottles or… well the doctors usually make the wound and sew it. Comes to stomach ache, the visit the hospital.

**I: Oh. so, it depends on what kind of illnesses are there. It will show who the kids go to first,**

R: yes.

**I: hm.**

R: well, the truth is, we usually go to the hospital…

**I: oh. the hospital first?**

R: yes, and after that. For example, when the child has a broken leg, we will bring him to the hospital and the doctor will see what’s wrong with him and other then, they will give us the cement and put cement on. But other then that the person will come and massage the kid and straighten the bone when he is in the hospital.

**I: the next question is, can you describe what kind of illnesses that comes from the food that affects the children in this community? like what illnesses that cause from foods? what illnesses that usually get from the children foods? that you’ve seen. Like your kids from school, from what they eat, what kind of illnesses that comes from them? It may not have been showed yet but, when you see it, it can show, like for example, less nutrition, if they don’t eat enough nutrition then these illnesses might affect them because they don’t have enough nutrition and they will get weak, this is just an example.**

R: hmm. Well, the student bodies are slowly growing and wen there is not enough nutrition in their foods it affects them as they grow up, their age can be grown but their height isn’t right for their age.

**I: hm.**

R: plus, when not enough nutrition, some kids can always get sick because they are so…

**I: when they eat chip and not eat real food but eat chips and that’ll make them have less nutrition. Hmm. what kind of food is not good for the children that will affect the health of the children.**

R: foods like candies and foods like cookies and like…

**I: the chips?**

R: yeah, the chips.

**I: the foods that has grease in them?**

R: yes. those are the food that the kids these days like them. Other than them eating real food, they eat junk foods. like chips and drink soda. And that’s why it makes some kids to have less nutrition and make them not grow healthier.

**I: can you tell me what types of food that makes a child’s body healthy?**

R: well there’s no question, local foods. like fish, eat fish, eat breadfruits, drink coconut juice, eat planted foods from the stores like apples and orange.

**I: is there any other illnesses that can affect the child from the food that are not planted? Planted food like for example, is there any illnesses like toothache? What will the child eat to make his mouth painful or is there any food that can make his teeth strong? Just from your understanding and experience.**

R: for toothache?

**I: I’m going to give you another one. Is there any other food than makes the children get sick? Or is there any other kind food that prevents the illness?**

R: yes. when it comes to toothache, some kids eat lots of lollipop and it will make them have cavities. But I believe if they eat a lot of coconut (laughing while talking) their teeth will get stronger. It won’t destroy their teeth, eat pandanus and stuffs…

**I: have you heard about a program, it’s usually at America, I don’t know the law for the Marshallese, education, do they teach the school about the wash? It stands for WASH HAND SANITATION HYGENE. The work of it, I don’t know… long time ago people use to make it. Like in the morning the students would wash their hands before they go to school and brush their teeth and soap their face every morning. But I remember when I was young, the thing is, the reason why for that was they wanted their kids to wash their hands and wash their face from when they were young until they grow up. I don’t know if this implements in the school, but do you think this will be good for this school if there was?**

R: yes. yes. thank you. this, when I was in Head Start, I also took the same program, like I said, we have our own pocket and we take it every morning and wash our face and brush our teeth, and I believe this will very much help if it comes back to the schools because some students, when they go home, they won’t remember to wash their hands because when they say eat, they will rush over and touch the foods other than go washing their hands first. But I believe if this was happening in the school and the students, if the student was going to eat, they won’t remember to wash their hands before they go eat.

**I: you think this program is good?**

R: yes. the program is very good.

**I: do you think it’s a thing that education can start a new start of it? Do you think the school need this and also make their restroom… will it, on how the students takes, you like the ministry of education, help each school on Marshall especially the elementary where children starting to grow. For the islands, it’ll need some requirements for them to give you guys this programs on how they make their restroom better. For the children to start making this.**

R: we need to make this program because the student can… so that the school can have a restroom because, how can we say it? For the student, if he is done using the restroom he can come wash his hands and…

**I: they way you see it, can education help make the school restroom better? If they were to renew it, will it be easier for the school to use the program on WASH for the kids? Start training them from when they are young. To wash their hands and…**

R: yes.

**I: clean themselves?**

R: hm…

**I: it will help us a lot?**

R: yes. it will very much help.

**I: our second question number 7, it comes from what kind of food in this community. can you tell me how people in this community have food in a day? do you think it’s hard for people to find food for them or?**

R: in this community?

**I: yes. here.**

R: yes.

**I: is hard to find food?**

R: it’s hard because, when it comes to planting, there’s no place for planting, not just few but when the animals crawl around like pigs and other animals, it’s hard for people to plant because, for example, if I made a place for planting and the animals comes and destroy the food in the where I planted them, that’s an issue because people in this community have hard times in finding food for them, other then buying food in the stores like mackerel and rice and flour, it’s a issue for them to take food for them like banana and things like that because they have no where to plant. That’s the issue.

**I: in your area. What kind of foods you have there?**

R: near my house?

**I: yes.**

R: well near my house where I live, there are bananas, breadfruit, papaya, pumpkins, pandanus and coconut juice. There are no other foods.

**I: if there was help for helping you, what kind of planting you would want to plant.?**

R: if there was help on making a place for planting, it would have been easy for me to plant taro, potato, foods that we bring and mix with our other plants like cabbage, and that is why we have issues and need place so that we can have others to plant in their homes.

**I: the things that you plants. If they have things growing in them do you take them and sell them to make money?**

R: at this moment I haven’t. the bananas, when the banana tree has a banana growing on it we will wait for it to turn yellow and eat them.

**I: not enough to sell them?**

R: yes. not enough. It’s only enough to eat it.

**I: but if they were plenty and you sold them, what would you have done to sell it?**

R: well, if it was enough and I was selling the food and there was money, I would use the money to buy what we need for our family. Our needs that are in Majuro, like for example we will go buy it for here.

**I: hm. What the issues and planting in this community? is there any issues on how you plant near your house? Do you need permission from people? Is it easy for you to make a planting place? If you had all the tools and all the things, will it be easy for you to build a planting place?**

**I: no issues?**

R: hm… the only issue would be the tools for working.

**I: some foods, like breadfruits and pandanus, there are times to make them grow? The pandanus have a different time to grow from breadfruit? If these foods weren’t available, are there other foods they will eat if these foods aren’t available? Like where can they find food other then the planted foods?**

R: well, around here they get food from the stores like rice and flour when there is no planted food available, but when there is breadfruit, like lots of them, then we will make a breadfruit paste because it will last long.

**I: that’s very good. What kind of animals are in this community?**

R: the animals that are a lot here are pigs, chickens and dogs.

**I: hmm…**

R: like those are the animals that are a lot here. Other than dogs and the animals from the other houses are pigs and chickens.

**I: pigs and chicken.**

R: those are the animals that we usually tap them for us to eat them.

**I: are there people that have pet pigs? Like On Majuro, you cant get a pig for a pet if you don’t have a fence for it.**

R: oh…

**I: and here is it like that?**

R: In this community I don’t think not yet. We have had issues, there’s plenty people that owns a lot a pigs that crawls around the area, there hasn’t been any issue on having pigs for pets.

**I: there hasn’t been any issues on people having pigs as pets?**

R: no.

**I: does this also apply for chickens?**

R: what?

**I: is it free for people to tap the animals like pigs?**

R: hm…

**I: is there any fence for chickens and pigs? Is it required?**

R: yes. here before, some years ago there was one rule that pigs aren’t allowed to crawl wherever because they will destroy our areas and especially the plants. And few years later, people let go of their animals and it was like they didn’t really enforce the rule but this, hear there is rule for the people but they don’t enforce the rule but they can let go, sometimes people doesn’t want to keep the animals in the fence because when they put them in the fence, and when they don’t have enough food to eat, here animals eat coconut, they don’t feel good when they are in the fence, now the owner will let the animals go because they will get sick.

**I: 0k. good. thank you. this last question about food. who in the family choose the foods for the family to eat? like who mostly choose the foods for the family to eat and fills up food for the family?**

R: well, here the man is the one that choose the foods because he is the one that brings food for the family for the wife to cook it and make food.

**I: who choose to food for the child to eat?**

R: the mother.

**I: the father brings the food and the mother chooses what to eat for the kids.**

R: Oh, that’s it. The mother is the one that gets the foods ready to feed the family. The father and mother jobs is bring food and the mother cooks it and let the children eats it.

**I: hm… we will now talk about water and cleaning. Can you explain how they get things for water? Or things to store the water? Where does your drinking water and bathing and cooking water comes from?**

R: here we usually store our water in the water catchments and bantoon. Sometimes water wells. Sometimes when the bantoon is empty they will have to use the water wells to shower and use them for cleaning.

**I: is there things to clean the bantoons? The bantoons for drinking? Is there things you clean the bantoons with?**

R: around here, we throw away the water, drain it out and clean inside. That’s the only way we do it other then that nothing. Nothing to clean it with. If it’s dirty inside, we will pour out the water and clean inside it.

**I: clean it and rain will fill it up?**

R: if it rains it will fill up.

**I: the water, do they boil? Clean? Boil or put them in a filter? What do you guys do? Boil it or?**

R: we drink it. Other then the kids, we boil their waters, other then the adults we just drink it straight from the bantoon.

**I: is there a lot of wells in this community?**

R: in this community there are few water wells in some houses. When they dig holes for wells, it’s salty.

**I: we will now come to washing hands, can you tell me how people in this community wash their hands? How do they? Can you tell me when and what makes others to wash their hands?**

R: the time when people usually wash their hands is when they are going to eat and after they eat. and after they use the restrooms they wash their hands. And after they do their chores.

**I: in this school. Is there any lessons on washing their hands? Now a days? Is there any lesson about washing hands for the students?**

R: yes. other then just giving lessons to the students we also give them advice and remind them to wash their hands before they eat. and at this moment, there is one clutch program that the community Is making, and before they feed them, the teacher will bring out the things to wash their hands and each parent. we have groups, one groups will bring things to wash their hands. Before the student eats and before the person serves the foods, the **student needs to wash their hands before they eat and go to the line. Who watches them, is the teachers and the lunch program committee. There is one committee that comes here to the school and look at the food that they make. And if the student goes out and play and they tell them to line, he will run to the line and the people watching will stop the student to go wash their hands first. There is always someone who watches them.**

**I: this program is good. this is the first time for me to hear about this program. I don’t know if they do it in the other schools, but I just found out.**

R: all the school here they do it.

**I: ine too? Do they make it on Majuro and on the other islands?**

R: the students usually eat at majuro but I don’t know. But the budget comes from each local government. The local government in the marshall they separated and give it to the school in the marshall islands. Some students they come in the morning and go home in the afternoon and eat but there is no food at their house. Because I don’t understand, some family they can have not enough food. the kid can come back to school but haven’t have lunch, when the child is in the classroom, you will look at him and he is sleeping because he is hungry. He didn’t eat. and I think that’s the reason why they made the program is to help the students.

**I: wow. That’s a good story. Thank you for the information that your sharing. They are new to me. I think this will be good for them to learn these lessons. Do you think there’s a difference between washing your hands with water and washing your hands with soap and water?**

R: yes. when it comes to washing with water only, it won’t be really clean but when we use soap, wash it soap, the child hands will be clean before they eat with it.

**I: what will prevent people from washing their hands with soap? What prevents kids and grown up to just wash their hands and just go but forgets to wash their hands with soap?**

R: sometimes there can be no soap to soap their hands. Sometimes, if it was a kid and he was hunger and thirsty, he can just go dip his hands in the water and go to…

**I: in a hurry?**

R: yes. in a hurry. But sometimes there is no soap and that can be a problem. Lets just say there is no soap.

**I: good. thank you. your good in giving me the information. Can you tell me what kind of toilets are there in this community?**

R: place for the restrooms?

**I: hmm**

R: they use the bathroom that uses…

**I: like a bucket?**

R: yeah. toilet paper or a bucket to flush the toilet.

**I: how many toilets do they use?**

R: just one.

**I: boys and girls share?**

R: yes.

**I: do they have a place to wash their hands in the bathroom?**

R: they don’t. other then, after they are done using the bathroom they will go to the tank and wash their hands.

**I: from your own thoughts, what kind of bathroom is accurate for children to use in this school and in this community?**

R: I think the bathroom that will be good for them if the boys can have separate bathrooms from the girls. And they can use the flush.

**I: flush?**

R: hm… and a bathroom that they make in other school. Like for ine, a construction came from Ine and build the bathroom and it’s nice. It’s cement.

**I: oh, other than plywood?**

R: yes.

**I: is it also important for them to have a place for them to wash their hands?**

R: yes. there should be a sink to wash their hands and water, they should bring one more bantoon because when they are done using the restroom they can wash their hands after. It can be just outside or in the bathroom.

**I: do you think this will help the kids and their health?**

R: yes. it will help a lot.

**I: if the bathroom that you say it’s good comes, will there be a difference between the bathroom that you want and the bathroom that you use at the moment for the kids? Will there be a difference between them?**

R: yes. the bathroom now is, we can say it’s good but in the other hand it’s not ready for the students because they flush it by themselves and sometimes the students won’t clean it after they use it. But if the new bathroom comes it will be good for the students to use because they can just press the button and it flush.

**I: do you think that kind of bathroom is good for the students? Do you think our leaders and government should take responsibility in this?**

R: yes

**I: is it important?**

R: yes. important for the government and the tutor for one island to take responsibility and make it possible for them to make it in this school.

**I: hm. In some communities, we have heard that people defecate on the lagoons. We want to know why they defecate on the lagoon? what makes them to use the lagoon?**

R: because the people don’t have bathrooms.

**I: what can help prevents them…**

R: every house needs bathroom. Some families don’t have enough money to make a bathroom.

**I: the issues on making a bathroom is not enough money?**

R: not enough money.

**I: and what else? Is there issues on if like the guy in the other house wants to make a bathroom, do he need to ask permission from whoever? like from your own home, do you need the leaders to sign papers or the chiefs? Or they can just make it?**

R: yes.

**I: is there any issue for someone to make their own bathroom? On Majuro for example, they won’t build until, for the bathroom to know how to flush, they need EPA and lot more workers to make it. Here is different.**

R: well, here we go to the community leader.

**I: do you think it’s easy for you to prevent doing it?**

R: well It won’t really because it’s something important and it will be ok with him. other than, if we don’t let him know he can…

**I: it can prevent that… but you think this will help the community?**

R: no question about it because this will help a lot the community and will be clean.

**I: that’s the only issue? The truth is there’s no issue but…**

R: yes. the truth is there is no issue but…

**I: only need materials to make them. If the government or which ever place helps, the problem will easily be gone for people to defecate on the lagoon?**

R: yes that’s the only difficulty, we need materials to make them.

**I: Okay thank you… You know where children plays around and they would defecate around. How are they dispose of? Like they would play under a breadfruit then they would just defecate wherever they’re standing at, how are they disposed of?**

R: Well around these parts they would use shuffle and shuffle it and throw it at the lagoon. (Laughs)

**I: But what if they’re defecating the lagoon?**

R: Well the lagoon is right next to it so why not just bury it.

**I: It’s good that your taking responsibility here in this school. Where do children usually play in the school or where in the community do you usually see them play at?**

R: Well here in this community where they usually play at is on the road also at the lagoon. Let’s just say that they usually play at the lagoon because they would play on the sand and swim. But if they’re playing baseball and sports like that they would play on the road and areas that are wide enough for them to play in.

**I: Is there any animals in the areas where the children are playing in?**

R: Yes. Pigs and other kinds of animals.

**I: can you explain to me about the playground the children play’s in that you see and that will be better for the children?**

R: the place for basketball, the things for the playgrounds for it to be good and so that no animals can go there and if they can make a fence so that the animal won’t go in the playground and defecate in the playground. Plus, if they make a playground like basketball court and volleyball well it will be nice for the kids.

**I: do you think there’s any difficulties to keeping the playing areas clean for children, during this time what are the things that we can do so that the playing areas for children can stay clean, is there anything that is not keeping the area clean?**

R: … Yes. Animals that would roam around and defecate the playing area where children would usually be. Plus the grasses would grow taller which would stop the children from playing on that area again.

**I: Is there any ways we can stop these things?**

R: Yes. If there’s tool we could use to clean it up well we would need cleaning tools.

**I: What about the animals?**

R: The animals we should keep them in a fence so that they won’t roam around.

**I: To end our question on cleaning, could you explain ways to prevent diseases? Like what can stop the diseases from spreading?**

R: Preventing diseases from spreading…

**I: Like can you view it as like for example is there any feces that would make diseases, is there anything we can do to prevent the diseases from spreading? What is spreading diseases, like trash or feces that are in various places, does these things bring diseases?**

R: Yes.

**I: What can you do to prevent the spread of disease, what can we do about it for the community?**

R: Hmm…

**I: Let’s say we know these animals would roam around and drop feces and the children are there playing, is there anything we can do so that the diseases that are spreading from these so that it won’t happened? What can we do to stop the things that spread diseases?**

R: What can we make? Like make a playing area where the children can only play in? Teach the children so that won’t forget to clean their hands before they eat.

**I: What about the areas? They should be cleaned?**

R: Yes. Clean the area like clean the areas before the children play and make sure that nothing would get them sick.

**I: Like cleaning the area will prevent the spread of diseases?**

R: Yea, the thing we should do is clean. Clean, like keep cleaning so that the community won’t be dirty.

**I: Yes. Now we will ask about the responsibilities of the mother’s and father’s and grandparents. Could you describe the care of children throughout the day in your community? Like who is mainly the one responsible for child care?**

R: Well here in this community it’s usually the mother and father or mother and grandmother would be the responsible for child care.

**I: Mothers and grandmothers.**

R: Hmmm. The women’s and the old ladies.

**I: What makes a good mother or grandmother on child care? What do they do so that it shows that they’re good at responsible with child care?**

R: on how we look at the child’s appearance.

**I: yes. the appearance. Shows that the mother and grandmother are clean.**

R: yes, because if we see that the child is clean, then we know that the mother is clean too

**I: so, what if it applies to the father? What do the fathers do to make the nice?**

R: on how he is bad?

**I: no, like what makes a father good?**

R: oh. for a child, what can we say? For a child to…

**I: like let’s say what are the responsibility for the fathers to the family?**

R: like support the family**,**

**I: with foods and those stuffs?**

R: hm…

**I: when you see a child that is not hunger and don’t need things shows that the father provides for their need. And their mother bath them and feed them and cook for them…**

R: some fathers watch their children, when he doesn’t have enough money to provide the needs of his children, this is also a issue to prevent a child to, but there is no question about it, when our children have what they need and eat the food they eat shows that the father has money and can provide for his children. When it comes to foods, he buys nutritious food for his children.

**I: can you tell me the role of the grandparents on taking care the children?**

R: on how I grew up and lived with one old lady, her responsibilities are teaching me manners, on how we grow up, she teaches us the manners and ways to deal with people and teach us what to do and what not to do. Like that.

**I: are there any difference on how grandparents and parents teach their kids? Same kind or different kind?**

R: we say it’s the same.

**I: they are like extra parent’s?**

R: giggles. Yeah.

**I: is there any help children see from they’re family in this community or the people next door or their neighbours that are not related but just live next to them? Do the people have raise the child in this community?**

R: here. Yes. some like for example, uncles, about caregiving, I can say, if my sister had a child I would say oh let me help you, or I would say I’m going to take the child to adopt him.

**I: meaning it’s easy for everyone to take care of each one and to help with care giving**,

R: yes.

**I: hmm. good. when it comes to information about nutrition and health for the children and the community, where is the best way to get the information that we trust get it from? the information that you trust**.

R: about what?

**I: caregiver and illness, any kind information on how to take care of your family. where do you go to get information?**

R: well, the hospital.

**I: yes.**

R: for me, I say hospital because when we go to the doctor, they said oh, you need to this and that.

**I: hm…**

R: and… where can I say…

**I: radio?**

R: yes, the radios, on how we listen to the programs that comes out from the radios.

**I: where is it easy that you are here in Arno Ine, you can go to what hospital? The hospital at Majuro? It’s far for you to go, other then the hospital, where do you think it’s easier for you to get the information?**

R: with the elders.

**I: the elders?**

R: yes. with the grandfathers and grandmothers. Or the elders that live here in this community that they have information about it. Because they have… yes, in this community.

**I: hospital? Radio?**

R: we can get information from the grandparents. Can it be?

**I: yes.**

R: mother and father.

**I: and which from these 4? Mother and father? Radio and hospital? Where is it easier among these? Where do you usually?**

R: well, with the grandparents

**I: that’s what is closer?**

R: yes. they are closer. Grandparents, because they are the ones who show their problems, if they have problems, they will say, let’s go the hospital.

**I: is there anything else you would want to know about watching the kids or take care them or anything else that you wanted to know but we didn’t talk about it in our interview. Is there anything I didn’t say or ask question about it**

R: I think it’s good.

**I: everything is good?**

R: hm…

**I: there’s a lot of us that came and one of them was the chief nurse from the hospital of Majuro, with us there are 3 ladies that came from the united nation from the UNICEFF. So that they can learn about the food that kids like them. We are staying at Ine. they will answer you with all the questions you will need and there are there, we are at Ine. If there are any other questions we will be at Ine. If you need, the next following week on Monday, we will now go home to our love ones. Arno is the place we are at, if you want to ask more question then the ladies are there to answer the community, but other then this, thank you very much, thank you for giving me your precious time to help with our work. For the school that you take care of, I want to thank for that, I know all of this work are not easy, but the island is blessed to have you to take responsibilities on saving the school I the future, other then that, thank you very much.**

R: Ok. thank you very much.

**I: wow, all your information was very good. (both giggles)**
