# Supplementary material for: Conceptualizing multi-level determinants of infant and young child nutrition in the Republic of Marshall Islands–a socio-ecological perspective
Source: PLOS Glob Public Health. 2022 Dec 19;2(12):e0001343. doi: 10.1371/journal.pgph.0001343 (PMC10022247; doi:10.1371/journal.pgph.0001343)
Supplement: S1 Data — (ZIP) [file pgph.0001343.s001.zip › RMI Supp Data/Interviews data/I58R_IDI_HW_Arno_Sep 17_Libon.docx]

Interview Code: I58R

Interview Type: IDI-HW

Interviewee: LIBON

Interview Date: SEP.17.2018

Location: ARNO

Interviewer: LIBON

Transcriber: LIBON

**I: Okay. Would you like to participate in this survey?**

R: Yes 100 %. This is very important.

**I: Okay. Thank you for giving me this opportunity to talk with each other. The information we will learn from you will help us find ways to improve the health of the women and the children especially the sanitation of our islands. And to start can you tell me about your job in the health center?**

R: My duty in the health department, I’m one of those doctor or clinic who is separated in one of the outer islands. Like all the doctors who are higher and also my title was a nurse or practical nurse. And after training on Ebeye, Kwajalein for over 18 years, I became an health assistant.

**I: Okay good. What are the biggest challenges in preventing children from having a healthy life and having a healthy diet?**

R: The difficulties in having healthy life in a child is number 1. The price of food is high, number 2. Not enough money to buy food.

**I: Are there any other reasons on preventing children from living healthy and having healthy diet?**

R: Yes. Some women or young lady don’t have enough food to feed their children and sometimes they don’t have enough breast milk.

**I: Why is it that sometimes they don’t have enough breast milk in their breast?**

R: There are a lot of different solutions that shows why there are not enough breast milk in their breast. Because not, some don’t have husbands to provide for them and care for them.

**I: Okay**.

R: And the biggest thing in these islands is when a child doesn’t or don’t eat regularly and that is what causes diarrhea.

**I: Okay. With all the problems you are mentioning, are there ways we can prevent these from happening, for example; if there is not enough budget to buy the foods that are nutritious?**

R: Yes. The most important thing is that if we can make a farm. And let the man and woman make their own farm so they can get paid for their work. These are some the things that we need in our community.

**I: Yes. Now. Sorry. Now regarding the women that you mentioned that don’t have husbands to support them or provide for them, are there ways that we can also…**

R: Yes. One of the laws from the government is for all people to protect and help these people.

**I: Okay. Now when you say help them, can you describe in detail how we can help these kind of women (single mothers or mothers who don’t have husbands)?**

R: In our customs, when there is mercy with their family members and those who are close to them, they feed them according with our custom and the plate between houses doesn’t go away. Also we feel sorry for them and feed them.

**I: Okay.**

R: I would spend over $9,000.00 on credit from the stores just to help these kinds of people.

**I: So as one the certify nurse or health assistant here in this community, what would you do to make this hospital better?**

R: The biggest thing I would do is talk to the local government (mayor and local government) and the community because they are the one that who is responsible and maintain everything. As I am an foreigner to this island, I will do my best where I can but to talk to the local government and the community, they are the one who is responsible for the sanitation. And for maintenance, the local government is the one filling gaps where its needed.

**I: So, what do you need here or for this hospital?**

R: The things that I really need are, inverter, fridge to cool the medicines for the little ones, Clorox and things to clean the bathroom and for mixing. I also convert Clorox to cleaning wound. This is one of the things that I experienced.

**I: Now can you describe how you convert Clorox to?**

R: The way I convert Clorox to cleaning wound is by adding amoxicillin and lactated ringers (Plain LR) 1000cc. I take 10cc from the Clorox and mix it. And I would open the container for 2 hours for the Clorox to weakens and after 2 hours for the Clorox to weakens, then I clean the wound.

**I: Now when you clean it does it work?**

R: Yes. When you mix it them and clean it right then, the person’s feet dries up.

**I: Oh okay. Now if you had all the supplies that you need for your hospital, what kind of project would you like to do?**

R: If I had all of these stuffs, now it would be good for emergency, it would be clean for the people to use without getting sick and 3^rd^, when the people see us clean and stuff they will follow and clean their houses like I clean this house and this area. I don’t tell them to clean but when they see me sweating they say, “oh the doctor is cleaning, lets also.”

**I: So, you mean just by moving around, do action and do the stuff, then they participate.**

R: They participate. They see me tired and sweating and participate. These are some of the things that I’ve learned from traveling in the Marshalls. All these years moving from Lep, Namdrik, Rongrong and all these.

**I: Like you giving them examples like that?**

R: Yes

**I: It’s a good example for these people to reflect on themselves, so that other than seeing they have to know what to do.**

R: The people of Ellep, I would go to the ocean and then wheelbarrow small rocks and after that I would clean from the ocean side to the lagoon side. And I would surround the hospital with just small rocks only. And the people next door would see me do it and do it and the other people next door would start cleaning. And our ancestor would say “eke ene elin Ellep.”

**I: What’s it call?**

R: Eke ene elin Ellep.

**I: What does that mean?**

R: It means, when the people next door clean, the other people next door would want to clean as well.

**I: Yes.**

R: And these are some of the examples that I learned from Lae and Ellep.

**I: Oh okay. Now we will talk about the illness. I am also interested in illnesses that children suffer from. In your community, what illnesses would you say children under 2 years commonly suffer from?**

R: The usually get illnesses like coughing and running nose also diarrhea but some and sometimes they would get it because now the mothers knows to protect them and to teach them.

I: What about Marshallese illnesses?

R: Yes, it also comes from Marshallese illnesses.

I: Like what kind of illnesses are there?

R: The reason why a child would get diarrhea because the mother is breast feeding but is sleeping with the father. Give or when the women and the men’s germs are combined together, it goes straight to the breast milk and from the breast milk it goes to the baby’s brain and cause it to. And even though they take American medicines, it won’t go away until they will drink baby medicine (local baby medicine).

I: Baby medicine?

R: There is baby medicine.

I: Can you explain what you mean by baby medicine?

R: Baby medicine. It’s the old ladies that really knows are the one that do it because they feel sad because I tell them to do it.

I: So, for running nose and coughing, what causes running nose and coughing?

R: Some children when they are having running nose and coughing for a long time, it’s a sign that they have bacteria in their stool, worm infection or ameba.

I: Now bacteria come from what?

R: It comes from dirty hands and how the mother is sleeping with the father while breastfeeding. She’s doing it while the baby is too little.

**I: Can you tell me the risk of the illnesses that you’ve mentioned, like coughing, running nose and diarrhea? what are the risk?**

R: Whenever coughing and running nose occurs with a baby that is 2 weeks old, then that mean they have liquid in the lungs and that means they have pneumonia. This causes fever, and if the mother doesn’t really take good care of the fever and give him antibiotic, then the fever goes to the brain and cause meningitis. And it’ll be difficult for the baby to survive because the fever has gone to the brain and all airways are blocked (oxygen). That’s a risk for a child.

**I: So you mean it can cause brain infection?**

R: It can. It depends on whether you live or die.

**I: Die or live.**

R: If they happen to live then they end up mental or crippled.

**I: How can we prevent these illnesses?**

R: To prevent these illnesses, we usually give them IV and then give them ampicillin into the IV push when they’re in critical condition and are at risk every after 2 hours for 24 hours. I would give them IV push tab after or when it gets better or not I change it to after 4 hours according to their critical conditions. Secondly, I would use the radio and make one radio call receive to have Arata give treatments or prepare him to make a medivac or look for a way to have a medivac whether by sea or air.

**I: Okay. So what usually heals these illnesses in this community?**

R: They usually or sometimes it’s hard to survive from these illnesses if they’re critical. But if we get them fast then we can treat them with coughing medicines and stool medicines. They should also give them lots of water/fluids.

**I: Do they usually use marshallese medicines (local)?**

R: Yes. The is the biggest medicine they use is marshallese medicine but if it doesn’t work then they turn to the health assistant. When they bring the baby he/she is already sick. Day after or 5 days after, its really hard for the health assistant because the child is really sick.

**I: That means that other then bringing them to you, they go to the traditional first?**

R: Yes

**I: Now why didn’t they bring them to you, but they brought them to the traditional healer?**

R: Well now they bring to me as we’re speaking. When they come, they all come at once.

**I: What about in the beginning?**

R: In the beginning, they would do it themselves. This is when I wasn’t here.

**I: You can say they usually depended on marshallese medicines?**

R: Inna

**I: Okay. Good. Now can you explain what type of treatment people in your community seek for their children. for examples; traditional healers, doctors, nurses?**

R: From the doctors and nurse.

**I: Doctors and nurse?**

R: Yes

**I: Now why do they believe in the doctors and nurses?**

R: because of the foreign medicines that they believe in. because the get well with them.

**I: Okay. What about who do they first take the child to when they’re sick and why?**

R: They usually bring them to the health assistant I’s look at it and for instant regarding asthma, there are some kinds of illnesses that occur we call those sinus symptoms. When they have asthma and we listen to their chest we hear a whistling sound.

**I: So, what does that mean?**

R: Shortness of breath or asthmas. And when the sound of the chest change, its pneumonia, like when its cloudy, it’s the sound of thunder.

**I: Oh, okay. So do they use the healers or the marshallese medicines?**

R: They would usually go to the health assistant and use foreign medicine.

**I: Foreign medicine? Okay. So can you explain the difficulties that the people in this community faces to find treatments to the illnesses you mentioned before? If for instant, diarrhea, what are the difficulties that they’re facing in finding treatments for the illnesses such as diarrhea, running nose and coughing.**

R: sometimes the reason why its difficult is because of late arrivals of medicines but we treat them with oral rehydration, mix our own. The amount of teaspoon in sugar, how many table salt and how big the container you’re mixing if they have diarrhea.

**I: What about coughing and running nose?**

R: Coughing and running nose. If there’s no medicine for adult or no medicine for children, the buttons, I mix them and change them for children’s medicine.

**I: Mix it and change it to liquid?**

R: Yes. I can mix it with luau, add a little sugar to the liquid. Show it and explain the meaning and teach them. I make my own mixture and now I follow how many buttons in cut up. If four times, then I would cut it up into 4 pieces. And then I would bound it on a piece of paper, so it becomes powder. And then add it to the water that I’ve added sugar to it and stir it so the powder would dissolve.

**I: To dissolve?**

R: Yes. And then give the medicine.

**I: What are the difficulties you face in providing health service to your patients?**

R: Regarding transportation, a pick-up truck. There should be a pick- up truck to go far. You remember the time you guys went out. Well Mantel En an Jabo they’re under my jurisdiction.

**I: Oh, so all these villages and combined with this village?**

R: Three villages. I bet we could use pick-up truck for these places?

**I: Yes. Good. These are the answers that we are looking for in your answers. Thank you. Can you describe any illnesses associated with nutrition that affect children in you community?**

R: There are no illnesses that appears if the food has nutrients in them. When we eat them we’re stronger but the reason why we get sick is because they don’t wash their hands. Sometimes the mother would forget to wash their hands.

**I: That’s true. Now what kind of foods that makes a child body unhealthy and why?**

R: the reason why they’re unhealthy is when the mother is not protective of their diet. Most of the time she would just bring lolly and chips and start training the child by just eating sweets and chip. And that makes the child think of just eating chips and sweets because he’s so used to it. He cries for it every hour, in between hours and all hours he cries for it because.

**I: Because he just wants to eat sweets?**

R: Yes. And the mother would constantly find ways to feed him/her.

**I: What kind of foods that makes a child’s body healthy and why?**

R: The food that they usually feed the children are, the men go fishing, feed the fish and make soup.

**I: Okay. What else?**

R: They also give them papaya, sometimes they would cook rice pudding and feed them.

**I: Now what are the biggest challenges regarding the health of a pregnant woman during pregnancy and after pregnancy?**

R: At the time of pregnancy, some woman don’t like food. And when they wonder around, her thoughts are so cloudy because of her cravings. If she sees a coconut she would eat it but its dirty and if she wants to pick up anything to eat, it will give illness to the baby. The baby can also get ameba from the mother being inside her stomach. And when the baby comes out, already she’s sick.

I: What about after birth?

R: After birth she just want to lie down and breastfeed until 6 months. After then she would make soft food or bob juice to put in a bottle.

I: From our understanding, there are probably more than 70% of people in this island over the ages of 55 that are diagnose with diabetes. What are your thoughts about what’s causing people to have diabetes?

R: The biggest problems that we face today are people have diabetes genetically and secondly from the food that they eat and how we don’t watch their diet. Foods that are imported from other countries. And when there’s no more breadfruit or pandanas and things like that, they won’t eat. They would usually eat rice, bread, donut or pancake for breakfast and when they drink, they would usually drink sugary drinks.

**I: Its like they’re used to those food?**

R: They’re use to it. Now some people they don’t walk and exercise. Now when they come and check their glucose, I end up teaching them and tell them that we need to recruit a walking team. And whoever’s glucose drops down to 120 and 100 some I will give them $100.00. The plan hasn’t yet been confirmed.

**I: Not yet confirmed?**

R: Not yet confirm. Its like a game. So they would say “you’re the one who’s being late doctor.”

**I: So they’re ready**

R: they’re ready. So only few are exercising. And to prevent diabetes, there’s one, that bottle. My younger sister dreamt about it. If they drink too much of it, it’ll dry up the diabetic’s body.

**I: So what’s the name of the medicine?**

R: There’s no name for it. But you know the medicine that the diabetic drink, the metformin, well I call this medicine informin.

**I: What’s it made of?**

R: From the tree leaves. But it’s going to be hard for me to tell you because it’s my sister’s secret.

**I: Yes, I understand. But what can you do to prevent diabetes? What kind of activities can you do and what kind of food can you eat to prevent diabetes?**

R: I can eat the local foods that are fresh and then our fresh water that God has prepared for our body and other stuff like ni is also fresh.

**I: Yes.**

R: But the products that the Americans bring that we drink was made 8 years from now are like they were made today for the right time. And they write on them and say they will expire years from now.

**I: So you mean they mostly put poison inside them.**

R: And when I went to the water factory in Australia. Then I went to all the factories and studies from the. I also went to Japan.

**I: what other activities can you do?**

R: Picking up trash. There is another activity that just started, they pick up trash and we pay them.

**I: So, when they pick up trash, from where to where? Is there a…**

R: For instant we cleaned from the ocean to the lagoon side and the girl at that house paid us. For this house, I paid Youth because they were looking for budget. I paid $20.00. sometimes $50.00 from the ocean side to the lagoon side.

**I: Because when they clean, that’s a lot of moving around?**

R: A lot. And when I pay them to bring little rock for outside this house, it’s $1.00 per bag. So, when they bring 100 bags they get $100.00.

**I: Good. Other than just picking up trashing they’re also lifting stuff.**

R: Yes. Exercise just so that they sweat.

**I: Now I have few more questions related to woman’s health, but can you tell me about your experience with women who have anaemia?**

R: Women with anaemia. I hardly see this kind of illness. If there is, it’s usually in Majuro or Ebeye. But for me to diagnose someone with anaemia, the signs that I look for is I look at the sinus symptom. There are ways that I diagnose them.

**I: Now can you really put in detail how you diagnose or know?**

R: Oh the way I diagnose is that I open the eyes and then bring the flashlight and look in it. There are somethings at the end I see that shows a sign. When its shiny and white and there is no redness. And when you see that it’s white and shiny, that person has anaemia and weak.

**I: Do women with anaemia think that this is serious illness?**

R: Yes. When they come to me or see the doctors and ask why their vision is weak and then I give them the blood pill and the medicine that makes their body strong. And tell them to eat raw fish and things that can produce blood.

**I: Now what causes anaemia in girls in their youth and the pregnant ones?**

R: when they get the illness of bleeding, blood or periods. Some women who are on their period even though it’s time to stop but doesn’t it just keep on bleeding. Then it becomes dark and then causes a little infection in the uterus.

**I: And it also affects anaemia?**

R: Yes, because its bleeding.

**I: What if was a pregnant woman?**

R: Like that. Doesn’t know that she’s over due but some go and ask the doctor for antibiotic. And some doctor gives them medicine for worm infection or antibiotic. Now the medicine for the worm infection and the medicine for ameba causes miscarriage and cause the thing to stay inside the uterus and it won’t come out. And that’s what causing the bleeding until all the yolk sac is gone from the uterus.

**I: what are the risks of anemia?**

R: It is risky. The risk of anemia is all the immune system in a person’s body won’t be as strong. For example; kidney, liver, lungs are some of the organs that helps the heart to circulate blood around a person’s body. And when one of them shuts down, then the heart will weaken because some of the powerful organs that are the foundation. The kidney can also help the heart and all the other immune system. If one of the organs fails, then the heart will weaken until it

**I: Just stops.**

R: Yes.

**I: Now are there any advice given to women for preventing and treatment of anemia?**

R: Yes. Whenever they have this type of illness, they always go to the health assistant. And the health assistant would give them pill that can produce blood and explain to them that they should move around a lot, exercise and don’t just sit and lay down. Because when we lay down and sit, then the blood in the body will be weak. This will cause weak vision and weakens the body (feet and hands). And these are some of the sign.

**I: For anemia?**

R: Yes.

**I: Now we will talk about how we breastfeed in this community. can you explain how long after birth most women start breastfeeding in you community?**

R: Some women have different ways of life. Some breastfeed them, but they stop because they feed the baby food and give them to their grandmothers to look after them. But some would breastfeed until they’re over 5 years old. They usually would breastfeed but when they’re thinking about going back to school, they would give them to their grandmothers.

**I: What if after the woman has given birth, how long after birth and she breastfeed?**

R: At the time when there’s a sign, because at the time of birth, she has to give her breast. When the baby sucks the nipple its just water. So sometimes the mother would say, “oh this is just water, I don’t have any breast milk”. But that the vitamin that the baby needs first and that also produces breast milk. And keeps breastfeeding until the mother has breast milk. When the father goes out fishing and feed her fish, coconut meat, coconut milk has produces breast milk. And, when the eat fish and foods that have vitamins.

**I: So, the mother can say that the first milk is just water but then again, it’s the vitamin.**

R: It’s the vitamin. And it makes way to produce breast milk in the mother’s breast.

**I: Are there any other liquid that the mother gives to the baby other than the breast milk during the few weeks after birth?**

R: Now a days, people get sick from climate change. Our islands are islands with poisons. And some mothers don’t have breast milk in their breast. They’re dry. Even though they will eat fish and meat but no.

**I: That’s why they would give other things like.**

R: Yes. The real problem that’s happening here in our community in the Marshall Islands is the climate change. When the bigger countries test their weapons and then the wind blow it here and then they breath the air.

**I: Breath the poison.**

R: The reason for having malnutrition and diarrhea is because of the climate change.

**I: Now can you explain how the women in the community exclusively breastfeed?**

R: The women in the community would exclusively breastfeed they don’t stop until the children are all grown up and they stop. But from 6 months and on, they breastfeed and feed them or make then food.

**I: So, who usually advice them to just breastfeed?**

R: They usually get these information from the Public Health when they come here, from the health assistant. And as they are taking these information and really try for their children.

**I: So as an health assistant, what do you advice the mothers to do to just breastfeed?**

R: At the time when the baby is born, I go to their house and teach them how to give birth and teach the old ladies to operate the baby, teach her how to breastfeed and they follow it and do it whenever they must help give birth to a child.

**I: So as a woman who is exclusively breastfeeding, what do you advice them and why do you advice them to just breastfeed, what do you tell them?**

R: I would usually explain to them ways of breastfeeding and when she say she doesn’t have any breast milk, then I would tell her “if you just let your breast down and breastfeed your baby and just let them breastfeed because you say that’s just water, well that’s the vitamin”. If you just let him breastfeed because you’re just letting your breast down, the it produce then the regular breast milk. So this is just how they do it.

**I: And that it true.**

R: Yes

**I: Are there any other liquid other then the breast milk they give to the baby during the first 6 months?**

R: No. Just that.

**I: Just constantly breastfeeding. So, what are the challenges the mothers in this community faces to exclusively breastfeeding during the first 6 months?**

R: The challenges they face when they prepare the food late because they’re always breastfeeding the babies every after 2 hours if they’re hungry. And the food is late for them to eat. And one of the things I would tell the young men is to make sure there is always food every hour today because there is no ending to eating.

**I: Are there ways or advice for the mothers to just breastfeed during the first 6 months?**

R: Yes. We advice them to just breastfeed and let their breast down.

**I: We want to know how the people in this community eat. Can you explain in detail what most families usually eat and drink throughout the day?**

R: Some family when they wake up, they cook pancake, wheel donut, round donut and drink tea and coffee. These are the food they usually eat.

**I: Now how do they prepare the food?**

R: They fry them. Cook them in grease.

**I: Grease?**

R: Yes. And when there is no more they would just feed them rice.

**I: Is there a time when there is no more food?**

R: Yes. They look for way to gather coconut because survive with copra. If they don’t gather coconut there won’t be money for food. They prepare this week and next week.

**I: What about our seafood?**

R: They also go fishing for fish. Usually the men would go fishing and look for food. They go diving.

**I: So, if they don’t have money to buy canned meat, rice, flour then they turn to the foods that we have.**

R: Yes.

**I: Who in the family get served first and who next?**

R: The workers in the government, the people who gather coconut are the one who will have lots to eat. They have lots of food because they have lots of coconut.

**I: What about in your family, who do you served first, next and last?**

R: Everybody is the same.

**I: Everybody has the same amount. Are there differences in the food you serve to different family members?**

R: There is no difference. We eat at the same time. When they reach the house the children are they’re not hungry. We can bring the pandanus and put it here. And we can cook some donut or pancake and leave it here and make a gallon, teapot or cooler and prepare there so they can just come in and out all day, just eat all day.

**I: Are there any difference in the amount of food you serve to different family?**

R: Yes.

**I: Can you explain it?**

R: The amount of food you serve to families if it was a case of quarter leg, I would give out little to each family according to our culture. I would take some to this house, like 2 or 3 pieces to every house and its up to them to cook them.

**I: Now do some children receive more food then others?**

R: Everybody is the same because their mom or grandmother feeds them.

**I: Can you please explain how the family serve food to neighbors during meal times (for example children eating together separately from the family, meals eaten from the same plate by all family members)?**

R: The small children are usually fed by the grandmothers or mothers. But older kids are served according to how much they eat and if they’re not full they fill their plates up again according to the amount.

**I: According to the amount they eat.**

R: Yes.

**I: Okay and does the family serve food to their neighbors?**

R: Yes. Passing around plates between family never ends.

**I: Okay. Now I want to know how children in this community eats? Can you describe in detail what children under 2 years commonly eat throughout the day?**

R: They usually eat. Their mother would feed them all the foods that are. If not they would feed them rice and bread if there’s no food.

**I: What if they have food, what kind of food do they feed them?**

R: If they have food, its nutrition food. But they would usually bring mackerel, tuna and mix it with rice and feed them.

**I: How many times a day are meals eaten by children under 2 years?**

R: Sometimes they would feed them breakfast, lunch and dinner and sometimes 2 times a day.

**I: Do children usually eat snack in between meal time?**

R: Yes. Sometimes they don’t.

**I: If there is, do they eat.**

R: if there is they eat

**I: Almost all the time there’s non?**

R: Yes.

**I: Are children fed differently when they are sick (for example when child has diarrhea and why)**

R: There is a difference. Because when a child has diarrhea, they feed them but they’re always vomiting. Usually they would give him water. Also, every after 2 hours we remind them and teach them that they must feed them every after 2 hours little at a time.

**I: To help their stomach?**

R: Yes. There has to be something in their stomach.

**I: Are there any difference in feeding the boys from the girls under 2 years?**

R: There is no difference. They eat with their mother.

**I: Oh okay. Its the same**

R: It’s the same.

**I: Can you tell me what influenced the families to feed their children in this community? Why is it important to feed your child?**

R: So that the children don’t get sick and their brain would develop properly as they’re growing. And there are different ways to make sure that the brain function properly to know what they’re doing.

**I: We hear that some families eat only local foods while others eat processed foods. Could you explain what is kind of food do family usually eat in this community?**

R: If there is no local food then processed foods. Usually.

**I: When you say no local food, why is it there is no more local foods?**

R: The reason is because the people don’t plant. The community doesn’t plant. There is food, but they don’t rush to plant or its not enough. Because they don’t plant.

**I: What about pandanas and breadfruit?**

R: They mostly depend on process foods.

**I: Oh, they mostly depend on process foods?**

R: What makes it difficult to cook local foods? Why is it that sometimes its difficult for you to cook local foods?

**I: It’s not difficult but some people just don’t want to make food.**

R: Why is easy to cook local foods?

R: The reason why its easy for me to cook local foods is because I usually want to treat the children that I look after local foods. The children that are with me knows how to eat breadfruit and eat pandanas all day long till evening.

**I: What are the good things about local foods?**

R: The good thing about local food is its healthy and it doesn’t cause diabetes. It has vitamin. It has vitamin A. Its has all the vitamin from A to 12A.

**I: Are there anything bad about local food?**

R: Nothing. Nothing.

**I: What are the good things about processed foods?**

R: Because they’re used to it. They grew up eating them. It’s like they’re addicted to cigarette.

**I: Yes. And what are the bad things about processed foods?**

R: It causes diabetes and makes a person constipate irregularly (ben lojeen??). And the reason why some people get sick is they don’t have enough, they use the bathroom regularly and the urine can damage the kidney.

**I: Can you talk about the messages about breastfeeding and complementary feeding you give to mothers and others in the community?**

R: The foods that I tell the mothers to eat during breastfeeding, if its fish or coconut meat. If they eat fish with coconut meat, these things produce breast milk. And when the baby breastfeeds, she/he gets all the strength from her mother. The baby gets all the vitamins from the mother. If the mother doesn’t eat local foods, the baby will be malnourished. If she doesn’t eat for one day, the baby will be hungry.

**I: Yes.**

R: And if she doesn’t have any local food for 2 weeks, she will also until she is born. And if there’s not enough vitamin it will cause premature.

**I: Are there nutrition education you gave to the people in this community as part of health worker?**

R: Yes. All the food that I explain to them I also tell them the important of them. If for instant breadfruit, pandanas and the other foods. When the people eat all the time they have difficulty in bowel movements because its new to their stomach. When they go use the bathroom all the food goes to waste while new foods are digested. This is what prevent the people from getting sick.

**I: Were there any difficulties in teaching the people about the foods that are healthy?**

R: There are huge difficulties that happens because some people don’t plant. They don’t plant their own foods. Their elders prepared them foods but because they’re letting the pigs destroy them. The most important thing is if they will cage the pigs and cage the chicken.

**I: What specific ways can this nutrition communication be more effective?**

R: We must talk to the local government, they mayor and gentlemen that is responsible for this community. And then the mayor, chief and the worker and whoever that is involved tell the Senator(s). And its up to the Senators to bring up issues at the Chamber meetings to see what they can do for their people.

**I: Yes. Okay good. Because when there is connection with the Mayor and counsels then it would be easy to spread the things that we study about food. Foods that are healthy. Now we want to know abut the pregnant women in this community? Can you explain what kind of food do they usually eat during pregnancy?**

R: During the time they’re pregnant, some pregnant women gets morning sickness and they may not eat for 3 days or 2 days. And when they eat, a little then vomit. When they eat fish, they vomit. But they try as best as they can. They usually would drink water. If the morning sickness gets worst, they go to the health assistant. Then we would treat them with vitamins, iron-sulfate and vitamin A because those are for pregnant women and they should try their best to drink. If they drink all of these supplements, they’ll never be nauseated.

**I: The women who are pregnant, does their diet change when they’re pregnant?**

R: Yes

**I: What usually change their diet?**

R: Their diet doesn’t usually change but they’re way of thinking. If they want to eat something inappropriate its up to them. But some people don’t see them eat those things. But some would hide and eat.

**I: What influences women’s diet during pregnancy?**

R: When there’s not enough money, the people of the house cooks late because they don’t have money. The process of copra is slow. But now we’re always eating these days.

**I: What kind of food are women encouraged to eat and why to they say it?**

R: They would tell to eat pandanas, eat fish. So, the men would go and look for these things until they find them.

**I: Why do want them to eat these foods?**

R: Because of their cravings. Without even thinking and they follow how would you say it in marshallese? Like…

**I: They want what the baby wants?**

R: Yes. They just think about their stomach.

**I: The growth of the baby inside their stomach?**

R: Yes. Also, when the baby grows a little inside the mother’s stomach it changes and the mother’s life also changes.

**I: When it changes, the diet also changes.**

R: Yes.

**I: What kind of foods are women encouraged not to eat?**

R: Salt. This is what they usually tell them what to eat only salt. But for all the food they say its okay to eat all kind of foods.

**I: Who would encouraged or discouraged the women to eat those food during pregnancy?**

R: The Public Health would bring advice when they come here to this community and also the health assistant they would take part.

**I: What about the family member?**

R: The family also take part. Now these 3 village here, there hasn’t been any birth since 2015 until now.

**I: They only give birth in Majuro?**

R: No. I usually give them family planning.

**I: Oh, the program that they mentioned.**

R: They would listen because I would give them advice.

**I: Yes. Yes. Now are there supplements that the women take during pregnancy?**

R: No. just those.

**I: The pills for blood and?**

R: Pills for blood

**I: The vitamin?**

R: The vitamin to give strength to their body.

**I: Okay. What are the challenges.**

R: Tylenol when they are in pain.

**I: Are there difficulties that prevent the women from taking their supplements?**

R: Sometimes there’s no difficulties. But they’re the ones making things difficult for them and not wanting to move around and going to see the doctor.

**I: Are there time when the women would drink alcohol, smoke or use other drugs during pregnancy?**

R: Not always.

**I: Now can you describe what kind of food the women who is breastfeeding eat in this community?**

R: The foods that the women who is breastfeeding eats in a community are, they eat fish, they eat breadfruit, and these are what they usually eat. Eat coconut meat because coconut meat also produces milk.

**I: It also produces milk?**

R: Yes.

**I: What kind of food they tell the women who are breastfeeding. Sorry! Do women change their diet during breastfeeding?**

R: Yes. According to what they want.

**I: So, they don’t think about what their body can.**

R: Yes.

**I: What kind of food do they advice the women who are breastfeeding to eat and why?**

R: The food they usually tell them to eat are fish, meat (local foods).

**I: Why do they tell them to eat lots of local foods?**

R: Because there are lots of vitamins in them and it gives them strength. To have the strength to look forward.

**I: Now. Who would encouraged or discouraged these women to eat these foods during breastfeeding?**

R: The health worker at Majuro hospital or the health assistant and their grandmothers and grandfathers.

**I: As a health worker. What are some of the biggest concerns of the diet of pregnant women and breastfeeding women in this community you work in?**

R: I’m always worried when they eat salt and eat the foods that can cause diabetes. Their glucose is high. When they go and check their glucose during pregnancy they have high results.

**I: Oh. Coming from the food they eat?**

R: Yes.

**I: Now for the last section, we would like to learn about ways we can develop health program in your community. Could you explain where community members usually get trusted information about health nutrition and health?**

R: The quickest and best ways to find solution for these things is to sit with the highest chief, the chief, mayor and other member to look for ways to have them make gardens/farms for people. Or the counsel or mayor can look for ways with our government as soon as possible. People are weak now a days.

**I: As a health worker where do you get your trusted information about nutrition or health from?**

R: I got these information from my working and how used to I am from working all these long years and from hardship. There are times when I would met wind and bad storm in the sea, but I kept on going. I was hungry and thirsty in the communities where I didn’t belong. But now its all good because I’ve reach my destination. I’ve been through hard times.

**I: Now you know.**

R: I know. I’ve been around in circle and I know what’s best and what foods are healthy.

**I: Where would these information/messages be delivered to so that the community members could see/hear easily?**

R: If not, the Public Health or others who also in this program should do outreach and give out information and make programs like this. And, advice the people because sometimes the people might forget and again do want they want.

**I: For the last question, could you describe what influences how people raise children in this community?**

R: How?

**I: How do they give advice to the people in this community? What do they say regarding how they raise the children?**

R: Oh. Those who have graduated from nursing usually come to this community to advice the people or conduct meeting with the young men and women. Do workshop with them and explain to them how to feed the children the right food. And when the health assistant has patients they would sit with them for 2 to 3 hours just to counsel them.

**I: What about how they raise the children?**

R: Regarding parenting. According to the information given they say they know how to raise children. these are the understanding that they really know how to raise children.

**I: Are there specific advice or information related to parenting typically give to community members?**

R: Yes. We would usually from the Public Health when they come.

**I: Are there any questions that pregnant or breastfeeding women typically ask from the health workers?**

R: Yes, there is. Usually they would ask from the health assistant about or when they don’t know something. Then we would explain to them.

**I: Now what is the best ways to communicate with the caregiver about health?**

R: Yes. If we don’t bring the mother and the father to teach. And then make programs at the churches and stand in the front and explain.

**I: On how you can raise your children.**

R: Yes. Parenting.

**I: Are there anything else you want to talk about that we forgot to mention?**

R: Yes. The most important thing is if the hospital and the community can work together to get things done. The most important thing is if we could work together through hardship and we’ll have a lot of accomplishments.

**I: Yes, that’s true. Okay, that was great, we are now done. Once again thank you for giving me this time to talk to you. I hope and have no doubt that all the information we gave us will help us find ways to improve the health of mothers and children in your community. Thank you for your information.**
