# Supplementary material for: Conceptualizing multi-level determinants of infant and young child nutrition in the Republic of Marshall Islands–a socio-ecological perspective
Source: PLOS Glob Public Health. 2022 Dec 19;2(12):e0001343. doi: 10.1371/journal.pgph.0001343 (PMC10022247; doi:10.1371/journal.pgph.0001343)
Supplement: S1 Data — (ZIP) [file pgph.0001343.s001.zip › RMI Supp Data/Interviews data/I59R_IDI_SLHS_Majuro_Sep 21_Maryam.docx]

- Interview Code: I59R
- Interview type and interviewee: IDI_SLHS
- Interview Date: Sept.21.18
- Location: Majuro
- Interviewer: Maryam
- Transcriber: Marcellina

**I: okay okay thank you so much. So, I guess to start with, so we know the ICHNS survey last year, 35% of children are standard in the Marshall so I just wanna get kind of your understanding and your perception of why standing is so high in the RMI?**

R: we do believe that children are not eating or fed well. I also believe that a lot of our mothers… maybe they are not… the stage between each child is probably not enough… they have frequent growth, so with that I’ve noticed that as soon as a child is born, the older child gets neglected… and so we see that a lot among many families so and they the mothers then now turn to the baby and take care of the baby and they forget the older child. And then as the older child gets older, the older child is given the responsibility to take care of another younger child, and then another baby is born and so the mother then focuses on the, and so you kinda see that cycle. So, I think that’s why children are standard. Also, our environment here is not very sanitary and you know, we have a lot of issues with sanitation. And you know who knows maybe that’s… you see the kids when it comes to rainy days, they play in the mud. You know it would be nice if the mud was clean, but you never know ‘cause the water is coming from everywhere. So yeah, all those contributing factors, I know there are lot of homes that are overcrowded. So, it’s overcrowding, it’s not enough foods, it’s sanitation issues, yeah, I think those are very real contributing factors.

**I: so, when you said children are not fed well, can you elaborate on that?**

R: whatever the family is eating, is what the child will also eat. And so if the family is not eating well; if they are just gonna have rice and a meat on the side, that’s what the child is also gonna eat. Also, in many of our typical homes, many children share one plate. Especially in the outer islands. They’ll put the food in one plate and then the children sit around the plate and they eat. So, whoever can catch as many food, I am sure probably gets most of the food, compare to the little ones. I rarely see in, especially in the outer islands’ homes, or in the small, more crowded area, I rarely see each child have their own plate and just sit. It wasn’t like with us, me as a child growing up, I remember, me plus my other three siblings, we will sit, and we each get a plate. So that was my mother’s work. But I think today, who knows, maybe mothers are getting lazy or, so I’ve seen a lot of children, which we also state the study at home on children, on what they are eating, and they found that, we found that they’re just sitting there, sharing a plate. So that could be a contributing factor too.

**I: so this sharing plate like sharing food, when did that shift, do you know?**

R: I don’t think it has shift, did I think, you mean by age?

**I: by when you said that, you know back when you were child you were, you have separate…**

R: I have no idea but, when I was a child, more than fifty years ago, so… I don’t know… I think maybe by the time you’ve came to the 80’s, so maybe that’s when the traditional ways our parents raised us kind of disappeared, I don’t know why but, I don’t know, I can not say when, but probably around the 80’s and 90’s…

**I: what happened in the 80s and 90s?**

R: I think more younger girls get more pregnant with more children… I think so. I mean a lot of my classmates, they didn’t go continue on to college because they stay at home and have children. And I think it’s very common too; as soon as you finish high school, you think you can now get married and stay home and raise a family. And so maybe around then, ‘cus I was like in the late 70s or 80s, people just kind of, some, very few go off to school but the rest stay home and raise a family. Maybe that was a contributing factor too. And when all this parenting skills changed, I think so, yeah just my opinion though.

**I: okay. And the food sharing with one plate, you know we get mothers who said you know they feed their children chicken or fish but, you’re right, if they’re on one plate, how much of that? Like the quantity, how much is the child getting?**

R: yeah we did find, older kids were eating most of the food, and the little ones were just not getting, but then they, when it’s finish, and that’s all they have, they’re not gonna get a refill.

**I: what study was that?**

R: when we did the household, it was household diabetes education… it was Johns Hopkins university, so… I think I have a paper on that one but, yeah I’ll look for it and I’ll share it with you, just remind me; just send me an email but, we were studying to see what we needed to develop in terms of educational tool kit for the family, so as a result of that study we develop a story board, and we would go and we would tell a story of so and so, we just came up with names. So we did have a story board and we would go back, okay today, today’s story we’re going to cook pancakes. And we’re going to add some bananas to the pancake, something like that but that’s a little bit different from the usual water and flour. So along with the story board, it’s also teaching them the household how to cook. So instead of just rice and chicken, rice and chicken and something else with it. They said “ oh it’s so expensive to get vegetables” so a lot of the foods that were used in the homes were either canned or frozen vegetables, and even just one bag was one dollar something, you know we also taught them that you know when you do it this way, it really doesn’t cost much, verses if you bought, how many chicken, and then the rice, but if add this much chicken and then add some vegetables to it, it’s still same cost or little bit less. So, it was all part of that household intervention study.

**I: okay yeah, I would love to get my hand on that**

R: then you sit down any you see the kids eat, after they do the cooking and then you can see how they serve the children. I don’t know if you’ve observe it yet but

**I: we’ve done household observation as well yeah**

R: have you seen any of that sharing?

**I: we’ll help you to get closer look at the data yeah**

R: but I think that’s really something, we don’t think about it. But if you really… now today if you kind of think back, you look “oh yeah no wonder this one is skinnier than that one, and that one was taller than that one”. I think so, because when the kids are eating nobody is seeing how much portion for you and you, doesn’t exist, you just eat.

**I: at what age the children start eating?**

R: even from three years old, so three four five six, the older ones like maybe, the ones in the, six seven grade so that would be what, how old would that be?

**I: ten twelve**

R: ten they’re not eating, but if there were bunch of little kids like two three and four or five, they all be just sitting there.

**I: interesting. And before two? Before two years of age**

R: the mother usually feeds them. By hand

**I: and that dies into, you know the hygiene, and like if she’s washing her hands?**

R: not only that but also, what ever they are eating, is exactly what the two years old is eating as well. There is no same. And so, when we say cook something, we cook something for the whole family. Because that’s what they can afford. We can not say, okay this one it’s just for two years old, this one is for five years old, everybody is gonna be eating the same thing. But of course, the… what you called it, was soft or hard

**I: okay so, the issues of food sharing that I think it’s one of the main issues, so for that study, did you create cook books?**

R: did we create cook books?

**I: yeah**

R: I don’t think we created cook books

**I: okay, okay,**

R: yeah, we didn’t.

**I: yeah, right**

R: no, we didn’t create cook books. We just kind of educated them what was on the story. Yeah, that would have been something but

**I: yeah that would be something open in our recommendation for yeah, for cook books to be like what the available ingredients, like healthy environment. Okay, so you mentioned children are not feed well, and if they’re eating like rice and chicken, so can you describe kind of like, from what you know like the typical diet for children under 2?**

R: children under 2, just rice, typical diet. Typical diet would be just rice, and they could probably put some milk with it, mash it up, or it could be… in a typical moment, or it could rice with something, whatever they have; maybe it’s tuna, maybe it’s chicken, it depends if the child has full set of teeth. If the child don’t have, full set of teeth yet and still small, then it’s anything soft. So, it’ll be, now a days they depend on baby food. But if you, the rice, you know they mash it up and it became really soft. Or the pandanus or pumpkin or whatever they can. Just make it soft, the consistency.

**I: but in terms of variety of food they’re giving it’s…**

R: I doubt it’s a lie, because here we also breastfeed, so there’s still a lot of breastfeed even all the way up to four years old. So, whenever there, they’ll first breastfeed and then they’ll complement with whatever soft food they can

**I: you mentioned one of the issues would be the frequent child birth, and the fact that the older children would get neglected as right when you know a new child arrived, so how does, we’re just interested in terms of breastfeeding, how does that impact**

R: it also ends

**I: yeah**

R: some kids, they’re lucky they can share, but as they get older, the mother tempts to again focus on the younger one. So even if that two years old they might stop breastfeeding. And it seems like our breastfeeding rate was lower, after…

**I: like so the continuing breastfeeding of a two years was 34%**

R: traditionally and the usual culture, everybody breastfed all the way up to maybe 4. So even with today again, that data says that it’s low. And so that’s means, it could mean that maybe another baby was born. So now the mother kind of focuses on breastfeeding that new born and wind off the other one. That’s also very common.

**I: are there any perception that explain to that?**

R: I think so, I think, well what I’ve heard was the bigger baby is gonna take away all the nutrients from the little baby. So yeah in one sense they believe that, but no it doesn’t, you know the breastmilk will always be there. It won’t run out unless you stop breastfeeding. So to me I think that’s also a myth that needs to be corrected. But yeah, I think, that’s what I’ve heard; the older child is gonna take away the nutrients from the younger child.

**I: that’s very interesting. In the terms of breastfeeding itself, so in the survey last year, exclusive breastfeeding for officially up to 6 months, was only 42%, so do have any idea why?**

R: well in the hospital, we know that it’s close to 100% when they’re in the hospital, but of course when they’re in the after birth, they’re only there for what, 24 hours 72 hours. We don’t know what happen and yeah we were very surprise to see that it’s low. So why did it happen? That’s why were asking ourselves. But from me I think, either the mother is a working mother and has to go back to work, which is very common now, or they just decide to change to bottle.

**I: do you know what would be influencing them to change to a bottle or**

R: no idea. The only thing I know is if they went back to work

**I: in some of our data or interviews, mothers would say that they ran out of milk, so there is the common percent, especially in the urban area, not so much in the rural area but in the urban area it came out that…**

R: they ran out of milk?

**I: yeah it’s the perception that they’re running out of milk, so they have to supplement with a bottle with formula**

R: when I was in nutrition program, one of the things that we really promoted and taught what women, it doesn’t run out. And we also encouraged them to finish one side and the next feed, you feed on the other side. Not to switch in between because you know the fat that came later on in the breastmilk, sometimes it doesn’t get there. You never know when it’s gonna- so the child only gets the poor milk and not the fatty part. So they never satisfied; they never fully satisfied. So those were some of the teachings that we went teaching mothers. But we really taught a lot about as long as the baby’s stays on, there’s going always going to be milk. And yeah, seems like a lot of the women knew that, I just don’t know why they would think they would run out of milk or not enough milk, maybe those that are really small breast, maybe that’s their thinking verses those that are big.

**I: I wonder if it’s like a messaging from the hospital or the nurse or I am not sure**

R: well during my time when we were doing hospital, baby friendly hospital initiative, one the thing that we taught all this about was to make sure you- this is what you say, “stay last on and don’t give anything not even traditional medicines in place of breastmilk or water for the first 6 months of life”. When I study breastfeeding more, I know that you can even exclusive breastfeed up to 8 months. So, there were kids that were really nice and chubby and child one, one of my staff, wherever we went we took the child with us. So, he was very healthy child and we use the child as a tool to teach mothers. This is exclusive breastfeeding, she has no water, no nothing, just exclusive milk. So those were some of the ways we taught mothers that just like john.

**I: and what was the other one?**

R: they sent their children to the outer islands, so they might have a lot of children here, they sent them off to mothers or parents living in the outer islands, and you do see that too. So I think when you go to arno, you might ask, is this your grandchild? Because there are majority of them, would have grandchildren in the outer islands, verses their own children.

**I: and the baby friendly hospital, so how long ago with that initiative?**

R: 96 97 98 up to 2005 when I left the nutrition program. That was my work.

**I: do you think that’s still going on or**

R: yes, we created the policies, so it is still happening, you can see the breastfeeding policy is still working and force because one of the thing that we stopped was vendors bringing in samples of formula. So immediately 96 no more formulas came to the hospital. And also, exclusive breastfeeding happens, is happening in the hospital. It just what happens when they go home. I think that’s still a problem.

**I: and then terms of the traditional medicines that you mentioned, can you tell us a little bit about like when they would give traditional medicines in the first few days of life?**

R: I am not really sure how long but just right after birth. The baby would take a bath, there is all of these things you have to rub on your baby, and then there is things you drop into the mouth, it’s not like they are drinking it, for a long time, it doesn’t replace breastmilk, but yeah they do that in the very few days, after they discharged from the hospital. And the mother goes through a whole bathing, which will bathing a whole month, hot water bathing, massage, for the whole month. And then the baby as well, have a shower in the traditional, mostly outside, they just, there is something that they dropped into the mouth.

**I: so as of our data collectors, they mentioned that during the interviews, mothers were kind of mentioned that the colostrum or the first milk was viewed as not helping, not good, so they would squeezed it up**

R: that was long time ago, we also told them that that’s like the first, that’s the richest milk a child could receive. You know that colostrum in the beginning, but yeah even in the 90s when I was starting to teach people about breastfeeding, that was the first thing they said, “Oh the yellow milk is not good” so we always squeezed it up. But that was like in the 90s, and if it’s still happening today, then maybe because we stopped teaching them. So we need to tell them that the colostrum is what you want to give the baby. Because then that really kind of get rid of the meconium right away as well. And they also don’t understand about the black stool that comes out, the sooner the black stool can come out the better. So all that kind of education to the new mothers, I think it’s very important.

**I: so why do you think that information is not getting to mothers right now?**

R: I think maybe they’re not prioritizing, I think a lot of things here happens, people take it for granted, that they know, that somebody others gonna teach them. That they’re gonna learn from their mothers.

**I: so like the nurses aren’t kind of telling them**

R: no the nurses teaching them what they can in hospital, but maybe it’s not all, maybe they missed the part about the colostrum, maybe they missed the part about teaching them how to you know do it fast so they can get rid of the meconium, well maybe, so I don’t know, we have to go look again and see do they have like teaching guides, you know when the nurses use to make sure you go through all the check lists and show the mothers understands

**I: because it seems like that information is missing**

R: but I know the midwife do educate about breastfeed, but again is to what extend what information do they missed out. But definitely they should not… I know one midwife who was one of my biggest champion, that’s where I learned about the colostrum, it’s from her, always “hey attach, attach to the colostrum, what is that, oh you know is the yellow milk, the first yellow milk” she would be like really teaching me about that, but not everybody say that unfortunately.

**I: you need that one champion. You need every, all nurses actually should be, at that level**

R: I think we really do need to educate the nurses again, they’re the ones who or they’re the one to, child comes out first, while the parents of the mothers is in labor, I know the mother is in pain, but in the labor room there’s the mother, there’s the grandmother, everybody is there. It’s gotta be a time when you, you know educate the mother waiting.

**I: yeah definitely. So we talked about kind of things that might cause stunting like the factors that you mentioned, you know, children not being fed well, they’re sharing the same kind of plates, the frequent birth like not spacing, kind of talked about breastfeeding, and in terms of exclusive breastfeeding, kind of talked about influencing and then perception of that, what about… so that’s kind of child heath section, what about maternal health? In terms of the biggest challenges to health of women, like women of child bearing age?**

R: I don’t know, because I can only speak for my family, I mean when my daughter got pregnant, we really took care of her, and we really make sure that you know, her and the father of the baby going to the clinic, “oh did you go and see your doctors? What’s the child immense eyes?” everything! We always make sure that she had the right food, you know she was comfortable, I mean she was educated because she’s educated because she graduated from the university but unfortunately not many homes are like that. So, I think mothers really need to be taken care of. I think we take it for granted that it just our natural ability or as our natural what, I don’t wanna say job, role and responsibility to be a mother, but we need to be taken care of too. Yeah so who knows, maybe these mothers are not been taken care of.

**I: yeah like not going to antenatal care**

R: even them, if they wanted to go to antenatal care, who would go with them, who would go and help them, and if they had children, who would take care of the other children, so they can go and take care of themselves. So all these factors too, if they have many children at home, of course they will be the last one, they’re gonna take care of their children first, and if they’ve had many children before, then they’ll say “oh I’ve been there, I know what it feels like, I don’t need to see the doctors, I know I am okay” so we have to again come back, and really tell these mothers, “when you’re pregnant, you’re in a very special condition, and don’t take it for granted, go see the doctors, get the care you need”, and maybe that’s what we should tell families to help support.

**I: and where would you get these messages out though?**

R: where would I get it out? To families, to homes, when we have our women’s group, even in church women groups, they should talk about these things, they shouldn’t just talk about fund raising for the church, which is so common here, when women get together is because they’re trying to do something for the community or for them, but it’s a great opportunity for them to also educate themselves about how to care for their daughters, how to care for their children, help each other, oh your child is what? Oh yours is what… you know share experiences. Sometimes I don’t want to be with women’s group because they spend too much time gossiping and not solving anything. And also many women do not want to admit that they have a weakness, when that there’s some fault in their family and there something they’re struggling with, they don’t want to admit that they need help. So maybe that’s when they’re just gonna keep to themselves and then they gossip about somebody else. But in actualities, them too. So I don’t know, maybe we need to again, we say this is a matriarchal society, women have power, women have the authority, but I think women need to support each other more.

**I: do think the concepts of society being matriarchal and be use to impower women to take care of themselves better?**

R: it should be. I don’t think so, I think it’s a very common attitude. I don’t know, it’s just depends. The group of young ladies that come together when we have an outreach or some kind of a focus group or something, they like it, they like coming together and they like talking and learning. Well these are younger women, they like to learn. So maybe there; we focus on these young women, focus on these young mothers. I think we have to really educate them. Because it seems like the older folks are not helping out, they’re not doing it. I think that’s the solution.

**I: so the women’s church group, and then so where are these young mother’s group?**

R: the ones I worked with like with our organization, the KUMIT, the COLLATIONS, they’re really not attach to any churches

**I: okay are they neighborhood collations or?**

R: they’re yeah, they’re like WETO, they’re like…

**I: the weto it’s like village?**

R: yeah, but these are individuals from each of these wetos who want to join the collation. So, they come together. When we have our retreat on the small island, I haven’t taken you there yet have I?

**I: we went to... yeah there was one day we took Kelsey but you weren’t available**

R: okay! Was it okay?

**I: it was great, it was amazing! Thank you so much.**

R: we would take young girls there, we would just talk stories, hang out, singing, so those kind of activities. They’re really kind of activities, but those are really important activities.

**I: what was the name of the collation?**

R: KUMIT, and also youth to youth in health; that’s also another nonprofit organization. And those kind of organization are really the community base 1. Even the WUTMI, the salvation army, even Coop school they would do retreat too; separate girls from boys, those kind of program.

**I: yeah there is just thing the younger generation can kind of be targeted more, because they’re more perceptive**

R: yeah and they need to know because I think adults are not doing their part, their parents; I think their parents are too busy now a days. I know parents, lots of parents are busy in churches, and so when it comes to church activities, they’ll all just go and leave the kids at home, for other people to take care of. So I think our priorities have really shifted, I don’t know why.

**I: so, a church can be a good place?**

R: a church can be a good place to empower them to educate these families, some churches do that like the Catholic Church, they really encourage family, every time, but some churches I think not.

**I: so I don’t wanna take much of your time, I just have one last question…**

R: only one?! Are you sure?!

**I: yeah, this is kind of the bigger thing that I just want to check with you, we keep hearing about the stomach bump?**

R: what?

**I: stomach bump. So they say when we ask them about child illnesses, and they say “oh you know, anytime a child falls over…”**

R: yeah that’s very common.

**I: yeah so we’re just wondering because we haven’t heard that in any context before…**

R: it’s like swelling.

**I: it’s a swelling??**

R: it’s a swelling. Could be an infection, but they just called it EBBOJ. So because every time a child falls, also it could be hernia, but because when they massage it, the hernia goes down. But they don’t know what it is, because they just know it’s a bump, and they’ll just, all they know is they have to massage it. That’s just like a traditional, so if a child falls, if a child is very hyperactive, and then they get skinny, and then they say “ oh maybe EBBOJ LOJEIN”- “ maybe the stomach is SWOLLEN”, the belly, the abdomen is swollen. It doesn’t necessarily mean the stomach. But if the abdomen is swollen, let’s have somebody massage the abdomen. And when they massage the abdomen, they’ll feel bumps, so they’ll keep massaging it until the bump goes away. So if the bump is from what? I don’t know because it’s not clinically tested or anything. But it’s just a bump. They believe it’s a bump because the child was always jumping around, so again all those jumping around maybe hernia, and then they massage it down. For boys especially, but for girls, it’s not too much for girls than boys.

**I: yeah that’s what people have been saying, it’s mostly with boys.**

R: because they say they’re always jumping all over the place, or falling from a tree, so they say the abdomen is swollen. It’s not bumps, but when you massage you feel bumps. Even us older people sometimes it gets really tight, so uncomfortable, or backache, then the person will; the traditional medicines person will massage you. And get rid of all of these bumps on your stomach, lots of them are at the back so they cannot get rid of, but yeah, they’ll keep massaging and then lay off and say your stomach is very soft. Yeah because they kind of get rid of all these bumps and then your back doesn’t hurt so much

**I: yeah it’s getting a massage**

R: yeah it’s a massage

**I: yeah okay.**

R: that’s what it is

**I: okay yeah because we heard it so much and we’re just like “what is this? A stomach bumps? What is this?”**

R: yeah it’s not like boil, or it’s not like the tumor, it’s just because it’s swelling, they feel swelling because maybe of jumping around. And then, I’ve proven it too, because my son was very skinny and all, “why is he losing weight? Is he eating?” so his grandmother, massaged him. And his grandmother was blind, so her hands were very good with hand feeling and if there was anything, and then from that time after she massage him, until now he’s huge; he just gains weight like that. He just wanted to eat.

**I: what you think it was?**

R: I don’t know. But he just wanted to eat and eat and eat. If you see my son today, he’s like a sumo wrestler, and he went to school in Japan, so people would kind of get out of his way. But I’ve proven that, that works, and they massage the stomach. Maybe I don’t know, maybe they put the stomach back in place, maybe they… moving things as they are massaging. And when the child is eating well again, and they get bigger.

**I: have you ask one of the doctors in hospital about…**

R: yeah. like have they seen it? Or what could be it?

**I: yeah**

R: but it works. It really works. I’ve proven it with my own son.

**I: okay yeah interesting. I just- in one more thing, in one of our, I think, I just got a chance to look at, just preliminary kind of transcript that we have so far, I think it’s 1 or 2 instances, caregiver mentioned that you know before they would use like physical disciplining of children, a grandmother was explaining this actually, so I just want to get your perception or kind of your knowledge on what is commonly, like what methods are commonly used to discipline young children?**

R: well during my time it was sticks, but now it’s not so much… physical, it’s a lot of verbal. But if they would want to a child, maybe belt, maybe stick, but just the verbal it’s really bad. So I am really glad you know we have the child protecting ads, you know those thing are really coming up, we just had a discussion a few days ago, about what is child protection, what rights do the child have to be protected, how do we protect the child, and we learned about the preventions, the response, you know all those things in order to stop these corporal punishments, physical, emotional, mental, but yeah, before long time ago when we were in school, it was very strict, so for example, I was in catholic school, so when the boys did something wrong, they would have them lay down on a barrel, that was under the sun for so long, so they got really burnt. Because the priest would say “okay! You didn’t listen, lay down on that barrel.” But the teacher that we had in second grade, every time she gets mad at somebody, she would just pulled the ear and twisted it, as hard as she can. For a child in the second grade, that’s what a 7 years old, and the ears just turned really red. But growing up, and even today I think, children just don’t complain, they don’t say anything because they think it’s just the respectful way, it’s an adult, so an adult, an adult probably they feel, they should know, it’s there, right to punish, and your child, so you just take it. But of course with all these ads and conventions of child’s rights, people have started to be more aware. But there are certain discipline that needs to take place, but not that kind. But it’s still happens. Even mothers, they think I really notice a lot but it’s just the verbal. They cuss their children, they’re telling their children they’re stupid, they tell their children “do you want me pick up this rock and throw it to your head?!” you know “your head is so stupid, you’re not listening to me”, so those kind of really verbal punishment that I think are worse than pain. So I see that a lot with the grandmothers, I see that a lot with young mothers, with many children. I guess it’s just the way they were raised in the home, that’s what they learned from their mother, pass it down to their children. But that’s what I’ve seen.

**I: have there been any kind of education campaign or anything?**

R: we do here, from our social protection offices, you know like how to talk to your children, how to take care of your children, and again now they’re saying you know, if we find that you’re abusing your child, your child will be remove, remove to where, to a another family maybe, or you’re gonna go to jail, and there is a lot protection about child abuse, child raped, sexual abuse, people are getting prosecuted, before no would ever talk, but now people are starting to understand that you can talk. The only thing that is really hard for family is, when is happing in the family, by a family member, so that’s the hardest thing. But people are stepping up now, and coming forth. So yeah people are starting to understand, but it just, more again, it’s gonna be part of the parenting skills, because we really like parenting skills, I don’t why because we didn’t go to parenting school, but it’s just the way you raise, it’s just the family that you were raised in. so no matter what kind of parenting, you gonna keep saying oh we need more parenting education, why?! What about your role? Why didn’t you teach your daughter how to take care of her family, I see that a lot too. I see that lot of mothers getting mad at their daughters, saying “you’re stupid in your family, no wonder your kids are stupid because you’re stupid”. You know those kind of talk. So when you hear those kind and like oh my god no wonder this family is a mess. So that kind of cycle needs to stop. And how do we stop it? I think we really need to start educating the young people now who will become future parents. Because I think we’re focusing on teaching the older now it’s just too late. So let’s take care of the little children and let’s educate the future parents.

**I: yeah that’s where you see the most potential**

R: it’s just, no more time to, teach old habits, or break old habits right?

**I: yeah. Let’s**

R: but people need to understand their, would they get access to services, or they don’t have so many children, family planning,

**I: yeah so family planning, is there any outreach going out…**

R: not so much anymore, it’s really, not so much from the hospital, but these community base from the organizations have taken it on. For example, youth to youth in health, that was one of their primary role, it’s a nonprofit organization but they been surviving for about maybe now almost 30 years. And that’s what they’ve been teaching. You know teaching their peers about family planning. But again, if they don’t have the commodities there, they need to come and… there needs to be collaborations between this ministry and them. You know commodity, if you do so well in outreach, go and do it. So instead of saying “oh we’ll do it” because we’re government and we’re health, that’s something needs to be stop.

**I: who can initiate that connection?**

R: well I can now, because that was my area, but I’m hoping that and I tell them, like the directions of MCH, the nurses, family planning, “you guys cannot do this work? So go out there” the public health nurses, “go out there and see who can help you, teach them, and let them do it”. I think that’s what you can do, it has to be in the shaded truth from our level but, to go out and do the work, we got to have people understand and take that passion. You know, they got to see it that it’s important. Because if I just tell them to go and do it, they’ll just go and drop it off somewhere and they won’t even do anything about it; they won’t say how to use it, how to disseminate it, “here is the box of condoms, okay let me do it, oh I don’t know”

**I: just make sure your hair go away**

R: that’s why I got so many, unless you wanna stop coloring. But yeah that’s really our role when we were working for youth to youth in health, peers to peers, so powerful. When peers, when young people have problems, they’re not come to their mother, they’re gonna go to their girlfriends or their boyfriends, and then they’ll talk about it.

**I: these kind of grass roots organization, are there, do you know about any programs they have about educating on nutrition or health?**

R: mostly I think for adult. But not so much children. So it would be really nice to see who can really help, you know WUTMI, they do have parents and teacher program, so they do teach the mothers how to feed their children, what to feed them, but that’s just one group again, you know there is still whole lot of people out there that we can reach. So yeah I think need to kind of continuing doing that. That was kind of work when we had our nutrition program.

**I: really?**

R: I don’t go, I don’t know, teach people how to make baby food from pandanus, how to mash bananas and just mix it with the rice or something whatever the household have, don’t just give them the rice and the water. Put some, maybe mash some bananas with it. So we did that all… that was part of our works, so maybe I think what we need to do is re-establish another nutrition unit, and then put those nutritionist there who can really focus on children. I think that’s really a, the way forward I can, it’s like we’re going back, but i don’t know why it stopped. I think this ministry is what they’ve done, is they integrated health promotion with nutrition. And they, nutrition itself was so important, that when they integrated as part of the promotion, it got dissolved or it got…

**I: it got lost in there**

R: yeah. It wasn’t so much of a priority. It could became holistic health promotion holistic. Eat right foods, eat this, not so much focus on the malnutrition, not so much focus on what’s, what the right foods are for the diabetics…

**I: so they tried to do anything but didn’t do anything?**

R: it’s like you know so much, what is the say? I know a lot, I know a little of everything, but I don’t know a lot of one thing. That’s the same thing, you know a little of everything and then you get lost, you don’t, you don’t solve anything. Because you know a little of everything. So I am gonna say, it’s not good.

**I: yeah. Do you think it should be worth focus like they should have…**

R: yeah. This ministry needs to re-establish a nutrition unit, and just put the nutrition local person, whose gonna take care of the early childhood development, whose gonna take care of the nutrition education, make sure the mothers who get discharged from the hospital continue breastfeeding at home, I think that’s gonna really solve that.

**I: yeah okay last, WUTMI, do you know what their reach is?**

R: well they do have a lot of, women chapters all over that, the country, in all the atolls, but I think wutmi also realizes that they need to do a little bit more to mobilize these; their chapters; they have everywhere! They have all the atolls. And the same with the kumit collation, but the kumit collation the reason why they were really established what should we do with substance use, among young people. But they’ve really expanded to family education. So they target parents, high risk family who are dealing with substance use, and they bring the two parent, plus the children, the young children, from 6 years up, so definitely more can be done out there to reach little children.

**I: I think that’s it, I think we kind of covered like the main causes, kind of the rates that we are seeing, and kind of identifying the caps, the nutrition, departments here in the ministry,**

R: I think that’s really important

**I: yeah, for them to do outreach**

R: people need to start spending more time to educate, because I think we’re so, we see the patients, we want them, we want to get their medicines and get them out of the door. Oh my goodness, we need more time, to at least just say, are you okay with your medicines? Do you have any side effects? Do you need help? You know with any, question about your medicines? What do eat? When do eat? When not to eat?

**I: that is so confusing just like yeah**

R: I think they’re just need to be, more time educating our patients. And more time again on prevention. Gosh! This country was a really nice peaceful community oriented, cleaned, our culture is a really protective culture, but I think people are losing that culture. I don’t know why. I think they’re really, a lot of influence from outside, and especially now with iphones, ipads, almost everybody has a ipad, almost every child has a ipad, not almost every child but this year in the urban city. Even in the outer islands, if you go to the outer islands, they have iphones, they’ll take pictures with a iphone.

**I: but they don’t have reception there do they?**

R: it doesn’t matter the reception, they just want the picture, they do have… you can do cell phones,

**I: yeah in arno arno**

R: and some outer islands you can, but not all. But yeah! They do have access to those, and those place have access to the internet, they’re on facebook! But they aren’t facebook from the outer islands, and I am like when did that happen?! You see them on facebook but they are in the outer islands.

**I: so that could be kind of a avenue for… kind of education, kind of getting information of…**

R: yeah so many kind of influence now, so many kind of information. So when they come here, I don’t know, I still like our culture, I still like our dresses, I still like our handicrafts, flowers, I don’t know why you want to use anything else? You know. You look good with what is yours. And then when you try to wear something that it’s not your, you look kind of awkward.

**I: out of place, yeah.**

R: but I think so, yeah. That’s my thinking. Yeah we’re very careful too because we do have grandchildren, my husband and I, our youngest grandchild, she’s 3 years old, but you know she’s very smart, she’s very hyper, very spoil I think, but still we have to tell them, you know, there’s a limit to everything. And don’t ask for anything you cannot have, and always respect the elders, when people talk don’t talk, you know we teach them these things. But if you go to a party today, majority of the people there will be children; will be the young people. Because families just oh… they’ll invite you as a… before when somebody has a birthday, everybody goes because we have to celebrate that first child right? That first birthday. Everybody goes; from the oldest to the youngest. But now, older people just send their kids “oh okay go” and the kids go for them. They have no understanding of what their role is in that birthday, you know you don’t just go and grabbed foods, and grabbed everything and run around like crazy and mess up the place and then go home. You know everybody have a role. It’s not like that anymore. Now a days people just go get the food and go home. They forget to go and shake the hand of the child, you know kiss the child, or give the gift to the child, they just go get the food and go home. They forgot to go and say happy birthday to the child. So sometimes we say “is it worth it to have just a big party?” have you been to a big birthday party?

**I: no, I haven’t**

R: we cannot even afford a big birthday party and all of us work at my house. And people take out loans to have these big party, and they’ll have a theme now, like cinderalla theme, spider man theme, you know, and then they just, they rent a big hall and they invite everybody, make all these tons of foods and…

**I: excessive**

R: too much

**I: yeah and when you said it’s so expensive, like how expensive the food is here, like the fresh fruits?**

R: my gosh! Even a strawberry, you can’t even eat a strawberry

**I: no of course not! Two heads of lettuce are $9! I am still stuck on that.**

R: so that’s why families cannot afford it. And so if they do, they do depend on their local foods, but lot of family don’t grow their local foods at home. And you don’t have access to it. So…

**I: in arno, I was actually shock that nobody was growing fresh vegetables…**

R: and they have really nice soil there

**I: no vegetables or what so ever. Yeah so the local foods would be you know, that coconut, or pandanus, or papaya, banana, but even that wasn’t eaten often, it would be eaten as snacks for children? But in terms of meals…**

R: they don’t regularly make them.

**I: no**

R: you can, you suppose to. But people get so… like use to rice now, that is so convenient, they forget to make the breadfruit, the banana, and they have it. And their land is very good compare to here. But they don’t grow it. They really do neglect their land to, lot of bushy areas, and yeah you’re right, like papaya I didn’t know that Marshallese people didn’t like papaya, I mean they don’t like the yellow one. So, I was in one of the outer islands, and then I saw yellow ones, I was the only one in the whole island eating the yellow one, I said “how come you guys let the yellow just falls to the?” oh it’s too old now; the yellow it’s too old. They want the green one. Because then they pickle it and they make it red, and make it like a pickle papaya. I said all of my guides, you guys are missing out on… so, when we eat yellow, they kind of like “is it good?” “How was your first one?” they asked me that, I said “what you mean is it good?” Its papaya!

**I: what were they doing before if they learned how to pickle?**

R: maybe they wouldn’t eat

**I: that’s a new kind of influence right?**

R: maybe they didn’t eat, because when I was in my 3^rd^ grade, my family moved, my family moved to Saipan.

**I: where is that?**

R: CNMI (common wealth of the northern Marianas), near Guam

**I: oh okay**

R: that was the trust territory headquarters. So during that period, the Marshall Islands was under the trust territory, so that was the headquarters. So all the governments went there. So that’s where we moved, and I didn’t move back here until I was in the 11^th^ grade. So when we would come here for summer, you know we would try find the things that we would eat, it would be like papaya, because we were crazy; “oh there’s so many ripe papaya here!” but I never saw my cousins eat ripe papaya, so I don’t know if they wanted to ate it, I don’t know, was it just for older people? Because I saw my grandparents eat but I never saw my cousins eat. I don’t know if that change or it just few people that I see. But yeah in the outer islands, all these papaya, they would just fall to the ground. I said “why don’t you guys eat them when they’re ripe? And they would say “they’re too old” as if the kids don’t like it, the kids don’t like the ripe papaya. I said “why?”, so what do they like? “Oh they want us to make big pickles”. But before you learned pickle, but I know pickle came here in the 1970s, so how about before it then?_

**I: no**

R: but I grew up with pumpkin, my mother would always make us pumpkin rice. Maybe that was because we had rice and we pumpkins in the yard. As pumpkin can grow everywhere. So that was very easy for her to make, she would just make the pumpkin and rice and she would put food on plates and line us up. That’s what I can remember. yeah. But today no, mothers don’t take their time, to make something nice but, just make it quick. Even today women are so busy with church function or something. They let their older kids do the work. They let the older kids cook the rice, take care of the children, wash the dishes, everything!

**I: yeah I mean in the outer islands, a lot of people like in arno, a lot of people were selling you know the copra, you know that’s where they get their income, and they would go straight to the stores to buy rice, flour, canned meats…**

R: and the kids don’t go to school on Somedays, because there is going to be a boat coming to pick up the copra. Or the family needs to prepare the copra. So that they can put them on the boat and get the money. So some kids don’t go to school so they help the family prepare the copra. And that’s fine! They need the money… but they need to go to school as well. But! They’re not also grow anything else in their land, that’s all they have; it’s just copra. I mean I didn’t see a lot of breadfruit tree, a lot of banana trees, at least on the side of Arno, in the middle, between Arno and Ine. So I was just thinking, it’s just mostly copra. Did you guys drive to Arno?

**I: the team did but I didn’t**

R: if you drive from Ine to Arno, of course it’s very narrow, but some areas are get wider, but nothing, just nothing. Just coconut trees. People do get really lazy, I don’t know why.

**I: could be the, you know in terms of education not prioritizing, not knowing how necessarily it is for your health?**

R: I think it’s gonna come down again to just education. Lack of understanding, and yeah who would have taught them because my grandparents taught us. In a way, we wake in the morning, you clean your yard, before you get ready to go to school. And then when it’s time to harvest, we always go help our grandmother harvest the first fruit, put it in a basket and take it to the chief. And the chief will say “okay thank you”, and he’ll take some and he goes “okay you take the rest”. Because that’s just the traditional; first harvest has to go to the chief. So we just learn that. Wherever we grew up, we always have lots of like plans around our home. We grew up with our own rules. So I just don’t understand why people don’t want to do that, I don’t know why they don’t want to keep their homes screen…

**I: for us to solve them by now**

R: okay let’s go

**I: okay thank you so much. Is there anything that you think we missed?**

R: people here needs to... I don’t know, we’re just a poor country. People here needs jobs, but at the same time, they shouldn’t depend on money. They should try and do as much as they can to… also be self-sustained, they go fishing, or what can we do? I mean this world is also changing. There’s no more fish, I mean no more fish, it’s just every all these fish are getting smaller. So with climate change, I don’t know that’s also has a big problem. But yeah no matter what, some area are affected, some are not, those that are not, I think we still need to do the best we can to grow our own food, we need to be a little bit more… we need to stop being lazy. I don’t know why, I think, we still accustom to, just work go home, work go home. But yeah life is changing. But then the more you work, I think people just need more salaries. I think pay level here is still very low but inflation, living cause is really high. So I think that’s, and I am glad that, and hope it does go through that this new early childhood development project will provide some help to these families that really need the foods and the financial help. Okay I think that’s it, I think families are poor that’s why they can’t afford.

**I: okay thank you!**
